# Gliosarcoma: The Distinct Genomic Alterations Identified by Comprehensive Analysis of Copy Number Variations

**DOI:** 10.1155/2022/2376288

**Published:** 2022-06-15

**Authors:** Chuan-dong Cheng, Cheng Chen, Li Wang, Yong-fei Dong, Yang Yang, Yi-nan Chen, Wan-xiang Niu, Wen-chao Wang, Qing-song Liu, Chao-shi Niu

**Affiliations:** ^1^Department of Neurosurgery, The First Affiliated Hospital of USTC, Division of Life Sciences and Medicine, University of Science and Technology of China, Hefei, Anhui 230036, China; ^2^Anhui Province Key Laboratory of Brain Function and Brain Disease, Hefei, Anhui 230036, China; ^3^High Magnetic Field Laboratory, Key Laboratory of High Magnetic Field and Ion Beam Physical Biology, Hefei Institutes of Physical Science, Chinese Academy of Sciences, Hefei, Anhui 230031, China; ^4^University of Science and Technology of China, Hefei, Anhui 230036, China; ^5^Precision Medicine Research Laboratory of Anhui Province, Hefei, Anhui 230088, China; ^6^Precision Targeted Therapy Discovery Center, Institute of Technology Innovation, Hefei Institutes of Physical Science, Chinese Academy of Sciences, Hefei, Anhui 230088, China

## Abstract

Gliosarcoma (GSM), a histologic variant of glioblastoma (GBM), carries a poor prognosis with less than one year of median survival. Though GSM is similar with GBM in most clinical and pathological symptoms, GBM has unique molecular and histological features. However, as the rarity of GSM samples, the genetic information of this tumor is still lacking. Here, we take a comprehensive analysis of DNA copy number variations (CNV) in GBM and GSM. Whole genome sequencing was performed on 21 cases of GBM and 15 cases of GSM. CNVKIT is used for CNV calling. Our data showed that chromosomes 7, 8, 9, and 10 were the regions where CNV frequently happened in both GBM and GSM. There was a distinct CNV signal in chromosome 2 especially in GSM. The pathway enrichment of genes with CNV was suggested that the GBM and GSM shared the similar mechanism of tumor development. However, the CNV of some screened genes displayed a disparate form between GBM and GSM, such as AMP, BEND2, HDAC6, FOXP3, ZBTB33, TFE3, and VEGFD. It meant that GSM was a distinct subgroup possessing typical biomarkers. The pathways and copy number alterations detected in this study may represent key drivers in gliosarcoma oncogenesis and may provide a starting point toward targeted oncologic analysis with therapeutic potential.

## 1. Introduction

Glioblastoma (GBM) is the most common and aggressive malignant tumor in central nervous system [1]. Gliosarcoma (GSM), a variant of GBM characterized with a well-circumscribed lesion with discernible gliomatous and mesenchymal components, accounts for 2-8% of all GBM types [2]. GSM is similar with GBM in most clinical and pathological symptoms, and the clinical principles of treatments with GSMs are followed with the guidelines of GBM treatment [3]. However, the unique features of GSM suggest that it may be a separate tumor type, such as extracranial metastasis, distinct radiological features, and poor prognosis [4].

As the poor prognosis of GSM, several researches were performed to detect characteristics of genomic alterations to understand the molecular etiology. Among these candidate genes, EGFR (epidermal growth factor receptor), PTEN, and TP53 are the most commonly reported. It was reported that the gain of 7p and 10q loss was associated with the amplification and overexpression of EGFR in IDH-wild-type GBM [5]. However, the EGFR amplification is rare in GSM. In addition, the mutations of EGFR were also not common in GSM [6, 7]. So, several drugs which were designed to specifically target EGFR mutations were failed in the clinical study of GSM [8]. Following the reports of other candidate genes associated with GSM located on chromosome 7 (such as CDK6, PDGF-A, and c-MET), it was suggested that the key oncogenic genes drive the process of GSM independent with EGFR pathway [7, 9]. In GSM, TP53 mutations were more common to be detected (70%), compared with GBM cases (32%). Furthermore, it was showed that TP53 mutations showed a positive correlation with the shorter survival time and epithelial mesenchymal transition (EMT) process of sarcomatous components of GSM patients [10]. Though some potential biomarker genes have been identified, the typical mechanism of GSM development was not well known.

In order to further study the diversity between GSM and GBM in genome level, we collected 21 GBM samples and 15 cases of GSM to examine the DNA copy number variations. We found that the abnormal genes which were detected in GBM and GSM were enriched in the similar pathways, such as JAK-STAT, PI3k-Akt, and cytokine. However, the pattern of genomic alterations (loss or gain) of candidate genes was displayed an obvious difference between GBM and GSM.

## 2. Materials and Methods

### 2.1. Tumor Samples

Patients with GSM and GBM were initially identified through the database of Anhui Province Hospital with dates of diagnosis from 2016 to 2019. The clinical history of the patients was gathered retrospectively by chart review. All GBM and GSM cases enrolled in our analysis were examined and graded independently by two neuropathologists (who were blind to tumor genotypes), according to the 2007 World Health Organization (WHO) Classification of Tumors of the Central Nervous System [11]. All samples were obtained with informed consent at the Anhui Province Hospital, and the study was approved by the International Agency for Research on Cancer Ethics Committee.

### 2.2. DNA Extraction

Genomic DNA was extracted from typical tumor areas that were scraped from formalin-fixed and paraffin-embedded tissue slides or cryostat section from a frozen sample. Total DNA was extracted from the sections using a QIAamp DNA Mini kit (QIAGEN, Hilden, Germany). DNA concentration and purity were measured by a ND8000 spectrophotometer (NanoDrop).

### 2.3. Analysis of Copy Number Variations

Paired reads were aligned to the hg19 reference genome using the BWA (V0.7.15-r1140)-mem command and then sorted and indexed using SAM tools. CNVKIT is used for CNV calling. CNVKIT algorithm was used to construct reference library with all samples, and then, the copy number of a single sample chromosome segment was calculated. The copy number > 2 was considered as AMP, and copy number < 2 was DEL. Fisher's exact test was used to calculate the correlation between copy number change and grouping. *P* < 0.05 was considered as significant correlation.

## 3. Results

### 3.1. Analysis of DNA Copy Number Variations in Chromosome Level

**Figure 1:**
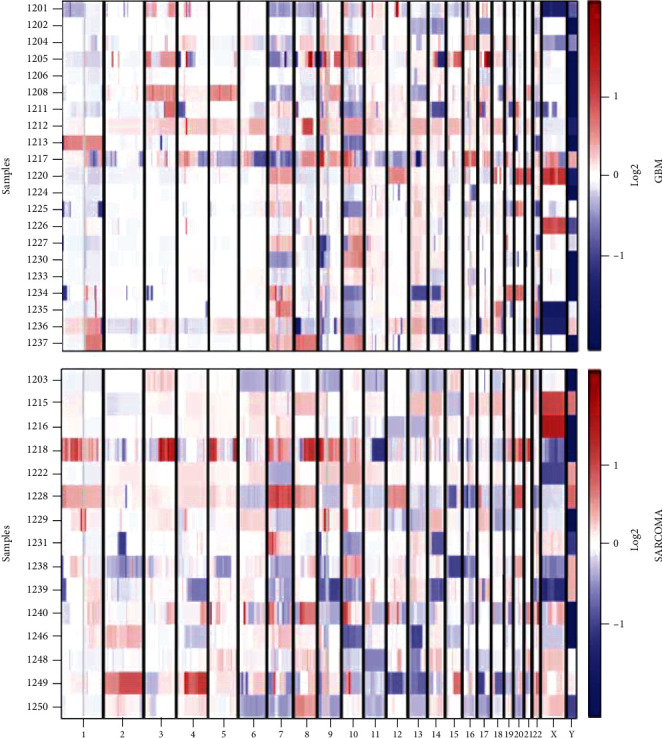
The distribution of genes with CNV on chromosomes. 21 cases of GBM and 15 cases of GSM.

To compare the genetic differences between GBM and GSM, we discovered genomic alterations of DNA with WGS technology. 21 cases of GBM and 15 cases of GSM were collected, and the detailed clinical information for each patient is provided in Supplementary Table S1. Firstly, we located all detected abnormal genes with CNV on chromosomes. As the Figure 1 displays, each chromosome had a similar pattern of corresponding copy number amplification/deletion in both tumors. The chromosomes 7, 8, 9, and 10 were the regions where CNV of DNA frequently happened in both GBM and GSM. However, the distribution of CNV in GSM showed an obvious signal in chromosome 2. It was suggested that there were some potential biomarker genes which could distinguish GSM from GBM in this chromosome.

### 3.2. The Pathway Enrichment of Genes with CNV Alteration

**Figure 2:**
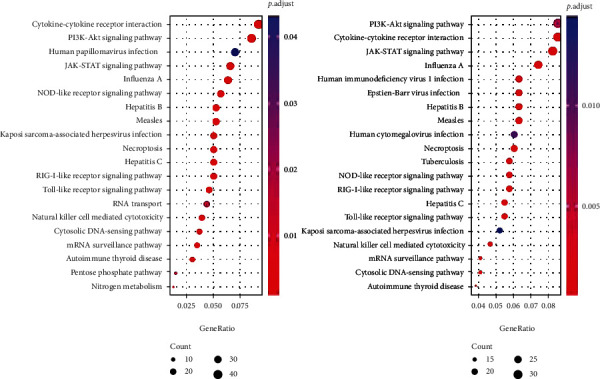
The pathway enrichment of screened genes with CNV in GBM and GSM.

To identify the significantly different genes, we defined that the copy number > 2 was considered as AMP (amplification), and copy number < 2 was DEL (deletion). We investigated the differences in the pathway enrichment. As the data shown (Figure 2(a)), the candidate genes were mainly enriched in the pathways of cytokine-cytokine receptor interaction, PI3K-Akt, JAK-STAT, and NOD-like receptor signaling in GBM samples. Most of the enriched pathways were the common reported signals included in the tumor development. For GSM cases, the pathway enrichment also displayed a high similarity with GBM (Figure 2(a)). It meant that GBM and GSM may share the same or similar mechanism of tumorigenesis and metastasis.

### 3.3. The Unique Alterations of CNV in GBM

**Figure 3:**
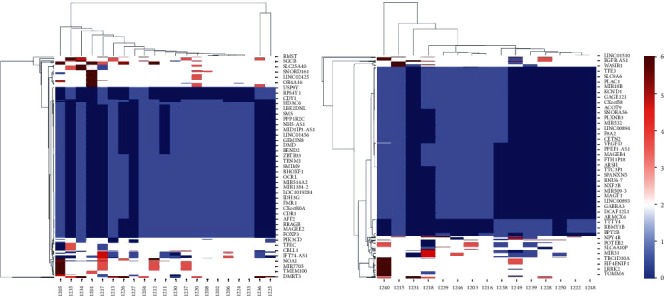
The gene list of screened genes with CNV in GBM and GSM.

To further probe the underlying distinctions between GBM and GSM, we focused on the patterns of copy number changes for each gene. We listed the aberrant genes and found that there were a number of gene amplification and deletion in GBM and gliosarcoma (Figure 3, Supplementary Table S2). We firstly studied the well-known CNVs, such as EGFR, PTEN, and TP53. The AMP frequency of EGFR was 38.10% in GBM, compared with 22.22% in GSM. The DEL frequency of EGFR was 9.52% in GBM, but no CNV signals of EGFR were detected in GSM. For PTEN, the AMP and DEL frequencies were 14.29% and 9.52% in GBM, by contrast, 16.67% and 5.56% in GSM. Interestingly, the AMP and DEL of TP53 were rare in both GBM (0% and 9.52%) and GSM (5.56% and 5.56%).

Besides, we identified some novel or few reported genes which displayed diverse CNV patterns in the two tumors. The early B-cell factors (EBF) are a family of highly conserved DNA-binding transcription factors with an atypical zinc-finger and helix-loop-helix motif. Here, we found the EBF mainly showed AMP in GBM (28.57%), while no AMP was found in GSM. In addition, lots of genes were identified as DEL. For example, the DEL of BEND2, HDAC6, FOXP3, ZBTB33, TFE3, and VEGFD was widely detected and showed a marked difference between GBM and GSM.

### 3.4. The Test of Compounds Targeting on Glioma

We collected the previous studies associated with the compounds targeting on glioma (Table 1). It was showed that most of the designed compounds targeting on the candidate genes or pathways failed. Among of these compounds, the target gene of romidepsin and vorinostat was HDAC family. In our work, we found that there was a frequent DEL event in HDAC6. So, the invalid effect of the two compounds may be due to the loss of target genes. Likewise, tofacitinib and idelalisib which targeted on the JAK and PI3K pathways also failed. The potential reason was the genome-level defect of genes in these pathways.

## 4. Discussion

GBM (WHO grade IV) is the most frequent and malignant glioma. Gliosarcoma is a rare histological variant of GBM [11]. In terms of clinical features, GBM is considered as a variant of primary GBM. Though GSM has unique pathological characteristics to distinguish with GBM, the genetic evidences that would allow a clear classification are still scarce.

Here, we collected 21 cases of GBM and 15 cases of GSM to explore the variation of DNA genetic codes. Whole genome sequencing was performed to discover the CNV patterns in tumors. Our data showed that chromosomes 7, 8, 9, and 10 were the regions where CNV frequently happened in both GBM and GSM. There was a distinct CNV signal in chromosome 2 especially in GSM. The pathway enrichment of genes with CNV was suggested that the GBM and GSM shared the similar mechanism of tumor development. However, the CNV of some screened genes displayed a disparate form between GBM and GSM, such as BEND2, HDAC6, FOXP3, ZBTB33, TFE3, and VEGFD. It meant that GSM was a distinct subgroup possessing typical biomarkers.

It was reported that chromosomes 9 and 10 had the highest number of losses, and the copy number of gains mainly occurred on chromosome 7 in GSM samples [9]. Loss of heterozygosity (LOH) on 10q is a frequent genetic alteration in both primary and secondary GBM, suggesting that 10q may contain tumor suppressor genes [12]. In GSM, LOH 10q was also frequently detected (88%) [4]. In our work, the events of gene loss were also primarily happened on chromosomes 9 and 10 in GBM and GSM. Furthermore, we found the AMP assuredly aggregated in chromosome 7 in GSM cases, compared with no obvious AMP in GBM. So, chromosome 7 may contained that some genes drove the tumorigenesis of GSM in a way different from GBM.

Previous researches have reported that the alterations of PI3K/Akt and RAS/MAPK pathways are crucial for tumor growth of GSM [13, 14]. Here, the genes with CNV changes in GBM and GSM were also enriched into pathways, such as PI3K-Akt, JAK-STAT, and NOD-like receptor signaling. It was further ensured that GSM shared a parallel molecular base with GBM, expect for pathological evidence.

EGFR was a gene detected with high frequency of CNV in the GBM. The amplification rate of EGFR is 35–45% in IDH-wild-type GBMs [2]. Interestingly, EGFR alterations were rare in IDH-mutated GBM but more prevalent in IDH-wild-type GBM [5]. In GSM, the amplification rate of EGFR was only 4–8% [7, 15]. In our cases, the AMP frequency of EGFR was 38.10% in GBM, and that of EGFR in GSM (most of our cases were IDH-mutated) was 22.22%. So, our study was consistent with preceding studies. Moreover, the mutation rates of PTEN and TP53 were 15–45% and 24–73% in GSM samples [4, 16]. Our data showed the amplification rate of PTEN was similar with previous work, but we nearly could not detect the CNV of TP53. So, more samples should be performed to discuss the role of TP53 in glioma.

Hypomethylation of EBF3 were observed in a number of metastatic tumors [17–19]. So, EBF gene was considered as a candidate epigenetic driver of tumor metastasis. The abnormal AMP of EBF in GBM may contribute to the metastasis. BEND2, HDAC6, and FOXP3 were the key genes controlling histone acetylation/deacetylation and chromatin restructuring [20–22]. ZBTB33 included in the Wnt signaling, TFE3, and VEGFD were the core genes controlling TGF-beta signal pathway [23–25]. The widely deletions of those genes displayed different patterns in GBM and GSM. It was suggested that GBM had its unique molecular traits. In addition, other biomarkers, such as circRNAs (circSMARCA5 and circHIPK3), were confirmed as good diagnostic biomarkers for GBM [26]. The study found that circSMARCA5 physically interacts with the oncoprotein SRSF1 and influence GBM cell migration and angiogenic potential [27]. In the end, combined with our analysis of compounds test, we speculated that more attention should be paid on the genetic characteristics of individual patient to avoid the probable situation of absent of drug targets.

## Figures and Tables

**Table 1:** The test of compounds targeting on glioma.

Compd.	Target	U251 (GI50,nM)	U87 MG (GI50, nM)
Abiraterone	CYP17	>10,000	>10,000
Alectinib	ALK	>10,000	3427
Afatinib	EGFR/HER2	1733	1413
Anlotinib	VEGFR/PDGFR/FGFR/Kit	>10,000	2513
Apatinib	VEGRF2	>10,000	>10,000
Axitinib	KIT/PDGFR/VEGRFR	>10,000	2319
Brigatinib	ALK	>10,000	6857
Bortezomib	Proteasome	40.61	0.7
Bosutinib	ABL	>10,000	>10,000
Brivanib	BRAF/KIT/PDGFR/RET/VEGFR	>10,000	>10,000
Cabozantinib	FLT3/KIT/MET/RET/VEGFR	>10,000	>10,000
Cediranib	PDGFR/VEGFR	9932	>10,000
Ceritinib	ALK?ROS1	4728	4654
Chidamide	HDAC	9010	3785
Cobimetinib	BRAF	6291	718.6
Dabrafenib	BRAF	>10,000	>10,000
Everolimus	mTOR	>10,000	>10,000
Dacomitnib	EGFR	7057	4849
Dasatinib	ABL	>10,000	>10,000
Dovitinib	FLT3/KIT	7373	524.6
Erlotinib	EGFR	1505	713.4
Larotrectinib	NTRK	>10,000	>10,000
Levatinib	VEGFR2	4627	>10,000
Neratinib	EGFR/HER2	1810	648.4
Nilotinib	ABL	9827	3251
Nintedanib	VEGFR/FGRF/PDGFR	6524	4481
Niraparib	BRCA1/BRCA2	>10,000	617.1
Olaparib	BRCA1/BRCA2	>10,000	>10,000
Osimertinib	EGFR	4041	8861
Palbociclib	CDK4, CDK6	5252	1615
Pamiparib	PARP1/PARP2	>10,000	453.7
Ponatinib	ABL	233.6	106.7
Pyrotinib	EGFR/HER2	2024	>10,000
Regorafenib	KIT/VEGFR/PDGFR/RAF/RET	>10,000	7954
Ribociclib	CDK4/CDK6	>10,000	>10,000
Romidepsin	HDAC	>10,000	>10,000
Rucaparib	BRCA1/BRCA2	>10,000	>10,000
Sirolimus	mTOR	>10,000	>10,000
Sorafenib	KIT/VEGFR/PDGFR/RAF	>10,000	>10,000
Sunitinib	PDGFR/VEGFR/KIT/FLT3/RET	3069	1408
Temsirolimus	mTOR	>10,000	809.7
Thalidomide	CRBN	>10,000	991.7
Tofacitinib	JAK1/JAK3	>10,000	>10,000
Trametinib	BRAF/MEK1/MEK2	7244	3311
Vandetanib	EGFR/RET/VEGFR2	2345	5183
Veliparib	PARP1/PARP2	7561	>10,000
Vemurafenib	BRAF	>10,000	>10,000
Vorinostat	HDAC	935.9	1251
Idelalisib	PI3K	>10,000	>10,000

## Data Availability

The data used to support the findings of this study are available from the corresponding author upon request.

## Supplementary Materials

Supplementary 1 Supplementary Table S1: detailed clinical information for 21 cases of GBM and 15 cases of GSM.

Supplementary 2 Supplementary Table S2: list of aberrant genes in GBM and gliosarcoma.

## References

[1] Walid M. S. (2008). Prognostic factors for long-term survival after glioblastoma. *The Permanente Journal*.

[2] Louis D. N., Perry A., Reifenberger G. (2016). The 2016 World Health Organization classification of tumors of the central nervous system: a summary. *Acta Neuropathologica*.

[3] Galanis E., Buckner J. C., Dinapoli R. P. (1998). Clinical outcome of gliosarcoma compared with glioblastoma multiforme: North Central Cancer Treatment Group results. *Journal of Neurosurgery*.

[4] Oh J. E., Ohta T., Nonoguchi N. (2016). Genetic alterations in gliosarcoma and giant cell glioblastoma. *Brain Pathology*.

[5] Watanabe K., Tachibana O., Sata K., Yonekawa Y., Kleihues P., Ohgaki H. (1996). Overexpression of the EGF receptor and p53 mutations are mutually exclusive in the evolution of primary and secondary glioblastomas. *Brain pathology*.

[6] Biernat W., Huang H., Yokoo H., Kleihues P., Ohgaki H. (2004). Predominant expression of mutant EGFR (EGFRvIII) is rare in primary glioblastomas. *Brain Pathology*.

[7] Reis R. M., Konu-Lebleblicioglu D., Lopes J. M., Kleihues P., Ohgaki H. (2000). Genetic profile of gliosarcomas. *The American Journal of Pathology*.

[8] Padfield E., Ellis H. P., Kurian K. M. (2015). Current therapeutic advances targeting EGFR and EGFRvIII in glioblastoma. *Frontiers in Oncology*.

[9] Lowder L., Hauenstein J., Woods A. (2019). Gliosarcoma: distinct molecular pathways and genomic alterations identified by DNA copy number/SNP microarray analysis. *Journal of Neuro-Oncology*.

[10] Cho S. Y., Park C., Na D. (2017). High prevalence of TP53 mutations is associated with poor survival and an EMT signature in gliosarcoma patients. *Experimental & Molecular Medicine*.

[11] Patrick Y. W., Roger J. P. (2021). The 2021 WHO classification of tumors of the central nervous system: clinical implications. *Neuro-Oncology*.

[12] Ohgaki H., Kleihues P. (2007). Genetic pathways to primary and secondary glioblastoma. *The American Journal of Pathology*.

[13] Johnson M. R., DeClue J. E., Felzmann S. (1994). Neurofibromin can inhibit Ras-dependent growth by a mechanism independent of its GTPase-accelerating function. *Molecular and Cellular Biology*.

[14] Maehama T., Dixon J. E. (1998). The tumor suppressor, PTEN/MMAC1, dephosphorylates the lipid second messenger, phosphatidylinositol 3,4,5-trisphosphate. *The Journal of Biological Chemistry*.

[15] Han S. J., Yang I., Tihan T., Prados M. D., Parsa A. T. (2010). Primary gliosarcoma: key clinical and pathologic distinctions from glioblastoma with implications as a unique oncologic entity. *Journal of Neuro-Oncology*.

[16] Actor B., Cobbers J. M., Buschges R. (2002). Comprehensive analysis of genomic alterations in gliosarcoma and its two tissue components. *Genes, Chromosomes & Cancer*.

[17] Rodger E. J., Chatterjee A., Stockwell P. A., Eccles M. R. (2019). Characterisation of DNA methylation changes in EBF3 and TBC1D16 associated with tumour progression and metastasis in multiple cancer types. *Clinical Epigenetics*.

[18] Chatterjee A., Stockwell P. A., Ahn A., Rodger E. J., Leichter A. L., Eccles M. R. (2017). Genome-wide methylation sequencing of paired primary and metastatic cell lines identifies common DNA methylation changes and a role for EBF3 as a candidate epigenetic driver of melanoma metastasis. *Oncotarget*.

[19] Tao Y. F., Xu L. X., Lu J. (2015). Early B-cell factor 3 (EBF3) is a novel tumor suppressor gene with promoter hypermethylation in pediatric acute myeloid leukemia. *Journal of Experimental & Clinical Cancer Research*.

[20] Miyake Y., Keusch J. J., Wang L. (2016). Structural insights into HDAC6 tubulin deacetylation and its selective inhibition. *Nature Chemical Biology*.

[21] Qian H., Chen Y., Nian Z. (2017). HDAC6-mediated acetylation of lipid droplet-binding protein CIDEC regulates fat-induced lipid storage. *The Journal of Clinical Investigation*.

[22] Jiang H., Xin S., Yan Y., Lun Y., Yang X., Zhang J. (2018). Abnormal acetylation of FOXP3 regulated by SIRT-1 induces Treg functional deficiency in patients with abdominal aortic aneurysms. *Atherosclerosis*.

[23] Hua X., Liu X., Ansari D. O., Lodish H. F. (1998). Synergistic cooperation of TFE3 and smad proteins in TGF-beta-induced transcription of the plasminogen activator inhibitor-1 gene. *Genes & Development*.

[24] Hua X., Miller Z. A., Benchabane H., Wrana J. L., Lodish H. F. (2000). Synergism between transcription factors TFE3 and Smad3 in transforming growth factor-beta-induced transcription of the Smad7 gene. *The Journal of Biological Chemistry*.

[25] Su F., Li X., You K. (2016). Expression of VEGF-D, SMAD4, and SMAD7 and their relationship with lymphangiogenesis and prognosis in colon cancer. *Journal of Gastrointestinal Surgery*.

[26] Michele S., Luca F., Angela C. (2021). Serum extracellular vesicle-derived circHIPK3 and circSMARCA5 are two novel diagnostic biomarkers for glioblastoma multiforme. *Pharmaceuticals*.

[27] Giuseppe B., Lucia S., Davide B. (2021). Diagnostic utility of the immunohistochemical expression of serine and arginine rich splicing factor 1 (SRSF1) in the differential diagnosis of adult gliomas. *Cancer*.

