# Supplementary material for: Gliosarcoma: The Distinct Genomic Alterations Identified by Comprehensive Analysis of Copy Number Variations
Source: Anal Cell Pathol (Amst). 2022 Jun 15;2022:2376288. doi: 10.1155/2022/2376288 (PMC9226978; doi:10.1155/2022/2376288)
Supplement: Supplementary 1 — Supplementary Table S1: detailed clinical information for 21 cases of GBM and 15 cases of GSM. [file 2376288.f1.pdf]

Supplymentary Table S1

GBM

Paitents NO.	WHO Grade	Immunohisto chemistry
1201	IV	GFAP +<br>Olig-2 +<br>ATRX + portion<br>p53 + 1%<br>Neun-/+<br>IDH-1-<br>EMA-<br>Ki-67+ 40%
1202	IV	GFAP+<br>P53+<br>ATRX+<br>Olig-2+ portion<br>NeuN+ neuron<br>CD34+ blood vessels<br>IDH-<br>Ki-67+ 15%
1204	IV	GFAP+<br>Olig-2+<br>IDH-<br>ATRX+<br>Vim+<br>Neun-<br>CD34+<br>p53+<br>Ki-67+ 10%
1205	IV	GFAP+<br>Olig-2+<br>ATRX+ portion<br>p53+ 70%<br>NeuN-<br>IDH-1-<br>Ki-67+ 50%
1206	IV	GFAP+<br>Olig-2+<br>ATRX+ weak<br>p53+ 70%<br>CD34+ blood vessels<br>IDH-1-<br>Neun-<br>Ki-67+ 15%
1208	IV	GFAP+<br>Olig-2+<br>IDH-1-<br>ATRX+<br>CD34+/-<br>NeuN-<br>EMA +<br>P53+ 30%<br>Ki-67+ 40%
1211	IV	GFAP+<br>Olig-2+<br>ATRX+<br>p53+<br>CD34+ blood vessels<br>Neun-<br>IDH-1-<br>Ki-67+ 60%
1212	IV	GFAP+<br>Olig-2+<br>IDH-1-<br>CD34+ blood vessels<br>P53 + portion<br>Vimentin +<br>ATRX + weak<br>Ki-67
1213	IV	GFAP+<br>Olig-2+<br>P53+<br>IDH-1-<br>CK-<br>CD34-<br>ATRX+<br>Ki-67+ 30%
1217	IV	GFAP+<br>Olig-2+<br>IDH+<br>ATRX-<br>p53+<br>Vim+<br>S-100+<br>H3K27M-<br>H3K27me 3-<br>Ki-67+ 30%
1220	IV	GFAP+<br>Olig-2+<br>IDH-<br>ATRX+<br>p53-<br>Vim+<br>EMA-<br>CD34+ blood vessels<br>H3K27M-<br>H3K27me 3-<br>Ki-67+ 40%
1224	IV	GFAP+<br>olig-2+<br>IDH-1-<br>P53+ portion<br>ATRX+<br>Ki-67+ 40%
1225	IV	GFAP+<br>Olig-2+<br>ATRX+ portion<br>IDH-1-<br>H3K27Me 3-<br>H3K27M-<br>NeuN-<br>CD34+ blood vessels<br>Ki-67+ 25%
1226	IV	GFAP+<br>GFAP+<br>Olig-2+<br>IDH-1+<br>CD34+<br>P53+<br>ATRX-<br>Ki-67
1227	IV	GFAP +<br>Olig-2 +<br>ATRX +<br>IDH-1 -<br>P53 -<br>NeuN -<br>CD34+ blood vessels<br>Ki-67+ 30%
1230	NA	GFAP +<br>Olig-2 +<br>ATRX +<br>CD34+ blood vessels<br>p53+ 60%<br>G34V -<br>G34R -<br>IDH-1 -<br>NeuN -<br>Ki-67+ 50%
1233	IV	GFAP+<br>Olig-2+ portion<br>ATRX+<br>IDH-1-<br>CD34+ blood vessels<br>p53+ 30%<br>Ki-67+ 25%
1234	IV	GFAP+<br>Olig-2+<br>IDH-<br>ATRX+<br>Vim+<br>CD34+ blood vessels<br>p53+ 15%<br>Ki-67+ 20%
1235	NA	NA
1236	IV	GFAP+<br>olig-2+<br>ATRX+<br>Vim+<br>p53+<br>CD34+ blood vessels<br>S-100+<br>IDH-<br>EMA-<br>Ki-67+ 20%
1237	IV	GFAP+<br>Olig-2+<br>ATRX+<br>IDH-1+ weak<br>P53+ 8%<br>Vimentin+<br>S100+<br>CD34+ blood vessels<br>NeuN-<br>Ki-67+ 40%

Sarcoma

Paitents NO.	WHO Grade	Component	Immunohisto chemistry
1203	IV		IDH wild type
1215	IV		GFAP+<br>Vim+<br>CD34+ blood vessels<br>EMA-<br>Olig2-<br>P53+ 80%<br>Ki67+ >50%
1216	IV		NA
1218	NA		GFAP+ focal<br>Vim+<br>SMA+<br>S100+ focal<br>CD34-<br>ki67+ 30%
1222	IV		GFAP +<br>EMA +<br>SSTR +<br>CD34 +<br>SMA +<br>Vim +<br>Olig-2 -<br>NueN -<br>IDH-1 -<br>P53 +<br>Ki-67+ 30%
1228	IV		GFAP +/-<br>CD34 +<br>olig-2-<br>Vim +<br>P53 +<br>IDH-1-<br>CK-<br>SMA+ blood vessels<br>Des-/+<br>ki-67+ 50%
1229	IV		GFAP+ gliocyte<br>olig-2+ portion<br>IDH-1-<br>P53+<br>ATRX+<br>NeuN-<br>Ki-67+ 60%
1231	IV		GFAP+<br>olig-2+ portion<br>IDH-1-<br>P53+<br>SMA+ portion<br>Des-<br>S100+<br>CK+ portion<br>ki-67+ 40%
1238	IV		GFAP+ portion<br>Vim+<br>SMA+ blood vessels<br>CD68+<br>ki67+ 60%
1239	IV		GFAP +<br>CD56 +<br>CK +<br>Vim +<br>S100 +<br>CD34 +<br>EMA -<br>NeuN -<br>P53 +<br>Ki-67 + >30%
1240	IV		GFAP-<br>Olig-2-<br>IDH-1-<br>Vim+<br>CD34+ blood vessels<br>P53-<br>Braf-<br>NeuN-<br>CK-<br>EMA-<br>SSTR2α-<br>S-100-<br>SOX-10-<br>TLE-1-<br>Ki-67+ 30%
1246	IV	Glioblastoma components	GFAP+<br>Olig-2 +<br>Vim +<br>S100 +<br>SOX-10 +<br>CD99 +<br>EMA+<br>ATRX +<br>NeuN +<br>CK +<br>Syn +<br>P53+ 70%<br>CD34 -<br>IDH-1 -<br>CgA -<br>Ki-67+ 50%<br>Ag staining (-)
		Sarcoma components	Vim +<br>S100 +<br>ATRX +<br>P53+ 70%<br>EMA +<br>Ki-67+ 15%<br>Ag staining (+)
1248	NA		Vim+<br>SMA-/+<br>GFAP+<br>olig2+ portion<br>Neun-<br>Syn+<br>CK-<br>Ki67+ 50%
1249	IV	Glioblastoma components	GFAP+<br>Vim+<br>S-100+<br>SOX-10+<br>Olig-2+<br>EMA+<br>ATRX+<br>P53+ portion<br>IDH-<br>CD34-<br>MyoD1-<br>Ki-67 + 15-40%<br>Ag staining (-)
		Sarcoma components	Vim+<br>ATRX+<br>P53+ portion<br>GFAP-<br>Olig-2-<br>S-100-<br>SOX-10-<br>EMA-<br>IDH-<br>CD34-<br>MyoD1-<br>Ki-67+ 15%<br>Ag staining (+)
1250	NA		S100+<br>GFAP+<br>Vim+<br>Syn+/-<br>Vim+<br>EMA-<br>PR-<br>Des-<br>P53+<br>Ki67+ 50%

NA, no available
