# Supplementary material for: Gliosarcoma: The Distinct Genomic Alterations Identified by Comprehensive Analysis of Copy Number Variations
Source: Anal Cell Pathol (Amst). 2022 Jun 15;2022:2376288. doi: 10.1155/2022/2376288 (PMC9226978; doi:10.1155/2022/2376288)
Supplement: Supplementary 2 — Supplementary Table S2: list of aberrant genes in GBM and gliosarcoma. [file 2376288.f2.pdf]

	Freq_GBM	GBM	Freq_Sarcoma	Sarcoma	Odds_ratio	Odds
A1BG	0.1428571429	0.1428571429	0.05555555556	0.05555555556	2.333333333	2.571428571
1BG-AS	0.1428571429	0.1428571429	0.05555555556	0.05555555556	2.333333333	2.571428571
A1CF	0.04761904762	0.04761904762	0.05555555556	0.05555555556	0.7	0.8571428571
A2M	0.04761904762	0.04761904762	0.1111111111	0.1111111111	0.325	0.4285714286
2M-AS1	0.04761904762	0.04761904762	0.1111111111	0.1111111111	0.325	0.4285714286
A2ML1	0.04761904762	0.04761904762	0.1111111111	0.1111111111	0.325	0.4285714286
A2MP1	0.04761904762	0.04761904762	0.1111111111	0.1111111111	0.325	0.4285714286
3GALT	0.04761904762	0.04761904762	0.05555555556	0.05555555556	0.7	0.8571428571
A4GALT	0	0	0.1666666667	0.1666666667	0	0
A4GNT	0.1428571429	0.1428571429	0.05555555556	0.05555555556	2.333333333	2.571428571
AA06	0.04761904762	0.04761904762	0	0	inf	4.7619E+31
AAAS	0.04761904762	0.04761904762	0.05555555556	0.05555555556	0.7	0.8571428571
AACS	0.09523809524	0.09523809524	0.05555555556	0.05555555556	1.473684211	1.714285714
AACSP1	0.09523809524	0.09523809524	0.05555555556	0.05555555556	1.473684211	1.714285714
AADAC	0.1904761905	0.1904761905	0.1111111111	0.1111111111	1.529411765	1.714285714
ADACL	0.1904761905	0.1904761905	0.1111111111	0.1111111111	1.529411765	1.714285714
DACL2-	0.1904761905	0.1904761905	0.1111111111	0.1111111111	1.529411765	1.714285714
ADACL	0.04761904762	0.04761904762	0.1111111111	0.1111111111	0.325	0.4285714286
ADACL	0.04761904762	0.04761904762	0.1111111111	0.1111111111	0.325	0.4285714286
ADACP	0.1904761905	0.1904761905	0.1111111111	0.1111111111	1.529411765	1.714285714
AADAT	0	0	0.05555555556	0.05555555556	0	0
AAGAB	0.04761904762	0.04761904762	0.05555555556	0.05555555556	0.7	0.8571428571
AAK1	0	0	0.05555555556	0.05555555556	0	0
AAMP	0	0	0.05555555556	0.05555555556	0	0
AANAT	0.04761904762	0.04761904762	0	0	inf	4.7619E+31
AAR2	0.09523809524	0.09523809524	0.1111111111	0.1111111111	0.6842105263	0.8571428571
AARD	0.1904761905	0.1904761905	0.1666666667	0.1666666667	0.9411764706	1.142857143
AARS	0.1428571429	0.1428571429	0.05555555556	0.05555555556	2.333333333	2.571428571
AARS2	0.04761904762	0.04761904762	0.05555555556	0.05555555556	0.7	0.8571428571
AARSD1	0.04761904762	0.04761904762	0	0	inf	4.7619E+31
AASDH	0.1904761905	0.1904761905	0.05555555556	0.05555555556	3.294117647	3.428571429
AASS	0.04761904762	0.04761904762	0.05555555556	0.05555555556	0.7	0.8571428571
AATBC	0.04761904762	0.04761904762	0.1111111111	0.1111111111	0.325	0.4285714286
AATF	0.04761904762	0.04761904762	0	0	inf	4.7619E+31
AATK	0.04761904762	0.04761904762	0	0	inf	4.7619E+31
BALON	0.09523809524	0.09523809524	0.1111111111	0.1111111111	0.6842105263	0.8571428571
ABAT	0.09523809524	0.09523809524	0	0	inf	9.52381E+31
ABCA1	0.04761904762	0.04761904762	0.05555555556	0.05555555556	0.7	0.8571428571
ABCA10	0	0	0	0		0.00001
BCA11	0.04761904762	0.04761904762	0	0	inf	4.7619E+31
ABCA12	0	0	0.05555555556	0.05555555556	0	0
ABCA13	0.09523809524	0.09523809524	0.2222222222	0.2222222222	0.2894736842	0.4285714286
BCA17	0.09523809524	0.09523809524	0.2222222222	0.2222222222	0.2894736842	0.4285714286
ABCA2	0.1428571429	0.1428571429	0.05555555556	0.05555555556	2.333333333	2.571428571
ABCA3	0.09523809524	0.09523809524	0.2222222222	0.2222222222	0.2894736842	0.4285714286
ABCA4	0.04761904762	0.04761904762	0.05555555556	0.05555555556	0.7	0.8571428571
ABCA5	0	0	0	0		0.00001
ABCA6	0	0	0	0		0.00001
ABCA7	0.09523809524	0.09523809524	0.1111111111	0.1111111111	0.6842105263	0.8571428571

ABCA8	0	0	0	0		0.00001
ABCA9	0	0	0	0		0.00001
BCA9-A	0	0	0	0		0.00001
ABCB1	0.09523809524	0.09523809524	0.05555555556	0.05555555556	1.473684211	1.714285714
ABCB10	0.1428571429	0.1428571429	0	0	inf	1.42857E+32
ABCB11	0.04761904762	0.04761904762	0.05555555556	0.05555555556	0.7	0.8571428571
ABCB4	0.09523809524	0.09523809524	0.05555555556	0.05555555556	1.473684211	1.714285714
ABCB5	0.04761904762	0.04761904762	0.2222222222	0.2222222222	0.1375	0.2142857143
ABCB8	0.09523809524	0.09523809524	0.05555555556	0.05555555556	1.473684211	1.714285714
ABCB9	0.04761904762	0.04761904762	0.1111111111	0.1111111111	0.325	0.4285714286
ABCC1	0.04761904762	0.04761904762	0	0	inf	4.7619E+31
ABCC10	0.04761904762	0.04761904762	0.05555555556	0.05555555556	0.7	0.8571428571
ABCC11	0.04761904762	0.04761904762	0	0	inf	4.7619E+31
ABCC12	0.04761904762	0.04761904762	0	0	inf	4.7619E+31
ABCC13	0.04761904762	0.04761904762	0.1111111111	0.1111111111	0.325	0.4285714286
ABCC2	0.1428571429	0.1428571429	0	0	inf	1.42857E+32
ABCC3	0.09523809524	0.09523809524	0	0	inf	9.52381E+31
ABCC5	0.1904761905	0.1904761905	0.05555555556	0.05555555556	3.294117647	3.428571429
BCC5-A	0.1428571429	0.1428571429	0.05555555556	0.05555555556	2.333333333	2.571428571
ABCC6	0.04761904762	0.04761904762	0	0	inf	4.7619E+31
BCC6P	0.09523809524	0.09523809524	0	0	inf	9.52381E+31
BCC6P	0.04761904762	0.04761904762	0	0	inf	4.7619E+31
ABCC9	0.04761904762	0.04761904762	0.05555555556	0.05555555556	0.7	0.8571428571
ABCD2	0.04761904762	0.04761904762	0.05555555556	0.05555555556	0.7	0.8571428571
ABCD3	0.04761904762	0.04761904762	0.05555555556	0.05555555556	0.7	0.8571428571
ABCD4	0.04761904762	0.04761904762	0	0	inf	4.7619E+31
ABCE1	0	0	0.05555555556	0.05555555556	0	0
ABCF2	0.09523809524	0.09523809524	0.05555555556	0.05555555556	1.473684211	1.714285714
ABCF3	0.1428571429	0.1428571429	0.05555555556	0.05555555556	2.333333333	2.571428571
ABCG1	0.04761904762	0.04761904762	0.1111111111	0.1111111111	0.325	0.4285714286
ABCG2	0	0	0.05555555556	0.05555555556	0	0
ABCG5	0	0	0.05555555556	0.05555555556	0	0
ABCG8	0	0	0.05555555556	0.05555555556	0	0
ABHD10	0.09523809524	0.09523809524	0.05555555556	0.05555555556	1.473684211	1.714285714
ABHD11	0.1428571429	0.1428571429	0.1111111111	0.1111111111	1.083333333	1.285714286
HD11-A	0.1428571429	0.1428571429	0.1111111111	0.1111111111	1.083333333	1.285714286
ABHD12	0.1904761905	0.1904761905	0.05555555556	0.05555555556	3.294117647	3.428571429
BHD14	0.04761904762	0.04761904762	0	0	inf	4.7619E+31
D14A-A	0.04761904762	0.04761904762	0	0	inf	4.7619E+31
BHD14	0.04761904762	0.04761904762	0	0	inf	4.7619E+31
ABHD15	0	0	0.05555555556	0.05555555556	0	0
BHD16	0.04761904762	0.04761904762	0	0	inf	4.7619E+31
BHD16	0.1428571429	0.1428571429	0.2222222222	0.2222222222	0.4583333333	0.6428571429
BHD17	0.09523809524	0.09523809524	0.1111111111	0.1111111111	0.6842105263	0.8571428571
BHD17	0	0	0.05555555556	0.05555555556	0	0
BHD17	0.04761904762	0.04761904762	0.05555555556	0.05555555556	0.7	0.8571428571
ABHD18	0	0	0.05555555556	0.05555555556	0	0
ABHD2	0.04761904762	0.04761904762	0.05555555556	0.05555555556	0.7	0.8571428571
ABHD3	0.04761904762	0.04761904762	0.1111111111	0.1111111111	0.325	0.4285714286
ABHD4	0	0	0.05555555556	0.05555555556	0	0
ABHD5	0.04761904762	0.04761904762	0	0	inf	4.7619E+31

ABHD6	0.04761904762	0.04761904762	0	0	inf	4.7619E+31
ABHD8	0.1428571429	0.1428571429	0.1111111111	0.1111111111	1.083333333	1.285714286
ABI1	0.09523809524	0.09523809524	0.1111111111	0.1111111111	0.6842105263	0.8571428571
ABI2	0.09523809524	0.09523809524	0.05555555556	0.05555555556	1.473684211	1.714285714
ABI3	0.1428571429	0.1428571429	0	0	inf	1.42857E+32
ABI3BP	0.09523809524	0.09523809524	0.05555555556	0.05555555556	1.473684211	1.714285714
BITRAI	0.04761904762	0.04761904762	0.05555555556	0.05555555556	0.7	0.8571428571
ABL1	0.09523809524	0.09523809524	0.05555555556	0.05555555556	1.473684211	1.714285714
ABL2	0.1428571429	0.1428571429	0	0	inf	1.42857E+32
ABLIM1	0.1904761905	0.1904761905	0.05555555556	0.05555555556	3.294117647	3.428571429
ABO	0.04761904762	0.04761904762	0	0	inf	4.7619E+31
ABR	0	0	0.1111111111	0.1111111111	0	0
ABRA	0.1904761905	0.1904761905	0.1111111111	0.1111111111	1.529411765	1.714285714
BRAXAS	0	0	0.05555555556	0.05555555556	0	0
BRAXAS	0.2380952381	0.2380952381	0.05555555556	0.05555555556	4.375	4.285714286
ACAA1	0.04761904762	0.04761904762	0	0	inf	4.7619E+31
ACAA2	0.04761904762	0.04761904762	0	0	inf	4.7619E+31
ACACA	0.04761904762	0.04761904762	0	0	inf	4.7619E+31
ACACB	0	0	0.05555555556	0.05555555556	0	0
ACAD10	0	0	0.1111111111	0.1111111111	0	0
ACAD11	0.1428571429	0.1428571429	0.05555555556	0.05555555556	2.333333333	2.571428571
ACAD9	0.09523809524	0.09523809524	0.05555555556	0.05555555556	1.473684211	1.714285714
ACADL	0	0	0.05555555556	0.05555555556	0	0
ACADM	0.04761904762	0.04761904762	0.05555555556	0.05555555556	0.7	0.8571428571
ACADS	0	0	0.1111111111	0.1111111111	0	0
ACADSB	0.2380952381	0.2380952381	0.05555555556	0.05555555556	4.375	4.285714286
ACADVL	0	0	0.05555555556	0.05555555556	0	0
ACAN	0.04761904762	0.04761904762	0.05555555556	0.05555555556	0.7	0.8571428571
ACAP1	0	0	0.05555555556	0.05555555556	0	0
ACAP2	0.1428571429	0.1428571429	0.05555555556	0.05555555556	2.333333333	2.571428571
ACAP3	0	0	0.1111111111	0.1111111111	0	0
ACBD3	0.1428571429	0.1428571429	0.05555555556	0.05555555556	2.333333333	2.571428571
CBD3-AS	0.1428571429	0.1428571429	0.05555555556	0.05555555556	2.333333333	2.571428571
ACBD4	0.1428571429	0.1428571429	0	0	inf	1.42857E+32
ACBD5	0.09523809524	0.09523809524	0.1111111111	0.1111111111	0.6842105263	0.8571428571
ACBD6	0.1428571429	0.1428571429	0	0	inf	1.42857E+32
ACBD7	0.09523809524	0.09523809524	0.1111111111	0.1111111111	0.6842105263	0.8571428571
7-DCLR	0.09523809524	0.09523809524	0.1111111111	0.1111111111	0.6842105263	0.8571428571
ACD	0.1428571429	0.1428571429	0.05555555556	0.05555555556	2.333333333	2.571428571
ACE	0.04761904762	0.04761904762	0	0	inf	4.7619E+31
ACER1	0.1428571429	0.1428571429	0.05555555556	0.05555555556	2.333333333	2.571428571
ACER2	0.04761904762	0.04761904762	0.05555555556	0.05555555556	0.7	0.8571428571
ACHE	0.04761904762	0.04761904762	0.05555555556	0.05555555556	0.7	0.8571428571
ACIN1	0	0	0.05555555556	0.05555555556	0	0
ACKR1	0.1428571429	0.1428571429	0	0	inf	1.42857E+32
ACKR2	0.04761904762	0.04761904762	0	0	inf	4.7619E+31
ACKR3	0	0	0.05555555556	0.05555555556	0	0
ACKR4	0.1428571429	0.1428571429	0.05555555556	0.05555555556	2.333333333	2.571428571
ACLY	0.09523809524	0.09523809524	0	0	inf	9.52381E+31
ACMSD	0.04761904762	0.04761904762	0.05555555556	0.05555555556	0.7	0.8571428571
ACO1	0.09523809524	0.09523809524	0.05555555556	0.05555555556	1.473684211	1.714285714

ACO2	0	0	0.1111111111	0.1111111111	0	0
ACOD1	0	0	0.05555555556	0.05555555556	0	0
ACOT1	0.09523809524	0.09523809524	0	0	inf	9.52381E+31
ACOT11	0.04761904762	0.04761904762	0.05555555556	0.05555555556	0.7	0.8571428571
ACOT12	0.04761904762	0.04761904762	0	0	inf	4.7619E+31
ACOT13	0	0	0.05555555556	0.05555555556	0	0
ACOT2	0.09523809524	0.09523809524	0	0	inf	9.52381E+31
ACOT4	0.09523809524	0.09523809524	0	0	inf	9.52381E+31
ACOT6	0.09523809524	0.09523809524	0	0	inf	9.52381E+31
ACOT7	0	0	0.05555555556	0.05555555556	0	0
ACOT8	0.1428571429	0.1428571429	0.1666666667	0.1666666667	0.6666666667	0.8571428571
ACOX1	0.04761904762	0.04761904762	0	0	inf	4.7619E+31
ACOX2	0.04761904762	0.04761904762	0	0	inf	4.7619E+31
ACOXL	0	0	0.05555555556	0.05555555556	0	0
COXL-A	0	0	0.05555555556	0.05555555556	0	0
ACP1	0	0	0.05555555556	0.05555555556	0	0
ACP2	0	0	0.05555555556	0.05555555556	0	0
ACP4	0.09523809524	0.09523809524	0.05555555556	0.05555555556	1.473684211	1.714285714
ACP5	0.1428571429	0.1428571429	0.1111111111	0.1111111111	1.083333333	1.285714286
ACP6	0.1428571429	0.1428571429	0.05555555556	0.05555555556	2.333333333	2.571428571
ACP7	0.04761904762	0.04761904762	0.05555555556	0.05555555556	0.7	0.8571428571
ACPP	0.1428571429	0.1428571429	0.05555555556	0.05555555556	2.333333333	2.571428571
ACR	0	0	0.1111111111	0.1111111111	0	0
ACRBP	0.04761904762	0.04761904762	0.1111111111	0.1111111111	0.325	0.4285714286
ACSBG1	0.04761904762	0.04761904762	0.05555555556	0.05555555556	0.7	0.8571428571
ACSBG2	0.1428571429	0.1428571429	0.05555555556	0.05555555556	2.333333333	2.571428571
ACSF2	0.09523809524	0.09523809524	0	0	inf	9.52381E+31
ACSF3	0.09523809524	0.09523809524	0	0	inf	9.52381E+31
ACSL1	0	0	0.05555555556	0.05555555556	0	0
ACSL3	0	0	0.05555555556	0.05555555556	0	0
ACSL5	0.1428571429	0.1428571429	0.05555555556	0.05555555556	2.333333333	2.571428571
ACSL6	0.04761904762	0.04761904762	0	0	inf	4.7619E+31
ACSM1	0.04761904762	0.04761904762	0	0	inf	4.7619E+31
ACSM2A	0.04761904762	0.04761904762	0	0	inf	4.7619E+31
ACSM2B	0.04761904762	0.04761904762	0	0	inf	4.7619E+31
ACSM3	0.04761904762	0.04761904762	0	0	inf	4.7619E+31
ACSM4	0.04761904762	0.04761904762	0.1111111111	0.1111111111	0.325	0.4285714286
ACSM5	0.04761904762	0.04761904762	0	0	inf	4.7619E+31
ACSM6	0.1428571429	0.1428571429	0	0	inf	1.42857E+32
ACSS1	0.1904761905	0.1904761905	0.05555555556	0.05555555556	3.294117647	3.428571429
ACSS2	0.09523809524	0.09523809524	0.1111111111	0.1111111111	0.6842105263	0.8571428571
ACSS3	0.04761904762	0.04761904762	0.05555555556	0.05555555556	0.7	0.8571428571
ACTA1	0.1428571429	0.1428571429	0	0	inf	1.42857E+32
ACTA2	0.1428571429	0.1428571429	0.1666666667	0.1666666667	0.6666666667	0.8571428571
CTA2-AS	0.1428571429	0.1428571429	0.1666666667	0.1666666667	0.6666666667	0.8571428571
ACTB	0.04761904762	0.04761904762	0.1666666667	0.1666666667	0.2	0.2857142857
ACTBL2	0.04761904762	0.04761904762	0	0	inf	4.7619E+31
ACTG1	0.04761904762	0.04761904762	0	0	inf	4.7619E+31
CTG1P1	0.04761904762	0.04761904762	0.05555555556	0.05555555556	0.7	0.8571428571
CTG1P2	0.04761904762	0.04761904762	0.1111111111	0.1111111111	0.325	0.4285714286
ACTG1P	0.04761904762	0.04761904762	0.05555555556	0.05555555556	0.7	0.8571428571

**ACTG2**	0	0	0.05555555556	0.05555555556	0	0
**ACTL10**	0.09523809524	0.09523809524	0.1111111111	0.1111111111	0.6842105263	0.8571428571
**ACTL6A**	0.1904761905	0.1904761905	0.05555555556	0.05555555556	3.294117647	3.428571429
**ACTL6B**	0.04761904762	0.04761904762	0.05555555556	0.05555555556	0.7	0.8571428571
**ACTL7A**	0.04761904762	0.04761904762	0.05555555556	0.05555555556	0.7	0.8571428571
**ACTL7B**	0.04761904762	0.04761904762	0.05555555556	0.05555555556	0.7	0.8571428571
**ACTL8**	0.04761904762	0.04761904762	0.05555555556	0.05555555556	0.7	0.8571428571
**ACTL9**	0.1428571429	0.1428571429	0.05555555556	0.05555555556	2.333333333	2.571428571
**ACTN2**	0.1428571429	0.1428571429	0	0	inf	1.42857E+32
**ACTN4**	0.04761904762	0.04761904762	0.05555555556	0.05555555556	0.7	0.8571428571
**ACTR10**	0.04761904762	0.04761904762	0	0	inf	4.7619E+31
**ACTR1A**	0.1428571429	0.1428571429	0.05555555556	0.05555555556	2.333333333	2.571428571
**ACTR1B**	0	0	0.1111111111	0.1111111111	0	0
**ACTR2**	0	0	0.05555555556	0.05555555556	0	0
**ACTR3**	0	0	0.05555555556	0.05555555556	0	0
**CTR3-AS**	0	0	0.05555555556	0.05555555556	0	0
**ACTR3B**	0.09523809524	0.09523809524	0.05555555556	0.05555555556	1.473684211	1.714285714
**CTR3BP**	0.04761904762	0.04761904762	0.05555555556	0.05555555556	0.7	0.8571428571
**CTR3BP**	0.09523809524	0.09523809524	0.1111111111	0.1111111111	0.6842105263	0.8571428571
**ACTR3C**	0.09523809524	0.09523809524	0.05555555556	0.05555555556	1.473684211	1.714285714
**ACTR5**	0.1428571429	0.1428571429	0.2222222222	0.2222222222	0.4583333333	0.6428571429
**ACTR6**	0.04761904762	0.04761904762	0.05555555556	0.05555555556	0.7	0.8571428571
**ACTR8**	0.04761904762	0.04761904762	0	0	inf	4.7619E+31
**ACTRT2**	0	0	0.05555555556	0.05555555556	0	0
**ACTRT3**	0.1904761905	0.1904761905	0.05555555556	0.05555555556	3.294117647	3.428571429
**ACVR1**	0	0	0.05555555556	0.05555555556	0	0
**ACVR1B**	0.04761904762	0.04761904762	0.05555555556	0.05555555556	0.7	0.8571428571
**ACVR1C**	0	0	0.05555555556	0.05555555556	0	0
**ACVR2A**	0	0	0.05555555556	0.05555555556	0	0
**ACVR2B**	0.04761904762	0.04761904762	0	0	inf	4.7619E+31
**VR2B-A**	0.04761904762	0.04761904762	0	0	inf	4.7619E+31
**ACVRL1**	0.04761904762	0.04761904762	0.05555555556	0.05555555556	0.7	0.8571428571
**ACY1**	0.04761904762	0.04761904762	0	0	inf	4.7619E+31
**ACYP2**	0.04761904762	0.04761904762	0.05555555556	0.05555555556	0.7	0.8571428571
**ADA**	0.1428571429	0.1428571429	0.1666666667	0.1666666667	0.6666666667	0.8571428571
**ADA2**	0.04761904762	0.04761904762	0	0	inf	4.7619E+31
**ADAD1**	0	0	0.05555555556	0.05555555556	0	0
**ADAD2**	0.04761904762	0.04761904762	0	0	inf	4.7619E+31
**ADAM10**	0.04761904762	0.04761904762	0.05555555556	0.05555555556	0.7	0.8571428571
**ADAM11**	0.09523809524	0.09523809524	0	0	inf	9.52381E+31
**ADAM12**	0.2380952381	0.2380952381	0.05555555556	0.05555555556	4.375	4.285714286
**ADAM15**	0.1904761905	0.1904761905	0.1111111111	0.1111111111	1.529411765	1.714285714
**ADAM17**	0	0	0.05555555556	0.05555555556	0	0
**ADAM18**	0.09523809524	0.09523809524	0.1111111111	0.1111111111	0.6842105263	0.8571428571
**ADAM19**	0.04761904762	0.04761904762	0.05555555556	0.05555555556	0.7	0.8571428571
**ADAM1A**	0	0	0.1111111111	0.1111111111	0	0
**ADAM2**	0.09523809524	0.09523809524	0.1111111111	0.1111111111	0.6842105263	0.8571428571
**ADAM22**	0.09523809524	0.09523809524	0.1111111111	0.1111111111	0.6842105263	0.8571428571
**ADAM23**	0	0	0.05555555556	0.05555555556	0	0
**ADAM28**	0.04761904762	0.04761904762	0.1111111111	0.1111111111	0.325	0.4285714286
**ADAM29**	0	0	0.05555555556	0.05555555556	0	0

ADAM30	0.04761904762	0.04761904762	0.05555555556	0.05555555556	0.7	0.8571428571
ADAM32	0.09523809524	0.09523809524	0.1111111111	0.1111111111	0.6842105263	0.8571428571
ADAM33	0.1904761905	0.1904761905	0.05555555556	0.05555555556	3.294117647	3.428571429
ADAM3A	0.09523809524	0.09523809524	0.1111111111	0.1111111111	0.6842105263	0.8571428571
ADAM3B	0.04761904762	0.04761904762	0	0	inf	4.7619E+31
ADAM5	0.09523809524	0.09523809524	0.1111111111	0.1111111111	0.6842105263	0.8571428571
ADAM6	0.04761904762	0.04761904762	0	0	inf	4.7619E+31
ADAM7	0.04761904762	0.04761904762	0.1111111111	0.1111111111	0.325	0.4285714286
ADAM8	0.1904761905	0.1904761905	0	0	inf	1.90476E+32
ADAM9	0.09523809524	0.09523809524	0.1111111111	0.1111111111	0.6842105263	0.8571428571
DAMDEC	0.04761904762	0.04761904762	0.1111111111	0.1111111111	0.325	0.4285714286
DAMTS	0.04761904762	0.04761904762	0.1111111111	0.1111111111	0.325	0.4285714286
DAMTS	0.1428571429	0.1428571429	0.05555555556	0.05555555556	2.333333333	2.571428571
DAMTS	0.04761904762	0.04761904762	0.1111111111	0.1111111111	0.325	0.4285714286
DAMTS	0.04761904762	0.04761904762	0	0	inf	4.7619E+31
DAMTS	0.09523809524	0.09523809524	0	0	inf	9.52381E+31
DAMTS	0	0	0.1111111111	0.1111111111	0	0
DAMTS	0.04761904762	0.04761904762	0.05555555556	0.05555555556	0.7	0.8571428571
DAMTS	0.09523809524	0.09523809524	0	0	inf	9.52381E+31
DAMTS	0.04761904762	0.04761904762	0	0	inf	4.7619E+31
MTS19-	0.04761904762	0.04761904762	0	0	inf	4.7619E+31
DAMTS	0.09523809524	0.09523809524	0.05555555556	0.05555555556	1.473684211	1.714285714
DAMTS2	0.04761904762	0.04761904762	0.05555555556	0.05555555556	0.7	0.8571428571
DAMTS	0.09523809524	0.09523809524	0.05555555556	0.05555555556	1.473684211	1.714285714
DAMTS	0.1428571429	0.1428571429	0	0	inf	1.42857E+32
DAMTS	0.04761904762	0.04761904762	0.1111111111	0.1111111111	0.325	0.4285714286
DAMTS	0.04761904762	0.04761904762	0	0	inf	4.7619E+31
DAMTS	0.04761904762	0.04761904762	0.05555555556	0.05555555556	0.7	0.8571428571
DAMTS7	0.04761904762	0.04761904762	0.05555555556	0.05555555556	0.7	0.8571428571
DAMTS	0.04761904762	0.04761904762	0	0	inf	4.7619E+31
MTS9-	0.04761904762	0.04761904762	0	0	inf	4.7619E+31
MTS9-	0.04761904762	0.04761904762	0	0	inf	4.7619E+31
DAMTSI	0.04761904762	0.04761904762	0.05555555556	0.05555555556	0.7	0.8571428571
DAMTSI	0.04761904762	0.04761904762	0	0	inf	4.7619E+31
DAMTSI	0.04761904762	0.04761904762	0.05555555556	0.05555555556	0.7	0.8571428571
DAMTSI	0.1904761905	0.1904761905	0.05555555556	0.05555555556	3.294117647	3.428571429
MTSL4-	0.1904761905	0.1904761905	0.05555555556	0.05555555556	3.294117647	3.428571429
DAMTSI	0.09523809524	0.09523809524	0.1111111111	0.1111111111	0.6842105263	0.8571428571
ADAP1	0.04761904762	0.04761904762	0.1666666667	0.1666666667	0.2	0.2857142857
ADAP2	0	0	0	0		0.00001
ADAR	0.1904761905	0.1904761905	0.1111111111	0.1111111111	1.529411765	1.714285714
ADARB1	0.04761904762	0.04761904762	0.1111111111	0.1111111111	0.325	0.4285714286
ADARB2	0.04761904762	0.04761904762	0.1111111111	0.1111111111	0.325	0.4285714286
ARB2-A	0.04761904762	0.04761904762	0.1111111111	0.1111111111	0.325	0.4285714286
ADAT1	0.04761904762	0.04761904762	0	0	inf	4.7619E+31
ADAT3	0.09523809524	0.09523809524	0.1111111111	0.1111111111	0.6842105263	0.8571428571
ADCK2	0.04761904762	0.04761904762	0.05555555556	0.05555555556	0.7	0.8571428571
ADCK5	0.04761904762	0.04761904762	0.1666666667	0.1666666667	0.2	0.2857142857
ADCY1	0.04761904762	0.04761904762	0.2222222222	0.2222222222	0.1375	0.2142857143
ADCY10	0.1428571429	0.1428571429	0	0	inf	1.42857E+32
DCY10P	0	0	0.05555555556	0.05555555556	0	0

**ADCY2**	0	0	0.05555555556	0.05555555556	0	0
**ADCY5**	0.04761904762	0.04761904762	0.05555555556	0.05555555556	0.7	0.8571428571
**ADCY6**	0.04761904762	0.04761904762	0.05555555556	0.05555555556	0.7	0.8571428571
**ADCY7**	0.04761904762	0.04761904762	0	0	inf	4.7619E+31
**ADCY8**	0.04761904762	0.04761904762	0.1111111111	0.1111111111	0.325	0.4285714286
**ADCY9**	0.04761904762	0.04761904762	0.1111111111	0.1111111111	0.325	0.4285714286
**DCYAP**	0.09523809524	0.09523809524	0	0	inf	9.52381E+31
**CYAP1**	0.04761904762	0.04761904762	0.1111111111	0.1111111111	0.325	0.4285714286
**ADD1**	0.04761904762	0.04761904762	0	0	inf	4.7619E+31
**ADD2**	0	0	0.05555555556	0.05555555556	0	0
**ADD3**	0.1428571429	0.1428571429	0.05555555556	0.05555555556	2.333333333	2.571428571
**DD3-AS**	0.1428571429	0.1428571429	0.05555555556	0.05555555556	2.333333333	2.571428571
**DGRA1**	0.1904761905	0.1904761905	0	0	inf	1.90476E+32
**GRA1-A**	0.1904761905	0.1904761905	0	0	inf	1.90476E+32
**DGRA2**	0.04761904762	0.04761904762	0.1111111111	0.1111111111	0.325	0.4285714286
**DGRB1**	0.04761904762	0.04761904762	0.1666666667	0.1666666667	0.2	0.2857142857
**DGRB2**	0.04761904762	0.04761904762	0.1111111111	0.1111111111	0.325	0.4285714286
**DGRD1**	0.09523809524	0.09523809524	0.05555555556	0.05555555556	1.473684211	1.714285714
**GRD1-A**	0.09523809524	0.09523809524	0.05555555556	0.05555555556	1.473684211	1.714285714
**DGRE1**	0.1428571429	0.1428571429	0.05555555556	0.05555555556	2.333333333	2.571428571
**DGRE2**	0.1428571429	0.1428571429	0.1111111111	0.1111111111	1.083333333	1.285714286
**DGRE3**	0.1428571429	0.1428571429	0.1111111111	0.1111111111	1.083333333	1.285714286
**DGRE4**	0.1428571429	0.1428571429	0.05555555556	0.05555555556	2.333333333	2.571428571
**DGRE5**	0.1428571429	0.1428571429	0.1111111111	0.1111111111	1.083333333	1.285714286
**DGRF1**	0.04761904762	0.04761904762	0.05555555556	0.05555555556	0.7	0.8571428571
**DGRF2**	0.04761904762	0.04761904762	0.05555555556	0.05555555556	0.7	0.8571428571
**DGRF4**	0.04761904762	0.04761904762	0.05555555556	0.05555555556	0.7	0.8571428571
**DGRF5**	0.04761904762	0.04761904762	0.05555555556	0.05555555556	0.7	0.8571428571
**DGRG1**	0.04761904762	0.04761904762	0	0	inf	4.7619E+31
**DGRG3**	0.04761904762	0.04761904762	0	0	inf	4.7619E+31
**DGRG5**	0.04761904762	0.04761904762	0	0	inf	4.7619E+31
**DGRG7**	0.09523809524	0.09523809524	0.05555555556	0.05555555556	1.473684211	1.714285714
**DGRL1**	0.1428571429	0.1428571429	0.1111111111	0.1111111111	1.083333333	1.285714286
**DGRL2**	0.04761904762	0.04761904762	0.05555555556	0.05555555556	0.7	0.8571428571
**DGRL3**	0.04761904762	0.04761904762	0.05555555556	0.05555555556	0.7	0.8571428571
**GRL3-A**	0.04761904762	0.04761904762	0.05555555556	0.05555555556	0.7	0.8571428571
**DGRL4**	0.04761904762	0.04761904762	0.05555555556	0.05555555556	0.7	0.8571428571
**DGRV1**	0.04761904762	0.04761904762	0	0	inf	4.7619E+31
**ADH1A**	0	0	0.05555555556	0.05555555556	0	0
**ADH1B**	0	0	0.05555555556	0.05555555556	0	0
**ADH1C**	0	0	0.05555555556	0.05555555556	0	0
**ADH4**	0	0	0.05555555556	0.05555555556	0	0
**ADH5**	0	0	0.05555555556	0.05555555556	0	0
**ADH6**	0	0	0.05555555556	0.05555555556	0	0
**ADH7**	0	0	0.05555555556	0.05555555556	0	0
**DHFE1**	0.1428571429	0.1428571429	0.1666666667	0.1666666667	0.6666666667	0.8571428571
**ADI1**	0	0	0.05555555556	0.05555555556	0	0
**ADIG**	0.1428571429	0.1428571429	0.1666666667	0.1666666667	0.6666666667	0.8571428571
**DIPOQ**	0.09523809524	0.09523809524	0.05555555556	0.05555555556	1.473684211	1.714285714
**IPOQ-A**	0.09523809524	0.09523809524	0.05555555556	0.05555555556	1.473684211	1.714285714
**DIPOR**	0.1428571429	0.1428571429	0	0	inf	1.42857E+32

ADIPOR	0.04761904762	0.04761904762	0.1666666667	0.1666666667	0.2	0.2857142857
ADIRF	0.1428571429	0.1428571429	0.05555555556	0.05555555556	2.333333333	2.571428571
ADK	0.1904761905	0.1904761905	0.05555555556	0.05555555556	3.294117647	3.428571429
ADM2	0	0	0.1111111111	0.1111111111	0	0
ADM5	0.09523809524	0.09523809524	0.1111111111	0.1111111111	0.6842105263	0.8571428571
ADNP	0.09523809524	0.09523809524	0.1666666667	0.1666666667	0.4210526316	0.5714285714
DNP-AS	0.09523809524	0.09523809524	0.1666666667	0.1666666667	0.4210526316	0.5714285714
ADNP2	0.04761904762	0.04761904762	0.05555555556	0.05555555556	0.7	0.8571428571
ADO	0.04761904762	0.04761904762	0.1666666667	0.1666666667	0.2	0.2857142857
ADORA1	0.1428571429	0.1428571429	0	0	inf	1.42857E+32
DORA2	0.04761904762	0.04761904762	0.1111111111	0.1111111111	0.325	0.4285714286
DRA2A-	0.04761904762	0.04761904762	0.1111111111	0.1111111111	0.325	0.4285714286
DORA2	0.04761904762	0.04761904762	0.05555555556	0.05555555556	0.7	0.8571428571
ADORA3	0.04761904762	0.04761904762	0	0	inf	4.7619E+31
ADPGK	0.04761904762	0.04761904762	0.05555555556	0.05555555556	0.7	0.8571428571
DPGK-A	0.04761904762	0.04761904762	0.05555555556	0.05555555556	0.7	0.8571428571
ADPRH	0.09523809524	0.09523809524	0.05555555556	0.05555555556	1.473684211	1.714285714
DPRHL	0.04761904762	0.04761904762	0.05555555556	0.05555555556	0.7	0.8571428571
ADPRM	0.04761904762	0.04761904762	0.05555555556	0.05555555556	0.7	0.8571428571
ADRA1A	0.04761904762	0.04761904762	0.1111111111	0.1111111111	0.325	0.4285714286
ADRA1B	0.04761904762	0.04761904762	0.05555555556	0.05555555556	0.7	0.8571428571
ADRA1D	0.1904761905	0.1904761905	0.05555555556	0.05555555556	3.294117647	3.428571429
ADRA2A	0.1428571429	0.1428571429	0.05555555556	0.05555555556	2.333333333	2.571428571
ADRA2B	0	0	0.1111111111	0.1111111111	0	0
ADRA2C	0.04761904762	0.04761904762	0	0	inf	4.7619E+31
ADRB1	0.1428571429	0.1428571429	0.05555555556	0.05555555556	2.333333333	2.571428571
ADRB3	0.09523809524	0.09523809524	0.1111111111	0.1111111111	0.6842105263	0.8571428571
ADRM1	0.1428571429	0.1428571429	0.2222222222	0.2222222222	0.4583333333	0.6428571429
ADSL	0	0	0.1111111111	0.1111111111	0	0
ADSS	0.1428571429	0.1428571429	0.05555555556	0.05555555556	2.333333333	2.571428571
ADTRP	0.04761904762	0.04761904762	0	0	inf	4.7619E+31
AEBP1	0.04761904762	0.04761904762	0.1666666667	0.1666666667	0.2	0.2857142857
AEBP2	0.04761904762	0.04761904762	0.1111111111	0.1111111111	0.325	0.4285714286
AEN	0.04761904762	0.04761904762	0.05555555556	0.05555555556	0.7	0.8571428571
AFAP1L	0.1904761905	0.1904761905	0.05555555556	0.05555555556	3.294117647	3.428571429
AFF1	0	0	0.05555555556	0.05555555556	0	0
FF1-AS	0	0	0.05555555556	0.05555555556	0	0
AFF3	0	0	0.05555555556	0.05555555556	0	0
AFF4	0.04761904762	0.04761904762	0	0	inf	4.7619E+31
AFG1L	0	0	0	0		0.00001
AFG3L1	0.09523809524	0.09523809524	0	0	inf	9.52381E+31
AFG3L2	0.09523809524	0.09523809524	0.05555555556	0.05555555556	1.473684211	1.714285714
AFM	0.09523809524	0.09523809524	0.05555555556	0.05555555556	1.473684211	1.714285714
AFMID	0.04761904762	0.04761904762	0	0	inf	4.7619E+31
AFP	0.09523809524	0.09523809524	0.05555555556	0.05555555556	1.473684211	1.714285714
AFTPH	0	0	0.05555555556	0.05555555556	0	0
AGA	0	0	0.05555555556	0.05555555556	0	0
AGAP1	0	0	0.05555555556	0.05555555556	0	0
GAP1-IT	0	0	0.05555555556	0.05555555556	0	0
AGAP10	0.1428571429	0.1428571429	0.05555555556	0.05555555556	2.333333333	2.571428571
AGAP11	0.1428571429	0.1428571429	0.05555555556	0.05555555556	2.333333333	2.571428571

AGAP12	0.1428571429	0.1428571429	0.05555555556	0.05555555556	2.333333333	2.571428571
AGAP2	0.1428571429	0.1428571429	0.05555555556	0.05555555556	2.333333333	2.571428571
GAP2-A	0.1428571429	0.1428571429	0.05555555556	0.05555555556	2.333333333	2.571428571
AGAP3	0.09523809524	0.09523809524	0.05555555556	0.05555555556	1.473684211	1.714285714
AGAP4	0.1428571429	0.1428571429	0.05555555556	0.05555555556	2.333333333	2.571428571
AGAP5	0.1904761905	0.1904761905	0.05555555556	0.05555555556	3.294117647	3.428571429
AGAP6	0.04761904762	0.04761904762	0.05555555556	0.05555555556	0.7	0.8571428571
AGAP7P	0.04761904762	0.04761904762	0.05555555556	0.05555555556	0.7	0.8571428571
AGAP9	0.1428571429	0.1428571429	0.05555555556	0.05555555556	2.333333333	2.571428571
AGBL1	0.09523809524	0.09523809524	0.05555555556	0.05555555556	1.473684211	1.714285714
GBL1-A	0.04761904762	0.04761904762	0.05555555556	0.05555555556	0.7	0.8571428571
AGBL2	0	0	0.05555555556	0.05555555556	0	0
AGBL3	0.04761904762	0.04761904762	0.05555555556	0.05555555556	0.7	0.8571428571
AGBL4	0	0	0.05555555556	0.05555555556	0	0
GBL4-IT	0	0	0	0		0.00001
AGER	0.04761904762	0.04761904762	0	0	inf	4.7619E+31
AGFG1	0	0	0.05555555556	0.05555555556	0	0
AGFG2	0.04761904762	0.04761904762	0.05555555556	0.05555555556	0.7	0.8571428571
AGGF1	0.04761904762	0.04761904762	0	0	inf	4.7619E+31
AGK	0.04761904762	0.04761904762	0.1111111111	0.1111111111	0.325	0.4285714286
AGMAT	0.04761904762	0.04761904762	0.1111111111	0.1111111111	0.325	0.4285714286
AGMO	0.04761904762	0.04761904762	0.2222222222	0.2222222222	0.1375	0.2142857143
AGO1	0.04761904762	0.04761904762	0.05555555556	0.05555555556	0.7	0.8571428571
AGO2	0.04761904762	0.04761904762	0.2222222222	0.2222222222	0.1375	0.2142857143
AGO3	0.04761904762	0.04761904762	0.05555555556	0.05555555556	0.7	0.8571428571
AGO4	0.04761904762	0.04761904762	0.05555555556	0.05555555556	0.7	0.8571428571
AGPAT1	0.04761904762	0.04761904762	0	0	inf	4.7619E+31
AGPAT2	0.1428571429	0.1428571429	0.05555555556	0.05555555556	2.333333333	2.571428571
AGPAT3	0.04761904762	0.04761904762	0.1111111111	0.1111111111	0.325	0.4285714286
AGPAT5	0.09523809524	0.09523809524	0.1111111111	0.1111111111	0.6842105263	0.8571428571
AGPS	0	0	0.1111111111	0.1111111111	0	0
AGR2	0.04761904762	0.04761904762	0.2222222222	0.2222222222	0.1375	0.2142857143
AGR3	0.04761904762	0.04761904762	0.2222222222	0.2222222222	0.1375	0.2142857143
AGRN	0	0	0.1111111111	0.1111111111	0	0
AGRP	0.1428571429	0.1428571429	0.05555555556	0.05555555556	2.333333333	2.571428571
AGT	0.1428571429	0.1428571429	0	0	inf	1.42857E+32
GTPBP	0	0	0.05555555556	0.05555555556	0	0
AGTR1	0.1428571429	0.1428571429	0.1111111111	0.1111111111	1.083333333	1.285714286
AGTRAP	0.04761904762	0.04761904762	0.1111111111	0.1111111111	0.325	0.4285714286
AGXT	0	0	0.05555555556	0.05555555556	0	0
AGXT2	0.04761904762	0.04761904762	0.05555555556	0.05555555556	0.7	0.8571428571
AHCTF1	0.1428571429	0.1428571429	0.05555555556	0.05555555556	2.333333333	2.571428571
HCTF1P	0	0	0.05555555556	0.05555555556	0	0
AHCY	0.09523809524	0.09523809524	0.1111111111	0.1111111111	0.6842105263	0.8571428571
AHCYL1	0.04761904762	0.04761904762	0.05555555556	0.05555555556	0.7	0.8571428571
AHCYL2	0.04761904762	0.04761904762	0.05555555556	0.05555555556	0.7	0.8571428571
AHDC1	0.04761904762	0.04761904762	0.1111111111	0.1111111111	0.325	0.4285714286
AHR	0.04761904762	0.04761904762	0.2222222222	0.2222222222	0.1375	0.2142857143
AHRR	0	0	0.05555555556	0.05555555556	0	0
AHSA2P	0.09523809524	0.09523809524	0.05555555556	0.05555555556	1.473684211	1.714285714
AHSG	0.09523809524	0.09523809524	0.05555555556	0.05555555556	1.473684211	1.714285714

**AHSP**	0.04761904762	0.04761904762	0	0	inf	4.7619E+31
**AICDA**	0.04761904762	0.04761904762	0.1111111111	0.1111111111	0.325	0.4285714286
**AIDA**	0.1428571429	0.1428571429	0.05555555556	0.05555555556	2.333333333	2.571428571
**AIF1**	0.04761904762	0.04761904762	0	0	inf	4.7619E+31
**AIF1L**	0.09523809524	0.09523809524	0.05555555556	0.05555555556	1.473684211	1.714285714
**AIFM2**	0.09523809524	0.09523809524	0	0	inf	9.52381E+31
**AIFM3**	0.04761904762	0.04761904762	0.05555555556	0.05555555556	0.7	0.8571428571
**AIM2**	0.1428571429	0.1428571429	0	0	inf	1.42857E+32
**AIMP1**	0	0	0.05555555556	0.05555555556	0	0
**AIMP2**	0.04761904762	0.04761904762	0.1666666667	0.1666666667	0.2	0.2857142857
**AIPL1**	0	0	0.05555555556	0.05555555556	0	0
**AIRE**	0.04761904762	0.04761904762	0.1111111111	0.1111111111	0.325	0.4285714286
**AJAP1**	0	0	0.05555555556	0.05555555556	0	0
**AJM1**	0.1428571429	0.1428571429	0.05555555556	0.05555555556	2.333333333	2.571428571
**AJUBA**	0	0	0.05555555556	0.05555555556	0	0
**AK1**	0.09523809524	0.09523809524	0.05555555556	0.05555555556	1.473684211	1.714285714
**AK2**	0.04761904762	0.04761904762	0.05555555556	0.05555555556	0.7	0.8571428571
**AK3**	0.04761904762	0.04761904762	0.05555555556	0.05555555556	0.7	0.8571428571
**AK4**	0.04761904762	0.04761904762	0.05555555556	0.05555555556	0.7	0.8571428571
**AK5**	0.04761904762	0.04761904762	0.05555555556	0.05555555556	0.7	0.8571428571
**AK6**	0.04761904762	0.04761904762	0.05555555556	0.05555555556	0.7	0.8571428571
**AK8**	0.04761904762	0.04761904762	0	0	inf	4.7619E+31
**AK9**	0	0	0	0		0.00001
**AKAIN1**	0.09523809524	0.09523809524	0	0	inf	9.52381E+31
**AKAP1**	0.09523809524	0.09523809524	0	0	inf	9.52381E+31
**AKAP10**	0.09523809524	0.09523809524	0.05555555556	0.05555555556	1.473684211	1.714285714
**AKAP13**	0.09523809524	0.09523809524	0.05555555556	0.05555555556	1.473684211	1.714285714
**AKAP2**	0.04761904762	0.04761904762	0.05555555556	0.05555555556	0.7	0.8571428571
**AKAP3**	0.04761904762	0.04761904762	0.1111111111	0.1111111111	0.325	0.4285714286
**AKAP5**	0.09523809524	0.09523809524	0	0	inf	9.52381E+31
**AKAP8**	0.1428571429	0.1428571429	0.1111111111	0.1111111111	1.083333333	1.285714286
**AKAP8L**	0.1428571429	0.1428571429	0.1111111111	0.1111111111	1.083333333	1.285714286
**AKAP9**	0.04761904762	0.04761904762	0.1111111111	0.1111111111	0.325	0.4285714286
**AKIRIN[illegible]**	0.04761904762	0.04761904762	0.05555555556	0.05555555556	0.7	0.8571428571
**AKNA**	0.04761904762	0.04761904762	0.05555555556	0.05555555556	0.7	0.8571428571
**AKNAD1**	0.04761904762	0.04761904762	0.05555555556	0.05555555556	0.7	0.8571428571
**AKR1A1**	0.04761904762	0.04761904762	0.05555555556	0.05555555556	0.7	0.8571428571
**AKR1B1**	0.04761904762	0.04761904762	0.05555555556	0.05555555556	0.7	0.8571428571
**AKR1B10**	0.04761904762	0.04761904762	0.05555555556	0.05555555556	0.7	0.8571428571
**AKR1B1[illegible]**	0.04761904762	0.04761904762	0.05555555556	0.05555555556	0.7	0.8571428571
**AKR1C1**	0	0	0.1666666667	0.1666666667	0	0
**AKR1C2**	0	0	0.1666666667	0.1666666667	0	0
**AKR1C3**	0	0	0.1666666667	0.1666666667	0	0
**AKR1C4**	0	0	0.1666666667	0.1666666667	0	0
**AKR1C6[illegible]**	0.04761904762	0.04761904762	0.1666666667	0.1666666667	0.2	0.2857142857
**AKR1C8[illegible]**	0	0	0.1666666667	0.1666666667	0	0
**AKR1D1**	0.04761904762	0.04761904762	0.05555555556	0.05555555556	0.7	0.8571428571
**AKR1E2**	0.04761904762	0.04761904762	0.1666666667	0.1666666667	0.2	0.2857142857
**AKR7A2**	0.04761904762	0.04761904762	0.1111111111	0.1111111111	0.325	0.4285714286
**AKR7A2[illegible]**	0.04761904762	0.04761904762	0.05555555556	0.05555555556	0.7	0.8571428571
**AKR7A3**	0.04761904762	0.04761904762	0.1111111111	0.1111111111	0.325	0.4285714286

AKR7L	0.04761904762	0.04761904762	0.1111111111	0.1111111111	0.325	0.4285714286
AKT1S1	0.09523809524	0.09523809524	0.1111111111	0.1111111111	0.6842105263	0.8571428571
AKT2	0.04761904762	0.04761904762	0.05555555556	0.05555555556	0.7	0.8571428571
AKT3	0.1428571429	0.1428571429	0.05555555556	0.05555555556	2.333333333	2.571428571
AKTIP	0.04761904762	0.04761904762	0.05555555556	0.05555555556	0.7	0.8571428571
ALAD	0.04761904762	0.04761904762	0.05555555556	0.05555555556	0.7	0.8571428571
ALAS1	0.04761904762	0.04761904762	0	0	inf	4.7619E+31
ALB	0.09523809524	0.09523809524	0.05555555556	0.05555555556	1.473684211	1.714285714
ALCAM	0.09523809524	0.09523809524	0.05555555556	0.05555555556	1.473684211	1.714285714
LDH16A	0.09523809524	0.09523809524	0.1111111111	0.1111111111	0.6842105263	0.8571428571
LDH18A	0.1428571429	0.1428571429	0	0	inf	1.42857E+32
LDH1A	0	0	0.05555555556	0.05555555556	0	0
LDH1A	0.04761904762	0.04761904762	0.05555555556	0.05555555556	0.7	0.8571428571
LDH1A	0.04761904762	0.04761904762	0.05555555556	0.05555555556	0.7	0.8571428571
LDH1B	0.04761904762	0.04761904762	0.05555555556	0.05555555556	0.7	0.8571428571
LDH1L	0.04761904762	0.04761904762	0.05555555556	0.05555555556	0.7	0.8571428571
DH1L1-A	0.04761904762	0.04761904762	0.05555555556	0.05555555556	0.7	0.8571428571
DH1L1-A	0.04761904762	0.04761904762	0.05555555556	0.05555555556	0.7	0.8571428571
LDH1L	0.04761904762	0.04761904762	0.05555555556	0.05555555556	0.7	0.8571428571
ALDH2	0	0	0.1111111111	0.1111111111	0	0
LDH3A	0.09523809524	0.09523809524	0.05555555556	0.05555555556	1.473684211	1.714285714
LDH3A	0.09523809524	0.09523809524	0.05555555556	0.05555555556	1.473684211	1.714285714
LDH4A	0.04761904762	0.04761904762	0.1111111111	0.1111111111	0.325	0.4285714286
LDH5A	0	0	0.05555555556	0.05555555556	0	0
LDH6A	0.09523809524	0.09523809524	0	0	inf	9.52381E+31
LDH7A	0.04761904762	0.04761904762	0	0	inf	4.7619E+31
LDH9A	0.1428571429	0.1428571429	0	0	inf	1.42857E+32
ALDOA	0.04761904762	0.04761904762	0	0	inf	4.7619E+31
ALDOB	0.04761904762	0.04761904762	0	0	inf	4.7619E+31
ALDOC	0.04761904762	0.04761904762	0.05555555556	0.05555555556	0.7	0.8571428571
ALG1	0.04761904762	0.04761904762	0	0	inf	4.7619E+31
ALG10	0.04761904762	0.04761904762	0.05555555556	0.05555555556	0.7	0.8571428571
ALG10B	0.04761904762	0.04761904762	0	0	inf	4.7619E+31
ALG12	0	0	0.1111111111	0.1111111111	0	0
ALG14	0.04761904762	0.04761904762	0.05555555556	0.05555555556	0.7	0.8571428571
ALG1L	0.04761904762	0.04761904762	0.05555555556	0.05555555556	0.7	0.8571428571
ALG1L2	0.09523809524	0.09523809524	0.05555555556	0.05555555556	1.473684211	1.714285714
ALG2	0.04761904762	0.04761904762	0	0	inf	4.7619E+31
ALG3	0.1428571429	0.1428571429	0.05555555556	0.05555555556	2.333333333	2.571428571
ALG6	0.04761904762	0.04761904762	0.05555555556	0.05555555556	0.7	0.8571428571
ALK	0	0	0.05555555556	0.05555555556	0	0
ALKAL1	0.09523809524	0.09523809524	0.1111111111	0.1111111111	0.6842105263	0.8571428571
ALKAL2	0	0	0.05555555556	0.05555555556	0	0
LKBH2	0	0	0.05555555556	0.05555555556	0	0
LKBH4	0.04761904762	0.04761904762	0.05555555556	0.05555555556	0.7	0.8571428571
LKBH6	0.04761904762	0.04761904762	0.05555555556	0.05555555556	0.7	0.8571428571
LKBH7	0.1428571429	0.1428571429	0.05555555556	0.05555555556	2.333333333	2.571428571
ALLC	0	0	0.05555555556	0.05555555556	0	0
ALMS1	0	0	0.05555555556	0.05555555556	0	0
LMS1-IT	0	0	0.05555555556	0.05555555556	0	0
LMS1P	0	0	0.05555555556	0.05555555556	0	0

ALOX12	0	0	0.05555555556	0.05555555556	0	0
.OX12-A	0	0	0.05555555556	0.05555555556	0	0
ALOX12I	0	0	0.05555555556	0.05555555556	0	0
LOX12P	0	0	0.05555555556	0.05555555556	0	0
ALOX15	0	0	0.1111111111	0.1111111111	0	0
ALOX15I	0	0	0.05555555556	0.05555555556	0	0
LOX15P	0	0	0.05555555556	0.05555555556	0	0
ALOX5	0.1428571429	0.1428571429	0.05555555556	0.05555555556	2.333333333	2.571428571
ALOXE3	0	0	0.05555555556	0.05555555556	0	0
ALPG	0	0	0.05555555556	0.05555555556	0	0
ALPI	0	0	0.05555555556	0.05555555556	0	0
ALPK1	0	0	0.05555555556	0.05555555556	0	0
ALPK2	0.09523809524	0.09523809524	0	0	inf	9.52381E+31
ALPK3	0.04761904762	0.04761904762	0.05555555556	0.05555555556	0.7	0.8571428571
ALPL	0.04761904762	0.04761904762	0.1111111111	0.1111111111	0.325	0.4285714286
ALPP	0	0	0.05555555556	0.05555555556	0	0
ALS2	0	0	0.05555555556	0.05555555556	0	0
ALX1	0.04761904762	0.04761904762	0.05555555556	0.05555555556	0.7	0.8571428571
ALX3	0.04761904762	0.04761904762	0.05555555556	0.05555555556	0.7	0.8571428571
ALYREF	0.04761904762	0.04761904762	0	0	inf	4.7619E+31
AMACR	0.04761904762	0.04761904762	0.1111111111	0.1111111111	0.325	0.4285714286
AMBN	0.09523809524	0.09523809524	0.05555555556	0.05555555556	1.473684211	1.714285714
AMBP	0.04761904762	0.04761904762	0.05555555556	0.05555555556	0.7	0.8571428571
AMBRA1	0	0	0.05555555556	0.05555555556	0	0
AMD1	0	0	0	0		0.00001
AMDHD	0.04761904762	0.04761904762	0.05555555556	0.05555555556	0.7	0.8571428571
AMDHD	0.04761904762	0.04761904762	0.1111111111	0.1111111111	0.325	0.4285714286
AMER3	0	0	0.05555555556	0.05555555556	0	0
AMFR	0.04761904762	0.04761904762	0	0	inf	4.7619E+31
AMH	0.09523809524	0.09523809524	0.1111111111	0.1111111111	0.6842105263	0.8571428571
AMHR2	0.04761904762	0.04761904762	0.05555555556	0.05555555556	0.7	0.8571428571
AMIGO1	0.04761904762	0.04761904762	0.05555555556	0.05555555556	0.7	0.8571428571
AMIGO2	0.04761904762	0.04761904762	0.05555555556	0.05555555556	0.7	0.8571428571
AMIGO3	0.09523809524	0.09523809524	0	0	inf	9.52381E+31
MECR	0	0	0.05555555556	0.05555555556	0	0
AMN1	0.1428571429	0.1428571429	0.1111111111	0.1111111111	1.083333333	1.285714286
AMOTL2	0.1428571429	0.1428571429	0.05555555556	0.05555555556	2.333333333	2.571428571
AMPD1	0.04761904762	0.04761904762	0.05555555556	0.05555555556	0.7	0.8571428571
AMPD2	0.04761904762	0.04761904762	0.05555555556	0.05555555556	0.7	0.8571428571
AMPH	0.04761904762	0.04761904762	0.2222222222	0.2222222222	0.1375	0.2142857143
AMT	0.1428571429	0.1428571429	0	0	inf	1.42857E+32
AMTN	0.04761904762	0.04761904762	0.05555555556	0.05555555556	0.7	0.8571428571
AMY1A	0.04761904762	0.04761904762	0.05555555556	0.05555555556	0.7	0.8571428571
AMY1B	0.04761904762	0.04761904762	0.05555555556	0.05555555556	0.7	0.8571428571
AMY1C	0.04761904762	0.04761904762	0.05555555556	0.05555555556	0.7	0.8571428571
AMY2A	0.04761904762	0.04761904762	0.05555555556	0.05555555556	0.7	0.8571428571
AMY2B	0.04761904762	0.04761904762	0.05555555556	0.05555555556	0.7	0.8571428571
AMZ1	0.04761904762	0.04761904762	0.1666666667	0.1666666667	0.2	0.2857142857
AMZ2	0	0	0	0		0.00001
AMZ2P1	0.04761904762	0.04761904762	0	0	inf	4.7619E+31
ANAPC1	0	0	0.05555555556	0.05555555556	0	0

ANAPC1	0	0	0.05555555556	0.05555555556	0	0
ANAPC1	0.04761904762	0.04761904762	0	0	inf	4.7619E+31
ANAPC1	0.1428571429	0.1428571429	0.05555555556	0.05555555556	2.333333333	2.571428571
ANAPC1	0.1904761905	0.1904761905	0.05555555556	0.05555555556	3.294117647	3.428571429
NAPC1E	0	0	0.05555555556	0.05555555556	0	0
ANAPC2	0.1428571429	0.1428571429	0.05555555556	0.05555555556	2.333333333	2.571428571
ANAPC5	0.04761904762	0.04761904762	0.1111111111	0.1111111111	0.325	0.4285714286
ANAPC7	0	0	0.1111111111	0.1111111111	0	0
ANGEL2	0.1428571429	0.1428571429	0	0	inf	1.42857E+32
ANGPT1	0.1904761905	0.1904761905	0.1111111111	0.1111111111	1.529411765	1.714285714
ANGPT2	0.09523809524	0.09523809524	0.1111111111	0.1111111111	0.6842105263	0.8571428571
ANGPT4	0.1904761905	0.1904761905	0.1111111111	0.1111111111	1.529411765	1.714285714
ANGPTL	0.1428571429	0.1428571429	0	0	inf	1.42857E+32
ANGPTL	0.09523809524	0.09523809524	0.05555555556	0.05555555556	1.473684211	1.714285714
ANGPTL	0.04761904762	0.04761904762	0.05555555556	0.05555555556	0.7	0.8571428571
ANGPTL	0.1428571429	0.1428571429	0.05555555556	0.05555555556	2.333333333	2.571428571
ANGPTL	0.1428571429	0.1428571429	0.1111111111	0.1111111111	1.083333333	1.285714286
ANGPTL	0.04761904762	0.04761904762	0.1111111111	0.1111111111	0.325	0.4285714286
ANGPTL	0.1428571429	0.1428571429	0.1111111111	0.1111111111	1.083333333	1.285714286
ANHX	0.09523809524	0.09523809524	0.05555555556	0.05555555556	1.473684211	1.714285714
ANK1	0.09523809524	0.09523809524	0.1666666667	0.1666666667	0.4210526316	0.5714285714
ANK2	0	0	0.05555555556	0.05555555556	0	0
ANK3	0.04761904762	0.04761904762	0.1111111111	0.1111111111	0.325	0.4285714286
ANKAR	0	0	0.05555555556	0.05555555556	0	0
NKDD1	0.04761904762	0.04761904762	0.05555555556	0.05555555556	0.7	0.8571428571
NKDD1	0.04761904762	0.04761904762	0	0	inf	4.7619E+31
ANKEF1	0.1904761905	0.1904761905	0.1111111111	0.1111111111	1.529411765	1.714285714
ANKFN1	0.09523809524	0.09523809524	0	0	inf	9.52381E+31
ANKFY1	0	0	0.1111111111	0.1111111111	0	0
ANKH	0.04761904762	0.04761904762	0.05555555556	0.05555555556	0.7	0.8571428571
ANKHD1	0.04761904762	0.04761904762	0	0	inf	4.7619E+31
D1-EIF4	0.04761904762	0.04761904762	0	0	inf	4.7619E+31
ANKIB1	0.04761904762	0.04761904762	0.1111111111	0.1111111111	0.325	0.4285714286
ANKLE1	0.1428571429	0.1428571429	0.1111111111	0.1111111111	1.083333333	1.285714286
ANKLE2	0.09523809524	0.09523809524	0.05555555556	0.05555555556	1.473684211	1.714285714
ANKMY	0.04761904762	0.04761904762	0.05555555556	0.05555555556	0.7	0.8571428571
ANKMY	0.04761904762	0.04761904762	0.2222222222	0.2222222222	0.1375	0.2142857143
ANKRA2	0	0	0.05555555556	0.05555555556	0	0
ANKRD1	0.1428571429	0.1428571429	0.1111111111	0.1111111111	1.083333333	1.285714286
ANKRD1	0.09523809524	0.09523809524	0	0	inf	9.52381E+31
ANKRD1	0.09523809524	0.09523809524	0	0	inf	9.52381E+31
NKRD13	0	0	0.1111111111	0.1111111111	0	0
NKRD13	0	0	0.05555555556	0.05555555556	0	0
NKRD13	0.04761904762	0.04761904762	0.05555555556	0.05555555556	0.7	0.8571428571
ANKRD1	0.09523809524	0.09523809524	0.1111111111	0.1111111111	0.6842105263	0.8571428571
ANKRD1	0.1428571429	0.1428571429	0.05555555556	0.05555555556	2.333333333	2.571428571
NKRD18	0.04761904762	0.04761904762	0.05555555556	0.05555555556	0.7	0.8571428571
NKRD18	0.09523809524	0.09523809524	0.05555555556	0.05555555556	1.473684211	1.714285714
NKRD18	0.04761904762	0.04761904762	0	0	inf	4.7619E+31
NKRD18	0.1428571429	0.1428571429	0.05555555556	0.05555555556	2.333333333	2.571428571
NKRD19	0.04761904762	0.04761904762	0.05555555556	0.05555555556	0.7	0.8571428571

ANKRD2	0.1428571429	0.1428571429	0	0	inf	1.42857E+32
NKRD20	0.1904761905	0.1904761905	0.2222222222	0.2222222222	0.6470588235	0.8571428571
KRD20A	0.04761904762	0.04761904762	0	0	inf	4.7619E+31
KRD20A	0.09523809524	0.09523809524	0.05555555556	0.05555555556	1.473684211	1.714285714
NKRD20	0.1904761905	0.1904761905	0.05555555556	0.05555555556	3.294117647	3.428571429
NKRD20	0.1904761905	0.1904761905	0.2222222222	0.2222222222	0.6470588235	0.8571428571
NKRD20	0.09523809524	0.09523809524	0.2222222222	0.2222222222	0.2894736842	0.4285714286
A4-ANK	0.09523809524	0.09523809524	0.2222222222	0.2222222222	0.2894736842	0.4285714286
KRD20A	0.09523809524	0.09523809524	0.05555555556	0.05555555556	1.473684211	1.714285714
KRD20A	0	0	0.1111111111	0.1111111111	0	0
KRD20A	0.04761904762	0.04761904762	0	0	inf	4.7619E+31
ANKRD2	0.1428571429	0.1428571429	0.1666666667	0.1666666667	0.6666666667	0.8571428571
ANKRD2	0	0	0.1111111111	0.1111111111	0	0
ANKRD2	0.09523809524	0.09523809524	0.05555555556	0.05555555556	1.473684211	1.714285714
ANKRD2	0.09523809524	0.09523809524	0.1111111111	0.1111111111	0.6842105263	0.8571428571
NKRD26	0.04761904762	0.04761904762	0	0	inf	4.7619E+31
NKRD26	0.04761904762	0.04761904762	0	0	inf	4.7619E+31
ANKRD2	0.04761904762	0.04761904762	0.05555555556	0.05555555556	0.7	0.8571428571
ANKRD2	0.04761904762	0.04761904762	0	0	inf	4.7619E+31
ANKRD2	0.09523809524	0.09523809524	0.05555555556	0.05555555556	1.473684211	1.714285714
NKRD30	0.09523809524	0.09523809524	0.1111111111	0.1111111111	0.6842105263	0.8571428571
NKRD30	0.09523809524	0.09523809524	0.05555555556	0.05555555556	1.473684211	1.714285714
NKRD30	0	0	0.05555555556	0.05555555556	0	0
KRD30E	0.04761904762	0.04761904762	0	0	inf	4.7619E+31
KRD30E	0.1428571429	0.1428571429	0.05555555556	0.05555555556	2.333333333	2.571428571
ANKRD3	0.04761904762	0.04761904762	0	0	inf	4.7619E+31
ANKRD3	0.04761904762	0.04761904762	0.05555555556	0.05555555556	0.7	0.8571428571
NKRD33	0.04761904762	0.04761904762	0.05555555556	0.05555555556	0.7	0.8571428571
NKRD34	0.04761904762	0.04761904762	0	0	inf	4.7619E+31
NKRD34	0.04761904762	0.04761904762	0.05555555556	0.05555555556	0.7	0.8571428571
KRD34C-	0.04761904762	0.04761904762	0.05555555556	0.05555555556	0.7	0.8571428571
ANKRD3	0.1428571429	0.1428571429	0.05555555556	0.05555555556	2.333333333	2.571428571
ANKRD3	0	0	0.1111111111	0.1111111111	0	0
NKRD36	0	0	0.1111111111	0.1111111111	0	0
KRD36E	0.1428571429	0.1428571429	0	0	inf	1.42857E+32
KRD36E	0	0	0.05555555556	0.05555555556	0	0
NKRD36	0	0	0.1111111111	0.1111111111	0	0
ANKRD3	0	0	0.05555555556	0.05555555556	0	0
ANKRD3	0	0	0.1111111111	0.1111111111	0	0
ANKRD4	0.09523809524	0.09523809524	0	0	inf	9.52381E+31
NKRD40	0.09523809524	0.09523809524	0	0	inf	9.52381E+31
ANKRD4	0	0	0.05555555556	0.05555555556	0	0
KRD44-	0	0	0.05555555556	0.05555555556	0	0
ANKRD4	0.1428571429	0.1428571429	0	0	inf	1.42857E+32
ANKRD4	0.1904761905	0.1904761905	0.1111111111	0.1111111111	1.529411765	1.714285714
ANKRD5	0	0	0.05555555556	0.05555555556	0	0
ANKRD5	0.09523809524	0.09523809524	0.05555555556	0.05555555556	1.473684211	1.714285714
ANKRD5	0	0	0.05555555556	0.05555555556	0	0
ANKRD5	0	0	0.1111111111	0.1111111111	0	0
ANKRD5	0.04761904762	0.04761904762	0.05555555556	0.05555555556	0.7	0.8571428571
ANKRD6	0	0	0	0		0.00001

NKRD6	0.1428571429	0.1428571429	0.2222222222	0.2222222222	0.4583333333	0.6428571429
NKRD6	0.04761904762	0.04761904762	0.1666666667	0.1666666667	0.2	0.2857142857
NKRD6	0.09523809524	0.09523809524	0.05555555556	0.05555555556	1.473684211	1.714285714
62P1-PA	0.04761904762	0.04761904762	0	0	inf	4.7619E+31
NKRD6	0	0	0.1111111111	0.1111111111	0	0
NKRD6	0.04761904762	0.04761904762	0.05555555556	0.05555555556	0.7	0.8571428571
NKRD7	0.09523809524	0.09523809524	0.05555555556	0.05555555556	1.473684211	1.714285714
ANKS1A	0.04761904762	0.04761904762	0	0	inf	4.7619E+31
ANKS1B	0.04761904762	0.04761904762	0.05555555556	0.05555555556	0.7	0.8571428571
ANKS3	0.04761904762	0.04761904762	0	0	inf	4.7619E+31
ANKS4B	0.04761904762	0.04761904762	0	0	inf	4.7619E+31
ANKS6	0.04761904762	0.04761904762	0	0	inf	4.7619E+31
ANKUB1	0.1904761905	0.1904761905	0.1111111111	0.1111111111	1.529411765	1.714285714
ANLN	0.04761904762	0.04761904762	0.2222222222	0.2222222222	0.1375	0.2142857143
ANO10	0.04761904762	0.04761904762	0	0	inf	4.7619E+31
ANO2	0.04761904762	0.04761904762	0.1111111111	0.1111111111	0.325	0.4285714286
ANO4	0.04761904762	0.04761904762	0.05555555556	0.05555555556	0.7	0.8571428571
ANO6	0.04761904762	0.04761904762	0.05555555556	0.05555555556	0.7	0.8571428571
ANO7	0	0	0.05555555556	0.05555555556	0	0
ANO8	0.1428571429	0.1428571429	0.1111111111	0.1111111111	1.083333333	1.285714286
ANO9	0.04761904762	0.04761904762	0	0	inf	4.7619E+31
ANP32A	0.04761904762	0.04761904762	0.05555555556	0.05555555556	0.7	0.8571428571
NP32A-IT	0.04761904762	0.04761904762	0.05555555556	0.05555555556	0.7	0.8571428571
NP32AP	0	0	0	0		0.00001
ANP32B	0.04761904762	0.04761904762	0	0	inf	4.7619E+31
ANP32C	0	0	0.05555555556	0.05555555556	0	0
ANP32D	0.04761904762	0.04761904762	0.05555555556	0.05555555556	0.7	0.8571428571
ANP32E	0.1904761905	0.1904761905	0.05555555556	0.05555555556	3.294117647	3.428571429
ANPEP	0.04761904762	0.04761904762	0.05555555556	0.05555555556	0.7	0.8571428571
ANTXR1	0	0	0.05555555556	0.05555555556	0	0
ANTXR2	0.04761904762	0.04761904762	0.05555555556	0.05555555556	0.7	0.8571428571
ANTXRI	0.1428571429	0.1428571429	0.05555555556	0.05555555556	2.333333333	2.571428571
NTXRLI	0.1428571429	0.1428571429	0.05555555556	0.05555555556	2.333333333	2.571428571
ANXA1	0	0	0.05555555556	0.05555555556	0	0
ANXA10	0	0	0.05555555556	0.05555555556	0	0
ANXA11	0.1428571429	0.1428571429	0	0	inf	1.42857E+32
ANXA13	0.1428571429	0.1428571429	0.1111111111	0.1111111111	1.083333333	1.285714286
ANXA2	0.04761904762	0.04761904762	0.05555555556	0.05555555556	0.7	0.8571428571
NXA2P	0	0	0.05555555556	0.05555555556	0	0
NXA2P	0.09523809524	0.09523809524	0.05555555556	0.05555555556	1.473684211	1.714285714
NXA2P	0.1428571429	0.1428571429	0.05555555556	0.05555555556	2.333333333	2.571428571
ANXA2F	0.04761904762	0.04761904762	0.05555555556	0.05555555556	0.7	0.8571428571
ANXA3	0.04761904762	0.04761904762	0.05555555556	0.05555555556	0.7	0.8571428571
ANXA4	0	0	0.05555555556	0.05555555556	0	0
ANXA5	0	0	0.05555555556	0.05555555556	0	0
ANXA7	0.1904761905	0.1904761905	0.05555555556	0.05555555556	3.294117647	3.428571429
ANXA8	0.1428571429	0.1428571429	0.05555555556	0.05555555556	2.333333333	2.571428571
NXA8L	0.1428571429	0.1428571429	0.05555555556	0.05555555556	2.333333333	2.571428571
ANXA9	0.1904761905	0.1904761905	0.05555555556	0.05555555556	3.294117647	3.428571429
AOAH	0.04761904762	0.04761904762	0.2222222222	0.2222222222	0.1375	0.2142857143
OAH-IT	0.04761904762	0.04761904762	0.2222222222	0.2222222222	0.1375	0.2142857143

**AOC1**	0.09523809524	0.09523809524	0.05555555556	0.05555555556	1.473684211	1.714285714
**AOC2**	0.04761904762	0.04761904762	0	0	inf	4.7619E+31
**AOC3**	0.04761904762	0.04761904762	0	0	inf	4.7619E+31
**AOC4P**	0.04761904762	0.04761904762	0	0	inf	4.7619E+31
**AOPEP**	0.04761904762	0.04761904762	0.05555555556	0.05555555556	0.7	0.8571428571
**AOX1**	0	0	0.05555555556	0.05555555556	0	0
**X3P-AO**	0	0	0.05555555556	0.05555555556	0	0
**AP1AR**	0	0	0.05555555556	0.05555555556	0	0
**AP1B1**	0.04761904762	0.04761904762	0.1111111111	0.1111111111	0.325	0.4285714286
**AP1B1P1**	0.04761904762	0.04761904762	0.1111111111	0.1111111111	0.325	0.4285714286
**AP1G1**	0.04761904762	0.04761904762	0	0	inf	4.7619E+31
**AP1M1**	0.1428571429	0.1428571429	0.1111111111	0.1111111111	1.083333333	1.285714286
**AP1M2**	0.1428571429	0.1428571429	0.1111111111	0.1111111111	1.083333333	1.285714286
**AP1S1**	0.04761904762	0.04761904762	0.05555555556	0.05555555556	0.7	0.8571428571
**AP1S3**	0	0	0.05555555556	0.05555555556	0	0
**AP2A1**	0.09523809524	0.09523809524	0.1111111111	0.1111111111	0.6842105263	0.8571428571
**AP2A2**	0.04761904762	0.04761904762	0	0	inf	4.7619E+31
**AP2B1**	0.09523809524	0.09523809524	0	0	inf	9.52381E+31
**AP2M1**	0.1428571429	0.1428571429	0.05555555556	0.05555555556	2.333333333	2.571428571
**AP2S1**	0.09523809524	0.09523809524	0.1111111111	0.1111111111	0.6842105263	0.8571428571
**AP3B1**	0.04761904762	0.04761904762	0	0	inf	4.7619E+31
**AP3B2**	0.04761904762	0.04761904762	0.05555555556	0.05555555556	0.7	0.8571428571
**AP3D1**	0.09523809524	0.09523809524	0.1111111111	0.1111111111	0.6842105263	0.8571428571
**AP3M1**	0.1904761905	0.1904761905	0.05555555556	0.05555555556	3.294117647	3.428571429
**AP3M2**	0.09523809524	0.09523809524	0.1666666667	0.1666666667	0.4210526316	0.5714285714
**AP3S1**	0.04761904762	0.04761904762	0	0	inf	4.7619E+31
**AP3S2**	0.04761904762	0.04761904762	0.05555555556	0.05555555556	0.7	0.8571428571
**AP4B1**	0.04761904762	0.04761904762	0.05555555556	0.05555555556	0.7	0.8571428571
**P4B1-AS**	0.04761904762	0.04761904762	0.05555555556	0.05555555556	0.7	0.8571428571
**AP4E1**	0.1428571429	0.1428571429	0.05555555556	0.05555555556	2.333333333	2.571428571
**AP4M1**	0.04761904762	0.04761904762	0.05555555556	0.05555555556	0.7	0.8571428571
**AP4S1**	0	0	0	0		0.00001
**AP5M1**	0.04761904762	0.04761904762	0	0	inf	4.7619E+31
**AP5S1**	0.1904761905	0.1904761905	0.05555555556	0.05555555556	3.294117647	3.428571429
**AP5Z1**	0.04761904762	0.04761904762	0.1666666667	0.1666666667	0.2	0.2857142857
**APAF1**	0.04761904762	0.04761904762	0.05555555556	0.05555555556	0.7	0.8571428571
**APBA1**	0	0	0.05555555556	0.05555555556	0	0
**APBA3**	0.09523809524	0.09523809524	0.05555555556	0.05555555556	1.473684211	1.714285714
**APBB1**	0.04761904762	0.04761904762	0	0	inf	4.7619E+31
**APBB1II**	0.09523809524	0.09523809524	0.1111111111	0.1111111111	0.6842105263	0.8571428571
**APBB2**	0.1904761905	0.1904761905	0.05555555556	0.05555555556	3.294117647	3.428571429
**APBB3**	0.04761904762	0.04761904762	0	0	inf	4.7619E+31
**APC**	0.04761904762	0.04761904762	0	0	inf	4.7619E+31
**APC2**	0.09523809524	0.09523809524	0.1111111111	0.1111111111	0.6842105263	0.8571428571
**APCDD1**	0.09523809524	0.09523809524	0.05555555556	0.05555555556	1.473684211	1.714285714
**PCDD1**	0.1428571429	0.1428571429	0.2222222222	0.2222222222	0.4583333333	0.6428571429
**CDD1L-**	0.1428571429	0.1428571429	0.2222222222	0.2222222222	0.4583333333	0.6428571429
**APCS**	0.1428571429	0.1428571429	0	0	inf	1.42857E+32
**APEH**	0.09523809524	0.09523809524	0	0	inf	9.52381E+31
**APELA**	0.04761904762	0.04761904762	0.05555555556	0.05555555556	0.7	0.8571428571
**APH1A**	0.1904761905	0.1904761905	0.05555555556	0.05555555556	3.294117647	3.428571429

APH1B	0.04761904762	0.04761904762	0.05555555556	0.05555555556	0.7	0.8571428571
APLF	0	0	0.05555555556	0.05555555556	0	0
APLP1	0.04761904762	0.04761904762	0.05555555556	0.05555555556	0.7	0.8571428571
APMAP	0.1904761905	0.1904761905	0.05555555556	0.05555555556	3.294117647	3.428571429
APOA2	0.1428571429	0.1428571429	0	0	inf	1.42857E+32
APOB	0	0	0.05555555556	0.05555555556	0	0
APOBEC	0.04761904762	0.04761904762	0.1111111111	0.1111111111	0.325	0.4285714286
APOBEC	0	0	0.05555555556	0.05555555556	0	0
POBEC3	0.04761904762	0.04761904762	0.1111111111	0.1111111111	0.325	0.4285714286
OBEC3A	0.04761904762	0.04761904762	0.1111111111	0.1111111111	0.325	0.4285714286
POBEC3	0.04761904762	0.04761904762	0.1111111111	0.1111111111	0.325	0.4285714286
BEC3B-	0.04761904762	0.04761904762	0.1111111111	0.1111111111	0.325	0.4285714286
POBEC3	0.04761904762	0.04761904762	0.1111111111	0.1111111111	0.325	0.4285714286
POBEC3	0.04761904762	0.04761904762	0.1111111111	0.1111111111	0.325	0.4285714286
POBEC3	0.04761904762	0.04761904762	0.1111111111	0.1111111111	0.325	0.4285714286
POBEC3	0.04761904762	0.04761904762	0.1111111111	0.1111111111	0.325	0.4285714286
POBEC3	0.04761904762	0.04761904762	0.1111111111	0.1111111111	0.325	0.4285714286
APOBR	0.04761904762	0.04761904762	0	0	inf	4.7619E+31
APOC1	0.09523809524	0.09523809524	0.1111111111	0.1111111111	0.6842105263	0.8571428571
APOC1P	0.09523809524	0.09523809524	0.1111111111	0.1111111111	0.6842105263	0.8571428571
APOC2	0.09523809524	0.09523809524	0.1111111111	0.1111111111	0.6842105263	0.8571428571
APOC4	0.09523809524	0.09523809524	0.1111111111	0.1111111111	0.6842105263	0.8571428571
OC4-APO	0.09523809524	0.09523809524	0.1111111111	0.1111111111	0.6842105263	0.8571428571
APOD	0.1428571429	0.1428571429	0.05555555556	0.05555555556	2.333333333	2.571428571
APOE	0.09523809524	0.09523809524	0.1111111111	0.1111111111	0.6842105263	0.8571428571
APOF	0.1428571429	0.1428571429	0.05555555556	0.05555555556	2.333333333	2.571428571
APOH	0	0	0	0		0.00001
APOL1	0	0	0.1111111111	0.1111111111	0	0
APOL2	0	0	0.1111111111	0.1111111111	0	0
APOL3	0	0	0.1111111111	0.1111111111	0	0
APOL4	0	0	0.1111111111	0.1111111111	0	0
APOL5	0	0	0.1111111111	0.1111111111	0	0
APOL6	0	0	0.1111111111	0.1111111111	0	0
APOLD1	0.04761904762	0.04761904762	0.05555555556	0.05555555556	0.7	0.8571428571
APOM	0.04761904762	0.04761904762	0	0	inf	4.7619E+31
APOOP5	0.04761904762	0.04761904762	0	0	inf	4.7619E+31
APP	0.04761904762	0.04761904762	0.1111111111	0.1111111111	0.325	0.4285714286
APPBP2	0.1428571429	0.1428571429	0	0	inf	1.42857E+32
APPL1	0.09523809524	0.09523809524	0	0	inf	9.52381E+31
APPL2	0.04761904762	0.04761904762	0.05555555556	0.05555555556	0.7	0.8571428571
APRT	0.1904761905	0.1904761905	0	0	inf	1.90476E+32
APTR	0.04761904762	0.04761904762	0.1111111111	0.1111111111	0.325	0.4285714286
APTX	0.09523809524	0.09523809524	0.05555555556	0.05555555556	1.473684211	1.714285714
AQP1	0.04761904762	0.04761904762	0.1111111111	0.1111111111	0.325	0.4285714286
AQP10	0.1904761905	0.1904761905	0.05555555556	0.05555555556	3.294117647	3.428571429
AQP12A	0	0	0.05555555556	0.05555555556	0	0
AQP12B	0	0	0.05555555556	0.05555555556	0	0
AQP2	0.04761904762	0.04761904762	0.05555555556	0.05555555556	0.7	0.8571428571
AQP3	0.09523809524	0.09523809524	0.05555555556	0.05555555556	1.473684211	1.714285714
AQP4	0.09523809524	0.09523809524	0.05555555556	0.05555555556	1.473684211	1.714285714
QP4-AS	0.09523809524	0.09523809524	0.05555555556	0.05555555556	1.473684211	1.714285714

**AQP5**	0.04761904762	0.04761904762	0.05555555556	0.05555555556	0.7	0.8571428571
**AQP6**	0.04761904762	0.04761904762	0.05555555556	0.05555555556	0.7	0.8571428571
**AQP7**	0.09523809524	0.09523809524	0.05555555556	0.05555555556	1.473684211	1.714285714
**AQP7P1**	0.09523809524	0.09523809524	0.2222222222	0.2222222222	0.2894736842	0.4285714286
**AQP7P3**	0.1904761905	0.1904761905	0.2222222222	0.2222222222	0.6470588235	0.8571428571
**AQP8**	0.04761904762	0.04761904762	0	0	inf	4.7619E+31
**AQP9**	0.04761904762	0.04761904762	0.05555555556	0.05555555556	0.7	0.8571428571
**ARAP3**	0.04761904762	0.04761904762	0	0	inf	4.7619E+31
**ARC**	0.04761904762	0.04761904762	0.1666666667	0.1666666667	0.2	0.2857142857
**AREG**	0.09523809524	0.09523809524	0.05555555556	0.05555555556	1.473684211	1.714285714
**ARF1**	0.1428571429	0.1428571429	0	0	inf	1.42857E+32
**ARF3**	0.04761904762	0.04761904762	0.05555555556	0.05555555556	0.7	0.8571428571
**ARF4**	0.09523809524	0.09523809524	0	0	inf	9.52381E+31
**RF4-AS**	0.09523809524	0.09523809524	0	0	inf	9.52381E+31
**ARF5**	0.04761904762	0.04761904762	0.05555555556	0.05555555556	0.7	0.8571428571
**ARF6**	0.04761904762	0.04761904762	0	0	inf	4.7619E+31
**RFGAP**	0.1428571429	0.1428571429	0.2222222222	0.2222222222	0.4583333333	0.6428571429
**RFGAP**	0	0	0.05555555556	0.05555555556	0	0
**RFGAP**	0	0	0.1666666667	0.1666666667	0	0
**RFGEF**	0.1428571429	0.1428571429	0.1666666667	0.1666666667	0.6666666667	0.8571428571
**RFGEF**	0.09523809524	0.09523809524	0.1666666667	0.1666666667	0.4210526316	0.5714285714
**ARFIP1**	0	0	0.05555555556	0.05555555556	0	0
**ARFIP2**	0.04761904762	0.04761904762	0	0	inf	4.7619E+31
**ARFRP1**	0.1428571429	0.1428571429	0.2222222222	0.2222222222	0.4583333333	0.6428571429
**ARGFX**	0.1428571429	0.1428571429	0.05555555556	0.05555555556	2.333333333	2.571428571
**RGFXP**	0	0	0	0		0.00001
**RHGAP**	0	0	0.05555555556	0.05555555556	0	0
**RHGAP**	0	0	0.05555555556	0.05555555556	0	0
**RHGAP**	0.09523809524	0.09523809524	0.2222222222	0.2222222222	0.2894736842	0.4285714286
**RHGAP**	0	0	0.05555555556	0.05555555556	0	0
**RHGAP**	0.04761904762	0.04761904762	0	0	inf	4.7619E+31
**RHGAP**	0.1428571429	0.1428571429	0	0	inf	1.42857E+32
**GAP19-S**	0.1428571429	0.1428571429	0	0	inf	1.42857E+32
**RHGAP**	0.09523809524	0.09523809524	0.1111111111	0.1111111111	0.6842105263	0.8571428571
**RHGAP**	0.09523809524	0.09523809524	0.05555555556	0.05555555556	1.473684211	1.714285714
**IGAP22-**	0.09523809524	0.09523809524	0.05555555556	0.05555555556	1.473684211	1.714285714
**RHGAP**	0	0	0.05555555556	0.05555555556	0	0
**RHGAP**	0	0	0.05555555556	0.05555555556	0	0
**RHGAP**	0.04761904762	0.04761904762	0	0	inf	4.7619E+31
**IGAP26-**	0.04761904762	0.04761904762	0	0	inf	4.7619E+31
**IGAP26-**	0.04761904762	0.04761904762	0	0	inf	4.7619E+31
**RHGAP**	0.1428571429	0.1428571429	0	0	inf	1.42857E+32
**HGAP2**	0.04761904762	0.04761904762	0	0	inf	4.7619E+31
**1-BPTF**	0.04761904762	0.04761904762	0	0	inf	4.7619E+31
**HGAP2**	0	0	0	0		0.00001
**RHGAP**	0.09523809524	0.09523809524	0	0	inf	9.52381E+31
**RHGAP**	0.04761904762	0.04761904762	0.05555555556	0.05555555556	0.7	0.8571428571
**RHGAP**	0.1428571429	0.1428571429	0	0	inf	1.42857E+32
**RHGAP**	0.09523809524	0.09523809524	0.05555555556	0.05555555556	1.473684211	1.714285714
**IGAP31-**	0.09523809524	0.09523809524	0.05555555556	0.05555555556	1.473684211	1.714285714
**RHGAP**	0.04761904762	0.04761904762	0.05555555556	0.05555555556	0.7	0.8571428571

**RHGAP**	0.09523809524	0.09523809524	0.1111111111	0.1111111111	0.6842105263	0.8571428571
**RHGAP**	0.09523809524	0.09523809524	0.2222222222	0.2222222222	0.2894736842	0.4285714286
**RHGAP**	0.1428571429	0.1428571429	0.1666666667	0.1666666667	0.6666666667	0.8571428571
**RHGAP**	0.04761904762	0.04761904762	0.1111111111	0.1111111111	0.325	0.4285714286
**RHGAP**	0.09523809524	0.09523809524	0.1111111111	0.1111111111	0.6842105263	0.8571428571
**RHGAP**	0	0	0.1666666667	0.1666666667	0	0
**RHGDI**	0.04761904762	0.04761904762	0	0	inf	4.7619E+31
**RHGDI**	0.04761904762	0.04761904762	0.1111111111	0.1111111111	0.325	0.4285714286
**RHGDI**	0.04761904762	0.04761904762	0.2222222222	0.2222222222	0.1375	0.2142857143
**RHGEF**	0.04761904762	0.04761904762	0.05555555556	0.05555555556	0.7	0.8571428571
**RHGEF**	0.1428571429	0.1428571429	0.1111111111	0.1111111111	1.083333333	1.285714286
**RHGEF1**	0.04761904762	0.04761904762	0.1111111111	0.1111111111	0.325	0.4285714286
**RHGEF**	0.1428571429	0.1428571429	0.05555555556	0.05555555556	2.333333333	2.571428571
**RHGEF**	0.04761904762	0.04761904762	0.05555555556	0.05555555556	0.7	0.8571428571
**RHGEF**	0	0	0.05555555556	0.05555555556	0	0
**RHGEF**	0.1428571429	0.1428571429	0.05555555556	0.05555555556	2.333333333	2.571428571
**RHGEF**	0.04761904762	0.04761904762	0.1111111111	0.1111111111	0.325	0.4285714286
**RHGEF**	0.1428571429	0.1428571429	0.1111111111	0.1111111111	1.083333333	1.285714286
**RHGEF**	0.1428571429	0.1428571429	0.05555555556	0.05555555556	2.333333333	2.571428571
**RHGEF**	0.1904761905	0.1904761905	0.05555555556	0.05555555556	3.294117647	3.428571429
**IGEF26-**	0.1904761905	0.1904761905	0.05555555556	0.05555555556	3.294117647	3.428571429
**RHGEF**	0	0	0.05555555556	0.05555555556	0	0
**RHGEF**	0.04761904762	0.04761904762	0	0	inf	4.7619E+31
**HGEF3-**	0.04761904762	0.04761904762	0	0	inf	4.7619E+31
**RHGEF**	0	0	0.05555555556	0.05555555556	0	0
**RHGEF3**	0.09523809524	0.09523809524	0.05555555556	0.05555555556	1.473684211	1.714285714
**RHGEF**	0.04761904762	0.04761904762	0.05555555556	0.05555555556	0.7	0.8571428571
**RHGEF**	0	0	0.05555555556	0.05555555556	0	0
**IGEF38**	0	0	0.05555555556	0.05555555556	0	0
**RHGEF**	0.09523809524	0.09523809524	0.05555555556	0.05555555556	1.473684211	1.714285714
**RHGEF**	0	0	0.05555555556	0.05555555556	0	0
**RHGEF**	0.09523809524	0.09523809524	0.05555555556	0.05555555556	1.473684211	1.714285714
**ARID1A**	0.04761904762	0.04761904762	0.1111111111	0.1111111111	0.325	0.4285714286
**ARID2**	0.04761904762	0.04761904762	0.05555555556	0.05555555556	0.7	0.8571428571
**ARID3A**	0.09523809524	0.09523809524	0.1111111111	0.1111111111	0.6842105263	0.8571428571
**ARID3B**	0.04761904762	0.04761904762	0.05555555556	0.05555555556	0.7	0.8571428571
**ARID3C**	0.09523809524	0.09523809524	0.05555555556	0.05555555556	1.473684211	1.714285714
**ARID4A**	0.04761904762	0.04761904762	0	0	inf	4.7619E+31
**ARID4B**	0.1428571429	0.1428571429	0.05555555556	0.05555555556	2.333333333	2.571428571
**ARID5A**	0	0	0.1111111111	0.1111111111	0	0
**ARID5B**	0.04761904762	0.04761904762	0.1666666667	0.1666666667	0.2	0.2857142857
**ARIH1**	0.04761904762	0.04761904762	0.05555555556	0.05555555556	0.7	0.8571428571
**ARIH2**	0.09523809524	0.09523809524	0	0	inf	9.52381E+31
**RIH2O**	0.09523809524	0.09523809524	0	0	inf	9.52381E+31
**ARL1**	0.04761904762	0.04761904762	0.05555555556	0.05555555556	0.7	0.8571428571
**ARL10**	0.09523809524	0.09523809524	0.05555555556	0.05555555556	1.473684211	1.714285714
**ARL13B**	0.04761904762	0.04761904762	0.05555555556	0.05555555556	0.7	0.8571428571
**ARL14**	0.1428571429	0.1428571429	0.05555555556	0.05555555556	2.333333333	2.571428571
**RL14EP**	0.04761904762	0.04761904762	0	0	inf	4.7619E+31
**ARL15**	0.04761904762	0.04761904762	0.05555555556	0.05555555556	0.7	0.8571428571
**ARL16**	0.04761904762	0.04761904762	0	0	inf	4.7619E+31

ARL17A	0.2380952381	0.2380952381	0	0	inf	2.38095E+32
ARL17B	0.2380952381	0.2380952381	0	0	inf	2.38095E+32
ARL2BP	0.04761904762	0.04761904762	0	0	inf	4.7619E+31
ARL3	0.1428571429	0.1428571429	0.05555555556	0.05555555556	2.333333333	2.571428571
ARL4A	0.04761904762	0.04761904762	0.2222222222	0.2222222222	0.1375	0.2142857143
ARL4C	0	0	0.05555555556	0.05555555556	0	0
ARL4D	0.04761904762	0.04761904762	0	0	inf	4.7619E+31
ARL5A	0	0	0.05555555556	0.05555555556	0	0
ARL5B	0.09523809524	0.09523809524	0.1111111111	0.1111111111	0.6842105263	0.8571428571
ARL5C	0.09523809524	0.09523809524	0	0	inf	9.52381E+31
ARL6	0.04761904762	0.04761904762	0.05555555556	0.05555555556	0.7	0.8571428571
ARL6IP1	0.04761904762	0.04761904762	0	0	inf	4.7619E+31
ARL6IP4	0.04761904762	0.04761904762	0.1111111111	0.1111111111	0.325	0.4285714286
ARL6IP5	0.04761904762	0.04761904762	0	0	inf	4.7619E+31
ARL6IP6	0	0	0.05555555556	0.05555555556	0	0
ARL8A	0.1428571429	0.1428571429	0	0	inf	1.42857E+32
ARL8B	0.04761904762	0.04761904762	0	0	inf	4.7619E+31
ARLNC1	0.09523809524	0.09523809524	0	0	inf	9.52381E+31
ARMC1	0.1428571429	0.1428571429	0.1666666667	0.1666666667	0.6666666667	0.8571428571
ARMC10	0.04761904762	0.04761904762	0.05555555556	0.05555555556	0.7	0.8571428571
ARMC12	0.04761904762	0.04761904762	0.05555555556	0.05555555556	0.7	0.8571428571
ARMC2	0	0	0	0		0.00001
RMC2-A	0	0	0	0		0.00001
ARMC3	0.09523809524	0.09523809524	0.1111111111	0.1111111111	0.6842105263	0.8571428571
ARMC4	0.09523809524	0.09523809524	0.1111111111	0.1111111111	0.6842105263	0.8571428571
RMC4P	0.09523809524	0.09523809524	0.1111111111	0.1111111111	0.6842105263	0.8571428571
ARMC5	0.04761904762	0.04761904762	0	0	inf	4.7619E+31
ARMC6	0.1428571429	0.1428571429	0.1111111111	0.1111111111	1.083333333	1.285714286
ARMC7	0.04761904762	0.04761904762	0.05555555556	0.05555555556	0.7	0.8571428571
ARMC8	0.1428571429	0.1428571429	0.05555555556	0.05555555556	2.333333333	2.571428571
ARMC9	0	0	0.05555555556	0.05555555556	0	0
ARMH1	0.04761904762	0.04761904762	0.05555555556	0.05555555556	0.7	0.8571428571
ARMH2	0	0	0.05555555556	0.05555555556	0	0
ARMH3	0.1428571429	0.1428571429	0.05555555556	0.05555555556	2.333333333	2.571428571
ARMH4	0.04761904762	0.04761904762	0	0	inf	4.7619E+31
ARMS2	0.1428571429	0.1428571429	0.05555555556	0.05555555556	2.333333333	2.571428571
ARNT	0.1904761905	0.1904761905	0.05555555556	0.05555555556	3.294117647	3.428571429
ARNT2	0.04761904762	0.04761904762	0.05555555556	0.05555555556	0.7	0.8571428571
ARNTL2	0.04761904762	0.04761904762	0.05555555556	0.05555555556	0.7	0.8571428571
NTL2-A	0.04761904762	0.04761904762	0.05555555556	0.05555555556	0.7	0.8571428571
ARPC1A	0.04761904762	0.04761904762	0.05555555556	0.05555555556	0.7	0.8571428571
ARPC1B	0.04761904762	0.04761904762	0.05555555556	0.05555555556	0.7	0.8571428571
ARPC2	0	0	0.05555555556	0.05555555556	0	0
ARPC3	0	0	0.1111111111	0.1111111111	0	0
ARPC4	0.09523809524	0.09523809524	0	0	inf	9.52381E+31
PC4-TTI	0.09523809524	0.09523809524	0	0	inf	9.52381E+31
ARPC5L	0.09523809524	0.09523809524	0.05555555556	0.05555555556	1.473684211	1.714285714
ARPIN	0.04761904762	0.04761904762	0.05555555556	0.05555555556	0.7	0.8571428571
PIN-AP3	0.04761904762	0.04761904762	0.05555555556	0.05555555556	0.7	0.8571428571
ARPP19	0.04761904762	0.04761904762	0.05555555556	0.05555555556	0.7	0.8571428571
ARPP21	0.04761904762	0.04761904762	0	0	inf	4.7619E+31

ARRB2	0	0	0.1111111111	0.1111111111	0	0
ARRDC1	0.09523809524	0.09523809524	0	0	inf	9.52381E+31
RRDC1-A	0.09523809524	0.09523809524	0	0	inf	9.52381E+31
ARRDC2	0.1428571429	0.1428571429	0.1111111111	0.1111111111	1.083333333	1.285714286
ARRDC3	0.04761904762	0.04761904762	0	0	inf	4.7619E+31
RRDC3-A	0.04761904762	0.04761904762	0	0	inf	4.7619E+31
ARRDC4	0.04761904762	0.04761904762	0.05555555556	0.05555555556	0.7	0.8571428571
ARRDC5	0.09523809524	0.09523809524	0.05555555556	0.05555555556	1.473684211	1.714285714
ARSA	0	0	0.1111111111	0.1111111111	0	0
ARSB	0.04761904762	0.04761904762	0	0	inf	4.7619E+31
ARSG	0	0	0	0		0.00001
ARSJ	0	0	0.05555555556	0.05555555556	0	0
ARSK	0.04761904762	0.04761904762	0	0	inf	4.7619E+31
ART1	0.04761904762	0.04761904762	0	0	inf	4.7619E+31
ART3	0.04761904762	0.04761904762	0.05555555556	0.05555555556	0.7	0.8571428571
ART4	0.04761904762	0.04761904762	0.1111111111	0.1111111111	0.325	0.4285714286
ART5	0.04761904762	0.04761904762	0	0	inf	4.7619E+31
ARTN	0.04761904762	0.04761904762	0.05555555556	0.05555555556	0.7	0.8571428571
ARV1	0.1428571429	0.1428571429	0	0	inf	1.42857E+32
ARVCF	0.04761904762	0.04761904762	0.05555555556	0.05555555556	0.7	0.8571428571
S-PTPR	0.2380952381	0.2380952381	0.05555555556	0.05555555556	4.375	4.285714286
AS3MT	0.1428571429	0.1428571429	0.05555555556	0.05555555556	2.333333333	2.571428571
ASAH1	0.09523809524	0.09523809524	0.1111111111	0.1111111111	0.6842105263	0.8571428571
ASAH2	0.04761904762	0.04761904762	0.05555555556	0.05555555556	0.7	0.8571428571
ASAH2B	0.04761904762	0.04761904762	0.05555555556	0.05555555556	0.7	0.8571428571
ASAP1	0.04761904762	0.04761904762	0.1111111111	0.1111111111	0.325	0.4285714286
SAP1-IT	0.04761904762	0.04761904762	0.1111111111	0.1111111111	0.325	0.4285714286
SAP1-IT	0.04761904762	0.04761904762	0.1111111111	0.1111111111	0.325	0.4285714286
ASAP2	0	0	0.05555555556	0.05555555556	0	0
ASAP3	0.04761904762	0.04761904762	0.1111111111	0.1111111111	0.325	0.4285714286
ASB1	0	0	0.05555555556	0.05555555556	0	0
ASB10	0.09523809524	0.09523809524	0.05555555556	0.05555555556	1.473684211	1.714285714
ASB13	0.04761904762	0.04761904762	0.1666666667	0.1666666667	0.2	0.2857142857
ASB14	0.09523809524	0.09523809524	0	0	inf	9.52381E+31
ASB15	0.04761904762	0.04761904762	0.05555555556	0.05555555556	0.7	0.8571428571
ASB16	0.09523809524	0.09523809524	0	0	inf	9.52381E+31
SB16-AS	0.09523809524	0.09523809524	0	0	inf	9.52381E+31
ASB17	0.04761904762	0.04761904762	0.05555555556	0.05555555556	0.7	0.8571428571
ASB18	0	0	0.05555555556	0.05555555556	0	0
ASB3	0.04761904762	0.04761904762	0.05555555556	0.05555555556	0.7	0.8571428571
ASB4	0.04761904762	0.04761904762	0.1111111111	0.1111111111	0.325	0.4285714286
ASB5	0	0	0.05555555556	0.05555555556	0	0
ASB6	0.09523809524	0.09523809524	0.05555555556	0.05555555556	1.473684211	1.714285714
ASB7	0.04761904762	0.04761904762	0.05555555556	0.05555555556	0.7	0.8571428571
ASB8	0.04761904762	0.04761904762	0.05555555556	0.05555555556	0.7	0.8571428571
ASB9P1	0.04761904762	0.04761904762	0.05555555556	0.05555555556	0.7	0.8571428571
ASCC1	0.1904761905	0.1904761905	0.05555555556	0.05555555556	3.294117647	3.428571429
ASCC2	0.04761904762	0.04761904762	0.1111111111	0.1111111111	0.325	0.4285714286
ASCL1	0.04761904762	0.04761904762	0.1111111111	0.1111111111	0.325	0.4285714286
ASCL2	0.04761904762	0.04761904762	0	0	inf	4.7619E+31
ASCL4	0.04761904762	0.04761904762	0.05555555556	0.05555555556	0.7	0.8571428571

ASCL5	0.1428571429	0.1428571429	0	0	inf	1.42857E+32
ASDURF	0	0	0.05555555556	0.05555555556	0	0
ASF1B	0.1428571429	0.1428571429	0.1111111111	0.1111111111	1.083333333	1.285714286
ASGR1	0	0	0.05555555556	0.05555555556	0	0
ASGR2	0	0	0.05555555556	0.05555555556	0	0
ASH1L	0.1904761905	0.1904761905	0.1111111111	0.1111111111	1.529411765	1.714285714
SH1L-AS	0.1904761905	0.1904761905	0.1111111111	0.1111111111	1.529411765	1.714285714
ASH2L	0.09523809524	0.09523809524	0.1111111111	0.1111111111	0.6842105263	0.8571428571
ASIC1	0.04761904762	0.04761904762	0.05555555556	0.05555555556	0.7	0.8571428571
ASIC2	0.09523809524	0.09523809524	0	0	inf	9.52381E+31
ASIC3	0.09523809524	0.09523809524	0.05555555556	0.05555555556	1.473684211	1.714285714
ASIC4	0	0	0.05555555556	0.05555555556	0	0
ASIC5	0	0	0.1111111111	0.1111111111	0	0
ASIP	0.09523809524	0.09523809524	0.1111111111	0.1111111111	0.6842105263	0.8571428571
ASL	0.04761904762	0.04761904762	0.1111111111	0.1111111111	0.325	0.4285714286
ASNA1	0.1428571429	0.1428571429	0.1111111111	0.1111111111	1.083333333	1.285714286
ASNS	0.04761904762	0.04761904762	0.05555555556	0.05555555556	0.7	0.8571428571
ASNSD1	0	0	0.05555555556	0.05555555556	0	0
ASNSP1	0.09523809524	0.09523809524	0.1666666667	0.1666666667	0.4210526316	0.5714285714
ASPA	0	0	0.1111111111	0.1111111111	0	0
ASPDH	0.09523809524	0.09523809524	0.1111111111	0.1111111111	0.6842105263	0.8571428571
ASPH	0.1428571429	0.1428571429	0.1666666667	0.1666666667	0.6666666667	0.8571428571
ASPHD1	0.04761904762	0.04761904762	0	0	inf	4.7619E+31
ASPHD2	0.04761904762	0.04761904762	0.05555555556	0.05555555556	0.7	0.8571428571
ASPM	0.1428571429	0.1428571429	0	0	inf	1.42857E+32
ASPN	0.04761904762	0.04761904762	0.05555555556	0.05555555556	0.7	0.8571428571
ASPRV1	0	0	0.05555555556	0.05555555556	0	0
ASPSCR	0.04761904762	0.04761904762	0	0	inf	4.7619E+31
ASS1	0.09523809524	0.09523809524	0.05555555556	0.05555555556	1.473684211	1.714285714
ASTE1	0.09523809524	0.09523809524	0.05555555556	0.05555555556	1.473684211	1.714285714
ASTL	0	0	0.1111111111	0.1111111111	0	0
ASTN1	0.1428571429	0.1428571429	0	0	inf	1.42857E+32
ASTN2	0.04761904762	0.04761904762	0.05555555556	0.05555555556	0.7	0.8571428571
STN2-AS	0.04761904762	0.04761904762	0.05555555556	0.05555555556	0.7	0.8571428571
ASXL1	0.09523809524	0.09523809524	0.1111111111	0.1111111111	0.6842105263	0.8571428571
ASXL3	0.09523809524	0.09523809524	0	0	inf	9.52381E+31
ASZ1	0.09523809524	0.09523809524	0.05555555556	0.05555555556	1.473684211	1.714285714
ATAD1	0.1428571429	0.1428571429	0.1666666667	0.1666666667	0.6666666667	0.8571428571
ATAD2	0.1428571429	0.1428571429	0.1111111111	0.1111111111	1.083333333	1.285714286
ATAD2E	0	0	0.05555555556	0.05555555556	0	0
ATAD3A	0	0	0.1111111111	0.1111111111	0	0
ATAD3E	0	0	0.1111111111	0.1111111111	0	0
ATAD3C	0	0	0.1111111111	0.1111111111	0	0
ATAD5	0	0	0.05555555556	0.05555555556	0	0
ATCAY	0.09523809524	0.09523809524	0.05555555556	0.05555555556	1.473684211	1.714285714
ATE1	0.2380952381	0.2380952381	0.05555555556	0.05555555556	4.375	4.285714286
TE1-AS	0.2380952381	0.2380952381	0.05555555556	0.05555555556	4.375	4.285714286
ATF1	0.09523809524	0.09523809524	0.05555555556	0.05555555556	1.473684211	1.714285714
ATF2	0	0	0.1111111111	0.1111111111	0	0
ATF3	0.09523809524	0.09523809524	0	0	inf	9.52381E+31
ATF4	0	0	0.1111111111	0.1111111111	0	0

ATF6	0.1428571429	0.1428571429	0	0	inf	1.42857E+32
ATF6B	0.04761904762	0.04761904762	0	0	inf	4.7619E+31
ATF7	0.04761904762	0.04761904762	0.05555555556	0.05555555556	0.7	0.8571428571
TF7-NP	0.04761904762	0.04761904762	0.05555555556	0.05555555556	0.7	0.8571428571
ATF7IP	0.04761904762	0.04761904762	0.1111111111	0.1111111111	0.325	0.4285714286
ATF7IP2	0.04761904762	0.04761904762	0	0	inf	4.7619E+31
ATG10	0.04761904762	0.04761904762	0.05555555556	0.05555555556	0.7	0.8571428571
ATG101	0.04761904762	0.04761904762	0.05555555556	0.05555555556	0.7	0.8571428571
ATG12	0.04761904762	0.04761904762	0	0	inf	4.7619E+31
ATG13	0	0	0.05555555556	0.05555555556	0	0
ATG14	0.04761904762	0.04761904762	0	0	inf	4.7619E+31
TG16L	0	0	0.05555555556	0.05555555556	0	0
ATG3	0.09523809524	0.09523809524	0.05555555556	0.05555555556	1.473684211	1.714285714
ATG4B	0	0	0.05555555556	0.05555555556	0	0
ATG4C	0.04761904762	0.04761904762	0.05555555556	0.05555555556	0.7	0.8571428571
ATG4D	0.1428571429	0.1428571429	0.1111111111	0.1111111111	1.083333333	1.285714286
ATG7	0.09523809524	0.09523809524	0	0	inf	9.52381E+31
ATG9B	0.09523809524	0.09523809524	0.05555555556	0.05555555556	1.473684211	1.714285714
ATIC	0	0	0.05555555556	0.05555555556	0	0
ATL1	0.04761904762	0.04761904762	0	0	inf	4.7619E+31
ATL2	0	0	0.05555555556	0.05555555556	0	0
ATMIN	0.09523809524	0.09523809524	0	0	inf	9.52381E+31
ATN1	0.04761904762	0.04761904762	0.1111111111	0.1111111111	0.325	0.4285714286
ATOH1	0	0	0.05555555556	0.05555555556	0	0
ATOH7	0.1904761905	0.1904761905	0.05555555556	0.05555555556	3.294117647	3.428571429
ATOH8	0	0	0.05555555556	0.05555555556	0	0
ATP10B	0.04761904762	0.04761904762	0.05555555556	0.05555555556	0.7	0.8571428571
ATP10D	0.04761904762	0.04761904762	0.05555555556	0.05555555556	0.7	0.8571428571
ATP11B	0.1904761905	0.1904761905	0.05555555556	0.05555555556	3.294117647	3.428571429
TP13A1	0.1428571429	0.1428571429	0.1111111111	0.1111111111	1.083333333	1.285714286
TP13A2	0.04761904762	0.04761904762	0.1111111111	0.1111111111	0.325	0.4285714286
TP13A3	0.09523809524	0.09523809524	0.05555555556	0.05555555556	1.473684211	1.714285714
TP13A4	0.09523809524	0.09523809524	0.05555555556	0.05555555556	1.473684211	1.714285714
P13A4-A	0.09523809524	0.09523809524	0.05555555556	0.05555555556	1.473684211	1.714285714
TP13A5	0.09523809524	0.09523809524	0.05555555556	0.05555555556	1.473684211	1.714285714
P13A5-A	0.09523809524	0.09523809524	0.05555555556	0.05555555556	1.473684211	1.714285714
ATP1A1	0.04761904762	0.04761904762	0.05555555556	0.05555555556	0.7	0.8571428571
P1A1-A	0.04761904762	0.04761904762	0.05555555556	0.05555555556	0.7	0.8571428571
ATP1A2	0.1428571429	0.1428571429	0	0	inf	1.42857E+32
ATP1A3	0.04761904762	0.04761904762	0.05555555556	0.05555555556	0.7	0.8571428571
ATP1A4	0.1428571429	0.1428571429	0	0	inf	1.42857E+32
ATP1B1	0.1428571429	0.1428571429	0	0	inf	1.42857E+32
ATP1B2	0	0	0.05555555556	0.05555555556	0	0
ATP1B3	0.1904761905	0.1904761905	0.05555555556	0.05555555556	3.294117647	3.428571429
ATP23	0.1428571429	0.1428571429	0.05555555556	0.05555555556	2.333333333	2.571428571
ATP2A1	0.04761904762	0.04761904762	0	0	inf	4.7619E+31
P2A1-A	0.04761904762	0.04761904762	0	0	inf	4.7619E+31
ATP2A2	0	0	0.1111111111	0.1111111111	0	0
ATP2A3	0	0	0.1111111111	0.1111111111	0	0
ATP2B1	0.04761904762	0.04761904762	0.05555555556	0.05555555556	0.7	0.8571428571
P2B1-A	0.04761904762	0.04761904762	0.05555555556	0.05555555556	0.7	0.8571428571

**ATP2B2**	0.04761904762	0.04761904762	0	0	inf	4.7619E+31
**TP2B2-I**	0.04761904762	0.04761904762	0	0	inf	4.7619E+31
**ATP2B4**	0.2380952381	0.2380952381	0	0	inf	2.38095E+32
**ATP2C1**	0.09523809524	0.09523809524	0.05555555556	0.05555555556	1.473684211	1.714285714
**ATP2C2**	0.04761904762	0.04761904762	0	0	inf	4.7619E+31
**P2C2-A**	0.04761904762	0.04761904762	0	0	inf	4.7619E+31
**ATP4A**	0.04761904762	0.04761904762	0.05555555556	0.05555555556	0.7	0.8571428571
**ATP5F1**	0.04761904762	0.04761904762	0.05555555556	0.05555555556	0.7	0.8571428571
**ATP5F1**	0.1428571429	0.1428571429	0.05555555556	0.05555555556	2.333333333	2.571428571
**ATP5F1**	0.09523809524	0.09523809524	0.1111111111	0.1111111111	0.6842105263	0.8571428571
**ATP5F1**	0.09523809524	0.09523809524	0.1111111111	0.1111111111	0.6842105263	0.8571428571
**ATP5F1**	0.1428571429	0.1428571429	0.2222222222	0.2222222222	0.4583333333	0.6428571429
**ATP5IF1**	0.04761904762	0.04761904762	0.1111111111	0.1111111111	0.325	0.4285714286
**TP5MC**	0.1428571429	0.1428571429	0	0	inf	1.42857E+32
**TP5MC**	0.04761904762	0.04761904762	0.05555555556	0.05555555556	0.7	0.8571428571
**TP5MC**	0	0	0.1111111111	0.1111111111	0	0
**ATP5MI**	0.1428571429	0.1428571429	0.05555555556	0.05555555556	2.333333333	2.571428571
**ATP5MI**	0.04761904762	0.04761904762	0	0	inf	4.7619E+31
**ATP5MI**	0.04761904762	0.04761904762	0.05555555556	0.05555555556	0.7	0.8571428571
**5MF-PT**	0.04761904762	0.04761904762	0.05555555556	0.05555555556	0.7	0.8571428571
**ATP5PB**	0.04761904762	0.04761904762	0	0	inf	4.7619E+31
**ATP5PD**	0.04761904762	0.04761904762	0	0	inf	4.7619E+31
**ATP5PF**	0.04761904762	0.04761904762	0.1111111111	0.1111111111	0.325	0.4285714286
**ATP5PO**	0.04761904762	0.04761904762	0.1111111111	0.1111111111	0.325	0.4285714286
**TP6AP1**	0.04761904762	0.04761904762	0.05555555556	0.05555555556	0.7	0.8571428571
**TP6V0A**	0.04761904762	0.04761904762	0	0	inf	4.7619E+31
**TP6V0A**	0.04761904762	0.04761904762	0.05555555556	0.05555555556	0.7	0.8571428571
**TP6V0A**	0.04761904762	0.04761904762	0.05555555556	0.05555555556	0.7	0.8571428571
**ATP6V0I**	0.04761904762	0.04761904762	0.05555555556	0.05555555556	0.7	0.8571428571
**ATP6V0C**	0.04761904762	0.04761904762	0.1111111111	0.1111111111	0.325	0.4285714286
**TP6V0C**	0.04761904762	0.04761904762	0.05555555556	0.05555555556	0.7	0.8571428571
**TP6V0D**	0.1428571429	0.1428571429	0.05555555556	0.05555555556	2.333333333	2.571428571
**TP6V0D**	0.1904761905	0.1904761905	0.1111111111	0.1111111111	1.529411765	1.714285714
**TP6V0E**	0	0	0.05555555556	0.05555555556	0	0
**TP6V0E**	0.09523809524	0.09523809524	0.05555555556	0.05555555556	1.473684211	1.714285714
**6V0E2-**	0.09523809524	0.09523809524	0.05555555556	0.05555555556	1.473684211	1.714285714
**ATP6V1**	0.09523809524	0.09523809524	0.05555555556	0.05555555556	1.473684211	1.714285714
**TP6V1B**	0	0	0.05555555556	0.05555555556	0	0
**6V1B1-**	0	0	0.05555555556	0.05555555556	0	0
**TP6V1B**	0.09523809524	0.09523809524	0.1111111111	0.1111111111	0.6842105263	0.8571428571
**TP6V1C**	0.1904761905	0.1904761905	0.1111111111	0.1111111111	1.529411765	1.714285714
**TP6V1C**	0	0	0.05555555556	0.05555555556	0	0
**TP6V1E**	0.04761904762	0.04761904762	0	0	inf	4.7619E+31
**TP6V1E**	0	0	0.05555555556	0.05555555556	0	0
**ATP6V1I**	0.04761904762	0.04761904762	0.05555555556	0.05555555556	0.7	0.8571428571
**TP6V1F**	0.04761904762	0.04761904762	0.05555555556	0.05555555556	0.7	0.8571428571
**TP6V1C**	0.04761904762	0.04761904762	0.05555555556	0.05555555556	0.7	0.8571428571
**TP6V1C**	0.04761904762	0.04761904762	0	0	inf	4.7619E+31
**V1G2-DI**	0.04761904762	0.04761904762	0	0	inf	4.7619E+31
**TP6V1C**	0.1428571429	0.1428571429	0.1111111111	0.1111111111	1.083333333	1.285714286
**ATP6V1I**	0.1428571429	0.1428571429	0.1111111111	0.1111111111	1.083333333	1.285714286

**ATP8B1**	0.09523809524	0.09523809524	0	0	inf	9.52381E+31
**ATP8B2**	0.1904761905	0.1904761905	0.05555555556	0.05555555556	3.294117647	3.428571429
**ATP8B3**	0.09523809524	0.09523809524	0.1111111111	0.1111111111	0.6842105263	0.8571428571
**ATP8B4**	0.04761904762	0.04761904762	0.05555555556	0.05555555556	0.7	0.8571428571
**ATP8B5I**	0.09523809524	0.09523809524	0.05555555556	0.05555555556	1.473684211	1.714285714
**ATP9A**	0.09523809524	0.09523809524	0.1666666667	0.1666666667	0.4210526316	0.5714285714
**ATP9B**	0.04761904762	0.04761904762	0.05555555556	0.05555555556	0.7	0.8571428571
**ATPAF1**	0.04761904762	0.04761904762	0.05555555556	0.05555555556	0.7	0.8571428571
**ATPAF2**	0.04761904762	0.04761904762	0.1111111111	0.1111111111	0.325	0.4285714286
**TPSCKM**	0.04761904762	0.04761904762	0.05555555556	0.05555555556	0.7	0.8571428571
**ATR**	0.2380952381	0.2380952381	0.05555555556	0.05555555556	4.375	4.285714286
**ATRIP**	0.09523809524	0.09523809524	0	0	inf	9.52381E+31
**RIP-TRE**	0.09523809524	0.09523809524	0	0	inf	9.52381E+31
**ATRN**	0.1904761905	0.1904761905	0.05555555556	0.05555555556	3.294117647	3.428571429
**ATRNL1**	0.2380952381	0.2380952381	0	0	inf	2.38095E+32
**ATXN1**	0	0	0	0		0.00001
**ATXN10**	0	0	0.1111111111	0.1111111111	0	0
**ATXN1L**	0.04761904762	0.04761904762	0	0	inf	4.7619E+31
**ATXN2**	0	0	0.1111111111	0.1111111111	0	0
**TXN2-A**	0	0	0.1111111111	0.1111111111	0	0
**ATXN2L**	0.04761904762	0.04761904762	0	0	inf	4.7619E+31
**ATXN7**	0.04761904762	0.04761904762	0	0	inf	4.7619E+31
**TXN7L**	0.04761904762	0.04761904762	0.05555555556	0.05555555556	0.7	0.8571428571
**TXN7L**	0.04761904762	0.04761904762	0.05555555556	0.05555555556	0.7	0.8571428571
**TXN7L**	0.09523809524	0.09523809524	0	0	inf	9.52381E+31
**TXN7L3**	0.04761904762	0.04761904762	0.05555555556	0.05555555556	0.7	0.8571428571
**AUH**	0.04761904762	0.04761904762	0.05555555556	0.05555555556	0.7	0.8571428571
**AUNIP**	0.04761904762	0.04761904762	0.1111111111	0.1111111111	0.325	0.4285714286
**AUP1**	0	0	0.05555555556	0.05555555556	0	0
**AURKA**	0.09523809524	0.09523809524	0.1666666667	0.1666666667	0.4210526316	0.5714285714
**URKAIF**	0	0	0.1111111111	0.1111111111	0	0
**URKAP**	0.1428571429	0.1428571429	0.05555555556	0.05555555556	2.333333333	2.571428571
**AURKB**	0.04761904762	0.04761904762	0.05555555556	0.05555555556	0.7	0.8571428571
**AURKC**	0.1428571429	0.1428571429	0.05555555556	0.05555555556	2.333333333	2.571428571
**AUTS2**	0.04761904762	0.04761904762	0.1111111111	0.1111111111	0.325	0.4285714286
**AVIL**	0.1428571429	0.1428571429	0.05555555556	0.05555555556	2.333333333	2.571428571
**AVL9**	0.04761904762	0.04761904762	0.1666666667	0.1666666667	0.2	0.2857142857
**AVP**	0.1904761905	0.1904761905	0.05555555556	0.05555555556	3.294117647	3.428571429
**AVPI1**	0.1428571429	0.1428571429	0	0	inf	1.42857E+32
**AVPR1A**	0.09523809524	0.09523809524	0.05555555556	0.05555555556	1.473684211	1.714285714
**AVPR1E**	0.1428571429	0.1428571429	0	0	inf	1.42857E+32
**AXDND1**	0.1428571429	0.1428571429	0	0	inf	1.42857E+32
**AXIN1**	0.04761904762	0.04761904762	0.2222222222	0.2222222222	0.1375	0.2142857143
**AXIN2**	0.04761904762	0.04761904762	0	0	inf	4.7619E+31
**AXL**	0.04761904762	0.04761904762	0.05555555556	0.05555555556	0.7	0.8571428571
**AZGP1**	0.04761904762	0.04761904762	0.05555555556	0.05555555556	0.7	0.8571428571
**AZGP1P**	0.04761904762	0.04761904762	0.05555555556	0.05555555556	0.7	0.8571428571
**AZI2**	0.04761904762	0.04761904762	0	0	inf	4.7619E+31
**AZIN1**	0.1904761905	0.1904761905	0.1111111111	0.1111111111	1.529411765	1.714285714
**ZIN1-AS**	0.1904761905	0.1904761905	0.1111111111	0.1111111111	1.529411765	1.714285714
**AZIN2**	0.04761904762	0.04761904762	0.05555555556	0.05555555556	0.7	0.8571428571

AZU1	0.09523809524	0.09523809524	0.1111111111	0.1111111111	0.6842105263	0.8571428571
3GALNT	0.1428571429	0.1428571429	0.05555555556	0.05555555556	2.333333333	2.571428571
3GALNT	0.1428571429	0.1428571429	0.05555555556	0.05555555556	2.333333333	2.571428571
B3GALT	0.04761904762	0.04761904762	0.05555555556	0.05555555556	0.7	0.8571428571
B3GALT	0.1428571429	0.1428571429	0	0	inf	1.42857E+32
B3GALT	0.04761904762	0.04761904762	0.05555555556	0.05555555556	0.7	0.8571428571
GALT5-A	0.04761904762	0.04761904762	0.05555555556	0.05555555556	0.7	0.8571428571
B3GALT	0	0	0.1111111111	0.1111111111	0	0
B3GNT10	0.09523809524	0.09523809524	0.05555555556	0.05555555556	1.473684211	1.714285714
B3GNT2	0	0	0.05555555556	0.05555555556	0	0
B3GNT3	0.1428571429	0.1428571429	0.1111111111	0.1111111111	1.083333333	1.285714286
B3GNT4	0.04761904762	0.04761904762	0.1111111111	0.1111111111	0.325	0.4285714286
B3GNT5	0.1904761905	0.1904761905	0.05555555556	0.05555555556	3.294117647	3.428571429
B3GNT7	0	0	0.05555555556	0.05555555556	0	0
B3GNT8	0.04761904762	0.04761904762	0.05555555556	0.05555555556	0.7	0.8571428571
B3GNT9	0.1428571429	0.1428571429	0.05555555556	0.05555555556	2.333333333	2.571428571
4GALNT	0.1428571429	0.1428571429	0.05555555556	0.05555555556	2.333333333	2.571428571
4GALNT	0.1428571429	0.1428571429	0	0	inf	1.42857E+32
4GALNT	0.04761904762	0.04761904762	0.1111111111	0.1111111111	0.325	0.4285714286
4GALNT	0.04761904762	0.04761904762	0	0	inf	4.7619E+31
B4GALT	0.09523809524	0.09523809524	0.05555555556	0.05555555556	1.473684211	1.714285714
GALT1-A	0.09523809524	0.09523809524	0.05555555556	0.05555555556	1.473684211	1.714285714
B4GALT	0.04761904762	0.04761904762	0.05555555556	0.05555555556	0.7	0.8571428571
B4GALT	0.1428571429	0.1428571429	0	0	inf	1.42857E+32
B4GALT	0.09523809524	0.09523809524	0.05555555556	0.05555555556	1.473684211	1.714285714
GALT4-A	0.09523809524	0.09523809524	0.05555555556	0.05555555556	1.473684211	1.714285714
B4GALT	0.09523809524	0.09523809524	0.1666666667	0.1666666667	0.4210526316	0.5714285714
B4GALT	0.09523809524	0.09523809524	0	0	inf	9.52381E+31
B4GALT	0.09523809524	0.09523809524	0.05555555556	0.05555555556	1.473684211	1.714285714
B9D1	0.04761904762	0.04761904762	0.1111111111	0.1111111111	0.325	0.4285714286
B9D2	0.04761904762	0.04761904762	0.05555555556	0.05555555556	0.7	0.8571428571
BAALC	0.1904761905	0.1904761905	0.1111111111	0.1111111111	1.529411765	1.714285714
AALC-A	0.1904761905	0.1904761905	0.1111111111	0.1111111111	1.529411765	1.714285714
AALC-A	0.1904761905	0.1904761905	0.1111111111	0.1111111111	1.529411765	1.714285714
BAAT	0.04761904762	0.04761904762	0	0	inf	4.7619E+31
BABAM	0.1428571429	0.1428571429	0.1111111111	0.1111111111	1.083333333	1.285714286
BABAM	0	0	0.1111111111	0.1111111111	0	0
BAM2-A	0	0	0.1111111111	0.1111111111	0	0
BACE2	0.04761904762	0.04761904762	0.1111111111	0.1111111111	0.325	0.4285714286
BACH1	0.04761904762	0.04761904762	0.1111111111	0.1111111111	0.325	0.4285714286
ACH1-IT	0.04761904762	0.04761904762	0.1111111111	0.1111111111	0.325	0.4285714286
ACH1-IT	0.04761904762	0.04761904762	0.05555555556	0.05555555556	0.7	0.8571428571
BACH2	0	0	0	0		0.00001
BAG1	0.09523809524	0.09523809524	0.05555555556	0.05555555556	1.473684211	1.714285714
BAG2	0.04761904762	0.04761904762	0.05555555556	0.05555555556	0.7	0.8571428571
BAG3	0.2380952381	0.2380952381	0	0	inf	2.38095E+32
BAG4	0.09523809524	0.09523809524	0.1111111111	0.1111111111	0.6842105263	0.8571428571
BAG6	0.04761904762	0.04761904762	0	0	inf	4.7619E+31
BAGE	0.04761904762	0.04761904762	0	0	inf	4.7619E+31
BAGE2	0.04761904762	0.04761904762	0	0	inf	4.7619E+31
BAGE3	0.04761904762	0.04761904762	0	0	inf	4.7619E+31

BAGE4	0.04761904762	0.04761904762	0	0	inf	4.7619E+31
BAGE5	0.04761904762	0.04761904762	0	0	inf	4.7619E+31
BAHCC1	0.04761904762	0.04761904762	0	0	inf	4.7619E+31
BAIAP2	0.04761904762	0.04761904762	0	0	inf	4.7619E+31
AIAP2-D	0.04761904762	0.04761904762	0	0	inf	4.7619E+31
AIAP2L	0.04761904762	0.04761904762	0.05555555556	0.05555555556	0.7	0.8571428571
AIAP2L	0.04761904762	0.04761904762	0.1111111111	0.1111111111	0.325	0.4285714286
BAIAP3	0.04761904762	0.04761904762	0.2222222222	0.2222222222	0.1375	0.2142857143
BAK1	0.04761904762	0.04761904762	0	0	inf	4.7619E+31
BAMBI	0.09523809524	0.09523809524	0.1111111111	0.1111111111	0.6842105263	0.8571428571
BANCR	0	0	0.05555555556	0.05555555556	0	0
BANF2	0.1904761905	0.1904761905	0.1111111111	0.1111111111	1.529411765	1.714285714
BANK1	0	0	0.05555555556	0.05555555556	0	0
BANP	0.04761904762	0.04761904762	0	0	inf	4.7619E+31
BAP1	0.04761904762	0.04761904762	0	0	inf	4.7619E+31
BARD1	0	0	0.05555555556	0.05555555556	0	0
BARHL1	0.04761904762	0.04761904762	0	0	inf	4.7619E+31
BARHL2	0.04761904762	0.04761904762	0.05555555556	0.05555555556	0.7	0.8571428571
BARX1	0.04761904762	0.04761904762	0.05555555556	0.05555555556	0.7	0.8571428571
ARX1-D	0.04761904762	0.04761904762	0.05555555556	0.05555555556	0.7	0.8571428571
BASP1	0.04761904762	0.04761904762	0.05555555556	0.05555555556	0.7	0.8571428571
ASP1-AS	0.04761904762	0.04761904762	0.05555555556	0.05555555556	0.7	0.8571428571
BATF3	0.09523809524	0.09523809524	0	0	inf	9.52381E+31
BAX	0.09523809524	0.09523809524	0.1111111111	0.1111111111	0.6842105263	0.8571428571
BAZ1A	0.09523809524	0.09523809524	0	0	inf	9.52381E+31
BAZ1B	0.1428571429	0.1428571429	0.1111111111	0.1111111111	1.083333333	1.285714286
BAZ2A	0.1428571429	0.1428571429	0.05555555556	0.05555555556	2.333333333	2.571428571
BAZ2B	0	0	0.05555555556	0.05555555556	0	0
BBC3	0.09523809524	0.09523809524	0.1111111111	0.1111111111	0.6842105263	0.8571428571
BBIP1	0.1428571429	0.1428571429	0.05555555556	0.05555555556	2.333333333	2.571428571
BBOF1	0.09523809524	0.09523809524	0	0	inf	9.52381E+31
BBS10	0.04761904762	0.04761904762	0.1111111111	0.1111111111	0.325	0.4285714286
BBS12	0	0	0.05555555556	0.05555555556	0	0
BBS2	0.04761904762	0.04761904762	0	0	inf	4.7619E+31
BBS4	0.04761904762	0.04761904762	0.05555555556	0.05555555556	0.7	0.8571428571
BBS5	0	0	0.05555555556	0.05555555556	0	0
BBS7	0	0	0.05555555556	0.05555555556	0	0
BBS9	0.04761904762	0.04761904762	0.1666666667	0.1666666667	0.2	0.2857142857
BBX	0.09523809524	0.09523809524	0.05555555556	0.05555555556	1.473684211	1.714285714
BCAM	0.09523809524	0.09523809524	0.1111111111	0.1111111111	0.6842105263	0.8571428571
BCAN	0.1428571429	0.1428571429	0.1111111111	0.1111111111	1.083333333	1.285714286
BCAP29	0.04761904762	0.04761904762	0.05555555556	0.05555555556	0.7	0.8571428571
BCAR1	0.04761904762	0.04761904762	0	0	inf	4.7619E+31
BCAR3	0.04761904762	0.04761904762	0.05555555556	0.05555555556	0.7	0.8571428571
BCAR4	0.04761904762	0.04761904762	0	0	inf	4.7619E+31
BCAS1	0.09523809524	0.09523809524	0.1111111111	0.1111111111	0.6842105263	0.8571428571
BCAS2	0.04761904762	0.04761904762	0.05555555556	0.05555555556	0.7	0.8571428571
BCAS3	0.1428571429	0.1428571429	0	0	inf	1.42857E+32
BCAS4	0.09523809524	0.09523809524	0.1666666667	0.1666666667	0.4210526316	0.5714285714
BCAT1	0.04761904762	0.04761904762	0.05555555556	0.05555555556	0.7	0.8571428571
BCAT2	0.09523809524	0.09523809524	0.1111111111	0.1111111111	0.6842105263	0.8571428571

BCCIP	0.2380952381	0.2380952381	0.05555555556	0.05555555556	4.375	4.285714286
CDIN3I	0.04761904762	0.04761904762	0.05555555556	0.05555555556	0.7	0.8571428571
DIN3D-A	0.04761904762	0.04761904762	0.05555555556	0.05555555556	0.7	0.8571428571
BCHE	0.1428571429	0.1428571429	0.05555555556	0.05555555556	2.333333333	2.571428571
CKDHA	0.04761904762	0.04761904762	0.05555555556	0.05555555556	0.7	0.8571428571
BCKDK	0.04761904762	0.04761904762	0	0	inf	4.7619E+31
BCL10	0.04761904762	0.04761904762	0.05555555556	0.05555555556	0.7	0.8571428571
BCL11A	0.04761904762	0.04761904762	0.05555555556	0.05555555556	0.7	0.8571428571
BCL2	0.09523809524	0.09523809524	0	0	inf	9.52381E+31
BCL2A1	0.04761904762	0.04761904762	0.05555555556	0.05555555556	0.7	0.8571428571
BCL2L1	0.09523809524	0.09523809524	0.1111111111	0.1111111111	0.6842105263	0.8571428571
CL2L1	0.04761904762	0.04761904762	0.05555555556	0.05555555556	0.7	0.8571428571
CL2L1	0	0	0.05555555556	0.05555555556	0	0
CL2L1	0.09523809524	0.09523809524	0.1111111111	0.1111111111	0.6842105263	0.8571428571
CL2L1	0.04761904762	0.04761904762	0	0	inf	4.7619E+31
CL2L1	0.04761904762	0.04761904762	0.05555555556	0.05555555556	0.7	0.8571428571
CL2L1	0.04761904762	0.04761904762	0.05555555556	0.05555555556	0.7	0.8571428571
BCL2L2	0	0	0.1111111111	0.1111111111	0	0
2L2-PAE	0	0	0.1111111111	0.1111111111	0	0
BCL3	0.09523809524	0.09523809524	0.1111111111	0.1111111111	0.6842105263	0.8571428571
BCL6	0.09523809524	0.09523809524	0.1111111111	0.1111111111	0.6842105263	0.8571428571
BCL6B	0	0	0.05555555556	0.05555555556	0	0
BCL7A	0.04761904762	0.04761904762	0.1111111111	0.1111111111	0.325	0.4285714286
BCL7B	0.1428571429	0.1428571429	0.1111111111	0.1111111111	1.083333333	1.285714286
BCL7C	0.04761904762	0.04761904762	0	0	inf	4.7619E+31
BCL9	0.1428571429	0.1428571429	0.05555555556	0.05555555556	2.333333333	2.571428571
BCO1	0.04761904762	0.04761904762	0	0	inf	4.7619E+31
BCR	0	0	0.05555555556	0.05555555556	0	0
BCRP2	0.04761904762	0.04761904762	0.05555555556	0.05555555556	0.7	0.8571428571
BCRP3	0.04761904762	0.04761904762	0.1111111111	0.1111111111	0.325	0.4285714286
BCS1L	0	0	0.05555555556	0.05555555556	0	0
CYRN1	0	0	0.05555555556	0.05555555556	0	0
BDH1	0.1428571429	0.1428571429	0.05555555556	0.05555555556	2.333333333	2.571428571
BDH2	0	0	0.05555555556	0.05555555556	0	0
BDP1	0.04761904762	0.04761904762	0	0	inf	4.7619E+31
BEAN1	0.04761904762	0.04761904762	0	0	inf	4.7619E+31
EAN1-AS	0.04761904762	0.04761904762	0	0	inf	4.7619E+31
BECN1	0.04761904762	0.04761904762	0	0	inf	4.7619E+31
BECN2	0.1428571429	0.1428571429	0	0	inf	1.42857E+32
BEND3	0	0	0	0		0.00001
END3P	0.1428571429	0.1428571429	0.05555555556	0.05555555556	2.333333333	2.571428571
BEND4	0.04761904762	0.04761904762	0	0	inf	4.7619E+31
BEND5	0	0	0.05555555556	0.05555555556	0	0
BEND6	0.04761904762	0.04761904762	0.05555555556	0.05555555556	0.7	0.8571428571
BEND7	0.09523809524	0.09523809524	0.1111111111	0.1111111111	0.6842105263	0.8571428571
BEST2	0.1428571429	0.1428571429	0.1111111111	0.1111111111	1.083333333	1.285714286
BEST3	0.09523809524	0.09523809524	0.1111111111	0.1111111111	0.6842105263	0.8571428571
BEST4	0.04761904762	0.04761904762	0.05555555556	0.05555555556	0.7	0.8571428571
BET1	0.04761904762	0.04761904762	0.1111111111	0.1111111111	0.325	0.4285714286
BET1L	0.04761904762	0.04761904762	0	0	inf	4.7619E+31
BFAR	0.04761904762	0.04761904762	0	0	inf	4.7619E+31

**BFSP1**	0.1904761905	0.1904761905	0.1111111111	0.1111111111	1.529411765	1.714285714
**BFSP2**	0.1428571429	0.1428571429	0.05555555556	0.05555555556	2.333333333	2.571428571
**FSP2-AS**	0.1428571429	0.1428571429	0.05555555556	0.05555555556	2.333333333	2.571428571
**HLHA1**	0.04761904762	0.04761904762	0.05555555556	0.05555555556	0.7	0.8571428571
**BHLHA9**	0	0	0.1111111111	0.1111111111	0	0
**HLHE2**	0.1428571429	0.1428571429	0.1666666667	0.1666666667	0.6666666667	0.8571428571
**HLHE2**	0.1428571429	0.1428571429	0.2222222222	0.2222222222	0.4583333333	0.6428571429
**HLHE4**	0.04761904762	0.04761904762	0	0	inf	4.7619E+31
**LHE40-A**	0.04761904762	0.04761904762	0	0	inf	4.7619E+31
**HLHE4**	0.04761904762	0.04761904762	0.05555555556	0.05555555556	0.7	0.8571428571
**BHMG1**	0.09523809524	0.09523809524	0.1111111111	0.1111111111	0.6842105263	0.8571428571
**BHMT**	0.04761904762	0.04761904762	0	0	inf	4.7619E+31
**BHMT2**	0.04761904762	0.04761904762	0	0	inf	4.7619E+31
**BICC1**	0.09523809524	0.09523809524	0.05555555556	0.05555555556	1.473684211	1.714285714
**BICD1**	0.1428571429	0.1428571429	0.1111111111	0.1111111111	1.083333333	1.285714286
**BICD2**	0.04761904762	0.04761904762	0.05555555556	0.05555555556	0.7	0.8571428571
**BICDL1**	0	0	0.1111111111	0.1111111111	0	0
**BICDL2**	0.04761904762	0.04761904762	0.1111111111	0.1111111111	0.325	0.4285714286
**BICRA**	0.09523809524	0.09523809524	0.1111111111	0.1111111111	0.6842105263	0.8571428571
**BICRAL**	0.04761904762	0.04761904762	0.05555555556	0.05555555556	0.7	0.8571428571
**BID**	0.04761904762	0.04761904762	0.05555555556	0.05555555556	0.7	0.8571428571
**BIK**	0	0	0.1666666667	0.1666666667	0	0
**BIN1**	0	0	0.05555555556	0.05555555556	0	0
**BIN2**	0.09523809524	0.09523809524	0.05555555556	0.05555555556	1.473684211	1.714285714
**BIN3**	0.09523809524	0.09523809524	0.1111111111	0.1111111111	0.6842105263	0.8571428571
**BIN3-IT1**	0.09523809524	0.09523809524	0.1111111111	0.1111111111	0.6842105263	0.8571428571
**BIRC5**	0.04761904762	0.04761904762	0	0	inf	4.7619E+31
**BIRC6**	0.1428571429	0.1428571429	0	0	inf	1.42857E+32
**IRC6-AS**	0.1428571429	0.1428571429	0	0	inf	1.42857E+32
**BIRC7**	0.1428571429	0.1428571429	0.2222222222	0.2222222222	0.4583333333	0.6428571429
**BIRC8**	0.09523809524	0.09523809524	0.05555555556	0.05555555556	1.473684211	1.714285714
**BISPR**	0.1428571429	0.1428571429	0.1111111111	0.1111111111	1.083333333	1.285714286
**LACAT**	0.1904761905	0.1904761905	0	0	inf	1.90476E+32
**BLACE**	0.1428571429	0.1428571429	0.05555555556	0.05555555556	2.333333333	2.571428571
**BLCAP**	0.09523809524	0.09523809524	0.1666666667	0.1666666667	0.4210526316	0.5714285714
**BLK**	0.09523809524	0.09523809524	0.1111111111	0.1111111111	0.6842105263	0.8571428571
**BLM**	0.04761904762	0.04761904762	0.05555555556	0.05555555556	0.7	0.8571428571
**BLMH**	0	0	0.05555555556	0.05555555556	0	0
**BLNK**	0.1428571429	0.1428571429	0	0	inf	1.42857E+32
**BLOC1S**	0.04761904762	0.04761904762	0.05555555556	0.05555555556	0.7	0.8571428571
**OC1S1-R**	0.04761904762	0.04761904762	0.05555555556	0.05555555556	0.7	0.8571428571
**LOC1S**	0.1428571429	0.1428571429	0	0	inf	1.42857E+32
**LOC1S**	0.09523809524	0.09523809524	0.1111111111	0.1111111111	0.6842105263	0.8571428571
**LOC1S**	0.04761904762	0.04761904762	0	0	inf	4.7619E+31
**BLVRA**	0.04761904762	0.04761904762	0.1666666667	0.1666666667	0.2	0.2857142857
**BLVRB**	0.04761904762	0.04761904762	0.05555555556	0.05555555556	0.7	0.8571428571
**BLZF1**	0.1428571429	0.1428571429	0	0	inf	1.42857E+32
**MERB1**	0.04761904762	0.04761904762	0	0	inf	4.7619E+31
**BMF**	0	0	0	0		0.00001
**BMI1**	0.09523809524	0.09523809524	0.1111111111	0.1111111111	0.6842105263	0.8571428571
**BMP1**	0.09523809524	0.09523809524	0.1111111111	0.1111111111	0.6842105263	0.8571428571

**BMP10**	0	0	0.05555555556	0.05555555556	0	0
**BMP2**	0.1904761905	0.1904761905	0.1111111111	0.1111111111	1.529411765	1.714285714
**BMP2K**	0.04761904762	0.04761904762	0.05555555556	0.05555555556	0.7	0.8571428571
**BMP3**	0	0	0.05555555556	0.05555555556	0	0
**BMP4**	0.04761904762	0.04761904762	0	0	inf	4.7619E+31
**BMP5**	0.04761904762	0.04761904762	0.05555555556	0.05555555556	0.7	0.8571428571
**BMP7**	0.1428571429	0.1428571429	0.2222222222	0.2222222222	0.4583333333	0.6428571429
**MP7-AS**	0.1428571429	0.1428571429	0.2222222222	0.2222222222	0.4583333333	0.6428571429
**BMP8A**	0.04761904762	0.04761904762	0.05555555556	0.05555555556	0.7	0.8571428571
**BMP8B**	0.04761904762	0.04761904762	0.05555555556	0.05555555556	0.7	0.8571428571
**BMPER**	0.04761904762	0.04761904762	0.1666666667	0.1666666667	0.2	0.2857142857
**MPR1A**	0.1428571429	0.1428571429	0.05555555556	0.05555555556	2.333333333	2.571428571
**MPR1**	0	0	0.05555555556	0.05555555556	0	0
**MPR1B-**	0	0	0.05555555556	0.05555555556	0	0
**BMPR2**	0.09523809524	0.09523809524	0.05555555556	0.05555555556	1.473684211	1.714285714
**BMS1**	0.1428571429	0.1428571429	0.05555555556	0.05555555556	2.333333333	2.571428571
**BMS1P1**	0.1428571429	0.1428571429	0.05555555556	0.05555555556	2.333333333	2.571428571
**MS1P1**	0.09523809524	0.09523809524	0.2222222222	0.2222222222	0.2894736842	0.4285714286
**MS1P1**	0.1904761905	0.1904761905	0.1111111111	0.1111111111	1.529411765	1.714285714
**MS1P1**	0.1904761905	0.1904761905	0.1111111111	0.1111111111	1.529411765	1.714285714
**BMS1P2**	0.1428571429	0.1428571429	0.05555555556	0.05555555556	2.333333333	2.571428571
**1P2-AG**	0.1428571429	0.1428571429	0.05555555556	0.05555555556	2.333333333	2.571428571
**MS1P2**	0.04761904762	0.04761904762	0.05555555556	0.05555555556	0.7	0.8571428571
**MS1P2**	0.1428571429	0.1428571429	0	0	inf	1.42857E+32
**MS1P2**	0.1904761905	0.1904761905	0.1111111111	0.1111111111	1.529411765	1.714285714
**1P4-AG**	0.1904761905	0.1904761905	0.05555555556	0.05555555556	3.294117647	3.428571429
**BMT2**	0.04761904762	0.04761904762	0.05555555556	0.05555555556	0.7	0.8571428571
**BNC1**	0.04761904762	0.04761904762	0.05555555556	0.05555555556	0.7	0.8571428571
**BNC2**	0.04761904762	0.04761904762	0.05555555556	0.05555555556	0.7	0.8571428571
**NC2-AS**	0.04761904762	0.04761904762	0.05555555556	0.05555555556	0.7	0.8571428571
**BNIP1**	0	0	0.05555555556	0.05555555556	0	0
**BNIP2**	0.04761904762	0.04761904762	0.05555555556	0.05555555556	0.7	0.8571428571
**BNIP3**	0.2380952381	0.2380952381	0	0	inf	2.38095E+32
**BNIP3L**	0.04761904762	0.04761904762	0.1111111111	0.1111111111	0.325	0.4285714286
**BNIPL**	0.1904761905	0.1904761905	0.05555555556	0.05555555556	3.294117647	3.428571429
**BOC**	0.09523809524	0.09523809524	0.05555555556	0.05555555556	1.473684211	1.714285714
**BOD1**	0	0	0.05555555556	0.05555555556	0	0
**BOD1L1**	0	0	0	0		0.00001
**BOD1L2**	0.09523809524	0.09523809524	0	0	inf	9.52381E+31
**BOK**	0	0	0.05555555556	0.05555555556	0	0
**OK-AS**	0	0	0.05555555556	0.05555555556	0	0
**BOLA1**	0.1428571429	0.1428571429	0.05555555556	0.05555555556	2.333333333	2.571428571
**BOLA2**	0.04761904762	0.04761904762	0	0	inf	4.7619E+31
**A2-SM**	0.04761904762	0.04761904762	0	0	inf	4.7619E+31
**BOLA2B**	0.04761904762	0.04761904762	0	0	inf	4.7619E+31
**BOLA3**	0	0	0.05555555556	0.05555555556	0	0
**LA3-A**	0	0	0.05555555556	0.05555555556	0	0
**BOLL**	0	0	0.05555555556	0.05555555556	0	0
**BOP1**	0.04761904762	0.04761904762	0.1666666667	0.1666666667	0.2	0.2857142857
**BORCS**	0.04761904762	0.04761904762	0.05555555556	0.05555555556	0.7	0.8571428571
**BORCS**	0.04761904762	0.04761904762	0.05555555556	0.05555555556	0.7	0.8571428571

**BORCS7**	0.1428571429	0.1428571429	0.05555555556	0.05555555556	2.333333333	2.571428571
**RCS7-AS**	0.1428571429	0.1428571429	0.05555555556	0.05555555556	2.333333333	2.571428571
**BORCS8**	0.1428571429	0.1428571429	0.1111111111	0.1111111111	1.083333333	1.285714286
**CS8-ME**	0.1428571429	0.1428571429	0.1111111111	0.1111111111	1.083333333	1.285714286
**BPESC1**	0.1428571429	0.1428571429	0.05555555556	0.05555555556	2.333333333	2.571428571
**BPGM**	0.04761904762	0.04761904762	0.05555555556	0.05555555556	0.7	0.8571428571
**BPI**	0.1428571429	0.1428571429	0.1666666667	0.1666666667	0.6666666667	0.8571428571
**BPIFA1**	0.09523809524	0.09523809524	0.1111111111	0.1111111111	0.6842105263	0.8571428571
**BPIFA2**	0.09523809524	0.09523809524	0.1111111111	0.1111111111	0.6842105263	0.8571428571
**BPIFA3**	0.09523809524	0.09523809524	0.1111111111	0.1111111111	0.6842105263	0.8571428571
**BPIFA4P**	0.09523809524	0.09523809524	0.1111111111	0.1111111111	0.6842105263	0.8571428571
**BPIFB1**	0.09523809524	0.09523809524	0.1111111111	0.1111111111	0.6842105263	0.8571428571
**BPIFB2**	0.09523809524	0.09523809524	0.1111111111	0.1111111111	0.6842105263	0.8571428571
**BPIFB3**	0.09523809524	0.09523809524	0.1111111111	0.1111111111	0.6842105263	0.8571428571
**BPIFB4**	0.09523809524	0.09523809524	0.1111111111	0.1111111111	0.6842105263	0.8571428571
**BPIFB6**	0.09523809524	0.09523809524	0.1111111111	0.1111111111	0.6842105263	0.8571428571
**BPIFC**	0.04761904762	0.04761904762	0.1111111111	0.1111111111	0.325	0.4285714286
**BPNT1**	0.1428571429	0.1428571429	0.05555555556	0.05555555556	2.333333333	2.571428571
**BPTF**	0	0	0	0		0.00001
**BRAF**	0.04761904762	0.04761904762	0.1111111111	0.1111111111	0.325	0.4285714286
**BRAP**	0	0	0.1111111111	0.1111111111	0	0
**BRAT1**	0.04761904762	0.04761904762	0.1666666667	0.1666666667	0.2	0.2857142857
**BRCA1**	0.04761904762	0.04761904762	0	0	inf	4.7619E+31
**BRD1**	0	0	0.1111111111	0.1111111111	0	0
**BRD2**	0.04761904762	0.04761904762	0	0	inf	4.7619E+31
**BRD3**	0.04761904762	0.04761904762	0	0	inf	4.7619E+31
**BRD3OS**	0.04761904762	0.04761904762	0	0	inf	4.7619E+31
**BRD4**	0.1428571429	0.1428571429	0.1111111111	0.1111111111	1.083333333	1.285714286
**BRD7**	0.04761904762	0.04761904762	0	0	inf	4.7619E+31
**BRD8**	0.04761904762	0.04761904762	0	0	inf	4.7619E+31
**BRD9**	0	0	0.05555555556	0.05555555556	0	0
**BRDT**	0.04761904762	0.04761904762	0.05555555556	0.05555555556	0.7	0.8571428571
**BREA2**	0.04761904762	0.04761904762	0.1666666667	0.1666666667	0.2	0.2857142857
**BRF2**	0.04761904762	0.04761904762	0.1111111111	0.1111111111	0.325	0.4285714286
**BRI3**	0.04761904762	0.04761904762	0.05555555556	0.05555555556	0.7	0.8571428571
**BRI3BP**	0.04761904762	0.04761904762	0.05555555556	0.05555555556	0.7	0.8571428571
**BRICD5**	0.09523809524	0.09523809524	0.2222222222	0.2222222222	0.2894736842	0.4285714286
**BRINP1**	0.04761904762	0.04761904762	0	0	inf	4.7619E+31
**BRINP2**	0.1428571429	0.1428571429	0	0	inf	1.42857E+32
**BRINP3**	0.1428571429	0.1428571429	0	0	inf	1.42857E+32
**BRIP1**	0.09523809524	0.09523809524	0	0	inf	9.52381E+31
**BRIX1**	0.04761904762	0.04761904762	0.05555555556	0.05555555556	0.7	0.8571428571
**BRK1**	0.09523809524	0.09523809524	0	0	inf	9.52381E+31
**BRMS1L**	0.09523809524	0.09523809524	0	0	inf	9.52381E+31
**BROX**	0.1428571429	0.1428571429	0.05555555556	0.05555555556	2.333333333	2.571428571
**BRPF1**	0.09523809524	0.09523809524	0	0	inf	9.52381E+31
**BRPF3**	0.04761904762	0.04761904762	0.05555555556	0.05555555556	0.7	0.8571428571
**BRSK1**	0.09523809524	0.09523809524	0.05555555556	0.05555555556	1.473684211	1.714285714
**BRSK2**	0.04761904762	0.04761904762	0	0	inf	4.7619E+31
**BRWD1**	0.04761904762	0.04761904762	0.1111111111	0.1111111111	0.325	0.4285714286
**RWD1-A**	0.04761904762	0.04761904762	0.1111111111	0.1111111111	0.325	0.4285714286

RWD1-A	0.04761904762	0.04761904762	0.1111111111	0.1111111111	0.325	0.4285714286
BSDC1	0.04761904762	0.04761904762	0.1111111111	0.1111111111	0.325	0.4285714286
BSG	0.09523809524	0.09523809524	0.1111111111	0.1111111111	0.6842105263	0.8571428571
BSN	0.09523809524	0.09523809524	0	0	inf	9.52381E+31
BSN-DT	0.09523809524	0.09523809524	0	0	inf	9.52381E+31
BSND	0.04761904762	0.04761904762	0.05555555556	0.05555555556	0.7	0.8571428571
BSPH1	0.09523809524	0.09523809524	0.1111111111	0.1111111111	0.6842105263	0.8571428571
BSPRY	0.04761904762	0.04761904762	0.05555555556	0.05555555556	0.7	0.8571428571
BST1	0	0	0	0		0.00001
BST2	0.1428571429	0.1428571429	0.1111111111	0.1111111111	1.083333333	1.285714286
BTAF1	0.1428571429	0.1428571429	0.1111111111	0.1111111111	1.083333333	1.285714286
BTBD1	0.04761904762	0.04761904762	0.05555555556	0.05555555556	0.7	0.8571428571
BTBD11	0.04761904762	0.04761904762	0.05555555556	0.05555555556	0.7	0.8571428571
BTBD16	0.1904761905	0.1904761905	0.05555555556	0.05555555556	3.294117647	3.428571429
BTBD17	0.04761904762	0.04761904762	0	0	inf	4.7619E+31
BTBD19	0.04761904762	0.04761904762	0.05555555556	0.05555555556	0.7	0.8571428571
BTBD2	0.09523809524	0.09523809524	0.1111111111	0.1111111111	0.6842105263	0.8571428571
BTBD3	0.1904761905	0.1904761905	0.1111111111	0.1111111111	1.529411765	1.714285714
BTBD8	0.04761904762	0.04761904762	0.05555555556	0.05555555556	0.7	0.8571428571
BTBD9	0.04761904762	0.04761904762	0.05555555556	0.05555555556	0.7	0.8571428571
TBD9-AS	0.04761904762	0.04761904762	0.05555555556	0.05555555556	0.7	0.8571428571
BTC	0.09523809524	0.09523809524	0.05555555556	0.05555555556	1.473684211	1.714285714
BTD	0.04761904762	0.04761904762	0	0	inf	4.7619E+31
BTF3	0	0	0.05555555556	0.05555555556	0	0
BTF3L4	0.04761904762	0.04761904762	0.05555555556	0.05555555556	0.7	0.8571428571
BTF3P11	0	0	0.05555555556	0.05555555556	0	0
BTG1	0.04761904762	0.04761904762	0.05555555556	0.05555555556	0.7	0.8571428571
BTG2	0.1428571429	0.1428571429	0	0	inf	1.42857E+32
BTG3	0.04761904762	0.04761904762	0.1111111111	0.1111111111	0.325	0.4285714286
TG3-AS	0.04761904762	0.04761904762	0.1111111111	0.1111111111	0.325	0.4285714286
BTLA	0.09523809524	0.09523809524	0.05555555556	0.05555555556	1.473684211	1.714285714
BTN1A1	0	0	0.05555555556	0.05555555556	0	0
BTN2A1	0	0	0.05555555556	0.05555555556	0	0
BTN2A2	0	0	0.05555555556	0.05555555556	0	0
BTN2A3I	0	0	0.05555555556	0.05555555556	0	0
BTN3A1	0	0	0.05555555556	0.05555555556	0	0
BTN3A2	0	0	0.05555555556	0.05555555556	0	0
BTN3A3	0	0	0.05555555556	0.05555555556	0	0
BTNL10	0.1428571429	0.1428571429	0	0	inf	1.42857E+32
BTNL2	0.04761904762	0.04761904762	0	0	inf	4.7619E+31
BTNL3	0.09523809524	0.09523809524	0.05555555556	0.05555555556	1.473684211	1.714285714
BTNL8	0.09523809524	0.09523809524	0.05555555556	0.05555555556	1.473684211	1.714285714
BTNL9	0.09523809524	0.09523809524	0.05555555556	0.05555555556	1.473684211	1.714285714
BTRC	0.1428571429	0.1428571429	0	0	inf	1.42857E+32
BUB1	0	0	0.05555555556	0.05555555556	0	0
BUB3	0.2380952381	0.2380952381	0.05555555556	0.05555555556	4.375	4.285714286
BUD23	0.1428571429	0.1428571429	0.1111111111	0.1111111111	1.083333333	1.285714286
BUD31	0.04761904762	0.04761904762	0.05555555556	0.05555555556	0.7	0.8571428571
BYSL	0.04761904762	0.04761904762	0.05555555556	0.05555555556	0.7	0.8571428571
BZW1	0	0	0.05555555556	0.05555555556	0	0
BZW2	0.04761904762	0.04761904762	0.2222222222	0.2222222222	0.1375	0.2142857143

C10orf10	0.09523809524	0.09523809524	0	0	inf	9.52381E+31
C10orf11	0.09523809524	0.09523809524	0.1111111111	0.1111111111	0.6842105263	0.8571428571
C10orf12	0.2380952381	0.2380952381	0.05555555556	0.05555555556	4.375	4.285714286
C10orf12	0.09523809524	0.09523809524	0.1111111111	0.1111111111	0.6842105263	0.8571428571
C10orf14	0.1428571429	0.1428571429	0.05555555556	0.05555555556	2.333333333	2.571428571
C10orf14	0.3333333333	0.3333333333	0	0	inf	3.33333E+32
C10orf25	0.1428571429	0.1428571429	0.05555555556	0.05555555556	2.333333333	2.571428571
C10orf53	0.09523809524	0.09523809524	0.05555555556	0.05555555556	1.473684211	1.714285714
C10orf55	0.1904761905	0.1904761905	0.05555555556	0.05555555556	3.294117647	3.428571429
C10orf62	0.1428571429	0.1428571429	0	0	inf	1.42857E+32
C10orf67	0.09523809524	0.09523809524	0.1111111111	0.1111111111	0.6842105263	0.8571428571
C10orf71	0.09523809524	0.09523809524	0.05555555556	0.05555555556	1.473684211	1.714285714
0orf71-A	0.09523809524	0.09523809524	0.05555555556	0.05555555556	1.473684211	1.714285714
C10orf82	0.2380952381	0.2380952381	0	0	inf	2.38095E+32
C10orf88	0.2380952381	0.2380952381	0.05555555556	0.05555555556	4.375	4.285714286
C10orf90	0.2380952381	0.2380952381	0.05555555556	0.05555555556	4.375	4.285714286
C10orf91	0.2380952381	0.2380952381	0	0	inf	2.38095E+32
C10orf95	0.1428571429	0.1428571429	0.05555555556	0.05555555556	2.333333333	2.571428571
C10orf99	0.1428571429	0.1428571429	0	0	inf	1.42857E+32
C11orf21	0.04761904762	0.04761904762	0	0	inf	4.7619E+31
C11orf42	0.04761904762	0.04761904762	0	0	inf	4.7619E+31
C11orf49	0	0	0.05555555556	0.05555555556	0	0
C12orf10	0.04761904762	0.04761904762	0.05555555556	0.05555555556	0.7	0.8571428571
C12orf29	0.04761904762	0.04761904762	0.05555555556	0.05555555556	0.7	0.8571428571
C12orf4	0.04761904762	0.04761904762	0.1111111111	0.1111111111	0.325	0.4285714286
C12orf40	0.04761904762	0.04761904762	0.05555555556	0.05555555556	0.7	0.8571428571
C12orf42	0.04761904762	0.04761904762	0.05555555556	0.05555555556	0.7	0.8571428571
C12orf43	0.04761904762	0.04761904762	0.1111111111	0.1111111111	0.325	0.4285714286
C12orf45	0.04761904762	0.04761904762	0.05555555556	0.05555555556	0.7	0.8571428571
C12orf49	0	0	0.05555555556	0.05555555556	0	0
C12orf50	0.04761904762	0.04761904762	0.05555555556	0.05555555556	0.7	0.8571428571
C12orf54	0.04761904762	0.04761904762	0.05555555556	0.05555555556	0.7	0.8571428571
C12orf56	0.1428571429	0.1428571429	0.05555555556	0.05555555556	2.333333333	2.571428571
C12orf57	0.04761904762	0.04761904762	0.1111111111	0.1111111111	0.325	0.4285714286
C12orf60	0.04761904762	0.04761904762	0.1111111111	0.1111111111	0.325	0.4285714286
C12orf65	0.04761904762	0.04761904762	0.1111111111	0.1111111111	0.325	0.4285714286
C12orf66	0.1428571429	0.1428571429	0.05555555556	0.05555555556	2.333333333	2.571428571
C12orf71	0.04761904762	0.04761904762	0.05555555556	0.05555555556	0.7	0.8571428571
C12orf73	0.04761904762	0.04761904762	0.05555555556	0.05555555556	0.7	0.8571428571
C12orf74	0.04761904762	0.04761904762	0.05555555556	0.05555555556	0.7	0.8571428571
C12orf75	0.04761904762	0.04761904762	0.05555555556	0.05555555556	0.7	0.8571428571
C12orf76	0	0	0.1111111111	0.1111111111	0	0
C12orf77	0.04761904762	0.04761904762	0.05555555556	0.05555555556	0.7	0.8571428571
C12orf80	0.04761904762	0.04761904762	0.05555555556	0.05555555556	0.7	0.8571428571
C14orf11	0	0	0.05555555556	0.05555555556	0	0
C14orf28	0.04761904762	0.04761904762	0	0	inf	4.7619E+31
C14orf93	0	0	0.05555555556	0.05555555556	0	0
C15orf32	0.04761904762	0.04761904762	0.05555555556	0.05555555556	0.7	0.8571428571
C15orf39	0.04761904762	0.04761904762	0.05555555556	0.05555555556	0.7	0.8571428571
C15orf40	0.04761904762	0.04761904762	0.05555555556	0.05555555556	0.7	0.8571428571
C15orf41	0	0	0	0		0.00001

**C15orf48**	0.04761904762	0.04761904762	0	0	inf	4.7619E+31
**C15orf54**	0	0	0	0		0.00001
**C15orf61**	0.04761904762	0.04761904762	0.05555555556	0.05555555556	0.7	0.8571428571
**C15orf65**	0.04761904762	0.04761904762	0.05555555556	0.05555555556	0.7	0.8571428571
**C16orf46**	0.09523809524	0.09523809524	0	0	inf	9.52381E+31
**C16orf47**	0.04761904762	0.04761904762	0	0	inf	4.7619E+31
**C16orf54**	0.04761904762	0.04761904762	0	0	inf	4.7619E+31
**C16orf58**	0.04761904762	0.04761904762	0	0	inf	4.7619E+31
**C16orf70**	0.1428571429	0.1428571429	0.05555555556	0.05555555556	2.333333333	2.571428571
**C16orf71**	0.04761904762	0.04761904762	0	0	inf	4.7619E+31
**C16orf72**	0.09523809524	0.09523809524	0	0	inf	9.52381E+31
**C16orf74**	0.04761904762	0.04761904762	0	0	inf	4.7619E+31
**C16orf78**	0.04761904762	0.04761904762	0	0	inf	4.7619E+31
**C16orf82**	0.04761904762	0.04761904762	0	0	inf	4.7619E+31
**C16orf86**	0.1428571429	0.1428571429	0.05555555556	0.05555555556	2.333333333	2.571428571
**C16orf87**	0.04761904762	0.04761904762	0	0	inf	4.7619E+31
**C16orf89**	0.04761904762	0.04761904762	0	0	inf	4.7619E+31
**C16orf90**	0.04761904762	0.04761904762	0.1111111111	0.1111111111	0.325	0.4285714286
**C16orf91**	0.09523809524	0.09523809524	0.2222222222	0.2222222222	0.2894736842	0.4285714286
**C16orf92**	0.04761904762	0.04761904762	0	0	inf	4.7619E+31
**C16orf95**	0.04761904762	0.04761904762	0	0	inf	4.7619E+31
**C16orf96**	0.04761904762	0.04761904762	0	0	inf	4.7619E+31
**C16orf97**	0.04761904762	0.04761904762	0	0	inf	4.7619E+31
**C17orf10**	0	0	0.05555555556	0.05555555556	0	0
**C17orf10**	0.04761904762	0.04761904762	0	0	inf	4.7619E+31
**C17orf10**	0	0	0.1111111111	0.1111111111	0	0
**C17orf11**	0.04761904762	0.04761904762	0	0	inf	4.7619E+31
**C17orf11**	0.09523809524	0.09523809524	0	0	inf	9.52381E+31
**C17orf49**	0	0	0.05555555556	0.05555555556	0	0
**C17orf50**	0.09523809524	0.09523809524	0	0	inf	9.52381E+31
**C17orf51**	0.09523809524	0.09523809524	0.05555555556	0.05555555556	1.473684211	1.714285714
**C17orf53**	0.09523809524	0.09523809524	0	0	inf	9.52381E+31
**C17orf58**	0	0	0	0		0.00001
**C17orf64**	0.1428571429	0.1428571429	0	0	inf	1.42857E+32
**C17orf67**	0.09523809524	0.09523809524	0	0	inf	9.52381E+31
**C17orf75**	0	0	0	0		0.00001
**C17orf77**	0.04761904762	0.04761904762	0	0	inf	4.7619E+31
**C17orf80**	0.04761904762	0.04761904762	0	0	inf	4.7619E+31
**C17orf82**	0.09523809524	0.09523809524	0	0	inf	9.52381E+31
**C17orf97**	0	0	0.1111111111	0.1111111111	0	0
**C17orf98**	0.09523809524	0.09523809524	0	0	inf	9.52381E+31
**C17orf99**	0.04761904762	0.04761904762	0	0	inf	4.7619E+31
**C18orf12**	0.04761904762	0.04761904762	0.05555555556	0.05555555556	0.7	0.8571428571
**C18orf15**	0.09523809524	0.09523809524	0.05555555556	0.05555555556	1.473684211	1.714285714
**C18orf21**	0.04761904762	0.04761904762	0	0	inf	4.7619E+31
**C18orf25**	0.04761904762	0.04761904762	0.05555555556	0.05555555556	0.7	0.8571428571
**C18orf32**	0.04761904762	0.04761904762	0	0	inf	4.7619E+31
**C18orf54**	0.09523809524	0.09523809524	0	0	inf	9.52381E+31
**C18orf61**	0.09523809524	0.09523809524	0.05555555556	0.05555555556	1.473684211	1.714285714
**C18orf65**	0.09523809524	0.09523809524	0.05555555556	0.05555555556	1.473684211	1.714285714
**C19orf12**	0.04761904762	0.04761904762	0.1666666667	0.1666666667	0.2	0.2857142857

C19orf18	0.1428571429	0.1428571429	0.05555555556	0.05555555556	2.333333333	2.571428571
C19orf24	0.09523809524	0.09523809524	0.1111111111	0.1111111111	0.6842105263	0.8571428571
C19orf25	0.09523809524	0.09523809524	0.1111111111	0.1111111111	0.6842105263	0.8571428571
C19orf33	0.04761904762	0.04761904762	0.05555555556	0.05555555556	0.7	0.8571428571
C19orf38	0.1428571429	0.1428571429	0.1111111111	0.1111111111	1.083333333	1.285714286
C19orf44	0.1428571429	0.1428571429	0.1111111111	0.1111111111	1.083333333	1.285714286
C19orf47	0.04761904762	0.04761904762	0.05555555556	0.05555555556	0.7	0.8571428571
C19orf48	0.09523809524	0.09523809524	0.05555555556	0.05555555556	1.473684211	1.714285714
C19orf53	0.1428571429	0.1428571429	0.1111111111	0.1111111111	1.083333333	1.285714286
C19orf57	0.1428571429	0.1428571429	0.1111111111	0.1111111111	1.083333333	1.285714286
C19orf66	0.1428571429	0.1428571429	0.1111111111	0.1111111111	1.083333333	1.285714286
C19orf67	0.1428571429	0.1428571429	0.1111111111	0.1111111111	1.083333333	1.285714286
C19orf71	0.09523809524	0.09523809524	0.05555555556	0.05555555556	1.473684211	1.714285714
C19orf73	0.09523809524	0.09523809524	0.1111111111	0.1111111111	0.6842105263	0.8571428571
C19orf81	0.09523809524	0.09523809524	0.05555555556	0.05555555556	1.473684211	1.714285714
C1D	0	0	0.05555555556	0.05555555556	0	0
C1GALT	0.04761904762	0.04761904762	0.1666666667	0.1666666667	0.2	0.2857142857
GALT1C	0	0	0.05555555556	0.05555555556	0	0
C1QA	0.04761904762	0.04761904762	0.1111111111	0.1111111111	0.325	0.4285714286
C1QB	0.04761904762	0.04761904762	0.1111111111	0.1111111111	0.325	0.4285714286
C1QBP	0	0	0.05555555556	0.05555555556	0	0
C1QC	0.04761904762	0.04761904762	0.1111111111	0.1111111111	0.325	0.4285714286
C1QL1	0.1428571429	0.1428571429	0	0	inf	1.42857E+32
C1QL2	0	0	0.05555555556	0.05555555556	0	0
C1QL3	0.09523809524	0.09523809524	0.1111111111	0.1111111111	0.6842105263	0.8571428571
C1QL4	0.04761904762	0.04761904762	0.05555555556	0.05555555556	0.7	0.8571428571
C1QTNF	0.04761904762	0.04761904762	0	0	inf	4.7619E+31
QTNF1-A	0.04761904762	0.04761904762	0	0	inf	4.7619E+31
1QTNF1	0	0	0.1111111111	0.1111111111	0	0
C1QTNF	0.04761904762	0.04761904762	0.05555555556	0.05555555556	0.7	0.8571428571
C1QTNF	0.04761904762	0.04761904762	0.1111111111	0.1111111111	0.325	0.4285714286
NF3-AM	0.04761904762	0.04761904762	0.1111111111	0.1111111111	0.325	0.4285714286
C1QTNF	0	0	0.05555555556	0.05555555556	0	0
C1QTNF	0	0	0.1111111111	0.1111111111	0	0
C1QTNF	0	0	0	0		0.00001
C1QTNF	0.04761904762	0.04761904762	0.2222222222	0.2222222222	0.1375	0.2142857143
C1R	0.04761904762	0.04761904762	0.1111111111	0.1111111111	0.325	0.4285714286
C1RL	0.04761904762	0.04761904762	0.1111111111	0.1111111111	0.325	0.4285714286
1RL-AS	0.04761904762	0.04761904762	0.1111111111	0.1111111111	0.325	0.4285714286
C1S	0.04761904762	0.04761904762	0.1111111111	0.1111111111	0.325	0.4285714286
C1orf100	0.1428571429	0.1428571429	0.05555555556	0.05555555556	2.333333333	2.571428571
C1orf105	0.1428571429	0.1428571429	0.05555555556	0.05555555556	2.333333333	2.571428571
C1orf109	0.04761904762	0.04761904762	0.05555555556	0.05555555556	0.7	0.8571428571
C1orf112	0.1428571429	0.1428571429	0.05555555556	0.05555555556	2.333333333	2.571428571
C1orf115	0.1428571429	0.1428571429	0.05555555556	0.05555555556	2.333333333	2.571428571
C1orf116	0.1428571429	0.1428571429	0	0	inf	1.42857E+32
C1orf122	0.04761904762	0.04761904762	0.05555555556	0.05555555556	0.7	0.8571428571
C1orf127	0.04761904762	0.04761904762	0.1111111111	0.1111111111	0.325	0.4285714286
C1orf131	0.1428571429	0.1428571429	0	0	inf	1.42857E+32
C1orf137	0.04761904762	0.04761904762	0.05555555556	0.05555555556	0.7	0.8571428571
C1orf140	0.1904761905	0.1904761905	0.05555555556	0.05555555556	3.294117647	3.428571429

C1orf141	0.04761904762	0.04761904762	0.05555555556	0.05555555556	0.7	0.8571428571
C1orf143	0.1428571429	0.1428571429	0.05555555556	0.05555555556	2.333333333	2.571428571
C1orf158	0.04761904762	0.04761904762	0.1111111111	0.1111111111	0.325	0.4285714286
C1orf159	0	0	0.1111111111	0.1111111111	0	0
C1orf162	0.04761904762	0.04761904762	0	0	inf	4.7619E+31
C1orf167	0.04761904762	0.04761904762	0.1111111111	0.1111111111	0.325	0.4285714286
C1orf174	0	0	0	0		0.00001
C1orf185	0.04761904762	0.04761904762	0.05555555556	0.05555555556	0.7	0.8571428571
C1orf189	0.1904761905	0.1904761905	0.05555555556	0.05555555556	3.294117647	3.428571429
C1orf194	0.04761904762	0.04761904762	0.05555555556	0.05555555556	0.7	0.8571428571
C1orf195	0.04761904762	0.04761904762	0.1111111111	0.1111111111	0.325	0.4285714286
C1orf198	0.1428571429	0.1428571429	0	0	inf	1.42857E+32
C1orf21	0.1428571429	0.1428571429	0	0	inf	1.42857E+32
C1orf210	0.04761904762	0.04761904762	0.05555555556	0.05555555556	0.7	0.8571428571
C1orf216	0.04761904762	0.04761904762	0.05555555556	0.05555555556	0.7	0.8571428571
C1orf220	0.1428571429	0.1428571429	0	0	inf	1.42857E+32
C1orf226	0.1428571429	0.1428571429	0	0	inf	1.42857E+32
C1orf229	0.1428571429	0.1428571429	0	0	inf	1.42857E+32
C1orf232	0.04761904762	0.04761904762	0.1111111111	0.1111111111	0.325	0.4285714286
C1orf35	0.1428571429	0.1428571429	0	0	inf	1.42857E+32
C1orf43	0.1904761905	0.1904761905	0.05555555556	0.05555555556	3.294117647	3.428571429
C1orf50	0.04761904762	0.04761904762	0.05555555556	0.05555555556	0.7	0.8571428571
C1orf52	0.04761904762	0.04761904762	0.05555555556	0.05555555556	0.7	0.8571428571
C1orf53	0.1428571429	0.1428571429	0	0	inf	1.42857E+32
C1orf54	0.1904761905	0.1904761905	0.05555555556	0.05555555556	3.294117647	3.428571429
C1orf56	0.1904761905	0.1904761905	0.05555555556	0.05555555556	3.294117647	3.428571429
C1orf61	0.1428571429	0.1428571429	0.1111111111	0.1111111111	1.083333333	1.285714286
C1orf68	0.1904761905	0.1904761905	0	0	inf	1.90476E+32
C1orf74	0.1428571429	0.1428571429	0	0	inf	1.42857E+32
C1orf87	0.04761904762	0.04761904762	0.05555555556	0.05555555556	0.7	0.8571428571
C1orf94	0.04761904762	0.04761904762	0	0	inf	4.7619E+31
C2	0.04761904762	0.04761904762	0	0	inf	4.7619E+31
C2-AS1	0.04761904762	0.04761904762	0	0	inf	4.7619E+31
C20orf14	0.1904761905	0.1904761905	0.05555555556	0.05555555556	3.294117647	3.428571429
C20orf14	0.09523809524	0.09523809524	0.1111111111	0.1111111111	0.6842105263	0.8571428571
C20orf17	0.09523809524	0.09523809524	0.1111111111	0.1111111111	0.6842105263	0.8571428571
C20orf18	0.1904761905	0.1904761905	0.1666666667	0.1666666667	0.9411764706	1.142857143
C20orf19	0.1904761905	0.1904761905	0.05555555556	0.05555555556	3.294117647	3.428571429
C20orf19	0.1428571429	0.1428571429	0.1666666667	0.1666666667	0.6666666667	0.8571428571
C20orf20	0.1904761905	0.1904761905	0.1111111111	0.1111111111	1.529411765	1.714285714
C20orf20	0.09523809524	0.09523809524	0.1111111111	0.1111111111	0.6842105263	0.8571428571
C20orf20	0.1428571429	0.1428571429	0.2222222222	0.2222222222	0.4583333333	0.6428571429
C20orf78	0.1904761905	0.1904761905	0.05555555556	0.05555555556	3.294117647	3.428571429
C20orf85	0.1428571429	0.1428571429	0.2222222222	0.2222222222	0.4583333333	0.6428571429
C20orf96	0.1904761905	0.1904761905	0.05555555556	0.05555555556	3.294117647	3.428571429
C21orf58	0.04761904762	0.04761904762	0.1111111111	0.1111111111	0.325	0.4285714286
rf59-TC	0.04761904762	0.04761904762	0.1111111111	0.1111111111	0.325	0.4285714286
C21orf62	0.04761904762	0.04761904762	0.1111111111	0.1111111111	0.325	0.4285714286
1orf62-A	0.04761904762	0.04761904762	0.1111111111	0.1111111111	0.325	0.4285714286
C21orf91	0.04761904762	0.04761904762	0.05555555556	0.05555555556	0.7	0.8571428571
1orf91-O	0.04761904762	0.04761904762	0.05555555556	0.05555555556	0.7	0.8571428571

C22orf15	0.04761904762	0.04761904762	0.1111111111	0.1111111111	0.325	0.4285714286
C22orf23	0	0	0.1111111111	0.1111111111	0	0
C22orf24	0.04761904762	0.04761904762	0.1111111111	0.1111111111	0.325	0.4285714286
C22orf31	0.04761904762	0.04761904762	0.1111111111	0.1111111111	0.325	0.4285714286
C22orf34	0.04761904762	0.04761904762	0.1666666667	0.1666666667	0.2	0.2857142857
C22orf39	0.04761904762	0.04761904762	0.05555555556	0.05555555556	0.7	0.8571428571
C22orf42	0.04761904762	0.04761904762	0.1111111111	0.1111111111	0.325	0.4285714286
C22orf46	0	0	0.1111111111	0.1111111111	0	0
C2CD2	0.04761904762	0.04761904762	0.1111111111	0.1111111111	0.325	0.4285714286
C2CD4A	0.04761904762	0.04761904762	0.05555555556	0.05555555556	0.7	0.8571428571
C2CD4B	0.04761904762	0.04761904762	0.05555555556	0.05555555556	0.7	0.8571428571
C2CD4C	0.09523809524	0.09523809524	0.1111111111	0.1111111111	0.6842105263	0.8571428571
C2CD4D	0.1904761905	0.1904761905	0	0	inf	1.90476E+32
CD4D-A	0.1904761905	0.1904761905	0	0	inf	1.90476E+32
C2CD5	0.04761904762	0.04761904762	0.05555555556	0.05555555556	0.7	0.8571428571
C2CD6	0	0	0.05555555556	0.05555555556	0	0
C2orf15	0	0	0.05555555556	0.05555555556	0	0
C2orf27A	0	0	0.05555555556	0.05555555556	0	0
C2orf27B	0	0	0.05555555556	0.05555555556	0	0
C2orf40	0	0	0.05555555556	0.05555555556	0	0
C2orf42	0	0	0.05555555556	0.05555555556	0	0
C2orf48	0	0	0.05555555556	0.05555555556	0	0
C2orf49	0	0	0.05555555556	0.05555555556	0	0
C2orf50	0	0	0.05555555556	0.05555555556	0	0
C2orf66	0	0	0.05555555556	0.05555555556	0	0
C2orf68	0	0	0.05555555556	0.05555555556	0	0
C2orf69	0	0	0.05555555556	0.05555555556	0	0
C2orf72	0	0	0.05555555556	0.05555555556	0	0
C2orf73	0.04761904762	0.04761904762	0.05555555556	0.05555555556	0.7	0.8571428571
C2orf74	0.09523809524	0.09523809524	0.05555555556	0.05555555556	1.473684211	1.714285714
C2orf76	0	0	0.05555555556	0.05555555556	0	0
C2orf78	0	0	0.05555555556	0.05555555556	0	0
C2orf80	0	0	0.05555555556	0.05555555556	0	0
C2orf81	0	0	0.05555555556	0.05555555556	0	0
C2orf83	0	0	0.05555555556	0.05555555556	0	0
C2orf88	0	0	0.05555555556	0.05555555556	0	0
C2orf91	0	0	0.05555555556	0.05555555556	0	0
C2orf92	0	0	0.1111111111	0.1111111111	0	0
C3	0.1428571429	0.1428571429	0.05555555556	0.05555555556	2.333333333	2.571428571
C3AR1	0.04761904762	0.04761904762	0.1111111111	0.1111111111	0.325	0.4285714286
C3P1	0.1428571429	0.1428571429	0.1111111111	0.1111111111	1.083333333	1.285714286
C3orf14	0.04761904762	0.04761904762	0	0	inf	4.7619E+31
C3orf18	0.09523809524	0.09523809524	0	0	inf	9.52381E+31
C3orf20	0.04761904762	0.04761904762	0	0	inf	4.7619E+31
C3orf22	0.04761904762	0.04761904762	0.05555555556	0.05555555556	0.7	0.8571428571
C3orf33	0.1904761905	0.1904761905	0.05555555556	0.05555555556	3.294117647	3.428571429
C3orf35	0.04761904762	0.04761904762	0	0	inf	4.7619E+31
C3orf36	0.1428571429	0.1428571429	0.05555555556	0.05555555556	2.333333333	2.571428571
C3orf38	0.04761904762	0.04761904762	0	0	inf	4.7619E+31
C3orf49	0.04761904762	0.04761904762	0	0	inf	4.7619E+31
C3orf52	0.09523809524	0.09523809524	0.05555555556	0.05555555556	1.473684211	1.714285714

C3orf56	0.09523809524	0.09523809524	0.05555555556	0.05555555556	1.473684211	1.714285714
C3orf62	0.09523809524	0.09523809524	0	0	inf	9.52381E+31
C3orf67	0.04761904762	0.04761904762	0	0	inf	4.7619E+31
3orf67-A	0.04761904762	0.04761904762	0	0	inf	4.7619E+31
C3orf70	0.1428571429	0.1428571429	0.05555555556	0.05555555556	2.333333333	2.571428571
C3orf79	0.1904761905	0.1904761905	0.1111111111	0.1111111111	1.529411765	1.714285714
C3orf80	0.1428571429	0.1428571429	0.05555555556	0.05555555556	2.333333333	2.571428571
C3orf84	0.09523809524	0.09523809524	0	0	inf	9.52381E+31
C3orf85	0.09523809524	0.09523809524	0.05555555556	0.05555555556	1.473684211	1.714285714
C4A	0.04761904762	0.04761904762	0	0	inf	4.7619E+31
C4B	0.04761904762	0.04761904762	0	0	inf	4.7619E+31
C4BPA	0.1428571429	0.1428571429	0	0	inf	1.42857E+32
C4BPB	0.1428571429	0.1428571429	0	0	inf	1.42857E+32
C4B_2	0.04761904762	0.04761904762	0	0	inf	4.7619E+31
C4orf17	0	0	0.05555555556	0.05555555556	0	0
C4orf3	0	0	0.05555555556	0.05555555556	0	0
C4orf33	0	0	0.05555555556	0.05555555556	0	0
C4orf36	0	0	0.05555555556	0.05555555556	0	0
C4orf45	0	0	0.05555555556	0.05555555556	0	0
C4orf46	0	0	0.05555555556	0.05555555556	0	0
C4orf47	0	0	0.05555555556	0.05555555556	0	0
C4orf48	0.04761904762	0.04761904762	0	0	inf	4.7619E+31
C4orf51	0	0	0.05555555556	0.05555555556	0	0
C4orf54	0	0	0.05555555556	0.05555555556	0	0
C5	0.09523809524	0.09523809524	0.05555555556	0.05555555556	1.473684211	1.714285714
C5-OT1	0.09523809524	0.09523809524	0.05555555556	0.05555555556	1.473684211	1.714285714
C5AR1	0.09523809524	0.09523809524	0.1111111111	0.1111111111	0.6842105263	0.8571428571
C5AR2	0.09523809524	0.09523809524	0.1111111111	0.1111111111	0.6842105263	0.8571428571
C5orf15	0.04761904762	0.04761904762	0	0	inf	4.7619E+31
C5orf17	0.04761904762	0.04761904762	0.05555555556	0.05555555556	0.7	0.8571428571
C5orf22	0.04761904762	0.04761904762	0.05555555556	0.05555555556	0.7	0.8571428571
C5orf24	0.04761904762	0.04761904762	0	0	inf	4.7619E+31
C5orf30	0.04761904762	0.04761904762	0	0	inf	4.7619E+31
C5orf34	0.04761904762	0.04761904762	0.05555555556	0.05555555556	0.7	0.8571428571
C5orf38	0	0	0.1111111111	0.1111111111	0	0
C5orf47	0	0	0.05555555556	0.05555555556	0	0
C5orf49	0	0	0.05555555556	0.05555555556	0	0
C5orf51	0.04761904762	0.04761904762	0.05555555556	0.05555555556	0.7	0.8571428571
C5orf52	0.04761904762	0.04761904762	0.05555555556	0.05555555556	0.7	0.8571428571
C5orf56	0.04761904762	0.04761904762	0	0	inf	4.7619E+31
C5orf58	0	0	0.05555555556	0.05555555556	0	0
C5orf60	0.09523809524	0.09523809524	0.05555555556	0.05555555556	1.473684211	1.714285714
C5orf63	0.04761904762	0.04761904762	0	0	inf	4.7619E+31
C5orf64	0.04761904762	0.04761904762	0	0	inf	4.7619E+31
5orf64-A	0.04761904762	0.04761904762	0	0	inf	4.7619E+31
C5orf66	0.04761904762	0.04761904762	0	0	inf	4.7619E+31
5orf66-A	0.04761904762	0.04761904762	0	0	inf	4.7619E+31
C5orf67	0.04761904762	0.04761904762	0.05555555556	0.05555555556	0.7	0.8571428571
C6	0.04761904762	0.04761904762	0.05555555556	0.05555555556	0.7	0.8571428571
C6orf132	0.04761904762	0.04761904762	0.05555555556	0.05555555556	0.7	0.8571428571
C6orf203	0	0	0	0		0.00001

C6orf222	0.04761904762	0.04761904762	0.05555555556	0.05555555556	0.7	0.8571428571
C6orf223	0.04761904762	0.04761904762	0.05555555556	0.05555555556	0.7	0.8571428571
C6orf226	0.04761904762	0.04761904762	0.05555555556	0.05555555556	0.7	0.8571428571
C6orf47	0.04761904762	0.04761904762	0	0	inf	4.7619E+31
C6orf48	0.04761904762	0.04761904762	0	0	inf	4.7619E+31
C6orf62	0	0	0.05555555556	0.05555555556	0	0
C6orf89	0.04761904762	0.04761904762	0.05555555556	0.05555555556	0.7	0.8571428571
C7	0.04761904762	0.04761904762	0.05555555556	0.05555555556	0.7	0.8571428571
C7orf25	0.04761904762	0.04761904762	0.2222222222	0.2222222222	0.1375	0.2142857143
C7orf26	0.04761904762	0.04761904762	0.1666666667	0.1666666667	0.2	0.2857142857
C7orf31	0.04761904762	0.04761904762	0.2222222222	0.2222222222	0.1375	0.2142857143
C7orf33	0.09523809524	0.09523809524	0.05555555556	0.05555555556	1.473684211	1.714285714
C7orf50	0.04761904762	0.04761904762	0.1666666667	0.1666666667	0.2	0.2857142857
C7orf57	0.04761904762	0.04761904762	0.2222222222	0.2222222222	0.1375	0.2142857143
C7orf61	0.04761904762	0.04761904762	0.05555555556	0.05555555556	0.7	0.8571428571
C7orf65	0.04761904762	0.04761904762	0.2222222222	0.2222222222	0.1375	0.2142857143
C7orf66	0.04761904762	0.04761904762	0.05555555556	0.05555555556	0.7	0.8571428571
C7orf69	0.04761904762	0.04761904762	0.2222222222	0.2222222222	0.1375	0.2142857143
C7orf71	0.04761904762	0.04761904762	0.2222222222	0.2222222222	0.1375	0.2142857143
C7orf77	0.04761904762	0.04761904762	0.05555555556	0.05555555556	0.7	0.8571428571
C8A	0.04761904762	0.04761904762	0.05555555556	0.05555555556	0.7	0.8571428571
C8B	0.04761904762	0.04761904762	0.05555555556	0.05555555556	0.7	0.8571428571
C8G	0.1428571429	0.1428571429	0.05555555556	0.05555555556	2.333333333	2.571428571
C8orf31	0.04761904762	0.04761904762	0.1666666667	0.1666666667	0.2	0.2857142857
C8orf33	0.09523809524	0.09523809524	0.2222222222	0.2222222222	0.2894736842	0.4285714286
C8orf34	0.1428571429	0.1428571429	0.1666666667	0.1666666667	0.6666666667	0.8571428571
8orf34-A	0.1428571429	0.1428571429	0.1666666667	0.1666666667	0.6666666667	0.8571428571
C8orf37	0.1904761905	0.1904761905	0.1111111111	0.1111111111	1.529411765	1.714285714
8orf37-A	0.1904761905	0.1904761905	0.1111111111	0.1111111111	1.529411765	1.714285714
C8orf44	0.1428571429	0.1428571429	0.1666666667	0.1666666667	0.6666666667	0.8571428571
orf44-SG	0.1428571429	0.1428571429	0.1666666667	0.1666666667	0.6666666667	0.8571428571
C8orf48	0.09523809524	0.09523809524	0.1111111111	0.1111111111	0.6842105263	0.8571428571
C8orf49	0.09523809524	0.09523809524	0.1111111111	0.1111111111	0.6842105263	0.8571428571
C8orf58	0.09523809524	0.09523809524	0.1111111111	0.1111111111	0.6842105263	0.8571428571
C8orf74	0.09523809524	0.09523809524	0.1111111111	0.1111111111	0.6842105263	0.8571428571
C8orf76	0.1428571429	0.1428571429	0.1111111111	0.1111111111	1.083333333	1.285714286
C8orf82	0.04761904762	0.04761904762	0.1666666667	0.1666666667	0.2	0.2857142857
C8orf86	0.09523809524	0.09523809524	0.1111111111	0.1111111111	0.6842105263	0.8571428571
C8orf87	0.1904761905	0.1904761905	0.1111111111	0.1111111111	1.529411765	1.714285714
C8orf88	0.1904761905	0.1904761905	0.1111111111	0.1111111111	1.529411765	1.714285714
C8orf89	0.1904761905	0.1904761905	0.1666666667	0.1666666667	0.9411764706	1.142857143
C9	0.04761904762	0.04761904762	0.05555555556	0.05555555556	0.7	0.8571428571
C9orf106	0.09523809524	0.09523809524	0.05555555556	0.05555555556	1.473684211	1.714285714
C9orf116	0.04761904762	0.04761904762	0	0	inf	4.7619E+31
C9orf129	0.04761904762	0.04761904762	0.05555555556	0.05555555556	0.7	0.8571428571
C9orf131	0.09523809524	0.09523809524	0.05555555556	0.05555555556	1.473684211	1.714285714
C9orf135	0	0	0.05555555556	0.05555555556	0	0
9orf135-I	0	0	0.05555555556	0.05555555556	0	0
C9orf139	0.1428571429	0.1428571429	0.05555555556	0.05555555556	2.333333333	2.571428571
C9orf147	0.04761904762	0.04761904762	0.05555555556	0.05555555556	0.7	0.8571428571
C9orf152	0.04761904762	0.04761904762	0.05555555556	0.05555555556	0.7	0.8571428571

C9orf153	0	0	0.05555555556	0.05555555556	0	0
C9orf16	0.09523809524	0.09523809524	0.05555555556	0.05555555556	1.473684211	1.714285714
C9orf163	0.1428571429	0.1428571429	0.05555555556	0.05555555556	2.333333333	2.571428571
C9orf170	0	0	0.05555555556	0.05555555556	0	0
C9orf24	0.09523809524	0.09523809524	0.05555555556	0.05555555556	1.473684211	1.714285714
C9orf40	0	0	0.05555555556	0.05555555556	0	0
C9orf43	0.04761904762	0.04761904762	0.05555555556	0.05555555556	0.7	0.8571428571
C9orf47	0	0	0.05555555556	0.05555555556	0	0
C9orf50	0.09523809524	0.09523809524	0.05555555556	0.05555555556	1.473684211	1.714285714
C9orf57	0	0	0.05555555556	0.05555555556	0	0
C9orf62	0.04761904762	0.04761904762	0	0	inf	4.7619E+31
C9orf64	0	0	0.05555555556	0.05555555556	0	0
C9orf66	0.04761904762	0.04761904762	0.05555555556	0.05555555556	0.7	0.8571428571
C9orf72	0.04761904762	0.04761904762	0.05555555556	0.05555555556	0.7	0.8571428571
C9orf78	0.09523809524	0.09523809524	0.05555555556	0.05555555556	1.473684211	1.714285714
C9orf85	0	0	0.05555555556	0.05555555556	0	0
C9orf92	0.04761904762	0.04761904762	0.05555555556	0.05555555556	0.7	0.8571428571
CA1	0.1904761905	0.1904761905	0.1111111111	0.1111111111	1.529411765	1.714285714
CA10	0.04761904762	0.04761904762	0	0	inf	4.7619E+31
CA11	0.09523809524	0.09523809524	0.1111111111	0.1111111111	0.6842105263	0.8571428571
CA12	0.04761904762	0.04761904762	0.05555555556	0.05555555556	0.7	0.8571428571
CA13	0.1904761905	0.1904761905	0.1111111111	0.1111111111	1.529411765	1.714285714
CA14	0.1904761905	0.1904761905	0.05555555556	0.05555555556	3.294117647	3.428571429
CA2	0.1904761905	0.1904761905	0.1111111111	0.1111111111	1.529411765	1.714285714
CA3	0.1904761905	0.1904761905	0.1111111111	0.1111111111	1.529411765	1.714285714
CA3-AS1	0.1904761905	0.1904761905	0.1111111111	0.1111111111	1.529411765	1.714285714
CA4	0.1428571429	0.1428571429	0	0	inf	1.42857E+32
CA5A	0.04761904762	0.04761904762	0	0	inf	4.7619E+31
CA6	0	0	0.05555555556	0.05555555556	0	0
CA7	0.09523809524	0.09523809524	0.05555555556	0.05555555556	1.473684211	1.714285714
CA8	0.1428571429	0.1428571429	0.1666666667	0.1666666667	0.6666666667	0.8571428571
CA9	0.09523809524	0.09523809524	0.05555555556	0.05555555556	1.473684211	1.714285714
CAAP1	0.04761904762	0.04761904762	0.05555555556	0.05555555556	0.7	0.8571428571
CAB39	0	0	0.05555555556	0.05555555556	0	0
ABCOC	0.04761904762	0.04761904762	0.1666666667	0.1666666667	0.2	0.2857142857
CABIN1	0.04761904762	0.04761904762	0.1111111111	0.1111111111	0.325	0.4285714286
CABLES	0.04761904762	0.04761904762	0.05555555556	0.05555555556	0.7	0.8571428571
CABLES	0.1428571429	0.1428571429	0.2222222222	0.2222222222	0.4583333333	0.6428571429
CABP1	0	0	0.1111111111	0.1111111111	0	0
CABP5	0.09523809524	0.09523809524	0.1111111111	0.1111111111	0.6842105263	0.8571428571
CABP7	0.04761904762	0.04761904762	0.1111111111	0.1111111111	0.325	0.4285714286
CABS1	0.04761904762	0.04761904762	0.05555555556	0.05555555556	0.7	0.8571428571
CABYR	0.09523809524	0.09523809524	0.05555555556	0.05555555556	1.473684211	1.714285714
CACFD1	0.04761904762	0.04761904762	0	0	inf	4.7619E+31
CACHD1	0.04761904762	0.04761904762	0.05555555556	0.05555555556	0.7	0.8571428571
ACNA1	0.1428571429	0.1428571429	0.1111111111	0.1111111111	1.083333333	1.285714286
ACNA1	0.09523809524	0.09523809524	0	0	inf	9.52381E+31
ACNA1	0.04761904762	0.04761904762	0.1666666667	0.1666666667	0.2	0.2857142857
CNA1C-	0.04761904762	0.04761904762	0.1666666667	0.1666666667	0.2	0.2857142857
CNA1C-	0.04761904762	0.04761904762	0.1666666667	0.1666666667	0.2	0.2857142857
CNA1C-	0.04761904762	0.04761904762	0.1666666667	0.1666666667	0.2	0.2857142857

CNA1C-	0.04761904762	0.04761904762	0.1666666667	0.1666666667	0.2	0.2857142857
CNA1C-	0.04761904762	0.04761904762	0.1666666667	0.1666666667	0.2	0.2857142857
ACNA1	0.04761904762	0.04761904762	0	0	inf	4.7619E+31
ACNA1	0.1428571429	0.1428571429	0	0	inf	1.42857E+32
ACNA1	0.09523809524	0.09523809524	0	0	inf	9.52381E+31
CNA1G-	0.09523809524	0.09523809524	0	0	inf	9.52381E+31
ACNA1	0.04761904762	0.04761904762	0.2222222222	0.2222222222	0.1375	0.2142857143
CACNA1	0	0	0.1111111111	0.1111111111	0	0
CACNA1	0.1428571429	0.1428571429	0	0	inf	1.42857E+32
ACNA2I	0.04761904762	0.04761904762	0.1666666667	0.1666666667	0.2	0.2857142857
ACNA2I	0.09523809524	0.09523809524	0	0	inf	9.52381E+31
ACNA2I	0.04761904762	0.04761904762	0	0	inf	4.7619E+31
NA2D3-	0.04761904762	0.04761904762	0	0	inf	4.7619E+31
ACNA2I	0.04761904762	0.04761904762	0.1666666667	0.1666666667	0.2	0.2857142857
CACNB1	0.09523809524	0.09523809524	0	0	inf	9.52381E+31
CACNB2	0.09523809524	0.09523809524	0.1111111111	0.1111111111	0.6842105263	0.8571428571
CACNB3	0.04761904762	0.04761904762	0.05555555556	0.05555555556	0.7	0.8571428571
CACNB4	0	0	0.05555555556	0.05555555556	0	0
CACNG1	0	0	0	0		0.00001
CACNG2	0	0	0.1111111111	0.1111111111	0	0
CACNG3	0.04761904762	0.04761904762	0	0	inf	4.7619E+31
CACNG4	0	0	0	0		0.00001
CACNG5	0	0	0	0		0.00001
CACNG6	0.09523809524	0.09523809524	0.05555555556	0.05555555556	1.473684211	1.714285714
CACNG7	0.09523809524	0.09523809524	0.05555555556	0.05555555556	1.473684211	1.714285714
CACNG8	0.09523809524	0.09523809524	0.05555555556	0.05555555556	1.473684211	1.714285714
CACTIN	0.09523809524	0.09523809524	0.05555555556	0.05555555556	1.473684211	1.714285714
CTIN-A	0.09523809524	0.09523809524	0.05555555556	0.05555555556	1.473684211	1.714285714
CACUL1	0.2380952381	0.2380952381	0	0	inf	2.38095E+32
CACYBI	0.1428571429	0.1428571429	0	0	inf	1.42857E+32
CADM2	0.04761904762	0.04761904762	0	0	inf	4.7619E+31
DM2-A	0.04761904762	0.04761904762	0	0	inf	4.7619E+31
CADM3	0.1428571429	0.1428571429	0	0	inf	1.42857E+32
DM3-A	0.1428571429	0.1428571429	0	0	inf	1.42857E+32
CADM4	0.09523809524	0.09523809524	0.05555555556	0.05555555556	1.473684211	1.714285714
CADPS	0.04761904762	0.04761904762	0	0	inf	4.7619E+31
CADPS2	0.04761904762	0.04761904762	0.05555555556	0.05555555556	0.7	0.8571428571
CAHM	0	0	0.1111111111	0.1111111111	0	0
CALB1	0.1904761905	0.1904761905	0.1111111111	0.1111111111	1.529411765	1.714285714
CALB2	0.04761904762	0.04761904762	0	0	inf	4.7619E+31
LCOC	0.04761904762	0.04761904762	0.05555555556	0.05555555556	0.7	0.8571428571
LCOC	0.1428571429	0.1428571429	0	0	inf	1.42857E+32
CALCR	0.04761904762	0.04761904762	0.1111111111	0.1111111111	0.325	0.4285714286
CALCRI	0	0	0.05555555556	0.05555555556	0	0
CALD1	0.04761904762	0.04761904762	0.05555555556	0.05555555556	0.7	0.8571428571
CALHM	0.1428571429	0.1428571429	0.05555555556	0.05555555556	2.333333333	2.571428571
CALHM	0.1428571429	0.1428571429	0.05555555556	0.05555555556	2.333333333	2.571428571
CALHM	0.1428571429	0.1428571429	0.05555555556	0.05555555556	2.333333333	2.571428571
CALM2	0	0	0.05555555556	0.05555555556	0	0
CALM3	0.09523809524	0.09523809524	0.1111111111	0.1111111111	0.6842105263	0.8571428571
CALML	0	0	0.1666666667	0.1666666667	0	0

LML3-A	0	0	0.1666666667	0.1666666667	0	0
CALML4	0.04761904762	0.04761904762	0.05555555556	0.05555555556	0.7	0.8571428571
CALML5	0	0	0.1666666667	0.1666666667	0	0
CALML6	0	0	0.1111111111	0.1111111111	0	0
CALN1	0.1428571429	0.1428571429	0.1111111111	0.1111111111	1.083333333	1.285714286
CALR	0.1428571429	0.1428571429	0.1111111111	0.1111111111	1.083333333	1.285714286
CALR3	0.1428571429	0.1428571429	0.1111111111	0.1111111111	1.083333333	1.285714286
CALU	0.04761904762	0.04761904762	0.05555555556	0.05555555556	0.7	0.8571428571
CALY	0.1904761905	0.1904761905	0	0	inf	1.90476E+32
CAMK1	0.09523809524	0.09523809524	0	0	inf	9.52381E+31
CAMK1I	0.09523809524	0.09523809524	0.1111111111	0.1111111111	0.6842105263	0.8571428571
CAMK1C	0.1428571429	0.1428571429	0	0	inf	1.42857E+32
CAMK2I	0.04761904762	0.04761904762	0.1666666667	0.1666666667	0.2	0.2857142857
CAMK2I	0	0	0.05555555556	0.05555555556	0	0
CAMK2C	0.1904761905	0.1904761905	0.05555555556	0.05555555556	3.294117647	3.428571429
AMK2N	0.04761904762	0.04761904762	0.1111111111	0.1111111111	0.325	0.4285714286
AMK2N	0.1428571429	0.1428571429	0.05555555556	0.05555555556	2.333333333	2.571428571
CAMK4	0.04761904762	0.04761904762	0	0	inf	4.7619E+31
CAMKK	0	0	0.1111111111	0.1111111111	0	0
CAMKK	0.04761904762	0.04761904762	0.1111111111	0.1111111111	0.325	0.4285714286
AMKMT	0	0	0.05555555556	0.05555555556	0	0
CAMKV	0.09523809524	0.09523809524	0	0	inf	9.52381E+31
CAMLG	0.04761904762	0.04761904762	0	0	inf	4.7619E+31
CAMP	0.09523809524	0.09523809524	0	0	inf	9.52381E+31
AMSAP	0.04761904762	0.04761904762	0	0	inf	4.7619E+31
AMSAP	0.1428571429	0.1428571429	0	0	inf	1.42857E+32
AMSAP	0.1428571429	0.1428571429	0.05555555556	0.05555555556	2.333333333	2.571428571
CAMTA1	0	0	0.05555555556	0.05555555556	0	0
AMTA1-I	0	0	0.05555555556	0.05555555556	0	0
CAMTA2	0	0	0.1111111111	0.1111111111	0	0
CAND1	0.09523809524	0.09523809524	0.05555555556	0.05555555556	1.473684211	1.714285714
CAND2	0.09523809524	0.09523809524	0	0	inf	9.52381E+31
CANT1	0.04761904762	0.04761904762	0	0	inf	4.7619E+31
CANX	0.09523809524	0.09523809524	0.05555555556	0.05555555556	1.473684211	1.714285714
CAP1	0.04761904762	0.04761904762	0.05555555556	0.05555555556	0.7	0.8571428571
CAPG	0	0	0.05555555556	0.05555555556	0	0
CAPN10	0	0	0.05555555556	0.05555555556	0	0
APN10-I	0	0	0.05555555556	0.05555555556	0	0
CAPN11	0.04761904762	0.04761904762	0.05555555556	0.05555555556	0.7	0.8571428571
CAPN12	0.04761904762	0.04761904762	0.05555555556	0.05555555556	0.7	0.8571428571
CAPN13	0	0	0.05555555556	0.05555555556	0	0
CAPN14	0	0	0.05555555556	0.05555555556	0	0
CAPN15	0.04761904762	0.04761904762	0.2222222222	0.2222222222	0.1375	0.2142857143
CAPN2	0.1428571429	0.1428571429	0.05555555556	0.05555555556	2.333333333	2.571428571
CAPN7	0.04761904762	0.04761904762	0	0	inf	4.7619E+31
CAPN8	0.1428571429	0.1428571429	0.05555555556	0.05555555556	2.333333333	2.571428571
CAPN9	0.1428571429	0.1428571429	0	0	inf	1.42857E+32
CAPNS1	0.04761904762	0.04761904762	0.05555555556	0.05555555556	0.7	0.8571428571
CAPNS2	0.04761904762	0.04761904762	0	0	inf	4.7619E+31
CAPRIN	0.04761904762	0.04761904762	0.05555555556	0.05555555556	0.7	0.8571428571
CAPS	0.1428571429	0.1428571429	0.05555555556	0.05555555556	2.333333333	2.571428571

CAPSL	0.04761904762	0.04761904762	0.05555555556	0.05555555556	0.7	0.8571428571
CAPZA1	0.04761904762	0.04761904762	0.05555555556	0.05555555556	0.7	0.8571428571
CAPZA2	0.09523809524	0.09523809524	0.2222222222	0.2222222222	0.2894736842	0.4285714286
CAPZA3	0.04761904762	0.04761904762	0.1111111111	0.1111111111	0.325	0.4285714286
CAPZB	0.04761904762	0.04761904762	0.1111111111	0.1111111111	0.325	0.4285714286
CARD10	0	0	0.1111111111	0.1111111111	0	0
CARD11	0.09523809524	0.09523809524	0.1666666667	0.1666666667	0.4210526316	0.5714285714
CARD14	0.04761904762	0.04761904762	0	0	inf	4.7619E+31
CARD19	0.04761904762	0.04761904762	0.05555555556	0.05555555556	0.7	0.8571428571
CARD6	0.04761904762	0.04761904762	0.05555555556	0.05555555556	0.7	0.8571428571
CARD8	0.09523809524	0.09523809524	0.1111111111	0.1111111111	0.6842105263	0.8571428571
ARD8-A	0.09523809524	0.09523809524	0.1111111111	0.1111111111	0.6842105263	0.8571428571
CARD9	0.1428571429	0.1428571429	0.05555555556	0.05555555556	2.333333333	2.571428571
CARF	0.09523809524	0.09523809524	0.05555555556	0.05555555556	1.473684211	1.714285714
ARHSP	0.09523809524	0.09523809524	0	0	inf	9.52381E+31
CARM1	0.1428571429	0.1428571429	0.1111111111	0.1111111111	1.083333333	1.285714286
ARMIL	0	0	0.05555555556	0.05555555556	0	0
ARMIL	0.1428571429	0.1428571429	0.05555555556	0.05555555556	2.333333333	2.571428571
ARNMT	0	0	0.05555555556	0.05555555556	0	0
RNMT1-	0	0	0.05555555556	0.05555555556	0	0
CARS	0.04761904762	0.04761904762	0	0	inf	4.7619E+31
ARS-AS	0.04761904762	0.04761904762	0	0	inf	4.7619E+31
CASC1	0.04761904762	0.04761904762	0.05555555556	0.05555555556	0.7	0.8571428571
CASC10	0.09523809524	0.09523809524	0.1111111111	0.1111111111	0.6842105263	0.8571428571
CASC11	0.09523809524	0.09523809524	0.1111111111	0.1111111111	0.6842105263	0.8571428571
CASC16	0.04761904762	0.04761904762	0	0	inf	4.7619E+31
CASC17	0	0	0	0		0.00001
CASC18	0.04761904762	0.04761904762	0.05555555556	0.05555555556	0.7	0.8571428571
CASC19	0.09523809524	0.09523809524	0.1111111111	0.1111111111	0.6842105263	0.8571428571
CASC2	0.2380952381	0.2380952381	0	0	inf	2.38095E+32
CASC20	0.1904761905	0.1904761905	0.1111111111	0.1111111111	1.529411765	1.714285714
CASC21	0.09523809524	0.09523809524	0.1111111111	0.1111111111	0.6842105263	0.8571428571
CASC22	0.04761904762	0.04761904762	0	0	inf	4.7619E+31
CASC3	0.09523809524	0.09523809524	0	0	inf	9.52381E+31
CASC6	0	0	0	0		0.00001
CASC8	0.09523809524	0.09523809524	0.1111111111	0.1111111111	0.6842105263	0.8571428571
CASC9	0.1904761905	0.1904761905	0.1111111111	0.1111111111	1.529411765	1.714285714
CASD1	0.04761904762	0.04761904762	0.1111111111	0.1111111111	0.325	0.4285714286
ASKIN	0.09523809524	0.09523809524	0.2222222222	0.2222222222	0.2894736842	0.4285714286
CASP10	0	0	0.05555555556	0.05555555556	0	0
CASP14	0.1428571429	0.1428571429	0.1111111111	0.1111111111	1.083333333	1.285714286
ASP16I	0.04761904762	0.04761904762	0.1111111111	0.1111111111	0.325	0.4285714286
CASP2	0.04761904762	0.04761904762	0.05555555556	0.05555555556	0.7	0.8571428571
CASP3	0	0	0.05555555556	0.05555555556	0	0
CASP6	0	0	0.05555555556	0.05555555556	0	0
CASP7	0.1428571429	0.1428571429	0.05555555556	0.05555555556	2.333333333	2.571428571
CASP8	0	0	0.05555555556	0.05555555556	0	0
ASP8AI	0	0	0	0		0.00001
CASP9	0.04761904762	0.04761904762	0.1111111111	0.1111111111	0.325	0.4285714286
CASQ1	0.1428571429	0.1428571429	0	0	inf	1.42857E+32
CASQ2	0.04761904762	0.04761904762	0.05555555556	0.05555555556	0.7	0.8571428571

**CASR**	0.09523809524	0.09523809524	0.05555555556	0.05555555556	1.473684211	1.714285714
**CASS4**	0.09523809524	0.09523809524	0.1666666667	0.1666666667	0.4210526316	0.5714285714
**CAST**	0.04761904762	0.04761904762	0	0	inf	4.7619E+31
**CASTOR**	0.04761904762	0.04761904762	0.1111111111	0.1111111111	0.325	0.4285714286
**CASTOR**	0.1428571429	0.1428571429	0.1666666667	0.1666666667	0.6666666667	0.8571428571
**CASTOR**	0.04761904762	0.04761904762	0.05555555556	0.05555555556	0.7	0.8571428571
**CASZ1**	0.04761904762	0.04761904762	0.1111111111	0.1111111111	0.325	0.4285714286
**CATIP**	0	0	0.05555555556	0.05555555556	0	0
**ATIP-AS**	0	0	0.05555555556	0.05555555556	0	0
**ATIP-AS**	0	0	0.05555555556	0.05555555556	0	0
**ATSPER**	0.04761904762	0.04761904762	0.1111111111	0.1111111111	0.325	0.4285714286
**ATSPER**	0.1428571429	0.1428571429	0.05555555556	0.05555555556	2.333333333	2.571428571
**ATSPER**	0.1428571429	0.1428571429	0.05555555556	0.05555555556	2.333333333	2.571428571
**ATSPER**	0.04761904762	0.04761904762	0.05555555556	0.05555555556	0.7	0.8571428571
**CAV1**	0.04761904762	0.04761904762	0.1111111111	0.1111111111	0.325	0.4285714286
**CAV2**	0.04761904762	0.04761904762	0.1111111111	0.1111111111	0.325	0.4285714286
**CAV3**	0.04761904762	0.04761904762	0	0	inf	4.7619E+31
**CAVIN1**	0.04761904762	0.04761904762	0	0	inf	4.7619E+31
**CAVIN2**	0	0	0.05555555556	0.05555555556	0	0
**CAVIN3**	0.04761904762	0.04761904762	0	0	inf	4.7619E+31
**CAVIN4**	0.04761904762	0.04761904762	0	0	inf	4.7619E+31
**CBARP**	0.09523809524	0.09523809524	0.1111111111	0.1111111111	0.6842105263	0.8571428571
**CBFA2T**	0.09523809524	0.09523809524	0.1111111111	0.1111111111	0.6842105263	0.8571428571
**CBFA2T**	0.1904761905	0.1904761905	0	0	inf	1.90476E+32
**CBFB**	0.1428571429	0.1428571429	0.05555555556	0.05555555556	2.333333333	2.571428571
**CBLB**	0.09523809524	0.09523809524	0.05555555556	0.05555555556	1.473684211	1.714285714
**CBLC**	0.09523809524	0.09523809524	0.1111111111	0.1111111111	0.6842105263	0.8571428571
**CBLL1**	0.04761904762	0.04761904762	0.05555555556	0.05555555556	0.7	0.8571428571
**CBLN1**	0.04761904762	0.04761904762	0	0	inf	4.7619E+31
**CBLN4**	0.09523809524	0.09523809524	0.1666666667	0.1666666667	0.4210526316	0.5714285714
**CBR1**	0.04761904762	0.04761904762	0.1666666667	0.1666666667	0.2	0.2857142857
**CBR3**	0.04761904762	0.04761904762	0.1666666667	0.1666666667	0.2	0.2857142857
**BR3-AS**	0.04761904762	0.04761904762	0.1666666667	0.1666666667	0.2	0.2857142857
**CBR4**	0	0	0.05555555556	0.05555555556	0	0
**CBS**	0.04761904762	0.04761904762	0.1666666667	0.1666666667	0.2	0.2857142857
**CBSL**	0.04761904762	0.04761904762	0.1666666667	0.1666666667	0.2	0.2857142857
**CBWD1**	0.04761904762	0.04761904762	0.05555555556	0.05555555556	0.7	0.8571428571
**CBWD2**	0	0	0.05555555556	0.05555555556	0	0
**CBWD3**	0.09523809524	0.09523809524	0.2222222222	0.2222222222	0.2894736842	0.4285714286
**CBWD5**	0.09523809524	0.09523809524	0.2222222222	0.2222222222	0.2894736842	0.4285714286
**CBWD6**	0.09523809524	0.09523809524	0.2222222222	0.2222222222	0.2894736842	0.4285714286
**CBX1**	0.1428571429	0.1428571429	0	0	inf	1.42857E+32
**CBX2**	0.04761904762	0.04761904762	0	0	inf	4.7619E+31
**CBX3**	0.04761904762	0.04761904762	0.2222222222	0.2222222222	0.1375	0.2142857143
**CBX3P2**	0.09523809524	0.09523809524	0	0	inf	9.52381E+31
**CBX4**	0.04761904762	0.04761904762	0	0	inf	4.7619E+31
**CBX5**	0.04761904762	0.04761904762	0.05555555556	0.05555555556	0.7	0.8571428571
**CBX6**	0.04761904762	0.04761904762	0.1111111111	0.1111111111	0.325	0.4285714286
**CBX7**	0.04761904762	0.04761904762	0.1111111111	0.1111111111	0.325	0.4285714286
**CBX8**	0.04761904762	0.04761904762	0	0	inf	4.7619E+31
**CBY1**	0.04761904762	0.04761904762	0.1111111111	0.1111111111	0.325	0.4285714286

CBY3	0.09523809524	0.09523809524	0.05555555556	0.05555555556	1.473684211	1.714285714
CC2D1A	0.1428571429	0.1428571429	0.1111111111	0.1111111111	1.083333333	1.285714286
CC2D1B	0.04761904762	0.04761904762	0.05555555556	0.05555555556	0.7	0.8571428571
CC2D2A	0	0	0	0		0.00001
CC2D2B	0.1428571429	0.1428571429	0	0	inf	1.42857E+32
CCAR1	0.1904761905	0.1904761905	0.05555555556	0.05555555556	3.294117647	3.428571429
CCAR2	0.09523809524	0.09523809524	0.1111111111	0.1111111111	0.6842105263	0.8571428571
CCAT1	0.09523809524	0.09523809524	0.1111111111	0.1111111111	0.6842105263	0.8571428571
CCAT2	0.09523809524	0.09523809524	0.1111111111	0.1111111111	0.6842105263	0.8571428571
CCBE1	0.09523809524	0.09523809524	0	0	inf	9.52381E+31
CDC102	0.04761904762	0.04761904762	0	0	inf	4.7619E+31
CDC10[illegible]	0.1428571429	0.1428571429	0	0	inf	1.42857E+32
CDC10[illegible]	0.1428571429	0.1428571429	0.1111111111	0.1111111111	1.083333333	1.285714286
CDC10[illegible]	0.09523809524	0.09523809524	0.05555555556	0.05555555556	1.473684211	1.714285714
CDC10[illegible]	0.09523809524	0.09523809524	0.05555555556	0.05555555556	1.473684211	1.714285714
CDC11[illegible]	0	0	0.05555555556	0.05555555556	0	0
CDC11[illegible]	0.04761904762	0.04761904762	0	0	inf	4.7619E+31
CDC11[illegible]	0.04761904762	0.04761904762	0	0	inf	4.7619E+31
CDC11[illegible]	0.09523809524	0.09523809524	0.1111111111	0.1111111111	0.6842105263	0.8571428571
CDC11[illegible]	0	0	0.05555555556	0.05555555556	0	0
CDC11[illegible]	0.04761904762	0.04761904762	0.05555555556	0.05555555556	0.7	0.8571428571
CDC11[illegible]	0.04761904762	0.04761904762	0.1111111111	0.1111111111	0.325	0.4285714286
CDC12[illegible]	0.1428571429	0.1428571429	0.1111111111	0.1111111111	1.083333333	1.285714286
CDC12[illegible]	0.04761904762	0.04761904762	0.05555555556	0.05555555556	0.7	0.8571428571
CDC12[illegible]	0.04761904762	0.04761904762	0.1666666667	0.1666666667	0.2	0.2857142857
CDC12[illegible]	0	0	0.05555555556	0.05555555556	0	0
CCDC13	0.04761904762	0.04761904762	0	0	inf	4.7619E+31
DC13-A	0.04761904762	0.04761904762	0	0	inf	4.7619E+31
CDC13[illegible]	0.1428571429	0.1428571429	0.1111111111	0.1111111111	1.083333333	1.285714286
CDC13[illegible]	0	0	0.1111111111	0.1111111111	0	0
CDC13[illegible]	0.04761904762	0.04761904762	0.05555555556	0.05555555556	0.7	0.8571428571
CDC13[illegible]	0.04761904762	0.04761904762	0	0	inf	4.7619E+31
CDC13[illegible]	0	0	0.05555555556	0.05555555556	0	0
CCDC14	0.04761904762	0.04761904762	0.05555555556	0.05555555556	0.7	0.8571428571
CDC14[illegible]	0	0	0.05555555556	0.05555555556	0	0
CDC14	0	0	0.1111111111	0.1111111111	0	0
CDC14[illegible]	0	0	0.05555555556	0.05555555556	0	0
CDC144	0.04761904762	0.04761904762	0.05555555556	0.05555555556	0.7	0.8571428571
CDC144	0.04761904762	0.04761904762	0.1111111111	0.1111111111	0.325	0.4285714286
CDC144C	0.09523809524	0.09523809524	0.05555555556	0.05555555556	1.473684211	1.714285714
CDC144N	0.09523809524	0.09523809524	0.05555555556	0.05555555556	1.473684211	1.714285714
C144NL	0.09523809524	0.09523809524	0.05555555556	0.05555555556	1.473684211	1.714285714
CDC14[illegible]	0.04761904762	0.04761904762	0.1111111111	0.1111111111	0.325	0.4285714286
CDC14[illegible]	0	0	0.05555555556	0.05555555556	0	0
DC148-[illegible]	0	0	0.05555555556	0.05555555556	0	0
CDC15[illegible]	0	0	0.05555555556	0.05555555556	0	0
CDC15	0.1428571429	0.1428571429	0.1111111111	0.1111111111	1.083333333	1.285714286
CDC15[illegible]	0.04761904762	0.04761904762	0.05555555556	0.05555555556	0.7	0.8571428571
CDC15[illegible]	0.09523809524	0.09523809524	0.2222222222	0.2222222222	0.2894736842	0.4285714286
CDC15[illegible]	0.09523809524	0.09523809524	0.1111111111	0.1111111111	0.6842105263	0.8571428571
CDC15[illegible]	0.04761904762	0.04761904762	0.1111111111	0.1111111111	0.325	0.4285714286

CCDC15	0.04761904762	0.04761904762	0.05555555556	0.05555555556	0.7	0.8571428571
CDC162	0	0	0	0		0.00001
CCDC16	0.04761904762	0.04761904762	0.05555555556	0.05555555556	0.7	0.8571428571
CCDC16	0.04761904762	0.04761904762	0.1666666667	0.1666666667	0.2	0.2857142857
CCDC16	0.04761904762	0.04761904762	0.05555555556	0.05555555556	0.7	0.8571428571
CCDC17	0.04761904762	0.04761904762	0.05555555556	0.05555555556	0.7	0.8571428571
CCDC17	0.04761904762	0.04761904762	0.05555555556	0.05555555556	0.7	0.8571428571
CCDC17	0.2380952381	0.2380952381	0	0	inf	2.38095E+32
CCDC17	0	0	0.05555555556	0.05555555556	0	0
CCDC17	0.04761904762	0.04761904762	0	0	inf	4.7619E+31
CCDC17	0.09523809524	0.09523809524	0	0	inf	9.52381E+31
CCDC18	0.04761904762	0.04761904762	0.05555555556	0.05555555556	0.7	0.8571428571
DC18-A	0.04761904762	0.04761904762	0.05555555556	0.05555555556	0.7	0.8571428571
CCDC18	0.04761904762	0.04761904762	0	0	inf	4.7619E+31
CCDC18	0.1428571429	0.1428571429	0	0	inf	1.42857E+32
CCDC18	0.09523809524	0.09523809524	0	0	inf	9.52381E+31
CCDC18	0.1428571429	0.1428571429	0.05555555556	0.05555555556	2.333333333	2.571428571
DC183-A	0.1428571429	0.1428571429	0.05555555556	0.05555555556	2.333333333	2.571428571
CCDC18	0.04761904762	0.04761904762	0.05555555556	0.05555555556	0.7	0.8571428571
CCDC18	0.1428571429	0.1428571429	0.05555555556	0.05555555556	2.333333333	2.571428571
CCDC18	0.1428571429	0.1428571429	0.05555555556	0.05555555556	2.333333333	2.571428571
CCDC18	0.04761904762	0.04761904762	0.05555555556	0.05555555556	0.7	0.8571428571
CCDC18	0.04761904762	0.04761904762	0	0	inf	4.7619E+31
CCDC19	0.1428571429	0.1428571429	0	0	inf	1.42857E+32
CCDC19	0.09523809524	0.09523809524	0.05555555556	0.05555555556	1.473684211	1.714285714
CCDC19	0.04761904762	0.04761904762	0	0	inf	4.7619E+31
CCDC19	0.04761904762	0.04761904762	0	0	inf	4.7619E+31
CCDC20	0.04761904762	0.04761904762	0	0	inf	4.7619E+31
CCDC24	0.04761904762	0.04761904762	0.05555555556	0.05555555556	0.7	0.8571428571
CCDC25	0.04761904762	0.04761904762	0.1111111111	0.1111111111	0.325	0.4285714286
CCDC26	0.04761904762	0.04761904762	0.1111111111	0.1111111111	0.325	0.4285714286
CCDC27	0	0	0.05555555556	0.05555555556	0	0
CCDC28	0.04761904762	0.04761904762	0.1111111111	0.1111111111	0.325	0.4285714286
CCDC3	0.09523809524	0.09523809524	0.1111111111	0.1111111111	0.6842105263	0.8571428571
CCDC30	0.04761904762	0.04761904762	0.05555555556	0.05555555556	0.7	0.8571428571
CCDC33	0.04761904762	0.04761904762	0.05555555556	0.05555555556	0.7	0.8571428571
CCDC36	0.09523809524	0.09523809524	0	0	inf	9.52381E+31
CDC37-I	0.04761904762	0.04761904762	0.05555555556	0.05555555556	0.7	0.8571428571
CCDC38	0.04761904762	0.04761904762	0.05555555556	0.05555555556	0.7	0.8571428571
CCDC39	0.1904761905	0.1904761905	0.05555555556	0.05555555556	3.294117647	3.428571429
CCDC40	0.04761904762	0.04761904762	0	0	inf	4.7619E+31
CCDC42	0.04761904762	0.04761904762	0.05555555556	0.05555555556	0.7	0.8571428571
CCDC43	0.09523809524	0.09523809524	0	0	inf	9.52381E+31
CCDC47	0.04761904762	0.04761904762	0	0	inf	4.7619E+31
CCDC50	0.09523809524	0.09523809524	0.05555555556	0.05555555556	1.473684211	1.714285714
CCDC51	0.09523809524	0.09523809524	0	0	inf	9.52381E+31
CCDC54	0.09523809524	0.09523809524	0.05555555556	0.05555555556	1.473684211	1.714285714
CCDC57	0.04761904762	0.04761904762	0	0	inf	4.7619E+31
CCDC58	0.09523809524	0.09523809524	0.05555555556	0.05555555556	1.473684211	1.714285714
CCDC59	0.04761904762	0.04761904762	0.05555555556	0.05555555556	0.7	0.8571428571
CCDC6	0.04761904762	0.04761904762	0.05555555556	0.05555555556	0.7	0.8571428571

CCDC60	0	0	0.05555555556	0.05555555556	0	0
CCDC62	0.04761904762	0.04761904762	0.1111111111	0.1111111111	0.325	0.4285714286
CCDC63	0	0	0.1111111111	0.1111111111	0	0
CCDC65	0.04761904762	0.04761904762	0.05555555556	0.05555555556	0.7	0.8571428571
CCDC66	0.04761904762	0.04761904762	0	0	inf	4.7619E+31
CCDC68	0.09523809524	0.09523809524	0.05555555556	0.05555555556	1.473684211	1.714285714
CCDC7	0.09523809524	0.09523809524	0.2222222222	0.2222222222	0.2894736842	0.4285714286
CCDC71	0.09523809524	0.09523809524	0	0	inf	9.52381E+31
CDC71I	0.04761904762	0.04761904762	0.05555555556	0.05555555556	0.7	0.8571428571
CCDC73	0.04761904762	0.04761904762	0	0	inf	4.7619E+31
CDC74A	0	0	0.05555555556	0.05555555556	0	0
CDC74I	0	0	0.05555555556	0.05555555556	0	0
CCDC77	0.04761904762	0.04761904762	0.1111111111	0.1111111111	0.325	0.4285714286
CCDC78	0.04761904762	0.04761904762	0.2222222222	0.2222222222	0.1375	0.2142857143
CCDC8	0.09523809524	0.09523809524	0.1111111111	0.1111111111	0.6842105263	0.8571428571
CCDC80	0.09523809524	0.09523809524	0.05555555556	0.05555555556	1.473684211	1.714285714
CDC85A	0.04761904762	0.04761904762	0.05555555556	0.05555555556	0.7	0.8571428571
CDC88A	0.04761904762	0.04761904762	0.05555555556	0.05555555556	0.7	0.8571428571
CCDC9	0.09523809524	0.09523809524	0.1111111111	0.1111111111	0.6842105263	0.8571428571
CCDC91	0.04761904762	0.04761904762	0.05555555556	0.05555555556	0.7	0.8571428571
CCDC92	0.04761904762	0.04761904762	0.05555555556	0.05555555556	0.7	0.8571428571
CDC92I	0	0	0.1111111111	0.1111111111	0	0
CCDC93	0	0	0.05555555556	0.05555555556	0	0
CCDC97	0.04761904762	0.04761904762	0.05555555556	0.05555555556	0.7	0.8571428571
CCEPR	0.04761904762	0.04761904762	0.05555555556	0.05555555556	0.7	0.8571428571
CCER1	0.04761904762	0.04761904762	0.05555555556	0.05555555556	0.7	0.8571428571
CCER2	0.04761904762	0.04761904762	0.05555555556	0.05555555556	0.7	0.8571428571
CCIN	0.09523809524	0.09523809524	0.05555555556	0.05555555556	1.473684211	1.714285714
CCK	0.04761904762	0.04761904762	0	0	inf	4.7619E+31
CCKBR	0.04761904762	0.04761904762	0	0	inf	4.7619E+31
CCL1	0.04761904762	0.04761904762	0	0	inf	4.7619E+31
CCL11	0.04761904762	0.04761904762	0	0	inf	4.7619E+31
CCL13	0.04761904762	0.04761904762	0	0	inf	4.7619E+31
CCL14	0.09523809524	0.09523809524	0	0	inf	9.52381E+31
CCL15	0.09523809524	0.09523809524	0	0	inf	9.52381E+31
L15-CCI	0.09523809524	0.09523809524	0	0	inf	9.52381E+31
CCL16	0.09523809524	0.09523809524	0	0	inf	9.52381E+31
CCL17	0.04761904762	0.04761904762	0	0	inf	4.7619E+31
CCL18	0.09523809524	0.09523809524	0	0	inf	9.52381E+31
CCL19	0.09523809524	0.09523809524	0.05555555556	0.05555555556	1.473684211	1.714285714
CCL2	0.04761904762	0.04761904762	0	0	inf	4.7619E+31
CCL20	0	0	0.05555555556	0.05555555556	0	0
CCL21	0.09523809524	0.09523809524	0.05555555556	0.05555555556	1.473684211	1.714285714
CCL22	0.04761904762	0.04761904762	0	0	inf	4.7619E+31
CCL23	0.09523809524	0.09523809524	0	0	inf	9.52381E+31
CCL24	0.1428571429	0.1428571429	0.1111111111	0.1111111111	1.083333333	1.285714286
CCL25	0.1428571429	0.1428571429	0.05555555556	0.05555555556	2.333333333	2.571428571
CCL26	0.1428571429	0.1428571429	0.1111111111	0.1111111111	1.083333333	1.285714286
CCL27	0.09523809524	0.09523809524	0.05555555556	0.05555555556	1.473684211	1.714285714
CCL28	0.04761904762	0.04761904762	0.05555555556	0.05555555556	0.7	0.8571428571
CCL3	0.09523809524	0.09523809524	0	0	inf	9.52381E+31

**CCL3L1**	0.09523809524	0.09523809524	0.05555555556	0.05555555556	1.473684211	1.714285714
**CCL3L3**	0.09523809524	0.09523809524	0.05555555556	0.05555555556	1.473684211	1.714285714
**CCL4**	0.09523809524	0.09523809524	0	0	inf	9.52381E+31
**CCL4L1**	0.09523809524	0.09523809524	0.05555555556	0.05555555556	1.473684211	1.714285714
**CCL4L2**	0.09523809524	0.09523809524	0.05555555556	0.05555555556	1.473684211	1.714285714
**CCL5**	0.09523809524	0.09523809524	0	0	inf	9.52381E+31
**CCL7**	0.04761904762	0.04761904762	0	0	inf	4.7619E+31
**CCL8**	0.04761904762	0.04761904762	0	0	inf	4.7619E+31
**CCM2**	0.04761904762	0.04761904762	0.2222222222	0.2222222222	0.1375	0.2142857143
**CCM2L**	0.09523809524	0.09523809524	0.1111111111	0.1111111111	0.6842105263	0.8571428571
**CCN1**	0.04761904762	0.04761904762	0.05555555556	0.05555555556	0.7	0.8571428571
**CCN3**	0.09523809524	0.09523809524	0.1666666667	0.1666666667	0.4210526316	0.5714285714
**CCN4**	0.04761904762	0.04761904762	0.2222222222	0.2222222222	0.1375	0.2142857143
**CCN5**	0.1428571429	0.1428571429	0.1666666667	0.1666666667	0.6666666667	0.8571428571
**CCNA2**	0	0	0.05555555556	0.05555555556	0	0
**CCNB1**	0.04761904762	0.04761904762	0.05555555556	0.05555555556	0.7	0.8571428571
**CNB1IP**	0.04761904762	0.04761904762	0	0	inf	4.7619E+31
**CCNB2**	0.04761904762	0.04761904762	0.05555555556	0.05555555556	0.7	0.8571428571
**CCND2**	0.04761904762	0.04761904762	0.1111111111	0.1111111111	0.325	0.4285714286
**CND2-A**	0.04761904762	0.04761904762	0.1111111111	0.1111111111	0.325	0.4285714286
**CCND3**	0.04761904762	0.04761904762	0.05555555556	0.05555555556	0.7	0.8571428571
**CCNE1**	0.04761904762	0.04761904762	0.1666666667	0.1666666667	0.2	0.2857142857
**CCNE2**	0.1904761905	0.1904761905	0.1111111111	0.1111111111	1.529411765	1.714285714
**CCNF**	0.09523809524	0.09523809524	0.2222222222	0.2222222222	0.2894736842	0.4285714286
**CCNG1**	0	0	0.05555555556	0.05555555556	0	0
**CCNG2**	0.04761904762	0.04761904762	0.05555555556	0.05555555556	0.7	0.8571428571
**CCNH**	0.04761904762	0.04761904762	0	0	inf	4.7619E+31
**CCNI**	0.04761904762	0.04761904762	0.05555555556	0.05555555556	0.7	0.8571428571
**CCNI2**	0.04761904762	0.04761904762	0	0	inf	4.7619E+31
**CCNJ**	0.1428571429	0.1428571429	0	0	inf	1.42857E+32
**CCNJL**	0.04761904762	0.04761904762	0.05555555556	0.05555555556	0.7	0.8571428571
**CCNL1**	0.1428571429	0.1428571429	0.05555555556	0.05555555556	2.333333333	2.571428571
**CCNL2**	0	0	0.1111111111	0.1111111111	0	0
**CCNO**	0.04761904762	0.04761904762	0.05555555556	0.05555555556	0.7	0.8571428571
**CCNT1**	0.04761904762	0.04761904762	0.05555555556	0.05555555556	0.7	0.8571428571
**CCNT2**	0.04761904762	0.04761904762	0.05555555556	0.05555555556	0.7	0.8571428571
**CNT2-A**	0.04761904762	0.04761904762	0.05555555556	0.05555555556	0.7	0.8571428571
**CCNY**	0.09523809524	0.09523809524	0.1666666667	0.1666666667	0.4210526316	0.5714285714
**CCNYL1**	0	0	0.05555555556	0.05555555556	0	0
**CCNYL2**	0.1428571429	0.1428571429	0.05555555556	0.05555555556	2.333333333	2.571428571
**CCNYL3**	0.04761904762	0.04761904762	0	0	inf	4.7619E+31
**CCP110**	0.04761904762	0.04761904762	0	0	inf	4.7619E+31
**CCPG1**	0.04761904762	0.04761904762	0.05555555556	0.05555555556	0.7	0.8571428571
**CCR10**	0.04761904762	0.04761904762	0	0	inf	4.7619E+31
**CCR7**	0.09523809524	0.09523809524	0	0	inf	9.52381E+31
**CCR8**	0.04761904762	0.04761904762	0	0	inf	4.7619E+31
**CCSAP**	0.1428571429	0.1428571429	0	0	inf	1.42857E+32
**CCSER1**	0	0	0.05555555556	0.05555555556	0	0
**CCSER2**	0.1428571429	0.1428571429	0	0	inf	1.42857E+32
**CCT2**	0.09523809524	0.09523809524	0.1111111111	0.1111111111	0.6842105263	0.8571428571
**CCT4**	0.04761904762	0.04761904762	0.05555555556	0.05555555556	0.7	0.8571428571

CCT5	0.04761904762	0.04761904762	0.05555555556	0.05555555556	0.7	0.8571428571
CCT6A	0.2380952381	0.2380952381	0.2222222222	0.2222222222	0.859375	1.071428571
CCT6B	0.04761904762	0.04761904762	0	0	inf	4.7619E+31
CCT6P1	0.04761904762	0.04761904762	0.1111111111	0.1111111111	0.325	0.4285714286
CCT6P3	0.04761904762	0.04761904762	0.1111111111	0.1111111111	0.325	0.4285714286
CCT7	0	0	0.05555555556	0.05555555556	0	0
CCT8	0.04761904762	0.04761904762	0.1111111111	0.1111111111	0.325	0.4285714286
CCT8L2	0.04761904762	0.04761904762	0	0	inf	4.7619E+31
CCZ1	0.04761904762	0.04761904762	0.1666666667	0.1666666667	0.2	0.2857142857
CCZ1B	0.04761904762	0.04761904762	0.1666666667	0.1666666667	0.2	0.2857142857
1P-OR7	0.04761904762	0.04761904762	0.05555555556	0.05555555556	0.7	0.8571428571
CD101	0.04761904762	0.04761904762	0.05555555556	0.05555555556	0.7	0.8571428571
CD109	0.04761904762	0.04761904762	0	0	inf	4.7619E+31
CD14	0.04761904762	0.04761904762	0	0	inf	4.7619E+31
CD151	0.04761904762	0.04761904762	0	0	inf	4.7619E+31
CD160	0.1428571429	0.1428571429	0.05555555556	0.05555555556	2.333333333	2.571428571
CD163	0.04761904762	0.04761904762	0.1111111111	0.1111111111	0.325	0.4285714286
CD163L1	0.04761904762	0.04761904762	0.1111111111	0.1111111111	0.325	0.4285714286
CD164	0	0	0	0		0.00001
CD164L2	0.04761904762	0.04761904762	0.1111111111	0.1111111111	0.325	0.4285714286
CD177	0.04761904762	0.04761904762	0.05555555556	0.05555555556	0.7	0.8571428571
CD180	0.04761904762	0.04761904762	0	0	inf	4.7619E+31
CD19	0.04761904762	0.04761904762	0	0	inf	4.7619E+31
CD1A	0.1428571429	0.1428571429	0.05555555556	0.05555555556	2.333333333	2.571428571
CD1B	0.1428571429	0.1428571429	0.05555555556	0.05555555556	2.333333333	2.571428571
CD1C	0.1428571429	0.1428571429	0.05555555556	0.05555555556	2.333333333	2.571428571
CD1D	0.1428571429	0.1428571429	0.05555555556	0.05555555556	2.333333333	2.571428571
CD1E	0.1428571429	0.1428571429	0.05555555556	0.05555555556	2.333333333	2.571428571
CD2	0.04761904762	0.04761904762	0.05555555556	0.05555555556	0.7	0.8571428571
CD200	0.09523809524	0.09523809524	0.05555555556	0.05555555556	1.473684211	1.714285714
CD200R1	0.09523809524	0.09523809524	0.05555555556	0.05555555556	1.473684211	1.714285714
D200R1	0.09523809524	0.09523809524	0.05555555556	0.05555555556	1.473684211	1.714285714
200R1L-	0.09523809524	0.09523809524	0.05555555556	0.05555555556	1.473684211	1.714285714
CD209	0.1428571429	0.1428571429	0.05555555556	0.05555555556	2.333333333	2.571428571
CD22	0.04761904762	0.04761904762	0.05555555556	0.05555555556	0.7	0.8571428571
CD244	0.1428571429	0.1428571429	0	0	inf	1.42857E+32
CD247	0.1428571429	0.1428571429	0	0	inf	1.42857E+32
CD27	0.04761904762	0.04761904762	0.1111111111	0.1111111111	0.325	0.4285714286
D27-AS	0.04761904762	0.04761904762	0.1111111111	0.1111111111	0.325	0.4285714286
CD274	0.04761904762	0.04761904762	0.05555555556	0.05555555556	0.7	0.8571428571
CD276	0.04761904762	0.04761904762	0.05555555556	0.05555555556	0.7	0.8571428571
CD28	0	0	0.05555555556	0.05555555556	0	0
CD2AP	0.04761904762	0.04761904762	0.05555555556	0.05555555556	0.7	0.8571428571
CD2BP2	0.04761904762	0.04761904762	0	0	inf	4.7619E+31
CD300A	0.04761904762	0.04761904762	0	0	inf	4.7619E+31
CD300C	0.04761904762	0.04761904762	0	0	inf	4.7619E+31
CD300E	0.04761904762	0.04761904762	0	0	inf	4.7619E+31
CD300H	0.04761904762	0.04761904762	0	0	inf	4.7619E+31
CD300LI	0.04761904762	0.04761904762	0	0	inf	4.7619E+31
CD300LI	0.04761904762	0.04761904762	0	0	inf	4.7619E+31
CD300LI	0.04761904762	0.04761904762	0	0	inf	4.7619E+31

CD300LC	0.09523809524	0.09523809524	0	0	inf	9.52381E+31
CD302	0	0	0.05555555556	0.05555555556	0	0
CD320	0.1428571429	0.1428571429	0.05555555556	0.05555555556	2.333333333	2.571428571
CD34	0.1428571429	0.1428571429	0	0	inf	1.42857E+32
CD36	0.04761904762	0.04761904762	0.1111111111	0.1111111111	0.325	0.4285714286
CD37	0.09523809524	0.09523809524	0.1111111111	0.1111111111	0.6842105263	0.8571428571
CD38	0	0	0	0		0.00001
CD3EAP	0.09523809524	0.09523809524	0.1111111111	0.1111111111	0.6842105263	0.8571428571
CD4	0.04761904762	0.04761904762	0.1111111111	0.1111111111	0.325	0.4285714286
CD40	0.1428571429	0.1428571429	0.1666666667	0.1666666667	0.6666666667	0.8571428571
CD46	0.1428571429	0.1428571429	0	0	inf	1.42857E+32
CD47	0.09523809524	0.09523809524	0.05555555556	0.05555555556	1.473684211	1.714285714
CD48	0.1428571429	0.1428571429	0	0	inf	1.42857E+32
CD52	0.04761904762	0.04761904762	0.1111111111	0.1111111111	0.325	0.4285714286
CD53	0.04761904762	0.04761904762	0	0	inf	4.7619E+31
CD55	0.1428571429	0.1428571429	0	0	inf	1.42857E+32
CD58	0.04761904762	0.04761904762	0.05555555556	0.05555555556	0.7	0.8571428571
CD5L	0.1428571429	0.1428571429	0.05555555556	0.05555555556	2.333333333	2.571428571
CD63	0.04761904762	0.04761904762	0.05555555556	0.05555555556	0.7	0.8571428571
CD68	0	0	0.05555555556	0.05555555556	0	0
CD69	0.04761904762	0.04761904762	0.1111111111	0.1111111111	0.325	0.4285714286
CD7	0.04761904762	0.04761904762	0	0	inf	4.7619E+31
CD70	0.1428571429	0.1428571429	0.05555555556	0.05555555556	2.333333333	2.571428571
CD72	0.09523809524	0.09523809524	0.05555555556	0.05555555556	1.473684211	1.714285714
CD79A	0.04761904762	0.04761904762	0.05555555556	0.05555555556	0.7	0.8571428571
CD79B	0.04761904762	0.04761904762	0	0	inf	4.7619E+31
CD80	0.09523809524	0.09523809524	0.05555555556	0.05555555556	1.473684211	1.714285714
CD81	0.04761904762	0.04761904762	0	0	inf	4.7619E+31
CD81-AS	0.04761904762	0.04761904762	0	0	inf	4.7619E+31
CD84	0.1428571429	0.1428571429	0	0	inf	1.42857E+32
CD86	0.09523809524	0.09523809524	0.05555555556	0.05555555556	1.473684211	1.714285714
CD8A	0	0	0.05555555556	0.05555555556	0	0
CD8B	0	0	0.05555555556	0.05555555556	0	0
CD8B2	0	0	0.05555555556	0.05555555556	0	0
CD9	0.04761904762	0.04761904762	0.1111111111	0.1111111111	0.325	0.4285714286
CD93	0.1904761905	0.1904761905	0.05555555556	0.05555555556	3.294117647	3.428571429
CD96	0.09523809524	0.09523809524	0.05555555556	0.05555555556	1.473684211	1.714285714
CDA	0.04761904762	0.04761904762	0.1111111111	0.1111111111	0.325	0.4285714286
CDC123	0.09523809524	0.09523809524	0.1111111111	0.1111111111	0.6842105263	0.8571428571
CDC14A	0.04761904762	0.04761904762	0.05555555556	0.05555555556	0.7	0.8571428571
CDC14B	0.04761904762	0.04761904762	0.05555555556	0.05555555556	0.7	0.8571428571
CDC14C	0.09523809524	0.09523809524	0.2222222222	0.2222222222	0.2894736842	0.4285714286
CDC20	0.04761904762	0.04761904762	0.05555555556	0.05555555556	0.7	0.8571428571
CDC20B	0.04761904762	0.04761904762	0.05555555556	0.05555555556	0.7	0.8571428571
CDC23	0.04761904762	0.04761904762	0	0	inf	4.7619E+31
CDC25A	0.09523809524	0.09523809524	0	0	inf	9.52381E+31
CDC25B	0.1904761905	0.1904761905	0.05555555556	0.05555555556	3.294117647	3.428571429
CDC25C	0.04761904762	0.04761904762	0	0	inf	4.7619E+31
CDC26	0.04761904762	0.04761904762	0.05555555556	0.05555555556	0.7	0.8571428571
CDC27	0.1428571429	0.1428571429	0	0	inf	1.42857E+32
CDC34	0.09523809524	0.09523809524	0.1111111111	0.1111111111	0.6842105263	0.8571428571

CDC37	0.1428571429	0.1428571429	0.1111111111	0.1111111111	1.083333333	1.285714286
CDC37L	0.04761904762	0.04761904762	0.05555555556	0.05555555556	0.7	0.8571428571
DC37L1-	0.04761904762	0.04761904762	0.05555555556	0.05555555556	0.7	0.8571428571
CDC40	0	0	0	0		0.00001
CDC42	0.04761904762	0.04761904762	0.1111111111	0.1111111111	0.325	0.4285714286
DC42BP	0.1428571429	0.1428571429	0	0	inf	1.42857E+32
DC42EP	0	0	0.1111111111	0.1111111111	0	0
DC42EP	0	0	0.05555555556	0.05555555556	0	0
DC42EP	0.04761904762	0.04761904762	0	0	inf	4.7619E+31
DC42EP	0.09523809524	0.09523809524	0.05555555556	0.05555555556	1.473684211	1.714285714
CDC42P3	0.04761904762	0.04761904762	0.1666666667	0.1666666667	0.2	0.2857142857
DC42SE	0.1904761905	0.1904761905	0.05555555556	0.05555555556	3.294117647	3.428571429
DC42SE	0.04761904762	0.04761904762	0	0	inf	4.7619E+31
CDC45	0.04761904762	0.04761904762	0.05555555556	0.05555555556	0.7	0.8571428571
CDC5L	0.04761904762	0.04761904762	0.05555555556	0.05555555556	0.7	0.8571428571
CDC6	0.09523809524	0.09523809524	0	0	inf	9.52381E+31
CDC7	0.04761904762	0.04761904762	0.05555555556	0.05555555556	0.7	0.8571428571
CDC73	0.1428571429	0.1428571429	0	0	inf	1.42857E+32
CDCA2	0.04761904762	0.04761904762	0.1111111111	0.1111111111	0.325	0.4285714286
CDCA3	0.04761904762	0.04761904762	0.1111111111	0.1111111111	0.325	0.4285714286
CDCA7	0	0	0.1111111111	0.1111111111	0	0
CDCA7L	0.04761904762	0.04761904762	0.1666666667	0.1666666667	0.2	0.2857142857
CDCA8	0.04761904762	0.04761904762	0.05555555556	0.05555555556	0.7	0.8571428571
CDCP2	0.04761904762	0.04761904762	0.05555555556	0.05555555556	0.7	0.8571428571
CDH1	0.1428571429	0.1428571429	0.05555555556	0.05555555556	2.333333333	2.571428571
CDH10	0.04761904762	0.04761904762	0.05555555556	0.05555555556	0.7	0.8571428571
CDH11	0.04761904762	0.04761904762	0.05555555556	0.05555555556	0.7	0.8571428571
CDH12	0.04761904762	0.04761904762	0.1111111111	0.1111111111	0.325	0.4285714286
CDH13	0.04761904762	0.04761904762	0	0	inf	4.7619E+31
CDH15	0.09523809524	0.09523809524	0	0	inf	9.52381E+31
CDH16	0.09523809524	0.09523809524	0.05555555556	0.05555555556	1.473684211	1.714285714
CDH17	0.1904761905	0.1904761905	0.1111111111	0.1111111111	1.529411765	1.714285714
CDH18	0.04761904762	0.04761904762	0.1111111111	0.1111111111	0.325	0.4285714286
DH18-AS	0.04761904762	0.04761904762	0.05555555556	0.05555555556	0.7	0.8571428571
CDH2	0.09523809524	0.09523809524	0	0	inf	9.52381E+31
CDH20	0.09523809524	0.09523809524	0	0	inf	9.52381E+31
CDH22	0.1428571429	0.1428571429	0.1666666667	0.1666666667	0.6666666667	0.8571428571
CDH23	0.1428571429	0.1428571429	0	0	inf	1.42857E+32
DH23-AS	0.09523809524	0.09523809524	0	0	inf	9.52381E+31
CDH24	0	0	0.05555555556	0.05555555556	0	0
CDH26	0.1428571429	0.1428571429	0.1666666667	0.1666666667	0.6666666667	0.8571428571
CDH3	0.1428571429	0.1428571429	0.05555555556	0.05555555556	2.333333333	2.571428571
CDH4	0.1428571429	0.1428571429	0.1111111111	0.1111111111	1.083333333	1.285714286
CDH5	0.04761904762	0.04761904762	0	0	inf	4.7619E+31
CDH6	0.04761904762	0.04761904762	0.05555555556	0.05555555556	0.7	0.8571428571
CDH8	0.04761904762	0.04761904762	0	0	inf	4.7619E+31
CDH9	0.04761904762	0.04761904762	0.05555555556	0.05555555556	0.7	0.8571428571
CDHR1	0.1428571429	0.1428571429	0	0	inf	1.42857E+32
CDHR2	0.09523809524	0.09523809524	0.05555555556	0.05555555556	1.473684211	1.714285714
CDHR3	0.04761904762	0.04761904762	0.05555555556	0.05555555556	0.7	0.8571428571
CDHR4	0.09523809524	0.09523809524	0	0	inf	9.52381E+31

**CDHR5**	0.04761904762	0.04761904762	0	0	inf	4.7619E+31
**CDIP1**	0.04761904762	0.04761904762	0	0	inf	4.7619E+31
**CDIPT**	0.04761904762	0.04761904762	0	0	inf	4.7619E+31
**DIPTOS**	0.04761904762	0.04761904762	0	0	inf	4.7619E+31
**CDK1**	0.04761904762	0.04761904762	0.1111111111	0.1111111111	0.325	0.4285714286
**CDK10**	0.09523809524	0.09523809524	0	0	inf	9.52381E+31
**CDK11A**	0	0	0.1111111111	0.1111111111	0	0
**CDK11B**	0	0	0.1111111111	0.1111111111	0	0
**CDK12**	0.09523809524	0.09523809524	0	0	inf	9.52381E+31
**CDK13**	0.04761904762	0.04761904762	0.2222222222	0.2222222222	0.1375	0.2142857143
**CDK14**	0.09523809524	0.09523809524	0.1111111111	0.1111111111	0.6842105263	0.8571428571
**CDK15**	0	0	0.05555555556	0.05555555556	0	0
**CDK17**	0.04761904762	0.04761904762	0.05555555556	0.05555555556	0.7	0.8571428571
**CDK18**	0.1428571429	0.1428571429	0	0	inf	1.42857E+32
**CDK19**	0	0	0	0		0.00001
**CDK2**	0.09523809524	0.09523809524	0.05555555556	0.05555555556	1.473684211	1.714285714
**CDK20**	0	0	0.05555555556	0.05555555556	0	0
**CDK2AP**	0.04761904762	0.04761904762	0.1111111111	0.1111111111	0.325	0.4285714286
**CDK3**	0.04761904762	0.04761904762	0	0	inf	4.7619E+31
**CDK4**	0.1428571429	0.1428571429	0.05555555556	0.05555555556	2.333333333	2.571428571
**CDK5**	0.09523809524	0.09523809524	0.05555555556	0.05555555556	1.473684211	1.714285714
**CDK5R1**	0	0	0	0		0.00001
**CDK5R2**	0	0	0.05555555556	0.05555555556	0	0
**DK5RAI**	0.09523809524	0.09523809524	0.1111111111	0.1111111111	0.6842105263	0.8571428571
**DK5RAI**	0.09523809524	0.09523809524	0.05555555556	0.05555555556	1.473684211	1.714285714
**DK5RAI**	0.1428571429	0.1428571429	0	0	inf	1.42857E+32
**CDK6**	0.04761904762	0.04761904762	0.1111111111	0.1111111111	0.325	0.4285714286
**CDK7**	0.04761904762	0.04761904762	0.05555555556	0.05555555556	0.7	0.8571428571
**CDK9**	0.09523809524	0.09523809524	0.05555555556	0.05555555556	1.473684211	1.714285714
**CDKL1**	0.04761904762	0.04761904762	0	0	inf	4.7619E+31
**CDKL2**	0.04761904762	0.04761904762	0.05555555556	0.05555555556	0.7	0.8571428571
**CDKL3**	0.04761904762	0.04761904762	0	0	inf	4.7619E+31
**CDKL4**	0	0	0.05555555556	0.05555555556	0	0
**CDKN1A**	0.04761904762	0.04761904762	0.05555555556	0.05555555556	0.7	0.8571428571
**CDKN1B**	0.04761904762	0.04761904762	0.05555555556	0.05555555556	0.7	0.8571428571
**CDKN1C**	0.04761904762	0.04761904762	0	0	inf	4.7619E+31
**DKN2AI**	0	0	0.05555555556	0.05555555556	0	0
**KN2AIP**	0.04761904762	0.04761904762	0	0	inf	4.7619E+31
**KN2B-A**	0.09523809524	0.09523809524	0.1111111111	0.1111111111	0.6842105263	0.8571428571
**CDKN2C**	0.04761904762	0.04761904762	0.05555555556	0.05555555556	0.7	0.8571428571
**CDKN2D**	0.1428571429	0.1428571429	0.1111111111	0.1111111111	1.083333333	1.285714286
**CDKN3**	0.04761904762	0.04761904762	0	0	inf	4.7619E+31
**CDNF**	0.09523809524	0.09523809524	0.1111111111	0.1111111111	0.6842105263	0.8571428571
**CDO1**	0.04761904762	0.04761904762	0	0	inf	4.7619E+31
**CDPF1**	0	0	0.1111111111	0.1111111111	0	0
**CDR2**	0.04761904762	0.04761904762	0	0	inf	4.7619E+31
**CDR2L**	0.04761904762	0.04761904762	0	0	inf	4.7619E+31
**CDRT1**	0.04761904762	0.04761904762	0.05555555556	0.05555555556	0.7	0.8571428571
**CDRT15**	0.04761904762	0.04761904762	0.05555555556	0.05555555556	0.7	0.8571428571
**DRT15L**	0.09523809524	0.09523809524	0.05555555556	0.05555555556	1.473684211	1.714285714
**DRT15P**	0.04761904762	0.04761904762	0.05555555556	0.05555555556	0.7	0.8571428571

DRT15P	0.04761904762	0.04761904762	0.05555555556	0.05555555556	0.7	0.8571428571
CDRT3	0.04761904762	0.04761904762	0.05555555556	0.05555555556	0.7	0.8571428571
CDRT4	0.04761904762	0.04761904762	0.05555555556	0.05555555556	0.7	0.8571428571
CDRT7	0.04761904762	0.04761904762	0.05555555556	0.05555555556	0.7	0.8571428571
CDRT8	0.04761904762	0.04761904762	0.05555555556	0.05555555556	0.7	0.8571428571
CDS1	0	0	0.05555555556	0.05555555556	0	0
CDS2	0.1904761905	0.1904761905	0.05555555556	0.05555555556	3.294117647	3.428571429
CDT1	0.1904761905	0.1904761905	0	0	inf	1.90476E+32
CDV3	0.1428571429	0.1428571429	0.05555555556	0.05555555556	2.333333333	2.571428571
CDYL2	0.09523809524	0.09523809524	0	0	inf	9.52381E+31
EACAM	0.04761904762	0.04761904762	0.05555555556	0.05555555556	0.7	0.8571428571
EACAM	0.09523809524	0.09523809524	0.05555555556	0.05555555556	1.473684211	1.714285714
EACAM	0.09523809524	0.09523809524	0.05555555556	0.05555555556	1.473684211	1.714285714
EACAM	0.09523809524	0.09523809524	0.05555555556	0.05555555556	1.473684211	1.714285714
EACAM	0.04761904762	0.04761904762	0.05555555556	0.05555555556	0.7	0.8571428571
ACAM2	0.09523809524	0.09523809524	0.05555555556	0.05555555556	1.473684211	1.714285714
EACAM	0.04761904762	0.04761904762	0.05555555556	0.05555555556	0.7	0.8571428571
EACAM	0.04761904762	0.04761904762	0.05555555556	0.05555555556	0.7	0.8571428571
EACAM	0.04761904762	0.04761904762	0.05555555556	0.05555555556	0.7	0.8571428571
EACAM	0.04761904762	0.04761904762	0.05555555556	0.05555555556	0.7	0.8571428571
EACAM	0.04761904762	0.04761904762	0.05555555556	0.05555555556	0.7	0.8571428571
EACAM	0.04761904762	0.04761904762	0.05555555556	0.05555555556	0.7	0.8571428571
CEBPA	0.04761904762	0.04761904762	0.05555555556	0.05555555556	0.7	0.8571428571
EBPA-D	0.04761904762	0.04761904762	0.05555555556	0.05555555556	0.7	0.8571428571
CEBPB	0.09523809524	0.09523809524	0.1666666667	0.1666666667	0.4210526316	0.5714285714
EBPB-AS	0.09523809524	0.09523809524	0.1666666667	0.1666666667	0.4210526316	0.5714285714
CEBPD	0.09523809524	0.09523809524	0.1666666667	0.1666666667	0.4210526316	0.5714285714
CEBPE	0	0	0.05555555556	0.05555555556	0	0
CEBPG	0.04761904762	0.04761904762	0.05555555556	0.05555555556	0.7	0.8571428571
CEBPZ	0	0	0.05555555556	0.05555555556	0	0
EBPZO	0	0	0.05555555556	0.05555555556	0	0
CECR2	0.04761904762	0.04761904762	0	0	inf	4.7619E+31
CECR3	0.04761904762	0.04761904762	0	0	inf	4.7619E+31
CECR7	0.04761904762	0.04761904762	0	0	inf	4.7619E+31
CEL	0.04761904762	0.04761904762	0	0	inf	4.7619E+31
CELA1	0.09523809524	0.09523809524	0.05555555556	0.05555555556	1.473684211	1.714285714
CELA2A	0.04761904762	0.04761904762	0.1111111111	0.1111111111	0.325	0.4285714286
CELA2B	0.04761904762	0.04761904762	0.1111111111	0.1111111111	0.325	0.4285714286
CELA3A	0.04761904762	0.04761904762	0.1111111111	0.1111111111	0.325	0.4285714286
CELA3B	0.04761904762	0.04761904762	0.1111111111	0.1111111111	0.325	0.4285714286
CELF1	0	0	0.05555555556	0.05555555556	0	0
CELF2	0.09523809524	0.09523809524	0.1111111111	0.1111111111	0.6842105263	0.8571428571
ELF2-AS	0.09523809524	0.09523809524	0.1111111111	0.1111111111	0.6842105263	0.8571428571
ELF2-AS	0.09523809524	0.09523809524	0.1111111111	0.1111111111	0.6842105263	0.8571428571
ELF2-D	0.09523809524	0.09523809524	0.1111111111	0.1111111111	0.6842105263	0.8571428571
CELF3	0.1428571429	0.1428571429	0	0	inf	1.42857E+32
CELF4	0.04761904762	0.04761904762	0	0	inf	4.7619E+31
CELF5	0.09523809524	0.09523809524	0.05555555556	0.05555555556	1.473684211	1.714285714
CELF6	0.04761904762	0.04761904762	0.05555555556	0.05555555556	0.7	0.8571428571
CELP	0.04761904762	0.04761904762	0	0	inf	4.7619E+31
CELSR1	0	0	0.1111111111	0.1111111111	0	0

CELSR2	0.04761904762	0.04761904762	0.05555555556	0.05555555556	0.7	0.8571428571
CELSR3	0.09523809524	0.09523809524	0	0	inf	9.52381E+31
CEMIP	0.04761904762	0.04761904762	0.05555555556	0.05555555556	0.7	0.8571428571
CEMIP2	0	0	0.05555555556	0.05555555556	0	0
CEMP1	0.04761904762	0.04761904762	0.1111111111	0.1111111111	0.325	0.4285714286
CEND1	0.04761904762	0.04761904762	0	0	inf	4.7619E+31
CENPB	0.1904761905	0.1904761905	0.05555555556	0.05555555556	3.294117647	3.428571429
CENPBD	0.09523809524	0.09523809524	0	0	inf	9.52381E+31
NPBD1	0.1428571429	0.1428571429	0.05555555556	0.05555555556	2.333333333	2.571428571
CENPC	0.04761904762	0.04761904762	0.05555555556	0.05555555556	0.7	0.8571428571
CENPE	0	0	0.05555555556	0.05555555556	0	0
CENPF	0.1428571429	0.1428571429	0	0	inf	1.42857E+32
CENPH	0.04761904762	0.04761904762	0.05555555556	0.05555555556	0.7	0.8571428571
CENPK	0.04761904762	0.04761904762	0	0	inf	4.7619E+31
CENPL	0.1428571429	0.1428571429	0	0	inf	1.42857E+32
CENPM	0	0	0.1111111111	0.1111111111	0	0
CENPN	0.09523809524	0.09523809524	0	0	inf	9.52381E+31
CENPP	0.04761904762	0.04761904762	0.05555555556	0.05555555556	0.7	0.8571428571
CENPS	0.04761904762	0.04761904762	0.1111111111	0.1111111111	0.325	0.4285714286
NPS-CO	0.04761904762	0.04761904762	0.1111111111	0.1111111111	0.325	0.4285714286
CENPT	0.1428571429	0.1428571429	0.05555555556	0.05555555556	2.333333333	2.571428571
CENPU	0	0	0.05555555556	0.05555555556	0	0
CENPX	0.04761904762	0.04761904762	0	0	inf	4.7619E+31
CEP104	0	0	0.05555555556	0.05555555556	0	0
CEP112	0.04761904762	0.04761904762	0	0	inf	4.7619E+31
CEP120	0.04761904762	0.04761904762	0	0	inf	4.7619E+31
CEP131	0.04761904762	0.04761904762	0	0	inf	4.7619E+31
CEP135	0.1904761905	0.1904761905	0.05555555556	0.05555555556	3.294117647	3.428571429
CEP152	0.04761904762	0.04761904762	0.05555555556	0.05555555556	0.7	0.8571428571
CEP170	0.1428571429	0.1428571429	0.05555555556	0.05555555556	2.333333333	2.571428571
EP170P	0	0	0.05555555556	0.05555555556	0	0
CEP19	0.1428571429	0.1428571429	0.05555555556	0.05555555556	2.333333333	2.571428571
CEP192	0.09523809524	0.09523809524	0.05555555556	0.05555555556	1.473684211	1.714285714
CEP250	0.09523809524	0.09523809524	0.1111111111	0.1111111111	0.6842105263	0.8571428571
CEP290	0.04761904762	0.04761904762	0.05555555556	0.05555555556	0.7	0.8571428571
EP295N	0.04761904762	0.04761904762	0	0	inf	4.7619E+31
CEP350	0.1428571429	0.1428571429	0	0	inf	1.42857E+32
CEP41	0.04761904762	0.04761904762	0.05555555556	0.05555555556	0.7	0.8571428571
CEP44	0	0	0.05555555556	0.05555555556	0	0
CEP55	0.1428571429	0.1428571429	0.1111111111	0.1111111111	1.083333333	1.285714286
EP57L1	0	0	0	0		0.00001
CEP63	0.1428571429	0.1428571429	0.05555555556	0.05555555556	2.333333333	2.571428571
CEP68	0	0	0.05555555556	0.05555555556	0	0
CEP70	0.1428571429	0.1428571429	0.05555555556	0.05555555556	2.333333333	2.571428571
CEP72	0	0	0.05555555556	0.05555555556	0	0
CEP76	0.09523809524	0.09523809524	0.05555555556	0.05555555556	1.473684211	1.714285714
CEP78	0	0	0.05555555556	0.05555555556	0	0
CEP83	0.04761904762	0.04761904762	0.05555555556	0.05555555556	0.7	0.8571428571
EP83-D	0.04761904762	0.04761904762	0.05555555556	0.05555555556	0.7	0.8571428571
CEP85	0.04761904762	0.04761904762	0.1111111111	0.1111111111	0.325	0.4285714286
CEP89	0.04761904762	0.04761904762	0.05555555556	0.05555555556	0.7	0.8571428571

**CEP95**	0.04761904762	0.04761904762	0	0	inf	4.7619E+31
**CEP97**	0.09523809524	0.09523809524	0.05555555556	0.05555555556	1.473684211	1.714285714
**CEPT1**	0.04761904762	0.04761904762	0	0	inf	4.7619E+31
**CER1**	0.1428571429	0.1428571429	0.05555555556	0.05555555556	2.333333333	2.571428571
**CERK**	0	0	0.1111111111	0.1111111111	0	0
**CERKL**	0	0	0.05555555556	0.05555555556	0	0
**CERNA1**	0.04761904762	0.04761904762	0.05555555556	0.05555555556	0.7	0.8571428571
**CERNA2**	0.1428571429	0.1428571429	0	0	inf	1.42857E+32
**CERNA3**	0.1428571429	0.1428571429	0.1111111111	0.1111111111	1.083333333	1.285714286
**CERS1**	0.1428571429	0.1428571429	0.1111111111	0.1111111111	1.083333333	1.285714286
**CERS2**	0.1904761905	0.1904761905	0.05555555556	0.05555555556	3.294117647	3.428571429
**CERS3**	0.04761904762	0.04761904762	0.05555555556	0.05555555556	0.7	0.8571428571
**ERS3-AS**	0.04761904762	0.04761904762	0.05555555556	0.05555555556	0.7	0.8571428571
**CERS4**	0.1428571429	0.1428571429	0.05555555556	0.05555555556	2.333333333	2.571428571
**CERS5**	0.09523809524	0.09523809524	0.05555555556	0.05555555556	1.473684211	1.714285714
**CERS6**	0.04761904762	0.04761904762	0.05555555556	0.05555555556	0.7	0.8571428571
**ERS6-AS**	0.04761904762	0.04761904762	0.05555555556	0.05555555556	0.7	0.8571428571
**CES1**	0.04761904762	0.04761904762	0	0	inf	4.7619E+31
**CES1P1**	0.04761904762	0.04761904762	0	0	inf	4.7619E+31
**CES1P2**	0.04761904762	0.04761904762	0	0	inf	4.7619E+31
**CES2**	0.09523809524	0.09523809524	0.05555555556	0.05555555556	1.473684211	1.714285714
**CES3**	0.1428571429	0.1428571429	0.05555555556	0.05555555556	2.333333333	2.571428571
**CES4A**	0.1428571429	0.1428571429	0.05555555556	0.05555555556	2.333333333	2.571428571
**CES5A**	0.04761904762	0.04761904762	0	0	inf	4.7619E+31
**CES5AP1**	0	0	0.05555555556	0.05555555556	0	0
**CETN1**	0.09523809524	0.09523809524	0	0	inf	9.52381E+31
**CETN3**	0.04761904762	0.04761904762	0	0	inf	4.7619E+31
**CETN4P**	0	0	0.05555555556	0.05555555556	0	0
**CETP**	0.04761904762	0.04761904762	0	0	inf	4.7619E+31
**CFAP100**	0.04761904762	0.04761904762	0.05555555556	0.05555555556	0.7	0.8571428571
**CFAP126**	0.1428571429	0.1428571429	0	0	inf	1.42857E+32
**CFAP157**	0.09523809524	0.09523809524	0.05555555556	0.05555555556	1.473684211	1.714285714
**CFAP161**	0.04761904762	0.04761904762	0.05555555556	0.05555555556	0.7	0.8571428571
**CFAP20**	0.04761904762	0.04761904762	0	0	inf	4.7619E+31
**CFAP221**	0	0	0.05555555556	0.05555555556	0	0
**CFAP298**	0.04761904762	0.04761904762	0.1111111111	0.1111111111	0.325	0.4285714286
**CFAP299**	0.04761904762	0.04761904762	0.05555555556	0.05555555556	0.7	0.8571428571
**CFAP36**	0.04761904762	0.04761904762	0.05555555556	0.05555555556	0.7	0.8571428571
**CFAP410**	0.04761904762	0.04761904762	0.1111111111	0.1111111111	0.325	0.4285714286
**CFAP43**	0.1428571429	0.1428571429	0.05555555556	0.05555555556	2.333333333	2.571428571
**CFAP44**	0.09523809524	0.09523809524	0.05555555556	0.05555555556	1.473684211	1.714285714
**FAP44-A**	0.09523809524	0.09523809524	0.05555555556	0.05555555556	1.473684211	1.714285714
**CFAP46**	0.2380952381	0.2380952381	0	0	inf	2.38095E+32
**CFAP52**	0.04761904762	0.04761904762	0.05555555556	0.05555555556	0.7	0.8571428571
**CFAP53**	0.04761904762	0.04761904762	0	0	inf	4.7619E+31
**CFAP54**	0.04761904762	0.04761904762	0.05555555556	0.05555555556	0.7	0.8571428571
**CFAP57**	0.04761904762	0.04761904762	0.05555555556	0.05555555556	0.7	0.8571428571
**CFAP58**	0.1428571429	0.1428571429	0.05555555556	0.05555555556	2.333333333	2.571428571
**FAP58-D**	0.1428571429	0.1428571429	0.05555555556	0.05555555556	2.333333333	2.571428571
**CFAP61**	0.1904761905	0.1904761905	0.05555555556	0.05555555556	3.294117647	3.428571429
**CFAP65**	0	0	0.05555555556	0.05555555556	0	0

CFAP69	0.09523809524	0.09523809524	0.1111111111	0.1111111111	0.6842105263	0.8571428571
CFAP70	0.1904761905	0.1904761905	0.05555555556	0.05555555556	3.294117647	3.428571429
CFAP73	0	0	0.05555555556	0.05555555556	0	0
CFAP74	0	0	0.1111111111	0.1111111111	0	0
CFAP77	0.09523809524	0.09523809524	0	0	inf	9.52381E+31
CFAP97	0	0	0.05555555556	0.05555555556	0	0
CFAP97D	0.09523809524	0.09523809524	0	0	inf	9.52381E+31
CFAP99	0.04761904762	0.04761904762	0	0	inf	4.7619E+31
CFB	0.04761904762	0.04761904762	0	0	inf	4.7619E+31
CFC1	0	0	0.05555555556	0.05555555556	0	0
CFC1B	0	0	0.05555555556	0.05555555556	0	0
CFD	0.09523809524	0.09523809524	0.1111111111	0.1111111111	0.6842105263	0.8571428571
CFDP1	0.04761904762	0.04761904762	0	0	inf	4.7619E+31
CFH	0.1428571429	0.1428571429	0	0	inf	1.42857E+32
CFHR1	0.1428571429	0.1428571429	0	0	inf	1.42857E+32
CFHR2	0.1428571429	0.1428571429	0	0	inf	1.42857E+32
CFHR3	0.1428571429	0.1428571429	0	0	inf	1.42857E+32
CFHR4	0.1428571429	0.1428571429	0	0	inf	1.42857E+32
CFHR5	0.1428571429	0.1428571429	0	0	inf	1.42857E+32
CFI	0	0	0.05555555556	0.05555555556	0	0
CFL1P1	0.1428571429	0.1428571429	0.1666666667	0.1666666667	0.6666666667	0.8571428571
CFL2	0.09523809524	0.09523809524	0	0	inf	9.52381E+31
CFLAR	0	0	0.05555555556	0.05555555556	0	0
CFLAR-AS	0	0	0.05555555556	0.05555555556	0	0
CFTR	0.09523809524	0.09523809524	0.05555555556	0.05555555556	1.473684211	1.714285714
CFTR-AS	0.09523809524	0.09523809524	0.05555555556	0.05555555556	1.473684211	1.714285714
CGAS	0.04761904762	0.04761904762	0	0	inf	4.7619E+31
CGB1	0.09523809524	0.09523809524	0.1111111111	0.1111111111	0.6842105263	0.8571428571
CGB2	0.09523809524	0.09523809524	0.1111111111	0.1111111111	0.6842105263	0.8571428571
CGB3	0.09523809524	0.09523809524	0.1111111111	0.1111111111	0.6842105263	0.8571428571
CGB5	0.09523809524	0.09523809524	0.1111111111	0.1111111111	0.6842105263	0.8571428571
CGB7	0.09523809524	0.09523809524	0.1111111111	0.1111111111	0.6842105263	0.8571428571
CGB8	0.09523809524	0.09523809524	0.1111111111	0.1111111111	0.6842105263	0.8571428571
CGGBP1	0.04761904762	0.04761904762	0	0	inf	4.7619E+31
CGN	0.1904761905	0.1904761905	0.05555555556	0.05555555556	3.294117647	3.428571429
CGNL1	0.04761904762	0.04761904762	0.05555555556	0.05555555556	0.7	0.8571428571
CGRRF1	0.04761904762	0.04761904762	0	0	inf	4.7619E+31
CH25H	0.1428571429	0.1428571429	0.1666666667	0.1666666667	0.6666666667	0.8571428571
CHAC2	0.04761904762	0.04761904762	0.05555555556	0.05555555556	0.7	0.8571428571
CHAD	0.09523809524	0.09523809524	0	0	inf	9.52381E+31
CHADL	0	0	0.1111111111	0.1111111111	0	0
CHAF1B	0.04761904762	0.04761904762	0.1666666667	0.1666666667	0.2	0.2857142857
CHAT	0.09523809524	0.09523809524	0.05555555556	0.05555555556	1.473684211	1.714285714
CHCHD1	0.04761904762	0.04761904762	0.1111111111	0.1111111111	0.325	0.4285714286
CHCHD2	0.2380952381	0.2380952381	0.1666666667	0.1666666667	1.25	1.428571429
CHCHD3	0.04761904762	0.04761904762	0.05555555556	0.05555555556	0.7	0.8571428571
CHCHD4	0.04761904762	0.04761904762	0	0	inf	4.7619E+31
CHCHD5	0	0	0.05555555556	0.05555555556	0	0
CHCHD6	0.09523809524	0.09523809524	0.05555555556	0.05555555556	1.473684211	1.714285714
CHCHD7	0.1428571429	0.1428571429	0.1111111111	0.1111111111	1.083333333	1.285714286
CHD1	0.04761904762	0.04761904762	0	0	inf	4.7619E+31

**CHD1L**	0.1428571429	0.1428571429	0.05555555556	0.05555555556	2.333333333	2.571428571
**CHD2**	0.04761904762	0.04761904762	0.05555555556	0.05555555556	0.7	0.8571428571
**CHD3**	0	0	0.05555555556	0.05555555556	0	0
**CHD4**	0.04761904762	0.04761904762	0.1111111111	0.1111111111	0.325	0.4285714286
**CHD5**	0	0	0.05555555556	0.05555555556	0	0
**CHD6**	0.1428571429	0.1428571429	0.1666666667	0.1666666667	0.6666666667	0.8571428571
**CHD7**	0.1428571429	0.1428571429	0.1666666667	0.1666666667	0.6666666667	0.8571428571
**CHD9**	0.04761904762	0.04761904762	0.05555555556	0.05555555556	0.7	0.8571428571
**CHDH**	0.04761904762	0.04761904762	0	0	inf	4.7619E+31
**CHEK2**	0.04761904762	0.04761904762	0.1111111111	0.1111111111	0.325	0.4285714286
**HEK2P**	0.2857142857	0.2857142857	0.1111111111	0.1111111111	2.6	2.571428571
**CHERP**	0.1428571429	0.1428571429	0.1111111111	0.1111111111	1.083333333	1.285714286
**CHFR**	0.09523809524	0.09523809524	0.05555555556	0.05555555556	1.473684211	1.714285714
**CHGB**	0.1904761905	0.1904761905	0.05555555556	0.05555555556	3.294117647	3.428571429
**CHI3L1**	0.1428571429	0.1428571429	0	0	inf	1.42857E+32
**CHI3L2**	0.04761904762	0.04761904762	0	0	inf	4.7619E+31
**CHIA**	0.04761904762	0.04761904762	0	0	inf	4.7619E+31
**CHIAP2**	0.04761904762	0.04761904762	0	0	inf	4.7619E+31
**CHIC2**	0.2380952381	0.2380952381	0.05555555556	0.05555555556	4.375	4.285714286
**CHID1**	0.04761904762	0.04761904762	0	0	inf	4.7619E+31
**CHIT1**	0.1428571429	0.1428571429	0	0	inf	1.42857E+32
**CHKB**	0	0	0.1111111111	0.1111111111	0	0
**KB-CPT**	0	0	0.1111111111	0.1111111111	0	0
**HKB-D**	0	0	0.1111111111	0.1111111111	0	0
**CHL1**	0.04761904762	0.04761904762	0.05555555556	0.05555555556	0.7	0.8571428571
**HL1-AS**	0.04761904762	0.04761904762	0.05555555556	0.05555555556	0.7	0.8571428571
**HL1-AS**	0.04761904762	0.04761904762	0.05555555556	0.05555555556	0.7	0.8571428571
**CHML**	0.1428571429	0.1428571429	0	0	inf	1.42857E+32
**CHMP1A**	0.09523809524	0.09523809524	0	0	inf	9.52381E+31
**CHMP1B**	0.09523809524	0.09523809524	0.05555555556	0.05555555556	1.473684211	1.714285714
**CHMP2A**	0.1428571429	0.1428571429	0.05555555556	0.05555555556	2.333333333	2.571428571
**CHMP2B**	0.04761904762	0.04761904762	0	0	inf	4.7619E+31
**CHMP3**	0	0	0.05555555556	0.05555555556	0	0
**CHMP4B**	0.09523809524	0.09523809524	0.1111111111	0.1111111111	0.6842105263	0.8571428571
**CHMP4C**	0.1428571429	0.1428571429	0.1111111111	0.1111111111	1.083333333	1.285714286
**CHMP5**	0.09523809524	0.09523809524	0.05555555556	0.05555555556	1.473684211	1.714285714
**CHMP6**	0.04761904762	0.04761904762	0	0	inf	4.7619E+31
**CHMP7**	0.04761904762	0.04761904762	0.1111111111	0.1111111111	0.325	0.4285714286
**CHN1**	0	0	0.1111111111	0.1111111111	0	0
**CHN2**	0.04761904762	0.04761904762	0.1666666667	0.1666666667	0.2	0.2857142857
**CHODL**	0.04761904762	0.04761904762	0.05555555556	0.05555555556	0.7	0.8571428571
**HODL-A**	0.04761904762	0.04761904762	0.05555555556	0.05555555556	0.7	0.8571428571
**CHP2**	0.04761904762	0.04761904762	0	0	inf	4.7619E+31
**CHPF**	0	0	0.05555555556	0.05555555556	0	0
**CHPF2**	0.09523809524	0.09523809524	0.05555555556	0.05555555556	1.473684211	1.714285714
**CHPT1**	0.04761904762	0.04761904762	0.05555555556	0.05555555556	0.7	0.8571428571
**CHRAC1**	0.04761904762	0.04761904762	0.1666666667	0.1666666667	0.2	0.2857142857
**CHRD**	0.1428571429	0.1428571429	0.05555555556	0.05555555556	2.333333333	2.571428571
**CHRM2**	0.04761904762	0.04761904762	0.05555555556	0.05555555556	0.7	0.8571428571
**CHRM3**	0.1428571429	0.1428571429	0	0	inf	1.42857E+32
**HRM3-A**	0.1428571429	0.1428571429	0	0	inf	1.42857E+32

HRM3-A	0.1428571429	0.1428571429	0	0	inf	1.42857E+32
CHRM4	0	0	0.05555555556	0.05555555556	0	0
CHRNA1	0	0	0.1111111111	0.1111111111	0	0
CHRNA1	0.04761904762	0.04761904762	0	0	inf	4.7619E+31
CHRNA2	0.04761904762	0.04761904762	0.1111111111	0.1111111111	0.325	0.4285714286
CHRNA3	0.04761904762	0.04761904762	0.05555555556	0.05555555556	0.7	0.8571428571
CHRNA4	0.1428571429	0.1428571429	0.2222222222	0.2222222222	0.4583333333	0.6428571429
CHRNA5	0.04761904762	0.04761904762	0.05555555556	0.05555555556	0.7	0.8571428571
CHRNA6	0.09523809524	0.09523809524	0.1666666667	0.1666666667	0.4210526316	0.5714285714
CHRNA9	0.1904761905	0.1904761905	0.05555555556	0.05555555556	3.294117647	3.428571429
CHRNB1	0	0	0.05555555556	0.05555555556	0	0
CHRNB2	0.1904761905	0.1904761905	0.1111111111	0.1111111111	1.529411765	1.714285714
CHRNB3	0.09523809524	0.09523809524	0.1666666667	0.1666666667	0.4210526316	0.5714285714
CHRNB4	0.04761904762	0.04761904762	0.05555555556	0.05555555556	0.7	0.8571428571
CHRND	0	0	0.05555555556	0.05555555556	0	0
CHRNE	0	0	0.1111111111	0.1111111111	0	0
CHRNG	0	0	0.05555555556	0.05555555556	0	0
CHST10	0	0	0.05555555556	0.05555555556	0	0
CHST11	0.04761904762	0.04761904762	0.05555555556	0.05555555556	0.7	0.8571428571
CHST12	0.04761904762	0.04761904762	0.1666666667	0.1666666667	0.2	0.2857142857
CHST13	0.04761904762	0.04761904762	0.05555555556	0.05555555556	0.7	0.8571428571
CHST15	0.2380952381	0.2380952381	0.05555555556	0.05555555556	4.375	4.285714286
CHST2	0.1428571429	0.1428571429	0.05555555556	0.05555555556	2.333333333	2.571428571
CHST3	0.1428571429	0.1428571429	0	0	inf	1.42857E+32
CHST4	0.04761904762	0.04761904762	0	0	inf	4.7619E+31
CHST5	0.04761904762	0.04761904762	0	0	inf	4.7619E+31
CHST6	0.04761904762	0.04761904762	0	0	inf	4.7619E+31
CHST8	0.04761904762	0.04761904762	0.05555555556	0.05555555556	0.7	0.8571428571
CHST9	0.09523809524	0.09523809524	0.05555555556	0.05555555556	1.473684211	1.714285714
CHSY1	0.04761904762	0.04761904762	0.05555555556	0.05555555556	0.7	0.8571428571
CHSY3	0.04761904762	0.04761904762	0	0	inf	4.7619E+31
CHTF18	0.04761904762	0.04761904762	0.2222222222	0.2222222222	0.1375	0.2142857143
CHTF8	0.1428571429	0.1428571429	0.05555555556	0.05555555556	2.333333333	2.571428571
CHTOP	0.1904761905	0.1904761905	0.05555555556	0.05555555556	3.294117647	3.428571429
CHUK	0.1428571429	0.1428571429	0	0	inf	1.42857E+32
CIAO1	0	0	0.1111111111	0.1111111111	0	0
CIAO2A	0.04761904762	0.04761904762	0.05555555556	0.05555555556	0.7	0.8571428571
CIAO2B	0.09523809524	0.09523809524	0.05555555556	0.05555555556	1.473684211	1.714285714
CIAO3	0.04761904762	0.04761904762	0.2222222222	0.2222222222	0.1375	0.2142857143
CIAPIN1	0.04761904762	0.04761904762	0	0	inf	4.7619E+31
CIART	0.1904761905	0.1904761905	0.05555555556	0.05555555556	3.294117647	3.428571429
CIB1	0.04761904762	0.04761904762	0.05555555556	0.05555555556	0.7	0.8571428571
CIB2	0.04761904762	0.04761904762	0.05555555556	0.05555555556	0.7	0.8571428571
CIB3	0.1428571429	0.1428571429	0.1111111111	0.1111111111	1.083333333	1.285714286
CIC	0.04761904762	0.04761904762	0.05555555556	0.05555555556	0.7	0.8571428571
CIDEA	0.09523809524	0.09523809524	0.05555555556	0.05555555556	1.473684211	1.714285714
CIDEC	0.09523809524	0.09523809524	0	0	inf	9.52381E+31
CIDECP	0.09523809524	0.09523809524	0	0	inf	9.52381E+31
CIITA	0.04761904762	0.04761904762	0	0	inf	4.7619E+31
CILP	0.04761904762	0.04761904762	0.05555555556	0.05555555556	0.7	0.8571428571
CILP2	0.1428571429	0.1428571429	0.1111111111	0.1111111111	1.083333333	1.285714286

**CIP2A**	0.09523809524	0.09523809524	0.05555555556	0.05555555556	1.473684211	1.714285714
**CIR1**	0	0	0.1111111111	0.1111111111	0	0
**CIRBP**	0.09523809524	0.09523809524	0.1111111111	0.1111111111	0.6842105263	0.8571428571
**IRBP-AS**	0.09523809524	0.09523809524	0.1111111111	0.1111111111	0.6842105263	0.8571428571
**CISD1**	0.09523809524	0.09523809524	0.05555555556	0.05555555556	1.473684211	1.714285714
**CISD2**	0	0	0.05555555556	0.05555555556	0	0
**CISD3**	0.04761904762	0.04761904762	0	0	inf	4.7619E+31
**CISH**	0.09523809524	0.09523809524	0	0	inf	9.52381E+31
**CISTR**	0.04761904762	0.04761904762	0.05555555556	0.05555555556	0.7	0.8571428571
**CIT**	0	0	0.1111111111	0.1111111111	0	0
**CITED4**	0.04761904762	0.04761904762	0.05555555556	0.05555555556	0.7	0.8571428571
**CIZ1**	0.09523809524	0.09523809524	0.05555555556	0.05555555556	1.473684211	1.714285714
**CKAP2L**	0	0	0.05555555556	0.05555555556	0	0
**CKAP4**	0.04761904762	0.04761904762	0.05555555556	0.05555555556	0.7	0.8571428571
**CKAP5**	0	0	0.05555555556	0.05555555556	0	0
**CKLF**	0.04761904762	0.04761904762	0	0	inf	4.7619E+31
**LF-CMT**	0.04761904762	0.04761904762	0	0	inf	4.7619E+31
**CKM**	0.09523809524	0.09523809524	0.1111111111	0.1111111111	0.6842105263	0.8571428571
**CKMT2**	0.04761904762	0.04761904762	0	0	inf	4.7619E+31
**KMT2-A**	0.04761904762	0.04761904762	0	0	inf	4.7619E+31
**CKS1B**	0.1904761905	0.1904761905	0.1111111111	0.1111111111	1.529411765	1.714285714
**CKS2**	0	0	0.05555555556	0.05555555556	0	0
**CLASP1**	0	0	0.05555555556	0.05555555556	0	0
**CLASP2**	0.09523809524	0.09523809524	0	0	inf	9.52381E+31
**CLASRP**	0.09523809524	0.09523809524	0.1111111111	0.1111111111	0.6842105263	0.8571428571
**CLC**	0.04761904762	0.04761904762	0.05555555556	0.05555555556	0.7	0.8571428571
**CLCA1**	0.04761904762	0.04761904762	0.05555555556	0.05555555556	0.7	0.8571428571
**CLCA2**	0.04761904762	0.04761904762	0.05555555556	0.05555555556	0.7	0.8571428571
**CLCA3P**	0.04761904762	0.04761904762	0.05555555556	0.05555555556	0.7	0.8571428571
**CLCA4**	0.04761904762	0.04761904762	0.05555555556	0.05555555556	0.7	0.8571428571
**LCA4-AS**	0.04761904762	0.04761904762	0.05555555556	0.05555555556	0.7	0.8571428571
**CLCC1**	0.04761904762	0.04761904762	0.05555555556	0.05555555556	0.7	0.8571428571
**CLCN1**	0.04761904762	0.04761904762	0.05555555556	0.05555555556	0.7	0.8571428571
**CLCN2**	0.1428571429	0.1428571429	0.05555555556	0.05555555556	2.333333333	2.571428571
**CLCN3**	0	0	0.05555555556	0.05555555556	0	0
**CLCN6**	0.04761904762	0.04761904762	0.1111111111	0.1111111111	0.325	0.4285714286
**CLCN7**	0.09523809524	0.09523809524	0.2222222222	0.2222222222	0.2894736842	0.4285714286
**CLCNKA**	0.04761904762	0.04761904762	0.1111111111	0.1111111111	0.325	0.4285714286
**CLCNKB**	0.04761904762	0.04761904762	0.1111111111	0.1111111111	0.325	0.4285714286
**CLDN1**	0.09523809524	0.09523809524	0.1111111111	0.1111111111	0.6842105263	0.8571428571
**CLDN11**	0.1904761905	0.1904761905	0.05555555556	0.05555555556	3.294117647	3.428571429
**CLDN12**	0.09523809524	0.09523809524	0.1111111111	0.1111111111	0.6842105263	0.8571428571
**CLDN14**	0.04761904762	0.04761904762	0.1666666667	0.1666666667	0.2	0.2857142857
**CLDN15**	0.04761904762	0.04761904762	0.05555555556	0.05555555556	0.7	0.8571428571
**CLDN16**	0.09523809524	0.09523809524	0.1111111111	0.1111111111	0.6842105263	0.8571428571
**CLDN17**	0.04761904762	0.04761904762	0.05555555556	0.05555555556	0.7	0.8571428571
**CLDN18**	0.1428571429	0.1428571429	0.05555555556	0.05555555556	2.333333333	2.571428571
**CLDN19**	0.04761904762	0.04761904762	0.05555555556	0.05555555556	0.7	0.8571428571
**CLDN22**	0	0	0.05555555556	0.05555555556	0	0
**CLDN23**	0.09523809524	0.09523809524	0.1111111111	0.1111111111	0.6842105263	0.8571428571
**CLDN24**	0	0	0.05555555556	0.05555555556	0	0

CLDN3	0.1428571429	0.1428571429	0.1111111111	0.1111111111	1.083333333	1.285714286
CLDN4	0.1428571429	0.1428571429	0.1111111111	0.1111111111	1.083333333	1.285714286
CLDN5	0.04761904762	0.04761904762	0.05555555556	0.05555555556	0.7	0.8571428571
CLDN6	0.04761904762	0.04761904762	0.1111111111	0.1111111111	0.325	0.4285714286
CLDN7	0	0	0.05555555556	0.05555555556	0	0
CLDN8	0.04761904762	0.04761904762	0.05555555556	0.05555555556	0.7	0.8571428571
CLDN9	0.04761904762	0.04761904762	0.1111111111	0.1111111111	0.325	0.4285714286
CLDND1	0.04761904762	0.04761904762	0.05555555556	0.05555555556	0.7	0.8571428571
CLEC10A	0	0	0.05555555556	0.05555555556	0	0
CLEC11A	0.09523809524	0.09523809524	0.05555555556	0.05555555556	1.473684211	1.714285714
CLEC12A	0.04761904762	0.04761904762	0.1111111111	0.1111111111	0.325	0.4285714286
EC12A-A	0.04761904762	0.04761904762	0.1111111111	0.1111111111	0.325	0.4285714286
CLEC12B	0.04761904762	0.04761904762	0.1111111111	0.1111111111	0.325	0.4285714286
CLEC16A	0.04761904762	0.04761904762	0	0	inf	4.7619E+31
CLEC17A	0.1428571429	0.1428571429	0.1111111111	0.1111111111	1.083333333	1.285714286
CLEC18A	0.1428571429	0.1428571429	0.05555555556	0.05555555556	2.333333333	2.571428571
CLEC18C	0.1428571429	0.1428571429	0.05555555556	0.05555555556	2.333333333	2.571428571
CLEC19A	0.04761904762	0.04761904762	0	0	inf	4.7619E+31
CLEC1A	0.04761904762	0.04761904762	0.1111111111	0.1111111111	0.325	0.4285714286
CLEC1B	0.04761904762	0.04761904762	0.1111111111	0.1111111111	0.325	0.4285714286
CLEC2A	0.04761904762	0.04761904762	0.1111111111	0.1111111111	0.325	0.4285714286
CLEC2B	0.04761904762	0.04761904762	0.1111111111	0.1111111111	0.325	0.4285714286
CLEC2D	0.04761904762	0.04761904762	0.1111111111	0.1111111111	0.325	0.4285714286
CLEC2L	0.04761904762	0.04761904762	0.05555555556	0.05555555556	0.7	0.8571428571
CLEC3A	0.09523809524	0.09523809524	0	0	inf	9.52381E+31
CLEC4A	0.04761904762	0.04761904762	0.1111111111	0.1111111111	0.325	0.4285714286
CLEC4C	0.04761904762	0.04761904762	0.1111111111	0.1111111111	0.325	0.4285714286
CLEC4D	0.04761904762	0.04761904762	0.1111111111	0.1111111111	0.325	0.4285714286
CLEC4E	0.04761904762	0.04761904762	0.1111111111	0.1111111111	0.325	0.4285714286
CLEC4G	0.1428571429	0.1428571429	0.05555555556	0.05555555556	2.333333333	2.571428571
LEC4GP	0.1428571429	0.1428571429	0.05555555556	0.05555555556	2.333333333	2.571428571
CLEC4M	0.1428571429	0.1428571429	0.05555555556	0.05555555556	2.333333333	2.571428571
CLEC5A	0.04761904762	0.04761904762	0.05555555556	0.05555555556	0.7	0.8571428571
CLEC6A	0.04761904762	0.04761904762	0.1111111111	0.1111111111	0.325	0.4285714286
CLEC7A	0.04761904762	0.04761904762	0.1111111111	0.1111111111	0.325	0.4285714286
CLEC9A	0.04761904762	0.04761904762	0.1111111111	0.1111111111	0.325	0.4285714286
CLECL1	0.04761904762	0.04761904762	0.1111111111	0.1111111111	0.325	0.4285714286
CLGN	0	0	0.05555555556	0.05555555556	0	0
CLHC1	0.04761904762	0.04761904762	0.05555555556	0.05555555556	0.7	0.8571428571
CLIC1	0.04761904762	0.04761904762	0	0	inf	4.7619E+31
CLIC3	0.1428571429	0.1428571429	0.05555555556	0.05555555556	2.333333333	2.571428571
CLIC4	0.04761904762	0.04761904762	0.1111111111	0.1111111111	0.325	0.4285714286
CLIC5	0.04761904762	0.04761904762	0.1111111111	0.1111111111	0.325	0.4285714286
CLIC6	0.04761904762	0.04761904762	0.1666666667	0.1666666667	0.2	0.2857142857
CLINT1	0.04761904762	0.04761904762	0.05555555556	0.05555555556	0.7	0.8571428571
CLIP1	0.04761904762	0.04761904762	0.1111111111	0.1111111111	0.325	0.4285714286
LIP1-AS	0.04761904762	0.04761904762	0.1111111111	0.1111111111	0.325	0.4285714286
CLIP2	0.1428571429	0.1428571429	0.1111111111	0.1111111111	1.083333333	1.285714286
CLIP3	0.04761904762	0.04761904762	0.05555555556	0.05555555556	0.7	0.8571428571
CLIP4	0	0	0.05555555556	0.05555555556	0	0
CLK1	0	0	0.05555555556	0.05555555556	0	0

CLK2	0.1904761905	0.1904761905	0.1111111111	0.1111111111	1.529411765	1.714285714
CLK2P1	0.04761904762	0.04761904762	0.1666666667	0.1666666667	0.2	0.2857142857
CLK3	0.04761904762	0.04761904762	0.05555555556	0.05555555556	0.7	0.8571428571
CLK4	0.09523809524	0.09523809524	0.05555555556	0.05555555556	1.473684211	1.714285714
CLLU1	0.04761904762	0.04761904762	0.05555555556	0.05555555556	0.7	0.8571428571
CLLU1O	0.04761904762	0.04761904762	0.05555555556	0.05555555556	0.7	0.8571428571
CLN3	0.04761904762	0.04761904762	0	0	inf	4.7619E+31
CLN5	0	0	0.05555555556	0.05555555556	0	0
CLN6	0.04761904762	0.04761904762	0.05555555556	0.05555555556	0.7	0.8571428571
CLN8	0.1428571429	0.1428571429	0.1111111111	0.1111111111	1.083333333	1.285714286
CLNK	0	0	0	0		0.00001
CLOCK	0.1904761905	0.1904761905	0.05555555556	0.05555555556	3.294117647	3.428571429
CLPP	0.1428571429	0.1428571429	0.05555555556	0.05555555556	2.333333333	2.571428571
CLPS	0.04761904762	0.04761904762	0.05555555556	0.05555555556	0.7	0.8571428571
CLPSL1	0.04761904762	0.04761904762	0.05555555556	0.05555555556	0.7	0.8571428571
CLPSL2	0.04761904762	0.04761904762	0.05555555556	0.05555555556	0.7	0.8571428571
CLPTM1	0.09523809524	0.09523809524	0.1111111111	0.1111111111	0.6842105263	0.8571428571
LPTM1	0	0	0.1111111111	0.1111111111	0	0
CLPX	0.04761904762	0.04761904762	0.05555555556	0.05555555556	0.7	0.8571428571
CLRN1	0.1904761905	0.1904761905	0.1111111111	0.1111111111	1.529411765	1.714285714
LRN1-A	0.1904761905	0.1904761905	0.1111111111	0.1111111111	1.529411765	1.714285714
CLRN2	0	0	0	0		0.00001
CLRN3	0.2380952381	0.2380952381	0.05555555556	0.05555555556	4.375	4.285714286
CLSPN	0.04761904762	0.04761904762	0.05555555556	0.05555555556	0.7	0.8571428571
CLSTN1	0	0	0.05555555556	0.05555555556	0	0
CLSTN2	0.1428571429	0.1428571429	0.05555555556	0.05555555556	2.333333333	2.571428571
STN2-A	0.1428571429	0.1428571429	0.05555555556	0.05555555556	2.333333333	2.571428571
CLSTN3	0.04761904762	0.04761904762	0.1111111111	0.1111111111	0.325	0.4285714286
CLTA	0.09523809524	0.09523809524	0.05555555556	0.05555555556	1.473684211	1.714285714
CLTB	0.09523809524	0.09523809524	0.05555555556	0.05555555556	1.473684211	1.714285714
CLTC	0.1428571429	0.1428571429	0	0	inf	1.42857E+32
CLTCL1	0.04761904762	0.04761904762	0.05555555556	0.05555555556	0.7	0.8571428571
CLU	0.04761904762	0.04761904762	0.1111111111	0.1111111111	0.325	0.4285714286
CLUAP1	0.04761904762	0.04761904762	0.1111111111	0.1111111111	0.325	0.4285714286
CLUH	0	0	0.1111111111	0.1111111111	0	0
CLUHP3	0.04761904762	0.04761904762	0	0	inf	4.7619E+31
CLUL1	0.09523809524	0.09523809524	0	0	inf	9.52381E+31
CLVS1	0.1428571429	0.1428571429	0.1666666667	0.1666666667	0.6666666667	0.8571428571
CMAHP	0	0	0.05555555556	0.05555555556	0	0
CMAS	0.04761904762	0.04761904762	0.05555555556	0.05555555556	0.7	0.8571428571
CMBL	0.04761904762	0.04761904762	0.05555555556	0.05555555556	0.7	0.8571428571
CMC1	0.04761904762	0.04761904762	0	0	inf	4.7619E+31
CMC2	0.09523809524	0.09523809524	0	0	inf	9.52381E+31
CMIP	0.04761904762	0.04761904762	0	0	inf	4.7619E+31
CMKLR	0.04761904762	0.04761904762	0.05555555556	0.05555555556	0.7	0.8571428571
CMPK1	0.04761904762	0.04761904762	0.05555555556	0.05555555556	0.7	0.8571428571
CMPK2	0	0	0.05555555556	0.05555555556	0	0
CMSS1	0.09523809524	0.09523809524	0.1111111111	0.1111111111	0.6842105263	0.8571428571
CMTM1	0.04761904762	0.04761904762	0	0	inf	4.7619E+31
CMTM2	0.04761904762	0.04761904762	0	0	inf	4.7619E+31
CMTM3	0.04761904762	0.04761904762	0	0	inf	4.7619E+31

CMTM4	0.09523809524	0.09523809524	0.05555555556	0.05555555556	1.473684211	1.714285714
CMTM5	0	0	0.05555555556	0.05555555556	0	0
CMTM6	0.09523809524	0.09523809524	0	0	inf	9.52381E+31
CMTM7	0.09523809524	0.09523809524	0	0	inf	9.52381E+31
CMTM8	0.09523809524	0.09523809524	0	0	inf	9.52381E+31
CMTR1	0.04761904762	0.04761904762	0.05555555556	0.05555555556	0.7	0.8571428571
CMTR2	0.04761904762	0.04761904762	0	0	inf	4.7619E+31
CMYA5	0.04761904762	0.04761904762	0	0	inf	4.7619E+31
CNBD1	0.1904761905	0.1904761905	0.1666666667	0.1666666667	0.9411764706	1.142857143
CNBD2	0.09523809524	0.09523809524	0.1111111111	0.1111111111	0.6842105263	0.8571428571
CNBP	0.09523809524	0.09523809524	0.05555555556	0.05555555556	1.473684211	1.714285714
CNEP1R	0.04761904762	0.04761904762	0	0	inf	4.7619E+31
CNFN	0.04761904762	0.04761904762	0.05555555556	0.05555555556	0.7	0.8571428571
CNGA1	0.04761904762	0.04761904762	0.1111111111	0.1111111111	0.325	0.4285714286
CNGA3	0	0	0.05555555556	0.05555555556	0	0
CNGA4	0.04761904762	0.04761904762	0	0	inf	4.7619E+31
CNGB1	0.04761904762	0.04761904762	0	0	inf	4.7619E+31
CNGB3	0.1904761905	0.1904761905	0.1111111111	0.1111111111	1.529411765	1.714285714
CNIH1	0.04761904762	0.04761904762	0	0	inf	4.7619E+31
CNIH3	0.1428571429	0.1428571429	0.05555555556	0.05555555556	2.333333333	2.571428571
CNIH4	0.1428571429	0.1428571429	0.05555555556	0.05555555556	2.333333333	2.571428571
CNKSR1	0.04761904762	0.04761904762	0.1111111111	0.1111111111	0.325	0.4285714286
CNN1	0.1428571429	0.1428571429	0.1111111111	0.1111111111	1.083333333	1.285714286
CNN2	0.09523809524	0.09523809524	0.1111111111	0.1111111111	0.6842105263	0.8571428571
CNN3	0.04761904762	0.04761904762	0.05555555556	0.05555555556	0.7	0.8571428571
CNNM1	0.1428571429	0.1428571429	0	0	inf	1.42857E+32
CNNM2	0.1428571429	0.1428571429	0.05555555556	0.05555555556	2.333333333	2.571428571
CNNM3	0	0	0.1111111111	0.1111111111	0	0
NNM3-D	0	0	0.1111111111	0.1111111111	0	0
CNNM4	0	0	0.1111111111	0.1111111111	0	0
CNOT1	0.04761904762	0.04761904762	0	0	inf	4.7619E+31
CNOT10	0.09523809524	0.09523809524	0	0	inf	9.52381E+31
CNOT11	0	0	0.05555555556	0.05555555556	0	0
CNOT2	0.09523809524	0.09523809524	0.1111111111	0.1111111111	0.6842105263	0.8571428571
CNOT3	0.09523809524	0.09523809524	0.05555555556	0.05555555556	1.473684211	1.714285714
CNOT4	0.04761904762	0.04761904762	0.05555555556	0.05555555556	0.7	0.8571428571
CNOT6	0.09523809524	0.09523809524	0.05555555556	0.05555555556	1.473684211	1.714285714
CNOT6L	0.04761904762	0.04761904762	0.05555555556	0.05555555556	0.7	0.8571428571
CNOT7	0.09523809524	0.09523809524	0.1111111111	0.1111111111	0.6842105263	0.8571428571
CNOT8	0	0	0.05555555556	0.05555555556	0	0
CNOT9	0	0	0.05555555556	0.05555555556	0	0
CNP	0.09523809524	0.09523809524	0	0	inf	9.52381E+31
CNPY1	0.1428571429	0.1428571429	0.05555555556	0.05555555556	2.333333333	2.571428571
CNPY2	0.1428571429	0.1428571429	0.05555555556	0.05555555556	2.333333333	2.571428571
CNPY3	0.04761904762	0.04761904762	0.05555555556	0.05555555556	0.7	0.8571428571
PY3-GN	0.04761904762	0.04761904762	0.05555555556	0.05555555556	0.7	0.8571428571
CNPY4	0.04761904762	0.04761904762	0.05555555556	0.05555555556	0.7	0.8571428571
CNR2	0.04761904762	0.04761904762	0.1111111111	0.1111111111	0.325	0.4285714286
CNRIP1	0	0	0.05555555556	0.05555555556	0	0
CNST	0.1428571429	0.1428571429	0.05555555556	0.05555555556	2.333333333	2.571428571
CNTD1	0.04761904762	0.04761904762	0	0	inf	4.7619E+31

**CNTD2**	0.04761904762	0.04761904762	0.05555555556	0.05555555556	0.7	0.8571428571
**CNTFR**	0.09523809524	0.09523809524	0.05555555556	0.05555555556	1.473684211	1.714285714
**NTFR-A**	0.09523809524	0.09523809524	0.05555555556	0.05555555556	1.473684211	1.714285714
**CNTLN**	0.04761904762	0.04761904762	0.05555555556	0.05555555556	0.7	0.8571428571
**CNTN1**	0.04761904762	0.04761904762	0.05555555556	0.05555555556	0.7	0.8571428571
**CNTN2**	0.2380952381	0.2380952381	0	0	inf	2.38095E+32
**CNTN3**	0.04761904762	0.04761904762	0	0	inf	4.7619E+31
**CNTN4**	0.04761904762	0.04761904762	0	0	inf	4.7619E+31
**NTN4-A**	0.04761904762	0.04761904762	0	0	inf	4.7619E+31
**NTN4-A**	0.04761904762	0.04761904762	0	0	inf	4.7619E+31
**CNTN6**	0.04761904762	0.04761904762	0	0	inf	4.7619E+31
**CNTNAP**	0.04761904762	0.04761904762	0	0	inf	4.7619E+31
**CNTNAP**	0.09523809524	0.09523809524	0.05555555556	0.05555555556	1.473684211	1.714285714
**CNTNAP**	0.04761904762	0.04761904762	0.1111111111	0.1111111111	0.325	0.4285714286
**NTNAP3**	0.1428571429	0.1428571429	0.05555555556	0.05555555556	2.333333333	2.571428571
**NTNAP3**	0.1428571429	0.1428571429	0.05555555556	0.05555555556	2.333333333	2.571428571
**CNTNAP**	0.04761904762	0.04761904762	0	0	inf	4.7619E+31
**CNTNAP**	0	0	0.05555555556	0.05555555556	0	0
**CNTRL**	0.09523809524	0.09523809524	0.05555555556	0.05555555556	1.473684211	1.714285714
**CNTROB**	0	0	0.05555555556	0.05555555556	0	0
**COA1**	0.04761904762	0.04761904762	0.1666666667	0.1666666667	0.2	0.2857142857
**COA3**	0.04761904762	0.04761904762	0	0	inf	4.7619E+31
**COA5**	0	0	0.05555555556	0.05555555556	0	0
**COA6**	0.09523809524	0.09523809524	0	0	inf	9.52381E+31
**OA6-AS**	0.09523809524	0.09523809524	0	0	inf	9.52381E+31
**COA7**	0.04761904762	0.04761904762	0.05555555556	0.05555555556	0.7	0.8571428571
**COBL**	0.1428571429	0.1428571429	0.1666666667	0.1666666667	0.6666666667	0.8571428571
**COBLL1**	0	0	0.05555555556	0.05555555556	0	0
**COCH**	0	0	0	0		0.00001
**COG1**	0.04761904762	0.04761904762	0	0	inf	4.7619E+31
**COG2**	0.1428571429	0.1428571429	0	0	inf	1.42857E+32
**COG4**	0.1428571429	0.1428571429	0.05555555556	0.05555555556	2.333333333	2.571428571
**COG5**	0.04761904762	0.04761904762	0.05555555556	0.05555555556	0.7	0.8571428571
**COG7**	0.04761904762	0.04761904762	0	0	inf	4.7619E+31
**COG8**	0.1428571429	0.1428571429	0.05555555556	0.05555555556	2.333333333	2.571428571
**COIL**	0.09523809524	0.09523809524	0	0	inf	9.52381E+31
**COL11A**	0.04761904762	0.04761904762	0.05555555556	0.05555555556	0.7	0.8571428571
**COL11A**	0.04761904762	0.04761904762	0	0	inf	4.7619E+31
**COL13A**	0.09523809524	0.09523809524	0	0	inf	9.52381E+31
**COL14A**	0.09523809524	0.09523809524	0.1666666667	0.1666666667	0.4210526316	0.5714285714
**COL15A**	0.04761904762	0.04761904762	0	0	inf	4.7619E+31
**COL16A**	0.04761904762	0.04761904762	0.1111111111	0.1111111111	0.325	0.4285714286
**COL17A**	0.1428571429	0.1428571429	0.1111111111	0.1111111111	1.083333333	1.285714286
**COL18A**	0.04761904762	0.04761904762	0.1111111111	0.1111111111	0.325	0.4285714286
**L18A1-A**	0.04761904762	0.04761904762	0.1111111111	0.1111111111	0.325	0.4285714286
**L18A1-A**	0.04761904762	0.04761904762	0.1111111111	0.1111111111	0.325	0.4285714286
**COL1A1**	0.09523809524	0.09523809524	0	0	inf	9.52381E+31
**COL1A2**	0.04761904762	0.04761904762	0.1111111111	0.1111111111	0.325	0.4285714286
**OL1A2-A**	0.04761904762	0.04761904762	0.1111111111	0.1111111111	0.325	0.4285714286
**COL20A**	0.1428571429	0.1428571429	0.2222222222	0.2222222222	0.4583333333	0.6428571429
**COL21A**	0.04761904762	0.04761904762	0.05555555556	0.05555555556	0.7	0.8571428571

COL22A	0.04761904762	0.04761904762	0.1111111111	0.1111111111	0.325	0.4285714286
COL23A	0.09523809524	0.09523809524	0.05555555556	0.05555555556	1.473684211	1.714285714
COL24A	0.04761904762	0.04761904762	0.05555555556	0.05555555556	0.7	0.8571428571
COL25A	0	0	0.05555555556	0.05555555556	0	0
COL26A	0.04761904762	0.04761904762	0.05555555556	0.05555555556	0.7	0.8571428571
COL27A	0.04761904762	0.04761904762	0.05555555556	0.05555555556	0.7	0.8571428571
COL28A	0.04761904762	0.04761904762	0.1666666667	0.1666666667	0.2	0.2857142857
COL2A1	0.04761904762	0.04761904762	0.05555555556	0.05555555556	0.7	0.8571428571
COL3A1	0	0	0.1666666667	0.1666666667	0	0
COL4A3	0	0	0.05555555556	0.05555555556	0	0
OL4A3B	0.04761904762	0.04761904762	0	0	inf	4.7619E+31
COL4A4	0	0	0.05555555556	0.05555555556	0	0
COL5A1	0.04761904762	0.04761904762	0	0	inf	4.7619E+31
L5A1-A	0.04761904762	0.04761904762	0	0	inf	4.7619E+31
COL5A2	0	0	0.1666666667	0.1666666667	0	0
COL5A3	0.1428571429	0.1428571429	0.1111111111	0.1111111111	1.083333333	1.285714286
COL6A1	0.04761904762	0.04761904762	0.1111111111	0.1111111111	0.325	0.4285714286
COL6A2	0.04761904762	0.04761904762	0.1111111111	0.1111111111	0.325	0.4285714286
COL6A3	0	0	0.05555555556	0.05555555556	0	0
OL6A4P	0.04761904762	0.04761904762	0	0	inf	4.7619E+31
OL6A4P	0.09523809524	0.09523809524	0.05555555556	0.05555555556	1.473684211	1.714285714
COL6A5	0.09523809524	0.09523809524	0.05555555556	0.05555555556	1.473684211	1.714285714
COL6A6	0.09523809524	0.09523809524	0.05555555556	0.05555555556	1.473684211	1.714285714
COL7A1	0.09523809524	0.09523809524	0	0	inf	9.52381E+31
COL8A1	0.09523809524	0.09523809524	0.1111111111	0.1111111111	0.6842105263	0.8571428571
COL8A2	0.04761904762	0.04761904762	0.05555555556	0.05555555556	0.7	0.8571428571
COL9A2	0.04761904762	0.04761904762	0.05555555556	0.05555555556	0.7	0.8571428571
COL9A3	0.1428571429	0.1428571429	0.2222222222	0.2222222222	0.4583333333	0.6428571429
COLEC1	0.09523809524	0.09523809524	0.1666666667	0.1666666667	0.4210526316	0.5714285714
COLEC1	0	0	0.05555555556	0.05555555556	0	0
COLEC1	0.09523809524	0.09523809524	0	0	inf	9.52381E+31
OLGAL	0.1428571429	0.1428571429	0	0	inf	1.42857E+32
COLQ	0.04761904762	0.04761904762	0	0	inf	4.7619E+31
COMMD	0.04761904762	0.04761904762	0.05555555556	0.05555555556	0.7	0.8571428571
OMMD1	0.04761904762	0.04761904762	0	0	inf	4.7619E+31
COMMD	0.1904761905	0.1904761905	0.1111111111	0.1111111111	1.529411765	1.714285714
COMMD	0.09523809524	0.09523809524	0.1111111111	0.1111111111	0.6842105263	0.8571428571
MMD3-B	0.09523809524	0.09523809524	0.1111111111	0.1111111111	0.6842105263	0.8571428571
COMMD	0.04761904762	0.04761904762	0.05555555556	0.05555555556	0.7	0.8571428571
COMMD	0.09523809524	0.09523809524	0.2222222222	0.2222222222	0.2894736842	0.4285714286
COMMD	0	0	0.05555555556	0.05555555556	0	0
COMMD	0.09523809524	0.09523809524	0.1111111111	0.1111111111	0.6842105263	0.8571428571
COMMD	0.04761904762	0.04761904762	0.05555555556	0.05555555556	0.7	0.8571428571
COMP	0.1428571429	0.1428571429	0.1111111111	0.1111111111	1.083333333	1.285714286
COMT	0.04761904762	0.04761904762	0.05555555556	0.05555555556	0.7	0.8571428571
COMTD	0.1904761905	0.1904761905	0.05555555556	0.05555555556	3.294117647	3.428571429
COP1	0.1428571429	0.1428571429	0	0	inf	1.42857E+32
COPA	0.1428571429	0.1428571429	0	0	inf	1.42857E+32
COPB2	0.1428571429	0.1428571429	0.05555555556	0.05555555556	2.333333333	2.571428571
COPE	0.1428571429	0.1428571429	0.1111111111	0.1111111111	1.083333333	1.285714286
COPG1	0.09523809524	0.09523809524	0.05555555556	0.05555555556	1.473684211	1.714285714

**COPG2**	0.04761904762	0.04761904762	0.05555555556	0.05555555556	0.7	0.8571428571
**COPRS**	0	0	0	0		0.00001
**COPS2**	0.04761904762	0.04761904762	0.05555555556	0.05555555556	0.7	0.8571428571
**COPS3**	0.04761904762	0.04761904762	0.1111111111	0.1111111111	0.325	0.4285714286
**COPS4**	0	0	0.05555555556	0.05555555556	0	0
**COPS5**	0.1428571429	0.1428571429	0.1666666667	0.1666666667	0.6666666667	0.8571428571
**COPS6**	0.04761904762	0.04761904762	0.05555555556	0.05555555556	0.7	0.8571428571
**COPS7A**	0.04761904762	0.04761904762	0.1111111111	0.1111111111	0.325	0.4285714286
**COPS7B**	0	0	0.05555555556	0.05555555556	0	0
**COPS8**	0	0	0.05555555556	0.05555555556	0	0
**COPS9**	0.04761904762	0.04761904762	0.05555555556	0.05555555556	0.7	0.8571428571
**COPZ1**	0.04761904762	0.04761904762	0.05555555556	0.05555555556	0.7	0.8571428571
**COPZ2**	0.1428571429	0.1428571429	0	0	inf	1.42857E+32
**COQ10A**	0.1428571429	0.1428571429	0.05555555556	0.05555555556	2.333333333	2.571428571
**COQ10B**	0	0	0.05555555556	0.05555555556	0	0
**COQ2**	0	0	0.05555555556	0.05555555556	0	0
**COQ4**	0.09523809524	0.09523809524	0.05555555556	0.05555555556	1.473684211	1.714285714
**COQ5**	0	0	0.1111111111	0.1111111111	0	0
**COQ6**	0.09523809524	0.09523809524	0	0	inf	9.52381E+31
**COQ7**	0.04761904762	0.04761904762	0	0	inf	4.7619E+31
**COQ8A**	0.1428571429	0.1428571429	0	0	inf	1.42857E+32
**COQ8B**	0.04761904762	0.04761904762	0.05555555556	0.05555555556	0.7	0.8571428571
**COQ9**	0.04761904762	0.04761904762	0	0	inf	4.7619E+31
**CORIN**	0.04761904762	0.04761904762	0.1111111111	0.1111111111	0.325	0.4285714286
**CORO1A**	0.04761904762	0.04761904762	0	0	inf	4.7619E+31
**CORO1C**	0.04761904762	0.04761904762	0.05555555556	0.05555555556	0.7	0.8571428571
**CORO2A**	0.04761904762	0.04761904762	0	0	inf	4.7619E+31
**CORO2B**	0.04761904762	0.04761904762	0.05555555556	0.05555555556	0.7	0.8571428571
**CORO6**	0	0	0.05555555556	0.05555555556	0	0
**CORO7**	0.04761904762	0.04761904762	0	0	inf	4.7619E+31
**RO7-PA**	0.04761904762	0.04761904762	0	0	inf	4.7619E+31
**CORT**	0.04761904762	0.04761904762	0.1111111111	0.1111111111	0.325	0.4285714286
**COTL1**	0.04761904762	0.04761904762	0	0	inf	4.7619E+31
**COX10**	0.04761904762	0.04761904762	0.05555555556	0.05555555556	0.7	0.8571428571
**OX10-AS**	0.04761904762	0.04761904762	0.05555555556	0.05555555556	0.7	0.8571428571
**COX11**	0.04761904762	0.04761904762	0	0	inf	4.7619E+31
**COX14**	0.04761904762	0.04761904762	0.05555555556	0.05555555556	0.7	0.8571428571
**COX15**	0.1428571429	0.1428571429	0	0	inf	1.42857E+32
**COX17**	0.09523809524	0.09523809524	0.05555555556	0.05555555556	1.473684211	1.714285714
**COX18**	0.1428571429	0.1428571429	0.05555555556	0.05555555556	2.333333333	2.571428571
**COX19**	0.04761904762	0.04761904762	0.1666666667	0.1666666667	0.2	0.2857142857
**COX20**	0.1428571429	0.1428571429	0.05555555556	0.05555555556	2.333333333	2.571428571
**COX4I1**	0.04761904762	0.04761904762	0	0	inf	4.7619E+31
**COX4I2**	0.09523809524	0.09523809524	0.1111111111	0.1111111111	0.6842105263	0.8571428571
**COX5A**	0.04761904762	0.04761904762	0.05555555556	0.05555555556	0.7	0.8571428571
**COX5B**	0	0	0.1111111111	0.1111111111	0	0
**COX6A1**	0	0	0.1111111111	0.1111111111	0	0
**COX6A2**	0.04761904762	0.04761904762	0	0	inf	4.7619E+31
**COX6B1**	0.04761904762	0.04761904762	0.05555555556	0.05555555556	0.7	0.8571428571
**COX6B2**	0.09523809524	0.09523809524	0.05555555556	0.05555555556	1.473684211	1.714285714
**COX6C**	0.1904761905	0.1904761905	0.1111111111	0.1111111111	1.529411765	1.714285714

COX7A1	0.04761904762	0.04761904762	0.05555555556	0.05555555556	0.7	0.8571428571
COX7A2	0	0	0.05555555556	0.05555555556	0	0
COX7B2	0.04761904762	0.04761904762	0	0	inf	4.7619E+31
COX7C	0.04761904762	0.04761904762	0	0	inf	4.7619E+31
CP	0.1428571429	0.1428571429	0.1111111111	0.1111111111	1.083333333	1.285714286
CPA1	0.04761904762	0.04761904762	0.05555555556	0.05555555556	0.7	0.8571428571
CPA2	0.04761904762	0.04761904762	0.05555555556	0.05555555556	0.7	0.8571428571
CPA3	0.1428571429	0.1428571429	0.1111111111	0.1111111111	1.083333333	1.285714286
CPA4	0.04761904762	0.04761904762	0.05555555556	0.05555555556	0.7	0.8571428571
CPA5	0.04761904762	0.04761904762	0.05555555556	0.05555555556	0.7	0.8571428571
CPA6	0.1428571429	0.1428571429	0.1666666667	0.1666666667	0.6666666667	0.8571428571
CPAMD8	0.1428571429	0.1428571429	0.1111111111	0.1111111111	1.083333333	1.285714286
CPB1	0.1428571429	0.1428571429	0.1111111111	0.1111111111	1.083333333	1.285714286
CPD	0	0	0.05555555556	0.05555555556	0	0
CPE	0	0	0.1111111111	0.1111111111	0	0
CPEB1	0.04761904762	0.04761904762	0.05555555556	0.05555555556	0.7	0.8571428571
CPEB1-AS	0.04761904762	0.04761904762	0.05555555556	0.05555555556	0.7	0.8571428571
CPEB2	0	0	0	0		0.00001
CPEB2-D	0	0	0	0		0.00001
CPEB3	0.1428571429	0.1428571429	0.1111111111	0.1111111111	1.083333333	1.285714286
CPEB4	0	0	0.05555555556	0.05555555556	0	0
CPED1	0.04761904762	0.04761904762	0.05555555556	0.05555555556	0.7	0.8571428571
CPHXL	0.04761904762	0.04761904762	0	0	inf	4.7619E+31
CPLANE	0.09523809524	0.09523809524	0.05555555556	0.05555555556	1.473684211	1.714285714
CPLANE	0.04761904762	0.04761904762	0.1111111111	0.1111111111	0.325	0.4285714286
CPLX1	0.04761904762	0.04761904762	0	0	inf	4.7619E+31
CPLX2	0.04761904762	0.04761904762	0.05555555556	0.05555555556	0.7	0.8571428571
CPLX3	0.04761904762	0.04761904762	0.05555555556	0.05555555556	0.7	0.8571428571
CPLX4	0.09523809524	0.09523809524	0	0	inf	9.52381E+31
CPM	0.09523809524	0.09523809524	0.1111111111	0.1111111111	0.6842105263	0.8571428571
CPN1	0.1428571429	0.1428571429	0	0	inf	1.42857E+32
CPN2	0.09523809524	0.09523809524	0.05555555556	0.05555555556	1.473684211	1.714285714
CPNE1	0.09523809524	0.09523809524	0.1111111111	0.1111111111	0.6842105263	0.8571428571
CPNE2	0.04761904762	0.04761904762	0	0	inf	4.7619E+31
CPNE3	0.1904761905	0.1904761905	0.1111111111	0.1111111111	1.529411765	1.714285714
CPNE4	0.1428571429	0.1428571429	0.05555555556	0.05555555556	2.333333333	2.571428571
CPNE5	0.04761904762	0.04761904762	0.05555555556	0.05555555556	0.7	0.8571428571
CPNE7	0.09523809524	0.09523809524	0	0	inf	9.52381E+31
CPNE8	0.04761904762	0.04761904762	0	0	inf	4.7619E+31
CPNE9	0.09523809524	0.09523809524	0	0	inf	9.52381E+31
CPO	0	0	0.05555555556	0.05555555556	0	0
CPOX	0.04761904762	0.04761904762	0.05555555556	0.05555555556	0.7	0.8571428571
CPPED1	0.04761904762	0.04761904762	0	0	inf	4.7619E+31
CPQ	0.1904761905	0.1904761905	0.1666666667	0.1666666667	0.9411764706	1.142857143
CPS1	0	0	0.05555555556	0.05555555556	0	0
CPS1-IT1	0	0	0.05555555556	0.05555555556	0	0
CPSF1	0.04761904762	0.04761904762	0.1666666667	0.1666666667	0.2	0.2857142857
CPSF3	0	0	0.05555555556	0.05555555556	0	0
CPSF4	0.04761904762	0.04761904762	0.05555555556	0.05555555556	0.7	0.8571428571
CPSF4L	0.04761904762	0.04761904762	0	0	inf	4.7619E+31
CPSF6	0.09523809524	0.09523809524	0.1111111111	0.1111111111	0.6842105263	0.8571428571

**CPT1B**	0	0	0.1111111111	0.1111111111	0	0
**CPT1C**	0.09523809524	0.09523809524	0.1111111111	0.1111111111	0.6842105263	0.8571428571
**CPT2**	0.04761904762	0.04761904762	0.05555555556	0.05555555556	0.7	0.8571428571
**CPTP**	0	0	0.1111111111	0.1111111111	0	0
**CPVL**	0.04761904762	0.04761904762	0.2222222222	0.2222222222	0.1375	0.2142857143
**CPXM1**	0.1904761905	0.1904761905	0.05555555556	0.05555555556	3.294117647	3.428571429
**CPXM2**	0.2380952381	0.2380952381	0.05555555556	0.05555555556	4.375	4.285714286
**CR1**	0.1428571429	0.1428571429	0	0	inf	1.42857E+32
**CR1L**	0.1428571429	0.1428571429	0	0	inf	1.42857E+32
**CR2**	0.1428571429	0.1428571429	0	0	inf	1.42857E+32
**CRABP1**	0.04761904762	0.04761904762	0.05555555556	0.05555555556	0.7	0.8571428571
**CRABP2**	0.1428571429	0.1428571429	0.1111111111	0.1111111111	1.083333333	1.285714286
**RACR2**	0.04761904762	0.04761904762	0.1111111111	0.1111111111	0.325	0.4285714286
**RACR2**	0.04761904762	0.04761904762	0	0	inf	4.7619E+31
**CRADD**	0.04761904762	0.04761904762	0.05555555556	0.05555555556	0.7	0.8571428571
**CRAMP1**	0.09523809524	0.09523809524	0.2222222222	0.2222222222	0.2894736842	0.4285714286
**CRAT37**	0.04761904762	0.04761904762	0.05555555556	0.05555555556	0.7	0.8571428571
**CRB1**	0.1428571429	0.1428571429	0	0	inf	1.42857E+32
**CRB2**	0.09523809524	0.09523809524	0.05555555556	0.05555555556	1.473684211	1.714285714
**CRB3**	0.1428571429	0.1428571429	0.05555555556	0.05555555556	2.333333333	2.571428571
**CRBN**	0.04761904762	0.04761904762	0	0	inf	4.7619E+31
**CRCP**	0.04761904762	0.04761904762	0.1111111111	0.1111111111	0.325	0.4285714286
**CRCT1**	0.1904761905	0.1904761905	0	0	inf	1.90476E+32
**CREB1**	0	0	0.05555555556	0.05555555556	0	0
**CREB3**	0.09523809524	0.09523809524	0.05555555556	0.05555555556	1.473684211	1.714285714
**CREB3L**	0	0	0.05555555556	0.05555555556	0	0
**CREB3L**	0.04761904762	0.04761904762	0.05555555556	0.05555555556	0.7	0.8571428571
**CREB3L**	0.09523809524	0.09523809524	0.05555555556	0.05555555556	1.473684211	1.714285714
**CREB3L**	0.1904761905	0.1904761905	0.05555555556	0.05555555556	3.294117647	3.428571429
**CREB5**	0.04761904762	0.04761904762	0.2222222222	0.2222222222	0.1375	0.2142857143
**CREBBP**	0.04761904762	0.04761904762	0.1111111111	0.1111111111	0.325	0.4285714286
**CREBL2**	0.04761904762	0.04761904762	0.05555555556	0.05555555556	0.7	0.8571428571
**CREBRF**	0	0	0.05555555556	0.05555555556	0	0
**CREG1**	0.1428571429	0.1428571429	0	0	inf	1.42857E+32
**CREG2**	0	0	0.05555555556	0.05555555556	0	0
**CRELD1**	0.09523809524	0.09523809524	0	0	inf	9.52381E+31
**CRELD2**	0	0	0.1111111111	0.1111111111	0	0
**CREM**	0.09523809524	0.09523809524	0.1666666667	0.1666666667	0.4210526316	0.5714285714
**CRH**	0.1428571429	0.1428571429	0.1666666667	0.1666666667	0.6666666667	0.8571428571
**CRHBP**	0.04761904762	0.04761904762	0	0	inf	4.7619E+31
**CRHR1**	0.1428571429	0.1428571429	0	0	inf	1.42857E+32
**CRHR2**	0.04761904762	0.04761904762	0.1111111111	0.1111111111	0.325	0.4285714286
**CRIM1**	0	0	0.05555555556	0.05555555556	0	0
**RIM1-D**	0	0	0.05555555556	0.05555555556	0	0
**CRIP3**	0.04761904762	0.04761904762	0.05555555556	0.05555555556	0.7	0.8571428571
**CRIPAK**	0.04761904762	0.04761904762	0	0	inf	4.7619E+31
**CRIPT**	0	0	0.05555555556	0.05555555556	0	0
**CRISP1**	0.04761904762	0.04761904762	0	0	inf	4.7619E+31
**CRISP2**	0.04761904762	0.04761904762	0	0	inf	4.7619E+31
**CRISP3**	0.04761904762	0.04761904762	0	0	inf	4.7619E+31
**RISPLD**	0.1904761905	0.1904761905	0.1111111111	0.1111111111	1.529411765	1.714285714

RISPLD	0.04761904762	0.04761904762	0	0	inf	4.7619E+31
CRK	0	0	0.1111111111	0.1111111111	0	0
CRKL	0.04761904762	0.04761904762	0.05555555556	0.05555555556	0.7	0.8571428571
CRLF1	0.1428571429	0.1428571429	0.1111111111	0.1111111111	1.083333333	1.285714286
CRLF3	0	0	0.05555555556	0.05555555556	0	0
CRLS1	0.1904761905	0.1904761905	0.05555555556	0.05555555556	3.294117647	3.428571429
CRNDE	0.04761904762	0.04761904762	0	0	inf	4.7619E+31
CRNKL1	0.1904761905	0.1904761905	0.05555555556	0.05555555556	3.294117647	3.428571429
CRNN	0.1904761905	0.1904761905	0	0	inf	1.90476E+32
CROCC	0.04761904762	0.04761904762	0.1111111111	0.1111111111	0.325	0.4285714286
CROCC2	0	0	0.05555555556	0.05555555556	0	0
ROCCP	0.04761904762	0.04761904762	0.1111111111	0.1111111111	0.325	0.4285714286
ROCCP	0.04761904762	0.04761904762	0.1111111111	0.1111111111	0.325	0.4285714286
CROT	0.09523809524	0.09523809524	0.05555555556	0.05555555556	1.473684211	1.714285714
CRP	0.1428571429	0.1428571429	0	0	inf	1.42857E+32
CRPPA	0.04761904762	0.04761904762	0.2222222222	0.2222222222	0.1375	0.2142857143
RPPA-AS	0.04761904762	0.04761904762	0.2222222222	0.2222222222	0.1375	0.2142857143
CRSP8P	0.04761904762	0.04761904762	0	0	inf	4.7619E+31
CRTAC1	0.1428571429	0.1428571429	0	0	inf	1.42857E+32
CRTAP	0.09523809524	0.09523809524	0	0	inf	9.52381E+31
CRTC1	0.1428571429	0.1428571429	0.1111111111	0.1111111111	1.083333333	1.285714286
CRTC2	0.1904761905	0.1904761905	0.05555555556	0.05555555556	3.294117647	3.428571429
CRTC3	0.04761904762	0.04761904762	0.05555555556	0.05555555556	0.7	0.8571428571
RTC3-AS	0.04761904762	0.04761904762	0.05555555556	0.05555555556	0.7	0.8571428571
CRX	0.09523809524	0.09523809524	0.1111111111	0.1111111111	0.6842105263	0.8571428571
CRY1	0.04761904762	0.04761904762	0.05555555556	0.05555555556	0.7	0.8571428571
CRYAA	0.04761904762	0.04761904762	0.1666666667	0.1666666667	0.2	0.2857142857
CRYAA2	0.04761904762	0.04761904762	0.1666666667	0.1666666667	0.2	0.2857142857
CRYBA1	0.04761904762	0.04761904762	0.05555555556	0.05555555556	0.7	0.8571428571
CRYBA2	0	0	0.05555555556	0.05555555556	0	0
CRYBA4	0.04761904762	0.04761904762	0.05555555556	0.05555555556	0.7	0.8571428571
CRYBB1	0.04761904762	0.04761904762	0.05555555556	0.05555555556	0.7	0.8571428571
CRYBB2	0.04761904762	0.04761904762	0.05555555556	0.05555555556	0.7	0.8571428571
RYBB2P	0.04761904762	0.04761904762	0.05555555556	0.05555555556	0.7	0.8571428571
CRYBB3	0.04761904762	0.04761904762	0.05555555556	0.05555555556	0.7	0.8571428571
CRYBG2	0.04761904762	0.04761904762	0.1111111111	0.1111111111	0.325	0.4285714286
CRYBG3	0.04761904762	0.04761904762	0.05555555556	0.05555555556	0.7	0.8571428571
CRYGA	0	0	0.05555555556	0.05555555556	0	0
CRYGB	0	0	0.05555555556	0.05555555556	0	0
CRYGC	0	0	0.05555555556	0.05555555556	0	0
CRYGD	0	0	0.05555555556	0.05555555556	0	0
CRYGN	0.09523809524	0.09523809524	0.05555555556	0.05555555556	1.473684211	1.714285714
CRYGS	0.09523809524	0.09523809524	0.05555555556	0.05555555556	1.473684211	1.714285714
CRYL1	0.04761904762	0.04761904762	0	0	inf	4.7619E+31
CRYM	0.04761904762	0.04761904762	0	0	inf	4.7619E+31
RYM-AS	0.04761904762	0.04761904762	0	0	inf	4.7619E+31
CRYZ	0.04761904762	0.04761904762	0.05555555556	0.05555555556	0.7	0.8571428571
CRYZL1	0.04761904762	0.04761904762	0.1111111111	0.1111111111	0.325	0.4285714286
CRYZL2	0.1428571429	0.1428571429	0	0	inf	1.42857E+32
ZL2P-SE	0.1428571429	0.1428571429	0	0	inf	1.42857E+32
CS	0.1428571429	0.1428571429	0.05555555556	0.05555555556	2.333333333	2.571428571

**CSAD**	0.04761904762	0.04761904762	0.05555555556	0.05555555556	0.7	0.8571428571
**CSDC2**	0	0	0.1111111111	0.1111111111	0	0
**CSDE1**	0.04761904762	0.04761904762	0.05555555556	0.05555555556	0.7	0.8571428571
**CSE1L**	0.09523809524	0.09523809524	0.1666666667	0.1666666667	0.4210526316	0.5714285714
**SE1L-AS**	0.09523809524	0.09523809524	0.1666666667	0.1666666667	0.4210526316	0.5714285714
**CSF1**	0.04761904762	0.04761904762	0.05555555556	0.05555555556	0.7	0.8571428571
**CSF2**	0.04761904762	0.04761904762	0	0	inf	4.7619E+31
**CSF2RB**	0	0	0.1111111111	0.1111111111	0	0
**CSF3**	0.09523809524	0.09523809524	0	0	inf	9.52381E+31
**CSF3R**	0.04761904762	0.04761904762	0	0	inf	4.7619E+31
**GALNAC**	0.09523809524	0.09523809524	0.1111111111	0.1111111111	0.6842105263	0.8571428571
**GALNAC**	0.1428571429	0.1428571429	0.05555555556	0.05555555556	2.333333333	2.571428571
**CSH1**	0.04761904762	0.04761904762	0	0	inf	4.7619E+31
**CSH2**	0.04761904762	0.04761904762	0	0	inf	4.7619E+31
**CSHL1**	0.04761904762	0.04761904762	0	0	inf	4.7619E+31
**CSK**	0.04761904762	0.04761904762	0.05555555556	0.05555555556	0.7	0.8571428571
**CSMD1**	0.1428571429	0.1428571429	0.1111111111	0.1111111111	1.083333333	1.285714286
**CSMD2**	0.04761904762	0.04761904762	0	0	inf	4.7619E+31
**SMD2-A**	0.04761904762	0.04761904762	0	0	inf	4.7619E+31
**CSMD3**	0.1904761905	0.1904761905	0.1111111111	0.1111111111	1.529411765	1.714285714
**CSN1S1**	0.04761904762	0.04761904762	0.05555555556	0.05555555556	0.7	0.8571428571
**SN1S2A**	0.04761904762	0.04761904762	0.05555555556	0.05555555556	0.7	0.8571428571
**SN1S2B**	0.04761904762	0.04761904762	0.05555555556	0.05555555556	0.7	0.8571428571
**CSN2**	0.04761904762	0.04761904762	0.05555555556	0.05555555556	0.7	0.8571428571
**CSN3**	0.04761904762	0.04761904762	0.05555555556	0.05555555556	0.7	0.8571428571
**SNK1A1**	0	0	0	0		0.00001
**CSNK1D**	0.04761904762	0.04761904762	0	0	inf	4.7619E+31
**CSNK1E**	0.04761904762	0.04761904762	0.1111111111	0.1111111111	0.325	0.4285714286
**CSNK1G**	0.04761904762	0.04761904762	0.05555555556	0.05555555556	0.7	0.8571428571
**CSNK1G**	0.09523809524	0.09523809524	0.1111111111	0.1111111111	0.6842105263	0.8571428571
**NK1G2-A**	0.09523809524	0.09523809524	0.1111111111	0.1111111111	0.6842105263	0.8571428571
**CSNK1G**	0.04761904762	0.04761904762	0	0	inf	4.7619E+31
**CSNK2A**	0.1904761905	0.1904761905	0.05555555556	0.05555555556	3.294117647	3.428571429
**CSNK2A**	0.04761904762	0.04761904762	0	0	inf	4.7619E+31
**CSNK2B**	0.04761904762	0.04761904762	0	0	inf	4.7619E+31
**SNKA2I**	0.04761904762	0.04761904762	0	0	inf	4.7619E+31
**CSPG4**	0.04761904762	0.04761904762	0.05555555556	0.05555555556	0.7	0.8571428571
**CSPG5**	0.09523809524	0.09523809524	0	0	inf	9.52381E+31
**CSPP1**	0.1428571429	0.1428571429	0.1666666667	0.1666666667	0.6666666667	0.8571428571
**CSRNP1**	0.04761904762	0.04761904762	0	0	inf	4.7619E+31
**CSRNP2**	0.09523809524	0.09523809524	0.05555555556	0.05555555556	1.473684211	1.714285714
**CSRNP3**	0	0	0.05555555556	0.05555555556	0	0
**CSRP1**	0.1428571429	0.1428571429	0	0	inf	1.42857E+32
**CSRP2**	0.04761904762	0.04761904762	0.1111111111	0.1111111111	0.325	0.4285714286
**CST1**	0.1904761905	0.1904761905	0.05555555556	0.05555555556	3.294117647	3.428571429
**CST11**	0.1904761905	0.1904761905	0.05555555556	0.05555555556	3.294117647	3.428571429
**CST13P**	0.1904761905	0.1904761905	0.05555555556	0.05555555556	3.294117647	3.428571429
**CST2**	0.1904761905	0.1904761905	0.05555555556	0.05555555556	3.294117647	3.428571429
**CST3**	0.1904761905	0.1904761905	0.05555555556	0.05555555556	3.294117647	3.428571429
**CST4**	0.1904761905	0.1904761905	0.05555555556	0.05555555556	3.294117647	3.428571429
**CST5**	0.1904761905	0.1904761905	0.05555555556	0.05555555556	3.294117647	3.428571429

CST7	0.1904761905	0.1904761905	0.05555555556	0.05555555556	3.294117647	3.428571429
CST8	0.1904761905	0.1904761905	0.05555555556	0.05555555556	3.294117647	3.428571429
CST9	0.1904761905	0.1904761905	0.05555555556	0.05555555556	3.294117647	3.428571429
CST9L	0.1904761905	0.1904761905	0.05555555556	0.05555555556	3.294117647	3.428571429
CSTA	0.09523809524	0.09523809524	0.05555555556	0.05555555556	1.473684211	1.714285714
CSTB	0.04761904762	0.04761904762	0.1111111111	0.1111111111	0.325	0.4285714286
CSTF1	0.09523809524	0.09523809524	0.1666666667	0.1666666667	0.4210526316	0.5714285714
CSTF2T	0.04761904762	0.04761904762	0.05555555556	0.05555555556	0.7	0.8571428571
CSTF3	0.04761904762	0.04761904762	0	0	inf	4.7619E+31
STF3-D	0.04761904762	0.04761904762	0	0	inf	4.7619E+31
CSTL1	0.1904761905	0.1904761905	0.05555555556	0.05555555556	3.294117647	3.428571429
CT62	0.04761904762	0.04761904762	0.05555555556	0.05555555556	0.7	0.8571428571
CT66	0.04761904762	0.04761904762	0.1111111111	0.1111111111	0.325	0.4285714286
CT75	0	0	0.05555555556	0.05555555556	0	0
CTAGE1	0.04761904762	0.04761904762	0.05555555556	0.05555555556	0.7	0.8571428571
TAGE1	0.04761904762	0.04761904762	0	0	inf	4.7619E+31
CTAGE4	0.09523809524	0.09523809524	0.05555555556	0.05555555556	1.473684211	1.714285714
CTAGE6	0.04761904762	0.04761904762	0	0	inf	4.7619E+31
CTAGE8	0.09523809524	0.09523809524	0.05555555556	0.05555555556	1.473684211	1.714285714
B-178M2	0	0	0.05555555556	0.05555555556	0	0
CTBP1	0.04761904762	0.04761904762	0	0	inf	4.7619E+31
TBP1-A	0.04761904762	0.04761904762	0	0	inf	4.7619E+31
TBP1-D	0.04761904762	0.04761904762	0	0	inf	4.7619E+31
CTBP2	0.2380952381	0.2380952381	0.05555555556	0.05555555556	4.375	4.285714286
CTBS	0.04761904762	0.04761904762	0.05555555556	0.05555555556	0.7	0.8571428571
C-338M1	0.09523809524	0.09523809524	0.05555555556	0.05555555556	1.473684211	1.714285714
CTC1	0.04761904762	0.04761904762	0.05555555556	0.05555555556	0.7	0.8571428571
CTCF	0.1428571429	0.1428571429	0.05555555556	0.05555555556	2.333333333	2.571428571
CTCFL	0.1428571429	0.1428571429	0.2222222222	0.2222222222	0.4583333333	0.6428571429
D-2194D	0	0	0.1111111111	0.1111111111	0	0
D-2201I1	0.04761904762	0.04761904762	0	0	inf	4.7619E+31
D-2270F1	0	0	0.05555555556	0.05555555556	0	0
D-2297D	0	0	0.1111111111	0.1111111111	0	0
D-2350J1	0.04761904762	0.04761904762	0.05555555556	0.05555555556	0.7	0.8571428571
D-3080P1	0	0	0.05555555556	0.05555555556	0	0
TDNEP	0	0	0.05555555556	0.05555555556	0	0
CTDP1	0.04761904762	0.04761904762	0.05555555556	0.05555555556	0.7	0.8571428571
CTDSP1	0	0	0.05555555556	0.05555555556	0	0
CTDSP2	0.1428571429	0.1428571429	0.05555555556	0.05555555556	2.333333333	2.571428571
CTDSPL	0.04761904762	0.04761904762	0	0	inf	4.7619E+31
CTF1	0.04761904762	0.04761904762	0	0	inf	4.7619E+31
CTH	0.04761904762	0.04761904762	0.05555555556	0.05555555556	0.7	0.8571428571
CTHRC1	0.1904761905	0.1904761905	0.1111111111	0.1111111111	1.529411765	1.714285714
CTIF	0.04761904762	0.04761904762	0.05555555556	0.05555555556	0.7	0.8571428571
CTLA4	0	0	0.05555555556	0.05555555556	0	0
CTNNA1	0.04761904762	0.04761904762	0	0	inf	4.7619E+31
CTNNA2	0	0	0.05555555556	0.05555555556	0	0
CTNNA3	0.1428571429	0.1428571429	0.05555555556	0.05555555556	2.333333333	2.571428571
TNNAL	0.04761904762	0.04761904762	0.05555555556	0.05555555556	0.7	0.8571428571
CTNNB1	0.04761904762	0.04761904762	0	0	inf	4.7619E+31
TNNBIP	0	0	0.1111111111	0.1111111111	0	0

TNNBL	0.1428571429	0.1428571429	0.1666666667	0.1666666667	0.6666666667	0.8571428571
TNND2	0.04761904762	0.04761904762	0.05555555556	0.05555555556	0.7	0.8571428571
CTNS	0	0	0.1111111111	0.1111111111	0	0
CTPS1	0.04761904762	0.04761904762	0.05555555556	0.05555555556	0.7	0.8571428571
CTRB1	0.04761904762	0.04761904762	0	0	inf	4.7619E+31
CTRB2	0.04761904762	0.04761904762	0	0	inf	4.7619E+31
CTRC	0.04761904762	0.04761904762	0.1111111111	0.1111111111	0.325	0.4285714286
CTRL	0.1428571429	0.1428571429	0.05555555556	0.05555555556	2.333333333	2.571428571
CTSA	0.1428571429	0.1428571429	0.1666666667	0.1666666667	0.6666666667	0.8571428571
CTSB	0.09523809524	0.09523809524	0.1111111111	0.1111111111	0.6842105263	0.8571428571
CTSD	0.04761904762	0.04761904762	0	0	inf	4.7619E+31
CTSE	0.1428571429	0.1428571429	0	0	inf	1.42857E+32
CTSH	0.04761904762	0.04761904762	0.05555555556	0.05555555556	0.7	0.8571428571
CTSK	0.1904761905	0.1904761905	0.05555555556	0.05555555556	3.294117647	3.428571429
CTSL	0	0	0.05555555556	0.05555555556	0	0
CTSL3P	0	0	0.05555555556	0.05555555556	0	0
CTSLP2	0.1428571429	0.1428571429	0.05555555556	0.05555555556	2.333333333	2.571428571
CTSLP8	0	0	0.05555555556	0.05555555556	0	0
CTSO	0	0	0.1111111111	0.1111111111	0	0
CTSS	0.1904761905	0.1904761905	0.05555555556	0.05555555556	3.294117647	3.428571429
CTSV	0.04761904762	0.04761904762	0	0	inf	4.7619E+31
CTSZ	0.1428571429	0.1428571429	0.2222222222	0.2222222222	0.4583333333	0.6428571429
TTNBP	0.09523809524	0.09523809524	0.05555555556	0.05555555556	1.473684211	1.714285714
TNBP2	0.04761904762	0.04761904762	0.05555555556	0.05555555556	0.7	0.8571428571
CTU1	0.09523809524	0.09523809524	0.05555555556	0.05555555556	1.473684211	1.714285714
CTU2	0.1904761905	0.1904761905	0	0	inf	1.90476E+32
CTXN1	0.1428571429	0.1428571429	0.05555555556	0.05555555556	2.333333333	2.571428571
CTXN2	0.04761904762	0.04761904762	0.05555555556	0.05555555556	0.7	0.8571428571
CTXN3	0.04761904762	0.04761904762	0	0	inf	4.7619E+31
TXND1	0.04761904762	0.04761904762	0.05555555556	0.05555555556	0.7	0.8571428571
TXND2	0.1904761905	0.1904761905	0.05555555556	0.05555555556	3.294117647	3.428571429
CUBN	0.09523809524	0.09523809524	0.1111111111	0.1111111111	0.6842105263	0.8571428571
UEDC1	0.09523809524	0.09523809524	0	0	inf	9.52381E+31
UEDC2	0.1428571429	0.1428571429	0.05555555556	0.05555555556	2.333333333	2.571428571
CUL1	0.09523809524	0.09523809524	0.05555555556	0.05555555556	1.473684211	1.714285714
CUL2	0.09523809524	0.09523809524	0.1666666667	0.1666666667	0.4210526316	0.5714285714
CUL3	0	0	0.05555555556	0.05555555556	0	0
CUL7	0.04761904762	0.04761904762	0.05555555556	0.05555555556	0.7	0.8571428571
CUL9	0.04761904762	0.04761904762	0.05555555556	0.05555555556	0.7	0.8571428571
CUTA	0.04761904762	0.04761904762	0	0	inf	4.7619E+31
UTALI	0.09523809524	0.09523809524	0.05555555556	0.05555555556	1.473684211	1.714285714
CUTC	0.1428571429	0.1428571429	0	0	inf	1.42857E+32
CUX1	0.04761904762	0.04761904762	0.05555555556	0.05555555556	0.7	0.8571428571
CUX2	0	0	0.1111111111	0.1111111111	0	0
CUZD1	0.2380952381	0.2380952381	0.05555555556	0.05555555556	4.375	4.285714286
CWC22	0	0	0.05555555556	0.05555555556	0	0
CWC25	0.09523809524	0.09523809524	0	0	inf	9.52381E+31
CWC27	0.04761904762	0.04761904762	0	0	inf	4.7619E+31
WF19L	0.1428571429	0.1428571429	0	0	inf	1.42857E+32
CWH43	0.09523809524	0.09523809524	0.1111111111	0.1111111111	0.6842105263	0.8571428571
X3CL1	0.04761904762	0.04761904762	0	0	inf	4.7619E+31

CX3CR1	0.04761904762	0.04761904762	0	0	inf	4.7619E+31
CXADR	0.04761904762	0.04761904762	0.1111111111	0.1111111111	0.325	0.4285714286
XADRP	0.2857142857	0.2857142857	0.1111111111	0.1111111111	2.6	2.571428571
XADRP	0.09523809524	0.09523809524	0.05555555556	0.05555555556	1.473684211	1.714285714
CXCL1	0.09523809524	0.09523809524	0.05555555556	0.05555555556	1.473684211	1.714285714
CXCL10	0.04761904762	0.04761904762	0.05555555556	0.05555555556	0.7	0.8571428571
CXCL11	0.04761904762	0.04761904762	0.05555555556	0.05555555556	0.7	0.8571428571
CXCL12	0.1428571429	0.1428571429	0.05555555556	0.05555555556	2.333333333	2.571428571
CXCL13	0.04761904762	0.04761904762	0.05555555556	0.05555555556	0.7	0.8571428571
CXCL14	0.04761904762	0.04761904762	0	0	inf	4.7619E+31
CXCL16	0	0	0.1111111111	0.1111111111	0	0
CXCL17	0.04761904762	0.04761904762	0.05555555556	0.05555555556	0.7	0.8571428571
CXCL2	0.09523809524	0.09523809524	0.05555555556	0.05555555556	1.473684211	1.714285714
CXCL3	0.09523809524	0.09523809524	0.05555555556	0.05555555556	1.473684211	1.714285714
CXCL5	0.09523809524	0.09523809524	0.05555555556	0.05555555556	1.473684211	1.714285714
CXCL6	0.09523809524	0.09523809524	0.05555555556	0.05555555556	1.473684211	1.714285714
CXCL8	0.09523809524	0.09523809524	0.05555555556	0.05555555556	1.473684211	1.714285714
CXCL9	0.04761904762	0.04761904762	0.05555555556	0.05555555556	0.7	0.8571428571
CXCR1	0	0	0.05555555556	0.05555555556	0	0
CXCR2	0	0	0.05555555556	0.05555555556	0	0
XCR2P	0	0	0.05555555556	0.05555555556	0	0
CXCR4	0	0	0.05555555556	0.05555555556	0	0
CXXC1	0.04761904762	0.04761904762	0	0	inf	4.7619E+31
CXXC4	0	0	0.05555555556	0.05555555556	0	0
XXC4-A	0	0	0.05555555556	0.05555555556	0	0
CXXC5	0.04761904762	0.04761904762	0	0	inf	4.7619E+31
XXC5-A	0.04761904762	0.04761904762	0	0	inf	4.7619E+31
CYB561	0.04761904762	0.04761904762	0	0	inf	4.7619E+31
YB561D	0.04761904762	0.04761904762	0.05555555556	0.05555555556	0.7	0.8571428571
YB561D	0.09523809524	0.09523809524	0	0	inf	9.52381E+31
CYB5B	0.1428571429	0.1428571429	0.05555555556	0.05555555556	2.333333333	2.571428571
CYB5D1	0	0	0.05555555556	0.05555555556	0	0
CYB5D2	0	0	0.1111111111	0.1111111111	0	0
CYB5R1	0.1428571429	0.1428571429	0	0	inf	1.42857E+32
CYB5R2	0.04761904762	0.04761904762	0	0	inf	4.7619E+31
CYB5RL	0.04761904762	0.04761904762	0.05555555556	0.05555555556	0.7	0.8571428571
CYBA	0.1428571429	0.1428571429	0	0	inf	1.42857E+32
CYBC1	0.04761904762	0.04761904762	0	0	inf	4.7619E+31
CYBRD1	0	0	0.05555555556	0.05555555556	0	0
CYC1	0.04761904762	0.04761904762	0.1666666667	0.1666666667	0.2	0.2857142857
CYCS	0.04761904762	0.04761904762	0.2222222222	0.2222222222	0.1375	0.2142857143
YCSP5	0.1428571429	0.1428571429	0.05555555556	0.05555555556	2.333333333	2.571428571
CYFIP1	0.09523809524	0.09523809524	0	0	inf	9.52381E+31
CYFIP2	0.04761904762	0.04761904762	0.05555555556	0.05555555556	0.7	0.8571428571
CYGB	0.04761904762	0.04761904762	0	0	inf	4.7619E+31
CYHR1	0.04761904762	0.04761904762	0.1666666667	0.1666666667	0.2	0.2857142857
CYLC2	0.04761904762	0.04761904762	0	0	inf	4.7619E+31
CYLD	0.04761904762	0.04761904762	0	0	inf	4.7619E+31
CYMP	0.04761904762	0.04761904762	0	0	inf	4.7619E+31
YMP-AS	0.04761904762	0.04761904762	0	0	inf	4.7619E+31
YP11A	0.04761904762	0.04761904762	0.05555555556	0.05555555556	0.7	0.8571428571

CYP11B1	0.04761904762	0.04761904762	0.1666666667	0.1666666667	0.2	0.2857142857
CYP11B2	0.04761904762	0.04761904762	0.1666666667	0.1666666667	0.2	0.2857142857
CYP17A1	0.1428571429	0.1428571429	0.05555555556	0.05555555556	2.333333333	2.571428571
CYP19A1	0.04761904762	0.04761904762	0.05555555556	0.05555555556	0.7	0.8571428571
CYP1A1	0.04761904762	0.04761904762	0.05555555556	0.05555555556	0.7	0.8571428571
CYP1A2	0.04761904762	0.04761904762	0.05555555556	0.05555555556	0.7	0.8571428571
CYP1B1	0	0	0.05555555556	0.05555555556	0	0
P1B1-A	0	0	0.05555555556	0.05555555556	0	0
CYP20A1	0.09523809524	0.09523809524	0.05555555556	0.05555555556	1.473684211	1.714285714
YP21A1	0.04761904762	0.04761904762	0	0	inf	4.7619E+31
CYP21A2	0.04761904762	0.04761904762	0	0	inf	4.7619E+31
CYP24A1	0.09523809524	0.09523809524	0.1111111111	0.1111111111	0.6842105263	0.8571428571
CYP26A1	0.1428571429	0.1428571429	0.1111111111	0.1111111111	1.083333333	1.285714286
CYP26B1	0	0	0.05555555556	0.05555555556	0	0
CYP26C1	0.1428571429	0.1428571429	0.1111111111	0.1111111111	1.083333333	1.285714286
CYP27A1	0	0	0.05555555556	0.05555555556	0	0
CYP27B1	0.1428571429	0.1428571429	0.05555555556	0.05555555556	2.333333333	2.571428571
CYP27C1	0	0	0.05555555556	0.05555555556	0	0
CYP2A13	0.04761904762	0.04761904762	0.05555555556	0.05555555556	0.7	0.8571428571
CYP2A6	0.04761904762	0.04761904762	0.05555555556	0.05555555556	0.7	0.8571428571
CYP2A7	0.04761904762	0.04761904762	0.05555555556	0.05555555556	0.7	0.8571428571
CYP2B6	0.04761904762	0.04761904762	0.05555555556	0.05555555556	0.7	0.8571428571
CYP2B7P	0.04761904762	0.04761904762	0.05555555556	0.05555555556	0.7	0.8571428571
CYP2C18	0.1428571429	0.1428571429	0	0	inf	1.42857E+32
CYP2C19	0.1428571429	0.1428571429	0	0	inf	1.42857E+32
CYP2C8	0.1428571429	0.1428571429	0	0	inf	1.42857E+32
CYP2C9	0.1428571429	0.1428571429	0	0	inf	1.42857E+32
CYP2D6	0	0	0.1111111111	0.1111111111	0	0
CYP2D7	0	0	0.1111111111	0.1111111111	0	0
CYP2E1	0.1904761905	0.1904761905	0	0	inf	1.90476E+32
CYP2F1	0.04761904762	0.04761904762	0.05555555556	0.05555555556	0.7	0.8571428571
CYP2G1P	0.04761904762	0.04761904762	0.05555555556	0.05555555556	0.7	0.8571428571
CYP2J2	0.04761904762	0.04761904762	0.05555555556	0.05555555556	0.7	0.8571428571
CYP2S1	0.04761904762	0.04761904762	0.05555555556	0.05555555556	0.7	0.8571428571
CYP2U1	0	0	0.05555555556	0.05555555556	0	0
CYP2W1	0.04761904762	0.04761904762	0.1666666667	0.1666666667	0.2	0.2857142857
CYP39A1	0.04761904762	0.04761904762	0.05555555556	0.05555555556	0.7	0.8571428571
CYP3A4	0.04761904762	0.04761904762	0.05555555556	0.05555555556	0.7	0.8571428571
CYP3A43	0.04761904762	0.04761904762	0.05555555556	0.05555555556	0.7	0.8571428571
CYP3A5	0.04761904762	0.04761904762	0.05555555556	0.05555555556	0.7	0.8571428571
CYP3A7	0.04761904762	0.04761904762	0.05555555556	0.05555555556	0.7	0.8571428571
A7-CYP3	0.04761904762	0.04761904762	0.05555555556	0.05555555556	0.7	0.8571428571
CYP4A11	0.04761904762	0.04761904762	0.05555555556	0.05555555556	0.7	0.8571428571
CYP4A22	0.04761904762	0.04761904762	0.05555555556	0.05555555556	0.7	0.8571428571
CYP4B1	0.04761904762	0.04761904762	0.05555555556	0.05555555556	0.7	0.8571428571
CYP4F11	0.1428571429	0.1428571429	0.1111111111	0.1111111111	1.083333333	1.285714286
CYP4F12	0.1428571429	0.1428571429	0.1111111111	0.1111111111	1.083333333	1.285714286
CYP4F2	0.1428571429	0.1428571429	0.1111111111	0.1111111111	1.083333333	1.285714286
CYP4F22	0.1428571429	0.1428571429	0.1111111111	0.1111111111	1.083333333	1.285714286
YP4F24	0.1428571429	0.1428571429	0.1111111111	0.1111111111	1.083333333	1.285714286
YP4F29	0.04761904762	0.04761904762	0	0	inf	4.7619E+31

CYP4F3	0.1428571429	0.1428571429	0.1111111111	0.1111111111	1.083333333	1.285714286
YP4F30	0	0	0.05555555556	0.05555555556	0	0
YP4F35	0.09523809524	0.09523809524	0.05555555556	0.05555555556	1.473684211	1.714285714
YP4F62	0	0	0.05555555556	0.05555555556	0	0
CYP4F8	0.1428571429	0.1428571429	0.1111111111	0.1111111111	1.083333333	1.285714286
CYP4V2	0	0	0.05555555556	0.05555555556	0	0
CYP4X1	0.04761904762	0.04761904762	0.05555555556	0.05555555556	0.7	0.8571428571
CYP4Z1	0.04761904762	0.04761904762	0.05555555556	0.05555555556	0.7	0.8571428571
YP4Z2I	0.04761904762	0.04761904762	0.05555555556	0.05555555556	0.7	0.8571428571
YP51A1	0.04761904762	0.04761904762	0.1111111111	0.1111111111	0.325	0.4285714286
P51A1-A	0.04761904762	0.04761904762	0.1111111111	0.1111111111	0.325	0.4285714286
CYP7A1	0.1428571429	0.1428571429	0.1666666667	0.1666666667	0.6666666667	0.8571428571
CYP7B1	0.1428571429	0.1428571429	0.1666666667	0.1666666667	0.6666666667	0.8571428571
CYP8B1	0.04761904762	0.04761904762	0	0	inf	4.7619E+31
CYREN	0.04761904762	0.04761904762	0.05555555556	0.05555555556	0.7	0.8571428571
CYS1	0	0	0.05555555556	0.05555555556	0	0
CYSRT1	0.1428571429	0.1428571429	0.05555555556	0.05555555556	2.333333333	2.571428571
CYSTM1	0.04761904762	0.04761904762	0	0	inf	4.7619E+31
CYTH1	0.04761904762	0.04761904762	0	0	inf	4.7619E+31
CYTH2	0.09523809524	0.09523809524	0.1111111111	0.1111111111	0.6842105263	0.8571428571
CYTH3	0.04761904762	0.04761904762	0.1666666667	0.1666666667	0.2	0.2857142857
CYTH4	0	0	0.1111111111	0.1111111111	0	0
CYTIP	0	0	0.05555555556	0.05555555556	0	0
CYTOR	0	0	0.05555555556	0.05555555556	0	0
CYYR1	0.04761904762	0.04761904762	0.1111111111	0.1111111111	0.325	0.4285714286
YYR1-A	0.04761904762	0.04761904762	0.1111111111	0.1111111111	0.325	0.4285714286
Z1P-ASN	0.04761904762	0.04761904762	0.05555555556	0.05555555556	0.7	0.8571428571
CZIB	0.04761904762	0.04761904762	0.05555555556	0.05555555556	0.7	0.8571428571
21S2088	0.04761904762	0.04761904762	0.1111111111	0.1111111111	0.325	0.4285714286
D2HGDH	0	0	0.05555555556	0.05555555556	0	0
DAAM2	0.04761904762	0.04761904762	0.05555555556	0.05555555556	0.7	0.8571428571
DAB1	0.04761904762	0.04761904762	0.05555555556	0.05555555556	0.7	0.8571428571
AB1-AS	0.04761904762	0.04761904762	0.05555555556	0.05555555556	0.7	0.8571428571
DAB2	0.04761904762	0.04761904762	0.05555555556	0.05555555556	0.7	0.8571428571
DAB2IP	0.09523809524	0.09523809524	0.05555555556	0.05555555556	1.473684211	1.714285714
DACT3	0.09523809524	0.09523809524	0.1111111111	0.1111111111	0.6842105263	0.8571428571
ACT3-AS	0.09523809524	0.09523809524	0.1111111111	0.1111111111	0.6842105263	0.8571428571
DAD1	0	0	0.05555555556	0.05555555556	0	0
DAG1	0.1428571429	0.1428571429	0	0	inf	1.42857E+32
DALRD3	0.09523809524	0.09523809524	0	0	inf	9.52381E+31
DANCR	0.2380952381	0.2380952381	0.05555555556	0.05555555556	4.375	4.285714286
DAND5	0.1428571429	0.1428571429	0.1111111111	0.1111111111	1.083333333	1.285714286
DAO	0	0	0.05555555556	0.05555555556	0	0
DAP	0.04761904762	0.04761904762	0.05555555556	0.05555555556	0.7	0.8571428571
DAP3	0.1904761905	0.1904761905	0.1111111111	0.1111111111	1.529411765	1.714285714
DAPK1	0	0	0.05555555556	0.05555555556	0	0
APK1-IT	0	0	0.05555555556	0.05555555556	0	0
DAPK2	0.04761904762	0.04761904762	0.05555555556	0.05555555556	0.7	0.8571428571
DAPK3	0.09523809524	0.09523809524	0.05555555556	0.05555555556	1.473684211	1.714285714
DAPL1	0	0	0.05555555556	0.05555555556	0	0
DAPP1	0	0	0.05555555556	0.05555555556	0	0

**DARS**	0.04761904762	0.04761904762	0.05555555556	0.05555555556	0.7	0.8571428571
**ARS-AS**	0.04761904762	0.04761904762	0.05555555556	0.05555555556	0.7	0.8571428571
**DARS2**	0.1428571429	0.1428571429	0	0	inf	1.42857E+32
**DAW1**	0	0	0.05555555556	0.05555555556	0	0
**DAXX**	0.04761904762	0.04761904762	0	0	inf	4.7619E+31
**DAZAP1**	0.09523809524	0.09523809524	0.1111111111	0.1111111111	0.6842105263	0.8571428571
**DAZAP2**	0.09523809524	0.09523809524	0.05555555556	0.05555555556	1.473684211	1.714285714
**DAZL**	0.04761904762	0.04761904762	0	0	inf	4.7619E+31
**DBET**	0.04761904762	0.04761904762	0.05555555556	0.05555555556	0.7	0.8571428571
**DBF4**	0.09523809524	0.09523809524	0.05555555556	0.05555555556	1.473684211	1.714285714
**DBF4B**	0.09523809524	0.09523809524	0	0	inf	9.52381E+31
**DBH**	0.04761904762	0.04761904762	0	0	inf	4.7619E+31
**DBH-AS1**	0.04761904762	0.04761904762	0	0	inf	4.7619E+31
**DBI**	0	0	0.05555555556	0.05555555556	0	0
**DBIL5P2**	0	0	0.05555555556	0.05555555556	0	0
**DBN1**	0.09523809524	0.09523809524	0.05555555556	0.05555555556	1.473684211	1.714285714
**DBNDD1**	0.09523809524	0.09523809524	0	0	inf	9.52381E+31
**DBNDD2**	0.1428571429	0.1428571429	0.1666666667	0.1666666667	0.6666666667	0.8571428571
**DBNL**	0.04761904762	0.04761904762	0.1666666667	0.1666666667	0.2	0.2857142857
**DBP**	0.09523809524	0.09523809524	0.1111111111	0.1111111111	0.6842105263	0.8571428571
**DBR1**	0.1428571429	0.1428571429	0.05555555556	0.05555555556	2.333333333	2.571428571
**DBT**	0.04761904762	0.04761904762	0.05555555556	0.05555555556	0.7	0.8571428571
**DBX2**	0.04761904762	0.04761904762	0.05555555556	0.05555555556	0.7	0.8571428571
**DCAF1**	0.04761904762	0.04761904762	0	0	inf	4.7619E+31
**DCAF10**	0.09523809524	0.09523809524	0.05555555556	0.05555555556	1.473684211	1.714285714
**DCAF12**	0.09523809524	0.09523809524	0.05555555556	0.05555555556	1.473684211	1.714285714
**DCAF13**	0.1904761905	0.1904761905	0.1111111111	0.1111111111	1.529411765	1.714285714
**CAF13P**	0.1428571429	0.1428571429	0.05555555556	0.05555555556	2.333333333	2.571428571
**DCAF15**	0.1428571429	0.1428571429	0.1111111111	0.1111111111	1.083333333	1.285714286
**DCAF16**	0	0	0	0		0.00001
**DCAF17**	0	0	0.05555555556	0.05555555556	0	0
**DCAF4**	0.09523809524	0.09523809524	0	0	inf	9.52381E+31
**CAF4L**	0.04761904762	0.04761904762	0	0	inf	4.7619E+31
**CAF4L**	0.1904761905	0.1904761905	0.1666666667	0.1666666667	0.9411764706	1.142857143
**DCAF6**	0.1428571429	0.1428571429	0	0	inf	1.42857E+32
**DCAF7**	0.04761904762	0.04761904762	0	0	inf	4.7619E+31
**DCAF8**	0.1428571429	0.1428571429	0	0	inf	1.42857E+32
**DCAKD**	0.1428571429	0.1428571429	0	0	inf	1.42857E+32
**DCANP1**	0.04761904762	0.04761904762	0	0	inf	4.7619E+31
**DCBLD2**	0.09523809524	0.09523809524	0.05555555556	0.05555555556	1.473684211	1.714285714
**DCC**	0.09523809524	0.09523809524	0	0	inf	9.52381E+31
**DCDC2**	0	0	0.05555555556	0.05555555556	0	0
**DCDC2B**	0.04761904762	0.04761904762	0.1111111111	0.1111111111	0.325	0.4285714286
**DCDC2C**	0	0	0.05555555556	0.05555555556	0	0
**DCHS1**	0.04761904762	0.04761904762	0	0	inf	4.7619E+31
**DCHS2**	0	0	0.05555555556	0.05555555556	0	0
**DCK**	0.09523809524	0.09523809524	0.05555555556	0.05555555556	1.473684211	1.714285714
**DCLK2**	0	0	0.05555555556	0.05555555556	0	0
**DCLK3**	0.04761904762	0.04761904762	0	0	inf	4.7619E+31
**CLRE1**	0.1428571429	0.1428571429	0.05555555556	0.05555555556	2.333333333	2.571428571
**CLRE1**	0.04761904762	0.04761904762	0.05555555556	0.05555555556	0.7	0.8571428571

CLRE1(	0.09523809524	0.09523809524	0.1111111111	0.1111111111	0.6842105263	0.8571428571
CLRE1C	0.09523809524	0.09523809524	0.1111111111	0.1111111111	0.6842105263	0.8571428571
DCN	0.04761904762	0.04761904762	0.05555555556	0.05555555556	0.7	0.8571428571
DCP1A	0.04761904762	0.04761904762	0	0	inf	4.7619E+31
DCP1B	0.04761904762	0.04761904762	0.1666666667	0.1666666667	0.2	0.2857142857
DCP2	0.04761904762	0.04761904762	0	0	inf	4.7619E+31
DCST1	0.1904761905	0.1904761905	0.1111111111	0.1111111111	1.529411765	1.714285714
CST1-AS	0.1904761905	0.1904761905	0.1111111111	0.1111111111	1.529411765	1.714285714
DCST2	0.1904761905	0.1904761905	0.1111111111	0.1111111111	1.529411765	1.714285714
CSTAM	0.1904761905	0.1904761905	0.1111111111	0.1111111111	1.529411765	1.714285714
DCTD	0	0	0.05555555556	0.05555555556	0	0
DCTN1	0	0	0.05555555556	0.05555555556	0	0
CTN1-AS	0	0	0.05555555556	0.05555555556	0	0
DCTN3	0.09523809524	0.09523809524	0.05555555556	0.05555555556	1.473684211	1.714285714
DCTN5	0.04761904762	0.04761904762	0	0	inf	4.7619E+31
DCTN6	0.04761904762	0.04761904762	0.1111111111	0.1111111111	0.325	0.4285714286
DCTPP1	0.04761904762	0.04761904762	0	0	inf	4.7619E+31
CUN1D	0.1904761905	0.1904761905	0.05555555556	0.05555555556	3.294117647	3.428571429
CUN1D	0.04761904762	0.04761904762	0	0	inf	4.7619E+31
CUN1D	0.2380952381	0.2380952381	0.05555555556	0.05555555556	4.375	4.285714286
DCXR	0.04761904762	0.04761904762	0	0	inf	4.7619E+31
CXR-DT	0.04761904762	0.04761904762	0	0	inf	4.7619E+31
DDA1	0.1428571429	0.1428571429	0.1111111111	0.1111111111	1.083333333	1.285714286
DDAH1	0.04761904762	0.04761904762	0.05555555556	0.05555555556	0.7	0.8571428571
DDAH2	0.04761904762	0.04761904762	0	0	inf	4.7619E+31
DDB2	0	0	0.05555555556	0.05555555556	0	0
DDC	0.1428571429	0.1428571429	0.2222222222	0.2222222222	0.4583333333	0.6428571429
DC-AS1	0.1428571429	0.1428571429	0.2222222222	0.2222222222	0.4583333333	0.6428571429
DDHD1	0.04761904762	0.04761904762	0	0	inf	4.7619E+31
DDHD2	0.09523809524	0.09523809524	0.1111111111	0.1111111111	0.6842105263	0.8571428571
DDI2	0.04761904762	0.04761904762	0.1111111111	0.1111111111	0.325	0.4285714286
DDIT4	0.1904761905	0.1904761905	0.05555555556	0.05555555556	3.294117647	3.428571429
DDIT4L	0	0	0.05555555556	0.05555555556	0	0
DDN	0.04761904762	0.04761904762	0.05555555556	0.05555555556	0.7	0.8571428571
DN-AS1	0.04761904762	0.04761904762	0.05555555556	0.05555555556	0.7	0.8571428571
DDO	0	0	0	0		0.00001
DDOST	0.04761904762	0.04761904762	0.1111111111	0.1111111111	0.325	0.4285714286
DDR2	0.1428571429	0.1428571429	0	0	inf	1.42857E+32
DRGK1	0.1904761905	0.1904761905	0.05555555556	0.05555555556	3.294117647	3.428571429
DDT	0.04761904762	0.04761904762	0.1111111111	0.1111111111	0.325	0.4285714286
DDTL	0.04761904762	0.04761904762	0.1111111111	0.1111111111	0.325	0.4285714286
DDX1	0	0	0.05555555556	0.05555555556	0	0
DDX11	0.04761904762	0.04761904762	0.05555555556	0.05555555556	0.7	0.8571428571
DX11-AS	0.04761904762	0.04761904762	0.05555555556	0.05555555556	0.7	0.8571428571
DDX11L	0.04761904762	0.04761904762	0.1666666667	0.1666666667	0.2	0.2857142857
DX11L1	0.04761904762	0.04761904762	0.2222222222	0.2222222222	0.1375	0.2142857143
DX11L	0	0	0.05555555556	0.05555555556	0	0
DX11L	0.04761904762	0.04761904762	0.05555555556	0.05555555556	0.7	0.8571428571
DX11L	0.04761904762	0.04761904762	0.05555555556	0.05555555556	0.7	0.8571428571
DDX12P	0.04761904762	0.04761904762	0.1111111111	0.1111111111	0.325	0.4285714286
DDX17	0.04761904762	0.04761904762	0.1111111111	0.1111111111	0.325	0.4285714286

**DDX18**	0	0	0.05555555556	0.05555555556	0	0
**DDX19A**	0.1428571429	0.1428571429	0.05555555556	0.05555555556	2.333333333	2.571428571
**DDX19B**	0.1428571429	0.1428571429	0.05555555556	0.05555555556	2.333333333	2.571428571
**DDX20**	0.04761904762	0.04761904762	0	0	inf	4.7619E+31
**DDX21**	0.1904761905	0.1904761905	0.05555555556	0.05555555556	3.294117647	3.428571429
**DDX23**	0.04761904762	0.04761904762	0.05555555556	0.05555555556	0.7	0.8571428571
**DDX27**	0.09523809524	0.09523809524	0.1666666667	0.1666666667	0.4210526316	0.5714285714
**DDX28**	0.1428571429	0.1428571429	0.05555555556	0.05555555556	2.333333333	2.571428571
**DDX31**	0.04761904762	0.04761904762	0	0	inf	4.7619E+31
**DDX39A**	0.1428571429	0.1428571429	0.1111111111	0.1111111111	1.083333333	1.285714286
**DDX39B**	0.04761904762	0.04761904762	0	0	inf	4.7619E+31
**DX39B-A**	0.04761904762	0.04761904762	0	0	inf	4.7619E+31
**DDX4**	0.04761904762	0.04761904762	0.05555555556	0.05555555556	0.7	0.8571428571
**DDX41**	0.09523809524	0.09523809524	0.05555555556	0.05555555556	1.473684211	1.714285714
**DDX42**	0.04761904762	0.04761904762	0	0	inf	4.7619E+31
**DDX43**	0.04761904762	0.04761904762	0	0	inf	4.7619E+31
**DDX46**	0.04761904762	0.04761904762	0	0	inf	4.7619E+31
**DDX47**	0.04761904762	0.04761904762	0.05555555556	0.05555555556	0.7	0.8571428571
**DDX49**	0.1428571429	0.1428571429	0.1111111111	0.1111111111	1.083333333	1.285714286
**DDX5**	0.04761904762	0.04761904762	0	0	inf	4.7619E+31
**DDX50**	0.1904761905	0.1904761905	0.05555555556	0.05555555556	3.294117647	3.428571429
**DDX51**	0.09523809524	0.09523809524	0.05555555556	0.05555555556	1.473684211	1.714285714
**DDX54**	0	0	0.05555555556	0.05555555556	0	0
**DDX55**	0.04761904762	0.04761904762	0.1111111111	0.1111111111	0.325	0.4285714286
**DDX56**	0.04761904762	0.04761904762	0.2222222222	0.2222222222	0.1375	0.2142857143
**DDX58**	0.09523809524	0.09523809524	0.05555555556	0.05555555556	1.473684211	1.714285714
**DDX59**	0.1428571429	0.1428571429	0	0	inf	1.42857E+32
**DDX60**	0	0	0.05555555556	0.05555555556	0	0
**DDX60L**	0	0	0.05555555556	0.05555555556	0	0
**DEAF1**	0.04761904762	0.04761904762	0	0	inf	4.7619E+31
**DECR1**	0.1904761905	0.1904761905	0.1111111111	0.1111111111	1.529411765	1.714285714
**DECR2**	0.04761904762	0.04761904762	0.2222222222	0.2222222222	0.1375	0.2142857143
**DEDD**	0.1428571429	0.1428571429	0	0	inf	1.42857E+32
**DEDD2**	0.04761904762	0.04761904762	0.05555555556	0.05555555556	0.7	0.8571428571
**DEF6**	0.04761904762	0.04761904762	0	0	inf	4.7619E+31
**DEF8**	0.09523809524	0.09523809524	0	0	inf	9.52381E+31
**DEFA1**	0.09523809524	0.09523809524	0.1111111111	0.1111111111	0.6842105263	0.8571428571
**DEFA10I**	0.09523809524	0.09523809524	0.1111111111	0.1111111111	0.6842105263	0.8571428571
**DEFA11I**	0.09523809524	0.09523809524	0.1111111111	0.1111111111	0.6842105263	0.8571428571
**DEFA1B**	0.09523809524	0.09523809524	0.1111111111	0.1111111111	0.6842105263	0.8571428571
**DEFA3**	0.09523809524	0.09523809524	0.1111111111	0.1111111111	0.6842105263	0.8571428571
**DEFA4**	0.09523809524	0.09523809524	0.1111111111	0.1111111111	0.6842105263	0.8571428571
**DEFA5**	0.09523809524	0.09523809524	0.1111111111	0.1111111111	0.6842105263	0.8571428571
**DEFA6**	0.09523809524	0.09523809524	0.1111111111	0.1111111111	0.6842105263	0.8571428571
**DEFA8P**	0.09523809524	0.09523809524	0.1111111111	0.1111111111	0.6842105263	0.8571428571
**DEFA9P**	0.09523809524	0.09523809524	0.1111111111	0.1111111111	0.6842105263	0.8571428571
**DEFB1**	0.09523809524	0.09523809524	0.1111111111	0.1111111111	0.6842105263	0.8571428571
**EFB103**	0.09523809524	0.09523809524	0.2222222222	0.2222222222	0.2894736842	0.4285714286
**EFB103**	0.09523809524	0.09523809524	0.2222222222	0.2222222222	0.2894736842	0.4285714286
**EFB104**	0.09523809524	0.09523809524	0.2222222222	0.2222222222	0.2894736842	0.4285714286
**EFB104**	0.09523809524	0.09523809524	0.2222222222	0.2222222222	0.2894736842	0.4285714286

**EFB105**	0.09523809524	0.09523809524	0.2222222222	0.2222222222	0.2894736842	0.4285714286
**EFB105**	0.09523809524	0.09523809524	0.2222222222	0.2222222222	0.2894736842	0.4285714286
**EFB106**	0.09523809524	0.09523809524	0.2222222222	0.2222222222	0.2894736842	0.4285714286
**EFB106**	0.09523809524	0.09523809524	0.2222222222	0.2222222222	0.2894736842	0.4285714286
**EFB107**	0.09523809524	0.09523809524	0.2222222222	0.2222222222	0.2894736842	0.4285714286
**EFB107**	0.09523809524	0.09523809524	0.2222222222	0.2222222222	0.2894736842	0.4285714286
**EFB109**	0.09523809524	0.09523809524	0.1111111111	0.1111111111	0.6842105263	0.8571428571
**EFB109**	0.09523809524	0.09523809524	0.2222222222	0.2222222222	0.2894736842	0.4285714286
**DEFB11**	0.04761904762	0.04761904762	0	0	inf	4.7619E+31
**DEFB11**	0.04761904762	0.04761904762	0	0	inf	4.7619E+31
**DEFB11**	0.04761904762	0.04761904762	0	0	inf	4.7619E+31
**DEFB11**	0.04761904762	0.04761904762	0	0	inf	4.7619E+31
**DEFB11**	0.09523809524	0.09523809524	0.05555555556	0.05555555556	1.473684211	1.714285714
**DEFB11**	0.09523809524	0.09523809524	0.05555555556	0.05555555556	1.473684211	1.714285714
**DEFB11**	0.09523809524	0.09523809524	0.05555555556	0.05555555556	1.473684211	1.714285714
**DEFB11**	0.09523809524	0.09523809524	0.05555555556	0.05555555556	1.473684211	1.714285714
**DEFB12**	0.09523809524	0.09523809524	0.05555555556	0.05555555556	1.473684211	1.714285714
**DEFB12**	0.09523809524	0.09523809524	0.05555555556	0.05555555556	1.473684211	1.714285714
**DEFB12**	0.09523809524	0.09523809524	0.05555555556	0.05555555556	1.473684211	1.714285714
**DEFB12**	0.09523809524	0.09523809524	0.05555555556	0.05555555556	1.473684211	1.714285714
**DEFB12**	0.1904761905	0.1904761905	0.05555555556	0.05555555556	3.294117647	3.428571429
**DEFB12**	0.1904761905	0.1904761905	0.05555555556	0.05555555556	3.294117647	3.428571429
**DEFB12**	0.1904761905	0.1904761905	0.05555555556	0.05555555556	3.294117647	3.428571429
**DEFB12**	0.1904761905	0.1904761905	0.05555555556	0.05555555556	3.294117647	3.428571429
**DEFB12**	0.1904761905	0.1904761905	0.05555555556	0.05555555556	3.294117647	3.428571429
**EFB130**	0.09523809524	0.09523809524	0.1111111111	0.1111111111	0.6842105263	0.8571428571
**EFB130**	0.09523809524	0.09523809524	0.1111111111	0.1111111111	0.6842105263	0.8571428571
**DEFB13**	0.1904761905	0.1904761905	0.05555555556	0.05555555556	3.294117647	3.428571429
**DEFB13**	0.04761904762	0.04761904762	0	0	inf	4.7619E+31
**DEFB13**	0.09523809524	0.09523809524	0.1111111111	0.1111111111	0.6842105263	0.8571428571
**DEFB13**	0.09523809524	0.09523809524	0.1111111111	0.1111111111	0.6842105263	0.8571428571
**DEFB13**	0.09523809524	0.09523809524	0.1111111111	0.1111111111	0.6842105263	0.8571428571
**DEFB4A**	0.09523809524	0.09523809524	0.2222222222	0.2222222222	0.2894736842	0.4285714286
**DEFB4B**	0.09523809524	0.09523809524	0.2222222222	0.2222222222	0.2894736842	0.4285714286
**DEFT1P**	0.09523809524	0.09523809524	0.1111111111	0.1111111111	0.6842105263	0.8571428571
**DEFT1P**	0.09523809524	0.09523809524	0.1111111111	0.1111111111	0.6842105263	0.8571428571
**DEGS1**	0.1428571429	0.1428571429	0.05555555556	0.05555555556	2.333333333	2.571428571
**DELE1**	0.04761904762	0.04761904762	0	0	inf	4.7619E+31
**ENND1**	0.09523809524	0.09523809524	0.05555555556	0.05555555556	1.473684211	1.714285714
**ENND1**	0.1428571429	0.1428571429	0	0	inf	1.42857E+32
**ENND1**	0.1428571429	0.1428571429	0.05555555556	0.05555555556	2.333333333	2.571428571
**ENND2**	0.04761904762	0.04761904762	0.05555555556	0.05555555556	0.7	0.8571428571
**ENND2**	0.04761904762	0.04761904762	0.05555555556	0.05555555556	0.7	0.8571428571
**ENND2**	0.04761904762	0.04761904762	0	0	inf	4.7619E+31
**DENND3**	0.04761904762	0.04761904762	0.2222222222	0.2222222222	0.1375	0.2142857143
**ENND4**	0.04761904762	0.04761904762	0.05555555556	0.05555555556	0.7	0.8571428571
**ENND4**	0.1904761905	0.1904761905	0.05555555556	0.05555555556	3.294117647	3.428571429
**ENND4**	0.04761904762	0.04761904762	0.05555555556	0.05555555556	0.7	0.8571428571
**ENND5**	0.04761904762	0.04761904762	0.05555555556	0.05555555556	0.7	0.8571428571
**ENND5**	0.1428571429	0.1428571429	0.1111111111	0.1111111111	1.083333333	1.285714286
**NND5B-**	0.1428571429	0.1428571429	0.1111111111	0.1111111111	1.083333333	1.285714286

ENND6	0.09523809524	0.09523809524	0	0	inf	9.52381E+31
NND6A-	0.09523809524	0.09523809524	0	0	inf	9.52381E+31
ENND6	0	0	0.1111111111	0.1111111111	0	0
DENR	0.04761904762	0.04761904762	0.1111111111	0.1111111111	0.325	0.4285714286
DEPDC1	0.04761904762	0.04761904762	0.05555555556	0.05555555556	0.7	0.8571428571
PDC1-A	0.04761904762	0.04761904762	0.05555555556	0.05555555556	0.7	0.8571428571
EPDC1	0.04761904762	0.04761904762	0	0	inf	4.7619E+31
DEPDC4	0.04761904762	0.04761904762	0.05555555556	0.05555555556	0.7	0.8571428571
DEPDC5	0.04761904762	0.04761904762	0.1111111111	0.1111111111	0.325	0.4285714286
DEPDC7	0.04761904762	0.04761904762	0	0	inf	4.7619E+31
DEPP1	0.1428571429	0.1428571429	0.05555555556	0.05555555556	2.333333333	2.571428571
EPTOR	0.09523809524	0.09523809524	0.1666666667	0.1666666667	0.4210526316	0.5714285714
DERA	0.04761904762	0.04761904762	0.1111111111	0.1111111111	0.325	0.4285714286
DERL1	0.1428571429	0.1428571429	0.1111111111	0.1111111111	1.083333333	1.285714286
DERL2	0	0	0.05555555556	0.05555555556	0	0
DERL3	0.04761904762	0.04761904762	0.1111111111	0.1111111111	0.325	0.4285714286
DERPC	0.1428571429	0.1428571429	0.05555555556	0.05555555556	2.333333333	2.571428571
DES	0	0	0.05555555556	0.05555555556	0	0
DESI1	0	0	0.1111111111	0.1111111111	0	0
DESI2	0.1428571429	0.1428571429	0.05555555556	0.05555555556	2.333333333	2.571428571
DET1	0.04761904762	0.04761904762	0.05555555556	0.05555555556	0.7	0.8571428571
DEXI	0.04761904762	0.04761904762	0	0	inf	4.7619E+31
DFFA	0.04761904762	0.04761904762	0.1111111111	0.1111111111	0.325	0.4285714286
DFFB	0	0	0	0		0.00001
DGAT1	0.04761904762	0.04761904762	0.1666666667	0.1666666667	0.2	0.2857142857
DGCR10	0.04761904762	0.04761904762	0.05555555556	0.05555555556	0.7	0.8571428571
DGCR11	0.04761904762	0.04761904762	0.05555555556	0.05555555556	0.7	0.8571428571
DGCR2	0.04761904762	0.04761904762	0.05555555556	0.05555555556	0.7	0.8571428571
DGCR5	0.04761904762	0.04761904762	0.05555555556	0.05555555556	0.7	0.8571428571
DGCR6	0.04761904762	0.04761904762	0.05555555556	0.05555555556	0.7	0.8571428571
GCR6I	0.04761904762	0.04761904762	0.05555555556	0.05555555556	0.7	0.8571428571
DGCR8	0.04761904762	0.04761904762	0.05555555556	0.05555555556	0.7	0.8571428571
DGCR9	0.04761904762	0.04761904762	0.05555555556	0.05555555556	0.7	0.8571428571
DGKA	0.04761904762	0.04761904762	0.05555555556	0.05555555556	0.7	0.8571428571
DGKB	0.04761904762	0.04761904762	0.2222222222	0.2222222222	0.1375	0.2142857143
DGKD	0	0	0.05555555556	0.05555555556	0	0
DGKE	0.09523809524	0.09523809524	0	0	inf	9.52381E+31
DGKG	0.09523809524	0.09523809524	0.05555555556	0.05555555556	1.473684211	1.714285714
DGKI	0.04761904762	0.04761904762	0.05555555556	0.05555555556	0.7	0.8571428571
DGKQ	0.04761904762	0.04761904762	0	0	inf	4.7619E+31
DGKZ	0	0	0.05555555556	0.05555555556	0	0
DGUOK	0	0	0.05555555556	0.05555555556	0	0
UOK-A	0	0	0.05555555556	0.05555555556	0	0
DHCR24	0.04761904762	0.04761904762	0.05555555556	0.05555555556	0.7	0.8571428571
DHDDS	0.04761904762	0.04761904762	0.1111111111	0.1111111111	0.325	0.4285714286
DHDH	0.09523809524	0.09523809524	0.1111111111	0.1111111111	0.6842105263	0.8571428571
DHFR	0.04761904762	0.04761904762	0	0	inf	4.7619E+31
DHFR2	0.04761904762	0.04761904762	0.05555555556	0.05555555556	0.7	0.8571428571
DHFRP3	0	0	0.05555555556	0.05555555556	0	0
DHH	0.04761904762	0.04761904762	0.05555555556	0.05555555556	0.7	0.8571428571
DHODH	0.04761904762	0.04761904762	0	0	inf	4.7619E+31

DHPS	0.1428571429	0.1428571429	0.1111111111	0.1111111111	1.083333333	1.285714286
DHRS11	0.04761904762	0.04761904762	0	0	inf	4.7619E+31
DHRS13	0.04761904762	0.04761904762	0.05555555556	0.05555555556	0.7	0.8571428571
DHRS3	0.04761904762	0.04761904762	0.1111111111	0.1111111111	0.325	0.4285714286
DHRS7B	0.09523809524	0.09523809524	0.05555555556	0.05555555556	1.473684211	1.714285714
DHRS7C	0.04761904762	0.04761904762	0.05555555556	0.05555555556	0.7	0.8571428571
DHRS9	0.04761904762	0.04761904762	0.05555555556	0.05555555556	0.7	0.8571428571
DHTKD1	0.09523809524	0.09523809524	0.1111111111	0.1111111111	0.6842105263	0.8571428571
DHX29	0.04761904762	0.04761904762	0.05555555556	0.05555555556	0.7	0.8571428571
DHX30	0.09523809524	0.09523809524	0	0	inf	9.52381E+31
DHX32	0.2380952381	0.2380952381	0.05555555556	0.05555555556	4.375	4.285714286
DHX33	0	0	0.05555555556	0.05555555556	0	0
DHX34	0.09523809524	0.09523809524	0.1111111111	0.1111111111	0.6842105263	0.8571428571
DHX35	0.1428571429	0.1428571429	0.2222222222	0.2222222222	0.4583333333	0.6428571429
DHX36	0.1904761905	0.1904761905	0.05555555556	0.05555555556	3.294117647	3.428571429
DHX37	0.04761904762	0.04761904762	0.05555555556	0.05555555556	0.7	0.8571428571
DHX38	0.04761904762	0.04761904762	0	0	inf	4.7619E+31
DHX40	0.1428571429	0.1428571429	0	0	inf	1.42857E+32
DHX57	0	0	0.05555555556	0.05555555556	0	0
DHX58	0.04761904762	0.04761904762	0	0	inf	4.7619E+31
DHX8	0.04761904762	0.04761904762	0	0	inf	4.7619E+31
DHX9	0.1428571429	0.1428571429	0	0	inf	1.42857E+32
DIABLO	0.04761904762	0.04761904762	0.1111111111	0.1111111111	0.325	0.4285714286
DIAPH1	0.04761904762	0.04761904762	0	0	inf	4.7619E+31
DIDO1	0.1428571429	0.1428571429	0.2222222222	0.2222222222	0.4583333333	0.6428571429
DIMT1	0.04761904762	0.04761904762	0	0	inf	4.7619E+31
DINOL	0.04761904762	0.04761904762	0.05555555556	0.05555555556	0.7	0.8571428571
DIO1	0.04761904762	0.04761904762	0.05555555556	0.05555555556	0.7	0.8571428571
DIP2A	0.04761904762	0.04761904762	0.1111111111	0.1111111111	0.325	0.4285714286
IP2A-IT	0.04761904762	0.04761904762	0.1111111111	0.1111111111	0.325	0.4285714286
DIP2B	0.09523809524	0.09523809524	0.05555555556	0.05555555556	1.473684211	1.714285714
DIP2C	0.04761904762	0.04761904762	0.1111111111	0.1111111111	0.325	0.4285714286
DIPK1A	0.04761904762	0.04761904762	0.05555555556	0.05555555556	0.7	0.8571428571
DIPK1B	0.1428571429	0.1428571429	0.05555555556	0.05555555556	2.333333333	2.571428571
DIPK2A	0.1428571429	0.1428571429	0.1111111111	0.1111111111	1.083333333	1.285714286
DIRAS1	0.09523809524	0.09523809524	0.05555555556	0.05555555556	1.473684211	1.714285714
DIRAS2	0.04761904762	0.04761904762	0.05555555556	0.05555555556	0.7	0.8571428571
DIRAS3	0.04761904762	0.04761904762	0.05555555556	0.05555555556	0.7	0.8571428571
DIRC1	0	0	0.1666666667	0.1666666667	0	0
DIRC3	0	0	0.1111111111	0.1111111111	0	0
IRC3-AS	0	0	0.1111111111	0.1111111111	0	0
DIS3L	0.04761904762	0.04761904762	0.05555555556	0.05555555556	0.7	0.8571428571
DIS3L2	0	0	0.05555555556	0.05555555556	0	0
DISC1	0.1428571429	0.1428571429	0	0	inf	1.42857E+32
ISC1-IT	0.1428571429	0.1428571429	0	0	inf	1.42857E+32
DISC2	0.1428571429	0.1428571429	0	0	inf	1.42857E+32
DISP1	0.1428571429	0.1428571429	0.05555555556	0.05555555556	2.333333333	2.571428571
DISP3	0.04761904762	0.04761904762	0.1111111111	0.1111111111	0.325	0.4285714286
FZP434A	0.1428571429	0.1428571429	0.05555555556	0.05555555556	2.333333333	2.571428571
FZP434H	0.04761904762	0.04761904762	0	0	inf	4.7619E+31
FZP586I	0.04761904762	0.04761904762	0.1666666667	0.1666666667	0.2	0.2857142857

FZp434L	0.1428571429	0.1428571429	0.1666666667	0.1666666667	0.6666666667	0.8571428571
FZp451B	0	0	0.1111111111	0.1111111111	0	0
DKK1	0.04761904762	0.04761904762	0.05555555556	0.05555555556	0.7	0.8571428571
DKK2	0	0	0.05555555556	0.05555555556	0	0
DKK4	0.09523809524	0.09523809524	0.1666666667	0.1666666667	0.4210526316	0.5714285714
DKKL1	0.09523809524	0.09523809524	0.1111111111	0.1111111111	0.6842105263	0.8571428571
DLC1	0.09523809524	0.09523809524	0.1111111111	0.1111111111	0.6842105263	0.8571428571
DLD	0.04761904762	0.04761904762	0.05555555556	0.05555555556	0.7	0.8571428571
DLEC1	0.04761904762	0.04761904762	0	0	inf	4.7619E+31
DLEU2L	0.04761904762	0.04761904762	0.05555555556	0.05555555556	0.7	0.8571428571
DLG1	0.1428571429	0.1428571429	0.05555555556	0.05555555556	2.333333333	2.571428571
LG1-AS	0.1428571429	0.1428571429	0.05555555556	0.05555555556	2.333333333	2.571428571
DLG4	0	0	0.05555555556	0.05555555556	0	0
DLG5	0.1428571429	0.1428571429	0.05555555556	0.05555555556	2.333333333	2.571428571
LG5-AS	0.1428571429	0.1428571429	0.05555555556	0.05555555556	2.333333333	2.571428571
DLGAP1	0.09523809524	0.09523809524	0	0	inf	9.52381E+31
GAP1-A	0.09523809524	0.09523809524	0	0	inf	9.52381E+31
GAP1-A	0.09523809524	0.09523809524	0	0	inf	9.52381E+31
GAP1-A	0.09523809524	0.09523809524	0	0	inf	9.52381E+31
GAP1-A	0.09523809524	0.09523809524	0	0	inf	9.52381E+31
GAP1-A	0.09523809524	0.09523809524	0	0	inf	9.52381E+31
DLGAP2	0.1428571429	0.1428571429	0.1111111111	0.1111111111	1.083333333	1.285714286
GAP2-A	0.1428571429	0.1428571429	0.1111111111	0.1111111111	1.083333333	1.285714286
DLGAP3	0.04761904762	0.04761904762	0	0	inf	4.7619E+31
DLGAP4	0.09523809524	0.09523809524	0.1111111111	0.1111111111	0.6842105263	0.8571428571
GAP4-A	0.09523809524	0.09523809524	0.1111111111	0.1111111111	0.6842105263	0.8571428571
DLGAP5	0.04761904762	0.04761904762	0	0	inf	4.7619E+31
DLK2	0.04761904762	0.04761904762	0.05555555556	0.05555555556	0.7	0.8571428571
DLL3	0.04761904762	0.04761904762	0.05555555556	0.05555555556	0.7	0.8571428571
DLSTP1	0.04761904762	0.04761904762	0.05555555556	0.05555555556	0.7	0.8571428571
DLX1	0	0	0.05555555556	0.05555555556	0	0
DLX2	0	0	0.05555555556	0.05555555556	0	0
DLX2-DT	0	0	0.05555555556	0.05555555556	0	0
DLX3	0.09523809524	0.09523809524	0	0	inf	9.52381E+31
DLX4	0.09523809524	0.09523809524	0	0	inf	9.52381E+31
DLX5	0.04761904762	0.04761904762	0.1111111111	0.1111111111	0.325	0.4285714286
DLX6	0.04761904762	0.04761904762	0.1111111111	0.1111111111	0.325	0.4285714286
LX6-AS	0.04761904762	0.04761904762	0.1111111111	0.1111111111	0.325	0.4285714286
DM1-AS	0.09523809524	0.09523809524	0.1111111111	0.1111111111	0.6842105263	0.8571428571
DMAC1	0.04761904762	0.04761904762	0.05555555556	0.05555555556	0.7	0.8571428571
DMAC2	0.04761904762	0.04761904762	0.05555555556	0.05555555556	0.7	0.8571428571
MAC2L	0.04761904762	0.04761904762	0	0	inf	4.7619E+31
DMAP1	0.04761904762	0.04761904762	0.05555555556	0.05555555556	0.7	0.8571428571
DMBT1	0.2380952381	0.2380952381	0.05555555556	0.05555555556	4.375	4.285714286
MBT1P	0.2380952381	0.2380952381	0.05555555556	0.05555555556	4.375	4.285714286
DMBX1	0.04761904762	0.04761904762	0.05555555556	0.05555555556	0.7	0.8571428571
DMC1	0.04761904762	0.04761904762	0.1111111111	0.1111111111	0.325	0.4285714286
DMGDH	0.04761904762	0.04761904762	0	0	inf	4.7619E+31
DMKN	0.04761904762	0.04761904762	0.05555555556	0.05555555556	0.7	0.8571428571
DMP1	0	0	0.05555555556	0.05555555556	0	0
DMPK	0.09523809524	0.09523809524	0.1111111111	0.1111111111	0.6842105263	0.8571428571

**DMRT1**	0.04761904762	0.04761904762	0.05555555556	0.05555555556	0.7	0.8571428571
**DMRT2**	0.04761904762	0.04761904762	0.05555555556	0.05555555556	0.7	0.8571428571
**DMRT3**	0.04761904762	0.04761904762	0.05555555556	0.05555555556	0.7	0.8571428571
**DMRTA**	0.09523809524	0.09523809524	0.05555555556	0.05555555556	1.473684211	1.714285714
**DMRTA**	0	0	0	0		0.00001
**DMRTB**	0.04761904762	0.04761904762	0.05555555556	0.05555555556	0.7	0.8571428571
**DMRTC**	0.04761904762	0.04761904762	0.05555555556	0.05555555556	0.7	0.8571428571
**DMTF1**	0.09523809524	0.09523809524	0.05555555556	0.05555555556	1.473684211	1.714285714
**DMTN**	0.09523809524	0.09523809524	0.1111111111	0.1111111111	0.6842105263	0.8571428571
**DMWD**	0.09523809524	0.09523809524	0.1111111111	0.1111111111	0.6842105263	0.8571428571
**DMXL1**	0.04761904762	0.04761904762	0	0	inf	4.7619E+31
**DMXL2**	0.04761904762	0.04761904762	0.05555555556	0.05555555556	0.7	0.8571428571
**DNA2**	0.1904761905	0.1904761905	0.05555555556	0.05555555556	3.294117647	3.428571429
**DNAAF1**	0.04761904762	0.04761904762	0	0	inf	4.7619E+31
**DNAAF2**	0.04761904762	0.04761904762	0	0	inf	4.7619E+31
**DNAAF3**	0.09523809524	0.09523809524	0.05555555556	0.05555555556	1.473684211	1.714285714
**DNAAF4**	0.04761904762	0.04761904762	0.05555555556	0.05555555556	0.7	0.8571428571
**AF4-CC**	0.04761904762	0.04761904762	0.05555555556	0.05555555556	0.7	0.8571428571
**DNAAF5**	0.04761904762	0.04761904762	0.1666666667	0.1666666667	0.2	0.2857142857
**DNAH1**	0.04761904762	0.04761904762	0	0	inf	4.7619E+31
**DNAH10**	0.04761904762	0.04761904762	0.05555555556	0.05555555556	0.7	0.8571428571
**DNAH11**	0.04761904762	0.04761904762	0.1666666667	0.1666666667	0.2	0.2857142857
**DNAH12**	0.09523809524	0.09523809524	0	0	inf	9.52381E+31
**DNAH14**	0.1428571429	0.1428571429	0.05555555556	0.05555555556	2.333333333	2.571428571
**DNAH17**	0.04761904762	0.04761904762	0	0	inf	4.7619E+31
**AH17-A**	0.04761904762	0.04761904762	0	0	inf	4.7619E+31
**DNAH2**	0	0	0.05555555556	0.05555555556	0	0
**DNAH3**	0.04761904762	0.04761904762	0	0	inf	4.7619E+31
**DNAH5**	0.04761904762	0.04761904762	0.05555555556	0.05555555556	0.7	0.8571428571
**DNAH6**	0	0	0.05555555556	0.05555555556	0	0
**DNAH7**	0	0	0.05555555556	0.05555555556	0	0
**DNAH8**	0.04761904762	0.04761904762	0.05555555556	0.05555555556	0.7	0.8571428571
**DNAH9**	0.04761904762	0.04761904762	0.05555555556	0.05555555556	0.7	0.8571428571
**DNAI1**	0.09523809524	0.09523809524	0.05555555556	0.05555555556	1.473684211	1.714285714
**DNAI2**	0.04761904762	0.04761904762	0	0	inf	4.7619E+31
**DNAJA1**	0.09523809524	0.09523809524	0.05555555556	0.05555555556	1.473684211	1.714285714
**NAJA1P**	0.04761904762	0.04761904762	0.05555555556	0.05555555556	0.7	0.8571428571
**DNAJA2**	0.04761904762	0.04761904762	0	0	inf	4.7619E+31
**DNAJA3**	0.04761904762	0.04761904762	0	0	inf	4.7619E+31
**DNAJA4**	0.04761904762	0.04761904762	0.05555555556	0.05555555556	0.7	0.8571428571
**DNAJB1**	0.1428571429	0.1428571429	0.1111111111	0.1111111111	1.083333333	1.285714286
**DNAJB1**	0.09523809524	0.09523809524	0.05555555556	0.05555555556	1.473684211	1.714285714
**DNAJB1**	0.1904761905	0.1904761905	0.05555555556	0.05555555556	3.294117647	3.428571429
**DNAJB1**	0	0	0.05555555556	0.05555555556	0	0
**DNAJB3**	0	0	0.05555555556	0.05555555556	0	0
**DNAJB4**	0.04761904762	0.04761904762	0.05555555556	0.05555555556	0.7	0.8571428571
**DNAJB5**	0.09523809524	0.09523809524	0.05555555556	0.05555555556	1.473684211	1.714285714
**NAJB5-D**	0.09523809524	0.09523809524	0.05555555556	0.05555555556	1.473684211	1.714285714
**DNAJB6**	0.1428571429	0.1428571429	0.05555555556	0.05555555556	2.333333333	2.571428571
**DNAJB7**	0	0	0.1111111111	0.1111111111	0	0
**DNAJB8**	0.09523809524	0.09523809524	0.05555555556	0.05555555556	1.473684211	1.714285714

NAJB8-A	0.09523809524	0.09523809524	0.05555555556	0.05555555556	1.473684211	1.714285714
DNAJB9	0.04761904762	0.04761904762	0.05555555556	0.05555555556	0.7	0.8571428571
DNAJC1	0.09523809524	0.09523809524	0.1111111111	0.1111111111	0.6842105263	0.8571428571
DNAJC1(	0	0	0.05555555556	0.05555555556	0	0
DNAJC1	0	0	0.05555555556	0.05555555556	0	0
DNAJC1:	0.1904761905	0.1904761905	0.05555555556	0.05555555556	3.294117647	3.428571429
DNAJC1.	0.1428571429	0.1428571429	0.05555555556	0.05555555556	2.333333333	2.571428571
DNAJC1	0.04761904762	0.04761904762	0.05555555556	0.05555555556	0.7	0.8571428571
DNAJC1	0.04761904762	0.04761904762	0.1111111111	0.1111111111	0.325	0.4285714286
DNAJC1	0.04761904762	0.04761904762	0	0	inf	4.7619E+31
DNAJC1	0.1904761905	0.1904761905	0.05555555556	0.05555555556	3.294117647	3.428571429
DNAJC2	0.04761904762	0.04761904762	0.05555555556	0.05555555556	0.7	0.8571428571
DNAJC2	0.04761904762	0.04761904762	0.05555555556	0.05555555556	0.7	0.8571428571
DNAJC2	0.04761904762	0.04761904762	0.05555555556	0.05555555556	0.7	0.8571428571
DNAJC2	0.04761904762	0.04761904762	0.05555555556	0.05555555556	0.7	0.8571428571
JC25-GN	0.04761904762	0.04761904762	0.05555555556	0.05555555556	0.7	0.8571428571
DNAJC2	0.04761904762	0.04761904762	0.1111111111	0.1111111111	0.325	0.4285714286
DNAJC3	0.1428571429	0.1428571429	0.1111111111	0.1111111111	1.083333333	1.285714286
DNAJC5	0.1428571429	0.1428571429	0.2222222222	0.2222222222	0.4583333333	0.6428571429
DNAJC5I	0.1428571429	0.1428571429	0.1666666667	0.1666666667	0.6666666667	0.8571428571
DNAJC6	0.04761904762	0.04761904762	0.05555555556	0.05555555556	0.7	0.8571428571
DNAJC7	0.09523809524	0.09523809524	0	0	inf	9.52381E+31
DNAJC8	0.04761904762	0.04761904762	0.1111111111	0.1111111111	0.325	0.4285714286
DNAJC9	0.1904761905	0.1904761905	0.05555555556	0.05555555556	3.294117647	3.428571429
NAJC9-A	0.1904761905	0.1904761905	0.05555555556	0.05555555556	3.294117647	3.428571429
DNAL1	0.09523809524	0.09523809524	0	0	inf	9.52381E+31
DNAL4	0.04761904762	0.04761904762	0.1111111111	0.1111111111	0.325	0.4285714286
DNALI1	0.04761904762	0.04761904762	0.05555555556	0.05555555556	0.7	0.8571428571
DNASE1	0.04761904762	0.04761904762	0.1111111111	0.1111111111	0.325	0.4285714286
NASE1L	0.09523809524	0.09523809524	0.2222222222	0.2222222222	0.2894736842	0.4285714286
NASE1L	0.04761904762	0.04761904762	0	0	inf	4.7619E+31
DNASE2	0.1428571429	0.1428571429	0.1111111111	0.1111111111	1.083333333	1.285714286
DNASE2I	0.04761904762	0.04761904762	0.05555555556	0.05555555556	0.7	0.8571428571
DND1	0.04761904762	0.04761904762	0	0	inf	4.7619E+31
DNER	0	0	0.05555555556	0.05555555556	0	0
DNHD1	0.04761904762	0.04761904762	0	0	inf	4.7619E+31
DNLZ	0.1428571429	0.1428571429	0.05555555556	0.05555555556	2.333333333	2.571428571
DNM1	0.09523809524	0.09523809524	0.05555555556	0.05555555556	1.473684211	1.714285714
DNM1L	0.1428571429	0.1428571429	0.1111111111	0.1111111111	1.083333333	1.285714286
DNM1P3	0.04761904762	0.04761904762	0.05555555556	0.05555555556	0.7	0.8571428571
DNM1P4	0.04761904762	0.04761904762	0.05555555556	0.05555555556	0.7	0.8571428571
DNM1P4	0.04761904762	0.04761904762	0.05555555556	0.05555555556	0.7	0.8571428571
DNM2	0.1428571429	0.1428571429	0.1111111111	0.1111111111	1.083333333	1.285714286
DNM3	0.1428571429	0.1428571429	0.05555555556	0.05555555556	2.333333333	2.571428571
DNM3-IT	0.1428571429	0.1428571429	0.05555555556	0.05555555556	2.333333333	2.571428571
DNM3OS	0.1428571429	0.1428571429	0.05555555556	0.05555555556	2.333333333	2.571428571
DNMBP	0.1428571429	0.1428571429	0	0	inf	1.42857E+32
NMBP-A	0.1428571429	0.1428571429	0	0	inf	1.42857E+32
DNMT1	0.1428571429	0.1428571429	0.1111111111	0.1111111111	1.083333333	1.285714286
DNMT3E	0.09523809524	0.09523809524	0.1111111111	0.1111111111	0.6842105263	0.8571428571
DNMT3I	0.04761904762	0.04761904762	0.1111111111	0.1111111111	0.325	0.4285714286

**DNPEP**	0	0	0.05555555556	0.05555555556	0	0
**DNPH1**	0.04761904762	0.04761904762	0.05555555556	0.05555555556	0.7	0.8571428571
**DNTT**	0.1428571429	0.1428571429	0	0	inf	1.42857E+32
**NTTIP1**	0.1428571429	0.1428571429	0.1666666667	0.1666666667	0.6666666667	0.8571428571
**NTTIP2**	0.04761904762	0.04761904762	0.05555555556	0.05555555556	0.7	0.8571428571
**DOC2A**	0.04761904762	0.04761904762	0	0	inf	4.7619E+31
**DOCK1**	0.2380952381	0.2380952381	0.05555555556	0.05555555556	4.375	4.285714286
**DOCK10**	0	0	0.05555555556	0.05555555556	0	0
**DOCK2**	0	0	0.05555555556	0.05555555556	0	0
**DOCK3**	0.09523809524	0.09523809524	0	0	inf	9.52381E+31
**DOCK4**	0.04761904762	0.04761904762	0.05555555556	0.05555555556	0.7	0.8571428571
**CK4-A**	0.04761904762	0.04761904762	0.05555555556	0.05555555556	0.7	0.8571428571
**DOCK5**	0.04761904762	0.04761904762	0.1111111111	0.1111111111	0.325	0.4285714286
**DOCK6**	0.1428571429	0.1428571429	0.1111111111	0.1111111111	1.083333333	1.285714286
**DOCK7**	0.04761904762	0.04761904762	0.05555555556	0.05555555556	0.7	0.8571428571
**DOCK8**	0.04761904762	0.04761904762	0.05555555556	0.05555555556	0.7	0.8571428571
**DOHH**	0.09523809524	0.09523809524	0.05555555556	0.05555555556	1.473684211	1.714285714
**DOK1**	0	0	0.05555555556	0.05555555556	0	0
**DOK2**	0.09523809524	0.09523809524	0.1111111111	0.1111111111	0.6842105263	0.8571428571
**DOK3**	0.09523809524	0.09523809524	0.05555555556	0.05555555556	1.473684211	1.714285714
**DOK4**	0.04761904762	0.04761904762	0	0	inf	4.7619E+31
**DOK5**	0.09523809524	0.09523809524	0.1111111111	0.1111111111	0.6842105263	0.8571428571
**DOK7**	0.04761904762	0.04761904762	0	0	inf	4.7619E+31
**ONSON**	0.04761904762	0.04761904762	0.1111111111	0.1111111111	0.325	0.4285714286
**DOP1B**	0.04761904762	0.04761904762	0.1666666667	0.1666666667	0.2	0.2857142857
**DOT1L**	0.09523809524	0.09523809524	0.1111111111	0.1111111111	0.6842105263	0.8571428571
**DPCD**	0.1428571429	0.1428571429	0	0	inf	1.42857E+32
**DPEP1**	0.09523809524	0.09523809524	0	0	inf	9.52381E+31
**DPEP2**	0.1428571429	0.1428571429	0.05555555556	0.05555555556	2.333333333	2.571428571
**PEP2NI**	0.1428571429	0.1428571429	0.05555555556	0.05555555556	2.333333333	2.571428571
**DPEP3**	0.1428571429	0.1428571429	0.05555555556	0.05555555556	2.333333333	2.571428571
**DPF1**	0.04761904762	0.04761904762	0.05555555556	0.05555555556	0.7	0.8571428571
**DPF3**	0.04761904762	0.04761904762	0	0	inf	4.7619E+31
**DPH1**	0	0	0.1111111111	0.1111111111	0	0
**DPH2**	0.04761904762	0.04761904762	0.05555555556	0.05555555556	0.7	0.8571428571
**DPH3**	0.04761904762	0.04761904762	0	0	inf	4.7619E+31
**DPH3P1**	0.1428571429	0.1428571429	0.2222222222	0.2222222222	0.4583333333	0.6428571429
**DPH5**	0.04761904762	0.04761904762	0.05555555556	0.05555555556	0.7	0.8571428571
**DPH6**	0	0	0.05555555556	0.05555555556	0	0
**PH6-DT**	0	0	0.05555555556	0.05555555556	0	0
**DPH7**	0.09523809524	0.09523809524	0	0	inf	9.52381E+31
**DPM1**	0.09523809524	0.09523809524	0.1666666667	0.1666666667	0.4210526316	0.5714285714
**DPM2**	0.09523809524	0.09523809524	0.05555555556	0.05555555556	1.473684211	1.714285714
**DPM3**	0.1904761905	0.1904761905	0.1111111111	0.1111111111	1.529411765	1.714285714
**DPP10**	0	0	0.05555555556	0.05555555556	0	0
**PP10-AS**	0	0	0.05555555556	0.05555555556	0	0
**PP10-AS**	0	0	0.05555555556	0.05555555556	0	0
**DPP4**	0	0	0.05555555556	0.05555555556	0	0
**DPP6**	0.1428571429	0.1428571429	0.05555555556	0.05555555556	2.333333333	2.571428571
**DPP7**	0.1428571429	0.1428571429	0.05555555556	0.05555555556	2.333333333	2.571428571
**DPP8**	0.04761904762	0.04761904762	0.05555555556	0.05555555556	0.7	0.8571428571

DPP9	0.09523809524	0.09523809524	0.05555555556	0.05555555556	1.473684211	1.714285714
)PP9-AS	0.09523809524	0.09523809524	0.05555555556	0.05555555556	1.473684211	1.714285714
DPPA2	0.1428571429	0.1428571429	0.05555555556	0.05555555556	2.333333333	2.571428571
)PPA2P	0.09523809524	0.09523809524	0.05555555556	0.05555555556	1.473684211	1.714285714
DPPA3	0.04761904762	0.04761904762	0.1111111111	0.1111111111	0.325	0.4285714286
DPPA4	0.1428571429	0.1428571429	0.05555555556	0.05555555556	2.333333333	2.571428571
DPPA5	0.04761904762	0.04761904762	0	0	inf	4.7619E+31
DPRX	0.09523809524	0.09523809524	0.05555555556	0.05555555556	1.473684211	1.714285714
DPRXP4	0	0	0	0		0.00001
DPT	0.1428571429	0.1428571429	0	0	inf	1.42857E+32
)PY19L	0.04761904762	0.04761904762	0.2222222222	0.2222222222	0.1375	0.2142857143
PY19L1	0.04761904762	0.04761904762	0.1666666667	0.1666666667	0.2	0.2857142857
PY19L1	0.04761904762	0.04761904762	0.1666666667	0.1666666667	0.2	0.2857142857
)PY19L	0.09523809524	0.09523809524	0.05555555556	0.05555555556	1.473684211	1.714285714
PY19L2	0.04761904762	0.04761904762	0.2222222222	0.2222222222	0.1375	0.2142857143
PY19L2	0.04761904762	0.04761904762	0.05555555556	0.05555555556	0.7	0.8571428571
PY19L2	0.04761904762	0.04761904762	0.1666666667	0.1666666667	0.2	0.2857142857
PY19L2	0.09523809524	0.09523809524	0.05555555556	0.05555555556	1.473684211	1.714285714
)PY19L	0.04761904762	0.04761904762	0.05555555556	0.05555555556	0.7	0.8571428571
)PY19L	0.1904761905	0.1904761905	0.1111111111	0.1111111111	1.529411765	1.714285714
DPY30	0.1428571429	0.1428571429	0	0	inf	1.42857E+32
DPYD	0.04761904762	0.04761904762	0.05555555556	0.05555555556	0.7	0.8571428571
PYD-AS	0.04761904762	0.04761904762	0.05555555556	0.05555555556	0.7	0.8571428571
PYD-AS	0.04761904762	0.04761904762	0.05555555556	0.05555555556	0.7	0.8571428571
DPYS	0.1904761905	0.1904761905	0.1111111111	0.1111111111	1.529411765	1.714285714
DPYSL2	0.04761904762	0.04761904762	0.1111111111	0.1111111111	0.325	0.4285714286
DPYSL4	0.2380952381	0.2380952381	0	0	inf	2.38095E+32
DQX1	0	0	0.05555555556	0.05555555556	0	0
DR1	0.04761904762	0.04761904762	0.05555555556	0.05555555556	0.7	0.8571428571
DRAIC	0.04761904762	0.04761904762	0.05555555556	0.05555555556	0.7	0.8571428571
DRAM1	0.04761904762	0.04761904762	0.05555555556	0.05555555556	0.7	0.8571428571
DRAM2	0.04761904762	0.04761904762	0	0	inf	4.7619E+31
)RAXIN	0.04761904762	0.04761904762	0.1111111111	0.1111111111	0.325	0.4285714286
DRC3	0.04761904762	0.04761904762	0.1111111111	0.1111111111	0.325	0.4285714286
DRC7	0.04761904762	0.04761904762	0	0	inf	4.7619E+31
DRD1	0	0	0.05555555556	0.05555555556	0	0
DRD3	0.09523809524	0.09523809524	0.05555555556	0.05555555556	1.473684211	1.714285714
DRD4	0.04761904762	0.04761904762	0	0	inf	4.7619E+31
DRD5P2	0.1428571429	0.1428571429	0.05555555556	0.05555555556	2.333333333	2.571428571
DRG1	0.04761904762	0.04761904762	0.1111111111	0.1111111111	0.325	0.4285714286
DRG2	0.04761904762	0.04761904762	0.1111111111	0.1111111111	0.325	0.4285714286
DRGX	0.09523809524	0.09523809524	0.05555555556	0.05555555556	1.473684211	1.714285714
)ROSHA	0.04761904762	0.04761904762	0.05555555556	0.05555555556	0.7	0.8571428571
DSC1	0.09523809524	0.09523809524	0	0	inf	9.52381E+31
DSC2	0.09523809524	0.09523809524	0	0	inf	9.52381E+31
DSC3	0.09523809524	0.09523809524	0	0	inf	9.52381E+31
DSCAM	0.04761904762	0.04761904762	0.05555555556	0.05555555556	0.7	0.8571428571
SCAM-A	0.04761904762	0.04761904762	0.05555555556	0.05555555556	0.7	0.8571428571
SCAM-I	0.04761904762	0.04761904762	0.05555555556	0.05555555556	0.7	0.8571428571
DSCAS	0.09523809524	0.09523809524	0	0	inf	9.52381E+31
DSCC1	0.09523809524	0.09523809524	0.1666666667	0.1666666667	0.4210526316	0.5714285714

DSCR10	0.04761904762	0.04761904762	0.1111111111	0.1111111111	0.325	0.4285714286
DSCR4	0.04761904762	0.04761904762	0.1111111111	0.1111111111	0.325	0.4285714286
DSCR8	0.04761904762	0.04761904762	0.1111111111	0.1111111111	0.325	0.4285714286
DSCR9	0.04761904762	0.04761904762	0.1666666667	0.1666666667	0.2	0.2857142857
DSG1	0.09523809524	0.09523809524	0	0	inf	9.52381E+31
DSG1-AS	0.09523809524	0.09523809524	0	0	inf	9.52381E+31
DSG2	0.09523809524	0.09523809524	0	0	inf	9.52381E+31
DSG2-AS	0.09523809524	0.09523809524	0	0	inf	9.52381E+31
DSG3	0.09523809524	0.09523809524	0	0	inf	9.52381E+31
DSG4	0.09523809524	0.09523809524	0	0	inf	9.52381E+31
DSN1	0.09523809524	0.09523809524	0.1111111111	0.1111111111	0.6842105263	0.8571428571
DSPP	0	0	0.05555555556	0.05555555556	0	0
DST	0.04761904762	0.04761904762	0.05555555556	0.05555555556	0.7	0.8571428571
DSTN	0.1904761905	0.1904761905	0.1111111111	0.1111111111	1.529411765	1.714285714
DSTNP2	0.04761904762	0.04761904762	0.1111111111	0.1111111111	0.325	0.4285714286
DSTYK	0.1904761905	0.1904761905	0	0	inf	1.90476E+32
DTD1	0.1904761905	0.1904761905	0.05555555556	0.05555555556	3.294117647	3.428571429
DTD2	0	0	0	0		0.00001
DTL	0.1428571429	0.1428571429	0	0	inf	1.42857E+32
DTNA	0.09523809524	0.09523809524	0	0	inf	9.52381E+31
DTWD1	0.04761904762	0.04761904762	0.05555555556	0.05555555556	0.7	0.8571428571
DTWD2	0.04761904762	0.04761904762	0	0	inf	4.7619E+31
DTX1	0	0	0.05555555556	0.05555555556	0	0
DTX2	0.09523809524	0.09523809524	0.1111111111	0.1111111111	0.6842105263	0.8571428571
PK3BP1	0.04761904762	0.04761904762	0.1111111111	0.1111111111	0.325	0.4285714286
DTX3	0.1428571429	0.1428571429	0.05555555556	0.05555555556	2.333333333	2.571428571
DTX3L	0.09523809524	0.09523809524	0.05555555556	0.05555555556	1.473684211	1.714285714
DTYMK	0	0	0.05555555556	0.05555555556	0	0
DUBR	0.09523809524	0.09523809524	0.05555555556	0.05555555556	1.473684211	1.714285714
DUOX1	0.04761904762	0.04761904762	0	0	inf	4.7619E+31
DUOX2	0.04761904762	0.04761904762	0	0	inf	4.7619E+31
DUOXA1	0.04761904762	0.04761904762	0	0	inf	4.7619E+31
DUOXA2	0.04761904762	0.04761904762	0	0	inf	4.7619E+31
DUPD1	0.1904761905	0.1904761905	0.05555555556	0.05555555556	3.294117647	3.428571429
DUS1L	0.04761904762	0.04761904762	0	0	inf	4.7619E+31
DUS2	0.1428571429	0.1428571429	0.05555555556	0.05555555556	2.333333333	2.571428571
DUS3L	0.1428571429	0.1428571429	0.05555555556	0.05555555556	2.333333333	2.571428571
DUS4L	0.04761904762	0.04761904762	0.05555555556	0.05555555556	0.7	0.8571428571
DUSP1	0	0	0.05555555556	0.05555555556	0	0
DUSP10	0.1904761905	0.1904761905	0.05555555556	0.05555555556	3.294117647	3.428571429
DUSP11	0	0	0.05555555556	0.05555555556	0	0
DUSP12	0.1428571429	0.1428571429	0	0	inf	1.42857E+32
DUSP13	0.1904761905	0.1904761905	0.05555555556	0.05555555556	3.294117647	3.428571429
DUSP15	0.09523809524	0.09523809524	0.1111111111	0.1111111111	0.6842105263	0.8571428571
DUSP16	0.04761904762	0.04761904762	0.05555555556	0.05555555556	0.7	0.8571428571
DUSP18	0.04761904762	0.04761904762	0.1111111111	0.1111111111	0.325	0.4285714286
DUSP19	0	0	0.05555555556	0.05555555556	0	0
DUSP2	0	0	0.1111111111	0.1111111111	0	0
DUSP23	0.1428571429	0.1428571429	0	0	inf	1.42857E+32
DUSP26	0.04761904762	0.04761904762	0.1111111111	0.1111111111	0.325	0.4285714286
DUSP27	0.1428571429	0.1428571429	0	0	inf	1.42857E+32

DUSP28	0.04761904762	0.04761904762	0.05555555556	0.05555555556	0.7	0.8571428571
DUSP3	0.09523809524	0.09523809524	0	0	inf	9.52381E+31
DUSP4	0.04761904762	0.04761904762	0.1111111111	0.1111111111	0.325	0.4285714286
DUSP5	0.1428571429	0.1428571429	0.05555555556	0.05555555556	2.333333333	2.571428571
DUSP5P1	0.1428571429	0.1428571429	0	0	inf	1.42857E+32
DUSP6	0.04761904762	0.04761904762	0.05555555556	0.05555555556	0.7	0.8571428571
DUSP7	0.04761904762	0.04761904762	0	0	inf	4.7619E+31
DUSP8	0.04761904762	0.04761904762	0	0	inf	4.7619E+31
DUT	0.04761904762	0.04761904762	0.05555555556	0.05555555556	0.7	0.8571428571
DUXA	0.1428571429	0.1428571429	0.05555555556	0.05555555556	2.333333333	2.571428571
DUXAP1	0.1428571429	0.1428571429	0.1111111111	0.1111111111	1.083333333	1.285714286
DUXAP8	0.09523809524	0.09523809524	0.05555555556	0.05555555556	1.473684211	1.714285714
DUXAP9	0.1428571429	0.1428571429	0.1111111111	0.1111111111	1.083333333	1.285714286
DUXB	0.04761904762	0.04761904762	0	0	inf	4.7619E+31
DVL1	0	0	0.1111111111	0.1111111111	0	0
DVL2	0	0	0.05555555556	0.05555555556	0	0
DVL3	0.1428571429	0.1428571429	0.05555555556	0.05555555556	2.333333333	2.571428571
DXO	0.04761904762	0.04761904762	0	0	inf	4.7619E+31
DYDC1	0.1428571429	0.1428571429	0	0	inf	1.42857E+32
DYDC2	0.1428571429	0.1428571429	0	0	inf	1.42857E+32
DYM	0.04761904762	0.04761904762	0	0	inf	4.7619E+31
DYNAP	0.09523809524	0.09523809524	0	0	inf	9.52381E+31
DYNC1I1	0.04761904762	0.04761904762	0.1111111111	0.1111111111	0.325	0.4285714286
DYNC1I2	0	0	0.05555555556	0.05555555556	0	0
DYNC1LI	0.09523809524	0.09523809524	0	0	inf	9.52381E+31
DYNC1LI	0.09523809524	0.09523809524	0.05555555556	0.05555555556	1.473684211	1.714285714
DYNC2LI	0	0	0.05555555556	0.05555555556	0	0
DYNLL1	0	0	0.1111111111	0.1111111111	0	0
DYNLL2	0.09523809524	0.09523809524	0	0	inf	9.52381E+31
DYNLRB	0.09523809524	0.09523809524	0.1111111111	0.1111111111	0.6842105263	0.8571428571
DYNLRB	0.09523809524	0.09523809524	0	0	inf	9.52381E+31
DYRK1A	0.04761904762	0.04761904762	0.1666666667	0.1666666667	0.2	0.2857142857
DYRK1B	0.04761904762	0.04761904762	0.05555555556	0.05555555556	0.7	0.8571428571
DYRK2	0.09523809524	0.09523809524	0.05555555556	0.05555555556	1.473684211	1.714285714
DYRK3	0.1428571429	0.1428571429	0	0	inf	1.42857E+32
DYRK4	0.04761904762	0.04761904762	0.1111111111	0.1111111111	0.325	0.4285714286
DYSF	0	0	0.05555555556	0.05555555556	0	0
DYTN	0	0	0.05555555556	0.05555555556	0	0
DZANK1	0.1904761905	0.1904761905	0.05555555556	0.05555555556	3.294117647	3.428571429
DZIP1L	0.1428571429	0.1428571429	0.05555555556	0.05555555556	2.333333333	2.571428571
DZIP3	0.09523809524	0.09523809524	0.05555555556	0.05555555556	1.473684211	1.714285714
E2F1	0.09523809524	0.09523809524	0.1111111111	0.1111111111	0.6842105263	0.8571428571
E2F2	0.04761904762	0.04761904762	0.1111111111	0.1111111111	0.325	0.4285714286
E2F4	0.1428571429	0.1428571429	0.05555555556	0.05555555556	2.333333333	2.571428571
E2F5	0.1428571429	0.1428571429	0.1111111111	0.1111111111	1.083333333	1.285714286
E2F6	0	0	0.05555555556	0.05555555556	0	0
E2F7	0.04761904762	0.04761904762	0.1111111111	0.1111111111	0.325	0.4285714286
E4F1	0.09523809524	0.09523809524	0.2222222222	0.2222222222	0.2894736842	0.4285714286
EAF1	0.04761904762	0.04761904762	0	0	inf	4.7619E+31
EAF2	0.09523809524	0.09523809524	0.05555555556	0.05555555556	1.473684211	1.714285714
EAPP	0.09523809524	0.09523809524	0	0	inf	9.52381E+31

**EARS2**	0.04761904762	0.04761904762	0	0	inf	4.7619E+31
**EBAG9**	0.1904761905	0.1904761905	0.1111111111	0.1111111111	1.529411765	1.714285714
**EBF1**	0.04761904762	0.04761904762	0.05555555556	0.05555555556	0.7	0.8571428571
**EBF2**	0.04761904762	0.04761904762	0.1111111111	0.1111111111	0.325	0.4285714286
**EBF3**	0.2857142857	0.2857142857	0	0	inf	2.85714E+32
**EBF4**	0.1904761905	0.1904761905	0.05555555556	0.05555555556	3.294117647	3.428571429
**EBI3**	0.09523809524	0.09523809524	0.05555555556	0.05555555556	1.473684211	1.714285714
**EBLN1**	0.09523809524	0.09523809524	0.1111111111	0.1111111111	0.6842105263	0.8571428571
**EBLN2**	0.04761904762	0.04761904762	0	0	inf	4.7619E+31
**EBLN3P**	0.09523809524	0.09523809524	0.05555555556	0.05555555556	1.473684211	1.714285714
**BNA1BP**	0.04761904762	0.04761904762	0.05555555556	0.05555555556	0.7	0.8571428571
**ECD**	0.1904761905	0.1904761905	0.05555555556	0.05555555556	3.294117647	3.428571429
**ECE1**	0.04761904762	0.04761904762	0.1111111111	0.1111111111	0.325	0.4285714286
**ECE2**	0.1428571429	0.1428571429	0.05555555556	0.05555555556	2.333333333	2.571428571
**ECEL1**	0	0	0.05555555556	0.05555555556	0	0
**CEL1P**	0	0	0.05555555556	0.05555555556	0	0
**ECH1**	0.04761904762	0.04761904762	0.05555555556	0.05555555556	0.7	0.8571428571
**ECHDC2**	0.04761904762	0.04761904762	0.05555555556	0.05555555556	0.7	0.8571428571
**ECHDC3**	0.09523809524	0.09523809524	0.1111111111	0.1111111111	0.6842105263	0.8571428571
**ECHS1**	0.1904761905	0.1904761905	0	0	inf	1.90476E+32
**ECI1**	0.09523809524	0.09523809524	0.2222222222	0.2222222222	0.2894736842	0.4285714286
**ECM1**	0.1904761905	0.1904761905	0.05555555556	0.05555555556	3.294117647	3.428571429
**ECM2**	0.04761904762	0.04761904762	0.05555555556	0.05555555556	0.7	0.8571428571
**ECPAS**	0.04761904762	0.04761904762	0.05555555556	0.05555555556	0.7	0.8571428571
**ECSCR**	0.04761904762	0.04761904762	0	0	inf	4.7619E+31
**ECSIT**	0.1428571429	0.1428571429	0.1111111111	0.1111111111	1.083333333	1.285714286
**ECT2**	0.1904761905	0.1904761905	0.05555555556	0.05555555556	3.294117647	3.428571429
**EDAR**	0	0	0.05555555556	0.05555555556	0	0
**DARAD**	0.1428571429	0.1428571429	0	0	inf	1.42857E+32
**EDC3**	0.04761904762	0.04761904762	0.05555555556	0.05555555556	0.7	0.8571428571
**EDC4**	0.1428571429	0.1428571429	0.05555555556	0.05555555556	2.333333333	2.571428571
**EDDM13**	0.1428571429	0.1428571429	0.05555555556	0.05555555556	2.333333333	2.571428571
**EDEM1**	0.04761904762	0.04761904762	0	0	inf	4.7619E+31
**EDEM2**	0.09523809524	0.09523809524	0.1111111111	0.1111111111	0.6842105263	0.8571428571
**EDEM3**	0.1428571429	0.1428571429	0.05555555556	0.05555555556	2.333333333	2.571428571
**EDF1**	0.1428571429	0.1428571429	0.05555555556	0.05555555556	2.333333333	2.571428571
**EDIL3**	0.04761904762	0.04761904762	0.05555555556	0.05555555556	0.7	0.8571428571
**EDN1**	0.04761904762	0.04761904762	0	0	inf	4.7619E+31
**EDN2**	0.04761904762	0.04761904762	0.05555555556	0.05555555556	0.7	0.8571428571
**EDN3**	0.1428571429	0.1428571429	0.1666666667	0.1666666667	0.6666666667	0.8571428571
**EDNRA**	0	0	0.05555555556	0.05555555556	0	0
**EDRF1**	0.2380952381	0.2380952381	0.05555555556	0.05555555556	4.375	4.285714286
**DRF1-AS**	0.2380952381	0.2380952381	0.05555555556	0.05555555556	4.375	4.285714286
**DRF1-D**	0.2380952381	0.2380952381	0.05555555556	0.05555555556	4.375	4.285714286
**EEA1**	0.04761904762	0.04761904762	0.05555555556	0.05555555556	0.7	0.8571428571
**EEF1A1**	0.04761904762	0.04761904762	0	0	inf	4.7619E+31
**EEF1A2**	0.1428571429	0.1428571429	0.2222222222	0.2222222222	0.4583333333	0.6428571429
**F1AKM**	0.04761904762	0.04761904762	0	0	inf	4.7619E+31
**F1AKM**	0.2380952381	0.2380952381	0.05555555556	0.05555555556	4.375	4.285714286
**F1AKM**	0.1428571429	0.1428571429	0.05555555556	0.05555555556	2.333333333	2.571428571
**F1AKM**	0.1428571429	0.1428571429	0.05555555556	0.05555555556	2.333333333	2.571428571

AKMT4-	0.1428571429	0.1428571429	0.05555555556	0.05555555556	2.333333333	2.571428571
F1AKNI	0.1428571429	0.1428571429	0.05555555556	0.05555555556	2.333333333	2.571428571
EEF1B2	0	0	0.05555555556	0.05555555556	0	0
EEF1D	0.04761904762	0.04761904762	0.1666666667	0.1666666667	0.2	0.2857142857
EEF2	0.09523809524	0.09523809524	0.05555555556	0.05555555556	1.473684211	1.714285714
EEF2K	0.04761904762	0.04761904762	0	0	inf	4.7619E+31
EF2KM	0.04761904762	0.04761904762	0	0	inf	4.7619E+31
EEFSEC	0.09523809524	0.09523809524	0.05555555556	0.05555555556	1.473684211	1.714285714
EEPD1	0.04761904762	0.04761904762	0.2222222222	0.2222222222	0.1375	0.2142857143
EFCAB1	0.09523809524	0.09523809524	0.1666666667	0.1666666667	0.4210526316	0.5714285714
EFCAB1	0.04761904762	0.04761904762	0.05555555556	0.05555555556	0.7	0.8571428571
EFCAB1	0.09523809524	0.09523809524	0.05555555556	0.05555555556	1.473684211	1.714285714
EFCAB1	0.1428571429	0.1428571429	0	0	inf	1.42857E+32
EFCAB1	0.04761904762	0.04761904762	0.05555555556	0.05555555556	0.7	0.8571428571
CAB14-A	0.04761904762	0.04761904762	0.05555555556	0.05555555556	0.7	0.8571428571
EFCAB2	0.1428571429	0.1428571429	0.05555555556	0.05555555556	2.333333333	2.571428571
EFCAB3	0.04761904762	0.04761904762	0	0	inf	4.7619E+31
EFCAB5	0	0	0.05555555556	0.05555555556	0	0
EFCAB6	0	0	0.1666666667	0.1666666667	0	0
CAB6-A	0	0	0.1666666667	0.1666666667	0	0
EFCAB7	0.04761904762	0.04761904762	0.05555555556	0.05555555556	0.7	0.8571428571
EFCAB8	0.09523809524	0.09523809524	0.1111111111	0.1111111111	0.6842105263	0.8571428571
EFCAB9	0	0	0.05555555556	0.05555555556	0	0
EFCC1	0.09523809524	0.09523809524	0.05555555556	0.05555555556	1.473684211	1.714285714
EFEMP1	0.04761904762	0.04761904762	0.05555555556	0.05555555556	0.7	0.8571428571
EFHB	0.04761904762	0.04761904762	0	0	inf	4.7619E+31
EFHC1	0.04761904762	0.04761904762	0	0	inf	4.7619E+31
EFHD1	0	0	0.05555555556	0.05555555556	0	0
EFHD2	0.04761904762	0.04761904762	0.1111111111	0.1111111111	0.325	0.4285714286
EFL1	0.04761904762	0.04761904762	0.05555555556	0.05555555556	0.7	0.8571428571
EFL1P1	0.04761904762	0.04761904762	0.05555555556	0.05555555556	0.7	0.8571428571
EFNA1	0.1904761905	0.1904761905	0.1111111111	0.1111111111	1.529411765	1.714285714
EFNA2	0.09523809524	0.09523809524	0.1111111111	0.1111111111	0.6842105263	0.8571428571
EFNA3	0.1904761905	0.1904761905	0.1111111111	0.1111111111	1.529411765	1.714285714
EFNA4	0.1904761905	0.1904761905	0.1111111111	0.1111111111	1.529411765	1.714285714
EFNA5	0.04761904762	0.04761904762	0	0	inf	4.7619E+31
EFNB3	0	0	0.05555555556	0.05555555556	0	0
EFR3A	0.04761904762	0.04761904762	0.1111111111	0.1111111111	0.325	0.4285714286
EFS	0	0	0.05555555556	0.05555555556	0	0
EFTUD2	0.1428571429	0.1428571429	0	0	inf	1.42857E+32
EGF	0	0	0.05555555556	0.05555555556	0	0
GFEM1	0.1428571429	0.1428571429	0.05555555556	0.05555555556	2.333333333	2.571428571
EGFL7	0.1428571429	0.1428571429	0.05555555556	0.05555555556	2.333333333	2.571428571
EGFL8	0.04761904762	0.04761904762	0	0	inf	4.7619E+31
EGFLAM	0.04761904762	0.04761904762	0.05555555556	0.05555555556	0.7	0.8571428571
FLAM-A	0.04761904762	0.04761904762	0.05555555556	0.05555555556	0.7	0.8571428571
FLAM-A	0.04761904762	0.04761904762	0.05555555556	0.05555555556	0.7	0.8571428571
EGFR	0.380952381	0.380952381	0.2222222222	0.2222222222	1.692307692	1.714285714
GFR-AS	0.380952381	0.380952381	0.2222222222	0.2222222222	1.692307692	1.714285714
EGOT	0.04761904762	0.04761904762	0	0	inf	4.7619E+31
EGR1	0.04761904762	0.04761904762	0	0	inf	4.7619E+31

EGR2	0.04761904762	0.04761904762	0.1666666667	0.1666666667	0.2	0.2857142857
EGR3	0.04761904762	0.04761904762	0.1111111111	0.1111111111	0.325	0.4285714286
EGR4	0	0	0.05555555556	0.05555555556	0	0
EHBP1	0	0	0.05555555556	0.05555555556	0	0
EHD2	0.09523809524	0.09523809524	0.1111111111	0.1111111111	0.6842105263	0.8571428571
EHD3	0	0	0.05555555556	0.05555555556	0	0
HHADI	0.1428571429	0.1428571429	0.05555555556	0.05555555556	2.333333333	2.571428571
HADH-A	0.1428571429	0.1428571429	0.05555555556	0.05555555556	2.333333333	2.571428571
EHMT1	0.09523809524	0.09523809524	0	0	inf	9.52381E+31
EHMT2	0.04761904762	0.04761904762	0	0	inf	4.7619E+31
EID1	0.04761904762	0.04761904762	0.05555555556	0.05555555556	0.7	0.8571428571
EID2	0.04761904762	0.04761904762	0.05555555556	0.05555555556	0.7	0.8571428571
EID2B	0.04761904762	0.04761904762	0.05555555556	0.05555555556	0.7	0.8571428571
EID3	0.04761904762	0.04761904762	0.05555555556	0.05555555556	0.7	0.8571428571
EIF1	0.09523809524	0.09523809524	0	0	inf	9.52381E+31
EIF1B	0.04761904762	0.04761904762	0	0	inf	4.7619E+31
IF1B-AS	0.04761904762	0.04761904762	0	0	inf	4.7619E+31
EIF2A	0.1904761905	0.1904761905	0.1111111111	0.1111111111	1.529411765	1.714285714
IF2AK1	0.04761904762	0.04761904762	0.1666666667	0.1666666667	0.2	0.2857142857
IF2AK2	0	0	0.05555555556	0.05555555556	0	0
IF2AK3	0	0	0.05555555556	0.05555555556	0	0
F2AK3-I	0	0	0.05555555556	0.05555555556	0	0
IF2AK4	0	0	0	0		0.00001
EIF2B1	0.04761904762	0.04761904762	0.1111111111	0.1111111111	0.325	0.4285714286
EIF2B3	0.04761904762	0.04761904762	0.05555555556	0.05555555556	0.7	0.8571428571
EIF2B5	0.1428571429	0.1428571429	0.05555555556	0.05555555556	2.333333333	2.571428571
EIF2D	0.1428571429	0.1428571429	0	0	inf	1.42857E+32
EIF2S2	0.09523809524	0.09523809524	0.1111111111	0.1111111111	0.6842105263	0.8571428571
EIF2S3B	0.04761904762	0.04761904762	0.1111111111	0.1111111111	0.325	0.4285714286
EIF3A	0.2380952381	0.2380952381	0	0	inf	2.38095E+32
EIF3B	0.04761904762	0.04761904762	0.1666666667	0.1666666667	0.2	0.2857142857
EIF3C	0.04761904762	0.04761904762	0	0	inf	4.7619E+31
EIF3CL	0.04761904762	0.04761904762	0	0	inf	4.7619E+31
EIF3D	0	0	0.1111111111	0.1111111111	0	0
EIF3E	0.1904761905	0.1904761905	0.1111111111	0.1111111111	1.529411765	1.714285714
EIF3G	0.1428571429	0.1428571429	0.1111111111	0.1111111111	1.083333333	1.285714286
EIF3H	0.1904761905	0.1904761905	0.1666666667	0.1666666667	0.9411764706	1.142857143
EIF3I	0.04761904762	0.04761904762	0.1111111111	0.1111111111	0.325	0.4285714286
EIF3IP1	0.04761904762	0.04761904762	0.05555555556	0.05555555556	0.7	0.8571428571
EIF3K	0.04761904762	0.04761904762	0.05555555556	0.05555555556	0.7	0.8571428571
EIF3L	0	0	0.1111111111	0.1111111111	0	0
EIF3M	0.04761904762	0.04761904762	0	0	inf	4.7619E+31
EIF4A1	0	0	0.05555555556	0.05555555556	0	0
EIF4A2	0.09523809524	0.09523809524	0.05555555556	0.05555555556	1.473684211	1.714285714
EIF4A3	0.04761904762	0.04761904762	0	0	inf	4.7619E+31
EIF4B	0.04761904762	0.04761904762	0.05555555556	0.05555555556	0.7	0.8571428571
EIF4E	0	0	0.05555555556	0.05555555556	0	0
EIF4E1B	0.09523809524	0.09523809524	0.05555555556	0.05555555556	1.473684211	1.714285714
EIF4E2	0	0	0.05555555556	0.05555555556	0	0
EIF4E3	0.04761904762	0.04761904762	0	0	inf	4.7619E+31
IF4EBP	0.09523809524	0.09523809524	0.1111111111	0.1111111111	0.6842105263	0.8571428571

IF4EBP	0.09523809524	0.09523809524	0	0	inf	9.52381E+31
IF4EBP	0.04761904762	0.04761904762	0	0	inf	4.7619E+31
IF4ENIF	0.04761904762	0.04761904762	0.1111111111	0.1111111111	0.325	0.4285714286
EIF4G1	0.1428571429	0.1428571429	0.05555555556	0.05555555556	2.333333333	2.571428571
EIF4G3	0.04761904762	0.04761904762	0.1111111111	0.1111111111	0.325	0.4285714286
EIF4H	0.1428571429	0.1428571429	0.1111111111	0.1111111111	1.083333333	1.285714286
EIF5A	0	0	0.05555555556	0.05555555556	0	0
EIF5A2	0.1904761905	0.1904761905	0.05555555556	0.05555555556	3.294117647	3.428571429
EIF5AL1	0.1428571429	0.1428571429	0.05555555556	0.05555555556	2.333333333	2.571428571
EIF5B	0	0	0.05555555556	0.05555555556	0	0
EIF6	0.09523809524	0.09523809524	0.1111111111	0.1111111111	0.6842105263	0.8571428571
EIPR1	0	0	0.05555555556	0.05555555556	0	0
ELAC1	0.04761904762	0.04761904762	0	0	inf	4.7619E+31
ELAC2	0.04761904762	0.04761904762	0.1111111111	0.1111111111	0.325	0.4285714286
ELANE	0.09523809524	0.09523809524	0.1111111111	0.1111111111	0.6842105263	0.8571428571
ELAVL1	0.1428571429	0.1428571429	0.05555555556	0.05555555556	2.333333333	2.571428571
ELAVL2	0.09523809524	0.09523809524	0.05555555556	0.05555555556	1.473684211	1.714285714
ELAVL3	0.1428571429	0.1428571429	0.1111111111	0.1111111111	1.083333333	1.285714286
ELAVL4	0	0	0	0		0.00001
ELDR	0.380952381	0.380952381	0.2222222222	0.2222222222	1.692307692	1.714285714
ELF2	0.04761904762	0.04761904762	0.05555555556	0.05555555556	0.7	0.8571428571
ELF3	0.1428571429	0.1428571429	0	0	inf	1.42857E+32
LF3-AS	0.1428571429	0.1428571429	0	0	inf	1.42857E+32
ELFN1	0.04761904762	0.04761904762	0.1666666667	0.1666666667	0.2	0.2857142857
LFN1-AS	0.04761904762	0.04761904762	0.1666666667	0.1666666667	0.2	0.2857142857
ELFN2	0	0	0.1111111111	0.1111111111	0	0
ELK3	0.04761904762	0.04761904762	0.05555555556	0.05555555556	0.7	0.8571428571
ELK4	0.1428571429	0.1428571429	0	0	inf	1.42857E+32
ELL	0.1428571429	0.1428571429	0.1111111111	0.1111111111	1.083333333	1.285714286
ELL2	0.04761904762	0.04761904762	0	0	inf	4.7619E+31
ELMO1	0.04761904762	0.04761904762	0.2222222222	0.2222222222	0.1375	0.2142857143
LMO1-A	0.04761904762	0.04761904762	0.2222222222	0.2222222222	0.1375	0.2142857143
ELMO2	0.1428571429	0.1428571429	0.1666666667	0.1666666667	0.6666666667	0.8571428571
ELMO3	0.1428571429	0.1428571429	0.05555555556	0.05555555556	2.333333333	2.571428571
LMOD2	0	0	0.05555555556	0.05555555556	0	0
LMOD3	0	0	0.05555555556	0.05555555556	0	0
LMSAN	0.09523809524	0.09523809524	0	0	inf	9.52381E+31
ELN	0.1428571429	0.1428571429	0.1111111111	0.1111111111	1.083333333	1.285714286
ELOA	0.04761904762	0.04761904762	0.1111111111	0.1111111111	0.325	0.4285714286
LOA-AS	0.04761904762	0.04761904762	0.1111111111	0.1111111111	0.325	0.4285714286
ELOA2	0.04761904762	0.04761904762	0.05555555556	0.05555555556	0.7	0.8571428571
ELOA3	0.04761904762	0.04761904762	0.05555555556	0.05555555556	0.7	0.8571428571
ELOA3B	0.04761904762	0.04761904762	0.05555555556	0.05555555556	0.7	0.8571428571
ELOA3C	0.04761904762	0.04761904762	0.05555555556	0.05555555556	0.7	0.8571428571
ELOA3D	0.04761904762	0.04761904762	0.05555555556	0.05555555556	0.7	0.8571428571
ELOB	0.04761904762	0.04761904762	0.1111111111	0.1111111111	0.325	0.4285714286
ELOC	0.1904761905	0.1904761905	0.1111111111	0.1111111111	1.529411765	1.714285714
ELOF1	0.1428571429	0.1428571429	0.1111111111	0.1111111111	1.083333333	1.285714286
ELOVL1	0.04761904762	0.04761904762	0.05555555556	0.05555555556	0.7	0.8571428571
ELOVL2	0.04761904762	0.04761904762	0	0	inf	4.7619E+31
OVL2-A	0.04761904762	0.04761904762	0	0	inf	4.7619E+31

ELOVL3	0.1428571429	0.1428571429	0.05555555556	0.05555555556	2.333333333	2.571428571
ELOVL5	0.04761904762	0.04761904762	0	0	inf	4.7619E+31
ELOVL6	0	0	0.05555555556	0.05555555556	0	0
ELOVL7	0.04761904762	0.04761904762	0	0	inf	4.7619E+31
ELP1	0.04761904762	0.04761904762	0.05555555556	0.05555555556	0.7	0.8571428571
ELP2	0.04761904762	0.04761904762	0	0	inf	4.7619E+31
ELP3	0.04761904762	0.04761904762	0.1111111111	0.1111111111	0.325	0.4285714286
ELP5	0	0	0.05555555556	0.05555555556	0	0
ELP6	0.09523809524	0.09523809524	0	0	inf	9.52381E+31
ELSPBP1	0.09523809524	0.09523809524	0.1111111111	0.1111111111	0.6842105263	0.8571428571
EMB	0.04761904762	0.04761904762	0.05555555556	0.05555555556	0.7	0.8571428571
EMBP1	0.04761904762	0.04761904762	0.1111111111	0.1111111111	0.325	0.4285714286
EMC1	0.04761904762	0.04761904762	0.1111111111	0.1111111111	0.325	0.4285714286
EMC1-AS	0.04761904762	0.04761904762	0.1111111111	0.1111111111	0.325	0.4285714286
EMC10	0.09523809524	0.09523809524	0.1111111111	0.1111111111	0.6842105263	0.8571428571
EMC2	0.1904761905	0.1904761905	0.1111111111	0.1111111111	1.529411765	1.714285714
EMC3	0.09523809524	0.09523809524	0	0	inf	9.52381E+31
EMC3-AS	0.09523809524	0.09523809524	0	0	inf	9.52381E+31
EMC6	0	0	0.1111111111	0.1111111111	0	0
EMC8	0.04761904762	0.04761904762	0	0	inf	4.7619E+31
EMCN	0	0	0.05555555556	0.05555555556	0	0
EME1	0.09523809524	0.09523809524	0	0	inf	9.52381E+31
EME2	0.09523809524	0.09523809524	0.2222222222	0.2222222222	0.2894736842	0.4285714286
EMG1	0.04761904762	0.04761904762	0.1111111111	0.1111111111	0.325	0.4285714286
EMID1	0.04761904762	0.04761904762	0.1111111111	0.1111111111	0.325	0.4285714286
EMILIN2	0.09523809524	0.09523809524	0	0	inf	9.52381E+31
EMILIN3	0.1428571429	0.1428571429	0.1666666667	0.1666666667	0.6666666667	0.8571428571
EML2	0.09523809524	0.09523809524	0.1111111111	0.1111111111	0.6842105263	0.8571428571
EML2-AS	0.09523809524	0.09523809524	0.1111111111	0.1111111111	0.6842105263	0.8571428571
EML4	0	0	0.05555555556	0.05555555556	0	0
EML6	0.04761904762	0.04761904762	0.05555555556	0.05555555556	0.7	0.8571428571
EMP1	0.04761904762	0.04761904762	0.05555555556	0.05555555556	0.7	0.8571428571
EMP2	0.04761904762	0.04761904762	0	0	inf	4.7619E+31
EMP3	0.09523809524	0.09523809524	0.1111111111	0.1111111111	0.6842105263	0.8571428571
EMX1	0	0	0.05555555556	0.05555555556	0	0
EMX2	0.2380952381	0.2380952381	0	0	inf	2.38095E+32
EMX2OS	0.2380952381	0.2380952381	0	0	inf	2.38095E+32
EN1	0	0	0.05555555556	0.05555555556	0	0
EN2	0.1428571429	0.1428571429	0.05555555556	0.05555555556	2.333333333	2.571428571
ENAH	0.1428571429	0.1428571429	0.05555555556	0.05555555556	2.333333333	2.571428571
ENAM	0.09523809524	0.09523809524	0.05555555556	0.05555555556	1.473684211	1.714285714
ENC1	0.04761904762	0.04761904762	0.05555555556	0.05555555556	0.7	0.8571428571
ENDOG	0.09523809524	0.09523809524	0.05555555556	0.05555555556	1.473684211	1.714285714
ENDOU	0.04761904762	0.04761904762	0.05555555556	0.05555555556	0.7	0.8571428571
ENDOV	0.04761904762	0.04761904762	0	0	inf	4.7619E+31
ENG	0.09523809524	0.09523809524	0.05555555556	0.05555555556	1.473684211	1.714285714
ENGASE	0.04761904762	0.04761904762	0	0	inf	4.7619E+31
ENHO	0.09523809524	0.09523809524	0.05555555556	0.05555555556	1.473684211	1.714285714
ENKD1	0.1428571429	0.1428571429	0.05555555556	0.05555555556	2.333333333	2.571428571
ENKUR	0.09523809524	0.09523809524	0.1111111111	0.1111111111	0.6842105263	0.8571428571
ENO1	0	0	0.05555555556	0.05555555556	0	0

NO1-AS	0	0	0.05555555556	0.05555555556	0	0
ENO2	0.04761904762	0.04761904762	0.1111111111	0.1111111111	0.325	0.4285714286
ENO3	0	0	0.1111111111	0.1111111111	0	0
ENO4	0.2380952381	0.2380952381	0	0	inf	2.38095E+32
ENOPH1	0	0	0.05555555556	0.05555555556	0	0
ENOSF1	0.09523809524	0.09523809524	0	0	inf	9.52381E+31
ENPEP	0	0	0.05555555556	0.05555555556	0	0
ENPP2	0.09523809524	0.09523809524	0.1666666667	0.1666666667	0.4210526316	0.5714285714
ENPP4	0.04761904762	0.04761904762	0.05555555556	0.05555555556	0.7	0.8571428571
ENPP5	0.04761904762	0.04761904762	0.05555555556	0.05555555556	0.7	0.8571428571
ENPP6	0	0	0.05555555556	0.05555555556	0	0
ENPP7	0.04761904762	0.04761904762	0	0	inf	4.7619E+31
NPP7P1	0.1428571429	0.1428571429	0	0	inf	1.42857E+32
ENSA	0.1904761905	0.1904761905	0.05555555556	0.05555555556	3.294117647	3.428571429
ENTHD1	0	0	0.1111111111	0.1111111111	0	0
ENTPD1	0.1428571429	0.1428571429	0	0	inf	1.42857E+32
TPD1-A	0.1428571429	0.1428571429	0	0	inf	1.42857E+32
ENTPD2	0.1428571429	0.1428571429	0.05555555556	0.05555555556	2.333333333	2.571428571
ENTPD3	0.04761904762	0.04761904762	0	0	inf	4.7619E+31
TPD3-A	0.04761904762	0.04761904762	0	0	inf	4.7619E+31
ENTPD4	0.04761904762	0.04761904762	0.1111111111	0.1111111111	0.325	0.4285714286
ENTPD5	0.09523809524	0.09523809524	0	0	inf	9.52381E+31
ENTPD6	0.1904761905	0.1904761905	0.05555555556	0.05555555556	3.294117647	3.428571429
ENTPD7	0.1428571429	0.1428571429	0	0	inf	1.42857E+32
ENTPD8	0.09523809524	0.09523809524	0	0	inf	9.52381E+31
ENTR1	0.1428571429	0.1428571429	0.05555555556	0.05555555556	2.333333333	2.571428571
ENY2	0.1904761905	0.1904761905	0.1111111111	0.1111111111	1.529411765	1.714285714
EOGT	0.04761904762	0.04761904762	0	0	inf	4.7619E+31
EOMES	0.04761904762	0.04761904762	0	0	inf	4.7619E+31
EP300	0	0	0.1111111111	0.1111111111	0	0
P300-AS	0	0	0.1111111111	0.1111111111	0	0
EP400	0.09523809524	0.09523809524	0.05555555556	0.05555555556	1.473684211	1.714285714
EP400P1	0.09523809524	0.09523809524	0.05555555556	0.05555555556	1.473684211	1.714285714
EPAS1	0	0	0.05555555556	0.05555555556	0	0
EPB41	0.04761904762	0.04761904762	0.1111111111	0.1111111111	0.325	0.4285714286
EPB41L1	0.09523809524	0.09523809524	0.1111111111	0.1111111111	0.6842105263	0.8571428571
EPB41L3	0.09523809524	0.09523809524	0	0	inf	9.52381E+31
PB41L4	0.04761904762	0.04761904762	0	0	inf	4.7619E+31
341L4A-	0.04761904762	0.04761904762	0	0	inf	4.7619E+31
341L4A-	0.04761904762	0.04761904762	0	0	inf	4.7619E+31
PB41L4	0.04761904762	0.04761904762	0.05555555556	0.05555555556	0.7	0.8571428571
EPB41L5	0	0	0.05555555556	0.05555555556	0	0
EPC1	0.09523809524	0.09523809524	0.2222222222	0.2222222222	0.2894736842	0.4285714286
EPC2	0	0	0.05555555556	0.05555555556	0	0
EPCAM	0	0	0.05555555556	0.05555555556	0	0
PCAM-D	0	0	0.05555555556	0.05555555556	0	0
EPDR1	0.04761904762	0.04761904762	0.2222222222	0.2222222222	0.1375	0.2142857143
EPG5	0.04761904762	0.04761904762	0.05555555556	0.05555555556	0.7	0.8571428571
EPGN	0.09523809524	0.09523809524	0.05555555556	0.05555555556	1.473684211	1.714285714
EPHA1	0.04761904762	0.04761904762	0.05555555556	0.05555555556	0.7	0.8571428571
PHA1-AS	0.04761904762	0.04761904762	0.05555555556	0.05555555556	0.7	0.8571428571

**EPHA10**	0.04761904762	0.04761904762	0.05555555556	0.05555555556	0.7	0.8571428571
**EPHA2**	0.04761904762	0.04761904762	0.1111111111	0.1111111111	0.325	0.4285714286
**EPHA3**	0.04761904762	0.04761904762	0	0	inf	4.7619E+31
**EPHA4**	0	0	0.05555555556	0.05555555556	0	0
**EPHA5**	0.04761904762	0.04761904762	0.05555555556	0.05555555556	0.7	0.8571428571
**PHA5-AS**	0.04761904762	0.04761904762	0.05555555556	0.05555555556	0.7	0.8571428571
**EPHA6**	0.04761904762	0.04761904762	0.1111111111	0.1111111111	0.325	0.4285714286
**EPHA7**	0	0	0	0		0.00001
**EPHA8**	0.04761904762	0.04761904762	0.1111111111	0.1111111111	0.325	0.4285714286
**EPHB1**	0.1428571429	0.1428571429	0	0	inf	1.42857E+32
**EPHB2**	0.04761904762	0.04761904762	0.1111111111	0.1111111111	0.325	0.4285714286
**EPHB3**	0.1428571429	0.1428571429	0.05555555556	0.05555555556	2.333333333	2.571428571
**EPHB4**	0.04761904762	0.04761904762	0.05555555556	0.05555555556	0.7	0.8571428571
**EPHB6**	0.04761904762	0.04761904762	0.05555555556	0.05555555556	0.7	0.8571428571
**EPHX1**	0.1428571429	0.1428571429	0.05555555556	0.05555555556	2.333333333	2.571428571
**EPHX2**	0.04761904762	0.04761904762	0.1111111111	0.1111111111	0.325	0.4285714286
**EPHX3**	0.1428571429	0.1428571429	0.1111111111	0.1111111111	1.083333333	1.285714286
**EPHX4**	0.04761904762	0.04761904762	0.05555555556	0.05555555556	0.7	0.8571428571
**PM2AIP**	0.04761904762	0.04761904762	0	0	inf	4.7619E+31
**EPN1**	0.09523809524	0.09523809524	0.05555555556	0.05555555556	1.473684211	1.714285714
**EPN2**	0.04761904762	0.04761904762	0.1111111111	0.1111111111	0.325	0.4285714286
**PN2-AS**	0.04761904762	0.04761904762	0.1111111111	0.1111111111	0.325	0.4285714286
**PN2-IT**	0.04761904762	0.04761904762	0.1111111111	0.1111111111	0.325	0.4285714286
**EPN3**	0.09523809524	0.09523809524	0	0	inf	9.52381E+31
**EPO**	0.04761904762	0.04761904762	0.05555555556	0.05555555556	0.7	0.8571428571
**EPOP**	0.04761904762	0.04761904762	0	0	inf	4.7619E+31
**EPPIN**	0.1428571429	0.1428571429	0.1666666667	0.1666666667	0.6666666667	0.8571428571
**PIN-WFI**	0.1428571429	0.1428571429	0.1666666667	0.1666666667	0.6666666667	0.8571428571
**EPPK1**	0.04761904762	0.04761904762	0.1666666667	0.1666666667	0.2	0.2857142857
**EPRS**	0.1428571429	0.1428571429	0.05555555556	0.05555555556	2.333333333	2.571428571
**EPS15**	0.04761904762	0.04761904762	0.05555555556	0.05555555556	0.7	0.8571428571
**EPS15L1**	0.1428571429	0.1428571429	0.1111111111	0.1111111111	1.083333333	1.285714286
**EPS8**	0.04761904762	0.04761904762	0.1111111111	0.1111111111	0.325	0.4285714286
**EPS8L1**	0.09523809524	0.09523809524	0.05555555556	0.05555555556	1.473684211	1.714285714
**EPS8L2**	0.04761904762	0.04761904762	0	0	inf	4.7619E+31
**EPS8L3**	0.04761904762	0.04761904762	0.05555555556	0.05555555556	0.7	0.8571428571
**EPX**	0.09523809524	0.09523809524	0	0	inf	9.52381E+31
**EPYC**	0.04761904762	0.04761904762	0.05555555556	0.05555555556	0.7	0.8571428571
**EQTN**	0.04761904762	0.04761904762	0.05555555556	0.05555555556	0.7	0.8571428571
**ERAL1**	0.04761904762	0.04761904762	0.05555555556	0.05555555556	0.7	0.8571428571
**ERAP1**	0.04761904762	0.04761904762	0	0	inf	4.7619E+31
**ERAP2**	0.04761904762	0.04761904762	0	0	inf	4.7619E+31
**ERBB2**	0.09523809524	0.09523809524	0	0	inf	9.52381E+31
**ERBB3**	0.09523809524	0.09523809524	0.05555555556	0.05555555556	1.473684211	1.714285714
**ERBB4**	0	0	0.05555555556	0.05555555556	0	0
**ERBIN**	0.04761904762	0.04761904762	0	0	inf	4.7619E+31
**ERC1**	0.04761904762	0.04761904762	0.1111111111	0.1111111111	0.325	0.4285714286
**ERC2**	0.04761904762	0.04761904762	0	0	inf	4.7619E+31
**RC2-IT**	0.04761904762	0.04761904762	0	0	inf	4.7619E+31
**ERCC1**	0.09523809524	0.09523809524	0.1111111111	0.1111111111	0.6842105263	0.8571428571
**ERCC2**	0.09523809524	0.09523809524	0.1111111111	0.1111111111	0.6842105263	0.8571428571

ERCC3	0	0	0.05555555556	0.05555555556	0	0
ERCC4	0.04761904762	0.04761904762	0	0	inf	4.7619E+31
ERCC6	0.09523809524	0.09523809524	0.05555555556	0.05555555556	1.473684211	1.714285714
RCC6L	0.04761904762	0.04761904762	0.05555555556	0.05555555556	0.7	0.8571428571
ERCC8	0.04761904762	0.04761904762	0	0	inf	4.7619E+31
EREG	0.09523809524	0.09523809524	0.05555555556	0.05555555556	1.473684211	1.714285714
ERF	0.04761904762	0.04761904762	0.05555555556	0.05555555556	0.7	0.8571428571
ERFE	0	0	0.05555555556	0.05555555556	0	0
ERG	0.04761904762	0.04761904762	0.1111111111	0.1111111111	0.325	0.4285714286
ERGIC1	0	0	0.05555555556	0.05555555556	0	0
ERGIC2	0.04761904762	0.04761904762	0.05555555556	0.05555555556	0.7	0.8571428571
ERGIC3	0.09523809524	0.09523809524	0.1111111111	0.1111111111	0.6842105263	0.8571428571
ERI1	0.09523809524	0.09523809524	0.1111111111	0.1111111111	0.6842105263	0.8571428571
ERI2	0.04761904762	0.04761904762	0	0	inf	4.7619E+31
ERI3	0.04761904762	0.04761904762	0.05555555556	0.05555555556	0.7	0.8571428571
RI3-IT1	0.04761904762	0.04761904762	0.05555555556	0.05555555556	0.7	0.8571428571
ERICH1	0.09523809524	0.09523809524	0.1111111111	0.1111111111	0.6842105263	0.8571428571
ERICH2	0	0	0.05555555556	0.05555555556	0	0
ERICH3	0.04761904762	0.04761904762	0.05555555556	0.05555555556	0.7	0.8571428571
RICH3-A	0.04761904762	0.04761904762	0.05555555556	0.05555555556	0.7	0.8571428571
ERICH4	0.04761904762	0.04761904762	0.05555555556	0.05555555556	0.7	0.8571428571
ERICH5	0.1904761905	0.1904761905	0.1111111111	0.1111111111	1.529411765	1.714285714
ERICH6	0.1904761905	0.1904761905	0.1111111111	0.1111111111	1.529411765	1.714285714
RICH6-A	0.1904761905	0.1904761905	0.1111111111	0.1111111111	1.529411765	1.714285714
ERLEC1	0.04761904762	0.04761904762	0.05555555556	0.05555555556	0.7	0.8571428571
ERLIN1	0.1428571429	0.1428571429	0	0	inf	1.42857E+32
ERLIN2	0.04761904762	0.04761904762	0.1111111111	0.1111111111	0.325	0.4285714286
RLNC1	0.2380952381	0.2380952381	0	0	inf	2.38095E+32
ERMAP	0.04761904762	0.04761904762	0.05555555556	0.05555555556	0.7	0.8571428571
ERMN	0	0	0.05555555556	0.05555555556	0	0
ERMP1	0.04761904762	0.04761904762	0.05555555556	0.05555555556	0.7	0.8571428571
ERN1	0.04761904762	0.04761904762	0	0	inf	4.7619E+31
ERN2	0.04761904762	0.04761904762	0	0	inf	4.7619E+31
ERO1A	0.04761904762	0.04761904762	0	0	inf	4.7619E+31
ERO1B	0.1428571429	0.1428571429	0	0	inf	1.42857E+32
ERP27	0.04761904762	0.04761904762	0.1111111111	0.1111111111	0.325	0.4285714286
ERP29	0	0	0.1111111111	0.1111111111	0	0
ERP44	0.04761904762	0.04761904762	0	0	inf	4.7619E+31
ERRFI1	0	0	0.05555555556	0.05555555556	0	0
ERV3-1	0.04761904762	0.04761904762	0.1111111111	0.1111111111	0.325	0.4285714286
3-1-ZNI	0.04761904762	0.04761904762	0.1111111111	0.1111111111	0.325	0.4285714286
RVFRD-	0.04761904762	0.04761904762	0	0	inf	4.7619E+31
RVH48-	0.04761904762	0.04761904762	0.1666666667	0.1666666667	0.2	0.2857142857
RVK13-	0.04761904762	0.04761904762	0.1111111111	0.1111111111	0.325	0.4285714286
RVK3-1	0.1428571429	0.1428571429	0.05555555556	0.05555555556	2.333333333	2.571428571
VMER3	0.2380952381	0.2380952381	0.05555555556	0.05555555556	4.375	4.285714286
ERVV-1	0.09523809524	0.09523809524	0.05555555556	0.05555555556	1.473684211	1.714285714
ERVV-2	0.09523809524	0.09523809524	0.05555555556	0.05555555556	1.473684211	1.714285714
ERVW-1	0.04761904762	0.04761904762	0.1111111111	0.1111111111	0.325	0.4285714286
ESCO1	0.04761904762	0.04761904762	0.1111111111	0.1111111111	0.325	0.4285714286
ESCO2	0.04761904762	0.04761904762	0.1111111111	0.1111111111	0.325	0.4285714286

**ESF1**	0.1904761905	0.1904761905	0.1111111111	0.1111111111	1.529411765	1.714285714
**ESM1**	0.04761904762	0.04761904762	0.05555555556	0.05555555556	0.7	0.8571428571
**ESPL1**	0.04761904762	0.04761904762	0.05555555556	0.05555555556	0.7	0.8571428571
**ESPN**	0	0	0.05555555556	0.05555555556	0	0
**ESPNL**	0	0	0.05555555556	0.05555555556	0	0
**ESPNP**	0.04761904762	0.04761904762	0.1111111111	0.1111111111	0.325	0.4285714286
**ESR2**	0.09523809524	0.09523809524	0	0	inf	9.52381E+31
**ESRG**	0.04761904762	0.04761904762	0	0	inf	4.7619E+31
**ESRP1**	0.1904761905	0.1904761905	0.1111111111	0.1111111111	1.529411765	1.714285714
**ESRP2**	0.1428571429	0.1428571429	0.05555555556	0.05555555556	2.333333333	2.571428571
**ESRRG**	0.1428571429	0.1428571429	0	0	inf	1.42857E+32
**ESS2**	0.04761904762	0.04761904762	0.05555555556	0.05555555556	0.7	0.8571428571
**ESYT1**	0.09523809524	0.09523809524	0.05555555556	0.05555555556	1.473684211	1.714285714
**ESYT2**	0.1428571429	0.1428571429	0.05555555556	0.05555555556	2.333333333	2.571428571
**ESYT3**	0.1428571429	0.1428571429	0.05555555556	0.05555555556	2.333333333	2.571428571
**ETAA1**	0	0	0.05555555556	0.05555555556	0	0
**ETF1**	0.04761904762	0.04761904762	0	0	inf	4.7619E+31
**ETFA**	0.04761904762	0.04761904762	0.05555555556	0.05555555556	0.7	0.8571428571
**TFBKM**	0.1428571429	0.1428571429	0.1111111111	0.1111111111	1.083333333	1.285714286
**ETFDH**	0	0	0.05555555556	0.05555555556	0	0
**ETFRF1**	0.04761904762	0.04761904762	0.05555555556	0.05555555556	0.7	0.8571428571
**ETHE1**	0.09523809524	0.09523809524	0.05555555556	0.05555555556	1.473684211	1.714285714
**ETNK1**	0.04761904762	0.04761904762	0.05555555556	0.05555555556	0.7	0.8571428571
**ETNK2**	0.2380952381	0.2380952381	0	0	inf	2.38095E+32
**ETNPPL**	0	0	0.05555555556	0.05555555556	0	0
**ETS2**	0.04761904762	0.04761904762	0.1111111111	0.1111111111	0.325	0.4285714286
**ETV1**	0.04761904762	0.04761904762	0.2222222222	0.2222222222	0.1375	0.2142857143
**ETV2**	0.04761904762	0.04761904762	0.05555555556	0.05555555556	0.7	0.8571428571
**ETV3**	0.1428571429	0.1428571429	0.05555555556	0.05555555556	2.333333333	2.571428571
**ETV3L**	0.1428571429	0.1428571429	0.05555555556	0.05555555556	2.333333333	2.571428571
**ETV4**	0.04761904762	0.04761904762	0	0	inf	4.7619E+31
**ETV5**	0.09523809524	0.09523809524	0.05555555556	0.05555555556	1.473684211	1.714285714
**ETV6**	0.04761904762	0.04761904762	0.1111111111	0.1111111111	0.325	0.4285714286
**ETV7**	0.04761904762	0.04761904762	0.05555555556	0.05555555556	0.7	0.8571428571
**EVA1A**	0	0	0.05555555556	0.05555555556	0	0
**EVA1B**	0.04761904762	0.04761904762	0.05555555556	0.05555555556	0.7	0.8571428571
**EVA1C**	0.04761904762	0.04761904762	0.1111111111	0.1111111111	0.325	0.4285714286
**EVI2A**	0	0	0	0		0.00001
**EVI2B**	0	0	0	0		0.00001
**EVI5**	0.04761904762	0.04761904762	0.05555555556	0.05555555556	0.7	0.8571428571
**EVI5L**	0.1428571429	0.1428571429	0.05555555556	0.05555555556	2.333333333	2.571428571
**EVPL**	0.04761904762	0.04761904762	0	0	inf	4.7619E+31
**EVPLL**	0.04761904762	0.04761904762	0.1111111111	0.1111111111	0.325	0.4285714286
**EVX1**	0.04761904762	0.04761904762	0.2222222222	0.2222222222	0.1375	0.2142857143
**VX1-AS**	0.04761904762	0.04761904762	0.2222222222	0.2222222222	0.1375	0.2142857143
**EVX2**	0	0	0.1111111111	0.1111111111	0	0
**WSAT1**	0.04761904762	0.04761904762	0.05555555556	0.05555555556	0.7	0.8571428571
**EWSR1**	0.04761904762	0.04761904762	0.1111111111	0.1111111111	0.325	0.4285714286
**EXD3**	0.1428571429	0.1428571429	0.05555555556	0.05555555556	2.333333333	2.571428571
**EXO1**	0.1428571429	0.1428571429	0	0	inf	1.42857E+32
**EXO5**	0.04761904762	0.04761904762	0.05555555556	0.05555555556	0.7	0.8571428571

EXOC1	0.1904761905	0.1904761905	0.05555555556	0.05555555556	3.294117647	3.428571429
EXOC1L	0.1904761905	0.1904761905	0.05555555556	0.05555555556	3.294117647	3.428571429
EXOC3	0	0	0.05555555556	0.05555555556	0	0
XOC3-A	0	0	0.05555555556	0.05555555556	0	0
XOC3L	0.1428571429	0.1428571429	0.05555555556	0.05555555556	2.333333333	2.571428571
XOC3L	0.09523809524	0.09523809524	0.1111111111	0.1111111111	0.6842105263	0.8571428571
EXOC4	0.04761904762	0.04761904762	0.05555555556	0.05555555556	0.7	0.8571428571
EXOC5	0.04761904762	0.04761904762	0	0	inf	4.7619E+31
EXOC6	0.1428571429	0.1428571429	0.1111111111	0.1111111111	1.083333333	1.285714286
EXOC6B	0	0	0.05555555556	0.05555555556	0	0
EXOC7	0.04761904762	0.04761904762	0	0	inf	4.7619E+31
EXOC8	0.1428571429	0.1428571429	0	0	inf	1.42857E+32
EXOG	0.04761904762	0.04761904762	0	0	inf	4.7619E+31
EXOSC1	0.1428571429	0.1428571429	0	0	inf	1.42857E+32
XOSC10	0.04761904762	0.04761904762	0.1111111111	0.1111111111	0.325	0.4285714286
OSC10-A	0.04761904762	0.04761904762	0.1111111111	0.1111111111	0.325	0.4285714286
EXOSC2	0.09523809524	0.09523809524	0.05555555556	0.05555555556	1.473684211	1.714285714
EXOSC3	0.09523809524	0.09523809524	0.05555555556	0.05555555556	1.473684211	1.714285714
EXOSC4	0.04761904762	0.04761904762	0.1666666667	0.1666666667	0.2	0.2857142857
EXOSC5	0.04761904762	0.04761904762	0.05555555556	0.05555555556	0.7	0.8571428571
EXOSC6	0.1428571429	0.1428571429	0.05555555556	0.05555555556	2.333333333	2.571428571
EXOSC9	0	0	0.05555555556	0.05555555556	0	0
EXT1	0.1428571429	0.1428571429	0.1666666667	0.1666666667	0.6666666667	0.8571428571
EXTL1	0.04761904762	0.04761904762	0.1111111111	0.1111111111	0.325	0.4285714286
EXTL2	0.04761904762	0.04761904762	0.05555555556	0.05555555556	0.7	0.8571428571
EXTL3	0.04761904762	0.04761904762	0.1111111111	0.1111111111	0.325	0.4285714286
XTL3-AS	0.04761904762	0.04761904762	0.1111111111	0.1111111111	0.325	0.4285714286
EYA1	0.1904761905	0.1904761905	0.1666666667	0.1666666667	0.9411764706	1.142857143
EYA2	0.1428571429	0.1428571429	0.1666666667	0.1666666667	0.6666666667	0.8571428571
EYA3	0.04761904762	0.04761904762	0.1111111111	0.1111111111	0.325	0.4285714286
EZH1	0.04761904762	0.04761904762	0	0	inf	4.7619E+31
EZH2	0.09523809524	0.09523809524	0.05555555556	0.05555555556	1.473684211	1.714285714
F11	0	0	0.05555555556	0.05555555556	0	0
F11-AS1	0	0	0.05555555556	0.05555555556	0	0
F11R	0.1428571429	0.1428571429	0	0	inf	1.42857E+32
F12	0.09523809524	0.09523809524	0.05555555556	0.05555555556	1.473684211	1.714285714
F13B	0.1428571429	0.1428571429	0	0	inf	1.42857E+32
F2	0	0	0.05555555556	0.05555555556	0	0
F2R	0.04761904762	0.04761904762	0	0	inf	4.7619E+31
F2RL1	0.04761904762	0.04761904762	0	0	inf	4.7619E+31
F2RL2	0.04761904762	0.04761904762	0	0	inf	4.7619E+31
F2RL3	0.1428571429	0.1428571429	0.1111111111	0.1111111111	1.083333333	1.285714286
F3	0.04761904762	0.04761904762	0.05555555556	0.05555555556	0.7	0.8571428571
F5	0.1428571429	0.1428571429	0	0	inf	1.42857E+32
FA2H	0.04761904762	0.04761904762	0	0	inf	4.7619E+31
FAAH	0.04761904762	0.04761904762	0.05555555556	0.05555555556	0.7	0.8571428571
FAAHP1	0.04761904762	0.04761904762	0.05555555556	0.05555555556	0.7	0.8571428571
FAAP10	0.04761904762	0.04761904762	0	0	inf	4.7619E+31
FAAP20	0	0	0.1111111111	0.1111111111	0	0
FAAP24	0.04761904762	0.04761904762	0.05555555556	0.05555555556	0.7	0.8571428571
FABP1	0	0	0.05555555556	0.05555555556	0	0

**FABP12**	0.1428571429	0.1428571429	0.1111111111	0.1111111111	1.083333333	1.285714286
**FABP2**	0	0	0.05555555556	0.05555555556	0	0
**FABP3**	0.04761904762	0.04761904762	0.1111111111	0.1111111111	0.325	0.4285714286
**FABP4**	0.1428571429	0.1428571429	0.1111111111	0.1111111111	1.083333333	1.285714286
**FABP5**	0.1428571429	0.1428571429	0.1111111111	0.1111111111	1.083333333	1.285714286
**FABP5P3**	0.09523809524	0.09523809524	0.05555555556	0.05555555556	1.473684211	1.714285714
**FABP6**	0.04761904762	0.04761904762	0.05555555556	0.05555555556	0.7	0.8571428571
**FABP9**	0.1428571429	0.1428571429	0.1111111111	0.1111111111	1.083333333	1.285714286
**FADS6**	0.04761904762	0.04761904762	0	0	inf	4.7619E+31
**FAF1**	0.04761904762	0.04761904762	0.05555555556	0.05555555556	0.7	0.8571428571
**FAF2**	0.09523809524	0.09523809524	0.05555555556	0.05555555556	1.473684211	1.714285714
**FAH**	0.04761904762	0.04761904762	0.05555555556	0.05555555556	0.7	0.8571428571
**FAHD1**	0.09523809524	0.09523809524	0.2222222222	0.2222222222	0.2894736842	0.4285714286
**FAHD2A**	0	0	0.1111111111	0.1111111111	0	0
**FAHD2B**	0	0	0.1111111111	0.1111111111	0	0
**AHD2C**	0	0	0.1111111111	0.1111111111	0	0
**FAIM**	0.1428571429	0.1428571429	0.05555555556	0.05555555556	2.333333333	2.571428571
**FAIM2**	0.04761904762	0.04761904762	0.05555555556	0.05555555556	0.7	0.8571428571
**FALEC**	0.1904761905	0.1904761905	0.05555555556	0.05555555556	3.294117647	3.428571429
**AM102A**	0.09523809524	0.09523809524	0.05555555556	0.05555555556	1.473684211	1.714285714
**AM102B**	0.04761904762	0.04761904762	0.05555555556	0.05555555556	0.7	0.8571428571
**AM104A**	0.04761904762	0.04761904762	0	0	inf	4.7619E+31
**AM106A**	0.04761904762	0.04761904762	0.1111111111	0.1111111111	0.325	0.4285714286
**AM106B**	0.09523809524	0.09523809524	0.05555555556	0.05555555556	1.473684211	1.714285714
**AM106C**	0.04761904762	0.04761904762	0.05555555556	0.05555555556	0.7	0.8571428571
**AM107A**	0.04761904762	0.04761904762	0	0	inf	4.7619E+31
**AM107B**	0.09523809524	0.09523809524	0.1111111111	0.1111111111	0.6842105263	0.8571428571
**AM110A**	0.1904761905	0.1904761905	0.05555555556	0.05555555556	3.294117647	3.428571429
**AM110B**	0.1428571429	0.1428571429	0.1666666667	0.1666666667	0.6666666667	0.8571428571
**AM110C**	0	0	0.05555555556	0.05555555556	0	0
**AM110D**	0.04761904762	0.04761904762	0.1111111111	0.1111111111	0.325	0.4285714286
**AM114A**	0.1904761905	0.1904761905	0.05555555556	0.05555555556	3.294117647	3.428571429
**AM117A**	0.09523809524	0.09523809524	0	0	inf	9.52381E+31
**AM117B**	0.09523809524	0.09523809524	0.05555555556	0.05555555556	1.473684211	1.714285714
**AM118A**	0	0	0.1111111111	0.1111111111	0	0
**AM120A**	0.04761904762	0.04761904762	0.05555555556	0.05555555556	0.7	0.8571428571
**AM120AO**	0.04761904762	0.04761904762	0.05555555556	0.05555555556	0.7	0.8571428571
**AM122A**	0	0	0.05555555556	0.05555555556	0	0
**AM124B**	0	0	0.05555555556	0.05555555556	0	0
**AM126A**	0.04761904762	0.04761904762	0.1666666667	0.1666666667	0.2	0.2857142857
**AM126B**	0	0	0.05555555556	0.05555555556	0	0
**AM129A**	0.1428571429	0.1428571429	0.05555555556	0.05555555556	2.333333333	2.571428571
**AM129B**	0.09523809524	0.09523809524	0.05555555556	0.05555555556	1.473684211	1.714285714
**AM131A**	0.1428571429	0.1428571429	0.05555555556	0.05555555556	2.333333333	2.571428571
**AM131B**	0.04761904762	0.04761904762	0.05555555556	0.05555555556	0.7	0.8571428571
**AM131C**	0.04761904762	0.04761904762	0.1111111111	0.1111111111	0.325	0.4285714286
**AM133B**	0.04761904762	0.04761904762	0.1111111111	0.1111111111	0.325	0.4285714286
**AM133C**	0.09523809524	0.09523809524	0.05555555556	0.05555555556	1.473684211	1.714285714
**AM133D**	0	0	0.05555555556	0.05555555556	0	0
**AM135B**	0.04761904762	0.04761904762	0.1111111111	0.1111111111	0.325	0.4285714286
**AM136A**	0	0	0.05555555556	0.05555555556	0	0

AM138A	0.1428571429	0.1428571429	0.2222222222	0.2222222222	0.4583333333	0.6428571429
AM138I	0	0	0.05555555556	0.05555555556	0	0
AM138C	0.1428571429	0.1428571429	0.2222222222	0.2222222222	0.4583333333	0.6428571429
AM138I	0.04761904762	0.04761904762	0.1111111111	0.1111111111	0.325	0.4285714286
AM138I	0.04761904762	0.04761904762	0.05555555556	0.05555555556	0.7	0.8571428571
AM138I	0.1428571429	0.1428571429	0.2222222222	0.2222222222	0.4583333333	0.6428571429
FAM13A	0	0	0.05555555556	0.05555555556	0	0
M13A-A	0	0	0.05555555556	0.05555555556	0	0
FAM13B	0.04761904762	0.04761904762	0	0	inf	4.7619E+31
FAM13C	0.04761904762	0.04761904762	0.05555555556	0.05555555556	0.7	0.8571428571
AM149A	0	0	0.05555555556	0.05555555556	0	0
AM149B	0.1904761905	0.1904761905	0.05555555556	0.05555555556	3.294117647	3.428571429
AM151A	0.04761904762	0.04761904762	0.05555555556	0.05555555556	0.7	0.8571428571
AM151I	0.04761904762	0.04761904762	0	0	inf	4.7619E+31
AM153A	0.09523809524	0.09523809524	0.05555555556	0.05555555556	1.473684211	1.714285714
AM153I	0.04761904762	0.04761904762	0.05555555556	0.05555555556	0.7	0.8571428571
AM153C	0.09523809524	0.09523809524	0.05555555556	0.05555555556	1.473684211	1.714285714
AM157A	0.1428571429	0.1428571429	0.05555555556	0.05555555556	2.333333333	2.571428571
AM157I	0.2380952381	0.2380952381	0.05555555556	0.05555555556	4.375	4.285714286
AM157C	0.09523809524	0.09523809524	0	0	inf	9.52381E+31
AM160A	0	0	0.05555555556	0.05555555556	0	0
AM160A	0.04761904762	0.04761904762	0	0	inf	4.7619E+31
AM160B	0.1904761905	0.1904761905	0.05555555556	0.05555555556	3.294117647	3.428571429
AM160B	0.09523809524	0.09523809524	0.1111111111	0.1111111111	0.6842105263	0.8571428571
AM161A	0.04761904762	0.04761904762	0.05555555556	0.05555555556	0.7	0.8571428571
AM161I	0.09523809524	0.09523809524	0	0	inf	9.52381E+31
AM162A	0.09523809524	0.09523809524	0.05555555556	0.05555555556	1.473684211	1.714285714
AM163A	0.1428571429	0.1428571429	0	0	inf	1.42857E+32
AM163I	0.04761904762	0.04761904762	0	0	inf	4.7619E+31
AM166A	0.1428571429	0.1428571429	0.05555555556	0.05555555556	2.333333333	2.571428571
AM166I	0.09523809524	0.09523809524	0.05555555556	0.05555555556	1.473684211	1.714285714
AM167A	0.09523809524	0.09523809524	0.1111111111	0.1111111111	0.6842105263	0.8571428571
M167A-A	0.09523809524	0.09523809524	0.1111111111	0.1111111111	0.6842105263	0.8571428571
AM167I	0.04761904762	0.04761904762	0.1111111111	0.1111111111	0.325	0.4285714286
AM168I	0	0	0.05555555556	0.05555555556	0	0
AM169A	0.04761904762	0.04761904762	0	0	inf	4.7619E+31
AM169I	0.04761904762	0.04761904762	0.05555555556	0.05555555556	0.7	0.8571428571
AM170A	0.04761904762	0.04761904762	0	0	inf	4.7619E+31
AM170I	0.09523809524	0.09523809524	0.05555555556	0.05555555556	1.473684211	1.714285714
M170B-A	0.09523809524	0.09523809524	0.05555555556	0.05555555556	1.473684211	1.714285714
AM171A	0.09523809524	0.09523809524	0.1111111111	0.1111111111	0.6842105263	0.8571428571
AM171A	0.09523809524	0.09523809524	0	0	inf	9.52381E+31
AM171I	0	0	0.05555555556	0.05555555556	0	0
AM172A	0.04761904762	0.04761904762	0	0	inf	4.7619E+31
AM172B	0.09523809524	0.09523809524	0.05555555556	0.05555555556	1.473684211	1.714285714
AM173A	0.04761904762	0.04761904762	0.2222222222	0.2222222222	0.1375	0.2142857143
AM174A	0.04761904762	0.04761904762	0	0	inf	4.7619E+31
AM174I	0.04761904762	0.04761904762	0.05555555556	0.05555555556	0.7	0.8571428571
AM177A	0.09523809524	0.09523809524	0	0	inf	9.52381E+31
AM177I	0.1428571429	0.1428571429	0.05555555556	0.05555555556	2.333333333	2.571428571
AM178I	0	0	0.1111111111	0.1111111111	0	0

AM180A	0.04761904762	0.04761904762	0.05555555556	0.05555555556	0.7	0.8571428571
AM180B	0	0	0.05555555556	0.05555555556	0	0
AM182A	0.1904761905	0.1904761905	0.05555555556	0.05555555556	3.294117647	3.428571429
AM182B	0.1904761905	0.1904761905	0.05555555556	0.05555555556	3.294117647	3.428571429
AM183A	0.04761904762	0.04761904762	0.05555555556	0.05555555556	0.7	0.8571428571
AM183B	0.04761904762	0.04761904762	0.2222222222	0.2222222222	0.1375	0.2142857143
AM184B	0	0	0	0		0.00001
AM185A	0.04761904762	0.04761904762	0.05555555556	0.05555555556	0.7	0.8571428571
AM185B	0.04761904762	0.04761904762	0.1111111111	0.1111111111	0.325	0.4285714286
AM186A	0.09523809524	0.09523809524	0.05555555556	0.05555555556	1.473684211	1.714285714
AM186B	0.04761904762	0.04761904762	0.05555555556	0.05555555556	0.7	0.8571428571
AM187A	0.1428571429	0.1428571429	0	0	inf	1.42857E+32
AM187B	0.04761904762	0.04761904762	0.05555555556	0.05555555556	0.7	0.8571428571
AM189A	0	0	0.05555555556	0.05555555556	0	0
AM189B	0.1904761905	0.1904761905	0.1111111111	0.1111111111	1.529411765	1.714285714
AM192A	0.04761904762	0.04761904762	0	0	inf	4.7619E+31
AM193A	0.04761904762	0.04761904762	0	0	inf	4.7619E+31
AM193B	0.09523809524	0.09523809524	0.05555555556	0.05555555556	1.473684211	1.714285714
M198B-A	0	0	0.05555555556	0.05555555556	0	0
AM200A	0.04761904762	0.04761904762	0.05555555556	0.05555555556	0.7	0.8571428571
AM200B	0	0	0	0		0.00001
AM201A	0.04761904762	0.04761904762	0.05555555556	0.05555555556	0.7	0.8571428571
AM201B	0	0	0.05555555556	0.05555555556	0	0
AM204A	0.2380952381	0.2380952381	0	0	inf	2.38095E+32
AM205A	0.09523809524	0.09523809524	0.05555555556	0.05555555556	1.473684211	1.714285714
AM205B	0.09523809524	0.09523809524	0.05555555556	0.05555555556	1.473684211	1.714285714
AM205C	0.09523809524	0.09523809524	0.05555555556	0.05555555556	1.473684211	1.714285714
AM207A	0.04761904762	0.04761904762	0.1111111111	0.1111111111	0.325	0.4285714286
AM209A	0.09523809524	0.09523809524	0.1666666667	0.1666666667	0.4210526316	0.5714285714
AM209B	0.09523809524	0.09523809524	0.1666666667	0.1666666667	0.4210526316	0.5714285714
FAM20A	0	0	0	0		0.00001
FAM20B	0.1428571429	0.1428571429	0	0	inf	1.42857E+32
FAM20C	0.04761904762	0.04761904762	0.1666666667	0.1666666667	0.2	0.2857142857
AM210A	0.09523809524	0.09523809524	0.05555555556	0.05555555556	1.473684211	1.714285714
AM210B	0.09523809524	0.09523809524	0.1666666667	0.1666666667	0.4210526316	0.5714285714
AM214A	0.04761904762	0.04761904762	0.05555555556	0.05555555556	0.7	0.8571428571
AM214B	0.09523809524	0.09523809524	0.05555555556	0.05555555556	1.473684211	1.714285714
AM215A	0.09523809524	0.09523809524	0	0	inf	9.52381E+31
AM216A	0	0	0.1111111111	0.1111111111	0	0
AM217B	0.1428571429	0.1428571429	0.1666666667	0.1666666667	0.6666666667	0.8571428571
AM218A	0.04761904762	0.04761904762	0.05555555556	0.05555555556	0.7	0.8571428571
AM219A	0.09523809524	0.09523809524	0.05555555556	0.05555555556	1.473684211	1.714285714
AM219B	0.04761904762	0.04761904762	0.05555555556	0.05555555556	0.7	0.8571428571
AM21E	0.04761904762	0.04761904762	0.05555555556	0.05555555556	0.7	0.8571428571
AM220A	0.04761904762	0.04761904762	0.1666666667	0.1666666667	0.2	0.2857142857
AM221A	0.04761904762	0.04761904762	0.1666666667	0.1666666667	0.2	0.2857142857
AM221B	0.09523809524	0.09523809524	0.05555555556	0.05555555556	1.473684211	1.714285714
AM222A	0	0	0.05555555556	0.05555555556	0	0
M222A-A	0	0	0.05555555556	0.05555555556	0	0
AM222B	0.04761904762	0.04761904762	0.05555555556	0.05555555556	0.7	0.8571428571
AM225A	0.04761904762	0.04761904762	0.05555555556	0.05555555556	0.7	0.8571428571

**AM225I**	0.04761904762	0.04761904762	0.05555555556	0.05555555556	0.7	0.8571428571
**AM227A**	0.04761904762	0.04761904762	0.1111111111	0.1111111111	0.325	0.4285714286
**AM227I**	0.04761904762	0.04761904762	0.05555555556	0.05555555556	0.7	0.8571428571
**AM229A**	0.04761904762	0.04761904762	0.1111111111	0.1111111111	0.325	0.4285714286
**AM230A**	0.04761904762	0.04761904762	0.05555555556	0.05555555556	0.7	0.8571428571
**AM230I**	0.04761904762	0.04761904762	0.05555555556	0.05555555556	0.7	0.8571428571
**AM230I**	0.04761904762	0.04761904762	0.05555555556	0.05555555556	0.7	0.8571428571
**AM230I**	0.04761904762	0.04761904762	0.05555555556	0.05555555556	0.7	0.8571428571
**AM230I**	0.04761904762	0.04761904762	0.05555555556	0.05555555556	0.7	0.8571428571
**AM230C**	0.04761904762	0.04761904762	0.05555555556	0.05555555556	0.7	0.8571428571
**AM230I**	0.04761904762	0.04761904762	0.05555555556	0.05555555556	0.7	0.8571428571
**FAM230**	0	0	0.05555555556	0.05555555556	0	0
**FAM230J**	0.04761904762	0.04761904762	0.05555555556	0.05555555556	0.7	0.8571428571
**AM234A**	0.04761904762	0.04761904762	0.2222222222	0.2222222222	0.1375	0.2142857143
**AM234I**	0.04761904762	0.04761904762	0.05555555556	0.05555555556	0.7	0.8571428571
**AM237A**	0	0	0.05555555556	0.05555555556	0	0
**AM238A**	0.09523809524	0.09523809524	0.1111111111	0.1111111111	0.6842105263	0.8571428571
**AM238I**	0.09523809524	0.09523809524	0.1111111111	0.1111111111	0.6842105263	0.8571428571
**AM238C**	0.09523809524	0.09523809524	0.1111111111	0.1111111111	0.6842105263	0.8571428571
**AM241A**	0	0	0.05555555556	0.05555555556	0	0
**AM241I**	0.09523809524	0.09523809524	0	0	inf	9.52381E+31
**AM242I**	0.09523809524	0.09523809524	0.05555555556	0.05555555556	1.473684211	1.714285714
**AM242I**	0.1428571429	0.1428571429	0.05555555556	0.05555555556	2.333333333	2.571428571
**AM243A**	0.04761904762	0.04761904762	0.1111111111	0.1111111111	0.325	0.4285714286
**AM243I**	0.04761904762	0.04761904762	0.1111111111	0.1111111111	0.325	0.4285714286
**AM245A**	0.1428571429	0.1428571429	0.1111111111	0.1111111111	1.083333333	1.285714286
**FAM24A**	0.2380952381	0.2380952381	0.05555555556	0.05555555556	4.375	4.285714286
**FAM24B**	0.2380952381	0.2380952381	0.05555555556	0.05555555556	4.375	4.285714286
**I24B-CU**	0.2380952381	0.2380952381	0.05555555556	0.05555555556	4.375	4.285714286
**AM25B**	0.1428571429	0.1428571429	0.05555555556	0.05555555556	2.333333333	2.571428571
**FAM25C**	0.1428571429	0.1428571429	0.05555555556	0.05555555556	2.333333333	2.571428571
**FAM25E**	0.1428571429	0.1428571429	0.05555555556	0.05555555556	2.333333333	2.571428571
**FAM25G**	0.1428571429	0.1428571429	0.05555555556	0.05555555556	2.333333333	2.571428571
**FAM27B**	0.09523809524	0.09523809524	0.2222222222	0.2222222222	0.2894736842	0.4285714286
**FAM27C**	0.1428571429	0.1428571429	0.05555555556	0.05555555556	2.333333333	2.571428571
**AM27E**	0.1428571429	0.1428571429	0.05555555556	0.05555555556	2.333333333	2.571428571
**AM27E**	0.09523809524	0.09523809524	0.2222222222	0.2222222222	0.2894736842	0.4285714286
**AM27E**	0.09523809524	0.09523809524	0.05555555556	0.05555555556	1.473684211	1.714285714
**FAM30A**	0.04761904762	0.04761904762	0	0	inf	4.7619E+31
**FAM30C**	0.2857142857	0.2857142857	0.1111111111	0.1111111111	2.6	2.571428571
**FAM32A**	0.1428571429	0.1428571429	0.1111111111	0.1111111111	1.083333333	1.285714286
**FAM3B**	0.04761904762	0.04761904762	0.1111111111	0.1111111111	0.325	0.4285714286
**FAM3C**	0.04761904762	0.04761904762	0.05555555556	0.05555555556	0.7	0.8571428571
**FAM3D**	0.04761904762	0.04761904762	0	0	inf	4.7619E+31
**M3D-AS**	0.04761904762	0.04761904762	0	0	inf	4.7619E+31
**FAM41C**	0	0	0.1111111111	0.1111111111	0	0
**FAM43A**	0.09523809524	0.09523809524	0.05555555556	0.05555555556	1.473684211	1.714285714
**FAM43B**	0.04761904762	0.04761904762	0.1111111111	0.1111111111	0.325	0.4285714286
**FAM45A**	0.2380952381	0.2380952381	0	0	inf	2.38095E+32
**FAM47E**	0.04761904762	0.04761904762	0.05555555556	0.05555555556	0.7	0.8571428571
**I47E-ST**	0.04761904762	0.04761904762	0.05555555556	0.05555555556	0.7	0.8571428571

FAM49A	0	0	0.05555555556	0.05555555556	0	0
FAM49B	0.04761904762	0.04761904762	0.1111111111	0.1111111111	0.325	0.4285714286
FAM53A	0.04761904762	0.04761904762	0	0	inf	4.7619E+31
FAM53B	0.2380952381	0.2380952381	0.05555555556	0.05555555556	4.375	4.285714286
M53B-A	0.2380952381	0.2380952381	0.05555555556	0.05555555556	4.375	4.285714286
FAM53C	0.04761904762	0.04761904762	0	0	inf	4.7619E+31
FAM57B	0.04761904762	0.04761904762	0	0	inf	4.7619E+31
FAM66A	0.09523809524	0.09523809524	0.1111111111	0.1111111111	0.6842105263	0.8571428571
FAM66B	0.09523809524	0.09523809524	0.2222222222	0.2222222222	0.2894736842	0.4285714286
FAM66C	0.04761904762	0.04761904762	0.1111111111	0.1111111111	0.325	0.4285714286
FAM66D	0.09523809524	0.09523809524	0.1111111111	0.1111111111	0.6842105263	0.8571428571
FAM66E	0.09523809524	0.09523809524	0.2222222222	0.2222222222	0.2894736842	0.4285714286
FAM71A	0.09523809524	0.09523809524	0	0	inf	9.52381E+31
FAM71B	0.04761904762	0.04761904762	0.05555555556	0.05555555556	0.7	0.8571428571
FAM71C	0.04761904762	0.04761904762	0.05555555556	0.05555555556	0.7	0.8571428571
AM71E	0.09523809524	0.09523809524	0.1111111111	0.1111111111	0.6842105263	0.8571428571
AM71E	0.09523809524	0.09523809524	0.05555555556	0.05555555556	1.473684211	1.714285714
AM71F	0.04761904762	0.04761904762	0.05555555556	0.05555555556	0.7	0.8571428571
AM71F	0.04761904762	0.04761904762	0.05555555556	0.05555555556	0.7	0.8571428571
FAM72A	0.1428571429	0.1428571429	0	0	inf	1.42857E+32
FAM72B	0.04761904762	0.04761904762	0.1111111111	0.1111111111	0.325	0.4285714286
FAM72C	0.1428571429	0.1428571429	0.05555555556	0.05555555556	2.333333333	2.571428571
FAM72D	0.09523809524	0.09523809524	0.05555555556	0.05555555556	1.473684211	1.714285714
AM74A	0.09523809524	0.09523809524	0.2222222222	0.2222222222	0.2894736842	0.4285714286
AM74A	0.04761904762	0.04761904762	0.1111111111	0.1111111111	0.325	0.4285714286
AM74A	0.09523809524	0.09523809524	0.2222222222	0.2222222222	0.2894736842	0.4285714286
AM74A	0.09523809524	0.09523809524	0.2222222222	0.2222222222	0.2894736842	0.4285714286
AM74A	0.1428571429	0.1428571429	0.05555555556	0.05555555556	2.333333333	2.571428571
FAM76A	0.04761904762	0.04761904762	0.1111111111	0.1111111111	0.325	0.4285714286
FAM78A	0.09523809524	0.09523809524	0.05555555556	0.05555555556	1.473684211	1.714285714
FAM78B	0.1428571429	0.1428571429	0	0	inf	1.42857E+32
FAM81A	0.04761904762	0.04761904762	0.05555555556	0.05555555556	0.7	0.8571428571
FAM81B	0.04761904762	0.04761904762	0	0	inf	4.7619E+31
FAM83A	0.1428571429	0.1428571429	0.1111111111	0.1111111111	1.083333333	1.285714286
M83A-A	0.1428571429	0.1428571429	0.1111111111	0.1111111111	1.083333333	1.285714286
FAM83B	0.04761904762	0.04761904762	0	0	inf	4.7619E+31
FAM83C	0.09523809524	0.09523809524	0.1111111111	0.1111111111	0.6842105263	0.8571428571
M83C-A	0.09523809524	0.09523809524	0.1111111111	0.1111111111	0.6842105263	0.8571428571
FAM83D	0.1428571429	0.1428571429	0.2222222222	0.2222222222	0.4583333333	0.6428571429
FAM83E	0.09523809524	0.09523809524	0.1111111111	0.1111111111	0.6842105263	0.8571428571
FAM83F	0	0	0.1111111111	0.1111111111	0	0
FAM83G	0.04761904762	0.04761904762	0.1111111111	0.1111111111	0.325	0.4285714286
FAM83H	0.04761904762	0.04761904762	0.1666666667	0.1666666667	0.2	0.2857142857
M83H-A	0.04761904762	0.04761904762	0.1666666667	0.1666666667	0.2	0.2857142857
FAM85A	0.09523809524	0.09523809524	0.1111111111	0.1111111111	0.6842105263	0.8571428571
FAM85B	0.09523809524	0.09523809524	0.1666666667	0.1666666667	0.4210526316	0.5714285714
AM86B	0.09523809524	0.09523809524	0.1111111111	0.1111111111	0.6842105263	0.8571428571
AM86B	0.09523809524	0.09523809524	0.1111111111	0.1111111111	0.6842105263	0.8571428571
AM86B3	0.09523809524	0.09523809524	0.1666666667	0.1666666667	0.4210526316	0.5714285714
AM86D	0.04761904762	0.04761904762	0	0	inf	4.7619E+31
AM86F	0.04761904762	0.04761904762	0.1111111111	0.1111111111	0.325	0.4285714286

AM86H	0.09523809524	0.09523809524	0.05555555556	0.05555555556	1.473684211	1.714285714
AM86J	0.04761904762	0.04761904762	0.05555555556	0.05555555556	0.7	0.8571428571
FAM87A	0.09523809524	0.09523809524	0.1111111111	0.1111111111	0.6842105263	0.8571428571
FAM87B	0	0	0.1111111111	0.1111111111	0	0
FAM89A	0.1428571429	0.1428571429	0	0	inf	1.42857E+32
AM90A	0.04761904762	0.04761904762	0.1111111111	0.1111111111	0.325	0.4285714286
AM90A1	0.09523809524	0.09523809524	0.2222222222	0.2222222222	0.2894736842	0.4285714286
AM90A2	0.09523809524	0.09523809524	0.1111111111	0.1111111111	0.6842105263	0.8571428571
AM90A2	0.09523809524	0.09523809524	0.05555555556	0.05555555556	1.473684211	1.714285714
AM90A2	0.09523809524	0.09523809524	0.1111111111	0.1111111111	0.6842105263	0.8571428571
AM90A7	0.09523809524	0.09523809524	0.2222222222	0.2222222222	0.2894736842	0.4285714286
AM91A	0.1428571429	0.1428571429	0.1111111111	0.1111111111	1.083333333	1.285714286
FAM92A	0.1904761905	0.1904761905	0.1111111111	0.1111111111	1.529411765	1.714285714
AM92A1	0	0	0.05555555556	0.05555555556	0	0
FAM92B	0.04761904762	0.04761904762	0	0	inf	4.7619E+31
FAM95A	0	0	0.1111111111	0.1111111111	0	0
AM95B	0.1904761905	0.1904761905	0.2222222222	0.2222222222	0.6470588235	0.8571428571
FAM95C	0.04761904762	0.04761904762	0.05555555556	0.05555555556	0.7	0.8571428571
FAM98B	0	0	0	0		0.00001
FAM98C	0.04761904762	0.04761904762	0.05555555556	0.05555555556	0.7	0.8571428571
FAM99A	0.04761904762	0.04761904762	0	0	inf	4.7619E+31
FAM99B	0.04761904762	0.04761904762	0	0	inf	4.7619E+31
FANCA	0.09523809524	0.09523809524	0	0	inf	9.52381E+31
FANCC	0.04761904762	0.04761904762	0.05555555556	0.05555555556	0.7	0.8571428571
FANCD2	0.09523809524	0.09523809524	0	0	inf	9.52381E+31
ANCD2C	0.09523809524	0.09523809524	0	0	inf	9.52381E+31
FANCE	0.04761904762	0.04761904762	0.05555555556	0.05555555556	0.7	0.8571428571
FANCG	0.09523809524	0.09523809524	0.05555555556	0.05555555556	1.473684211	1.714285714
FANCI	0.04761904762	0.04761904762	0.05555555556	0.05555555556	0.7	0.8571428571
FANCL	0.04761904762	0.04761904762	0.05555555556	0.05555555556	0.7	0.8571428571
FANCM	0.04761904762	0.04761904762	0	0	inf	4.7619E+31
FANK1	0.2380952381	0.2380952381	0.05555555556	0.05555555556	4.375	4.285714286
ANK1-A	0.2380952381	0.2380952381	0.05555555556	0.05555555556	4.375	4.285714286
FAP	0	0	0.05555555556	0.05555555556	0	0
FAR2	0.04761904762	0.04761904762	0.05555555556	0.05555555556	0.7	0.8571428571
FAR2P1	0	0	0.05555555556	0.05555555556	0	0
FAR2P2	0	0	0.05555555556	0.05555555556	0	0
FARP2	0	0	0.05555555556	0.05555555556	0	0
FARSA	0.1428571429	0.1428571429	0.1111111111	0.1111111111	1.083333333	1.285714286
RSA-A	0.1428571429	0.1428571429	0.1111111111	0.1111111111	1.083333333	1.285714286
FARSB	0	0	0.05555555556	0.05555555556	0	0
FAS	0.1428571429	0.1428571429	0.1666666667	0.1666666667	0.6666666667	0.8571428571
FAS-AS1	0.1428571429	0.1428571429	0.1666666667	0.1666666667	0.6666666667	0.8571428571
FASLG	0.1428571429	0.1428571429	0	0	inf	1.42857E+32
FASN	0.04761904762	0.04761904762	0	0	inf	4.7619E+31
FASTK	0.09523809524	0.09523809524	0.05555555556	0.05555555556	1.473684211	1.714285714
ASTKD	0	0	0.05555555556	0.05555555556	0	0
ASTKD	0	0	0.05555555556	0.05555555556	0	0
ASTKD	0	0	0.05555555556	0.05555555556	0	0
ASTKD	0.1904761905	0.1904761905	0.05555555556	0.05555555556	3.294117647	3.428571429
FAT1	0	0	0.05555555556	0.05555555556	0	0

**FAT4**	0	0	0.05555555556	0.05555555556	0	0
**FAXDC2**	0	0	0.05555555556	0.05555555556	0	0
**FBF1**	0.04761904762	0.04761904762	0	0	inf	4.7619E+31
**FBH1**	0.09523809524	0.09523809524	0.1111111111	0.1111111111	0.6842105263	0.8571428571
**FBL**	0.04761904762	0.04761904762	0.05555555556	0.05555555556	0.7	0.8571428571
**FBLIM1**	0.04761904762	0.04761904762	0.1111111111	0.1111111111	0.325	0.4285714286
**FBLL1**	0	0	0.05555555556	0.05555555556	0	0
**FBLN1**	0	0	0.1111111111	0.1111111111	0	0
**FBLN2**	0.04761904762	0.04761904762	0	0	inf	4.7619E+31
**FBLN7**	0	0	0.05555555556	0.05555555556	0	0
**FBN1**	0.04761904762	0.04761904762	0.05555555556	0.05555555556	0.7	0.8571428571
**FBN2**	0.04761904762	0.04761904762	0	0	inf	4.7619E+31
**FBN3**	0.1428571429	0.1428571429	0.05555555556	0.05555555556	2.333333333	2.571428571
**FBP1**	0.04761904762	0.04761904762	0.05555555556	0.05555555556	0.7	0.8571428571
**FBP2**	0.04761904762	0.04761904762	0.05555555556	0.05555555556	0.7	0.8571428571
**FBRS**	0.04761904762	0.04761904762	0	0	inf	4.7619E+31
**FBRSL1**	0.09523809524	0.09523809524	0.05555555556	0.05555555556	1.473684211	1.714285714
**FBXL12**	0.1428571429	0.1428571429	0.1111111111	0.1111111111	1.083333333	1.285714286
**FBXL13**	0.04761904762	0.04761904762	0.05555555556	0.05555555556	0.7	0.8571428571
**FBXL14**	0.04761904762	0.04761904762	0.1111111111	0.1111111111	0.325	0.4285714286
**FBXL15**	0.1428571429	0.1428571429	0.05555555556	0.05555555556	2.333333333	2.571428571
**FBXL16**	0.04761904762	0.04761904762	0.2222222222	0.2222222222	0.1375	0.2142857143
**FBXL17**	0.04761904762	0.04761904762	0	0	inf	4.7619E+31
**FBXL18**	0.04761904762	0.04761904762	0.1666666667	0.1666666667	0.2	0.2857142857
**FBXL19**	0.04761904762	0.04761904762	0	0	inf	4.7619E+31
**3XL19-A**	0.04761904762	0.04761904762	0	0	inf	4.7619E+31
**FBXL2**	0.09523809524	0.09523809524	0	0	inf	9.52381E+31
**FBXL20**	0.09523809524	0.09523809524	0	0	inf	9.52381E+31
**'BXL21I**	0.04761904762	0.04761904762	0	0	inf	4.7619E+31
**FBXL22**	0.04761904762	0.04761904762	0.05555555556	0.05555555556	0.7	0.8571428571
**FBXL3**	0	0	0.05555555556	0.05555555556	0	0
**FBXL5**	0	0	0	0		0.00001
**FBXL6**	0.04761904762	0.04761904762	0.1666666667	0.1666666667	0.2	0.2857142857
**FBXL7**	0.04761904762	0.04761904762	0.05555555556	0.05555555556	0.7	0.8571428571
**FBXL8**	0.1428571429	0.1428571429	0.05555555556	0.05555555556	2.333333333	2.571428571
**FBXO10**	0.09523809524	0.09523809524	0.05555555556	0.05555555556	1.473684211	1.714285714
**FBXO11**	0	0	0.05555555556	0.05555555556	0	0
**FBXO16**	0.04761904762	0.04761904762	0.1111111111	0.1111111111	0.325	0.4285714286
**FBXO17**	0.04761904762	0.04761904762	0.05555555556	0.05555555556	0.7	0.8571428571
**FBXO2**	0.04761904762	0.04761904762	0.1111111111	0.1111111111	0.325	0.4285714286
**FBXO21**	0	0	0.05555555556	0.05555555556	0	0
**FBXO22**	0.04761904762	0.04761904762	0.05555555556	0.05555555556	0.7	0.8571428571
**FBXO24**	0.04761904762	0.04761904762	0.05555555556	0.05555555556	0.7	0.8571428571
**FBXO25**	0.09523809524	0.09523809524	0.1111111111	0.1111111111	0.6842105263	0.8571428571
**FBXO27**	0.04761904762	0.04761904762	0.05555555556	0.05555555556	0.7	0.8571428571
**FBXO28**	0.1428571429	0.1428571429	0.05555555556	0.05555555556	2.333333333	2.571428571
**FBXO31**	0.04761904762	0.04761904762	0	0	inf	4.7619E+31
**FBXO32**	0.1428571429	0.1428571429	0.1111111111	0.1111111111	1.083333333	1.285714286
**FBXO33**	0.04761904762	0.04761904762	0	0	inf	4.7619E+31
**FBXO34**	0.04761904762	0.04761904762	0	0	inf	4.7619E+31
**FBXO36**	0	0	0.05555555556	0.05555555556	0	0

FBXO39	0	0	0.05555555556	0.05555555556	0	0
FBXO4	0.04761904762	0.04761904762	0.05555555556	0.05555555556	0.7	0.8571428571
FBXO40	0.1428571429	0.1428571429	0.05555555556	0.05555555556	2.333333333	2.571428571
FBXO41	0	0	0.05555555556	0.05555555556	0	0
FBXO42	0.04761904762	0.04761904762	0.1111111111	0.1111111111	0.325	0.4285714286
FBXO43	0.1904761905	0.1904761905	0.1111111111	0.1111111111	1.529411765	1.714285714
FBXO44	0.04761904762	0.04761904762	0.1111111111	0.1111111111	0.325	0.4285714286
FBXO45	0.1428571429	0.1428571429	0.05555555556	0.05555555556	2.333333333	2.571428571
FBXO46	0.09523809524	0.09523809524	0.1111111111	0.1111111111	0.6842105263	0.8571428571
FBXO47	0.09523809524	0.09523809524	0	0	inf	9.52381E+31
FBXO48	0	0	0.05555555556	0.05555555556	0	0
FBXO6	0.04761904762	0.04761904762	0.1111111111	0.1111111111	0.325	0.4285714286
FBXO7	0.04761904762	0.04761904762	0.1111111111	0.1111111111	0.325	0.4285714286
FBXO8	0	0	0.05555555556	0.05555555556	0	0
FBXO9	0.04761904762	0.04761904762	0	0	inf	4.7619E+31
FBXW10	0.04761904762	0.04761904762	0.1111111111	0.1111111111	0.325	0.4285714286
FBXW11	0	0	0.05555555556	0.05555555556	0	0
FBXW12	0.09523809524	0.09523809524	0	0	inf	9.52381E+31
FBXW2	0.09523809524	0.09523809524	0.05555555556	0.05555555556	1.473684211	1.714285714
FBXW4	0.1428571429	0.1428571429	0	0	inf	1.42857E+32
BXW4P	0	0	0.05555555556	0.05555555556	0	0
FBXW5	0.1428571429	0.1428571429	0.05555555556	0.05555555556	2.333333333	2.571428571
FBXW7	0	0	0.05555555556	0.05555555556	0	0
BXW7-A	0	0	0.05555555556	0.05555555556	0	0
FBXW8	0	0	0.05555555556	0.05555555556	0	0
FBXW9	0.1428571429	0.1428571429	0.1111111111	0.1111111111	1.083333333	1.285714286
FCAMR	0.1428571429	0.1428571429	0	0	inf	1.42857E+32
FCAR	0.09523809524	0.09523809524	0.05555555556	0.05555555556	1.473684211	1.714285714
FCER1A	0.1428571429	0.1428571429	0	0	inf	1.42857E+32
FCER1G	0.1428571429	0.1428571429	0	0	inf	1.42857E+32
FCER2	0.1428571429	0.1428571429	0.05555555556	0.05555555556	2.333333333	2.571428571
FCF1P2	0.09523809524	0.09523809524	0	0	inf	9.52381E+31
FCGBP	0.04761904762	0.04761904762	0.05555555556	0.05555555556	0.7	0.8571428571
FCGR1A	0.1428571429	0.1428571429	0.05555555556	0.05555555556	2.333333333	2.571428571
FCGR1B	0.04761904762	0.04761904762	0.1111111111	0.1111111111	0.325	0.4285714286
CGR1C	0.09523809524	0.09523809524	0.05555555556	0.05555555556	1.473684211	1.714285714
FCGR2A	0.1428571429	0.1428571429	0	0	inf	1.42857E+32
FCGR2B	0.1428571429	0.1428571429	0	0	inf	1.42857E+32
FCGR2C	0.1428571429	0.1428571429	0	0	inf	1.42857E+32
FCGR3A	0.1428571429	0.1428571429	0	0	inf	1.42857E+32
FCGR3B	0.1428571429	0.1428571429	0	0	inf	1.42857E+32
FCGRT	0.09523809524	0.09523809524	0.1111111111	0.1111111111	0.6842105263	0.8571428571
FCHO1	0.1428571429	0.1428571429	0.1111111111	0.1111111111	1.083333333	1.285714286
FCHO2	0	0	0.05555555556	0.05555555556	0	0
FCHSD1	0.04761904762	0.04761904762	0	0	inf	4.7619E+31
FCMR	0.1428571429	0.1428571429	0	0	inf	1.42857E+32
FCN1	0.04761904762	0.04761904762	0	0	inf	4.7619E+31
FCN2	0.04761904762	0.04761904762	0	0	inf	4.7619E+31
FCN3	0.04761904762	0.04761904762	0.1111111111	0.1111111111	0.325	0.4285714286
FCRL1	0.1428571429	0.1428571429	0.05555555556	0.05555555556	2.333333333	2.571428571
FCRL2	0.1428571429	0.1428571429	0.05555555556	0.05555555556	2.333333333	2.571428571

FCRL3	0.1428571429	0.1428571429	0.05555555556	0.05555555556	2.333333333	2.571428571
FCRL4	0.1428571429	0.1428571429	0.05555555556	0.05555555556	2.333333333	2.571428571
FCRL5	0.1428571429	0.1428571429	0.05555555556	0.05555555556	2.333333333	2.571428571
FCRL6	0.1428571429	0.1428571429	0	0	inf	1.42857E+32
FCRLA	0.1428571429	0.1428571429	0	0	inf	1.42857E+32
FCRLB	0.1428571429	0.1428571429	0	0	inf	1.42857E+32
FCSK	0.1428571429	0.1428571429	0.05555555556	0.05555555556	2.333333333	2.571428571
FDCSP	0.04761904762	0.04761904762	0.05555555556	0.05555555556	0.7	0.8571428571
FDFT1	0.09523809524	0.09523809524	0.1111111111	0.1111111111	0.6842105263	0.8571428571
FDPS	0.1904761905	0.1904761905	0.1111111111	0.1111111111	1.529411765	1.714285714
FDPSP2	0.09523809524	0.09523809524	0.1111111111	0.1111111111	0.6842105263	0.8571428571
FDXR	0.04761904762	0.04761904762	0	0	inf	4.7619E+31
FECH	0.09523809524	0.09523809524	0	0	inf	9.52381E+31
FEM1A	0.09523809524	0.09523809524	0.05555555556	0.05555555556	1.473684211	1.714285714
FEM1B	0.04761904762	0.04761904762	0.05555555556	0.05555555556	0.7	0.8571428571
FEM1C	0.04761904762	0.04761904762	0	0	inf	4.7619E+31
FENDRR	0.04761904762	0.04761904762	0	0	inf	4.7619E+31
FER	0.04761904762	0.04761904762	0	0	inf	4.7619E+31
FER1L4	0.09523809524	0.09523809524	0.1111111111	0.1111111111	0.6842105263	0.8571428571
FER1L5	0	0	0.1111111111	0.1111111111	0	0
FER1L6	0.1428571429	0.1428571429	0.1111111111	0.1111111111	1.083333333	1.285714286
R1L6-A	0.1428571429	0.1428571429	0.1111111111	0.1111111111	1.083333333	1.285714286
R1L6-A	0.1428571429	0.1428571429	0.1111111111	0.1111111111	1.083333333	1.285714286
FERD3L	0.04761904762	0.04761904762	0.2222222222	0.2222222222	0.1375	0.2142857143
FERMT1	0.1904761905	0.1904761905	0.05555555556	0.05555555556	3.294117647	3.428571429
FERMT2	0.04761904762	0.04761904762	0	0	inf	4.7619E+31
FES	0.04761904762	0.04761904762	0.05555555556	0.05555555556	0.7	0.8571428571
FETUB	0.09523809524	0.09523809524	0.05555555556	0.05555555556	1.473684211	1.714285714
FEV	0	0	0.05555555556	0.05555555556	0	0
FEZ2	0	0	0.05555555556	0.05555555556	0	0
FEZF1	0.04761904762	0.04761904762	0.05555555556	0.05555555556	0.7	0.8571428571
EZF1-AS	0.04761904762	0.04761904762	0.05555555556	0.05555555556	0.7	0.8571428571
FEZF2	0.04761904762	0.04761904762	0	0	inf	4.7619E+31
FFAR1	0.04761904762	0.04761904762	0.05555555556	0.05555555556	0.7	0.8571428571
FFAR2	0.04761904762	0.04761904762	0.05555555556	0.05555555556	0.7	0.8571428571
FFAR3	0.04761904762	0.04761904762	0.05555555556	0.05555555556	0.7	0.8571428571
FFAR4	0.1428571429	0.1428571429	0.05555555556	0.05555555556	2.333333333	2.571428571
FGA	0	0	0.05555555556	0.05555555556	0	0
FGB	0	0	0.05555555556	0.05555555556	0	0
FGD2	0.04761904762	0.04761904762	0.05555555556	0.05555555556	0.7	0.8571428571
FGD3	0.04761904762	0.04761904762	0.05555555556	0.05555555556	0.7	0.8571428571
FGD4	0.1428571429	0.1428571429	0.1111111111	0.1111111111	1.083333333	1.285714286
FGD5	0.04761904762	0.04761904762	0	0	inf	4.7619E+31
GD5-AS	0.04761904762	0.04761904762	0	0	inf	4.7619E+31
FGD5P1	0.04761904762	0.04761904762	0	0	inf	4.7619E+31
FGD6	0.04761904762	0.04761904762	0.05555555556	0.05555555556	0.7	0.8571428571
FGF1	0.04761904762	0.04761904762	0	0	inf	4.7619E+31
FGF10	0.04761904762	0.04761904762	0.05555555556	0.05555555556	0.7	0.8571428571
GF10-AS	0.04761904762	0.04761904762	0.05555555556	0.05555555556	0.7	0.8571428571
FGF11	0	0	0.05555555556	0.05555555556	0	0
FGF12	0.09523809524	0.09523809524	0.05555555556	0.05555555556	1.473684211	1.714285714

GF12-AS	0.09523809524	0.09523809524	0.05555555556	0.05555555556	1.473684211	1.714285714
GF12-AS	0.09523809524	0.09523809524	0.05555555556	0.05555555556	1.473684211	1.714285714
FGF17	0.09523809524	0.09523809524	0.1111111111	0.1111111111	0.6842105263	0.8571428571
FGF18	0	0	0.05555555556	0.05555555556	0	0
FGF2	0	0	0.05555555556	0.05555555556	0	0
FGF20	0.09523809524	0.09523809524	0.1111111111	0.1111111111	0.6842105263	0.8571428571
FGF21	0.09523809524	0.09523809524	0.1111111111	0.1111111111	0.6842105263	0.8571428571
FGF22	0.09523809524	0.09523809524	0.1111111111	0.1111111111	0.6842105263	0.8571428571
FGF23	0.04761904762	0.04761904762	0.1111111111	0.1111111111	0.325	0.4285714286
FGF5	0.04761904762	0.04761904762	0.05555555556	0.05555555556	0.7	0.8571428571
FGF6	0.04761904762	0.04761904762	0.1111111111	0.1111111111	0.325	0.4285714286
FGF7	0.04761904762	0.04761904762	0.05555555556	0.05555555556	0.7	0.8571428571
FGF7P3	0.1904761905	0.1904761905	0.05555555556	0.05555555556	3.294117647	3.428571429
FGF7P6	0.1428571429	0.1428571429	0.05555555556	0.05555555556	2.333333333	2.571428571
FGF8	0.1428571429	0.1428571429	0	0	inf	1.42857E+32
FGFBP1	0	0	0	0		0.00001
FGFBP2	0	0	0	0		0.00001
FGFBP3	0.1428571429	0.1428571429	0.1111111111	0.1111111111	1.083333333	1.285714286
FGFR1	0.09523809524	0.09523809524	0.1111111111	0.1111111111	0.6842105263	0.8571428571
GFR1OP	0.04761904762	0.04761904762	0.05555555556	0.05555555556	0.7	0.8571428571
FGFR2	0.2380952381	0.2380952381	0	0	inf	2.38095E+32
FGFR3	0.04761904762	0.04761904762	0	0	inf	4.7619E+31
FGFR4	0.09523809524	0.09523809524	0.05555555556	0.05555555556	1.473684211	1.714285714
FGFRL1	0.04761904762	0.04761904762	0	0	inf	4.7619E+31
FGG	0	0	0.05555555556	0.05555555556	0	0
FGGY	0.04761904762	0.04761904762	0.05555555556	0.05555555556	0.7	0.8571428571
FGR	0.04761904762	0.04761904762	0.1111111111	0.1111111111	0.325	0.4285714286
FH	0.1428571429	0.1428571429	0	0	inf	1.42857E+32
FHAD1	0.04761904762	0.04761904762	0.1111111111	0.1111111111	0.325	0.4285714286
FHDC1	0	0	0.05555555556	0.05555555556	0	0
FHIT	0.04761904762	0.04761904762	0	0	inf	4.7619E+31
FHL2	0	0	0.05555555556	0.05555555556	0	0
FHL3	0.04761904762	0.04761904762	0.05555555556	0.05555555556	0.7	0.8571428571
FHOD1	0.1428571429	0.1428571429	0.05555555556	0.05555555556	2.333333333	2.571428571
FHOD3	0.04761904762	0.04761904762	0	0	inf	4.7619E+31
FIBCD1	0.09523809524	0.09523809524	0.05555555556	0.05555555556	1.473684211	1.714285714
FICD	0.04761904762	0.04761904762	0.05555555556	0.05555555556	0.7	0.8571428571
FIG4	0	0	0	0		0.00001
FIGN	0	0	0.05555555556	0.05555555556	0	0
FIGNL1	0.1428571429	0.1428571429	0.2222222222	0.2222222222	0.4583333333	0.6428571429
FIGNL2	0.04761904762	0.04761904762	0.05555555556	0.05555555556	0.7	0.8571428571
FILIP1L	0.09523809524	0.09523809524	0.1111111111	0.1111111111	0.6842105263	0.8571428571
FIP1L1	0.2380952381	0.2380952381	0.05555555556	0.05555555556	4.375	4.285714286
FIS1	0.04761904762	0.04761904762	0.05555555556	0.05555555556	0.7	0.8571428571
FITM2	0.1428571429	0.1428571429	0.1666666667	0.1666666667	0.6666666667	0.8571428571
FIZ1	0.09523809524	0.09523809524	0.05555555556	0.05555555556	1.473684211	1.714285714
FKBP10	0.09523809524	0.09523809524	0	0	inf	9.52381E+31
FKBP11	0.04761904762	0.04761904762	0.05555555556	0.05555555556	0.7	0.8571428571
FKBP14	0.04761904762	0.04761904762	0.1666666667	0.1666666667	0.2	0.2857142857
FKBP15	0.04761904762	0.04761904762	0.05555555556	0.05555555556	0.7	0.8571428571
FKBP1A	0.1904761905	0.1904761905	0.1111111111	0.1111111111	1.529411765	1.714285714

**P1A-SD(**	0.1904761905	0.1904761905	0.1111111111	0.1111111111	1.529411765	1.714285714
**KBP1AI**	0.1428571429	0.1428571429	0.05555555556	0.05555555556	2.333333333	2.571428571
**FKBP3**	0.04761904762	0.04761904762	0	0	inf	4.7619E+31
**FKBP4**	0.04761904762	0.04761904762	0.1666666667	0.1666666667	0.2	0.2857142857
**FKBP5**	0.04761904762	0.04761904762	0.05555555556	0.05555555556	0.7	0.8571428571
**FKBP6**	0.1428571429	0.1428571429	0.1111111111	0.1111111111	1.083333333	1.285714286
**FKBP7**	0	0	0.1111111111	0.1111111111	0	0
**FKBP8**	0.1428571429	0.1428571429	0.1111111111	0.1111111111	1.083333333	1.285714286
**FKBP9**	0.04761904762	0.04761904762	0.1666666667	0.1666666667	0.2	0.2857142857
**KBP9P1**	0.3333333333	0.3333333333	0.2222222222	0.2222222222	1.375	1.5
**FKBPL**	0.04761904762	0.04761904762	0	0	inf	4.7619E+31
**FKRP**	0.09523809524	0.09523809524	0.1111111111	0.1111111111	0.6842105263	0.8571428571
**FKTN**	0.04761904762	0.04761904762	0.05555555556	0.05555555556	0.7	0.8571428571
**FLACC1**	0	0	0.05555555556	0.05555555556	0	0
**FLAD1**	0.1904761905	0.1904761905	0.1111111111	0.1111111111	1.529411765	1.714285714
**FLCN**	0.04761904762	0.04761904762	0.1111111111	0.1111111111	0.325	0.4285714286
**FLG**	0.1904761905	0.1904761905	0	0	inf	1.90476E+32
**FLG-AS1**	0.1904761905	0.1904761905	0	0	inf	1.90476E+32
**FLG2**	0.1904761905	0.1904761905	0	0	inf	1.90476E+32
**FLJ1282**	0.04761904762	0.04761904762	0.05555555556	0.05555555556	0.7	0.8571428571
**FLJ1322**	0.1428571429	0.1428571429	0.1111111111	0.1111111111	1.083333333	1.285714286
**FLJ1677**	0.1428571429	0.1428571429	0.2222222222	0.2222222222	0.4583333333	0.6428571429
**FLJ2002**	0	0	0.05555555556	0.05555555556	0	0
**FLJ2575**	0.1428571429	0.1428571429	0.05555555556	0.05555555556	2.333333333	2.571428571
**FLJ3067**	0.04761904762	0.04761904762	0	0	inf	4.7619E+31
**FLJ3090**	0	0	0.1666666667	0.1666666667	0	0
**FLJ3110**	0.04761904762	0.04761904762	0.05555555556	0.05555555556	0.7	0.8571428571
**FLJ3135**	0	0	0.05555555556	0.05555555556	0	0
**FLJ3225**	0.04761904762	0.04761904762	0.05555555556	0.05555555556	0.7	0.8571428571
**FLJ3353**	0	0	0.05555555556	0.05555555556	0	0
**FLJ3600**	0.09523809524	0.09523809524	0.05555555556	0.05555555556	1.473684211	1.714285714
**FLJ3720**	0.1428571429	0.1428571429	0.1111111111	0.1111111111	1.083333333	1.285714286
**FLJ3745**	0.04761904762	0.04761904762	0.1111111111	0.1111111111	0.325	0.4285714286
**FLJ3857**	0	0	0.05555555556	0.05555555556	0	0
**FLJ3909**	0.1428571429	0.1428571429	0	0	inf	1.42857E+32
**FLJ4019**	0.1428571429	0.1428571429	0	0	inf	1.42857E+32
**FLJ4028**	0.04761904762	0.04761904762	0.05555555556	0.05555555556	0.7	0.8571428571
**FLJ4235**	0	0	0.05555555556	0.05555555556	0	0
**FLJ4239**	0.09523809524	0.09523809524	0.1111111111	0.1111111111	0.6842105263	0.8571428571
**FLJ4262**	0.04761904762	0.04761904762	0.1111111111	0.1111111111	0.325	0.4285714286
**FLJ4296**	0.1904761905	0.1904761905	0.1111111111	0.1111111111	1.529411765	1.714285714
**FLJ4551**	0.09523809524	0.09523809524	0	0	inf	9.52381E+31
**FLJ4628**	0.1904761905	0.1904761905	0.1111111111	0.1111111111	1.529411765	1.714285714
**FLJ4687**	0	0	0.05555555556	0.05555555556	0	0
**FLNB**	0.09523809524	0.09523809524	0	0	inf	9.52381E+31
**LNB-AS**	0.04761904762	0.04761904762	0	0	inf	4.7619E+31
**FLNC**	0.04761904762	0.04761904762	0.05555555556	0.05555555556	0.7	0.8571428571
**LNC-AS**	0.04761904762	0.04761904762	0.05555555556	0.05555555556	0.7	0.8571428571
**FLOT2**	0.04761904762	0.04761904762	0.05555555556	0.05555555556	0.7	0.8571428571
**FLRT3**	0.1904761905	0.1904761905	0.1111111111	0.1111111111	1.529411765	1.714285714
**FLT3LG**	0.09523809524	0.09523809524	0.1111111111	0.1111111111	0.6842105263	0.8571428571

**FLT4**	0.09523809524	0.09523809524	0.05555555556	0.05555555556	1.473684211	1.714285714
**FLVCR1**	0.09523809524	0.09523809524	0	0	inf	9.52381E+31
**LVCR1-D**	0.09523809524	0.09523809524	0	0	inf	9.52381E+31
**LYWCH**	0.04761904762	0.04761904762	0.1111111111	0.1111111111	0.325	0.4285714286
**LYWCH**	0.04761904762	0.04761904762	0.1111111111	0.1111111111	0.325	0.4285714286
**FMC1**	0.04761904762	0.04761904762	0.05555555556	0.05555555556	0.7	0.8571428571
**C1-LUC**	0.04761904762	0.04761904762	0.05555555556	0.05555555556	0.7	0.8571428571
**FMN2**	0.1428571429	0.1428571429	0	0	inf	1.42857E+32
**FMNL1**	0.1428571429	0.1428571429	0	0	inf	1.42857E+32
**FMNL2**	0	0	0.05555555556	0.05555555556	0	0
**FMNL3**	0.04761904762	0.04761904762	0.05555555556	0.05555555556	0.7	0.8571428571
**FMO1**	0.1428571429	0.1428571429	0.05555555556	0.05555555556	2.333333333	2.571428571
**FMO2**	0.1428571429	0.1428571429	0.05555555556	0.05555555556	2.333333333	2.571428571
**FMO3**	0.1428571429	0.1428571429	0.05555555556	0.05555555556	2.333333333	2.571428571
**FMO4**	0.1428571429	0.1428571429	0.05555555556	0.05555555556	2.333333333	2.571428571
**FMO5**	0.1428571429	0.1428571429	0.05555555556	0.05555555556	2.333333333	2.571428571
**FMO6P**	0.1428571429	0.1428571429	0.05555555556	0.05555555556	2.333333333	2.571428571
**FMO9P**	0.1428571429	0.1428571429	0	0	inf	1.42857E+32
**FMOD**	0.1428571429	0.1428571429	0	0	inf	1.42857E+32
**FN1**	0	0	0.05555555556	0.05555555556	0	0
**FN3K**	0	0	0	0		0.00001
**FN3KRP**	0	0	0	0		0.00001
**FNBP1**	0.09523809524	0.09523809524	0.05555555556	0.05555555556	1.473684211	1.714285714
**FNBP1L**	0.04761904762	0.04761904762	0.05555555556	0.05555555556	0.7	0.8571428571
**FNBP4**	0	0	0.05555555556	0.05555555556	0	0
**FNDC10**	0	0	0.1111111111	0.1111111111	0	0
**FNDC11**	0.1428571429	0.1428571429	0.2222222222	0.2222222222	0.4583333333	0.6428571429
**FNDC3B**	0.1904761905	0.1904761905	0.1111111111	0.1111111111	1.529411765	1.714285714
**FNDC5**	0.04761904762	0.04761904762	0.1111111111	0.1111111111	0.325	0.4285714286
**FNDC7**	0.04761904762	0.04761904762	0.05555555556	0.05555555556	0.7	0.8571428571
**FNDC9**	0.04761904762	0.04761904762	0.05555555556	0.05555555556	0.7	0.8571428571
**FNIP1**	0.04761904762	0.04761904762	0	0	inf	4.7619E+31
**FNIP2**	0	0	0.05555555556	0.05555555556	0	0
**FNTA**	0.09523809524	0.09523809524	0.1666666667	0.1666666667	0.4210526316	0.5714285714
**FOCAD**	0.09523809524	0.09523809524	0.1111111111	0.1111111111	0.6842105263	0.8571428571
**OCAD-A**	0.04761904762	0.04761904762	0.05555555556	0.05555555556	0.7	0.8571428571
**FOPNL**	0.04761904762	0.04761904762	0	0	inf	4.7619E+31
**FOSB**	0.09523809524	0.09523809524	0.1111111111	0.1111111111	0.6842105263	0.8571428571
**FOSL2**	0	0	0.05555555556	0.05555555556	0	0
**FOXA2**	0.1904761905	0.1904761905	0.05555555556	0.05555555556	3.294117647	3.428571429
**FOXA3**	0.09523809524	0.09523809524	0.1111111111	0.1111111111	0.6842105263	0.8571428571
**FOXB1**	0.04761904762	0.04761904762	0.05555555556	0.05555555556	0.7	0.8571428571
**FOXB2**	0	0	0.05555555556	0.05555555556	0	0
**FOXC2**	0.04761904762	0.04761904762	0	0	inf	4.7619E+31
**OXC2-AS**	0.04761904762	0.04761904762	0	0	inf	4.7619E+31
**FOXD1**	0	0	0.05555555556	0.05555555556	0	0
**FOXD2**	0	0	0.05555555556	0.05555555556	0	0
**OXD2-AS**	0	0	0.05555555556	0.05555555556	0	0
**FOXD3**	0.04761904762	0.04761904762	0.05555555556	0.05555555556	0.7	0.8571428571
**OXD3-AS**	0.04761904762	0.04761904762	0.05555555556	0.05555555556	0.7	0.8571428571
**FOXD4**	0.04761904762	0.04761904762	0.05555555556	0.05555555556	0.7	0.8571428571

OXD4L	0	0	0.05555555556	0.05555555556	0	0
OXD4L	0.09523809524	0.09523809524	0.2222222222	0.2222222222	0.2894736842	0.4285714286
OXD4L	0.1904761905	0.1904761905	0.2222222222	0.2222222222	0.6470588235	0.8571428571
OXD4L	0.09523809524	0.09523809524	0.2222222222	0.2222222222	0.2894736842	0.4285714286
OXD4L	0.09523809524	0.09523809524	0.2222222222	0.2222222222	0.2894736842	0.4285714286
FOXE1	0.04761904762	0.04761904762	0	0	inf	4.7619E+31
FOXE3	0	0	0.05555555556	0.05555555556	0	0
FOXF1	0.04761904762	0.04761904762	0	0	inf	4.7619E+31
FOXH1	0.04761904762	0.04761904762	0.1666666667	0.1666666667	0.2	0.2857142857
FOXI1	0	0	0.05555555556	0.05555555556	0	0
FOXI2	0.2380952381	0.2380952381	0.05555555556	0.05555555556	4.375	4.285714286
FOXI3	0	0	0.05555555556	0.05555555556	0	0
FOXJ1	0.04761904762	0.04761904762	0	0	inf	4.7619E+31
FOXJ2	0.04761904762	0.04761904762	0.1111111111	0.1111111111	0.325	0.4285714286
FOXJ3	0.04761904762	0.04761904762	0.05555555556	0.05555555556	0.7	0.8571428571
FOXK1	0.04761904762	0.04761904762	0.1666666667	0.1666666667	0.2	0.2857142857
FOXK2	0	0	0	0		0.00001
FOXL1	0.04761904762	0.04761904762	0	0	inf	4.7619E+31
FOXL2	0.1428571429	0.1428571429	0.05555555556	0.05555555556	2.333333333	2.571428571
OXL2N	0.1428571429	0.1428571429	0.05555555556	0.05555555556	2.333333333	2.571428571
FOXM1	0.04761904762	0.04761904762	0.1666666667	0.1666666667	0.2	0.2857142857
FOXN1	0.04761904762	0.04761904762	0.05555555556	0.05555555556	0.7	0.8571428571
FOXN2	0	0	0.05555555556	0.05555555556	0	0
FOXN4	0	0	0.05555555556	0.05555555556	0	0
FOXO3	0	0	0	0		0.00001
FOXO3E	0.04761904762	0.04761904762	0.1111111111	0.1111111111	0.325	0.4285714286
FOXO6	0.04761904762	0.04761904762	0.05555555556	0.05555555556	0.7	0.8571428571
FOXP1	0.04761904762	0.04761904762	0	0	inf	4.7619E+31
OXP1-AS	0.04761904762	0.04761904762	0	0	inf	4.7619E+31
FOXP2	0.04761904762	0.04761904762	0.1111111111	0.1111111111	0.325	0.4285714286
FOXP4	0	0	0.05555555556	0.05555555556	0	0
OXP4-AS	0	0	0.05555555556	0.05555555556	0	0
OXRED	0	0	0.1111111111	0.1111111111	0	0
FOXS1	0.09523809524	0.09523809524	0.1111111111	0.1111111111	0.6842105263	0.8571428571
FPGS	0.09523809524	0.09523809524	0.05555555556	0.05555555556	1.473684211	1.714285714
FPGT	0.04761904762	0.04761904762	0.05555555556	0.05555555556	0.7	0.8571428571
GT-TNN	0.04761904762	0.04761904762	0.05555555556	0.05555555556	0.7	0.8571428571
FPR1	0.09523809524	0.09523809524	0.05555555556	0.05555555556	1.473684211	1.714285714
FPR2	0.09523809524	0.09523809524	0.05555555556	0.05555555556	1.473684211	1.714285714
FPR3	0.09523809524	0.09523809524	0.05555555556	0.05555555556	1.473684211	1.714285714
RA10AC	0.1428571429	0.1428571429	0	0	inf	1.42857E+32
FRAS1	0.04761904762	0.04761904762	0.05555555556	0.05555555556	0.7	0.8571428571
FRAT1	0.1428571429	0.1428571429	0	0	inf	1.42857E+32
FRAT2	0.1428571429	0.1428571429	0	0	inf	1.42857E+32
FREM1	0.1428571429	0.1428571429	0.05555555556	0.05555555556	2.333333333	2.571428571
FREM3	0	0	0.05555555556	0.05555555556	0	0
FRG1	0.04761904762	0.04761904762	0.05555555556	0.05555555556	0.7	0.8571428571
RG1-DT	0.04761904762	0.04761904762	0.05555555556	0.05555555556	0.7	0.8571428571
FRG1BP	0.09523809524	0.09523809524	0.05555555556	0.05555555556	1.473684211	1.714285714
FRG1DP	0.09523809524	0.09523809524	0.05555555556	0.05555555556	1.473684211	1.714285714
RG1HP	0.09523809524	0.09523809524	0.2222222222	0.2222222222	0.2894736842	0.4285714286

FRG1JP	0.09523809524	0.09523809524	0.2222222222	0.2222222222	0.2894736842	0.4285714286
FRG2	0.04761904762	0.04761904762	0.05555555556	0.05555555556	0.7	0.8571428571
FRG2B	0.1904761905	0.1904761905	0	0	inf	1.90476E+32
FRG2C	0.04761904762	0.04761904762	0	0	inf	4.7619E+31
FRG2DP	0.04761904762	0.04761904762	0	0	inf	4.7619E+31
FRG2EP	0.04761904762	0.04761904762	0	0	inf	4.7619E+31
FRG2KP	0.04761904762	0.04761904762	0	0	inf	4.7619E+31
FRGCA	0.04761904762	0.04761904762	0.1666666667	0.1666666667	0.2	0.2857142857
FRMD3	0	0	0.05555555556	0.05555555556	0	0
FRMD4A	0.09523809524	0.09523809524	0.1111111111	0.1111111111	0.6842105263	0.8571428571
FRMD4B	0.04761904762	0.04761904762	0	0	inf	4.7619E+31
FRMD6	0.04761904762	0.04761904762	0	0	inf	4.7619E+31
RMD6-A	0.04761904762	0.04761904762	0	0	inf	4.7619E+31
RMD6-A	0.04761904762	0.04761904762	0	0	inf	4.7619E+31
FRMPD1	0.09523809524	0.09523809524	0.05555555556	0.05555555556	1.473684211	1.714285714
FRMPD2	0.1428571429	0.1428571429	0.05555555556	0.05555555556	2.333333333	2.571428571
RMPD2	0.1428571429	0.1428571429	0.05555555556	0.05555555556	2.333333333	2.571428571
FRRS1	0.04761904762	0.04761904762	0.05555555556	0.05555555556	0.7	0.8571428571
FRRS1L	0.04761904762	0.04761904762	0.05555555556	0.05555555556	0.7	0.8571428571
FRS2	0.09523809524	0.09523809524	0.1111111111	0.1111111111	0.6842105263	0.8571428571
FRS3	0.04761904762	0.04761904762	0.05555555556	0.05555555556	0.7	0.8571428571
FRYL	0.04761904762	0.04761904762	0.1111111111	0.1111111111	0.325	0.4285714286
FRZB	0	0	0.05555555556	0.05555555556	0	0
FSBP	0.1904761905	0.1904761905	0.1111111111	0.1111111111	1.529411765	1.714285714
FSCB	0.04761904762	0.04761904762	0		inf	#DIV/0!
FSCN1	0.04761904762	0.04761904762	0.1666666667		0.2	#DIV/0!
FSCN2	0.04761904762	0.04761904762	0		inf	#DIV/0!
FSCN3	0.04761904762	0.04761904762	0.05555555556		0.7	#DIV/0!
FSD1	0.09523809524	0.09523809524	0.05555555556		1.473684211	#DIV/0!
FSD1L	0.04761904762	0.04761904762	0.05555555556		0.7	#DIV/0!
FSD2	0.04761904762	0.04761904762	0.05555555556		0.7	#DIV/0!
FSHR	0	0	0.05555555556		0	#DIV/0!
FSIP1	0	0	0			#DIV/0!
FSIP2	0	0	0.05555555556		0	#DIV/0!
SIP2-AS	0	0	0.05555555556		0	#DIV/0!
SIP2-AS	0	0	0.05555555556		0	#DIV/0!
FST	0.04761904762	0.04761904762	0.05555555556		0.7	#DIV/0!
FSTL1	0.09523809524	0.09523809524	0.05555555556		1.473684211	#DIV/0!
FSTL3	0.09523809524	0.09523809524	0.1111111111		0.6842105263	#DIV/0!
FSTL4	0.04761904762	0.04761904762	0		inf	#DIV/0!
FSTL5	0	0	0.05555555556		0	#DIV/0!
FTCD	0.04761904762	0.04761904762	0.1111111111		0.325	#DIV/0!
TCD-AS	0.04761904762	0.04761904762	0.1111111111		0.325	#DIV/0!
TCDNL	0	0	0.05555555556		0	#DIV/0!
FTL	0.09523809524	0.09523809524	0.1111111111		0.6842105263	#DIV/0!
FTLP10	0.04761904762	0.04761904762	0.05555555556		0.7	#DIV/0!
FTMT	0.04761904762	0.04761904762	0		inf	#DIV/0!
FTO	0.04761904762	0.04761904762	0.05555555556		0.7	#DIV/0!
FTO-IT1	0.04761904762	0.04761904762	0.05555555556		0.7	#DIV/0!
FTSJ3	0.04761904762	0.04761904762	0		inf	#DIV/0!
FUBP1	0.04761904762	0.04761904762	0.05555555556		0.7	#DIV/0!

FUBP3	0.09523809524	0.09523809524	0.05555555556		1.473684211	#DIV/0!
FUCA1	0.04761904762	0.04761904762	0.1111111111		0.325	#DIV/0!
UNDC2F	0	0	0.05555555556		0	#DIV/0!
FUOM	0.1904761905	0.1904761905	0		inf	#DIV/0!
FURIN	0.04761904762	0.04761904762	0.05555555556		0.7	#DIV/0!
FUS	0.04761904762	0.04761904762	0		inf	#DIV/0!
FUT1	0.09523809524	0.09523809524	0.1111111111		0.6842105263	#DIV/0!
FUT10	0.04761904762	0.04761904762	0.1111111111		0.325	#DIV/0!
FUT2	0.09523809524	0.09523809524	0.1111111111		0.6842105263	#DIV/0!
FUT3	0.1428571429	0.1428571429	0.05555555556		2.333333333	#DIV/0!
FUT5	0.1428571429	0.1428571429	0.05555555556		2.333333333	#DIV/0!
FUT6	0.1428571429	0.1428571429	0.05555555556		2.333333333	#DIV/0!
FUT7	0.1428571429	0.1428571429	0.05555555556		2.333333333	#DIV/0!
FUZ	0.09523809524	0.09523809524	0.1111111111		0.6842105263	#DIV/0!
FXN	0	0	0.05555555556		0	#DIV/0!
FXR1	0.1904761905	0.1904761905	0.05555555556		3.294117647	#DIV/0!
FXR2	0	0	0.05555555556		0	#DIV/0!
FXYD1	0.04761904762	0.04761904762	0.05555555556		0.7	#DIV/0!
FXYD3	0.04761904762	0.04761904762	0.05555555556		0.7	#DIV/0!
FXYD4	0.1428571429	0.1428571429	0.05555555556		2.333333333	#DIV/0!
FXYD5	0.04761904762	0.04761904762	0.05555555556		0.7	#DIV/0!
FXYD7	0.04761904762	0.04761904762	0.05555555556		0.7	#DIV/0!
FYB1	0.04761904762	0.04761904762	0.05555555556		0.7	#DIV/0!
FYB2	0.04761904762	0.04761904762	0.05555555556		0.7	#DIV/0!
FYTTD1	0.1428571429	0.1428571429	0.05555555556		2.333333333	#DIV/0!
FZD1	0.04761904762	0.04761904762	0.1111111111		0.325	#DIV/0!
FZD10	0.09523809524	0.09523809524	0.05555555556		1.473684211	#DIV/0!
ZD10-AS	0.09523809524	0.09523809524	0.05555555556		1.473684211	#DIV/0!
FZD2	0.09523809524	0.09523809524	0		inf	#DIV/0!
FZD3	0.04761904762	0.04761904762	0.1111111111		0.325	#DIV/0!
FZD5	0	0	0.05555555556		0	#DIV/0!
FZD6	0.1904761905	0.1904761905	0.1111111111		1.529411765	#DIV/0!
FZD7	0	0	0.05555555556		0	#DIV/0!
FZD8	0.09523809524	0.09523809524	0.1666666667		0.4210526316	#DIV/0!
FZD9	0.1428571429	0.1428571429	0.1111111111		1.083333333	#DIV/0!
FZR1	0.09523809524	0.09523809524	0.05555555556		1.473684211	#DIV/0!
G0S2	0.1428571429	0.1428571429	0		inf	#DIV/0!
G3BP2	0.04761904762	0.04761904762	0.05555555556		0.7	#DIV/0!
G6PC	0.04761904762	0.04761904762	0		inf	#DIV/0!
G6PC2	0.04761904762	0.04761904762	0.05555555556		0.7	#DIV/0!
G6PC3	0.09523809524	0.09523809524	0		inf	#DIV/0!
GAA	0.04761904762	0.04761904762	0		inf	#DIV/0!
GAB1	0	0	0.05555555556		0	#DIV/0!
GAB4	0.04761904762	0.04761904762	0		inf	#DIV/0!
ABARA	0	0	0.05555555556		0	#DIV/0!
BARAP	0.04761904762	0.04761904762	0.1111111111		0.325	#DIV/0!
BARAP	0.04761904762	0.04761904762	0		inf	#DIV/0!
BARAP	0.04761904762	0.04761904762	0.05555555556		0.7	#DIV/0!
GABBR2	0.04761904762	0.04761904762	0		inf	#DIV/0!
GABPA	0.04761904762	0.04761904762	0.1111111111		0.325	#DIV/0!
GABPB1	0.1428571429	0.1428571429	0.05555555556		2.333333333	#DIV/0!

ABPB1-A	0.1428571429	0.1428571429	0.05555555556		2.333333333	#DIV/0!
ABPB1-I	0.1428571429	0.1428571429	0.05555555556		2.333333333	#DIV/0!
GABPB2	0.1904761905	0.1904761905	0.05555555556		3.294117647	#DIV/0!
GABRA1	0.04761904762	0.04761904762	0.05555555556		0.7	#DIV/0!
GABRA2	0.04761904762	0.04761904762	0		inf	#DIV/0!
GABRA4	0.04761904762	0.04761904762	0		inf	#DIV/0!
GABRA6	0.04761904762	0.04761904762	0.05555555556		0.7	#DIV/0!
GABRB1	0.04761904762	0.04761904762	0.05555555556		0.7	#DIV/0!
GABRB2	0.04761904762	0.04761904762	0.05555555556		0.7	#DIV/0!
GABRD	0	0	0.1111111111		0	#DIV/0!
GABRG1	0.04761904762	0.04761904762	0		inf	#DIV/0!
GABRG2	0.04761904762	0.04761904762	0.05555555556		0.7	#DIV/0!
GABRP	0	0	0.05555555556		0	#DIV/0!
GABRR3	0.04761904762	0.04761904762	0.05555555556		0.7	#DIV/0!
GACAT1	0	0	0.05555555556		0	#DIV/0!
GACAT2	0.09523809524	0.09523809524	0		inf	#DIV/0!
GACAT3	0	0	0.05555555556		0	#DIV/0!
GAD1	0	0	0.05555555556		0	#DIV/0!
GAD2	0.09523809524	0.09523809524	0.1111111111		0.6842105263	#DIV/0!
GADD45A	0.04761904762	0.04761904762	0.05555555556		0.7	#DIV/0!
GADD45B	0.09523809524	0.09523809524	0.05555555556		1.473684211	#DIV/0!
GADD45G	0.04761904762	0.04761904762	0.05555555556		0.7	#DIV/0!
ADD45GI	0.1428571429	0.1428571429	0.1111111111		1.083333333	#DIV/0!
GADL1	0.04761904762	0.04761904762	0		inf	#DIV/0!
GAK	0.04761904762	0.04761904762	0		inf	#DIV/0!
GAL3ST	0.04761904762	0.04761904762	0.1111111111		0.325	#DIV/0!
GAL3ST	0	0	0.05555555556		0	#DIV/0!
GAL3ST	0.04761904762	0.04761904762	0.05555555556		0.7	#DIV/0!
GALE	0.04761904762	0.04761904762	0.1111111111		0.325	#DIV/0!
GALK1	0.04761904762	0.04761904762	0		inf	#DIV/0!
GALK2	0.04761904762	0.04761904762	0.05555555556		0.7	#DIV/0!
GALM	0	0	0.05555555556		0	#DIV/0!
GALNS	0.1904761905	0.1904761905	0		inf	#DIV/0!
GALNT1	0.09523809524	0.09523809524	0		inf	#DIV/0!
GALNT1	0.09523809524	0.09523809524	0.05555555556		1.473684211	#DIV/0!
GALNT1	0.04761904762	0.04761904762	0		inf	#DIV/0!
GALNT1	0	0	0.05555555556		0	#DIV/0!
GALNT1	0	0	0.05555555556		0	#DIV/0!
GALNT1	0.04761904762	0.04761904762	0		inf	#DIV/0!
GALNT1	0.09523809524	0.09523809524	0.1111111111		0.6842105263	#DIV/0!
GALNT2	0.1428571429	0.1428571429	0		inf	#DIV/0!
GALNT3	0	0	0.05555555556		0	#DIV/0!
GALNT4	0.04761904762	0.04761904762	0.05555555556		0.7	#DIV/0!
GALNT5	0	0	0.05555555556		0	#DIV/0!
GALNT6	0.09523809524	0.09523809524	0.05555555556		1.473684211	#DIV/0!
GALNT7	0	0	0.05555555556		0	#DIV/0!
GALNT8	0.04761904762	0.04761904762	0.1111111111		0.325	#DIV/0!
GALNT9	0.09523809524	0.09523809524	0.05555555556		1.473684211	#DIV/0!
GALNTL	0.09523809524	0.09523809524	0.05555555556		1.473684211	#DIV/0!
GALNTL	0	0	0.05555555556		0	#DIV/0!
LNTL6-A	0	0	0.05555555556		0	#DIV/0!

**GALP**	0.1428571429	0.1428571429	0.05555555556		2.333333333	#DIV/0!
**GALR1**	0.09523809524	0.09523809524	0.05555555556		1.473684211	#DIV/0!
**GALR2**	0.04761904762	0.04761904762	0		inf	#DIV/0!
**GALR3**	0	0	0.1111111111		0	#DIV/0!
**GALT**	0.09523809524	0.09523809524	0.05555555556		1.473684211	#DIV/0!
**GAMT**	0.09523809524	0.09523809524	0.1111111111		0.6842105263	#DIV/0!
**GAN**	0.04761904762	0.04761904762	0		inf	#DIV/0!
**GAP43**	0.09523809524	0.09523809524	0.05555555556		1.473684211	#DIV/0!
**GAPDH**	0.04761904762	0.04761904762	0.1111111111		0.325	#DIV/0!
**GAPDHS**	0.04761904762	0.04761904762	0.05555555556		0.7	#DIV/0!
**GAPLIN**	0.09523809524	0.09523809524	0		inf	#DIV/0!
**GAPT**	0.04761904762	0.04761904762	0		inf	#DIV/0!
**GAPVD1**	0.09523809524	0.09523809524	0.05555555556		1.473684211	#DIV/0!
**GAR1**	0	0	0.05555555556		0	#DIV/0!
**GAREM**	0.09523809524	0.09523809524	0		inf	#DIV/0!
**GARNL3**	0.09523809524	0.09523809524	0.05555555556		1.473684211	#DIV/0!
**GARS**	0.04761904762	0.04761904762	0.1666666667		0.2	#DIV/0!
**GARS-DT**	0.04761904762	0.04761904762	0.1666666667		0.2	#DIV/0!
**GART**	0.04761904762	0.04761904762	0.1111111111		0.325	#DIV/0!
**GAS1**	0	0	0.05555555556		0	#DIV/0!
**GAS1RR**	0	0	0.05555555556		0	#DIV/0!
**GAS2L1**	0.04761904762	0.04761904762	0.1111111111		0.325	#DIV/0!
**GAS2L1P**	0.04761904762	0.04761904762	0		inf	#DIV/0!
**GAS2L2**	0.09523809524	0.09523809524	0		inf	#DIV/0!
**GAS2L3**	0.04761904762	0.04761904762	0.05555555556		0.7	#DIV/0!
**GAS5**	0.1428571429	0.1428571429	0		inf	#DIV/0!
**GAS5-AS**	0.1428571429	0.1428571429	0		inf	#DIV/0!
**GAS7**	0.04761904762	0.04761904762	0.05555555556		0.7	#DIV/0!
**GAS8**	0.09523809524	0.09523809524	0		inf	#DIV/0!
**GAS8-AS**	0.09523809524	0.09523809524	0		inf	#DIV/0!
**GASAL1**	0.1904761905	0.1904761905	0.1111111111		1.529411765	#DIV/0!
**GASK1A**	0.04761904762	0.04761904762	0		inf	#DIV/0!
**GASK1B**	0	0	0.05555555556		0	#DIV/0!
**GAST**	0.09523809524	0.09523809524	0		inf	#DIV/0!
**GATA2**	0.09523809524	0.09523809524	0.05555555556		1.473684211	#DIV/0!
**GATA2-AS**	0.09523809524	0.09523809524	0.05555555556		1.473684211	#DIV/0!
**GATA3**	0.09523809524	0.09523809524	0.1111111111		0.6842105263	#DIV/0!
**GATA3-AS**	0.09523809524	0.09523809524	0.1111111111		0.6842105263	#DIV/0!
**GATA4**	0.09523809524	0.09523809524	0.1111111111		0.6842105263	#DIV/0!
**GATA5**	0.1428571429	0.1428571429	0.2222222222		0.4583333333	#DIV/0!
**GATA6**	0.09523809524	0.09523809524	0.05555555556		1.473684211	#DIV/0!
**GATA6-AS**	0.09523809524	0.09523809524	0.05555555556		1.473684211	#DIV/0!
**GATAD**	0.04761904762	0.04761904762	0.1111111111		0.325	#DIV/0!
**GATAD2**	0.1428571429	0.1428571429	0.1111111111		1.083333333	#DIV/0!
**GATAD2**	0.1904761905	0.1904761905	0.05555555556		3.294117647	#DIV/0!
**GATB**	0	0	0.05555555556		0	#DIV/0!
**GATC**	0	0	0.1111111111		0	#DIV/0!
**GATD1**	0.04761904762	0.04761904762	0		inf	#DIV/0!
**GATD3A**	0.04761904762	0.04761904762	0.1111111111		0.325	#DIV/0!
**GATD3B**	0.04761904762	0.04761904762	0.1111111111		0.325	#DIV/0!
**GATM**	0.04761904762	0.04761904762	0		inf	#DIV/0!

**GAU1**	0.04761904762	0.04761904762	0.1111111111		0.325	#DIV/0!
**GBA**	0.1904761905	0.1904761905	0.1111111111		1.529411765	#DIV/0!
**GBA2**	0.09523809524	0.09523809524	0.05555555556		1.473684211	#DIV/0!
**GBAP1**	0.1904761905	0.1904761905	0.1111111111		1.529411765	#DIV/0!
**GBAT2**	0.1904761905	0.1904761905	0.05555555556		3.294117647	#DIV/0!
**GBE1**	0.04761904762	0.04761904762	0		inf	#DIV/0!
**GBF1**	0.1428571429	0.1428571429	0.05555555556		2.333333333	#DIV/0!
**GBGT1**	0.04761904762	0.04761904762	0		inf	#DIV/0!
**GBP1**	0.04761904762	0.04761904762	0.05555555556		0.7	#DIV/0!
**GBP1P1**	0.04761904762	0.04761904762	0.05555555556		0.7	#DIV/0!
**GBP2**	0.04761904762	0.04761904762	0.05555555556		0.7	#DIV/0!
**GBP3**	0.04761904762	0.04761904762	0.05555555556		0.7	#DIV/0!
**GBP4**	0.04761904762	0.04761904762	0.05555555556		0.7	#DIV/0!
**GBP5**	0.04761904762	0.04761904762	0.05555555556		0.7	#DIV/0!
**GBP6**	0.04761904762	0.04761904762	0.05555555556		0.7	#DIV/0!
**GBP7**	0.04761904762	0.04761904762	0.05555555556		0.7	#DIV/0!
**GBX1**	0.09523809524	0.09523809524	0.05555555556		1.473684211	#DIV/0!
**GBX2**	0	0	0.05555555556		0	#DIV/0!
**GC**	0.04761904762	0.04761904762	0.05555555556		0.7	#DIV/0!
**GCA**	0	0	0.05555555556		0	#DIV/0!
**GCAT**	0	0	0.1111111111		0	#DIV/0!
**GCC1**	0.04761904762	0.04761904762	0.05555555556		0.7	#DIV/0!
**GCC2**	0	0	0.05555555556		0	#DIV/0!
**CC2-AS**	0	0	0.05555555556		0	#DIV/0!
**GCDH**	0.1428571429	0.1428571429	0.1111111111		1.083333333	#DIV/0!
**GCFC2**	0	0	0.05555555556		0	#DIV/0!
**GCG**	0	0	0.05555555556		0	#DIV/0!
**GCGR**	0.04761904762	0.04761904762	0		inf	#DIV/0!
**GCH1**	0.04761904762	0.04761904762	0		inf	#DIV/0!
**GCK**	0.04761904762	0.04761904762	0.1666666667		0.2	#DIV/0!
**GCLC**	0.04761904762	0.04761904762	0		inf	#DIV/0!
**GCLM**	0.04761904762	0.04761904762	0.05555555556		0.7	#DIV/0!
**GCM1**	0.04761904762	0.04761904762	0		inf	#DIV/0!
**GCM2**	0.04761904762	0.04761904762	0		inf	#DIV/0!
**GCN1**	0	0	0.1111111111		0	#DIV/0!
**GCNT1**	0	0	0.05555555556		0	#DIV/0!
**GCNT3**	0.04761904762	0.04761904762	0.05555555556		0.7	#DIV/0!
**GCNT4**	0.04761904762	0.04761904762	0		inf	#DIV/0!
**GCNT7**	0.09523809524	0.09523809524	0.1666666667		0.4210526316	#DIV/0!
**GCOM1**	0.04761904762	0.04761904762	0.05555555556		0.7	#DIV/0!
**GCSAM**	0.09523809524	0.09523809524	0.05555555556		1.473684211	#DIV/0!
**GCSAMI**	0.1428571429	0.1428571429	0		inf	#DIV/0!
**SAML-A**	0.1428571429	0.1428571429	0		inf	#DIV/0!
**GCSH**	0.09523809524	0.09523809524	0		inf	#DIV/0!
**GCSHP3**	0	0	0.05555555556		0	#DIV/0!
**GDA**	0	0	0.05555555556		0	#DIV/0!
**GDAP1**	0.1904761905	0.1904761905	0.1111111111		1.529411765	#DIV/0!
**GDAP1L**	0.1428571429	0.1428571429	0.1666666667		0.6666666667	#DIV/0!
**GDAP2**	0.04761904762	0.04761904762	0.05555555556		0.7	#DIV/0!
**GDE1**	0.04761904762	0.04761904762	0		inf	#DIV/0!
**GDF1**	0.1428571429	0.1428571429	0.1111111111		1.083333333	#DIV/0!

**GDF10**	0.1428571429	0.1428571429	0.05555555556		2.333333333	#DIV/0!
**GDF11**	0.04761904762	0.04761904762	0.05555555556		0.7	#DIV/0!
**GDF15**	0.1428571429	0.1428571429	0.1111111111		1.083333333	#DIV/0!
**GDF2**	0.1428571429	0.1428571429	0.05555555556		2.333333333	#DIV/0!
**GDF3**	0.04761904762	0.04761904762	0.1111111111		0.325	#DIV/0!
**GDF5**	0.09523809524	0.09523809524	0.1111111111		0.6842105263	#DIV/0!
**GDF5OS**	0.09523809524	0.09523809524	0.1111111111		0.6842105263	#DIV/0!
**GDF6**	0.1904761905	0.1904761905	0.1666666667		0.9411764706	#DIV/0!
**GDF7**	0	0	0.05555555556		0	#DIV/0!
**GDF9**	0.04761904762	0.04761904762	0		inf	#DIV/0!
**GDI2**	0.09523809524	0.09523809524	0.1111111111		0.6842105263	#DIV/0!
**GDNF**	0.04761904762	0.04761904762	0.05555555556		0.7	#DIV/0!
**DNF-AS**	0.04761904762	0.04761904762	0.05555555556		0.7	#DIV/0!
**GDPD1**	0.1428571429	0.1428571429	0		inf	#DIV/0!
**GDPD3**	0.04761904762	0.04761904762	0		inf	#DIV/0!
**GDPGP1**	0.04761904762	0.04761904762	0.05555555556		0.7	#DIV/0!
**GEM**	0.1904761905	0.1904761905	0.1111111111		1.529411765	#DIV/0!
**GEMIN2**	0.04761904762	0.04761904762	0.05555555556		0.7	#DIV/0!
**GEMIN5**	0	0	0.05555555556		0	#DIV/0!
**GEMIN6**	0	0	0.05555555556		0	#DIV/0!
**GEMIN7**	0.09523809524	0.09523809524	0.1111111111		0.6842105263	#DIV/0!
**MIN7-A**	0.09523809524	0.09523809524	0.1111111111		0.6842105263	#DIV/0!
**EMIN8P**	0.04761904762	0.04761904762	0.05555555556		0.7	#DIV/0!
**GEN1**	0	0	0.05555555556		0	#DIV/0!
**GET4**	0.04761904762	0.04761904762	0.1666666667		0.2	#DIV/0!
**GFAP**	0.1428571429	0.1428571429	0		inf	#DIV/0!
**GFER**	0.09523809524	0.09523809524	0.2222222222		0.2894736842	#DIV/0!
**GFI1**	0.04761904762	0.04761904762	0.05555555556		0.7	#DIV/0!
**GFI1B**	0.04761904762	0.04761904762	0		inf	#DIV/0!
**GFM1**	0.1428571429	0.1428571429	0.05555555556		2.333333333	#DIV/0!
**GFM2**	0.04761904762	0.04761904762	0.05555555556		0.7	#DIV/0!
**GFOD2**	0.1428571429	0.1428571429	0.05555555556		2.333333333	#DIV/0!
**GFPT1**	0	0	0.05555555556		0	#DIV/0!
**GFPT2**	0.09523809524	0.09523809524	0.05555555556		1.473684211	#DIV/0!
**GFRA1**	0.2380952381	0.2380952381	0		inf	#DIV/0!
**GFRA2**	0.09523809524	0.09523809524	0.1111111111		0.6842105263	#DIV/0!
**GFRA3**	0.04761904762	0.04761904762	0		inf	#DIV/0!
**GFRA4**	0.1904761905	0.1904761905	0.05555555556		3.294117647	#DIV/0!
**GFRAL**	0.04761904762	0.04761904762	0		inf	#DIV/0!
**GFY**	0.09523809524	0.09523809524	0.1111111111		0.6842105263	#DIV/0!
**GGA1**	0	0	0.1111111111		0	#DIV/0!
**GGA2**	0.04761904762	0.04761904762	0		inf	#DIV/0!
**GGA3**	0.04761904762	0.04761904762	0.05555555556		0.7	#DIV/0!
**GGCT**	0.04761904762	0.04761904762	0.1666666667		0.2	#DIV/0!
**GGCX**	0	0	0.05555555556		0	#DIV/0!
**GGH**	0.1428571429	0.1428571429	0.1666666667		0.6666666667	#DIV/0!
**GGN**	0.04761904762	0.04761904762	0.05555555556		0.7	#DIV/0!
**GGNBP1**	0.04761904762	0.04761904762	0		inf	#DIV/0!
**GGNBP2**	0.04761904762	0.04761904762	0		inf	#DIV/0!
**GGPS1**	0.1428571429	0.1428571429	0.05555555556		2.333333333	#DIV/0!
**GGT1**	0.04761904762	0.04761904762	0.1111111111		0.325	#DIV/0!

GGT2	0.04761904762	0.04761904762	0.05555555556		0.7	#DIV/0!
GGT3P	0.04761904762	0.04761904762	0.05555555556		0.7	#DIV/0!
GGT5	0.04761904762	0.04761904762	0.1111111111		0.325	#DIV/0!
GGT6	0	0	0.1111111111		0	#DIV/0!
GGT7	0.09523809524	0.09523809524	0.1111111111		0.6842105263	#DIV/0!
GGT8P	0.04761904762	0.04761904762	0.05555555556		0.7	#DIV/0!
GGTA1I	0.09523809524	0.09523809524	0.05555555556		1.473684211	#DIV/0!
GGTLC1	0.1904761905	0.1904761905	0.05555555556		3.294117647	#DIV/0!
GGTLC2	0.04761904762	0.04761904762	0.05555555556		0.7	#DIV/0!
GGTLC3	0.04761904762	0.04761904762	0.05555555556		0.7	#DIV/0!
GH1	0.04761904762	0.04761904762	0		inf	#DIV/0!
GH2	0.04761904762	0.04761904762	0		inf	#DIV/0!
GHDC	0.04761904762	0.04761904762	0		inf	#DIV/0!
GHET1	0.09523809524	0.09523809524	0.05555555556		1.473684211	#DIV/0!
GHITM	0.1428571429	0.1428571429	0		inf	#DIV/0!
GHR	0.04761904762	0.04761904762	0.05555555556		0.7	#DIV/0!
GHRH	0.09523809524	0.09523809524	0.1666666667		0.4210526316	#DIV/0!
GHRHR	0.04761904762	0.04761904762	0.1111111111		0.325	#DIV/0!
GHRL	0.09523809524	0.09523809524	0		inf	#DIV/0!
GHRLOS	0.09523809524	0.09523809524	0		inf	#DIV/0!
GHSR	0.1904761905	0.1904761905	0.1111111111		1.529411765	#DIV/0!
GID4	0.04761904762	0.04761904762	0.1111111111		0.325	#DIV/0!
GID8	0.1428571429	0.1428571429	0.2222222222		0.4583333333	#DIV/0!
GIGYF1	0.04761904762	0.04761904762	0.05555555556		0.7	#DIV/0!
GIGYF2	0	0	0.05555555556		0	#DIV/0!
GIHCG	0.1428571429	0.1428571429	0.05555555556		2.333333333	#DIV/0!
GIMAP1	0.09523809524	0.09523809524	0.05555555556		1.473684211	#DIV/0!
AP1-GIM	0.09523809524	0.09523809524	0.05555555556		1.473684211	#DIV/0!
GIMAP2	0.09523809524	0.09523809524	0.05555555556		1.473684211	#DIV/0!
GIMAP4	0.09523809524	0.09523809524	0.05555555556		1.473684211	#DIV/0!
GIMAP5	0.09523809524	0.09523809524	0.05555555556		1.473684211	#DIV/0!
GIMAP6	0.09523809524	0.09523809524	0.05555555556		1.473684211	#DIV/0!
GIMAP7	0.09523809524	0.09523809524	0.05555555556		1.473684211	#DIV/0!
GIMAP8	0.09523809524	0.09523809524	0.05555555556		1.473684211	#DIV/0!
GIMD1	0	0	0.05555555556		0	#DIV/0!
GIN1	0.04761904762	0.04761904762	0		inf	#DIV/0!
GINM1	0	0	0			#DIV/0!
GINS1	0.1904761905	0.1904761905	0.05555555556		3.294117647	#DIV/0!
GINS2	0.04761904762	0.04761904762	0		inf	#DIV/0!
GINS3	0.04761904762	0.04761904762	0		inf	#DIV/0!
GINS4	0.09523809524	0.09523809524	0.1666666667		0.4210526316	#DIV/0!
GIP	0.1428571429	0.1428571429	0		inf	#DIV/0!
GIPC1	0.1428571429	0.1428571429	0.1111111111		1.083333333	#DIV/0!
GIPC2	0.04761904762	0.04761904762	0.05555555556		0.7	#DIV/0!
GIPC3	0.09523809524	0.09523809524	0.05555555556		1.473684211	#DIV/0!
GIPR	0.09523809524	0.09523809524	0.1111111111		0.6842105263	#DIV/0!
GIT1	0	0	0.05555555556		0	#DIV/0!
GIT2	0	0	0.1111111111		0	#DIV/0!
GJA1	0	0	0.05555555556		0	#DIV/0!
GJA10	0	0	0			#DIV/0!
GJA3	0.04761904762	0.04761904762	0		inf	#DIV/0!

GJA4	0.04761904762	0.04761904762	0		inf	#DIV/0!
GJA5	0.1428571429	0.1428571429	0.05555555556		2.333333333	#DIV/0!
GJA8	0.1428571429	0.1428571429	0.05555555556		2.333333333	#DIV/0!
GJA9	0.04761904762	0.04761904762	0.05555555556		0.7	#DIV/0!
A9-MYC	0.04761904762	0.04761904762	0.05555555556		0.7	#DIV/0!
GJB2	0.04761904762	0.04761904762	0		inf	#DIV/0!
GJB3	0.04761904762	0.04761904762	0		inf	#DIV/0!
GJB4	0.04761904762	0.04761904762	0		inf	#DIV/0!
GJB5	0.04761904762	0.04761904762	0		inf	#DIV/0!
GJB6	0.04761904762	0.04761904762	0		inf	#DIV/0!
GJC1	0.09523809524	0.09523809524	0		inf	#DIV/0!
GJC2	0.1428571429	0.1428571429	0		inf	#DIV/0!
GJC3	0.04761904762	0.04761904762	0.05555555556		0.7	#DIV/0!
GJD3	0.09523809524	0.09523809524	0		inf	#DIV/0!
GJD4	0.09523809524	0.09523809524	0.1666666667		0.4210526316	#DIV/0!
GK2	0.04761904762	0.04761904762	0.05555555556		0.7	#DIV/0!
GK3P	0	0	0.1111111111		0	#DIV/0!
GK5	0.1904761905	0.1904761905	0.05555555556		3.294117647	#DIV/0!
GKAP1	0	0	0.05555555556		0	#DIV/0!
GKN1	0	0	0.05555555556		0	#DIV/0!
GKN2	0	0	0.05555555556		0	#DIV/0!
GMCL1	0	0	0.05555555556		0	#DIV/0!
GMCL2	0.09523809524	0.09523809524	0.05555555556		1.473684211	#DIV/0!
GMEB1	0.04761904762	0.04761904762	0.1111111111		0.325	#DIV/0!
GMEB2	0.1428571429	0.1428571429	0.2222222222		0.4583333333	#DIV/0!
GMFB	0.04761904762	0.04761904762	0		inf	#DIV/0!
GMFG	0.04761904762	0.04761904762	0.05555555556		0.7	#DIV/0!
GMIP	0.1428571429	0.1428571429	0.1111111111		1.083333333	#DIV/0!
GML	0.04761904762	0.04761904762	0.1666666667		0.2	#DIV/0!
GMNC	0.09523809524	0.09523809524	0.05555555556		1.473684211	#DIV/0!
GMNN	0	0	0.05555555556		0	#DIV/0!
GMPPA	0	0	0.05555555556		0	#DIV/0!
GMPPB	0.09523809524	0.09523809524	0		inf	#DIV/0!
GMPS	0.1904761905	0.1904761905	0.05555555556		3.294117647	#DIV/0!
GNA11	0.09523809524	0.09523809524	0.05555555556		1.473684211	#DIV/0!
GNA12	0.04761904762	0.04761904762	0.1666666667		0.2	#DIV/0!
GNA13	0.04761904762	0.04761904762	0		inf	#DIV/0!
GNA14	0	0	0.05555555556		0	#DIV/0!
NA14-AS	0	0	0.05555555556		0	#DIV/0!
GNA15	0.09523809524	0.09523809524	0.05555555556		1.473684211	#DIV/0!
GNAI1	0.04761904762	0.04761904762	0.1111111111		0.325	#DIV/0!
GNAI2	0.09523809524	0.09523809524	0		inf	#DIV/0!
GNAI3	0.04761904762	0.04761904762	0.05555555556		0.7	#DIV/0!
GNAL	0.09523809524	0.09523809524	0.05555555556		1.473684211	#DIV/0!
GNAO1	0.04761904762	0.04761904762	0		inf	#DIV/0!
GNAQ	0	0	0.05555555556		0	#DIV/0!
GNAS	0.1428571429	0.1428571429	0.2222222222		0.4583333333	#DIV/0!
NAS-AS	0.1428571429	0.1428571429	0.2222222222		0.4583333333	#DIV/0!
GNAT1	0.09523809524	0.09523809524	0		inf	#DIV/0!
GNAT2	0.04761904762	0.04761904762	0.05555555556		0.7	#DIV/0!
GNAT3	0.04761904762	0.04761904762	0.1111111111		0.325	#DIV/0!

GNAZ	0	0	0.05555555556		0	#DIV/0!
GNB1	0	0	0.1111111111		0	#DIV/0!
GNB1L	0.04761904762	0.04761904762	0.05555555556		0.7	#DIV/0!
GNB2	0.04761904762	0.04761904762	0.05555555556		0.7	#DIV/0!
GNB3	0.04761904762	0.04761904762	0.1111111111		0.325	#DIV/0!
GNB4	0.1904761905	0.1904761905	0.05555555556		3.294117647	#DIV/0!
GNB5	0.04761904762	0.04761904762	0.05555555556		0.7	#DIV/0!
GNE	0.09523809524	0.09523809524	0.05555555556		1.473684211	#DIV/0!
GNG10	0.04761904762	0.04761904762	0.05555555556		0.7	#DIV/0!
GNG11	0.04761904762	0.04761904762	0.1111111111		0.325	#DIV/0!
GNG12	0.04761904762	0.04761904762	0.05555555556		0.7	#DIV/0!
NG12-AS	0.04761904762	0.04761904762	0.05555555556		0.7	#DIV/0!
GNG13	0.04761904762	0.04761904762	0.2222222222		0.1375	#DIV/0!
GNG14	0.1428571429	0.1428571429	0.1111111111		1.083333333	#DIV/0!
GNG2	0.04761904762	0.04761904762	0		inf	#DIV/0!
GNG4	0.1428571429	0.1428571429	0.05555555556		2.333333333	#DIV/0!
GNG5	0.04761904762	0.04761904762	0.05555555556		0.7	#DIV/0!
GNG7	0.09523809524	0.09523809524	0.05555555556		1.473684211	#DIV/0!
GNG8	0.09523809524	0.09523809524	0.1111111111		0.6842105263	#DIV/0!
GNGT1	0.04761904762	0.04761904762	0.1111111111		0.325	#DIV/0!
GNGT2	0.1428571429	0.1428571429	0		inf	#DIV/0!
GNL2	0.04761904762	0.04761904762	0.05555555556		0.7	#DIV/0!
GNLY	0	0	0.05555555556		0	#DIV/0!
GNMT	0.04761904762	0.04761904762	0.05555555556		0.7	#DIV/0!
GNPAT	0.1428571429	0.1428571429	0		inf	#DIV/0!
GNPDA1	0.04761904762	0.04761904762	0		inf	#DIV/0!
GNPDA2	0.04761904762	0.04761904762	0.05555555556		0.7	#DIV/0!
NPNAT	0.04761904762	0.04761904762	0		inf	#DIV/0!
GNPTAI	0.04761904762	0.04761904762	0.05555555556		0.7	#DIV/0!
GNPTG	0.04761904762	0.04761904762	0.2222222222		0.1375	#DIV/0!
GNRH1	0.04761904762	0.04761904762	0.1111111111		0.325	#DIV/0!
GNRH2	0.1904761905	0.1904761905	0.05555555556		3.294117647	#DIV/0!
GNRHR	0.04761904762	0.04761904762	0.05555555556		0.7	#DIV/0!
GNS	0.1428571429	0.1428571429	0.05555555556		2.333333333	#DIV/0!
GOLGA1	0.09523809524	0.09523809524	0.05555555556		1.473684211	#DIV/0!
GOLGA2	0.09523809524	0.09523809524	0.05555555556		1.473684211	#DIV/0!
OLGA2P	0.04761904762	0.04761904762	0.05555555556		0.7	#DIV/0!
OLGA2P	0.04761904762	0.04761904762	0.05555555556		0.7	#DIV/0!
OLGA2I	0.04761904762	0.04761904762	0.05555555556		0.7	#DIV/0!
OLGA2I	0.09523809524	0.09523809524	0.2222222222		0.2894736842	#DIV/0!
OLGA2I	0.04761904762	0.04761904762	0.05555555556		0.7	#DIV/0!
OLGA2I	0.09523809524	0.09523809524	0		inf	#DIV/0!
GOLGA3	0.09523809524	0.09523809524	0.05555555556		1.473684211	#DIV/0!
GOLGA4	0.04761904762	0.04761904762	0		inf	#DIV/0!
OLGA6	0.04761904762	0.04761904762	0.05555555556		0.7	#DIV/0!
OLGA6	0.04761904762	0.04761904762	0.05555555556		0.7	#DIV/0!
OLGA6	0.04761904762	0.04761904762	0.05555555556		0.7	#DIV/0!
OLGA6	0.04761904762	0.04761904762	0.05555555556		0.7	#DIV/0!
OLGA6I	0.1904761905	0.1904761905	0		inf	#DIV/0!
OLGA6L	0.04761904762	0.04761904762	0.05555555556		0.7	#DIV/0!
LGA6L	0.04761904762	0.04761904762	0.05555555556		0.7	#DIV/0!

OLGA6I	0.04761904762	0.04761904762	0		inf	#DIV/0!
OLGA6L	0.1904761905	0.1904761905	0		inf	#DIV/0!
OLGA6I	0.04761904762	0.04761904762	0.05555555556		0.7	#DIV/0!
OLGA6I	0.04761904762	0.04761904762	0.05555555556		0.7	#DIV/0!
OLGA6L	0.04761904762	0.04761904762	0.05555555556		0.7	#DIV/0!
OLGA6I	0.2857142857	0.2857142857	0.1111111111		2.6	#DIV/0!
OLGA6I	0.04761904762	0.04761904762	0.05555555556		0.7	#DIV/0!
GOLGA7	0.09523809524	0.09523809524	0.1666666667		0.4210526316	#DIV/0!
OLGA7	0.1428571429	0.1428571429	0		inf	#DIV/0!
OLGA8C	0.2857142857	0.2857142857	0.1111111111		2.6	#DIV/0!
OLGA8I	0.1428571429	0.1428571429	0		inf	#DIV/0!
OLGA8I	0.04761904762	0.04761904762	0		inf	#DIV/0!
OLGA8I	0.09523809524	0.09523809524	0		inf	#DIV/0!
OLGA8	0.04761904762	0.04761904762	0		inf	#DIV/0!
GOLGB1	0.09523809524	0.09523809524	0.05555555556		1.473684211	#DIV/0!
GOLIM4	0.1428571429	0.1428571429	0.05555555556		2.333333333	#DIV/0!
GOLM1	0	0	0.05555555556		0	#DIV/0!
GOLPH3	0.04761904762	0.04761904762	0.05555555556		0.7	#DIV/0!
OLPH3	0.1904761905	0.1904761905	0.05555555556		3.294117647	#DIV/0!
GOLT1A	0.2380952381	0.2380952381	0		inf	#DIV/0!
GOLT1B	0.04761904762	0.04761904762	0.05555555556		0.7	#DIV/0!
GON4L	0.1904761905	0.1904761905	0.1111111111		1.529411765	#DIV/0!
GORAB	0.1428571429	0.1428571429	0.05555555556		2.333333333	#DIV/0!
ORAB-A	0.1428571429	0.1428571429	0.05555555556		2.333333333	#DIV/0!
ORASP	0.04761904762	0.04761904762	0		inf	#DIV/0!
ORASP	0	0	0.05555555556		0	#DIV/0!
GOSR1	0	0	0.05555555556		0	#DIV/0!
GOSR2	0.1428571429	0.1428571429	0		inf	#DIV/0!
GOT1	0.1428571429	0.1428571429	0		inf	#DIV/0!
GOT1L1	0.09523809524	0.09523809524	0.1111111111		0.6842105263	#DIV/0!
GOT2	0.04761904762	0.04761904762	0		inf	#DIV/0!
GP1BA	0	0	0.1111111111		0	#DIV/0!
GP1BB	0.04761904762	0.04761904762	0.05555555556		0.7	#DIV/0!
GP2	0.04761904762	0.04761904762	0		inf	#DIV/0!
GP5	0.09523809524	0.09523809524	0.05555555556		1.473684211	#DIV/0!
GP6	0.09523809524	0.09523809524	0.05555555556		1.473684211	#DIV/0!
GP9	0.09523809524	0.09523809524	0.05555555556		1.473684211	#DIV/0!
GPA33	0.1428571429	0.1428571429	0		inf	#DIV/0!
GPAA1	0.04761904762	0.04761904762	0.1666666667		0.2	#DIV/0!
GPAM	0.1428571429	0.1428571429	0.05555555556		2.333333333	#DIV/0!
GPANK1	0.04761904762	0.04761904762	0		inf	#DIV/0!
GPAT2	0	0	0.1111111111		0	#DIV/0!
GPAT3	0	0	0.05555555556		0	#DIV/0!
GPAT4	0.09523809524	0.09523809524	0.1666666667		0.4210526316	#DIV/0!
PATCH	0.04761904762	0.04761904762	0.05555555556		0.7	#DIV/0!
PATCH	0	0	0.05555555556		0	#DIV/0!
PATCH	0.1428571429	0.1428571429	0		inf	#DIV/0!
PATCH	0.04761904762	0.04761904762	0.1111111111		0.325	#DIV/0!
PATCH	0.1428571429	0.1428571429	0.1111111111		1.083333333	#DIV/0!
PATCH	0.09523809524	0.09523809524	0		inf	#DIV/0!
GPBAR1	0	0	0.05555555556		0	#DIV/0!

**GPBP1**	0.04761904762	0.04761904762	0		inf	#DIV/0!
**GPBP1L**	0.04761904762	0.04761904762	0.05555555556		0.7	#DIV/0!
**GPC1**	0.04761904762	0.04761904762	0.05555555556		0.7	#DIV/0!
**GPC2**	0.04761904762	0.04761904762	0.05555555556		0.7	#DIV/0!
**GPCPD1**	0.1904761905	0.1904761905	0.05555555556		3.294117647	#DIV/0!
**GPD1**	0.04761904762	0.04761904762	0.05555555556		0.7	#DIV/0!
**GPD1L**	0.09523809524	0.09523809524	0		inf	#DIV/0!
**GPD2**	0	0	0.05555555556		0	#DIV/0!
**GPER1**	0.04761904762	0.04761904762	0.1666666667		0.2	#DIV/0!
**GPI**	0.04761904762	0.04761904762	0.05555555556		0.7	#DIV/0!
**GPIHBP**	0.04761904762	0.04761904762	0.1666666667		0.2	#DIV/0!
**GPLD1**	0	0	0.05555555556		0	#DIV/0!
**GPM6A**	0	0	0.05555555556		0	#DIV/0!
**GPN2**	0.04761904762	0.04761904762	0.1111111111		0.325	#DIV/0!
**GPN3**	0	0	0.1111111111		0	#DIV/0!
**GPNMB**	0.04761904762	0.04761904762	0.1666666667		0.2	#DIV/0!
**GPR1**	0	0	0.05555555556		0	#DIV/0!
**GPR1-AS**	0	0	0.05555555556		0	#DIV/0!
**GPR107**	0.09523809524	0.09523809524	0.05555555556		1.473684211	#DIV/0!
**GPR108**	0.1428571429	0.1428571429	0.05555555556		2.333333333	#DIV/0!
**GPR137B**	0.1428571429	0.1428571429	0		inf	#DIV/0!
**GPR137C**	0.04761904762	0.04761904762	0		inf	#DIV/0!
**GPR139**	0.04761904762	0.04761904762	0		inf	#DIV/0!
**GPR141**	0.04761904762	0.04761904762	0.2222222222		0.1375	#DIV/0!
**GPR142**	0.04761904762	0.04761904762	0		inf	#DIV/0!
**GPR146**	0.04761904762	0.04761904762	0.1666666667		0.2	#DIV/0!
**GPR148**	0	0	0.05555555556		0	#DIV/0!
**GPR149**	0.1904761905	0.1904761905	0.05555555556		3.294117647	#DIV/0!
**GPR15**	0.04761904762	0.04761904762	0.05555555556		0.7	#DIV/0!
**GPR150**	0.04761904762	0.04761904762	0		inf	#DIV/0!
**GPR153**	0	0	0.05555555556		0	#DIV/0!
**GPR155**	0	0	0.1111111111		0	#DIV/0!
**GPR156**	0.09523809524	0.09523809524	0.05555555556		1.473684211	#DIV/0!
**GPR157**	0	0	0.05555555556		0	#DIV/0!
**GPR158**	0.09523809524	0.09523809524	0.1111111111		0.6842105263	#DIV/0!
**PR158-A**	0.09523809524	0.09523809524	0.1111111111		0.6842105263	#DIV/0!
**GPR160**	0.1904761905	0.1904761905	0.05555555556		3.294117647	#DIV/0!
**GPR161**	0.1428571429	0.1428571429	0		inf	#DIV/0!
**GPR162**	0.04761904762	0.04761904762	0.1111111111		0.325	#DIV/0!
**GPR17**	0	0	0.05555555556		0	#DIV/0!
**GPR171**	0.1904761905	0.1904761905	0.1111111111		1.529411765	#DIV/0!
**GPR176**	0	0	0			#DIV/0!
**GPR182**	0.1428571429	0.1428571429	0.05555555556		2.333333333	#DIV/0!
**GPR19**	0.04761904762	0.04761904762	0.05555555556		0.7	#DIV/0!
**GPR20**	0.04761904762	0.04761904762	0.2222222222		0.1375	#DIV/0!
**GPR21**	0.09523809524	0.09523809524	0.05555555556		1.473684211	#DIV/0!
**GPR22**	0.04761904762	0.04761904762	0.05555555556		0.7	#DIV/0!
**GPR25**	0.1428571429	0.1428571429	0		inf	#DIV/0!
**GPR26**	0.2380952381	0.2380952381	0.05555555556		4.375	#DIV/0!
**GPR27**	0.04761904762	0.04761904762	0		inf	#DIV/0!
**GPR3**	0.04761904762	0.04761904762	0.1111111111		0.325	#DIV/0!

GPR32	0.09523809524	0.09523809524	0.05555555556		1.473684211	#DIV/0!
GPR33	0	0	0			#DIV/0!
GPR35	0	0	0.05555555556		0	#DIV/0!
GPR37	0.04761904762	0.04761904762	0.05555555556		0.7	#DIV/0!
GPR37L1	0.1428571429	0.1428571429	0		inf	#DIV/0!
GPR39	0	0	0.05555555556		0	#DIV/0!
GPR4	0.09523809524	0.09523809524	0.1111111111		0.6842105263	#DIV/0!
GPR42	0.04761904762	0.04761904762	0.05555555556		0.7	#DIV/0!
GPR45	0	0	0.05555555556		0	#DIV/0!
GPR52	0.1428571429	0.1428571429	0		inf	#DIV/0!
GPR55	0	0	0.05555555556		0	#DIV/0!
GPR6	0	0	0			#DIV/0!
GPR61	0.04761904762	0.04761904762	0.05555555556		0.7	#DIV/0!
GPR62	0.04761904762	0.04761904762	0		inf	#DIV/0!
GPR75	0.04761904762	0.04761904762	0.05555555556		0.7	#DIV/0!
PR75-AS	0.04761904762	0.04761904762	0.05555555556		0.7	#DIV/0!
GPR84	0.04761904762	0.04761904762	0.05555555556		0.7	#DIV/0!
GPR85	0.04761904762	0.04761904762	0.05555555556		0.7	#DIV/0!
GPR87	0.1904761905	0.1904761905	0.1111111111		1.529411765	#DIV/0!
GPR88	0.04761904762	0.04761904762	0.05555555556		0.7	#DIV/0!
GPR89A	0.1428571429	0.1428571429	0.05555555556		2.333333333	#DIV/0!
GPR89B	0.1428571429	0.1428571429	0.05555555556		2.333333333	#DIV/0!
GPRACR	0.09523809524	0.09523809524	0.05555555556		1.473684211	#DIV/0!
GPRC5A	0.04761904762	0.04761904762	0.05555555556		0.7	#DIV/0!
GPRC5B	0.04761904762	0.04761904762	0		inf	#DIV/0!
GPRC5C	0.04761904762	0.04761904762	0		inf	#DIV/0!
GPRC5D	0.04761904762	0.04761904762	0.05555555556		0.7	#DIV/0!
RC5D-A	0.04761904762	0.04761904762	0.05555555556		0.7	#DIV/0!
GPRIN1	0.09523809524	0.09523809524	0.05555555556		1.473684211	#DIV/0!
GPRIN2	0.1428571429	0.1428571429	0.05555555556		2.333333333	#DIV/0!
GPRIN3	0	0	0.05555555556		0	#DIV/0!
GPS1	0.04761904762	0.04761904762	0		inf	#DIV/0!
GPS2	0	0	0.05555555556		0	#DIV/0!
GPSM1	0.1428571429	0.1428571429	0.05555555556		2.333333333	#DIV/0!
GPSM2	0.04761904762	0.04761904762	0.05555555556		0.7	#DIV/0!
GPSM3	0.04761904762	0.04761904762	0		inf	#DIV/0!
GPT	0.04761904762	0.04761904762	0.1666666667		0.2	#DIV/0!
GPT2	0.04761904762	0.04761904762	0		inf	#DIV/0!
GPX1	0.1428571429	0.1428571429	0		inf	#DIV/0!
GPX4	0.09523809524	0.09523809524	0.1111111111		0.6842105263	#DIV/0!
GPX7	0.04761904762	0.04761904762	0.05555555556		0.7	#DIV/0!
GPX8	0.04761904762	0.04761904762	0.05555555556		0.7	#DIV/0!
RAMD1	0.04761904762	0.04761904762	0.05555555556		0.7	#DIV/0!
RAMD1	0.09523809524	0.09523809524	0.05555555556		1.473684211	#DIV/0!
RAMD2	0.04761904762	0.04761904762	0.05555555556		0.7	#DIV/0!
RAMD2	0.04761904762	0.04761904762	0		inf	#DIV/0!
GRAMD4	0	0	0.1111111111		0	#DIV/0!
GRAP	0.04761904762	0.04761904762	0.1111111111		0.325	#DIV/0!
GRAP2	0	0	0.1111111111		0	#DIV/0!
GRAPL	0.04761904762	0.04761904762	0.1111111111		0.325	#DIV/0!
GRASP	0.04761904762	0.04761904762	0.05555555556		0.7	#DIV/0!

RASP-A	0.04761904762	0.04761904762	0.05555555556		0.7	#DIV/0!
GRB10	0.1428571429	0.1428571429	0.2222222222		0.4583333333	#DIV/0!
GRB14	0	0	0.05555555556		0	#DIV/0!
GRB2	0.04761904762	0.04761904762	0.05555555556		0.7	#DIV/0!
GRB7	0.09523809524	0.09523809524	0		inf	#DIV/0!
GREB1	0	0	0.05555555556		0	#DIV/0!
GREB1L	0.04761904762	0.04761904762	0.1111111111		0.325	#DIV/0!
GREM2	0.1428571429	0.1428571429	0		inf	#DIV/0!
GRHL1	0	0	0.05555555556		0	#DIV/0!
GRHL2	0.1904761905	0.1904761905	0.1111111111		1.529411765	#DIV/0!
GRHL3	0.04761904762	0.04761904762	0.1111111111		0.325	#DIV/0!
GRHPR	0.09523809524	0.09523809524	0.05555555556		1.473684211	#DIV/0!
GRIA2	0	0	0.05555555556		0	#DIV/0!
GRID1	0.1428571429	0.1428571429	0.05555555556		2.333333333	#DIV/0!
RID1-AS	0.1428571429	0.1428571429	0		inf	#DIV/0!
GRID2	0	0	0.05555555556		0	#DIV/0!
GRID2IP	0.04761904762	0.04761904762	0.1666666667		0.2	#DIV/0!
GRIFIN	0.04761904762	0.04761904762	0.1666666667		0.2	#DIV/0!
GRIK1	0.04761904762	0.04761904762	0.05555555556		0.7	#DIV/0!
RIK1-AS	0.04761904762	0.04761904762	0.05555555556		0.7	#DIV/0!
RIK1-AS	0.04761904762	0.04761904762	0.05555555556		0.7	#DIV/0!
GRIK3	0.04761904762	0.04761904762	0		inf	#DIV/0!
GRIK5	0.04761904762	0.04761904762	0.05555555556		0.7	#DIV/0!
GRIN1	0.1428571429	0.1428571429	0.05555555556		2.333333333	#DIV/0!
GRIN2A	0.04761904762	0.04761904762	0		inf	#DIV/0!
GRIN2B	0.04761904762	0.04761904762	0.05555555556		0.7	#DIV/0!
GRIN2C	0.04761904762	0.04761904762	0		inf	#DIV/0!
GRIN2D	0.09523809524	0.09523809524	0.1111111111		0.6842105263	#DIV/0!
GRIN3A	0.04761904762	0.04761904762	0		inf	#DIV/0!
GRIN3B	0.09523809524	0.09523809524	0.1111111111		0.6842105263	#DIV/0!
GRINA	0.04761904762	0.04761904762	0.1666666667		0.2	#DIV/0!
GRIP1	0.09523809524	0.09523809524	0.05555555556		1.473684211	#DIV/0!
GRIP2	0.04761904762	0.04761904762	0		inf	#DIV/0!
GRK3	0.04761904762	0.04761904762	0.05555555556		0.7	#DIV/0!
GRK4	0.04761904762	0.04761904762	0		inf	#DIV/0!
GRK5	0.2380952381	0.2380952381	0		inf	#DIV/0!
GRK6	0.09523809524	0.09523809524	0.05555555556		1.473684211	#DIV/0!
GRK7	0.1904761905	0.1904761905	0.05555555556		3.294117647	#DIV/0!
GRM2	0.04761904762	0.04761904762	0		inf	#DIV/0!
GRM3	0.09523809524	0.09523809524	0.05555555556		1.473684211	#DIV/0!
GRM4	0.04761904762	0.04761904762	0		inf	#DIV/0!
GRM6	0.09523809524	0.09523809524	0.05555555556		1.473684211	#DIV/0!
GRM7	0.04761904762	0.04761904762	0		inf	#DIV/0!
RM7-AS	0.04761904762	0.04761904762	0		inf	#DIV/0!
RM7-AS	0.04761904762	0.04761904762	0		inf	#DIV/0!
RM7-AS	0.04761904762	0.04761904762	0		inf	#DIV/0!
GRM8	0.04761904762	0.04761904762	0.05555555556		0.7	#DIV/0!
GRN	0.09523809524	0.09523809524	0		inf	#DIV/0!
GRP	0.09523809524	0.09523809524	0		inf	#DIV/0!
GRSF1	0.09523809524	0.09523809524	0.05555555556		1.473684211	#DIV/0!
GRWD1	0.09523809524	0.09523809524	0.1111111111		0.6842105263	#DIV/0!

1-124K5	0.04761904762	0.04761904762	0.1111111111		0.325	#DIV/0!
1-124K5	0.04761904762	0.04761904762	0.1111111111		0.325	#DIV/0!
S1-24F4	0.09523809524	0.09523809524	0.1111111111		0.6842105263	#DIV/0!
1-279B7	0.1428571429	0.1428571429	0.05555555556		2.333333333	#DIV/0!
GSAP	0.04761904762	0.04761904762	0.1111111111		0.325	#DIV/0!
GSC2	0.04761904762	0.04761904762	0.05555555556		0.7	#DIV/0!
GSDMA	0.09523809524	0.09523809524	0		inf	#DIV/0!
GSDMB	0.09523809524	0.09523809524	0		inf	#DIV/0!
GSDMC	0.04761904762	0.04761904762	0.1111111111		0.325	#DIV/0!
GSDMD	0.04761904762	0.04761904762	0.1666666667		0.2	#DIV/0!
GSDME	0.04761904762	0.04761904762	0.2222222222		0.1375	#DIV/0!
GSE1	0.04761904762	0.04761904762	0		inf	#DIV/0!
GSG1	0.04761904762	0.04761904762	0.05555555556		0.7	#DIV/0!
GSG1L	0.04761904762	0.04761904762	0		inf	#DIV/0!
GSK3A	0.04761904762	0.04761904762	0.05555555556		0.7	#DIV/0!
GSK3B	0.09523809524	0.09523809524	0.05555555556		1.473684211	#DIV/0!
GSN	0.09523809524	0.09523809524	0.05555555556		1.473684211	#DIV/0!
GSN-AS1	0.09523809524	0.09523809524	0.05555555556		1.473684211	#DIV/0!
GSPT1	0.04761904762	0.04761904762	0		inf	#DIV/0!
GSR	0.04761904762	0.04761904762	0.1111111111		0.325	#DIV/0!
GSS	0.09523809524	0.09523809524	0.1111111111		0.6842105263	#DIV/0!
GSTA1	0.04761904762	0.04761904762	0		inf	#DIV/0!
GSTA2	0.04761904762	0.04761904762	0		inf	#DIV/0!
GSTA3	0.04761904762	0.04761904762	0		inf	#DIV/0!
GSTA4	0.04761904762	0.04761904762	0		inf	#DIV/0!
GSTA5	0.04761904762	0.04761904762	0		inf	#DIV/0!
GSTA7P	0.04761904762	0.04761904762	0		inf	#DIV/0!
GSTCD	0	0	0.05555555556		0	#DIV/0!
GSTK1	0.04761904762	0.04761904762	0.05555555556		0.7	#DIV/0!
GSTM1	0.04761904762	0.04761904762	0.05555555556		0.7	#DIV/0!
GSTM2	0.04761904762	0.04761904762	0.05555555556		0.7	#DIV/0!
GSTM2P	0	0	0			#DIV/0!
GSTM3	0.04761904762	0.04761904762	0.05555555556		0.7	#DIV/0!
GSTM4	0.04761904762	0.04761904762	0.05555555556		0.7	#DIV/0!
GSTM5	0.04761904762	0.04761904762	0.05555555556		0.7	#DIV/0!
GSTO1	0.1428571429	0.1428571429	0.05555555556		2.333333333	#DIV/0!
GSTO2	0.1428571429	0.1428571429	0.05555555556		2.333333333	#DIV/0!
GSTT1	0.04761904762	0.04761904762	0.1111111111		0.325	#DIV/0!
STT1-AS	0.04761904762	0.04761904762	0.1111111111		0.325	#DIV/0!
GSTT2	0.04761904762	0.04761904762	0.1111111111		0.325	#DIV/0!
GSTT2B	0.04761904762	0.04761904762	0.1111111111		0.325	#DIV/0!
GSTT4	0.04761904762	0.04761904762	0.1111111111		0.325	#DIV/0!
GSTTP2	0.04761904762	0.04761904762	0.1111111111		0.325	#DIV/0!
GSX2	0.2380952381	0.2380952381	0.05555555556		4.375	#DIV/0!
GTDC1	0	0	0.05555555556		0	#DIV/0!
GTF2A1L	0	0	0.05555555556		0	#DIV/0!
GTF2A2	0.04761904762	0.04761904762	0.05555555556		0.7	#DIV/0!
GTF2B	0.04761904762	0.04761904762	0.05555555556		0.7	#DIV/0!
GTF2E1	0.09523809524	0.09523809524	0.05555555556		1.473684211	#DIV/0!
GTF2E2	0.04761904762	0.04761904762	0.1111111111		0.325	#DIV/0!
GTF2F1	0.1428571429	0.1428571429	0.05555555556		2.333333333	#DIV/0!

GTF2H2	0.09523809524	0.09523809524	0.1666666667		0.4210526316	#DIV/0!
GTF2H2I	0.09523809524	0.09523809524	0.1666666667		0.4210526316	#DIV/0!
GTF2H2C	0.09523809524	0.09523809524	0.1666666667		0.4210526316	#DIV/0!
TF2H2C	0.09523809524	0.09523809524	0.1666666667		0.4210526316	#DIV/0!
GTF2H3	0.04761904762	0.04761904762	0.1111111111		0.325	#DIV/0!
GTF2I	0.1428571429	0.1428571429	0.1666666667		0.6666666667	#DIV/0!
GTF2IP1	0.1428571429	0.1428571429	0.1666666667		0.6666666667	#DIV/0!
GTF2IP1	0	0	0.05555555556		0	#DIV/0!
GTF2IP2	0.1428571429	0.1428571429	0.05555555556		2.333333333	#DIV/0!
GTF2IP2	0.04761904762	0.04761904762	0.1111111111		0.325	#DIV/0!
GTF2IP4	0.1428571429	0.1428571429	0.1666666667		0.6666666667	#DIV/0!
GTF2IP7	0.1428571429	0.1428571429	0.1111111111		1.083333333	#DIV/0!
TF2IRD	0.1428571429	0.1428571429	0.1111111111		1.083333333	#DIV/0!
TF2IRD1	0.04761904762	0.04761904762	0.1111111111		0.325	#DIV/0!
TF2IRD	0.1428571429	0.1428571429	0.1666666667		0.6666666667	#DIV/0!
TF2IRD2	0.1428571429	0.1428571429	0.1666666667		0.6666666667	#DIV/0!
GTF3C1	0.04761904762	0.04761904762	0		inf	#DIV/0!
GTF3C3	0	0	0.05555555556		0	#DIV/0!
GTF3C4	0.04761904762	0.04761904762	0		inf	#DIV/0!
GTF3C5	0.04761904762	0.04761904762	0		inf	#DIV/0!
GTF3C6	0	0	0			#DIV/0!
GTPBP1	0.04761904762	0.04761904762	0.1111111111		0.325	#DIV/0!
GTPBP10	0.09523809524	0.09523809524	0.1111111111		0.6842105263	#DIV/0!
GTPBP2	0.04761904762	0.04761904762	0.05555555556		0.7	#DIV/0!
GTPBP3	0.1428571429	0.1428571429	0.1111111111		1.083333333	#DIV/0!
GTPBP4	0.04761904762	0.04761904762	0.1111111111		0.325	#DIV/0!
GTPBP8	0.09523809524	0.09523809524	0.05555555556		1.473684211	#DIV/0!
GTSE1	0	0	0.1111111111		0	#DIV/0!
TSE1-D	0	0	0.1111111111		0	#DIV/0!
GTSF1	0.04761904762	0.04761904762	0.05555555556		0.7	#DIV/0!
GTSF1L	0.1428571429	0.1428571429	0.1111111111		1.083333333	#DIV/0!
GUCA1A	0.04761904762	0.04761904762	0.05555555556		0.7	#DIV/0!
GUCA1B	0.04761904762	0.04761904762	0.05555555556		0.7	#DIV/0!
GUCA1C	0.09523809524	0.09523809524	0.05555555556		1.473684211	#DIV/0!
GUCA2A	0.04761904762	0.04761904762	0.05555555556		0.7	#DIV/0!
GUCA2B	0.04761904762	0.04761904762	0.05555555556		0.7	#DIV/0!
GUCD1	0.04761904762	0.04761904762	0.1111111111		0.325	#DIV/0!
GUCY1A	0	0	0.1111111111		0	#DIV/0!
GUCY1B	0	0	0.1111111111		0	#DIV/0!
GUCY2C	0.04761904762	0.04761904762	0.1111111111		0.325	#DIV/0!
GUCY2D	0	0	0.05555555556		0	#DIV/0!
UCY2G	0.1428571429	0.1428571429	0.05555555556		2.333333333	#DIV/0!
GUF1	0.04761904762	0.04761904762	0.05555555556		0.7	#DIV/0!
GUK1	0.1428571429	0.1428571429	0		inf	#DIV/0!
GULP1	0	0	0.1666666667		0	#DIV/0!
GUSB	0.04761904762	0.04761904762	0.1111111111		0.325	#DIV/0!
GUSBP1	0.04761904762	0.04761904762	0.1111111111		0.325	#DIV/0!
GUSBP1	0.1428571429	0.1428571429	0.1666666667		0.6666666667	#DIV/0!
GUSBP3	0.09523809524	0.09523809524	0.1666666667		0.4210526316	#DIV/0!
GUSBP4	0.04761904762	0.04761904762	0		inf	#DIV/0!
GUSBP5	0	0	0.05555555556		0	#DIV/0!

GUSBP9	0.09523809524	0.09523809524	0.1666666667		0.4210526316	#DIV/0!
GVINP1	0.04761904762	0.04761904762	0		inf	#DIV/0!
GXYLT1	0.04761904762	0.04761904762	0.1111111111		0.325	#DIV/0!
XYLT1I	0.1904761905	0.1904761905	0.05555555556		3.294117647	#DIV/0!
GXYLT2	0.04761904762	0.04761904762	0		inf	#DIV/0!
GYG1	0.1428571429	0.1428571429	0.1111111111		1.083333333	#DIV/0!
GYPA	0	0	0.05555555556		0	#DIV/0!
GYPB	0	0	0.05555555556		0	#DIV/0!
GYPC	0	0	0.05555555556		0	#DIV/0!
GYPE	0	0	0.05555555556		0	#DIV/0!
GYS1	0.09523809524	0.09523809524	0.1111111111		0.6842105263	#DIV/0!
GYS2	0.04761904762	0.04761904762	0.05555555556		0.7	#DIV/0!
GZF1	0.1904761905	0.1904761905	0.05555555556		3.294117647	#DIV/0!
GZMA	0.04761904762	0.04761904762	0.05555555556		0.7	#DIV/0!
GZMK	0.04761904762	0.04761904762	0.05555555556		0.7	#DIV/0!
GZMM	0.09523809524	0.09523809524	0.1111111111		0.6842105263	#DIV/0!
H19	0.04761904762	0.04761904762	0		inf	#DIV/0!
H1F0	0	0	0.1111111111		0	#DIV/0!
H1FNT	0.04761904762	0.04761904762	0.05555555556		0.7	#DIV/0!
H1FOO	0.09523809524	0.09523809524	0.05555555556		1.473684211	#DIV/0!
H1FX	0.09523809524	0.09523809524	0.05555555556		1.473684211	#DIV/0!
I1FX-AS	0.09523809524	0.09523809524	0.05555555556		1.473684211	#DIV/0!
H2AFJ	0.04761904762	0.04761904762	0.1111111111		0.325	#DIV/0!
H2AFV	0.04761904762	0.04761904762	0.2222222222		0.1375	#DIV/0!
H2AFY	0.04761904762	0.04761904762	0		inf	#DIV/0!
H2AFY2	0.09523809524	0.09523809524	0		inf	#DIV/0!
H2AFZ	0	0	0.05555555556		0	#DIV/0!
H2BFS	0.04761904762	0.04761904762	0.1666666667		0.2	#DIV/0!
H3.Y	0.04761904762	0.04761904762	0.05555555556		0.7	#DIV/0!
H3F3A	0.1428571429	0.1428571429	0.05555555556		2.333333333	#DIV/0!
I3F3AP4	0.1428571429	0.1428571429	0.1666666667		0.6666666667	#DIV/0!
H3F3B	0.04761904762	0.04761904762	0		inf	#DIV/0!
H3F3C	0.1428571429	0.1428571429	0.1111111111		1.083333333	#DIV/0!
H6PD	0	0	0.05555555556		0	#DIV/0!
HAAO	0	0	0.05555555556		0	#DIV/0!
HABP2	0.1428571429	0.1428571429	0.05555555556		2.333333333	#DIV/0!
HABP4	0.04761904762	0.04761904762	0.05555555556		0.7	#DIV/0!
HACD1	0.09523809524	0.09523809524	0.1111111111		0.6842105263	#DIV/0!
HACD2	0.04761904762	0.04761904762	0.05555555556		0.7	#DIV/0!
HACD3	0.04761904762	0.04761904762	0.05555555556		0.7	#DIV/0!
HACD4	0.09523809524	0.09523809524	0.1111111111		0.6842105263	#DIV/0!
HACL1	0.04761904762	0.04761904762	0		inf	#DIV/0!
HADH	0	0	0.05555555556		0	#DIV/0!
HAGH	0.09523809524	0.09523809524	0.2222222222		0.2894736842	#DIV/0!
HAGHL	0.04761904762	0.04761904762	0.2222222222		0.1375	#DIV/0!
HAL	0.04761904762	0.04761904762	0.05555555556		0.7	#DIV/0!
HAMP	0.04761904762	0.04761904762	0.05555555556		0.7	#DIV/0!
HAND2	0	0	0.05555555556		0	#DIV/0!
AND2-AS	0	0	0.05555555556		0	#DIV/0!
HAO1	0.1904761905	0.1904761905	0.1111111111		1.529411765	#DIV/0!
HAO2	0.04761904762	0.04761904762	0.05555555556		0.7	#DIV/0!

IAO2-IT	0.04761904762	0.04761904762	0.05555555556		0.7	#DIV/0!
HAP1	0.09523809524	0.09523809524	0		inf	#DIV/0!
HAPLN1	0.04761904762	0.04761904762	0.05555555556		0.7	#DIV/0!
HAPLN2	0.1428571429	0.1428571429	0.1111111111		1.083333333	#DIV/0!
HAPLN3	0.04761904762	0.04761904762	0.05555555556		0.7	#DIV/0!
HAPLN4	0.1428571429	0.1428571429	0.1111111111		1.083333333	#DIV/0!
HAR1A	0.1428571429	0.1428571429	0.2222222222		0.4583333333	#DIV/0!
HAR1B	0.1428571429	0.1428571429	0.2222222222		0.4583333333	#DIV/0!
HARBI1	0	0	0.05555555556		0	#DIV/0!
HARS	0.04761904762	0.04761904762	0		inf	#DIV/0!
HARS2	0.04761904762	0.04761904762	0		inf	#DIV/0!
HAS1	0.09523809524	0.09523809524	0.05555555556		1.473684211	#DIV/0!
HAS2	0.1428571429	0.1428571429	0.1111111111		1.083333333	#DIV/0!
IAS2-AS	0.1428571429	0.1428571429	0.1111111111		1.083333333	#DIV/0!
HAS3	0.1428571429	0.1428571429	0.05555555556		2.333333333	#DIV/0!
HASPIN	0	0	0.1111111111		0	#DIV/0!
HAT1	0	0	0.05555555556		0	#DIV/0!
HAUS1	0.04761904762	0.04761904762	0.05555555556		0.7	#DIV/0!
HAUS3	0.04761904762	0.04761904762	0		inf	#DIV/0!
HAUS4	0	0	0.05555555556		0	#DIV/0!
HAUS5	0.04761904762	0.04761904762	0.05555555556		0.7	#DIV/0!
HAUS6	0.04761904762	0.04761904762	0.05555555556		0.7	#DIV/0!
HAUS8	0.1428571429	0.1428571429	0.1111111111		1.083333333	#DIV/0!
HAVCR	0.04761904762	0.04761904762	0.05555555556		0.7	#DIV/0!
AVCR1I	0.09523809524	0.09523809524	0		inf	#DIV/0!
HAVCR2	0.04761904762	0.04761904762	0.05555555556		0.7	#DIV/0!
HAX1	0.1904761905	0.1904761905	0.05555555556		3.294117647	#DIV/0!
HBA1	0.04761904762	0.04761904762	0.2222222222		0.1375	#DIV/0!
HBA2	0.04761904762	0.04761904762	0.2222222222		0.1375	#DIV/0!
HBEGF	0.04761904762	0.04761904762	0		inf	#DIV/0!
HBM	0.04761904762	0.04761904762	0.2222222222		0.1375	#DIV/0!
HBP1	0.04761904762	0.04761904762	0.05555555556		0.7	#DIV/0!
HBQ1	0.04761904762	0.04761904762	0.2222222222		0.1375	#DIV/0!
HBZ	0.04761904762	0.04761904762	0.2222222222		0.1375	#DIV/0!
HCAR1	0.04761904762	0.04761904762	0.1111111111		0.325	#DIV/0!
HCAR2	0.04761904762	0.04761904762	0.1111111111		0.325	#DIV/0!
HCAR3	0.04761904762	0.04761904762	0.1111111111		0.325	#DIV/0!
HCCAT5	0.04761904762	0.04761904762	0		inf	#DIV/0!
ICFC1R	0.04761904762	0.04761904762	0.1111111111		0.325	#DIV/0!
HCFC2	0.04761904762	0.04761904762	0.05555555556		0.7	#DIV/0!
HCG11	0	0	0.05555555556		0	#DIV/0!
CG20400	0	0	0.05555555556		0	#DIV/0!
HCG23	0.04761904762	0.04761904762	0		inf	#DIV/0!
HCG24	0.04761904762	0.04761904762	0		inf	#DIV/0!
HCG26	0.04761904762	0.04761904762	0		inf	#DIV/0!
HCK	0.09523809524	0.09523809524	0.1111111111		0.6842105263	#DIV/0!
HCLS1	0.09523809524	0.09523809524	0.05555555556		1.473684211	#DIV/0!
HCN1	0.04761904762	0.04761904762	0.05555555556		0.7	#DIV/0!
HCN2	0.09523809524	0.09523809524	0.1111111111		0.6842105263	#DIV/0!
HCN3	0.1904761905	0.1904761905	0.1111111111		1.529411765	#DIV/0!
HCN4	0.04761904762	0.04761904762	0.05555555556		0.7	#DIV/0!

HCP5	0.04761904762	0.04761904762	0		inf	#DIV/0!
HCRT	0.04761904762	0.04761904762	0		inf	#DIV/0!
HCRTR2	0.04761904762	0.04761904762	0		inf	#DIV/0!
HCST	0.04761904762	0.04761904762	0.05555555556		0.7	#DIV/0!
HDAC1	0.04761904762	0.04761904762	0.1111111111		0.325	#DIV/0!
HDAC10	0	0	0.1111111111		0	#DIV/0!
HDAC11	0.04761904762	0.04761904762	0		inf	#DIV/0!
DAC11-A	0.04761904762	0.04761904762	0		inf	#DIV/0!
HDAC3	0.04761904762	0.04761904762	0		inf	#DIV/0!
HDAC4	0	0	0.05555555556		0	#DIV/0!
DAC4-A	0	0	0.05555555556		0	#DIV/0!
HDAC5	0.09523809524	0.09523809524	0		inf	#DIV/0!
HDAC7	0.04761904762	0.04761904762	0.05555555556		0.7	#DIV/0!
HDAC9	0.04761904762	0.04761904762	0.2222222222		0.1375	#DIV/0!
HDC	0.1428571429	0.1428571429	0.05555555556		2.333333333	#DIV/0!
HDDC3	0.04761904762	0.04761904762	0.05555555556		0.7	#DIV/0!
HDGF	0.1428571429	0.1428571429	0.05555555556		2.333333333	#DIV/0!
HDGFL2	0.09523809524	0.09523809524	0.05555555556		1.473684211	#DIV/0!
HDGFL3	0.04761904762	0.04761904762	0.05555555556		0.7	#DIV/0!
HDHD2	0.04761904762	0.04761904762	0.05555555556		0.7	#DIV/0!
HDHD3	0.04761904762	0.04761904762	0.05555555556		0.7	#DIV/0!
HDHD5	0.04761904762	0.04761904762	0		inf	#DIV/0!
DHD5-A	0.04761904762	0.04761904762	0		inf	#DIV/0!
HDLBP	0	0	0.05555555556		0	#DIV/0!
HEAT2	0.1428571429	0.1428571429	0.05555555556		2.333333333	#DIV/0!
HEATR1	0.1428571429	0.1428571429	0		inf	#DIV/0!
HEATR3	0.04761904762	0.04761904762	0		inf	#DIV/0!
HEATR4	0.09523809524	0.09523809524	0		inf	#DIV/0!
IEATR5	0	0	0			#DIV/0!
IEATR5	0	0	0.05555555556		0	#DIV/0!
HEATR6	0.1428571429	0.1428571429	0		inf	#DIV/0!
HEATR9	0.09523809524	0.09523809524	0		inf	#DIV/0!
HEBP1	0.04761904762	0.04761904762	0.05555555556		0.7	#DIV/0!
HECTD1	0	0	0			#DIV/0!
HECTD2	0.1428571429	0.1428571429	0.1111111111		1.083333333	#DIV/0!
CTD2-A	0.1428571429	0.1428571429	0.1111111111		1.083333333	#DIV/0!
HECTD3	0.04761904762	0.04761904762	0.05555555556		0.7	#DIV/0!
HECTD4	0	0	0.1111111111		0	#DIV/0!
HECW1	0.04761904762	0.04761904762	0.1666666667		0.2	#DIV/0!
ECW1-IT	0.04761904762	0.04761904762	0.1666666667		0.2	#DIV/0!
HECW2	0	0	0.05555555556		0	#DIV/0!
ECW2-A	0	0	0.05555555556		0	#DIV/0!
HEG1	0.04761904762	0.04761904762	0.05555555556		0.7	#DIV/0!
HEIH	0.09523809524	0.09523809524	0.05555555556		1.473684211	#DIV/0!
HELB	0.09523809524	0.09523809524	0.05555555556		1.473684211	#DIV/0!
HELLS	0.1428571429	0.1428571429	0		inf	#DIV/0!
HELQ	0	0	0.05555555556		0	#DIV/0!
HELT	0	0	0.05555555556		0	#DIV/0!
HELZ	0	0	0			#DIV/0!
HELZ2	0.1428571429	0.1428571429	0.2222222222		0.4583333333	#DIV/0!
HEMGN	0.04761904762	0.04761904762	0		inf	#DIV/0!

HEMK1	0.09523809524	0.09523809524	0		inf	#DIV/0!
IENMT1	0.04761904762	0.04761904762	0.05555555556		0.7	#DIV/0!
EPACAM	0.04761904762	0.04761904762	0.1111111111		0.325	#DIV/0!
HERC1	0.04761904762	0.04761904762	0.05555555556		0.7	#DIV/0!
IERC2P	0.09523809524	0.09523809524	0		inf	#DIV/0!
IERC2P	0.2857142857	0.2857142857	0.1111111111		2.6	#DIV/0!
IERC2P	0.1428571429	0.1428571429	0		inf	#DIV/0!
IERC2P	0.04761904762	0.04761904762	0		inf	#DIV/0!
HERC3	0	0	0.05555555556		0	#DIV/0!
HERC4	0.1904761905	0.1904761905	0.05555555556		3.294117647	#DIV/0!
HERC5	0	0	0.05555555556		0	#DIV/0!
HERC6	0	0	0.05555555556		0	#DIV/0!
IERPUD	0.04761904762	0.04761904762	0		inf	#DIV/0!
IERPUD	0.04761904762	0.04761904762	0.2222222222		0.1375	#DIV/0!
HES1	0.09523809524	0.09523809524	0.05555555556		1.473684211	#DIV/0!
HES2	0	0	0.05555555556		0	#DIV/0!
HES3	0	0	0.05555555556		0	#DIV/0!
HES4	0	0	0.1111111111		0	#DIV/0!
HES5	0	0	0.1111111111		0	#DIV/0!
HES6	0	0	0.05555555556		0	#DIV/0!
HES7	0	0	0.05555555556		0	#DIV/0!
HESX1	0.04761904762	0.04761904762	0		inf	#DIV/0!
HEXA	0.04761904762	0.04761904762	0.05555555556		0.7	#DIV/0!
EXA-AS	0.04761904762	0.04761904762	0.05555555556		0.7	#DIV/0!
HEXB	0.04761904762	0.04761904762	0.05555555556		0.7	#DIV/0!
HEXD	0.04761904762	0.04761904762	0		inf	#DIV/0!
IEXIM1	0.1428571429	0.1428571429	0		inf	#DIV/0!
IEXIM2	0.1428571429	0.1428571429	0		inf	#DIV/0!
HEY1	0.1428571429	0.1428571429	0.1111111111		1.083333333	#DIV/0!
HEYL	0.04761904762	0.04761904762	0.05555555556		0.7	#DIV/0!
HFE	0	0	0.05555555556		0	#DIV/0!
HFM1	0.04761904762	0.04761904762	0.05555555556		0.7	#DIV/0!
HGD	0.09523809524	0.09523809524	0.05555555556		1.473684211	#DIV/0!
HGF	0.04761904762	0.04761904762	0.1666666667		0.2	#DIV/0!
HGFAC	0.04761904762	0.04761904762	0		inf	#DIV/0!
HGH1	0.04761904762	0.04761904762	0.1666666667		0.2	#DIV/0!
HGS	0.04761904762	0.04761904762	0		inf	#DIV/0!
IGSNAT	0.09523809524	0.09523809524	0.1666666667		0.4210526316	#DIV/0!
HHAT	0.1428571429	0.1428571429	0		inf	#DIV/0!
HHATL	0.04761904762	0.04761904762	0		inf	#DIV/0!
IATL-A	0.04761904762	0.04761904762	0		inf	#DIV/0!
HHEX	0.1428571429	0.1428571429	0.1111111111		1.083333333	#DIV/0!
HHIP	0	0	0.05555555556		0	#DIV/0!
IHIP-AS	0	0	0.05555555556		0	#DIV/0!
HHIPL2	0.1428571429	0.1428571429	0.05555555556		2.333333333	#DIV/0!
HHLA1	0.04761904762	0.04761904762	0.1111111111		0.325	#DIV/0!
HHLA2	0.09523809524	0.09523809524	0.05555555556		1.473684211	#DIV/0!
HHLA3	0.04761904762	0.04761904762	0.05555555556		0.7	#DIV/0!
IIBADH	0.04761904762	0.04761904762	0.2222222222		0.1375	#DIV/0!
HIBCH	0	0	0.05555555556		0	#DIV/0!
HIC1	0	0	0.1111111111		0	#DIV/0!

**HIC2**	0.04761904762	0.04761904762	0.05555555556		0.7	#DIV/0!
**HID1**	0.04761904762	0.04761904762	0		inf	#DIV/0!
**HID1-AS**	0.04761904762	0.04761904762	0		inf	#DIV/0!
**HIF1AN**	0.1428571429	0.1428571429	0		inf	#DIV/0!
**HIF3A**	0.09523809524	0.09523809524	0.1111111111		0.6842105263	#DIV/0!
**HIGD1A**	0.04761904762	0.04761904762	0		inf	#DIV/0!
**HIGD1B**	0.1428571429	0.1428571429	0		inf	#DIV/0!
**HIGD1C**	0.09523809524	0.09523809524	0.05555555556		1.473684211	#DIV/0!
**HIGD2A**	0.09523809524	0.09523809524	0.05555555556		1.473684211	#DIV/0!
**HIGD2B**	0.04761904762	0.04761904762	0.05555555556		0.7	#DIV/0!
**HILPDA**	0.04761904762	0.04761904762	0.05555555556		0.7	#DIV/0!
**HILS1**	0.09523809524	0.09523809524	0		inf	#DIV/0!
**HINT1**	0.04761904762	0.04761904762	0		inf	#DIV/0!
**HINT2**	0.09523809524	0.09523809524	0.05555555556		1.473684211	#DIV/0!
**HIP1**	0.1428571429	0.1428571429	0.1666666667		0.6666666667	#DIV/0!
**HIP1R**	0.04761904762	0.04761904762	0.1111111111		0.325	#DIV/0!
**HIPK1**	0.04761904762	0.04761904762	0.05555555556		0.7	#DIV/0!
**IPK1-AS**	0.04761904762	0.04761904762	0.05555555556		0.7	#DIV/0!
**HIPK2**	0.04761904762	0.04761904762	0.05555555556		0.7	#DIV/0!
**HIPK3**	0.04761904762	0.04761904762	0		inf	#DIV/0!
**HIPK4**	0.04761904762	0.04761904762	0.05555555556		0.7	#DIV/0!
**HIRA**	0.04761904762	0.04761904762	0.05555555556		0.7	#DIV/0!
**HIRIP3**	0.04761904762	0.04761904762	0		inf	#DIV/0!
**IST1H1**	0	0	0.05555555556		0	#DIV/0!
**IST1H1**	0	0	0.05555555556		0	#DIV/0!
**IST1H1**	0	0	0.05555555556		0	#DIV/0!
**IST1H1**	0	0	0.05555555556		0	#DIV/0!
**IST1H1**	0	0	0.05555555556		0	#DIV/0!
**ST1H2A**	0	0	0.05555555556		0	#DIV/0!
**ST1H2A**	0	0	0.05555555556		0	#DIV/0!
**ST1H2A**	0	0	0.05555555556		0	#DIV/0!
**ST1H2A**	0	0	0.05555555556		0	#DIV/0!
**IST1H2B**	0	0	0.05555555556		0	#DIV/0!
**ST1H2B**	0	0	0.05555555556		0	#DIV/0!
**ST1H2B**	0	0	0.05555555556		0	#DIV/0!
**IST1H2B**	0	0	0.05555555556		0	#DIV/0!
**IST1H2B**	0	0	0.05555555556		0	#DIV/0!
**ST1H2B**	0	0	0.05555555556		0	#DIV/0!
**ST1H2B**	0	0	0.05555555556		0	#DIV/0!
**IST1H2I**	0	0	0.05555555556		0	#DIV/0!
**IST1H3**	0	0	0.05555555556		0	#DIV/0!
**IST1H3**	0	0	0.05555555556		0	#DIV/0!
**IST1H3**	0	0	0.05555555556		0	#DIV/0!
**IST1H3**	0	0	0.05555555556		0	#DIV/0!
**IST1H3**	0	0	0.05555555556		0	#DIV/0!
**IST1H3**	0	0	0.05555555556		0	#DIV/0!
**IST1H3**	0	0	0.05555555556		0	#DIV/0!
**IST1H4**	0	0	0.05555555556		0	#DIV/0!
**IST1H4**	0	0	0.05555555556		0	#DIV/0!
**IST1H4**	0	0	0.05555555556		0	#DIV/0!
**IST1H4**	0	0	0.05555555556		0	#DIV/0!

**IST1H4**	0	0	0.05555555556		0	#DIV/0!
**IIST1H4**	0	0	0.05555555556		0	#DIV/0!
**IST1H4**	0	0	0.05555555556		0	#DIV/0!
**IST1H4**	0	0	0.05555555556		0	#DIV/0!
**ST2H2A**	0.1428571429	0.1428571429	0.05555555556		2.333333333	#DIV/0!
**ST2H2A**	0.1428571429	0.1428571429	0.05555555556		2.333333333	#DIV/0!
**IST2H2A**	0.1428571429	0.1428571429	0.05555555556		2.333333333	#DIV/0!
**IST2H2A**	0.1428571429	0.1428571429	0.05555555556		2.333333333	#DIV/0!
**IST2H2B**	0.04761904762	0.04761904762	0.1111111111		0.325	#DIV/0!
**IST2H2B**	0.09523809524	0.09523809524	0.05555555556		1.473684211	#DIV/0!
**IST2H2B**	0.1428571429	0.1428571429	0.05555555556		2.333333333	#DIV/0!
**IST2H2B**	0.1428571429	0.1428571429	0.05555555556		2.333333333	#DIV/0!
**IST2H2B**	0.1428571429	0.1428571429	0.05555555556		2.333333333	#DIV/0!
**IST2H3**	0.1428571429	0.1428571429	0.05555555556		2.333333333	#DIV/0!
**IST2H3**	0.1428571429	0.1428571429	0.05555555556		2.333333333	#DIV/0!
**IST2H3**	0.1428571429	0.1428571429	0.05555555556		2.333333333	#DIV/0!
**ST2H3D**	0.1428571429	0.1428571429	0.1111111111		1.083333333	#DIV/0!
**ST2H3P**	0.09523809524	0.09523809524	0.05555555556		1.473684211	#DIV/0!
**IST2H4**	0.1428571429	0.1428571429	0.05555555556		2.333333333	#DIV/0!
**IST2H4**	0.1428571429	0.1428571429	0.05555555556		2.333333333	#DIV/0!
**IST3H2**	0.1428571429	0.1428571429	0		inf	#DIV/0!
**IST3H2B**	0.1428571429	0.1428571429	0		inf	#DIV/0!
**HIST3H3**	0.1428571429	0.1428571429	0		inf	#DIV/0!
**HIST4H4**	0.04761904762	0.04761904762	0.1111111111		0.325	#DIV/0!
**HIVEP1**	0.04761904762	0.04761904762	0		inf	#DIV/0!
**HIVEP3**	0.04761904762	0.04761904762	0.05555555556		0.7	#DIV/0!
**HJURP**	0	0	0.05555555556		0	#DIV/0!
**HJV**	0.1428571429	0.1428571429	0.05555555556		2.333333333	#DIV/0!
**HK1**	0.1904761905	0.1904761905	0		inf	#DIV/0!
**HK2**	0	0	0.05555555556		0	#DIV/0!
**HK3**	0.09523809524	0.09523809524	0.05555555556		1.473684211	#DIV/0!
**HKDC1**	0.1904761905	0.1904761905	0		inf	#DIV/0!
**LA-DM**	0.04761904762	0.04761904762	0		inf	#DIV/0!
**LA-DM**	0.04761904762	0.04761904762	0		inf	#DIV/0!
**ILA-DO**	0.04761904762	0.04761904762	0		inf	#DIV/0!
**ILA-DO**	0.04761904762	0.04761904762	0		inf	#DIV/0!
**LA-DPA**	0.04761904762	0.04761904762	0		inf	#DIV/0!
**LA-DPB**	0.04761904762	0.04761904762	0		inf	#DIV/0!
**LA-DPB**	0.04761904762	0.04761904762	0		inf	#DIV/0!
**LA-DQA**	0.04761904762	0.04761904762	0		inf	#DIV/0!
**LA-DQA**	0.04761904762	0.04761904762	0		inf	#DIV/0!
**LA-DQB**	0.04761904762	0.04761904762	0		inf	#DIV/0!
**A-DQB1-**	0.04761904762	0.04761904762	0		inf	#DIV/0!
**LA-DQB**	0.04761904762	0.04761904762	0		inf	#DIV/0!
**ILA-DR**	0.04761904762	0.04761904762	0		inf	#DIV/0!
**LA-DRB**	0.04761904762	0.04761904762	0		inf	#DIV/0!
**LA-DRB**	0.04761904762	0.04761904762	0		inf	#DIV/0!
**LA-DRB**	0.04761904762	0.04761904762	0		inf	#DIV/0!
**HLCS**	0.04761904762	0.04761904762	0.1666666667		0.2	#DIV/0!
**HLF**	0.09523809524	0.09523809524	0		inf	#DIV/0!
**HLTF**	0.1428571429	0.1428571429	0.1111111111		1.083333333	#DIV/0!

LTF-AS	0.1428571429	0.1428571429	0.1111111111		1.083333333	#DIV/0!
HLX	0.1428571429	0.1428571429	0.05555555556		2.333333333	#DIV/0!
HLX-AS1	0.1428571429	0.1428571429	0.05555555556		2.333333333	#DIV/0!
HM13	0.09523809524	0.09523809524	0.1111111111		0.6842105263	#DIV/0!
M13-AS	0.09523809524	0.09523809524	0.1111111111		0.6842105263	#DIV/0!
HMBOX	0.04761904762	0.04761904762	0.1111111111		0.325	#DIV/0!
HMCES	0.09523809524	0.09523809524	0.05555555556		1.473684211	#DIV/0!
HMCN1	0.1428571429	0.1428571429	0.05555555556		2.333333333	#DIV/0!
HMCN2	0.09523809524	0.09523809524	0.05555555556		1.473684211	#DIV/0!
HMG20A	0.04761904762	0.04761904762	0.05555555556		0.7	#DIV/0!
HMG20B	0.09523809524	0.09523809524	0.05555555556		1.473684211	#DIV/0!
HMGA1	0.04761904762	0.04761904762	0		inf	#DIV/0!
HMGA2	0.09523809524	0.09523809524	0.05555555556		1.473684211	#DIV/0!
MGA2-A	0.09523809524	0.09523809524	0.05555555556		1.473684211	#DIV/0!
HMGB2	0	0	0.05555555556		0	#DIV/0!
HMGB3P	0.09523809524	0.09523809524	0.1111111111		0.6842105263	#DIV/0!
HMGB4	0.04761904762	0.04761904762	0		inf	#DIV/0!
HMGCL	0.04761904762	0.04761904762	0.1111111111		0.325	#DIV/0!
HMGCLL	0.04761904762	0.04761904762	0.05555555556		0.7	#DIV/0!
HMGCR	0.04761904762	0.04761904762	0		inf	#DIV/0!
HMGCS1	0.04761904762	0.04761904762	0.05555555556		0.7	#DIV/0!
HMGCS2	0.04761904762	0.04761904762	0.05555555556		0.7	#DIV/0!
HMGN1	0.04761904762	0.04761904762	0.1111111111		0.325	#DIV/0!
HMGN2	0.04761904762	0.04761904762	0.1111111111		0.325	#DIV/0!
MGN2P	0.04761904762	0.04761904762	0		inf	#DIV/0!
HMGN4	0	0	0.05555555556		0	#DIV/0!
HMGXB	0	0	0.1666666667		0	#DIV/0!
HMMR	0	0	0.05555555556		0	#DIV/0!
MMR-A	0	0	0.05555555556		0	#DIV/0!
HMOX1	0	0	0.1111111111		0	#DIV/0!
HMOX2	0.04761904762	0.04761904762	0		inf	#DIV/0!
HMSD	0.04761904762	0.04761904762	0		inf	#DIV/0!
HMX2	0.2380952381	0.2380952381	0.05555555556		4.375	#DIV/0!
HMX3	0.2380952381	0.2380952381	0.05555555556		4.375	#DIV/0!
HNF1A	0.04761904762	0.04761904762	0.1111111111		0.325	#DIV/0!
NF1A-A	0.04761904762	0.04761904762	0.1111111111		0.325	#DIV/0!
HNF4A	0.1428571429	0.1428571429	0.1666666667		0.6666666667	#DIV/0!
NF4A-A	0.1428571429	0.1428571429	0.1666666667		0.6666666667	#DIV/0!
HNF4G	0.1904761905	0.1904761905	0.1111111111		1.529411765	#DIV/0!
HNMT	0	0	0.05555555556		0	#DIV/0!
HNRNPA	0.04761904762	0.04761904762	0		inf	#DIV/0!
HNRNPA	0.04761904762	0.04761904762	0.05555555556		0.7	#DIV/0!
RNPA1	0.1428571429	0.1428571429	0.1111111111		1.083333333	#DIV/0!
RNPA1	0.1428571429	0.1428571429	0.05555555556		2.333333333	#DIV/0!
NRNPA2	0.04761904762	0.04761904762	0.2222222222		0.1375	#DIV/0!
HNRNPA	0	0	0.1111111111		0	#DIV/0!
NRNPA3	0.1428571429	0.1428571429	0.05555555556		2.333333333	#DIV/0!
HNRNPA	0.09523809524	0.09523809524	0.05555555556		1.473684211	#DIV/0!
NRNPC	0.04761904762	0.04761904762	0.1111111111		0.325	#DIV/0!
NRNPC	0.04761904762	0.04761904762	0.1111111111		0.325	#DIV/0!
NRNPC	0.04761904762	0.04761904762	0.1111111111		0.325	#DIV/0!

NRNPCI	0.04761904762	0.04761904762	0.1111111111		0.325	#DIV/0!
INRNPI	0	0	0.05555555556		0	#DIV/0!
INRNPD	0	0	0.05555555556		0	#DIV/0!
INRNPI	0.1428571429	0.1428571429	0.05555555556		2.333333333	#DIV/0!
INRNPH	0.09523809524	0.09523809524	0.05555555556		1.473684211	#DIV/0!
INRNPH	0.1904761905	0.1904761905	0.05555555556		3.294117647	#DIV/0!
INRNPK	0	0	0.05555555556		0	#DIV/0!
INRNPI	0.04761904762	0.04761904762	0.05555555556		0.7	#DIV/0!
INRNPL	0	0	0.05555555556		0	#DIV/0!
INRNPM	0.1428571429	0.1428571429	0.05555555556		2.333333333	#DIV/0!
INRNPR	0.04761904762	0.04761904762	0.1111111111		0.325	#DIV/0!
INRNPU	0.1428571429	0.1428571429	0.05555555556		2.333333333	#DIV/0!
NRNPUI	0.04761904762	0.04761904762	0.05555555556		0.7	#DIV/0!
HOGA1	0.1428571429	0.1428571429	0		inf	#DIV/0!
IOMER	0.04761904762	0.04761904762	0		inf	#DIV/0!
IOMER	0.04761904762	0.04761904762	0.05555555556		0.7	#DIV/0!
IOMER	0.1428571429	0.1428571429	0.1111111111		1.083333333	#DIV/0!
MER3-A	0.1428571429	0.1428571429	0.1111111111		1.083333333	#DIV/0!
HOMEZ	0	0	0.1111111111		0	#DIV/0!
HOOK1	0.04761904762	0.04761904762	0.05555555556		0.7	#DIV/0!
HOOK2	0.1428571429	0.1428571429	0.1111111111		1.083333333	#DIV/0!
HOOK3	0.09523809524	0.09523809524	0.1666666667		0.4210526316	#DIV/0!
HOPX	0.1904761905	0.1904761905	0.05555555556		3.294117647	#DIV/0!
ORMAI	0.1904761905	0.1904761905	0.05555555556		3.294117647	#DIV/0!
ORMAI	0.04761904762	0.04761904762	0.1111111111		0.325	#DIV/0!
RMAD2-	0.04761904762	0.04761904762	0.1111111111		0.325	#DIV/0!
HOTAIR	0.04761904762	0.04761904762	0.05555555556		0.7	#DIV/0!
OTAIRM	0.04761904762	0.04761904762	0.2222222222		0.1375	#DIV/0!
HOTS	0.04761904762	0.04761904762	0		inf	#DIV/0!
HOTTIP	0.04761904762	0.04761904762	0.2222222222		0.1375	#DIV/0!
OXA-AS	0.04761904762	0.04761904762	0.2222222222		0.1375	#DIV/0!
OXA-AS	0.04761904762	0.04761904762	0.2222222222		0.1375	#DIV/0!
HOXA1	0.04761904762	0.04761904762	0.2222222222		0.1375	#DIV/0!
HOXA10	0.04761904762	0.04761904762	0.2222222222		0.1375	#DIV/0!
OXA10-A	0.04761904762	0.04761904762	0.2222222222		0.1375	#DIV/0!
A10-HO	0.04761904762	0.04761904762	0.2222222222		0.1375	#DIV/0!
HOXA11	0.04761904762	0.04761904762	0.2222222222		0.1375	#DIV/0!
OXA11-A	0.04761904762	0.04761904762	0.2222222222		0.1375	#DIV/0!
HOXA13	0.04761904762	0.04761904762	0.2222222222		0.1375	#DIV/0!
HOXA2	0.04761904762	0.04761904762	0.2222222222		0.1375	#DIV/0!
HOXA3	0.04761904762	0.04761904762	0.2222222222		0.1375	#DIV/0!
HOXA4	0.04761904762	0.04761904762	0.2222222222		0.1375	#DIV/0!
HOXA5	0.04761904762	0.04761904762	0.2222222222		0.1375	#DIV/0!
HOXA6	0.04761904762	0.04761904762	0.2222222222		0.1375	#DIV/0!
HOXA7	0.04761904762	0.04761904762	0.2222222222		0.1375	#DIV/0!
HOXA9	0.04761904762	0.04761904762	0.2222222222		0.1375	#DIV/0!
OXB-AS	0.1428571429	0.1428571429	0		inf	#DIV/0!
OXB-AS	0.1428571429	0.1428571429	0		inf	#DIV/0!
HOXB1	0.1428571429	0.1428571429	0		inf	#DIV/0!
HOXB13	0.1428571429	0.1428571429	0		inf	#DIV/0!
HOXB2	0.1428571429	0.1428571429	0		inf	#DIV/0!

**HOXB3**	0.1428571429	0.1428571429	0		inf	#DIV/0!
**HOXB4**	0.1428571429	0.1428571429	0		inf	#DIV/0!
**HOXB5**	0.1428571429	0.1428571429	0		inf	#DIV/0!
**HOXB6**	0.1428571429	0.1428571429	0		inf	#DIV/0!
**HOXB7**	0.1428571429	0.1428571429	0		inf	#DIV/0!
**HOXB8**	0.1428571429	0.1428571429	0		inf	#DIV/0!
**HOXB9**	0.1428571429	0.1428571429	0		inf	#DIV/0!
**OXC-AS**	0.04761904762	0.04761904762	0.05555555556		0.7	#DIV/0!
**OXC-AS**	0.04761904762	0.04761904762	0.05555555556		0.7	#DIV/0!
**OXC-AS**	0.04761904762	0.04761904762	0.05555555556		0.7	#DIV/0!
**HOXC10**	0.04761904762	0.04761904762	0.05555555556		0.7	#DIV/0!
**HOXC11**	0.04761904762	0.04761904762	0.05555555556		0.7	#DIV/0!
**HOXC12**	0.04761904762	0.04761904762	0.05555555556		0.7	#DIV/0!
**HOXC13**	0.04761904762	0.04761904762	0.05555555556		0.7	#DIV/0!
**OXC13-A**	0.04761904762	0.04761904762	0.05555555556		0.7	#DIV/0!
**HOXC4**	0.04761904762	0.04761904762	0.05555555556		0.7	#DIV/0!
**HOXC5**	0.04761904762	0.04761904762	0.05555555556		0.7	#DIV/0!
**HOXC6**	0.04761904762	0.04761904762	0.05555555556		0.7	#DIV/0!
**HOXC8**	0.04761904762	0.04761904762	0.05555555556		0.7	#DIV/0!
**HOXC9**	0.04761904762	0.04761904762	0.05555555556		0.7	#DIV/0!
**HOXD10**	0	0	0.1111111111		0	#DIV/0!
**HOXD11**	0	0	0.1111111111		0	#DIV/0!
**HOXD12**	0	0	0.1111111111		0	#DIV/0!
**HOXD13**	0	0	0.1111111111		0	#DIV/0!
**HOXD8**	0	0	0.1111111111		0	#DIV/0!
**HOXD9**	0	0	0.1111111111		0	#DIV/0!
**HP**	0.04761904762	0.04761904762	0		inf	#DIV/0!
**HP09053**	0.09523809524	0.09523809524	0.1111111111		0.6842105263	#DIV/0!
**HP1BP3**	0.04761904762	0.04761904762	0.1111111111		0.325	#DIV/0!
**HPCA**	0.04761904762	0.04761904762	0.1111111111		0.325	#DIV/0!
**HPCAL1**	0	0	0.05555555556		0	#DIV/0!
**HPCAL4**	0.04761904762	0.04761904762	0.05555555556		0.7	#DIV/0!
**HPD**	0.04761904762	0.04761904762	0.1111111111		0.325	#DIV/0!
**HPDL**	0.04761904762	0.04761904762	0.05555555556		0.7	#DIV/0!
**HPF1**	0	0	0.05555555556		0	#DIV/0!
**HPGD**	0	0	0.05555555556		0	#DIV/0!
**HPGDS**	0	0	0.05555555556		0	#DIV/0!
**HPN**	0.04761904762	0.04761904762	0.05555555556		0.7	#DIV/0!
**HPN-AS1**	0.04761904762	0.04761904762	0.05555555556		0.7	#DIV/0!
**HPR**	0.04761904762	0.04761904762	0		inf	#DIV/0!
**HPS1**	0.1428571429	0.1428571429	0		inf	#DIV/0!
**HPS3**	0.1428571429	0.1428571429	0.1111111111		1.083333333	#DIV/0!
**HPS4**	0.04761904762	0.04761904762	0.05555555556		0.7	#DIV/0!
**HPS6**	0.1428571429	0.1428571429	0.05555555556		2.333333333	#DIV/0!
**HPSE**	0	0	0.05555555556		0	#DIV/0!
**HPSE2**	0.1428571429	0.1428571429	0		inf	#DIV/0!
**HPX**	0.04761904762	0.04761904762	0		inf	#DIV/0!
**HPYR1**	0.04761904762	0.04761904762	0.1111111111		0.325	#DIV/0!
**HR**	0.09523809524	0.09523809524	0.1111111111		0.6842105263	#DIV/0!
**HRAS**	0.04761904762	0.04761904762	0		inf	#DIV/0!
**HRAT17**	0.04761904762	0.04761904762	0.05555555556		0.7	#DIV/0!

HRAT5	0	0	0.05555555556		0	#DIV/0!
HRAT92	0.04761904762	0.04761904762	0.1666666667		0.2	#DIV/0!
HRC	0.09523809524	0.09523809524	0.1111111111		0.6842105263	#DIV/0!
HRCT1	0.09523809524	0.09523809524	0.05555555556		1.473684211	#DIV/0!
HRG	0.09523809524	0.09523809524	0.05555555556		1.473684211	#DIV/0!
HRH1	0.09523809524	0.09523809524	0		inf	#DIV/0!
HRH2	0	0	0.05555555556		0	#DIV/0!
HRH3	0.1428571429	0.1428571429	0.2222222222		0.4583333333	#DIV/0!
HRH4	0.09523809524	0.09523809524	0.05555555556		1.473684211	#DIV/0!
HRK	0	0	0.05555555556		0	#DIV/0!
HRNR	0.1904761905	0.1904761905	0		inf	#DIV/0!
HS1BP3	0	0	0.05555555556		0	#DIV/0!
S1BP3-I	0	0	0.05555555556		0	#DIV/0!
HS2ST1	0.04761904762	0.04761904762	0.05555555556		0.7	#DIV/0!
HS3ST1	0	0	0			#DIV/0!
HS3ST2	0.04761904762	0.04761904762	0		inf	#DIV/0!
IS3ST3A	0.04761904762	0.04761904762	0.1111111111		0.325	#DIV/0!
IS3ST3B	0.04761904762	0.04761904762	0.05555555556		0.7	#DIV/0!
HS3ST4	0.04761904762	0.04761904762	0		inf	#DIV/0!
HS3ST6	0.09523809524	0.09523809524	0.2222222222		0.2894736842	#DIV/0!
HS6ST1	0	0	0.05555555556		0	#DIV/0!
HSBP1	0.04761904762	0.04761904762	0		inf	#DIV/0!
ISBP1L	0.04761904762	0.04761904762	0.05555555556		0.7	#DIV/0!
HSCB	0.04761904762	0.04761904762	0.1111111111		0.325	#DIV/0!
ISD11B	0.1428571429	0.1428571429	0		inf	#DIV/0!
D11B1-A	0.1428571429	0.1428571429	0		inf	#DIV/0!
SD11B1	0.1428571429	0.1428571429	0.05555555556		2.333333333	#DIV/0!
ISD11B	0.1428571429	0.1428571429	0.05555555556		2.333333333	#DIV/0!
ISD17B1	0	0	0.05555555556		0	#DIV/0!
ISD17B1	0	0	0.05555555556		0	#DIV/0!
ISD17B1	0.09523809524	0.09523809524	0.1111111111		0.6842105263	#DIV/0!
ISD17B	0.04761904762	0.04761904762	0		inf	#DIV/0!
ISD17B	0.04761904762	0.04761904762	0.05555555556		0.7	#DIV/0!
D17B3-A	0.04761904762	0.04761904762	0.05555555556		0.7	#DIV/0!
ISD17B	0.04761904762	0.04761904762	0		inf	#DIV/0!
ISD17B	0.1428571429	0.1428571429	0.05555555556		2.333333333	#DIV/0!
ISD17B	0.1428571429	0.1428571429	0		inf	#DIV/0!
SD17B7	0.09523809524	0.09523809524	0.1111111111		0.6842105263	#DIV/0!
ISD17B	0.04761904762	0.04761904762	0		inf	#DIV/0!
HSD3B1	0.04761904762	0.04761904762	0.05555555556		0.7	#DIV/0!
HSD3B2	0.04761904762	0.04761904762	0.05555555556		0.7	#DIV/0!
HSD3B7	0.04761904762	0.04761904762	0		inf	#DIV/0!
ISD3BP	0.04761904762	0.04761904762	0.05555555556		0.7	#DIV/0!
HSD52	0.04761904762	0.04761904762	0.05555555556		0.7	#DIV/0!
HSDL1	0.04761904762	0.04761904762	0		inf	#DIV/0!
HSDL2	0.04761904762	0.04761904762	0.05555555556		0.7	#DIV/0!
HSF1	0.04761904762	0.04761904762	0.1666666667		0.2	#DIV/0!
HSF2BP	0.04761904762	0.04761904762	0.1666666667		0.2	#DIV/0!
HSF4	0.1428571429	0.1428571429	0.05555555556		2.333333333	#DIV/0!
HSF5	0.09523809524	0.09523809524	0		inf	#DIV/0!
ISFY1P	0.04761904762	0.04761904762	0		inf	#DIV/0!

**HSH2D**	0.1428571429	0.1428571429	0.1111111111		1.083333333	#DIV/0!
**SP90AB**	0.04761904762	0.04761904762	0.05555555556		0.7	#DIV/0!
**SP90AB4**	0.04761904762	0.04761904762	0.05555555556		0.7	#DIV/0!
**HSP90B1**	0.04761904762	0.04761904762	0.05555555556		0.7	#DIV/0!
**ISP90B2**	0.04761904762	0.04761904762	0.05555555556		0.7	#DIV/0!
**HSPA12A**	0.2380952381	0.2380952381	0		inf	#DIV/0!
**HSPA13**	0.04761904762	0.04761904762	0.1111111111		0.325	#DIV/0!
**HSPA14**	0.09523809524	0.09523809524	0.1111111111		0.6842105263	#DIV/0!
**HSPA1A**	0.04761904762	0.04761904762	0		inf	#DIV/0!
**HSPA1B**	0.04761904762	0.04761904762	0		inf	#DIV/0!
**HSPA1L**	0.04761904762	0.04761904762	0		inf	#DIV/0!
**HSPA2**	0.09523809524	0.09523809524	0		inf	#DIV/0!
**HSPA4**	0.04761904762	0.04761904762	0		inf	#DIV/0!
**HSPA4L**	0	0	0.05555555556		0	#DIV/0!
**HSPA5**	0.09523809524	0.09523809524	0.05555555556		1.473684211	#DIV/0!
**HSPA6**	0.1428571429	0.1428571429	0		inf	#DIV/0!
**HSPA7**	0.1428571429	0.1428571429	0		inf	#DIV/0!
**HSPA9**	0.04761904762	0.04761904762	0		inf	#DIV/0!
**HSPB1**	0.09523809524	0.09523809524	0.1111111111		0.6842105263	#DIV/0!
**HSPB11**	0.04761904762	0.04761904762	0.05555555556		0.7	#DIV/0!
**HSPB3**	0.04761904762	0.04761904762	0.05555555556		0.7	#DIV/0!
**HSPB6**	0.04761904762	0.04761904762	0.05555555556		0.7	#DIV/0!
**HSPB7**	0.04761904762	0.04761904762	0.1111111111		0.325	#DIV/0!
**HSPB8**	0	0	0.05555555556		0	#DIV/0!
**HSPB9**	0.04761904762	0.04761904762	0		inf	#DIV/0!
**ISPBAP**	0.09523809524	0.09523809524	0.05555555556		1.473684211	#DIV/0!
**HSPBP1**	0.09523809524	0.09523809524	0.05555555556		1.473684211	#DIV/0!
**ISPC324**	0.1428571429	0.1428571429	0.05555555556		2.333333333	#DIV/0!
**HSPD1**	0	0	0.05555555556		0	#DIV/0!
**HSPE1**	0	0	0.05555555556		0	#DIV/0!
**PE1-MO**	0	0	0.05555555556		0	#DIV/0!
**HSPG2**	0.04761904762	0.04761904762	0.1111111111		0.325	#DIV/0!
**HTD2**	0.04761904762	0.04761904762	0		inf	#DIV/0!
**HTN1**	0.04761904762	0.04761904762	0.05555555556		0.7	#DIV/0!
**HTN3**	0.04761904762	0.04761904762	0.05555555556		0.7	#DIV/0!
**HTR1A**	0.04761904762	0.04761904762	0		inf	#DIV/0!
**HTR1D**	0.04761904762	0.04761904762	0.1111111111		0.325	#DIV/0!
**HTR1F**	0.04761904762	0.04761904762	0		inf	#DIV/0!
**HTR2B**	0	0	0.05555555556		0	#DIV/0!
**HTR3C**	0.1428571429	0.1428571429	0.05555555556		2.333333333	#DIV/0!
**HTR3D**	0.1428571429	0.1428571429	0.05555555556		2.333333333	#DIV/0!
**HTR3E**	0.1428571429	0.1428571429	0.05555555556		2.333333333	#DIV/0!
**TR3E-AS**	0.1428571429	0.1428571429	0.05555555556		2.333333333	#DIV/0!
**HTR5A**	0.1428571429	0.1428571429	0.05555555556		2.333333333	#DIV/0!
**TR5A-A**	0.1428571429	0.1428571429	0.05555555556		2.333333333	#DIV/0!
**HTR6**	0.04761904762	0.04761904762	0.1111111111		0.325	#DIV/0!
**HTR7**	0.1428571429	0.1428571429	0.1111111111		1.083333333	#DIV/0!
**HTR7P1**	0.04761904762	0.04761904762	0.05555555556		0.7	#DIV/0!
**HTRA1**	0.1904761905	0.1904761905	0.05555555556		3.294117647	#DIV/0!
**HTRA2**	0	0	0.05555555556		0	#DIV/0!
**HTRA4**	0.09523809524	0.09523809524	0.1111111111		0.6842105263	#DIV/0!

**HTT**	0.04761904762	0.04761904762	0		inf	#DIV/0!
**HTT-AS**	0.04761904762	0.04761904762	0		inf	#DIV/0!
**HUNK**	0.04761904762	0.04761904762	0.1111111111		0.325	#DIV/0!
**HUS1**	0.04761904762	0.04761904762	0.2222222222		0.1375	#DIV/0!
**HVCN1**	0	0	0.1111111111		0	#DIV/0!
**HYAL1**	0.09523809524	0.09523809524	0		inf	#DIV/0!
**HYAL2**	0.09523809524	0.09523809524	0		inf	#DIV/0!
**HYAL3**	0.09523809524	0.09523809524	0		inf	#DIV/0!
**HYAL4**	0.04761904762	0.04761904762	0.05555555556		0.7	#DIV/0!
**HYAL6P**	0.04761904762	0.04761904762	0.05555555556		0.7	#DIV/0!
**HYDIN**	0.04761904762	0.04761904762	0		inf	#DIV/0!
**HYI**	0.04761904762	0.04761904762	0.05555555556		0.7	#DIV/0!
**HYKK**	0.04761904762	0.04761904762	0.05555555556		0.7	#DIV/0!
**IAH1**	0	0	0.05555555556		0	#DIV/0!
**IAPP**	0.04761904762	0.04761904762	0.05555555556		0.7	#DIV/0!
**IARS**	0.04761904762	0.04761904762	0.05555555556		0.7	#DIV/0!
**IARS2**	0.1428571429	0.1428571429	0.05555555556		2.333333333	#DIV/0!
**IATPR**	0.09523809524	0.09523809524	0.2222222222		0.2894736842	#DIV/0!
**IBA57**	0.1428571429	0.1428571429	0		inf	#DIV/0!
**BA57-D**	0.1428571429	0.1428571429	0		inf	#DIV/0!
**IBSP**	0	0	0.05555555556		0	#DIV/0!
**ICA1**	0.04761904762	0.04761904762	0.1666666667		0.2	#DIV/0!
**ICA1L**	0.09523809524	0.09523809524	0.05555555556		1.473684211	#DIV/0!
**ICAM2**	0.04761904762	0.04761904762	0		inf	#DIV/0!
**ICAM3**	0.1428571429	0.1428571429	0.1111111111		1.083333333	#DIV/0!
**ICE1**	0	0	0.1111111111		0	#DIV/0!
**ICE2**	0.04761904762	0.04761904762	0.05555555556		0.7	#DIV/0!
**ICK**	0.04761904762	0.04761904762	0		inf	#DIV/0!
**ICMT**	0	0	0.05555555556		0	#DIV/0!
**ICOS**	0	0	0.05555555556		0	#DIV/0!
**ICOSLG**	0.04761904762	0.04761904762	0.1111111111		0.325	#DIV/0!
**ID1**	0.09523809524	0.09523809524	0.1111111111		0.6842105263	#DIV/0!
**ID2**	0	0	0.05555555556		0	#DIV/0!
**ID2-AS1**	0	0	0.05555555556		0	#DIV/0!
**ID3**	0.04761904762	0.04761904762	0.1111111111		0.325	#DIV/0!
**IDE**	0.1428571429	0.1428571429	0.1111111111		1.083333333	#DIV/0!
**IDH1**	0	0	0.05555555556		0	#DIV/0!
**DH1-AS**	0	0	0.05555555556		0	#DIV/0!
**IDH2**	0.04761904762	0.04761904762	0.05555555556		0.7	#DIV/0!
**DH2-DT**	0.04761904762	0.04761904762	0.05555555556		0.7	#DIV/0!
**IDH3A**	0.04761904762	0.04761904762	0.05555555556		0.7	#DIV/0!
**IDH3B**	0.1904761905	0.1904761905	0.05555555556		3.294117647	#DIV/0!
**IDI1**	0.04761904762	0.04761904762	0.1111111111		0.325	#DIV/0!
**IDI2**	0.04761904762	0.04761904762	0.1111111111		0.325	#DIV/0!
**DI2-AS1**	0.04761904762	0.04761904762	0.1111111111		0.325	#DIV/0!
**IDNK**	0	0	0.05555555556		0	#DIV/0!
**IDO1**	0.09523809524	0.09523809524	0.1111111111		0.6842105263	#DIV/0!
**IDO2**	0.09523809524	0.09523809524	0.1111111111		0.6842105263	#DIV/0!
**IDUA**	0.04761904762	0.04761904762	0		inf	#DIV/0!
**IER2**	0.1428571429	0.1428571429	0.1111111111		1.083333333	#DIV/0!
**IER3IP1**	0.04761904762	0.04761904762	0.05555555556		0.7	#DIV/0!

**IER5**	0.1428571429	0.1428571429	0		inf	#DIV/0!
**IER5L**	0.09523809524	0.09523809524	0.05555555556		1.473684211	#DIV/0!
**IFFO1**	0.04761904762	0.04761904762	0.1111111111		0.325	#DIV/0!
**IFFO2**	0.04761904762	0.04761904762	0.1111111111		0.325	#DIV/0!
**IFI16**	0.1428571429	0.1428571429	0		inf	#DIV/0!
**IFI30**	0.1428571429	0.1428571429	0.1111111111		1.083333333	#DIV/0!
**IFI35**	0.04761904762	0.04761904762	0		inf	#DIV/0!
**IFI44**	0.04761904762	0.04761904762	0.05555555556		0.7	#DIV/0!
**IFI44L**	0.04761904762	0.04761904762	0.05555555556		0.7	#DIV/0!
**IFI6**	0.04761904762	0.04761904762	0.1111111111		0.325	#DIV/0!
**IFIH1**	0	0	0.05555555556		0	#DIV/0!
**IFIT1**	0.1428571429	0.1428571429	0.1666666667		0.6666666667	#DIV/0!
**IFIT1B**	0.1428571429	0.1428571429	0.1666666667		0.6666666667	#DIV/0!
**IFIT2**	0.1428571429	0.1428571429	0.1666666667		0.6666666667	#DIV/0!
**IFIT3**	0.1428571429	0.1428571429	0.1666666667		0.6666666667	#DIV/0!
**IFIT5**	0.1428571429	0.1428571429	0.1111111111		1.083333333	#DIV/0!
**IFITM1**	0.04761904762	0.04761904762	0		inf	#DIV/0!
**IFITM10**	0.04761904762	0.04761904762	0		inf	#DIV/0!
**IFITM2**	0.04761904762	0.04761904762	0		inf	#DIV/0!
**IFITM3**	0.04761904762	0.04761904762	0		inf	#DIV/0!
**IFITM5**	0.04761904762	0.04761904762	0		inf	#DIV/0!
**IFNA1**	0.09523809524	0.09523809524	0.1111111111		0.6842105263	#DIV/0!
**IFNA10**	0.09523809524	0.09523809524	0.1111111111		0.6842105263	#DIV/0!
**IFNA13**	0.09523809524	0.09523809524	0.1111111111		0.6842105263	#DIV/0!
**IFNA14**	0.09523809524	0.09523809524	0.1111111111		0.6842105263	#DIV/0!
**IFNA16**	0.09523809524	0.09523809524	0.1111111111		0.6842105263	#DIV/0!
**IFNA17**	0.09523809524	0.09523809524	0.1111111111		0.6842105263	#DIV/0!
**IFNA2**	0.09523809524	0.09523809524	0.1111111111		0.6842105263	#DIV/0!
**IFNA21**	0.09523809524	0.09523809524	0.1111111111		0.6842105263	#DIV/0!
**FNA22P**	0.09523809524	0.09523809524	0.1111111111		0.6842105263	#DIV/0!
**IFNA4**	0.09523809524	0.09523809524	0.1111111111		0.6842105263	#DIV/0!
**IFNA5**	0.09523809524	0.09523809524	0.1111111111		0.6842105263	#DIV/0!
**IFNA6**	0.09523809524	0.09523809524	0.1111111111		0.6842105263	#DIV/0!
**IFNA7**	0.09523809524	0.09523809524	0.1111111111		0.6842105263	#DIV/0!
**IFNA8**	0.09523809524	0.09523809524	0.1111111111		0.6842105263	#DIV/0!
**IFNAR1**	0.04761904762	0.04761904762	0.1111111111		0.325	#DIV/0!
**IFNAR2**	0.04761904762	0.04761904762	0.1111111111		0.325	#DIV/0!
**IFNB1**	0.09523809524	0.09523809524	0.1111111111		0.6842105263	#DIV/0!
**IFNE**	0.09523809524	0.09523809524	0.1111111111		0.6842105263	#DIV/0!
**IFNG**	0.09523809524	0.09523809524	0.05555555556		1.473684211	#DIV/0!
**FNG-AS**	0.09523809524	0.09523809524	0.05555555556		1.473684211	#DIV/0!
**IFNGR2**	0.04761904762	0.04761904762	0.1111111111		0.325	#DIV/0!
**IFNK**	0.04761904762	0.04761904762	0.05555555556		0.7	#DIV/0!
**IFNL1**	0.04761904762	0.04761904762	0.05555555556		0.7	#DIV/0!
**IFNL2**	0.04761904762	0.04761904762	0.05555555556		0.7	#DIV/0!
**IFNL3**	0.04761904762	0.04761904762	0.05555555556		0.7	#DIV/0!
**IFNL4**	0.04761904762	0.04761904762	0.05555555556		0.7	#DIV/0!
**IFNLR1**	0.04761904762	0.04761904762	0.1111111111		0.325	#DIV/0!
**IFNW1**	0.09523809524	0.09523809524	0.1111111111		0.6842105263	#DIV/0!
**IFRD1**	0.04761904762	0.04761904762	0.05555555556		0.7	#DIV/0!
**IFRD2**	0.09523809524	0.09523809524	0		inf	#DIV/0!

IFT122	0.09523809524	0.09523809524	0.05555555556		1.473684211	#DIV/0!
IFT140	0.09523809524	0.09523809524	0.2222222222		0.2894736842	#DIV/0!
IFT20	0.04761904762	0.04761904762	0.05555555556		0.7	#DIV/0!
IFT22	0.04761904762	0.04761904762	0.05555555556		0.7	#DIV/0!
IFT27	0	0	0.1111111111		0	#DIV/0!
IFT52	0.1428571429	0.1428571429	0.1111111111		1.083333333	#DIV/0!
IFT57	0.09523809524	0.09523809524	0.05555555556		1.473684211	#DIV/0!
IFT74	0.04761904762	0.04761904762	0.05555555556		0.7	#DIV/0!
FT74-AS	0.04761904762	0.04761904762	0.05555555556		0.7	#DIV/0!
IFT80	0.1428571429	0.1428571429	0.05555555556		2.333333333	#DIV/0!
IFT81	0	0	0.1111111111		0	#DIV/0!
IFT88	0.04761904762	0.04761904762	0		inf	#DIV/0!
GBP1P1	0.09523809524	0.09523809524	0		inf	#DIV/0!
IGDCC3	0.04761904762	0.04761904762	0.05555555556		0.7	#DIV/0!
IGDCC4	0.04761904762	0.04761904762	0.05555555556		0.7	#DIV/0!
IGF1	0.04761904762	0.04761904762	0.05555555556		0.7	#DIV/0!
IGF1R	0.04761904762	0.04761904762	0.05555555556		0.7	#DIV/0!
IGF2	0.04761904762	0.04761904762	0		inf	#DIV/0!
IGF2-AS	0.04761904762	0.04761904762	0		inf	#DIV/0!
GF2BP1	0.1428571429	0.1428571429	0		inf	#DIV/0!
GF2BP2	0.1428571429	0.1428571429	0.05555555556		2.333333333	#DIV/0!
F2BP2-A	0.1428571429	0.1428571429	0.05555555556		2.333333333	#DIV/0!
GF2BP3	0.04761904762	0.04761904762	0.1666666667		0.2	#DIV/0!
IGFALS	0.09523809524	0.09523809524	0.2222222222		0.2894736842	#DIV/0!
IGFBP1	0.04761904762	0.04761904762	0.2222222222		0.1375	#DIV/0!
IGFBP2	0	0	0.1111111111		0	#DIV/0!
IGFBP3	0.04761904762	0.04761904762	0.2222222222		0.1375	#DIV/0!
IGFBP4	0.09523809524	0.09523809524	0		inf	#DIV/0!
IGFBP5	0	0	0.1111111111		0	#DIV/0!
IGFBP6	0.04761904762	0.04761904762	0.05555555556		0.7	#DIV/0!
IGFBP7	0.1904761905	0.1904761905	0.05555555556		3.294117647	#DIV/0!
FBP7-A	0.1904761905	0.1904761905	0.05555555556		3.294117647	#DIV/0!
GFBPL1	0.04761904762	0.04761904762	0.05555555556		0.7	#DIV/0!
IGFL1	0.09523809524	0.09523809524	0.1111111111		0.6842105263	#DIV/0!
IGFL2	0.09523809524	0.09523809524	0.1111111111		0.6842105263	#DIV/0!
GFL2-AS	0.09523809524	0.09523809524	0.1111111111		0.6842105263	#DIV/0!
IGFL3	0.09523809524	0.09523809524	0.1111111111		0.6842105263	#DIV/0!
IGFLR1	0.04761904762	0.04761904762	0.05555555556		0.7	#DIV/0!
IGFN1	0.1428571429	0.1428571429	0		inf	#DIV/0!
HV1OR1	0.2380952381	0.2380952381	0.1111111111		2.03125	#DIV/0!
HV1OR1	0.2380952381	0.2380952381	0.1111111111		2.03125	#DIV/0!
IGIP	0.04761904762	0.04761904762	0		inf	#DIV/0!
IGSF10	0.1904761905	0.1904761905	0.1111111111		1.529411765	#DIV/0!
IGSF11	0.09523809524	0.09523809524	0.05555555556		1.473684211	#DIV/0!
SF11-AS	0.09523809524	0.09523809524	0.05555555556		1.473684211	#DIV/0!
IGSF21	0.04761904762	0.04761904762	0.05555555556		0.7	#DIV/0!
IGSF23	0.09523809524	0.09523809524	0.05555555556		1.473684211	#DIV/0!
IGSF3	0.04761904762	0.04761904762	0.05555555556		0.7	#DIV/0!
IGSF5	0.04761904762	0.04761904762	0.05555555556		0.7	#DIV/0!
IGSF6	0.04761904762	0.04761904762	0		inf	#DIV/0!
IGSF8	0.1428571429	0.1428571429	0		inf	#DIV/0!

**IGSF9**	0.1428571429	0.1428571429	0		inf	#DIV/0!
**IHH**	0	0	0.05555555556		0	#DIV/0!
**IK**	0.04761904762	0.04761904762	0		inf	#DIV/0!
**IKBIP**	0.04761904762	0.04761904762	0.05555555556		0.7	#DIV/0!
**IKBKB**	0.09523809524	0.09523809524	0.1666666667		0.4210526316	#DIV/0!
**IKBKE**	0.1428571429	0.1428571429	0		inf	#DIV/0!
**IKZF1**	0.09523809524	0.09523809524	0.2222222222		0.2894736842	#DIV/0!
**IKZF2**	0	0	0.05555555556		0	#DIV/0!
**IKZF3**	0.09523809524	0.09523809524	0		inf	#DIV/0!
**IKZF4**	0.09523809524	0.09523809524	0.05555555556		1.473684211	#DIV/0!
**IKZF5**	0.2380952381	0.2380952381	0.05555555556		4.375	#DIV/0!
**IL10**	0.1428571429	0.1428571429	0		inf	#DIV/0!
**IL10RB**	0.04761904762	0.04761904762	0.1111111111		0.325	#DIV/0!
**L10RB-D**	0.04761904762	0.04761904762	0.1111111111		0.325	#DIV/0!
**IL11**	0.09523809524	0.09523809524	0.05555555556		1.473684211	#DIV/0!
**IL11RA**	0.09523809524	0.09523809524	0.05555555556		1.473684211	#DIV/0!
**IL12A**	0.1428571429	0.1428571429	0.05555555556		2.333333333	#DIV/0!
**L12A-AS**	0.1428571429	0.1428571429	0.05555555556		2.333333333	#DIV/0!
**IL12B**	0.04761904762	0.04761904762	0.05555555556		0.7	#DIV/0!
**IL12RB1**	0.1428571429	0.1428571429	0.1111111111		1.083333333	#DIV/0!
**IL12RB2**	0.04761904762	0.04761904762	0.05555555556		0.7	#DIV/0!
**IL13**	0.04761904762	0.04761904762	0		inf	#DIV/0!
**IL15**	0	0	0.05555555556		0	#DIV/0!
**IL15RA**	0.09523809524	0.09523809524	0.1111111111		0.6842105263	#DIV/0!
**IL16**	0.04761904762	0.04761904762	0.05555555556		0.7	#DIV/0!
**IL17A**	0.04761904762	0.04761904762	0		inf	#DIV/0!
**IL17C**	0.1428571429	0.1428571429	0		inf	#DIV/0!
**IL17D**	0.04761904762	0.04761904762	0		inf	#DIV/0!
**IL17F**	0.04761904762	0.04761904762	0		inf	#DIV/0!
**IL17RA**	0.04761904762	0.04761904762	0		inf	#DIV/0!
**IL17RB**	0.04761904762	0.04761904762	0		inf	#DIV/0!
**IL17RC**	0.09523809524	0.09523809524	0		inf	#DIV/0!
**IL17RD**	0.04761904762	0.04761904762	0		inf	#DIV/0!
**IL17RE**	0.09523809524	0.09523809524	0		inf	#DIV/0!
**IL17REI**	0	0	0.1111111111		0	#DIV/0!
**IL18R1**	0	0	0.05555555556		0	#DIV/0!
**IL18RAI**	0	0	0.05555555556		0	#DIV/0!
**IL19**	0.1428571429	0.1428571429	0		inf	#DIV/0!
**IL1A**	0	0	0.05555555556		0	#DIV/0!
**IL1B**	0	0	0.05555555556		0	#DIV/0!
**IL1F10**	0	0	0.05555555556		0	#DIV/0!
**IL1R1**	0	0	0.05555555556		0	#DIV/0!
**IL1R2**	0	0	0.05555555556		0	#DIV/0!
**IL1RAP**	0.09523809524	0.09523809524	0.1111111111		0.6842105263	#DIV/0!
**IL1RL1**	0	0	0.05555555556		0	#DIV/0!
**IL1RL2**	0	0	0.05555555556		0	#DIV/0!
**IL1RN**	0	0	0.05555555556		0	#DIV/0!
**IL2**	0	0	0.05555555556		0	#DIV/0!
**IL20**	0.1428571429	0.1428571429	0		inf	#DIV/0!
**IL20RB**	0.1904761905	0.1904761905	0.05555555556		3.294117647	#DIV/0!
**IL21**	0	0	0.05555555556		0	#DIV/0!

**L21-AS1**	0	0	0.05555555556		0	#DIV/0!
**IL21R**	0.04761904762	0.04761904762	0		inf	#DIV/0!
**L21R-AS**	0.04761904762	0.04761904762	0		inf	#DIV/0!
**IL22**	0.09523809524	0.09523809524	0.05555555556		1.473684211	#DIV/0!
**L22RA1**	0.04761904762	0.04761904762	0.1111111111		0.325	#DIV/0!
**IL23A**	0.1428571429	0.1428571429	0.05555555556		2.333333333	#DIV/0!
**IL23R**	0.04761904762	0.04761904762	0.05555555556		0.7	#DIV/0!
**IL24**	0.1428571429	0.1428571429	0		inf	#DIV/0!
**IL25**	0	0	0.05555555556		0	#DIV/0!
**IL26**	0.09523809524	0.09523809524	0.05555555556		1.473684211	#DIV/0!
**IL27**	0.04761904762	0.04761904762	0		inf	#DIV/0!
**IL27RA**	0.1428571429	0.1428571429	0.1111111111		1.083333333	#DIV/0!
**IL2RA**	0.09523809524	0.09523809524	0.1111111111		0.6842105263	#DIV/0!
**IL2RB**	0	0	0.1111111111		0	#DIV/0!
**IL3**	0.04761904762	0.04761904762	0		inf	#DIV/0!
**IL31**	0.04761904762	0.04761904762	0.1111111111		0.325	#DIV/0!
**IL31RA**	0.04761904762	0.04761904762	0.05555555556		0.7	#DIV/0!
**IL32**	0.04761904762	0.04761904762	0.1111111111		0.325	#DIV/0!
**IL33**	0.04761904762	0.04761904762	0.05555555556		0.7	#DIV/0!
**IL34**	0.1428571429	0.1428571429	0.05555555556		2.333333333	#DIV/0!
**IL36A**	0	0	0.05555555556		0	#DIV/0!
**IL36B**	0	0	0.05555555556		0	#DIV/0!
**IL36G**	0	0	0.05555555556		0	#DIV/0!
**IL36RN**	0	0	0.05555555556		0	#DIV/0!
**IL37**	0	0	0.05555555556		0	#DIV/0!
**IL4**	0.04761904762	0.04761904762	0		inf	#DIV/0!
**IL4I1**	0.09523809524	0.09523809524	0.1111111111		0.6842105263	#DIV/0!
**IL4R**	0.04761904762	0.04761904762	0		inf	#DIV/0!
**IL5**	0.04761904762	0.04761904762	0		inf	#DIV/0!
**IL5RA**	0.04761904762	0.04761904762	0		inf	#DIV/0!
**IL6**	0.04761904762	0.04761904762	0.1666666667		0.2	#DIV/0!
**IL6R**	0.1904761905	0.1904761905	0.05555555556		3.294117647	#DIV/0!
**L6R-AS**	0.1904761905	0.1904761905	0.05555555556		3.294117647	#DIV/0!
**IL6ST**	0.04761904762	0.04761904762	0.05555555556		0.7	#DIV/0!
**IL7**	0.1428571429	0.1428571429	0.1111111111		1.083333333	#DIV/0!
**IL7R**	0.04761904762	0.04761904762	0.05555555556		0.7	#DIV/0!
**IL9**	0.04761904762	0.04761904762	0		inf	#DIV/0!
**ILDR1**	0.09523809524	0.09523809524	0.05555555556		1.473684211	#DIV/0!
**ILDR2**	0.1428571429	0.1428571429	0		inf	#DIV/0!
**ILF2**	0.1904761905	0.1904761905	0.05555555556		3.294117647	#DIV/0!
**ILF3**	0.1428571429	0.1428571429	0.1111111111		1.083333333	#DIV/0!
**ILF3-DT**	0.1428571429	0.1428571429	0.1111111111		1.083333333	#DIV/0!
**ILK**	0.04761904762	0.04761904762	0		inf	#DIV/0!
**ILKAP**	0	0	0.05555555556		0	#DIV/0!
**ILRUN**	0.04761904762	0.04761904762	0		inf	#DIV/0!
**ILVBL**	0.1428571429	0.1428571429	0.1111111111		1.083333333	#DIV/0!
**MMP2L**	0.04761904762	0.04761904762	0.05555555556		0.7	#DIV/0!
**IMMT**	0	0	0.05555555556		0	#DIV/0!
**IMP3**	0.04761904762	0.04761904762	0.05555555556		0.7	#DIV/0!
**IMP4**	0	0	0.05555555556		0	#DIV/0!
**IMPA1**	0.1428571429	0.1428571429	0.1111111111		1.083333333	#DIV/0!

**MPA1P**	0.1428571429	0.1428571429	0.1111111111		1.083333333	#DIV/0!
**IMPA2**	0.09523809524	0.09523809524	0.05555555556		1.473684211	#DIV/0!
**IMPACT**	0.09523809524	0.09523809524	0.05555555556		1.473684211	#DIV/0!
**IMPAD1**	0.1428571429	0.1428571429	0.1111111111		1.083333333	#DIV/0!
**IMPDH1**	0.04761904762	0.04761904762	0.05555555556		0.7	#DIV/0!
**IMPDH2**	0.09523809524	0.09523809524	0		inf	#DIV/0!
**IMPG2**	0.09523809524	0.09523809524	0.05555555556		1.473684211	#DIV/0!
**INA**	0.1428571429	0.1428571429	0.05555555556		2.333333333	#DIV/0!
**INAFM1**	0.09523809524	0.09523809524	0.1111111111		0.6842105263	#DIV/0!
**INAVA**	0.1428571429	0.1428571429	0		inf	#DIV/0!
**INCA1**	0	0	0.1111111111		0	#DIV/0!
**ING2**	0	0	0.05555555556		0	#DIV/0!
**ING3**	0.04761904762	0.04761904762	0.05555555556		0.7	#DIV/0!
**ING4**	0.04761904762	0.04761904762	0.1111111111		0.325	#DIV/0!
**ING5**	0	0	0.05555555556		0	#DIV/0!
**INHA**	0	0	0.05555555556		0	#DIV/0!
**INHBA**	0.09523809524	0.09523809524	0.2222222222		0.2894736842	#DIV/0!
**HBA-AS**	0.09523809524	0.09523809524	0.2222222222		0.2894736842	#DIV/0!
**INHBB**	0	0	0.05555555556		0	#DIV/0!
**INIP**	0.04761904762	0.04761904762	0.05555555556		0.7	#DIV/0!
**INKA1**	0.09523809524	0.09523809524	0		inf	#DIV/0!
**INKA2**	0.04761904762	0.04761904762	0		inf	#DIV/0!
**KA2-AS**	0.04761904762	0.04761904762	0		inf	#DIV/0!
**INMT**	0.04761904762	0.04761904762	0.1111111111		0.325	#DIV/0!
**IT-MINI**	0.04761904762	0.04761904762	0.1111111111		0.325	#DIV/0!
**INO80B**	0	0	0.05555555556		0	#DIV/0!
**)80B-WI**	0	0	0.05555555556		0	#DIV/0!
**INO80C**	0.09523809524	0.09523809524	0		inf	#DIV/0!
**INO80D**	0	0	0.05555555556		0	#DIV/0!
**INO80E**	0.04761904762	0.04761904762	0		inf	#DIV/0!
**INPP1**	0	0	0.05555555556		0	#DIV/0!
**INPP4A**	0	0	0.05555555556		0	#DIV/0!
**INPP4B**	0	0	0.05555555556		0	#DIV/0!
**INPP5A**	0.2380952381	0.2380952381	0		inf	#DIV/0!
**INPP5B**	0.04761904762	0.04761904762	0.05555555556		0.7	#DIV/0!
**INPP5D**	0	0	0.05555555556		0	#DIV/0!
**INPP5E**	0.1428571429	0.1428571429	0.05555555556		2.333333333	#DIV/0!
**INPP5F**	0.2380952381	0.2380952381	0		inf	#DIV/0!
**INPP5J**	0.04761904762	0.04761904762	0.1111111111		0.325	#DIV/0!
**INPP5K**	0	0	0.1111111111		0	#DIV/0!
**INS**	0.04761904762	0.04761904762	0		inf	#DIV/0!
**NS-IGF2**	0.04761904762	0.04761904762	0		inf	#DIV/0!
**INSIG1**	0.1428571429	0.1428571429	0.05555555556		2.333333333	#DIV/0!
**INSIG2**	0	0	0.05555555556		0	#DIV/0!
**INSL3**	0.1428571429	0.1428571429	0.1111111111		1.083333333	#DIV/0!
**INSL4**	0.04761904762	0.04761904762	0.05555555556		0.7	#DIV/0!
**INSL5**	0.04761904762	0.04761904762	0.05555555556		0.7	#DIV/0!
**INSL6**	0.04761904762	0.04761904762	0.05555555556		0.7	#DIV/0!
**INSM1**	0.1904761905	0.1904761905	0.05555555556		3.294117647	#DIV/0!
**INSM2**	0.09523809524	0.09523809524	0		inf	#DIV/0!
**INSR**	0.1428571429	0.1428571429	0.05555555556		2.333333333	#DIV/0!

**INSRR**	0.1428571429	0.1428571429	0.05555555556		2.333333333	#DIV/0!
**INSYN1**	0.04761904762	0.04761904762	0.05555555556		0.7	#DIV/0!
**SYN1-A**	0.04761904762	0.04761904762	0.05555555556		0.7	#DIV/0!
**NSYN2A**	0.2380952381	0.2380952381	0.05555555556		4.375	#DIV/0!
**NSYN2B**	0	0	0.05555555556		0	#DIV/0!
**INTS1**	0.04761904762	0.04761904762	0.1666666667		0.2	#DIV/0!
**INTS10**	0.09523809524	0.09523809524	0.1111111111		0.6842105263	#DIV/0!
**INTS11**	0	0	0.1111111111		0	#DIV/0!
**INTS12**	0	0	0.05555555556		0	#DIV/0!
**INTS13**	0.04761904762	0.04761904762	0.05555555556		0.7	#DIV/0!
**INTS14**	0.04761904762	0.04761904762	0.05555555556		0.7	#DIV/0!
**INTS2**	0.09523809524	0.09523809524	0		inf	#DIV/0!
**INTS3**	0.1904761905	0.1904761905	0.05555555556		3.294117647	#DIV/0!
**NTS4P1**	0.04761904762	0.04761904762	0.1111111111		0.325	#DIV/0!
**NTS4P2**	0.04761904762	0.04761904762	0.1111111111		0.325	#DIV/0!
**INTS7**	0.1428571429	0.1428571429	0		inf	#DIV/0!
**INTS8**	0.1904761905	0.1904761905	0.1111111111		1.529411765	#DIV/0!
**INTS9**	0.04761904762	0.04761904762	0.1111111111		0.325	#DIV/0!
**INTU**	0	0	0.05555555556		0	#DIV/0!
**INVS**	0.04761904762	0.04761904762	0		inf	#DIV/0!
**IP6K1**	0.09523809524	0.09523809524	0		inf	#DIV/0!
**IP6K2**	0.09523809524	0.09523809524	0		inf	#DIV/0!
**IP6K3**	0.04761904762	0.04761904762	0		inf	#DIV/0!
**IPMK**	0.09523809524	0.09523809524	0.05555555556		1.473684211	#DIV/0!
**IPO11**	0.04761904762	0.04761904762	0		inf	#DIV/0!
**11-LRR**	0.04761904762	0.04761904762	0		inf	#DIV/0!
**IPO13**	0.04761904762	0.04761904762	0.05555555556		0.7	#DIV/0!
**IPO5P1**	0.09523809524	0.09523809524	0		inf	#DIV/0!
**IPO7**	0.04761904762	0.04761904762	0.05555555556		0.7	#DIV/0!
**IPO8**	0.04761904762	0.04761904762	0.05555555556		0.7	#DIV/0!
**IPO9**	0.1428571429	0.1428571429	0		inf	#DIV/0!
**PO9-AS**	0.1428571429	0.1428571429	0		inf	#DIV/0!
**IPP**	0.04761904762	0.04761904762	0.05555555556		0.7	#DIV/0!
**IPPK**	0.04761904762	0.04761904762	0.05555555556		0.7	#DIV/0!
**IQCA1**	0	0	0.05555555556		0	#DIV/0!
**IQCA1L**	0.09523809524	0.09523809524	0.05555555556		1.473684211	#DIV/0!
**IQCB1**	0.09523809524	0.09523809524	0.05555555556		1.473684211	#DIV/0!
**IQCC**	0.04761904762	0.04761904762	0.1111111111		0.325	#DIV/0!
**IQCD**	0	0	0.05555555556		0	#DIV/0!
**IQCE**	0.04761904762	0.04761904762	0.1666666667		0.2	#DIV/0!
**IQCF1**	0.04761904762	0.04761904762	0		inf	#DIV/0!
**IQCF2**	0.04761904762	0.04761904762	0		inf	#DIV/0!
**IQCF3**	0.04761904762	0.04761904762	0		inf	#DIV/0!
**IQCF4**	0.04761904762	0.04761904762	0		inf	#DIV/0!
**IQCF5**	0.04761904762	0.04761904762	0		inf	#DIV/0!
**QCF5-AS**	0.04761904762	0.04761904762	0		inf	#DIV/0!
**IQCF6**	0.04761904762	0.04761904762	0		inf	#DIV/0!
**IQCG**	0.1428571429	0.1428571429	0.05555555556		2.333333333	#DIV/0!
**IQCH**	0.04761904762	0.04761904762	0.05555555556		0.7	#DIV/0!
**QCH-AS**	0.04761904762	0.04761904762	0.05555555556		0.7	#DIV/0!
**IQCJ**	0.1428571429	0.1428571429	0.05555555556		2.333333333	#DIV/0!

CJ-SCHI	0.1428571429	0.1428571429	0.05555555556		2.333333333	#DIV/0!
-SCHIP1	0.1428571429	0.1428571429	0.05555555556		2.333333333	#DIV/0!
IQCK	0.04761904762	0.04761904762	0		inf	#DIV/0!
IQCM	0	0	0.05555555556		0	#DIV/0!
IQCN	0.1428571429	0.1428571429	0.1111111111		1.083333333	#DIV/0!
IQGAP1	0.04761904762	0.04761904762	0.05555555556		0.7	#DIV/0!
IQGAP2	0.04761904762	0.04761904762	0		inf	#DIV/0!
IQGAP3	0.1428571429	0.1428571429	0.1111111111		1.083333333	#DIV/0!
IQSEC1	0.04761904762	0.04761904762	0		inf	#DIV/0!
IQSEC3	0.04761904762	0.04761904762	0.1111111111		0.325	#DIV/0!
IQUB	0.04761904762	0.04761904762	0.05555555556		0.7	#DIV/0!
IRAIN	0.04761904762	0.04761904762	0.05555555556		0.7	#DIV/0!
IRAK2	0.09523809524	0.09523809524	0		inf	#DIV/0!
IRAK3	0.09523809524	0.09523809524	0.05555555556		1.473684211	#DIV/0!
IRAK4	0.04761904762	0.04761904762	0.05555555556		0.7	#DIV/0!
IREB2	0.04761904762	0.04761904762	0.05555555556		0.7	#DIV/0!
IRF1	0.04761904762	0.04761904762	0		inf	#DIV/0!
IRF2	0	0	0.05555555556		0	#DIV/0!
IRF2BP1	0.09523809524	0.09523809524	0.1111111111		0.6842105263	#DIV/0!
IRF2BP2	0.09523809524	0.09523809524	0.05555555556		1.473684211	#DIV/0!
IRF3	0.09523809524	0.09523809524	0.1111111111		0.6842105263	#DIV/0!
IRF5	0.04761904762	0.04761904762	0.05555555556		0.7	#DIV/0!
IRF6	0.1428571429	0.1428571429	0		inf	#DIV/0!
IRF7	0.04761904762	0.04761904762	0		inf	#DIV/0!
IRF8	0.04761904762	0.04761904762	0		inf	#DIV/0!
IRGC	0.09523809524	0.09523809524	0.05555555556		1.473684211	#DIV/0!
IRGQ	0.09523809524	0.09523809524	0.05555555556		1.473684211	#DIV/0!
IRS1	0	0	0.05555555556		0	#DIV/0!
IRX1	0	0	0.1111111111		0	#DIV/0!
IRX2	0	0	0.1111111111		0	#DIV/0!
IRX3	0.04761904762	0.04761904762	0		inf	#DIV/0!
IRX4	0	0	0.1111111111		0	#DIV/0!
IRX5	0.04761904762	0.04761904762	0		inf	#DIV/0!
IRX6	0.04761904762	0.04761904762	0		inf	#DIV/0!
ISCA1	0	0	0.05555555556		0	#DIV/0!
ISCU	0.04761904762	0.04761904762	0.05555555556		0.7	#DIV/0!
ISG15	0	0	0.1111111111		0	#DIV/0!
ISG20	0.04761904762	0.04761904762	0.05555555556		0.7	#DIV/0!
ISG20L2	0.1428571429	0.1428571429	0.1111111111		1.083333333	#DIV/0!
ISL1	0.04761904762	0.04761904762	0.05555555556		0.7	#DIV/0!
ISL2	0.04761904762	0.04761904762	0.05555555556		0.7	#DIV/0!
ISLR	0.04761904762	0.04761904762	0.05555555556		0.7	#DIV/0!
ISLR2	0.04761904762	0.04761904762	0.05555555556		0.7	#DIV/0!
ISM1	0.1904761905	0.1904761905	0.1111111111		1.529411765	#DIV/0!
SM1-AS	0.1904761905	0.1904761905	0.1111111111		1.529411765	#DIV/0!
ISOC1	0.04761904762	0.04761904762	0		inf	#DIV/0!
ISOC2	0.09523809524	0.09523809524	0.05555555556		1.473684211	#DIV/0!
IST1	0.04761904762	0.04761904762	0		inf	#DIV/0!
ISX	0	0	0.1666666667		0	#DIV/0!
ISX-AS1	0	0	0.1666666667		0	#DIV/0!
ISY1	0.09523809524	0.09523809524	0.05555555556		1.473684211	#DIV/0!

**Y1-RAB**	0.09523809524	0.09523809524	0.05555555556		1.473684211	#DIV/0!
**ISYNA1**	0.1428571429	0.1428571429	0.1111111111		1.083333333	#DIV/0!
**ITCH**	0.09523809524	0.09523809524	0.1111111111		0.6842105263	#DIV/0!
**ITFG1**	0.04761904762	0.04761904762	0		inf	#DIV/0!
**TFG1-AS**	0.04761904762	0.04761904762	0		inf	#DIV/0!
**ITFG2**	0.04761904762	0.04761904762	0.1666666667		0.2	#DIV/0!
**TFG2-AS**	0.04761904762	0.04761904762	0.1666666667		0.2	#DIV/0!
**ITGA1**	0.04761904762	0.04761904762	0.05555555556		0.7	#DIV/0!
**ITGA10**	0.1428571429	0.1428571429	0.05555555556		2.333333333	#DIV/0!
**ITGA11**	0.04761904762	0.04761904762	0.05555555556		0.7	#DIV/0!
**ITGA2**	0.04761904762	0.04761904762	0.05555555556		0.7	#DIV/0!
**ITGA2B**	0.09523809524	0.09523809524	0		inf	#DIV/0!
**ITGA3**	0.09523809524	0.09523809524	0		inf	#DIV/0!
**ITGA4**	0	0	0.05555555556		0	#DIV/0!
**ITGA5**	0.04761904762	0.04761904762	0.05555555556		0.7	#DIV/0!
**ITGA6**	0	0	0.1111111111		0	#DIV/0!
**TGA6-AS**	0	0	0.1111111111		0	#DIV/0!
**ITGA7**	0.04761904762	0.04761904762	0.05555555556		0.7	#DIV/0!
**ITGA8**	0.09523809524	0.09523809524	0.1111111111		0.6842105263	#DIV/0!
**ITGA9**	0.04761904762	0.04761904762	0		inf	#DIV/0!
**TGA9-AS**	0.04761904762	0.04761904762	0		inf	#DIV/0!
**ITGAD**	0.04761904762	0.04761904762	0		inf	#DIV/0!
**ITGAE**	0	0	0.1111111111		0	#DIV/0!
**ITGAL**	0.04761904762	0.04761904762	0		inf	#DIV/0!
**ITGAM**	0.04761904762	0.04761904762	0		inf	#DIV/0!
**ITGAV**	0	0	0.05555555556		0	#DIV/0!
**ITGAX**	0.04761904762	0.04761904762	0		inf	#DIV/0!
**ITGB1**	0.09523809524	0.09523809524	0.2222222222		0.2894736842	#DIV/0!
**TGB1BP**	0	0	0.05555555556		0	#DIV/0!
**ITGB2**	0.04761904762	0.04761904762	0.1111111111		0.325	#DIV/0!
**TGB2-AS**	0.04761904762	0.04761904762	0.1111111111		0.325	#DIV/0!
**ITGB3**	0.1428571429	0.1428571429	0		inf	#DIV/0!
**TGB3BI**	0.04761904762	0.04761904762	0.05555555556		0.7	#DIV/0!
**ITGB4**	0.04761904762	0.04761904762	0.05555555556		0.7	#DIV/0!
**ITGB5**	0.04761904762	0.04761904762	0.05555555556		0.7	#DIV/0!
**ITGB6**	0	0	0.05555555556		0	#DIV/0!
**ITGB7**	0.04761904762	0.04761904762	0.05555555556		0.7	#DIV/0!
**ITGB8**	0.04761904762	0.04761904762	0.2222222222		0.1375	#DIV/0!
**ITIH1**	0.04761904762	0.04761904762	0		inf	#DIV/0!
**ITIH2**	0.09523809524	0.09523809524	0.1111111111		0.6842105263	#DIV/0!
**ITIH3**	0.04761904762	0.04761904762	0		inf	#DIV/0!
**ITIH4**	0.04761904762	0.04761904762	0		inf	#DIV/0!
**TIH4-AS**	0.04761904762	0.04761904762	0		inf	#DIV/0!
**ITIH5**	0.09523809524	0.09523809524	0.1111111111		0.6842105263	#DIV/0!
**ITK**	0.04761904762	0.04761904762	0.05555555556		0.7	#DIV/0!
**ITLN1**	0.1428571429	0.1428571429	0		inf	#DIV/0!
**ITLN2**	0.1428571429	0.1428571429	0		inf	#DIV/0!
**ITM2C**	0	0	0.05555555556		0	#DIV/0!
**ITPA**	0.1904761905	0.1904761905	0.05555555556		3.294117647	#DIV/0!
**ITPKB**	0.1428571429	0.1428571429	0		inf	#DIV/0!
**TPKB-IT**	0.1428571429	0.1428571429	0		inf	#DIV/0!

ITPKC	0.04761904762	0.04761904762	0.05555555556		0.7	#DIV/0!
ITPR1	0.04761904762	0.04761904762	0		inf	#DIV/0!
TPR1-D'	0.04761904762	0.04761904762	0		inf	#DIV/0!
ITPR2	0.04761904762	0.04761904762	0.05555555556		0.7	#DIV/0!
ITPR3	0.04761904762	0.04761904762	0		inf	#DIV/0!
TPRID1	0.09523809524	0.09523809524	0.1111111111		0.6842105263	#DIV/0!
TPRID2	0	0	0.05555555556		0	#DIV/0!
ITPRIP	0.1428571429	0.1428571429	0.05555555556		2.333333333	#DIV/0!
PRIP-A:	0.1428571429	0.1428571429	0.05555555556		2.333333333	#DIV/0!
TPRIPL	0	0	0.1111111111		0	#DIV/0!
TPRIPL	0.04761904762	0.04761904762	0		inf	#DIV/0!
ITSN1	0.04761904762	0.04761904762	0.1111111111		0.325	#DIV/0!
IVL	0.1904761905	0.1904761905	0		inf	#DIV/0!
VNS1AB	0.1428571429	0.1428571429	0.05555555556		2.333333333	#DIV/0!
IWS1	0	0	0.05555555556		0	#DIV/0!
ZUMO1	0.09523809524	0.09523809524	0.1111111111		0.6842105263	#DIV/0!
ZUMO2	0.09523809524	0.09523809524	0.1111111111		0.6842105263	#DIV/0!
ZUMO3	0.04761904762	0.04761904762	0.05555555556		0.7	#DIV/0!
ZUMO4	0.09523809524	0.09523809524	0.1111111111		0.6842105263	#DIV/0!
JADE1	0	0	0.05555555556		0	#DIV/0!
JADE2	0.04761904762	0.04761904762	0		inf	#DIV/0!
JAG1	0.1904761905	0.1904761905	0.1666666667		0.9411764706	#DIV/0!
JAGN1	0.09523809524	0.09523809524	0		inf	#DIV/0!
JAK1	0.04761904762	0.04761904762	0.05555555556		0.7	#DIV/0!
JAK2	0.04761904762	0.04761904762	0.05555555556		0.7	#DIV/0!
JAK3	0.1428571429	0.1428571429	0.1111111111		1.083333333	#DIV/0!
AKMIP:	0.2380952381	0.2380952381	0		inf	#DIV/0!
JAM2	0.04761904762	0.04761904762	0.1111111111		0.325	#DIV/0!
JAZF1	0.04761904762	0.04761904762	0.2222222222		0.1375	#DIV/0!
AZF1-AS	0.04761904762	0.04761904762	0.2222222222		0.1375	#DIV/0!
JCAD	0.09523809524	0.09523809524	0.2222222222		0.2894736842	#DIV/0!
JCHAIN	0.09523809524	0.09523809524	0.05555555556		1.473684211	#DIV/0!
JMJD1C	0.09523809524	0.09523809524	0.1111111111		0.6842105263	#DIV/0!
JD1C-A	0.09523809524	0.09523809524	0.1111111111		0.6842105263	#DIV/0!
JMJD4	0.1428571429	0.1428571429	0		inf	#DIV/0!
JMJD6	0.04761904762	0.04761904762	0		inf	#DIV/0!
JMJD8	0.04761904762	0.04761904762	0.2222222222		0.1375	#DIV/0!
JMY	0.04761904762	0.04761904762	0		inf	#DIV/0!
JOSD1	0.04761904762	0.04761904762	0.1111111111		0.325	#DIV/0!
JOSD2	0.09523809524	0.09523809524	0.1111111111		0.6842105263	#DIV/0!
JPH1	0.1904761905	0.1904761905	0.1111111111		1.529411765	#DIV/0!
JPH2	0.1428571429	0.1428571429	0.1666666667		0.6666666667	#DIV/0!
JPH3	0.04761904762	0.04761904762	0		inf	#DIV/0!
JPT1	0.04761904762	0.04761904762	0.05555555556		0.7	#DIV/0!
JPT2	0.09523809524	0.09523809524	0.2222222222		0.2894736842	#DIV/0!
JRK	0.04761904762	0.04761904762	0.1666666667		0.2	#DIV/0!
JSRP1	0.09523809524	0.09523809524	0.1111111111		0.6842105263	#DIV/0!
JTB	0.1904761905	0.1904761905	0.05555555556		3.294117647	#DIV/0!
JUN	0.04761904762	0.04761904762	0.05555555556		0.7	#DIV/0!
JUNB	0.1428571429	0.1428571429	0.1111111111		1.083333333	#DIV/0!
JUND	0.1428571429	0.1428571429	0.1111111111		1.083333333	#DIV/0!

**JUP**	0.09523809524	0.09523809524	0		inf	#DIV/0!
**KALRN**	0.04761904762	0.04761904762	0.05555555556		0.7	#DIV/0!
**KANK1**	0.04761904762	0.04761904762	0.05555555556		0.7	#DIV/0!
**KANK2**	0.1428571429	0.1428571429	0.1111111111		1.083333333	#DIV/0!
**KANK3**	0.1428571429	0.1428571429	0.05555555556		2.333333333	#DIV/0!
**KANK4**	0.04761904762	0.04761904762	0.05555555556		0.7	#DIV/0!
**KANSL1**	0.1428571429	0.1428571429	0		inf	#DIV/0!
**NSL1-A**	0.1428571429	0.1428571429	0		inf	#DIV/0!
**ANSL1**	0	0	0.05555555556		0	#DIV/0!
**KANSL2**	0.04761904762	0.04761904762	0.05555555556		0.7	#DIV/0!
**KANSL3**	0	0	0.1111111111		0	#DIV/0!
**KARS**	0.04761904762	0.04761904762	0		inf	#DIV/0!
**KAT14**	0.1904761905	0.1904761905	0.05555555556		3.294117647	#DIV/0!
**KAT2A**	0.04761904762	0.04761904762	0		inf	#DIV/0!
**KAT2B**	0.04761904762	0.04761904762	0		inf	#DIV/0!
**KAT6A**	0.09523809524	0.09523809524	0.1666666667		0.4210526316	#DIV/0!
**KAT6B**	0.1904761905	0.1904761905	0.05555555556		3.294117647	#DIV/0!
**KAT7**	0.09523809524	0.09523809524	0		inf	#DIV/0!
**KAT8**	0.04761904762	0.04761904762	0		inf	#DIV/0!
**KATNA1**	0	0	0			#DIV/0!
**ATNAL**	0.04761904762	0.04761904762	0.05555555556		0.7	#DIV/0!
**KATNB1**	0.04761904762	0.04761904762	0		inf	#DIV/0!
**AZALD**	0.1428571429	0.1428571429	0		inf	#DIV/0!
**KAZN**	0.04761904762	0.04761904762	0.1111111111		0.325	#DIV/0!
**AZN-AS**	0.04761904762	0.04761904762	0.1111111111		0.325	#DIV/0!
**KBTBD1**	0.1428571429	0.1428571429	0.1111111111		1.083333333	#DIV/0!
**TBD11-C**	0.1428571429	0.1428571429	0.1111111111		1.083333333	#DIV/0!
**KBTBD1**	0.09523809524	0.09523809524	0.05555555556		1.473684211	#DIV/0!
**KBTBD1**	0.04761904762	0.04761904762	0.05555555556		0.7	#DIV/0!
**KBTBD2**	0.04761904762	0.04761904762	0.1666666667		0.2	#DIV/0!
**KBTBD4**	0	0	0.05555555556		0	#DIV/0!
**KBTBD8**	0.04761904762	0.04761904762	0		inf	#DIV/0!
**KC6**	0.04761904762	0.04761904762	0		inf	#DIV/0!
**CCAT1**	0.04761904762	0.04761904762	0.05555555556		0.7	#DIV/0!
**CCAT3**	0.04761904762	0.04761904762	0.2222222222		0.1375	#DIV/0!
**KCMF1**	0	0	0.05555555556		0	#DIV/0!
**KCNA1**	0.04761904762	0.04761904762	0.1111111111		0.325	#DIV/0!
**KCNA10**	0.04761904762	0.04761904762	0		inf	#DIV/0!
**KCNA2**	0.04761904762	0.04761904762	0		inf	#DIV/0!
**KCNA3**	0.04761904762	0.04761904762	0		inf	#DIV/0!
**KCNA5**	0.04761904762	0.04761904762	0.1111111111		0.325	#DIV/0!
**KCNA6**	0.04761904762	0.04761904762	0.1111111111		0.325	#DIV/0!
**KCNA7**	0.09523809524	0.09523809524	0.1111111111		0.6842105263	#DIV/0!
**KCNAB1**	0.1428571429	0.1428571429	0.05555555556		2.333333333	#DIV/0!
**NAB1-A**	0.1428571429	0.1428571429	0.05555555556		2.333333333	#DIV/0!
**NAB1-A**	0.1428571429	0.1428571429	0.05555555556		2.333333333	#DIV/0!
**KCNAB2**	0	0	0.05555555556		0	#DIV/0!
**KCNAB3**	0	0	0.05555555556		0	#DIV/0!
**KCNB1**	0.09523809524	0.09523809524	0.1666666667		0.4210526316	#DIV/0!
**KCNB2**	0.1904761905	0.1904761905	0.1666666667		0.9411764706	#DIV/0!
**KCNC2**	0.04761904762	0.04761904762	0.1111111111		0.325	#DIV/0!

KCNC3	0.09523809524	0.09523809524	0.1111111111		0.6842105263	#DIV/0!
KCNC4	0.04761904762	0.04761904762	0		inf	#DIV/0!
KCND2	0.04761904762	0.04761904762	0.05555555556		0.7	#DIV/0!
KCND3	0.04761904762	0.04761904762	0		inf	#DIV/0!
CND3-A	0.04761904762	0.04761904762	0		inf	#DIV/0!
CND3-IT	0.04761904762	0.04761904762	0		inf	#DIV/0!
KCNE1	0.04761904762	0.04761904762	0.1666666667		0.2	#DIV/0!
KCNE1B	0.04761904762	0.04761904762	0.1111111111		0.325	#DIV/0!
KCNE2	0.04761904762	0.04761904762	0.1111111111		0.325	#DIV/0!
KCNE4	0	0	0.05555555556		0	#DIV/0!
KCNF1	0	0	0.05555555556		0	#DIV/0!
KCNG1	0.09523809524	0.09523809524	0.1666666667		0.4210526316	#DIV/0!
KCNG2	0.04761904762	0.04761904762	0.05555555556		0.7	#DIV/0!
KCNG3	0	0	0.05555555556		0	#DIV/0!
KCNG4	0.04761904762	0.04761904762	0		inf	#DIV/0!
KCNH1	0.1428571429	0.1428571429	0		inf	#DIV/0!
CNH1-IT	0.1428571429	0.1428571429	0		inf	#DIV/0!
KCNH2	0.09523809524	0.09523809524	0.05555555556		1.473684211	#DIV/0!
KCNH3	0.04761904762	0.04761904762	0.05555555556		0.7	#DIV/0!
KCNH4	0.04761904762	0.04761904762	0		inf	#DIV/0!
KCNH6	0.04761904762	0.04761904762	0		inf	#DIV/0!
KCNH7	0	0	0.05555555556		0	#DIV/0!
KCNH8	0.04761904762	0.04761904762	0		inf	#DIV/0!
KCNIP1	0	0	0.05555555556		0	#DIV/0!
KCNIP2	0.1428571429	0.1428571429	0		inf	#DIV/0!
CNIP2-A	0.1428571429	0.1428571429	0		inf	#DIV/0!
KCNIP3	0	0	0.1111111111		0	#DIV/0!
KCNJ10	0.1428571429	0.1428571429	0		inf	#DIV/0!
KCNJ12	0.09523809524	0.09523809524	0.05555555556		1.473684211	#DIV/0!
KCNJ13	0	0	0.05555555556		0	#DIV/0!
KCNJ14	0.09523809524	0.09523809524	0.1111111111		0.6842105263	#DIV/0!
KCNJ15	0.04761904762	0.04761904762	0.1111111111		0.325	#DIV/0!
KCNJ16	0	0	0			#DIV/0!
KCNJ18	0.09523809524	0.09523809524	0.05555555556		1.473684211	#DIV/0!
KCNJ2	0	0	0			#DIV/0!
CNJ2-AS	0	0	0			#DIV/0!
KCNJ3	0	0	0.05555555556		0	#DIV/0!
KCNJ4	0.04761904762	0.04761904762	0.1111111111		0.325	#DIV/0!
KCNJ6	0.04761904762	0.04761904762	0.1111111111		0.325	#DIV/0!
KCNJ8	0.04761904762	0.04761904762	0.05555555556		0.7	#DIV/0!
KCNJ9	0.1428571429	0.1428571429	0		inf	#DIV/0!
KCNK1	0.1428571429	0.1428571429	0		inf	#DIV/0!
KCNK12	0	0	0.05555555556		0	#DIV/0!
KCNK15	0.1428571429	0.1428571429	0.1666666667		0.6666666667	#DIV/0!
NK15-A	0.1428571429	0.1428571429	0.1666666667		0.6666666667	#DIV/0!
KCNK16	0.04761904762	0.04761904762	0.05555555556		0.7	#DIV/0!
KCNK17	0.04761904762	0.04761904762	0.05555555556		0.7	#DIV/0!
KCNK18	0.2380952381	0.2380952381	0		inf	#DIV/0!
KCNK2	0.1428571429	0.1428571429	0		inf	#DIV/0!
KCNK5	0.04761904762	0.04761904762	0.05555555556		0.7	#DIV/0!
KCNK6	0.04761904762	0.04761904762	0.05555555556		0.7	#DIV/0!

KCNK9	0.04761904762	0.04761904762	0.1111111111		0.325	#DIV/0!
KCNMA	0.1428571429	0.1428571429	0.1111111111		1.083333333	#DIV/0!
NMA1-A	0.1428571429	0.1428571429	0.1111111111		1.083333333	#DIV/0!
NMA1-A	0.1428571429	0.1428571429	0.1111111111		1.083333333	#DIV/0!
NMA1-A	0.1428571429	0.1428571429	0.1111111111		1.083333333	#DIV/0!
KCNMB	0	0	0.05555555556		0	#DIV/0!
KCNMB	0.1904761905	0.1904761905	0.05555555556		3.294117647	#DIV/0!
NMB2-A	0.1904761905	0.1904761905	0.05555555556		3.294117647	#DIV/0!
KCNMB	0.1904761905	0.1904761905	0.05555555556		3.294117647	#DIV/0!
KCNMB	0.09523809524	0.09523809524	0.1111111111		0.6842105263	#DIV/0!
KCNN1	0.1428571429	0.1428571429	0.1111111111		1.083333333	#DIV/0!
KCNN2	0.04761904762	0.04761904762	0		inf	#DIV/0!
KCNN3	0.1904761905	0.1904761905	0.1111111111		1.529411765	#DIV/0!
KCNN4	0.09523809524	0.09523809524	0.05555555556		1.473684211	#DIV/0!
KCNQ1	0.04761904762	0.04761904762	0		inf	#DIV/0!
CNQ1-A	0.04761904762	0.04761904762	0		inf	#DIV/0!
CNQ1D	0.04761904762	0.04761904762	0		inf	#DIV/0!
CNQ1OT	0.04761904762	0.04761904762	0		inf	#DIV/0!
KCNQ2	0.1428571429	0.1428571429	0.2222222222		0.4583333333	#DIV/0!
KCNQ3	0.04761904762	0.04761904762	0.1111111111		0.325	#DIV/0!
KCNQ4	0.04761904762	0.04761904762	0.05555555556		0.7	#DIV/0!
KCNQ5	0.04761904762	0.04761904762	0		inf	#DIV/0!
KCNS1	0.1428571429	0.1428571429	0.1666666667		0.6666666667	#DIV/0!
KCNS2	0.1904761905	0.1904761905	0.1111111111		1.529411765	#DIV/0!
KCNS3	0	0	0.05555555556		0	#DIV/0!
KCNT1	0.04761904762	0.04761904762	0		inf	#DIV/0!
KCNT2	0.1428571429	0.1428571429	0		inf	#DIV/0!
KCNU1	0.04761904762	0.04761904762	0.1111111111		0.325	#DIV/0!
KCNV1	0.1904761905	0.1904761905	0.1111111111		1.529411765	#DIV/0!
KCNV2	0.04761904762	0.04761904762	0.05555555556		0.7	#DIV/0!
KCP	0.04761904762	0.04761904762	0.05555555556		0.7	#DIV/0!
KCTD1	0.09523809524	0.09523809524	0.05555555556		1.473684211	#DIV/0!
KCTD10	0	0	0.05555555556		0	#DIV/0!
KCTD11	0	0	0.05555555556		0	#DIV/0!
KCTD12	0	0	0.05555555556		0	#DIV/0!
KCTD13	0.04761904762	0.04761904762	0		inf	#DIV/0!
KCTD15	0.04761904762	0.04761904762	0.05555555556		0.7	#DIV/0!
KCTD17	0	0	0.1111111111		0	#DIV/0!
KCTD18	0	0	0.05555555556		0	#DIV/0!
KCTD19	0.1428571429	0.1428571429	0.05555555556		2.333333333	#DIV/0!
KCTD2	0.04761904762	0.04761904762	0.05555555556		0.7	#DIV/0!
KCTD20	0.04761904762	0.04761904762	0.05555555556		0.7	#DIV/0!
KCTD3	0.1428571429	0.1428571429	0		inf	#DIV/0!
KCTD5	0.04761904762	0.04761904762	0.1111111111		0.325	#DIV/0!
KCTD6	0.04761904762	0.04761904762	0		inf	#DIV/0!
KCTD7	0.04761904762	0.04761904762	0.1111111111		0.325	#DIV/0!
KCTD8	0.04761904762	0.04761904762	0		inf	#DIV/0!
KCTD9	0.04761904762	0.04761904762	0.1111111111		0.325	#DIV/0!
KDELR1	0.09523809524	0.09523809524	0.1111111111		0.6842105263	#DIV/0!
KDELR3	0.04761904762	0.04761904762	0.1111111111		0.325	#DIV/0!
KDF1	0.04761904762	0.04761904762	0.1111111111		0.325	#DIV/0!

KDM1A	0.04761904762	0.04761904762	0.1111111111		0.325	#DIV/0!
KDM2B	0.04761904762	0.04761904762	0.1111111111		0.325	#DIV/0!
KDM3A	0	0	0.05555555556		0	#DIV/0!
KDM3B	0.04761904762	0.04761904762	0		inf	#DIV/0!
KDM4A	0.04761904762	0.04761904762	0.05555555556		0.7	#DIV/0!
DM4A-A	0.04761904762	0.04761904762	0.05555555556		0.7	#DIV/0!
KDM4B	0.1428571429	0.1428571429	0.05555555556		2.333333333	#DIV/0!
KDM4C	0.04761904762	0.04761904762	0.05555555556		0.7	#DIV/0!
KDM5A	0.04761904762	0.04761904762	0.1111111111		0.325	#DIV/0!
KDM5B	0.1428571429	0.1428571429	0		inf	#DIV/0!
KDM6B	0	0	0.05555555556		0	#DIV/0!
KDM7A	0.04761904762	0.04761904762	0.05555555556		0.7	#DIV/0!
DM7A-D	0.04761904762	0.04761904762	0.05555555556		0.7	#DIV/0!
KDM8	0.04761904762	0.04761904762	0		inf	#DIV/0!
KDR	0.1904761905	0.1904761905	0.05555555556		3.294117647	#DIV/0!
KDSR	0.09523809524	0.09523809524	0		inf	#DIV/0!
KEAP1	0.1428571429	0.1428571429	0.1111111111		1.083333333	#DIV/0!
KEL	0.04761904762	0.04761904762	0.05555555556		0.7	#DIV/0!
KERA	0.04761904762	0.04761904762	0.05555555556		0.7	#DIV/0!
KHDC1	0.04761904762	0.04761904762	0		inf	#DIV/0!
KHDC1L	0.04761904762	0.04761904762	0		inf	#DIV/0!
KHDC3L	0.04761904762	0.04761904762	0		inf	#DIV/0!
KHDC4	0.1904761905	0.1904761905	0.1111111111		1.529411765	#DIV/0!
HDRBS	0.04761904762	0.04761904762	0.1111111111		0.325	#DIV/0!
HDRBS	0.04761904762	0.04761904762	0.2222222222		0.1375	#DIV/0!
KHSRP	0.1428571429	0.1428571429	0.05555555556		2.333333333	#DIV/0!
IAA004	0.1428571429	0.1428571429	0		inf	#DIV/0!
IAA008	0.04761904762	0.04761904762	0.2222222222		0.1375	#DIV/0!
IAA010	0.04761904762	0.04761904762	0.05555555556		0.7	#DIV/0!
IAA031	0	0	0.05555555556		0	#DIV/0!
IAA0319	0.04761904762	0.04761904762	0.05555555556		0.7	#DIV/0!
IAA035	0.04761904762	0.04761904762	0.05555555556		0.7	#DIV/0!
IAA039	0.09523809524	0.09523809524	0		inf	#DIV/0!
IAA051	0.04761904762	0.04761904762	0		inf	#DIV/0!
IAA055	0.04761904762	0.04761904762	0		inf	#DIV/0!
IAA058	0.04761904762	0.04761904762	0		inf	#DIV/0!
IAA075	0	0	0.05555555556		0	#DIV/0!
IAA075	0.04761904762	0.04761904762	0.05555555556		0.7	#DIV/0!
IAA082	0.04761904762	0.04761904762	0		inf	#DIV/0!
IAA089	0.04761904762	0.04761904762	0.2222222222		0.1375	#DIV/0!
IAA0895	0.1428571429	0.1428571429	0.05555555556		2.333333333	#DIV/0!
IAA093	0	0	0.1111111111		0	#DIV/0!
IAA110	0.04761904762	0.04761904762	0.05555555556		0.7	#DIV/0!
IAA110	0	0	0.05555555556		0	#DIV/0!
IAA114	0.04761904762	0.04761904762	0.1111111111		0.325	#DIV/0!
IAA119	0.09523809524	0.09523809524	0.05555555556		1.473684211	#DIV/0!
IAA121	0.1904761905	0.1904761905	0.05555555556		3.294117647	#DIV/0!
IAA1211	0	0	0.05555555556		0	#DIV/0!
IAA121	0.09523809524	0.09523809524	0.1111111111		0.6842105263	#DIV/0!
IAA125	0.09523809524	0.09523809524	0.05555555556		1.473684211	#DIV/0!
IAA132	0.04761904762	0.04761904762	0.05555555556		0.7	#DIV/0!

**IAA1324**	0.09523809524	0.09523809524	0.05555555556		1.473684211	#DIV/0!
**KIAA132**	0.04761904762	0.04761904762	0		inf	#DIV/0!
**KIAA152**	0.04761904762	0.04761904762	0.1111111111		0.325	#DIV/0!
**KIAA154**	0.04761904762	0.04761904762	0.05555555556		0.7	#DIV/0!
**KIAA158**	0.04761904762	0.04761904762	0.05555555556		0.7	#DIV/0!
**KIAA161**	0.1428571429	0.1428571429	0		inf	#DIV/0!
**AA1614-**	0.1428571429	0.1428571429	0		inf	#DIV/0!
**KIAA165**	0.04761904762	0.04761904762	0.1111111111		0.325	#DIV/0!
**KIAA167**	0.04761904762	0.04761904762	0.05555555556		0.7	#DIV/0!
**KIAA175**	0.1428571429	0.1428571429	0.1666666667		0.6666666667	#DIV/0!
**KIAA184**	0.09523809524	0.09523809524	0.05555555556		1.473684211	#DIV/0!
**KIAA195**	0.04761904762	0.04761904762	0.05555555556		0.7	#DIV/0!
**KIAA201**	0.09523809524	0.09523809524	0.05555555556		1.473684211	#DIV/0!
**A2012-**	0	0	0.05555555556		0	#DIV/0!
**KIAA201**	0.04761904762	0.04761904762	0.1111111111		0.325	#DIV/0!
**KIAA202**	0.04761904762	0.04761904762	0.05555555556		0.7	#DIV/0!
**IDINS2**	0	0	0.05555555556		0	#DIV/0!
**KIF11**	0.1428571429	0.1428571429	0.1111111111		1.083333333	#DIV/0!
**KIF12**	0.04761904762	0.04761904762	0.05555555556		0.7	#DIV/0!
**KIF13B**	0.04761904762	0.04761904762	0.1111111111		0.325	#DIV/0!
**KIF14**	0.1428571429	0.1428571429	0		inf	#DIV/0!
**KIF16B**	0.1904761905	0.1904761905	0.1111111111		1.529411765	#DIV/0!
**KIF17**	0.04761904762	0.04761904762	0.1111111111		0.325	#DIV/0!
**KIF18B**	0.1428571429	0.1428571429	0		inf	#DIV/0!
**KIF19**	0.04761904762	0.04761904762	0		inf	#DIV/0!
**KIF1A**	0	0	0.05555555556		0	#DIV/0!
**KIF1B**	0	0	0.1111111111		0	#DIV/0!
**KIF1BP**	0.1904761905	0.1904761905	0.05555555556		3.294117647	#DIV/0!
**KIF1C**	0	0	0.1111111111		0	#DIV/0!
**KIF20A**	0.04761904762	0.04761904762	0		inf	#DIV/0!
**KIF20B**	0.1428571429	0.1428571429	0.1111111111		1.083333333	#DIV/0!
**KIF21A**	0.04761904762	0.04761904762	0.05555555556		0.7	#DIV/0!
**KIF21B**	0.1428571429	0.1428571429	0		inf	#DIV/0!
**KIF22**	0.04761904762	0.04761904762	0		inf	#DIV/0!
**KIF23**	0.04761904762	0.04761904762	0.05555555556		0.7	#DIV/0!
**KIF24**	0.09523809524	0.09523809524	0.05555555556		1.473684211	#DIV/0!
**KIF26B**	0.1428571429	0.1428571429	0.05555555556		2.333333333	#DIV/0!
**F26B-A**	0.1428571429	0.1428571429	0.05555555556		2.333333333	#DIV/0!
**KIF27**	0	0	0.05555555556		0	#DIV/0!
**KIF2A**	0.04761904762	0.04761904762	0		inf	#DIV/0!
**KIF2B**	0.09523809524	0.09523809524	0		inf	#DIV/0!
**KIF2C**	0.04761904762	0.04761904762	0.05555555556		0.7	#DIV/0!
**KIF3A**	0.04761904762	0.04761904762	0		inf	#DIV/0!
**KIF3B**	0.09523809524	0.09523809524	0.1111111111		0.6842105263	#DIV/0!
**KIF4B**	0	0	0.05555555556		0	#DIV/0!
**KIF5A**	0.1428571429	0.1428571429	0.05555555556		2.333333333	#DIV/0!
**KIF5B**	0.09523809524	0.09523809524	0.2222222222		0.2894736842	#DIV/0!
**KIF5C**	0	0	0.05555555556		0	#DIV/0!
**KIF6**	0.04761904762	0.04761904762	0.05555555556		0.7	#DIV/0!
**KIF7**	0.04761904762	0.04761904762	0.05555555556		0.7	#DIV/0!
**KIF9**	0.09523809524	0.09523809524	0		inf	#DIV/0!

KIF9-AS	0.09523809524	0.09523809524	0		inf	#DIV/0!
KIFAP3	0.1428571429	0.1428571429	0.05555555556		2.333333333	#DIV/0!
KIFC1	0.04761904762	0.04761904762	0		inf	#DIV/0!
KIFC2	0.04761904762	0.04761904762	0.1666666667		0.2	#DIV/0!
KIFC3	0.04761904762	0.04761904762	0		inf	#DIV/0!
KIN	0.09523809524	0.09523809524	0.1111111111		0.6842105263	#DIV/0!
KIR2DL1	0.09523809524	0.09523809524	0.05555555556		1.473684211	#DIV/0!
KIR2DL3	0.09523809524	0.09523809524	0.05555555556		1.473684211	#DIV/0!
KIR2DL4	0.09523809524	0.09523809524	0.05555555556		1.473684211	#DIV/0!
KIR2DS4	0.09523809524	0.09523809524	0.05555555556		1.473684211	#DIV/0!
KIR3DL1	0.09523809524	0.09523809524	0.05555555556		1.473684211	#DIV/0!
KIR3DL2	0.09523809524	0.09523809524	0.05555555556		1.473684211	#DIV/0!
KIR3DL3	0.09523809524	0.09523809524	0.05555555556		1.473684211	#DIV/0!
KIR3DX	0.09523809524	0.09523809524	0.05555555556		1.473684211	#DIV/0!
KIRREL	0.1428571429	0.1428571429	0.05555555556		2.333333333	#DIV/0!
RREL1-I	0.1428571429	0.1428571429	0.05555555556		2.333333333	#DIV/0!
KIRREL	0.04761904762	0.04761904762	0.05555555556		0.7	#DIV/0!
KISS1	0.2380952381	0.2380952381	0		inf	#DIV/0!
KISS1R	0.09523809524	0.09523809524	0.1111111111		0.6842105263	#DIV/0!
KIT	0.1904761905	0.1904761905	0.05555555556		3.294117647	#DIV/0!
KITLG	0.04761904762	0.04761904762	0.05555555556		0.7	#DIV/0!
KIZ	0.1904761905	0.1904761905	0.05555555556		3.294117647	#DIV/0!
KIZ-AS1	0.1904761905	0.1904761905	0.05555555556		3.294117647	#DIV/0!
KLB	0.1904761905	0.1904761905	0.05555555556		3.294117647	#DIV/0!
KLC3	0.09523809524	0.09523809524	0.1111111111		0.6842105263	#DIV/0!
KLC4	0.04761904762	0.04761904762	0.05555555556		0.7	#DIV/0!
KLF1	0.1428571429	0.1428571429	0.1111111111		1.083333333	#DIV/0!
KLF10	0.1904761905	0.1904761905	0.1111111111		1.529411765	#DIV/0!
KLF11	0	0	0.05555555556		0	#DIV/0!
KLF14	0.04761904762	0.04761904762	0.05555555556		0.7	#DIV/0!
KLF15	0.04761904762	0.04761904762	0.05555555556		0.7	#DIV/0!
KLF16	0.09523809524	0.09523809524	0.1111111111		0.6842105263	#DIV/0!
KLF17	0.04761904762	0.04761904762	0.05555555556		0.7	#DIV/0!
KLF18	0.04761904762	0.04761904762	0.05555555556		0.7	#DIV/0!
KLF2	0.1428571429	0.1428571429	0.1111111111		1.083333333	#DIV/0!
KLF3	0.04761904762	0.04761904762	0		inf	#DIV/0!
KLF3-AS	0.04761904762	0.04761904762	0		inf	#DIV/0!
KLF4	0.04761904762	0.04761904762	0.05555555556		0.7	#DIV/0!
KLF6	0.04761904762	0.04761904762	0.1666666667		0.2	#DIV/0!
KLF7	0	0	0.05555555556		0	#DIV/0!
KLF9	0	0	0.05555555556		0	#DIV/0!
KLHDC1	0.04761904762	0.04761904762	0		inf	#DIV/0!
KLHDC1	0.04761904762	0.04761904762	0.05555555556		0.7	#DIV/0!
KLHDC2	0.04761904762	0.04761904762	0		inf	#DIV/0!
KLHDC3	0.04761904762	0.04761904762	0.05555555556		0.7	#DIV/0!
KLHDC4	0.04761904762	0.04761904762	0		inf	#DIV/0!
LHDC7	0.04761904762	0.04761904762	0.05555555556		0.7	#DIV/0!
LHDC7	0	0	0.1111111111		0	#DIV/0!
LHDC8	0.1904761905	0.1904761905	0		inf	#DIV/0!
LHDC8	0.09523809524	0.09523809524	0		inf	#DIV/0!
KLHDC9	0.1428571429	0.1428571429	0		inf	#DIV/0!

KLHL10	0.09523809524	0.09523809524	0		inf	#DIV/0!
KLHL11	0.09523809524	0.09523809524	0		inf	#DIV/0!
KLHL12	0.1428571429	0.1428571429	0		inf	#DIV/0!
KLHL14	0.09523809524	0.09523809524	0		inf	#DIV/0!
KLHL17	0	0	0.1111111111		0	#DIV/0!
KLHL18	0.09523809524	0.09523809524	0		inf	#DIV/0!
KLHL2	0	0	0.1111111111		0	#DIV/0!
KLHL20	0.1428571429	0.1428571429	0		inf	#DIV/0!
KLHL21	0	0	0.05555555556		0	#DIV/0!
KLHL22	0.04761904762	0.04761904762	0.05555555556		0.7	#DIV/0!
KLHL23	0	0	0.05555555556		0	#DIV/0!
KLHL24	0.1904761905	0.1904761905	0.05555555556		3.294117647	#DIV/0!
KLHL25	0.09523809524	0.09523809524	0.05555555556		1.473684211	#DIV/0!
KLHL26	0.1428571429	0.1428571429	0.1111111111		1.083333333	#DIV/0!
KLHL28	0.04761904762	0.04761904762	0		inf	#DIV/0!
KLHL29	0	0	0.05555555556		0	#DIV/0!
KLHL3	0	0	0			#DIV/0!
KLHL30	0	0	0.05555555556		0	#DIV/0!
KLHL31	0.04761904762	0.04761904762	0		inf	#DIV/0!
KLHL33	0.04761904762	0.04761904762	0		inf	#DIV/0!
KLHL36	0.04761904762	0.04761904762	0		inf	#DIV/0!
KLHL38	0.1428571429	0.1428571429	0.1111111111		1.083333333	#DIV/0!
KLHL40	0.04761904762	0.04761904762	0		inf	#DIV/0!
KLHL41	0	0	0.05555555556		0	#DIV/0!
KLHL42	0.04761904762	0.04761904762	0.05555555556		0.7	#DIV/0!
KLHL5	0.1904761905	0.1904761905	0.05555555556		3.294117647	#DIV/0!
KLHL6	0.1904761905	0.1904761905	0.05555555556		3.294117647	#DIV/0!
LHL6-A	0.1904761905	0.1904761905	0.05555555556		3.294117647	#DIV/0!
KLHL7	0.04761904762	0.04761904762	0.1666666667		0.2	#DIV/0!
LHL7-D	0.04761904762	0.04761904762	0.1666666667		0.2	#DIV/0!
KLHL8	0	0	0.05555555556		0	#DIV/0!
KLHL9	0.09523809524	0.09523809524	0.1111111111		0.6842105263	#DIV/0!
KLK1	0.09523809524	0.09523809524	0.05555555556		1.473684211	#DIV/0!
KLK10	0.09523809524	0.09523809524	0.05555555556		1.473684211	#DIV/0!
KLK11	0.09523809524	0.09523809524	0.05555555556		1.473684211	#DIV/0!
KLK12	0.09523809524	0.09523809524	0.05555555556		1.473684211	#DIV/0!
KLK13	0.09523809524	0.09523809524	0.05555555556		1.473684211	#DIV/0!
KLK14	0.09523809524	0.09523809524	0.05555555556		1.473684211	#DIV/0!
KLK15	0.09523809524	0.09523809524	0.05555555556		1.473684211	#DIV/0!
KLK2	0.09523809524	0.09523809524	0.05555555556		1.473684211	#DIV/0!
KLK3	0.09523809524	0.09523809524	0.05555555556		1.473684211	#DIV/0!
KLK4	0.09523809524	0.09523809524	0.05555555556		1.473684211	#DIV/0!
KLK5	0.09523809524	0.09523809524	0.05555555556		1.473684211	#DIV/0!
KLK6	0.09523809524	0.09523809524	0.05555555556		1.473684211	#DIV/0!
KLK7	0.09523809524	0.09523809524	0.05555555556		1.473684211	#DIV/0!
KLK8	0.09523809524	0.09523809524	0.05555555556		1.473684211	#DIV/0!
KLK9	0.09523809524	0.09523809524	0.05555555556		1.473684211	#DIV/0!
KLKB1	0	0	0.05555555556		0	#DIV/0!
KLKP1	0.09523809524	0.09523809524	0.05555555556		1.473684211	#DIV/0!
KLLN	0.1428571429	0.1428571429	0.1666666667		0.6666666667	#DIV/0!
KLRA1P	0.04761904762	0.04761904762	0.1111111111		0.325	#DIV/0!

**KLRB1**	0.04761904762	0.04761904762	0.1111111111		0.325	#DIV/0!
**KLRC1**	0.04761904762	0.04761904762	0.1111111111		0.325	#DIV/0!
**KLRC2**	0.04761904762	0.04761904762	0.1111111111		0.325	#DIV/0!
**KLRC3**	0.04761904762	0.04761904762	0.1111111111		0.325	#DIV/0!
**KLRC4**	0.04761904762	0.04761904762	0.1111111111		0.325	#DIV/0!
**RC4-KLI**	0.04761904762	0.04761904762	0.1111111111		0.325	#DIV/0!
**KLRD1**	0.04761904762	0.04761904762	0.1111111111		0.325	#DIV/0!
**KLRF1**	0.04761904762	0.04761904762	0.1111111111		0.325	#DIV/0!
**KLRF2**	0.04761904762	0.04761904762	0.1111111111		0.325	#DIV/0!
**KLRG1**	0.04761904762	0.04761904762	0.1111111111		0.325	#DIV/0!
**KLRG2**	0.04761904762	0.04761904762	0.05555555556		0.7	#DIV/0!
**KLRK1**	0.04761904762	0.04761904762	0.1111111111		0.325	#DIV/0!
**KMO**	0.1428571429	0.1428571429	0		inf	#DIV/0!
**KMT2B**	0.04761904762	0.04761904762	0.05555555556		0.7	#DIV/0!
**KMT2C**	0.09523809524	0.09523809524	0.05555555556		1.473684211	#DIV/0!
**KMT2D**	0.04761904762	0.04761904762	0.05555555556		0.7	#DIV/0!
**KMT2E**	0.04761904762	0.04761904762	0.05555555556		0.7	#DIV/0!
**MT2E-A**	0.04761904762	0.04761904762	0.05555555556		0.7	#DIV/0!
**KMT5A**	0.04761904762	0.04761904762	0.1111111111		0.325	#DIV/0!
**KMT5C**	0.09523809524	0.09523809524	0.05555555556		1.473684211	#DIV/0!
**KNCN**	0.04761904762	0.04761904762	0.05555555556		0.7	#DIV/0!
**KNDC1**	0.1904761905	0.1904761905	0		inf	#DIV/0!
**KNG1**	0.09523809524	0.09523809524	0.05555555556		1.473684211	#DIV/0!
**KNOP1**	0.04761904762	0.04761904762	0		inf	#DIV/0!
**KNTC1**	0.04761904762	0.04761904762	0.1111111111		0.325	#DIV/0!
**KPNA1**	0.09523809524	0.09523809524	0.05555555556		1.473684211	#DIV/0!
**KPNA2**	0	0	0			#DIV/0!
**KPNA4**	0.1428571429	0.1428571429	0.05555555556		2.333333333	#DIV/0!
**KPNA6**	0.04761904762	0.04761904762	0.1111111111		0.325	#DIV/0!
**KPNA7**	0.04761904762	0.04761904762	0.05555555556		0.7	#DIV/0!
**KPNB1**	0.1428571429	0.1428571429	0		inf	#DIV/0!
**KPRP**	0.1904761905	0.1904761905	0		inf	#DIV/0!
**KPTN**	0.09523809524	0.09523809524	0.1111111111		0.6842105263	#DIV/0!
**KRAS**	0.04761904762	0.04761904762	0.05555555556		0.7	#DIV/0!
**KRBA1**	0.09523809524	0.09523809524	0.05555555556		1.473684211	#DIV/0!
**KRBA2**	0.04761904762	0.04761904762	0.05555555556		0.7	#DIV/0!
**KRBOX1**	0.04761904762	0.04761904762	0		inf	#DIV/0!
**BOX1-A**	0.04761904762	0.04761904762	0		inf	#DIV/0!
**KRCC1**	0	0	0.05555555556		0	#DIV/0!
**REMEN**	0.04761904762	0.04761904762	0.1111111111		0.325	#DIV/0!
**REMEN**	0.04761904762	0.04761904762	0.1111111111		0.325	#DIV/0!
**KRI1**	0.1428571429	0.1428571429	0.1111111111		1.083333333	#DIV/0!
**KRIT1**	0.04761904762	0.04761904762	0.1111111111		0.325	#DIV/0!
**KRT1**	0.04761904762	0.04761904762	0.05555555556		0.7	#DIV/0!
**KRT10**	0.09523809524	0.09523809524	0		inf	#DIV/0!
**KRT12**	0.09523809524	0.09523809524	0		inf	#DIV/0!
**KRT13**	0.09523809524	0.09523809524	0		inf	#DIV/0!
**KRT14**	0.09523809524	0.09523809524	0		inf	#DIV/0!
**KRT15**	0.09523809524	0.09523809524	0		inf	#DIV/0!
**KRT16**	0.09523809524	0.09523809524	0		inf	#DIV/0!
**KRT16P**	0.04761904762	0.04761904762	0.1111111111		0.325	#DIV/0!

KRT16P	0.04761904762	0.04761904762	0.05555555556		0.7	#DIV/0!
KRT16P	0.09523809524	0.09523809524	0.05555555556		1.473684211	#DIV/0!
KRT17	0.09523809524	0.09523809524	0		inf	#DIV/0!
KRT17P	0.04761904762	0.04761904762	0.05555555556		0.7	#DIV/0!
KRT17P	0.04761904762	0.04761904762	0.1111111111		0.325	#DIV/0!
KRT17P	0.04761904762	0.04761904762	0.1111111111		0.325	#DIV/0!
KRT18	0.04761904762	0.04761904762	0.05555555556		0.7	#DIV/0!
RT18P5	0.04761904762	0.04761904762	0.05555555556		0.7	#DIV/0!
KRT19	0.09523809524	0.09523809524	0		inf	#DIV/0!
KRT19P	0.04761904762	0.04761904762	0.05555555556		0.7	#DIV/0!
KRT2	0.04761904762	0.04761904762	0.05555555556		0.7	#DIV/0!
KRT20	0.09523809524	0.09523809524	0		inf	#DIV/0!
KRT222	0.09523809524	0.09523809524	0		inf	#DIV/0!
KRT23	0.09523809524	0.09523809524	0		inf	#DIV/0!
KRT24	0.09523809524	0.09523809524	0		inf	#DIV/0!
KRT25	0.09523809524	0.09523809524	0		inf	#DIV/0!
KRT26	0.09523809524	0.09523809524	0		inf	#DIV/0!
KRT27	0.09523809524	0.09523809524	0		inf	#DIV/0!
KRT28	0.09523809524	0.09523809524	0		inf	#DIV/0!
KRT3	0.04761904762	0.04761904762	0.05555555556		0.7	#DIV/0!
KRT31	0.09523809524	0.09523809524	0		inf	#DIV/0!
KRT32	0.09523809524	0.09523809524	0		inf	#DIV/0!
KRT33A	0.09523809524	0.09523809524	0		inf	#DIV/0!
KRT33B	0.09523809524	0.09523809524	0		inf	#DIV/0!
KRT34	0.09523809524	0.09523809524	0		inf	#DIV/0!
KRT35	0.09523809524	0.09523809524	0		inf	#DIV/0!
KRT36	0.09523809524	0.09523809524	0		inf	#DIV/0!
KRT37	0.09523809524	0.09523809524	0		inf	#DIV/0!
KRT38	0.09523809524	0.09523809524	0		inf	#DIV/0!
KRT39	0.09523809524	0.09523809524	0		inf	#DIV/0!
KRT4	0.04761904762	0.04761904762	0.05555555556		0.7	#DIV/0!
KRT40	0.09523809524	0.09523809524	0		inf	#DIV/0!
KRT42P	0.09523809524	0.09523809524	0		inf	#DIV/0!
KRT5	0.04761904762	0.04761904762	0.05555555556		0.7	#DIV/0!
KRT6A	0.04761904762	0.04761904762	0.05555555556		0.7	#DIV/0!
KRT6B	0.04761904762	0.04761904762	0.05555555556		0.7	#DIV/0!
KRT6C	0.04761904762	0.04761904762	0.05555555556		0.7	#DIV/0!
KRT7	0.04761904762	0.04761904762	0.05555555556		0.7	#DIV/0!
KRT7-AS	0.04761904762	0.04761904762	0.05555555556		0.7	#DIV/0!
KRT71	0.04761904762	0.04761904762	0.05555555556		0.7	#DIV/0!
KRT72	0.04761904762	0.04761904762	0.05555555556		0.7	#DIV/0!
KRT73	0.04761904762	0.04761904762	0.05555555556		0.7	#DIV/0!
RT73-AS	0.04761904762	0.04761904762	0.05555555556		0.7	#DIV/0!
KRT74	0.04761904762	0.04761904762	0.05555555556		0.7	#DIV/0!
KRT75	0.04761904762	0.04761904762	0.05555555556		0.7	#DIV/0!
KRT76	0.04761904762	0.04761904762	0.05555555556		0.7	#DIV/0!
KRT77	0.04761904762	0.04761904762	0.05555555556		0.7	#DIV/0!
KRT78	0.04761904762	0.04761904762	0.05555555556		0.7	#DIV/0!
KRT79	0.04761904762	0.04761904762	0.05555555556		0.7	#DIV/0!
KRT8	0.04761904762	0.04761904762	0.05555555556		0.7	#DIV/0!
KRT80	0.04761904762	0.04761904762	0.05555555556		0.7	#DIV/0!

KRT81	0.04761904762	0.04761904762	0.05555555556		0.7	#DIV/0!
KRT82	0.04761904762	0.04761904762	0.05555555556		0.7	#DIV/0!
KRT83	0.04761904762	0.04761904762	0.05555555556		0.7	#DIV/0!
KRT84	0.04761904762	0.04761904762	0.05555555556		0.7	#DIV/0!
KRT85	0.04761904762	0.04761904762	0.05555555556		0.7	#DIV/0!
KRT86	0.04761904762	0.04761904762	0.05555555556		0.7	#DIV/0!
KRT87P	0.04761904762	0.04761904762	0.05555555556		0.7	#DIV/0!
KRT9	0.09523809524	0.09523809524	0		inf	#DIV/0!
RTAP1-	0.09523809524	0.09523809524	0		inf	#DIV/0!
RTAP1-	0.09523809524	0.09523809524	0		inf	#DIV/0!
RTAP1-	0.09523809524	0.09523809524	0		inf	#DIV/0!
RTAP1-	0.09523809524	0.09523809524	0		inf	#DIV/0!
RTAP10	0.04761904762	0.04761904762	0.1111111111		0.325	#DIV/0!
RTAP10-	0.04761904762	0.04761904762	0.1111111111		0.325	#DIV/0!
RTAP10-	0.04761904762	0.04761904762	0.1111111111		0.325	#DIV/0!
RTAP10-	0.04761904762	0.04761904762	0.1111111111		0.325	#DIV/0!
RTAP10	0.04761904762	0.04761904762	0.1111111111		0.325	#DIV/0!
RTAP10	0.04761904762	0.04761904762	0.1111111111		0.325	#DIV/0!
RTAP10	0.04761904762	0.04761904762	0.1111111111		0.325	#DIV/0!
RTAP10	0.04761904762	0.04761904762	0.1111111111		0.325	#DIV/0!
RTAP10	0.04761904762	0.04761904762	0.1111111111		0.325	#DIV/0!
RTAP10	0.04761904762	0.04761904762	0.1111111111		0.325	#DIV/0!
RTAP10	0.04761904762	0.04761904762	0.1111111111		0.325	#DIV/0!
RTAP10	0.04761904762	0.04761904762	0.1111111111		0.325	#DIV/0!
RTAP11	0.04761904762	0.04761904762	0.05555555556		0.7	#DIV/0!
RTAP12	0.04761904762	0.04761904762	0.1111111111		0.325	#DIV/0!
RTAP12	0.04761904762	0.04761904762	0.1111111111		0.325	#DIV/0!
RTAP12	0.04761904762	0.04761904762	0.1111111111		0.325	#DIV/0!
RTAP12	0.04761904762	0.04761904762	0.1111111111		0.325	#DIV/0!
RTAP13	0.04761904762	0.04761904762	0.05555555556		0.7	#DIV/0!
RTAP13	0.04761904762	0.04761904762	0.05555555556		0.7	#DIV/0!
RTAP13	0.04761904762	0.04761904762	0.05555555556		0.7	#DIV/0!
RTAP13	0.04761904762	0.04761904762	0.05555555556		0.7	#DIV/0!
RTAP15	0.04761904762	0.04761904762	0.05555555556		0.7	#DIV/0!
RTAP16	0.09523809524	0.09523809524	0		inf	#DIV/0!
RTAP17	0.09523809524	0.09523809524	0		inf	#DIV/0!
RTAP19	0.04761904762	0.04761904762	0.05555555556		0.7	#DIV/0!
RTAP19	0.04761904762	0.04761904762	0.05555555556		0.7	#DIV/0!
RTAP19	0.04761904762	0.04761904762	0.05555555556		0.7	#DIV/0!
RTAP19	0.04761904762	0.04761904762	0.05555555556		0.7	#DIV/0!
RTAP19	0.04761904762	0.04761904762	0.05555555556		0.7	#DIV/0!
RTAP19	0.04761904762	0.04761904762	0.05555555556		0.7	#DIV/0!
RTAP19	0.04761904762	0.04761904762	0.05555555556		0.7	#DIV/0!
RTAP19	0.04761904762	0.04761904762	0.05555555556		0.7	#DIV/0!
RTAP2-	0.09523809524	0.09523809524	0		inf	#DIV/0!
RTAP2-	0.09523809524	0.09523809524	0		inf	#DIV/0!
RTAP2-	0.09523809524	0.09523809524	0		inf	#DIV/0!
RTAP2-	0.09523809524	0.09523809524	0		inf	#DIV/0!
RTAP20	0.04761904762	0.04761904762	0.05555555556		0.7	#DIV/0!
RTAP20	0.04761904762	0.04761904762	0.05555555556		0.7	#DIV/0!
RTAP20	0.04761904762	0.04761904762	0.05555555556		0.7	#DIV/0!

**RTAP20**	0.04761904762	0.04761904762	0.05555555556		0.7	#DIV/0!
**RTAP21**	0.04761904762	0.04761904762	0.05555555556		0.7	#DIV/0!
**RTAP21**	0.04761904762	0.04761904762	0.05555555556		0.7	#DIV/0!
**RTAP21**	0.04761904762	0.04761904762	0.05555555556		0.7	#DIV/0!
**RTAP22**	0.04761904762	0.04761904762	0.05555555556		0.7	#DIV/0!
**RTAP22**	0.04761904762	0.04761904762	0.05555555556		0.7	#DIV/0!
**RTAP23**	0.04761904762	0.04761904762	0.05555555556		0.7	#DIV/0!
**RTAP24**	0.04761904762	0.04761904762	0.05555555556		0.7	#DIV/0!
**RTAP25**	0.04761904762	0.04761904762	0.05555555556		0.7	#DIV/0!
**RTAP26**	0.04761904762	0.04761904762	0.05555555556		0.7	#DIV/0!
**RTAP27**	0.04761904762	0.04761904762	0.05555555556		0.7	#DIV/0!
**RTAP29**	0.09523809524	0.09523809524	0		inf	#DIV/0!
**RTAP3-**	0.09523809524	0.09523809524	0		inf	#DIV/0!
**RTAP3-**	0.09523809524	0.09523809524	0		inf	#DIV/0!
**RTAP3-**	0.09523809524	0.09523809524	0		inf	#DIV/0!
**RTAP4-**	0.09523809524	0.09523809524	0		inf	#DIV/0!
**RTAP4-**	0.09523809524	0.09523809524	0		inf	#DIV/0!
**RTAP4-**	0.09523809524	0.09523809524	0		inf	#DIV/0!
**RTAP4-**	0.09523809524	0.09523809524	0		inf	#DIV/0!
**RTAP4-**	0.09523809524	0.09523809524	0		inf	#DIV/0!
**RTAP4-**	0.09523809524	0.09523809524	0		inf	#DIV/0!
**RTAP4-**	0.09523809524	0.09523809524	0		inf	#DIV/0!
**RTAP4-**	0.09523809524	0.09523809524	0		inf	#DIV/0!
**RTAP4-**	0.09523809524	0.09523809524	0		inf	#DIV/0!
**RTAP4-**	0.09523809524	0.09523809524	0		inf	#DIV/0!
**RTAP4-**	0.09523809524	0.09523809524	0		inf	#DIV/0!
**RTAP5-**	0.04761904762	0.04761904762	0		inf	#DIV/0!
**RTAP5-**	0.04761904762	0.04761904762	0		inf	#DIV/0!
**RTAP5-**	0.04761904762	0.04761904762	0		inf	#DIV/0!
**RTAP5-**	0.04761904762	0.04761904762	0		inf	#DIV/0!
**RTAP5-**	0.04761904762	0.04761904762	0		inf	#DIV/0!
**RTAP5-**	0.04761904762	0.04761904762	0		inf	#DIV/0!
**TAP5-A**	0.04761904762	0.04761904762	0		inf	#DIV/0!
**RTAP6-**	0.04761904762	0.04761904762	0.05555555556		0.7	#DIV/0!
**RTAP6-**	0.04761904762	0.04761904762	0.05555555556		0.7	#DIV/0!
**RTAP6-**	0.04761904762	0.04761904762	0.05555555556		0.7	#DIV/0!
**RTAP7-**	0.04761904762	0.04761904762	0.05555555556		0.7	#DIV/0!
**RTAP8-**	0.04761904762	0.04761904762	0.05555555556		0.7	#DIV/0!
**RTAP9-**	0.09523809524	0.09523809524	0		inf	#DIV/0!
**RTAP9-**	0.09523809524	0.09523809524	0		inf	#DIV/0!
**RTAP9-**	0.09523809524	0.09523809524	0		inf	#DIV/0!
**RTAP9-**	0.09523809524	0.09523809524	0		inf	#DIV/0!
**RTAP9-**	0.09523809524	0.09523809524	0		inf	#DIV/0!
**RTAP9-**	0.09523809524	0.09523809524	0		inf	#DIV/0!
**RTAP9-**	0.09523809524	0.09523809524	0		inf	#DIV/0!
**RTAP9-**	0.09523809524	0.09523809524	0		inf	#DIV/0!
**RTCAP**	0.1904761905	0.1904761905	0.1111111111		1.529411765	#DIV/0!
**KRTDAI**	0.04761904762	0.04761904762	0.05555555556		0.7	#DIV/0!
**KSR1**	0.04761904762	0.04761904762	0.05555555556		0.7	#DIV/0!
**KSR2**	0	0	0.05555555556		0	#DIV/0!
**KTI12**	0.04761904762	0.04761904762	0.05555555556		0.7	#DIV/0!

KTN1	0.04761904762	0.04761904762	0		inf	#DIV/0!
TN1-AS	0.04761904762	0.04761904762	0		inf	#DIV/0!
KXD1	0.1428571429	0.1428571429	0.1111111111		1.083333333	#DIV/0!
KY	0.1428571429	0.1428571429	0.05555555556		2.333333333	#DIV/0!
KYAT1	0.09523809524	0.09523809524	0.05555555556		1.473684211	#DIV/0!
KYAT3	0.04761904762	0.04761904762	0.05555555556		0.7	#DIV/0!
KYNU	0	0	0.05555555556		0	#DIV/0!
L1TD1	0.04761904762	0.04761904762	0.05555555556		0.7	#DIV/0!
2HGDH	0.04761904762	0.04761904762	0		inf	#DIV/0!
3MBTL	0.1428571429	0.1428571429	0.1111111111		1.083333333	#DIV/0!
3MBTL	0	0	0.1111111111		0	#DIV/0!
3MBTL	0.09523809524	0.09523809524	0		inf	#DIV/0!
IBTL4-	0.09523809524	0.09523809524	0		inf	#DIV/0!
LACTB	0.04761904762	0.04761904762	0.05555555556		0.7	#DIV/0!
LACTB2	0.1904761905	0.1904761905	0.1666666667		0.9411764706	#DIV/0!
CTB2-A	0.1904761905	0.1904761905	0.1666666667		0.9411764706	#DIV/0!
ACTBL	0.04761904762	0.04761904762	0.1111111111		0.325	#DIV/0!
LAD1	0.1428571429	0.1428571429	0		inf	#DIV/0!
LAG3	0.04761904762	0.04761904762	0.1111111111		0.325	#DIV/0!
LAIR1	0.09523809524	0.09523809524	0.05555555556		1.473684211	#DIV/0!
LAIR2	0.09523809524	0.09523809524	0.05555555556		1.473684211	#DIV/0!
LALBA	0.04761904762	0.04761904762	0.05555555556		0.7	#DIV/0!
LAMA1	0.09523809524	0.09523809524	0		inf	#DIV/0!
LAMA3	0.09523809524	0.09523809524	0.05555555556		1.473684211	#DIV/0!
LAMA5	0.1428571429	0.1428571429	0.2222222222		0.4583333333	#DIV/0!
MA5-A	0.1428571429	0.1428571429	0.2222222222		0.4583333333	#DIV/0!
LAMB1	0.04761904762	0.04761904762	0.05555555556		0.7	#DIV/0!
LAMB2	0.09523809524	0.09523809524	0		inf	#DIV/0!
AMB2P	0.09523809524	0.09523809524	0		inf	#DIV/0!
LAMB3	0.1428571429	0.1428571429	0		inf	#DIV/0!
LAMB4	0.04761904762	0.04761904762	0.05555555556		0.7	#DIV/0!
LAMC1	0.1428571429	0.1428571429	0		inf	#DIV/0!
MC1-A	0.1428571429	0.1428571429	0		inf	#DIV/0!
LAMC2	0.1428571429	0.1428571429	0		inf	#DIV/0!
LAMC3	0.09523809524	0.09523809524	0.05555555556		1.473684211	#DIV/0!
LAMP3	0.1904761905	0.1904761905	0.05555555556		3.294117647	#DIV/0!
LAMP5	0.1904761905	0.1904761905	0.1111111111		1.529411765	#DIV/0!
MP5-A	0.1904761905	0.1904761905	0.1111111111		1.529411765	#DIV/0!
AMTOR	0.1428571429	0.1428571429	0.1111111111		1.083333333	#DIV/0!
AMTOR	0	0	0.05555555556		0	#DIV/0!
AMTOR	0.04761904762	0.04761904762	0.05555555556		0.7	#DIV/0!
AMTOR	0.04761904762	0.04761904762	0		inf	#DIV/0!
ITOR5-	0.04761904762	0.04761904762	0		inf	#DIV/0!
ANCL1	0	0	0.05555555556		0	#DIV/0!
NCL1-A	0	0	0.05555555556		0	#DIV/0!
ANCL2	0.380952381	0.380952381	0.2222222222		1.692307692	#DIV/0!
LAP3	0	0	0			#DIV/0!
APTM4	0	0	0.05555555556		0	#DIV/0!
APTM4	0.1904761905	0.1904761905	0.1111111111		1.529411765	#DIV/0!
APTM5	0.04761904762	0.04761904762	0.1111111111		0.325	#DIV/0!
RGE-A	0.04761904762	0.04761904762	0.1666666667		0.2	#DIV/0!

LARGE1	0.04761904762	0.04761904762	0.1666666667		0.2	#DIV/0!
LARP1	0	0	0.05555555556		0	#DIV/0!
LARP1B	0	0	0.05555555556		0	#DIV/0!
LARP4	0.09523809524	0.09523809524	0.05555555556		1.473684211	#DIV/0!
LARP4B	0.04761904762	0.04761904762	0.1111111111		0.325	#DIV/0!
LARP6	0.04761904762	0.04761904762	0.05555555556		0.7	#DIV/0!
LARP7	0	0	0.05555555556		0	#DIV/0!
LASP1	0.09523809524	0.09523809524	0		inf	#DIV/0!
LAT	0.04761904762	0.04761904762	0		inf	#DIV/0!
LAT2	0.1428571429	0.1428571429	0.1111111111		1.083333333	#DIV/0!
LATS1	0	0	0			#DIV/0!
LATS2	0.04761904762	0.04761904762	0		inf	#DIV/0!
LAX1	0.2380952381	0.2380952381	0		inf	#DIV/0!
LBH	0	0	0.05555555556		0	#DIV/0!
LBP	0.1428571429	0.1428571429	0.1666666667		0.6666666667	#DIV/0!
LBR	0.1428571429	0.1428571429	0.05555555556		2.333333333	#DIV/0!
LBX1	0.1428571429	0.1428571429	0		inf	#DIV/0!
LBX1-AS	0.1428571429	0.1428571429	0		inf	#DIV/0!
LBX2	0	0	0.05555555556		0	#DIV/0!
LBX2-AS	0	0	0.05555555556		0	#DIV/0!
LCA5L	0.04761904762	0.04761904762	0.05555555556		0.7	#DIV/0!
LCAT	0.1428571429	0.1428571429	0.05555555556		2.333333333	#DIV/0!
LCE1A	0.1904761905	0.1904761905	0		inf	#DIV/0!
LCE1B	0.1904761905	0.1904761905	0		inf	#DIV/0!
LCE1C	0.1904761905	0.1904761905	0		inf	#DIV/0!
LCE1D	0.1904761905	0.1904761905	0		inf	#DIV/0!
LCE1E	0.1904761905	0.1904761905	0		inf	#DIV/0!
LCE1F	0.1904761905	0.1904761905	0		inf	#DIV/0!
LCE2A	0.1904761905	0.1904761905	0		inf	#DIV/0!
LCE2B	0.1904761905	0.1904761905	0		inf	#DIV/0!
LCE2C	0.1904761905	0.1904761905	0		inf	#DIV/0!
LCE2D	0.1904761905	0.1904761905	0		inf	#DIV/0!
LCE3A	0.1904761905	0.1904761905	0		inf	#DIV/0!
LCE3B	0.1904761905	0.1904761905	0		inf	#DIV/0!
LCE3C	0.1904761905	0.1904761905	0		inf	#DIV/0!
LCE3D	0.1904761905	0.1904761905	0		inf	#DIV/0!
LCE3E	0.1904761905	0.1904761905	0		inf	#DIV/0!
LCE4A	0.1904761905	0.1904761905	0		inf	#DIV/0!
LCE5A	0.1904761905	0.1904761905	0		inf	#DIV/0!
LCE6A	0.1904761905	0.1904761905	0		inf	#DIV/0!
LCK	0.04761904762	0.04761904762	0.1111111111		0.325	#DIV/0!
LCLAT1	0	0	0.05555555556		0	#DIV/0!
LCMT1	0.04761904762	0.04761904762	0		inf	#DIV/0!
CMT1-A	0.04761904762	0.04761904762	0		inf	#DIV/0!
CMT1-A	0.04761904762	0.04761904762	0		inf	#DIV/0!
LCN1	0.04761904762	0.04761904762	0		inf	#DIV/0!
LCN10	0.1428571429	0.1428571429	0.05555555556		2.333333333	#DIV/0!
LCN12	0.1428571429	0.1428571429	0.05555555556		2.333333333	#DIV/0!
LCN15	0.1428571429	0.1428571429	0.05555555556		2.333333333	#DIV/0!
LCN2	0.09523809524	0.09523809524	0.05555555556		1.473684211	#DIV/0!
LCN6	0.1428571429	0.1428571429	0.05555555556		2.333333333	#DIV/0!

**LCN8**	0.1428571429	0.1428571429	0.05555555556		2.333333333	#DIV/0!
**LCNL1**	0.1428571429	0.1428571429	0.05555555556		2.333333333	#DIV/0!
**LCOR**	0.1428571429	0.1428571429	0		inf	#DIV/0!
**LCORL**	0	0	0			#DIV/0!
**LCP2**	0	0	0.05555555556		0	#DIV/0!
**LCT**	0.04761904762	0.04761904762	0.05555555556		0.7	#DIV/0!
**LCTL**	0.04761904762	0.04761904762	0.05555555556		0.7	#DIV/0!
**LDAH**	0	0	0.05555555556		0	#DIV/0!
**LDB1**	0.1428571429	0.1428571429	0.05555555556		2.333333333	#DIV/0!
**LDB2**	0	0	0			#DIV/0!
**LDB3**	0.1428571429	0.1428571429	0.05555555556		2.333333333	#DIV/0!
**DHAL6**	0.04761904762	0.04761904762	0.05555555556		0.7	#DIV/0!
**LDHB**	0.04761904762	0.04761904762	0.05555555556		0.7	#DIV/0!
**LDHD**	0.04761904762	0.04761904762	0		inf	#DIV/0!
**LDLR**	0.1428571429	0.1428571429	0.1111111111		1.083333333	#DIV/0!
**DLRAD**	0.04761904762	0.04761904762	0.05555555556		0.7	#DIV/0!
**DLRAD**	0.04761904762	0.04761904762	0.1111111111		0.325	#DIV/0!
**DLRAD**	0.09523809524	0.09523809524	0.05555555556		1.473684211	#DIV/0!
**LRAD4-**	0.09523809524	0.09523809524	0.05555555556		1.473684211	#DIV/0!
**DLRAP**	0.04761904762	0.04761904762	0.1111111111		0.325	#DIV/0!
**LEAP2**	0.04761904762	0.04761904762	0		inf	#DIV/0!
**LECT2**	0.04761904762	0.04761904762	0		inf	#DIV/0!
**LEF1**	0	0	0.05555555556		0	#DIV/0!
**EF1-AS**	0	0	0.05555555556		0	#DIV/0!
**LEFTY1**	0.1428571429	0.1428571429	0.05555555556		2.333333333	#DIV/0!
**LEFTY2**	0.1428571429	0.1428571429	0.05555555556		2.333333333	#DIV/0!
**LEKR1**	0.1428571429	0.1428571429	0.05555555556		2.333333333	#DIV/0!
**LELP1**	0.1904761905	0.1904761905	0		inf	#DIV/0!
**LEMD1**	0.1904761905	0.1904761905	0		inf	#DIV/0!
**MD1-A**	0.1904761905	0.1904761905	0		inf	#DIV/0!
**LEMD2**	0.04761904762	0.04761904762	0		inf	#DIV/0!
**LEMD3**	0.09523809524	0.09523809524	0.05555555556		1.473684211	#DIV/0!
**LENEP**	0.1904761905	0.1904761905	0.1111111111		1.529411765	#DIV/0!
**LENG1**	0.09523809524	0.09523809524	0.05555555556		1.473684211	#DIV/0!
**LENG8**	0.09523809524	0.09523809524	0.05555555556		1.473684211	#DIV/0!
**NG8-A**	0.09523809524	0.09523809524	0.05555555556		1.473684211	#DIV/0!
**LENG9**	0.09523809524	0.09523809524	0.05555555556		1.473684211	#DIV/0!
**LEO1**	0.04761904762	0.04761904762	0.05555555556		0.7	#DIV/0!
**LEP**	0.04761904762	0.04761904762	0.05555555556		0.7	#DIV/0!
**LEPR**	0.04761904762	0.04761904762	0.05555555556		0.7	#DIV/0!
**LEPROT**	0.04761904762	0.04761904762	0.05555555556		0.7	#DIV/0!
**EPROTI**	0.04761904762	0.04761904762	0.1111111111		0.325	#DIV/0!
**LETM1**	0.04761904762	0.04761904762	0		inf	#DIV/0!
**LETM2**	0.09523809524	0.09523809524	0.1111111111		0.6842105263	#DIV/0!
**LETMD1**	0.09523809524	0.09523809524	0.05555555556		1.473684211	#DIV/0!
**LEUTX**	0.04761904762	0.04761904762	0.05555555556		0.7	#DIV/0!
**LEXM**	0.04761904762	0.04761904762	0.05555555556		0.7	#DIV/0!
**LFNG**	0.04761904762	0.04761904762	0.1666666667		0.2	#DIV/0!
**LGALS1**	0	0	0.1111111111		0	#DIV/0!
**LGALS1**	0.04761904762	0.04761904762	0.05555555556		0.7	#DIV/0!
**LGALS1**	0.04761904762	0.04761904762	0.05555555556		0.7	#DIV/0!

LGALS1	0.04761904762	0.04761904762	0.05555555556		0.7	#DIV/0!
GALS17	0.04761904762	0.04761904762	0.05555555556		0.7	#DIV/0!
LGALS2	0	0	0.1111111111		0	#DIV/0!
LGALS3	0.04761904762	0.04761904762	0		inf	#DIV/0!
GALS3B	0.04761904762	0.04761904762	0		inf	#DIV/0!
LGALS4	0.04761904762	0.04761904762	0.05555555556		0.7	#DIV/0!
LGALS7	0.04761904762	0.04761904762	0.05555555556		0.7	#DIV/0!
GALS7I	0.04761904762	0.04761904762	0.05555555556		0.7	#DIV/0!
LGALS8	0.1428571429	0.1428571429	0		inf	#DIV/0!
ALS8-A	0.1428571429	0.1428571429	0		inf	#DIV/0!
LGALS9	0.04761904762	0.04761904762	0.05555555556		0.7	#DIV/0!
GALS9I	0.09523809524	0.09523809524	0.05555555556		1.473684211	#DIV/0!
GALS9C	0.04761904762	0.04761904762	0.1111111111		0.325	#DIV/0!
LGALSL	0	0	0.05555555556		0	#DIV/0!
LGI1	0.1428571429	0.1428571429	0		inf	#DIV/0!
LGI3	0.09523809524	0.09523809524	0.1111111111		0.6842105263	#DIV/0!
LGI4	0.04761904762	0.04761904762	0.05555555556		0.7	#DIV/0!
LGR5	0.04761904762	0.04761904762	0.1111111111		0.325	#DIV/0!
LGR6	0.1428571429	0.1428571429	0		inf	#DIV/0!
LHB	0.09523809524	0.09523809524	0.1111111111		0.6842105263	#DIV/0!
LHCGR	0	0	0.05555555556		0	#DIV/0!
LHFPL2	0.04761904762	0.04761904762	0		inf	#DIV/0!
LHFPL3	0.09523809524	0.09523809524	0.05555555556		1.473684211	#DIV/0!
IFPL3-A	0.09523809524	0.09523809524	0.05555555556		1.473684211	#DIV/0!
IFPL3-A	0.04761904762	0.04761904762	0.05555555556		0.7	#DIV/0!
LHFPL4	0.09523809524	0.09523809524	0		inf	#DIV/0!
LHFPL5	0.04761904762	0.04761904762	0.05555555556		0.7	#DIV/0!
LHPP	0.2380952381	0.2380952381	0.05555555556		4.375	#DIV/0!
LHX1	0.04761904762	0.04761904762	0		inf	#DIV/0!
HX1-DT	0.04761904762	0.04761904762	0		inf	#DIV/0!
LHX2	0.09523809524	0.09523809524	0.05555555556		1.473684211	#DIV/0!
LHX3	0.1428571429	0.1428571429	0.05555555556		2.333333333	#DIV/0!
LHX4	0.1428571429	0.1428571429	0		inf	#DIV/0!
HX4-AS	0.1428571429	0.1428571429	0		inf	#DIV/0!
LHX5	0	0	0.05555555556		0	#DIV/0!
HX5-AS	0	0	0.05555555556		0	#DIV/0!
LHX6	0.09523809524	0.09523809524	0.05555555556		1.473684211	#DIV/0!
LHX8	0.04761904762	0.04761904762	0.05555555556		0.7	#DIV/0!
LHX9	0.1428571429	0.1428571429	0		inf	#DIV/0!
LIAS	0.1904761905	0.1904761905	0.05555555556		3.294117647	#DIV/0!
LIF	0.04761904762	0.04761904762	0.1111111111		0.325	#DIV/0!
LIF-AS1	0.04761904762	0.04761904762	0.1111111111		0.325	#DIV/0!
LIFR	0.04761904762	0.04761904762	0.05555555556		0.7	#DIV/0!
IFR-AS	0.04761904762	0.04761904762	0.05555555556		0.7	#DIV/0!
LIG1	0.09523809524	0.09523809524	0.1111111111		0.6842105263	#DIV/0!
LILRA1	0.09523809524	0.09523809524	0.05555555556		1.473684211	#DIV/0!
LILRA2	0.09523809524	0.09523809524	0.05555555556		1.473684211	#DIV/0!
LILRA3	0.09523809524	0.09523809524	0.05555555556		1.473684211	#DIV/0!
LILRA4	0.09523809524	0.09523809524	0.05555555556		1.473684211	#DIV/0!
LILRA5	0.09523809524	0.09523809524	0.05555555556		1.473684211	#DIV/0!
LILRA6	0.09523809524	0.09523809524	0.05555555556		1.473684211	#DIV/0!

**LILRB1**	0.09523809524	0.09523809524	0.05555555556		1.473684211	#DIV/0!
**LILRB2**	0.09523809524	0.09523809524	0.05555555556		1.473684211	#DIV/0!
**LILRB3**	0.09523809524	0.09523809524	0.05555555556		1.473684211	#DIV/0!
**LILRB4**	0.09523809524	0.09523809524	0.05555555556		1.473684211	#DIV/0!
**LILRB5**	0.09523809524	0.09523809524	0.05555555556		1.473684211	#DIV/0!
**LILRP2**	0.09523809524	0.09523809524	0.05555555556		1.473684211	#DIV/0!
**LIMA1**	0.09523809524	0.09523809524	0.05555555556		1.473684211	#DIV/0!
**LIMCH1**	0.04761904762	0.04761904762	0		inf	#DIV/0!
**LIMD2**	0.04761904762	0.04761904762	0		inf	#DIV/0!
**LIME1**	0.1428571429	0.1428571429	0.2222222222		0.4583333333	#DIV/0!
**LIMK1**	0.1428571429	0.1428571429	0.1111111111		1.083333333	#DIV/0!
**LIMK2**	0.04761904762	0.04761904762	0.1111111111		0.325	#DIV/0!
**LIMS1**	0	0	0.05555555556		0	#DIV/0!
**IMS1-AS**	0	0	0.05555555556		0	#DIV/0!
**LIMS2**	0	0	0.05555555556		0	#DIV/0!
**LIMS3**	0	0	0.05555555556		0	#DIV/0!
**3-LOC4**	0	0	0.05555555556		0	#DIV/0!
**LIMS4**	0	0	0.05555555556		0	#DIV/0!
**LIN28A**	0.04761904762	0.04761904762	0.1111111111		0.325	#DIV/0!
**LIN37**	0.04761904762	0.04761904762	0.05555555556		0.7	#DIV/0!
**LIN52**	0.09523809524	0.09523809524	0		inf	#DIV/0!
**LIN54**	0	0	0.05555555556		0	#DIV/0!
**LIN7A**	0.04761904762	0.04761904762	0.05555555556		0.7	#DIV/0!
**LIN7B**	0.09523809524	0.09523809524	0.1111111111		0.6842105263	#DIV/0!
**LIN9**	0.1904761905	0.1904761905	0		inf	#DIV/0!
**INC-PIN**	0.04761904762	0.04761904762	0.05555555556		0.7	#DIV/0!
**INC-RO**	0.09523809524	0.09523809524	0		inf	#DIV/0!
**INC0002**	0.09523809524	0.09523809524	0.05555555556		1.473684211	#DIV/0!
**INC0002**	0.1428571429	0.1428571429	0.2222222222		0.4583333333	#DIV/0!
**INC0003**	0.04761904762	0.04761904762	0.05555555556		0.7	#DIV/0!
**INC0005**	0.04761904762	0.04761904762	0.1666666667		0.2	#DIV/0!
**INC0005**	0.04761904762	0.04761904762	0.05555555556		0.7	#DIV/0!
**INC0009**	0.04761904762	0.04761904762	0.05555555556		0.7	#DIV/0!
**INC0011**	0.04761904762	0.04761904762	0.1111111111		0.325	#DIV/0!
**INC0011**	0.04761904762	0.04761904762	0.1111111111		0.325	#DIV/0!
**INC0011**	0.04761904762	0.04761904762	0.1111111111		0.325	#DIV/0!
**INC0011**	0.04761904762	0.04761904762	0.1111111111		0.325	#DIV/0!
**INC0011**	0	0	0.1111111111		0	#DIV/0!
**INC0015**	0.04761904762	0.04761904762	0.1111111111		0.325	#DIV/0!
**INC0015**	0.04761904762	0.04761904762	0.1111111111		0.325	#DIV/0!
**INC0016**	0.04761904762	0.04761904762	0.1666666667		0.2	#DIV/0!
**INC0016**	0.04761904762	0.04761904762	0.1111111111		0.325	#DIV/0!
**INC0016**	0.04761904762	0.04761904762	0.1111111111		0.325	#DIV/0!
**INC0017**	0	0	0.05555555556		0	#DIV/0!
**INC0017**	0.04761904762	0.04761904762	0.1111111111		0.325	#DIV/0!
**INC0018**	0.09523809524	0.09523809524	0.05555555556		1.473684211	#DIV/0!
**INC0018**	0.04761904762	0.04761904762	0.1111111111		0.325	#DIV/0!
**INC0020**	0.04761904762	0.04761904762	0.1111111111		0.325	#DIV/0!
**INC0020**	0.04761904762	0.04761904762	0.1111111111		0.325	#DIV/0!
**INC0020**	0	0	0.1666666667		0	#DIV/0!
**INC0020**	0.09523809524	0.09523809524	0.1111111111		0.6842105263	#DIV/0!

**INC0021**	0.1428571429	0.1428571429	0		inf	#DIV/0!
**INC0021**	0	0	0.05555555556		0	#DIV/0!
**INC0021**	0.04761904762	0.04761904762	0		inf	#DIV/0!
**INC0022**	0.04761904762	0.04761904762	0		inf	#DIV/0!
**INC0022**	0	0	0			#DIV/0!
**INC0022**	0.04761904762	0.04761904762	0		inf	#DIV/0!
**INC0022**	0	0	0.1666666667		0	#DIV/0!
**INC0023**	0.04761904762	0.04761904762	0.2222222222		0.1375	#DIV/0!
**INC0023**	0.1904761905	0.1904761905	0.05555555556		3.294117647	#DIV/0!
**INC0024**	0.1428571429	0.1428571429	0.05555555556		2.333333333	#DIV/0!
**INC0025**	0.1428571429	0.1428571429	0.1666666667		0.6666666667	#DIV/0!
**INC0025**	0.09523809524	0.09523809524	0.2222222222		0.2894736842	#DIV/0!
**INC0026**	0.2380952381	0.2380952381	0		inf	#DIV/0!
**INC0026**	0.1904761905	0.1904761905	0.05555555556		3.294117647	#DIV/0!
**INC0026**	0.04761904762	0.04761904762	0.2222222222		0.1375	#DIV/0!
**NC00266**	0.1428571429	0.1428571429	0.2222222222		0.4583333333	#DIV/0!
**INC0027**	0	0	0.05555555556		0	#DIV/0!
**INC0029**	0	0	0.05555555556		0	#DIV/0!
**INC0029**	0.09523809524	0.09523809524	0.1666666667		0.4210526316	#DIV/0!
**INC0029**	0.04761904762	0.04761904762	0		inf	#DIV/0!
**INC0029**	0	0	0.05555555556		0	#DIV/0!
**INC0029**	0	0	0.05555555556		0	#DIV/0!
**INC0030**	0.1904761905	0.1904761905	0		inf	#DIV/0!
**INC0030**	0.2380952381	0.2380952381	0		inf	#DIV/0!
**INC0030**	0.09523809524	0.09523809524	0		inf	#DIV/0!
**INC0030**	0.04761904762	0.04761904762	0		inf	#DIV/0!
**INC0030**	0.04761904762	0.04761904762	0.05555555556		0.7	#DIV/0!
**INC0030**	0.04761904762	0.04761904762	0.1111111111		0.325	#DIV/0!
**INC0030**	0	0	0.05555555556		0	#DIV/0!
**INC0031**	0.04761904762	0.04761904762	0.1111111111		0.325	#DIV/0!
**INC0031**	0.04761904762	0.04761904762	0		inf	#DIV/0!
**INC0031**	0.04761904762	0.04761904762	0		inf	#DIV/0!
**INC0031**	0.04761904762	0.04761904762	0.1666666667		0.2	#DIV/0!
**INC0031**	0.04761904762	0.04761904762	0.1111111111		0.325	#DIV/0!
**INC0031**	0.04761904762	0.04761904762	0.1111111111		0.325	#DIV/0!
**INC0031**	0.04761904762	0.04761904762	0.1111111111		0.325	#DIV/0!
**INC0031**	0.04761904762	0.04761904762	0.1666666667		0.2	#DIV/0!
**INC0032**	0.04761904762	0.04761904762	0.1666666667		0.2	#DIV/0!
**INC0032**	0.04761904762	0.04761904762	0.1666666667		0.2	#DIV/0!
**INC0032**	0.04761904762	0.04761904762	0.1111111111		0.325	#DIV/0!
**INC0032**	0.04761904762	0.04761904762	0.05555555556		0.7	#DIV/0!
**INC0033**	0.04761904762	0.04761904762	0.1111111111		0.325	#DIV/0!
**INC0033**	0.04761904762	0.04761904762	0		inf	#DIV/0!
**INC0033**	0	0	0.05555555556		0	#DIV/0!
**INC0033**	0.04761904762	0.04761904762	0.1111111111		0.325	#DIV/0!
**INC0034**	0	0	0.1111111111		0	#DIV/0!
**INC0035**	0.04761904762	0.04761904762	0		inf	#DIV/0!
**INC0036**	0.04761904762	0.04761904762	0		inf	#DIV/0!
**INC0040**	0.04761904762	0.04761904762	0		inf	#DIV/0!
**INC0041**	0.04761904762	0.04761904762	0		inf	#DIV/0!
**INC0042**	0.04761904762	0.04761904762	0		inf	#DIV/0!

**INC0044**	0.04761904762	0.04761904762	0		inf	#DIV/0!
**INC0046**	0.04761904762	0.04761904762	0		inf	#DIV/0!
**INC0046**	0.04761904762	0.04761904762	0.05555555556		0.7	#DIV/0!
**INC0046**	0.1428571429	0.1428571429	0		inf	#DIV/0!
**INC0046**	0.04761904762	0.04761904762	0		inf	#DIV/0!
**INC0047**	0.09523809524	0.09523809524	0		inf	#DIV/0!
**INC0047**	0	0	0.05555555556		0	#DIV/0!
**INC0047**	0.04761904762	0.04761904762	0.05555555556		0.7	#DIV/0!
**INC0047**	0.04761904762	0.04761904762	0.05555555556		0.7	#DIV/0!
**INC0047**	0.04761904762	0.04761904762	0.05555555556		0.7	#DIV/0!
**INC0047**	0.04761904762	0.04761904762	0.05555555556		0.7	#DIV/0!
**INC0047**	0.04761904762	0.04761904762	0.1111111111		0.325	#DIV/0!
**INC0048**	0.04761904762	0.04761904762	0		inf	#DIV/0!
**INC0048**	0.04761904762	0.04761904762	0.05555555556		0.7	#DIV/0!
**INC0048**	0.04761904762	0.04761904762	0.1111111111		0.325	#DIV/0!
**INC0048**	0	0	0.05555555556		0	#DIV/0!
**INC0048**	0.09523809524	0.09523809524	0.05555555556		1.473684211	#DIV/0!
**INC0048**	0.1428571429	0.1428571429	0.1666666667		0.6666666667	#DIV/0!
**INC0049**	0.04761904762	0.04761904762	0		inf	#DIV/0!
**INC0049**	0.04761904762	0.04761904762	0		inf	#DIV/0!
**INC0049**	0.1428571429	0.1428571429	0.1666666667		0.6666666667	#DIV/0!
**INC0049**	0	0	0.05555555556		0	#DIV/0!
**INC0050**	0.1904761905	0.1904761905	0.1111111111		1.529411765	#DIV/0!
**INC0050**	0.1428571429	0.1428571429	0.1111111111		1.083333333	#DIV/0!
**INC0050**	0	0	0			#DIV/0!
**INC0050**	0.04761904762	0.04761904762	0		inf	#DIV/0!
**INC0050**	0.09523809524	0.09523809524	0.05555555556		1.473684211	#DIV/0!
**INC0050**	0.09523809524	0.09523809524	0.05555555556		1.473684211	#DIV/0!
**INC0051**	0	0	0			#DIV/0!
**INC0051**	0.04761904762	0.04761904762	0.05555555556		0.7	#DIV/0!
**INC0051**	0.04761904762	0.04761904762	0.1111111111		0.325	#DIV/0!
**INC0051**	0.04761904762	0.04761904762	0.1111111111		0.325	#DIV/0!
**INC0051**	0.04761904762	0.04761904762	0		inf	#DIV/0!
**INC0052**	0.04761904762	0.04761904762	0		inf	#DIV/0!
**INC0052**	0.04761904762	0.04761904762	0.2222222222		0.1375	#DIV/0!
**INC0052**	0.09523809524	0.09523809524	0		inf	#DIV/0!
**INC0052**	0.04761904762	0.04761904762	0.05555555556		0.7	#DIV/0!
**INC0053**	0.1904761905	0.1904761905	0.1111111111		1.529411765	#DIV/0!
**INC0053**	0.1904761905	0.1904761905	0.1111111111		1.529411765	#DIV/0!
**INC0053**	0.1904761905	0.1904761905	0.1666666667		0.9411764706	#DIV/0!
**INC0053**	0.09523809524	0.09523809524	0.2222222222		0.2894736842	#DIV/0!
**INC0053**	0.1428571429	0.1428571429	0		inf	#DIV/0!
**INC0056**	0	0	0.05555555556		0	#DIV/0!
**INC0057**	0	0	0.05555555556		0	#DIV/0!
**INC0057**	0	0	0.05555555556		0	#DIV/0!
**INC0057**	0.1904761905	0.1904761905	0.1111111111		1.529411765	#DIV/0!
**INC0058**	0.1428571429	0.1428571429	0		inf	#DIV/0!
**INC0058**	0.09523809524	0.09523809524	0.05555555556		1.473684211	#DIV/0!
**INC0058**	0.04761904762	0.04761904762	0		inf	#DIV/0!
**INC0058**	0.1428571429	0.1428571429	0.1111111111		1.083333333	#DIV/0!
**INC0058**	0.04761904762	0.04761904762	0.1111111111		0.325	#DIV/0!

INC0059	0.04761904762	0.04761904762	0.05555555556		0.7	#DIV/0!
INC0059	0.04761904762	0.04761904762	0.05555555556		0.7	#DIV/0!
INC0059	0.1428571429	0.1428571429	0.05555555556		2.333333333	#DIV/0!
INC0059	0.04761904762	0.04761904762	0.05555555556		0.7	#DIV/0!
INC0059	0.09523809524	0.09523809524	0.1111111111		0.6842105263	#DIV/0!
INC0060	0.2380952381	0.2380952381	0.05555555556		4.375	#DIV/0!
INC0060	0.04761904762	0.04761904762	0.05555555556		0.7	#DIV/0!
INC0060	0.04761904762	0.04761904762	0		inf	#DIV/0!
INC0060	0	0	0.05555555556		0	#DIV/0!
INC0060	0	0	0.05555555556		0	#DIV/0!
INC0061	0.04761904762	0.04761904762	0.1111111111		0.325	#DIV/0!
INC0061	0	0	0.05555555556		0	#DIV/0!
INC0061	0.04761904762	0.04761904762	0.05555555556		0.7	#DIV/0!
INC0061	0	0	0.05555555556		0	#DIV/0!
INC0061	0.1428571429	0.1428571429	0.05555555556		2.333333333	#DIV/0!
INC0062	0.04761904762	0.04761904762	0		inf	#DIV/0!
INC0062	0.04761904762	0.04761904762	0.05555555556		0.7	#DIV/0!
INC0062	0.1428571429	0.1428571429	0.05555555556		2.333333333	#DIV/0!
INC0062	0.1428571429	0.1428571429	0.05555555556		2.333333333	#DIV/0!
INC0062	0.1428571429	0.1428571429	0		inf	#DIV/0!
INC0062	0.2380952381	0.2380952381	0		inf	#DIV/0!
INC0063	0	0	0.1111111111		0	#DIV/0!
INC0063	0.09523809524	0.09523809524	0.05555555556		1.473684211	#DIV/0!
INC0063	0.09523809524	0.09523809524	0.05555555556		1.473684211	#DIV/0!
INC0063	0.04761904762	0.04761904762	0.05555555556		0.7	#DIV/0!
INC0064	0.04761904762	0.04761904762	0		inf	#DIV/0!
INC0064	0.04761904762	0.04761904762	0		inf	#DIV/0!
INC0064	0.04761904762	0.04761904762	0.1111111111		0.325	#DIV/0!
INC0065	0.1904761905	0.1904761905	0.05555555556		3.294117647	#DIV/0!
INC0065	0.1904761905	0.1904761905	0.05555555556		3.294117647	#DIV/0!
INC0065	0.1904761905	0.1904761905	0.05555555556		3.294117647	#DIV/0!
INC0065	0.1904761905	0.1904761905	0.05555555556		3.294117647	#DIV/0!
INC0065	0.1904761905	0.1904761905	0.05555555556		3.294117647	#DIV/0!
INC0065	0.1428571429	0.1428571429	0.2222222222		0.4583333333	#DIV/0!
INC0066	0.1428571429	0.1428571429	0.1111111111		1.083333333	#DIV/0!
INC0066	0.09523809524	0.09523809524	0.05555555556		1.473684211	#DIV/0!
INC0066	0.1428571429	0.1428571429	0.1111111111		1.083333333	#DIV/0!
INC0066	0.09523809524	0.09523809524	0		inf	#DIV/0!
INC0066	0.04761904762	0.04761904762	0.05555555556		0.7	#DIV/0!
INC0066	0.09523809524	0.09523809524	0		inf	#DIV/0!
INC0066	0.09523809524	0.09523809524	0		inf	#DIV/0!
INC0067	0.04761904762	0.04761904762	0.05555555556		0.7	#DIV/0!
INC0067	0.04761904762	0.04761904762	0		inf	#DIV/0!
INC0067	0.09523809524	0.09523809524	0		inf	#DIV/0!
INC0067	0	0	0			#DIV/0!
INC0067	0	0	0			#DIV/0!
INC0068	0.04761904762	0.04761904762	0		inf	#DIV/0!
0680-GU	0.04761904762	0.04761904762	0		inf	#DIV/0!
INC0068	0.09523809524	0.09523809524	0.1111111111		0.6842105263	#DIV/0!
INC0068	0.04761904762	0.04761904762	0		inf	#DIV/0!
INC0068	0.09523809524	0.09523809524	0.05555555556		1.473684211	#DIV/0!

**INC0068**	0.1904761905	0.1904761905	0.1111111111		1.529411765	#DIV/0!
**INC0068**	0.1428571429	0.1428571429	0.05555555556		2.333333333	#DIV/0!
**INC0069**	0.04761904762	0.04761904762	0		inf	#DIV/0!
**INC0069**	0.04761904762	0.04761904762	0		inf	#DIV/0!
**INC0069**	0.04761904762	0.04761904762	0		inf	#DIV/0!
**INC0069**	0.04761904762	0.04761904762	0		inf	#DIV/0!
**INC0069**	0.04761904762	0.04761904762	0		inf	#DIV/0!
**INC0069**	0.04761904762	0.04761904762	0		inf	#DIV/0!
**INC0070**	0.04761904762	0.04761904762	0.1111111111		0.325	#DIV/0!
**INC0070**	0	0	0.1666666667		0	#DIV/0!
**INC0070**	0.04761904762	0.04761904762	0.1666666667		0.2	#DIV/0!
**INC0070**	0.04761904762	0.04761904762	0.1666666667		0.2	#DIV/0!
**INC0070**	0.04761904762	0.04761904762	0.1666666667		0.2	#DIV/0!
**INC0070**	0.09523809524	0.09523809524	0.1111111111		0.6842105263	#DIV/0!
**INC0070**	0.09523809524	0.09523809524	0.1111111111		0.6842105263	#DIV/0!
**INC0070**	0.09523809524	0.09523809524	0.1111111111		0.6842105263	#DIV/0!
**INC0070**	0.09523809524	0.09523809524	0.1111111111		0.6842105263	#DIV/0!
**INC0071**	0.09523809524	0.09523809524	0.1111111111		0.6842105263	#DIV/0!
**INC0082**	0.09523809524	0.09523809524	0.1111111111		0.6842105263	#DIV/0!
**INC0083**	0.09523809524	0.09523809524	0.1111111111		0.6842105263	#DIV/0!
**INC0083**	0.09523809524	0.09523809524	0.1111111111		0.6842105263	#DIV/0!
**INC0083**	0.09523809524	0.09523809524	0.2222222222		0.2894736842	#DIV/0!
**INC0083**	0.1428571429	0.1428571429	0.05555555556		2.333333333	#DIV/0!
**INC0084**	0.1428571429	0.1428571429	0.05555555556		2.333333333	#DIV/0!
**INC0084**	0.1428571429	0.1428571429	0.05555555556		2.333333333	#DIV/0!
**INC0084**	0.1428571429	0.1428571429	0.05555555556		2.333333333	#DIV/0!
**INC0084**	0.04761904762	0.04761904762	0.05555555556		0.7	#DIV/0!
**INC0084**	0.04761904762	0.04761904762	0.1111111111		0.325	#DIV/0!
**INC0084**	0.09523809524	0.09523809524	0.05555555556		1.473684211	#DIV/0!
**INC0085**	0.1904761905	0.1904761905	0.05555555556		3.294117647	#DIV/0!
**INC0085**	0.09523809524	0.09523809524	0		inf	#DIV/0!
**INC0085**	0.04761904762	0.04761904762	0.05555555556		0.7	#DIV/0!
**INC0085**	0.1428571429	0.1428571429	0.05555555556		2.333333333	#DIV/0!
**INC0085**	0.1428571429	0.1428571429	0		inf	#DIV/0!
**INC0085**	0.1428571429	0.1428571429	0		inf	#DIV/0!
**INC0086**	0.1428571429	0.1428571429	0.1111111111		1.083333333	#DIV/0!
**INC0086**	0.1428571429	0.1428571429	0		inf	#DIV/0!
**INC0086**	0.1428571429	0.1428571429	0.1111111111		1.083333333	#DIV/0!
**INC0086**	0.1428571429	0.1428571429	0.1111111111		1.083333333	#DIV/0!
**INC0086**	0.1428571429	0.1428571429	0		inf	#DIV/0!
**INC0086**	0.2380952381	0.2380952381	0		inf	#DIV/0!
**INC0086**	0.04761904762	0.04761904762	0		inf	#DIV/0!
**INC0086**	0.1428571429	0.1428571429	0.05555555556		2.333333333	#DIV/0!
**INC0087**	0.04761904762	0.04761904762	0		inf	#DIV/0!
**INC0087**	0.04761904762	0.04761904762	0		inf	#DIV/0!
**INC0087**	0.04761904762	0.04761904762	0		inf	#DIV/0!
**INC0087**	0.04761904762	0.04761904762	0.05555555556		0.7	#DIV/0!
**INC0088**	0.1428571429	0.1428571429	0.05555555556		2.333333333	#DIV/0!
**INC0088**	0.1428571429	0.1428571429	0.05555555556		2.333333333	#DIV/0!
**INC0088**	0.09523809524	0.09523809524	0.05555555556		1.473684211	#DIV/0!
**INC0088**	0.09523809524	0.09523809524	0.05555555556		1.473684211	#DIV/0!

**INC0088**	0.1428571429	0.1428571429	0.05555555556		2.333333333	#DIV/0!
**INC0088**	0.1428571429	0.1428571429	0.05555555556		2.333333333	#DIV/0!
**INC0088**	0.09523809524	0.09523809524	0.05555555556		1.473684211	#DIV/0!
**INC0088**	0.1904761905	0.1904761905	0.05555555556		3.294117647	#DIV/0!
**INC0089**	0.04761904762	0.04761904762	0.05555555556		0.7	#DIV/0!
**INC0089**	0.04761904762	0.04761904762	0.05555555556		0.7	#DIV/0!
**INC0089**	0.04761904762	0.04761904762	0.1666666667		0.2	#DIV/0!
**INC0089**	0	0	0.1111111111		0	#DIV/0!
**INC0090**	0.09523809524	0.09523809524	0.05555555556		1.473684211	#DIV/0!
**INC0090**	0.09523809524	0.09523809524	0.05555555556		1.473684211	#DIV/0!
**INC0090**	0.04761904762	0.04761904762	0.05555555556		0.7	#DIV/0!
**INC0090**	0.1428571429	0.1428571429	0.1111111111		1.083333333	#DIV/0!
**INC0090**	0.04761904762	0.04761904762	0.1666666667		0.2	#DIV/0!
**INC0090**	0.04761904762	0.04761904762	0		inf	#DIV/0!
**INC0090**	0.09523809524	0.09523809524	0.05555555556		1.473684211	#DIV/0!
**INC0091**	0.04761904762	0.04761904762	0		inf	#DIV/0!
**INC0091**	0.04761904762	0.04761904762	0		inf	#DIV/0!
**INC0091**	0.04761904762	0.04761904762	0		inf	#DIV/0!
**INC0092**	0.04761904762	0.04761904762	0		inf	#DIV/0!
**INC0092**	0.04761904762	0.04761904762	0.1111111111		0.325	#DIV/0!
**INC0092**	0.04761904762	0.04761904762	0.05555555556		0.7	#DIV/0!
**INC0092**	0.04761904762	0.04761904762	0.05555555556		0.7	#DIV/0!
**INC0092**	0.04761904762	0.04761904762	0.05555555556		0.7	#DIV/0!
**INC0092**	0.04761904762	0.04761904762	0.05555555556		0.7	#DIV/0!
**INC0092**	0.04761904762	0.04761904762	0.05555555556		0.7	#DIV/0!
**INC0092**	0.04761904762	0.04761904762	0.05555555556		0.7	#DIV/0!
**INC0093**	0.04761904762	0.04761904762	0.05555555556		0.7	#DIV/0!
**INC0093**	0.04761904762	0.04761904762	0.05555555556		0.7	#DIV/0!
**INC0093**	0	0	0.05555555556		0	#DIV/0!
**INC0093**	0.04761904762	0.04761904762	0.1111111111		0.325	#DIV/0!
**INC0093**	0.04761904762	0.04761904762	0.05555555556		0.7	#DIV/0!
**INC0093**	0.09523809524	0.09523809524	0.05555555556		1.473684211	#DIV/0!
**INC0094**	0.04761904762	0.04761904762	0.1666666667		0.2	#DIV/0!
**INC0094**	0.04761904762	0.04761904762	0.05555555556		0.7	#DIV/0!
**INC0094**	0.04761904762	0.04761904762	0.1111111111		0.325	#DIV/0!
**INC0094**	0.09523809524	0.09523809524	0.05555555556		1.473684211	#DIV/0!
**INC0094**	0.09523809524	0.09523809524	0.05555555556		1.473684211	#DIV/0!
**INC0094**	0.04761904762	0.04761904762	0.1111111111		0.325	#DIV/0!
**INC0095**	0.09523809524	0.09523809524	0.05555555556		1.473684211	#DIV/0!
**INC0095**	0	0	0.05555555556		0	#DIV/0!
**INC0095**	0	0	0.05555555556		0	#DIV/0!
**INC0095**	0.04761904762	0.04761904762	0		inf	#DIV/0!
**INC0095**	0.04761904762	0.04761904762	0.1666666667		0.2	#DIV/0!
**INC0096**	0.04761904762	0.04761904762	0		inf	#DIV/0!
**INC0096**	0.09523809524	0.09523809524	0.05555555556		1.473684211	#DIV/0!
**INC0096**	0.1428571429	0.1428571429	0.1111111111		1.083333333	#DIV/0!
**INC0096**	0.09523809524	0.09523809524	0.2222222222		0.2894736842	#DIV/0!
**INC0096**	0.1428571429	0.1428571429	0.1666666667		0.6666666667	#DIV/0!
**INC0096**	0.1428571429	0.1428571429	0.1111111111		1.083333333	#DIV/0!
**INC0097**	0.1428571429	0.1428571429	0		inf	#DIV/0!
**INC0097**	0.04761904762	0.04761904762	0		inf	#DIV/0!

**INC0097**	0.09523809524	0.09523809524	0.1111111111		0.6842105263	#DIV/0!
**INC0097**	0.09523809524	0.09523809524	0		inf	#DIV/0!
**INC0097**	0.09523809524	0.09523809524	0.1111111111		0.6842105263	#DIV/0!
**INC0097**	0.09523809524	0.09523809524	0.1111111111		0.6842105263	#DIV/0!
**INC0098**	0.04761904762	0.04761904762	0.1111111111		0.325	#DIV/0!
**INC0098**	0.04761904762	0.04761904762	0.05555555556		0.7	#DIV/0!
**INC0099**	0.04761904762	0.04761904762	0		inf	#DIV/0!
**INC0099**	0.09523809524	0.09523809524	0.1111111111		0.6842105263	#DIV/0!
**INC0099**	0.04761904762	0.04761904762	0		inf	#DIV/0!
**INC0099**	0.09523809524	0.09523809524	0.05555555556		1.473684211	#DIV/0!
**INC0099**	0.04761904762	0.04761904762	0.1666666667		0.2	#DIV/0!
**INC0099**	0.09523809524	0.09523809524	0.1111111111		0.6842105263	#DIV/0!
**INC0100**	0.04761904762	0.04761904762	0.05555555556		0.7	#DIV/0!
**INC0100**	0.04761904762	0.04761904762	0		inf	#DIV/0!
**INC0100**	0.09523809524	0.09523809524	0.1111111111		0.6842105263	#DIV/0!
**INC0100**	0.09523809524	0.09523809524	0.05555555556		1.473684211	#DIV/0!
**INC0100**	0.04761904762	0.04761904762	0.05555555556		0.7	#DIV/0!
**INC0100**	0.09523809524	0.09523809524	0.05555555556		1.473684211	#DIV/0!
**INC0100**	0.1428571429	0.1428571429	0.05555555556		2.333333333	#DIV/0!
**INC0100**	0.04761904762	0.04761904762	0.05555555556		0.7	#DIV/0!
**INC0101**	0.1904761905	0.1904761905	0.05555555556		3.294117647	#DIV/0!
**INC0101**	0.04761904762	0.04761904762	0		inf	#DIV/0!
**INC0101**	0	0	0.1111111111		0	#DIV/0!
**INC0101**	0	0	0.05555555556		0	#DIV/0!
**INC0101**	0	0	0.1111111111		0	#DIV/0!
**INC0102**	0	0	0.1111111111		0	#DIV/0!
**INC0102**	0.1428571429	0.1428571429	0.05555555556		2.333333333	#DIV/0!
**INC0102**	0.04761904762	0.04761904762	0		inf	#DIV/0!
**INC0102**	0	0	0			#DIV/0!
**INC0102**	0.04761904762	0.04761904762	0.05555555556		0.7	#DIV/0!
**INC0103**	0.1904761905	0.1904761905	0.1111111111		1.529411765	#DIV/0!
**INC0103**	0.1428571429	0.1428571429	0		inf	#DIV/0!
**INC0103**	0.1428571429	0.1428571429	0		inf	#DIV/0!
**INC0103**	0.04761904762	0.04761904762	0.05555555556		0.7	#DIV/0!
**INC0103**	0.1428571429	0.1428571429	0		inf	#DIV/0!
**INC0103**	0.1428571429	0.1428571429	0		inf	#DIV/0!
**INC0104**	0.04761904762	0.04761904762	0		inf	#DIV/0!
**INC0105**	0.1428571429	0.1428571429	0.2222222222		0.4583333333	#DIV/0!
**INC0106**	0	0	0.05555555556		0	#DIV/0!
**INC0106**	0	0	0.05555555556		0	#DIV/0!
**INC0106**	0.1428571429	0.1428571429	0.05555555556		2.333333333	#DIV/0!
**INC0107**	0.04761904762	0.04761904762	0		inf	#DIV/0!
**INC0108**	0.04761904762	0.04761904762	0		inf	#DIV/0!
**INC0108**	0.04761904762	0.04761904762	0		inf	#DIV/0!
**INC0108**	0	0	0			#DIV/0!
**INC0108**	0	0	0.05555555556		0	#DIV/0!
**INC0108**	0.04761904762	0.04761904762	0.05555555556		0.7	#DIV/0!
**INC0108**	0.04761904762	0.04761904762	0.1111111111		0.325	#DIV/0!
**INC0109**	0	0	0.05555555556		0	#DIV/0!
**INC0109**	0	0	0.05555555556		0	#DIV/0!
**INC0109**	0	0	0.05555555556		0	#DIV/0!

**INC0109**	0.04761904762	0.04761904762	0.05555555556		0.7	#DIV/0!
**INC0109**	0	0	0.05555555556		0	#DIV/0!
**INC0109**	0	0	0			#DIV/0!
**INC0109**	0	0	0			#DIV/0!
**INC0109**	0	0	0.05555555556		0	#DIV/0!
**INC0109**	0	0	0.05555555556		0	#DIV/0!
**INC0110**	0.1428571429	0.1428571429	0.05555555556		2.333333333	#DIV/0!
**INC0110**	0	0	0.05555555556		0	#DIV/0!
**INC0110**	0	0	0.05555555556		0	#DIV/0!
**INC0110**	0	0	0.05555555556		0	#DIV/0!
**INC0110**	0	0	0.05555555556		0	#DIV/0!
**INC0110**	0	0	0.05555555556		0	#DIV/0!
**INC0110**	0	0	0.05555555556		0	#DIV/0!
**INC0111**	0.1904761905	0.1904761905	0.1111111111		1.529411765	#DIV/0!
**INC0111**	0	0	0.05555555556		0	#DIV/0!
**INC0111**	0	0	0.05555555556		0	#DIV/0!
**INC0111**	0	0	0.1111111111		0	#DIV/0!
**INC0111**	0	0	0.1111111111		0	#DIV/0!
**INC0111**	0	0	0.05555555556		0	#DIV/0!
**INC0111**	0	0	0.05555555556		0	#DIV/0!
**INC0112**	0	0	0.05555555556		0	#DIV/0!
**INC0112**	0	0	0.05555555556		0	#DIV/0!
**INC0112**	0.04761904762	0.04761904762	0.05555555556		0.7	#DIV/0!
**INC0112**	0	0	0.05555555556		0	#DIV/0!
**INC0112**	0	0	0.05555555556		0	#DIV/0!
**INC0112**	0	0	0.05555555556		0	#DIV/0!
**INC0112**	0	0	0.05555555556		0	#DIV/0!
**INC0112**	0	0	0.1111111111		0	#DIV/0!
**INC0113**	0.09523809524	0.09523809524	0.05555555556		1.473684211	#DIV/0!
**INC0113**	0.1428571429	0.1428571429	0		inf	#DIV/0!
**INC0113**	0	0	0			#DIV/0!
**INC0113**	0.04761904762	0.04761904762	0.05555555556		0.7	#DIV/0!
**INC0113**	0.1428571429	0.1428571429	0		inf	#DIV/0!
**INC0113**	0.04761904762	0.04761904762	0.05555555556		0.7	#DIV/0!
**INC0113**	0.09523809524	0.09523809524	0.05555555556		1.473684211	#DIV/0!
**INC0113**	0.1428571429	0.1428571429	0		inf	#DIV/0!
**INC0114**	0.04761904762	0.04761904762	0.05555555556		0.7	#DIV/0!
**INC0114**	0.04761904762	0.04761904762	0.1111111111		0.325	#DIV/0!
**INC0114**	0.1428571429	0.1428571429	0.05555555556		2.333333333	#DIV/0!
**INC0114**	0	0	0.05555555556		0	#DIV/0!
**INC0114**	0.04761904762	0.04761904762	0.05555555556		0.7	#DIV/0!
**INC0114**	0.1428571429	0.1428571429	0.05555555556		2.333333333	#DIV/0!
**INC0114**	0.04761904762	0.04761904762	0		inf	#DIV/0!
**INC0115**	0.04761904762	0.04761904762	0		inf	#DIV/0!
**INC0115**	0.1428571429	0.1428571429	0.1111111111		1.083333333	#DIV/0!
**INC0115**	0	0	0			#DIV/0!
**INC0115**	0	0	0.05555555556		0	#DIV/0!
**INC0116**	0.04761904762	0.04761904762	0		inf	#DIV/0!
**INC0116**	0.04761904762	0.04761904762	0.1666666667		0.2	#DIV/0!
**INC0116**	0.2857142857	0.2857142857	0		inf	#DIV/0!
**INC0116**	0.2857142857	0.2857142857	0		inf	#DIV/0!

INC0116	0.1904761905	0.1904761905	0		inf	#DIV/0!
INC0116	0.1904761905	0.1904761905	0		inf	#DIV/0!
INC0116	0.1904761905	0.1904761905	0		inf	#DIV/0!
INC0116	0.04761904762	0.04761904762	0.05555555556		0.7	#DIV/0!
INC0117	0.04761904762	0.04761904762	0		inf	#DIV/0!
INC0117	0	0	0.05555555556		0	#DIV/0!
INC0117	0.04761904762	0.04761904762	0.1666666667		0.2	#DIV/0!
INC0117	0.04761904762	0.04761904762	0		inf	#DIV/0!
INC0117	0	0	0.1111111111		0	#DIV/0!
INC0118	0.09523809524	0.09523809524	0		inf	#DIV/0!
INC0118	0.1904761905	0.1904761905	0.1111111111		1.529411765	#DIV/0!
INC0118	0	0	0			#DIV/0!
INC0118	0.04761904762	0.04761904762	0		inf	#DIV/0!
INC0118	0.09523809524	0.09523809524	0.05555555556		1.473684211	#DIV/0!
INC0118	0	0	0.05555555556		0	#DIV/0!
INC0118	0.1428571429	0.1428571429	0.05555555556		2.333333333	#DIV/0!
INC0119	0	0	0.05555555556		0	#DIV/0!
INC0119	0.1428571429	0.1428571429	0.05555555556		2.333333333	#DIV/0!
INC0119	0.2857142857	0.2857142857	0.1111111111		2.6	#DIV/0!
INC0119	0.04761904762	0.04761904762	0.05555555556		0.7	#DIV/0!
INC0119	0.04761904762	0.04761904762	0		inf	#DIV/0!
INC0119	0.04761904762	0.04761904762	0.05555555556		0.7	#DIV/0!
INC0120	0.04761904762	0.04761904762	0.05555555556		0.7	#DIV/0!
INC0120	0.1428571429	0.1428571429	0.05555555556		2.333333333	#DIV/0!
INC0120	0.1428571429	0.1428571429	0.05555555556		2.333333333	#DIV/0!
INC0120	0.1904761905	0.1904761905	0.05555555556		3.294117647	#DIV/0!
INC0120	0.1904761905	0.1904761905	0.05555555556		3.294117647	#DIV/0!
INC0121	0.1428571429	0.1428571429	0.05555555556		2.333333333	#DIV/0!
INC0121	0.1904761905	0.1904761905	0.1111111111		1.529411765	#DIV/0!
INC0121	0.1904761905	0.1904761905	0.1111111111		1.529411765	#DIV/0!
INC0121	0.09523809524	0.09523809524	0.05555555556		1.473684211	#DIV/0!
INC0121	0	0	0.05555555556		0	#DIV/0!
INC0121	0.04761904762	0.04761904762	0		inf	#DIV/0!
INC0122	0.1428571429	0.1428571429	0.1111111111		1.083333333	#DIV/0!
INC0122	0.1428571429	0.1428571429	0.1111111111		1.083333333	#DIV/0!
INC0122	0.09523809524	0.09523809524	0		inf	#DIV/0!
INC0122	0.04761904762	0.04761904762	0.1111111111		0.325	#DIV/0!
INC0122	0.04761904762	0.04761904762	0.1111111111		0.325	#DIV/0!
INC0122	0.09523809524	0.09523809524	0		inf	#DIV/0!
INC0122	0.09523809524	0.09523809524	0		inf	#DIV/0!
INC0123	0.04761904762	0.04761904762	0.05555555556		0.7	#DIV/0!
INC0123	0.04761904762	0.04761904762	0.05555555556		0.7	#DIV/0!
INC0123	0.09523809524	0.09523809524	0		inf	#DIV/0!
INC0123	0	0	0.05555555556		0	#DIV/0!
INC0123	0.09523809524	0.09523809524	0.05555555556		1.473684211	#DIV/0!
INC0123	0	0	0.05555555556		0	#DIV/0!
INC0123	0	0	0.05555555556		0	#DIV/0!
INC0123	0.09523809524	0.09523809524	0.05555555556		1.473684211	#DIV/0!
INC0124	0.04761904762	0.04761904762	0.05555555556		0.7	#DIV/0!
INC0124	0.09523809524	0.09523809524	0.05555555556		1.473684211	#DIV/0!
INC0124	0.09523809524	0.09523809524	0.05555555556		1.473684211	#DIV/0!

INC0124	0	0	0.05555555556		0	#DIV/0!
INC0124	0	0	0.05555555556		0	#DIV/0!
INC0124	0	0	0.05555555556		0	#DIV/0!
INC0124	0	0	0.05555555556		0	#DIV/0!
INC0125	0	0	0.05555555556		0	#DIV/0!
INC0125	0.09523809524	0.09523809524	0.05555555556		1.473684211	#DIV/0!
INC0125	0.04761904762	0.04761904762	0.1111111111		0.325	#DIV/0!
INC0125	0.09523809524	0.09523809524	0.05555555556		1.473684211	#DIV/0!
INC0125	0.09523809524	0.09523809524	0		inf	#DIV/0!
INC0125	0	0	0.05555555556		0	#DIV/0!
INC0125	0.09523809524	0.09523809524	0.05555555556		1.473684211	#DIV/0!
INC0125	0.04761904762	0.04761904762	0		inf	#DIV/0!
INC0125	0.04761904762	0.04761904762	0		inf	#DIV/0!
INC0126	0.1428571429	0.1428571429	0.1666666667		0.6666666667	#DIV/0!
INC0126	0.04761904762	0.04761904762	0.05555555556		0.7	#DIV/0!
INC0126	0.1428571429	0.1428571429	0.05555555556		2.333333333	#DIV/0!
INC0126	0.04761904762	0.04761904762	0.05555555556		0.7	#DIV/0!
INC0126	0.04761904762	0.04761904762	0		inf	#DIV/0!
INC0126	0.04761904762	0.04761904762	0		inf	#DIV/0!
INC0127	0.09523809524	0.09523809524	0.1666666667		0.4210526316	#DIV/0!
INC0127	0.09523809524	0.09523809524	0.1666666667		0.4210526316	#DIV/0!
INC0127	0.09523809524	0.09523809524	0.1666666667		0.4210526316	#DIV/0!
INC0127	0	0	0.05555555556		0	#DIV/0!
INC0127	0.09523809524	0.09523809524	0.05555555556		1.473684211	#DIV/0!
INC0128	0	0	0.05555555556		0	#DIV/0!
INC0128	0.1428571429	0.1428571429	0.05555555556		2.333333333	#DIV/0!
INC0128	0.04761904762	0.04761904762	0.1111111111		0.325	#DIV/0!
INC0128	0.1428571429	0.1428571429	0.1666666667		0.6666666667	#DIV/0!
INC0129	0.04761904762	0.04761904762	0		inf	#DIV/0!
INC0129	0	0	0.05555555556		0	#DIV/0!
INC0129	0.09523809524	0.09523809524	0.05555555556		1.473684211	#DIV/0!
INC0129	0.1904761905	0.1904761905	0.1111111111		1.529411765	#DIV/0!
INC0129	0.1428571429	0.1428571429	0.1666666667		0.6666666667	#DIV/0!
INC0130	0.04761904762	0.04761904762	0.2222222222		0.1375	#DIV/0!
INC0130	0.1428571429	0.1428571429	0.1666666667		0.6666666667	#DIV/0!
INC0130	0	0	0.05555555556		0	#DIV/0!
INC0130	0	0	0.1111111111		0	#DIV/0!
INC0130	0.04761904762	0.04761904762	0.05555555556		0.7	#DIV/0!
INC0131	0.04761904762	0.04761904762	0.1666666667		0.2	#DIV/0!
INC0131	0.04761904762	0.04761904762	0.05555555556		0.7	#DIV/0!
INC0131	0	0	0.1111111111		0	#DIV/0!
INC0132	0.1428571429	0.1428571429	0.05555555556		2.333333333	#DIV/0!
INC0132	0.1428571429	0.1428571429	0.05555555556		2.333333333	#DIV/0!
INC0132	0.1428571429	0.1428571429	0.05555555556		2.333333333	#DIV/0!
INC0133	0	0	0.05555555556		0	#DIV/0!
INC0133	0	0	0.05555555556		0	#DIV/0!
INC0133	0	0	0.05555555556		0	#DIV/0!
INC0133	0.04761904762	0.04761904762	0		inf	#DIV/0!
INC0133	0.04761904762	0.04761904762	0.05555555556		0.7	#DIV/0!
INC0133	0.04761904762	0.04761904762	0		inf	#DIV/0!
INC0134	0.04761904762	0.04761904762	0		inf	#DIV/0!

**INC0134**	0.1428571429	0.1428571429	0.05555555556		2.333333333	#DIV/0!
**INC0134**	0	0	0.1111111111		0	#DIV/0!
**INC0134**	0.04761904762	0.04761904762	0.05555555556		0.7	#DIV/0!
**INC0134**	0.1428571429	0.1428571429	0		inf	#DIV/0!
**INC0134**	0	0	0.05555555556		0	#DIV/0!
**INC0134**	0	0	0.05555555556		0	#DIV/0!
**INC0134**	0.1428571429	0.1428571429	0		inf	#DIV/0!
**INC0134**	0.04761904762	0.04761904762	0.05555555556		0.7	#DIV/0!
**INC0135**	0.1428571429	0.1428571429	0.05555555556		2.333333333	#DIV/0!
**INC0135**	0.1428571429	0.1428571429	0		inf	#DIV/0!
**INC0135**	0.1428571429	0.1428571429	0.05555555556		2.333333333	#DIV/0!
**INC0135**	0.1428571429	0.1428571429	0		inf	#DIV/0!
**INC0135**	0.09523809524	0.09523809524	0.05555555556		1.473684211	#DIV/0!
**INC0135**	0.04761904762	0.04761904762	0.1111111111		0.325	#DIV/0!
**INC0135**	0.04761904762	0.04761904762	0.05555555556		0.7	#DIV/0!
**INC0135**	0.04761904762	0.04761904762	0.05555555556		0.7	#DIV/0!
**INC0135**	0.04761904762	0.04761904762	0.05555555556		0.7	#DIV/0!
**INC0136**	0.04761904762	0.04761904762	0.05555555556		0.7	#DIV/0!
**INC0136**	0.04761904762	0.04761904762	0.05555555556		0.7	#DIV/0!
**INC0136**	0.04761904762	0.04761904762	0.05555555556		0.7	#DIV/0!
**INC0136**	0.1428571429	0.1428571429	0		inf	#DIV/0!
**INC0136**	0.04761904762	0.04761904762	0.05555555556		0.7	#DIV/0!
**INC0136**	0	0	0.05555555556		0	#DIV/0!
**INC0136**	0	0	0.05555555556		0	#DIV/0!
**INC0137**	0.1428571429	0.1428571429	0.1666666667		0.6666666667	#DIV/0!
**INC0137**	0.04761904762	0.04761904762	0.05555555556		0.7	#DIV/0!
**INC0137**	0.1428571429	0.1428571429	0.1111111111		1.083333333	#DIV/0!
**INC0137**	0.1428571429	0.1428571429	0.1111111111		1.083333333	#DIV/0!
**INC0137**	0	0	0.05555555556		0	#DIV/0!
**INC0137**	0	0	0.1111111111		0	#DIV/0!
**INC0137**	0	0	0.05555555556		0	#DIV/0!
**INC0138**	0	0	0.05555555556		0	#DIV/0!
**INC0138**	0.09523809524	0.09523809524	0		inf	#DIV/0!
**INC0138**	0	0	0.05555555556		0	#DIV/0!
**INC0139**	0.1428571429	0.1428571429	0.05555555556		2.333333333	#DIV/0!
**INC0139**	0.04761904762	0.04761904762	0.1111111111		0.325	#DIV/0!
**INC0139**	0.04761904762	0.04761904762	0.1111111111		0.325	#DIV/0!
**INC0139**	0.04761904762	0.04761904762	0		inf	#DIV/0!
**INC0139**	0.04761904762	0.04761904762	0.05555555556		0.7	#DIV/0!
**INC0139**	0	0	0.1666666667		0	#DIV/0!
**INC0140**	0	0	0.1111111111		0	#DIV/0!
**INC0141**	0.09523809524	0.09523809524	0.2222222222		0.2894736842	#DIV/0!
**INC0141**	0	0	0.05555555556		0	#DIV/0!
**INC0141**	0	0	0.05555555556		0	#DIV/0!
**INC0141**	0.04761904762	0.04761904762	0.05555555556		0.7	#DIV/0!
**INC0141**	0.1428571429	0.1428571429	0.1666666667		0.6666666667	#DIV/0!
**INC0141**	0.09523809524	0.09523809524	0		inf	#DIV/0!
**INC0141**	0.09523809524	0.09523809524	0		inf	#DIV/0!
**INC0141**	0.1428571429	0.1428571429	0.1111111111		1.083333333	#DIV/0!
**INC0142**	0.04761904762	0.04761904762	0.05555555556		0.7	#DIV/0!
**INC0142**	0.04761904762	0.04761904762	0.1111111111		0.325	#DIV/0!

INC0142	0.04761904762	0.04761904762	0.1111111111		0.325	#DIV/0!
INC0142	0.04761904762	0.04761904762	0.1111111111		0.325	#DIV/0!
INC0142	0.04761904762	0.04761904762	0.1666666667		0.2	#DIV/0!
INC0142	0.1904761905	0.1904761905	0.05555555556		3.294117647	#DIV/0!
INC0142	0.1904761905	0.1904761905	0.1111111111		1.529411765	#DIV/0!
INC0142	0.09523809524	0.09523809524	0.1111111111		0.6842105263	#DIV/0!
INC0143	0.1428571429	0.1428571429	0.1666666667		0.6666666667	#DIV/0!
INC0143	0.1904761905	0.1904761905	0.05555555556		3.294117647	#DIV/0!
INC0143	0.1904761905	0.1904761905	0.05555555556		3.294117647	#DIV/0!
INC0143	0.1904761905	0.1904761905	0.05555555556		3.294117647	#DIV/0!
INC0143	0.1904761905	0.1904761905	0		inf	#DIV/0!
INC0143	0.04761904762	0.04761904762	0.1666666667		0.2	#DIV/0!
INC0144	0.09523809524	0.09523809524	0.1666666667		0.4210526316	#DIV/0!
INC0144	0.09523809524	0.09523809524	0.1666666667		0.4210526316	#DIV/0!
INC0144	0.09523809524	0.09523809524	0.05555555556		1.473684211	#DIV/0!
INC0144	0.09523809524	0.09523809524	0.05555555556		1.473684211	#DIV/0!
INC0144	0.3333333333	0.3333333333	0.1666666667		2	#DIV/0!
INC0144	0.1428571429	0.1428571429	0.1666666667		0.6666666667	#DIV/0!
INC0144	0.04761904762	0.04761904762	0.2222222222		0.1375	#DIV/0!
INC0144	0.09523809524	0.09523809524	0.2222222222		0.2894736842	#DIV/0!
INC0144	0.04761904762	0.04761904762	0.2222222222		0.1375	#DIV/0!
INC0145	0.04761904762	0.04761904762	0.2222222222		0.1375	#DIV/0!
INC0145	0.1428571429	0.1428571429	0.05555555556		2.333333333	#DIV/0!
INC0145	0.04761904762	0.04761904762	0		inf	#DIV/0!
INC0146	0	0	0.05555555556		0	#DIV/0!
INC0146	0.04761904762	0.04761904762	0.05555555556		0.7	#DIV/0!
INC0146	0.09523809524	0.09523809524	0		inf	#DIV/0!
INC0147	0.09523809524	0.09523809524	0.05555555556		1.473684211	#DIV/0!
INC0147	0	0	0.05555555556		0	#DIV/0!
INC0147	0	0	0.05555555556		0	#DIV/0!
INC0147	0.1428571429	0.1428571429	0		inf	#DIV/0!
INC0147	0.1428571429	0.1428571429	0		inf	#DIV/0!
INC0147	0.04761904762	0.04761904762	0		inf	#DIV/0!
INC0147	0.04761904762	0.04761904762	0		inf	#DIV/0!
INC0147	0.09523809524	0.09523809524	0.05555555556		1.473684211	#DIV/0!
INC0148	0.04761904762	0.04761904762	0.05555555556		0.7	#DIV/0!
INC0148	0.09523809524	0.09523809524	0.1111111111		0.6842105263	#DIV/0!
INC0148	0	0	0			#DIV/0!
INC0148	0	0	0			#DIV/0!
INC0148	0	0	0.05555555556		0	#DIV/0!
INC0148	0	0	0.05555555556		0	#DIV/0!
INC0148	0	0	0.05555555556		0	#DIV/0!
INC0148	0.1904761905	0.1904761905	0.05555555556		3.294117647	#DIV/0!
INC0148	0.04761904762	0.04761904762	0.1111111111		0.325	#DIV/0!
INC0149	0.04761904762	0.04761904762	0.05555555556		0.7	#DIV/0!
INC0149	0.04761904762	0.04761904762	0.05555555556		0.7	#DIV/0!
INC0149	0.04761904762	0.04761904762	0		inf	#DIV/0!
INC0149	0.04761904762	0.04761904762	0		inf	#DIV/0!
INC0149	0	0	0.05555555556		0	#DIV/0!
INC0149	0	0	0			#DIV/0!
INC0149	0.04761904762	0.04761904762	0.05555555556		0.7	#DIV/0!

**INC0150**	0.04761904762	0.04761904762	0.05555555556		0.7	#DIV/0!
**INC0150**	0.04761904762	0.04761904762	0		inf	#DIV/0!
**INC0150**	0.09523809524	0.09523809524	0.05555555556		1.473684211	#DIV/0!
**INC0150**	0	0	0.05555555556		0	#DIV/0!
**INC0150**	0.04761904762	0.04761904762	0.05555555556		0.7	#DIV/0!
**INC0150**	0	0	0.05555555556		0	#DIV/0!
**INC0150**	0	0	0.05555555556		0	#DIV/0!
**INC0150**	0.04761904762	0.04761904762	0.05555555556		0.7	#DIV/0!
**INC0150**	0.04761904762	0.04761904762	0.05555555556		0.7	#DIV/0!
**INC0151**	0.04761904762	0.04761904762	0.1666666667		0.2	#DIV/0!
**INC0151**	0	0	0.1111111111		0	#DIV/0!
**INC0151**	0.04761904762	0.04761904762	0.05555555556		0.7	#DIV/0!
**INC0151**	0.1428571429	0.1428571429	0		inf	#DIV/0!
**INC0151**	0.1428571429	0.1428571429	0		inf	#DIV/0!
**INC0151**	0.09523809524	0.09523809524	0.1111111111		0.6842105263	#DIV/0!
**INC0151**	0.09523809524	0.09523809524	0.1111111111		0.6842105263	#DIV/0!
**INC0151**	0.1428571429	0.1428571429	0.05555555556		2.333333333	#DIV/0!
**INC0151**	0.1428571429	0.1428571429	0		inf	#DIV/0!
**INC0152**	0.1428571429	0.1428571429	0		inf	#DIV/0!
**INC0152**	0.04761904762	0.04761904762	0.1111111111		0.325	#DIV/0!
**INC0152**	0.1428571429	0.1428571429	0.1666666667		0.6666666667	#DIV/0!
**INC0152**	0.1428571429	0.1428571429	0.1666666667		0.6666666667	#DIV/0!
**INC0152**	0.09523809524	0.09523809524	0.1111111111		0.6842105263	#DIV/0!
**INC0152**	0.04761904762	0.04761904762	0.05555555556		0.7	#DIV/0!
**INC0152**	0.04761904762	0.04761904762	0.05555555556		0.7	#DIV/0!
**INC0153**	0.04761904762	0.04761904762	0.05555555556		0.7	#DIV/0!
**INC0153**	0.04761904762	0.04761904762	0.1666666667		0.2	#DIV/0!
**INC0153**	0.04761904762	0.04761904762	0.05555555556		0.7	#DIV/0!
**INC0153**	0.04761904762	0.04761904762	0.05555555556		0.7	#DIV/0!
**INC0153**	0.04761904762	0.04761904762	0.05555555556		0.7	#DIV/0!
**INC0153**	0.04761904762	0.04761904762	0		inf	#DIV/0!
**INC0153**	0.09523809524	0.09523809524	0		inf	#DIV/0!
**INC0154**	0.09523809524	0.09523809524	0.05555555556		1.473684211	#DIV/0!
**INC0154**	0.09523809524	0.09523809524	0		inf	#DIV/0!
**INC0154**	0.04761904762	0.04761904762	0.1111111111		0.325	#DIV/0!
**INC0154**	0.04761904762	0.04761904762	0.1111111111		0.325	#DIV/0!
**INC0154**	0.04761904762	0.04761904762	0.1111111111		0.325	#DIV/0!
**INC0155**	0.04761904762	0.04761904762	0.05555555556		0.7	#DIV/0!
**INC0155**	0.04761904762	0.04761904762	0		inf	#DIV/0!
**INC0155**	0.04761904762	0.04761904762	0.05555555556		0.7	#DIV/0!
**INC0155**	0.04761904762	0.04761904762	0.05555555556		0.7	#DIV/0!
**INC0156**	0.2380952381	0.2380952381	0		inf	#DIV/0!
**INC0156**	0.04761904762	0.04761904762	0.05555555556		0.7	#DIV/0!
**INC0156**	0.09523809524	0.09523809524	0.05555555556		1.473684211	#DIV/0!
**INC0156**	0.04761904762	0.04761904762	0		inf	#DIV/0!
**INC0156**	0.09523809524	0.09523809524	0.05555555556		1.473684211	#DIV/0!
**INC0156**	0.04761904762	0.04761904762	0		inf	#DIV/0!
**INC0156**	0.04761904762	0.04761904762	0		inf	#DIV/0!
**INC0156**	0.04761904762	0.04761904762	0		inf	#DIV/0!
**INC0156**	0.04761904762	0.04761904762	0		inf	#DIV/0!
**INC0157**	0.04761904762	0.04761904762	0		inf	#DIV/0!

INC0157	0.04761904762	0.04761904762	0		inf	#DIV/0!
INC0157	0.04761904762	0.04761904762	0		inf	#DIV/0!
INC0157	0.09523809524	0.09523809524	0.05555555556		1.473684211	#DIV/0!
INC0157	0.04761904762	0.04761904762	0.05555555556		0.7	#DIV/0!
INC0157	0.04761904762	0.04761904762	0.05555555556		0.7	#DIV/0!
INC0158	0.04761904762	0.04761904762	0.05555555556		0.7	#DIV/0!
INC0158	0.04761904762	0.04761904762	0.05555555556		0.7	#DIV/0!
INC0158	0.04761904762	0.04761904762	0.05555555556		0.7	#DIV/0!
INC0158	0.04761904762	0.04761904762	0.05555555556		0.7	#DIV/0!
INC0158	0.09523809524	0.09523809524	0.05555555556		1.473684211	#DIV/0!
INC0158	0.04761904762	0.04761904762	0.05555555556		0.7	#DIV/0!
INC0158	0.04761904762	0.04761904762	0.05555555556		0.7	#DIV/0!
INC0158	0.04761904762	0.04761904762	0		inf	#DIV/0!
INC0158	0	0	0.1111111111		0	#DIV/0!
INC0159	0.04761904762	0.04761904762	0.2222222222		0.1375	#DIV/0!
INC0159	0.1428571429	0.1428571429	0.1666666667		0.6666666667	#DIV/0!
INC0159	0	0	0.05555555556		0	#DIV/0!
INC0159	0	0	0.05555555556		0	#DIV/0!
INC0159	0.04761904762	0.04761904762	0.05555555556		0.7	#DIV/0!
INC0159	0.09523809524	0.09523809524	0.05555555556		1.473684211	#DIV/0!
INC0159	0.04761904762	0.04761904762	0		inf	#DIV/0!
INC0160	0.04761904762	0.04761904762	0		inf	#DIV/0!
INC0160	0.1428571429	0.1428571429	0.1666666667		0.6666666667	#DIV/0!
INC0160	0.1428571429	0.1428571429	0.1666666667		0.6666666667	#DIV/0!
INC0160	0.04761904762	0.04761904762	0.1111111111		0.325	#DIV/0!
INC0160	0.1428571429	0.1428571429	0.1111111111		1.083333333	#DIV/0!
INC0160	0.1428571429	0.1428571429	0.1111111111		1.083333333	#DIV/0!
INC0160	0.1904761905	0.1904761905	0.1111111111		1.529411765	#DIV/0!
INC0160	0.1904761905	0.1904761905	0.1111111111		1.529411765	#DIV/0!
INC0161	0	0	0.05555555556		0	#DIV/0!
INC0161	0.04761904762	0.04761904762	0		inf	#DIV/0!
INC0161	0	0	0.05555555556		0	#DIV/0!
INC0161	0.1904761905	0.1904761905	0.1111111111		1.529411765	#DIV/0!
INC0161	0.2380952381	0.2380952381	0.05555555556		4.375	#DIV/0!
INC0161	0.04761904762	0.04761904762	0.05555555556		0.7	#DIV/0!
INC0162	0.1428571429	0.1428571429	0.1666666667		0.6666666667	#DIV/0!
INC0162	0	0	0.05555555556		0	#DIV/0!
INC0163	0.09523809524	0.09523809524	0		inf	#DIV/0!
INC0163	0.1428571429	0.1428571429	0.05555555556		2.333333333	#DIV/0!
INC0163	0.04761904762	0.04761904762	0.05555555556		0.7	#DIV/0!
INC0163	0.04761904762	0.04761904762	0.1111111111		0.325	#DIV/0!
INC0163	0.04761904762	0.04761904762	0.05555555556		0.7	#DIV/0!
INC0163	0.04761904762	0.04761904762	0.1111111111		0.325	#DIV/0!
INC0163	0	0	0.1666666667		0	#DIV/0!
INC0164	0.04761904762	0.04761904762	0.1666666667		0.2	#DIV/0!
INC0164	0.04761904762	0.04761904762	0.1666666667		0.2	#DIV/0!
INC0164	0.1428571429	0.1428571429	0		inf	#DIV/0!
INC0164	0	0	0.05555555556		0	#DIV/0!
INC0164	0.04761904762	0.04761904762	0.1111111111		0.325	#DIV/0!
INC0164	0	0	0.05555555556		0	#DIV/0!
INC0164	0.04761904762	0.04761904762	0.05555555556		0.7	#DIV/0!

INC0165	0.04761904762	0.04761904762	0.05555555556		0.7	#DIV/0!
INC0165	0.1428571429	0.1428571429	0		inf	#DIV/0!
INC0165	0.04761904762	0.04761904762	0.05555555556		0.7	#DIV/0!
INC0165	0.1904761905	0.1904761905	0.05555555556		3.294117647	#DIV/0!
INC0165	0	0	0.1666666667		0	#DIV/0!
INC0165	0.1428571429	0.1428571429	0		inf	#DIV/0!
INC0165	0	0	0.05555555556		0	#DIV/0!
INC0166	0.04761904762	0.04761904762	0.05555555556		0.7	#DIV/0!
INC0166	0.04761904762	0.04761904762	0		inf	#DIV/0!
INC0166	0.04761904762	0.04761904762	0		inf	#DIV/0!
INC0166	0.04761904762	0.04761904762	0.1666666667		0.2	#DIV/0!
INC0166	0.04761904762	0.04761904762	0.1666666667		0.2	#DIV/0!
INC0167	0.04761904762	0.04761904762	0.1666666667		0.2	#DIV/0!
INC0167	0	0	0.05555555556		0	#DIV/0!
INC0167	0.04761904762	0.04761904762	0		inf	#DIV/0!
INC0167	0.1428571429	0.1428571429	0		inf	#DIV/0!
INC0167	0.04761904762	0.04761904762	0.05555555556		0.7	#DIV/0!
INC0167	0.04761904762	0.04761904762	0.05555555556		0.7	#DIV/0!
INC0167	0.04761904762	0.04761904762	0.1111111111		0.325	#DIV/0!
INC0167	0.04761904762	0.04761904762	0.1666666667		0.2	#DIV/0!
INC0168	0.1428571429	0.1428571429	0		inf	#DIV/0!
INC0168	0.1428571429	0.1428571429	0.05555555556		2.333333333	#DIV/0!
INC0168	0.1428571429	0.1428571429	0		inf	#DIV/0!
INC0168	0.04761904762	0.04761904762	0.1111111111		0.325	#DIV/0!
INC0168	0.04761904762	0.04761904762	0.1111111111		0.325	#DIV/0!
INC0168	0.04761904762	0.04761904762	0.05555555556		0.7	#DIV/0!
INC0168	0.1428571429	0.1428571429	0		inf	#DIV/0!
INC0168	0.04761904762	0.04761904762	0.1111111111		0.325	#DIV/0!
INC0168	0.1428571429	0.1428571429	0		inf	#DIV/0!
INC0168	0.04761904762	0.04761904762	0.1111111111		0.325	#DIV/0!
INC0169	0.04761904762	0.04761904762	0.1111111111		0.325	#DIV/0!
INC0169	0.04761904762	0.04761904762	0.1111111111		0.325	#DIV/0!
INC0169	0.1428571429	0.1428571429	0		inf	#DIV/0!
INC0169	0.04761904762	0.04761904762	0.1111111111		0.325	#DIV/0!
INC0169	0.04761904762	0.04761904762	0.1111111111		0.325	#DIV/0!
INC0169	0.1428571429	0.1428571429	0		inf	#DIV/0!
INC0169	0.04761904762	0.04761904762	0.1111111111		0.325	#DIV/0!
INC0169	0.1428571429	0.1428571429	0		inf	#DIV/0!
INC0169	0.1428571429	0.1428571429	0		inf	#DIV/0!
INC0170	0.04761904762	0.04761904762	0.1111111111		0.325	#DIV/0!
INC0170	0.1428571429	0.1428571429	0.05555555556		2.333333333	#DIV/0!
INC0170	0.1428571429	0.1428571429	0.05555555556		2.333333333	#DIV/0!
INC0170	0.1904761905	0.1904761905	0.1111111111		1.529411765	#DIV/0!
INC0170	0.04761904762	0.04761904762	0.05555555556		0.7	#DIV/0!
INC0170	0.04761904762	0.04761904762	0.05555555556		0.7	#DIV/0!
INC0171	0.1428571429	0.1428571429	0.05555555556		2.333333333	#DIV/0!
INC0171	0.1428571429	0.1428571429	0.2222222222		0.4583333333	#DIV/0!
INC0171	0.04761904762	0.04761904762	0.05555555556		0.7	#DIV/0!
INC0171	0.1904761905	0.1904761905	0.1111111111		1.529411765	#DIV/0!
INC0171	0	0	0.05555555556		0	#DIV/0!
INC0171	0.04761904762	0.04761904762	0.1111111111		0.325	#DIV/0!

INC0171	0.09523809524	0.09523809524	0.1666666667		0.4210526316	#DIV/0!
INC0171	0.1428571429	0.1428571429	0		inf	#DIV/0!
INC0171	0.1428571429	0.1428571429	0.1111111111		1.083333333	#DIV/0!
INC0171	0.1428571429	0.1428571429	0.05555555556		2.333333333	#DIV/0!
INC0172	0.1428571429	0.1428571429	0		inf	#DIV/0!
INC0172	0.1904761905	0.1904761905	0.05555555556		3.294117647	#DIV/0!
INC0172	0.1904761905	0.1904761905	0.1111111111		1.529411765	#DIV/0!
INC0172	0.1904761905	0.1904761905	0.1111111111		1.529411765	#DIV/0!
INC0172	0.1428571429	0.1428571429	0		inf	#DIV/0!
INC0172	0.04761904762	0.04761904762	0.05555555556		0.7	#DIV/0!
INC0172	0.1904761905	0.1904761905	0.05555555556		3.294117647	#DIV/0!
INC0172	0.1904761905	0.1904761905	0.05555555556		3.294117647	#DIV/0!
INC0172	0.1428571429	0.1428571429	0.1666666667		0.6666666667	#DIV/0!
INC0172	0.1904761905	0.1904761905	0.05555555556		3.294117647	#DIV/0!
INC0173	0.1904761905	0.1904761905	0.05555555556		3.294117647	#DIV/0!
INC0173	0.1428571429	0.1428571429	0		inf	#DIV/0!
INC0173	0.1904761905	0.1904761905	0.05555555556		3.294117647	#DIV/0!
INC0173	0.1428571429	0.1428571429	0.1666666667		0.6666666667	#DIV/0!
INC0173	0.1428571429	0.1428571429	0		inf	#DIV/0!
INC0173	0.1428571429	0.1428571429	0		inf	#DIV/0!
INC0173	0.04761904762	0.04761904762	0.05555555556		0.7	#DIV/0!
INC0174	0.09523809524	0.09523809524	0		inf	#DIV/0!
INC0174	0.1428571429	0.1428571429	0		inf	#DIV/0!
INC0174	0.1428571429	0.1428571429	0.2222222222		0.4583333333	#DIV/0!
INC0174	0.1428571429	0.1428571429	0.05555555556		2.333333333	#DIV/0!
INC0174	0.1428571429	0.1428571429	0		inf	#DIV/0!
INC0174	0.1428571429	0.1428571429	0		inf	#DIV/0!
INC0174	0.1904761905	0.1904761905	0.05555555556		3.294117647	#DIV/0!
INC0174	0.04761904762	0.04761904762	0.05555555556		0.7	#DIV/0!
INC0174	0.1428571429	0.1428571429	0.2222222222		0.4583333333	#DIV/0!
INC0175	0.04761904762	0.04761904762	0		inf	#DIV/0!
INC0175	0.1904761905	0.1904761905	0.1111111111		1.529411765	#DIV/0!
INC0175	0.1904761905	0.1904761905	0.1666666667		0.9411764706	#DIV/0!
INC0175	0.04761904762	0.04761904762	0.05555555556		0.7	#DIV/0!
INC0175	0.1428571429	0.1428571429	0.1666666667		0.6666666667	#DIV/0!
INC0175	0.04761904762	0.04761904762	0.05555555556		0.7	#DIV/0!
INC0175	0	0	0.05555555556		0	#DIV/0!
INC0175	0.04761904762	0.04761904762	0.1111111111		0.325	#DIV/0!
INC0175	0.04761904762	0.04761904762	0.05555555556		0.7	#DIV/0!
INC0176	0.04761904762	0.04761904762	0.05555555556		0.7	#DIV/0!
INC0176	0.04761904762	0.04761904762	0.05555555556		0.7	#DIV/0!
INC0176	0.04761904762	0.04761904762	0.05555555556		0.7	#DIV/0!
INC0176	0.04761904762	0.04761904762	0.05555555556		0.7	#DIV/0!
INC0176	0.04761904762	0.04761904762	0.05555555556		0.7	#DIV/0!
INC0176	0.04761904762	0.04761904762	0.05555555556		0.7	#DIV/0!
INC0177	0	0	0.1111111111		0	#DIV/0!
INC0177	0.04761904762	0.04761904762	0.05555555556		0.7	#DIV/0!
INC0177	0.04761904762	0.04761904762	0.1111111111		0.325	#DIV/0!
INC0177	0.1428571429	0.1428571429	0		inf	#DIV/0!
INC0177	0.09523809524	0.09523809524	0.05555555556		1.473684211	#DIV/0!
INC0177	0.04761904762	0.04761904762	0.05555555556		0.7	#DIV/0!

INC0177	0	0	0.05555555556		0	#DIV/0!
INC0177	0.04761904762	0.04761904762	0.1111111111		0.325	#DIV/0!
INC0178	0.04761904762	0.04761904762	0.05555555556		0.7	#DIV/0!
INC0178	0.04761904762	0.04761904762	0.05555555556		0.7	#DIV/0!
INC0178	0.04761904762	0.04761904762	0.05555555556		0.7	#DIV/0!
INC0178	0.04761904762	0.04761904762	0.1111111111		0.325	#DIV/0!
INC0178	0.04761904762	0.04761904762	0.1111111111		0.325	#DIV/0!
INC0178	0.09523809524	0.09523809524	0		inf	#DIV/0!
INC0178	0.04761904762	0.04761904762	0.05555555556		0.7	#DIV/0!
INC0178	0.04761904762	0.04761904762	0.05555555556		0.7	#DIV/0!
INC0179	0	0	0.05555555556		0	#DIV/0!
INC0179	0.04761904762	0.04761904762	0.05555555556		0.7	#DIV/0!
INC0179	0	0	0.05555555556		0	#DIV/0!
INC0179	0.04761904762	0.04761904762	0.05555555556		0.7	#DIV/0!
INC0179	0.04761904762	0.04761904762	0.05555555556		0.7	#DIV/0!
INC0179	0	0	0.05555555556		0	#DIV/0!
INC0179	0	0	0.05555555556		0	#DIV/0!
INC0179	0	0	0.05555555556		0	#DIV/0!
INC0179	0	0	0.05555555556		0	#DIV/0!
INC0180	0	0	0.05555555556		0	#DIV/0!
INC0180	0.04761904762	0.04761904762	0.05555555556		0.7	#DIV/0!
INC0180	0	0	0.05555555556		0	#DIV/0!
INC0180	0	0	0.05555555556		0	#DIV/0!
INC0180	0	0	0.05555555556		0	#DIV/0!
INC0180	0	0	0.05555555556		0	#DIV/0!
INC0180	0	0	0.05555555556		0	#DIV/0!
INC0180	0	0	0.05555555556		0	#DIV/0!
INC0180	0	0	0.05555555556		0	#DIV/0!
INC0181	0	0	0.05555555556		0	#DIV/0!
INC0181	0	0	0.05555555556		0	#DIV/0!
INC0181	0.04761904762	0.04761904762	0.05555555556		0.7	#DIV/0!
INC0181	0	0	0.05555555556		0	#DIV/0!
INC0181	0	0	0.05555555556		0	#DIV/0!
INC0181	0	0	0.05555555556		0	#DIV/0!
INC0181	0	0	0.05555555556		0	#DIV/0!
INC0181	0	0	0.05555555556		0	#DIV/0!
INC0182	0	0	0.05555555556		0	#DIV/0!
INC0182	0	0	0.05555555556		0	#DIV/0!
INC0182	0	0	0.05555555556		0	#DIV/0!
INC0182	0	0	0.05555555556		0	#DIV/0!
INC0182	0	0	0.05555555556		0	#DIV/0!
INC0182	0	0	0.05555555556		0	#DIV/0!
INC0183	0	0	0.05555555556		0	#DIV/0!
INC0183	0	0	0.05555555556		0	#DIV/0!
INC0183	0	0	0.05555555556		0	#DIV/0!
INC0183	0.1428571429	0.1428571429	0.1111111111		1.083333333	#DIV/0!
INC0183	0.04761904762	0.04761904762	0.05555555556		0.7	#DIV/0!
INC0184	0.1428571429	0.1428571429	0.05555555556		2.333333333	#DIV/0!
INC0184	0.1428571429	0.1428571429	0.1111111111		1.083333333	#DIV/0!
INC0184	0.1428571429	0.1428571429	0.1111111111		1.083333333	#DIV/0!
INC0184	0.04761904762	0.04761904762	0		inf	#DIV/0!

INC0184	0.04761904762	0.04761904762	0		inf	#DIV/0!
INC0184	0.04761904762	0.04761904762	0.05555555556		0.7	#DIV/0!
INC0184	0.04761904762	0.04761904762	0		inf	#DIV/0!
INC0184	0.04761904762	0.04761904762	0.05555555556		0.7	#DIV/0!
INC0184	0.04761904762	0.04761904762	0		inf	#DIV/0!
INC0184	0	0	0.05555555556		0	#DIV/0!
INC0185	0	0	0.05555555556		0	#DIV/0!
INC0185	0	0	0			#DIV/0!
INC0185	0	0	0.05555555556		0	#DIV/0!
INC0185	0.1428571429	0.1428571429	0.1111111111		1.083333333	#DIV/0!
INC0185	0	0	0.05555555556		0	#DIV/0!
INC0185	0	0	0.05555555556		0	#DIV/0!
INC0185	0.09523809524	0.09523809524	0		inf	#DIV/0!
INC0185	0.09523809524	0.09523809524	0		inf	#DIV/0!
INC0186	0.1428571429	0.1428571429	0.05555555556		2.333333333	#DIV/0!
INC0186	0	0	0.05555555556		0	#DIV/0!
INC0186	0.04761904762	0.04761904762	0.05555555556		0.7	#DIV/0!
INC0186	0.09523809524	0.09523809524	0.05555555556		1.473684211	#DIV/0!
INC0187	0	0	0.05555555556		0	#DIV/0!
INC0187	0	0	0.05555555556		0	#DIV/0!
INC0187	0	0	0.05555555556		0	#DIV/0!
INC0187	0	0	0.05555555556		0	#DIV/0!
INC0187	0	0	0.05555555556		0	#DIV/0!
INC0187	0	0	0.05555555556		0	#DIV/0!
INC0187	0.09523809524	0.09523809524	0.05555555556		1.473684211	#DIV/0!
INC0188	0	0	0.05555555556		0	#DIV/0!
INC0188	0	0	0.05555555556		0	#DIV/0!
INC0188	0.09523809524	0.09523809524	0.05555555556		1.473684211	#DIV/0!
INC0188	0	0	0.05555555556		0	#DIV/0!
INC0188	0	0	0.05555555556		0	#DIV/0!
INC0188	0	0	0.05555555556		0	#DIV/0!
INC0188	0.09523809524	0.09523809524	0		inf	#DIV/0!
INC0189	0	0	0.05555555556		0	#DIV/0!
INC0189	0.09523809524	0.09523809524	0		inf	#DIV/0!
INC0189	0.09523809524	0.09523809524	0.05555555556		1.473684211	#DIV/0!
INC0189	0.09523809524	0.09523809524	0.1111111111		0.6842105263	#DIV/0!
INC0189	0.09523809524	0.09523809524	0		inf	#DIV/0!
INC0189	0.09523809524	0.09523809524	0		inf	#DIV/0!
INC0190	0.09523809524	0.09523809524	0		inf	#DIV/0!
INC0190	0.09523809524	0.09523809524	0		inf	#DIV/0!
INC0190	0.09523809524	0.09523809524	0		inf	#DIV/0!
INC0190	0	0	0.05555555556		0	#DIV/0!
INC0191	0	0	0.05555555556		0	#DIV/0!
INC0191	0	0	0.05555555556		0	#DIV/0!
INC0191	0.09523809524	0.09523809524	0.05555555556		1.473684211	#DIV/0!
INC0191	0.09523809524	0.09523809524	0		inf	#DIV/0!
INC0191	0	0	0.05555555556		0	#DIV/0!
INC0191	0.09523809524	0.09523809524	0		inf	#DIV/0!
INC0192	0	0	0.05555555556		0	#DIV/0!
INC0192	0	0	0.1111111111		0	#DIV/0!
INC0192	0	0	0.05555555556		0	#DIV/0!

INC0192	0.04761904762	0.04761904762	0		inf	#DIV/0!
INC0192	0.09523809524	0.09523809524	0		inf	#DIV/0!
INC0192	0.09523809524	0.09523809524	0		inf	#DIV/0!
INC0192	0.09523809524	0.09523809524	0.05555555556		1.473684211	#DIV/0!
INC0192	0.09523809524	0.09523809524	0		inf	#DIV/0!
INC0192	0.09523809524	0.09523809524	0.05555555556		1.473684211	#DIV/0!
INC0193	0.04761904762	0.04761904762	0.05555555556		0.7	#DIV/0!
INC0193	0	0	0.05555555556		0	#DIV/0!
INC0193	0.04761904762	0.04761904762	0.05555555556		0.7	#DIV/0!
INC0193	0	0	0.05555555556		0	#DIV/0!
INC0193	0	0	0.05555555556		0	#DIV/0!
INC0193	0	0	0.05555555556		0	#DIV/0!
INC0193	0	0	0.05555555556		0	#DIV/0!
INC0194	0	0	0.05555555556		0	#DIV/0!
INC0194	0	0	0.05555555556		0	#DIV/0!
INC0194	0	0	0.05555555556		0	#DIV/0!
INC0194	0	0	0.05555555556		0	#DIV/0!
INC0194	0	0	0.05555555556		0	#DIV/0!
INC0194	0.09523809524	0.09523809524	0		inf	#DIV/0!
INC0194	0	0	0.05555555556		0	#DIV/0!
INC0194	0.04761904762	0.04761904762	0.05555555556		0.7	#DIV/0!
INC0194	0.04761904762	0.04761904762	0		inf	#DIV/0!
INC0195	0.04761904762	0.04761904762	0		inf	#DIV/0!
INC0195	0	0	0.05555555556		0	#DIV/0!
INC0195	0	0	0.05555555556		0	#DIV/0!
INC0195	0	0	0.05555555556		0	#DIV/0!
INC0195	0.04761904762	0.04761904762	0		inf	#DIV/0!
INC0195	0.04761904762	0.04761904762	0		inf	#DIV/0!
INC0196	0	0	0.1111111111		0	#DIV/0!
INC0196	0.09523809524	0.09523809524	0.05555555556		1.473684211	#DIV/0!
INC0196	0	0	0.05555555556		0	#DIV/0!
INC0196	0	0	0.05555555556		0	#DIV/0!
INC0196	0.09523809524	0.09523809524	0.05555555556		1.473684211	#DIV/0!
INC0196	0.09523809524	0.09523809524	0		inf	#DIV/0!
INC0197	0.04761904762	0.04761904762	0		inf	#DIV/0!
INC0197	0.09523809524	0.09523809524	0.05555555556		1.473684211	#DIV/0!
INC0197	0.04761904762	0.04761904762	0		inf	#DIV/0!
INC0197	0	0	0.1111111111		0	#DIV/0!
INC0197	0.09523809524	0.09523809524	0		inf	#DIV/0!
INC0197	0.04761904762	0.04761904762	0		inf	#DIV/0!
INC0197	0.04761904762	0.04761904762	0		inf	#DIV/0!
INC0197	0.04761904762	0.04761904762	0		inf	#DIV/0!
INC0198	0.04761904762	0.04761904762	0		inf	#DIV/0!
INC0198	0.04761904762	0.04761904762	0		inf	#DIV/0!
INC0198	0.04761904762	0.04761904762	0		inf	#DIV/0!
INC0198	0.1428571429	0.1428571429	0.05555555556		2.333333333	#DIV/0!
INC0198	0.04761904762	0.04761904762	0		inf	#DIV/0!
INC0198	0.04761904762	0.04761904762	0.05555555556		0.7	#DIV/0!
INC0198	0.04761904762	0.04761904762	0		inf	#DIV/0!
INC0198	0.04761904762	0.04761904762	0		inf	#DIV/0!
INC0199	0.09523809524	0.09523809524	0.05555555556		1.473684211	#DIV/0!

INC0199	0.09523809524	0.09523809524	0.1111111111		0.6842105263	#DIV/0!
INC0199	0.04761904762	0.04761904762	0.05555555556		0.7	#DIV/0!
INC0199	0.04761904762	0.04761904762	0		inf	#DIV/0!
INC0199	0.1428571429	0.1428571429	0.05555555556		2.333333333	#DIV/0!
INC0199	0.1428571429	0.1428571429	0.05555555556		2.333333333	#DIV/0!
INC0199	0	0	0.1111111111		0	#DIV/0!
INC0199	0.1428571429	0.1428571429	0		inf	#DIV/0!
INC0200	0.1428571429	0.1428571429	0.05555555556		2.333333333	#DIV/0!
INC0200	0	0	0			#DIV/0!
INC0200	0.1428571429	0.1428571429	0.05555555556		2.333333333	#DIV/0!
INC0200	0.04761904762	0.04761904762	0		inf	#DIV/0!
INC0200	0.1904761905	0.1904761905	0.1111111111		1.529411765	#DIV/0!
INC0200	0.04761904762	0.04761904762	0		inf	#DIV/0!
INC0201	0.1428571429	0.1428571429	0.1111111111		1.083333333	#DIV/0!
INC0201	0.04761904762	0.04761904762	0		inf	#DIV/0!
INC0201	0.1428571429	0.1428571429	0.05555555556		2.333333333	#DIV/0!
INC0201	0.09523809524	0.09523809524	0.05555555556		1.473684211	#DIV/0!
INC0201	0.1904761905	0.1904761905	0.1111111111		1.529411765	#DIV/0!
INC0201	0.09523809524	0.09523809524	0.05555555556		1.473684211	#DIV/0!
INC0201	0.04761904762	0.04761904762	0		inf	#DIV/0!
INC0201	0.04761904762	0.04761904762	0		inf	#DIV/0!
INC0202	0.09523809524	0.09523809524	0.05555555556		1.473684211	#DIV/0!
INC0202	0.09523809524	0.09523809524	0.05555555556		1.473684211	#DIV/0!
INC0202	0.04761904762	0.04761904762	0		inf	#DIV/0!
INC0202	0.09523809524	0.09523809524	0.05555555556		1.473684211	#DIV/0!
INC0202	0.04761904762	0.04761904762	0		inf	#DIV/0!
INC0202	0.09523809524	0.09523809524	0.05555555556		1.473684211	#DIV/0!
INC0202	0.04761904762	0.04761904762	0		inf	#DIV/0!
INC0202	0.09523809524	0.09523809524	0.05555555556		1.473684211	#DIV/0!
INC0202	0.1428571429	0.1428571429	0.05555555556		2.333333333	#DIV/0!
INC0203	0.1428571429	0.1428571429	0.1111111111		1.083333333	#DIV/0!
INC0203	0.04761904762	0.04761904762	0		inf	#DIV/0!
INC0203	0.09523809524	0.09523809524	0.05555555556		1.473684211	#DIV/0!
INC0203	0.09523809524	0.09523809524	0.05555555556		1.473684211	#DIV/0!
INC0203	0.09523809524	0.09523809524	0.05555555556		1.473684211	#DIV/0!
INC0203	0.09523809524	0.09523809524	0.05555555556		1.473684211	#DIV/0!
INC0203	0.04761904762	0.04761904762	0		inf	#DIV/0!
INC0204	0.09523809524	0.09523809524	0.05555555556		1.473684211	#DIV/0!
INC0204	0.09523809524	0.09523809524	0.05555555556		1.473684211	#DIV/0!
INC0204	0.09523809524	0.09523809524	0.05555555556		1.473684211	#DIV/0!
INC0204	0.09523809524	0.09523809524	0.05555555556		1.473684211	#DIV/0!
INC0204	0.1428571429	0.1428571429	0.1111111111		1.083333333	#DIV/0!
INC0204	0.09523809524	0.09523809524	0.05555555556		1.473684211	#DIV/0!
INC0205	0.04761904762	0.04761904762	0		inf	#DIV/0!
INC0205	0.09523809524	0.09523809524	0.05555555556		1.473684211	#DIV/0!
INC0205	0.1904761905	0.1904761905	0.05555555556		3.294117647	#DIV/0!
INC0205	0.1428571429	0.1428571429	0.05555555556		2.333333333	#DIV/0!
INC0205	0.04761904762	0.04761904762	0.1111111111		0.325	#DIV/0!
INC0205	0	0	0.05555555556		0	#DIV/0!
INC0205	0.04761904762	0.04761904762	0		inf	#DIV/0!
INC0205	0.04761904762	0.04761904762	0		inf	#DIV/0!

INC0206	0.04761904762	0.04761904762	0		inf	#DIV/0!
INC0206	0.04761904762	0.04761904762	0		inf	#DIV/0!
INC0206	0.04761904762	0.04761904762	0.05555555556		0.7	#DIV/0!
INC0206	0.04761904762	0.04761904762	0		inf	#DIV/0!
INC0206	0.1428571429	0.1428571429	0.05555555556		2.333333333	#DIV/0!
INC0206	0.1904761905	0.1904761905	0.1111111111		1.529411765	#DIV/0!
INC0206	0.1428571429	0.1428571429	0.05555555556		2.333333333	#DIV/0!
INC0207	0.04761904762	0.04761904762	0		inf	#DIV/0!
INC0207	0.04761904762	0.04761904762	0		inf	#DIV/0!
INC0207	0.04761904762	0.04761904762	0		inf	#DIV/0!
INC0207	0.04761904762	0.04761904762	0		inf	#DIV/0!
INC0207	0.09523809524	0.09523809524	0		inf	#DIV/0!
INC0207	0.04761904762	0.04761904762	0.1111111111		0.325	#DIV/0!
INC0207	0.04761904762	0.04761904762	0		inf	#DIV/0!
INC0207	0.04761904762	0.04761904762	0		inf	#DIV/0!
INC0207	0.09523809524	0.09523809524	0		inf	#DIV/0!
INC0208	0.04761904762	0.04761904762	0		inf	#DIV/0!
INC0208	0.1428571429	0.1428571429	0.05555555556		2.333333333	#DIV/0!
INC0208	0.09523809524	0.09523809524	0.05555555556		1.473684211	#DIV/0!
INC0208	0.04761904762	0.04761904762	0.05555555556		0.7	#DIV/0!
INC0208	0.09523809524	0.09523809524	0.05555555556		1.473684211	#DIV/0!
INC0208	0.04761904762	0.04761904762	0		inf	#DIV/0!
INC0209	0.04761904762	0.04761904762	0.05555555556		0.7	#DIV/0!
INC0209	0	0	0.1111111111		0	#DIV/0!
INC0209	0.04761904762	0.04761904762	0		inf	#DIV/0!
INC0209	0.04761904762	0.04761904762	0.1111111111		0.325	#DIV/0!
INC0209	0	0	0			#DIV/0!
INC0209	0.04761904762	0.04761904762	0.1111111111		0.325	#DIV/0!
INC0210	0.04761904762	0.04761904762	0		inf	#DIV/0!
INC0210	0	0	0.05555555556		0	#DIV/0!
INC0210	0.04761904762	0.04761904762	0.05555555556		0.7	#DIV/0!
INC0210	0.04761904762	0.04761904762	0.05555555556		0.7	#DIV/0!
INC0210	0.04761904762	0.04761904762	0.05555555556		0.7	#DIV/0!
INC0210	0.04761904762	0.04761904762	0.05555555556		0.7	#DIV/0!
INC0210	0.04761904762	0.04761904762	0.05555555556		0.7	#DIV/0!
INC0210	0.04761904762	0.04761904762	0		inf	#DIV/0!
INC0210	0.04761904762	0.04761904762	0.05555555556		0.7	#DIV/0!
INC0211	0.04761904762	0.04761904762	0.05555555556		0.7	#DIV/0!
INC0211	0.04761904762	0.04761904762	0.05555555556		0.7	#DIV/0!
INC0211	0	0	0.05555555556		0	#DIV/0!
INC0211	0.04761904762	0.04761904762	0		inf	#DIV/0!
INC0211	0	0	0.1111111111		0	#DIV/0!
INC0211	0.04761904762	0.04761904762	0		inf	#DIV/0!
INC0211	0	0	0.1111111111		0	#DIV/0!
INC0211	0.04761904762	0.04761904762	0.05555555556		0.7	#DIV/0!
INC0211	0.04761904762	0.04761904762	0.05555555556		0.7	#DIV/0!
INC0212	0.04761904762	0.04761904762	0.05555555556		0.7	#DIV/0!
INC0212	0	0	0.1111111111		0	#DIV/0!
INC0212	0	0	0.05555555556		0	#DIV/0!
INC0212	0.09523809524	0.09523809524	0.2222222222		0.2894736842	#DIV/0!
INC0212	0.04761904762	0.04761904762	0		inf	#DIV/0!

**INC0212**	0.04761904762	0.04761904762	0.05555555556		0.7	#DIV/0!
**INC0212**	0.04761904762	0.04761904762	0		inf	#DIV/0!
**INC0213**	0.04761904762	0.04761904762	0		inf	#DIV/0!
**INC0213**	0.04761904762	0.04761904762	0		inf	#DIV/0!
**INC0213**	0.04761904762	0.04761904762	0		inf	#DIV/0!
**INC0213**	0.04761904762	0.04761904762	0		inf	#DIV/0!
**INC0213**	0.04761904762	0.04761904762	0		inf	#DIV/0!
**INC0213**	0.09523809524	0.09523809524	0		inf	#DIV/0!
**INC0213**	0.04761904762	0.04761904762	0		inf	#DIV/0!
**INC0214**	0.04761904762	0.04761904762	0		inf	#DIV/0!
**INC0214**	0.04761904762	0.04761904762	0		inf	#DIV/0!
**INC0214**	0	0	0.05555555556		0	#DIV/0!
**INC0214**	0	0	0.1111111111		0	#DIV/0!
**INC0214**	0.04761904762	0.04761904762	0.1111111111		0.325	#DIV/0!
**INC0214**	0.04761904762	0.04761904762	0		inf	#DIV/0!
**INC0214**	0.04761904762	0.04761904762	0		inf	#DIV/0!
**INC0214**	0.04761904762	0.04761904762	0.05555555556		0.7	#DIV/0!
**INC0215**	0.04761904762	0.04761904762	0.05555555556		0.7	#DIV/0!
**INC0215**	0.09523809524	0.09523809524	0.1111111111		0.6842105263	#DIV/0!
**INC0215**	0.1428571429	0.1428571429	0.1666666667		0.6666666667	#DIV/0!
**INC0215**	0.04761904762	0.04761904762	0.05555555556		0.7	#DIV/0!
**INC0215**	0.04761904762	0.04761904762	0		inf	#DIV/0!
**INC0215**	0.04761904762	0.04761904762	0.05555555556		0.7	#DIV/0!
**INC0216**	0.04761904762	0.04761904762	0.1111111111		0.325	#DIV/0!
**INC0216**	0	0	0.1111111111		0	#DIV/0!
**INC0216**	0.04761904762	0.04761904762	0		inf	#DIV/0!
**INC0216**	0.04761904762	0.04761904762	0		inf	#DIV/0!
**INC0216**	0.04761904762	0.04761904762	0		inf	#DIV/0!
**INC0217**	0.04761904762	0.04761904762	0		inf	#DIV/0!
**INC0217**	0	0	0.05555555556		0	#DIV/0!
**INC0217**	0	0	0.05555555556		0	#DIV/0!
**INC0217**	0	0	0.05555555556		0	#DIV/0!
**INC0217**	0.04761904762	0.04761904762	0		inf	#DIV/0!
**INC0217**	0.04761904762	0.04761904762	0		inf	#DIV/0!
**INC0217**	0.04761904762	0.04761904762	0		inf	#DIV/0!
**INC0218**	0.04761904762	0.04761904762	0		inf	#DIV/0!
**INC0218**	0.04761904762	0.04761904762	0		inf	#DIV/0!
**INC0218**	0.09523809524	0.09523809524	0		inf	#DIV/0!
**INC0218**	0.04761904762	0.04761904762	0		inf	#DIV/0!
**INC0218**	0.04761904762	0.04761904762	0		inf	#DIV/0!
**INC0218**	0.04761904762	0.04761904762	0		inf	#DIV/0!
**INC0218**	0.04761904762	0.04761904762	0		inf	#DIV/0!
**INC0219**	0.04761904762	0.04761904762	0		inf	#DIV/0!
**INC0219**	0.04761904762	0.04761904762	0		inf	#DIV/0!
**INC0219**	0.09523809524	0.09523809524	0.1666666667		0.4210526316	#DIV/0!
**INC0219**	0.04761904762	0.04761904762	0.05555555556		0.7	#DIV/0!
**INC0219**	0	0	0.05555555556		0	#DIV/0!
**INC0220**	0.04761904762	0.04761904762	0		inf	#DIV/0!
**INC0220**	0.04761904762	0.04761904762	0		inf	#DIV/0!
**INC0220**	0.04761904762	0.04761904762	0.05555555556		0.7	#DIV/0!
**INC0220**	0.2857142857	0.2857142857	0.1111111111		2.6	#DIV/0!

**INC0220**	0.04761904762	0.04761904762	0.05555555556		0.7	#DIV/0!
**INC0220**	0.04761904762	0.04761904762	0.05555555556		0.7	#DIV/0!
**INC0220**	0.04761904762	0.04761904762	0.05555555556		0.7	#DIV/0!
**INC0220**	0.04761904762	0.04761904762	0.05555555556		0.7	#DIV/0!
**INC0220**	0.04761904762	0.04761904762	0		inf	#DIV/0!
**INC0220**	0.04761904762	0.04761904762	0.1111111111		0.325	#DIV/0!
**INC0221**	0.1428571429	0.1428571429	0		inf	#DIV/0!
**02210-C**	0.1428571429	0.1428571429	0		inf	#DIV/0!
**INC0221**	0.04761904762	0.04761904762	0.05555555556		0.7	#DIV/0!
**INC0221**	0.04761904762	0.04761904762	0.05555555556		0.7	#DIV/0!
**INC0221**	0.04761904762	0.04761904762	0.05555555556		0.7	#DIV/0!
**INC0221**	0.04761904762	0.04761904762	0		inf	#DIV/0!
**INC0221**	0.04761904762	0.04761904762	0		inf	#DIV/0!
**INC0221**	0.04761904762	0.04761904762	0.05555555556		0.7	#DIV/0!
**INC0221**	0.04761904762	0.04761904762	0.05555555556		0.7	#DIV/0!
**INC0221**	0.04761904762	0.04761904762	0		inf	#DIV/0!
**INC0222**	0.04761904762	0.04761904762	0.05555555556		0.7	#DIV/0!
**INC0222**	0	0	0.05555555556		0	#DIV/0!
**INC0222**	0.09523809524	0.09523809524	0.05555555556		1.473684211	#DIV/0!
**INC0222**	0.04761904762	0.04761904762	0.05555555556		0.7	#DIV/0!
**INC0222**	0.04761904762	0.04761904762	0.05555555556		0.7	#DIV/0!
**INC0222**	0.04761904762	0.04761904762	0		inf	#DIV/0!
**INC0222**	0	0	0.05555555556		0	#DIV/0!
**INC0222**	0.04761904762	0.04761904762	0.05555555556		0.7	#DIV/0!
**INC0222**	0.04761904762	0.04761904762	0.05555555556		0.7	#DIV/0!
**INC0222**	0.04761904762	0.04761904762	0		inf	#DIV/0!
**INC0223**	0	0	0.05555555556		0	#DIV/0!
**INC0223**	0.09523809524	0.09523809524	0.05555555556		1.473684211	#DIV/0!
**INC0223**	0.04761904762	0.04761904762	0.05555555556		0.7	#DIV/0!
**INC0223**	0	0	0.05555555556		0	#DIV/0!
**INC0223**	0.04761904762	0.04761904762	0		inf	#DIV/0!
**INC0223**	0	0	0.05555555556		0	#DIV/0!
**INC0223**	0.1904761905	0.1904761905	0.1111111111		1.529411765	#DIV/0!
**INC0223**	0.04761904762	0.04761904762	0.05555555556		0.7	#DIV/0!
**INC0223**	0.04761904762	0.04761904762	0.05555555556		0.7	#DIV/0!
**INC0224**	0.04761904762	0.04761904762	0		inf	#DIV/0!
**INC0224**	0.04761904762	0.04761904762	0.1111111111		0.325	#DIV/0!
**INC0224**	0.04761904762	0.04761904762	0		inf	#DIV/0!
**INC0224**	0	0	0.05555555556		0	#DIV/0!
**INC0224**	0.04761904762	0.04761904762	0.1111111111		0.325	#DIV/0!
**INC0224**	0.04761904762	0.04761904762	0		inf	#DIV/0!
**INC0225**	0.04761904762	0.04761904762	0.05555555556		0.7	#DIV/0!
**INC0225**	0.04761904762	0.04761904762	0.05555555556		0.7	#DIV/0!
**INC0225**	0.04761904762	0.04761904762	0.05555555556		0.7	#DIV/0!
**INC0225**	0.1904761905	0.1904761905	0.05555555556		3.294117647	#DIV/0!
**INC0225**	0.04761904762	0.04761904762	0.05555555556		0.7	#DIV/0!
**INC0225**	0.04761904762	0.04761904762	0.05555555556		0.7	#DIV/0!
**INC0226**	0.1904761905	0.1904761905	0.05555555556		3.294117647	#DIV/0!
**INC0226**	0	0	0.05555555556		0	#DIV/0!
**INC0226**	0	0	0.05555555556		0	#DIV/0!
**INC0226**	0	0	0.05555555556		0	#DIV/0!

INC0226	0.1904761905	0.1904761905	0.05555555556		3.294117647	#DIV/0!
INC0226	0	0	0.05555555556		0	#DIV/0!
INC0226	0	0	0.05555555556		0	#DIV/0!
INC0226	0	0	0.05555555556		0	#DIV/0!
INC0226	0	0	0.05555555556		0	#DIV/0!
INC0227	0	0	0			#DIV/0!
INC0227	0.04761904762	0.04761904762	0.05555555556		0.7	#DIV/0!
INC0227	0	0	0.05555555556		0	#DIV/0!
INC0227	0.09523809524	0.09523809524	0		inf	#DIV/0!
INC0227	0	0	0.05555555556		0	#DIV/0!
INC0227	0.04761904762	0.04761904762	0		inf	#DIV/0!
INC0228	0.2380952381	0.2380952381	0.05555555556		4.375	#DIV/0!
INC0229	0.1428571429	0.1428571429	0.1111111111		1.083333333	#DIV/0!
INC0230	0.04761904762	0.04761904762	0		inf	#DIV/0!
INC0230	0.04761904762	0.04761904762	0		inf	#DIV/0!
INC0231	0.04761904762	0.04761904762	0		inf	#DIV/0!
INC0231	0.04761904762	0.04761904762	0		inf	#DIV/0!
INC0233	0	0	0.05555555556		0	#DIV/0!
INC0234	0	0	0			#DIV/0!
INC0234	0.09523809524	0.09523809524	0.05555555556		1.473684211	#DIV/0!
INC0234	0.04761904762	0.04761904762	0.05555555556		0.7	#DIV/0!
INC0234	0.04761904762	0.04761904762	0.05555555556		0.7	#DIV/0!
INC0235	0.09523809524	0.09523809524	0.05555555556		1.473684211	#DIV/0!
INC0235	0.04761904762	0.04761904762	0.05555555556		0.7	#DIV/0!
INC0235	0	0	0.05555555556		0	#DIV/0!
INC0235	0.09523809524	0.09523809524	0.05555555556		1.473684211	#DIV/0!
INC0236	0	0	0			#DIV/0!
INC0236	0.09523809524	0.09523809524	0.05555555556		1.473684211	#DIV/0!
INC0236	0	0	0.05555555556		0	#DIV/0!
INC0236	0	0	0.05555555556		0	#DIV/0!
INC0236	0	0	0.05555555556		0	#DIV/0!
INC0236	0.04761904762	0.04761904762	0.1111111111		0.325	#DIV/0!
INC0236	0.04761904762	0.04761904762	0.1111111111		0.325	#DIV/0!
INC0236	0.09523809524	0.09523809524	0.05555555556		1.473684211	#DIV/0!
INC0236	0.09523809524	0.09523809524	0.05555555556		1.473684211	#DIV/0!
INC0237	0.09523809524	0.09523809524	0.05555555556		1.473684211	#DIV/0!
INC0237	0.09523809524	0.09523809524	0.05555555556		1.473684211	#DIV/0!
INC0237	0	0	0.05555555556		0	#DIV/0!
INC0237	0.09523809524	0.09523809524	0.05555555556		1.473684211	#DIV/0!
INC0237	0.09523809524	0.09523809524	0.05555555556		1.473684211	#DIV/0!
INC0237	0.04761904762	0.04761904762	0.1111111111		0.325	#DIV/0!
INC0238	0.1904761905	0.1904761905	0.05555555556		3.294117647	#DIV/0!
INC0238	0.04761904762	0.04761904762	0.05555555556		0.7	#DIV/0!
INC0238	0	0	0.05555555556		0	#DIV/0!
INC0238	0.04761904762	0.04761904762	0		inf	#DIV/0!
INC0238	0.09523809524	0.09523809524	0.05555555556		1.473684211	#DIV/0!
INC0238	0.1428571429	0.1428571429	0.1111111111		1.083333333	#DIV/0!
INC0238	0.09523809524	0.09523809524	0		inf	#DIV/0!
INC0238	0.09523809524	0.09523809524	0.05555555556		1.473684211	#DIV/0!
INC0239	0.04761904762	0.04761904762	0.05555555556		0.7	#DIV/0!
INC0239	0.09523809524	0.09523809524	0.05555555556		1.473684211	#DIV/0!

INC0239	0.04761904762	0.04761904762	0.05555555556		0.7	#DIV/0!
INC0239	0.04761904762	0.04761904762	0.05555555556		0.7	#DIV/0!
INC0239	0.04761904762	0.04761904762	0.05555555556		0.7	#DIV/0!
INC0239	0.04761904762	0.04761904762	0.1111111111		0.325	#DIV/0!
INC0239	0.04761904762	0.04761904762	0.05555555556		0.7	#DIV/0!
INC0240	0.04761904762	0.04761904762	0.1111111111		0.325	#DIV/0!
INC0240	0.04761904762	0.04761904762	0.05555555556		0.7	#DIV/0!
INC0240	0.04761904762	0.04761904762	0.05555555556		0.7	#DIV/0!
INC0240	0.09523809524	0.09523809524	0.05555555556		1.473684211	#DIV/0!
INC0240	0.09523809524	0.09523809524	0.05555555556		1.473684211	#DIV/0!
INC0240	0.09523809524	0.09523809524	0.05555555556		1.473684211	#DIV/0!
INC0241	0.04761904762	0.04761904762	0.05555555556		0.7	#DIV/0!
INC0241	0.09523809524	0.09523809524	0.05555555556		1.473684211	#DIV/0!
INC0241	0.04761904762	0.04761904762	0.05555555556		0.7	#DIV/0!
INC0241	0.09523809524	0.09523809524	0.05555555556		1.473684211	#DIV/0!
INC0241	0.09523809524	0.09523809524	0.05555555556		1.473684211	#DIV/0!
INC0241	0.04761904762	0.04761904762	0.1111111111		0.325	#DIV/0!
INC0241	0.09523809524	0.09523809524	0.05555555556		1.473684211	#DIV/0!
INC0241	0.09523809524	0.09523809524	0.05555555556		1.473684211	#DIV/0!
INC0242	0.09523809524	0.09523809524	0.05555555556		1.473684211	#DIV/0!
INC0242	0.09523809524	0.09523809524	0.05555555556		1.473684211	#DIV/0!
INC0242	0.1428571429	0.1428571429	0.1111111111		1.083333333	#DIV/0!
INC0242	0	0	0.05555555556		0	#DIV/0!
INC0242	0.04761904762	0.04761904762	0.05555555556		0.7	#DIV/0!
INC0242	0.09523809524	0.09523809524	0.05555555556		1.473684211	#DIV/0!
INC0242	0.04761904762	0.04761904762	0.05555555556		0.7	#DIV/0!
INC0242	0	0	0.05555555556		0	#DIV/0!
INC0242	0	0	0.05555555556		0	#DIV/0!
INC0242	0.09523809524	0.09523809524	0.05555555556		1.473684211	#DIV/0!
INC0243	0	0	0.05555555556		0	#DIV/0!
INC0243	0	0	0.05555555556		0	#DIV/0!
INC0243	0	0	0.05555555556		0	#DIV/0!
INC0243	0	0	0.05555555556		0	#DIV/0!
INC0243	0	0	0.05555555556		0	#DIV/0!
INC0244	0.09523809524	0.09523809524	0.05555555556		1.473684211	#DIV/0!
INC0244	0.04761904762	0.04761904762	0.1111111111		0.325	#DIV/0!
INC0244	0.04761904762	0.04761904762	0.05555555556		0.7	#DIV/0!
INC0244	0.04761904762	0.04761904762	0.1111111111		0.325	#DIV/0!
INC0244	0.04761904762	0.04761904762	0.1111111111		0.325	#DIV/0!
INC0245	0.04761904762	0.04761904762	0.05555555556		0.7	#DIV/0!
INC0245	0.04761904762	0.04761904762	0.05555555556		0.7	#DIV/0!
INC0245	0.04761904762	0.04761904762	0.05555555556		0.7	#DIV/0!
INC0245	0.09523809524	0.09523809524	0.05555555556		1.473684211	#DIV/0!
INC0245	0.04761904762	0.04761904762	0.05555555556		0.7	#DIV/0!
INC0245	0	0	0.05555555556		0	#DIV/0!
INC0246	0.04761904762	0.04761904762	0.05555555556		0.7	#DIV/0!
INC0246	0	0	0.05555555556		0	#DIV/0!
INC0246	0	0	0.05555555556		0	#DIV/0!
INC0246	0	0	0.05555555556		0	#DIV/0!
INC0246	0.04761904762	0.04761904762	0.1111111111		0.325	#DIV/0!
INC0247	0.04761904762	0.04761904762	0.1111111111		0.325	#DIV/0!

INC0247	0.04761904762	0.04761904762	0.05555555556		0.7	#DIV/0!
INC0247	0.1904761905	0.1904761905	0.05555555556		3.294117647	#DIV/0!
INC0247	0.04761904762	0.04761904762	0		inf	#DIV/0!
INC0247	0.04761904762	0.04761904762	0.05555555556		0.7	#DIV/0!
INC0247	0	0	0.05555555556		0	#DIV/0!
INC0247	0	0	0.05555555556		0	#DIV/0!
INC0248	0.2380952381	0.2380952381	0.05555555556		4.375	#DIV/0!
INC0248	0.04761904762	0.04761904762	0.05555555556		0.7	#DIV/0!
INC0248	0	0	0.05555555556		0	#DIV/0!
INC0248	0.04761904762	0.04761904762	0		inf	#DIV/0!
INC0249	0.04761904762	0.04761904762	0.05555555556		0.7	#DIV/0!
INC0249	0	0	0.05555555556		0	#DIV/0!
INC0249	0	0	0			#DIV/0!
INC0249	0.1428571429	0.1428571429	0.05555555556		2.333333333	#DIV/0!
INC0249	0.09523809524	0.09523809524	0.05555555556		1.473684211	#DIV/0!
INC0250	0	0	0.05555555556		0	#DIV/0!
INC0250	0	0	0.05555555556		0	#DIV/0!
INC0250	0	0	0.05555555556		0	#DIV/0!
INC0250	0	0	0.05555555556		0	#DIV/0!
INC0251	0	0	0.05555555556		0	#DIV/0!
INC0251	0	0	0.05555555556		0	#DIV/0!
INC0251	0	0	0.05555555556		0	#DIV/0!
INC0251	0	0	0.05555555556		0	#DIV/0!
INC0252	0.04761904762	0.04761904762	0.05555555556		0.7	#DIV/0!
INC0252	0	0	0			#DIV/0!
INC0253	0	0	0			#DIV/0!
INC0253	0.04761904762	0.04761904762	0.05555555556		0.7	#DIV/0!
INC0255	0.04761904762	0.04761904762	0.1111111111		0.325	#DIV/0!
INC0255	0	0	0.05555555556		0	#DIV/0!
INC0255	0.04761904762	0.04761904762	0.1111111111		0.325	#DIV/0!
INC0256	0.1428571429	0.1428571429	0.05555555556		2.333333333	#DIV/0!
INC0256	0	0	0.1666666667		0	#DIV/0!
INC0256	0.09523809524	0.09523809524	0.05555555556		1.473684211	#DIV/0!
INC0256	0.04761904762	0.04761904762	0		inf	#DIV/0!
INC0256	0.09523809524	0.09523809524	0		inf	#DIV/0!
INC0256	0.09523809524	0.09523809524	0		inf	#DIV/0!
INC0256	0.04761904762	0.04761904762	0.05555555556		0.7	#DIV/0!
INC0256	0.04761904762	0.04761904762	0.05555555556		0.7	#DIV/0!
INC0257	0	0	0.05555555556		0	#DIV/0!
INC0257	0.04761904762	0.04761904762	0.1111111111		0.325	#DIV/0!
INC0257	0.04761904762	0.04761904762	0.1111111111		0.325	#DIV/0!
INC0257	0.04761904762	0.04761904762	0.1111111111		0.325	#DIV/0!
INC0257	0.04761904762	0.04761904762	0		inf	#DIV/0!
INC0257	0	0	0.05555555556		0	#DIV/0!
INC0258	0	0	0.05555555556		0	#DIV/0!
INC0258	0	0	0.05555555556		0	#DIV/0!
INC0258	0.09523809524	0.09523809524	0		inf	#DIV/0!
INC0258	0.04761904762	0.04761904762	0		inf	#DIV/0!
INC0258	0.04761904762	0.04761904762	0.2222222222		0.1375	#DIV/0!
INC0259	0	0	0.1111111111		0	#DIV/0!
INC0260	0.04761904762	0.04761904762	0.05555555556		0.7	#DIV/0!

INC0260	0.04761904762	0.04761904762	0.1111111111		0.325	#DIV/0!
INC0260	0.1428571429	0.1428571429	0.1111111111		1.083333333	#DIV/0!
INC0260	0	0	0.05555555556		0	#DIV/0!
INC0260	0.04761904762	0.04761904762	0.05555555556		0.7	#DIV/0!
INC0260	0.09523809524	0.09523809524	0		inf	#DIV/0!
INC0260	0.04761904762	0.04761904762	0.05555555556		0.7	#DIV/0!
INC0261	0	0	0.05555555556		0	#DIV/0!
INC0261	0	0	0.05555555556		0	#DIV/0!
INC0261	0	0	0.05555555556		0	#DIV/0!
INC0261	0	0	0.05555555556		0	#DIV/0!
INC0261	0.04761904762	0.04761904762	0.05555555556		0.7	#DIV/0!
INC0261	0	0	0.05555555556		0	#DIV/0!
INC0261	0.04761904762	0.04761904762	0.1111111111		0.325	#DIV/0!
INC0261	0.1904761905	0.1904761905	0.05555555556		3.294117647	#DIV/0!
INC0261	0.09523809524	0.09523809524	0.05555555556		1.473684211	#DIV/0!
INC0262	0.1428571429	0.1428571429	0.05555555556		2.333333333	#DIV/0!
INC0262	0.09523809524	0.09523809524	0		inf	#DIV/0!
INC0262	0.1428571429	0.1428571429	0.05555555556		2.333333333	#DIV/0!
INC0262	0.1428571429	0.1428571429	0		inf	#DIV/0!
INC0262	0.1904761905	0.1904761905	0.05555555556		3.294117647	#DIV/0!
INC0262	0.1428571429	0.1428571429	0		inf	#DIV/0!
INC0263	0.04761904762	0.04761904762	0.1666666667		0.2	#DIV/0!
INC0264	0.09523809524	0.09523809524	0.1111111111		0.6842105263	#DIV/0!
INC0264	0.09523809524	0.09523809524	0.2222222222		0.2894736842	#DIV/0!
INC0264	0	0	0.1666666667		0	#DIV/0!
INC0264	0.1428571429	0.1428571429	0		inf	#DIV/0!
INC0264	0.09523809524	0.09523809524	0.1111111111		0.6842105263	#DIV/0!
INC0264	0.09523809524	0.09523809524	0.1111111111		0.6842105263	#DIV/0!
INC0265	0.1428571429	0.1428571429	0		inf	#DIV/0!
INC0265	0.09523809524	0.09523809524	0.1111111111		0.6842105263	#DIV/0!
INC0265	0.1428571429	0.1428571429	0.1111111111		1.083333333	#DIV/0!
INC0265	0.1428571429	0.1428571429	0		inf	#DIV/0!
INC0265	0.09523809524	0.09523809524	0.1111111111		0.6842105263	#DIV/0!
INC0265	0	0	0.1666666667		0	#DIV/0!
INC0265	0.1428571429	0.1428571429	0.05555555556		2.333333333	#DIV/0!
INC0266	0.09523809524	0.09523809524	0.2222222222		0.2894736842	#DIV/0!
INC0266	0.2857142857	0.2857142857	0		inf	#DIV/0!
INC0266	0.04761904762	0.04761904762	0.1666666667		0.2	#DIV/0!
INC0267	0.09523809524	0.09523809524	0.1111111111		0.6842105263	#DIV/0!
INC0267	0.1428571429	0.1428571429	0.05555555556		2.333333333	#DIV/0!
INC0267	0.09523809524	0.09523809524	0.1111111111		0.6842105263	#DIV/0!
INC0267	0.1428571429	0.1428571429	0.05555555556		2.333333333	#DIV/0!
INC0268	0.1428571429	0.1428571429	0		inf	#DIV/0!
INC0268	0.04761904762	0.04761904762	0		inf	#DIV/0!
INC0269	0.1428571429	0.1428571429	0.05555555556		2.333333333	#DIV/0!
INC219	0.04761904762	0.04761904762	0		inf	#DIV/0!
INCAD	0.04761904762	0.04761904762	0		inf	#DIV/0!
INCMD	0.04761904762	0.04761904762	0		inf	#DIV/0!
NCR-00	0.09523809524	0.09523809524	0.1111111111		0.6842105263	#DIV/0!
NCR-00	0.09523809524	0.09523809524	0.05555555556		1.473684211	#DIV/0!
NCR-00	0.04761904762	0.04761904762	0		inf	#DIV/0!

LINGO1	0.04761904762	0.04761904762	0.05555555556		0.7	#DIV/0!
NGO1-A	0.04761904762	0.04761904762	0.05555555556		0.7	#DIV/0!
NGO1-A	0.04761904762	0.04761904762	0.05555555556		0.7	#DIV/0!
LINGO2	0.09523809524	0.09523809524	0.05555555556		1.473684211	#DIV/0!
LINGO3	0.09523809524	0.09523809524	0.1111111111		0.6842105263	#DIV/0!
LINGO4	0.1904761905	0.1904761905	0		inf	#DIV/0!
LINP1	0.09523809524	0.09523809524	0.1111111111		0.6842105263	#DIV/0!
LINS1	0.04761904762	0.04761904762	0.05555555556		0.7	#DIV/0!
LIPA	0.1428571429	0.1428571429	0.1666666667		0.6666666667	#DIV/0!
LIPC	0.04761904762	0.04761904762	0.05555555556		0.7	#DIV/0!
IPC-AS	0.04761904762	0.04761904762	0.05555555556		0.7	#DIV/0!
LIPE	0.04761904762	0.04761904762	0.05555555556		0.7	#DIV/0!
IPE-AS	0.04761904762	0.04761904762	0.05555555556		0.7	#DIV/0!
LIPF	0.1428571429	0.1428571429	0.1666666667		0.6666666667	#DIV/0!
LIPG	0.04761904762	0.04761904762	0		inf	#DIV/0!
LIPH	0.1428571429	0.1428571429	0.05555555556		2.333333333	#DIV/0!
LIPI	0.04761904762	0.04761904762	0.1111111111		0.325	#DIV/0!
LIPJ	0.1428571429	0.1428571429	0.1666666667		0.6666666667	#DIV/0!
LIPK	0.1428571429	0.1428571429	0.1666666667		0.6666666667	#DIV/0!
LIPM	0.1428571429	0.1428571429	0.1666666667		0.6666666667	#DIV/0!
LIPN	0.1428571429	0.1428571429	0.1666666667		0.6666666667	#DIV/0!
LIPT1	0	0	0.05555555556		0	#DIV/0!
LITAF	0.04761904762	0.04761904762	0		inf	#DIV/0!
LIX1	0.04761904762	0.04761904762	0		inf	#DIV/0!
KAAEAI	0.1428571429	0.1428571429	0.2222222222		0.4583333333	#DIV/0!
NC01-81	0	0	0.1111111111		0	#DIV/0!
NC03-6	0.04761904762	0.04761904762	0.05555555556		0.7	#DIV/0!
LLCFC1	0.04761904762	0.04761904762	0.05555555556		0.7	#DIV/0!
LLPH	0.09523809524	0.09523809524	0.05555555556		1.473684211	#DIV/0!
LPH-DT	0.09523809524	0.09523809524	0.05555555556		1.473684211	#DIV/0!
LMAN1	0.09523809524	0.09523809524	0		inf	#DIV/0!
LMAN1I	0.04761904762	0.04761904762	0.05555555556		0.7	#DIV/0!
LMAN2	0.09523809524	0.09523809524	0.05555555556		1.473684211	#DIV/0!
LMAN2I	0	0	0.1111111111		0	#DIV/0!
LMBR1	0.1428571429	0.1428571429	0.05555555556		2.333333333	#DIV/0!
LMBR1I	0.04761904762	0.04761904762	0.05555555556		0.7	#DIV/0!
LMBRD	0.04761904762	0.04761904762	0.05555555556		0.7	#DIV/0!
LMCD1	0.04761904762	0.04761904762	0		inf	#DIV/0!
ICD1-A	0.04761904762	0.04761904762	0		inf	#DIV/0!
LMF1	0.04761904762	0.04761904762	0.2222222222		0.1375	#DIV/0!
MF1-AS	0.04761904762	0.04761904762	0.2222222222		0.1375	#DIV/0!
LMF2	0	0	0.1111111111		0	#DIV/0!
LMLN	0.1428571429	0.1428571429	0.05555555556		2.333333333	#DIV/0!
MLN-AS	0.1428571429	0.1428571429	0.05555555556		2.333333333	#DIV/0!
LMLN2	0	0	0.05555555556		0	#DIV/0!
LMNA	0.1428571429	0.1428571429	0.1111111111		1.083333333	#DIV/0!
LMNB1	0.04761904762	0.04761904762	0		inf	#DIV/0!
MNB1-D	0.04761904762	0.04761904762	0		inf	#DIV/0!
LMNB2	0.09523809524	0.09523809524	0.05555555556		1.473684211	#DIV/0!
LMNTD	0.04761904762	0.04761904762	0.05555555556		0.7	#DIV/0!
LMNTD	0.04761904762	0.04761904762	0		inf	#DIV/0!

**LMO3**	0.04761904762	0.04761904762	0.1111111111		0.325	#DIV/0!
**LMO4**	0.04761904762	0.04761904762	0.05555555556		0.7	#DIV/0!
**LMO7**	0	0	0.05555555556		0	#DIV/0!
**MO7-AS**	0	0	0.05555555556		0	#DIV/0!
**LMO7DN**	0	0	0.05555555556		0	#DIV/0!
**IO7DN-I**	0	0	0.05555555556		0	#DIV/0!
**LMOD1**	0.1428571429	0.1428571429	0		inf	#DIV/0!
**LMOD2**	0.04761904762	0.04761904762	0.05555555556		0.7	#DIV/0!
**LMOD3**	0.04761904762	0.04761904762	0		inf	#DIV/0!
**LMTK2**	0.04761904762	0.04761904762	0.05555555556		0.7	#DIV/0!
**LMTK3**	0.09523809524	0.09523809524	0.1111111111		0.6842105263	#DIV/0!
**LMX1A**	0.1428571429	0.1428571429	0		inf	#DIV/0!
**LMX1B**	0.09523809524	0.09523809524	0.05555555556		1.473684211	#DIV/0!
**NCARO**	0.04761904762	0.04761904762	0.05555555556		0.7	#DIV/0!
**LNCNEI**	0.1904761905	0.1904761905	0.05555555556		3.294117647	#DIV/0!
**LNCOC1**	0.04761904762	0.04761904762	0.1666666667		0.2	#DIV/0!
**LNCOG**	0.04761904762	0.04761904762	0.1111111111		0.325	#DIV/0!
**NCPRES**	0.04761904762	0.04761904762	0.05555555556		0.7	#DIV/0!
**NCPRES**	0	0	0.05555555556		0	#DIV/0!
**CRNA-A**	0.1428571429	0.1428571429	0.1111111111		1.083333333	#DIV/0!
**NCSRL**	0.1428571429	0.1428571429	0.1111111111		1.083333333	#DIV/0!
**CTAM3**	0	0	0.05555555556		0	#DIV/0!
**LNP1**	0.09523809524	0.09523809524	0.05555555556		1.473684211	#DIV/0!
**LNPEP**	0.04761904762	0.04761904762	0		inf	#DIV/0!
**LNPK**	0	0	0.1111111111		0	#DIV/0!
**LNX1**	0.2380952381	0.2380952381	0.05555555556		4.375	#DIV/0!
**NX1-AS**	0.2380952381	0.2380952381	0.05555555556		4.375	#DIV/0!
**NX1-AS**	0.2380952381	0.2380952381	0.05555555556		4.375	#DIV/0!
**C100049**	0.04761904762	0.04761904762	0.1111111111		0.325	#DIV/0!
**C100101**	0.1428571429	0.1428571429	0.1111111111		1.083333333	#DIV/0!
**C100128**	0.04761904762	0.04761904762	0.05555555556		0.7	#DIV/0!
**C100128**	0	0	0.05555555556		0	#DIV/0!
**C100128**	0.04761904762	0.04761904762	0.05555555556		0.7	#DIV/0!
**C100128**	0.04761904762	0.04761904762	0		inf	#DIV/0!
**C100128**	0.04761904762	0.04761904762	0.05555555556		0.7	#DIV/0!
**C100128**	0.1904761905	0.1904761905	0.05555555556		3.294117647	#DIV/0!
**C100128**	0.04761904762	0.04761904762	0.1666666667		0.2	#DIV/0!
**C100128**	0.04761904762	0.04761904762	0.05555555556		0.7	#DIV/0!
**C100128**	0.1428571429	0.1428571429	0.1111111111		1.083333333	#DIV/0!
**C100128**	0.04761904762	0.04761904762	0.1666666667		0.2	#DIV/0!
**C100128**	0.04761904762	0.04761904762	0.05555555556		0.7	#DIV/0!
**C100128**	0.09523809524	0.09523809524	0.05555555556		1.473684211	#DIV/0!
**C100128**	0.04761904762	0.04761904762	0.05555555556		0.7	#DIV/0!
**C100128**	0.1428571429	0.1428571429	0.05555555556		2.333333333	#DIV/0!
**C100128**	0.04761904762	0.04761904762	0.05555555556		0.7	#DIV/0!
**C100128**	0.1428571429	0.1428571429	0.05555555556		2.333333333	#DIV/0!
**C100128**	0.1428571429	0.1428571429	0.05555555556		2.333333333	#DIV/0!
**C100128**	0.04761904762	0.04761904762	0.1666666667		0.2	#DIV/0!
**C100128**	0.04761904762	0.04761904762	0.1111111111		0.325	#DIV/0!
**C100128**	0.1428571429	0.1428571429	0		inf	#DIV/0!
**C100128**	0.04761904762	0.04761904762	0.05555555556		0.7	#DIV/0!

**C100128**	0.04761904762	0.04761904762	0		inf	#DIV/0!
**C100128**	0.1428571429	0.1428571429	0.1666666667		0.6666666667	#DIV/0!
**C100128**	0.09523809524	0.09523809524	0.1111111111		0.6842105263	#DIV/0!
**C100129**	0.04761904762	0.04761904762	0.1111111111		0.325	#DIV/0!
**C100129**	0.09523809524	0.09523809524	0.05555555556		1.473684211	#DIV/0!
**C100129**	0.04761904762	0.04761904762	0.05555555556		0.7	#DIV/0!
**C100129**	0.09523809524	0.09523809524	0.1111111111		0.6842105263	#DIV/0!
**C100129**	0.09523809524	0.09523809524	0.1111111111		0.6842105263	#DIV/0!
**C100129**	0.04761904762	0.04761904762	0.05555555556		0.7	#DIV/0!
**C100129**	0.04761904762	0.04761904762	0.05555555556		0.7	#DIV/0!
**C100129**	0	0	0.05555555556		0	#DIV/0!
**C100129**	0.04761904762	0.04761904762	0.05555555556		0.7	#DIV/0!
**C100129**	0.04761904762	0.04761904762	0.05555555556		0.7	#DIV/0!
**C100129**	0.04761904762	0.04761904762	0.1666666667		0.2	#DIV/0!
**C100129**	0	0	0.1111111111		0	#DIV/0!
**C100129**	0.04761904762	0.04761904762	0.05555555556		0.7	#DIV/0!
**C100129**	0.09523809524	0.09523809524	0.1666666667		0.4210526316	#DIV/0!
**C100129**	0.04761904762	0.04761904762	0		inf	#DIV/0!
**C100129**	0.04761904762	0.04761904762	0.05555555556		0.7	#DIV/0!
**C100129**	0.1904761905	0.1904761905	0		inf	#DIV/0!
**C100129**	0.04761904762	0.04761904762	0		inf	#DIV/0!
**C100129**	0.04761904762	0.04761904762	0		inf	#DIV/0!
**C100129**	0.04761904762	0.04761904762	0.05555555556		0.7	#DIV/0!
**C100130**	0.09523809524	0.09523809524	0.2222222222		0.2894736842	#DIV/0!
**C100130**	0.09523809524	0.09523809524	0.1111111111		0.6842105263	#DIV/0!
**C100130**	0.04761904762	0.04761904762	0		inf	#DIV/0!
**C100130**	0.04761904762	0.04761904762	0		inf	#DIV/0!
**C100130**	0.09523809524	0.09523809524	0.05555555556		1.473684211	#DIV/0!
**C100130**	0.1904761905	0.1904761905	0.05555555556		3.294117647	#DIV/0!
**C100130**	0.04761904762	0.04761904762	0.1111111111		0.325	#DIV/0!
**C100130**	0.09523809524	0.09523809524	0		inf	#DIV/0!
**C100130**	0.1428571429	0.1428571429	0.1666666667		0.6666666667	#DIV/0!
**C100130**	0.1428571429	0.1428571429	0		inf	#DIV/0!
**C100130**	0.04761904762	0.04761904762	0		inf	#DIV/0!
**C100130**	0.09523809524	0.09523809524	0.1111111111		0.6842105263	#DIV/0!
**C100130**	0.04761904762	0.04761904762	0.05555555556		0.7	#DIV/0!
**C100130**	0	0	0.05555555556		0	#DIV/0!
**C100130**	0	0	0.05555555556		0	#DIV/0!
**C100130**	0.04761904762	0.04761904762	0		inf	#DIV/0!
**C100130**	0.04761904762	0.04761904762	0		inf	#DIV/0!
**C100130**	0.1428571429	0.1428571429	0.2222222222		0.4583333333	#DIV/0!
**C100130**	0.04761904762	0.04761904762	0.1666666667		0.2	#DIV/0!
**C100130**	0	0	0.1111111111		0	#DIV/0!
**C100130**	0.1428571429	0.1428571429	0		inf	#DIV/0!
**C100130**	0.04761904762	0.04761904762	0.05555555556		0.7	#DIV/0!
**C100130**	0.1428571429	0.1428571429	0.1666666667		0.6666666667	#DIV/0!
**C100130**	0.04761904762	0.04761904762	0		inf	#DIV/0!
**C100130**	0.04761904762	0.04761904762	0.05555555556		0.7	#DIV/0!
**C100130**	0	0	0.1111111111		0	#DIV/0!
**C100130**	0	0	0.1111111111		0	#DIV/0!
**C100130**	0.09523809524	0.09523809524	0.1111111111		0.6842105263	#DIV/0!

**C100130**	0.09523809524	0.09523809524	0.1111111111		0.6842105263	#DIV/0!
**C100131**	0.04761904762	0.04761904762	0.05555555556		0.7	#DIV/0!
**C100131**	0.1904761905	0.1904761905	0		inf	#DIV/0!
**C100131**	0.04761904762	0.04761904762	0.1666666667		0.2	#DIV/0!
**C100131**	0.04761904762	0.04761904762	0.2222222222		0.1375	#DIV/0!
**C100131**	0.1428571429	0.1428571429	0.1666666667		0.6666666667	#DIV/0!
**C100131**	0	0	0.05555555556		0	#DIV/0!
**C100131**	0.09523809524	0.09523809524	0.1111111111		0.6842105263	#DIV/0!
**C100132**	0.1428571429	0.1428571429	0.05555555556		2.333333333	#DIV/0!
**C100132**	0.09523809524	0.09523809524	0.1666666667		0.4210526316	#DIV/0!
**C100132**	0.04761904762	0.04761904762	0.05555555556		0.7	#DIV/0!
**C100132**	0	0	0.05555555556		0	#DIV/0!
**C100132**	0.09523809524	0.09523809524	0.2222222222		0.2894736842	#DIV/0!
**C100132**	0.09523809524	0.09523809524	0.1666666667		0.4210526316	#DIV/0!
**C100132**	0.04761904762	0.04761904762	0.05555555556		0.7	#DIV/0!
**C100132**	0.04761904762	0.04761904762	0		inf	#DIV/0!
**C100132**	0.1904761905	0.1904761905	0.1111111111		1.529411765	#DIV/0!
**C100133**	0.04761904762	0.04761904762	0		inf	#DIV/0!
**C100133**	0.09523809524	0.09523809524	0		inf	#DIV/0!
**C100133**	0.09523809524	0.09523809524	0.1111111111		0.6842105263	#DIV/0!
**C100133**	0.04761904762	0.04761904762	0.1666666667		0.2	#DIV/0!
**C100133**	0.04761904762	0.04761904762	0.1666666667		0.2	#DIV/0!
**C100133**	0.09523809524	0.09523809524	0.2222222222		0.2894736842	#DIV/0!
**C100134**	0.09523809524	0.09523809524	0.05555555556		1.473684211	#DIV/0!
**C100134**	0.04761904762	0.04761904762	0.05555555556		0.7	#DIV/0!
**C100134**	0.04761904762	0.04761904762	0.2222222222		0.1375	#DIV/0!
**C100134**	0.04761904762	0.04761904762	0		inf	#DIV/0!
**C100134**	0.1904761905	0.1904761905	0.05555555556		3.294117647	#DIV/0!
**C100144**	0	0	0.05555555556		0	#DIV/0!
**C100190**	0.04761904762	0.04761904762	0		inf	#DIV/0!
**C100192**	0.09523809524	0.09523809524	0		inf	#DIV/0!
**C100240**	0.1428571429	0.1428571429	0.1666666667		0.6666666667	#DIV/0!
**C100240**	0.04761904762	0.04761904762	0.05555555556		0.7	#DIV/0!
**C100240**	0.04761904762	0.04761904762	0.05555555556		0.7	#DIV/0!
**C100268**	0	0	0.05555555556		0	#DIV/0!
**C100272**	0.09523809524	0.09523809524	0.05555555556		1.473684211	#DIV/0!
**C100286**	0.1428571429	0.1428571429	0.05555555556		2.333333333	#DIV/0!
**C100286**	0	0	0.05555555556		0	#DIV/0!
**C100286**	0.04761904762	0.04761904762	0.05555555556		0.7	#DIV/0!
**C100287**	0	0	0.05555555556		0	#DIV/0!
**C100287**	0.09523809524	0.09523809524	0.1111111111		0.6842105263	#DIV/0!
**C100287**	0.09523809524	0.09523809524	0		inf	#DIV/0!
**C100287**	0.04761904762	0.04761904762	0.05555555556		0.7	#DIV/0!
**C100287**	0.1428571429	0.1428571429	0		inf	#DIV/0!
**C100287**	0.09523809524	0.09523809524	0.05555555556		1.473684211	#DIV/0!
**C100287**	0.04761904762	0.04761904762	0.2222222222		0.1375	#DIV/0!
**C100287**	0.04761904762	0.04761904762	0		inf	#DIV/0!
**C100287**	0	0	0.05555555556		0	#DIV/0!
**C100287**	0.1428571429	0.1428571429	0.05555555556		2.333333333	#DIV/0!
**C100287**	0.09523809524	0.09523809524	0.05555555556		1.473684211	#DIV/0!
**C100287**	0.1428571429	0.1428571429	0.1666666667		0.6666666667	#DIV/0!

**C100287**	0.04761904762	0.04761904762	0		inf	#DIV/0!
**C100287**	0.09523809524	0.09523809524	0.05555555556		1.473684211	#DIV/0!
**C100287**	0.09523809524	0.09523809524	0.1666666667		0.4210526316	#DIV/0!
**C100287**	0.04761904762	0.04761904762	0.05555555556		0.7	#DIV/0!
**C100288**	0.04761904762	0.04761904762	0.1666666667		0.2	#DIV/0!
**C100288**	0.09523809524	0.09523809524	0.1111111111		0.6842105263	#DIV/0!
**C100288**	0.09523809524	0.09523809524	0		inf	#DIV/0!
**C100288**	0	0	0.1111111111		0	#DIV/0!
**C100288**	0	0	0.05555555556		0	#DIV/0!
**C100288**	0	0	0.05555555556		0	#DIV/0!
**C100288**	0	0	0.1111111111		0	#DIV/0!
**C100288**	0.1904761905	0.1904761905	0.1111111111		1.529411765	#DIV/0!
**C100288**	0.04761904762	0.04761904762	0.05555555556		0.7	#DIV/0!
**C100288**	0.04761904762	0.04761904762	0		inf	#DIV/0!
**C100288**	0.09523809524	0.09523809524	0		inf	#DIV/0!
**C100289**	0.04761904762	0.04761904762	0		inf	#DIV/0!
**C100289**	0.1428571429	0.1428571429	0.1111111111		1.083333333	#DIV/0!
**C100289**	0.1428571429	0.1428571429	0.05555555556		2.333333333	#DIV/0!
**C100289**	0.1904761905	0.1904761905	0.1111111111		1.529411765	#DIV/0!
**C100289**	0.04761904762	0.04761904762	0.05555555556		0.7	#DIV/0!
**C100289**	0.1904761905	0.1904761905	0		inf	#DIV/0!
**C100291**	0.1428571429	0.1428571429	0.2222222222		0.4583333333	#DIV/0!
**C100294**	0.04761904762	0.04761904762	0		inf	#DIV/0!
**C100294**	0.04761904762	0.04761904762	0		inf	#DIV/0!
**C100303**	0.04761904762	0.04761904762	0		inf	#DIV/0!
**C100310**	0.04761904762	0.04761904762	0.1666666667		0.2	#DIV/0!
**C100335**	0.04761904762	0.04761904762	0.05555555556		0.7	#DIV/0!
**C100379**	0.09523809524	0.09523809524	0.05555555556		1.473684211	#DIV/0!
**C100419**	0	0	0.05555555556		0	#DIV/0!
**C100420**	0.09523809524	0.09523809524	0.1111111111		0.6842105263	#DIV/0!
**C100422**	0.1428571429	0.1428571429	0		inf	#DIV/0!
**C100422**	0.04761904762	0.04761904762	0.05555555556		0.7	#DIV/0!
**C100499**	0	0	0.05555555556		0	#DIV/0!
**C100499**	0.09523809524	0.09523809524	0.1111111111		0.6842105263	#DIV/0!
**C100500**	0.1904761905	0.1904761905	0.1666666667		0.9411764706	#DIV/0!
**C100505**	0.1428571429	0.1428571429	0.1666666667		0.6666666667	#DIV/0!
**C100505**	0.09523809524	0.09523809524	0.2222222222		0.2894736842	#DIV/0!
**C100505**	0.09523809524	0.09523809524	0		inf	#DIV/0!
**C100505**	0.1428571429	0.1428571429	0.1111111111		1.083333333	#DIV/0!
**C100505**	0.04761904762	0.04761904762	0.05555555556		0.7	#DIV/0!
**C100505**	0.09523809524	0.09523809524	0.05555555556		1.473684211	#DIV/0!
**C100505**	0.04761904762	0.04761904762	0.05555555556		0.7	#DIV/0!
**C100505**	0	0	0.05555555556		0	#DIV/0!
**C100505**	0.04761904762	0.04761904762	0		inf	#DIV/0!
**C100505**	0.09523809524	0.09523809524	0.05555555556		1.473684211	#DIV/0!
**C100505**	0	0	0.1111111111		0	#DIV/0!
**C100505**	0	0	0.1111111111		0	#DIV/0!
**C100505**	0	0	0.05555555556		0	#DIV/0!
**C100505**	0.09523809524	0.09523809524	0		inf	#DIV/0!
**C100505**	0.1428571429	0.1428571429	0		inf	#DIV/0!
**C100505**	0.1428571429	0.1428571429	0.05555555556		2.333333333	#DIV/0!

**C100505**	0.04761904762	0.04761904762	0		inf	#DIV/0!
**C100505**	0.09523809524	0.09523809524	0		inf	#DIV/0!
**C100505**	0.04761904762	0.04761904762	0		inf	#DIV/0!
**C100505**	0.1428571429	0.1428571429	0		inf	#DIV/0!
**C100505**	0.04761904762	0.04761904762	0.1666666667		0.2	#DIV/0!
**C100505**	0.1428571429	0.1428571429	0.05555555556		2.333333333	#DIV/0!
**C100505**	0.04761904762	0.04761904762	0.05555555556		0.7	#DIV/0!
**C100506**	0.1428571429	0.1428571429	0		inf	#DIV/0!
**C100506**	0	0	0			#DIV/0!
**C100506**	0	0	0.1111111111		0	#DIV/0!
**C100506**	0.04761904762	0.04761904762	0		inf	#DIV/0!
**C100506**	0.1428571429	0.1428571429	0.05555555556		2.333333333	#DIV/0!
**C100506**	0.04761904762	0.04761904762	0.2222222222		0.1375	#DIV/0!
**C100506**	0.09523809524	0.09523809524	0.05555555556		1.473684211	#DIV/0!
**C100506**	0	0	0.1111111111		0	#DIV/0!
**C100506**	0	0	0.05555555556		0	#DIV/0!
**C100506**	0.04761904762	0.04761904762	0.05555555556		0.7	#DIV/0!
**C100506**	0	0	0.05555555556		0	#DIV/0!
**C100506**	0.09523809524	0.09523809524	0.1666666667		0.4210526316	#DIV/0!
**C100506**	0.04761904762	0.04761904762	0.1666666667		0.2	#DIV/0!
**C100506**	0.04761904762	0.04761904762	0.05555555556		0.7	#DIV/0!
**C100506**	0.04761904762	0.04761904762	0		inf	#DIV/0!
**C100506**	0	0	0.1111111111		0	#DIV/0!
**C100506**	0	0	0.05555555556		0	#DIV/0!
**C100506**	0.04761904762	0.04761904762	0		inf	#DIV/0!
**C100506**	0.1428571429	0.1428571429	0.05555555556		2.333333333	#DIV/0!
**C100506**	0.1428571429	0.1428571429	0.1666666667		0.6666666667	#DIV/0!
**C100506**	0	0	0.1111111111		0	#DIV/0!
**C100506**	0.04761904762	0.04761904762	0.1666666667		0.2	#DIV/0!
**C100506**	0.04761904762	0.04761904762	0.05555555556		0.7	#DIV/0!
**C100506**	0.04761904762	0.04761904762	0.05555555556		0.7	#DIV/0!
**C100506**	0.2380952381	0.2380952381	0.05555555556		4.375	#DIV/0!
**C100506**	0	0	0.1111111111		0	#DIV/0!
**C100506**	0	0	0.05555555556		0	#DIV/0!
**C100506**	0.09523809524	0.09523809524	0		inf	#DIV/0!
**C100506**	0.04761904762	0.04761904762	0.2222222222		0.1375	#DIV/0!
**C100506**	0	0	0.1111111111		0	#DIV/0!
**C100506**	0	0	0.05555555556		0	#DIV/0!
**C100506**	0.1428571429	0.1428571429	0		inf	#DIV/0!
**C100506**	0.1428571429	0.1428571429	0.05555555556		2.333333333	#DIV/0!
**C100506**	0.04761904762	0.04761904762	0.05555555556		0.7	#DIV/0!
**C100506**	0.04761904762	0.04761904762	0.05555555556		0.7	#DIV/0!
**C100506**	0	0	0.1666666667		0	#DIV/0!
**C100506**	0.04761904762	0.04761904762	0.05555555556		0.7	#DIV/0!
**C100506**	0	0	0.05555555556		0	#DIV/0!
**C100506**	0.04761904762	0.04761904762	0.1111111111		0.325	#DIV/0!
**C100506**	0.04761904762	0.04761904762	0.2222222222		0.1375	#DIV/0!
**C100506**	0.04761904762	0.04761904762	0.1111111111		0.325	#DIV/0!
**C100506**	0.1428571429	0.1428571429	0		inf	#DIV/0!
**C100506**	0.04761904762	0.04761904762	0.1111111111		0.325	#DIV/0!
**C100506**	0	0	0.1111111111		0	#DIV/0!

**C100506**	0.09523809524	0.09523809524	0		inf	#DIV/0!
**C100506**	0.04761904762	0.04761904762	0.05555555556		0.7	#DIV/0!
**C100506**	0.04761904762	0.04761904762	0.1111111111		0.325	#DIV/0!
**C100506**	0.09523809524	0.09523809524	0.1111111111		0.6842105263	#DIV/0!
**C100507**	0.04761904762	0.04761904762	0		inf	#DIV/0!
**C100507**	0	0	0.05555555556		0	#DIV/0!
**C100507**	0	0	0.05555555556		0	#DIV/0!
**C100507**	0.09523809524	0.09523809524	0.05555555556		1.473684211	#DIV/0!
**C100507**	0.09523809524	0.09523809524	0.1111111111		0.6842105263	#DIV/0!
**C100507**	0.04761904762	0.04761904762	0.1111111111		0.325	#DIV/0!
**C100507**	0.09523809524	0.09523809524	0.05555555556		1.473684211	#DIV/0!
**C100507**	0.1428571429	0.1428571429	0.05555555556		2.333333333	#DIV/0!
**C100507**	0	0	0.05555555556		0	#DIV/0!
**C100507**	0.04761904762	0.04761904762	0.05555555556		0.7	#DIV/0!
**C100507**	0.04761904762	0.04761904762	0		inf	#DIV/0!
**C100507**	0.1428571429	0.1428571429	0.1111111111		1.083333333	#DIV/0!
**C100507**	0.04761904762	0.04761904762	0.05555555556		0.7	#DIV/0!
**C100507**	0.04761904762	0.04761904762	0.05555555556		0.7	#DIV/0!
**C100507**	0.09523809524	0.09523809524	0.05555555556		1.473684211	#DIV/0!
**C100507**	0.1428571429	0.1428571429	0.05555555556		2.333333333	#DIV/0!
**C100507**	0	0	0.05555555556		0	#DIV/0!
**C100507**	0.09523809524	0.09523809524	0.1111111111		0.6842105263	#DIV/0!
**C100507**	0.04761904762	0.04761904762	0.05555555556		0.7	#DIV/0!
**C100507**	0.04761904762	0.04761904762	0.05555555556		0.7	#DIV/0!
**C100507**	0.09523809524	0.09523809524	0.1111111111		0.6842105263	#DIV/0!
**C100507**	0.04761904762	0.04761904762	0		inf	#DIV/0!
**C100507**	0.04761904762	0.04761904762	0.05555555556		0.7	#DIV/0!
**C100507**	0.04761904762	0.04761904762	0.05555555556		0.7	#DIV/0!
**C100507**	0.04761904762	0.04761904762	0.05555555556		0.7	#DIV/0!
**C100507**	0.04761904762	0.04761904762	0.05555555556		0.7	#DIV/0!
**C100507**	0.04761904762	0.04761904762	0.1666666667		0.2	#DIV/0!
**C100508**	0.1428571429	0.1428571429	0.1111111111		1.083333333	#DIV/0!
**C100630**	0.04761904762	0.04761904762	0.05555555556		0.7	#DIV/0!
**C100631**	0.04761904762	0.04761904762	0.05555555556		0.7	#DIV/0!
**C100652**	0.04761904762	0.04761904762	0.05555555556		0.7	#DIV/0!
**C100996**	0.04761904762	0.04761904762	0.05555555556		0.7	#DIV/0!
**C100996**	0.04761904762	0.04761904762	0.05555555556		0.7	#DIV/0!
**C100996**	0.04761904762	0.04761904762	0.05555555556		0.7	#DIV/0!
**C100996**	0.04761904762	0.04761904762	0.1111111111		0.325	#DIV/0!
**C100996**	0	0	0.05555555556		0	#DIV/0!
**C100996**	0.09523809524	0.09523809524	0		inf	#DIV/0!
**C100996**	0.09523809524	0.09523809524	0.05555555556		1.473684211	#DIV/0!
**C100996**	0.04761904762	0.04761904762	0.05555555556		0.7	#DIV/0!
**C100996**	0.09523809524	0.09523809524	0.05555555556		1.473684211	#DIV/0!
**C100996**	0.04761904762	0.04761904762	0.1111111111		0.325	#DIV/0!
**C100996**	0.1428571429	0.1428571429	0.05555555556		2.333333333	#DIV/0!
**C100996**	0	0	0.1111111111		0	#DIV/0!
**C100996**	0.09523809524	0.09523809524	0.2222222222		0.2894736842	#DIV/0!
**C100996**	0.380952381	0.380952381	0.2222222222		1.692307692	#DIV/0!
**C100996**	0	0	0.1666666667		0	#DIV/0!
**C100996**	0.09523809524	0.09523809524	0.05555555556		1.473684211	#DIV/0!

**C100996**	0.09523809524	0.09523809524	0.05555555556		1.473684211	#DIV/0!
**C100996**	0.09523809524	0.09523809524	0.05555555556		1.473684211	#DIV/0!
**C100996**	0.1428571429	0.1428571429	0.05555555556		2.333333333	#DIV/0!
**C100996**	0.09523809524	0.09523809524	0		inf	#DIV/0!
**C100996**	0	0	0.05555555556		0	#DIV/0!
**C101059**	0.09523809524	0.09523809524	0.1111111111		0.6842105263	#DIV/0!
**C101060**	0.1428571429	0.1428571429	0.05555555556		2.333333333	#DIV/0!
**C101241**	0.1428571429	0.1428571429	0.1111111111		1.083333333	#DIV/0!
**C101409**	0.09523809524	0.09523809524	0.1111111111		0.6842105263	#DIV/0!
**C101448**	0.04761904762	0.04761904762	0		inf	#DIV/0!
**C101559**	0	0	0.1111111111		0	#DIV/0!
**C101593**	0.04761904762	0.04761904762	0.1111111111		0.325	#DIV/0!
**C101926**	0.1904761905	0.1904761905	0.05555555556		3.294117647	#DIV/0!
**C101926**	0.1904761905	0.1904761905	0.1666666667		0.9411764706	#DIV/0!
**C101926**	0.04761904762	0.04761904762	0.05555555556		0.7	#DIV/0!
**C101926**	0.04761904762	0.04761904762	0		inf	#DIV/0!
**C101926**	0.1904761905	0.1904761905	0.1666666667		0.9411764706	#DIV/0!
**C101926**	0	0	0.05555555556		0	#DIV/0!
**C101926**	0.04761904762	0.04761904762	0.05555555556		0.7	#DIV/0!
**C101926**	0	0	0.05555555556		0	#DIV/0!
**C101926**	0.04761904762	0.04761904762	0.05555555556		0.7	#DIV/0!
**C101926**	0.1904761905	0.1904761905	0.05555555556		3.294117647	#DIV/0!
**C101926**	0.1428571429	0.1428571429	0.1666666667		0.6666666667	#DIV/0!
**C101926**	0.04761904762	0.04761904762	0.05555555556		0.7	#DIV/0!
**C101926**	0.09523809524	0.09523809524	0.05555555556		1.473684211	#DIV/0!
**C101926**	0.04761904762	0.04761904762	0.05555555556		0.7	#DIV/0!
**C101926**	0.04761904762	0.04761904762	0.05555555556		0.7	#DIV/0!
**C101926**	0.04761904762	0.04761904762	0.1666666667		0.2	#DIV/0!
**C101926**	0.04761904762	0.04761904762	0.1111111111		0.325	#DIV/0!
**C101926**	0.09523809524	0.09523809524	0.05555555556		1.473684211	#DIV/0!
**C101927**	0.04761904762	0.04761904762	0.1666666667		0.2	#DIV/0!
**C101927**	0.04761904762	0.04761904762	0.05555555556		0.7	#DIV/0!
**C101927**	0.04761904762	0.04761904762	0		inf	#DIV/0!
**C101927**	0.04761904762	0.04761904762	0.1666666667		0.2	#DIV/0!
**C101927**	0.04761904762	0.04761904762	0		inf	#DIV/0!
**C101927**	0.1428571429	0.1428571429	0.1111111111		1.083333333	#DIV/0!
**C101927**	0.04761904762	0.04761904762	0		inf	#DIV/0!
**C101927**	0.04761904762	0.04761904762	0.05555555556		0.7	#DIV/0!
**C101927**	0	0	0.1111111111		0	#DIV/0!
**C101927**	0	0	0.1111111111		0	#DIV/0!
**C101927**	0	0	0.1111111111		0	#DIV/0!
**C101927**	0.1904761905	0.1904761905	0.1666666667		0.9411764706	#DIV/0!
**C101927**	0	0	0.05555555556		0	#DIV/0!
**C101927**	0.04761904762	0.04761904762	0		inf	#DIV/0!
**C101927**	0.2380952381	0.2380952381	0.1111111111		2.03125	#DIV/0!
**C101927**	0.04761904762	0.04761904762	0		inf	#DIV/0!
**C101927**	0.04761904762	0.04761904762	0		inf	#DIV/0!
**C101927**	0	0	0			#DIV/0!
**C101927**	0.04761904762	0.04761904762	0		inf	#DIV/0!
**C101927**	0.04761904762	0.04761904762	0		inf	#DIV/0!
**C101927**	0.1428571429	0.1428571429	0.1111111111		1.083333333	#DIV/0!

C101927	0.04761904762	0.04761904762	0.05555555556		0.7	#DIV/0!
C101927	0.1428571429	0.1428571429	0.1111111111		1.083333333	#DIV/0!
C101927	0	0	0.05555555556		0	#DIV/0!
C101927	0.1428571429	0.1428571429	0.05555555556		2.333333333	#DIV/0!
C101927	0.09523809524	0.09523809524	0.05555555556		1.473684211	#DIV/0!
C101927	0.04761904762	0.04761904762	0.1111111111		0.325	#DIV/0!
C101927	0.1428571429	0.1428571429	0.1111111111		1.083333333	#DIV/0!
C101927	0.1428571429	0.1428571429	0.05555555556		2.333333333	#DIV/0!
C101927	0.1428571429	0.1428571429	0		inf	#DIV/0!
C101927	0.09523809524	0.09523809524	0		inf	#DIV/0!
C101927	0.09523809524	0.09523809524	0		inf	#DIV/0!
C101927	0.04761904762	0.04761904762	0.1111111111		0.325	#DIV/0!
C101927	0.09523809524	0.09523809524	0		inf	#DIV/0!
C101927	0.04761904762	0.04761904762	0		inf	#DIV/0!
C101927	0.04761904762	0.04761904762	0		inf	#DIV/0!
C101927	0.04761904762	0.04761904762	0.05555555556		0.7	#DIV/0!
C101927	0.04761904762	0.04761904762	0.1111111111		0.325	#DIV/0!
C101927	0.1904761905	0.1904761905	0.1111111111		1.529411765	#DIV/0!
C101927	0.04761904762	0.04761904762	0.05555555556		0.7	#DIV/0!
C101927	0.04761904762	0.04761904762	0.1111111111		0.325	#DIV/0!
C101927	0.04761904762	0.04761904762	0		inf	#DIV/0!
C101927	0.1428571429	0.1428571429	0		inf	#DIV/0!
C101927	0.04761904762	0.04761904762	0		inf	#DIV/0!
C101927	0.04761904762	0.04761904762	0.05555555556		0.7	#DIV/0!
C101927	0.04761904762	0.04761904762	0.05555555556		0.7	#DIV/0!
C101927	0.09523809524	0.09523809524	0		inf	#DIV/0!
C101927	0.04761904762	0.04761904762	0.05555555556		0.7	#DIV/0!
C101927	0.04761904762	0.04761904762	0.1666666667		0.2	#DIV/0!
C101927	0.04761904762	0.04761904762	0.1666666667		0.2	#DIV/0!
C101927	0.04761904762	0.04761904762	0		inf	#DIV/0!
C101927	0	0	0.05555555556		0	#DIV/0!
C101927	0	0	0.05555555556		0	#DIV/0!
C101927	0.04761904762	0.04761904762	0		inf	#DIV/0!
C101927	0.1428571429	0.1428571429	0.1666666667		0.6666666667	#DIV/0!
C101927	0.04761904762	0.04761904762	0.1111111111		0.325	#DIV/0!
C101927	0.04761904762	0.04761904762	0.1666666667		0.2	#DIV/0!
C101927	0.04761904762	0.04761904762	0		inf	#DIV/0!
C101927	0	0	0.05555555556		0	#DIV/0!
C101927	0.09523809524	0.09523809524	0		inf	#DIV/0!
C101927	0.04761904762	0.04761904762	0.1111111111		0.325	#DIV/0!
C101927	0.04761904762	0.04761904762	0.1111111111		0.325	#DIV/0!
C101927	0.04761904762	0.04761904762	0		inf	#DIV/0!
C101927	0.09523809524	0.09523809524	0.05555555556		1.473684211	#DIV/0!
C101927	0.04761904762	0.04761904762	0		inf	#DIV/0!
C101927	0.1428571429	0.1428571429	0.05555555556		2.333333333	#DIV/0!
C101927	0.04761904762	0.04761904762	0.05555555556		0.7	#DIV/0!
C101927	0.09523809524	0.09523809524	0.1111111111		0.6842105263	#DIV/0!
C101927	0	0	0.1666666667		0	#DIV/0!
C101927	0	0	0.05555555556		0	#DIV/0!
C101927	0.1428571429	0.1428571429	0.05555555556		2.333333333	#DIV/0!
C101927	0.1428571429	0.1428571429	0		inf	#DIV/0!

**C101927**	0.04761904762	0.04761904762	0		inf	#DIV/0!
**C101927**	0.04761904762	0.04761904762	0		inf	#DIV/0!
**C101927**	0.04761904762	0.04761904762	0.1111111111		0.325	#DIV/0!
**C101927**	0	0	0.05555555556		0	#DIV/0!
**C101927**	0.09523809524	0.09523809524	0.1666666667		0.4210526316	#DIV/0!
**C101927**	0	0	0.05555555556		0	#DIV/0!
**C101927**	0.09523809524	0.09523809524	0		inf	#DIV/0!
**C101927**	0.09523809524	0.09523809524	0.1111111111		0.6842105263	#DIV/0!
**C101927**	0.04761904762	0.04761904762	0.05555555556		0.7	#DIV/0!
**C101927**	0	0	0.1666666667		0	#DIV/0!
**C101927**	0.09523809524	0.09523809524	0		inf	#DIV/0!
**C101927**	0.04761904762	0.04761904762	0.05555555556		0.7	#DIV/0!
**C101927**	0.04761904762	0.04761904762	0.05555555556		0.7	#DIV/0!
**C101927**	0.04761904762	0.04761904762	0.05555555556		0.7	#DIV/0!
**C101927**	0	0	0.05555555556		0	#DIV/0!
**C101927**	0.1428571429	0.1428571429	0.05555555556		2.333333333	#DIV/0!
**C101927**	0.1428571429	0.1428571429	0.1111111111		1.083333333	#DIV/0!
**C101927**	0	0	0.05555555556		0	#DIV/0!
**C101927**	0.1428571429	0.1428571429	0		inf	#DIV/0!
**C101927**	0.04761904762	0.04761904762	0		inf	#DIV/0!
**C101927**	0	0	0.05555555556		0	#DIV/0!
**C101927**	0.04761904762	0.04761904762	0.2222222222		0.1375	#DIV/0!
**C101927**	0	0	0.05555555556		0	#DIV/0!
**C101927**	0.1428571429	0.1428571429	0.1111111111		1.083333333	#DIV/0!
**C101927**	0	0	0.05555555556		0	#DIV/0!
**C101927**	0.09523809524	0.09523809524	0		inf	#DIV/0!
**C101927**	0.04761904762	0.04761904762	0.2222222222		0.1375	#DIV/0!
**C101927**	0.04761904762	0.04761904762	0		inf	#DIV/0!
**C101927**	0.1904761905	0.1904761905	0.05555555556		3.294117647	#DIV/0!
**C101927**	0.1428571429	0.1428571429	0.05555555556		2.333333333	#DIV/0!
**C101927**	0.04761904762	0.04761904762	0		inf	#DIV/0!
**C101927**	0	0	0.05555555556		0	#DIV/0!
**C101927**	0	0	0.1111111111		0	#DIV/0!
**C101927**	0.04761904762	0.04761904762	0.05555555556		0.7	#DIV/0!
**C101927**	0.04761904762	0.04761904762	0.05555555556		0.7	#DIV/0!
**C101927**	0.1428571429	0.1428571429	0.1111111111		1.083333333	#DIV/0!
**C101927**	0.04761904762	0.04761904762	0.1111111111		0.325	#DIV/0!
**C101927**	0.04761904762	0.04761904762	0.2222222222		0.1375	#DIV/0!
**C101927**	0.09523809524	0.09523809524	0.1111111111		0.6842105263	#DIV/0!
**C101927**	0.09523809524	0.09523809524	0.05555555556		1.473684211	#DIV/0!
**C101927**	0.1904761905	0.1904761905	0		inf	#DIV/0!
**C101927**	0	0	0.05555555556		0	#DIV/0!
**C101927**	0.04761904762	0.04761904762	0.2222222222		0.1375	#DIV/0!
**C101927**	0.04761904762	0.04761904762	0		inf	#DIV/0!
**C101927**	0.04761904762	0.04761904762	0.2222222222		0.1375	#DIV/0!
**C101927**	0.04761904762	0.04761904762	0		inf	#DIV/0!
**C101927**	0.1428571429	0.1428571429	0.1111111111		1.083333333	#DIV/0!
**C101927**	0.09523809524	0.09523809524	0		inf	#DIV/0!
**C101927**	0.04761904762	0.04761904762	0.2222222222		0.1375	#DIV/0!
**C101927**	0.04761904762	0.04761904762	0.1666666667		0.2	#DIV/0!
**C101927**	0.09523809524	0.09523809524	0.05555555556		1.473684211	#DIV/0!

**C101927**	0.04761904762	0.04761904762	0		inf	#DIV/0!
**C101927**	0	0	0.1111111111		0	#DIV/0!
**C101927**	0.04761904762	0.04761904762	0.05555555556		0.7	#DIV/0!
**C101927**	0.04761904762	0.04761904762	0.2222222222		0.1375	#DIV/0!
**C101927**	0.09523809524	0.09523809524	0.05555555556		1.473684211	#DIV/0!
**C101927**	0.09523809524	0.09523809524	0.05555555556		1.473684211	#DIV/0!
**C101927**	0.04761904762	0.04761904762	0		inf	#DIV/0!
**C101927**	0.09523809524	0.09523809524	0		inf	#DIV/0!
**C101927**	0.04761904762	0.04761904762	0.05555555556		0.7	#DIV/0!
**C101927**	0.04761904762	0.04761904762	0		inf	#DIV/0!
**C101927**	0	0	0.05555555556		0	#DIV/0!
**C101927**	0	0	0.05555555556		0	#DIV/0!
**C101927**	0	0	0.05555555556		0	#DIV/0!
**C101927**	0	0	0.05555555556		0	#DIV/0!
**C101927**	0	0	0.1111111111		0	#DIV/0!
**C101927**	0.1428571429	0.1428571429	0.05555555556		2.333333333	#DIV/0!
**C101927**	0.04761904762	0.04761904762	0.1111111111		0.325	#DIV/0!
**C101927**	0.1428571429	0.1428571429	0.2222222222		0.4583333333	#DIV/0!
**C101927**	0	0	0.05555555556		0	#DIV/0!
**C101927**	0.04761904762	0.04761904762	0.05555555556		0.7	#DIV/0!
**C101927**	0	0	0.05555555556		0	#DIV/0!
**C101927**	0.04761904762	0.04761904762	0.1666666667		0.2	#DIV/0!
**C101927**	0	0	0.05555555556		0	#DIV/0!
**C101927**	0	0	0.05555555556		0	#DIV/0!
**C101927**	0.04761904762	0.04761904762	0.05555555556		0.7	#DIV/0!
**C101927**	0	0	0.05555555556		0	#DIV/0!
**C101927**	0.09523809524	0.09523809524	0.05555555556		1.473684211	#DIV/0!
**C101928**	0	0	0.1111111111		0	#DIV/0!
**C101928**	0.09523809524	0.09523809524	0.1111111111		0.6842105263	#DIV/0!
**C101928**	0.04761904762	0.04761904762	0.05555555556		0.7	#DIV/0!
**C101928**	0.1904761905	0.1904761905	0		inf	#DIV/0!
**C101928**	0.04761904762	0.04761904762	0.05555555556		0.7	#DIV/0!
**C101928**	0	0	0.05555555556		0	#DIV/0!
**C101928**	0	0	0			#DIV/0!
**C101928**	0.04761904762	0.04761904762	0.1111111111		0.325	#DIV/0!
**C101928**	0.1904761905	0.1904761905	0.05555555556		3.294117647	#DIV/0!
**C101928**	0.04761904762	0.04761904762	0		inf	#DIV/0!
**C101928**	0.1428571429	0.1428571429	0.1111111111		1.083333333	#DIV/0!
**C101928**	0	0	0.05555555556		0	#DIV/0!
**C101928**	0.1428571429	0.1428571429	0.1111111111		1.083333333	#DIV/0!
**C101928**	0.09523809524	0.09523809524	0.05555555556		1.473684211	#DIV/0!
**C101928**	0.1428571429	0.1428571429	0.05555555556		2.333333333	#DIV/0!
**C101928**	0.04761904762	0.04761904762	0.1666666667		0.2	#DIV/0!
**C101928**	0	0	0.05555555556		0	#DIV/0!
**C101928**	0.04761904762	0.04761904762	0.05555555556		0.7	#DIV/0!
**C101928**	0.04761904762	0.04761904762	0.1111111111		0.325	#DIV/0!
**C101928**	0.1904761905	0.1904761905	0.1111111111		1.529411765	#DIV/0!
**C101928**	0.04761904762	0.04761904762	0.1111111111		0.325	#DIV/0!
**C101928**	0.04761904762	0.04761904762	0.05555555556		0.7	#DIV/0!
**C101928**	0.04761904762	0.04761904762	0.05555555556		0.7	#DIV/0!
**C101928**	0.1904761905	0.1904761905	0.1111111111		1.529411765	#DIV/0!

**C101928**	0.09523809524	0.09523809524	0		inf	#DIV/0!
**C101928**	0.04761904762	0.04761904762	0.1111111111		0.325	#DIV/0!
**C101928**	0	0	0.1111111111		0	#DIV/0!
**C101928**	0.04761904762	0.04761904762	0		inf	#DIV/0!
**C101928**	0.09523809524	0.09523809524	0		inf	#DIV/0!
**C101928**	0.04761904762	0.04761904762	0.1666666667		0.2	#DIV/0!
**C101928**	0.04761904762	0.04761904762	0.1111111111		0.325	#DIV/0!
**C101928**	0.1904761905	0.1904761905	0.1111111111		1.529411765	#DIV/0!
**C101928**	0.04761904762	0.04761904762	0.2222222222		0.1375	#DIV/0!
**C101928**	0.1428571429	0.1428571429	0.1111111111		1.083333333	#DIV/0!
**C101928**	0.04761904762	0.04761904762	0		inf	#DIV/0!
**C101928**	0.1904761905	0.1904761905	0.2222222222		0.6470588235	#DIV/0!
**C101928**	0.04761904762	0.04761904762	0.05555555556		0.7	#DIV/0!
**C101928**	0	0	0.05555555556		0	#DIV/0!
**C101928**	0.1428571429	0.1428571429	0		inf	#DIV/0!
**C101928**	0.04761904762	0.04761904762	0		inf	#DIV/0!
**C101928**	0.04761904762	0.04761904762	0		inf	#DIV/0!
**C101928**	0.04761904762	0.04761904762	0.05555555556		0.7	#DIV/0!
**C101928**	0.04761904762	0.04761904762	0.05555555556		0.7	#DIV/0!
**C101928**	0.04761904762	0.04761904762	0.1666666667		0.2	#DIV/0!
**C101928**	0.09523809524	0.09523809524	0.1111111111		0.6842105263	#DIV/0!
**C101928**	0	0	0.05555555556		0	#DIV/0!
**C101928**	0.04761904762	0.04761904762	0.05555555556		0.7	#DIV/0!
**C101928**	0.09523809524	0.09523809524	0.1111111111		0.6842105263	#DIV/0!
**C101928**	0.04761904762	0.04761904762	0.1111111111		0.325	#DIV/0!
**C101928**	0.04761904762	0.04761904762	0		inf	#DIV/0!
**C101928**	0.04761904762	0.04761904762	0.1111111111		0.325	#DIV/0!
**C101928**	0.04761904762	0.04761904762	0.05555555556		0.7	#DIV/0!
**C101928**	0.04761904762	0.04761904762	0		inf	#DIV/0!
**C101928**	0.04761904762	0.04761904762	0.05555555556		0.7	#DIV/0!
**C101928**	0	0	0.05555555556		0	#DIV/0!
**C101928**	0.1428571429	0.1428571429	0		inf	#DIV/0!
**C101928**	0.1904761905	0.1904761905	0.2222222222		0.6470588235	#DIV/0!
**C101928**	0	0	0.05555555556		0	#DIV/0!
**C101928**	0.04761904762	0.04761904762	0.1111111111		0.325	#DIV/0!
**C101928**	0.1428571429	0.1428571429	0.1666666667		0.6666666667	#DIV/0!
**C101928**	0.1428571429	0.1428571429	0		inf	#DIV/0!
**C101928**	0.04761904762	0.04761904762	0		inf	#DIV/0!
**C101928**	0.09523809524	0.09523809524	0.05555555556		1.473684211	#DIV/0!
**C101928**	0.04761904762	0.04761904762	0		inf	#DIV/0!
**C101928**	0.04761904762	0.04761904762	0.05555555556		0.7	#DIV/0!
**C101928**	0	0	0			#DIV/0!
**C101928**	0.04761904762	0.04761904762	0.05555555556		0.7	#DIV/0!
**C101928**	0.04761904762	0.04761904762	0		inf	#DIV/0!
**C101928**	0.04761904762	0.04761904762	0.05555555556		0.7	#DIV/0!
**C101928**	0.09523809524	0.09523809524	0.05555555556		1.473684211	#DIV/0!
**C101928**	0.04761904762	0.04761904762	0		inf	#DIV/0!
**C101928**	0.09523809524	0.09523809524	0.1111111111		0.6842105263	#DIV/0!
**C101928**	0.04761904762	0.04761904762	0.05555555556		0.7	#DIV/0!
**C101928**	0.04761904762	0.04761904762	0		inf	#DIV/0!
**C101928**	0.04761904762	0.04761904762	0		inf	#DIV/0!

**C101928**	0.04761904762	0.04761904762	0		inf	#DIV/0!
**C101928**	0.04761904762	0.04761904762	0		inf	#DIV/0!
**C101928**	0.04761904762	0.04761904762	0		inf	#DIV/0!
**C101928**	0.09523809524	0.09523809524	0.05555555556		1.473684211	#DIV/0!
**C101928**	0.04761904762	0.04761904762	0		inf	#DIV/0!
**C101928**	0.1428571429	0.1428571429	0		inf	#DIV/0!
**C101928**	0	0	0.05555555556		0	#DIV/0!
**C101928**	0.04761904762	0.04761904762	0		inf	#DIV/0!
**C101928**	0.1428571429	0.1428571429	0		inf	#DIV/0!
**C101928**	0.09523809524	0.09523809524	0.05555555556		1.473684211	#DIV/0!
**C101928**	0.09523809524	0.09523809524	0.05555555556		1.473684211	#DIV/0!
**C101928**	0.04761904762	0.04761904762	0.05555555556		0.7	#DIV/0!
**C101928**	0.04761904762	0.04761904762	0.2222222222		0.1375	#DIV/0!
**C101928**	0.04761904762	0.04761904762	0.1666666667		0.2	#DIV/0!
**C101928**	0.04761904762	0.04761904762	0		inf	#DIV/0!
**C101928**	0.04761904762	0.04761904762	0		inf	#DIV/0!
**C101928**	0	0	0.05555555556		0	#DIV/0!
**C101928**	0.1428571429	0.1428571429	0		inf	#DIV/0!
**C101928**	0.04761904762	0.04761904762	0		inf	#DIV/0!
**C101928**	0.04761904762	0.04761904762	0		inf	#DIV/0!
**C101928**	0.1428571429	0.1428571429	0		inf	#DIV/0!
**C101928**	0.04761904762	0.04761904762	0		inf	#DIV/0!
**C101928**	0.09523809524	0.09523809524	0.05555555556		1.473684211	#DIV/0!
**C101928**	0.04761904762	0.04761904762	0		inf	#DIV/0!
**C101928**	0.04761904762	0.04761904762	0		inf	#DIV/0!
**C101928**	0.04761904762	0.04761904762	0.05555555556		0.7	#DIV/0!
**C101928**	0.04761904762	0.04761904762	0.1111111111		0.325	#DIV/0!
**C101928**	0.04761904762	0.04761904762	0.05555555556		0.7	#DIV/0!
**C101928**	0.04761904762	0.04761904762	0		inf	#DIV/0!
**C101928**	0.1904761905	0.1904761905	0.05555555556		3.294117647	#DIV/0!
**C101928**	0.04761904762	0.04761904762	0.05555555556		0.7	#DIV/0!
**C101928**	0.04761904762	0.04761904762	0.05555555556		0.7	#DIV/0!
**C101928**	0.04761904762	0.04761904762	0.05555555556		0.7	#DIV/0!
**C101928**	0.09523809524	0.09523809524	0		inf	#DIV/0!
**C101928**	0.04761904762	0.04761904762	0.1111111111		0.325	#DIV/0!
**C101928**	0.04761904762	0.04761904762	0		inf	#DIV/0!
**C101928**	0.09523809524	0.09523809524	0.05555555556		1.473684211	#DIV/0!
**C101928**	0.04761904762	0.04761904762	0.05555555556		0.7	#DIV/0!
**C101928**	0.04761904762	0.04761904762	0.05555555556		0.7	#DIV/0!
**C101928**	0.09523809524	0.09523809524	0.1111111111		0.6842105263	#DIV/0!
**C101928**	0.1428571429	0.1428571429	0.05555555556		2.333333333	#DIV/0!
**C101928**	0.04761904762	0.04761904762	0		inf	#DIV/0!
**C101928**	0.04761904762	0.04761904762	0		inf	#DIV/0!
**C101928**	0.04761904762	0.04761904762	0.05555555556		0.7	#DIV/0!
**C101928**	0	0	0.05555555556		0	#DIV/0!
**C101928**	0.1904761905	0.1904761905	0.05555555556		3.294117647	#DIV/0!
**C101928**	0	0	0.05555555556		0	#DIV/0!
**C101928**	0.04761904762	0.04761904762	0.1666666667		0.2	#DIV/0!
**C101928**	0.04761904762	0.04761904762	0.05555555556		0.7	#DIV/0!
**C101928**	0.04761904762	0.04761904762	0.05555555556		0.7	#DIV/0!
**C101928**	0.09523809524	0.09523809524	0		inf	#DIV/0!

**C101928**	0.04761904762	0.04761904762	0.05555555556		0.7	#DIV/0!
**C101928**	0	0	0.05555555556		0	#DIV/0!
**C101928**	0	0	0.05555555556		0	#DIV/0!
**C101928**	0.1428571429	0.1428571429	0		inf	#DIV/0!
**C101928**	0.04761904762	0.04761904762	0.05555555556		0.7	#DIV/0!
**C101928**	0	0	0.05555555556		0	#DIV/0!
**C101928**	0.04761904762	0.04761904762	0.05555555556		0.7	#DIV/0!
**C101928**	0.1904761905	0.1904761905	0		inf	#DIV/0!
**C101929**	0	0	0			#DIV/0!
**C101929**	0.04761904762	0.04761904762	0.05555555556		0.7	#DIV/0!
**C101929**	0	0	0.05555555556		0	#DIV/0!
**C101929**	0.09523809524	0.09523809524	0.1111111111		0.6842105263	#DIV/0!
**C101929**	0.09523809524	0.09523809524	0.1111111111		0.6842105263	#DIV/0!
**C101929**	0.04761904762	0.04761904762	0.05555555556		0.7	#DIV/0!
**C101929**	0	0	0			#DIV/0!
**C101929**	0.04761904762	0.04761904762	0.05555555556		0.7	#DIV/0!
**C101929**	0.09523809524	0.09523809524	0.05555555556		1.473684211	#DIV/0!
**C101929**	0.04761904762	0.04761904762	0		inf	#DIV/0!
**C101929**	0.09523809524	0.09523809524	0.05555555556		1.473684211	#DIV/0!
**C101929**	0.09523809524	0.09523809524	0.1111111111		0.6842105263	#DIV/0!
**C101929**	0.09523809524	0.09523809524	0		inf	#DIV/0!
**C101929**	0.09523809524	0.09523809524	0.1111111111		0.6842105263	#DIV/0!
**C101929**	0.09523809524	0.09523809524	0.05555555556		1.473684211	#DIV/0!
**C101929**	0.04761904762	0.04761904762	0		inf	#DIV/0!
**C101929**	0.04761904762	0.04761904762	0.05555555556		0.7	#DIV/0!
**C101929**	0.1904761905	0.1904761905	0.05555555556		3.294117647	#DIV/0!
**C101929**	0.09523809524	0.09523809524	0.1111111111		0.6842105263	#DIV/0!
**C101929**	0.04761904762	0.04761904762	0		inf	#DIV/0!
**C101929**	0	0	0.05555555556		0	#DIV/0!
**C101929**	0.09523809524	0.09523809524	0.1666666667		0.4210526316	#DIV/0!
**C101929**	0.1428571429	0.1428571429	0		inf	#DIV/0!
**C101929**	0.09523809524	0.09523809524	0.1111111111		0.6842105263	#DIV/0!
**C101929**	0	0	0.05555555556		0	#DIV/0!
**C101929**	0.1428571429	0.1428571429	0.05555555556		2.333333333	#DIV/0!
**C101929**	0.04761904762	0.04761904762	0.1111111111		0.325	#DIV/0!
**C101929**	0.04761904762	0.04761904762	0		inf	#DIV/0!
**C101929**	0.09523809524	0.09523809524	0.1666666667		0.4210526316	#DIV/0!
**C101929**	0.09523809524	0.09523809524	0.1111111111		0.6842105263	#DIV/0!
**C101929**	0.09523809524	0.09523809524	0.2222222222		0.2894736842	#DIV/0!
**C101929**	0.04761904762	0.04761904762	0.1111111111		0.325	#DIV/0!
**C101929**	0.04761904762	0.04761904762	0.1111111111		0.325	#DIV/0!
**C101929**	0	0	0.05555555556		0	#DIV/0!
**C101929**	0.04761904762	0.04761904762	0.1111111111		0.325	#DIV/0!
**C101929**	0.09523809524	0.09523809524	0.05555555556		1.473684211	#DIV/0!
**C101929**	0.09523809524	0.09523809524	0.1111111111		0.6842105263	#DIV/0!
**C101929**	0	0	0.05555555556		0	#DIV/0!
**C101929**	0.04761904762	0.04761904762	0		inf	#DIV/0!
**C101929**	0.04761904762	0.04761904762	0		inf	#DIV/0!
**C101929**	0.04761904762	0.04761904762	0.1111111111		0.325	#DIV/0!
**C101929**	0.09523809524	0.09523809524	0.05555555556		1.473684211	#DIV/0!
**C101929**	0.1904761905	0.1904761905	0.1666666667		0.9411764706	#DIV/0!

**C101929**	0.1428571429	0.1428571429	0.1111111111		1.083333333	#DIV/0!
**C101929**	0.09523809524	0.09523809524	0.2222222222		0.2894736842	#DIV/0!
**C101929**	0.04761904762	0.04761904762	0.05555555556		0.7	#DIV/0!
**C101929**	0	0	0.05555555556		0	#DIV/0!
**C101929**	0.04761904762	0.04761904762	0.05555555556		0.7	#DIV/0!
**C101929**	0.04761904762	0.04761904762	0.05555555556		0.7	#DIV/0!
**C101929**	0.04761904762	0.04761904762	0.1111111111		0.325	#DIV/0!
**C101929**	0.04761904762	0.04761904762	0.05555555556		0.7	#DIV/0!
**C101929**	0.1428571429	0.1428571429	0.1111111111		1.083333333	#DIV/0!
**C101929**	0	0	0.05555555556		0	#DIV/0!
**C101929**	0.04761904762	0.04761904762	0.05555555556		0.7	#DIV/0!
**C101929**	0.1904761905	0.1904761905	0.05555555556		3.294117647	#DIV/0!
**C101929**	0.1428571429	0.1428571429	0.1666666667		0.6666666667	#DIV/0!
**C101929**	0	0	0.05555555556		0	#DIV/0!
**C101929**	0	0	0.05555555556		0	#DIV/0!
**C101929**	0.04761904762	0.04761904762	0.05555555556		0.7	#DIV/0!
**C101929**	0.04761904762	0.04761904762	0.1111111111		0.325	#DIV/0!
**C101929**	0	0	0.05555555556		0	#DIV/0!
**C101929**	0	0	0			#DIV/0!
**C101929**	0	0	0.05555555556		0	#DIV/0!
**C101929**	0.09523809524	0.09523809524	0.05555555556		1.473684211	#DIV/0!
**C101929**	0	0	0.05555555556		0	#DIV/0!
**C101929**	0.1428571429	0.1428571429	0.1111111111		1.083333333	#DIV/0!
**C101929**	0.1428571429	0.1428571429	0		inf	#DIV/0!
**C101929**	0	0	0.05555555556		0	#DIV/0!
**C101929**	0.09523809524	0.09523809524	0.05555555556		1.473684211	#DIV/0!
**C101929**	0.1904761905	0.1904761905	0.2222222222		0.6470588235	#DIV/0!
**C101929**	0.04761904762	0.04761904762	0.05555555556		0.7	#DIV/0!
**C101929**	0.04761904762	0.04761904762	0.05555555556		0.7	#DIV/0!
**C101929**	0	0	0.05555555556		0	#DIV/0!
**C101929**	0.09523809524	0.09523809524	0.1666666667		0.4210526316	#DIV/0!
**C101929**	0.04761904762	0.04761904762	0.1111111111		0.325	#DIV/0!
**C101929**	0	0	0.05555555556		0	#DIV/0!
**C101929**	0.04761904762	0.04761904762	0.1111111111		0.325	#DIV/0!
**C101929**	0.04761904762	0.04761904762	0.05555555556		0.7	#DIV/0!
**C101929**	0	0	0.05555555556		0	#DIV/0!
**C101929**	0.04761904762	0.04761904762	0.05555555556		0.7	#DIV/0!
**C101929**	0.1428571429	0.1428571429	0		inf	#DIV/0!
**C101929**	0.09523809524	0.09523809524	0.05555555556		1.473684211	#DIV/0!
**C101929**	0.09523809524	0.09523809524	0.1111111111		0.6842105263	#DIV/0!
**C101929**	0.04761904762	0.04761904762	0.05555555556		0.7	#DIV/0!
**C101929**	0.1904761905	0.1904761905	0.1111111111		1.529411765	#DIV/0!
**C101929**	0.04761904762	0.04761904762	0		inf	#DIV/0!
**C101929**	0.04761904762	0.04761904762	0		inf	#DIV/0!
**C101929**	0	0	0			#DIV/0!
**C101929**	0.04761904762	0.04761904762	0.05555555556		0.7	#DIV/0!
**C101929**	0	0	0.05555555556		0	#DIV/0!
**C101929**	0.04761904762	0.04761904762	0		inf	#DIV/0!
**C101929**	0.1428571429	0.1428571429	0.05555555556		2.333333333	#DIV/0!
**C101929**	0	0	0.1111111111		0	#DIV/0!
**C101929**	0	0	0.05555555556		0	#DIV/0!

**C101929**	0.09523809524	0.09523809524	0.1666666667		0.4210526316	#DIV/0!
**C101929**	0.04761904762	0.04761904762	0.05555555556		0.7	#DIV/0!
**C101929**	0.04761904762	0.04761904762	0.05555555556		0.7	#DIV/0!
**C101929**	0.1428571429	0.1428571429	0.05555555556		2.333333333	#DIV/0!
**C101930**	0.04761904762	0.04761904762	0.05555555556		0.7	#DIV/0!
**C101930**	0.04761904762	0.04761904762	0.05555555556		0.7	#DIV/0!
**C101930**	0.04761904762	0.04761904762	0.2222222222		0.1375	#DIV/0!
**C101930**	0.04761904762	0.04761904762	0.1111111111		0.325	#DIV/0!
**C101930**	0	0	0.05555555556		0	#DIV/0!
**C101930**	0.04761904762	0.04761904762	0.1111111111		0.325	#DIV/0!
**C102031**	0.09523809524	0.09523809524	0.2222222222		0.2894736842	#DIV/0!
**C102467**	0.04761904762	0.04761904762	0.05555555556		0.7	#DIV/0!
**C102467**	0.04761904762	0.04761904762	0.05555555556		0.7	#DIV/0!
**C102467**	0.04761904762	0.04761904762	0		inf	#DIV/0!
**C102467**	0.04761904762	0.04761904762	0		inf	#DIV/0!
**C102467**	0.09523809524	0.09523809524	0.1111111111		0.6842105263	#DIV/0!
**C102467**	0.04761904762	0.04761904762	0		inf	#DIV/0!
**C102503**	0	0	0.05555555556		0	#DIV/0!
**C102546**	0.04761904762	0.04761904762	0		inf	#DIV/0!
**C102546**	0	0	0.05555555556		0	#DIV/0!
**C102606**	0.04761904762	0.04761904762	0.05555555556		0.7	#DIV/0!
**C102606**	0.1904761905	0.1904761905	0.1111111111		1.529411765	#DIV/0!
**C102723**	0.09523809524	0.09523809524	0.1111111111		0.6842105263	#DIV/0!
**C102723**	0.1428571429	0.1428571429	0.1111111111		1.083333333	#DIV/0!
**C102723**	0.04761904762	0.04761904762	0.05555555556		0.7	#DIV/0!
**C102723**	0.04761904762	0.04761904762	0.05555555556		0.7	#DIV/0!
**C102723**	0.09523809524	0.09523809524	0		inf	#DIV/0!
**C102723**	0.04761904762	0.04761904762	0		inf	#DIV/0!
**C102723**	0.04761904762	0.04761904762	0.05555555556		0.7	#DIV/0!
**C102723**	0.1904761905	0.1904761905	0.05555555556		3.294117647	#DIV/0!
**C102723**	0.04761904762	0.04761904762	0.05555555556		0.7	#DIV/0!
**C102723**	0	0	0			#DIV/0!
**C102723**	0.2857142857	0.2857142857	0.1111111111		2.6	#DIV/0!
**C102723**	0.04761904762	0.04761904762	0.1111111111		0.325	#DIV/0!
**C102723**	0.09523809524	0.09523809524	0.05555555556		1.473684211	#DIV/0!
**C102723**	0.09523809524	0.09523809524	0.05555555556		1.473684211	#DIV/0!
**C102723**	0.04761904762	0.04761904762	0		inf	#DIV/0!
**C102723**	0.1428571429	0.1428571429	0		inf	#DIV/0!
**C102723**	0.04761904762	0.04761904762	0.05555555556		0.7	#DIV/0!
**C102723**	0.1428571429	0.1428571429	0		inf	#DIV/0!
**C102723**	0.04761904762	0.04761904762	0.1666666667		0.2	#DIV/0!
**C102723**	0.04761904762	0.04761904762	0		inf	#DIV/0!
**C102723**	0.04761904762	0.04761904762	0.1111111111		0.325	#DIV/0!
**C102723**	0.1428571429	0.1428571429	0		inf	#DIV/0!
**C102723**	0	0	0.05555555556		0	#DIV/0!
**C102723**	0.1428571429	0.1428571429	0.2222222222		0.4583333333	#DIV/0!
**C102723**	0.1428571429	0.1428571429	0		inf	#DIV/0!
**C102723**	0.09523809524	0.09523809524	0.1666666667		0.4210526316	#DIV/0!
**C102723**	0.1428571429	0.1428571429	0		inf	#DIV/0!
**C102723**	0.09523809524	0.09523809524	0.05555555556		1.473684211	#DIV/0!
**C102723**	0.09523809524	0.09523809524	0		inf	#DIV/0!

C102723	0	0	0.05555555556		0	#DIV/0!
C102723	0.1904761905	0.1904761905	0		inf	#DIV/0!
C102723	0.09523809524	0.09523809524	0.1111111111		0.6842105263	#DIV/0!
C102723	0.09523809524	0.09523809524	0.1111111111		0.6842105263	#DIV/0!
C102723	0.04761904762	0.04761904762	0.1111111111		0.325	#DIV/0!
C102724	0	0	0.1111111111		0	#DIV/0!
C102724	0.04761904762	0.04761904762	0.1111111111		0.325	#DIV/0!
C102724	0.04761904762	0.04761904762	0.05555555556		0.7	#DIV/0!
C102724	0.04761904762	0.04761904762	0.05555555556		0.7	#DIV/0!
C102724	0	0	0.05555555556		0	#DIV/0!
C102724	0.09523809524	0.09523809524	0		inf	#DIV/0!
C102724	0.04761904762	0.04761904762	0.05555555556		0.7	#DIV/0!
C102724	0.04761904762	0.04761904762	0.05555555556		0.7	#DIV/0!
C102724	0	0	0.1111111111		0	#DIV/0!
C102724	0	0	0.05555555556		0	#DIV/0!
C102724	0.04761904762	0.04761904762	0.1111111111		0.325	#DIV/0!
C102724	0.04761904762	0.04761904762	0		inf	#DIV/0!
C102724	0.1904761905	0.1904761905	0.05555555556		3.294117647	#DIV/0!
C102724	0.1428571429	0.1428571429	0.05555555556		2.333333333	#DIV/0!
C102724	0	0	0.1111111111		0	#DIV/0!
C102724	0.04761904762	0.04761904762	0.05555555556		0.7	#DIV/0!
C102724	0.09523809524	0.09523809524	0.05555555556		1.473684211	#DIV/0!
C102724	0.04761904762	0.04761904762	0.1111111111		0.325	#DIV/0!
C102724	0	0	0.05555555556		0	#DIV/0!
C102724	0.04761904762	0.04761904762	0.05555555556		0.7	#DIV/0!
C102724	0.04761904762	0.04761904762	0		inf	#DIV/0!
C102724	0.04761904762	0.04761904762	0.1666666667		0.2	#DIV/0!
C102724	0.1428571429	0.1428571429	0		inf	#DIV/0!
C102724	0.04761904762	0.04761904762	0.05555555556		0.7	#DIV/0!
C102724	0.04761904762	0.04761904762	0.1111111111		0.325	#DIV/0!
C102724	0	0	0.05555555556		0	#DIV/0!
C102724	0.09523809524	0.09523809524	0.2222222222		0.2894736842	#DIV/0!
C102724	0.1428571429	0.1428571429	0.05555555556		2.333333333	#DIV/0!
C102724	0.1428571429	0.1428571429	0		inf	#DIV/0!
C102724	0.1428571429	0.1428571429	0		inf	#DIV/0!
C102724	0.1428571429	0.1428571429	0.05555555556		2.333333333	#DIV/0!
C102724	0.1428571429	0.1428571429	0.1666666667		0.6666666667	#DIV/0!
C102724	0.04761904762	0.04761904762	0.1111111111		0.325	#DIV/0!
C102724	0	0	0.05555555556		0	#DIV/0!
C102724	0.1428571429	0.1428571429	0.1666666667		0.6666666667	#DIV/0!
C102724	0.1904761905	0.1904761905	0.1111111111		1.529411765	#DIV/0!
C102724	0.04761904762	0.04761904762	0.05555555556		0.7	#DIV/0!
C102724	0.04761904762	0.04761904762	0.05555555556		0.7	#DIV/0!
C102724	0.09523809524	0.09523809524	0.05555555556		1.473684211	#DIV/0!
C102724	0.2380952381	0.2380952381	0.1111111111		2.03125	#DIV/0!
C102724	0.04761904762	0.04761904762	0.05555555556		0.7	#DIV/0!
C102724	0	0	0.05555555556		0	#DIV/0!
C102724	0.04761904762	0.04761904762	0.05555555556		0.7	#DIV/0!
C102724	0.1904761905	0.1904761905	0.1666666667		0.9411764706	#DIV/0!
C102724	0	0	0.05555555556		0	#DIV/0!
C102724	0.1904761905	0.1904761905	0.1111111111		1.529411765	#DIV/0!

**C102724**	0.09523809524	0.09523809524	0.2222222222		0.2894736842	#DIV/0!
**C102724**	0.09523809524	0.09523809524	0.05555555556		1.473684211	#DIV/0!
**C102724**	0.1428571429	0.1428571429	0.05555555556		2.333333333	#DIV/0!
**C102724**	0.04761904762	0.04761904762	0.1111111111		0.325	#DIV/0!
**C102724**	0.04761904762	0.04761904762	0.1666666667		0.2	#DIV/0!
**C102724**	0.09523809524	0.09523809524	0.1666666667		0.4210526316	#DIV/0!
**C102725**	0.04761904762	0.04761904762	0.05555555556		0.7	#DIV/0!
**C102725**	0.09523809524	0.09523809524	0.1111111111		0.6842105263	#DIV/0!
**C102725**	0.04761904762	0.04761904762	0.2222222222		0.1375	#DIV/0!
**C102725**	0.04761904762	0.04761904762	0.2222222222		0.1375	#DIV/0!
**C102725**	0	0	0.05555555556		0	#DIV/0!
**C102725**	0.1428571429	0.1428571429	0.1111111111		1.083333333	#DIV/0!
**C103021**	0	0	0.1111111111		0	#DIV/0!
**C103171**	0.04761904762	0.04761904762	0.05555555556		0.7	#DIV/0!
**C103312**	0.04761904762	0.04761904762	0		inf	#DIV/0!
**C103344**	0.1428571429	0.1428571429	0.05555555556		2.333333333	#DIV/0!
**C103908**	0.1428571429	0.1428571429	0.05555555556		2.333333333	#DIV/0!
**C103908**	0.1428571429	0.1428571429	0.05555555556		2.333333333	#DIV/0!
**C104054**	0.1904761905	0.1904761905	0.1111111111		1.529411765	#DIV/0!
**C104613**	0.04761904762	0.04761904762	0.05555555556		0.7	#DIV/0!
**C104968**	0.09523809524	0.09523809524	0		inf	#DIV/0!
**C105274**	0.04761904762	0.04761904762	0		inf	#DIV/0!
**C105369**	0.09523809524	0.09523809524	0.05555555556		1.473684211	#DIV/0!
**C105369**	0.04761904762	0.04761904762	0.1666666667		0.2	#DIV/0!
**C105369**	0.1904761905	0.1904761905	0.1111111111		1.529411765	#DIV/0!
**C105369**	0.09523809524	0.09523809524	0.05555555556		1.473684211	#DIV/0!
**C105369**	0.09523809524	0.09523809524	0.05555555556		1.473684211	#DIV/0!
**C105369**	0.04761904762	0.04761904762	0.05555555556		0.7	#DIV/0!
**C105369**	0.1428571429	0.1428571429	0.2222222222		0.4583333333	#DIV/0!
**C105369**	0.04761904762	0.04761904762	0.05555555556		0.7	#DIV/0!
**C105369**	0.1428571429	0.1428571429	0		inf	#DIV/0!
**C105369**	0.2380952381	0.2380952381	0.1111111111		2.03125	#DIV/0!
**C105369**	0.04761904762	0.04761904762	0.1666666667		0.2	#DIV/0!
**C105369**	0.04761904762	0.04761904762	0.1111111111		0.325	#DIV/0!
**C105369**	0.04761904762	0.04761904762	0.1111111111		0.325	#DIV/0!
**C105369**	0.04761904762	0.04761904762	0.05555555556		0.7	#DIV/0!
**C105369**	0.04761904762	0.04761904762	0.1111111111		0.325	#DIV/0!
**C105369**	0.04761904762	0.04761904762	0.05555555556		0.7	#DIV/0!
**C105369**	0.09523809524	0.09523809524	0.05555555556		1.473684211	#DIV/0!
**C105369**	0.04761904762	0.04761904762	0.05555555556		0.7	#DIV/0!
**C105369**	0.04761904762	0.04761904762	0.05555555556		0.7	#DIV/0!
**C105369**	0.04761904762	0.04761904762	0.05555555556		0.7	#DIV/0!
**C105369**	0.04761904762	0.04761904762	0.05555555556		0.7	#DIV/0!
**C105369**	0.04761904762	0.04761904762	0.05555555556		0.7	#DIV/0!
**C105369**	0	0	0.1111111111		0	#DIV/0!
**C105370**	0	0	0.05555555556		0	#DIV/0!
**C105370**	0.09523809524	0.09523809524	0.05555555556		1.473684211	#DIV/0!
**C105370**	0	0	0.05555555556		0	#DIV/0!
**C105370**	0.04761904762	0.04761904762	0.05555555556		0.7	#DIV/0!
**C105370**	0.04761904762	0.04761904762	0		inf	#DIV/0!
**C105370**	0.04761904762	0.04761904762	0		inf	#DIV/0!

**C105370**	0.04761904762	0.04761904762	0		inf	#DIV/0!
**C105370**	0.04761904762	0.04761904762	0.05555555556		0.7	#DIV/0!
**C105370**	0	0	0			#DIV/0!
**C105370**	0.04761904762	0.04761904762	0.05555555556		0.7	#DIV/0!
**C105370**	0.04761904762	0.04761904762	0.05555555556		0.7	#DIV/0!
**C105371**	0.04761904762	0.04761904762	0.05555555556		0.7	#DIV/0!
**C105371**	0.04761904762	0.04761904762	0.2222222222		0.1375	#DIV/0!
**C105371**	0.09523809524	0.09523809524	0.2222222222		0.2894736842	#DIV/0!
**C105371**	0.09523809524	0.09523809524	0.2222222222		0.2894736842	#DIV/0!
**C105371**	0.09523809524	0.09523809524	0.2222222222		0.2894736842	#DIV/0!
**C105371**	0.04761904762	0.04761904762	0		inf	#DIV/0!
**C105371**	0.04761904762	0.04761904762	0.2222222222		0.1375	#DIV/0!
**C105371**	0.1428571429	0.1428571429	0.05555555556		2.333333333	#DIV/0!
**C105371**	0.04761904762	0.04761904762	0.05555555556		0.7	#DIV/0!
**C105371**	0.1428571429	0.1428571429	0.05555555556		2.333333333	#DIV/0!
**C105371**	0.04761904762	0.04761904762	0		inf	#DIV/0!
**C105371**	0.09523809524	0.09523809524	0		inf	#DIV/0!
**C105371**	0	0	0.1111111111		0	#DIV/0!
**C105371**	0.1904761905	0.1904761905	0.05555555556		3.294117647	#DIV/0!
**C105371**	0.1428571429	0.1428571429	0.05555555556		2.333333333	#DIV/0!
**C105371**	0	0	0.1111111111		0	#DIV/0!
**C105371**	0	0	0.05555555556		0	#DIV/0!
**C105371**	0	0	0.1111111111		0	#DIV/0!
**C105371**	0.09523809524	0.09523809524	0.05555555556		1.473684211	#DIV/0!
**C105371**	0	0	0			#DIV/0!
**C105371**	0.04761904762	0.04761904762	0		inf	#DIV/0!
**C105371**	0.09523809524	0.09523809524	0		inf	#DIV/0!
**C105371**	0.1428571429	0.1428571429	0		inf	#DIV/0!
**C105371**	0.1428571429	0.1428571429	0		inf	#DIV/0!
**C105371**	0.09523809524	0.09523809524	0		inf	#DIV/0!
**C105371**	0.1428571429	0.1428571429	0		inf	#DIV/0!
**C105371**	0.04761904762	0.04761904762	0		inf	#DIV/0!
**C105371**	0	0	0			#DIV/0!
**C105371**	0.04761904762	0.04761904762	0		inf	#DIV/0!
**C105371**	0.04761904762	0.04761904762	0		inf	#DIV/0!
**C105371**	0.04761904762	0.04761904762	0		inf	#DIV/0!
**C105371**	0.09523809524	0.09523809524	0.05555555556		1.473684211	#DIV/0!
**C105372**	0.09523809524	0.09523809524	0.1111111111		0.6842105263	#DIV/0!
**C105372**	0.09523809524	0.09523809524	0.05555555556		1.473684211	#DIV/0!
**C105372**	0.04761904762	0.04761904762	0		inf	#DIV/0!
**C105372**	0.04761904762	0.04761904762	0		inf	#DIV/0!
**C105372**	0.04761904762	0.04761904762	0		inf	#DIV/0!
**C105372**	0.1428571429	0.1428571429	0.1111111111		1.083333333	#DIV/0!
**C105372**	0.04761904762	0.04761904762	0.05555555556		0.7	#DIV/0!
**C105372**	0.04761904762	0.04761904762	0.05555555556		0.7	#DIV/0!
**C105372**	0.09523809524	0.09523809524	0.05555555556		1.473684211	#DIV/0!
**C105372**	0.09523809524	0.09523809524	0.05555555556		1.473684211	#DIV/0!
**C105372**	0.1428571429	0.1428571429	0.05555555556		2.333333333	#DIV/0!
**C105372**	0.1428571429	0.1428571429	0.05555555556		2.333333333	#DIV/0!
**C105372**	0.1904761905	0.1904761905	0.1111111111		1.529411765	#DIV/0!
**C105372**	0.1904761905	0.1904761905	0.05555555556		3.294117647	#DIV/0!

C105372	0.1428571429	0.1428571429	0.1666666667		0.6666666667	#DIV/0!
C105372	0.1428571429	0.1428571429	0.1666666667		0.6666666667	#DIV/0!
C105372	0.09523809524	0.09523809524	0.1666666667		0.4210526316	#DIV/0!
C105372	0.09523809524	0.09523809524	0.1111111111		0.6842105263	#DIV/0!
C105372	0.1428571429	0.1428571429	0.2222222222		0.4583333333	#DIV/0!
C105372	0.04761904762	0.04761904762	0.1666666667		0.2	#DIV/0!
C105372	0.04761904762	0.04761904762	0.1111111111		0.325	#DIV/0!
C105372	0.04761904762	0.04761904762	0.1111111111		0.325	#DIV/0!
C105372	0.04761904762	0.04761904762	0.1111111111		0.325	#DIV/0!
C105373	0	0	0.1111111111		0	#DIV/0!
C105373	0.04761904762	0.04761904762	0.1111111111		0.325	#DIV/0!
C105373	0	0	0.1111111111		0	#DIV/0!
C105373	0	0	0.1666666667		0	#DIV/0!
C105373	0	0	0.1111111111		0	#DIV/0!
C105373	0	0	0.1111111111		0	#DIV/0!
C105373	0.1428571429	0.1428571429	0		inf	#DIV/0!
C105373	0	0	0.05555555556		0	#DIV/0!
C105373	0	0	0.05555555556		0	#DIV/0!
C105373	0	0	0.1111111111		0	#DIV/0!
C105373	0	0	0.05555555556		0	#DIV/0!
C105373	0	0	0.05555555556		0	#DIV/0!
C105373	0	0	0.05555555556		0	#DIV/0!
C105373	0	0	0.05555555556		0	#DIV/0!
C105373	0	0	0.1111111111		0	#DIV/0!
C105373	0	0	0.1111111111		0	#DIV/0!
C105373	0	0	0.05555555556		0	#DIV/0!
C105373	0	0	0.1111111111		0	#DIV/0!
C105373	0	0	0.1111111111		0	#DIV/0!
C105374	0.09523809524	0.09523809524	0.05555555556		1.473684211	#DIV/0!
C105374	0.09523809524	0.09523809524	0.05555555556		1.473684211	#DIV/0!
C105374	0.1428571429	0.1428571429	0.05555555556		2.333333333	#DIV/0!
C105374	0.1428571429	0.1428571429	0.05555555556		2.333333333	#DIV/0!
C105374	0.1428571429	0.1428571429	0.05555555556		2.333333333	#DIV/0!
C105374	0.04761904762	0.04761904762	0.05555555556		0.7	#DIV/0!
C105374	0.1904761905	0.1904761905	0.1111111111		1.529411765	#DIV/0!
C105374	0.04761904762	0.04761904762	0		inf	#DIV/0!
C105374	0.04761904762	0.04761904762	0		inf	#DIV/0!
C105374	0	0	0.05555555556		0	#DIV/0!
C105374	0	0	0.05555555556		0	#DIV/0!
C105374	0.04761904762	0.04761904762	0		inf	#DIV/0!
C105374	0	0	0.1111111111		0	#DIV/0!
C105374	0.04761904762	0.04761904762	0.05555555556		0.7	#DIV/0!
C105374	0.09523809524	0.09523809524	0.05555555556		1.473684211	#DIV/0!
C105374	0	0	0.05555555556		0	#DIV/0!
C105374	0	0	0.05555555556		0	#DIV/0!
C105375	0.04761904762	0.04761904762	0.05555555556		0.7	#DIV/0!
C105375	0.04761904762	0.04761904762	0.1666666667		0.2	#DIV/0!
C105375	0.09523809524	0.09523809524	0.1666666667		0.4210526316	#DIV/0!
C105375	0.04761904762	0.04761904762	0.2222222222		0.1375	#DIV/0!
C105375	0.04761904762	0.04761904762	0.1666666667		0.2	#DIV/0!
C105375	0.1428571429	0.1428571429	0.1666666667		0.6666666667	#DIV/0!

C105375	0.04761904762	0.04761904762	0.2222222222		0.1375	#DIV/0!
C105375	0.04761904762	0.04761904762	0.1111111111		0.325	#DIV/0!
C105375	0.04761904762	0.04761904762	0.05555555556		0.7	#DIV/0!
C105375	0.04761904762	0.04761904762	0.05555555556		0.7	#DIV/0!
C105375	0.04761904762	0.04761904762	0.05555555556		0.7	#DIV/0!
C105375	0.04761904762	0.04761904762	0.05555555556		0.7	#DIV/0!
C105375	0.04761904762	0.04761904762	0.05555555556		0.7	#DIV/0!
C105375	0.04761904762	0.04761904762	0.05555555556		0.7	#DIV/0!
C105375	0.09523809524	0.09523809524	0.05555555556		1.473684211	#DIV/0!
C105375	0.1904761905	0.1904761905	0.1111111111		1.529411765	#DIV/0!
C105375	0.1904761905	0.1904761905	0.1666666667		0.9411764706	#DIV/0!
C105375	0.1428571429	0.1428571429	0.1111111111		1.083333333	#DIV/0!
C105375	0.04761904762	0.04761904762	0.2222222222		0.1375	#DIV/0!
C105375	0.04761904762	0.04761904762	0.2222222222		0.1375	#DIV/0!
C105375	0.04761904762	0.04761904762	0.1666666667		0.2	#DIV/0!
C105375	0.1428571429	0.1428571429	0.1111111111		1.083333333	#DIV/0!
C105375	0.04761904762	0.04761904762	0.05555555556		0.7	#DIV/0!
C105376	0	0	0.05555555556		0	#DIV/0!
C105376	0.04761904762	0.04761904762	0		inf	#DIV/0!
C105376	0.09523809524	0.09523809524	0.05555555556		1.473684211	#DIV/0!
C105376	0.04761904762	0.04761904762	0		inf	#DIV/0!
C105376	0.09523809524	0.09523809524	0		inf	#DIV/0!
C105376	0.04761904762	0.04761904762	0.1666666667		0.2	#DIV/0!
C105376	0.09523809524	0.09523809524	0.1111111111		0.6842105263	#DIV/0!
C105376	0.09523809524	0.09523809524	0.1111111111		0.6842105263	#DIV/0!
C105376	0.04761904762	0.04761904762	0.05555555556		0.7	#DIV/0!
C105376	0.04761904762	0.04761904762	0.1111111111		0.325	#DIV/0!
C105376	0.09523809524	0.09523809524	0		inf	#DIV/0!
C105376	0.04761904762	0.04761904762	0		inf	#DIV/0!
C105377	0.09523809524	0.09523809524	0		inf	#DIV/0!
C105377	0.04761904762	0.04761904762	0		inf	#DIV/0!
C105377	0.04761904762	0.04761904762	0		inf	#DIV/0!
C105377	0.04761904762	0.04761904762	0		inf	#DIV/0!
C105377	0.04761904762	0.04761904762	0.05555555556		0.7	#DIV/0!
C105377	0	0	0.05555555556		0	#DIV/0!
C105377	0	0	0.05555555556		0	#DIV/0!
C105377	0.04761904762	0.04761904762	0.05555555556		0.7	#DIV/0!
C105377	0	0	0.05555555556		0	#DIV/0!
C105377	0	0	0.05555555556		0	#DIV/0!
C105377	0	0	0.05555555556		0	#DIV/0!
C105377	0	0	0.05555555556		0	#DIV/0!
C105377	0	0	0.05555555556		0	#DIV/0!
C105377	0	0	0.05555555556		0	#DIV/0!
C105377	0.09523809524	0.09523809524	0.05555555556		1.473684211	#DIV/0!
C105377	0.09523809524	0.09523809524	0.05555555556		1.473684211	#DIV/0!
C105377	0.09523809524	0.09523809524	0.1111111111		0.6842105263	#DIV/0!
C105378	0.1428571429	0.1428571429	0.05555555556		2.333333333	#DIV/0!
C105378	0.1428571429	0.1428571429	0.05555555556		2.333333333	#DIV/0!
C105378	0.04761904762	0.04761904762	0.05555555556		0.7	#DIV/0!
C105378	0.09523809524	0.09523809524	0.05555555556		1.473684211	#DIV/0!
C105378	0.09523809524	0.09523809524	0.1111111111		0.6842105263	#DIV/0!

**C105378**	0.1428571429	0.1428571429	0.05555555556		2.333333333	#DIV/0!
**C105378**	0.1428571429	0.1428571429	0.1111111111		1.083333333	#DIV/0!
**C105378**	0.1428571429	0.1428571429	0.05555555556		2.333333333	#DIV/0!
**C105378**	0	0	0.1111111111		0	#DIV/0!
**C105378**	0	0	0.1111111111		0	#DIV/0!
**C105378**	0.04761904762	0.04761904762	0.1111111111		0.325	#DIV/0!
**C105378**	0.04761904762	0.04761904762	0.05555555556		0.7	#DIV/0!
**C105378**	0.04761904762	0.04761904762	0.05555555556		0.7	#DIV/0!
**C105378**	0.04761904762	0.04761904762	0.05555555556		0.7	#DIV/0!
**C105378**	0.04761904762	0.04761904762	0.05555555556		0.7	#DIV/0!
**C105379**	0	0	0.05555555556		0	#DIV/0!
**C105379**	0.04761904762	0.04761904762	0		inf	#DIV/0!
**C105379**	0.04761904762	0.04761904762	0		inf	#DIV/0!
**C105379**	0.04761904762	0.04761904762	0		inf	#DIV/0!
**C105379**	0.04761904762	0.04761904762	0		inf	#DIV/0!
**C105379**	0.04761904762	0.04761904762	0		inf	#DIV/0!
**C105379**	0.1904761905	0.1904761905	0.2222222222		0.6470588235	#DIV/0!
**C105379**	0.04761904762	0.04761904762	0.1111111111		0.325	#DIV/0!
**C105379**	0.09523809524	0.09523809524	0.1666666667		0.4210526316	#DIV/0!
**C105379**	0.1904761905	0.1904761905	0.2222222222		0.6470588235	#DIV/0!
**C105379**	0.04761904762	0.04761904762	0.1111111111		0.325	#DIV/0!
**C105379**	0.1904761905	0.1904761905	0.05555555556		3.294117647	#DIV/0!
**C105379**	0.04761904762	0.04761904762	0.05555555556		0.7	#DIV/0!
**C105379**	0.04761904762	0.04761904762	0.05555555556		0.7	#DIV/0!
**C105379**	0.1428571429	0.1428571429	0.2222222222		0.4583333333	#DIV/0!
**C105447**	0.09523809524	0.09523809524	0.1111111111		0.6842105263	#DIV/0!
**C105616**	0.04761904762	0.04761904762	0.05555555556		0.7	#DIV/0!
**C105747**	0	0	0.05555555556		0	#DIV/0!
**C106660**	0.09523809524	0.09523809524	0.2222222222		0.2894736842	#DIV/0!
**C106699**	0.09523809524	0.09523809524	0.05555555556		1.473684211	#DIV/0!
**C106780**	0.04761904762	0.04761904762	0.1666666667		0.2	#DIV/0!
**C107001**	0.1428571429	0.1428571429	0.05555555556		2.333333333	#DIV/0!
**C107105**	0	0	0.05555555556		0	#DIV/0!
**C107984**	0.1428571429	0.1428571429	0.05555555556		2.333333333	#DIV/0!
**C107984**	0	0	0.05555555556		0	#DIV/0!
**C107984**	0.04761904762	0.04761904762	0		inf	#DIV/0!
**C107984**	0.09523809524	0.09523809524	0.1111111111		0.6842105263	#DIV/0!
**C107984**	0.04761904762	0.04761904762	0.1111111111		0.325	#DIV/0!
**C107984**	0.1428571429	0.1428571429	0.05555555556		2.333333333	#DIV/0!
**C107984**	0.2380952381	0.2380952381	0		inf	#DIV/0!
**C107984**	0.04761904762	0.04761904762	0.1111111111		0.325	#DIV/0!
**C107984**	0.04761904762	0.04761904762	0.05555555556		0.7	#DIV/0!
**C107984**	0.1428571429	0.1428571429	0.05555555556		2.333333333	#DIV/0!
**C107984**	0	0	0			#DIV/0!
**C107985**	0.04761904762	0.04761904762	0		inf	#DIV/0!
**C107985**	0.04761904762	0.04761904762	0		inf	#DIV/0!
**C107985**	0.1428571429	0.1428571429	0		inf	#DIV/0!
**C107985**	0.1428571429	0.1428571429	0		inf	#DIV/0!
**C107985**	0.04761904762	0.04761904762	0.1111111111		0.325	#DIV/0!
**C107985**	0	0	0.05555555556		0	#DIV/0!
**C107985**	0	0	0.05555555556		0	#DIV/0!

C107985	0.04761904762	0.04761904762	0.05555555556		0.7	#DIV/0!
C107985	0	0	0.05555555556		0	#DIV/0!
C107985	0.04761904762	0.04761904762	0.05555555556		0.7	#DIV/0!
C107986	0.1428571429	0.1428571429	0.05555555556		2.333333333	#DIV/0!
C107986	0.04761904762	0.04761904762	0		inf	#DIV/0!
C107986	0.1428571429	0.1428571429	0.1666666667		0.6666666667	#DIV/0!
C107986	0.04761904762	0.04761904762	0.05555555556		0.7	#DIV/0!
C107986	0.09523809524	0.09523809524	0.1111111111		0.6842105263	#DIV/0!
C107987	0.04761904762	0.04761904762	0.05555555556		0.7	#DIV/0!
C108783	0	0	0.05555555556		0	#DIV/0!
C110091	0.04761904762	0.04761904762	0.05555555556		0.7	#DIV/0!
C110091	0.04761904762	0.04761904762	0		inf	#DIV/0!
C110091	0.04761904762	0.04761904762	0		inf	#DIV/0!
)117498-	0.04761904762	0.04761904762	0.05555555556		0.7	#DIV/0!
C110384	0.04761904762	0.04761904762	0		inf	#DIV/0!
C112267	0.04761904762	0.04761904762	0.1111111111		0.325	#DIV/0!
C112267	0.09523809524	0.09523809524	0.05555555556		1.473684211	#DIV/0!
C112267	0.04761904762	0.04761904762	0.2222222222		0.1375	#DIV/0!
C112268	0.09523809524	0.09523809524	0.05555555556		1.473684211	#DIV/0!
C112268	0.04761904762	0.04761904762	0.1111111111		0.325	#DIV/0!
C112268	0.09523809524	0.09523809524	0		inf	#DIV/0!
C112268	0.09523809524	0.09523809524	0		inf	#DIV/0!
C112268	0.04761904762	0.04761904762	0		inf	#DIV/0!
C112268	0.09523809524	0.09523809524	0.05555555556		1.473684211	#DIV/0!
C112543	0.1428571429	0.1428571429	0		inf	#DIV/0!
C112577	0.2380952381	0.2380952381	0.05555555556		4.375	#DIV/0!
C112577	0.09523809524	0.09523809524	0		inf	#DIV/0!
C112694	0.04761904762	0.04761904762	0		inf	#DIV/0!
C114483	0.09523809524	0.09523809524	0.05555555556		1.473684211	#DIV/0!
C114483	0.09523809524	0.09523809524	0.05555555556		1.473684211	#DIV/0!
OC1436(	0.04761904762	0.04761904762	0		inf	#DIV/0!
OC1456(	0.04761904762	0.04761904762	0.05555555556		0.7	#DIV/0!
OC1457(	0.04761904762	0.04761904762	0.05555555556		0.7	#DIV/0!
OC1458	0	0	0			#DIV/0!
OC1486(	0.1428571429	0.1428571429	0		inf	#DIV/0!
OC1487(	0.1428571429	0.1428571429	0		inf	#DIV/0!
OC1493	0.1428571429	0.1428571429	0		inf	#DIV/0!
OC1496(	0.1428571429	0.1428571429	0.1666666667		0.6666666667	#DIV/0!
OC1500(	0.04761904762	0.04761904762	0.1111111111		0.325	#DIV/0!
OC1507	0	0	0.05555555556		0	#DIV/0!
OC1509	0	0	0.05555555556		0	#DIV/0!
OC1514	0	0	0.05555555556		0	#DIV/0!
OC1520	0.04761904762	0.04761904762	0		inf	#DIV/0!
OC1536(	0.04761904762	0.04761904762	0.05555555556		0.7	#DIV/0!
OC1547(	0.04761904762	0.04761904762	0		inf	#DIV/0!
OC1550(	0.09523809524	0.09523809524	0.05555555556		1.473684211	#DIV/0!
OC1572	0.09523809524	0.09523809524	0.1111111111		0.6842105263	#DIV/0!
OC1584	0.04761904762	0.04761904762	0.05555555556		0.7	#DIV/0!
OC1584	0.04761904762	0.04761904762	0.05555555556		0.7	#DIV/0!
OC1713(	0.04761904762	0.04761904762	0		inf	#DIV/0!
OC2007	0	0	0.05555555556		0	#DIV/0!

**OC20218**	0.09523809524	0.09523809524	0.05555555556		1.473684211	#DIV/0!
**OC22072**	0.1428571429	0.1428571429	0.05555555556		2.333333333	#DIV/0!
**OC22194**	0.04761904762	0.04761904762	0.1666666667		0.2	#DIV/0!
**OC25489**	0.04761904762	0.04761904762	0.1111111111		0.325	#DIV/0!
**OC25688**	0	0	0.05555555556		0	#DIV/0!
**OC25739**	0.04761904762	0.04761904762	0.05555555556		0.7	#DIV/0!
**OC28302**	0.1428571429	0.1428571429	0.05555555556		2.333333333	#DIV/0!
**OC28303**	0.2380952381	0.2380952381	0.05555555556		4.375	#DIV/0!
**OC28304**	0.04761904762	0.04761904762	0.1666666667		0.2	#DIV/0!
**OC28329**	0.04761904762	0.04761904762	0		inf	#DIV/0!
**OC28333**	0.04761904762	0.04761904762	0.05555555556		0.7	#DIV/0!
**OC28338**	0.1428571429	0.1428571429	0.05555555556		2.333333333	#DIV/0!
**OC28343**	0.09523809524	0.09523809524	0.05555555556		1.473684211	#DIV/0!
**OC28366**	0.04761904762	0.04761904762	0.05555555556		0.7	#DIV/0!
**OC28368**	0.09523809524	0.09523809524	0		inf	#DIV/0!
**OC28373**	0.04761904762	0.04761904762	0.05555555556		0.7	#DIV/0!
**OC28385**	0.04761904762	0.04761904762	0		inf	#DIV/0!
**OC28392**	0.04761904762	0.04761904762	0		inf	#DIV/0!
**OC28400**	0	0	0.1111111111		0	#DIV/0!
**OC28419**	0.04761904762	0.04761904762	0.05555555556		0.7	#DIV/0!
**OC28424**	0.04761904762	0.04761904762	0.05555555556		0.7	#DIV/0!
**OC28424**	0.04761904762	0.04761904762	0.05555555556		0.7	#DIV/0!
**OC28434**	0.04761904762	0.04761904762	0.05555555556		0.7	#DIV/0!
**OC28437**	0.09523809524	0.09523809524	0.05555555556		1.473684211	#DIV/0!
**OC28439**	0.04761904762	0.04761904762	0.1666666667		0.2	#DIV/0!
**OC28441**	0.04761904762	0.04761904762	0.05555555556		0.7	#DIV/0!
**OC28445**	0.1428571429	0.1428571429	0.1111111111		1.083333333	#DIV/0!
**OC28457**	0.1428571429	0.1428571429	0		inf	#DIV/0!
**OC28458**	0.1428571429	0.1428571429	0		inf	#DIV/0!
**OC28463**	0.04761904762	0.04761904762	0.1111111111		0.325	#DIV/0!
**OC28478**	0.1904761905	0.1904761905	0.05555555556		3.294117647	#DIV/0!
**OC28479**	0.1904761905	0.1904761905	0.05555555556		3.294117647	#DIV/0!
**OC28486**	0.04761904762	0.04761904762	0.05555555556		0.7	#DIV/0!
**OC28489**	0.04761904762	0.04761904762	0.05555555556		0.7	#DIV/0!
**OC28493**	0.04761904762	0.04761904762	0.1666666667		0.2	#DIV/0!
**OC28493**	0.04761904762	0.04761904762	0.1666666667		0.2	#DIV/0!
**OC28495**	0	0	0.05555555556		0	#DIV/0!
**OC28500**	0	0	0.05555555556		0	#DIV/0!
**OC28507**	0	0	0.05555555556		0	#DIV/0!
**OC28509**	0	0	0.05555555556		0	#DIV/0!
**OC28509**	0	0	0.05555555556		0	#DIV/0!
**OC28519**	0	0	0.05555555556		0	#DIV/0!
**OC28559**	0	0	0.05555555556		0	#DIV/0!
**OC28562**	0.04761904762	0.04761904762	0.05555555556		0.7	#DIV/0!
**OC28563**	0.04761904762	0.04761904762	0		inf	#DIV/0!
**OC28581**	0	0	0.05555555556		0	#DIV/0!
**OC28584**	0.04761904762	0.04761904762	0.05555555556		0.7	#DIV/0!
**OC28588**	0.1428571429	0.1428571429	0.05555555556		2.333333333	#DIV/0!
**OC28605**	0.04761904762	0.04761904762	0.1111111111		0.325	#DIV/0!
**OC28608**	0.09523809524	0.09523809524	0.1111111111		0.6842105263	#DIV/0!
**OC28617**	0.1428571429	0.1428571429	0.1111111111		1.083333333	#DIV/0!

**OC28617**	0.1428571429	0.1428571429	0.1666666667		0.6666666667	#DIV/0!
**OC28629**	0.1904761905	0.1904761905	0.2222222222		0.6470588235	#DIV/0!
**OC28635**	0.04761904762	0.04761904762	0		inf	#DIV/0!
**OC33896**	0.04761904762	0.04761904762	0.05555555556		0.7	#DIV/0!
**OC33905**	0.1904761905	0.1904761905	0		inf	#DIV/0!
**OC33916**	0	0	0.05555555556		0	#DIV/0!
**OC33919**	0.1428571429	0.1428571429	0		inf	#DIV/0!
**OC33926**	0.09523809524	0.09523809524	0.05555555556		1.473684211	#DIV/0!
**OC33929**	0.09523809524	0.09523809524	0.05555555556		1.473684211	#DIV/0!
**OC33952**	0.1428571429	0.1428571429	0.05555555556		2.333333333	#DIV/0!
**OC33953**	0.04761904762	0.04761904762	0.05555555556		0.7	#DIV/0!
**OC33959**	0.1904761905	0.1904761905	0.1666666667		0.9411764706	#DIV/0!
**OC33966**	0.04761904762	0.04761904762	0.1111111111		0.325	#DIV/0!
**OC33968**	0.04761904762	0.04761904762	0.1666666667		0.2	#DIV/0!
**OC33980**	0.09523809524	0.09523809524	0.05555555556		1.473684211	#DIV/0!
**OC33986**	0.04761904762	0.04761904762	0		inf	#DIV/0!
**OC33987**	0.09523809524	0.09523809524	0.05555555556		1.473684211	#DIV/0!
**OC33990**	0.04761904762	0.04761904762	0		inf	#DIV/0!
**OC33997**	0	0	0.05555555556		0	#DIV/0!
**OC34009**	0	0	0.05555555556		0	#DIV/0!
**OC34035**	0.09523809524	0.09523809524	0.1111111111		0.6842105263	#DIV/0!
**OC34051**	0.04761904762	0.04761904762	0.05555555556		0.7	#DIV/0!
**OC34305**	0.1904761905	0.1904761905	0.05555555556		3.294117647	#DIV/0!
**OC34496**	0.1904761905	0.1904761905	0.05555555556		3.294117647	#DIV/0!
**OC34916**	0.04761904762	0.04761904762	0.05555555556		0.7	#DIV/0!
**OC37444**	0.04761904762	0.04761904762	0.1111111111		0.325	#DIV/0!
**OC37519**	0	0	0.05555555556		0	#DIV/0!
**OC38824**	0.04761904762	0.04761904762	0		inf	#DIV/0!
**OC38828**	0.04761904762	0.04761904762	0		inf	#DIV/0!
**OC38843**	0.04761904762	0.04761904762	0.1111111111		0.325	#DIV/0!
**OC38878**	0.1904761905	0.1904761905	0.05555555556		3.294117647	#DIV/0!
**OC38881**	0.04761904762	0.04761904762	0.1111111111		0.325	#DIV/0!
**OC38924**	0	0	0.05555555556		0	#DIV/0!
**OC38960**	0.1428571429	0.1428571429	0.05555555556		2.333333333	#DIV/0!
**OC38964**	0.04761904762	0.04761904762	0.1111111111		0.325	#DIV/0!
**OC38970**	0.04761904762	0.04761904762	0.05555555556		0.7	#DIV/0!
**OC38976**	0	0	0.05555555556		0	#DIV/0!
**OC39070**	0.1428571429	0.1428571429	0		inf	#DIV/0!
**OC39087**	0.1428571429	0.1428571429	0.05555555556		2.333333333	#DIV/0!
**OC39093**	0.04761904762	0.04761904762	0.05555555556		0.7	#DIV/0!
**OC39132**	0.04761904762	0.04761904762	0.1111111111		0.325	#DIV/0!
**OC39219**	0.09523809524	0.09523809524	0.1111111111		0.6842105263	#DIV/0!
**OC39223**	0.1904761905	0.1904761905	0.1666666667		0.9411764706	#DIV/0!
**OC39236**	0	0	0.05555555556		0	#DIV/0!
**OC39981**	0.2380952381	0.2380952381	0.05555555556		4.375	#DIV/0!
**OC40046**	0.04761904762	0.04761904762	0.05555555556		0.7	#DIV/0!
**OC40054**	0.1428571429	0.1428571429	0.05555555556		2.333333333	#DIV/0!
**OC40055**	0.04761904762	0.04761904762	0		inf	#DIV/0!
**OC40062**	0.04761904762	0.04761904762	0		inf	#DIV/0!
**OC40062**	0.04761904762	0.04761904762	0		inf	#DIV/0!
**OC40068**	0.09523809524	0.09523809524	0		inf	#DIV/0!

OC40068	0.04761904762	0.04761904762	0.05555555556		0.7	#DIV/0!
OC40071	0.09523809524	0.09523809524	0.1111111111		0.6842105263	#DIV/0!
OC40074	0.04761904762	0.04761904762	0.1111111111		0.325	#DIV/0!
OC40080	0.1428571429	0.1428571429	0.1111111111		1.083333333	#DIV/0!
OC40086	0.04761904762	0.04761904762	0.1111111111		0.325	#DIV/0!
OC40094	0	0	0.05555555556		0	#DIV/0!
OC40112	0.1904761905	0.1904761905	0.05555555556		3.294117647	#DIV/0!
OC40117	0.04761904762	0.04761904762	0.05555555556		0.7	#DIV/0!
OC40126	0.04761904762	0.04761904762	0.05555555556		0.7	#DIV/0!
OC40131	0.04761904762	0.04761904762	0.1666666667		0.2	#DIV/0!
OC40132	0.04761904762	0.04761904762	0.2222222222		0.1375	#DIV/0!
OC40135	0.1428571429	0.1428571429	0.1666666667		0.6666666667	#DIV/0!
OC40144	0.09523809524	0.09523809524	0.1111111111		0.6842105263	#DIV/0!
OC40146	0.1428571429	0.1428571429	0.1666666667		0.6666666667	#DIV/0!
OC40147	0.04761904762	0.04761904762	0.1111111111		0.325	#DIV/0!
OC40155	0.04761904762	0.04761904762	0		inf	#DIV/0!
OC40332	0.09523809524	0.09523809524	0.2222222222		0.2894736842	#DIV/0!
OC40783	0.04761904762	0.04761904762	0.05555555556		0.7	#DIV/0!
OC41430	0.04761904762	0.04761904762	0.05555555556		0.7	#DIV/0!
OC44002	0.04761904762	0.04761904762	0.05555555556		0.7	#DIV/0!
OC44008	0.04761904762	0.04761904762	0.1111111111		0.325	#DIV/0!
OC44017	0	0	0.05555555556		0	#DIV/0!
OC44029	0.04761904762	0.04761904762	0.05555555556		0.7	#DIV/0!
OC44030	0.04761904762	0.04761904762	0.05555555556		0.7	#DIV/0!
OC44031	0.04761904762	0.04761904762	0.05555555556		0.7	#DIV/0!
OC44034	0.04761904762	0.04761904762	0		inf	#DIV/0!
OC44057	0.04761904762	0.04761904762	0.1111111111		0.325	#DIV/0!
OC44070	0.1428571429	0.1428571429	0		inf	#DIV/0!
OC44089	0	0	0.05555555556		0	#DIV/0!
OC44089	0.09523809524	0.09523809524	0.2222222222		0.2894736842	#DIV/0!
OC44091	0	0	0.05555555556		0	#DIV/0!
OC44098	0.1428571429	0.1428571429	0.1111111111		1.083333333	#DIV/0!
OC44105	0	0	0.05555555556		0	#DIV/0!
OC44108	0.09523809524	0.09523809524	0.1666666667		0.4210526316	#DIV/0!
OC44108	0.04761904762	0.04761904762	0		inf	#DIV/0!
OC44108	0.04761904762	0.04761904762	0		inf	#DIV/0!
OC44120	0.04761904762	0.04761904762	0.2222222222		0.1375	#DIV/0!
OC44124	0.04761904762	0.04761904762	0.1111111111		0.325	#DIV/0!
OC44145	0.04761904762	0.04761904762	0		inf	#DIV/0!
OC44166	0.1428571429	0.1428571429	0.05555555556		2.333333333	#DIV/0!
OC44202	0	0	0.1111111111		0	#DIV/0!
OC44213	0	0	0.05555555556		0	#DIV/0!
OC44249	0.04761904762	0.04761904762	0.1666666667		0.2	#DIV/0!
OC49412	0	0	0.05555555556		0	#DIV/0!
OC49725	0	0	0.05555555556		0	#DIV/0!
OC5114	0.09523809524	0.09523809524	0.05555555556		1.473684211	#DIV/0!
OC54147	0.04761904762	0.04761904762	0.1666666667		0.2	#DIV/0!
OC54147	0.1428571429	0.1428571429	0.1666666667		0.6666666667	#DIV/0!
OC55011	0.04761904762	0.04761904762	0.05555555556		0.7	#DIV/0!
OC55420	0.04761904762	0.04761904762	0		inf	#DIV/0!
OC55424	0.1904761905	0.1904761905	0.05555555556		3.294117647	#DIV/0!

**OC57453**	0.04761904762	0.04761904762	0.1111111111		0.325	#DIV/0!
**OC60672**	0.04761904762	0.04761904762	0		inf	#DIV/0!
**OC61303**	0.04761904762	0.04761904762	0		inf	#DIV/0!
**OC61320**	0.09523809524	0.09523809524	0.05555555556		1.473684211	#DIV/0!
**OC61326**	0.1904761905	0.1904761905	0.1111111111		1.529411765	#DIV/0!
**OC64130**	0.09523809524	0.09523809524	0		inf	#DIV/0!
**OC64174**	0.04761904762	0.04761904762	0.05555555556		0.7	#DIV/0!
**OC64213**	0.2380952381	0.2380952381	0.1111111111		2.03125	#DIV/0!
**OC64236**	0.1428571429	0.1428571429	0		inf	#DIV/0!
**OC64230**	0.04761904762	0.04761904762	0.05555555556		0.7	#DIV/0!
**OC64248**	0.09523809524	0.09523809524	0		inf	#DIV/0!
**OC64264**	0	0	0.1111111111		0	#DIV/0!
**OC64284**	0.04761904762	0.04761904762	0.1111111111		0.325	#DIV/0!
**OC64292**	0.1904761905	0.1904761905	0.05555555556		3.294117647	#DIV/0!
**OC64294**	0.04761904762	0.04761904762	0.05555555556		0.7	#DIV/0!
**OC64307**	0	0	0.05555555556		0	#DIV/0!
**OC64320**	0.09523809524	0.09523809524	0.05555555556		1.473684211	#DIV/0!
**OC64333**	0.04761904762	0.04761904762	0.05555555556		0.7	#DIV/0!
**OC64338**	0	0	0.05555555556		0	#DIV/0!
**OC64340**	0.1904761905	0.1904761905	0.05555555556		3.294117647	#DIV/0!
**OC64344**	0.04761904762	0.04761904762	0.05555555556		0.7	#DIV/0!
**OC64371**	0.1904761905	0.1904761905	0.05555555556		3.294117647	#DIV/0!
**OC64380**	0.04761904762	0.04761904762	0.05555555556		0.7	#DIV/0!
**OC64409**	0.09523809524	0.09523809524	0.05555555556		1.473684211	#DIV/0!
**OC64418**	0.04761904762	0.04761904762	0.05555555556		0.7	#DIV/0!
**OC64421**	0.1428571429	0.1428571429	0		inf	#DIV/0!
**OC64428**	0.04761904762	0.04761904762	0		inf	#DIV/0!
**OC64455**	0.04761904762	0.04761904762	0.05555555556		0.7	#DIV/0!
**OC64463**	0.09523809524	0.09523809524	0.05555555556		1.473684211	#DIV/0!
**OC64465**	0.04761904762	0.04761904762	0.05555555556		0.7	#DIV/0!
**OC64466**	0.09523809524	0.09523809524	0.05555555556		1.473684211	#DIV/0!
**OC64493**	0.04761904762	0.04761904762	0		inf	#DIV/0!
**OC64517**	0.04761904762	0.04761904762	0.05555555556		0.7	#DIV/0!
**OC64548**	0.04761904762	0.04761904762	0.05555555556		0.7	#DIV/0!
**OC64551**	0	0	0.05555555556		0	#DIV/0!
**OC64575**	0.04761904762	0.04761904762	0.05555555556		0.7	#DIV/0!
**OC64596**	0	0	0			#DIV/0!
**OC64603**	0	0	0			#DIV/0!
**OC64621**	0.2857142857	0.2857142857	0.1111111111		2.6	#DIV/0!
**OC64647**	0.04761904762	0.04761904762	0.1111111111		0.325	#DIV/0!
**OC64658**	0.04761904762	0.04761904762	0.2222222222		0.1375	#DIV/0!
**OC64662**	0.04761904762	0.04761904762	0.05555555556		0.7	#DIV/0!
**OC64665**	0.04761904762	0.04761904762	0.1111111111		0.325	#DIV/0!
**OC64673**	0	0	0.05555555556		0	#DIV/0!
**OC64676**	0.04761904762	0.04761904762	0.1666666667		0.2	#DIV/0!
**OC64690**	0.1904761905	0.1904761905	0.1111111111		1.529411765	#DIV/0!
**OC64693**	0.04761904762	0.04761904762	0.05555555556		0.7	#DIV/0!
**OC64707**	0.1428571429	0.1428571429	0		inf	#DIV/0!
**OC64898**	0.04761904762	0.04761904762	0.05555555556		0.7	#DIV/0!
**OC64935**	0.09523809524	0.09523809524	0.1111111111		0.6842105263	#DIV/0!
**OC65022**	0.1428571429	0.1428571429	0.1666666667		0.6666666667	#DIV/0!

**OC65133**	0.09523809524	0.09523809524	0		inf	#DIV/0!
**OC65227**	0.04761904762	0.04761904762	0.1111111111		0.325	#DIV/0!
**OC65308**	0.09523809524	0.09523809524	0.1666666667		0.4210526316	#DIV/0!
**OC65316**	0.04761904762	0.04761904762	0.05555555556		0.7	#DIV/0!
**OC65351**	0.09523809524	0.09523809524	0.05555555556		1.473684211	#DIV/0!
**OC65365**	0.1428571429	0.1428571429	0		inf	#DIV/0!
**OC65371**	0.09523809524	0.09523809524	0.05555555556		1.473684211	#DIV/0!
**OC65478**	0.04761904762	0.04761904762	0		inf	#DIV/0!
**OC65484**	0	0	0.05555555556		0	#DIV/0!
**OC69224**	0.04761904762	0.04761904762	0		inf	#DIV/0!
**OC72775**	0.04761904762	0.04761904762	0.05555555556		0.7	#DIV/0!
**OC72789**	0.09523809524	0.09523809524	0		inf	#DIV/0!
**OC72802**	0.04761904762	0.04761904762	0.1111111111		0.325	#DIV/0!
**OC72809**	0	0	0.05555555556		0	#DIV/0!
**OC72815**	0.2380952381	0.2380952381	0.05555555556		4.375	#DIV/0!
**OC72839**	0	0	0.05555555556		0	#DIV/0!
**OC72848**	0.04761904762	0.04761904762	0.05555555556		0.7	#DIV/0!
**OC72855**	0.09523809524	0.09523809524	0.05555555556		1.473684211	#DIV/0!
**OC72861**	0	0	0.1111111111		0	#DIV/0!
**OC72867**	0.09523809524	0.09523809524	0.2222222222		0.2894736842	#DIV/0!
**OC72871**	0.04761904762	0.04761904762	0.1111111111		0.325	#DIV/0!
**OC72873**	0.04761904762	0.04761904762	0.05555555556		0.7	#DIV/0!
**OC72874**	0.09523809524	0.09523809524	0.05555555556		1.473684211	#DIV/0!
**OC72875**	0.04761904762	0.04761904762	0.05555555556		0.7	#DIV/0!
**OC72898**	0.1428571429	0.1428571429	0.05555555556		2.333333333	#DIV/0!
**OC72908**	0.04761904762	0.04761904762	0		inf	#DIV/0!
**OC72921**	0	0	0.05555555556		0	#DIV/0!
**OC72925**	0	0	0.05555555556		0	#DIV/0!
**OC72929**	0.04761904762	0.04761904762	0.05555555556		0.7	#DIV/0!
**OC72929**	0.1428571429	0.1428571429	0.1666666667		0.6666666667	#DIV/0!
**OC72955**	0	0	0.05555555556		0	#DIV/0!
**OC72965**	0.09523809524	0.09523809524	0.2222222222		0.2894736842	#DIV/0!
**OC72968**	0.04761904762	0.04761904762	0		inf	#DIV/0!
**OC72973**	0.09523809524	0.09523809524	0.1111111111		0.6842105263	#DIV/0!
**OC72973**	0.04761904762	0.04761904762	0.1666666667		0.2	#DIV/0!
**OC72981**	0.1428571429	0.1428571429	0.05555555556		2.333333333	#DIV/0!
**OC72986**	0.1428571429	0.1428571429	0		inf	#DIV/0!
**OC72987**	0	0	0.05555555556		0	#DIV/0!
**OC72993**	0.04761904762	0.04761904762	0.05555555556		0.7	#DIV/0!
**OC72996**	0.1428571429	0.1428571429	0.1111111111		1.083333333	#DIV/0!
**OC72996**	0	0	0.05555555556		0	#DIV/0!
**OC72997**	0.04761904762	0.04761904762	0.05555555556		0.7	#DIV/0!
**OC73009**	0.09523809524	0.09523809524	0.05555555556		1.473684211	#DIV/0!
**OC73010**	0.04761904762	0.04761904762	0.05555555556		0.7	#DIV/0!
**OC73010**	0.04761904762	0.04761904762	0		inf	#DIV/0!
**OC73018**	0.04761904762	0.04761904762	0		inf	#DIV/0!
**OC73023**	0.04761904762	0.04761904762	0.2222222222		0.1375	#DIV/0!
**OC73026**	0	0	0.05555555556		0	#DIV/0!
**OC73033**	0.04761904762	0.04761904762	0.2222222222		0.1375	#DIV/0!
**OC73066**	0	0	0.1111111111		0	#DIV/0!
**OC73115**	0.04761904762	0.04761904762	0		inf	#DIV/0!

OC7322(	0.04761904762	0.04761904762	0.05555555556		0.7	#DIV/0!
OC7999	0.04761904762	0.04761904762	0.1111111111		0.325	#DIV/0!
OC9024	0.09523809524	0.09523809524	0.05555555556		1.473684211	#DIV/0!
OC9137	0.04761904762	0.04761904762	0.1111111111		0.325	#DIV/0!
OC9145	0.04761904762	0.04761904762	0.05555555556		0.7	#DIV/0!
OC9154	0.1428571429	0.1428571429	0		inf	#DIV/0!
OC9342	0.09523809524	0.09523809524	0.1111111111		0.6842105263	#DIV/0!
OC9346	0	0	0.05555555556		0	#DIV/0!
OH12CF	0.04761904762	0.04761904762	0.05555555556		0.7	#DIV/0!
LONP1	0.1428571429	0.1428571429	0.05555555556		2.333333333	#DIV/0!
LONP2	0.04761904762	0.04761904762	0		inf	#DIV/0!
LONRF1	0.09523809524	0.09523809524	0.1111111111		0.6842105263	#DIV/0!
LONRF2	0	0	0.05555555556		0	#DIV/0!
LOX	0.04761904762	0.04761904762	0		inf	#DIV/0!
LOXHD1	0.04761904762	0.04761904762	0.05555555556		0.7	#DIV/0!
LOXL1	0.04761904762	0.04761904762	0.05555555556		0.7	#DIV/0!
OXL1-AS	0.04761904762	0.04761904762	0.05555555556		0.7	#DIV/0!
LOXL2	0.04761904762	0.04761904762	0.1111111111		0.325	#DIV/0!
LOXL3	0	0	0.05555555556		0	#DIV/0!
LOXL4	0.1428571429	0.1428571429	0		inf	#DIV/0!
LPAR1	0.04761904762	0.04761904762	0.05555555556		0.7	#DIV/0!
LPAR2	0.1428571429	0.1428571429	0.1111111111		1.083333333	#DIV/0!
LPAR3	0.04761904762	0.04761904762	0.05555555556		0.7	#DIV/0!
LPAR5	0.04761904762	0.04761904762	0.1111111111		0.325	#DIV/0!
LPCAT1	0	0	0.1111111111		0	#DIV/0!
LPCAT2	0.04761904762	0.04761904762	0		inf	#DIV/0!
LPCAT3	0.04761904762	0.04761904762	0.1111111111		0.325	#DIV/0!
LPGAT1	0.1428571429	0.1428571429	0		inf	#DIV/0!
GAT1-A	0.1428571429	0.1428571429	0		inf	#DIV/0!
LPIN1	0	0	0.05555555556		0	#DIV/0!
LPIN2	0.09523809524	0.09523809524	0		inf	#DIV/0!
LPIN3	0.1428571429	0.1428571429	0.1666666667		0.6666666667	#DIV/0!
LPL	0.09523809524	0.09523809524	0.1111111111		0.6842105263	#DIV/0!
LPO	0.09523809524	0.09523809524	0		inf	#DIV/0!
LPP	0.09523809524	0.09523809524	0.1111111111		0.6842105263	#DIV/0!
LPP-AS1	0.09523809524	0.09523809524	0.1111111111		0.6842105263	#DIV/0!
LPP-AS2	0.09523809524	0.09523809524	0.1111111111		0.6842105263	#DIV/0!
LRAT	0	0	0.05555555556		0	#DIV/0!
LRATD1	0	0	0.05555555556		0	#DIV/0!
LRATD2	0.1428571429	0.1428571429	0.1111111111		1.083333333	#DIV/0!
LRBA	0	0	0.05555555556		0	#DIV/0!
LRCH3	0.1428571429	0.1428571429	0.05555555556		2.333333333	#DIV/0!
LRCH4	0.04761904762	0.04761904762	0.05555555556		0.7	#DIV/0!
LRCOL1	0.09523809524	0.09523809524	0.05555555556		1.473684211	#DIV/0!
LRFN1	0.04761904762	0.04761904762	0.05555555556		0.7	#DIV/0!
LRFN2	0	0	0.05555555556		0	#DIV/0!
LRFN3	0.04761904762	0.04761904762	0.05555555556		0.7	#DIV/0!
LRFN5	0.04761904762	0.04761904762	0		inf	#DIV/0!
LRG1	0.09523809524	0.09523809524	0.05555555556		1.473684211	#DIV/0!
LRGUK	0.04761904762	0.04761904762	0.05555555556		0.7	#DIV/0!
LRIF1	0.04761904762	0.04761904762	0		inf	#DIV/0!

LRIG1	0.04761904762	0.04761904762	0		inf	#DIV/0!
LRIG2	0.04761904762	0.04761904762	0.05555555556		0.7	#DIV/0!
LRIG3	0.09523809524	0.09523809524	0		inf	#DIV/0!
LRIT1	0.1428571429	0.1428571429	0		inf	#DIV/0!
LRIT2	0.1428571429	0.1428571429	0		inf	#DIV/0!
LRIT3	0	0	0.05555555556		0	#DIV/0!
LRMDA	0.1904761905	0.1904761905	0.1111111111		1.529411765	#DIV/0!
LRMP	0.04761904762	0.04761904762	0.05555555556		0.7	#DIV/0!
LRP1	0.1428571429	0.1428571429	0.05555555556		2.333333333	#DIV/0!
LRP1-AS	0.1428571429	0.1428571429	0.05555555556		2.333333333	#DIV/0!
LRP10	0	0	0.05555555556		0	#DIV/0!
LRP11	0	0	0			#DIV/0!
LRP12	0.1904761905	0.1904761905	0.1111111111		1.529411765	#DIV/0!
LRP1B	0	0	0.05555555556		0	#DIV/0!
LRP2	0.04761904762	0.04761904762	0.05555555556		0.7	#DIV/0!
LRP2BP	0	0	0.05555555556		0	#DIV/0!
LRP3	0.04761904762	0.04761904762	0.05555555556		0.7	#DIV/0!
LRP4	0	0	0.05555555556		0	#DIV/0!
LRP4-AS	0	0	0.05555555556		0	#DIV/0!
LRP6	0.04761904762	0.04761904762	0.05555555556		0.7	#DIV/0!
LRP8	0.04761904762	0.04761904762	0.05555555556		0.7	#DIV/0!
LRPAP1	0.04761904762	0.04761904762	0		inf	#DIV/0!
LRPPRC	0	0	0.05555555556		0	#DIV/0!
LRR1	0.04761904762	0.04761904762	0		inf	#DIV/0!
LRRC1	0.04761904762	0.04761904762	0		inf	#DIV/0!
LRRC10	0.09523809524	0.09523809524	0.1111111111		0.6842105263	#DIV/0!
LRRC14	0.04761904762	0.04761904762	0.1666666667		0.2	#DIV/0!
LRRC14I	0	0	0.05555555556		0	#DIV/0!
LRRC15	0.09523809524	0.09523809524	0.05555555556		1.473684211	#DIV/0!
LRRC17	0.04761904762	0.04761904762	0.05555555556		0.7	#DIV/0!
LRRC18	0.09523809524	0.09523809524	0.05555555556		1.473684211	#DIV/0!
LRRC19	0.04761904762	0.04761904762	0.05555555556		0.7	#DIV/0!
LRRC20	0.09523809524	0.09523809524	0		inf	#DIV/0!
LRRC23	0.04761904762	0.04761904762	0.1111111111		0.325	#DIV/0!
LRRC24	0.04761904762	0.04761904762	0.1666666667		0.2	#DIV/0!
LRRC25	0.1428571429	0.1428571429	0.1111111111		1.083333333	#DIV/0!
LRRC26	0.1428571429	0.1428571429	0.05555555556		2.333333333	#DIV/0!
LRRC27	0.2380952381	0.2380952381	0		inf	#DIV/0!
LRRC28	0.04761904762	0.04761904762	0.05555555556		0.7	#DIV/0!
LRRC29	0.1428571429	0.1428571429	0.05555555556		2.333333333	#DIV/0!
LRRC3	0.04761904762	0.04761904762	0.1111111111		0.325	#DIV/0!
RRC3-D	0.04761904762	0.04761904762	0.1111111111		0.325	#DIV/0!
LRRC30	0.09523809524	0.09523809524	0		inf	#DIV/0!
LRRC31	0.1904761905	0.1904761905	0.05555555556		3.294117647	#DIV/0!
LRRC34	0.1904761905	0.1904761905	0.05555555556		3.294117647	#DIV/0!
LRRC36	0.1428571429	0.1428571429	0.05555555556		2.333333333	#DIV/0!
LRRC37A	0.1428571429	0.1428571429	0		inf	#DIV/0!
RC37A1	0.09523809524	0.09523809524	0		inf	#DIV/0!
RRC37A	0.2380952381	0.2380952381	0		inf	#DIV/0!
RRC37A	0.04761904762	0.04761904762	0		inf	#DIV/0!
RC37A-	0.1428571429	0.1428571429	0		inf	#DIV/0!

RRC37A:	0.04761904762	0.04761904762	0.05555555556		0.7	#DIV/0!
RRC37A	0.09523809524	0.09523809524	0.1111111111		0.6842105263	#DIV/0!
RRC37A	0.09523809524	0.09523809524	0		inf	#DIV/0!
LRRC37I	0	0	0			#DIV/0!
LRRC38	0.04761904762	0.04761904762	0.1111111111		0.325	#DIV/0!
LRRC39	0.04761904762	0.04761904762	0.05555555556		0.7	#DIV/0!
LRRC3B	0.04761904762	0.04761904762	0		inf	#DIV/0!
LRRC3C	0.09523809524	0.09523809524	0		inf	#DIV/0!
LRRC4	0.04761904762	0.04761904762	0.05555555556		0.7	#DIV/0!
LRRC40	0.04761904762	0.04761904762	0.05555555556		0.7	#DIV/0!
LRRC41	0.04761904762	0.04761904762	0.05555555556		0.7	#DIV/0!
LRRC42	0.04761904762	0.04761904762	0.05555555556		0.7	#DIV/0!
LRRC43	0.04761904762	0.04761904762	0.1111111111		0.325	#DIV/0!
LRRC45	0.04761904762	0.04761904762	0		inf	#DIV/0!
LRRC46	0.1428571429	0.1428571429	0		inf	#DIV/0!
LRRC47	0	0	0.05555555556		0	#DIV/0!
LRRC49	0.04761904762	0.04761904762	0.05555555556		0.7	#DIV/0!
LRRC4B	0.09523809524	0.09523809524	0.1111111111		0.6842105263	#DIV/0!
LRRC52	0.1428571429	0.1428571429	0		inf	#DIV/0!
RRC52-A	0.1428571429	0.1428571429	0		inf	#DIV/0!
LRRC53	0.04761904762	0.04761904762	0.05555555556		0.7	#DIV/0!
LRRC56	0.04761904762	0.04761904762	0		inf	#DIV/0!
LRRC58	0.09523809524	0.09523809524	0.05555555556		1.473684211	#DIV/0!
LRRC59	0.09523809524	0.09523809524	0		inf	#DIV/0!
LRRC6	0.04761904762	0.04761904762	0.1111111111		0.325	#DIV/0!
LRRC61	0.09523809524	0.09523809524	0.05555555556		1.473684211	#DIV/0!
LRRC66	0.2380952381	0.2380952381	0.05555555556		4.375	#DIV/0!
LRRC69	0.1904761905	0.1904761905	0.1111111111		1.529411765	#DIV/0!
LRRC7	0.04761904762	0.04761904762	0.05555555556		0.7	#DIV/0!
LRRC70	0.04761904762	0.04761904762	0		inf	#DIV/0!
LRRC71	0.1428571429	0.1428571429	0.05555555556		2.333333333	#DIV/0!
LRRC72	0.04761904762	0.04761904762	0.2222222222		0.1375	#DIV/0!
LRRC73	0.04761904762	0.04761904762	0.05555555556		0.7	#DIV/0!
LRRC74I	0.04761904762	0.04761904762	0.05555555556		0.7	#DIV/0!
LRRC75A	0.04761904762	0.04761904762	0.05555555556		0.7	#DIV/0!
LRRC75I	0.04761904762	0.04761904762	0.1111111111		0.325	#DIV/0!
LRRC77I	0.1428571429	0.1428571429	0.05555555556		2.333333333	#DIV/0!
LRRC8A	0.09523809524	0.09523809524	0.05555555556		1.473684211	#DIV/0!
LRRC8B	0.04761904762	0.04761904762	0.05555555556		0.7	#DIV/0!
LRRC8C	0.04761904762	0.04761904762	0.05555555556		0.7	#DIV/0!
RRC8C-I	0.04761904762	0.04761904762	0.05555555556		0.7	#DIV/0!
LRRC8D	0.04761904762	0.04761904762	0.05555555556		0.7	#DIV/0!
LRRC8E	0.1428571429	0.1428571429	0.05555555556		2.333333333	#DIV/0!
LRRCC1	0.1428571429	0.1428571429	0.1111111111		1.083333333	#DIV/0!
LRRD1	0.04761904762	0.04761904762	0.1111111111		0.325	#DIV/0!
LRRFIP1	0	0	0.05555555556		0	#DIV/0!
LRRFIP2	0.04761904762	0.04761904762	0		inf	#DIV/0!
LRRIQ1	0.04761904762	0.04761904762	0.05555555556		0.7	#DIV/0!
LRRIQ3	0.04761904762	0.04761904762	0.05555555556		0.7	#DIV/0!
LRRIQ4	0.1904761905	0.1904761905	0.05555555556		3.294117647	#DIV/0!
LRRK1	0.04761904762	0.04761904762	0.05555555556		0.7	#DIV/0!

LRRK2	0.04761904762	0.04761904762	0.05555555556		0.7	#DIV/0!
LRRN1	0.04761904762	0.04761904762	0		inf	#DIV/0!
LRRN2	0.2380952381	0.2380952381	0		inf	#DIV/0!
LRRN3	0.04761904762	0.04761904762	0.05555555556		0.7	#DIV/0!
LRRN4	0.1904761905	0.1904761905	0.05555555556		3.294117647	#DIV/0!
LRRTM1	0	0	0.05555555556		0	#DIV/0!
LRRTM2	0.04761904762	0.04761904762	0		inf	#DIV/0!
LRRTM3	0.1428571429	0.1428571429	0		inf	#DIV/0!
LRRTM4	0	0	0.05555555556		0	#DIV/0!
LRSAM1	0.09523809524	0.09523809524	0.05555555556		1.473684211	#DIV/0!
LRTM1	0.04761904762	0.04761904762	0		inf	#DIV/0!
LRTM2	0.04761904762	0.04761904762	0.1666666667		0.2	#DIV/0!
LRWD1	0.04761904762	0.04761904762	0.05555555556		0.7	#DIV/0!
LSAMP	0.09523809524	0.09523809524	0.05555555556		1.473684211	#DIV/0!
SAMP-A	0.09523809524	0.09523809524	0.05555555556		1.473684211	#DIV/0!
LSG1	0.09523809524	0.09523809524	0.05555555556		1.473684211	#DIV/0!
LSINCT5	0	0	0.1111111111		0	#DIV/0!
LSM1	0.09523809524	0.09523809524	0.1111111111		0.6842105263	#DIV/0!
LSM10	0.04761904762	0.04761904762	0.05555555556		0.7	#DIV/0!
LSM11	0.04761904762	0.04761904762	0.05555555556		0.7	#DIV/0!
LSM12	0.09523809524	0.09523809524	0		inf	#DIV/0!
LSM14A	0.04761904762	0.04761904762	0.05555555556		0.7	#DIV/0!
LSM14B	0.1428571429	0.1428571429	0.2222222222		0.4583333333	#DIV/0!
LSM2	0.04761904762	0.04761904762	0		inf	#DIV/0!
LSM3	0.04761904762	0.04761904762	0		inf	#DIV/0!
LSM4	0.1428571429	0.1428571429	0.1111111111		1.083333333	#DIV/0!
LSM5	0.04761904762	0.04761904762	0.1666666667		0.2	#DIV/0!
LSM6	0	0	0.05555555556		0	#DIV/0!
LSM7	0.09523809524	0.09523809524	0.1111111111		0.6842105263	#DIV/0!
LSM8	0.09523809524	0.09523809524	0.05555555556		1.473684211	#DIV/0!
LSMEM1	0.04761904762	0.04761904762	0.05555555556		0.7	#DIV/0!
LSMEM2	0.09523809524	0.09523809524	0		inf	#DIV/0!
LSP1	0.04761904762	0.04761904762	0		inf	#DIV/0!
LSP1P3	0.04761904762	0.04761904762	0.05555555556		0.7	#DIV/0!
LSP1P4	0.04761904762	0.04761904762	0.05555555556		0.7	#DIV/0!
LSP1P5	0.1428571429	0.1428571429	0.05555555556		2.333333333	#DIV/0!
LSR	0.04761904762	0.04761904762	0.05555555556		0.7	#DIV/0!
LSS	0.04761904762	0.04761904762	0.1111111111		0.325	#DIV/0!
LST1	0.04761904762	0.04761904762	0		inf	#DIV/0!
LTA	0.04761904762	0.04761904762	0		inf	#DIV/0!
LTA4H	0.04761904762	0.04761904762	0.05555555556		0.7	#DIV/0!
LTB	0.04761904762	0.04761904762	0		inf	#DIV/0!
LTBP4	0.04761904762	0.04761904762	0.05555555556		0.7	#DIV/0!
LTBR	0.04761904762	0.04761904762	0.1111111111		0.325	#DIV/0!
LTC4S	0.09523809524	0.09523809524	0.05555555556		1.473684211	#DIV/0!
LTN1	0.04761904762	0.04761904762	0.1111111111		0.325	#DIV/0!
LUARIS	0.04761904762	0.04761904762	0.1666666667		0.2	#DIV/0!
LUC7L	0.04761904762	0.04761904762	0.2222222222		0.1375	#DIV/0!
LUC7L2	0.04761904762	0.04761904762	0.05555555556		0.7	#DIV/0!
LUC7L3	0.09523809524	0.09523809524	0		inf	#DIV/0!
LUCAT1	0.04761904762	0.04761904762	0		inf	#DIV/0!

**LUM**	0.04761904762	0.04761904762	0.05555555556		0.7	#DIV/0!
**LUNAR1**	0.04761904762	0.04761904762	0.05555555556		0.7	#DIV/0!
**LURAP1**	0.04761904762	0.04761904762	0.05555555556		0.7	#DIV/0!
**URAP1L**	0.09523809524	0.09523809524	0.05555555556		1.473684211	#DIV/0!
**RAP1L-A**	0.09523809524	0.09523809524	0.05555555556		1.473684211	#DIV/0!
**LUZP1**	0.04761904762	0.04761904762	0.1111111111		0.325	#DIV/0!
**LUZP6**	0.04761904762	0.04761904762	0.05555555556		0.7	#DIV/0!
**LVRN**	0.04761904762	0.04761904762	0		inf	#DIV/0!
**LXN**	0.1428571429	0.1428571429	0.05555555556		2.333333333	#DIV/0!
**LY6D**	0.04761904762	0.04761904762	0.1666666667		0.2	#DIV/0!
**LY6E**	0.04761904762	0.04761904762	0.1666666667		0.2	#DIV/0!
**LY6E-DT**	0.04761904762	0.04761904762	0.1666666667		0.2	#DIV/0!
**LY6G5B**	0.04761904762	0.04761904762	0		inf	#DIV/0!
**LY6G5C**	0.04761904762	0.04761904762	0		inf	#DIV/0!
**LY6G6C**	0.04761904762	0.04761904762	0		inf	#DIV/0!
**LY6G6D**	0.04761904762	0.04761904762	0		inf	#DIV/0!
**LY6G6E**	0.04761904762	0.04761904762	0		inf	#DIV/0!
**LY6G6F**	0.04761904762	0.04761904762	0		inf	#DIV/0!
**G6F-LY6**	0.04761904762	0.04761904762	0		inf	#DIV/0!
**LY6H**	0.04761904762	0.04761904762	0.1666666667		0.2	#DIV/0!
**LY6K**	0.04761904762	0.04761904762	0.1666666667		0.2	#DIV/0!
**LY6L**	0.04761904762	0.04761904762	0.1666666667		0.2	#DIV/0!
**LY75**	0	0	0.05555555556		0	#DIV/0!
**Y75-CD3**	0	0	0.05555555556		0	#DIV/0!
**LY9**	0.1428571429	0.1428571429	0		inf	#DIV/0!
**LY96**	0.1904761905	0.1904761905	0.1111111111		1.529411765	#DIV/0!
**LYG1**	0	0	0.05555555556		0	#DIV/0!
**LYG2**	0	0	0.05555555556		0	#DIV/0!
**LYL1**	0.1428571429	0.1428571429	0.1111111111		1.083333333	#DIV/0!
**LYN**	0.1428571429	0.1428571429	0.1111111111		1.083333333	#DIV/0!
**LYNX1**	0.04761904762	0.04761904762	0.1666666667		0.2	#DIV/0!
**X1-SLU**	0.04761904762	0.04761904762	0.1666666667		0.2	#DIV/0!
**LYPD1**	0	0	0.05555555556		0	#DIV/0!
**LYPD2**	0.04761904762	0.04761904762	0.1666666667		0.2	#DIV/0!
**LYPD3**	0.09523809524	0.09523809524	0.05555555556		1.473684211	#DIV/0!
**LYPD4**	0.04761904762	0.04761904762	0.05555555556		0.7	#DIV/0!
**LYPD5**	0.09523809524	0.09523809524	0.05555555556		1.473684211	#DIV/0!
**LYPD6**	0	0	0.05555555556		0	#DIV/0!
**LYPD6B**	0	0	0.05555555556		0	#DIV/0!
**LYPD8**	0.1428571429	0.1428571429	0		inf	#DIV/0!
**LYPLA1**	0.1428571429	0.1428571429	0.1111111111		1.083333333	#DIV/0!
**LYPLA2**	0.04761904762	0.04761904762	0.1111111111		0.325	#DIV/0!
**YPLA2P**	0.1428571429	0.1428571429	0.05555555556		2.333333333	#DIV/0!
**YPLAL**	0.1428571429	0.1428571429	0.05555555556		2.333333333	#DIV/0!
**PLAL1-A**	0.1428571429	0.1428571429	0.05555555556		2.333333333	#DIV/0!
**PLAL1-**	0.1428571429	0.1428571429	0.05555555556		2.333333333	#DIV/0!
**LYRM1**	0.04761904762	0.04761904762	0		inf	#DIV/0!
**LYRM2**	0	0	0			#DIV/0!
**LYRM7**	0.04761904762	0.04761904762	0		inf	#DIV/0!
**LYRM9**	0.04761904762	0.04761904762	0.05555555556		0.7	#DIV/0!
**YSMD1**	0.1904761905	0.1904761905	0.05555555556		3.294117647	#DIV/0!

LYSMD2	0.04761904762	0.04761904762	0.05555555556		0.7	#DIV/0!
LYSMD3	0.04761904762	0.04761904762	0		inf	#DIV/0!
LYSMD4	0.04761904762	0.04761904762	0.05555555556		0.7	#DIV/0!
LYST	0.1428571429	0.1428571429	0.05555555556		2.333333333	#DIV/0!
LYZ	0.09523809524	0.09523809524	0.1111111111		0.6842105263	#DIV/0!
LYZL1	0.09523809524	0.09523809524	0.2222222222		0.2894736842	#DIV/0!
LYZL2	0.09523809524	0.09523809524	0.2222222222		0.2894736842	#DIV/0!
LYZL4	0.04761904762	0.04761904762	0		inf	#DIV/0!
LYZL6	0.09523809524	0.09523809524	0		inf	#DIV/0!
LZIC	0	0	0.1111111111		0	#DIV/0!
LZTR1	0.04761904762	0.04761904762	0.05555555556		0.7	#DIV/0!
LZTS1	0.09523809524	0.09523809524	0.1111111111		0.6842105263	#DIV/0!
ZTS1-AS	0.09523809524	0.09523809524	0.1111111111		0.6842105263	#DIV/0!
LZTS2	0.1428571429	0.1428571429	0		inf	#DIV/0!
LZTS3	0.1904761905	0.1904761905	0.05555555556		3.294117647	#DIV/0!
M1AP	0	0	0.05555555556		0	#DIV/0!
M6PR	0.04761904762	0.04761904762	0.1111111111		0.325	#DIV/0!
MAATS1	0.09523809524	0.09523809524	0.05555555556		1.473684211	#DIV/0!
IAB21L	0	0	0.05555555556		0	#DIV/0!
IAB21L	0.04761904762	0.04761904762	0.05555555556		0.7	#DIV/0!
IAB21L	0	0	0.05555555556		0	#DIV/0!
MACC1	0.04761904762	0.04761904762	0.2222222222		0.1375	#DIV/0!
ACC1-A	0.04761904762	0.04761904762	0.2222222222		0.1375	#DIV/0!
MACF1	0.04761904762	0.04761904762	0.05555555556		0.7	#DIV/0!
MACO1	0.04761904762	0.04761904762	0.1111111111		0.325	#DIV/0!
IACROD	0.1904761905	0.1904761905	0.1111111111		1.529411765	#DIV/0!
CROD2-	0.1904761905	0.1904761905	0.1111111111		1.529411765	#DIV/0!
CROD2-	0.1904761905	0.1904761905	0.1111111111		1.529411765	#DIV/0!
MAD1L1	0.04761904762	0.04761904762	0.1666666667		0.2	#DIV/0!
MAD2L1	0	0	0.05555555556		0	#DIV/0!
AD2L1B	0.04761904762	0.04761904762	0.05555555556		0.7	#DIV/0!
MAD2L2	0.04761904762	0.04761904762	0.1111111111		0.325	#DIV/0!
ADCAM	0.09523809524	0.09523809524	0.1111111111		0.6842105263	#DIV/0!
MADD	0	0	0.05555555556		0	#DIV/0!
MAEA	0.04761904762	0.04761904762	0		inf	#DIV/0!
MAEL	0.1428571429	0.1428571429	0		inf	#DIV/0!
MAF	0.09523809524	0.09523809524	0		inf	#DIV/0!
MAF1	0.04761904762	0.04761904762	0.1666666667		0.2	#DIV/0!
MAFA	0.04761904762	0.04761904762	0.05555555556		0.7	#DIV/0!
IAFA-AS	0.04761904762	0.04761904762	0.05555555556		0.7	#DIV/0!
MAFB	0.1428571429	0.1428571429	0.1666666667		0.6666666667	#DIV/0!
MAFF	0.04761904762	0.04761904762	0.1111111111		0.325	#DIV/0!
MAFG	0.04761904762	0.04761904762	0		inf	#DIV/0!
IAFG-D	0.04761904762	0.04761904762	0		inf	#DIV/0!
MAFK	0.04761904762	0.04761904762	0.1666666667		0.2	#DIV/0!
MAFTRI	0.09523809524	0.09523809524	0		inf	#DIV/0!
MAG	0.04761904762	0.04761904762	0.05555555556		0.7	#DIV/0!
IAGEF1	0.1428571429	0.1428571429	0.05555555556		2.333333333	#DIV/0!
MAGI1	0.04761904762	0.04761904762	0		inf	#DIV/0!
AGI1-A	0.04761904762	0.04761904762	0		inf	#DIV/0!
AGI1-IT	0.04761904762	0.04761904762	0		inf	#DIV/0!

MAGI2	0.04761904762	0.04761904762	0.1111111111		0.325	#DIV/0!
AGI2-A	0.04761904762	0.04761904762	0.1111111111		0.325	#DIV/0!
AGI2-A	0.04761904762	0.04761904762	0.1111111111		0.325	#DIV/0!
MAGI3	0.04761904762	0.04761904762	0.05555555556		0.7	#DIV/0!
MAGOH	0.04761904762	0.04761904762	0.05555555556		0.7	#DIV/0!
AGOH2	0.04761904762	0.04761904762	0.05555555556		0.7	#DIV/0!
IAGOH	0.04761904762	0.04761904762	0.1111111111		0.325	#DIV/0!
MAIP1	0	0	0.05555555556		0	#DIV/0!
MAK	0.04761904762	0.04761904762	0		inf	#DIV/0!
MAK16	0.04761904762	0.04761904762	0.1111111111		0.325	#DIV/0!
MAL	0	0	0.1111111111		0	#DIV/0!
MAL2	0.09523809524	0.09523809524	0.1666666667		0.4210526316	#DIV/0!
IAL2-AS	0.09523809524	0.09523809524	0.1666666667		0.4210526316	#DIV/0!
IALINC	0.04761904762	0.04761904762	0		inf	#DIV/0!
MALL	0	0	0.05555555556		0	#DIV/0!
IALRD	0.09523809524	0.09523809524	0.1111111111		0.6842105263	#DIV/0!
MALSU	0.04761904762	0.04761904762	0.1666666667		0.2	#DIV/0!
MALT1	0.09523809524	0.09523809524	0		inf	#DIV/0!
IAMDC	0	0	0.05555555556		0	#DIV/0!
MDC2-	0	0	0.05555555556		0	#DIV/0!
IAMDC	0.1428571429	0.1428571429	0.05555555556		2.333333333	#DIV/0!
MAML1	0.09523809524	0.09523809524	0.05555555556		1.473684211	#DIV/0!
MAML3	0	0	0.05555555556		0	#DIV/0!
IAMSTI	0.09523809524	0.09523809524	0.1111111111		0.6842105263	#DIV/0!
MAN1A2	0.04761904762	0.04761904762	0.05555555556		0.7	#DIV/0!
MAN1B1	0.1428571429	0.1428571429	0.05555555556		2.333333333	#DIV/0!
AN1B1-I	0.1428571429	0.1428571429	0.05555555556		2.333333333	#DIV/0!
MAN1C1	0.04761904762	0.04761904762	0.1111111111		0.325	#DIV/0!
MAN2A1	0.04761904762	0.04761904762	0		inf	#DIV/0!
MAN2A2	0.04761904762	0.04761904762	0.05555555556		0.7	#DIV/0!
MAN2B1	0.1428571429	0.1428571429	0.1111111111		1.083333333	#DIV/0!
MAN2C1	0.04761904762	0.04761904762	0.05555555556		0.7	#DIV/0!
MANBA	0	0	0.05555555556		0	#DIV/0!
IANBAI	0.09523809524	0.09523809524	0.1666666667		0.4210526316	#DIV/0!
MANCR	0.04761904762	0.04761904762	0.1666666667		0.2	#DIV/0!
IANEAI	0.04761904762	0.04761904762	0.05555555556		0.7	#DIV/0!
MANF	0.04761904762	0.04761904762	0		inf	#DIV/0!
IANSC1	0.04761904762	0.04761904762	0.05555555556		0.7	#DIV/0!
IANSC4	0.04761904762	0.04761904762	0.05555555556		0.7	#DIV/0!
MAP10	0.1428571429	0.1428571429	0		inf	#DIV/0!
MAP11	0.04761904762	0.04761904762	0.05555555556		0.7	#DIV/0!
MAP1B	0	0	0.05555555556		0	#DIV/0!
AP1LC3	0.09523809524	0.09523809524	0.1111111111		0.6842105263	#DIV/0!
AP1LC3	0.04761904762	0.04761904762	0		inf	#DIV/0!
AP1LC3	0	0	0.05555555556		0	#DIV/0!
AP1LC3	0.1428571429	0.1428571429	0		inf	#DIV/0!
MAP1S	0.1428571429	0.1428571429	0.1111111111		1.083333333	#DIV/0!
MAP2	0	0	0.05555555556		0	#DIV/0!
MAP2K1	0.04761904762	0.04761904762	0.05555555556		0.7	#DIV/0!
MAP2K2	0.09523809524	0.09523809524	0.05555555556		1.473684211	#DIV/0!
MAP2K3	0.09523809524	0.09523809524	0.05555555556		1.473684211	#DIV/0!

MAP2K4	0.04761904762	0.04761904762	0.05555555556		0.7	#DIV/0!
MAP2K5	0.04761904762	0.04761904762	0.05555555556		0.7	#DIV/0!
MAP2K6	0	0	0			#DIV/0!
MAP2K7	0.1428571429	0.1428571429	0.05555555556		2.333333333	#DIV/0!
MAP3K1	0.04761904762	0.04761904762	0.05555555556		0.7	#DIV/0!
IAP3K1	0.04761904762	0.04761904762	0.05555555556		0.7	#DIV/0!
IAP3K1	0.04761904762	0.04761904762	0.05555555556		0.7	#DIV/0!
IAP3K1	0.1428571429	0.1428571429	0.05555555556		2.333333333	#DIV/0!
IAP3K1	0.1428571429	0.1428571429	0		inf	#DIV/0!
P3K14-A	0.1428571429	0.1428571429	0		inf	#DIV/0!
IAP3K1	0.04761904762	0.04761904762	0.05555555556		0.7	#DIV/0!
MAP3K2	0	0	0.05555555556		0	#DIV/0!
IAP3K2	0	0	0.1111111111		0	#DIV/0!
P3K20-A	0	0	0.1111111111		0	#DIV/0!
IAP3K2	0.1428571429	0.1428571429	0		inf	#DIV/0!
MAP3K3	0.04761904762	0.04761904762	0		inf	#DIV/0!
MAP3K6	0.04761904762	0.04761904762	0.1111111111		0.325	#DIV/0!
MAP3K7	0	0	0			#DIV/0!
AP3K7C	0.04761904762	0.04761904762	0.1111111111		0.325	#DIV/0!
MAP3K8	0.09523809524	0.09523809524	0.2222222222		0.2894736842	#DIV/0!
MAP4	0.09523809524	0.09523809524	0		inf	#DIV/0!
MAP4K1	0.04761904762	0.04761904762	0.05555555556		0.7	#DIV/0!
MAP4K3	0	0	0.05555555556		0	#DIV/0!
AP4K3-I	0	0	0.05555555556		0	#DIV/0!
MAP4K4	0	0	0.05555555556		0	#DIV/0!
MAP4K5	0.04761904762	0.04761904762	0		inf	#DIV/0!
MAP6D1	0.1904761905	0.1904761905	0.05555555556		3.294117647	#DIV/0!
MAP7D1	0.04761904762	0.04761904762	0.05555555556		0.7	#DIV/0!
MAP9	0	0	0.05555555556		0	#DIV/0!
MAPK1	0.04761904762	0.04761904762	0.05555555556		0.7	#DIV/0!
IAPK10	0	0	0.05555555556		0	#DIV/0!
IAPK11	0	0	0.1111111111		0	#DIV/0!
IAPK12	0	0	0.1111111111		0	#DIV/0!
MAPK13	0.04761904762	0.04761904762	0.05555555556		0.7	#DIV/0!
IAPK14	0.04761904762	0.04761904762	0.05555555556		0.7	#DIV/0!
MAPK15	0.04761904762	0.04761904762	0.1666666667		0.2	#DIV/0!
APK1IP	0.04761904762	0.04761904762	0		inf	#DIV/0!
MAPK3	0.04761904762	0.04761904762	0		inf	#DIV/0!
MAPK4	0.04761904762	0.04761904762	0		inf	#DIV/0!
MAPK6	0.04761904762	0.04761904762	0.05555555556		0.7	#DIV/0!
APK6-D	0.04761904762	0.04761904762	0.05555555556		0.7	#DIV/0!
MAPK7	0.04761904762	0.04761904762	0.05555555556		0.7	#DIV/0!
MAPK8	0.09523809524	0.09523809524	0.05555555556		1.473684211	#DIV/0!
APK8IP1	0.1428571429	0.1428571429	0		inf	#DIV/0!
IAPK8II	0	0	0.1111111111		0	#DIV/0!
IAPK8II	0.09523809524	0.09523809524	0.2222222222		0.2894736842	#DIV/0!
MAPK9	0.09523809524	0.09523809524	0.05555555556		1.473684211	#DIV/0!
IAPKAP	0.09523809524	0.09523809524	0.05555555556		1.473684211	#DIV/0!
APKAPI	0.1428571429	0.1428571429	0		inf	#DIV/0!
APKAPI	0.09523809524	0.09523809524	0		inf	#DIV/0!
APKAPI	0	0	0.1111111111		0	#DIV/0!

KAPK5	0	0	0.1111111111		0	#DIV/0!
MAPRE1	0.09523809524	0.09523809524	0.1111111111		0.6842105263	#DIV/0!
MAPRE2	0.09523809524	0.09523809524	0		inf	#DIV/0!
MAPT	0.1428571429	0.1428571429	0		inf	#DIV/0!
IAPT-AS	0.1428571429	0.1428571429	0		inf	#DIV/0!
IAPT-IT	0.1428571429	0.1428571429	0		inf	#DIV/0!
MARC1	0.1428571429	0.1428571429	0.05555555556		2.333333333	#DIV/0!
MARC2	0.1428571429	0.1428571429	0.05555555556		2.333333333	#DIV/0!
IARCH	0.04761904762	0.04761904762	0.05555555556		0.7	#DIV/0!
IARCH1	0.04761904762	0.04761904762	0		inf	#DIV/0!
IARCH1	0.04761904762	0.04761904762	0.05555555556		0.7	#DIV/0!
IARCH	0.1428571429	0.1428571429	0.05555555556		2.333333333	#DIV/0!
IARCH	0.04761904762	0.04761904762	0		inf	#DIV/0!
IARCH	0	0	0.05555555556		0	#DIV/0!
IARCH	0.1428571429	0.1428571429	0.1111111111		1.083333333	#DIV/0!
IARCH	0.04761904762	0.04761904762	0.05555555556		0.7	#DIV/0!
IARCH	0	0	0.05555555556		0	#DIV/0!
IARCH	0.1428571429	0.1428571429	0.05555555556		2.333333333	#DIV/0!
IARCH	0.1428571429	0.1428571429	0.05555555556		2.333333333	#DIV/0!
ARCKS	0.04761904762	0.04761904762	0.1111111111		0.325	#DIV/0!
MARCO	0	0	0.05555555556		0	#DIV/0!
MARF1	0.04761904762	0.04761904762	0		inf	#DIV/0!
MARK1	0.1428571429	0.1428571429	0.05555555556		2.333333333	#DIV/0!
IARK2P	0.1428571429	0.1428571429	0.1111111111		1.083333333	#DIV/0!
MARK4	0.09523809524	0.09523809524	0.1111111111		0.6842105263	#DIV/0!
MARS2	0	0	0.05555555556		0	#DIV/0!
ARVEL	0.1428571429	0.1428571429	0		inf	#DIV/0!
ARVEL	0.04761904762	0.04761904762	0.05555555556		0.7	#DIV/0!
ARVEL	0.04761904762	0.04761904762	0		inf	#DIV/0!
MASP1	0.09523809524	0.09523809524	0.05555555556		1.473684211	#DIV/0!
MASP2	0.04761904762	0.04761904762	0.1111111111		0.325	#DIV/0!
MAST1	0.1428571429	0.1428571429	0.1111111111		1.083333333	#DIV/0!
MAST2	0.04761904762	0.04761904762	0.05555555556		0.7	#DIV/0!
MAST3	0.1428571429	0.1428571429	0.1111111111		1.083333333	#DIV/0!
MAST4	0.04761904762	0.04761904762	0		inf	#DIV/0!
AST4-A	0.04761904762	0.04761904762	0		inf	#DIV/0!
MASTL	0.09523809524	0.09523809524	0.1111111111		0.6842105263	#DIV/0!
MAT1A	0.1428571429	0.1428571429	0		inf	#DIV/0!
MAT2A	0	0	0.05555555556		0	#DIV/0!
MAT2B	0	0	0.05555555556		0	#DIV/0!
MATK	0.09523809524	0.09523809524	0.05555555556		1.473684211	#DIV/0!
MATN1	0	0	0.1111111111		0	#DIV/0!
ATN1-A	0	0	0.1111111111		0	#DIV/0!
MATN2	0.1904761905	0.1904761905	0.1111111111		1.529411765	#DIV/0!
MATN3	0	0	0.05555555556		0	#DIV/0!
MATN4	0.1428571429	0.1428571429	0.1666666667		0.6666666667	#DIV/0!
MATR3	0.04761904762	0.04761904762	0		inf	#DIV/0!
MAU2	0.1428571429	0.1428571429	0.1111111111		1.083333333	#DIV/0!
MAVS	0.1904761905	0.1904761905	0.05555555556		3.294117647	#DIV/0!
MAZ	0.04761904762	0.04761904762	0		inf	#DIV/0!
MB	0	0	0.1111111111		0	#DIV/0!

MB21D2	0.09523809524	0.09523809524	0.05555555556		1.473684211	#DIV/0!
MBD1	0.04761904762	0.04761904762	0		inf	#DIV/0!
MBD2	0.09523809524	0.09523809524	0		inf	#DIV/0!
MBD3	0.09523809524	0.09523809524	0.1111111111		0.6842105263	#DIV/0!
MBD3L1	0.1428571429	0.1428571429	0.05555555556		2.333333333	#DIV/0!
MBD3L2	0.1428571429	0.1428571429	0.05555555556		2.333333333	#DIV/0!
MBD3L2	0.1428571429	0.1428571429	0.05555555556		2.333333333	#DIV/0!
MBD3L3	0.1428571429	0.1428571429	0.05555555556		2.333333333	#DIV/0!
MBD3L4	0.1428571429	0.1428571429	0.05555555556		2.333333333	#DIV/0!
MBD3L5	0.1428571429	0.1428571429	0.05555555556		2.333333333	#DIV/0!
MBD4	0.09523809524	0.09523809524	0.05555555556		1.473684211	#DIV/0!
MBD5	0	0	0.05555555556		0	#DIV/0!
MBL1P	0.1428571429	0.1428571429	0		inf	#DIV/0!
MBL2	0.04761904762	0.04761904762	0.05555555556		0.7	#DIV/0!
MBLAC1	0.04761904762	0.04761904762	0.05555555556		0.7	#DIV/0!
MBLAC2	0.04761904762	0.04761904762	0		inf	#DIV/0!
MBNL1	0.1904761905	0.1904761905	0.1111111111		1.529411765	#DIV/0!
MBNL1-A	0.1904761905	0.1904761905	0.1111111111		1.529411765	#DIV/0!
MBOAT1	0	0	0.05555555556		0	#DIV/0!
MBOAT4	0.04761904762	0.04761904762	0.1111111111		0.325	#DIV/0!
MBOAT7	0.09523809524	0.09523809524	0.05555555556		1.473684211	#DIV/0!
MBP	0.09523809524	0.09523809524	0.05555555556		1.473684211	#DIV/0!
MBTD1	0.04761904762	0.04761904762	0		inf	#DIV/0!
MBTPS1	0.04761904762	0.04761904762	0		inf	#DIV/0!
MC1R	0.09523809524	0.09523809524	0		inf	#DIV/0!
MC2R	0.09523809524	0.09523809524	0.05555555556		1.473684211	#DIV/0!
MC3R	0.09523809524	0.09523809524	0.1666666667		0.4210526316	#DIV/0!
MC4R	0.09523809524	0.09523809524	0		inf	#DIV/0!
MC5R	0.09523809524	0.09523809524	0.05555555556		1.473684211	#DIV/0!
MCAT	0	0	0.1666666667		0	#DIV/0!
MCC	0.04761904762	0.04761904762	0		inf	#DIV/0!
MCCC1	0.1904761905	0.1904761905	0.05555555556		3.294117647	#DIV/0!
MCCC2	0.04761904762	0.04761904762	0		inf	#DIV/0!
MCCD1	0.04761904762	0.04761904762	0		inf	#DIV/0!
MCEE	0	0	0.05555555556		0	#DIV/0!
MCEMP	0.1428571429	0.1428571429	0.05555555556		2.333333333	#DIV/0!
MCF2L2	0.1904761905	0.1904761905	0.05555555556		3.294117647	#DIV/0!
MCFD2	0	0	0.05555555556		0	#DIV/0!
MCHR1	0	0	0.1111111111		0	#DIV/0!
MCIDAS	0.04761904762	0.04761904762	0.05555555556		0.7	#DIV/0!
MCL1	0.1904761905	0.1904761905	0.05555555556		3.294117647	#DIV/0!
MCM10	0.09523809524	0.09523809524	0.1111111111		0.6842105263	#DIV/0!
MCM2	0.09523809524	0.09523809524	0.05555555556		1.473684211	#DIV/0!
MCM3	0.04761904762	0.04761904762	0		inf	#DIV/0!
MCM3AP	0.04761904762	0.04761904762	0.1111111111		0.325	#DIV/0!
MCM3AP-A	0.04761904762	0.04761904762	0.1111111111		0.325	#DIV/0!
MCM4	0.09523809524	0.09523809524	0.1666666667		0.4210526316	#DIV/0!
MCM5	0	0	0.1111111111		0	#DIV/0!
MCM6	0.04761904762	0.04761904762	0.05555555556		0.7	#DIV/0!
MCM7	0.04761904762	0.04761904762	0.05555555556		0.7	#DIV/0!
MCM8	0.1904761905	0.1904761905	0.05555555556		3.294117647	#DIV/0!

CM8-AS	0.1904761905	0.1904761905	0.05555555556		3.294117647	#DIV/0!
MCMBP	0.2380952381	0.2380952381	0		inf	#DIV/0!
MCMDC	0.1428571429	0.1428571429	0.1666666667		0.6666666667	#DIV/0!
MCOLN	0.1428571429	0.1428571429	0.05555555556		2.333333333	#DIV/0!
MCOLN	0.04761904762	0.04761904762	0.05555555556		0.7	#DIV/0!
MCOLN	0.04761904762	0.04761904762	0.05555555556		0.7	#DIV/0!
MCPH1	0.09523809524	0.09523809524	0.1111111111		0.6842105263	#DIV/0!
CPH1-A	0.09523809524	0.09523809524	0.1111111111		0.6842105263	#DIV/0!
MCRIP1	0.04761904762	0.04761904762	0		inf	#DIV/0!
MCRIP2	0.04761904762	0.04761904762	0.2222222222		0.1375	#DIV/0!
MCRS1	0.04761904762	0.04761904762	0.05555555556		0.7	#DIV/0!
MCTP1	0.04761904762	0.04761904762	0		inf	#DIV/0!
MCTP2	0.04761904762	0.04761904762	0.05555555556		0.7	#DIV/0!
MCU	0.1904761905	0.1904761905	0.05555555556		3.294117647	#DIV/0!
MCUB	0	0	0.05555555556		0	#DIV/0!
MDFI	0	0	0.05555555556		0	#DIV/0!
MDFIC	0.04761904762	0.04761904762	0.1111111111		0.325	#DIV/0!
MDFIC2	0.04761904762	0.04761904762	0		inf	#DIV/0!
MDGA1	0.04761904762	0.04761904762	0.05555555556		0.7	#DIV/0!
MDGA2	0.04761904762	0.04761904762	0		inf	#DIV/0!
MDH1	0	0	0.05555555556		0	#DIV/0!
MDH1B	0	0	0.05555555556		0	#DIV/0!
MDH2	0.1428571429	0.1428571429	0.1111111111		1.083333333	#DIV/0!
MDK	0	0	0.05555555556		0	#DIV/0!
MDM1	0.09523809524	0.09523809524	0.05555555556		1.473684211	#DIV/0!
MDM2	0.09523809524	0.09523809524	0.1111111111		0.6842105263	#DIV/0!
MDM4	0.2380952381	0.2380952381	0		inf	#DIV/0!
MDN1	0	0	0			#DIV/0!
MDS2	0.04761904762	0.04761904762	0.1111111111		0.325	#DIV/0!
ME2	0.04761904762	0.04761904762	0		inf	#DIV/0!
MEA1	0.04761904762	0.04761904762	0.05555555556		0.7	#DIV/0!
MEAF6	0.04761904762	0.04761904762	0.05555555556		0.7	#DIV/0!
MEAK7	0.04761904762	0.04761904762	0		inf	#DIV/0!
MECOM	0.1428571429	0.1428571429	0.05555555556		2.333333333	#DIV/0!
MECR	0.04761904762	0.04761904762	0.1111111111		0.325	#DIV/0!
MED1	0.09523809524	0.09523809524	0		inf	#DIV/0!
MED10	0	0	0.1111111111		0	#DIV/0!
MED11	0	0	0.1111111111		0	#DIV/0!
MED12L	0.1904761905	0.1904761905	0.1111111111		1.529411765	#DIV/0!
MED13	0.09523809524	0.09523809524	0		inf	#DIV/0!
MED13L	0	0	0.05555555556		0	#DIV/0!
MED15	0.04761904762	0.04761904762	0.05555555556		0.7	#DIV/0!
MED15P	0	0	0.05555555556		0	#DIV/0!
MED16	0.09523809524	0.09523809524	0.1111111111		0.6842105263	#DIV/0!
MED18	0.04761904762	0.04761904762	0.1111111111		0.325	#DIV/0!
MED20	0.04761904762	0.04761904762	0.05555555556		0.7	#DIV/0!
MED21	0.04761904762	0.04761904762	0.05555555556		0.7	#DIV/0!
MED22	0.04761904762	0.04761904762	0		inf	#DIV/0!
MED24	0.09523809524	0.09523809524	0		inf	#DIV/0!
MED25	0.09523809524	0.09523809524	0.1111111111		0.6842105263	#DIV/0!
MED26	0.1428571429	0.1428571429	0.1111111111		1.083333333	#DIV/0!

**MED27**	0.09523809524	0.09523809524	0		inf	#DIV/0!
**MED28**	0	0	0			#DIV/0!
**MED29**	0.04761904762	0.04761904762	0.05555555556		0.7	#DIV/0!
**MED30**	0.1428571429	0.1428571429	0.1666666667		0.6666666667	#DIV/0!
**MED31**	0	0	0.05555555556		0	#DIV/0!
**MED7**	0.04761904762	0.04761904762	0.05555555556		0.7	#DIV/0!
**MED8**	0.04761904762	0.04761904762	0.05555555556		0.7	#DIV/0!
**MED9**	0.04761904762	0.04761904762	0.1111111111		0.325	#DIV/0!
**MEF2A**	0.04761904762	0.04761904762	0.05555555556		0.7	#DIV/0!
**MEF2B**	0.1428571429	0.1428571429	0.1111111111		1.083333333	#DIV/0!
**MEF2C**	0.04761904762	0.04761904762	0		inf	#DIV/0!
**EF2C-A**	0.04761904762	0.04761904762	0		inf	#DIV/0!
**EF2C-A**	0.04761904762	0.04761904762	0		inf	#DIV/0!
**MEF2D**	0.1428571429	0.1428571429	0.1111111111		1.083333333	#DIV/0!
**MEFV**	0.04761904762	0.04761904762	0.1111111111		0.325	#DIV/0!
**MEGF10**	0.04761904762	0.04761904762	0		inf	#DIV/0!
**MEGF11**	0.04761904762	0.04761904762	0.05555555556		0.7	#DIV/0!
**MEGF6**	0	0	0.05555555556		0	#DIV/0!
**MEGF8**	0.04761904762	0.04761904762	0.05555555556		0.7	#DIV/0!
**MEGF9**	0.09523809524	0.09523809524	0.05555555556		1.473684211	#DIV/0!
**MEI1**	0	0	0.1111111111		0	#DIV/0!
**MEIG1**	0.09523809524	0.09523809524	0.1111111111		0.6842105263	#DIV/0!
**MEIKIN**	0.04761904762	0.04761904762	0		inf	#DIV/0!
**MEIOB**	0.09523809524	0.09523809524	0.2222222222		0.2894736842	#DIV/0!
**MEIOC**	0.09523809524	0.09523809524	0		inf	#DIV/0!
**MEIS1**	0	0	0.05555555556		0	#DIV/0!
**EIS1-AS**	0	0	0.05555555556		0	#DIV/0!
**EIS1-AS**	0	0	0.05555555556		0	#DIV/0!
**MEIS2**	0	0	0			#DIV/0!
**MEIS3**	0.09523809524	0.09523809524	0.1111111111		0.6842105263	#DIV/0!
**MEIS3P1**	0.04761904762	0.04761904762	0.05555555556		0.7	#DIV/0!
**MELK**	0.09523809524	0.09523809524	0.05555555556		1.473684211	#DIV/0!
**MELTF**	0.1428571429	0.1428571429	0.05555555556		2.333333333	#DIV/0!
**ELTF-A**	0.1428571429	0.1428571429	0.05555555556		2.333333333	#DIV/0!
**MEMO1**	0.1428571429	0.1428571429	0		inf	#DIV/0!
**MEOX1**	0.04761904762	0.04761904762	0		inf	#DIV/0!
**MEOX2**	0.04761904762	0.04761904762	0.2222222222		0.1375	#DIV/0!
**MEP1A**	0.04761904762	0.04761904762	0.05555555556		0.7	#DIV/0!
**MEP1B**	0.09523809524	0.09523809524	0		inf	#DIV/0!
**MEPCE**	0.04761904762	0.04761904762	0.05555555556		0.7	#DIV/0!
**MEPE**	0	0	0.05555555556		0	#DIV/0!
**MERTK**	0	0	0.05555555556		0	#DIV/0!
**MESD**	0.04761904762	0.04761904762	0.05555555556		0.7	#DIV/0!
**MESP1**	0.04761904762	0.04761904762	0.05555555556		0.7	#DIV/0!
**MESP2**	0.04761904762	0.04761904762	0.05555555556		0.7	#DIV/0!
**MEST**	0.04761904762	0.04761904762	0.05555555556		0.7	#DIV/0!
**IESTIT**	0.04761904762	0.04761904762	0.05555555556		0.7	#DIV/0!
**MET**	0.09523809524	0.09523809524	0.2222222222		0.2894736842	#DIV/0!
**METAP1**	0	0	0.05555555556		0	#DIV/0!
**IETAP1**	0	0	0.05555555556		0	#DIV/0!
**METAP2**	0.04761904762	0.04761904762	0.05555555556		0.7	#DIV/0!

METRN	0.04761904762	0.04761904762	0.2222222222		0.1375	#DIV/0!
METTL1	0.1428571429	0.1428571429	0.05555555556		2.333333333	#DIV/0!
ETTL11	0.1428571429	0.1428571429	0.05555555556		2.333333333	#DIV/0!
METTL1	0	0	0.05555555556		0	#DIV/0!
TTL14-	0	0	0.05555555556		0	#DIV/0!
METTL1	0	0	0.1111111111		0	#DIV/0!
METTL1	0.1428571429	0.1428571429	0.05555555556		2.333333333	#DIV/0!
ETTL21	0	0	0.05555555556		0	#DIV/0!
METTL2	0.04761904762	0.04761904762	0		inf	#DIV/0!
METTL2	0.04761904762	0.04761904762	0		inf	#DIV/0!
METTL2	0	0	0			#DIV/0!
METTL2	0.04761904762	0.04761904762	0.05555555556		0.7	#DIV/0!
METTL2	0.04761904762	0.04761904762	0.2222222222		0.1375	#DIV/0!
METTL2	0.1428571429	0.1428571429	0.1111111111		1.083333333	#DIV/0!
IETTL2	0.04761904762	0.04761904762	0		inf	#DIV/0!
METTL2	0.04761904762	0.04761904762	0.05555555556		0.7	#DIV/0!
METTL4	0.09523809524	0.09523809524	0		inf	#DIV/0!
METTL5	0	0	0.05555555556		0	#DIV/0!
METTL6	0.04761904762	0.04761904762	0		inf	#DIV/0!
IETTL7	0.09523809524	0.09523809524	0.05555555556		1.473684211	#DIV/0!
METTL7	0.04761904762	0.04761904762	0.05555555556		0.7	#DIV/0!
METTL8	0	0	0.05555555556		0	#DIV/0!
METTL9	0.04761904762	0.04761904762	0		inf	#DIV/0!
MEX3A	0.1428571429	0.1428571429	0.1111111111		1.083333333	#DIV/0!
MEX3B	0.04761904762	0.04761904762	0.05555555556		0.7	#DIV/0!
MEX3C	0.09523809524	0.09523809524	0		inf	#DIV/0!
MEX3D	0.09523809524	0.09523809524	0.1111111111		0.6842105263	#DIV/0!
MFAP2	0.04761904762	0.04761904762	0.1111111111		0.325	#DIV/0!
MFAP3L	0	0	0.05555555556		0	#DIV/0!
MFAP4	0.04761904762	0.04761904762	0.05555555556		0.7	#DIV/0!
MFAP5	0.04761904762	0.04761904762	0.1111111111		0.325	#DIV/0!
MFF	0	0	0.05555555556		0	#DIV/0!
MFGE8	0.04761904762	0.04761904762	0.05555555556		0.7	#DIV/0!
MFHAS1	0.09523809524	0.09523809524	0.1111111111		0.6842105263	#DIV/0!
MFN1	0.1904761905	0.1904761905	0.05555555556		3.294117647	#DIV/0!
MFN2	0.04761904762	0.04761904762	0.1111111111		0.325	#DIV/0!
MFNG	0	0	0.1111111111		0	#DIV/0!
MFSD1	0.1428571429	0.1428571429	0.05555555556		2.333333333	#DIV/0!
MFSD10	0.04761904762	0.04761904762	0		inf	#DIV/0!
MFSD11	0.04761904762	0.04761904762	0		inf	#DIV/0!
MFSD12	0.09523809524	0.09523809524	0.05555555556		1.473684211	#DIV/0!
MFSD13A	0.1428571429	0.1428571429	0.05555555556		2.333333333	#DIV/0!
MFSD13B	0.04761904762	0.04761904762	0		inf	#DIV/0!
MFSD14A	0.04761904762	0.04761904762	0.05555555556		0.7	#DIV/0!
MFSD14B	0.04761904762	0.04761904762	0.05555555556		0.7	#DIV/0!
MFSD14C	0.04761904762	0.04761904762	0		inf	#DIV/0!
MFSD2A	0.04761904762	0.04761904762	0.05555555556		0.7	#DIV/0!
MFSD3	0.04761904762	0.04761904762	0.1666666667		0.2	#DIV/0!
MFSD4A	0.1428571429	0.1428571429	0		inf	#DIV/0!
FSD4A-A	0.1428571429	0.1428571429	0		inf	#DIV/0!
MFSD4B	0	0	0			#DIV/0!

**MFSD5**	0.04761904762	0.04761904762	0.05555555556		0.7	#DIV/0!
**MFSD6**	0	0	0.05555555556		0	#DIV/0!
**MFSD6L**	0.04761904762	0.04761904762	0.05555555556		0.7	#DIV/0!
**MFSD8**	0	0	0.05555555556		0	#DIV/0!
**MFSD9**	0	0	0.05555555556		0	#DIV/0!
**MGAM**	0.04761904762	0.04761904762	0.05555555556		0.7	#DIV/0!
**MGAM2**	0.04761904762	0.04761904762	0.05555555556		0.7	#DIV/0!
**MGARP**	0.04761904762	0.04761904762	0.05555555556		0.7	#DIV/0!
**MGAT1**	0.09523809524	0.09523809524	0.05555555556		1.473684211	#DIV/0!
**MGAT2**	0.04761904762	0.04761904762	0		inf	#DIV/0!
**MGAT3**	0	0	0.1111111111		0	#DIV/0!
**GAT3-A**	0	0	0.1111111111		0	#DIV/0!
**MGAT4A**	0	0	0.05555555556		0	#DIV/0!
**MGAT4B**	0.09523809524	0.09523809524	0.05555555556		1.473684211	#DIV/0!
**MGAT4C**	0.04761904762	0.04761904762	0.05555555556		0.7	#DIV/0!
**MGAT4D**	0	0	0.05555555556		0	#DIV/0!
**IGAT4E**	0.1428571429	0.1428571429	0		inf	#DIV/0!
**MGAT5**	0	0	0.05555555556		0	#DIV/0!
**MGAT5B**	0.04761904762	0.04761904762	0		inf	#DIV/0!
**IGC1291**	0.04761904762	0.04761904762	0.05555555556		0.7	#DIV/0!
**IGC1588**	0.04761904762	0.04761904762	0.05555555556		0.7	#DIV/0!
**IGC1602**	0	0	0.05555555556		0	#DIV/0!
**IGC1627**	0.04761904762	0.04761904762	0		inf	#DIV/0!
**IGC2734**	0.04761904762	0.04761904762	0.05555555556		0.7	#DIV/0!
**IGC2738**	0.04761904762	0.04761904762	0.05555555556		0.7	#DIV/0!
**MGC2889**	0.09523809524	0.09523809524	0.05555555556		1.473684211	#DIV/0!
**IGC3280**	0.04761904762	0.04761904762	0		inf	#DIV/0!
**IGC3479**	0.04761904762	0.04761904762	0.05555555556		0.7	#DIV/0!
**MGC4859**	0.04761904762	0.04761904762	0.2222222222		0.1375	#DIV/0!
**MGME1**	0.1904761905	0.1904761905	0.1111111111		1.529411765	#DIV/0!
**MGMT**	0.2857142857	0.2857142857	0		inf	#DIV/0!
**MGP**	0.04761904762	0.04761904762	0.1111111111		0.325	#DIV/0!
**MGRN1**	0.04761904762	0.04761904762	0		inf	#DIV/0!
**MGST1**	0.04761904762	0.04761904762	0.1111111111		0.325	#DIV/0!
**MGST2**	0	0	0.05555555556		0	#DIV/0!
**MGST3**	0.1428571429	0.1428571429	0		inf	#DIV/0!
**IHENCI**	0.1428571429	0.1428571429	0.2222222222		0.4583333333	#DIV/0!
**MHRT**	0	0	0.05555555556		0	#DIV/0!
**MIA2**	0.04761904762	0.04761904762	0		inf	#DIV/0!
**MIA3**	0.1428571429	0.1428571429	0.05555555556		2.333333333	#DIV/0!
**MIAT**	0.04761904762	0.04761904762	0.05555555556		0.7	#DIV/0!
**MIATNB**	0.04761904762	0.04761904762	0.05555555556		0.7	#DIV/0!
**MIB1**	0.04761904762	0.04761904762	0.1111111111		0.325	#DIV/0!
**MIB2**	0	0	0.1111111111		0	#DIV/0!
**MICA**	0.04761904762	0.04761904762	0		inf	#DIV/0!
**MICAL1**	0	0	0			#DIV/0!
**MICAL3**	0.04761904762	0.04761904762	0.05555555556		0.7	#DIV/0!
**IICALL**	0	0	0.1111111111		0	#DIV/0!
**IICALL**	0.04761904762	0.04761904762	0.1666666667		0.2	#DIV/0!
**MICB**	0.04761904762	0.04761904762	0		inf	#DIV/0!
**IICB-DT**	0.04761904762	0.04761904762	0		inf	#DIV/0!

MICOS1	0.04761904762	0.04761904762	0.1111111111		0.325	#DIV/0!
COS10-N	0.04761904762	0.04761904762	0.1111111111		0.325	#DIV/0!
MICOS1	0.1428571429	0.1428571429	0.05555555556		2.333333333	#DIV/0!
MICU1	0.1904761905	0.1904761905	0.05555555556		3.294117647	#DIV/0!
MICU3	0.09523809524	0.09523809524	0.1111111111		0.6842105263	#DIV/0!
MIDN	0.09523809524	0.09523809524	0.1111111111		0.6842105263	#DIV/0!
MIEF1	0	0	0.1111111111		0	#DIV/0!
MIEN1	0.09523809524	0.09523809524	0		inf	#DIV/0!
MIER1	0.04761904762	0.04761904762	0.05555555556		0.7	#DIV/0!
MIER2	0.09523809524	0.09523809524	0.1111111111		0.6842105263	#DIV/0!
MIER3	0.04761904762	0.04761904762	0		inf	#DIV/0!
MIF	0.04761904762	0.04761904762	0.1111111111		0.325	#DIV/0!
MIF-AS1	0.04761904762	0.04761904762	0.1111111111		0.325	#DIV/0!
MIF4GD	0.04761904762	0.04761904762	0.05555555556		0.7	#DIV/0!
MIG7	0.04761904762	0.04761904762	0.05555555556		0.7	#DIV/0!
MIGA1	0.04761904762	0.04761904762	0.05555555556		0.7	#DIV/0!
MIIP	0.04761904762	0.04761904762	0.1111111111		0.325	#DIV/0!
MILR1	0.04761904762	0.04761904762	0		inf	#DIV/0!
MIMT1	0.1428571429	0.1428571429	0.05555555556		2.333333333	#DIV/0!
MINAR1	0.04761904762	0.04761904762	0.05555555556		0.7	#DIV/0!
MINAR2	0.04761904762	0.04761904762	0		inf	#DIV/0!
MINDY1	0.1904761905	0.1904761905	0.05555555556		3.294117647	#DIV/0!
MINDY2	0.04761904762	0.04761904762	0.05555555556		0.7	#DIV/0!
MINDY3	0.09523809524	0.09523809524	0.1111111111		0.6842105263	#DIV/0!
MINDY4	0.04761904762	0.04761904762	0.1111111111		0.325	#DIV/0!
IINDY4	0.1904761905	0.1904761905	0.1111111111		1.529411765	#DIV/0!
MINK1	0	0	0.1111111111		0	#DIV/0!
MINPP1	0.1428571429	0.1428571429	0.1111111111		1.083333333	#DIV/0!
MIOS	0.04761904762	0.04761904762	0.1666666667		0.2	#DIV/0!
MIOX	0	0	0.1111111111		0	#DIV/0!
MIP	0.1428571429	0.1428571429	0.05555555556		2.333333333	#DIV/0!
MIR1-1	0.1428571429	0.1428571429	0.2222222222		0.4583333333	#DIV/0!
IIR1-1H	0.1428571429	0.1428571429	0.2222222222		0.4583333333	#DIV/0!
R1-1HG-	0.1428571429	0.1428571429	0.2222222222		0.4583333333	#DIV/0!
MIR1-2	0.04761904762	0.04761904762	0.1111111111		0.325	#DIV/0!
MIR101-	0.04761904762	0.04761904762	0.05555555556		0.7	#DIV/0!
MIR101-	0.04761904762	0.04761904762	0.05555555556		0.7	#DIV/0!
IIR103A	0	0	0.05555555556		0	#DIV/0!
IIR103A	0.1904761905	0.1904761905	0.05555555556		3.294117647	#DIV/0!
IIR103B	0	0	0.05555555556		0	#DIV/0!
IIR103B	0.1904761905	0.1904761905	0.05555555556		3.294117647	#DIV/0!
MIR106I	0.04761904762	0.04761904762	0.05555555556		0.7	#DIV/0!
MIR107	0.1428571429	0.1428571429	0.1111111111		1.083333333	#DIV/0!
MIR10A	0.1428571429	0.1428571429	0		inf	#DIV/0!
MIR1178	0	0	0.05555555556		0	#DIV/0!
MIR1179	0.04761904762	0.04761904762	0.05555555556		0.7	#DIV/0!
MIR1180	0.04761904762	0.04761904762	0.1111111111		0.325	#DIV/0!
MIR1181	0.1428571429	0.1428571429	0.1111111111		1.083333333	#DIV/0!
MIR1182	0.1428571429	0.1428571429	0		inf	#DIV/0!
MIR1183	0.04761904762	0.04761904762	0.1666666667		0.2	#DIV/0!
MIR1199	0.1428571429	0.1428571429	0.1111111111		1.083333333	#DIV/0!

MIR1200	0.04761904762	0.04761904762	0.2222222222		0.1375	#DIV/0!
MIR1203	0.1428571429	0.1428571429	0		inf	#DIV/0!
MIR1204	0.09523809524	0.09523809524	0.1111111111		0.6842105263	#DIV/0!
MIR1205	0.09523809524	0.09523809524	0.1111111111		0.6842105263	#DIV/0!
MIR1206	0.09523809524	0.09523809524	0.1111111111		0.6842105263	#DIV/0!
MIR1207	0.09523809524	0.09523809524	0.1111111111		0.6842105263	#DIV/0!
MIR1208	0.09523809524	0.09523809524	0.1111111111		0.6842105263	#DIV/0!
MIR122	0.09523809524	0.09523809524	0		inf	#DIV/0!
MIR1224	0.1428571429	0.1428571429	0.05555555556		2.333333333	#DIV/0!
MIR1225	0.09523809524	0.09523809524	0.2222222222		0.2894736842	#DIV/0!
MIR1226	0.09523809524	0.09523809524	0		inf	#DIV/0!
MIR1227	0.09523809524	0.09523809524	0.1111111111		0.6842105263	#DIV/0!
MIR1228	0.1428571429	0.1428571429	0.05555555556		2.333333333	#DIV/0!
MIR1229	0.09523809524	0.09523809524	0.05555555556		1.473684211	#DIV/0!
MIR1231	0.1428571429	0.1428571429	0		inf	#DIV/0!
MIR1234	0.04761904762	0.04761904762	0		inf	#DIV/0!
MIR1236	0.04761904762	0.04761904762	0		inf	#DIV/0!
MIR1238	0.1428571429	0.1428571429	0.1111111111		1.083333333	#DIV/0!
MIR124-	0.09523809524	0.09523809524	0.1111111111		0.6842105263	#DIV/0!
MIR124-	0.1428571429	0.1428571429	0.1666666667		0.6666666667	#DIV/0!
IR124-2I	0.1428571429	0.1428571429	0.1666666667		0.6666666667	#DIV/0!
MIR124-	0.1428571429	0.1428571429	0.2222222222		0.4583333333	#DIV/0!
MIR1243	0	0	0.05555555556		0	#DIV/0!
MIR1244-	0.09523809524	0.09523809524	0.1666666667		0.4210526316	#DIV/0!
MIR1244-	0.09523809524	0.09523809524	0.1666666667		0.4210526316	#DIV/0!
MIR1244-	0.09523809524	0.09523809524	0.1666666667		0.4210526316	#DIV/0!
MIR1244-	0.09523809524	0.09523809524	0.1666666667		0.4210526316	#DIV/0!
MIR1245	0	0	0.1666666667		0	#DIV/0!
MIR1245	0	0	0.1666666667		0	#DIV/0!
MIR1246	0	0	0.1111111111		0	#DIV/0!
MIR1248	0.09523809524	0.09523809524	0.05555555556		1.473684211	#DIV/0!
MIR1249	0	0	0.1111111111		0	#DIV/0!
MIR1250	0.04761904762	0.04761904762	0		inf	#DIV/0!
MIR1251	0.1904761905	0.1904761905	0.05555555556		3.294117647	#DIV/0!
MIR1252	0.04761904762	0.04761904762	0		inf	#DIV/0!
MIR1253	0	0	0.1111111111		0	#DIV/0!
MIR1255	0	0	0.05555555556		0	#DIV/0!
IR1255B	0.1428571429	0.1428571429	0		inf	#DIV/0!
MIR1256	0.04761904762	0.04761904762	0.1111111111		0.325	#DIV/0!
MIR1257	0.1428571429	0.1428571429	0.1111111111		1.083333333	#DIV/0!
MIR1258	0	0	0.05555555556		0	#DIV/0!
MIR125B	0.04761904762	0.04761904762	0.1111111111		0.325	#DIV/0!
MIR126	0.1428571429	0.1428571429	0.05555555556		2.333333333	#DIV/0!
MIR1262	0.04761904762	0.04761904762	0.05555555556		0.7	#DIV/0!
MIR1263	0.1428571429	0.1428571429	0.05555555556		2.333333333	#DIV/0!
MIR1265	0.09523809524	0.09523809524	0.1111111111		0.6842105263	#DIV/0!
MIR1266	0.04761904762	0.04761904762	0.05555555556		0.7	#DIV/0!
MIR1268	0.380952381	0.380952381	0.3333333333		0.9230769231	#DIV/0!
MIR1268	0.04761904762	0.04761904762	0		inf	#DIV/0!
MIR1269	0.04761904762	0.04761904762	0.05555555556		0.7	#DIV/0!
MIR1269	0.04761904762	0.04761904762	0.1111111111		0.325	#DIV/0!

MIR127(	0.1428571429	0.1428571429	0		inf	#DIV/0!
MIR1271	0.09523809524	0.09523809524	0.05555555556		1.473684211	#DIV/0!
MIR1272	0.04761904762	0.04761904762	0.05555555556		0.7	#DIV/0!
IIR1273I	0.04761904762	0.04761904762	0		inf	#DIV/0!
MIR1275	0.04761904762	0.04761904762	0		inf	#DIV/0!
MIR1276	0.09523809524	0.09523809524	0.05555555556		1.473684211	#DIV/0!
MIR1279	0.09523809524	0.09523809524	0.1111111111		0.6842105263	#DIV/0!
MIR128-	0.04761904762	0.04761904762	0.05555555556		0.7	#DIV/0!
MIR128-	0.04761904762	0.04761904762	0		inf	#DIV/0!
MIR1281	0	0	0.1111111111		0	#DIV/0!
IIR1283-	0.09523809524	0.09523809524	0.05555555556		1.473684211	#DIV/0!
IIR1283-	0.09523809524	0.09523809524	0.05555555556		1.473684211	#DIV/0!
MIR1284	0.04761904762	0.04761904762	0		inf	#DIV/0!
IIR1285-	0.04761904762	0.04761904762	0.1111111111		0.325	#DIV/0!
IIR1285-	0	0	0.05555555556		0	#DIV/0!
MIR1286	0.04761904762	0.04761904762	0.05555555556		0.7	#DIV/0!
MIR1287	0.1428571429	0.1428571429	0		inf	#DIV/0!
IIR1289-	0.09523809524	0.09523809524	0.1111111111		0.6842105263	#DIV/0!
IIR1289-	0.04761904762	0.04761904762	0		inf	#DIV/0!
MIR129-	0.04761904762	0.04761904762	0.05555555556		0.7	#DIV/0!
MIR1290	0.04761904762	0.04761904762	0.1111111111		0.325	#DIV/0!
MIR1291	0.04761904762	0.04761904762	0.05555555556		0.7	#DIV/0!
MIR1292	0.1904761905	0.1904761905	0.05555555556		3.294117647	#DIV/0!
MIR1293	0.09523809524	0.09523809524	0.05555555556		1.473684211	#DIV/0!
IIR1295.	0.1428571429	0.1428571429	0.05555555556		2.333333333	#DIV/0!
IIR1295I	0.1428571429	0.1428571429	0.05555555556		2.333333333	#DIV/0!
MIR1296	0.09523809524	0.09523809524	0.1111111111		0.6842105263	#DIV/0!
MIR1299	0.09523809524	0.09523809524	0.2222222222		0.2894736842	#DIV/0!
IIR1302-	0	0	0.05555555556		0	#DIV/0!
IR1302-	0.1428571429	0.1428571429	0.2777777778		0.3333333333	#DIV/0!
IR1302-	0.1428571429	0.1428571429	0.2777777778		0.3333333333	#DIV/0!
IIR1302-	0.1428571429	0.1428571429	0.2777777778		0.3333333333	#DIV/0!
IIR1302-	0	0	0.05555555556		0	#DIV/0!
IIR1302-	0	0	0.05555555556		0	#DIV/0!
IIR1302-	0.09523809524	0.09523809524	0.1666666667		0.4210526316	#DIV/0!
IIR1302-	0.04761904762	0.04761904762	0.2222222222		0.1375	#DIV/0!
IIR1302-	0.04761904762	0.04761904762	0.1666666667		0.2	#DIV/0!
IIR1302-	0.04761904762	0.04761904762	0		inf	#DIV/0!
IIR1302-	0.1428571429	0.1428571429	0.2777777778		0.3333333333	#DIV/0!
MIR1303	0	0	0.05555555556		0	#DIV/0!
MIR1305	0	0	0.05555555556		0	#DIV/0!
MIR1306	0.04761904762	0.04761904762	0.05555555556		0.7	#DIV/0!
MIR1307	0.1428571429	0.1428571429	0.05555555556		2.333333333	#DIV/0!
MIR130I	0.04761904762	0.04761904762	0.05555555556		0.7	#DIV/0!
MIR132	0	0	0.1111111111		0	#DIV/0!
MIR1322	0.09523809524	0.09523809524	0.1111111111		0.6842105263	#DIV/0!
MIR1323	0.09523809524	0.09523809524	0.05555555556		1.473684211	#DIV/0!
MIR1324	0.04761904762	0.04761904762	0		inf	#DIV/0!
IIR133A	0.04761904762	0.04761904762	0.1111111111		0.325	#DIV/0!
R133A1I	0.04761904762	0.04761904762	0.1111111111		0.325	#DIV/0!
IIR133A	0.1428571429	0.1428571429	0.2222222222		0.4583333333	#DIV/0!

MIR133B	0.04761904762	0.04761904762	0		inf	#DIV/0!
MIR135A	0.1904761905	0.1904761905	0.05555555556		3.294117647	#DIV/0!
MIR135B	0.1904761905	0.1904761905	0		inf	#DIV/0!
MIR137	0.04761904762	0.04761904762	0.05555555556		0.7	#DIV/0!
IR137H	0.04761904762	0.04761904762	0.05555555556		0.7	#DIV/0!
MIR138-	0.04761904762	0.04761904762	0		inf	#DIV/0!
MIR138-	0.04761904762	0.04761904762	0		inf	#DIV/0!
MIR140	0.1428571429	0.1428571429	0.05555555556		2.333333333	#DIV/0!
MIR141	0.04761904762	0.04761904762	0.1111111111		0.325	#DIV/0!
MIR142	0.09523809524	0.09523809524	0		inf	#DIV/0!
MIR144	0.04761904762	0.04761904762	0.05555555556		0.7	#DIV/0!
MIR1469	0.04761904762	0.04761904762	0.05555555556		0.7	#DIV/0!
MIR146A	0.04761904762	0.04761904762	0.05555555556		0.7	#DIV/0!
MIR146B	0.1428571429	0.1428571429	0.05555555556		2.333333333	#DIV/0!
MIR1471	0	0	0.05555555556		0	#DIV/0!
MIR147A	0.04761904762	0.04761904762	0		inf	#DIV/0!
MIR147B	0.04761904762	0.04761904762	0		inf	#DIV/0!
MIR148A	0.04761904762	0.04761904762	0.2222222222		0.1375	#DIV/0!
MIR148B	0.04761904762	0.04761904762	0.05555555556		0.7	#DIV/0!
MIR149	0.04761904762	0.04761904762	0.05555555556		0.7	#DIV/0!
MIR150	0.09523809524	0.09523809524	0.1111111111		0.6842105263	#DIV/0!
MIR151A	0.04761904762	0.04761904762	0.2222222222		0.1375	#DIV/0!
MIR152	0.1428571429	0.1428571429	0		inf	#DIV/0!
MIR153-	0.1428571429	0.1428571429	0.05555555556		2.333333333	#DIV/0!
MIR1537	0.1428571429	0.1428571429	0.05555555556		2.333333333	#DIV/0!
MIR1538	0.1428571429	0.1428571429	0.05555555556		2.333333333	#DIV/0!
MIR1539	0.04761904762	0.04761904762	0		inf	#DIV/0!
MIR155	0.04761904762	0.04761904762	0.1111111111		0.325	#DIV/0!
IR155H	0.04761904762	0.04761904762	0.1111111111		0.325	#DIV/0!
MIR15B	0.1428571429	0.1428571429	0.05555555556		2.333333333	#DIV/0!
MIR16-2	0.1428571429	0.1428571429	0.05555555556		2.333333333	#DIV/0!
IR181A	0.1428571429	0.1428571429	0.1111111111		1.083333333	#DIV/0!
R181A1	0.1428571429	0.1428571429	0.1111111111		1.083333333	#DIV/0!
IR181A	0.09523809524	0.09523809524	0.05555555556		1.473684211	#DIV/0!
R181A2	0.09523809524	0.09523809524	0.05555555556		1.473684211	#DIV/0!
IR181B	0.1428571429	0.1428571429	0.1111111111		1.083333333	#DIV/0!
IR181B	0.09523809524	0.09523809524	0.05555555556		1.473684211	#DIV/0!
MIR181C	0.1428571429	0.1428571429	0.1111111111		1.083333333	#DIV/0!
MIR181D	0.1428571429	0.1428571429	0.1111111111		1.083333333	#DIV/0!
MIR182	0.04761904762	0.04761904762	0.05555555556		0.7	#DIV/0!
MIR1825	0.09523809524	0.09523809524	0.1111111111		0.6842105263	#DIV/0!
MIR1827	0.04761904762	0.04761904762	0.05555555556		0.7	#DIV/0!
MIR183	0.04761904762	0.04761904762	0.05555555556		0.7	#DIV/0!
MIR184	0.04761904762	0.04761904762	0.05555555556		0.7	#DIV/0!
MIR185	0.04761904762	0.04761904762	0.05555555556		0.7	#DIV/0!
MIR186	0.04761904762	0.04761904762	0.05555555556		0.7	#DIV/0!
MIR187	0.09523809524	0.09523809524	0		inf	#DIV/0!
MIR1909	0.09523809524	0.09523809524	0.1111111111		0.6842105263	#DIV/0!
MIR190A	0.04761904762	0.04761904762	0.05555555556		0.7	#DIV/0!
MIR190B	0.1904761905	0.1904761905	0.05555555556		3.294117647	#DIV/0!
MIR191	0.09523809524	0.09523809524	0		inf	#DIV/0!

MIR1910	0.04761904762	0.04761904762	0		inf	#DIV/0!
MIR1914	0.1428571429	0.1428571429	0.2222222222		0.4583333333	#DIV/0!
MIR1915	0.09523809524	0.09523809524	0.1111111111		0.6842105263	#DIV/0!
MIR193A	0	0	0			#DIV/0!
MIR193B	0.04761904762	0.04761904762	0		inf	#DIV/0!
IR193BH	0.04761904762	0.04761904762	0		inf	#DIV/0!
MIR194-	0.1428571429	0.1428571429	0.05555555556		2.333333333	#DIV/0!
MIR195	0	0	0.05555555556		0	#DIV/0!
MIR196A	0.1428571429	0.1428571429	0		inf	#DIV/0!
MIR196A	0.04761904762	0.04761904762	0.05555555556		0.7	#DIV/0!
MIR196B	0.04761904762	0.04761904762	0.2222222222		0.1375	#DIV/0!
MIR197	0.04761904762	0.04761904762	0.05555555556		0.7	#DIV/0!
MIR1972-	0.1428571429	0.1428571429	0.05555555556		2.333333333	#DIV/0!
MIR1972-	0.1428571429	0.1428571429	0.05555555556		2.333333333	#DIV/0!
MIR1973	0	0	0.05555555556		0	#DIV/0!
MIR1976	0.04761904762	0.04761904762	0.1111111111		0.325	#DIV/0!
MIR198	0.09523809524	0.09523809524	0.05555555556		1.473684211	#DIV/0!
MIR199A	0.1428571429	0.1428571429	0.1111111111		1.083333333	#DIV/0!
MIR199A	0.1428571429	0.1428571429	0.05555555556		2.333333333	#DIV/0!
MIR199B	0.09523809524	0.09523809524	0.05555555556		1.473684211	#DIV/0!
MIR200A	0	0	0.1111111111		0	#DIV/0!
MIR200B	0	0	0.1111111111		0	#DIV/0!
MIR200C	0.04761904762	0.04761904762	0.1111111111		0.325	#DIV/0!
IR200CH	0.04761904762	0.04761904762	0.1111111111		0.325	#DIV/0!
MIR202	0.1904761905	0.1904761905	0		inf	#DIV/0!
IR202H	0.1904761905	0.1904761905	0		inf	#DIV/0!
MIR204	0	0	0.05555555556		0	#DIV/0!
MIR205	0.1428571429	0.1428571429	0		inf	#DIV/0!
MIR2052	0.1904761905	0.1904761905	0.1111111111		1.529411765	#DIV/0!
IR2052H	0.1904761905	0.1904761905	0.1111111111		1.529411765	#DIV/0!
MIR2053	0.1904761905	0.1904761905	0.1111111111		1.529411765	#DIV/0!
MIR2054	0	0	0.05555555556		0	#DIV/0!
IR205H	0.1428571429	0.1428571429	0		inf	#DIV/0!
MIR206	0.04761904762	0.04761904762	0		inf	#DIV/0!
MIR208A	0	0	0.05555555556		0	#DIV/0!
MIR208B	0	0	0.05555555556		0	#DIV/0!
MIR21	0.1428571429	0.1428571429	0		inf	#DIV/0!
MIR210	0.04761904762	0.04761904762	0		inf	#DIV/0!
IR210H	0.04761904762	0.04761904762	0		inf	#DIV/0!
MIR2110	0.1428571429	0.1428571429	0.05555555556		2.333333333	#DIV/0!
MIR2115	0.09523809524	0.09523809524	0		inf	#DIV/0!
MIR2116	0.04761904762	0.04761904762	0.05555555556		0.7	#DIV/0!
MIR2117	0.04761904762	0.04761904762	0		inf	#DIV/0!
IR2117H	0.04761904762	0.04761904762	0		inf	#DIV/0!
MIR212	0	0	0.1111111111		0	#DIV/0!
MIR214	0.1428571429	0.1428571429	0.05555555556		2.333333333	#DIV/0!
MIR215	0.1428571429	0.1428571429	0.05555555556		2.333333333	#DIV/0!
MIR216A	0.04761904762	0.04761904762	0.05555555556		0.7	#DIV/0!
MIR216B	0.04761904762	0.04761904762	0.05555555556		0.7	#DIV/0!
MIR217	0.04761904762	0.04761904762	0.05555555556		0.7	#DIV/0!
IR217H	0.04761904762	0.04761904762	0.05555555556		0.7	#DIV/0!

**IIR218-**	0	0	0.05555555556		0	#DIV/0!
**IIR219A**	0.04761904762	0.04761904762	0		inf	#DIV/0!
**IIR219A**	0.09523809524	0.09523809524	0.05555555556		1.473684211	#DIV/0!
**IIR219I**	0.09523809524	0.09523809524	0.05555555556		1.473684211	#DIV/0!
**MIR22**	0	0	0.1111111111		0	#DIV/0!
**MIR2277**	0.04761904762	0.04761904762	0		inf	#DIV/0!
**MIR2278**	0.04761904762	0.04761904762	0.05555555556		0.7	#DIV/0!
**MIR22H(**	0	0	0.1111111111		0	#DIV/0!
**MIR2355**	0	0	0.05555555556		0	#DIV/0!
**MIR23A**	0.1428571429	0.1428571429	0.1111111111		1.083333333	#DIV/0!
**MIR23B**	0.04761904762	0.04761904762	0.05555555556		0.7	#DIV/0!
**MIR24-1**	0.04761904762	0.04761904762	0.05555555556		0.7	#DIV/0!
**MIR24-2**	0.1428571429	0.1428571429	0.1111111111		1.083333333	#DIV/0!
**MIR2467**	0	0	0.05555555556		0	#DIV/0!
**MIR25**	0.04761904762	0.04761904762	0.05555555556		0.7	#DIV/0!
**MIR2682**	0.04761904762	0.04761904762	0.05555555556		0.7	#DIV/0!
**MIR26A1**	0.04761904762	0.04761904762	0		inf	#DIV/0!
**IIR26A2**	0.1428571429	0.1428571429	0.05555555556		2.333333333	#DIV/0!
**MIR26B**	0	0	0.05555555556		0	#DIV/0!
**MIR27A**	0.1428571429	0.1428571429	0.1111111111		1.083333333	#DIV/0!
**MIR27B**	0.04761904762	0.04761904762	0.05555555556		0.7	#DIV/0!
**MIR28**	0.09523809524	0.09523809524	0.1111111111		0.6842105263	#DIV/0!
**MIR2861**	0.09523809524	0.09523809524	0.05555555556		1.473684211	#DIV/0!
**MIR2909**	0.04761904762	0.04761904762	0		inf	#DIV/0!
**MIR296**	0.1428571429	0.1428571429	0.2222222222		0.4583333333	#DIV/0!
**MIR297**	0	0	0.05555555556		0	#DIV/0!
**MIR298**	0.1428571429	0.1428571429	0.2222222222		0.4583333333	#DIV/0!
**MIR29A**	0.04761904762	0.04761904762	0.05555555556		0.7	#DIV/0!
**MIR29B1**	0.04761904762	0.04761904762	0.05555555556		0.7	#DIV/0!
**MIR29B2**	0.1428571429	0.1428571429	0		inf	#DIV/0!
**R29B2C**	0.1428571429	0.1428571429	0		inf	#DIV/0!
**MIR29C**	0.1428571429	0.1428571429	0		inf	#DIV/0!
**MIR301A**	0.1428571429	0.1428571429	0		inf	#DIV/0!
**MIR301I**	0.04761904762	0.04761904762	0.05555555556		0.7	#DIV/0!
**MIR302A**	0	0	0.05555555556		0	#DIV/0!
**MIR302I**	0	0	0.05555555556		0	#DIV/0!
**MIR302C**	0	0	0.05555555556		0	#DIV/0!
**IR302CH**	0	0	0.05555555556		0	#DIV/0!
**MIR302I**	0	0	0.05555555556		0	#DIV/0!
**MIR302I**	0.04761904762	0.04761904762	0		inf	#DIV/0!
**MIR302I**	0.09523809524	0.09523809524	0		inf	#DIV/0!
**MIR3064**	0.04761904762	0.04761904762	0		inf	#DIV/0!
**MIR3065**	0.04761904762	0.04761904762	0		inf	#DIV/0!
**MIR3074**	0.04761904762	0.04761904762	0.05555555556		0.7	#DIV/0!
**MIR30B**	0.04761904762	0.04761904762	0.2222222222		0.1375	#DIV/0!
**MIR30C1**	0.04761904762	0.04761904762	0.05555555556		0.7	#DIV/0!
**MIR30D**	0.04761904762	0.04761904762	0.2222222222		0.1375	#DIV/0!
**MIR30E**	0.04761904762	0.04761904762	0.05555555556		0.7	#DIV/0!
**MIR31**	0.09523809524	0.09523809524	0.1111111111		0.6842105263	#DIV/0!
**MIR3115**	0.04761904762	0.04761904762	0.1111111111		0.325	#DIV/0!
**IIR3116-**	0.04761904762	0.04761904762	0.05555555556		0.7	#DIV/0!

IIR3116-	0.04761904762	0.04761904762	0.05555555556		0.7	#DIV/0!
MIR3117	0.04761904762	0.04761904762	0.05555555556		0.7	#DIV/0!
IIR3118-	0.04761904762	0.04761904762	0		inf	#DIV/0!
IIR3118-	0.2857142857	0.2857142857	0.1111111111		2.6	#DIV/0!
IIR3118-	0.2857142857	0.2857142857	0.1111111111		2.6	#DIV/0!
IIR3118-	0.2857142857	0.2857142857	0.1111111111		2.6	#DIV/0!
IIR3119-	0.1428571429	0.1428571429	0.05555555556		2.333333333	#DIV/0!
IIR3119-	0.1428571429	0.1428571429	0.05555555556		2.333333333	#DIV/0!
MIR3120	0.1428571429	0.1428571429	0.05555555556		2.333333333	#DIV/0!
MIR3121	0.1428571429	0.1428571429	0		inf	#DIV/0!
MIR3122	0.1428571429	0.1428571429	0		inf	#DIV/0!
MIR3123	0.1428571429	0.1428571429	0		inf	#DIV/0!
MIR3124	0.1428571429	0.1428571429	0		inf	#DIV/0!
MIR3125	0	0	0.05555555556		0	#DIV/0!
MIR3126	0	0	0.05555555556		0	#DIV/0!
MIR3127	0	0	0.1111111111		0	#DIV/0!
MIR3128	0	0	0.1111111111		0	#DIV/0!
MIR3129	0	0	0.1666666667		0	#DIV/0!
IIR3130-	0	0	0.05555555556		0	#DIV/0!
IIR3130-	0	0	0.05555555556		0	#DIV/0!
MIR3131	0	0	0.05555555556		0	#DIV/0!
MIR3132	0	0	0.05555555556		0	#DIV/0!
MIR3133	0	0	0.05555555556		0	#DIV/0!
MIR3134	0.04761904762	0.04761904762	0		inf	#DIV/0!
IIR3135.	0.04761904762	0.04761904762	0		inf	#DIV/0!
IIR3135I	0.04761904762	0.04761904762	0		inf	#DIV/0!
MIR3136	0.04761904762	0.04761904762	0		inf	#DIV/0!
MIR3137	0.09523809524	0.09523809524	0.05555555556		1.473684211	#DIV/0!
MIR3139	0	0	0.05555555556		0	#DIV/0!
MIR3140	0	0	0.05555555556		0	#DIV/0!
MIR3141	0	0	0.05555555556		0	#DIV/0!
MIR3142	0.04761904762	0.04761904762	0.05555555556		0.7	#DIV/0!
IR3142H	0.04761904762	0.04761904762	0.05555555556		0.7	#DIV/0!
MIR3146	0.04761904762	0.04761904762	0.2222222222		0.1375	#DIV/0!
MIR3147	0.1428571429	0.1428571429	0.1666666667		0.6666666667	#DIV/0!
MIR3148	0.04761904762	0.04761904762	0.1111111111		0.325	#DIV/0!
MIR3149	0.1904761905	0.1904761905	0.1111111111		1.529411765	#DIV/0!
IIR3150.	0.1904761905	0.1904761905	0.1111111111		1.529411765	#DIV/0!
IIR3150I	0.1904761905	0.1904761905	0.1111111111		1.529411765	#DIV/0!
R3150BI	0.1904761905	0.1904761905	0.1111111111		1.529411765	#DIV/0!
MIR3151	0.1904761905	0.1904761905	0.1111111111		1.529411765	#DIV/0!
MIR3152	0.04761904762	0.04761904762	0.05555555556		0.7	#DIV/0!
MIR3153	0	0	0.05555555556		0	#DIV/0!
MIR3154	0.09523809524	0.09523809524	0.05555555556		1.473684211	#DIV/0!
IIR3155.	0.09523809524	0.09523809524	0.1111111111		0.6842105263	#DIV/0!
IIR3155I	0.09523809524	0.09523809524	0.1111111111		0.6842105263	#DIV/0!
IIR3156-	0.1428571429	0.1428571429	0.05555555556		2.333333333	#DIV/0!
IIR3156-	0.09523809524	0.09523809524	0.05555555556		1.473684211	#DIV/0!
IIR3156-	0.04761904762	0.04761904762	0		inf	#DIV/0!
MIR3157	0.1428571429	0.1428571429	0		inf	#DIV/0!
IIR3158-	0.1428571429	0.1428571429	0		inf	#DIV/0!

IIR3158-	0.1428571429	0.1428571429	0		inf	#DIV/0!
IIR3160-	0	0	0.05555555556		0	#DIV/0!
IIR3160-	0	0	0.05555555556		0	#DIV/0!
MIR3161	0	0	0.05555555556		0	#DIV/0!
MIR3174	0.04761904762	0.04761904762	0.05555555556		0.7	#DIV/0!
MIR3175	0.04761904762	0.04761904762	0.05555555556		0.7	#DIV/0!
MIR3176	0.04761904762	0.04761904762	0.2222222222		0.1375	#DIV/0!
MIR3177	0.09523809524	0.09523809524	0.2222222222		0.2894736842	#DIV/0!
MIR3178	0.04761904762	0.04761904762	0.1111111111		0.325	#DIV/0!
IIR3179-	0.09523809524	0.09523809524	0		inf	#DIV/0!
IIR3179-	0.09523809524	0.09523809524	0		inf	#DIV/0!
IIR3179-	0.09523809524	0.09523809524	0		inf	#DIV/0!
IIR3179-	0.09523809524	0.09523809524	0		inf	#DIV/0!
IIR3180-	0.09523809524	0.09523809524	0		inf	#DIV/0!
IIR3180-	0.09523809524	0.09523809524	0		inf	#DIV/0!
IIR3180-	0.09523809524	0.09523809524	0		inf	#DIV/0!
IIR3180-	0.04761904762	0.04761904762	0		inf	#DIV/0!
IIR3180-	0.09523809524	0.09523809524	0.2222222222		0.2894736842	#DIV/0!
MIR3181	0.04761904762	0.04761904762	0		inf	#DIV/0!
MIR3182	0.04761904762	0.04761904762	0		inf	#DIV/0!
MIR3183	0	0	0.1111111111		0	#DIV/0!
MIR3184	0	0	0.05555555556		0	#DIV/0!
MIR3185	0.1428571429	0.1428571429	0		inf	#DIV/0!
MIR3186	0.04761904762	0.04761904762	0		inf	#DIV/0!
MIR3187	0.09523809524	0.09523809524	0.1111111111		0.6842105263	#DIV/0!
MIR3188	0.1428571429	0.1428571429	0.1111111111		1.083333333	#DIV/0!
MIR3189	0.1428571429	0.1428571429	0.1111111111		1.083333333	#DIV/0!
MIR3190	0.09523809524	0.09523809524	0.1111111111		0.6842105263	#DIV/0!
MIR3191	0.09523809524	0.09523809524	0.1111111111		0.6842105263	#DIV/0!
MIR3192	0.1904761905	0.1904761905	0.05555555556		3.294117647	#DIV/0!
MIR3193	0.09523809524	0.09523809524	0.1111111111		0.6842105263	#DIV/0!
MIR3194	0.09523809524	0.09523809524	0.1666666667		0.4210526316	#DIV/0!
MIR3195	0.1428571429	0.1428571429	0.2222222222		0.4583333333	#DIV/0!
MIR3196	0.1428571429	0.1428571429	0.2222222222		0.4583333333	#DIV/0!
MIR3197	0.04761904762	0.04761904762	0.1111111111		0.325	#DIV/0!
IIR3198-	0.04761904762	0.04761904762	0.05555555556		0.7	#DIV/0!
IIR3198-	0.04761904762	0.04761904762	0.05555555556		0.7	#DIV/0!
IIR3199-	0.04761904762	0.04761904762	0.1111111111		0.325	#DIV/0!
IIR3199-	0.04761904762	0.04761904762	0.1111111111		0.325	#DIV/0!
IIR31HG	0.09523809524	0.09523809524	0.1111111111		0.6842105263	#DIV/0!
MIR32	0.04761904762	0.04761904762	0.05555555556		0.7	#DIV/0!
MIR3200	0.04761904762	0.04761904762	0.1111111111		0.325	#DIV/0!
MIR3201	0.04761904762	0.04761904762	0.1666666667		0.2	#DIV/0!
MIR320A	0.09523809524	0.09523809524	0.1111111111		0.6842105263	#DIV/0!
IIR320B	0.04761904762	0.04761904762	0.05555555556		0.7	#DIV/0!
IIR320B	0.1428571429	0.1428571429	0.05555555556		2.333333333	#DIV/0!
IIR320C	0.04761904762	0.04761904762	0.1111111111		0.325	#DIV/0!
IIR320C	0.09523809524	0.09523809524	0.05555555556		1.473684211	#DIV/0!
MIR320D	0.09523809524	0.09523809524	0.1111111111		0.6842105263	#DIV/0!
MIR324	0	0	0.05555555556		0	#DIV/0!
MIR328	0.1428571429	0.1428571429	0.05555555556		2.333333333	#DIV/0!

MIR330	0.09523809524	0.09523809524	0.1111111111		0.6842105263	#DIV/0!
MIR331	0.04761904762	0.04761904762	0.05555555556		0.7	#DIV/0!
MIR335	0.04761904762	0.04761904762	0.05555555556		0.7	#DIV/0!
MIR338	0.04761904762	0.04761904762	0		inf	#DIV/0!
MIR339	0.04761904762	0.04761904762	0.1666666667		0.2	#DIV/0!
MIR33A	0	0	0.1111111111		0	#DIV/0!
MIR33B	0.04761904762	0.04761904762	0.1111111111		0.325	#DIV/0!
MIR340	0.09523809524	0.09523809524	0.05555555556		1.473684211	#DIV/0!
MIR346	0.1428571429	0.1428571429	0.05555555556		2.333333333	#DIV/0!
MIR34A	0	0	0.05555555556		0	#DIV/0!
IR34AH	0	0	0.05555555556		0	#DIV/0!
MIR3529	0.04761904762	0.04761904762	0.05555555556		0.7	#DIV/0!
MIR3591	0.09523809524	0.09523809524	0		inf	#DIV/0!
MIR3605	0.04761904762	0.04761904762	0.05555555556		0.7	#DIV/0!
MIR3606	0	0	0.1666666667		0	#DIV/0!
MIR3609	0.04761904762	0.04761904762	0.05555555556		0.7	#DIV/0!
MIR3610	0.1904761905	0.1904761905	0.1666666667		0.9411764706	#DIV/0!
MIR3611	0.09523809524	0.09523809524	0.1666666667		0.4210526316	#DIV/0!
MIR3612	0.09523809524	0.09523809524	0.05555555556		1.473684211	#DIV/0!
MIR3614	0.09523809524	0.09523809524	0		inf	#DIV/0!
MIR3615	0.04761904762	0.04761904762	0		inf	#DIV/0!
MIR3616	0.1428571429	0.1428571429	0.1666666667		0.6666666667	#DIV/0!
MIR3617	0.1428571429	0.1428571429	0.1666666667		0.6666666667	#DIV/0!
MIR3618	0.04761904762	0.04761904762	0.05555555556		0.7	#DIV/0!
MIR3619	0	0	0.1111111111		0	#DIV/0!
MIR3620	0.1428571429	0.1428571429	0		inf	#DIV/0!
MIR3621	0.1428571429	0.1428571429	0.05555555556		2.333333333	#DIV/0!
IIR3622	0.04761904762	0.04761904762	0.1111111111		0.325	#DIV/0!
IIR3622	0.04761904762	0.04761904762	0.1111111111		0.325	#DIV/0!
MIR3646	0.1428571429	0.1428571429	0.1666666667		0.6666666667	#DIV/0!
IIR3648-	0.04761904762	0.04761904762	0		inf	#DIV/0!
IIR3648-	0.04761904762	0.04761904762	0		inf	#DIV/0!
MIR3649	0.04761904762	0.04761904762	0.1111111111		0.325	#DIV/0!
MIR3650	0.04761904762	0.04761904762	0.05555555556		0.7	#DIV/0!
MIR3651	0.04761904762	0.04761904762	0.05555555556		0.7	#DIV/0!
MIR3652	0.04761904762	0.04761904762	0.05555555556		0.7	#DIV/0!
MIR3654	0.04761904762	0.04761904762	0.05555555556		0.7	#DIV/0!
MIR3655	0.04761904762	0.04761904762	0		inf	#DIV/0!
MIR3657	0	0	0.1111111111		0	#DIV/0!
MIR3658	0.1428571429	0.1428571429	0		inf	#DIV/0!
MIR3659	0.04761904762	0.04761904762	0.05555555556		0.7	#DIV/0!
MIR365A	0.04761904762	0.04761904762	0		inf	#DIV/0!
MIR365B	0	0	0			#DIV/0!
MIR3660	0.04761904762	0.04761904762	0		inf	#DIV/0!
MIR3661	0.04761904762	0.04761904762	0		inf	#DIV/0!
MIR3663	0.2380952381	0.2380952381	0		inf	#DIV/0!
IR3663H	0.2380952381	0.2380952381	0		inf	#DIV/0!
MIR3666	0.04761904762	0.04761904762	0.1111111111		0.325	#DIV/0!
MIR3667	0.04761904762	0.04761904762	0.1666666667		0.2	#DIV/0!
MIR367	0	0	0.05555555556		0	#DIV/0!
IIR3670-	0.09523809524	0.09523809524	0		inf	#DIV/0!

**IIR3670-**	0.09523809524	0.09523809524	0		inf	#DIV/0!
**IIR3670-**	0.09523809524	0.09523809524	0		inf	#DIV/0!
**IIR3670-**	0.09523809524	0.09523809524	0		inf	#DIV/0!
**MIR3671**	0.04761904762	0.04761904762	0.05555555556		0.7	#DIV/0!
**MIR3674**	0.1428571429	0.1428571429	0.1111111111		1.083333333	#DIV/0!
**MIR3675**	0.04761904762	0.04761904762	0.1111111111		0.325	#DIV/0!
**MIR3677**	0.09523809524	0.09523809524	0.2222222222		0.2894736842	#DIV/0!
**MIR3678**	0.04761904762	0.04761904762	0.05555555556		0.7	#DIV/0!
**MIR3679**	0	0	0.05555555556		0	#DIV/0!
**IIR3680-**	0.04761904762	0.04761904762	0		inf	#DIV/0!
**IIR3680-**	0.04761904762	0.04761904762	0		inf	#DIV/0!
**MIR3681**	0	0	0.05555555556		0	#DIV/0!
**IR3681H**	0	0	0.05555555556		0	#DIV/0!
**MIR3682**	0.04761904762	0.04761904762	0.05555555556		0.7	#DIV/0!
**MIR3683**	0.04761904762	0.04761904762	0.1666666667		0.2	#DIV/0!
**MIR3684**	0	0	0.05555555556		0	#DIV/0!
**MIR3685**	0.04761904762	0.04761904762	0.05555555556		0.7	#DIV/0!
**MIR3686**	0.04761904762	0.04761904762	0.1111111111		0.325	#DIV/0!
**IIR3688-**	0	0	0.05555555556		0	#DIV/0!
**IIR3688-**	0	0	0.05555555556		0	#DIV/0!
**IIR3689**	0.04761904762	0.04761904762	0		inf	#DIV/0!
**IIR3689**	0.04761904762	0.04761904762	0		inf	#DIV/0!
**IIR3689**	0.04761904762	0.04761904762	0		inf	#DIV/0!
**IR3689D**	0.04761904762	0.04761904762	0		inf	#DIV/0!
**IR3689D**	0.04761904762	0.04761904762	0		inf	#DIV/0!
**IIR3689**	0.04761904762	0.04761904762	0		inf	#DIV/0!
**IIR3689**	0.04761904762	0.04761904762	0		inf	#DIV/0!
**MIR3713**	0.04761904762	0.04761904762	0.05555555556		0.7	#DIV/0!
**MIR3714**	0.04761904762	0.04761904762	0		inf	#DIV/0!
**MIR371A**	0.09523809524	0.09523809524	0.05555555556		1.473684211	#DIV/0!
**MIR371B**	0.09523809524	0.09523809524	0.05555555556		1.473684211	#DIV/0!
**MIR372**	0.09523809524	0.09523809524	0.05555555556		1.473684211	#DIV/0!
**MIR373**	0.09523809524	0.09523809524	0.05555555556		1.473684211	#DIV/0!
**MIR375**	0	0	0.05555555556		0	#DIV/0!
**MIR378B**	0.04761904762	0.04761904762	0		inf	#DIV/0!
**MIR378C**	0.2857142857	0.2857142857	0		inf	#DIV/0!
**IIR378D**	0.1904761905	0.1904761905	0.1111111111		1.529411765	#DIV/0!
**MIR378E**	0	0	0.05555555556		0	#DIV/0!
**MIR378F**	0.04761904762	0.04761904762	0.1111111111		0.325	#DIV/0!
**MIR378G**	0.04761904762	0.04761904762	0.05555555556		0.7	#DIV/0!
**MIR378H**	0	0	0.05555555556		0	#DIV/0!
**MIR378I**	0	0	0.1111111111		0	#DIV/0!
**MIR383**	0.09523809524	0.09523809524	0.1111111111		0.6842105263	#DIV/0!
**MIR3907**	0.09523809524	0.09523809524	0.05555555556		1.473684211	#DIV/0!
**MIR3908**	0.04761904762	0.04761904762	0.1111111111		0.325	#DIV/0!
**MIR3909**	0	0	0.1111111111		0	#DIV/0!
**IIR3910-**	0.04761904762	0.04761904762	0.05555555556		0.7	#DIV/0!
**IIR3910-**	0.04761904762	0.04761904762	0.05555555556		0.7	#DIV/0!
**MIR3911**	0.09523809524	0.09523809524	0.05555555556		1.473684211	#DIV/0!
**MIR3912**	0	0	0.05555555556		0	#DIV/0!
**IIR3913-**	0.09523809524	0.09523809524	0.1111111111		0.6842105263	#DIV/0!

IIR3913-	0.09523809524	0.09523809524	0.1111111111		0.6842105263	#DIV/0!
IIR3914-	0.09523809524	0.09523809524	0.1111111111		0.6842105263	#DIV/0!
IIR3914-	0.09523809524	0.09523809524	0.1111111111		0.6842105263	#DIV/0!
MIR3916	0.1428571429	0.1428571429	0		inf	#DIV/0!
MIR3917	0.04761904762	0.04761904762	0.1111111111		0.325	#DIV/0!
MIR3919	0.1428571429	0.1428571429	0.05555555556		2.333333333	#DIV/0!
MIR3921	0.09523809524	0.09523809524	0.1111111111		0.6842105263	#DIV/0!
MIR3922	0.04761904762	0.04761904762	0.05555555556		0.7	#DIV/0!
MIR3923	0.04761904762	0.04761904762	0		inf	#DIV/0!
MIR3924	0.09523809524	0.09523809524	0.05555555556		1.473684211	#DIV/0!
MIR3925	0.04761904762	0.04761904762	0.05555555556		0.7	#DIV/0!
IIR3926-	0.09523809524	0.09523809524	0.1111111111		0.6842105263	#DIV/0!
IIR3926-	0.09523809524	0.09523809524	0.1111111111		0.6842105263	#DIV/0!
MIR3927	0.04761904762	0.04761904762	0.05555555556		0.7	#DIV/0!
MIR3928	0.04761904762	0.04761904762	0.1111111111		0.325	#DIV/0!
MIR3929	0.09523809524	0.09523809524	0		inf	#DIV/0!
MIR3934	0.04761904762	0.04761904762	0		inf	#DIV/0!
MIR3935	0.04761904762	0.04761904762	0		inf	#DIV/0!
MIR3936	0.04761904762	0.04761904762	0		inf	#DIV/0!
IR3936H	0.04761904762	0.04761904762	0		inf	#DIV/0!
MIR3938	0.04761904762	0.04761904762	0		inf	#DIV/0!
MIR3940	0.1428571429	0.1428571429	0.05555555556		2.333333333	#DIV/0!
MIR3941	0.1428571429	0.1428571429	0.05555555556		2.333333333	#DIV/0!
MIR3942	0	0	0			#DIV/0!
MIR3943	0.04761904762	0.04761904762	0.1666666667		0.2	#DIV/0!
MIR3944	0.1904761905	0.1904761905	0		inf	#DIV/0!
MIR3945	0	0	0.05555555556		0	#DIV/0!
IR3945H	0	0	0.05555555556		0	#DIV/0!
MIR3960	0.09523809524	0.09523809524	0.05555555556		1.473684211	#DIV/0!
MIR3972	0.04761904762	0.04761904762	0.1111111111		0.325	#DIV/0!
MIR3974	0.04761904762	0.04761904762	0.1111111111		0.325	#DIV/0!
MIR3975	0.09523809524	0.09523809524	0		inf	#DIV/0!
MIR3976	0.09523809524	0.09523809524	0		inf	#DIV/0!
IR3976H	0.09523809524	0.09523809524	0		inf	#DIV/0!
MIR3977	0.04761904762	0.04761904762	0.05555555556		0.7	#DIV/0!
MIR422A	0.04761904762	0.04761904762	0.05555555556		0.7	#DIV/0!
MIR423	0	0	0.05555555556		0	#DIV/0!
MIR425	0.09523809524	0.09523809524	0		inf	#DIV/0!
MIR4251	0	0	0.05555555556		0	#DIV/0!
MIR4252	0	0	0.05555555556		0	#DIV/0!
MIR4253	0.04761904762	0.04761904762	0.1111111111		0.325	#DIV/0!
MIR4254	0.04761904762	0.04761904762	0.1111111111		0.325	#DIV/0!
MIR4255	0.04761904762	0.04761904762	0		inf	#DIV/0!
MIR4256	0.04761904762	0.04761904762	0.05555555556		0.7	#DIV/0!
MIR4257	0.1904761905	0.1904761905	0.05555555556		3.294117647	#DIV/0!
MIR4258	0.1904761905	0.1904761905	0.1111111111		1.529411765	#DIV/0!
MIR4260	0.1428571429	0.1428571429	0		inf	#DIV/0!
MIR4261	0	0	0.05555555556		0	#DIV/0!
MIR4262	0	0	0.05555555556		0	#DIV/0!
MIR4263	0	0	0.1111111111		0	#DIV/0!
MIR4264	0	0	0.05555555556		0	#DIV/0!

MIR4265	0	0	0.05555555556		0	#DIV/0!
MIR4266	0	0	0.05555555556		0	#DIV/0!
MIR4267	0	0	0.05555555556		0	#DIV/0!
MIR4268	0	0	0.05555555556		0	#DIV/0!
MIR4269	0	0	0.05555555556		0	#DIV/0!
MIR4270	0.04761904762	0.04761904762	0		inf	#DIV/0!
MIR4271	0.09523809524	0.09523809524	0		inf	#DIV/0!
MIR4272	0.04761904762	0.04761904762	0		inf	#DIV/0!
MIR4273	0.04761904762	0.04761904762	0		inf	#DIV/0!
MIR4276	0	0	0.05555555556		0	#DIV/0!
MIR4277	0	0	0.1111111111		0	#DIV/0!
MIR4278	0	0	0.05555555556		0	#DIV/0!
MIR4279	0.04761904762	0.04761904762	0.05555555556		0.7	#DIV/0!
MIR4280	0.04761904762	0.04761904762	0		inf	#DIV/0!
MIR4281	0.09523809524	0.09523809524	0.05555555556		1.473684211	#DIV/0!
IR4283-	0.1428571429	0.1428571429	0.1666666667		0.6666666667	#DIV/0!
IR4283-	0.1428571429	0.1428571429	0.1666666667		0.6666666667	#DIV/0!
MIR4284	0.1428571429	0.1428571429	0.1111111111		1.083333333	#DIV/0!
MIR4285	0.04761904762	0.04761904762	0.05555555556		0.7	#DIV/0!
MIR4286	0.09523809524	0.09523809524	0.1111111111		0.6842105263	#DIV/0!
MIR4287	0.04761904762	0.04761904762	0.1111111111		0.325	#DIV/0!
MIR4288	0.04761904762	0.04761904762	0.1111111111		0.325	#DIV/0!
MIR4289	0	0	0.05555555556		0	#DIV/0!
MIR429	0	0	0.1111111111		0	#DIV/0!
MIR4290	0.09523809524	0.09523809524	0.05555555556		1.473684211	#DIV/0!
IR4290H	0.09523809524	0.09523809524	0.05555555556		1.473684211	#DIV/0!
MIR4291	0.04761904762	0.04761904762	0.05555555556		0.7	#DIV/0!
MIR4292	0.1428571429	0.1428571429	0.05555555556		2.333333333	#DIV/0!
MIR4293	0.09523809524	0.09523809524	0.1111111111		0.6842105263	#DIV/0!
MIR4294	0.09523809524	0.09523809524	0.05555555556		1.473684211	#DIV/0!
MIR4295	0.1428571429	0.1428571429	0.05555555556		2.333333333	#DIV/0!
MIR4296	0.2380952381	0.2380952381	0.05555555556		4.375	#DIV/0!
MIR4297	0.2857142857	0.2857142857	0		inf	#DIV/0!
MIR4298	0.04761904762	0.04761904762	0		inf	#DIV/0!
MIR4302	0.04761904762	0.04761904762	0.05555555556		0.7	#DIV/0!
MIR4303	0.1904761905	0.1904761905	0.05555555556		3.294117647	#DIV/0!
MIR4304	0.04761904762	0.04761904762	0.1111111111		0.325	#DIV/0!
MIR4308	0.04761904762	0.04761904762	0		inf	#DIV/0!
MIR4311	0.04761904762	0.04761904762	0.05555555556		0.7	#DIV/0!
MIR4312	0.04761904762	0.04761904762	0.05555555556		0.7	#DIV/0!
MIR4313	0.04761904762	0.04761904762	0.05555555556		0.7	#DIV/0!
MIR4314	0	0	0.05555555556		0	#DIV/0!
IR4315-	0.1428571429	0.1428571429	0		inf	#DIV/0!
IR4315-	0.1428571429	0.1428571429	0		inf	#DIV/0!
MIR4316	0.04761904762	0.04761904762	0		inf	#DIV/0!
MIR4317	0.09523809524	0.09523809524	0		inf	#DIV/0!
MIR4318	0.04761904762	0.04761904762	0		inf	#DIV/0!
MIR4319	0.04761904762	0.04761904762	0		inf	#DIV/0!
MIR4320	0.04761904762	0.04761904762	0		inf	#DIV/0!
MIR4321	0.09523809524	0.09523809524	0.1111111111		0.6842105263	#DIV/0!
MIR4323	0.04761904762	0.04761904762	0.05555555556		0.7	#DIV/0!

MIR4324	0.09523809524	0.09523809524	0.1111111111		0.6842105263	#DIV/0!
MIR4325	0.1428571429	0.1428571429	0.2222222222		0.4583333333	#DIV/0!
MIR4326	0.1428571429	0.1428571429	0.2222222222		0.4583333333	#DIV/0!
MIR4327	0.04761904762	0.04761904762	0.05555555556		0.7	#DIV/0!
MIR4418	0.04761904762	0.04761904762	0.1111111111		0.325	#DIV/0!
MIR4420	0	0	0.1111111111		0	#DIV/0!
MIR4421	0.04761904762	0.04761904762	0.05555555556		0.7	#DIV/0!
MIR4422	0.04761904762	0.04761904762	0.05555555556		0.7	#DIV/0!
IR4422H	0.04761904762	0.04761904762	0.05555555556		0.7	#DIV/0!
MIR4423	0.04761904762	0.04761904762	0.05555555556		0.7	#DIV/0!
MIR4424	0.1428571429	0.1428571429	0		inf	#DIV/0!
MIR4425	0.04761904762	0.04761904762	0.1111111111		0.325	#DIV/0!
MIR4426	0.2380952381	0.2380952381	0.05555555556		4.375	#DIV/0!
MIR4427	0.1428571429	0.1428571429	0		inf	#DIV/0!
MIR4428	0.1428571429	0.1428571429	0		inf	#DIV/0!
MIR4429	0	0	0.05555555556		0	#DIV/0!
MIR4431	0.04761904762	0.04761904762	0.05555555556		0.7	#DIV/0!
MIR4432	0.04761904762	0.04761904762	0.05555555556		0.7	#DIV/0!
IR4432H	0.04761904762	0.04761904762	0.05555555556		0.7	#DIV/0!
IIR4433	0	0	0.05555555556		0	#DIV/0!
IIR4433	0	0	0.05555555556		0	#DIV/0!
MIR4434	0	0	0.05555555556		0	#DIV/0!
IIR4435-	0	0	0.05555555556		0	#DIV/0!
IIR4435-	0	0	0.05555555556		0	#DIV/0!
R4435-2	0	0	0.05555555556		0	#DIV/0!
IIR4436	0	0	0.05555555556		0	#DIV/0!
IR4436E	0	0	0.05555555556		0	#DIV/0!
IR4436E	0	0	0.05555555556		0	#DIV/0!
MIR4437	0	0	0.05555555556		0	#DIV/0!
MIR4438	0	0	0.05555555556		0	#DIV/0!
MIR4439	0	0	0.05555555556		0	#DIV/0!
MIR4440	0	0	0.05555555556		0	#DIV/0!
MIR4441	0	0	0.05555555556		0	#DIV/0!
MIR4443	0.09523809524	0.09523809524	0		inf	#DIV/0!
IIR4444-	0.04761904762	0.04761904762	0.1111111111		0.325	#DIV/0!
IIR4444-	0.04761904762	0.04761904762	0.1111111111		0.325	#DIV/0!
MIR4445	0.09523809524	0.09523809524	0.05555555556		1.473684211	#DIV/0!
MIR4446	0.09523809524	0.09523809524	0.05555555556		1.473684211	#DIV/0!
MIR4447	0.09523809524	0.09523809524	0.05555555556		1.473684211	#DIV/0!
MIR4448	0.1904761905	0.1904761905	0.05555555556		3.294117647	#DIV/0!
MIR4449	0.2380952381	0.2380952381	0.05555555556		4.375	#DIV/0!
MIR4450	0.04761904762	0.04761904762	0.05555555556		0.7	#DIV/0!
MIR4451	0	0	0.05555555556		0	#DIV/0!
MIR4452	0	0	0.05555555556		0	#DIV/0!
MIR4453	0	0	0.05555555556		0	#DIV/0!
IR4453H	0	0	0.05555555556		0	#DIV/0!
MIR4454	0.2857142857	0.2857142857	0.2222222222		1.1	#DIV/0!
MIR4455	0	0	0.05555555556		0	#DIV/0!
MIR4456	0	0	0.05555555556		0	#DIV/0!
MIR4457	0	0	0.1111111111		0	#DIV/0!
MIR4458	0	0	0.05555555556		0	#DIV/0!

IR4458H	0	0	0.05555555556		0	#DIV/0!
MIR4460	0.04761904762	0.04761904762	0		inf	#DIV/0!
MIR4462	0.04761904762	0.04761904762	0.05555555556		0.7	#DIV/0!
MIR4464	0	0	0			#DIV/0!
MIR4467	0.04761904762	0.04761904762	0.05555555556		0.7	#DIV/0!
MIR4468	0.04761904762	0.04761904762	0.05555555556		0.7	#DIV/0!
MIR4469	0.09523809524	0.09523809524	0.1666666667		0.4210526316	#DIV/0!
MIR4470	0.1428571429	0.1428571429	0.1666666667		0.6666666667	#DIV/0!
MIR4471	0.1904761905	0.1904761905	0.1111111111		1.529411765	#DIV/0!
IIR4472-	0.04761904762	0.04761904762	0.1666666667		0.2	#DIV/0!
IIR4472-	0	0	0.05555555556		0	#DIV/0!
MIR4473	0.04761904762	0.04761904762	0.05555555556		0.7	#DIV/0!
MIR4474	0.04761904762	0.04761904762	0.05555555556		0.7	#DIV/0!
MIR4475	0.09523809524	0.09523809524	0.05555555556		1.473684211	#DIV/0!
MIR4476	0.09523809524	0.09523809524	0.05555555556		1.473684211	#DIV/0!
IIR4477	0.09523809524	0.09523809524	0.2222222222		0.2894736842	#DIV/0!
IIR4477	0.09523809524	0.09523809524	0.2222222222		0.2894736842	#DIV/0!
MIR4478	0.09523809524	0.09523809524	0.05555555556		1.473684211	#DIV/0!
MIR4479	0.1428571429	0.1428571429	0.05555555556		2.333333333	#DIV/0!
MIR4480	0.09523809524	0.09523809524	0.1111111111		0.6842105263	#DIV/0!
MIR4481	0.09523809524	0.09523809524	0.1111111111		0.6842105263	#DIV/0!
MIR4482	0.1428571429	0.1428571429	0.05555555556		2.333333333	#DIV/0!
MIR4483	0.1428571429	0.1428571429	0.05555555556		2.333333333	#DIV/0!
MIR4484	0.2380952381	0.2380952381	0.05555555556		4.375	#DIV/0!
MIR4487	0	0	0.05555555556		0	#DIV/0!
MIR4494	0.04761904762	0.04761904762	0.05555555556		0.7	#DIV/0!
MIR4495	0.1904761905	0.1904761905	0.05555555556		3.294117647	#DIV/0!
MIR4496	0.04761904762	0.04761904762	0.05555555556		0.7	#DIV/0!
MIR4498	0	0	0.1111111111		0	#DIV/0!
MIR4499	0.04761904762	0.04761904762	0		inf	#DIV/0!
MIR449A	0.04761904762	0.04761904762	0.05555555556		0.7	#DIV/0!
MIR449B	0.04761904762	0.04761904762	0.05555555556		0.7	#DIV/0!
MIR449C	0.04761904762	0.04761904762	0.05555555556		0.7	#DIV/0!
MIR4504	0.04761904762	0.04761904762	0		inf	#DIV/0!
MIR4505	0.09523809524	0.09523809524	0		inf	#DIV/0!
MIR4507	0.04761904762	0.04761904762	0		inf	#DIV/0!
IIR4509-	0.1428571429	0.1428571429	0		inf	#DIV/0!
IIR4509-	0.1428571429	0.1428571429	0		inf	#DIV/0!
IIR4509-	0.1428571429	0.1428571429	0		inf	#DIV/0!
MIR4510	0	0	0			#DIV/0!
MIR4511	0.04761904762	0.04761904762	0.05555555556		0.7	#DIV/0!
MIR4512	0.04761904762	0.04761904762	0.05555555556		0.7	#DIV/0!
MIR4513	0.04761904762	0.04761904762	0.05555555556		0.7	#DIV/0!
MIR4514	0.04761904762	0.04761904762	0.05555555556		0.7	#DIV/0!
MIR4515	0.04761904762	0.04761904762	0.05555555556		0.7	#DIV/0!
MIR4516	0.09523809524	0.09523809524	0.2222222222		0.2894736842	#DIV/0!
MIR4517	0.04761904762	0.04761904762	0		inf	#DIV/0!
MIR4518	0.04761904762	0.04761904762	0		inf	#DIV/0!
MIR4519	0.04761904762	0.04761904762	0		inf	#DIV/0!
MIR451A	0.04761904762	0.04761904762	0.05555555556		0.7	#DIV/0!
MIR451B	0.04761904762	0.04761904762	0.05555555556		0.7	#DIV/0!

IIR4520-	0	0	0.05555555556		0	#DIV/0!
IIR4520-	0	0	0.05555555556		0	#DIV/0!
MIR4521	0.04761904762	0.04761904762	0.05555555556		0.7	#DIV/0!
MIR4522	0.04761904762	0.04761904762	0.05555555556		0.7	#DIV/0!
MIR4523	0.04761904762	0.04761904762	0.05555555556		0.7	#DIV/0!
IIR4524	0	0	0			#DIV/0!
IIR4524	0	0	0			#DIV/0!
MIR4525	0	0	0			#DIV/0!
MIR4526	0.09523809524	0.09523809524	0.05555555556		1.473684211	#DIV/0!
MIR4527	0.04761904762	0.04761904762	0.05555555556		0.7	#DIV/0!
IR4527H	0.04761904762	0.04761904762	0.05555555556		0.7	#DIV/0!
MIR4528	0.09523809524	0.09523809524	0		inf	#DIV/0!
MIR4529	0.09523809524	0.09523809524	0.05555555556		1.473684211	#DIV/0!
MIR4530	0.04761904762	0.04761904762	0.05555555556		0.7	#DIV/0!
MIR4531	0.09523809524	0.09523809524	0.05555555556		1.473684211	#DIV/0!
MIR4533	0.1428571429	0.1428571429	0.1111111111		1.083333333	#DIV/0!
MIR4534	0.04761904762	0.04761904762	0.1111111111		0.325	#DIV/0!
MIR4535	0.04761904762	0.04761904762	0.1666666667		0.2	#DIV/0!
MIR4537	0.04761904762	0.04761904762	0		inf	#DIV/0!
MIR4538	0.09523809524	0.09523809524	0		inf	#DIV/0!
MIR4539	0.09523809524	0.09523809524	0.1666666667		0.4210526316	#DIV/0!
MIR454	0.1428571429	0.1428571429	0		inf	#DIV/0!
MIR4540	0.09523809524	0.09523809524	0.05555555556		1.473684211	#DIV/0!
MIR455	0.04761904762	0.04761904762	0.05555555556		0.7	#DIV/0!
MIR4632	0.04761904762	0.04761904762	0.1111111111		0.325	#DIV/0!
MIR4633	0.04761904762	0.04761904762	0		inf	#DIV/0!
MIR4634	0	0	0.05555555556		0	#DIV/0!
MIR4635	0	0	0.05555555556		0	#DIV/0!
MIR4636	0	0	0.05555555556		0	#DIV/0!
MIR4637	0.04761904762	0.04761904762	0.05555555556		0.7	#DIV/0!
MIR4638	0.09523809524	0.09523809524	0.05555555556		1.473684211	#DIV/0!
MIR4641	0	0	0.05555555556		0	#DIV/0!
MIR4642	0.04761904762	0.04761904762	0.05555555556		0.7	#DIV/0!
MIR4643	0	0	0			#DIV/0!
MIR4646	0.04761904762	0.04761904762	0		inf	#DIV/0!
MIR4647	0.04761904762	0.04761904762	0.05555555556		0.7	#DIV/0!
MIR4648	0.04761904762	0.04761904762	0.1666666667		0.2	#DIV/0!
MIR4649	0.04761904762	0.04761904762	0.1666666667		0.2	#DIV/0!
IIR4650-	0.1428571429	0.1428571429	0.1111111111		1.083333333	#DIV/0!
IIR4650-	0.1428571429	0.1428571429	0.1111111111		1.083333333	#DIV/0!
MIR4651	0.1428571429	0.1428571429	0.1111111111		1.083333333	#DIV/0!
MIR4652	0.04761904762	0.04761904762	0.1111111111		0.325	#DIV/0!
MIR4653	0.04761904762	0.04761904762	0.05555555556		0.7	#DIV/0!
MIR4654	0.1428571429	0.1428571429	0		inf	#DIV/0!
MIR4655	0.04761904762	0.04761904762	0.1666666667		0.2	#DIV/0!
MIR4656	0.04761904762	0.04761904762	0.1666666667		0.2	#DIV/0!
MIR4657	0.04761904762	0.04761904762	0.2222222222		0.1375	#DIV/0!
MIR4658	0.04761904762	0.04761904762	0.05555555556		0.7	#DIV/0!
IIR4659	0.09523809524	0.09523809524	0.1111111111		0.6842105263	#DIV/0!
IIR4659	0.09523809524	0.09523809524	0.1111111111		0.6842105263	#DIV/0!
MIR466	0.04761904762	0.04761904762	0		inf	#DIV/0!

MIR466(	0.09523809524	0.09523809524	0.1111111111		0.6842105263	#DIV/0!
MIR4661	0.1904761905	0.1904761905	0.1111111111		1.529411765	#DIV/0!
IIR4662.	0.1428571429	0.1428571429	0.1111111111		1.083333333	#DIV/0!
IIR4662	0.1428571429	0.1428571429	0.1111111111		1.083333333	#DIV/0!
MIR4663	0.1428571429	0.1428571429	0.1111111111		1.083333333	#DIV/0!
MIR4664	0.04761904762	0.04761904762	0.1666666667		0.2	#DIV/0!
MIR4665	0.04761904762	0.04761904762	0.05555555556		0.7	#DIV/0!
IIR4666.	0.1428571429	0.1428571429	0		inf	#DIV/0!
MIR4667	0.09523809524	0.09523809524	0.05555555556		1.473684211	#DIV/0!
MIR4668	0.04761904762	0.04761904762	0.05555555556		0.7	#DIV/0!
MIR4669	0.04761904762	0.04761904762	0		inf	#DIV/0!
MIR467(	0.04761904762	0.04761904762	0.05555555556		0.7	#DIV/0!
MIR4671	0.09523809524	0.09523809524	0		inf	#DIV/0!
MIR4672	0.09523809524	0.09523809524	0.05555555556		1.473684211	#DIV/0!
MIR4673	0.1428571429	0.1428571429	0.05555555556		2.333333333	#DIV/0!
MIR4674	0.1428571429	0.1428571429	0.05555555556		2.333333333	#DIV/0!
MIR4675	0.09523809524	0.09523809524	0.1111111111		0.6842105263	#DIV/0!
MIR4676	0.1904761905	0.1904761905	0.05555555556		3.294117647	#DIV/0!
MIR4677	0.1428571429	0.1428571429	0.05555555556		2.333333333	#DIV/0!
MIR4678	0.1428571429	0.1428571429	0.1111111111		1.083333333	#DIV/0!
IIR4679-	0.1428571429	0.1428571429	0.1666666667		0.6666666667	#DIV/0!
IIR4679-	0.1428571429	0.1428571429	0.1666666667		0.6666666667	#DIV/0!
MIR468(	0.1428571429	0.1428571429	0.05555555556		2.333333333	#DIV/0!
MIR4681	0.2380952381	0.2380952381	0		inf	#DIV/0!
MIR4682	0.2380952381	0.2380952381	0		inf	#DIV/0!
MIR4683	0.09523809524	0.09523809524	0.1666666667		0.4210526316	#DIV/0!
MIR4684	0.04761904762	0.04761904762	0.1111111111		0.325	#DIV/0!
MIR4685	0.1428571429	0.1428571429	0		inf	#DIV/0!
MIR4686	0.04761904762	0.04761904762	0		inf	#DIV/0!
MIR4687	0.04761904762	0.04761904762	0		inf	#DIV/0!
MIR4688	0	0	0.05555555556		0	#DIV/0!
MIR4689	0	0	0.05555555556		0	#DIV/0!
MIR4695	0.04761904762	0.04761904762	0.1111111111		0.325	#DIV/0!
MIR4698	0.04761904762	0.04761904762	0.05555555556		0.7	#DIV/0!
MIR4699	0.04761904762	0.04761904762	0.05555555556		0.7	#DIV/0!
MIR470(	0	0	0.1111111111		0	#DIV/0!
MIR4701	0.04761904762	0.04761904762	0.05555555556		0.7	#DIV/0!
MIR4707	0	0	0.05555555556		0	#DIV/0!
MIR4711	0.04761904762	0.04761904762	0.05555555556		0.7	#DIV/0!
MIR4712	0.1428571429	0.1428571429	0.05555555556		2.333333333	#DIV/0!
MIR4713	0.04761904762	0.04761904762	0.05555555556		0.7	#DIV/0!
IR4713H	0.04761904762	0.04761904762	0.05555555556		0.7	#DIV/0!
MIR4714	0.04761904762	0.04761904762	0.05555555556		0.7	#DIV/0!
MIR4716	0.04761904762	0.04761904762	0.05555555556		0.7	#DIV/0!
MIR4717	0.09523809524	0.09523809524	0.2222222222		0.2894736842	#DIV/0!
MIR4718	0.04761904762	0.04761904762	0		inf	#DIV/0!
MIR4719	0.04761904762	0.04761904762	0		inf	#DIV/0!
MIR472(	0.04761904762	0.04761904762	0		inf	#DIV/0!
MIR4721	0.04761904762	0.04761904762	0		inf	#DIV/0!
MIR4722	0.1904761905	0.1904761905	0		inf	#DIV/0!
MIR4723	0.04761904762	0.04761904762	0.05555555556		0.7	#DIV/0!

MIR4724	0	0	0			#DIV/0!
MIR4725	0	0	0			#DIV/0!
MIR4726	0.04761904762	0.04761904762	0		inf	#DIV/0!
MIR4727	0.09523809524	0.09523809524	0		inf	#DIV/0!
MIR4728	0.09523809524	0.09523809524	0		inf	#DIV/0!
MIR4729	0.1428571429	0.1428571429	0		inf	#DIV/0!
MIR4730	0.04761904762	0.04761904762	0		inf	#DIV/0!
MIR4731	0.04761904762	0.04761904762	0.05555555556		0.7	#DIV/0!
MIR4732	0.04761904762	0.04761904762	0.05555555556		0.7	#DIV/0!
MIR4733	0	0	0			#DIV/0!
MIR4734	0.04761904762	0.04761904762	0		inf	#DIV/0!
MIR4735	0.1428571429	0.1428571429	0		inf	#DIV/0!
MIR4736	0.09523809524	0.09523809524	0		inf	#DIV/0!
MIR4737	0.1428571429	0.1428571429	0		inf	#DIV/0!
MIR4738	0.04761904762	0.04761904762	0		inf	#DIV/0!
MIR4739	0.04761904762	0.04761904762	0		inf	#DIV/0!
MIR4740	0.04761904762	0.04761904762	0		inf	#DIV/0!
MIR4741	0.04761904762	0.04761904762	0.05555555556		0.7	#DIV/0!
MIR4742	0.1428571429	0.1428571429	0.05555555556		2.333333333	#DIV/0!
MIR4743	0.04761904762	0.04761904762	0		inf	#DIV/0!
MIR4744	0.04761904762	0.04761904762	0		inf	#DIV/0!
MIR4745	0.09523809524	0.09523809524	0.1111111111		0.6842105263	#DIV/0!
MIR4747	0.09523809524	0.09523809524	0.05555555556		1.473684211	#DIV/0!
MIR4748	0.1428571429	0.1428571429	0.1111111111		1.083333333	#DIV/0!
MIR4749	0.09523809524	0.09523809524	0.1111111111		0.6842105263	#DIV/0!
MIR4750	0.09523809524	0.09523809524	0.1111111111		0.6842105263	#DIV/0!
MIR4752	0.09523809524	0.09523809524	0.05555555556		1.473684211	#DIV/0!
MIR4753	0.1428571429	0.1428571429	0.05555555556		2.333333333	#DIV/0!
MIR4754	0.1428571429	0.1428571429	0.05555555556		2.333333333	#DIV/0!
MIR4755	0.09523809524	0.09523809524	0.1111111111		0.6842105263	#DIV/0!
MIR4756	0.09523809524	0.09523809524	0.1111111111		0.6842105263	#DIV/0!
MIR4757	0	0	0.05555555556		0	#DIV/0!
MIR4758	0.1428571429	0.1428571429	0.2222222222		0.4583333333	#DIV/0!
MIR4759	0.04761904762	0.04761904762	0.1111111111		0.325	#DIV/0!
MIR4760	0.04761904762	0.04761904762	0.05555555556		0.7	#DIV/0!
MIR4761	0.04761904762	0.04761904762	0.05555555556		0.7	#DIV/0!
MIR4762	0	0	0.1111111111		0	#DIV/0!
MIR4763	0	0	0.1111111111		0	#DIV/0!
MIR4764	0.04761904762	0.04761904762	0.1666666667		0.2	#DIV/0!
MIR4765	0.1428571429	0.1428571429	0		inf	#DIV/0!
MIR4766	0	0	0.1111111111		0	#DIV/0!
IIR4771-	0	0	0.05555555556		0	#DIV/0!
IIR4771-	0	0	0.05555555556		0	#DIV/0!
MIR4772	0	0	0.05555555556		0	#DIV/0!
IIR4773-	0	0	0.05555555556		0	#DIV/0!
IIR4773-	0	0	0.05555555556		0	#DIV/0!
MIR4774	0.04761904762	0.04761904762	0.05555555556		0.7	#DIV/0!
MIR4775	0	0	0.05555555556		0	#DIV/0!
IIR4776-	0	0	0.05555555556		0	#DIV/0!
IIR4776-	0	0	0.05555555556		0	#DIV/0!
MIR4777	0	0	0.05555555556		0	#DIV/0!

MIR4778	0	0	0.05555555556		0	#DIV/0!
MIR4779	0	0	0.05555555556		0	#DIV/0!
MIR4780	0	0	0.05555555556		0	#DIV/0!
MIR4781	0.04761904762	0.04761904762	0.05555555556		0.7	#DIV/0!
MIR4782	0	0	0.05555555556		0	#DIV/0!
MIR4783	0	0	0.05555555556		0	#DIV/0!
MIR4784	0	0	0.05555555556		0	#DIV/0!
MIR4785	0	0	0.05555555556		0	#DIV/0!
MIR4786	0	0	0.05555555556		0	#DIV/0!
MIR4787	0.09523809524	0.09523809524	0		inf	#DIV/0!
MIR4788	0.1428571429	0.1428571429	0.05555555556		2.333333333	#DIV/0!
MIR4789	0.1904761905	0.1904761905	0.05555555556		3.294117647	#DIV/0!
MIR4790	0.04761904762	0.04761904762	0		inf	#DIV/0!
MIR4791	0.04761904762	0.04761904762	0		inf	#DIV/0!
MIR4793	0.09523809524	0.09523809524	0		inf	#DIV/0!
MIR4794	0.04761904762	0.04761904762	0.05555555556		0.7	#DIV/0!
MIR4795	0.04761904762	0.04761904762	0		inf	#DIV/0!
MIR4796	0.09523809524	0.09523809524	0.05555555556		1.473684211	#DIV/0!
MIR4797	0.1428571429	0.1428571429	0.05555555556		2.333333333	#DIV/0!
MIR4799	0	0	0.05555555556		0	#DIV/0!
MIR4800	0.04761904762	0.04761904762	0		inf	#DIV/0!
MIR4802	0.1904761905	0.1904761905	0.05555555556		3.294117647	#DIV/0!
MIR4803	0	0	0.05555555556		0	#DIV/0!
MIR4804	0	0	0.05555555556		0	#DIV/0!
MIR483	0.04761904762	0.04761904762	0		inf	#DIV/0!
MIR484	0.04761904762	0.04761904762	0		inf	#DIV/0!
MIR486-	0.09523809524	0.09523809524	0.1666666667		0.4210526316	#DIV/0!
MIR486-	0.09523809524	0.09523809524	0.1666666667		0.4210526316	#DIV/0!
MIR488	0.1428571429	0.1428571429	0		inf	#DIV/0!
MIR489	0.04761904762	0.04761904762	0.1111111111		0.325	#DIV/0!
MIR490	0.04761904762	0.04761904762	0.05555555556		0.7	#DIV/0!
MIR491	0.04761904762	0.04761904762	0.05555555556		0.7	#DIV/0!
MIR492	0.04761904762	0.04761904762	0.05555555556		0.7	#DIV/0!
MIR497	0	0	0.05555555556		0	#DIV/0!
MIR497H	0	0	0.05555555556		0	#DIV/0!
MIR498	0.09523809524	0.09523809524	0.05555555556		1.473684211	#DIV/0!
MIR4999	0.1428571429	0.1428571429	0.05555555556		2.333333333	#DIV/0!
MIR499A	0.09523809524	0.09523809524	0.1111111111		0.6842105263	#DIV/0!
MIR499B	0.09523809524	0.09523809524	0.1111111111		0.6842105263	#DIV/0!
MIR5000	0	0	0.05555555556		0	#DIV/0!
MIR5001	0	0	0.05555555556		0	#DIV/0!
MIR5002	0.04761904762	0.04761904762	0.05555555556		0.7	#DIV/0!
MIR5003	0	0	0.05555555556		0	#DIV/0!
MIR5004	0.04761904762	0.04761904762	0		inf	#DIV/0!
MIR5008	0.1428571429	0.1428571429	0		inf	#DIV/0!
MIR5009	0.04761904762	0.04761904762	0.05555555556		0.7	#DIV/0!
MIR5047	0.04761904762	0.04761904762	0		inf	#DIV/0!
MIR5087	0.1428571429	0.1428571429	0.05555555556		2.333333333	#DIV/0!
MIR5088	0.09523809524	0.09523809524	0.1111111111		0.6842105263	#DIV/0!
MIR5089	0.1428571429	0.1428571429	0		inf	#DIV/0!
MIR5090	0.04761904762	0.04761904762	0.05555555556		0.7	#DIV/0!

MIR5091	0	0	0			#DIV/0!
MIR5092	0.04761904762	0.04761904762	0.05555555556		0.7	#DIV/0!
MIR5093	0.04761904762	0.04761904762	0		inf	#DIV/0!
MIR5094	0.04761904762	0.04761904762	0.05555555556		0.7	#DIV/0!
MIR510(	0.1428571429	0.1428571429	0.05555555556		2.333333333	#DIV/0!
MIR511	0.09523809524	0.09523809524	0.1111111111		0.6842105263	#DIV/0!
MIR512-	0.09523809524	0.09523809524	0.05555555556		1.473684211	#DIV/0!
MIR512-	0.09523809524	0.09523809524	0.05555555556		1.473684211	#DIV/0!
MIR515-	0.09523809524	0.09523809524	0.05555555556		1.473684211	#DIV/0!
MIR515-	0.09523809524	0.09523809524	0.05555555556		1.473684211	#DIV/0!
MIR516A	0.09523809524	0.09523809524	0.05555555556		1.473684211	#DIV/0!
MIR516A	0.09523809524	0.09523809524	0.05555555556		1.473684211	#DIV/0!
MIR516B	0.09523809524	0.09523809524	0.05555555556		1.473684211	#DIV/0!
MIR516B	0.09523809524	0.09523809524	0.05555555556		1.473684211	#DIV/0!
MIR517A	0.09523809524	0.09523809524	0.05555555556		1.473684211	#DIV/0!
MIR517B	0.09523809524	0.09523809524	0.05555555556		1.473684211	#DIV/0!
MIR517C	0.09523809524	0.09523809524	0.05555555556		1.473684211	#DIV/0!
MIR518(	0.1904761905	0.1904761905	0.1111111111		1.529411765	#DIV/0!
MIR5187	0.1428571429	0.1428571429	0		inf	#DIV/0!
MIR5188	0.04761904762	0.04761904762	0.05555555556		0.7	#DIV/0!
MIR5189	0.1428571429	0.1428571429	0		inf	#DIV/0!
MIR518A	0.09523809524	0.09523809524	0.05555555556		1.473684211	#DIV/0!
MIR518A	0.09523809524	0.09523809524	0.05555555556		1.473684211	#DIV/0!
MIR518B	0.09523809524	0.09523809524	0.05555555556		1.473684211	#DIV/0!
MIR518C	0.09523809524	0.09523809524	0.05555555556		1.473684211	#DIV/0!
MIR518D	0.09523809524	0.09523809524	0.05555555556		1.473684211	#DIV/0!
MIR518E	0.09523809524	0.09523809524	0.05555555556		1.473684211	#DIV/0!
MIR518F	0.09523809524	0.09523809524	0.05555555556		1.473684211	#DIV/0!
MIR519(	0.09523809524	0.09523809524	0.05555555556		1.473684211	#DIV/0!
MIR5191	0.1428571429	0.1428571429	0		inf	#DIV/0!
MIR5192	0	0	0.05555555556		0	#DIV/0!
MIR5193	0.09523809524	0.09523809524	0		inf	#DIV/0!
MIR5194	0.04761904762	0.04761904762	0.1111111111		0.325	#DIV/0!
MIR5195	0.04761904762	0.04761904762	0		inf	#DIV/0!
MIR5196	0.04761904762	0.04761904762	0.05555555556		0.7	#DIV/0!
MIR519A	0.09523809524	0.09523809524	0.05555555556		1.473684211	#DIV/0!
MIR519A	0.09523809524	0.09523809524	0.05555555556		1.473684211	#DIV/0!
MIR519B	0.09523809524	0.09523809524	0.05555555556		1.473684211	#DIV/0!
MIR519C	0.09523809524	0.09523809524	0.05555555556		1.473684211	#DIV/0!
MIR519D	0.09523809524	0.09523809524	0.05555555556		1.473684211	#DIV/0!
MIR519E	0.09523809524	0.09523809524	0.05555555556		1.473684211	#DIV/0!
MIR520A	0.09523809524	0.09523809524	0.05555555556		1.473684211	#DIV/0!
MIR520B	0.09523809524	0.09523809524	0.05555555556		1.473684211	#DIV/0!
MIR520C	0.09523809524	0.09523809524	0.05555555556		1.473684211	#DIV/0!
MIR520D	0.09523809524	0.09523809524	0.05555555556		1.473684211	#DIV/0!
MIR520E	0.09523809524	0.09523809524	0.05555555556		1.473684211	#DIV/0!
MIR520F	0.09523809524	0.09523809524	0.05555555556		1.473684211	#DIV/0!
MIR520G	0.09523809524	0.09523809524	0.05555555556		1.473684211	#DIV/0!
MIR520H	0.09523809524	0.09523809524	0.05555555556		1.473684211	#DIV/0!
MIR521-	0.09523809524	0.09523809524	0.05555555556		1.473684211	#DIV/0!
MIR521-	0.09523809524	0.09523809524	0.05555555556		1.473684211	#DIV/0!

MIR522	0.09523809524	0.09523809524	0.05555555556		1.473684211	#DIV/0!
MIR523	0.09523809524	0.09523809524	0.05555555556		1.473684211	#DIV/0!
MIR524	0.09523809524	0.09523809524	0.05555555556		1.473684211	#DIV/0!
MIR525	0.09523809524	0.09523809524	0.05555555556		1.473684211	#DIV/0!
IIR526A	0.09523809524	0.09523809524	0.05555555556		1.473684211	#DIV/0!
IIR526A	0.09523809524	0.09523809524	0.05555555556		1.473684211	#DIV/0!
MIR526I	0.09523809524	0.09523809524	0.05555555556		1.473684211	#DIV/0!
MIR527	0.09523809524	0.09523809524	0.05555555556		1.473684211	#DIV/0!
MIR544I	0.04761904762	0.04761904762	0.05555555556		0.7	#DIV/0!
IIR548A	0.1904761905	0.1904761905	0.1111111111		1.529411765	#DIV/0!
IR548A	0.1428571429	0.1428571429	0.1111111111		1.083333333	#DIV/0!
IR548A	0	0	0			#DIV/0!
IIR548A	0.09523809524	0.09523809524	0.05555555556		1.473684211	#DIV/0!
IIR548A	0.04761904762	0.04761904762	0.05555555556		0.7	#DIV/0!
IR548AI	0	0	0.05555555556		0	#DIV/0!
IR548AI	0.04761904762	0.04761904762	0		inf	#DIV/0!
IR548AC	0.04761904762	0.04761904762	0.05555555556		0.7	#DIV/0!
IR548AC	0.1428571429	0.1428571429	0.1111111111		1.083333333	#DIV/0!
IIR548A	0.04761904762	0.04761904762	0.05555555556		0.7	#DIV/0!
IIR548A	0.09523809524	0.09523809524	0.1111111111		0.6842105263	#DIV/0!
IIR548A	0.09523809524	0.09523809524	0.1666666667		0.4210526316	#DIV/0!
IIR548A	0.09523809524	0.09523809524	0.05555555556		1.473684211	#DIV/0!
IIR548A	0.09523809524	0.09523809524	0.1666666667		0.4210526316	#DIV/0!
IIR548A	0.04761904762	0.04761904762	0.1111111111		0.325	#DIV/0!
IIR548A	0.04761904762	0.04761904762	0		inf	#DIV/0!
IIR548A	0.09523809524	0.09523809524	0		inf	#DIV/0!
IIR548A	0.09523809524	0.09523809524	0.1666666667		0.4210526316	#DIV/0!
IIR548B	0	0	0.05555555556		0	#DIV/0!
IIR548B	0.04761904762	0.04761904762	0		inf	#DIV/0!
MIR548C	0.1428571429	0.1428571429	0.05555555556		2.333333333	#DIV/0!
IIR548D	0.1428571429	0.1428571429	0.1111111111		1.083333333	#DIV/0!
IIR548D	0	0	0			#DIV/0!
MIR548I	0.1428571429	0.1428571429	0.05555555556		2.333333333	#DIV/0!
IIR548F	0.09523809524	0.09523809524	0.05555555556		1.473684211	#DIV/0!
IIR548F	0	0	0.05555555556		0	#DIV/0!
IIR548F	0.1904761905	0.1904761905	0.05555555556		3.294117647	#DIV/0!
IIR548F	0.09523809524	0.09523809524	0.05555555556		1.473684211	#DIV/0!
MIR548C	0	0	0.05555555556		0	#DIV/0!
IIR548H	0.09523809524	0.09523809524	0		inf	#DIV/0!
IIR548H	0.04761904762	0.04761904762	0		inf	#DIV/0!
IIR548H	0.04761904762	0.04761904762	0.1111111111		0.325	#DIV/0!
IIR548H	0.04761904762	0.04761904762	0.1111111111		0.325	#DIV/0!
MIR548I	0.04761904762	0.04761904762	0.05555555556		0.7	#DIV/0!
MIR548I	0.09523809524	0.09523809524	0.1666666667		0.4210526316	#DIV/0!
MIR548J	0.04761904762	0.04761904762	0.05555555556		0.7	#DIV/0!
MIR548N	0.09523809524	0.09523809524	0.2222222222		0.2894736842	#DIV/0!
MIR548C	0.04761904762	0.04761904762	0.05555555556		0.7	#DIV/0!
IIR548O	0.1428571429	0.1428571429	0.1666666667		0.6666666667	#DIV/0!
MIR548I	0.04761904762	0.04761904762	0		inf	#DIV/0!
MIR548C	0.09523809524	0.09523809524	0.1111111111		0.6842105263	#DIV/0!
MIR548S	0	0	0.05555555556		0	#DIV/0!

MIR548T	0	0	0.05555555556		0	#DIV/0!
MIR548U	0.04761904762	0.04761904762	0.05555555556		0.7	#DIV/0!
MIR548V	0.09523809524	0.09523809524	0.1111111111		0.6842105263	#DIV/0!
MIR548W	0.04761904762	0.04761904762	0		inf	#DIV/0!
MIR548X	0.04761904762	0.04761904762	0.1111111111		0.325	#DIV/0!
IR548XI	0.04761904762	0.04761904762	0.1111111111		0.325	#DIV/0!
MIR548Y	0.04761904762	0.04761904762	0		inf	#DIV/0!
MIR548Z	0.1428571429	0.1428571429	0.05555555556		2.333333333	#DIV/0!
MIR549A	0.04761904762	0.04761904762	0.05555555556		0.7	#DIV/0!
MIR550A	0.04761904762	0.04761904762	0.1666666667		0.2	#DIV/0!
MIR550A	0.04761904762	0.04761904762	0.1666666667		0.2	#DIV/0!
MIR550A	0.04761904762	0.04761904762	0.1666666667		0.2	#DIV/0!
MIR550B	0.04761904762	0.04761904762	0.1666666667		0.2	#DIV/0!
MIR550B	0.04761904762	0.04761904762	0.1666666667		0.2	#DIV/0!
MIR551A	0	0	0.05555555556		0	#DIV/0!
MIR551B	0.1428571429	0.1428571429	0.05555555556		2.333333333	#DIV/0!
MIR552	0.04761904762	0.04761904762	0		inf	#DIV/0!
MIR553	0.04761904762	0.04761904762	0.05555555556		0.7	#DIV/0!
MIR554	0.1428571429	0.1428571429	0.05555555556		2.333333333	#DIV/0!
MIR555	0.1904761905	0.1904761905	0.1111111111		1.529411765	#DIV/0!
MIR556	0.1428571429	0.1428571429	0		inf	#DIV/0!
MIR557	0.1428571429	0.1428571429	0		inf	#DIV/0!
MIR5572	0.04761904762	0.04761904762	0.05555555556		0.7	#DIV/0!
MIR558	0.1428571429	0.1428571429	0		inf	#DIV/0!
MIR5580	0.04761904762	0.04761904762	0		inf	#DIV/0!
MIR5581	0.04761904762	0.04761904762	0.05555555556		0.7	#DIV/0!
MIR5582	0	0	0.05555555556		0	#DIV/0!
MIR5583-	0.09523809524	0.09523809524	0		inf	#DIV/0!
MIR5583-	0.09523809524	0.09523809524	0		inf	#DIV/0!
MIR5584	0.04761904762	0.04761904762	0.05555555556		0.7	#DIV/0!
MIR5585	0.04761904762	0.04761904762	0.1111111111		0.325	#DIV/0!
MIR5587	0.04761904762	0.04761904762	0.2222222222		0.1375	#DIV/0!
MIR5588	0.1428571429	0.1428571429	0.05555555556		2.333333333	#DIV/0!
MIR5589	0.1428571429	0.1428571429	0.1111111111		1.083333333	#DIV/0!
MIR559	0	0	0.05555555556		0	#DIV/0!
MIR5590	0.04761904762	0.04761904762	0.05555555556		0.7	#DIV/0!
MIR5591	0.1904761905	0.1904761905	0.05555555556		3.294117647	#DIV/0!
MIR561	0	0	0.05555555556		0	#DIV/0!
MIR562	0	0	0.05555555556		0	#DIV/0!
MIR563	0.04761904762	0.04761904762	0		inf	#DIV/0!
MIR567	0.09523809524	0.09523809524	0.05555555556		1.473684211	#DIV/0!
MIR568	0.09523809524	0.09523809524	0.05555555556		1.473684211	#DIV/0!
MIR5680	0.1904761905	0.1904761905	0.1111111111		1.529411765	#DIV/0!
MIR5681	0.1904761905	0.1904761905	0.1111111111		1.529411765	#DIV/0!
MIR5681	0.1904761905	0.1904761905	0.1111111111		1.529411765	#DIV/0!
MIR5682	0.1428571429	0.1428571429	0.05555555556		2.333333333	#DIV/0!
MIR5684	0.1428571429	0.1428571429	0.1111111111		1.083333333	#DIV/0!
MIR5685	0.04761904762	0.04761904762	0		inf	#DIV/0!
MIR5687	0.04761904762	0.04761904762	0.05555555556		0.7	#DIV/0!
MIR5688	0.04761904762	0.04761904762	0		inf	#DIV/0!
MIR569	0.1904761905	0.1904761905	0.05555555556		3.294117647	#DIV/0!

MIR569(	0.04761904762	0.04761904762	0.05555555556		0.7	#DIV/0!
IR5692A	0.1428571429	0.1428571429	0.1111111111		1.083333333	#DIV/0!
IR5692A	0.1428571429	0.1428571429	0.1111111111		1.083333333	#DIV/0!
IIR5692I	0.04761904762	0.04761904762	0.1666666667		0.2	#DIV/0!
IR5692C	0.04761904762	0.04761904762	0		inf	#DIV/0!
IR5692C	0.04761904762	0.04761904762	0.05555555556		0.7	#DIV/0!
MIR5695	0.1428571429	0.1428571429	0.1111111111		1.083333333	#DIV/0!
MIR5696	0	0	0.05555555556		0	#DIV/0!
MIR5697	0	0	0.1111111111		0	#DIV/0!
MIR5698	0.1904761905	0.1904761905	0.05555555556		3.294117647	#DIV/0!
MIR5699	0.04761904762	0.04761904762	0.1111111111		0.325	#DIV/0!
MIR570	0.1428571429	0.1428571429	0.05555555556		2.333333333	#DIV/0!
MIR570(	0.04761904762	0.04761904762	0.05555555556		0.7	#DIV/0!
IIR5701-	0.2857142857	0.2857142857	0.1111111111		2.6	#DIV/0!
IIR5701-	0.2857142857	0.2857142857	0.1111111111		2.6	#DIV/0!
IIR5701-	0.2857142857	0.2857142857	0.1111111111		2.6	#DIV/0!
MIR5702	0	0	0.05555555556		0	#DIV/0!
MIR5703	0	0	0.05555555556		0	#DIV/0!
MIR5704	0.09523809524	0.09523809524	0.05555555556		1.473684211	#DIV/0!
MIR5705	0	0	0.05555555556		0	#DIV/0!
MIR570(	0.04761904762	0.04761904762	0		inf	#DIV/0!
MIR5707	0.1428571429	0.1428571429	0.05555555556		2.333333333	#DIV/0!
MIR5708	0.1428571429	0.1428571429	0.1111111111		1.083333333	#DIV/0!
IR570H	0.1428571429	0.1428571429	0.05555555556		2.333333333	#DIV/0!
MIR571	0.04761904762	0.04761904762	0		inf	#DIV/0!
MIR572	0	0	0			#DIV/0!
MIR5739	0.04761904762	0.04761904762	0.1111111111		0.325	#DIV/0!
MIR574	0.1428571429	0.1428571429	0.05555555556		2.333333333	#DIV/0!
MIR575	0	0	0.05555555556		0	#DIV/0!
MIR576	0	0	0.05555555556		0	#DIV/0!
MIR577	0	0	0.05555555556		0	#DIV/0!
MIR578	0	0	0.1111111111		0	#DIV/0!
MIR5787	0.09523809524	0.09523809524	0		inf	#DIV/0!
MIR579	0.04761904762	0.04761904762	0.05555555556		0.7	#DIV/0!
MIR580	0.04761904762	0.04761904762	0.05555555556		0.7	#DIV/0!
MIR581	0.04761904762	0.04761904762	0.05555555556		0.7	#DIV/0!
MIR582	0.04761904762	0.04761904762	0.05555555556		0.7	#DIV/0!
MIR583	0.04761904762	0.04761904762	0		inf	#DIV/0!
MIR585	0	0	0.05555555556		0	#DIV/0!
MIR586	0.04761904762	0.04761904762	0.1111111111		0.325	#DIV/0!
MIR587	0	0	0			#DIV/0!
MIR589	0.04761904762	0.04761904762	0.1666666667		0.2	#DIV/0!
MIR590	0.1428571429	0.1428571429	0.1111111111		1.083333333	#DIV/0!
MIR591	0.04761904762	0.04761904762	0.1111111111		0.325	#DIV/0!
MIR592	0.04761904762	0.04761904762	0.05555555556		0.7	#DIV/0!
MIR593	0.04761904762	0.04761904762	0.05555555556		0.7	#DIV/0!
MIR595	0.1428571429	0.1428571429	0.05555555556		2.333333333	#DIV/0!
MIR596	0.1428571429	0.1428571429	0.1111111111		1.083333333	#DIV/0!
MIR597	0.09523809524	0.09523809524	0.1111111111		0.6842105263	#DIV/0!
MIR598	0.09523809524	0.09523809524	0.1111111111		0.6842105263	#DIV/0!
MIR599	0.1904761905	0.1904761905	0.1111111111		1.529411765	#DIV/0!

**MIR600**	0.09523809524	0.09523809524	0.05555555556		1.473684211	#DIV/0!
**IR600H**	0.09523809524	0.09523809524	0.05555555556		1.473684211	#DIV/0!
**MIR601**	0.09523809524	0.09523809524	0.05555555556		1.473684211	#DIV/0!
**MIR602**	0.09523809524	0.09523809524	0		inf	#DIV/0!
**MIR603**	0.09523809524	0.09523809524	0.1111111111		0.6842105263	#DIV/0!
**MIR604**	0.09523809524	0.09523809524	0.2222222222		0.2894736842	#DIV/0!
**MIR605**	0.04761904762	0.04761904762	0.05555555556		0.7	#DIV/0!
**MIR606**	0.09523809524	0.09523809524	0.05555555556		1.473684211	#DIV/0!
**MIR6068**	0.04761904762	0.04761904762	0.05555555556		0.7	#DIV/0!
**MIR6069**	0	0	0.1111111111		0	#DIV/0!
**MIR607**	0.1428571429	0.1428571429	0		inf	#DIV/0!
**MIR6070**	0.04761904762	0.04761904762	0.1666666667		0.2	#DIV/0!
**MIR6071**	0	0	0.05555555556		0	#DIV/0!
**MIR6072**	0	0	0.1111111111		0	#DIV/0!
**MIR6074**	0.09523809524	0.09523809524	0.05555555556		1.473684211	#DIV/0!
**MIR6075**	0	0	0.1111111111		0	#DIV/0!
**MIR6076**	0.04761904762	0.04761904762	0		inf	#DIV/0!
**MIR6077**	0.1428571429	0.1428571429	0.05555555556		2.333333333	#DIV/0!
**MIR6078**	0.04761904762	0.04761904762	0.1666666667		0.2	#DIV/0!
**MIR6079**	0.04761904762	0.04761904762	0.05555555556		0.7	#DIV/0!
**MIR608**	0.1428571429	0.1428571429	0		inf	#DIV/0!
**MIR6080**	0.04761904762	0.04761904762	0		inf	#DIV/0!
**MIR6081**	0.04761904762	0.04761904762	0.05555555556		0.7	#DIV/0!
**MIR6082**	0	0	0.05555555556		0	#DIV/0!
**MIR6083**	0.04761904762	0.04761904762	0.05555555556		0.7	#DIV/0!
**MIR6084**	0.04761904762	0.04761904762	0.1111111111		0.325	#DIV/0!
**MIR6085**	0.04761904762	0.04761904762	0.05555555556		0.7	#DIV/0!
**MIR6088**	0.09523809524	0.09523809524	0.1111111111		0.6842105263	#DIV/0!
**MIR609**	0.1428571429	0.1428571429	0.05555555556		2.333333333	#DIV/0!
**MIR6125**	0.09523809524	0.09523809524	0		inf	#DIV/0!
**MIR6126**	0.04761904762	0.04761904762	0.1111111111		0.325	#DIV/0!
**MIR6127**	0.04761904762	0.04761904762	0.1111111111		0.325	#DIV/0!
**MIR6129**	0.1428571429	0.1428571429	0		inf	#DIV/0!
**MIR613**	0.04761904762	0.04761904762	0.05555555556		0.7	#DIV/0!
**MIR6130**	0.04761904762	0.04761904762	0.1111111111		0.325	#DIV/0!
**MIR6131**	0.04761904762	0.04761904762	0.05555555556		0.7	#DIV/0!
**MIR6132**	0.09523809524	0.09523809524	0.1666666667		0.4210526316	#DIV/0!
**MIR6133**	0.04761904762	0.04761904762	0.05555555556		0.7	#DIV/0!
**MIR614**	0.04761904762	0.04761904762	0.05555555556		0.7	#DIV/0!
**MIR615**	0.04761904762	0.04761904762	0.05555555556		0.7	#DIV/0!
**MIR6165**	0.09523809524	0.09523809524	0		inf	#DIV/0!
**MIR617**	0.04761904762	0.04761904762	0.05555555556		0.7	#DIV/0!
**MIR618**	0.04761904762	0.04761904762	0.05555555556		0.7	#DIV/0!
**MIR619**	0	0	0.05555555556		0	#DIV/0!
**MIR620**	0	0	0.05555555556		0	#DIV/0!
**MIR624**	0	0	0			#DIV/0!
**MIR628**	0.04761904762	0.04761904762	0.05555555556		0.7	#DIV/0!
**MIR629**	0.04761904762	0.04761904762	0.05555555556		0.7	#DIV/0!
**MIR630**	0.04761904762	0.04761904762	0.05555555556		0.7	#DIV/0!
**MIR631**	0.04761904762	0.04761904762	0.05555555556		0.7	#DIV/0!
**MIR632**	0	0	0			#DIV/0!

MIR633	0.04761904762	0.04761904762	0		inf	#DIV/0!
MIR634	0	0	0			#DIV/0!
MIR635	0	0	0			#DIV/0!
MIR636	0.04761904762	0.04761904762	0		inf	#DIV/0!
MIR637	0.09523809524	0.09523809524	0.05555555556		1.473684211	#DIV/0!
MIR638	0.1428571429	0.1428571429	0.1111111111		1.083333333	#DIV/0!
MIR639	0.1428571429	0.1428571429	0.1111111111		1.083333333	#DIV/0!
MIR640	0.1428571429	0.1428571429	0.1111111111		1.083333333	#DIV/0!
MIR641	0.04761904762	0.04761904762	0.05555555556		0.7	#DIV/0!
MIR642A	0.09523809524	0.09523809524	0.1111111111		0.6842105263	#DIV/0!
MIR642B	0.09523809524	0.09523809524	0.1111111111		0.6842105263	#DIV/0!
MIR643	0.09523809524	0.09523809524	0.05555555556		1.473684211	#DIV/0!
MIR644A	0.09523809524	0.09523809524	0.1111111111		0.6842105263	#DIV/0!
MIR645	0.09523809524	0.09523809524	0.1666666667		0.4210526316	#DIV/0!
MIR646	0.1428571429	0.1428571429	0.1111111111		1.083333333	#DIV/0!
IR646H	0.1428571429	0.1428571429	0.1666666667		0.6666666667	#DIV/0!
MIR647	0.1428571429	0.1428571429	0.2222222222		0.4583333333	#DIV/0!
MIR648	0.04761904762	0.04761904762	0.05555555556		0.7	#DIV/0!
MIR649	0.04761904762	0.04761904762	0.05555555556		0.7	#DIV/0!
MIR650	0	0	0.05555555556		0	#DIV/0!
MIR6500	0.04761904762	0.04761904762	0.05555555556		0.7	#DIV/0!
MIR6501	0.04761904762	0.04761904762	0.1111111111		0.325	#DIV/0!
MIR6502	0.09523809524	0.09523809524	0.05555555556		1.473684211	#DIV/0!
MIR6504	0.04761904762	0.04761904762	0		inf	#DIV/0!
MIR6505	0.04761904762	0.04761904762	0.05555555556		0.7	#DIV/0!
MIR6506	0.04761904762	0.04761904762	0		inf	#DIV/0!
MIR6507	0.1428571429	0.1428571429	0		inf	#DIV/0!
MIR6508	0.04761904762	0.04761904762	0.05555555556		0.7	#DIV/0!
MIR6509	0.04761904762	0.04761904762	0.05555555556		0.7	#DIV/0!
MIR6510	0.09523809524	0.09523809524	0		inf	#DIV/0!
IR6511A	0.09523809524	0.09523809524	0		inf	#DIV/0!
IR6511A	0.09523809524	0.09523809524	0		inf	#DIV/0!
IR6511A	0.09523809524	0.09523809524	0		inf	#DIV/0!
IR6511A	0.09523809524	0.09523809524	0		inf	#DIV/0!
IR6511B	0.09523809524	0.09523809524	0.2222222222		0.2894736842	#DIV/0!
IR6511B	0.09523809524	0.09523809524	0.2222222222		0.2894736842	#DIV/0!
MIR6512	0	0	0.1111111111		0	#DIV/0!
MIR6513	0	0	0.05555555556		0	#DIV/0!
MIR6515	0.1428571429	0.1428571429	0.1111111111		1.083333333	#DIV/0!
MIR6516	0.04761904762	0.04761904762	0		inf	#DIV/0!
MIR653	0.04761904762	0.04761904762	0.1111111111		0.325	#DIV/0!
MIR657	0.04761904762	0.04761904762	0		inf	#DIV/0!
MIR658	0	0	0.1111111111		0	#DIV/0!
MIR659	0	0	0.1111111111		0	#DIV/0!
MIR661	0.04761904762	0.04761904762	0.1666666667		0.2	#DIV/0!
MIR662	0.04761904762	0.04761904762	0.2222222222		0.1375	#DIV/0!
MIR663A	0.1904761905	0.1904761905	0.05555555556		3.294117647	#DIV/0!
IR663AH	0.1904761905	0.1904761905	0.05555555556		3.294117647	#DIV/0!
MIR663B	0	0	0.05555555556		0	#DIV/0!
MIR664A	0.1428571429	0.1428571429	0.05555555556		2.333333333	#DIV/0!
MIR671	0.09523809524	0.09523809524	0.05555555556		1.473684211	#DIV/0!

**IIR6715**	0.1428571429	0.1428571429	0.05555555556		2.333333333	#DIV/0!
**IIR6715**	0.1428571429	0.1428571429	0.05555555556		2.333333333	#DIV/0!
**MIR6718**	0.09523809524	0.09523809524	0		inf	#DIV/0!
**MIR6719**	0.04761904762	0.04761904762	0.05555555556		0.7	#DIV/0!
**MIR6721**	0.04761904762	0.04761904762	0		inf	#DIV/0!
**MIR6722**	0.1428571429	0.1428571429	0.05555555556		2.333333333	#DIV/0!
**MIR6726**	0	0	0.1111111111		0	#DIV/0!
**MIR6727**	0	0	0.1111111111		0	#DIV/0!
**MIR6728**	0	0	0.05555555556		0	#DIV/0!
**MIR6729**	0.04761904762	0.04761904762	0.1111111111		0.325	#DIV/0!
**MIR6730**	0.09523809524	0.09523809524	0.1111111111		0.6842105263	#DIV/0!
**MIR6731**	0.04761904762	0.04761904762	0.1111111111		0.325	#DIV/0!
**MIR6732**	0.04761904762	0.04761904762	0.05555555556		0.7	#DIV/0!
**MIR6733**	0.04761904762	0.04761904762	0.05555555556		0.7	#DIV/0!
**MIR6734**	0.04761904762	0.04761904762	0.05555555556		0.7	#DIV/0!
**MIR6735**	0.04761904762	0.04761904762	0.05555555556		0.7	#DIV/0!
**MIR6736**	0.1428571429	0.1428571429	0.05555555556		2.333333333	#DIV/0!
**MIR6737**	0.1904761905	0.1904761905	0.05555555556		3.294117647	#DIV/0!
**MIR6738**	0.1428571429	0.1428571429	0.1111111111		1.083333333	#DIV/0!
**MIR6739**	0.1428571429	0.1428571429	0		inf	#DIV/0!
**MIR6740**	0.1428571429	0.1428571429	0		inf	#DIV/0!
**MIR6741**	0.1428571429	0.1428571429	0.05555555556		2.333333333	#DIV/0!
**MIR6742**	0.1428571429	0.1428571429	0		inf	#DIV/0!
**MIR6743**	0.04761904762	0.04761904762	0		inf	#DIV/0!
**MIR6744**	0.04761904762	0.04761904762	0		inf	#DIV/0!
**MIR6745**	0	0	0.05555555556		0	#DIV/0!
**MIR675**	0.04761904762	0.04761904762	0		inf	#DIV/0!
**MIR6757**	0.04761904762	0.04761904762	0.05555555556		0.7	#DIV/0!
**MIR6759**	0.1428571429	0.1428571429	0.05555555556		2.333333333	#DIV/0!
**MIR6760**	0	0	0.1111111111		0	#DIV/0!
**MIR6761**	0	0	0.1111111111		0	#DIV/0!
**MIR6762**	0	0	0.05555555556		0	#DIV/0!
**MIR6763**	0.09523809524	0.09523809524	0.05555555556		1.473684211	#DIV/0!
**MIR6766**	0.04761904762	0.04761904762	0.05555555556		0.7	#DIV/0!
**MIR6767**	0.09523809524	0.09523809524	0.2222222222		0.2894736842	#DIV/0!
**MIR6768**	0.09523809524	0.09523809524	0.2222222222		0.2894736842	#DIV/0!
**IIR6769**	0.04761904762	0.04761904762	0		inf	#DIV/0!
**IIR6769**	0.1428571429	0.1428571429	0		inf	#DIV/0!
**IIR6770-**	0.09523809524	0.09523809524	0		inf	#DIV/0!
**IIR6770-**	0.09523809524	0.09523809524	0		inf	#DIV/0!
**IIR6770-**	0.09523809524	0.09523809524	0		inf	#DIV/0!
**MIR6771**	0.04761904762	0.04761904762	0		inf	#DIV/0!
**MIR6772**	0.04761904762	0.04761904762	0		inf	#DIV/0!
**MIR6773**	0.1428571429	0.1428571429	0.05555555556		2.333333333	#DIV/0!
**MIR6774**	0.04761904762	0.04761904762	0		inf	#DIV/0!
**MIR6775**	0.04761904762	0.04761904762	0		inf	#DIV/0!
**MIR6776**	0	0	0.1111111111		0	#DIV/0!
**MIR6777**	0.04761904762	0.04761904762	0.1111111111		0.325	#DIV/0!
**MIR6778**	0.04761904762	0.04761904762	0.1111111111		0.325	#DIV/0!
**MIR6779**	0.09523809524	0.09523809524	0		inf	#DIV/0!
**IIR6780**	0.04761904762	0.04761904762	0		inf	#DIV/0!

MIR6780	0.04761904762	0.04761904762	0.05555555556		0.7	#DIV/0!
MIR6781	0.04761904762	0.04761904762	0		inf	#DIV/0!
MIR6782	0.09523809524	0.09523809524	0		inf	#DIV/0!
MIR6783	0.1428571429	0.1428571429	0		inf	#DIV/0!
MIR6784	0.1428571429	0.1428571429	0		inf	#DIV/0!
MIR6786	0.04761904762	0.04761904762	0		inf	#DIV/0!
MIR6787	0.04761904762	0.04761904762	0		inf	#DIV/0!
MIR6788	0.09523809524	0.09523809524	0		inf	#DIV/0!
MIR6789	0.09523809524	0.09523809524	0.1111111111		0.6842105263	#DIV/0!
MIR6790	0.1428571429	0.1428571429	0.05555555556		2.333333333	#DIV/0!
MIR6791	0.1428571429	0.1428571429	0.05555555556		2.333333333	#DIV/0!
MIR6792	0.1428571429	0.1428571429	0.05555555556		2.333333333	#DIV/0!
MIR6793	0.1428571429	0.1428571429	0.1111111111		1.083333333	#DIV/0!
MIR6794	0.1428571429	0.1428571429	0.1111111111		1.083333333	#DIV/0!
MIR6795	0.1428571429	0.1428571429	0.1111111111		1.083333333	#DIV/0!
MIR6796	0.04761904762	0.04761904762	0.05555555556		0.7	#DIV/0!
MIR6797	0.04761904762	0.04761904762	0.05555555556		0.7	#DIV/0!
MIR6798	0.09523809524	0.09523809524	0.1111111111		0.6842105263	#DIV/0!
MIR6799	0.09523809524	0.09523809524	0.1111111111		0.6842105263	#DIV/0!
MIR6800	0.09523809524	0.09523809524	0.1111111111		0.6842105263	#DIV/0!
MIR6801	0.09523809524	0.09523809524	0.05555555556		1.473684211	#DIV/0!
MIR6802	0.09523809524	0.09523809524	0.05555555556		1.473684211	#DIV/0!
MIR6803	0.09523809524	0.09523809524	0.05555555556		1.473684211	#DIV/0!
MIR6804	0.09523809524	0.09523809524	0.05555555556		1.473684211	#DIV/0!
MIR6805	0.09523809524	0.09523809524	0.05555555556		1.473684211	#DIV/0!
MIR6806	0.1428571429	0.1428571429	0.05555555556		2.333333333	#DIV/0!
MIR6807	0.1428571429	0.1428571429	0.05555555556		2.333333333	#DIV/0!
MIR6808	0	0	0.1111111111		0	#DIV/0!
MIR6809	0	0	0.1111111111		0	#DIV/0!
MIR6810	0	0	0.05555555556		0	#DIV/0!
MIR6811	0	0	0.05555555556		0	#DIV/0!
MIR6812	0.1428571429	0.1428571429	0.1666666667		0.6666666667	#DIV/0!
MIR6813	0.1428571429	0.1428571429	0.2222222222		0.4583333333	#DIV/0!
MIR6814	0.04761904762	0.04761904762	0.1111111111		0.325	#DIV/0!
MIR6815	0.04761904762	0.04761904762	0.1111111111		0.325	#DIV/0!
MIR6816	0.04761904762	0.04761904762	0.05555555556		0.7	#DIV/0!
MIR6817	0.04761904762	0.04761904762	0.05555555556		0.7	#DIV/0!
MIR6818	0.04761904762	0.04761904762	0.1111111111		0.325	#DIV/0!
MIR6819	0	0	0.1111111111		0	#DIV/0!
MIR6820	0	0	0.1111111111		0	#DIV/0!
MIR6821	0	0	0.1111111111		0	#DIV/0!
MIR6822	0.04761904762	0.04761904762	0		inf	#DIV/0!
MIR6823	0.09523809524	0.09523809524	0		inf	#DIV/0!
MIR6824	0.09523809524	0.09523809524	0		inf	#DIV/0!
MIR6825	0.09523809524	0.09523809524	0.05555555556		1.473684211	#DIV/0!
MIR6826	0.09523809524	0.09523809524	0.05555555556		1.473684211	#DIV/0!
MIR6827	0.1428571429	0.1428571429	0.05555555556		2.333333333	#DIV/0!
MIR6828	0.1904761905	0.1904761905	0.05555555556		3.294117647	#DIV/0!
MIR6829	0.1428571429	0.1428571429	0.05555555556		2.333333333	#DIV/0!
MIR6830	0.04761904762	0.04761904762	0		inf	#DIV/0!
MIR6831	0.04761904762	0.04761904762	0		inf	#DIV/0!

**MIR6832**	0.04761904762	0.04761904762	0		inf	#DIV/0!
**MIR6833**	0.04761904762	0.04761904762	0		inf	#DIV/0!
**MIR6835**	0.04761904762	0.04761904762	0		inf	#DIV/0!
**MIR6836**	0.04761904762	0.04761904762	0.1666666667		0.2	#DIV/0!
**MIR6837**	0.04761904762	0.04761904762	0.1666666667		0.2	#DIV/0!
**MIR6838**	0.04761904762	0.04761904762	0.1666666667		0.2	#DIV/0!
**MIR6839**	0.04761904762	0.04761904762	0.05555555556		0.7	#DIV/0!
**MIR6840**	0.04761904762	0.04761904762	0.05555555556		0.7	#DIV/0!
**MIR6841**	0.04761904762	0.04761904762	0.1111111111		0.325	#DIV/0!
**MIR6842**	0.04761904762	0.04761904762	0.1111111111		0.325	#DIV/0!
**MIR6843**	0.04761904762	0.04761904762	0.1111111111		0.325	#DIV/0!
**MIR6844**	0.1428571429	0.1428571429	0.1111111111		1.083333333	#DIV/0!
**MIR6845**	0.04761904762	0.04761904762	0.1666666667		0.2	#DIV/0!
**MIR6846**	0.04761904762	0.04761904762	0.1666666667		0.2	#DIV/0!
**MIR6847**	0.04761904762	0.04761904762	0.1666666667		0.2	#DIV/0!
**MIR6848**	0.04761904762	0.04761904762	0.1666666667		0.2	#DIV/0!
**MIR6849**	0.04761904762	0.04761904762	0.1666666667		0.2	#DIV/0!
**MIR6850**	0.09523809524	0.09523809524	0.2222222222		0.2894736842	#DIV/0!
**MIR6851**	0.09523809524	0.09523809524	0.05555555556		1.473684211	#DIV/0!
**MIR6852**	0.09523809524	0.09523809524	0.05555555556		1.473684211	#DIV/0!
**MIR6853**	0.09523809524	0.09523809524	0.05555555556		1.473684211	#DIV/0!
**MIR6854**	0.04761904762	0.04761904762	0		inf	#DIV/0!
**MIR6855**	0.09523809524	0.09523809524	0.05555555556		1.473684211	#DIV/0!
**MIR6856**	0.09523809524	0.09523809524	0.05555555556		1.473684211	#DIV/0!
**IIR6859-**	0.09523809524	0.09523809524	0.3888888889		0.1203007519	#DIV/0!
**IIR6859-**	0.09523809524	0.09523809524	0.3888888889		0.1203007519	#DIV/0!
**IIR6859-**	0.09523809524	0.09523809524	0.3888888889		0.1203007519	#DIV/0!
**IIR6859-**	0.09523809524	0.09523809524	0.3888888889		0.1203007519	#DIV/0!
**MIR6861**	0	0	0.1111111111		0	#DIV/0!
**IIR6862-**	0.04761904762	0.04761904762	0		inf	#DIV/0!
**IIR6862-**	0.04761904762	0.04761904762	0		inf	#DIV/0!
**MIR6863**	0.04761904762	0.04761904762	0		inf	#DIV/0!
**MIR6864**	0	0	0.1111111111		0	#DIV/0!
**MIR6865**	0	0	0.1111111111		0	#DIV/0!
**MIR6866**	0.09523809524	0.09523809524	0		inf	#DIV/0!
**MIR6867**	0.09523809524	0.09523809524	0		inf	#DIV/0!
**MIR6868**	0.04761904762	0.04761904762	0		inf	#DIV/0!
**MIR6869**	0.1904761905	0.1904761905	0.1111111111		1.529411765	#DIV/0!
**MIR6870**	0.1904761905	0.1904761905	0.1666666667		0.9411764706	#DIV/0!
**MIR6871**	0.1428571429	0.1428571429	0.1666666667		0.6666666667	#DIV/0!
**MIR6872**	0.09523809524	0.09523809524	0		inf	#DIV/0!
**MIR6874**	0.04761904762	0.04761904762	0.1666666667		0.2	#DIV/0!
**MIR6875**	0.04761904762	0.04761904762	0.05555555556		0.7	#DIV/0!
**MIR6876**	0.04761904762	0.04761904762	0.1111111111		0.325	#DIV/0!
**MIR6877**	0.04761904762	0.04761904762	0		inf	#DIV/0!
**MIR6878**	0.1904761905	0.1904761905	0.05555555556		3.294117647	#DIV/0!
**MIR6880**	0.04761904762	0.04761904762	0.05555555556		0.7	#DIV/0!
**MIR6881**	0.04761904762	0.04761904762	0.05555555556		0.7	#DIV/0!
**MIR6882**	0.04761904762	0.04761904762	0.05555555556		0.7	#DIV/0!
**MIR6883**	0	0	0.05555555556		0	#DIV/0!
**MIR6884**	0.09523809524	0.09523809524	0		inf	#DIV/0!

MIR6885	0.1428571429	0.1428571429	0.05555555556		2.333333333	#DIV/0!
MIR6886	0.1428571429	0.1428571429	0.1111111111		1.083333333	#DIV/0!
MIR6887	0.04761904762	0.04761904762	0.05555555556		0.7	#DIV/0!
MIR6888	0	0	0.05555555556		0	#DIV/0!
MIR6889	0	0	0.1111111111		0	#DIV/0!
MIR6890	0.09523809524	0.09523809524	0		inf	#DIV/0!
MIR6892	0.04761904762	0.04761904762	0.05555555556		0.7	#DIV/0!
MIR6893	0.04761904762	0.04761904762	0.1666666667		0.2	#DIV/0!
MIR7-1	0	0	0.05555555556		0	#DIV/0!
MIR7-2	0.04761904762	0.04761904762	0.05555555556		0.7	#DIV/0!
MIR7-3	0.09523809524	0.09523809524	0.05555555556		1.473684211	#DIV/0!
IR7-3H	0.09523809524	0.09523809524	0.05555555556		1.473684211	#DIV/0!
MIR7106	0	0	0.05555555556		0	#DIV/0!
MIR7107	0.04761904762	0.04761904762	0.1111111111		0.325	#DIV/0!
MIR7108	0.09523809524	0.09523809524	0.05555555556		1.473684211	#DIV/0!
MIR7109	0.04761904762	0.04761904762	0.1111111111		0.325	#DIV/0!
MIR711	0.09523809524	0.09523809524	0		inf	#DIV/0!
MIR7110	0.04761904762	0.04761904762	0.05555555556		0.7	#DIV/0!
MIR7111	0.04761904762	0.04761904762	0.05555555556		0.7	#DIV/0!
MIR7112	0.04761904762	0.04761904762	0.1666666667		0.2	#DIV/0!
MIR7114	0.09523809524	0.09523809524	0		inf	#DIV/0!
MIR7150	0.09523809524	0.09523809524	0.05555555556		1.473684211	#DIV/0!
MIR7151	0.1428571429	0.1428571429	0		inf	#DIV/0!
MIR7152	0.09523809524	0.09523809524	0		inf	#DIV/0!
MIR7153	0.09523809524	0.09523809524	0.05555555556		1.473684211	#DIV/0!
MIR7156	0.04761904762	0.04761904762	0.05555555556		0.7	#DIV/0!
MIR7157	0	0	0.05555555556		0	#DIV/0!
MIR7158	0	0	0.05555555556		0	#DIV/0!
MIR7159	0.04761904762	0.04761904762	0		inf	#DIV/0!
MIR7160	0.1428571429	0.1428571429	0.1111111111		1.083333333	#DIV/0!
MIR7162	0.09523809524	0.09523809524	0.2222222222		0.2894736842	#DIV/0!
MIR744	0.04761904762	0.04761904762	0.05555555556		0.7	#DIV/0!
MIR7515	0	0	0.05555555556		0	#DIV/0!
IR7515H	0	0	0.05555555556		0	#DIV/0!
MIR760	0.04761904762	0.04761904762	0.05555555556		0.7	#DIV/0!
MIR761	0.04761904762	0.04761904762	0.05555555556		0.7	#DIV/0!
MIR762	0.04761904762	0.04761904762	0		inf	#DIV/0!
IR762H	0.04761904762	0.04761904762	0		inf	#DIV/0!
MIR765	0.1428571429	0.1428571429	0.05555555556		2.333333333	#DIV/0!
MIR7702	0.04761904762	0.04761904762	0.05555555556		0.7	#DIV/0!
MIR7705	0.1904761905	0.1904761905	0.1111111111		1.529411765	#DIV/0!
MIR7706	0.04761904762	0.04761904762	0.05555555556		0.7	#DIV/0!
MIR7843	0.04761904762	0.04761904762	0		inf	#DIV/0!
MIR7844	0.04761904762	0.04761904762	0.05555555556		0.7	#DIV/0!
MIR7845	0	0	0.05555555556		0	#DIV/0!
MIR7846	0.04761904762	0.04761904762	0.1111111111		0.325	#DIV/0!
MIR7847	0.04761904762	0.04761904762	0		inf	#DIV/0!
MIR7848	0.04761904762	0.04761904762	0.1666666667		0.2	#DIV/0!
MIR7849	0	0	0.05555555556		0	#DIV/0!
MIR7850	0.09523809524	0.09523809524	0.05555555556		1.473684211	#DIV/0!
MIR7851	0.04761904762	0.04761904762	0.1111111111		0.325	#DIV/0!

MIR7852	0.04761904762	0.04761904762	0.05555555556		0.7	#DIV/0!
MIR7854	0.04761904762	0.04761904762	0		inf	#DIV/0!
MIR7856	0.04761904762	0.04761904762	0.05555555556		0.7	#DIV/0!
IIR7973-	0.04761904762	0.04761904762	0.05555555556		0.7	#DIV/0!
IIR7973-	0.04761904762	0.04761904762	0.05555555556		0.7	#DIV/0!
MIR7974	0.1428571429	0.1428571429	0.1111111111		1.083333333	#DIV/0!
MIR7975	0.09523809524	0.09523809524	0.05555555556		1.473684211	#DIV/0!
MIR7976	0.09523809524	0.09523809524	0.05555555556		1.473684211	#DIV/0!
MIR7977	0.1904761905	0.1904761905	0.05555555556		3.294117647	#DIV/0!
MIR802	0.04761904762	0.04761904762	0.1666666667		0.2	#DIV/0!
MIR8053	0.04761904762	0.04761904762	0.05555555556		0.7	#DIV/0!
MIR8055	0.09523809524	0.09523809524	0.1111111111		0.6842105263	#DIV/0!
MIR8056	0	0	0.05555555556		0	#DIV/0!
MIR8057	0.09523809524	0.09523809524	0.05555555556		1.473684211	#DIV/0!
MIR8058	0.04761904762	0.04761904762	0		inf	#DIV/0!
MIR8059	0.09523809524	0.09523809524	0		inf	#DIV/0!
MIR8060	0.04761904762	0.04761904762	0.05555555556		0.7	#DIV/0!
MIR8061	0.09523809524	0.09523809524	0.05555555556		1.473684211	#DIV/0!
MIR8062	0.1904761905	0.1904761905	0.1111111111		1.529411765	#DIV/0!
MIR8063	0	0	0			#DIV/0!
MIR8064	0.04761904762	0.04761904762	0		inf	#DIV/0!
MIR8065	0.04761904762	0.04761904762	0		inf	#DIV/0!
MIR8066	0	0	0.05555555556		0	#DIV/0!
MIR8067	0.04761904762	0.04761904762	0.05555555556		0.7	#DIV/0!
IIR8069-	0.04761904762	0.04761904762	0		inf	#DIV/0!
IIR8069-	0.04761904762	0.04761904762	0		inf	#DIV/0!
MIR8072	0.04761904762	0.04761904762	0.1111111111		0.325	#DIV/0!
MIR8076	0.09523809524	0.09523809524	0.05555555556		1.473684211	#DIV/0!
MIR8077	0.04761904762	0.04761904762	0.05555555556		0.7	#DIV/0!
MIR8078	0.09523809524	0.09523809524	0		inf	#DIV/0!
MIR8080	0	0	0.05555555556		0	#DIV/0!
MIR8081	0.04761904762	0.04761904762	0.05555555556		0.7	#DIV/0!
MIR8082	0	0	0.05555555556		0	#DIV/0!
MIR8083	0.1904761905	0.1904761905	0.05555555556		3.294117647	#DIV/0!
MIR8084	0.1904761905	0.1904761905	0.1111111111		1.529411765	#DIV/0!
MIR8085	0.09523809524	0.09523809524	0.1111111111		0.6842105263	#DIV/0!
MIR8086	0.09523809524	0.09523809524	0.1111111111		0.6842105263	#DIV/0!
MIR8089	0.09523809524	0.09523809524	0.05555555556		1.473684211	#DIV/0!
MIR8485	0	0	0.05555555556		0	#DIV/0!
MIR873	0.09523809524	0.09523809524	0.05555555556		1.473684211	#DIV/0!
MIR874	0	0	0			#DIV/0!
MIR875	0.1904761905	0.1904761905	0.1111111111		1.529411765	#DIV/0!
MIR876	0.09523809524	0.09523809524	0.05555555556		1.473684211	#DIV/0!
MIR885	0.04761904762	0.04761904762	0		inf	#DIV/0!
MIR887	0.04761904762	0.04761904762	0.05555555556		0.7	#DIV/0!
MIR9-1	0.1428571429	0.1428571429	0.1111111111		1.083333333	#DIV/0!
MIR9-2	0.04761904762	0.04761904762	0		inf	#DIV/0!
MIR9-3	0.04761904762	0.04761904762	0.05555555556		0.7	#DIV/0!
IIR9-3H	0.04761904762	0.04761904762	0.05555555556		0.7	#DIV/0!
MIR920	0.04761904762	0.04761904762	0.05555555556		0.7	#DIV/0!
MIR921	0.1428571429	0.1428571429	0		inf	#DIV/0!

MIR922	0.1428571429	0.1428571429	0.05555555556		2.333333333	#DIV/0!
MIR924	0.09523809524	0.09523809524	0		inf	#DIV/0!
IR924H	0.09523809524	0.09523809524	0		inf	#DIV/0!
MIR92B	0.1904761905	0.1904761905	0.1111111111		1.529411765	#DIV/0!
MIR93	0.04761904762	0.04761904762	0.05555555556		0.7	#DIV/0!
MIR933	0	0	0.1111111111		0	#DIV/0!
MIR935	0.09523809524	0.09523809524	0.05555555556		1.473684211	#DIV/0!
MIR936	0.1428571429	0.1428571429	0.1111111111		1.083333333	#DIV/0!
MIR937	0.04761904762	0.04761904762	0.1666666667		0.2	#DIV/0!
MIR938	0.09523809524	0.09523809524	0.2222222222		0.2894736842	#DIV/0!
MIR939	0.04761904762	0.04761904762	0.1666666667		0.2	#DIV/0!
MIR940	0.09523809524	0.09523809524	0.2222222222		0.2894736842	#DIV/0!
MIR941-	0.1428571429	0.1428571429	0.2222222222		0.4583333333	#DIV/0!
MIR941-	0.1428571429	0.1428571429	0.2222222222		0.4583333333	#DIV/0!
MIR941-	0.1428571429	0.1428571429	0.2222222222		0.4583333333	#DIV/0!
MIR941-	0.1428571429	0.1428571429	0.2222222222		0.4583333333	#DIV/0!
MIR941-	0.1428571429	0.1428571429	0.2222222222		0.4583333333	#DIV/0!
MIR942	0.04761904762	0.04761904762	0.05555555556		0.7	#DIV/0!
MIR943	0.04761904762	0.04761904762	0		inf	#DIV/0!
MIR944	0.09523809524	0.09523809524	0.1111111111		0.6842105263	#DIV/0!
MIR9500	0	0	0.05555555556		0	#DIV/0!
MIR96	0.04761904762	0.04761904762	0.05555555556		0.7	#DIV/0!
MIR99A	0.04761904762	0.04761904762	0.1111111111		0.325	#DIV/0!
IR99AH	0.04761904762	0.04761904762	0.1111111111		0.325	#DIV/0!
IRLET7	0.04761904762	0.04761904762	0.05555555556		0.7	#DIV/0!
IRLET7	0	0	0.1111111111		0	#DIV/0!
IRLET7	0	0	0.1111111111		0	#DIV/0!
RLET7B	0	0	0.1111111111		0	#DIV/0!
IRLET7	0.04761904762	0.04761904762	0.1111111111		0.325	#DIV/0!
IRLET7	0.04761904762	0.04761904762	0.05555555556		0.7	#DIV/0!
IRLET7	0.04761904762	0.04761904762	0.05555555556		0.7	#DIV/0!
IRLET7	0.09523809524	0.09523809524	0		inf	#DIV/0!
MIS12	0	0	0.05555555556		0	#DIV/0!
MIS18A	0.04761904762	0.04761904762	0.1111111111		0.325	#DIV/0!
IIS18BP	0.04761904762	0.04761904762	0		inf	#DIV/0!
MISP	0.09523809524	0.09523809524	0.1111111111		0.6842105263	#DIV/0!
MISP3	0.1428571429	0.1428571429	0.1111111111		1.083333333	#DIV/0!
MITD1	0	0	0.05555555556		0	#DIV/0!
MITF	0.04761904762	0.04761904762	0		inf	#DIV/0!
MIXL1	0.1428571429	0.1428571429	0		inf	#DIV/0!
MKI67	0.2380952381	0.2380952381	0.05555555556		4.375	#DIV/0!
MKKS	0.1904761905	0.1904761905	0.1111111111		1.529411765	#DIV/0!
MKLN1	0.04761904762	0.04761904762	0.05555555556		0.7	#DIV/0!
IKLN1-A	0.04761904762	0.04761904762	0.05555555556		0.7	#DIV/0!
MKNK1	0.04761904762	0.04761904762	0.05555555556		0.7	#DIV/0!
KNK1-A	0.04761904762	0.04761904762	0.05555555556		0.7	#DIV/0!
MKNK2	0.09523809524	0.09523809524	0.1111111111		0.6842105263	#DIV/0!
MKRN1	0.04761904762	0.04761904762	0.05555555556		0.7	#DIV/0!
MKRN2	0.09523809524	0.09523809524	0		inf	#DIV/0!
IKRN2O	0.09523809524	0.09523809524	0		inf	#DIV/0!
IKRN7I	0.1428571429	0.1428571429	0.1666666667		0.6666666667	#DIV/0!

1KRN9I	0.04761904762	0.04761904762	0.05555555556		0.7	#DIV/0!
MKS1	0.09523809524	0.09523809524	0		inf	#DIV/0!
MKX	0.09523809524	0.09523809524	0.1111111111		0.6842105263	#DIV/0!
IKX-AS	0.09523809524	0.09523809524	0.1111111111		0.6842105263	#DIV/0!
MLANA	0.04761904762	0.04761904762	0.05555555556		0.7	#DIV/0!
MLC1	0	0	0.1111111111		0	#DIV/0!
MLEC	0	0	0.1111111111		0	#DIV/0!
MLF1	0.1428571429	0.1428571429	0.05555555556		2.333333333	#DIV/0!
MLF2	0.04761904762	0.04761904762	0.1111111111		0.325	#DIV/0!
MLH1	0.04761904762	0.04761904762	0		inf	#DIV/0!
MLIP	0.04761904762	0.04761904762	0		inf	#DIV/0!
ILIP-AS	0.04761904762	0.04761904762	0		inf	#DIV/0!
ILIP-IT	0.04761904762	0.04761904762	0		inf	#DIV/0!
MLKL	0.04761904762	0.04761904762	0		inf	#DIV/0!
MLLT1	0.1428571429	0.1428571429	0.05555555556		2.333333333	#DIV/0!
MLLT10	0.09523809524	0.09523809524	0.1111111111		0.6842105263	#DIV/0!
ILLT10I	0.09523809524	0.09523809524	0.05555555556		1.473684211	#DIV/0!
MLLT11	0.1904761905	0.1904761905	0.05555555556		3.294117647	#DIV/0!
MLLT3	0.04761904762	0.04761904762	0.05555555556		0.7	#DIV/0!
MLLT6	0.04761904762	0.04761904762	0		inf	#DIV/0!
MLN	0.04761904762	0.04761904762	0		inf	#DIV/0!
MLPH	0	0	0.05555555556		0	#DIV/0!
MLST8	0.09523809524	0.09523809524	0.2222222222		0.2894736842	#DIV/0!
MLX	0.04761904762	0.04761904762	0		inf	#DIV/0!
MLXIP	0.04761904762	0.04761904762	0.1111111111		0.325	#DIV/0!
MLXIPL	0.1428571429	0.1428571429	0.1111111111		1.083333333	#DIV/0!
MLYCD	0.04761904762	0.04761904762	0		inf	#DIV/0!
MMAA	0	0	0.05555555556		0	#DIV/0!
MMAB	0	0	0.05555555556		0	#DIV/0!
IMACH	0.04761904762	0.04761904762	0.05555555556		0.7	#DIV/0!
IMADH	0	0	0.05555555556		0	#DIV/0!
MMD	0.09523809524	0.09523809524	0		inf	#DIV/0!
MMD2	0.04761904762	0.04761904762	0.1666666667		0.2	#DIV/0!
MME	0.1904761905	0.1904761905	0.05555555556		3.294117647	#DIV/0!
MMEL1	0	0	0.1111111111		0	#DIV/0!
MMP11	0.04761904762	0.04761904762	0.1111111111		0.325	#DIV/0!
MMP14	0	0	0.05555555556		0	#DIV/0!
MMP15	0.04761904762	0.04761904762	0		inf	#DIV/0!
MMP16	0.1904761905	0.1904761905	0.1666666667		0.9411764706	#DIV/0!
MMP17	0.09523809524	0.09523809524	0.05555555556		1.473684211	#DIV/0!
MMP19	0.04761904762	0.04761904762	0.05555555556		0.7	#DIV/0!
MMP2	0.04761904762	0.04761904762	0		inf	#DIV/0!
IMP2-AS	0.04761904762	0.04761904762	0		inf	#DIV/0!
MMP21	0.2380952381	0.2380952381	0.05555555556		4.375	#DIV/0!
MMP23A	0	0	0.1111111111		0	#DIV/0!
MMP23I	0	0	0.1111111111		0	#DIV/0!
MMP24	0.09523809524	0.09523809524	0.1111111111		0.6842105263	#DIV/0!
4-AS1-E	0.09523809524	0.09523809524	0.1111111111		0.6842105263	#DIV/0!
IMP24O	0.09523809524	0.09523809524	0.1111111111		0.6842105263	#DIV/0!
MMP25	0.04761904762	0.04761904762	0.1111111111		0.325	#DIV/0!
MP25-A	0.04761904762	0.04761904762	0.1111111111		0.325	#DIV/0!

**MMP26**	0.04761904762	0.04761904762	0		inf	#DIV/0!
**MMP28**	0.09523809524	0.09523809524	0		inf	#DIV/0!
**MMP9**	0.1428571429	0.1428571429	0.1666666667		0.6666666667	#DIV/0!
**MMRN1**	0	0	0.05555555556		0	#DIV/0!
**MMRN2**	0.1428571429	0.1428571429	0.05555555556		2.333333333	#DIV/0!
**MMS19**	0.1428571429	0.1428571429	0		inf	#DIV/0!
**MN1**	0.04761904762	0.04761904762	0.1111111111		0.325	#DIV/0!
**MND1**	0	0	0.05555555556		0	#DIV/0!
**MNDA**	0.1428571429	0.1428571429	0		inf	#DIV/0!
**MNS1**	0.04761904762	0.04761904762	0.05555555556		0.7	#DIV/0!
**MNT**	0	0	0.1111111111		0	#DIV/0!
**MNX1**	0.1428571429	0.1428571429	0.05555555556		2.333333333	#DIV/0!
**INX1-AS**	0.1428571429	0.1428571429	0.05555555556		2.333333333	#DIV/0!
**INX1-AS**	0.1428571429	0.1428571429	0.05555555556		2.333333333	#DIV/0!
**MOB1A**	0	0	0.05555555556		0	#DIV/0!
**MOB1B**	0.09523809524	0.09523809524	0.05555555556		1.473684211	#DIV/0!
**MOB2**	0.04761904762	0.04761904762	0		inf	#DIV/0!
**MOB3A**	0.09523809524	0.09523809524	0.1111111111		0.6842105263	#DIV/0!
**MOB3B**	0.04761904762	0.04761904762	0.05555555556		0.7	#DIV/0!
**MOB3C**	0.04761904762	0.04761904762	0.05555555556		0.7	#DIV/0!
**MOB4**	0	0	0.05555555556		0	#DIV/0!
**MOBP**	0.04761904762	0.04761904762	0		inf	#DIV/0!
**MOCOS**	0.04761904762	0.04761904762	0		inf	#DIV/0!
**MOCS1**	0	0	0.05555555556		0	#DIV/0!
**MOCS2**	0.04761904762	0.04761904762	0.05555555556		0.7	#DIV/0!
**MOCS3**	0.09523809524	0.09523809524	0.1666666667		0.4210526316	#DIV/0!
**IOGAT**	0	0	0.05555555556		0	#DIV/0!
**IOGAT**	0.04761904762	0.04761904762	0.05555555556		0.7	#DIV/0!
**MOGS**	0	0	0.05555555556		0	#DIV/0!
**MON1A**	0.09523809524	0.09523809524	0		inf	#DIV/0!
**MON1B**	0.04761904762	0.04761904762	0		inf	#DIV/0!
**MON2**	0.09523809524	0.09523809524	0		inf	#DIV/0!
**MORC1**	0.09523809524	0.09523809524	0.05555555556		1.473684211	#DIV/0!
**ORC1-A**	0.09523809524	0.09523809524	0.05555555556		1.473684211	#DIV/0!
**MORC2**	0.04761904762	0.04761904762	0.1111111111		0.325	#DIV/0!
**ORC2-A**	0.04761904762	0.04761904762	0.1111111111		0.325	#DIV/0!
**MORC3**	0.04761904762	0.04761904762	0.1666666667		0.2	#DIV/0!
**IORF4L**	0.04761904762	0.04761904762	0.05555555556		0.7	#DIV/0!
**MORN1**	0	0	0.1111111111		0	#DIV/0!
**MORN2**	0	0	0.05555555556		0	#DIV/0!
**MORN3**	0.04761904762	0.04761904762	0.1111111111		0.325	#DIV/0!
**MORN4**	0.1428571429	0.1428571429	0		inf	#DIV/0!
**MORN5**	0.09523809524	0.09523809524	0.05555555556		1.473684211	#DIV/0!
**MOS**	0.1428571429	0.1428571429	0.1111111111		1.083333333	#DIV/0!
**MOSMO**	0.04761904762	0.04761904762	0		inf	#DIV/0!
**MOSPD3**	0.04761904762	0.04761904762	0.05555555556		0.7	#DIV/0!
**MOV10**	0.04761904762	0.04761904762	0.05555555556		0.7	#DIV/0!
**IOV10L**	0	0	0.1111111111		0	#DIV/0!
**IOXD2I**	0.04761904762	0.04761904762	0.05555555556		0.7	#DIV/0!
**MPC2**	0.1428571429	0.1428571429	0		inf	#DIV/0!
**MPDU1**	0	0	0.05555555556		0	#DIV/0!

**MPDZ**	0.09523809524	0.09523809524	0.05555555556		1.473684211	#DIV/0!
**MPG**	0.04761904762	0.04761904762	0.2222222222		0.1375	#DIV/0!
**PHOSPH**	0	0	0.05555555556		0	#DIV/0!
**PHOSPI**	0.04761904762	0.04761904762	0		inf	#DIV/0!
**PHOSPI**	0.04761904762	0.04761904762	0		inf	#DIV/0!
**PHOSPI**	0.04761904762	0.04761904762	0.1111111111		0.325	#DIV/0!
**MPI**	0.04761904762	0.04761904762	0.05555555556		0.7	#DIV/0!
**MPIG6B**	0.04761904762	0.04761904762	0		inf	#DIV/0!
**MPL**	0.04761904762	0.04761904762	0.05555555556		0.7	#DIV/0!
**MPLKIP**	0.04761904762	0.04761904762	0.2222222222		0.1375	#DIV/0!
**MPND**	0.09523809524	0.09523809524	0.05555555556		1.473684211	#DIV/0!
**MPO**	0.09523809524	0.09523809524	0		inf	#DIV/0!
**MPP2**	0.09523809524	0.09523809524	0		inf	#DIV/0!
**MPP3**	0.09523809524	0.09523809524	0		inf	#DIV/0!
**MPP4**	0	0	0.05555555556		0	#DIV/0!
**MPP6**	0.04761904762	0.04761904762	0.2222222222		0.1375	#DIV/0!
**MPP7**	0.09523809524	0.09523809524	0.1111111111		0.6842105263	#DIV/0!
**MPPE1**	0.09523809524	0.09523809524	0.05555555556		1.473684211	#DIV/0!
**MPPED1**	0	0	0.1666666667		0	#DIV/0!
**MPRIP**	0.04761904762	0.04761904762	0.1111111111		0.325	#DIV/0!
**MPST**	0	0	0.1111111111		0	#DIV/0!
**MPV17L**	0.04761904762	0.04761904762	0		inf	#DIV/0!
**IPV17L**	0.1428571429	0.1428571429	0.1111111111		1.083333333	#DIV/0!
**MPZ**	0.1428571429	0.1428571429	0		inf	#DIV/0!
**MPZL1**	0.1428571429	0.1428571429	0		inf	#DIV/0!
**MR1**	0.1428571429	0.1428571429	0		inf	#DIV/0!
**MRAP**	0.04761904762	0.04761904762	0.1111111111		0.325	#DIV/0!
**MRAS**	0.1428571429	0.1428571429	0.05555555556		2.333333333	#DIV/0!
**MRC1**	0.09523809524	0.09523809524	0.1111111111		0.6842105263	#DIV/0!
**MRC2**	0.04761904762	0.04761904762	0		inf	#DIV/0!
**MREG**	0	0	0.05555555556		0	#DIV/0!
**MRGBP**	0.1428571429	0.1428571429	0.2222222222		0.4583333333	#DIV/0!
**IRGPRI**	0.04761904762	0.04761904762	0		inf	#DIV/0!
**IRGPRC**	0.04761904762	0.04761904762	0		inf	#DIV/0!
**GPRG-A**	0.04761904762	0.04761904762	0		inf	#DIV/0!
**MRI1**	0.1428571429	0.1428571429	0.1111111111		1.083333333	#DIV/0!
**MRLN**	0.04761904762	0.04761904762	0.05555555556		0.7	#DIV/0!
**MRM1**	0.04761904762	0.04761904762	0		inf	#DIV/0!
**MRM2**	0.04761904762	0.04761904762	0.1666666667		0.2	#DIV/0!
**MRNIP**	0.09523809524	0.09523809524	0.05555555556		1.473684211	#DIV/0!
**MRO**	0.04761904762	0.04761904762	0		inf	#DIV/0!
**MROH1**	0.04761904762	0.04761904762	0.1666666667		0.2	#DIV/0!
**IROH2A**	0	0	0.05555555556		0	#DIV/0!
**IROH2I**	0.04761904762	0.04761904762	0.05555555556		0.7	#DIV/0!
**IROH3I**	0.1428571429	0.1428571429	0		inf	#DIV/0!
**MROH5**	0.04761904762	0.04761904762	0.2222222222		0.1375	#DIV/0!
**MROH6**	0.04761904762	0.04761904762	0.1666666667		0.2	#DIV/0!
**MROH7**	0.04761904762	0.04761904762	0.05555555556		0.7	#DIV/0!
**OH7-TT**	0.04761904762	0.04761904762	0.05555555556		0.7	#DIV/0!
**MROH8**	0.09523809524	0.09523809524	0.1111111111		0.6842105263	#DIV/0!
**MROH9**	0.1428571429	0.1428571429	0.05555555556		2.333333333	#DIV/0!

MRPL1	0.04761904762	0.04761904762	0.05555555556		0.7	#DIV/0!
MRPL10	0.1428571429	0.1428571429	0		inf	#DIV/0!
MRPL12	0.04761904762	0.04761904762	0		inf	#DIV/0!
MRPL13	0.09523809524	0.09523809524	0.1111111111		0.6842105263	#DIV/0!
MRPL14	0.04761904762	0.04761904762	0.05555555556		0.7	#DIV/0!
MRPL15	0.1428571429	0.1428571429	0.1111111111		1.083333333	#DIV/0!
MRPL17	0.04761904762	0.04761904762	0		inf	#DIV/0!
MRPL19	0	0	0.05555555556		0	#DIV/0!
MRPL2	0.04761904762	0.04761904762	0.05555555556		0.7	#DIV/0!
MRPL20	0	0	0.1111111111		0	#DIV/0!
RPL20-A	0	0	0.1111111111		0	#DIV/0!
MRPL22	0	0	0.05555555556		0	#DIV/0!
MRPL23	0.04761904762	0.04761904762	0		inf	#DIV/0!
RPL23-A	0.04761904762	0.04761904762	0		inf	#DIV/0!
MRPL24	0.1428571429	0.1428571429	0.1111111111		1.083333333	#DIV/0!
MRPL27	0.09523809524	0.09523809524	0		inf	#DIV/0!
MRPL28	0.04761904762	0.04761904762	0.2222222222		0.1375	#DIV/0!
MRPL3	0.09523809524	0.09523809524	0.05555555556		1.473684211	#DIV/0!
MRPL30	0	0	0.05555555556		0	#DIV/0!
MRPL32	0.04761904762	0.04761904762	0.2222222222		0.1375	#DIV/0!
MRPL33	0	0	0.1111111111		0	#DIV/0!
MRPL34	0.1428571429	0.1428571429	0.1111111111		1.083333333	#DIV/0!
MRPL35	0	0	0.05555555556		0	#DIV/0!
MRPL36	0	0	0.1111111111		0	#DIV/0!
MRPL37	0.04761904762	0.04761904762	0.05555555556		0.7	#DIV/0!
MRPL38	0.04761904762	0.04761904762	0		inf	#DIV/0!
MRPL39	0.04761904762	0.04761904762	0.1111111111		0.325	#DIV/0!
MRPL40	0.04761904762	0.04761904762	0.05555555556		0.7	#DIV/0!
MRPL41	0.09523809524	0.09523809524	0		inf	#DIV/0!
MRPL42	0.04761904762	0.04761904762	0.05555555556		0.7	#DIV/0!
MRPL43	0.1428571429	0.1428571429	0		inf	#DIV/0!
MRPL44	0	0	0.05555555556		0	#DIV/0!
IRPL45I	0.1428571429	0.1428571429	0		inf	#DIV/0!
MRPL46	0.04761904762	0.04761904762	0.05555555556		0.7	#DIV/0!
MRPL47	0.1904761905	0.1904761905	0.05555555556		3.294117647	#DIV/0!
MRPL50	0.04761904762	0.04761904762	0		inf	#DIV/0!
MRPL51	0.04761904762	0.04761904762	0.1111111111		0.325	#DIV/0!
MRPL52	0	0	0.05555555556		0	#DIV/0!
MRPL53	0	0	0.05555555556		0	#DIV/0!
MRPL54	0.09523809524	0.09523809524	0.05555555556		1.473684211	#DIV/0!
MRPL55	0.1428571429	0.1428571429	0		inf	#DIV/0!
MRPL57	0.04761904762	0.04761904762	0		inf	#DIV/0!
MRPL58	0.04761904762	0.04761904762	0		inf	#DIV/0!
MRPL9	0.1904761905	0.1904761905	0		inf	#DIV/0!
MRPS10	0.04761904762	0.04761904762	0.05555555556		0.7	#DIV/0!
MRPS11	0.04761904762	0.04761904762	0.05555555556		0.7	#DIV/0!
MRPS12	0.04761904762	0.04761904762	0.05555555556		0.7	#DIV/0!
MRPS14	0.1428571429	0.1428571429	0		inf	#DIV/0!
MRPS15	0.04761904762	0.04761904762	0.05555555556		0.7	#DIV/0!
MRPS16	0.1904761905	0.1904761905	0.05555555556		3.294117647	#DIV/0!
MRPS17	0.3333333333	0.3333333333	0.2222222222		1.375	#DIV/0!

IRPS18A	0.04761904762	0.04761904762	0.05555555556		0.7	#DIV/0!
IRPS18C	0	0	0.05555555556		0	#DIV/0!
MRPS2	0.04761904762	0.04761904762	0		inf	#DIV/0!
MRPS21	0.1904761905	0.1904761905	0.05555555556		3.294117647	#DIV/0!
MRPS22	0.1428571429	0.1428571429	0.05555555556		2.333333333	#DIV/0!
MRPS23	0.09523809524	0.09523809524	0		inf	#DIV/0!
MRPS24	0.04761904762	0.04761904762	0.1666666667		0.2	#DIV/0!
MRPS25	0.04761904762	0.04761904762	0		inf	#DIV/0!
MRPS26	0.1904761905	0.1904761905	0.05555555556		3.294117647	#DIV/0!
MRPS27	0	0	0.05555555556		0	#DIV/0!
MRPS28	0.1428571429	0.1428571429	0.1111111111		1.083333333	#DIV/0!
MRPS30	0.04761904762	0.04761904762	0.05555555556		0.7	#DIV/0!
RPS30-D	0.04761904762	0.04761904762	0.05555555556		0.7	#DIV/0!
MRPS33	0.04761904762	0.04761904762	0.1111111111		0.325	#DIV/0!
MRPS34	0.09523809524	0.09523809524	0.2222222222		0.2894736842	#DIV/0!
MRPS35	0.04761904762	0.04761904762	0.05555555556		0.7	#DIV/0!
MRPS36	0.04761904762	0.04761904762	0.05555555556		0.7	#DIV/0!
MRPS5	0	0	0.1111111111		0	#DIV/0!
MRPS6	0.04761904762	0.04761904762	0.1111111111		0.325	#DIV/0!
MRPS7	0.04761904762	0.04761904762	0.05555555556		0.7	#DIV/0!
MRPS9	0	0	0.05555555556		0	#DIV/0!
MRRF	0.09523809524	0.09523809524	0.05555555556		1.473684211	#DIV/0!
MRS2	0	0	0.05555555556		0	#DIV/0!
MRS2P2	0.04761904762	0.04761904762	0.1111111111		0.325	#DIV/0!
MRTFA	0	0	0.1111111111		0	#DIV/0!
RTFA-A	0	0	0.1111111111		0	#DIV/0!
MRTFB	0.04761904762	0.04761904762	0		inf	#DIV/0!
MRTO4	0.04761904762	0.04761904762	0.1111111111		0.325	#DIV/0!
ISANTD	0.04761904762	0.04761904762	0		inf	#DIV/0!
ISANTD	0.04761904762	0.04761904762	0		inf	#DIV/0!
TD3-TM	0.04761904762	0.04761904762	0		inf	#DIV/0!
MSC	0.1904761905	0.1904761905	0.1666666667		0.9411764706	#DIV/0!
ISC-AS	0.1904761905	0.1904761905	0.1666666667		0.9411764706	#DIV/0!
MSGN1	0	0	0.05555555556		0	#DIV/0!
MSH2	0	0	0.05555555556		0	#DIV/0!
MSH3	0.04761904762	0.04761904762	0		inf	#DIV/0!
MSH4	0.04761904762	0.04761904762	0.05555555556		0.7	#DIV/0!
MSH5	0.04761904762	0.04761904762	0		inf	#DIV/0!
H5-SAPC	0.04761904762	0.04761904762	0		inf	#DIV/0!
MSH6	0	0	0.05555555556		0	#DIV/0!
MSI1	0	0	0.1111111111		0	#DIV/0!
MSI2	0.09523809524	0.09523809524	0		inf	#DIV/0!
MSL1	0.09523809524	0.09523809524	0		inf	#DIV/0!
MSL2	0.1904761905	0.1904761905	0.05555555556		3.294117647	#DIV/0!
MSL3P1	0	0	0.05555555556		0	#DIV/0!
MSLN	0.04761904762	0.04761904762	0.2222222222		0.1375	#DIV/0!
MSMB	0.04761904762	0.04761904762	0.05555555556		0.7	#DIV/0!
MSMO1	0	0	0.1111111111		0	#DIV/0!
MSMP	0.09523809524	0.09523809524	0.05555555556		1.473684211	#DIV/0!
MSR1	0.09523809524	0.09523809524	0.1111111111		0.6842105263	#DIV/0!
MSRA	0.09523809524	0.09523809524	0.1111111111		0.6842105263	#DIV/0!

**MSRB1**	0.09523809524	0.09523809524	0.2222222222		0.2894736842	#DIV/0!
**MSRB2**	0.09523809524	0.09523809524	0.1111111111		0.6842105263	#DIV/0!
**MSRB3**	0.09523809524	0.09523809524	0.05555555556		1.473684211	#DIV/0!
**MSS51**	0.1904761905	0.1904761905	0.05555555556		3.294117647	#DIV/0!
**MST1**	0.09523809524	0.09523809524	0		inf	#DIV/0!
**MST1L**	0.04761904762	0.04761904762	0.1111111111		0.325	#DIV/0!
**MST1P2**	0.04761904762	0.04761904762	0.1111111111		0.325	#DIV/0!
**MST1R**	0.09523809524	0.09523809524	0		inf	#DIV/0!
**MSTN**	0	0	0.05555555556		0	#DIV/0!
**MSTO1**	0.1904761905	0.1904761905	0.1111111111		1.529411765	#DIV/0!
**MSTO2P**	0.1904761905	0.1904761905	0.1111111111		1.529411765	#DIV/0!
**MSX2**	0	0	0.05555555556		0	#DIV/0!
**MSX2P1**	0.09523809524	0.09523809524	0		inf	#DIV/0!
**MT1A**	0.04761904762	0.04761904762	0		inf	#DIV/0!
**MT1B**	0.04761904762	0.04761904762	0		inf	#DIV/0!
**MT1DP**	0.04761904762	0.04761904762	0		inf	#DIV/0!
**MT1E**	0.04761904762	0.04761904762	0		inf	#DIV/0!
**MT1F**	0.04761904762	0.04761904762	0		inf	#DIV/0!
**MT1G**	0.04761904762	0.04761904762	0		inf	#DIV/0!
**MT1H**	0.04761904762	0.04761904762	0		inf	#DIV/0!
**MT1HL1**	0.1428571429	0.1428571429	0		inf	#DIV/0!
**MT1IP**	0.04761904762	0.04761904762	0		inf	#DIV/0!
**MT1JP**	0.04761904762	0.04761904762	0		inf	#DIV/0!
**MT1L**	0.04761904762	0.04761904762	0		inf	#DIV/0!
**MT1M**	0.04761904762	0.04761904762	0		inf	#DIV/0!
**MT1X**	0.04761904762	0.04761904762	0		inf	#DIV/0!
**MT2A**	0.04761904762	0.04761904762	0		inf	#DIV/0!
**MT3**	0.04761904762	0.04761904762	0		inf	#DIV/0!
**MT4**	0.04761904762	0.04761904762	0		inf	#DIV/0!
**MTA3**	0	0	0.05555555556		0	#DIV/0!
**MTBP**	0.09523809524	0.09523809524	0.1111111111		0.6842105263	#DIV/0!
**MTCH1**	0.04761904762	0.04761904762	0.05555555556		0.7	#DIV/0!
**MTCH2**	0	0	0.05555555556		0	#DIV/0!
**MTCL1**	0.09523809524	0.09523809524	0		inf	#DIV/0!
**MTDH**	0.1904761905	0.1904761905	0.1111111111		1.529411765	#DIV/0!
**MTERF1**	0.04761904762	0.04761904762	0.1111111111		0.325	#DIV/0!
**MTERF2**	0.04761904762	0.04761904762	0.05555555556		0.7	#DIV/0!
**MTERF3**	0.1904761905	0.1904761905	0.1666666667		0.9411764706	#DIV/0!
**MTERF4**	0	0	0.05555555556		0	#DIV/0!
**MTF1**	0.04761904762	0.04761904762	0.05555555556		0.7	#DIV/0!
**MTF2**	0.04761904762	0.04761904762	0.05555555556		0.7	#DIV/0!
**MTFMT**	0.04761904762	0.04761904762	0.05555555556		0.7	#DIV/0!
**MTFP1**	0.04761904762	0.04761904762	0.1111111111		0.325	#DIV/0!
**MTFR1**	0.1428571429	0.1428571429	0.1666666667		0.6666666667	#DIV/0!
**MTFR1L**	0.04761904762	0.04761904762	0.1111111111		0.325	#DIV/0!
**MTG1**	0.1904761905	0.1904761905	0		inf	#DIV/0!
**MTG2**	0.1428571429	0.1428571429	0.2222222222		0.4583333333	#DIV/0!
**MTHFD1**	0.09523809524	0.09523809524	0		inf	#DIV/0!
**MTHFD2**	0	0	0.05555555556		0	#DIV/0!
**MTHFD2**	0.09523809524	0.09523809524	0.05555555556		1.473684211	#DIV/0!
**MTHFD2L**	0.04761904762	0.04761904762	0.05555555556		0.7	#DIV/0!

MTHFR	0.04761904762	0.04761904762	0.1111111111		0.325	#DIV/0!
MTHFS	0.04761904762	0.04761904762	0.05555555556		0.7	#DIV/0!
ITHFSI	0.04761904762	0.04761904762	0		inf	#DIV/0!
MTIF2	0.04761904762	0.04761904762	0.05555555556		0.7	#DIV/0!
MTLN	0	0	0.05555555556		0	#DIV/0!
ITMR1	0.1428571429	0.1428571429	0.05555555556		2.333333333	#DIV/0!
ITMR12	0.04761904762	0.04761904762	0.05555555556		0.7	#DIV/0!
ITMR14	0.09523809524	0.09523809524	0		inf	#DIV/0!
MTMR3	0.04761904762	0.04761904762	0.1111111111		0.325	#DIV/0!
MTMR4	0.09523809524	0.09523809524	0		inf	#DIV/0!
MTMR7	0.09523809524	0.09523809524	0.1111111111		0.6842105263	#DIV/0!
MTMR9	0.09523809524	0.09523809524	0.1111111111		0.6842105263	#DIV/0!
ITMR9L	0.04761904762	0.04761904762	0.1111111111		0.325	#DIV/0!
ITNR1A	0	0	0.05555555556		0	#DIV/0!
MTO1	0.04761904762	0.04761904762	0		inf	#DIV/0!
MTOR	0.04761904762	0.04761904762	0.1111111111		0.325	#DIV/0!
TOR-AS	0.04761904762	0.04761904762	0.1111111111		0.325	#DIV/0!
MTPAP	0.09523809524	0.09523809524	0.2222222222		0.2894736842	#DIV/0!
MTPN	0.04761904762	0.04761904762	0.05555555556		0.7	#DIV/0!
MTR	0.1428571429	0.1428571429	0		inf	#DIV/0!
MTREX	0.04761904762	0.04761904762	0.05555555556		0.7	#DIV/0!
TRNR2I	0.09523809524	0.09523809524	0.05555555556		1.473684211	#DIV/0!
TRNR2I	0.04761904762	0.04761904762	0		inf	#DIV/0!
TRNR2I	0.1428571429	0.1428571429	0.2222222222		0.4583333333	#DIV/0!
TRNR2I	0.04761904762	0.04761904762	0.1111111111		0.325	#DIV/0!
TRNR2I	0.09523809524	0.09523809524	0.05555555556		1.473684211	#DIV/0!
TRNR2I	0.04761904762	0.04761904762	0.05555555556		0.7	#DIV/0!
TRNR2I	0.09523809524	0.09523809524	0.1111111111		0.6842105263	#DIV/0!
MTRR	0	0	0.05555555556		0	#DIV/0!
MTSS1	0.1428571429	0.1428571429	0.1111111111		1.083333333	#DIV/0!
MTSS2	0.04761904762	0.04761904762	0		inf	#DIV/0!
MTTP	0	0	0.05555555556		0	#DIV/0!
MTURN	0.04761904762	0.04761904762	0.1666666667		0.2	#DIV/0!
MTUS1	0.09523809524	0.09523809524	0.1111111111		0.6842105263	#DIV/0!
MTVR2	0.09523809524	0.09523809524	0		inf	#DIV/0!
MTX1	0.1904761905	0.1904761905	0.1111111111		1.529411765	#DIV/0!
MTX2	0	0	0.1111111111		0	#DIV/0!
MTX3	0.04761904762	0.04761904762	0		inf	#DIV/0!
MUC1	0.1904761905	0.1904761905	0.1111111111		1.529411765	#DIV/0!
MUC12	0.04761904762	0.04761904762	0.05555555556		0.7	#DIV/0!
MUC13	0.04761904762	0.04761904762	0.05555555556		0.7	#DIV/0!
MUC16	0.1428571429	0.1428571429	0.05555555556		2.333333333	#DIV/0!
MUC17	0.04761904762	0.04761904762	0.05555555556		0.7	#DIV/0!
MUC19	0.04761904762	0.04761904762	0.05555555556		0.7	#DIV/0!
MUC2	0.04761904762	0.04761904762	0		inf	#DIV/0!
MUC20	0.1428571429	0.1428571429	0.05555555556		2.333333333	#DIV/0!
MUC3A	0.04761904762	0.04761904762	0.05555555556		0.7	#DIV/0!
MUC4	0.1428571429	0.1428571429	0.05555555556		2.333333333	#DIV/0!
IUC5AC	0.04761904762	0.04761904762	0		inf	#DIV/0!
MUC5B	0.04761904762	0.04761904762	0		inf	#DIV/0!
UC5B-A	0.04761904762	0.04761904762	0		inf	#DIV/0!

**MUC6**	0.04761904762	0.04761904762	0		inf	#DIV/0!
**MUC7**	0.04761904762	0.04761904762	0.05555555556		0.7	#DIV/0!
**MUCL1**	0.04761904762	0.04761904762	0.05555555556		0.7	#DIV/0!
**MUL1**	0.04761904762	0.04761904762	0.1111111111		0.325	#DIV/0!
**MUSK**	0.04761904762	0.04761904762	0.05555555556		0.7	#DIV/0!
**MUSTN1**	0.04761904762	0.04761904762	0		inf	#DIV/0!
**MUTYH**	0.04761904762	0.04761904762	0.05555555556		0.7	#DIV/0!
**MVB12A**	0.1428571429	0.1428571429	0.1111111111		1.083333333	#DIV/0!
**MVB12B**	0.09523809524	0.09523809524	0.05555555556		1.473684211	#DIV/0!
**MVD**	0.1428571429	0.1428571429	0		inf	#DIV/0!
**MVK**	0	0	0.05555555556		0	#DIV/0!
**MVP**	0.04761904762	0.04761904762	0		inf	#DIV/0!
**MX1**	0.04761904762	0.04761904762	0.1111111111		0.325	#DIV/0!
**MX2**	0.04761904762	0.04761904762	0.1111111111		0.325	#DIV/0!
**MXD1**	0	0	0.05555555556		0	#DIV/0!
**MXD3**	0.09523809524	0.09523809524	0.05555555556		1.473684211	#DIV/0!
**MXD4**	0.04761904762	0.04761904762	0		inf	#DIV/0!
**MXI1**	0.1428571429	0.1428571429	0.05555555556		2.333333333	#DIV/0!
**MXRA7**	0.04761904762	0.04761904762	0		inf	#DIV/0!
**MXRA8**	0	0	0.1111111111		0	#DIV/0!
**MYADM**	0.09523809524	0.09523809524	0.05555555556		1.473684211	#DIV/0!
**YADML**	0.04761904762	0.04761904762	0		inf	#DIV/0!
**YBBP1A**	0	0	0.1111111111		0	#DIV/0!
**MYBL1**	0.1428571429	0.1428571429	0.1666666667		0.6666666667	#DIV/0!
**MYBL2**	0.1428571429	0.1428571429	0.1111111111		1.083333333	#DIV/0!
**MYBPC1**	0.04761904762	0.04761904762	0.05555555556		0.7	#DIV/0!
**MYBPC2**	0.09523809524	0.09523809524	0.1111111111		0.6842105263	#DIV/0!
**MYBPC3**	0	0	0.05555555556		0	#DIV/0!
**MYBPH**	0.1428571429	0.1428571429	0		inf	#DIV/0!
**MYBPHL**	0.04761904762	0.04761904762	0.05555555556		0.7	#DIV/0!
**MYC**	0.09523809524	0.09523809524	0.1111111111		0.6842105263	#DIV/0!
**MYCBP**	0.04761904762	0.04761904762	0.05555555556		0.7	#DIV/0!
**MYCBP2**	0	0	0.05555555556		0	#DIV/0!
**YCBP2-A**	0	0	0.05555555556		0	#DIV/0!
**MYCBPAP**	0.09523809524	0.09523809524	0		inf	#DIV/0!
**MYCL**	0.04761904762	0.04761904762	0.05555555556		0.7	#DIV/0!
**MYCN**	0	0	0.05555555556		0	#DIV/0!
**MYCNOS**	0	0	0.05555555556		0	#DIV/0!
**MYCNUT**	0	0	0.05555555556		0	#DIV/0!
**MYD88**	0.04761904762	0.04761904762	0		inf	#DIV/0!
**MYDGF**	0.09523809524	0.09523809524	0.05555555556		1.473684211	#DIV/0!
**MYEF2**	0.04761904762	0.04761904762	0.05555555556		0.7	#DIV/0!
**MYF5**	0.04761904762	0.04761904762	0.05555555556		0.7	#DIV/0!
**MYF6**	0.04761904762	0.04761904762	0.05555555556		0.7	#DIV/0!
**MYH1**	0.04761904762	0.04761904762	0.05555555556		0.7	#DIV/0!
**MYH10**	0.04761904762	0.04761904762	0.05555555556		0.7	#DIV/0!
**MYH11**	0.04761904762	0.04761904762	0		inf	#DIV/0!
**MYH13**	0.04761904762	0.04761904762	0.05555555556		0.7	#DIV/0!
**MYH14**	0.09523809524	0.09523809524	0.1111111111		0.6842105263	#DIV/0!
**MYH15**	0.09523809524	0.09523809524	0.05555555556		1.473684211	#DIV/0!
**MYH16**	0.04761904762	0.04761904762	0.05555555556		0.7	#DIV/0!

**MYH2**	0.04761904762	0.04761904762	0.05555555556		0.7	#DIV/0!
**MYH3**	0.04761904762	0.04761904762	0.05555555556		0.7	#DIV/0!
**MYH4**	0.04761904762	0.04761904762	0.05555555556		0.7	#DIV/0!
**MYH6**	0	0	0.05555555556		0	#DIV/0!
**MYH7**	0	0	0.05555555556		0	#DIV/0!
**MYH7B**	0.09523809524	0.09523809524	0.1111111111		0.6842105263	#DIV/0!
**MYH8**	0.04761904762	0.04761904762	0.05555555556		0.7	#DIV/0!
**MYH9**	0	0	0.1111111111		0	#DIV/0!
**MYHAS**	0.04761904762	0.04761904762	0.05555555556		0.7	#DIV/0!
**MYL1**	0	0	0.05555555556		0	#DIV/0!
**MYL10**	0.04761904762	0.04761904762	0.05555555556		0.7	#DIV/0!
**MYL12A**	0.09523809524	0.09523809524	0		inf	#DIV/0!
**MYL12B**	0.09523809524	0.09523809524	0		inf	#DIV/0!
**MYL2**	0	0	0.1111111111		0	#DIV/0!
**MYL4**	0.1428571429	0.1428571429	0		inf	#DIV/0!
**MYL5**	0.04761904762	0.04761904762	0		inf	#DIV/0!
**MYL6**	0.09523809524	0.09523809524	0.05555555556		1.473684211	#DIV/0!
**MYL6B**	0.09523809524	0.09523809524	0.05555555556		1.473684211	#DIV/0!
**MYL7**	0.04761904762	0.04761904762	0.1666666667		0.2	#DIV/0!
**MYL9**	0.09523809524	0.09523809524	0.1111111111		0.6842105263	#DIV/0!
**MYLK**	0.04761904762	0.04761904762	0.05555555556		0.7	#DIV/0!
**YLK-AS**	0.04761904762	0.04761904762	0.05555555556		0.7	#DIV/0!
**YLK-AS**	0.04761904762	0.04761904762	0.05555555556		0.7	#DIV/0!
**MYLK2**	0.09523809524	0.09523809524	0.1111111111		0.6842105263	#DIV/0!
**MYLK3**	0.04761904762	0.04761904762	0		inf	#DIV/0!
**MYLPF**	0.04761904762	0.04761904762	0		inf	#DIV/0!
**MYMK**	0.04761904762	0.04761904762	0		inf	#DIV/0!
**MYMX**	0.04761904762	0.04761904762	0.05555555556		0.7	#DIV/0!
**MYNN**	0.1904761905	0.1904761905	0.05555555556		3.294117647	#DIV/0!
**MYO10**	0.04761904762	0.04761904762	0.05555555556		0.7	#DIV/0!
**MYO15A**	0.04761904762	0.04761904762	0.1111111111		0.325	#DIV/0!
**MYO18A**	0.04761904762	0.04761904762	0.05555555556		0.7	#DIV/0!
**MYO18B**	0.04761904762	0.04761904762	0.05555555556		0.7	#DIV/0!
**MYO19**	0.04761904762	0.04761904762	0		inf	#DIV/0!
**MYO1A**	0.1428571429	0.1428571429	0.05555555556		2.333333333	#DIV/0!
**MYO1B**	0	0	0.05555555556		0	#DIV/0!
**MYO1C**	0	0	0.1111111111		0	#DIV/0!
**MYO1D**	0.04761904762	0.04761904762	0		inf	#DIV/0!
**MYO1E**	0.04761904762	0.04761904762	0.05555555556		0.7	#DIV/0!
**MYO1F**	0.1428571429	0.1428571429	0.05555555556		2.333333333	#DIV/0!
**MYO1G**	0.04761904762	0.04761904762	0.2222222222		0.1375	#DIV/0!
**MYO1H**	0	0	0.05555555556		0	#DIV/0!
**MYO3A**	0.09523809524	0.09523809524	0.1111111111		0.6842105263	#DIV/0!
**MYO3B**	0	0	0.05555555556		0	#DIV/0!
**YO3B-A**	0	0	0.05555555556		0	#DIV/0!
**MYO5A**	0.04761904762	0.04761904762	0.05555555556		0.7	#DIV/0!
**MYO5B**	0.04761904762	0.04761904762	0		inf	#DIV/0!
**MYO5C**	0.04761904762	0.04761904762	0.05555555556		0.7	#DIV/0!
**MYO7B**	0	0	0.05555555556		0	#DIV/0!
**MYO9A**	0.04761904762	0.04761904762	0.05555555556		0.7	#DIV/0!
**MYO9B**	0.1428571429	0.1428571429	0.1111111111		1.083333333	#DIV/0!

**MYOC**	0.1428571429	0.1428571429	0.05555555556		2.333333333	#DIV/0!
**MYOCD**	0.04761904762	0.04761904762	0.05555555556		0.7	#DIV/0!
**IYOCO:**	0.1428571429	0.1428571429	0.05555555556		2.333333333	#DIV/0!
**MYOF**	0.1428571429	0.1428571429	0.1111111111		1.083333333	#DIV/0!
**MYOG**	0.1428571429	0.1428571429	0		inf	#DIV/0!
**MYOM1**	0.09523809524	0.09523809524	0		inf	#DIV/0!
**MYOM2**	0.1428571429	0.1428571429	0.1111111111		1.083333333	#DIV/0!
**MYOM3**	0.04761904762	0.04761904762	0.1111111111		0.325	#DIV/0!
**YOPAR**	0.1428571429	0.1428571429	0		inf	#DIV/0!
**MYORG**	0.09523809524	0.09523809524	0.05555555556		1.473684211	#DIV/0!
**IYOSLI**	0	0	0.05555555556		0	#DIV/0!
**OSLID-**	0	0	0.05555555556		0	#DIV/0!
**MYOT**	0.04761904762	0.04761904762	0		inf	#DIV/0!
**MYOZ1**	0.1904761905	0.1904761905	0.05555555556		3.294117647	#DIV/0!
**MYOZ2**	0	0	0.05555555556		0	#DIV/0!
**MYPN**	0.1904761905	0.1904761905	0.05555555556		3.294117647	#DIV/0!
**MYPOP**	0.09523809524	0.09523809524	0.1111111111		0.6842105263	#DIV/0!
**MYRFL**	0.09523809524	0.09523809524	0.1111111111		0.6842105263	#DIV/0!
**MYRIP**	0.04761904762	0.04761904762	0		inf	#DIV/0!
**MYSM1**	0.04761904762	0.04761904762	0.05555555556		0.7	#DIV/0!
**MYT1**	0.1428571429	0.1428571429	0.2222222222		0.4583333333	#DIV/0!
**MYT1L**	0	0	0.05555555556		0	#DIV/0!
**YT1L-A**	0	0	0.05555555556		0	#DIV/0!
**MYZAP**	0.04761904762	0.04761904762	0.05555555556		0.7	#DIV/0!
**MZB1**	0.04761904762	0.04761904762	0		inf	#DIV/0!
**MZF1**	0.1428571429	0.1428571429	0.05555555556		2.333333333	#DIV/0!
**IZF1-AS**	0.1428571429	0.1428571429	0.05555555556		2.333333333	#DIV/0!
**MZT2A**	0	0	0.05555555556		0	#DIV/0!
**MZT2B**	0	0	0.05555555556		0	#DIV/0!
**N4BP1**	0.04761904762	0.04761904762	0		inf	#DIV/0!
**N4BP2**	0.1904761905	0.1904761905	0.05555555556		3.294117647	#DIV/0!
**N4BP3**	0.09523809524	0.09523809524	0.05555555556		1.473684211	#DIV/0!
**N6AMT1**	0.04761904762	0.04761904762	0.1111111111		0.325	#DIV/0!
**NAA11**	0.04761904762	0.04761904762	0.05555555556		0.7	#DIV/0!
**NAA15**	0.04761904762	0.04761904762	0.05555555556		0.7	#DIV/0!
**NAA20**	0.1904761905	0.1904761905	0.05555555556		3.294117647	#DIV/0!
**NAA25**	0	0	0.1111111111		0	#DIV/0!
**NAA30**	0.04761904762	0.04761904762	0		inf	#DIV/0!
**NAA35**	0	0	0.05555555556		0	#DIV/0!
**NAA38**	0	0	0.05555555556		0	#DIV/0!
**NAA50**	0.09523809524	0.09523809524	0.05555555556		1.473684211	#DIV/0!
**NAA60**	0.04761904762	0.04761904762	0.1111111111		0.325	#DIV/0!
**NAA80**	0.09523809524	0.09523809524	0		inf	#DIV/0!
**NAAA**	0.04761904762	0.04761904762	0.05555555556		0.7	#DIV/0!
**AALADI**	0.1904761905	0.1904761905	0.05555555556		3.294117647	#DIV/0!
**LADL2-**	0.1904761905	0.1904761905	0.05555555556		3.294117647	#DIV/0!
**LADL2-**	0.1904761905	0.1904761905	0.05555555556		3.294117647	#DIV/0!
**LADL2-**	0.1904761905	0.1904761905	0.05555555556		3.294117647	#DIV/0!
**NAB1**	0	0	0.05555555556		0	#DIV/0!
**NAB2**	0.1428571429	0.1428571429	0.05555555556		2.333333333	#DIV/0!
**NABP1**	0	0	0.05555555556		0	#DIV/0!

**NABP2**	0.09523809524	0.09523809524	0.05555555556		1.473684211	#DIV/0!
**NACA**	0.1428571429	0.1428571429	0.05555555556		2.333333333	#DIV/0!
**NACA2**	0.09523809524	0.09523809524	0		inf	#DIV/0!
**NACA4P**	0.1904761905	0.1904761905	0.1111111111		1.529411765	#DIV/0!
**NACAD**	0.04761904762	0.04761904762	0.2222222222		0.1375	#DIV/0!
**NACC1**	0.1428571429	0.1428571429	0.1111111111		1.083333333	#DIV/0!
**NACC2**	0.1428571429	0.1428571429	0.05555555556		2.333333333	#DIV/0!
**NADK**	0	0	0.1111111111		0	#DIV/0!
**NADK2**	0.04761904762	0.04761904762	0.05555555556		0.7	#DIV/0!
**NAE1**	0.09523809524	0.09523809524	0.05555555556		1.473684211	#DIV/0!
**NAF1**	0	0	0.05555555556		0	#DIV/0!
**NAGA**	0	0	0.1111111111		0	#DIV/0!
**NAGK**	0	0	0.05555555556		0	#DIV/0!
**NAGPA**	0.04761904762	0.04761904762	0		inf	#DIV/0!
**AGPA-A**	0.04761904762	0.04761904762	0		inf	#DIV/0!
**NAGS**	0.09523809524	0.09523809524	0		inf	#DIV/0!
**NAIF1**	0.09523809524	0.09523809524	0.05555555556		1.473684211	#DIV/0!
**NAIP**	0.09523809524	0.09523809524	0.1666666667		0.4210526316	#DIV/0!
**NALT1**	0.1428571429	0.1428571429	0.05555555556		2.333333333	#DIV/0!
**NAMA**	0.04761904762	0.04761904762	0		inf	#DIV/0!
**NAMPT**	0.04761904762	0.04761904762	0.05555555556		0.7	#DIV/0!
**NANOG**	0.04761904762	0.04761904762	0.1111111111		0.325	#DIV/0!
**ANOGN**	0.04761904762	0.04761904762	0.1111111111		0.325	#DIV/0!
**NANOS1**	0.2380952381	0.2380952381	0		inf	#DIV/0!
**NANOS2**	0.09523809524	0.09523809524	0.1111111111		0.6842105263	#DIV/0!
**NANOS3**	0.1428571429	0.1428571429	0.1111111111		1.083333333	#DIV/0!
**NANP**	0.1904761905	0.1904761905	0.05555555556		3.294117647	#DIV/0!
**NANS**	0.04761904762	0.04761904762	0		inf	#DIV/0!
**NAP1L1**	0.04761904762	0.04761904762	0.1111111111		0.325	#DIV/0!
**NAP1L4**	0.04761904762	0.04761904762	0		inf	#DIV/0!
**NAP1L5**	0	0	0.05555555556		0	#DIV/0!
**NAPA**	0.09523809524	0.09523809524	0.1111111111		0.6842105263	#DIV/0!
**APA-AS**	0.09523809524	0.09523809524	0.1111111111		0.6842105263	#DIV/0!
**NAPB**	0.1904761905	0.1904761905	0.05555555556		3.294117647	#DIV/0!
**APEPLI**	0.04761904762	0.04761904762	0.05555555556		0.7	#DIV/0!
**NAPG**	0.09523809524	0.09523809524	0		inf	#DIV/0!
**NAPRT**	0.04761904762	0.04761904762	0.1666666667		0.2	#DIV/0!
**NAPSA**	0.09523809524	0.09523809524	0.1111111111		0.6842105263	#DIV/0!
**NAPSB**	0.09523809524	0.09523809524	0.1111111111		0.6842105263	#DIV/0!
**NARF**	0.04761904762	0.04761904762	0		inf	#DIV/0!
**NARS**	0.09523809524	0.09523809524	0		inf	#DIV/0!
**NASP**	0.04761904762	0.04761904762	0.05555555556		0.7	#DIV/0!
**NAT1**	0.09523809524	0.09523809524	0.1111111111		0.6842105263	#DIV/0!
**NAT14**	0.09523809524	0.09523809524	0.05555555556		1.473684211	#DIV/0!
**NAT16**	0.04761904762	0.04761904762	0.05555555556		0.7	#DIV/0!
**NAT2**	0.09523809524	0.09523809524	0.1111111111		0.6842105263	#DIV/0!
**NAT8**	0	0	0.05555555556		0	#DIV/0!
**NAT8B**	0	0	0.05555555556		0	#DIV/0!
**NAT8L**	0.04761904762	0.04761904762	0		inf	#DIV/0!
**NAT9**	0.04761904762	0.04761904762	0		inf	#DIV/0!
**NATD1**	0.09523809524	0.09523809524	0.05555555556		1.473684211	#DIV/0!

NAV1	0.1428571429	0.1428571429	0		inf	#DIV/0!
NAV3	0.04761904762	0.04761904762	0.05555555556		0.7	#DIV/0!
NAXE	0.1428571429	0.1428571429	0.1111111111		1.083333333	#DIV/0!
NBAS	0	0	0.05555555556		0	#DIV/0!
NBEAL1	0.09523809524	0.09523809524	0.05555555556		1.473684211	#DIV/0!
NBEAP1	0.2857142857	0.2857142857	0.1111111111		2.6	#DIV/0!
NBL1	0.04761904762	0.04761904762	0.1111111111		0.325	#DIV/0!
NBN	0.1904761905	0.1904761905	0.1111111111		1.529411765	#DIV/0!
NBPF1	0.04761904762	0.04761904762	0.1111111111		0.325	#DIV/0!
NBPF10	0.1428571429	0.1428571429	0.05555555556		2.333333333	#DIV/0!
NBPF11	0.1428571429	0.1428571429	0.05555555556		2.333333333	#DIV/0!
NBPF12	0.1428571429	0.1428571429	0.05555555556		2.333333333	#DIV/0!
NBPF13I	0.1428571429	0.1428571429	0.05555555556		2.333333333	#DIV/0!
NBPF14	0.1428571429	0.1428571429	0.05555555556		2.333333333	#DIV/0!
NBPF15	0.1428571429	0.1428571429	0.05555555556		2.333333333	#DIV/0!
NBPF18I	0.1904761905	0.1904761905	0		inf	#DIV/0!
NBPF19	0.1428571429	0.1428571429	0.05555555556		2.333333333	#DIV/0!
NBPF20	0.1428571429	0.1428571429	0.05555555556		2.333333333	#DIV/0!
NBPF22I	0.04761904762	0.04761904762	0		inf	#DIV/0!
NBPF25I	0.1428571429	0.1428571429	0.05555555556		2.333333333	#DIV/0!
NBPF26	0.1428571429	0.1428571429	0.05555555556		2.333333333	#DIV/0!
NBPF3	0.04761904762	0.04761904762	0.1111111111		0.325	#DIV/0!
NBPF4	0.04761904762	0.04761904762	0.05555555556		0.7	#DIV/0!
NBPF6	0.04761904762	0.04761904762	0.05555555556		0.7	#DIV/0!
NBPF7	0.04761904762	0.04761904762	0.05555555556		0.7	#DIV/0!
NBPF8	0.09523809524	0.09523809524	0.05555555556		1.473684211	#DIV/0!
NBPF9	0.1428571429	0.1428571429	0.05555555556		2.333333333	#DIV/0!
NBR1	0.04761904762	0.04761904762	0		inf	#DIV/0!
NBR2	0.04761904762	0.04761904762	0		inf	#DIV/0!
NCALD	0.1904761905	0.1904761905	0.1111111111		1.529411765	#DIV/0!
NCAM2	0.04761904762	0.04761904762	0.1666666667		0.2	#DIV/0!
NCAN	0.1428571429	0.1428571429	0.1111111111		1.083333333	#DIV/0!
NCAPD2	0.04761904762	0.04761904762	0.1111111111		0.325	#DIV/0!
NCAPG	0	0	0			#DIV/0!
NCAPG2	0.1428571429	0.1428571429	0.05555555556		2.333333333	#DIV/0!
NCAPH	0	0	0.1111111111		0	#DIV/0!
NCAPH2	0	0	0.1111111111		0	#DIV/0!
NCBP1	0.04761904762	0.04761904762	0		inf	#DIV/0!
NCBP2	0.1428571429	0.1428571429	0.05555555556		2.333333333	#DIV/0!
CBP2-AS	0.1428571429	0.1428571429	0.05555555556		2.333333333	#DIV/0!
CBP2AS	0.1428571429	0.1428571429	0.05555555556		2.333333333	#DIV/0!
NCBP3	0	0	0.1111111111		0	#DIV/0!
NCCRP1	0.04761904762	0.04761904762	0.05555555556		0.7	#DIV/0!
NCDN	0.04761904762	0.04761904762	0.05555555556		0.7	#DIV/0!
NCEH1	0.1904761905	0.1904761905	0.05555555556		3.294117647	#DIV/0!
NCF1	0.1428571429	0.1428571429	0.1666666667		0.6666666667	#DIV/0!
NCF1B	0.1428571429	0.1428571429	0.1111111111		1.083333333	#DIV/0!
NCF1C	0.1428571429	0.1428571429	0.1666666667		0.6666666667	#DIV/0!
NCF4	0	0	0.1111111111		0	#DIV/0!
CF4-AS	0	0	0.1111111111		0	#DIV/0!
NCK1	0.1904761905	0.1904761905	0.05555555556		3.294117647	#DIV/0!

**\CK1-D7**	0.1904761905	0.1904761905	0.05555555556		3.294117647	#DIV/0!
**NCK2**	0	0	0.05555555556		0	#DIV/0!
**NCKAP1**	0	0	0.05555555556		0	#DIV/0!
**CKAP1**	0.04761904762	0.04761904762	0.05555555556		0.7	#DIV/0!
**NCKAP5**	0	0	0.05555555556		0	#DIV/0!
**KAP5-A**	0	0	0.05555555556		0	#DIV/0!
**KAP5-A**	0	0	0.05555555556		0	#DIV/0!
**CKAP5**	0.04761904762	0.04761904762	0.05555555556		0.7	#DIV/0!
**CKIPSI**	0.09523809524	0.09523809524	0		inf	#DIV/0!
**NCL**	0	0	0.05555555556		0	#DIV/0!
**NCLN**	0.09523809524	0.09523809524	0.05555555556		1.473684211	#DIV/0!
**NCMAP**	0.04761904762	0.04761904762	0.1111111111		0.325	#DIV/0!
**NCOA2**	0.1904761905	0.1904761905	0.1666666667		0.9411764706	#DIV/0!
**NCOA3**	0.1428571429	0.1428571429	0.1666666667		0.6666666667	#DIV/0!
**NCOA4**	0.04761904762	0.04761904762	0.05555555556		0.7	#DIV/0!
**NCOA5**	0.1428571429	0.1428571429	0.1666666667		0.6666666667	#DIV/0!
**NCOA6**	0.09523809524	0.09523809524	0.1111111111		0.6842105263	#DIV/0!
**NCOR1**	0.04761904762	0.04761904762	0.05555555556		0.7	#DIV/0!
**ICOR1P**	0.1904761905	0.1904761905	0.05555555556		3.294117647	#DIV/0!
**NCOR2**	0.04761904762	0.04761904762	0.05555555556		0.7	#DIV/0!
**NCR1**	0.09523809524	0.09523809524	0.05555555556		1.473684211	#DIV/0!
**NCR2**	0	0	0.05555555556		0	#DIV/0!
**NCR3**	0.04761904762	0.04761904762	0		inf	#DIV/0!
**RNA002**	0.04761904762	0.04761904762	0.2222222222		0.1375	#DIV/0!
**CRUPA**	0.04761904762	0.04761904762	0		inf	#DIV/0!
**NCS1**	0.09523809524	0.09523809524	0.05555555556		1.473684211	#DIV/0!
**NDC1**	0.04761904762	0.04761904762	0.05555555556		0.7	#DIV/0!
**NDC80**	0.09523809524	0.09523809524	0		inf	#DIV/0!
**NDE1**	0.04761904762	0.04761904762	0		inf	#DIV/0!
**NDEL1**	0.04761904762	0.04761904762	0.05555555556		0.7	#DIV/0!
**NDFIP1**	0.04761904762	0.04761904762	0		inf	#DIV/0!
**NDNF**	0	0	0.05555555556		0	#DIV/0!
**NDOR1**	0.1428571429	0.1428571429	0.05555555556		2.333333333	#DIV/0!
**NDRG1**	0.04761904762	0.04761904762	0.2222222222		0.1375	#DIV/0!
**NDRG3**	0.09523809524	0.09523809524	0.1111111111		0.6842105263	#DIV/0!
**NDRG4**	0.04761904762	0.04761904762	0		inf	#DIV/0!
**NDST2**	0.1904761905	0.1904761905	0.05555555556		3.294117647	#DIV/0!
**NDST3**	0	0	0.05555555556		0	#DIV/0!
**NDST4**	0	0	0.05555555556		0	#DIV/0!
**NDUFA1**	0	0	0.05555555556		0	#DIV/0!
**NDUFA1**	0.1428571429	0.1428571429	0.05555555556		2.333333333	#DIV/0!
**NDUFA1**	0.04761904762	0.04761904762	0.05555555556		0.7	#DIV/0!
**NDUFA1**	0.1428571429	0.1428571429	0.1111111111		1.083333333	#DIV/0!
**NDUFA2**	0.04761904762	0.04761904762	0		inf	#DIV/0!
**NDUFA3**	0.09523809524	0.09523809524	0.05555555556		1.473684211	#DIV/0!
**NDUFA4**	0.04761904762	0.04761904762	0.2222222222		0.1375	#DIV/0!
**DUFA4I**	0.1428571429	0.1428571429	0.05555555556		2.333333333	#DIV/0!
**NDUFA5**	0.04761904762	0.04761904762	0.05555555556		0.7	#DIV/0!
**NDUFA6**	0	0	0.1111111111		0	#DIV/0!
**DUFA6-I**	0	0	0.1111111111		0	#DIV/0!
**NDUFA7**	0.1428571429	0.1428571429	0.05555555556		2.333333333	#DIV/0!

NDUFA8	0.09523809524	0.09523809524	0.05555555556		1.473684211	#DIV/0!
NDUFA9	0.04761904762	0.04761904762	0.1111111111		0.325	#DIV/0!
IDUFAB	0.04761904762	0.04761904762	0		inf	#DIV/0!
NDUFAF	0.04761904762	0.04761904762	0		inf	#DIV/0!
NDUFAF	0.09523809524	0.09523809524	0		inf	#DIV/0!
DUFAF4	0.04761904762	0.04761904762	0.05555555556		0.7	#DIV/0!
NDUFAF	0.1904761905	0.1904761905	0.1111111111		1.529411765	#DIV/0!
NDUFAF	0.1904761905	0.1904761905	0.1111111111		1.529411765	#DIV/0!
NDUFAF	0	0	0.05555555556		0	#DIV/0!
NDUFAF	0.04761904762	0.04761904762	0		inf	#DIV/0!
NDUFB1	0.09523809524	0.09523809524	0.2222222222		0.2894736842	#DIV/0!
NDUFB2	0.04761904762	0.04761904762	0.05555555556		0.7	#DIV/0!
UFB2-A	0.04761904762	0.04761904762	0.05555555556		0.7	#DIV/0!
NDUFB3	0	0	0.05555555556		0	#DIV/0!
NDUFB4	0.09523809524	0.09523809524	0.05555555556		1.473684211	#DIV/0!
NDUFB5	0.1904761905	0.1904761905	0.05555555556		3.294117647	#DIV/0!
NDUFB6	0.09523809524	0.09523809524	0.05555555556		1.473684211	#DIV/0!
NDUFB7	0.1428571429	0.1428571429	0.1111111111		1.083333333	#DIV/0!
NDUFB8	0.1428571429	0.1428571429	0		inf	#DIV/0!
NDUFB9	0.1428571429	0.1428571429	0.1111111111		1.083333333	#DIV/0!
NDUFC1	0.04761904762	0.04761904762	0.05555555556		0.7	#DIV/0!
NDUFS1	0	0	0.05555555556		0	#DIV/0!
NDUFS2	0.1428571429	0.1428571429	0		inf	#DIV/0!
NDUFS3	0	0	0.05555555556		0	#DIV/0!
NDUFS4	0.04761904762	0.04761904762	0.05555555556		0.7	#DIV/0!
NDUFS5	0.04761904762	0.04761904762	0.05555555556		0.7	#DIV/0!
NDUFS6	0	0	0.1111111111		0	#DIV/0!
NDUFS7	0.09523809524	0.09523809524	0.1111111111		0.6842105263	#DIV/0!
NDUFV2	0.09523809524	0.09523809524	0		inf	#DIV/0!
UFV2-A	0.09523809524	0.09523809524	0		inf	#DIV/0!
NDUFV3	0.04761904762	0.04761904762	0.1666666667		0.2	#DIV/0!
NEB	0	0	0.05555555556		0	#DIV/0!
NEBL	0.09523809524	0.09523809524	0.1111111111		0.6842105263	#DIV/0!
EBL-AS	0.09523809524	0.09523809524	0.1111111111		0.6842105263	#DIV/0!
NECAB1	0.1904761905	0.1904761905	0.1111111111		1.529411765	#DIV/0!
NECAB2	0.04761904762	0.04761904762	0		inf	#DIV/0!
NECAB3	0.09523809524	0.09523809524	0.1111111111		0.6842105263	#DIV/0!
NECAP1	0.04761904762	0.04761904762	0.1111111111		0.325	#DIV/0!
NECAP2	0.04761904762	0.04761904762	0.1111111111		0.325	#DIV/0!
NECTIN	0.09523809524	0.09523809524	0.1111111111		0.6842105263	#DIV/0!
NECTIN	0.09523809524	0.09523809524	0.05555555556		1.473684211	#DIV/0!
CTIN3-A	0.09523809524	0.09523809524	0.05555555556		1.473684211	#DIV/0!
NECTIN	0.1428571429	0.1428571429	0		inf	#DIV/0!
NEDD1	0.04761904762	0.04761904762	0.05555555556		0.7	#DIV/0!
NEDD4	0.04761904762	0.04761904762	0.05555555556		0.7	#DIV/0!
NEDD4L	0.09523809524	0.09523809524	0		inf	#DIV/0!
NEDD9	0.04761904762	0.04761904762	0		inf	#DIV/0!
NEFH	0.04761904762	0.04761904762	0.1111111111		0.325	#DIV/0!
NEFL	0.04761904762	0.04761904762	0.1111111111		0.325	#DIV/0!
NEFM	0.04761904762	0.04761904762	0.1111111111		0.325	#DIV/0!
NEGR1	0.04761904762	0.04761904762	0.05555555556		0.7	#DIV/0!

EGR1-IT	0.04761904762	0.04761904762	0.05555555556		0.7	#DIV/0!
NEIL1	0.04761904762	0.04761904762	0.05555555556		0.7	#DIV/0!
NEIL2	0.09523809524	0.09523809524	0.1111111111		0.6842105263	#DIV/0!
NEIL3	0	0	0.05555555556		0	#DIV/0!
NEK1	0	0	0.05555555556		0	#DIV/0!
NEK10	0.04761904762	0.04761904762	0		inf	#DIV/0!
NEK11	0.09523809524	0.09523809524	0.05555555556		1.473684211	#DIV/0!
NEK2	0.1428571429	0.1428571429	0		inf	#DIV/0!
NEK4	0.04761904762	0.04761904762	0		inf	#DIV/0!
NEK6	0.09523809524	0.09523809524	0.05555555556		1.473684211	#DIV/0!
NEK7	0.1428571429	0.1428571429	0.1111111111		1.083333333	#DIV/0!
NEK8	0.04761904762	0.04761904762	0.05555555556		0.7	#DIV/0!
NELFA	0.04761904762	0.04761904762	0		inf	#DIV/0!
NELFB	0.1428571429	0.1428571429	0.05555555556		2.333333333	#DIV/0!
NELFCD	0.1428571429	0.1428571429	0.2222222222		0.4583333333	#DIV/0!
NELFE	0.04761904762	0.04761904762	0		inf	#DIV/0!
NELL2	0.04761904762	0.04761904762	0.05555555556		0.7	#DIV/0!
NEMF	0.04761904762	0.04761904762	0		inf	#DIV/0!
NEMP1	0.1428571429	0.1428571429	0.05555555556		2.333333333	#DIV/0!
NEMP2	0	0	0.05555555556		0	#DIV/0!
NENF	0.09523809524	0.09523809524	0		inf	#DIV/0!
NEO1	0.04761904762	0.04761904762	0.05555555556		0.7	#DIV/0!
NEPRO	0.09523809524	0.09523809524	0.05555555556		1.473684211	#DIV/0!
NES	0.1428571429	0.1428571429	0.1111111111		1.083333333	#DIV/0!
NET1	0	0	0.1666666667		0	#DIV/0!
NETO2	0.04761904762	0.04761904762	0		inf	#DIV/0!
NEU1	0.04761904762	0.04761904762	0		inf	#DIV/0!
NEU2	0	0	0.05555555556		0	#DIV/0!
NEU4	0	0	0.05555555556		0	#DIV/0!
NEURL1	0.1428571429	0.1428571429	0.05555555556		2.333333333	#DIV/0!
URL1-A	0.1428571429	0.1428571429	0.05555555556		2.333333333	#DIV/0!
NEURL1	0	0	0.05555555556		0	#DIV/0!
NEURL2	0.1428571429	0.1428571429	0.1666666667		0.6666666667	#DIV/0!
NEURL3	0	0	0.1111111111		0	#DIV/0!
NEURL4	0	0	0.05555555556		0	#DIV/0!
EUROD	0	0	0.05555555556		0	#DIV/0!
EUROD	0.09523809524	0.09523809524	0		inf	#DIV/0!
EUROD	0.04761904762	0.04761904762	0.05555555556		0.7	#DIV/0!
EUROD	0.04761904762	0.04761904762	0.1111111111		0.325	#DIV/0!
EUROG	0.04761904762	0.04761904762	0		inf	#DIV/0!
EUROG	0	0	0.05555555556		0	#DIV/0!
EUROG	0.09523809524	0.09523809524	0		inf	#DIV/0!
NEXN	0.04761904762	0.04761904762	0.05555555556		0.7	#DIV/0!
EXN-AS	0.04761904762	0.04761904762	0.05555555556		0.7	#DIV/0!
NF1	0	0	0			#DIV/0!
NF1P2	0.2857142857	0.2857142857	0.1111111111		2.6	#DIV/0!
NF2	0.04761904762	0.04761904762	0.1111111111		0.325	#DIV/0!
NFAM1	0	0	0.1666666667		0	#DIV/0!
NFASC	0.2380952381	0.2380952381	0		inf	#DIV/0!
NFAT5	0.1428571429	0.1428571429	0.05555555556		2.333333333	#DIV/0!
NFATC1	0.04761904762	0.04761904762	0.05555555556		0.7	#DIV/0!

NFATC2	0.09523809524	0.09523809524	0.1666666667		0.4210526316	#DIV/0!
FATC2I	0.04761904762	0.04761904762	0		inf	#DIV/0!
NFATC3	0.1428571429	0.1428571429	0.05555555556		2.333333333	#DIV/0!
NFE2	0.04761904762	0.04761904762	0.05555555556		0.7	#DIV/0!
NFE2L1	0.1428571429	0.1428571429	0		inf	#DIV/0!
NFE2L2	0	0	0.1111111111		0	#DIV/0!
NFE2L3	0.04761904762	0.04761904762	0.2222222222		0.1375	#DIV/0!
NFE4	0.04761904762	0.04761904762	0.05555555556		0.7	#DIV/0!
NFIA	0.04761904762	0.04761904762	0.1111111111		0.325	#DIV/0!
IFIA-AS	0.04761904762	0.04761904762	0.1111111111		0.325	#DIV/0!
IFIA-AS	0.04761904762	0.04761904762	0.1111111111		0.325	#DIV/0!
NFIB	0.1428571429	0.1428571429	0.05555555556		2.333333333	#DIV/0!
NFIC	0.09523809524	0.09523809524	0.05555555556		1.473684211	#DIV/0!
NFIL3	0.04761904762	0.04761904762	0.05555555556		0.7	#DIV/0!
NFIX	0.1428571429	0.1428571429	0.1111111111		1.083333333	#DIV/0!
NFKB1	0	0	0.05555555556		0	#DIV/0!
NFKB2	0.1428571429	0.1428571429	0.05555555556		2.333333333	#DIV/0!
NFKBIA	0.09523809524	0.09523809524	0		inf	#DIV/0!
NFKBIB	0.04761904762	0.04761904762	0.05555555556		0.7	#DIV/0!
NFKBID	0.04761904762	0.04761904762	0.05555555556		0.7	#DIV/0!
NFKBIE	0.04761904762	0.04761904762	0.05555555556		0.7	#DIV/0!
NFKBIL	0.04761904762	0.04761904762	0		inf	#DIV/0!
NFKBIZ	0.09523809524	0.09523809524	0.05555555556		1.473684211	#DIV/0!
NFS1	0.09523809524	0.09523809524	0.1111111111		0.6842105263	#DIV/0!
NFU1	0	0	0.05555555556		0	#DIV/0!
NFX1	0.09523809524	0.09523809524	0.05555555556		1.473684211	#DIV/0!
NFXL1	0.04761904762	0.04761904762	0.1111111111		0.325	#DIV/0!
NFYA	0	0	0.05555555556		0	#DIV/0!
NFYB	0.04761904762	0.04761904762	0.05555555556		0.7	#DIV/0!
NFYC	0.04761904762	0.04761904762	0.05555555556		0.7	#DIV/0!
FYC-AS	0.04761904762	0.04761904762	0.05555555556		0.7	#DIV/0!
NGDN	0	0	0.05555555556		0	#DIV/0!
NGEF	0	0	0.05555555556		0	#DIV/0!
NGF	0.04761904762	0.04761904762	0.05555555556		0.7	#DIV/0!
NGF-AS1	0.04761904762	0.04761904762	0.05555555556		0.7	#DIV/0!
NGFR	0.09523809524	0.09523809524	0		inf	#DIV/0!
NGRN	0.04761904762	0.04761904762	0.05555555556		0.7	#DIV/0!
NHEJ1	0	0	0.05555555556		0	#DIV/0!
NHLH2	0.04761904762	0.04761904762	0.05555555556		0.7	#DIV/0!
NHLRC2	0.1428571429	0.1428571429	0.05555555556		2.333333333	#DIV/0!
NHLRC4	0.04761904762	0.04761904762	0.2222222222		0.1375	#DIV/0!
NHP2	0.09523809524	0.09523809524	0.05555555556		1.473684211	#DIV/0!
NICN1	0.1428571429	0.1428571429	0		inf	#DIV/0!
NID1	0.1428571429	0.1428571429	0.05555555556		2.333333333	#DIV/0!
NID2	0.04761904762	0.04761904762	0		inf	#DIV/0!
NIF3L1	0	0	0.05555555556		0	#DIV/0!
NIFK	0	0	0.05555555556		0	#DIV/0!
IIFK-AS	0	0	0.05555555556		0	#DIV/0!
NIM1K	0.04761904762	0.04761904762	0.05555555556		0.7	#DIV/0!
NIN	0.04761904762	0.04761904762	0		inf	#DIV/0!
NINJ1	0.04761904762	0.04761904762	0.05555555556		0.7	#DIV/0!

NINJ2	0.04761904762	0.04761904762	0.1111111111		0.325	#DIV/0!
NINL	0.1904761905	0.1904761905	0.05555555556		3.294117647	#DIV/0!
NIP7	0.1428571429	0.1428571429	0.05555555556		2.333333333	#DIV/0!
NIPA1	0.09523809524	0.09523809524	0		inf	#DIV/0!
NIPA2	0.09523809524	0.09523809524	0		inf	#DIV/0!
NIPAL1	0.04761904762	0.04761904762	0.1111111111		0.325	#DIV/0!
NIPAL2	0.1904761905	0.1904761905	0.1111111111		1.529411765	#DIV/0!
NIPAL3	0.04761904762	0.04761904762	0.1111111111		0.325	#DIV/0!
NIPAL4	0.04761904762	0.04761904762	0.05555555556		0.7	#DIV/0!
NIPBL	0.04761904762	0.04761904762	0.05555555556		0.7	#DIV/0!
IPBL-D	0.04761904762	0.04761904762	0.05555555556		0.7	#DIV/0!
IPSNAP	0.04761904762	0.04761904762	0.1111111111		0.325	#DIV/0!
IPSNAP	0.3333333333	0.3333333333	0.2222222222		1.375	#DIV/0!
PSNAP3	0.04761904762	0.04761904762	0.05555555556		0.7	#DIV/0!
PSNAP3	0.04761904762	0.04761904762	0.05555555556		0.7	#DIV/0!
NISCH	0.04761904762	0.04761904762	0		inf	#DIV/0!
NIT1	0.1428571429	0.1428571429	0		inf	#DIV/0!
NIT2	0.09523809524	0.09523809524	0.05555555556		1.473684211	#DIV/0!
NKAIN1	0.04761904762	0.04761904762	0.1111111111		0.325	#DIV/0!
NKAIN3	0.1428571429	0.1428571429	0.1666666667		0.6666666667	#DIV/0!
KAIN3-I	0.1428571429	0.1428571429	0.1666666667		0.6666666667	#DIV/0!
NKAIN4	0.1428571429	0.1428571429	0.2222222222		0.4583333333	#DIV/0!
NKD1	0.04761904762	0.04761904762	0		inf	#DIV/0!
NKD2	0	0	0.05555555556		0	#DIV/0!
NKILA	0.1428571429	0.1428571429	0.2222222222		0.4583333333	#DIV/0!
NKIRAS	0.04761904762	0.04761904762	0		inf	#DIV/0!
NKIRAS	0.09523809524	0.09523809524	0		inf	#DIV/0!
NKPD1	0.09523809524	0.09523809524	0.1111111111		0.6842105263	#DIV/0!
NKTR	0.04761904762	0.04761904762	0		inf	#DIV/0!
NKX1-1	0.04761904762	0.04761904762	0		inf	#DIV/0!
NKX1-2	0.2380952381	0.2380952381	0.05555555556		4.375	#DIV/0!
NKX2-2	0.1904761905	0.1904761905	0.05555555556		3.294117647	#DIV/0!
NKX2-3	0.1428571429	0.1428571429	0		inf	#DIV/0!
NKX2-4	0.1904761905	0.1904761905	0.05555555556		3.294117647	#DIV/0!
NKX2-5	0	0	0.05555555556		0	#DIV/0!
NKX2-6	0.04761904762	0.04761904762	0.1111111111		0.325	#DIV/0!
NKX3-1	0.04761904762	0.04761904762	0.1111111111		0.325	#DIV/0!
NKX3-2	0	0	0			#DIV/0!
NKX6-1	0	0	0.05555555556		0	#DIV/0!
NKX6-2	0.2380952381	0.2380952381	0		inf	#DIV/0!
NKX6-3	0.09523809524	0.09523809524	0.1666666667		0.4210526316	#DIV/0!
NLGN1	0.1904761905	0.1904761905	0.05555555556		3.294117647	#DIV/0!
LGN1-A	0.1904761905	0.1904761905	0.05555555556		3.294117647	#DIV/0!
NLGN2	0	0	0.05555555556		0	#DIV/0!
NLK	0.04761904762	0.04761904762	0.05555555556		0.7	#DIV/0!
NLN	0.04761904762	0.04761904762	0		inf	#DIV/0!
NLRC3	0.04761904762	0.04761904762	0.1111111111		0.325	#DIV/0!
NLRC4	0.1428571429	0.1428571429	0		inf	#DIV/0!
NLRC5	0.04761904762	0.04761904762	0		inf	#DIV/0!
NLRP1	0	0	0.05555555556		0	#DIV/0!
NLRP11	0.1428571429	0.1428571429	0.05555555556		2.333333333	#DIV/0!

**NLRP12**	0.09523809524	0.09523809524	0.05555555556		1.473684211	#DIV/0!
**NLRP13**	0.1428571429	0.1428571429	0.05555555556		2.333333333	#DIV/0!
**NLRP14**	0.04761904762	0.04761904762	0		inf	#DIV/0!
**NLRP2**	0.09523809524	0.09523809524	0.05555555556		1.473684211	#DIV/0!
**NLRP3**	0.1428571429	0.1428571429	0		inf	#DIV/0!
**NLRP4**	0.1428571429	0.1428571429	0.05555555556		2.333333333	#DIV/0!
**NLRP5**	0.1428571429	0.1428571429	0.05555555556		2.333333333	#DIV/0!
**NLRP6**	0.04761904762	0.04761904762	0		inf	#DIV/0!
**NLRP7**	0.09523809524	0.09523809524	0.05555555556		1.473684211	#DIV/0!
**NLRP8**	0.1428571429	0.1428571429	0.05555555556		2.333333333	#DIV/0!
**NLRP9**	0.1428571429	0.1428571429	0.05555555556		2.333333333	#DIV/0!
**NMB**	0.04761904762	0.04761904762	0.05555555556		0.7	#DIV/0!
**NMD3**	0.1428571429	0.1428571429	0.05555555556		2.333333333	#DIV/0!
**NME1**	0.04761904762	0.04761904762	0		inf	#DIV/0!
**IE1-NM**	0.04761904762	0.04761904762	0		inf	#DIV/0!
**NME2**	0.04761904762	0.04761904762	0		inf	#DIV/0!
**NME3**	0.09523809524	0.09523809524	0.2222222222		0.2894736842	#DIV/0!
**NME4**	0.04761904762	0.04761904762	0.2222222222		0.1375	#DIV/0!
**NME5**	0.04761904762	0.04761904762	0		inf	#DIV/0!
**NME6**	0.09523809524	0.09523809524	0		inf	#DIV/0!
**NME7**	0.1428571429	0.1428571429	0		inf	#DIV/0!
**NME8**	0.04761904762	0.04761904762	0.2222222222		0.1375	#DIV/0!
**NME9**	0.1428571429	0.1428571429	0.05555555556		2.333333333	#DIV/0!
**NMI**	0	0	0.05555555556		0	#DIV/0!
**NMNAT**	0	0	0.1111111111		0	#DIV/0!
**NMNAT**	0.1428571429	0.1428571429	0		inf	#DIV/0!
**NMNAT**	0.1428571429	0.1428571429	0.05555555556		2.333333333	#DIV/0!
**NMRAL**	0.04761904762	0.04761904762	0		inf	#DIV/0!
**MRAL2**	0.09523809524	0.09523809524	0.05555555556		1.473684211	#DIV/0!
**NMRK1**	0	0	0.05555555556		0	#DIV/0!
**NMRK2**	0.09523809524	0.09523809524	0.05555555556		1.473684211	#DIV/0!
**NMS**	0	0	0.05555555556		0	#DIV/0!
**NMT1**	0.1428571429	0.1428571429	0		inf	#DIV/0!
**NMT2**	0.09523809524	0.09523809524	0.1111111111		0.6842105263	#DIV/0!
**NMU**	0.1904761905	0.1904761905	0.05555555556		3.294117647	#DIV/0!
**NMUR1**	0	0	0.05555555556		0	#DIV/0!
**NNAT**	0.09523809524	0.09523809524	0.1666666667		0.4210526316	#DIV/0!
**NNT**	0.04761904762	0.04761904762	0.05555555556		0.7	#DIV/0!
**NNT-AS1**	0.04761904762	0.04761904762	0.05555555556		0.7	#DIV/0!
**NOA1**	0.1904761905	0.1904761905	0.05555555556		3.294117647	#DIV/0!
**NOB1**	0.1428571429	0.1428571429	0.05555555556		2.333333333	#DIV/0!
**NOBOX**	0.09523809524	0.09523809524	0.05555555556		1.473684211	#DIV/0!
**NOC2L**	0	0	0.1111111111		0	#DIV/0!
**NOC2LP**	0	0	0.05555555556		0	#DIV/0!
**NOC3L**	0.1428571429	0.1428571429	0		inf	#DIV/0!
**NOC4L**	0.09523809524	0.09523809524	0.05555555556		1.473684211	#DIV/0!
**NOCT**	0.04761904762	0.04761904762	0.05555555556		0.7	#DIV/0!
**NOD1**	0.04761904762	0.04761904762	0.1666666667		0.2	#DIV/0!
**NOD2**	0.04761904762	0.04761904762	0		inf	#DIV/0!
**NODAL**	0.09523809524	0.09523809524	0		inf	#DIV/0!
**NOG**	0.09523809524	0.09523809524	0		inf	#DIV/0!

**NOL10**	0	0	0.05555555556		0	#DIV/0!
**NOL11**	0	0	0			#DIV/0!
**NOL12**	0	0	0.1111111111		0	#DIV/0!
**NOL3**	0.1428571429	0.1428571429	0.05555555556		2.333333333	#DIV/0!
**NOL4**	0.09523809524	0.09523809524	0		inf	#DIV/0!
**NOL4L**	0.09523809524	0.09523809524	0.1111111111		0.6842105263	#DIV/0!
**OL4L-D**	0.09523809524	0.09523809524	0.1111111111		0.6842105263	#DIV/0!
**NOL6**	0.09523809524	0.09523809524	0.05555555556		1.473684211	#DIV/0!
**NOL8**	0.04761904762	0.04761904762	0.05555555556		0.7	#DIV/0!
**NOL9**	0	0	0.05555555556		0	#DIV/0!
**NOLC1**	0.1428571429	0.1428571429	0.05555555556		2.333333333	#DIV/0!
**NOM1**	0.1428571429	0.1428571429	0.05555555556		2.333333333	#DIV/0!
**NOMO1**	0.04761904762	0.04761904762	0		inf	#DIV/0!
**NOMO2**	0.09523809524	0.09523809524	0		inf	#DIV/0!
**NOMO3**	0.04761904762	0.04761904762	0		inf	#DIV/0!
**NOP14**	0.04761904762	0.04761904762	0		inf	#DIV/0!
**OP14-AS**	0.04761904762	0.04761904762	0		inf	#DIV/0!
**NOP16**	0.09523809524	0.09523809524	0.05555555556		1.473684211	#DIV/0!
**NOP2**	0.04761904762	0.04761904762	0.1111111111		0.325	#DIV/0!
**NOP53**	0.09523809524	0.09523809524	0.1111111111		0.6842105263	#DIV/0!
**OP53-AS**	0.09523809524	0.09523809524	0.1111111111		0.6842105263	#DIV/0!
**NOP56**	0.1904761905	0.1904761905	0.05555555556		3.294117647	#DIV/0!
**NOP58**	0.09523809524	0.09523809524	0.05555555556		1.473684211	#DIV/0!
**NORAD**	0.09523809524	0.09523809524	0.1111111111		0.6842105263	#DIV/0!
**NOS1**	0	0	0.05555555556		0	#DIV/0!
**NOS1AP**	0.1428571429	0.1428571429	0		inf	#DIV/0!
**NOS2**	0.04761904762	0.04761904762	0.05555555556		0.7	#DIV/0!
**NOS2P3**	0.09523809524	0.09523809524	0.05555555556		1.473684211	#DIV/0!
**NOS3**	0.09523809524	0.09523809524	0.05555555556		1.473684211	#DIV/0!
**NOSIP**	0.09523809524	0.09523809524	0.1111111111		0.6842105263	#DIV/0!
**IOSTRIN**	0.04761904762	0.04761904762	0.05555555556		0.7	#DIV/0!
**NOTCH1**	0.1428571429	0.1428571429	0.05555555556		2.333333333	#DIV/0!
**NOTCH2**	0.04761904762	0.04761904762	0.1111111111		0.325	#DIV/0!
**TCH2N**	0.1428571429	0.1428571429	0.05555555556		2.333333333	#DIV/0!
**TCH2N**	0.1428571429	0.1428571429	0.05555555556		2.333333333	#DIV/0!
**TCH2N**	0.1428571429	0.1428571429	0.05555555556		2.333333333	#DIV/0!
**TCH2N**	0.1428571429	0.1428571429	0.1111111111		1.083333333	#DIV/0!
**NOTCH3**	0.1428571429	0.1428571429	0.1111111111		1.083333333	#DIV/0!
**NOTCH4**	0.04761904762	0.04761904762	0		inf	#DIV/0!
**NOTO**	0	0	0.05555555556		0	#DIV/0!
**NOTUM**	0.04761904762	0.04761904762	0		inf	#DIV/0!
**NOX5**	0.04761904762	0.04761904762	0.05555555556		0.7	#DIV/0!
**NOXA1**	0.09523809524	0.09523809524	0		inf	#DIV/0!
**NOXO1**	0.09523809524	0.09523809524	0.2222222222		0.2894736842	#DIV/0!
**NPAP1L**	0.04761904762	0.04761904762	0		inf	#DIV/0!
**NPAS1**	0.09523809524	0.09523809524	0.1111111111		0.6842105263	#DIV/0!
**NPAS2**	0	0	0.05555555556		0	#DIV/0!
**NPB**	0.04761904762	0.04761904762	0		inf	#DIV/0!
**NPBWR1**	0.09523809524	0.09523809524	0.1111111111		0.6842105263	#DIV/0!
**NPBWR2**	0.1428571429	0.1428571429	0.2222222222		0.4583333333	#DIV/0!
**NPC1**	0.04761904762	0.04761904762	0.05555555556		0.7	#DIV/0!

**NPC1L1**	0.04761904762	0.04761904762	0.2222222222		0.1375	#DIV/0!
**NPDC1**	0.1428571429	0.1428571429	0.05555555556		2.333333333	#DIV/0!
**NPEPL1**	0.1428571429	0.1428571429	0.2222222222		0.4583333333	#DIV/0!
**NPEPPS**	0.1428571429	0.1428571429	0		inf	#DIV/0!
**NPFF**	0.04761904762	0.04761904762	0.05555555556		0.7	#DIV/0!
**NPFFR1**	0.09523809524	0.09523809524	0		inf	#DIV/0!
**NPFFR2**	0.04761904762	0.04761904762	0.05555555556		0.7	#DIV/0!
**NPHP1**	0	0	0.05555555556		0	#DIV/0!
**NPHP3**	0.1428571429	0.1428571429	0.05555555556		2.333333333	#DIV/0!
**IP3-ACA**	0.1428571429	0.1428571429	0.05555555556		2.333333333	#DIV/0!
**PHP3-AS**	0.1428571429	0.1428571429	0.05555555556		2.333333333	#DIV/0!
**NPHP4**	0	0	0.05555555556		0	#DIV/0!
**NPHS1**	0.04761904762	0.04761904762	0.05555555556		0.7	#DIV/0!
**NPHS2**	0.1428571429	0.1428571429	0		inf	#DIV/0!
**NPIPA1**	0.04761904762	0.04761904762	0		inf	#DIV/0!
**NPIPA2**	0.04761904762	0.04761904762	0		inf	#DIV/0!
**NPIPA3**	0.04761904762	0.04761904762	0		inf	#DIV/0!
**NPIPA5**	0.04761904762	0.04761904762	0		inf	#DIV/0!
**NPIPA7**	0.09523809524	0.09523809524	0		inf	#DIV/0!
**NPIPA8**	0.09523809524	0.09523809524	0		inf	#DIV/0!
**NPIPB11**	0.04761904762	0.04761904762	0		inf	#DIV/0!
**NPIPB12**	0.04761904762	0.04761904762	0		inf	#DIV/0!
**NPIPB13**	0.04761904762	0.04761904762	0		inf	#DIV/0!
**NPIPB2**	0.04761904762	0.04761904762	0		inf	#DIV/0!
**NPIPB3**	0.04761904762	0.04761904762	0		inf	#DIV/0!
**NPIPB4**	0.04761904762	0.04761904762	0		inf	#DIV/0!
**NPIPB5**	0.04761904762	0.04761904762	0		inf	#DIV/0!
**NPIPB6**	0.04761904762	0.04761904762	0		inf	#DIV/0!
**NPIPB8**	0.04761904762	0.04761904762	0		inf	#DIV/0!
**NPIPB9**	0.04761904762	0.04761904762	0		inf	#DIV/0!
**NPL**	0.1428571429	0.1428571429	0		inf	#DIV/0!
**NPLOC4**	0.04761904762	0.04761904762	0		inf	#DIV/0!
**NPM1**	0	0	0.05555555556		0	#DIV/0!
**NPM2**	0.09523809524	0.09523809524	0.1111111111		0.6842105263	#DIV/0!
**NPM3**	0.1428571429	0.1428571429	0		inf	#DIV/0!
**NPNT**	0	0	0.05555555556		0	#DIV/0!
**NPPA**	0.04761904762	0.04761904762	0.1111111111		0.325	#DIV/0!
**PPA-AS**	0.04761904762	0.04761904762	0.1111111111		0.325	#DIV/0!
**NPPB**	0.04761904762	0.04761904762	0.1111111111		0.325	#DIV/0!
**NPPC**	0	0	0.05555555556		0	#DIV/0!
**NPR1**	0.1904761905	0.1904761905	0.05555555556		3.294117647	#DIV/0!
**NPR2**	0.09523809524	0.09523809524	0.05555555556		1.473684211	#DIV/0!
**NPR3**	0.04761904762	0.04761904762	0.05555555556		0.7	#DIV/0!
**NPRL2**	0.09523809524	0.09523809524	0		inf	#DIV/0!
**NPRL3**	0.04761904762	0.04761904762	0.2222222222		0.1375	#DIV/0!
**NPS**	0.2380952381	0.2380952381	0.05555555556		4.375	#DIV/0!
**NPSR1**	0.04761904762	0.04761904762	0.1666666667		0.2	#DIV/0!
**PSR1-AS**	0.04761904762	0.04761904762	0.1666666667		0.2	#DIV/0!
**NPTN**	0.04761904762	0.04761904762	0.05555555556		0.7	#DIV/0!
**IPTN-IT**	0.04761904762	0.04761904762	0.05555555556		0.7	#DIV/0!
**NPTX1**	0.04761904762	0.04761904762	0		inf	#DIV/0!

**NPTX2**	0.04761904762	0.04761904762	0.05555555556		0.7	#DIV/0!
**NPTXR**	0.04761904762	0.04761904762	0.1111111111		0.325	#DIV/0!
**NPVF**	0.04761904762	0.04761904762	0.2222222222		0.1375	#DIV/0!
**NPW**	0.09523809524	0.09523809524	0.2222222222		0.2894736842	#DIV/0!
**NPY**	0.04761904762	0.04761904762	0.1666666667		0.2	#DIV/0!
**NPY1R**	0	0	0.05555555556		0	#DIV/0!
**NPY2R**	0	0	0.05555555556		0	#DIV/0!
**NPY4R**	0.1428571429	0.1428571429	0.05555555556		2.333333333	#DIV/0!
**NPY4R2**	0.1428571429	0.1428571429	0.05555555556		2.333333333	#DIV/0!
**NPY5R**	0	0	0.05555555556		0	#DIV/0!
**NPY6R**	0.04761904762	0.04761904762	0		inf	#DIV/0!
**NQO1**	0.1428571429	0.1428571429	0.05555555556		2.333333333	#DIV/0!
**NR0B2**	0.04761904762	0.04761904762	0.1111111111		0.325	#DIV/0!
**NR1D1**	0.09523809524	0.09523809524	0		inf	#DIV/0!
**NR1D2**	0.04761904762	0.04761904762	0		inf	#DIV/0!
**NR1H2**	0.09523809524	0.09523809524	0.1111111111		0.6842105263	#DIV/0!
**NR1H3**	0	0	0.05555555556		0	#DIV/0!
**NR1H4**	0.04761904762	0.04761904762	0.05555555556		0.7	#DIV/0!
**NR1I2**	0.09523809524	0.09523809524	0.05555555556		1.473684211	#DIV/0!
**NR1I3**	0.1428571429	0.1428571429	0		inf	#DIV/0!
**NR2C1**	0.04761904762	0.04761904762	0.05555555556		0.7	#DIV/0!
**NR2C2**	0.04761904762	0.04761904762	0		inf	#DIV/0!
**NR2C2A**	0.1428571429	0.1428571429	0.1111111111		1.083333333	#DIV/0!
**NR2E1**	0	0	0			#DIV/0!
**NR2E3**	0.04761904762	0.04761904762	0.05555555556		0.7	#DIV/0!
**NR2F1**	0.04761904762	0.04761904762	0		inf	#DIV/0!
**R2F1-AS**	0.04761904762	0.04761904762	0		inf	#DIV/0!
**NR2F2**	0.04761904762	0.04761904762	0.05555555556		0.7	#DIV/0!
**R2F2-AS**	0.04761904762	0.04761904762	0.05555555556		0.7	#DIV/0!
**NR2F6**	0.1428571429	0.1428571429	0.1111111111		1.083333333	#DIV/0!
**NR3C1**	0.04761904762	0.04761904762	0		inf	#DIV/0!
**NR3C2**	0	0	0.05555555556		0	#DIV/0!
**NR4A1**	0.04761904762	0.04761904762	0.05555555556		0.7	#DIV/0!
**NR4A2**	0	0	0.05555555556		0	#DIV/0!
**NR4A3**	0.04761904762	0.04761904762	0		inf	#DIV/0!
**NR5A1**	0.09523809524	0.09523809524	0.05555555556		1.473684211	#DIV/0!
**NR5A2**	0.1428571429	0.1428571429	0		inf	#DIV/0!
**NR6A1**	0.09523809524	0.09523809524	0.05555555556		1.473684211	#DIV/0!
**NRAP**	0.1428571429	0.1428571429	0.05555555556		2.333333333	#DIV/0!
**NRARP**	0.1428571429	0.1428571429	0.05555555556		2.333333333	#DIV/0!
**NRAS**	0.04761904762	0.04761904762	0.05555555556		0.7	#DIV/0!
**NRAV**	0	0	0.1111111111		0	#DIV/0!
**NRBF2**	0.09523809524	0.09523809524	0.1111111111		0.6842105263	#DIV/0!
**NRBP2**	0.04761904762	0.04761904762	0.1666666667		0.2	#DIV/0!
**NRCAM**	0.04761904762	0.04761904762	0.05555555556		0.7	#DIV/0!
**NRDC**	0.04761904762	0.04761904762	0.05555555556		0.7	#DIV/0!
**NREP**	0.04761904762	0.04761904762	0		inf	#DIV/0!
**REP-AS**	0.04761904762	0.04761904762	0		inf	#DIV/0!
**NRF1**	0.04761904762	0.04761904762	0.05555555556		0.7	#DIV/0!
**NRG1**	0.04761904762	0.04761904762	0.1111111111		0.325	#DIV/0!
**IRG1-IT**	0.04761904762	0.04761904762	0.1111111111		0.325	#DIV/0!

RG1-IT	0.04761904762	0.04761904762	0.1111111111		0.325	#DIV/0!
NRG2	0.04761904762	0.04761904762	0		inf	#DIV/0!
NRG3	0.1428571429	0.1428571429	0		inf	#DIV/0!
RG3-AS	0.1428571429	0.1428571429	0		inf	#DIV/0!
NRG4	0.04761904762	0.04761904762	0.05555555556		0.7	#DIV/0!
NRIP1	0.04761904762	0.04761904762	0.1111111111		0.325	#DIV/0!
NRIP2	0.04761904762	0.04761904762	0.1666666667		0.2	#DIV/0!
NRIR	0	0	0.05555555556		0	#DIV/0!
NRN1L	0.1428571429	0.1428571429	0.05555555556		2.333333333	#DIV/0!
NRON	0.09523809524	0.09523809524	0.05555555556		1.473684211	#DIV/0!
NRP1	0.09523809524	0.09523809524	0.2222222222		0.2894736842	#DIV/0!
NRP2	0	0	0.05555555556		0	#DIV/0!
NRROS	0.1428571429	0.1428571429	0.05555555556		2.333333333	#DIV/0!
NRSN2	0.1904761905	0.1904761905	0.05555555556		3.294117647	#DIV/0!
RSN2-A	0.1904761905	0.1904761905	0.05555555556		3.294117647	#DIV/0!
NRTN	0.1428571429	0.1428571429	0.05555555556		2.333333333	#DIV/0!
NRXN1	0.04761904762	0.04761904762	0.05555555556		0.7	#DIV/0!
NSA2	0.04761904762	0.04761904762	0		inf	#DIV/0!
NSD1	0.09523809524	0.09523809524	0.05555555556		1.473684211	#DIV/0!
NSD2	0.04761904762	0.04761904762	0		inf	#DIV/0!
NSD3	0.09523809524	0.09523809524	0.1111111111		0.6842105263	#DIV/0!
NSF	0.2380952381	0.2380952381	0		inf	#DIV/0!
NSFL1C	0.1904761905	0.1904761905	0.1111111111		1.529411765	#DIV/0!
NSFP1	0.2380952381	0.2380952381	0		inf	#DIV/0!
NSG2	0	0	0.05555555556		0	#DIV/0!
NSL1	0.09523809524	0.09523809524	0		inf	#DIV/0!
NSMAF	0.1428571429	0.1428571429	0.1666666667		0.6666666667	#DIV/0!
NSMCE1	0.04761904762	0.04761904762	0		inf	#DIV/0!
MCE1-I	0.04761904762	0.04761904762	0		inf	#DIV/0!
NSMCE2	0.1428571429	0.1428571429	0.1111111111		1.083333333	#DIV/0!
SMCE4	0.2380952381	0.2380952381	0.05555555556		4.375	#DIV/0!
NSMF	0.09523809524	0.09523809524	0		inf	#DIV/0!
NSRP1	0	0	0.05555555556		0	#DIV/0!
NSUN2	0	0	0.05555555556		0	#DIV/0!
NSUN3	0.04761904762	0.04761904762	0.05555555556		0.7	#DIV/0!
NSUN4	0.04761904762	0.04761904762	0.05555555556		0.7	#DIV/0!
NSUN5	0.1428571429	0.1428571429	0.1111111111		1.083333333	#DIV/0!
NSUN5P	0.1428571429	0.1428571429	0.1666666667		0.6666666667	#DIV/0!
NSUN5P	0.1428571429	0.1428571429	0.1111111111		1.083333333	#DIV/0!
NSUN6	0.09523809524	0.09523809524	0.1111111111		0.6842105263	#DIV/0!
NSUN7	0.1904761905	0.1904761905	0.05555555556		3.294117647	#DIV/0!
NT5C	0.04761904762	0.04761904762	0.05555555556		0.7	#DIV/0!
NT5C1A	0.04761904762	0.04761904762	0.05555555556		0.7	#DIV/0!
NT5C1B	0	0	0.05555555556		0	#DIV/0!
C1B-RD	0	0	0.05555555556		0	#DIV/0!
NT5C2	0.1428571429	0.1428571429	0.05555555556		2.333333333	#DIV/0!
NT5C3A	0.04761904762	0.04761904762	0.1666666667		0.2	#DIV/0!
NT5C3B	0.09523809524	0.09523809524	0		inf	#DIV/0!
NT5DC2	0.04761904762	0.04761904762	0		inf	#DIV/0!
NT5DC3	0.04761904762	0.04761904762	0.05555555556		0.7	#DIV/0!
NT5DC4	0	0	0.05555555556		0	#DIV/0!

**NT5M**	0.04761904762	0.04761904762	0.1111111111		0.325	#DIV/0!
**NTAN1**	0.04761904762	0.04761904762	0		inf	#DIV/0!
**NTF3**	0.04761904762	0.04761904762	0.1111111111		0.325	#DIV/0!
**NTF4**	0.09523809524	0.09523809524	0.1111111111		0.6842105263	#DIV/0!
**NTHL1**	0.09523809524	0.09523809524	0.2222222222		0.2894736842	#DIV/0!
**NTMT1**	0.09523809524	0.09523809524	0.05555555556		1.473684211	#DIV/0!
**NTN1**	0.04761904762	0.04761904762	0.05555555556		0.7	#DIV/0!
**NTN3**	0.09523809524	0.09523809524	0.2222222222		0.2894736842	#DIV/0!
**NTN4**	0.04761904762	0.04761904762	0.05555555556		0.7	#DIV/0!
**NTN5**	0.09523809524	0.09523809524	0.1111111111		0.6842105263	#DIV/0!
**NTNG1**	0.04761904762	0.04761904762	0.05555555556		0.7	#DIV/0!
**NTNG2**	0.09523809524	0.09523809524	0		inf	#DIV/0!
**NTPCR**	0.1428571429	0.1428571429	0		inf	#DIV/0!
**NTRK1**	0.1428571429	0.1428571429	0.05555555556		2.333333333	#DIV/0!
**NTRK2**	0	0	0.05555555556		0	#DIV/0!
**NTRK3**	0.04761904762	0.04761904762	0.05555555556		0.7	#DIV/0!
**TRK3-A**	0.04761904762	0.04761904762	0.05555555556		0.7	#DIV/0!
**NTS**	0.04761904762	0.04761904762	0.05555555556		0.7	#DIV/0!
**NTSR1**	0.1428571429	0.1428571429	0.2222222222		0.4583333333	#DIV/0!
**NTSR2**	0	0	0.05555555556		0	#DIV/0!
**NUAK1**	0.04761904762	0.04761904762	0.05555555556		0.7	#DIV/0!
**NUAK2**	0.1904761905	0.1904761905	0		inf	#DIV/0!
**NUB1**	0.09523809524	0.09523809524	0.05555555556		1.473684211	#DIV/0!
**NUBP1**	0.04761904762	0.04761904762	0		inf	#DIV/0!
**NUBP2**	0.09523809524	0.09523809524	0.2222222222		0.2894736842	#DIV/0!
**NUBPL**	0	0	0			#DIV/0!
**NUCB1**	0.09523809524	0.09523809524	0.1111111111		0.6842105263	#DIV/0!
**UCB1-A**	0.09523809524	0.09523809524	0.1111111111		0.6842105263	#DIV/0!
**NUCKS1**	0.1428571429	0.1428571429	0		inf	#DIV/0!
**NUDC**	0.04761904762	0.04761904762	0.1111111111		0.325	#DIV/0!
**NUDCD1**	0.1904761905	0.1904761905	0.1111111111		1.529411765	#DIV/0!
**NUDCD2**	0	0	0.05555555556		0	#DIV/0!
**NUDCD3**	0.04761904762	0.04761904762	0.2222222222		0.1375	#DIV/0!
**NUDT1**	0.04761904762	0.04761904762	0.1666666667		0.2	#DIV/0!
**NUDT12**	0.04761904762	0.04761904762	0		inf	#DIV/0!
**NUDT13**	0.1904761905	0.1904761905	0.05555555556		3.294117647	#DIV/0!
**NUDT16**	0.09523809524	0.09523809524	0.05555555556		1.473684211	#DIV/0!
**UDT16L**	0.04761904762	0.04761904762	0		inf	#DIV/0!
**UDT16P**	0.09523809524	0.09523809524	0.05555555556		1.473684211	#DIV/0!
**NUDT17**	0.1428571429	0.1428571429	0.05555555556		2.333333333	#DIV/0!
**NUDT18**	0.09523809524	0.09523809524	0.1111111111		0.6842105263	#DIV/0!
**NUDT19**	0.04761904762	0.04761904762	0.05555555556		0.7	#DIV/0!
**NUDT2**	0.09523809524	0.09523809524	0.05555555556		1.473684211	#DIV/0!
**NUDT21**	0.04761904762	0.04761904762	0		inf	#DIV/0!
**NUDT3**	0.04761904762	0.04761904762	0		inf	#DIV/0!
**NUDT4**	0.04761904762	0.04761904762	0.05555555556		0.7	#DIV/0!
**NUDT4B**	0.04761904762	0.04761904762	0.05555555556		0.7	#DIV/0!
**NUDT4P**	0.04761904762	0.04761904762	0.05555555556		0.7	#DIV/0!
**NUDT5**	0.09523809524	0.09523809524	0.1111111111		0.6842105263	#DIV/0!
**NUDT6**	0	0	0.05555555556		0	#DIV/0!
**NUDT7**	0.09523809524	0.09523809524	0		inf	#DIV/0!

NUDT9	0	0	0.05555555556		0	#DIV/0!
NUDT9P	0.1428571429	0.1428571429	0.1111111111		1.083333333	#DIV/0!
NUF2	0.1428571429	0.1428571429	0		inf	#DIV/0!
NUFIP2	0.04761904762	0.04761904762	0.05555555556		0.7	#DIV/0!
NUGGC	0.04761904762	0.04761904762	0.1111111111		0.325	#DIV/0!
NUMB	0.09523809524	0.09523809524	0		inf	#DIV/0!
NUMBL	0.04761904762	0.04761904762	0.05555555556		0.7	#DIV/0!
NUP107	0.09523809524	0.09523809524	0.05555555556		1.473684211	#DIV/0!
NUP133	0.1428571429	0.1428571429	0		inf	#DIV/0!
NUP155	0.09523809524	0.09523809524	0.05555555556		1.473684211	#DIV/0!
NUP160	0	0	0.05555555556		0	#DIV/0!
NUP205	0.04761904762	0.04761904762	0.05555555556		0.7	#DIV/0!
NUP210	0.04761904762	0.04761904762	0		inf	#DIV/0!
NUP210L	0.1904761905	0.1904761905	0.05555555556		3.294117647	#DIV/0!
NUP210P	0.04761904762	0.04761904762	0.05555555556		0.7	#DIV/0!
NUP214	0.09523809524	0.09523809524	0.05555555556		1.473684211	#DIV/0!
NUP35	0	0	0.05555555556		0	#DIV/0!
NUP37	0.04761904762	0.04761904762	0.05555555556		0.7	#DIV/0!
NUP43	0	0	0			#DIV/0!
NUP50	0	0	0.1111111111		0	#DIV/0!
NUP50-D	0	0	0.1111111111		0	#DIV/0!
NUP54	0.04761904762	0.04761904762	0.05555555556		0.7	#DIV/0!
NUP62	0.09523809524	0.09523809524	0.1111111111		0.6842105263	#DIV/0!
NUP85	0.04761904762	0.04761904762	0.05555555556		0.7	#DIV/0!
NUP88	0	0	0.1111111111		0	#DIV/0!
NUP93	0.04761904762	0.04761904762	0		inf	#DIV/0!
NUP98	0.04761904762	0.04761904762	0		inf	#DIV/0!
NUPL2	0.04761904762	0.04761904762	0.1666666667		0.2	#DIV/0!
NUPR1	0.04761904762	0.04761904762	0		inf	#DIV/0!
NUPR2	0.2380952381	0.2380952381	0.1666666667		1.25	#DIV/0!
NUTF2	0.1428571429	0.1428571429	0.05555555556		2.333333333	#DIV/0!
NUTM2A	0.1428571429	0.1428571429	0.1111111111		1.083333333	#DIV/0!
TM2A-A	0.1428571429	0.1428571429	0.1111111111		1.083333333	#DIV/0!
NUTM2B	0.1428571429	0.1428571429	0		inf	#DIV/0!
TM2B-A	0.1428571429	0.1428571429	0.05555555556		2.333333333	#DIV/0!
NUTM2D	0.1428571429	0.1428571429	0.1111111111		1.083333333	#DIV/0!
NUTM2E	0.1428571429	0.1428571429	0		inf	#DIV/0!
NUTM2F	0.04761904762	0.04761904762	0.05555555556		0.7	#DIV/0!
NUTM2G	0.04761904762	0.04761904762	0		inf	#DIV/0!
NVL	0.1428571429	0.1428571429	0.05555555556		2.333333333	#DIV/0!
NWD1	0.1428571429	0.1428571429	0.1111111111		1.083333333	#DIV/0!
NXN	0	0	0.1111111111		0	#DIV/0!
NXNL2	0	0	0.05555555556		0	#DIV/0!
NXPE3	0.09523809524	0.09523809524	0.05555555556		1.473684211	#DIV/0!
NXPH1	0.04761904762	0.04761904762	0.1666666667		0.2	#DIV/0!
NXPH2	0	0	0.05555555556		0	#DIV/0!
NXPH3	0.09523809524	0.09523809524	0		inf	#DIV/0!
NXPH4	0.1428571429	0.1428571429	0.05555555556		2.333333333	#DIV/0!
NXT1	0.1904761905	0.1904761905	0.05555555556		3.294117647	#DIV/0!
NYAP1	0.04761904762	0.04761904762	0.05555555556		0.7	#DIV/0!
NYAP2	0	0	0.05555555556		0	#DIV/0!

OACYLI	0.09523809524	0.09523809524	0		inf	#DIV/0!
OARD1	0	0	0.05555555556		0	#DIV/0!
OAS1	0	0	0.05555555556		0	#DIV/0!
OAS2	0	0	0.05555555556		0	#DIV/0!
OAS3	0	0	0.05555555556		0	#DIV/0!
OASL	0.04761904762	0.04761904762	0.1111111111		0.325	#DIV/0!
OAT	0.2380952381	0.2380952381	0.05555555556		4.375	#DIV/0!
OAZ1	0.09523809524	0.09523809524	0.1111111111		0.6842105263	#DIV/0!
OAZ2	0.04761904762	0.04761904762	0.05555555556		0.7	#DIV/0!
OAZ3	0.1904761905	0.1904761905	0		inf	#DIV/0!
OBP2A	0.04761904762	0.04761904762	0		inf	#DIV/0!
OBP2B	0.04761904762	0.04761904762	0		inf	#DIV/0!
OBSCN	0.1428571429	0.1428571429	0		inf	#DIV/0!
BSCN-A	0.1428571429	0.1428571429	0		inf	#DIV/0!
OBSL1	0	0	0.05555555556		0	#DIV/0!
OC90	0.04761904762	0.04761904762	0.1111111111		0.325	#DIV/0!
OCEL1	0.1428571429	0.1428571429	0.1111111111		1.083333333	#DIV/0!
OCIAD1	0.09523809524	0.09523809524	0.1111111111		0.6842105263	#DIV/0!
CIAD1-A	0.09523809524	0.09523809524	0.1111111111		0.6842105263	#DIV/0!
OCIAD2	0.09523809524	0.09523809524	0.1111111111		0.6842105263	#DIV/0!
OCLM	0.1428571429	0.1428571429	0.05555555556		2.333333333	#DIV/0!
OCLN	0.09523809524	0.09523809524	0.1666666667		0.4210526316	#DIV/0!
OCLNP1	0.09523809524	0.09523809524	0.1666666667		0.4210526316	#DIV/0!
OCM	0.04761904762	0.04761904762	0.1666666667		0.2	#DIV/0!
OCM2	0.04761904762	0.04761904762	0.05555555556		0.7	#DIV/0!
OCSTAM	0.1428571429	0.1428571429	0.1666666667		0.6666666667	#DIV/0!
ODAM	0.04761904762	0.04761904762	0.05555555556		0.7	#DIV/0!
ODAPH	0.04761904762	0.04761904762	0.05555555556		0.7	#DIV/0!
ODC1	0	0	0.05555555556		0	#DIV/0!
ODC1-D	0	0	0.05555555556		0	#DIV/0!
ODF1	0.1904761905	0.1904761905	0.1111111111		1.529411765	#DIV/0!
ODF2L	0.04761904762	0.04761904762	0.05555555556		0.7	#DIV/0!
ODF3	0.04761904762	0.04761904762	0		inf	#DIV/0!
ODF3B	0	0	0.1111111111		0	#DIV/0!
ODF3L1	0.04761904762	0.04761904762	0.05555555556		0.7	#DIV/0!
ODF3L2	0.09523809524	0.09523809524	0.1111111111		0.6842105263	#DIV/0!
ODF4	0.04761904762	0.04761904762	0.05555555556		0.7	#DIV/0!
ODR4	0.1428571429	0.1428571429	0.05555555556		2.333333333	#DIV/0!
OGA	0.1428571429	0.1428571429	0		inf	#DIV/0!
OGDH	0.04761904762	0.04761904762	0.2222222222		0.1375	#DIV/0!
OGDHL	0.04761904762	0.04761904762	0.05555555556		0.7	#DIV/0!
OGFOD1	0.04761904762	0.04761904762	0		inf	#DIV/0!
OGFOD2	0.04761904762	0.04761904762	0.1111111111		0.325	#DIV/0!
OGFOD3	0.04761904762	0.04761904762	0		inf	#DIV/0!
OGFR	0.1428571429	0.1428571429	0.2222222222		0.4583333333	#DIV/0!
GFR-AS	0.1428571429	0.1428571429	0.2222222222		0.4583333333	#DIV/0!
OGFRP1	0	0	0.1111111111		0	#DIV/0!
OGG1	0.09523809524	0.09523809524	0		inf	#DIV/0!
OGN	0.04761904762	0.04761904762	0.05555555556		0.7	#DIV/0!
OIT3	0.1904761905	0.1904761905	0.05555555556		3.294117647	#DIV/0!
OLA1	0	0	0.1111111111		0	#DIV/0!

**OLAH**	0.09523809524	0.09523809524	0.1111111111		0.6842105263	#DIV/0!
**OLFM1**	0.04761904762	0.04761904762	0		inf	#DIV/0!
**OLFM2**	0.1428571429	0.1428571429	0.1111111111		1.083333333	#DIV/0!
**OLFM3**	0.04761904762	0.04761904762	0.05555555556		0.7	#DIV/0!
**)LFM5I**	0.04761904762	0.04761904762	0		inf	#DIV/0!
**)LFML1**	0.04761904762	0.04761904762	0		inf	#DIV/0!
**)LFML2**	0.09523809524	0.09523809524	0.05555555556		1.473684211	#DIV/0!
**)LFML2**	0.1428571429	0.1428571429	0		inf	#DIV/0!
**)LFML3**	0.04761904762	0.04761904762	0.05555555556		0.7	#DIV/0!
**OLIG1**	0.04761904762	0.04761904762	0.1111111111		0.325	#DIV/0!
**OLIG2**	0.04761904762	0.04761904762	0.1111111111		0.325	#DIV/0!
**LMALIN**	0.1428571429	0.1428571429	0		inf	#DIV/0!
**OLR1**	0.04761904762	0.04761904762	0.1111111111		0.325	#DIV/0!
**OMA1**	0.04761904762	0.04761904762	0.05555555556		0.7	#DIV/0!
**OMD**	0.04761904762	0.04761904762	0.05555555556		0.7	#DIV/0!
**OMG**	0	0	0			#DIV/0!
**)NECUT**	0.04761904762	0.04761904762	0.05555555556		0.7	#DIV/0!
**)NECUT**	0.09523809524	0.09523809524	0		inf	#DIV/0!
**)NECUT**	0.09523809524	0.09523809524	0.1111111111		0.6842105263	#DIV/0!
**OOEP**	0.04761904762	0.04761904762	0		inf	#DIV/0!
**OPA1**	0.09523809524	0.09523809524	0.05555555556		1.473684211	#DIV/0!
**)PA1-AS**	0.09523809524	0.09523809524	0.05555555556		1.473684211	#DIV/0!
**OPA3**	0.09523809524	0.09523809524	0.1111111111		0.6842105263	#DIV/0!
**OPALIN**	0.1428571429	0.1428571429	0		inf	#DIV/0!
**OPLAH**	0.04761904762	0.04761904762	0.1666666667		0.2	#DIV/0!
**)PN1SW**	0.04761904762	0.04761904762	0.05555555556		0.7	#DIV/0!
**OPN3**	0.1428571429	0.1428571429	0		inf	#DIV/0!
**OPN4**	0.1428571429	0.1428571429	0.05555555556		2.333333333	#DIV/0!
**OPN5**	0.04761904762	0.04761904762	0.05555555556		0.7	#DIV/0!
**OPRD1**	0.04761904762	0.04761904762	0.1111111111		0.325	#DIV/0!
**OPRK1**	0.09523809524	0.09523809524	0.1111111111		0.6842105263	#DIV/0!
**OPRL1**	0.1428571429	0.1428571429	0.2222222222		0.4583333333	#DIV/0!
**OPRPN**	0.04761904762	0.04761904762	0.05555555556		0.7	#DIV/0!
**OPTC**	0.1904761905	0.1904761905	0		inf	#DIV/0!
**OPTN**	0.09523809524	0.09523809524	0.1111111111		0.6842105263	#DIV/0!
**OR10A2**	0.04761904762	0.04761904762	0		inf	#DIV/0!
**OR10A4**	0.04761904762	0.04761904762	0		inf	#DIV/0!
**OR10A5**	0.04761904762	0.04761904762	0		inf	#DIV/0!
**OR10A7**	0.04761904762	0.04761904762	0.05555555556		0.7	#DIV/0!
**)R10AC**	0.04761904762	0.04761904762	0.05555555556		0.7	#DIV/0!
**)R10AD**	0.04761904762	0.04761904762	0.05555555556		0.7	#DIV/0!
**OR10H1**	0.1428571429	0.1428571429	0.1111111111		1.083333333	#DIV/0!
**OR10H2**	0.1428571429	0.1428571429	0.1111111111		1.083333333	#DIV/0!
**OR10H3**	0.1428571429	0.1428571429	0.1111111111		1.083333333	#DIV/0!
**OR10H4**	0.1428571429	0.1428571429	0.1111111111		1.083333333	#DIV/0!
**OR10H5**	0.1428571429	0.1428571429	0.1111111111		1.083333333	#DIV/0!
**OR10J1**	0.1428571429	0.1428571429	0		inf	#DIV/0!
**OR10J3**	0.1428571429	0.1428571429	0		inf	#DIV/0!
**OR10J4**	0.1428571429	0.1428571429	0		inf	#DIV/0!
**OR10J5**	0.1428571429	0.1428571429	0		inf	#DIV/0!
**OR10K1**	0.1428571429	0.1428571429	0.05555555556		2.333333333	#DIV/0!

OR10K2	0.1428571429	0.1428571429	0.05555555556		2.333333333	#DIV/0!
OR10P1	0.04761904762	0.04761904762	0.05555555556		0.7	#DIV/0!
OR10R2	0.1428571429	0.1428571429	0.05555555556		2.333333333	#DIV/0!
OR10T2	0.1428571429	0.1428571429	0.05555555556		2.333333333	#DIV/0!
OR10X1	0.1428571429	0.1428571429	0.05555555556		2.333333333	#DIV/0!
OR10Z1	0.1428571429	0.1428571429	0.05555555556		2.333333333	#DIV/0!
OR11G2	0.04761904762	0.04761904762	0		inf	#DIV/0!
OR11H1	0.09523809524	0.09523809524	0.05555555556		1.473684211	#DIV/0!
OR11H1[illegible]	0.1428571429	0.1428571429	0.1111111111		1.083333333	#DIV/0!
OR11H2	0.1428571429	0.1428571429	0.1111111111		1.083333333	#DIV/0!
OR11H4	0.04761904762	0.04761904762	0		inf	#DIV/0!
OR11H6	0.04761904762	0.04761904762	0		inf	#DIV/0!
OR11H7	0.04761904762	0.04761904762	0		inf	#DIV/0!
OR11L1	0.1428571429	0.1428571429	0		inf	#DIV/0!
OR13A1	0.1428571429	0.1428571429	0.05555555556		2.333333333	#DIV/0!
OR13C2	0.04761904762	0.04761904762	0		inf	#DIV/0!
OR13C3	0.04761904762	0.04761904762	0		inf	#DIV/0!
OR13C4	0.04761904762	0.04761904762	0		inf	#DIV/0!
OR13C5	0.04761904762	0.04761904762	0		inf	#DIV/0!
OR13C8	0.04761904762	0.04761904762	0		inf	#DIV/0!
OR13C9	0.04761904762	0.04761904762	0		inf	#DIV/0!
OR13D1	0.04761904762	0.04761904762	0		inf	#DIV/0!
OR13F1	0.04761904762	0.04761904762	0		inf	#DIV/0!
OR13G1	0.1428571429	0.1428571429	0		inf	#DIV/0!
OR13J1	0.09523809524	0.09523809524	0.05555555556		1.473684211	#DIV/0!
OR14A1[illegible]	0.1428571429	0.1428571429	0		inf	#DIV/0!
OR14A2	0.1428571429	0.1428571429	0		inf	#DIV/0!
OR14C3[illegible]	0.1428571429	0.1428571429	0		inf	#DIV/0!
OR14I1	0.1428571429	0.1428571429	0		inf	#DIV/0!
OR14K1	0.1428571429	0.1428571429	0		inf	#DIV/0!
OR1A1	0	0	0.1111111111		0	#DIV/0!
OR1A2	0	0	0.1111111111		0	#DIV/0!
OR1B1	0.09523809524	0.09523809524	0.05555555556		1.473684211	#DIV/0!
OR1C1	0.1428571429	0.1428571429	0		inf	#DIV/0!
OR1D2	0	0	0.1111111111		0	#DIV/0!
OR1D4	0	0	0.1111111111		0	#DIV/0!
OR1D5	0	0	0.1111111111		0	#DIV/0!
OR1E1	0	0	0.1111111111		0	#DIV/0!
OR1E2	0	0	0.1111111111		0	#DIV/0!
OR1E3	0	0	0.1111111111		0	#DIV/0!
OR1F1	0.04761904762	0.04761904762	0.1111111111		0.325	#DIV/0!
OR1F2P	0.04761904762	0.04761904762	0.1111111111		0.325	#DIV/0!
OR1G1	0	0	0.1111111111		0	#DIV/0!
OR1I1	0.1428571429	0.1428571429	0.1111111111		1.083333333	#DIV/0!
OR1J1	0.09523809524	0.09523809524	0.05555555556		1.473684211	#DIV/0!
OR1J2	0.09523809524	0.09523809524	0.05555555556		1.473684211	#DIV/0!
OR1J4	0.09523809524	0.09523809524	0.05555555556		1.473684211	#DIV/0!
OR1K1	0.09523809524	0.09523809524	0.05555555556		1.473684211	#DIV/0!
OR1L1	0.09523809524	0.09523809524	0.05555555556		1.473684211	#DIV/0!
OR1L3	0.09523809524	0.09523809524	0.05555555556		1.473684211	#DIV/0!
OR1L4	0.09523809524	0.09523809524	0.05555555556		1.473684211	#DIV/0!

**OR1L6**	0.09523809524	0.09523809524	0.05555555556		1.473684211	#DIV/0!
**OR1L8**	0.09523809524	0.09523809524	0.05555555556		1.473684211	#DIV/0!
**OR1M1**	0.1428571429	0.1428571429	0.05555555556		2.333333333	#DIV/0!
**OR1N1**	0.09523809524	0.09523809524	0.05555555556		1.473684211	#DIV/0!
**OR1N2**	0.09523809524	0.09523809524	0.05555555556		1.473684211	#DIV/0!
**OR1Q1**	0.09523809524	0.09523809524	0.05555555556		1.473684211	#DIV/0!
**OR2A1**	0.09523809524	0.09523809524	0.05555555556		1.473684211	#DIV/0!
**R2A1-AS**	0.09523809524	0.09523809524	0.05555555556		1.473684211	#DIV/0!
**OR2A12**	0.04761904762	0.04761904762	0.05555555556		0.7	#DIV/0!
**OR2A14**	0.04761904762	0.04761904762	0.05555555556		0.7	#DIV/0!
**OR2A2**	0.04761904762	0.04761904762	0.05555555556		0.7	#DIV/0!
**)R2A20I**	0.09523809524	0.09523809524	0.05555555556		1.473684211	#DIV/0!
**OR2A25**	0.04761904762	0.04761904762	0.05555555556		0.7	#DIV/0!
**OR2A42**	0.09523809524	0.09523809524	0.05555555556		1.473684211	#DIV/0!
**OR2A5**	0.04761904762	0.04761904762	0.05555555556		0.7	#DIV/0!
**OR2A7**	0.09523809524	0.09523809524	0.05555555556		1.473684211	#DIV/0!
**OR2A9P**	0.09523809524	0.09523809524	0.05555555556		1.473684211	#DIV/0!
**OR2AE1**	0.04761904762	0.04761904762	0.05555555556		0.7	#DIV/0!
**)R2AG1**	0.04761904762	0.04761904762	0		inf	#DIV/0!
**)R2AG2**	0.04761904762	0.04761904762	0		inf	#DIV/0!
**OR2AJ1**	0.1428571429	0.1428571429	0		inf	#DIV/0!
**)R2AK2**	0.1428571429	0.1428571429	0		inf	#DIV/0!
**OR2AP1**	0.04761904762	0.04761904762	0.05555555556		0.7	#DIV/0!
**OR2B11**	0.1428571429	0.1428571429	0		inf	#DIV/0!
**OR2C1**	0.04761904762	0.04761904762	0.1111111111		0.325	#DIV/0!
**OR2C3**	0.1428571429	0.1428571429	0		inf	#DIV/0!
**OR2D2**	0.04761904762	0.04761904762	0		inf	#DIV/0!
**OR2D3**	0.04761904762	0.04761904762	0		inf	#DIV/0!
**OR2F1**	0.04761904762	0.04761904762	0.05555555556		0.7	#DIV/0!
**OR2F2**	0.04761904762	0.04761904762	0.05555555556		0.7	#DIV/0!
**OR2G2**	0.1428571429	0.1428571429	0		inf	#DIV/0!
**OR2G3**	0.1428571429	0.1428571429	0		inf	#DIV/0!
**OR2G6**	0.1428571429	0.1428571429	0		inf	#DIV/0!
**OR2K2**	0.04761904762	0.04761904762	0.05555555556		0.7	#DIV/0!
**OR2L13**	0.1428571429	0.1428571429	0		inf	#DIV/0!
**OR2L1P**	0.1428571429	0.1428571429	0		inf	#DIV/0!
**OR2L2**	0.1428571429	0.1428571429	0		inf	#DIV/0!
**OR2L3**	0.1428571429	0.1428571429	0		inf	#DIV/0!
**OR2L5**	0.1428571429	0.1428571429	0		inf	#DIV/0!
**OR2L8**	0.1428571429	0.1428571429	0		inf	#DIV/0!
**)R2M1P**	0.1428571429	0.1428571429	0		inf	#DIV/0!
**OR2M2**	0.1428571429	0.1428571429	0		inf	#DIV/0!
**OR2M3**	0.1428571429	0.1428571429	0		inf	#DIV/0!
**OR2M4**	0.1428571429	0.1428571429	0		inf	#DIV/0!
**OR2M5**	0.1428571429	0.1428571429	0		inf	#DIV/0!
**OR2M7**	0.1428571429	0.1428571429	0		inf	#DIV/0!
**OR2S2**	0.09523809524	0.09523809524	0.05555555556		1.473684211	#DIV/0!
**OR2T1**	0.1428571429	0.1428571429	0		inf	#DIV/0!
**OR2T10**	0.1428571429	0.1428571429	0		inf	#DIV/0!
**OR2T11**	0.1428571429	0.1428571429	0		inf	#DIV/0!
**OR2T12**	0.1428571429	0.1428571429	0		inf	#DIV/0!

**OR2T2**	0.1428571429	0.1428571429	0		inf	#DIV/0!
**OR2T27**	0.1428571429	0.1428571429	0		inf	#DIV/0!
**OR2T29**	0.1428571429	0.1428571429	0		inf	#DIV/0!
**OR2T3**	0.1428571429	0.1428571429	0		inf	#DIV/0!
**OR2T33**	0.1428571429	0.1428571429	0		inf	#DIV/0!
**OR2T34**	0.1428571429	0.1428571429	0		inf	#DIV/0!
**OR2T35**	0.1428571429	0.1428571429	0		inf	#DIV/0!
**OR2T4**	0.1428571429	0.1428571429	0		inf	#DIV/0!
**OR2T5**	0.1428571429	0.1428571429	0		inf	#DIV/0!
**OR2T6**	0.1428571429	0.1428571429	0		inf	#DIV/0!
**OR2T7**	0.1428571429	0.1428571429	0		inf	#DIV/0!
**OR2T8**	0.1428571429	0.1428571429	0		inf	#DIV/0!
**OR2V1**	0.09523809524	0.09523809524	0.05555555556		1.473684211	#DIV/0!
**OR2V2**	0.09523809524	0.09523809524	0.05555555556		1.473684211	#DIV/0!
**OR2W3**	0.1428571429	0.1428571429	0		inf	#DIV/0!
**OR2W5**	0.1428571429	0.1428571429	0		inf	#DIV/0!
**OR2Y1**	0.09523809524	0.09523809524	0.05555555556		1.473684211	#DIV/0!
**OR2Z1**	0.1428571429	0.1428571429	0.05555555556		2.333333333	#DIV/0!
**OR3A1**	0	0	0.1111111111		0	#DIV/0!
**OR3A2**	0	0	0.1111111111		0	#DIV/0!
**OR3A3**	0	0	0.1111111111		0	#DIV/0!
**OR3A4P**	0	0	0.1111111111		0	#DIV/0!
**OR4A15**	0.04761904762	0.04761904762	0		inf	#DIV/0!
**OR4A16**	0.04761904762	0.04761904762	0		inf	#DIV/0!
**OR4A47**	0	0	0.05555555556		0	#DIV/0!
**OR4A5**	0.09523809524	0.09523809524	0		inf	#DIV/0!
**OR4B1**	0	0	0.05555555556		0	#DIV/0!
**OR4C11**	0.04761904762	0.04761904762	0		inf	#DIV/0!
**OR4C15**	0.04761904762	0.04761904762	0		inf	#DIV/0!
**OR4C16**	0.04761904762	0.04761904762	0		inf	#DIV/0!
**OR4C3**	0	0	0.05555555556		0	#DIV/0!
**OR4C45**	0	0	0.05555555556		0	#DIV/0!
**OR4C46**	0.09523809524	0.09523809524	0		inf	#DIV/0!
**OR4C5**	0	0	0.05555555556		0	#DIV/0!
**OR4D1**	0.09523809524	0.09523809524	0		inf	#DIV/0!
**OR4D2**	0.09523809524	0.09523809524	0		inf	#DIV/0!
**OR4F13P**	0.04761904762	0.04761904762	0.05555555556		0.7	#DIV/0!
**OR4F15**	0.04761904762	0.04761904762	0.05555555556		0.7	#DIV/0!
**OR4F16**	0.09523809524	0.09523809524	0.1666666667		0.4210526316	#DIV/0!
**OR4F17**	0.09523809524	0.09523809524	0.1111111111		0.6842105263	#DIV/0!
**OR4F21**	0.09523809524	0.09523809524	0.1111111111		0.6842105263	#DIV/0!
**OR4F29**	0.09523809524	0.09523809524	0.1666666667		0.4210526316	#DIV/0!
**OR4F3**	0.09523809524	0.09523809524	0.1666666667		0.4210526316	#DIV/0!
**OR4F4**	0.04761904762	0.04761904762	0.05555555556		0.7	#DIV/0!
**OR4F5**	0.04761904762	0.04761904762	0.1666666667		0.2	#DIV/0!
**OR4F6**	0.04761904762	0.04761904762	0.05555555556		0.7	#DIV/0!
**OR4K1**	0.09523809524	0.09523809524	0		inf	#DIV/0!
**OR4K13**	0.04761904762	0.04761904762	0		inf	#DIV/0!
**OR4K14**	0.09523809524	0.09523809524	0		inf	#DIV/0!
**OR4K15**	0.09523809524	0.09523809524	0		inf	#DIV/0!
**OR4K17**	0.04761904762	0.04761904762	0		inf	#DIV/0!

**OR4K2**	0.09523809524	0.09523809524	0.05555555556		1.473684211	#DIV/0!
**OR4K3**	0.09523809524	0.09523809524	0.05555555556		1.473684211	#DIV/0!
**OR4K5**	0.09523809524	0.09523809524	0.05555555556		1.473684211	#DIV/0!
**OR4L1**	0.04761904762	0.04761904762	0		inf	#DIV/0!
**OR4M1**	0.09523809524	0.09523809524	0.05555555556		1.473684211	#DIV/0!
**OR4M2**	0.2380952381	0.2380952381	0.1111111111		2.03125	#DIV/0!
**OR4N2**	0.09523809524	0.09523809524	0.05555555556		1.473684211	#DIV/0!
**OR4N3P**	0.2380952381	0.2380952381	0.1111111111		2.03125	#DIV/0!
**OR4N4**	0.2380952381	0.2380952381	0.1111111111		2.03125	#DIV/0!
**OR4N5**	0.04761904762	0.04761904762	0		inf	#DIV/0!
**OR4Q2**	0.09523809524	0.09523809524	0		inf	#DIV/0!
**OR4Q3**	0.09523809524	0.09523809524	0.05555555556		1.473684211	#DIV/0!
**OR4S1**	0	0	0.05555555556		0	#DIV/0!
**OR4X1**	0	0	0.05555555556		0	#DIV/0!
**OR4X2**	0	0	0.05555555556		0	#DIV/0!
**OR51A2**	0.04761904762	0.04761904762	0		inf	#DIV/0!
**OR51A4**	0.04761904762	0.04761904762	0		inf	#DIV/0!
**OR51A7**	0.04761904762	0.04761904762	0		inf	#DIV/0!
**OR51B2**	0.04761904762	0.04761904762	0		inf	#DIV/0!
**OR51B4**	0.04761904762	0.04761904762	0		inf	#DIV/0!
**OR51B5**	0.04761904762	0.04761904762	0		inf	#DIV/0!
**OR51B6**	0.04761904762	0.04761904762	0		inf	#DIV/0!
**OR51D1**	0.04761904762	0.04761904762	0		inf	#DIV/0!
**OR51E1**	0.04761904762	0.04761904762	0		inf	#DIV/0!
**OR51E2**	0.04761904762	0.04761904762	0		inf	#DIV/0!
**OR51F1**	0.04761904762	0.04761904762	0		inf	#DIV/0!
**OR51F2**	0.04761904762	0.04761904762	0		inf	#DIV/0!
**OR51G1**	0.04761904762	0.04761904762	0		inf	#DIV/0!
**OR51G2**	0.04761904762	0.04761904762	0		inf	#DIV/0!
**OR51H1**	0.04761904762	0.04761904762	0		inf	#DIV/0!
**OR51I1**	0.04761904762	0.04761904762	0		inf	#DIV/0!
**OR51I2**	0.04761904762	0.04761904762	0		inf	#DIV/0!
**OR51J1**	0.04761904762	0.04761904762	0		inf	#DIV/0!
**OR51L1**	0.04761904762	0.04761904762	0		inf	#DIV/0!
**OR51M1**	0.04761904762	0.04761904762	0		inf	#DIV/0!
**OR51Q1**	0.04761904762	0.04761904762	0		inf	#DIV/0!
**OR51S1**	0.04761904762	0.04761904762	0		inf	#DIV/0!
**OR51T1**	0.04761904762	0.04761904762	0		inf	#DIV/0!
**OR52A1**	0.04761904762	0.04761904762	0		inf	#DIV/0!
**OR52A5**	0.04761904762	0.04761904762	0		inf	#DIV/0!
**OR52B2**	0.04761904762	0.04761904762	0		inf	#DIV/0!
**OR52B4**	0.04761904762	0.04761904762	0		inf	#DIV/0!
**OR52B6**	0.04761904762	0.04761904762	0		inf	#DIV/0!
**OR52D1**	0.04761904762	0.04761904762	0		inf	#DIV/0!
**OR52E1**	0.04761904762	0.04761904762	0		inf	#DIV/0!
**OR52E2**	0.04761904762	0.04761904762	0		inf	#DIV/0!
**OR52E4**	0.04761904762	0.04761904762	0		inf	#DIV/0!
**OR52E5**	0.04761904762	0.04761904762	0		inf	#DIV/0!
**OR52E6**	0.04761904762	0.04761904762	0		inf	#DIV/0!
**OR52E8**	0.04761904762	0.04761904762	0		inf	#DIV/0!
**OR52H1**	0.04761904762	0.04761904762	0		inf	#DIV/0!

**OR52I1**	0.04761904762	0.04761904762	0		inf	#DIV/0!
**OR52I2**	0.04761904762	0.04761904762	0		inf	#DIV/0!
**OR52J3**	0.04761904762	0.04761904762	0		inf	#DIV/0!
**OR52K1**	0.04761904762	0.04761904762	0		inf	#DIV/0!
**OR52K2**	0.04761904762	0.04761904762	0		inf	#DIV/0!
**OR52L1**	0.04761904762	0.04761904762	0		inf	#DIV/0!
**OR52M1**	0.04761904762	0.04761904762	0		inf	#DIV/0!
**OR52N1**	0.04761904762	0.04761904762	0		inf	#DIV/0!
**OR52N2**	0.04761904762	0.04761904762	0		inf	#DIV/0!
**OR52N4**	0.04761904762	0.04761904762	0		inf	#DIV/0!
**OR52N5**	0.04761904762	0.04761904762	0		inf	#DIV/0!
**OR52R1**	0.04761904762	0.04761904762	0		inf	#DIV/0!
**OR52W1**	0.04761904762	0.04761904762	0		inf	#DIV/0!
**OR52Z1**	0.04761904762	0.04761904762	0		inf	#DIV/0!
**OR56A1**	0.04761904762	0.04761904762	0		inf	#DIV/0!
**OR56A3**	0.04761904762	0.04761904762	0		inf	#DIV/0!
**OR56A4**	0.04761904762	0.04761904762	0		inf	#DIV/0!
**OR56A5**	0.04761904762	0.04761904762	0		inf	#DIV/0!
**OR56B1**	0.04761904762	0.04761904762	0		inf	#DIV/0!
**OR56B4**	0.04761904762	0.04761904762	0		inf	#DIV/0!
**OR5AC2**	0.04761904762	0.04761904762	0.05555555556		0.7	#DIV/0!
**OR5C1**	0.09523809524	0.09523809524	0.05555555556		1.473684211	#DIV/0!
**OR5E1P**	0.04761904762	0.04761904762	0		inf	#DIV/0!
**OR5H1**	0.04761904762	0.04761904762	0.05555555556		0.7	#DIV/0!
**OR5H14**	0.04761904762	0.04761904762	0.05555555556		0.7	#DIV/0!
**OR5H15**	0.04761904762	0.04761904762	0.05555555556		0.7	#DIV/0!
**OR5H2**	0.04761904762	0.04761904762	0.05555555556		0.7	#DIV/0!
**OR5H6**	0.04761904762	0.04761904762	0.05555555556		0.7	#DIV/0!
**OR5K1**	0.04761904762	0.04761904762	0.05555555556		0.7	#DIV/0!
**OR5K2**	0.04761904762	0.04761904762	0.05555555556		0.7	#DIV/0!
**OR5K3**	0.04761904762	0.04761904762	0.05555555556		0.7	#DIV/0!
**OR5K4**	0.04761904762	0.04761904762	0.05555555556		0.7	#DIV/0!
**OR5P2**	0.04761904762	0.04761904762	0		inf	#DIV/0!
**OR5P3**	0.04761904762	0.04761904762	0		inf	#DIV/0!
**OR6A2**	0.04761904762	0.04761904762	0		inf	#DIV/0!
**OR6B1**	0.04761904762	0.04761904762	0.05555555556		0.7	#DIV/0!
**OR6B2**	0	0	0.05555555556		0	#DIV/0!
**OR6B3**	0	0	0.05555555556		0	#DIV/0!
**OR6C1**	0.04761904762	0.04761904762	0.05555555556		0.7	#DIV/0!
**OR6C2**	0.04761904762	0.04761904762	0.05555555556		0.7	#DIV/0!
**OR6C3**	0.04761904762	0.04761904762	0.05555555556		0.7	#DIV/0!
**OR6C4**	0.04761904762	0.04761904762	0.05555555556		0.7	#DIV/0!
**OR6C6**	0.04761904762	0.04761904762	0.05555555556		0.7	#DIV/0!
**OR6C65**	0.04761904762	0.04761904762	0.05555555556		0.7	#DIV/0!
**OR6C68**	0.04761904762	0.04761904762	0.05555555556		0.7	#DIV/0!
**OR6C70**	0.04761904762	0.04761904762	0.05555555556		0.7	#DIV/0!
**OR6C74**	0.04761904762	0.04761904762	0.05555555556		0.7	#DIV/0!
**OR6C75**	0.04761904762	0.04761904762	0.05555555556		0.7	#DIV/0!
**OR6C76**	0.04761904762	0.04761904762	0.05555555556		0.7	#DIV/0!
**OR6F1**	0.1428571429	0.1428571429	0		inf	#DIV/0!
**OR6J1**	0	0	0.05555555556		0	#DIV/0!

**OR6K2**	0.1428571429	0.1428571429	0.05555555556		2.333333333	#DIV/0!
**OR6K3**	0.1428571429	0.1428571429	0.05555555556		2.333333333	#DIV/0!
**OR6K6**	0.1428571429	0.1428571429	0		inf	#DIV/0!
**OR6N1**	0.1428571429	0.1428571429	0		inf	#DIV/0!
**OR6N2**	0.1428571429	0.1428571429	0		inf	#DIV/0!
**OR6P1**	0.1428571429	0.1428571429	0.05555555556		2.333333333	#DIV/0!
**OR6V1**	0.04761904762	0.04761904762	0.05555555556		0.7	#DIV/0!
**OR6W1P**	0.04761904762	0.04761904762	0.05555555556		0.7	#DIV/0!
**OR6Y1**	0.1428571429	0.1428571429	0.05555555556		2.333333333	#DIV/0!
**OR7A10**	0.1428571429	0.1428571429	0.1111111111		1.083333333	#DIV/0!
**OR7A17**	0.1428571429	0.1428571429	0.1111111111		1.083333333	#DIV/0!
**OR7A5**	0.1428571429	0.1428571429	0.1111111111		1.083333333	#DIV/0!
**OR7C1**	0.1428571429	0.1428571429	0.1111111111		1.083333333	#DIV/0!
**OR7C2**	0.1428571429	0.1428571429	0.1111111111		1.083333333	#DIV/0!
**OR7D2**	0.1428571429	0.1428571429	0.05555555556		2.333333333	#DIV/0!
**OR7D4**	0.1428571429	0.1428571429	0.05555555556		2.333333333	#DIV/0!
**OR7E12P**	0.04761904762	0.04761904762	0		inf	#DIV/0!
**OR7E24**	0.1428571429	0.1428571429	0.05555555556		2.333333333	#DIV/0!
**OR7E47P**	0.04761904762	0.04761904762	0.05555555556		0.7	#DIV/0!
**OR7E91P**	0	0	0.05555555556		0	#DIV/0!
**OR7G1**	0.1428571429	0.1428571429	0.05555555556		2.333333333	#DIV/0!
**OR7G2**	0.1428571429	0.1428571429	0.05555555556		2.333333333	#DIV/0!
**OR7G3**	0.1428571429	0.1428571429	0.05555555556		2.333333333	#DIV/0!
**OR8S1**	0.04761904762	0.04761904762	0.05555555556		0.7	#DIV/0!
**OR9A2**	0.04761904762	0.04761904762	0.05555555556		0.7	#DIV/0!
**OR9A4**	0.04761904762	0.04761904762	0.05555555556		0.7	#DIV/0!
**OR9K2**	0.04761904762	0.04761904762	0.05555555556		0.7	#DIV/0!
**ORAI1**	0.04761904762	0.04761904762	0.1111111111		0.325	#DIV/0!
**ORAI2**	0.04761904762	0.04761904762	0.05555555556		0.7	#DIV/0!
**ORAI3**	0.04761904762	0.04761904762	0		inf	#DIV/0!
**ORC1**	0.04761904762	0.04761904762	0.05555555556		0.7	#DIV/0!
**ORC2**	0	0	0.05555555556		0	#DIV/0!
**ORC4**	0	0	0.05555555556		0	#DIV/0!
**ORC5**	0.09523809524	0.09523809524	0.05555555556		1.473684211	#DIV/0!
**ORC6**	0.04761904762	0.04761904762	0		inf	#DIV/0!
**ORM1**	0.04761904762	0.04761904762	0.05555555556		0.7	#DIV/0!
**ORM2**	0.04761904762	0.04761904762	0.05555555556		0.7	#DIV/0!
**ORMDL**	0	0	0.05555555556		0	#DIV/0!
**ORMDL**	0.04761904762	0.04761904762	0.05555555556		0.7	#DIV/0!
**ORMDL**	0.09523809524	0.09523809524	0		inf	#DIV/0!
**OS9**	0.1428571429	0.1428571429	0.05555555556		2.333333333	#DIV/0!
**OSBP2**	0.04761904762	0.04761904762	0.1111111111		0.325	#DIV/0!
**OSBPL10**	0.09523809524	0.09523809524	0		inf	#DIV/0!
**BPL10-A**	0.04761904762	0.04761904762	0		inf	#DIV/0!
**OSBPL1**	0.04761904762	0.04761904762	0.05555555556		0.7	#DIV/0!
**OSBPL1A**	0.09523809524	0.09523809524	0.05555555556		1.473684211	#DIV/0!
**OSBPL2**	0.1428571429	0.1428571429	0.2222222222		0.4583333333	#DIV/0!
**OSBPL3**	0.04761904762	0.04761904762	0.2222222222		0.1375	#DIV/0!
**OSBPL5**	0.04761904762	0.04761904762	0		inf	#DIV/0!
**OSBPL6**	0	0	0.1111111111		0	#DIV/0!
**OSBPL7**	0.1428571429	0.1428571429	0		inf	#DIV/0!

OSBPL8	0.04761904762	0.04761904762	0.1111111111		0.325	#DIV/0!
OSBPL9	0.04761904762	0.04761904762	0.05555555556		0.7	#DIV/0!
OSCAR	0.09523809524	0.09523809524	0.05555555556		1.473684211	#DIV/0!
OSCP1	0.04761904762	0.04761904762	0.05555555556		0.7	#DIV/0!
OSER1	0.1428571429	0.1428571429	0.1666666667		0.6666666667	#DIV/0!
SER1-D	0.1428571429	0.1428571429	0.1666666667		0.6666666667	#DIV/0!
SGEPL	0	0	0.05555555556		0	#DIV/0!
GEPL1-	0	0	0.05555555556		0	#DIV/0!
OSGIN1	0.04761904762	0.04761904762	0		inf	#DIV/0!
OSGIN2	0.1904761905	0.1904761905	0.1111111111		1.529411765	#DIV/0!
OSM	0.04761904762	0.04761904762	0.1111111111		0.325	#DIV/0!
OSMR	0.04761904762	0.04761904762	0.05555555556		0.7	#DIV/0!
SMR-AS	0.04761904762	0.04761904762	0.05555555556		0.7	#DIV/0!
OSR1	0	0	0.05555555556		0	#DIV/0!
OSR2	0.1904761905	0.1904761905	0.1111111111		1.529411765	#DIV/0!
OSTC	0	0	0.05555555556		0	#DIV/0!
OSTF1	0	0	0.05555555556		0	#DIV/0!
OSTM1	0	0	0			#DIV/0!
STM1-A	0	0	0			#DIV/0!
OSTN	0.09523809524	0.09523809524	0.05555555556		1.473684211	#DIV/0!
STN-AS	0.09523809524	0.09523809524	0.05555555556		1.473684211	#DIV/0!
OTOA	0.04761904762	0.04761904762	0		inf	#DIV/0!
OTOAP1	0.04761904762	0.04761904762	0		inf	#DIV/0!
OTOL1	0.1428571429	0.1428571429	0.05555555556		2.333333333	#DIV/0!
OTOP2	0.04761904762	0.04761904762	0		inf	#DIV/0!
OTOP3	0.04761904762	0.04761904762	0		inf	#DIV/0!
OTOR	0.1904761905	0.1904761905	0.1111111111		1.529411765	#DIV/0!
OTOS	0.04761904762	0.04761904762	0.05555555556		0.7	#DIV/0!
OTP	0.04761904762	0.04761904762	0		inf	#DIV/0!
OTUD1	0.09523809524	0.09523809524	0.1111111111		0.6842105263	#DIV/0!
OTUD3	0.04761904762	0.04761904762	0.1111111111		0.325	#DIV/0!
OTUD4	0	0	0.05555555556		0	#DIV/0!
OTUD6B	0.1904761905	0.1904761905	0.1111111111		1.529411765	#DIV/0!
UD6B-A	0.1904761905	0.1904761905	0.1111111111		1.529411765	#DIV/0!
OTUD7B	0.1904761905	0.1904761905	0.1111111111		1.529411765	#DIV/0!
OTULIN	0.04761904762	0.04761904762	0.05555555556		0.7	#DIV/0!
TULINI	0.04761904762	0.04761904762	0.05555555556		0.7	#DIV/0!
OTX1	0	0	0.05555555556		0	#DIV/0!
OTX2	0.04761904762	0.04761904762	0		inf	#DIV/0!
TX2-AS	0.04761904762	0.04761904762	0		inf	#DIV/0!
OVAAL	0.1428571429	0.1428571429	0		inf	#DIV/0!
OVCA2	0	0	0.1111111111		0	#DIV/0!
OVCH1	0.04761904762	0.04761904762	0.05555555556		0.7	#DIV/0!
VCH1-A	0.04761904762	0.04761904762	0.05555555556		0.7	#DIV/0!
OVCH2	0.04761904762	0.04761904762	0		inf	#DIV/0!
OVGP1	0.04761904762	0.04761904762	0		inf	#DIV/0!
OVOL2	0.1904761905	0.1904761905	0.1111111111		1.529411765	#DIV/0!
OVOL3	0.04761904762	0.04761904762	0.05555555556		0.7	#DIV/0!
OVOS	0.04761904762	0.04761904762	0.1111111111		0.325	#DIV/0!
OVOS2	0.04761904762	0.04761904762	0.05555555556		0.7	#DIV/0!
OXA1L	0	0	0.05555555556		0	#DIV/0!

OXCT1	0.04761904762	0.04761904762	0.05555555556		0.7	#DIV/0!
XCT1-A	0.04761904762	0.04761904762	0.05555555556		0.7	#DIV/0!
OXCT2	0.04761904762	0.04761904762	0.05555555556		0.7	#DIV/0!
XCT2P	0.04761904762	0.04761904762	0.05555555556		0.7	#DIV/0!
OXER1	0	0	0.05555555556		0	#DIV/0!
OXLD1	0.04761904762	0.04761904762	0		inf	#DIV/0!
XNAD1	0.04761904762	0.04761904762	0		inf	#DIV/0!
OXR1	0.1904761905	0.1904761905	0.1111111111		1.529411765	#DIV/0!
OXSR1	0.04761904762	0.04761904762	0		inf	#DIV/0!
OXT	0.1904761905	0.1904761905	0.05555555556		3.294117647	#DIV/0!
OXTR	0.04761904762	0.04761904762	0		inf	#DIV/0!
P2RX1	0	0	0.1111111111		0	#DIV/0!
P2RX2	0.09523809524	0.09523809524	0.05555555556		1.473684211	#DIV/0!
P2RX4	0.04761904762	0.04761904762	0.1111111111		0.325	#DIV/0!
P2RX5	0	0	0.1111111111		0	#DIV/0!
X5-TAX1	0	0	0.1111111111		0	#DIV/0!
P2RX6	0.04761904762	0.04761904762	0.05555555556		0.7	#DIV/0!
P2RX6P	0.04761904762	0.04761904762	0.05555555556		0.7	#DIV/0!
P2RX7	0.04761904762	0.04761904762	0.1111111111		0.325	#DIV/0!
P2RY1	0.1904761905	0.1904761905	0.1111111111		1.529411765	#DIV/0!
P2RY11	0.1428571429	0.1428571429	0.1111111111		1.083333333	#DIV/0!
P2RY12	0.1904761905	0.1904761905	0.1111111111		1.529411765	#DIV/0!
P2RY13	0.1904761905	0.1904761905	0.1111111111		1.529411765	#DIV/0!
P2RY14	0.1904761905	0.1904761905	0.1111111111		1.529411765	#DIV/0!
P3H1	0.04761904762	0.04761904762	0.05555555556		0.7	#DIV/0!
P3H2	0.09523809524	0.09523809524	0.1111111111		0.6842105263	#DIV/0!
3H2-AS	0.09523809524	0.09523809524	0.1111111111		0.6842105263	#DIV/0!
P3H3	0.04761904762	0.04761904762	0.1111111111		0.325	#DIV/0!
P3H4	0.09523809524	0.09523809524	0		inf	#DIV/0!
3R3URI	0.04761904762	0.04761904762	0.05555555556		0.7	#DIV/0!
P4HA1	0.1904761905	0.1904761905	0.05555555556		3.294117647	#DIV/0!
P4HA2	0.04761904762	0.04761904762	0		inf	#DIV/0!
4HA2-AS	0.04761904762	0.04761904762	0		inf	#DIV/0!
P4HB	0.04761904762	0.04761904762	0		inf	#DIV/0!
P4HTM	0.09523809524	0.09523809524	0		inf	#DIV/0!
PA2G4	0.09523809524	0.09523809524	0.05555555556		1.473684211	#DIV/0!
PA2G4P4	0.1428571429	0.1428571429	0.05555555556		2.333333333	#DIV/0!
PABPC1	0.1904761905	0.1904761905	0.1111111111		1.529411765	#DIV/0!
ABPC1I	0.1428571429	0.1428571429	0.1666666667		0.6666666667	#DIV/0!
ABPC1P	0	0	0.05555555556		0	#DIV/0!
PABPC4	0.04761904762	0.04761904762	0.05555555556		0.7	#DIV/0!
BPC4-A	0.04761904762	0.04761904762	0.05555555556		0.7	#DIV/0!
ABPC4I	0	0	0.05555555556		0	#DIV/0!
PABPN1	0	0	0.1111111111		0	#DIV/0!
ABPN1I	0.1904761905	0.1904761905	0		inf	#DIV/0!
ACERI	0.1428571429	0.1428571429	0.05555555556		2.333333333	#DIV/0!
PACRG	0	0	0.1111111111		0	#DIV/0!
CRG-A	0	0	0.1111111111		0	#DIV/0!
CRG-A	0	0	0.05555555556		0	#DIV/0!
CRG-A	0	0	0.1111111111		0	#DIV/0!
ACSIN	0.04761904762	0.04761904762	0		inf	#DIV/0!

PACSIN2	0	0	0.1666666667		0	#DIV/0!
PACSIN3	0	0	0.05555555556		0	#DIV/0!
PADI1	0.04761904762	0.04761904762	0.1111111111		0.325	#DIV/0!
PADI2	0.04761904762	0.04761904762	0.1111111111		0.325	#DIV/0!
PADI3	0.04761904762	0.04761904762	0.1111111111		0.325	#DIV/0!
PADI4	0.04761904762	0.04761904762	0.1111111111		0.325	#DIV/0!
PADI6	0.04761904762	0.04761904762	0.1111111111		0.325	#DIV/0!
PAEP	0.04761904762	0.04761904762	0		inf	#DIV/0!
PAF1	0.04761904762	0.04761904762	0.05555555556		0.7	#DIV/0!
AFAH1I	0	0	0.1111111111		0	#DIV/0!
AFAH1I	0.04761904762	0.04761904762	0.05555555556		0.7	#DIV/0!
PAFAH2	0.04761904762	0.04761904762	0.1111111111		0.325	#DIV/0!
PAG1	0.1428571429	0.1428571429	0.1111111111		1.083333333	#DIV/0!
PAGR1	0.04761904762	0.04761904762	0		inf	#DIV/0!
PAH	0.04761904762	0.04761904762	0.1111111111		0.325	#DIV/0!
PAICS	0.1904761905	0.1904761905	0.05555555556		3.294117647	#DIV/0!
PAIP1	0.04761904762	0.04761904762	0.05555555556		0.7	#DIV/0!
PAIP2	0.04761904762	0.04761904762	0		inf	#DIV/0!
PAIP2B	0	0	0.05555555556		0	#DIV/0!
PAK2	0.1428571429	0.1428571429	0.05555555556		2.333333333	#DIV/0!
PAK4	0.04761904762	0.04761904762	0.05555555556		0.7	#DIV/0!
PAK5	0.1904761905	0.1904761905	0.1111111111		1.529411765	#DIV/0!
PALB2	0.04761904762	0.04761904762	0		inf	#DIV/0!
PALD1	0.09523809524	0.09523809524	0		inf	#DIV/0!
PALLD	0	0	0.05555555556		0	#DIV/0!
PALM	0.09523809524	0.09523809524	0.1111111111		0.6842105263	#DIV/0!
PALM2	0.04761904762	0.04761904762	0.05555555556		0.7	#DIV/0!
LM2-AK	0.04761904762	0.04761904762	0.05555555556		0.7	#DIV/0!
PALM3	0.1428571429	0.1428571429	0.1111111111		1.083333333	#DIV/0!
PALMD	0.04761904762	0.04761904762	0.05555555556		0.7	#DIV/0!
PAM	0.04761904762	0.04761904762	0		inf	#DIV/0!
PAN2	0.1428571429	0.1428571429	0.05555555556		2.333333333	#DIV/0!
PANCR	0	0	0.05555555556		0	#DIV/0!
PANDAI	0.04761904762	0.04761904762	0.05555555556		0.7	#DIV/0!
PANK1	0.1428571429	0.1428571429	0.1111111111		1.083333333	#DIV/0!
PANK2	0.1904761905	0.1904761905	0.05555555556		3.294117647	#DIV/0!
PANK3	0	0	0.05555555556		0	#DIV/0!
PANK4	0	0	0.1111111111		0	#DIV/0!
PANO1	0.04761904762	0.04761904762	0		inf	#DIV/0!
PANTR1	0	0	0.05555555556		0	#DIV/0!
PANX2	0	0	0.1111111111		0	#DIV/0!
PAOX	0.1904761905	0.1904761905	0		inf	#DIV/0!
PAPLN	0.09523809524	0.09523809524	0		inf	#DIV/0!
PAPOLE	0.04761904762	0.04761904762	0.1666666667		0.2	#DIV/0!
PAPOLC	0.09523809524	0.09523809524	0.05555555556		1.473684211	#DIV/0!
PAPPA	0.04761904762	0.04761904762	0.05555555556		0.7	#DIV/0!
APPA-AS	0.04761904762	0.04761904762	0.05555555556		0.7	#DIV/0!
PAPPA2	0.1428571429	0.1428571429	0		inf	#DIV/0!
PAPSS1	0	0	0.05555555556		0	#DIV/0!
PAPSS2	0.1428571429	0.1428571429	0.1666666667		0.6666666667	#DIV/0!
PAQR3	0.04761904762	0.04761904762	0.05555555556		0.7	#DIV/0!

PAQR4	0.04761904762	0.04761904762	0.1111111111		0.325	#DIV/0!
PAQR5	0.04761904762	0.04761904762	0.05555555556		0.7	#DIV/0!
PAQR7	0.04761904762	0.04761904762	0.1111111111		0.325	#DIV/0!
PAQR8	0.04761904762	0.04761904762	0		inf	#DIV/0!
PAQR9	0.1428571429	0.1428571429	0.05555555556		2.333333333	#DIV/0!
AQR9-AS	0.1428571429	0.1428571429	0.05555555556		2.333333333	#DIV/0!
PARAL1	0.1904761905	0.1904761905	0.1111111111		1.529411765	#DIV/0!
PARD3	0.09523809524	0.09523809524	0.2222222222		0.2894736842	#DIV/0!
ARD3-AS	0.09523809524	0.09523809524	0.1666666667		0.4210526316	#DIV/0!
PARD3B	0	0	0.05555555556		0	#DIV/0!
PARD6A	0.1428571429	0.1428571429	0.05555555556		2.333333333	#DIV/0!
PARD6B	0.09523809524	0.09523809524	0.1666666667		0.4210526316	#DIV/0!
PARD6G	0.04761904762	0.04761904762	0.05555555556		0.7	#DIV/0!
RD6G-A	0.04761904762	0.04761904762	0.05555555556		0.7	#DIV/0!
PARG	0.04761904762	0.04761904762	0.05555555556		0.7	#DIV/0!
PARGP1	0.04761904762	0.04761904762	0.05555555556		0.7	#DIV/0!
GP1-AG	0.04761904762	0.04761904762	0.05555555556		0.7	#DIV/0!
PARK7	0	0	0.05555555556		0	#DIV/0!
PARL	0.1904761905	0.1904761905	0.05555555556		3.294117647	#DIV/0!
PARM1	0.09523809524	0.09523809524	0.05555555556		1.473684211	#DIV/0!
PARN	0.04761904762	0.04761904762	0		inf	#DIV/0!
PARP1	0.1904761905	0.1904761905	0		inf	#DIV/0!
PARP10	0.04761904762	0.04761904762	0.1666666667		0.2	#DIV/0!
PARP11	0.04761904762	0.04761904762	0.1111111111		0.325	#DIV/0!
PARP12	0.04761904762	0.04761904762	0.05555555556		0.7	#DIV/0!
PARP14	0.09523809524	0.09523809524	0.05555555556		1.473684211	#DIV/0!
PARP15	0.09523809524	0.09523809524	0.05555555556		1.473684211	#DIV/0!
PARP16	0.04761904762	0.04761904762	0.05555555556		0.7	#DIV/0!
PARP2	0.04761904762	0.04761904762	0		inf	#DIV/0!
PARP3	0.04761904762	0.04761904762	0		inf	#DIV/0!
PARP6	0.04761904762	0.04761904762	0.05555555556		0.7	#DIV/0!
PARP8	0.04761904762	0.04761904762	0.05555555556		0.7	#DIV/0!
PARP9	0.09523809524	0.09523809524	0.05555555556		1.473684211	#DIV/0!
PARPBP	0.04761904762	0.04761904762	0.05555555556		0.7	#DIV/0!
PARS2	0.04761904762	0.04761904762	0.05555555556		0.7	#DIV/0!
PART1	0.04761904762	0.04761904762	0		inf	#DIV/0!
PARTICL	0	0	0.05555555556		0	#DIV/0!
PARVB	0	0	0.1666666667		0	#DIV/0!
PARVG	0	0	0.1666666667		0	#DIV/0!
PASK	0	0	0.05555555556		0	#DIV/0!
PATJ	0.04761904762	0.04761904762	0.05555555556		0.7	#DIV/0!
PATZ1	0.04761904762	0.04761904762	0.1111111111		0.325	#DIV/0!
PAWR	0.04761904762	0.04761904762	0.05555555556		0.7	#DIV/0!
PAX1	0.1904761905	0.1904761905	0.05555555556		3.294117647	#DIV/0!
PAX2	0.1428571429	0.1428571429	0		inf	#DIV/0!
PAX3	0	0	0.05555555556		0	#DIV/0!
PAX4	0.04761904762	0.04761904762	0.05555555556		0.7	#DIV/0!
PAX5	0.09523809524	0.09523809524	0.05555555556		1.473684211	#DIV/0!
PAX7	0.04761904762	0.04761904762	0.05555555556		0.7	#DIV/0!
PAX8	0	0	0.05555555556		0	#DIV/0!
AX8-AS	0	0	0.05555555556		0	#DIV/0!

PAXBP1	0.04761904762	0.04761904762	0.1111111111		0.325	#DIV/0!
XBP1-A	0.04761904762	0.04761904762	0.1111111111		0.325	#DIV/0!
PAXIP1	0.1428571429	0.1428571429	0.05555555556		2.333333333	#DIV/0!
XIP1-A	0.1428571429	0.1428571429	0.05555555556		2.333333333	#DIV/0!
XIP1-A	0.1428571429	0.1428571429	0.05555555556		2.333333333	#DIV/0!
PAXX	0.1428571429	0.1428571429	0.05555555556		2.333333333	#DIV/0!
PBK	0.04761904762	0.04761904762	0.1111111111		0.325	#DIV/0!
PBLD	0.1904761905	0.1904761905	0.05555555556		3.294117647	#DIV/0!
PBRM1	0.04761904762	0.04761904762	0		inf	#DIV/0!
PBX1	0.1428571429	0.1428571429	0		inf	#DIV/0!
PBX2	0.04761904762	0.04761904762	0		inf	#DIV/0!
PBX3	0.09523809524	0.09523809524	0.05555555556		1.473684211	#DIV/0!
PBX4	0.1428571429	0.1428571429	0.1111111111		1.083333333	#DIV/0!
PBXIP1	0.1904761905	0.1904761905	0.1111111111		1.529411765	#DIV/0!
PCA3	0	0	0.05555555556		0	#DIV/0!
PCARE	0	0	0.05555555556		0	#DIV/0!
PCAT1	0.09523809524	0.09523809524	0.1111111111		0.6842105263	#DIV/0!
PCAT14	0	0	0.05555555556		0	#DIV/0!
PCAT18	0.09523809524	0.09523809524	0.05555555556		1.473684211	#DIV/0!
PCAT19	0.04761904762	0.04761904762	0.05555555556		0.7	#DIV/0!
PCAT2	0.09523809524	0.09523809524	0.1111111111		0.6842105263	#DIV/0!
PCAT29	0.04761904762	0.04761904762	0.05555555556		0.7	#DIV/0!
PCAT4	0.04761904762	0.04761904762	0.05555555556		0.7	#DIV/0!
PCAT5	0.09523809524	0.09523809524	0.1666666667		0.4210526316	#DIV/0!
PCAT6	0.1428571429	0.1428571429	0		inf	#DIV/0!
PCAT7	0.04761904762	0.04761904762	0.05555555556		0.7	#DIV/0!
PCBD1	0.09523809524	0.09523809524	0		inf	#DIV/0!
PCBD2	0.04761904762	0.04761904762	0		inf	#DIV/0!
PCBP1	0	0	0.05555555556		0	#DIV/0!
CBP1-AS	0	0	0.05555555556		0	#DIV/0!
PCBP2	0.04761904762	0.04761904762	0.05555555556		0.7	#DIV/0!
CBP2-OT	0.04761904762	0.04761904762	0.05555555556		0.7	#DIV/0!
PCBP3	0.04761904762	0.04761904762	0.1111111111		0.325	#DIV/0!
PCBP4	0.04761904762	0.04761904762	0		inf	#DIV/0!
PCCB	0.1904761905	0.1904761905	0.05555555556		3.294117647	#DIV/0!
PCDH1	0.04761904762	0.04761904762	0		inf	#DIV/0!
PCDH10	0	0	0.05555555556		0	#DIV/0!
PCDH12	0.04761904762	0.04761904762	0		inf	#DIV/0!
PCDH15	0.09523809524	0.09523809524	0.05555555556		1.473684211	#DIV/0!
PCDH18	0	0	0.05555555556		0	#DIV/0!
PCDHA1	0.04761904762	0.04761904762	0		inf	#DIV/0!
CDHA1	0.04761904762	0.04761904762	0		inf	#DIV/0!
PCDHA1	0.04761904762	0.04761904762	0		inf	#DIV/0!
CDHA1	0.04761904762	0.04761904762	0		inf	#DIV/0!
CDHA1	0.04761904762	0.04761904762	0		inf	#DIV/0!
PCDHA2	0.04761904762	0.04761904762	0		inf	#DIV/0!
PCDHA3	0.04761904762	0.04761904762	0		inf	#DIV/0!
PCDHA4	0.04761904762	0.04761904762	0		inf	#DIV/0!
PCDHA5	0.04761904762	0.04761904762	0		inf	#DIV/0!
PCDHA6	0.04761904762	0.04761904762	0		inf	#DIV/0!
PCDHA7	0.04761904762	0.04761904762	0		inf	#DIV/0!

PCDHA8	0.04761904762	0.04761904762	0		inf	#DIV/0!
PCDHA9	0.04761904762	0.04761904762	0		inf	#DIV/0!
CDHAC	0.04761904762	0.04761904762	0		inf	#DIV/0!
CDHAC	0.04761904762	0.04761904762	0		inf	#DIV/0!
PCDHB1	0.04761904762	0.04761904762	0		inf	#DIV/0!
PCDHB1	0.04761904762	0.04761904762	0		inf	#DIV/0!
PCDHB1	0.04761904762	0.04761904762	0		inf	#DIV/0!
PCDHB1	0.04761904762	0.04761904762	0		inf	#DIV/0!
PCDHB1	0.04761904762	0.04761904762	0		inf	#DIV/0!
PCDHB1	0.04761904762	0.04761904762	0		inf	#DIV/0!
PCDHB1	0.04761904762	0.04761904762	0		inf	#DIV/0!
PCDHB1	0.04761904762	0.04761904762	0		inf	#DIV/0!
CDHB17	0.04761904762	0.04761904762	0		inf	#DIV/0!
CDHB18	0.04761904762	0.04761904762	0		inf	#DIV/0!
CDHB19	0.04761904762	0.04761904762	0		inf	#DIV/0!
PCDHB2	0.04761904762	0.04761904762	0		inf	#DIV/0!
PCDHB3	0.04761904762	0.04761904762	0		inf	#DIV/0!
PCDHB4	0.04761904762	0.04761904762	0		inf	#DIV/0!
PCDHB5	0.04761904762	0.04761904762	0		inf	#DIV/0!
PCDHB6	0.04761904762	0.04761904762	0		inf	#DIV/0!
PCDHB7	0.04761904762	0.04761904762	0		inf	#DIV/0!
PCDHB8	0.04761904762	0.04761904762	0		inf	#DIV/0!
PCDHB9	0.04761904762	0.04761904762	0		inf	#DIV/0!
CDHGA	0.04761904762	0.04761904762	0		inf	#DIV/0!
CDHGA	0.04761904762	0.04761904762	0		inf	#DIV/0!
CDHGA	0.04761904762	0.04761904762	0		inf	#DIV/0!
CDHGA	0.04761904762	0.04761904762	0		inf	#DIV/0!
CDHGA	0.04761904762	0.04761904762	0		inf	#DIV/0!
CDHGA	0.04761904762	0.04761904762	0		inf	#DIV/0!
CDHGA	0.04761904762	0.04761904762	0		inf	#DIV/0!
CDHGA	0.04761904762	0.04761904762	0		inf	#DIV/0!
CDHGA	0.04761904762	0.04761904762	0		inf	#DIV/0!
CDHGA	0.04761904762	0.04761904762	0		inf	#DIV/0!
CDHGA	0.04761904762	0.04761904762	0		inf	#DIV/0!
CDHGA	0.04761904762	0.04761904762	0		inf	#DIV/0!
CDHGB	0.04761904762	0.04761904762	0		inf	#DIV/0!
CDHGB	0.04761904762	0.04761904762	0		inf	#DIV/0!
CDHGB	0.04761904762	0.04761904762	0		inf	#DIV/0!
CDHGB	0.04761904762	0.04761904762	0		inf	#DIV/0!
CDHGB	0.04761904762	0.04761904762	0		inf	#DIV/0!
CDHGB	0.04761904762	0.04761904762	0		inf	#DIV/0!
CDHGB	0.04761904762	0.04761904762	0		inf	#DIV/0!
CDHGB8	0.04761904762	0.04761904762	0		inf	#DIV/0!
CDHGC	0.04761904762	0.04761904762	0		inf	#DIV/0!
CDHGC	0.04761904762	0.04761904762	0		inf	#DIV/0!
CDHGC	0.04761904762	0.04761904762	0		inf	#DIV/0!
PCED1A	0.1904761905	0.1904761905	0.05555555556		3.294117647	#DIV/0!
PCED1B	0.04761904762	0.04761904762	0.05555555556		0.7	#DIV/0!
ED1B-A	0.04761904762	0.04761904762	0.05555555556		0.7	#DIV/0!
PCGEM1	0	0	0.05555555556		0	#DIV/0!
PCGF1	0	0	0.05555555556		0	#DIV/0!

**PCGF2**	0.04761904762	0.04761904762	0		inf	#DIV/0!
**PCGF3**	0.04761904762	0.04761904762	0		inf	#DIV/0!
**PCGF5**	0.1428571429	0.1428571429	0.1111111111		1.083333333	#DIV/0!
**PCGF6**	0.1428571429	0.1428571429	0.05555555556		2.333333333	#DIV/0!
**PCIF1**	0.1428571429	0.1428571429	0.1666666667		0.6666666667	#DIV/0!
**PCK1**	0.1428571429	0.1428571429	0.2222222222		0.4583333333	#DIV/0!
**PCLAF**	0.04761904762	0.04761904762	0.05555555556		0.7	#DIV/0!
**PCLO**	0.04761904762	0.04761904762	0.1111111111		0.325	#DIV/0!
**PCM1**	0.09523809524	0.09523809524	0.1111111111		0.6842105263	#DIV/0!
**PCMT1**	0	0	0			#DIV/0!
**PCMTD1**	0.09523809524	0.09523809524	0.1111111111		0.6842105263	#DIV/0!
**PCMTD2**	0.1428571429	0.1428571429	0.2222222222		0.4583333333	#DIV/0!
**PCNA**	0.1904761905	0.1904761905	0.05555555556		3.294117647	#DIV/0!
**CNA-AS**	0.1904761905	0.1904761905	0.05555555556		3.294117647	#DIV/0!
**PCNAP1**	0	0	0.05555555556		0	#DIV/0!
**PCNP**	0.09523809524	0.09523809524	0.05555555556		1.473684211	#DIV/0!
**PCNT**	0.04761904762	0.04761904762	0.1111111111		0.325	#DIV/0!
**PCNX2**	0.1428571429	0.1428571429	0		inf	#DIV/0!
**PCOLCE**	0.04761904762	0.04761904762	0.05555555556		0.7	#DIV/0!
**OLCE-A**	0.04761904762	0.04761904762	0.05555555556		0.7	#DIV/0!
**COLCE**	0.1428571429	0.1428571429	0.05555555556		2.333333333	#DIV/0!
**PCP2**	0.1428571429	0.1428571429	0.05555555556		2.333333333	#DIV/0!
**PCP4**	0.04761904762	0.04761904762	0.05555555556		0.7	#DIV/0!
**PCP4L1**	0.1428571429	0.1428571429	0		inf	#DIV/0!
**PCSK1**	0.04761904762	0.04761904762	0		inf	#DIV/0!
**PCSK2**	0.1904761905	0.1904761905	0.1111111111		1.529411765	#DIV/0!
**PCSK4**	0.09523809524	0.09523809524	0.1111111111		0.6842105263	#DIV/0!
**PCSK5**	0	0	0.05555555556		0	#DIV/0!
**PCSK6**	0.04761904762	0.04761904762	0.05555555556		0.7	#DIV/0!
**CSK6-AS**	0.04761904762	0.04761904762	0.05555555556		0.7	#DIV/0!
**PCSK9**	0.04761904762	0.04761904762	0.05555555556		0.7	#DIV/0!
**PCTP**	0.09523809524	0.09523809524	0		inf	#DIV/0!
**PCYOX1**	0	0	0.05555555556		0	#DIV/0!
**PCYT1A**	0.1428571429	0.1428571429	0.05555555556		2.333333333	#DIV/0!
**PCYT2**	0.04761904762	0.04761904762	0		inf	#DIV/0!
**PDAP1**	0.04761904762	0.04761904762	0.05555555556		0.7	#DIV/0!
**PDC**	0.1428571429	0.1428571429	0.05555555556		2.333333333	#DIV/0!
**PDCD1**	0	0	0.05555555556		0	#DIV/0!
**PDCD10**	0.1428571429	0.1428571429	0.05555555556		2.333333333	#DIV/0!
**PDCD11**	0.1428571429	0.1428571429	0.05555555556		2.333333333	#DIV/0!
**DCD1LG**	0.04761904762	0.04761904762	0.05555555556		0.7	#DIV/0!
**PDCD2L**	0.04761904762	0.04761904762	0.05555555556		0.7	#DIV/0!
**PDCD4**	0.1428571429	0.1428571429	0.05555555556		2.333333333	#DIV/0!
**DCD4-AS**	0.1428571429	0.1428571429	0.05555555556		2.333333333	#DIV/0!
**PDCD5**	0.04761904762	0.04761904762	0.05555555556		0.7	#DIV/0!
**PDCD6**	0	0	0.05555555556		0	#DIV/0!
**PDCD6IP**	0.09523809524	0.09523809524	0		inf	#DIV/0!
**PDCD7**	0.04761904762	0.04761904762	0.05555555556		0.7	#DIV/0!
**PDCL**	0.09523809524	0.09523809524	0.05555555556		1.473684211	#DIV/0!
**PDCL2**	0.1904761905	0.1904761905	0.05555555556		3.294117647	#DIV/0!
**PDCL3**	0	0	0.05555555556		0	#DIV/0!

**'DCL3P**	0.09523809524	0.09523809524	0.05555555556		1.473684211	#DIV/0!
**PDE11A**	0	0	0.1111111111		0	#DIV/0!
**PDE12**	0.09523809524	0.09523809524	0		inf	#DIV/0!
**PDE1A**	0	0	0.05555555556		0	#DIV/0!
**PDE1B**	0.04761904762	0.04761904762	0.05555555556		0.7	#DIV/0!
**PDE1C**	0.09523809524	0.09523809524	0.1111111111		0.6842105263	#DIV/0!
**PDE3A**	0.04761904762	0.04761904762	0.1111111111		0.325	#DIV/0!
**PDE4A**	0.1428571429	0.1428571429	0.1111111111		1.083333333	#DIV/0!
**PDE4B**	0.04761904762	0.04761904762	0.05555555556		0.7	#DIV/0!
**PDE4C**	0.1428571429	0.1428571429	0.1111111111		1.083333333	#DIV/0!
**PDE4D**	0.04761904762	0.04761904762	0.05555555556		0.7	#DIV/0!
**'DE4DII**	0.09523809524	0.09523809524	0.05555555556		1.473684211	#DIV/0!
**DE4DIP**	0.1428571429	0.1428571429	0.05555555556		2.333333333	#DIV/0!
**PDE5A**	0	0	0.05555555556		0	#DIV/0!
**PDE6B**	0.04761904762	0.04761904762	0		inf	#DIV/0!
**PDE6C**	0.1428571429	0.1428571429	0		inf	#DIV/0!
**PDE6D**	0	0	0.05555555556		0	#DIV/0!
**PDE6G**	0.04761904762	0.04761904762	0		inf	#DIV/0!
**PDE6H**	0.04761904762	0.04761904762	0.1111111111		0.325	#DIV/0!
**PDE7A**	0.1428571429	0.1428571429	0.1666666667		0.6666666667	#DIV/0!
**PDE8A**	0.04761904762	0.04761904762	0.05555555556		0.7	#DIV/0!
**PDE8B**	0.04761904762	0.04761904762	0		inf	#DIV/0!
**PDE9A**	0.04761904762	0.04761904762	0.1666666667		0.2	#DIV/0!
**PDF**	0.1428571429	0.1428571429	0.05555555556		2.333333333	#DIV/0!
**PDGFA**	0.04761904762	0.04761904762	0.1666666667		0.2	#DIV/0!
**PDGFB**	0.04761904762	0.04761904762	0.1111111111		0.325	#DIV/0!
**PDGFC**	0	0	0.1111111111		0	#DIV/0!
**'DGFRA**	0.2380952381	0.2380952381	0.05555555556		4.375	#DIV/0!
**'DGFRI**	0.09523809524	0.09523809524	0.1111111111		0.6842105263	#DIV/0!
**PDHA2**	0	0	0.05555555556		0	#DIV/0!
**PDHB**	0.04761904762	0.04761904762	0		inf	#DIV/0!
**PDIA2**	0.04761904762	0.04761904762	0.2222222222		0.1375	#DIV/0!
**'DIA3P1**	0.1428571429	0.1428571429	0.05555555556		2.333333333	#DIV/0!
**PDIA4**	0.09523809524	0.09523809524	0.05555555556		1.473684211	#DIV/0!
**PDIA5**	0.04761904762	0.04761904762	0.05555555556		0.7	#DIV/0!
**PDIA6**	0	0	0.05555555556		0	#DIV/0!
**PDIK1L**	0.04761904762	0.04761904762	0.1111111111		0.325	#DIV/0!
**PDILT**	0.04761904762	0.04761904762	0		inf	#DIV/0!
**PDK1**	0	0	0.1111111111		0	#DIV/0!
**PDK2**	0.09523809524	0.09523809524	0		inf	#DIV/0!
**PDK4**	0.04761904762	0.04761904762	0.1111111111		0.325	#DIV/0!
**PDLIM1**	0.1428571429	0.1428571429	0		inf	#DIV/0!
**PDLIM2**	0.09523809524	0.09523809524	0.1111111111		0.6842105263	#DIV/0!
**PDLIM3**	0	0	0.05555555556		0	#DIV/0!
**PDLIM4**	0.04761904762	0.04761904762	0		inf	#DIV/0!
**PDLIM5**	0	0	0.05555555556		0	#DIV/0!
**PDLIM7**	0.09523809524	0.09523809524	0.05555555556		1.473684211	#DIV/0!
**PDP1**	0.1904761905	0.1904761905	0.1111111111		1.529411765	#DIV/0!
**PDP2**	0.09523809524	0.09523809524	0.05555555556		1.473684211	#DIV/0!
**PDPK1**	0.04761904762	0.04761904762	0.1111111111		0.325	#DIV/0!
**PDPN**	0.04761904762	0.04761904762	0.1111111111		0.325	#DIV/0!

PDPR	0.1428571429	0.1428571429	0.05555555556		2.333333333	#DIV/0!
PDRG1	0.09523809524	0.09523809524	0.1111111111		0.6842105263	#DIV/0!
PDS5A	0.1904761905	0.1904761905	0.05555555556		3.294117647	#DIV/0!
PDSS1	0.09523809524	0.09523809524	0.1111111111		0.6842105263	#DIV/0!
PDSS2	0	0	0			#DIV/0!
PDXDC1	0.04761904762	0.04761904762	0		inf	#DIV/0!
C2P-NPI	0.1428571429	0.1428571429	0.05555555556		2.333333333	#DIV/0!
PDXK	0.04761904762	0.04761904762	0.1111111111		0.325	#DIV/0!
PDXP	0	0	0.1111111111		0	#DIV/0!
PDYN	0.1904761905	0.1904761905	0.1111111111		1.529411765	#DIV/0!
DYN-AS	0.1904761905	0.1904761905	0.1111111111		1.529411765	#DIV/0!
PDZD2	0.04761904762	0.04761904762	0.05555555556		0.7	#DIV/0!
PDZD7	0.1428571429	0.1428571429	0		inf	#DIV/0!
PDZD8	0.2380952381	0.2380952381	0		inf	#DIV/0!
PDZD9	0.04761904762	0.04761904762	0		inf	#DIV/0!
PDZK1	0.1428571429	0.1428571429	0.05555555556		2.333333333	#DIV/0!
DZK1IP	0.04761904762	0.04761904762	0.05555555556		0.7	#DIV/0!
PDZK1P	0.1428571429	0.1428571429	0.05555555556		2.333333333	#DIV/0!
PDZRN3	0.04761904762	0.04761904762	0		inf	#DIV/0!
ZRN3-A	0.04761904762	0.04761904762	0		inf	#DIV/0!
PDZRN4	0.04761904762	0.04761904762	0.05555555556		0.7	#DIV/0!
PEA15	0.1428571429	0.1428571429	0		inf	#DIV/0!
PEAK1	0.04761904762	0.04761904762	0.05555555556		0.7	#DIV/0!
PEAK3	0.09523809524	0.09523809524	0.1111111111		0.6842105263	#DIV/0!
PEAR1	0.1428571429	0.1428571429	0.05555555556		2.333333333	#DIV/0!
PEBP1	0	0	0.05555555556		0	#DIV/0!
PEBP4	0.04761904762	0.04761904762	0.1111111111		0.325	#DIV/0!
PECAM1	0.04761904762	0.04761904762	0		inf	#DIV/0!
PECR	0	0	0.05555555556		0	#DIV/0!
PEG10	0.04761904762	0.04761904762	0.1111111111		0.325	#DIV/0!
PEG13	0.04761904762	0.04761904762	0.1111111111		0.325	#DIV/0!
PEG3	0.1428571429	0.1428571429	0.05555555556		2.333333333	#DIV/0!
EG3-AS	0.1428571429	0.1428571429	0.05555555556		2.333333333	#DIV/0!
PELI1	0	0	0.05555555556		0	#DIV/0!
PELI2	0.04761904762	0.04761904762	0		inf	#DIV/0!
PELO	0.04761904762	0.04761904762	0.05555555556		0.7	#DIV/0!
PELP1	0	0	0.1111111111		0	#DIV/0!
PEMT	0.04761904762	0.04761904762	0.1111111111		0.325	#DIV/0!
PENK	0.1428571429	0.1428571429	0.1111111111		1.083333333	#DIV/0!
PEPD	0.04761904762	0.04761904762	0.05555555556		0.7	#DIV/0!
PER1	0	0	0.05555555556		0	#DIV/0!
PER2	0	0	0.05555555556		0	#DIV/0!
PER3	0	0	0.05555555556		0	#DIV/0!
PER4	0.04761904762	0.04761904762	0.2222222222		0.1375	#DIV/0!
PERM1	0	0	0.1111111111		0	#DIV/0!
PES1	0.04761904762	0.04761904762	0.1111111111		0.325	#DIV/0!
PET100	0.1428571429	0.1428571429	0.05555555556		2.333333333	#DIV/0!
PET117	0.1904761905	0.1904761905	0.05555555556		3.294117647	#DIV/0!
PEX1	0.04761904762	0.04761904762	0.1111111111		0.325	#DIV/0!
PEX10	0	0	0.1111111111		0	#DIV/0!
PEX11A	0.04761904762	0.04761904762	0.05555555556		0.7	#DIV/0!

PEX11G	0.1428571429	0.1428571429	0.05555555556		2.333333333	#DIV/0!
PEX12	0.04761904762	0.04761904762	0		inf	#DIV/0!
PEX13	0.09523809524	0.09523809524	0.05555555556		1.473684211	#DIV/0!
PEX14	0.04761904762	0.04761904762	0.1111111111		0.325	#DIV/0!
PEX19	0.1428571429	0.1428571429	0		inf	#DIV/0!
PEX2	0.1904761905	0.1904761905	0.1111111111		1.529411765	#DIV/0!
PEX26	0.04761904762	0.04761904762	0.05555555556		0.7	#DIV/0!
PEX5	0.04761904762	0.04761904762	0.1111111111		0.325	#DIV/0!
PEX5L	0.1904761905	0.1904761905	0.05555555556		3.294117647	#DIV/0!
EX5L-AS	0.1904761905	0.1904761905	0.05555555556		3.294117647	#DIV/0!
PEX6	0.04761904762	0.04761904762	0.05555555556		0.7	#DIV/0!
PF4	0.09523809524	0.09523809524	0.05555555556		1.473684211	#DIV/0!
PF4V1	0.09523809524	0.09523809524	0.05555555556		1.473684211	#DIV/0!
PFAS	0.04761904762	0.04761904762	0.05555555556		0.7	#DIV/0!
PFDN1	0.04761904762	0.04761904762	0		inf	#DIV/0!
PFDN2	0.1428571429	0.1428571429	0		inf	#DIV/0!
PFDN4	0.09523809524	0.09523809524	0.1111111111		0.6842105263	#DIV/0!
PFDN5	0.04761904762	0.04761904762	0.05555555556		0.7	#DIV/0!
PFKFB2	0.1428571429	0.1428571429	0		inf	#DIV/0!
PFKFB3	0.09523809524	0.09523809524	0.1111111111		0.6842105263	#DIV/0!
PFKFB4	0.09523809524	0.09523809524	0		inf	#DIV/0!
PFKL	0.04761904762	0.04761904762	0.1111111111		0.325	#DIV/0!
PFKM	0.04761904762	0.04761904762	0.05555555556		0.7	#DIV/0!
PFKP	0.04761904762	0.04761904762	0.1666666667		0.2	#DIV/0!
PFN1	0	0	0.1111111111		0	#DIV/0!
PFN1P2	0.09523809524	0.09523809524	0.05555555556		1.473684211	#DIV/0!
PFN2	0.1904761905	0.1904761905	0.1111111111		1.529411765	#DIV/0!
PFN3	0.09523809524	0.09523809524	0.05555555556		1.473684211	#DIV/0!
PGAM1	0.1428571429	0.1428571429	0		inf	#DIV/0!
GAM1P	0.04761904762	0.04761904762	0.05555555556		0.7	#DIV/0!
PGAM2	0.04761904762	0.04761904762	0.1666666667		0.2	#DIV/0!
PGAM5	0.09523809524	0.09523809524	0.05555555556		1.473684211	#DIV/0!
PGAP1	0	0	0.05555555556		0	#DIV/0!
PGAP2	0.04761904762	0.04761904762	0		inf	#DIV/0!
PGAP3	0.09523809524	0.09523809524	0		inf	#DIV/0!
PGBD2	0.1428571429	0.1428571429	0		inf	#DIV/0!
PGBD3	0.09523809524	0.09523809524	0.05555555556		1.473684211	#DIV/0!
PGBD5	0.1428571429	0.1428571429	0		inf	#DIV/0!
PGC	0.04761904762	0.04761904762	0.05555555556		0.7	#DIV/0!
PGCP1	0.04761904762	0.04761904762	0		inf	#DIV/0!
PGD	0.04761904762	0.04761904762	0.1111111111		0.325	#DIV/0!
PGGHG	0.04761904762	0.04761904762	0		inf	#DIV/0!
PGGT1B	0.04761904762	0.04761904762	0		inf	#DIV/0!
PGK2	0.04761904762	0.04761904762	0		inf	#DIV/0!
PGM1	0.04761904762	0.04761904762	0.05555555556		0.7	#DIV/0!
PGM5	0.09523809524	0.09523809524	0.1666666667		0.4210526316	#DIV/0!
GM5-AS	0.09523809524	0.09523809524	0.1666666667		0.4210526316	#DIV/0!
PGM5P2	0.09523809524	0.09523809524	0.2222222222		0.2894736842	#DIV/0!
M5P3-A	0.04761904762	0.04761904762	0.1111111111		0.325	#DIV/0!
PGM5P4	0	0	0.05555555556		0	#DIV/0!
M5P4-A	0	0	0.05555555556		0	#DIV/0!

PGP	0.09523809524	0.09523809524	0.2222222222		0.2894736842	#DIV/0!
PGPEP1	0.1428571429	0.1428571429	0.1111111111		1.083333333	#DIV/0!
PGPEP1I	0.04761904762	0.04761904762	0.05555555556		0.7	#DIV/0!
PGRMC2	0	0	0.05555555556		0	#DIV/0!
PGS1	0.04761904762	0.04761904762	0		inf	#DIV/0!
HACTR	0.1428571429	0.1428571429	0.1666666667		0.6666666667	#DIV/0!
HACTR	0.04761904762	0.04761904762	0.1111111111		0.325	#DIV/0!
PHAX	0.04761904762	0.04761904762	0		inf	#DIV/0!
PHB	0.1428571429	0.1428571429	0		inf	#DIV/0!
PHB2	0.04761904762	0.04761904762	0.1111111111		0.325	#DIV/0!
PHC1	0.04761904762	0.04761904762	0.1111111111		0.325	#DIV/0!
PHC2	0.04761904762	0.04761904762	0.05555555556		0.7	#DIV/0!
PHC3	0.1904761905	0.1904761905	0.05555555556		3.294117647	#DIV/0!
PHETA1	0	0	0.1111111111		0	#DIV/0!
PHETA2	0	0	0.1111111111		0	#DIV/0!
PHF1	0.04761904762	0.04761904762	0		inf	#DIV/0!
PHF12	0.04761904762	0.04761904762	0.05555555556		0.7	#DIV/0!
PHF13	0	0	0.05555555556		0	#DIV/0!
PHF14	0.04761904762	0.04761904762	0.2222222222		0.1375	#DIV/0!
PHF19	0.09523809524	0.09523809524	0.05555555556		1.473684211	#DIV/0!
PHF2	0.04761904762	0.04761904762	0.05555555556		0.7	#DIV/0!
PHF20	0.09523809524	0.09523809524	0.1111111111		0.6842105263	#DIV/0!
PHF20L1	0.04761904762	0.04761904762	0.1666666667		0.2	#DIV/0!
PHF21B	0	0	0.1666666667		0	#DIV/0!
PHF23	0	0	0.05555555556		0	#DIV/0!
PHF24	0.09523809524	0.09523809524	0.05555555556		1.473684211	#DIV/0!
PHF5A	0	0	0.1111111111		0	#DIV/0!
PHF7	0.04761904762	0.04761904762	0		inf	#DIV/0!
PHGDH	0.04761904762	0.04761904762	0.05555555556		0.7	#DIV/0!
PHKB	0.04761904762	0.04761904762	0		inf	#DIV/0!
PHKG1	0.2380952381	0.2380952381	0.1666666667		1.25	#DIV/0!
PHKG2	0.04761904762	0.04761904762	0		inf	#DIV/0!
PHLDA1	0.04761904762	0.04761904762	0.1111111111		0.325	#DIV/0!
PHLDA2	0.04761904762	0.04761904762	0		inf	#DIV/0!
PHLDA3	0.1428571429	0.1428571429	0		inf	#DIV/0!
PHLDB2	0.09523809524	0.09523809524	0.05555555556		1.473684211	#DIV/0!
PHLDB3	0.09523809524	0.09523809524	0.05555555556		1.473684211	#DIV/0!
PHLPP1	0.09523809524	0.09523809524	0		inf	#DIV/0!
PHLPP2	0.04761904762	0.04761904762	0		inf	#DIV/0!
HOSPHO	0.1428571429	0.1428571429	0		inf	#DIV/0!
HOSPHO	0	0	0.05555555556		0	#DIV/0!
PHO2-KI	0	0	0.05555555556		0	#DIV/0!
PHOX2B	0.04761904762	0.04761904762	0		inf	#DIV/0!
PHPT1	0.1428571429	0.1428571429	0.05555555556		2.333333333	#DIV/0!
PHRF1	0.04761904762	0.04761904762	0		inf	#DIV/0!
PHTF1	0.04761904762	0.04761904762	0.05555555556		0.7	#DIV/0!
PHTF2	0.04761904762	0.04761904762	0.1111111111		0.325	#DIV/0!
PHYH	0.09523809524	0.09523809524	0.1111111111		0.6842105263	#DIV/0!
PHYHIP	0.09523809524	0.09523809524	0.1111111111		0.6842105263	#DIV/0!
HYHIPI	0.04761904762	0.04761904762	0.05555555556		0.7	#DIV/0!
PHYKPI	0.09523809524	0.09523809524	0.05555555556		1.473684211	#DIV/0!

**PI15**	0.1904761905	0.1904761905	0.1111111111		1.529411765	#DIV/0!
**PI16**	0.04761904762	0.04761904762	0.05555555556		0.7	#DIV/0!
**PI3**	0.1428571429	0.1428571429	0.1666666667		0.6666666667	#DIV/0!
**PI4K2A**	0.1428571429	0.1428571429	0		inf	#DIV/0!
**PI4KA**	0.04761904762	0.04761904762	0.05555555556		0.7	#DIV/0!
**PI4KAP1**	0.04761904762	0.04761904762	0.05555555556		0.7	#DIV/0!
**PI4KAP2**	0.04761904762	0.04761904762	0.05555555556		0.7	#DIV/0!
**PI4KB**	0.1904761905	0.1904761905	0.05555555556		3.294117647	#DIV/0!
**PIANP**	0.04761904762	0.04761904762	0.1111111111		0.325	#DIV/0!
**PIAS1**	0.04761904762	0.04761904762	0.05555555556		0.7	#DIV/0!
**PIAS2**	0.04761904762	0.04761904762	0.05555555556		0.7	#DIV/0!
**PIAS3**	0.1428571429	0.1428571429	0.05555555556		2.333333333	#DIV/0!
**PIAS4**	0.09523809524	0.09523809524	0.05555555556		1.473684211	#DIV/0!
**PICART**	0.09523809524	0.09523809524	0		inf	#DIV/0!
**PICK1**	0.04761904762	0.04761904762	0.1111111111		0.325	#DIV/0!
**PICSAR**	0.04761904762	0.04761904762	0.1111111111		0.325	#DIV/0!
**PID1**	0	0	0.05555555556		0	#DIV/0!
**PIDD1**	0.04761904762	0.04761904762	0		inf	#DIV/0!
**PIEZO1**	0.1904761905	0.1904761905	0		inf	#DIV/0!
**PIEZO2**	0.09523809524	0.09523809524	0		inf	#DIV/0!
**PIF1**	0.04761904762	0.04761904762	0.05555555556		0.7	#DIV/0!
**PIFO**	0.04761904762	0.04761904762	0		inf	#DIV/0!
**PIGB**	0.04761904762	0.04761904762	0.05555555556		0.7	#DIV/0!
**PIGBOS1**	0.04761904762	0.04761904762	0.05555555556		0.7	#DIV/0!
**PIGC**	0.1428571429	0.1428571429	0.05555555556		2.333333333	#DIV/0!
**PIGF**	0	0	0.05555555556		0	#DIV/0!
**PIGG**	0.04761904762	0.04761904762	0		inf	#DIV/0!
**PIGK**	0.04761904762	0.04761904762	0.05555555556		0.7	#DIV/0!
**PIGM**	0.1428571429	0.1428571429	0		inf	#DIV/0!
**PIGN**	0.09523809524	0.09523809524	0		inf	#DIV/0!
**PIGO**	0.09523809524	0.09523809524	0.05555555556		1.473684211	#DIV/0!
**PIGP**	0.04761904762	0.04761904762	0.1666666667		0.2	#DIV/0!
**PIGQ**	0.04761904762	0.04761904762	0.2222222222		0.1375	#DIV/0!
**PIGR**	0.1428571429	0.1428571429	0		inf	#DIV/0!
**PIGS**	0.04761904762	0.04761904762	0.05555555556		0.7	#DIV/0!
**PIGT**	0.1428571429	0.1428571429	0.1666666667		0.6666666667	#DIV/0!
**PIGU**	0.09523809524	0.09523809524	0.1111111111		0.6842105263	#DIV/0!
**PIGV**	0.04761904762	0.04761904762	0.1111111111		0.325	#DIV/0!
**PIGW**	0.04761904762	0.04761904762	0		inf	#DIV/0!
**PIGX**	0.1428571429	0.1428571429	0.05555555556		2.333333333	#DIV/0!
**PIGY**	0	0	0.05555555556		0	#DIV/0!
**PIGY-DT**	0	0	0.05555555556		0	#DIV/0!
**PIGZ**	0.1428571429	0.1428571429	0.05555555556		2.333333333	#DIV/0!
**PIH1D1**	0.09523809524	0.09523809524	0.1111111111		0.6842105263	#DIV/0!
**PIK3AP1**	0.1428571429	0.1428571429	0		inf	#DIV/0!
**PIK3C2B**	0.2380952381	0.2380952381	0		inf	#DIV/0!
**PIK3C2G**	0.04761904762	0.04761904762	0.1111111111		0.325	#DIV/0!
**PIK3C3**	0.04761904762	0.04761904762	0		inf	#DIV/0!
**PIK3CA**	0.1904761905	0.1904761905	0.05555555556		3.294117647	#DIV/0!
**PIK3CB**	0.1428571429	0.1428571429	0.05555555556		2.333333333	#DIV/0!
**PIK3CD**	0	0	0.05555555556		0	#DIV/0!

K3CD-A	0	0	0.05555555556		0	#DIV/0!
K3CD-A	0	0	0.05555555556		0	#DIV/0!
PIK3CG	0.04761904762	0.04761904762	0.05555555556		0.7	#DIV/0!
PIK3IP1	0.04761904762	0.04761904762	0.1111111111		0.325	#DIV/0!
K3IP1-A	0.04761904762	0.04761904762	0.1111111111		0.325	#DIV/0!
PIK3R1	0.04761904762	0.04761904762	0		inf	#DIV/0!
PIK3R2	0.1428571429	0.1428571429	0.1111111111		1.083333333	#DIV/0!
PIK3R3	0.04761904762	0.04761904762	0.05555555556		0.7	#DIV/0!
PIK3R4	0.09523809524	0.09523809524	0.05555555556		1.473684211	#DIV/0!
PIK3R5	0.04761904762	0.04761904762	0.05555555556		0.7	#DIV/0!
PIK3R6	0.04761904762	0.04761904762	0.05555555556		0.7	#DIV/0!
PIKFYVE	0	0	0.05555555556		0	#DIV/0!
PILRA	0.04761904762	0.04761904762	0.05555555556		0.7	#DIV/0!
PILRB	0.04761904762	0.04761904762	0.05555555556		0.7	#DIV/0!
PIM1	0.04761904762	0.04761904762	0.05555555556		0.7	#DIV/0!
PIM3	0	0	0.1111111111		0	#DIV/0!
PIMREG	0	0	0.05555555556		0	#DIV/0!
PIN1	0.1428571429	0.1428571429	0.1111111111		1.083333333	#DIV/0!
PIN1P1	0.04761904762	0.04761904762	0.05555555556		0.7	#DIV/0!
PINK1	0.04761904762	0.04761904762	0.1111111111		0.325	#DIV/0!
INK1-A	0.04761904762	0.04761904762	0.1111111111		0.325	#DIV/0!
PINLYP	0.09523809524	0.09523809524	0.05555555556		1.473684211	#DIV/0!
PINX1	0.09523809524	0.09523809524	0.1111111111		0.6842105263	#DIV/0!
PIP	0.04761904762	0.04761904762	0.05555555556		0.7	#DIV/0!
PIP4K2A	0.09523809524	0.09523809524	0.1111111111		0.6842105263	#DIV/0!
PIP4K2B	0.09523809524	0.09523809524	0		inf	#DIV/0!
PIP4K2C	0.1428571429	0.1428571429	0.05555555556		2.333333333	#DIV/0!
PIP4P2	0.1904761905	0.1904761905	0.1111111111		1.529411765	#DIV/0!
PIP5K1A	0.1904761905	0.1904761905	0.05555555556		3.294117647	#DIV/0!
PIP5K1B	0	0	0.05555555556		0	#DIV/0!
PIP5K1C	0.09523809524	0.09523809524	0.05555555556		1.473684211	#DIV/0!
PIP5KL1	0.09523809524	0.09523809524	0.05555555556		1.473684211	#DIV/0!
PIPOX	0.04761904762	0.04761904762	0.05555555556		0.7	#DIV/0!
PIPSL	0.1428571429	0.1428571429	0		inf	#DIV/0!
PIRT	0.04761904762	0.04761904762	0.05555555556		0.7	#DIV/0!
PISD	0.04761904762	0.04761904762	0.1111111111		0.325	#DIV/0!
PISRT1	0.1428571429	0.1428571429	0.05555555556		2.333333333	#DIV/0!
PITHD1	0.04761904762	0.04761904762	0.1111111111		0.325	#DIV/0!
PITPNA	0	0	0.1111111111		0	#DIV/0!
TPNA-A	0	0	0.1111111111		0	#DIV/0!
PITPNB	0.04761904762	0.04761904762	0.1111111111		0.325	#DIV/0!
PITPNC1	0	0	0			#DIV/0!
PITPNM1	0.04761904762	0.04761904762	0.1111111111		0.325	#DIV/0!
TPNM2-A	0.04761904762	0.04761904762	0.1111111111		0.325	#DIV/0!
PITPNM3	0	0	0.05555555556		0	#DIV/0!
PITRM1	0.04761904762	0.04761904762	0.1666666667		0.2	#DIV/0!
TRM1-A	0.04761904762	0.04761904762	0.1666666667		0.2	#DIV/0!
PITX2	0	0	0.05555555556		0	#DIV/0!
PITX3	0.1428571429	0.1428571429	0.05555555556		2.333333333	#DIV/0!
PIWIL1	0.09523809524	0.09523809524	0.05555555556		1.473684211	#DIV/0!
PIWIL2	0.09523809524	0.09523809524	0.1111111111		0.6842105263	#DIV/0!

PIWIL3	0.04761904762	0.04761904762	0.05555555556		0.7	#DIV/0!
PJA2	0.04761904762	0.04761904762	0		inf	#DIV/0!
PJVK	0	0	0.1111111111		0	#DIV/0!
PKD1	0.09523809524	0.09523809524	0.2222222222		0.2894736842	#DIV/0!
PKD1L1	0.04761904762	0.04761904762	0.2222222222		0.1375	#DIV/0!
PKD1L2	0.09523809524	0.09523809524	0		inf	#DIV/0!
PKD1L3	0.04761904762	0.04761904762	0		inf	#DIV/0!
PKD1P1	0.04761904762	0.04761904762	0		inf	#DIV/0!
1P3-NPI	0.04761904762	0.04761904762	0		inf	#DIV/0!
1P4-NPI	0.09523809524	0.09523809524	0		inf	#DIV/0!
-LOC10	0.09523809524	0.09523809524	0		inf	#DIV/0!
1P6-NPI	0.04761904762	0.04761904762	0		inf	#DIV/0!
PKD2	0	0	0.05555555556		0	#DIV/0!
PKD2L1	0.1428571429	0.1428571429	0		inf	#DIV/0!
PKD2L2	0.04761904762	0.04761904762	0		inf	#DIV/0!
PKDCC	0	0	0.05555555556		0	#DIV/0!
KDREJ	0	0	0.1111111111		0	#DIV/0!
PKHD1	0.04761904762	0.04761904762	0		inf	#DIV/0!
KHD1L	0.1904761905	0.1904761905	0.1111111111		1.529411765	#DIV/0!
PKIA	0.1428571429	0.1428571429	0.1111111111		1.083333333	#DIV/0!
KIA-AS	0.1428571429	0.1428571429	0.1111111111		1.083333333	#DIV/0!
PKIG	0.1428571429	0.1428571429	0.1666666667		0.6666666667	#DIV/0!
PKLR	0.1904761905	0.1904761905	0.1111111111		1.529411765	#DIV/0!
PKM	0.04761904762	0.04761904762	0.05555555556		0.7	#DIV/0!
KMYT1	0.04761904762	0.04761904762	0.1111111111		0.325	#DIV/0!
PKN1	0.1428571429	0.1428571429	0.1111111111		1.083333333	#DIV/0!
PKN2	0.04761904762	0.04761904762	0.05555555556		0.7	#DIV/0!
KN2-AS	0.04761904762	0.04761904762	0.05555555556		0.7	#DIV/0!
PKN3	0.09523809524	0.09523809524	0.05555555556		1.473684211	#DIV/0!
KNOX1	0.04761904762	0.04761904762	0.1666666667		0.2	#DIV/0!
PKP1	0.1428571429	0.1428571429	0		inf	#DIV/0!
PKP2	0.1428571429	0.1428571429	0.1111111111		1.083333333	#DIV/0!
PKP3	0.04761904762	0.04761904762	0		inf	#DIV/0!
PKP4	0	0	0.05555555556		0	#DIV/0!
KP4-AS	0	0	0.05555555556		0	#DIV/0!
PLA1A	0.09523809524	0.09523809524	0.05555555556		1.473684211	#DIV/0!
LA2G10	0.04761904762	0.04761904762	0		inf	#DIV/0!
LA2G12	0	0	0.05555555556		0	#DIV/0!
LA2G12	0.1904761905	0.1904761905	0.05555555556		3.294117647	#DIV/0!
LA2G15	0.1428571429	0.1428571429	0.05555555556		2.333333333	#DIV/0!
LA2G1I	0	0	0.1111111111		0	#DIV/0!
LA2G2A	0.04761904762	0.04761904762	0.1111111111		0.325	#DIV/0!
LA2G2C	0.04761904762	0.04761904762	0.1111111111		0.325	#DIV/0!
LA2G2I	0.04761904762	0.04761904762	0.1111111111		0.325	#DIV/0!
LA2G2I	0.04761904762	0.04761904762	0.1111111111		0.325	#DIV/0!
LA2G2I	0.04761904762	0.04761904762	0.1111111111		0.325	#DIV/0!
PLA2G3	0.04761904762	0.04761904762	0.1111111111		0.325	#DIV/0!
LA2G4A	0.1428571429	0.1428571429	0.05555555556		2.333333333	#DIV/0!
LA2G4C	0.09523809524	0.09523809524	0.1111111111		0.6842105263	#DIV/0!
A2G4C-A	0.09523809524	0.09523809524	0.1111111111		0.6842105263	#DIV/0!
PLA2G5	0.04761904762	0.04761904762	0.1111111111		0.325	#DIV/0!

PLA2G6	0.04761904762	0.04761904762	0.1111111111		0.325	#DIV/0!
PLA2G7	0.04761904762	0.04761904762	0.05555555556		0.7	#DIV/0!
PLA2R1	0	0	0.05555555556		0	#DIV/0!
PLAA	0.04761904762	0.04761904762	0.05555555556		0.7	#DIV/0!
PLAAT1	0.09523809524	0.09523809524	0.05555555556		1.473684211	#DIV/0!
PLAC4	0.04761904762	0.04761904762	0.1111111111		0.325	#DIV/0!
PLAC8	0	0	0.05555555556		0	#DIV/0!
PLAC9	0.1428571429	0.1428571429	0		inf	#DIV/0!
PLAC9P	0	0	0.05555555556		0	#DIV/0!
PLAG1	0.1428571429	0.1428571429	0.1111111111		1.083333333	#DIV/0!
PLAT	0.09523809524	0.09523809524	0.1666666667		0.4210526316	#DIV/0!
PLAU	0.1904761905	0.1904761905	0.05555555556		3.294117647	#DIV/0!
PLAUR	0.09523809524	0.09523809524	0.05555555556		1.473684211	#DIV/0!
PLB1	0	0	0.05555555556		0	#DIV/0!
PLBD1	0.04761904762	0.04761904762	0.1111111111		0.325	#DIV/0!
LBD1-AS	0.04761904762	0.04761904762	0.1111111111		0.325	#DIV/0!
PLBD2	0	0	0.05555555556		0	#DIV/0!
PLCB1	0.1904761905	0.1904761905	0.1666666667		0.9411764706	#DIV/0!
LCB1-IT	0.1904761905	0.1904761905	0.1666666667		0.9411764706	#DIV/0!
PLCB4	0.1904761905	0.1904761905	0.1111111111		1.529411765	#DIV/0!
PLCD1	0.04761904762	0.04761904762	0		inf	#DIV/0!
PLCD3	0.1428571429	0.1428571429	0		inf	#DIV/0!
PLCD4	0	0	0.05555555556		0	#DIV/0!
PLCE1	0.1428571429	0.1428571429	0		inf	#DIV/0!
LCE1-AS	0.1428571429	0.1428571429	0		inf	#DIV/0!
LCE1-AS	0.1428571429	0.1428571429	0		inf	#DIV/0!
PLCG1	0.1428571429	0.1428571429	0.1666666667		0.6666666667	#DIV/0!
LCG1-AS	0.1428571429	0.1428571429	0.1666666667		0.6666666667	#DIV/0!
PLCG2	0.04761904762	0.04761904762	0		inf	#DIV/0!
PLCH1	0.1904761905	0.1904761905	0.05555555556		3.294117647	#DIV/0!
LCH1-AS	0.1904761905	0.1904761905	0.05555555556		3.294117647	#DIV/0!
PLCH2	0	0	0.1111111111		0	#DIV/0!
PLCL1	0	0	0.05555555556		0	#DIV/0!
PLCL2	0.04761904762	0.04761904762	0		inf	#DIV/0!
PLCXD2	0.09523809524	0.09523809524	0.05555555556		1.473684211	#DIV/0!
CXD2-A	0.09523809524	0.09523809524	0.05555555556		1.473684211	#DIV/0!
PLCXD3	0.04761904762	0.04761904762	0.05555555556		0.7	#DIV/0!
PLCZ1	0.04761904762	0.04761904762	0.1111111111		0.325	#DIV/0!
PLD1	0.1904761905	0.1904761905	0.1111111111		1.529411765	#DIV/0!
PLD3	0.04761904762	0.04761904762	0.05555555556		0.7	#DIV/0!
PLD5	0.1428571429	0.1428571429	0		inf	#DIV/0!
PLD6	0.04761904762	0.04761904762	0.1111111111		0.325	#DIV/0!
PLEC	0.04761904762	0.04761904762	0.1666666667		0.2	#DIV/0!
PLEK	0	0	0.05555555556		0	#DIV/0!
LEKHA	0.1904761905	0.1904761905	0.05555555556		3.294117647	#DIV/0!
LEKHA	0.09523809524	0.09523809524	0.1111111111		0.6842105263	#DIV/0!
LEKHA	0	0	0.1111111111		0	#DIV/0!
LEKHA	0.09523809524	0.09523809524	0.1111111111		0.6842105263	#DIV/0!
LEKHA	0.04761904762	0.04761904762	0.1111111111		0.325	#DIV/0!
LEKHA	0.2380952381	0.2380952381	0		inf	#DIV/0!
LEKHA	0.04761904762	0.04761904762	0.1666666667		0.2	#DIV/0!

EKHA8	0.04761904762	0.04761904762	0.05555555556		0.7	#DIV/0!
LEKHB	0	0	0.05555555556		0	#DIV/0!
LEKHF	0.04761904762	0.04761904762	0.1666666667		0.2	#DIV/0!
LEKHF	0.1904761905	0.1904761905	0.1111111111		1.529411765	#DIV/0!
LEKHG	0.04761904762	0.04761904762	0.05555555556		0.7	#DIV/0!
LEKHG	0.1428571429	0.1428571429	0.05555555556		2.333333333	#DIV/0!
LEKHG4	0	0	0.05555555556		0	#DIV/0!
LEKHG	0	0	0.05555555556		0	#DIV/0!
LEKHG	0.04761904762	0.04761904762	0.1111111111		0.325	#DIV/0!
LEKHG	0.04761904762	0.04761904762	0.05555555556		0.7	#DIV/0!
LEKHH	0	0	0.05555555556		0	#DIV/0!
LEKHH	0.04761904762	0.04761904762	0		inf	#DIV/0!
PLEKHJ	0.09523809524	0.09523809524	0.1111111111		0.6842105263	#DIV/0!
LEKHM	0.1428571429	0.1428571429	0		inf	#DIV/0!
EKHM1	0.04761904762	0.04761904762	0		inf	#DIV/0!
LEKHM	0.04761904762	0.04761904762	0.1111111111		0.325	#DIV/0!
LEKHM	0	0	0.05555555556		0	#DIV/0!
LEKHN	0	0	0.1111111111		0	#DIV/0!
LEKHO	0.1904761905	0.1904761905	0.05555555556		3.294117647	#DIV/0!
LEKHO	0.04761904762	0.04761904762	0.05555555556		0.7	#DIV/0!
LEKHS	0.1428571429	0.1428571429	0.05555555556		2.333333333	#DIV/0!
PLGRKT	0.04761904762	0.04761904762	0.05555555556		0.7	#DIV/0!
PLIN1	0.04761904762	0.04761904762	0.05555555556		0.7	#DIV/0!
PLIN2	0.04761904762	0.04761904762	0.05555555556		0.7	#DIV/0!
PLIN3	0.09523809524	0.09523809524	0.05555555556		1.473684211	#DIV/0!
PLIN4	0.09523809524	0.09523809524	0.05555555556		1.473684211	#DIV/0!
PLIN5	0.09523809524	0.09523809524	0.05555555556		1.473684211	#DIV/0!
PLK1	0.04761904762	0.04761904762	0		inf	#DIV/0!
PLK2	0.04761904762	0.04761904762	0		inf	#DIV/0!
PLK3	0.04761904762	0.04761904762	0.05555555556		0.7	#DIV/0!
PLK4	0	0	0.05555555556		0	#DIV/0!
PLK5	0.09523809524	0.09523809524	0.1111111111		0.6842105263	#DIV/0!
PLLP	0.04761904762	0.04761904762	0		inf	#DIV/0!
PLOD1	0.04761904762	0.04761904762	0.1111111111		0.325	#DIV/0!
PLOD2	0.1428571429	0.1428571429	0.1111111111		1.083333333	#DIV/0!
PLOD3	0.04761904762	0.04761904762	0.05555555556		0.7	#DIV/0!
PLPBP	0.04761904762	0.04761904762	0.1111111111		0.325	#DIV/0!
PLPP1	0.04761904762	0.04761904762	0.05555555556		0.7	#DIV/0!
PLPP2	0.09523809524	0.09523809524	0.1111111111		0.6842105263	#DIV/0!
PLPP3	0.04761904762	0.04761904762	0.05555555556		0.7	#DIV/0!
PLPP4	0.2380952381	0.2380952381	0		inf	#DIV/0!
PLPP5	0.09523809524	0.09523809524	0.1111111111		0.6842105263	#DIV/0!
PLPP6	0.04761904762	0.04761904762	0.05555555556		0.7	#DIV/0!
PLPP7	0.09523809524	0.09523809524	0.05555555556		1.473684211	#DIV/0!
PLPPR1	0.04761904762	0.04761904762	0		inf	#DIV/0!
PLPPR3	0.09523809524	0.09523809524	0.1111111111		0.6842105263	#DIV/0!
PLPPR4	0.04761904762	0.04761904762	0.05555555556		0.7	#DIV/0!
PLPPR5	0.04761904762	0.04761904762	0.05555555556		0.7	#DIV/0!
PLRG1	0	0	0.05555555556		0	#DIV/0!
PLS1	0.2380952381	0.2380952381	0.05555555556		4.375	#DIV/0!
PLSCR1	0.1428571429	0.1428571429	0.1111111111		1.083333333	#DIV/0!

**PLSCR2**	0.1428571429	0.1428571429	0.1111111111		1.083333333	#DIV/0!
**PLSCR3**	0	0	0.05555555556		0	#DIV/0!
**PLSCR4**	0.1428571429	0.1428571429	0.1111111111		1.083333333	#DIV/0!
**PLSCR5**	0.1428571429	0.1428571429	0.1111111111		1.083333333	#DIV/0!
**PLTP**	0.1428571429	0.1428571429	0.1666666667		0.6666666667	#DIV/0!
**PLVAP**	0.1428571429	0.1428571429	0.1111111111		1.083333333	#DIV/0!
**PLXDC1**	0.09523809524	0.09523809524	0		inf	#DIV/0!
**PLXDC2**	0.09523809524	0.09523809524	0.1111111111		0.6842105263	#DIV/0!
**PLXNA1**	0.09523809524	0.09523809524	0.05555555556		1.473684211	#DIV/0!
**PLXNA2**	0.1428571429	0.1428571429	0		inf	#DIV/0!
**PLXNA4**	0.04761904762	0.04761904762	0.05555555556		0.7	#DIV/0!
**PLXNB1**	0.09523809524	0.09523809524	0		inf	#DIV/0!
**PLXNB2**	0	0	0.1111111111		0	#DIV/0!
**PLXNC1**	0.04761904762	0.04761904762	0.05555555556		0.7	#DIV/0!
**PLXND1**	0.09523809524	0.09523809524	0.05555555556		1.473684211	#DIV/0!
**PM20D1**	0.1428571429	0.1428571429	0		inf	#DIV/0!
**PMAIP1**	0.09523809524	0.09523809524	0		inf	#DIV/0!
**PMCH**	0.04761904762	0.04761904762	0.05555555556		0.7	#DIV/0!
**PMCHL1**	0.04761904762	0.04761904762	0.1111111111		0.325	#DIV/0!
**PMCHL2**	0.04761904762	0.04761904762	0.05555555556		0.7	#DIV/0!
**PMEL**	0.04761904762	0.04761904762	0.05555555556		0.7	#DIV/0!
**PMEPA1**	0.1428571429	0.1428571429	0.2222222222		0.4583333333	#DIV/0!
**PMFBP1**	0.04761904762	0.04761904762	0		inf	#DIV/0!
**PML**	0.04761904762	0.04761904762	0.05555555556		0.7	#DIV/0!
**PMM1**	0	0	0.1111111111		0	#DIV/0!
**PMM2**	0.09523809524	0.09523809524	0		inf	#DIV/0!
**PMP2**	0.1428571429	0.1428571429	0.1111111111		1.083333333	#DIV/0!
**PMP22**	0.04761904762	0.04761904762	0.05555555556		0.7	#DIV/0!
**PMPCA**	0.1428571429	0.1428571429	0.05555555556		2.333333333	#DIV/0!
**PMPCB**	0.04761904762	0.04761904762	0.05555555556		0.7	#DIV/0!
**PMS1**	0	0	0.05555555556		0	#DIV/0!
**PMS2**	0.04761904762	0.04761904762	0.1666666667		0.2	#DIV/0!
**PMS2CL**	0.04761904762	0.04761904762	0.1666666667		0.2	#DIV/0!
**PMS2P1**	0.04761904762	0.04761904762	0.05555555556		0.7	#DIV/0!
**PMS2P2**	0.1428571429	0.1428571429	0.1111111111		1.083333333	#DIV/0!
**PMS2P3**	0.1428571429	0.1428571429	0.1666666667		0.6666666667	#DIV/0!
**PMS2P4**	0.04761904762	0.04761904762	0.1111111111		0.325	#DIV/0!
**PMS2P5**	0.1428571429	0.1428571429	0.1666666667		0.6666666667	#DIV/0!
**PMS2P7**	0.1428571429	0.1428571429	0.1666666667		0.6666666667	#DIV/0!
**PMS2P9**	0.04761904762	0.04761904762	0.1111111111		0.325	#DIV/0!
**PMVK**	0.1904761905	0.1904761905	0.1111111111		1.529411765	#DIV/0!
**PNKD**	0	0	0.05555555556		0	#DIV/0!
**PNKP**	0.09523809524	0.09523809524	0.1111111111		0.6842105263	#DIV/0!
**PNLIP**	0.2380952381	0.2380952381	0		inf	#DIV/0!
**NLIPRP**	0.2380952381	0.2380952381	0		inf	#DIV/0!
**NLIPRP**	0.2380952381	0.2380952381	0		inf	#DIV/0!
**NLIPRP**	0.2380952381	0.2380952381	0		inf	#DIV/0!
**PNMA1**	0.09523809524	0.09523809524	0		inf	#DIV/0!
**PNMA2**	0.04761904762	0.04761904762	0.1111111111		0.325	#DIV/0!
**PNMA8A**	0.09523809524	0.09523809524	0.1111111111		0.6842105263	#DIV/0!
**PNMA8B**	0.09523809524	0.09523809524	0.1111111111		0.6842105263	#DIV/0!

PNMT	0.09523809524	0.09523809524	0		inf	#DIV/0!
PNN	0.04761904762	0.04761904762	0		inf	#DIV/0!
PNO1	0	0	0.05555555556		0	#DIV/0!
PNOC	0.04761904762	0.04761904762	0.1111111111		0.325	#DIV/0!
PNPLA1	0.04761904762	0.04761904762	0.05555555556		0.7	#DIV/0!
PNPLA2	0.04761904762	0.04761904762	0		inf	#DIV/0!
PNPLA3	0	0	0.1666666667		0	#DIV/0!
PNPLA5	0	0	0.1666666667		0	#DIV/0!
PNPLA6	0.1428571429	0.1428571429	0.05555555556		2.333333333	#DIV/0!
PNPLA7	0.09523809524	0.09523809524	0		inf	#DIV/0!
PNPLA8	0.04761904762	0.04761904762	0.05555555556		0.7	#DIV/0!
PNPO	0.1428571429	0.1428571429	0		inf	#DIV/0!
PNPT1	0.04761904762	0.04761904762	0.05555555556		0.7	#DIV/0!
PNRC2	0.04761904762	0.04761904762	0.1111111111		0.325	#DIV/0!
POC1A	0.04761904762	0.04761904762	0		inf	#DIV/0!
POC1B	0.04761904762	0.04761904762	0.05555555556		0.7	#DIV/0!
)C1B-AS	0.04761904762	0.04761904762	0.05555555556		0.7	#DIV/0!
1B-GAL	0.04761904762	0.04761904762	0.05555555556		0.7	#DIV/0!
POC5	0.04761904762	0.04761904762	0		inf	#DIV/0!
PODN	0.04761904762	0.04761904762	0.05555555556		0.7	#DIV/0!
PODNL1	0.1428571429	0.1428571429	0.1111111111		1.083333333	#DIV/0!
PODXL	0.04761904762	0.04761904762	0.05555555556		0.7	#DIV/0!
POFUT1	0.09523809524	0.09523809524	0.1111111111		0.6842105263	#DIV/0!
POFUT2	0.04761904762	0.04761904762	0.1111111111		0.325	#DIV/0!
POGK	0.1428571429	0.1428571429	0		inf	#DIV/0!
POGZ	0.1904761905	0.1904761905	0.05555555556		3.294117647	#DIV/0!
POLB	0.09523809524	0.09523809524	0.1666666667		0.4210526316	#DIV/0!
POLD1	0.09523809524	0.09523809524	0.1111111111		0.6842105263	#DIV/0!
POLD2	0.04761904762	0.04761904762	0.1666666667		0.2	#DIV/0!
POLDIP2	0.04761904762	0.04761904762	0.05555555556		0.7	#DIV/0!
POLE	0.09523809524	0.09523809524	0.05555555556		1.473684211	#DIV/0!
POLE2	0.04761904762	0.04761904762	0		inf	#DIV/0!
POLE3	0.04761904762	0.04761904762	0.05555555556		0.7	#DIV/0!
POLE4	0	0	0.05555555556		0	#DIV/0!
POLG	0.04761904762	0.04761904762	0.05555555556		0.7	#DIV/0!
POLG2	0.04761904762	0.04761904762	0		inf	#DIV/0!
POLH	0.04761904762	0.04761904762	0.05555555556		0.7	#DIV/0!
POLI	0.09523809524	0.09523809524	0		inf	#DIV/0!
POLK	0.04761904762	0.04761904762	0		inf	#DIV/0!
POLL	0.1428571429	0.1428571429	0		inf	#DIV/0!
POLM	0.04761904762	0.04761904762	0.1666666667		0.2	#DIV/0!
POLN	0.04761904762	0.04761904762	0		inf	#DIV/0!
POLQ	0.1428571429	0.1428571429	0.05555555556		2.333333333	#DIV/0!
POLR1A	0	0	0.05555555556		0	#DIV/0!
POLR1B	0	0	0.05555555556		0	#DIV/0!
POLR1C	0.04761904762	0.04761904762	0.05555555556		0.7	#DIV/0!
POLR1E	0.09523809524	0.09523809524	0.05555555556		1.473684211	#DIV/0!
POLR2A	0	0	0.05555555556		0	#DIV/0!
POLR2B	0.1904761905	0.1904761905	0.05555555556		3.294117647	#DIV/0!
POLR2C	0.04761904762	0.04761904762	0		inf	#DIV/0!
POLR2D	0	0	0.05555555556		0	#DIV/0!

POLR2E	0.09523809524	0.09523809524	0.1111111111		0.6842105263	#DIV/0!
POLR2F	0.04761904762	0.04761904762	0.1111111111		0.325	#DIV/0!
POLR2H	0.1428571429	0.1428571429	0.05555555556		2.333333333	#DIV/0!
POLR2I	0.04761904762	0.04761904762	0.05555555556		0.7	#DIV/0!
POLR2J	0.04761904762	0.04761904762	0.05555555556		0.7	#DIV/0!
POLR2J2	0.04761904762	0.04761904762	0.05555555556		0.7	#DIV/0!
POLR2J3	0.04761904762	0.04761904762	0.05555555556		0.7	#DIV/0!
POLR2J4	0.04761904762	0.04761904762	0.1666666667		0.2	#DIV/0!
POLR2K	0.1904761905	0.1904761905	0.1111111111		1.529411765	#DIV/0!
POLR2L	0.04761904762	0.04761904762	0		inf	#DIV/0!
POLR2M	0.04761904762	0.04761904762	0.05555555556		0.7	#DIV/0!
POLR3A	0.1428571429	0.1428571429	0.05555555556		2.333333333	#DIV/0!
POLR3B	0.04761904762	0.04761904762	0.05555555556		0.7	#DIV/0!
POLR3C	0.1428571429	0.1428571429	0.05555555556		2.333333333	#DIV/0!
POLR3D	0.09523809524	0.09523809524	0.1111111111		0.6842105263	#DIV/0!
POLR3E	0.04761904762	0.04761904762	0		inf	#DIV/0!
POLR3F	0.1904761905	0.1904761905	0.05555555556		3.294117647	#DIV/0!
POLR3G	0.04761904762	0.04761904762	0		inf	#DIV/0!
POLR3H	0	0	0.1111111111		0	#DIV/0!
POLR3K	0.04761904762	0.04761904762	0.2222222222		0.1375	#DIV/0!
POLRMT	0.09523809524	0.09523809524	0.1111111111		0.6842105263	#DIV/0!
POM121	0.1428571429	0.1428571429	0.1111111111		1.083333333	#DIV/0!
POM121C	0.1428571429	0.1428571429	0.1666666667		0.6666666667	#DIV/0!
M121L1	0.04761904762	0.04761904762	0.1111111111		0.325	#DIV/0!
OM121L	0.1428571429	0.1428571429	0.1666666667		0.6666666667	#DIV/0!
OM121L	0.04761904762	0.04761904762	0.05555555556		0.7	#DIV/0!
OM121L	0.04761904762	0.04761904762	0.05555555556		0.7	#DIV/0!
OM121L	0.04761904762	0.04761904762	0.05555555556		0.7	#DIV/0!
OM121L	0.04761904762	0.04761904762	0.1111111111		0.325	#DIV/0!
OMGNT	0.04761904762	0.04761904762	0.05555555556		0.7	#DIV/0!
OMGNT	0.04761904762	0.04761904762	0		inf	#DIV/0!
POMK	0.09523809524	0.09523809524	0.1666666667		0.4210526316	#DIV/0!
POMT1	0.09523809524	0.09523809524	0.05555555556		1.473684211	#DIV/0!
POMZP3	0.09523809524	0.09523809524	0.1111111111		0.6842105263	#DIV/0!
PON1	0.04761904762	0.04761904762	0.1111111111		0.325	#DIV/0!
PON2	0.04761904762	0.04761904762	0.1111111111		0.325	#DIV/0!
PON3	0.04761904762	0.04761904762	0.1111111111		0.325	#DIV/0!
POP1	0.1904761905	0.1904761905	0.1111111111		1.529411765	#DIV/0!
POP4	0.04761904762	0.04761904762	0.1666666667		0.2	#DIV/0!
POP5	0	0	0.1111111111		0	#DIV/0!
POP7	0.04761904762	0.04761904762	0.05555555556		0.7	#DIV/0!
POPDC2	0.09523809524	0.09523809524	0.05555555556		1.473684211	#DIV/0!
POR	0.1428571429	0.1428571429	0.1111111111		1.083333333	#DIV/0!
POT1	0.04761904762	0.04761904762	0.05555555556		0.7	#DIV/0!
OT1-AS	0.04761904762	0.04761904762	0.05555555556		0.7	#DIV/0!
POTEA	0.09523809524	0.09523809524	0.1666666667		0.4210526316	#DIV/0!
POTEB	0.2857142857	0.2857142857	0.1111111111		2.6	#DIV/0!
POTEB2	0.2857142857	0.2857142857	0.1111111111		2.6	#DIV/0!
POTEB3	0.2857142857	0.2857142857	0.1111111111		2.6	#DIV/0!
POTEC	0.09523809524	0.09523809524	0.05555555556		1.473684211	#DIV/0!
POTED	0.04761904762	0.04761904762	0		inf	#DIV/0!

POTEE	0	0	0.05555555556		0	#DIV/0!
POTEF	0	0	0.05555555556		0	#DIV/0!
POTEG	0.1428571429	0.1428571429	0.1111111111		1.083333333	#DIV/0!
POTEH	0.09523809524	0.09523809524	0.05555555556		1.473684211	#DIV/0!
)TEH-A	0.1904761905	0.1904761905	0.1111111111		1.529411765	#DIV/0!
POTEI	0	0	0.05555555556		0	#DIV/0!
POTEJ	0	0	0.05555555556		0	#DIV/0!
'OTEKI	0	0	0.05555555556		0	#DIV/0!
POTEM	0.1428571429	0.1428571429	0.1111111111		1.083333333	#DIV/0!
POU1F1	0.04761904762	0.04761904762	0		inf	#DIV/0!
POU2F1	0.1428571429	0.1428571429	0		inf	#DIV/0!
POU2F2	0.04761904762	0.04761904762	0.05555555556		0.7	#DIV/0!
POU3F1	0.04761904762	0.04761904762	0.05555555556		0.7	#DIV/0!
POU3F3	0	0	0.05555555556		0	#DIV/0!
POU4F2	0	0	0.05555555556		0	#DIV/0!
'OU5F1I	0.09523809524	0.09523809524	0.1111111111		0.6842105263	#DIV/0!
OU5F1P	0.04761904762	0.04761904762	0.1111111111		0.325	#DIV/0!
OU5F1P	0.1904761905	0.1904761905	0.1111111111		1.529411765	#DIV/0!
OU5F1P	0.1904761905	0.1904761905	0.05555555556		3.294117647	#DIV/0!
POU5F2	0.04761904762	0.04761904762	0		inf	#DIV/0!
POU6F1	0.09523809524	0.09523809524	0.05555555556		1.473684211	#DIV/0!
POU6F2	0.04761904762	0.04761904762	0.2222222222		0.1375	#DIV/0!
)U6F2-A	0.04761904762	0.04761904762	0.2222222222		0.1375	#DIV/0!
)U6F2-A	0.04761904762	0.04761904762	0.2222222222		0.1375	#DIV/0!
PP12613	0	0	0.05555555556		0	#DIV/0!
PP2672	0.09523809524	0.09523809524	0.05555555556		1.473684211	#DIV/0!
PP2D1	0.04761904762	0.04761904762	0		inf	#DIV/0!
PP7080	0	0	0.05555555556		0	#DIV/0!
PPA1	0.09523809524	0.09523809524	0		inf	#DIV/0!
PPA2	0	0	0.05555555556		0	#DIV/0!
PPAN	0.1428571429	0.1428571429	0.1111111111		1.083333333	#DIV/0!
AN-P2RY	0.1428571429	0.1428571429	0.1111111111		1.083333333	#DIV/0!
PPARA	0	0	0.1111111111		0	#DIV/0!
PPARD	0.04761904762	0.04761904762	0.05555555556		0.7	#DIV/0!
PPARG	0.09523809524	0.09523809524	0		inf	#DIV/0!
PPAT	0.1904761905	0.1904761905	0.05555555556		3.294117647	#DIV/0!
PPBP	0.09523809524	0.09523809524	0.05555555556		1.473684211	#DIV/0!
PPBPP2	0.09523809524	0.09523809524	0.05555555556		1.473684211	#DIV/0!
PPCDC	0.04761904762	0.04761904762	0.05555555556		0.7	#DIV/0!
PPCS	0.04761904762	0.04761904762	0.05555555556		0.7	#DIV/0!
PPDPF	0.1428571429	0.1428571429	0.2222222222		0.4583333333	#DIV/0!
PPDPFL	0.09523809524	0.09523809524	0.1111111111		0.6842105263	#DIV/0!
PPEF2	0.04761904762	0.04761904762	0.05555555556		0.7	#DIV/0!
PPFIA2	0.04761904762	0.04761904762	0.05555555556		0.7	#DIV/0!
'FIA2-A	0.04761904762	0.04761904762	0.05555555556		0.7	#DIV/0!
PPFIA3	0.09523809524	0.09523809524	0.1111111111		0.6842105263	#DIV/0!
PPFIA4	0.1428571429	0.1428571429	0		inf	#DIV/0!
'PFIBP1	0.04761904762	0.04761904762	0.05555555556		0.7	#DIV/0!
'PFIBP2	0.04761904762	0.04761904762	0		inf	#DIV/0!
PPHLN1	0.04761904762	0.04761904762	0.1111111111		0.325	#DIV/0!
PPIA	0.04761904762	0.04761904762	0.2222222222		0.1375	#DIV/0!

**PPIAL4A**	0.1428571429	0.1428571429	0.05555555556		2.333333333	#DIV/0!
**PPIAL4C**	0.1428571429	0.1428571429	0.05555555556		2.333333333	#DIV/0!
**PPIAL4D**	0.1428571429	0.1428571429	0.05555555556		2.333333333	#DIV/0!
**PPIAL4E**	0.1428571429	0.1428571429	0.05555555556		2.333333333	#DIV/0!
**PPIAL4F**	0.1428571429	0.1428571429	0.05555555556		2.333333333	#DIV/0!
**PPIAL4G**	0.09523809524	0.09523809524	0.05555555556		1.473684211	#DIV/0!
**PPIAL4H**	0.1428571429	0.1428571429	0.05555555556		2.333333333	#DIV/0!
**PPIAP30**	0.09523809524	0.09523809524	0.1111111111		0.6842105263	#DIV/0!
**PPIAP46**	0.04761904762	0.04761904762	0.05555555556		0.7	#DIV/0!
**PPIB**	0.04761904762	0.04761904762	0.05555555556		0.7	#DIV/0!
**PPIC**	0.04761904762	0.04761904762	0		inf	#DIV/0!
**PPID**	0	0	0.05555555556		0	#DIV/0!
**PPIE**	0.04761904762	0.04761904762	0.05555555556		0.7	#DIV/0!
**PPIEL**	0.04761904762	0.04761904762	0.05555555556		0.7	#DIV/0!
**PPIF**	0.1428571429	0.1428571429	0.05555555556		2.333333333	#DIV/0!
**PPIG**	0	0	0.05555555556		0	#DIV/0!
**PPIH**	0.04761904762	0.04761904762	0.05555555556		0.7	#DIV/0!
**PPIL1**	0.04761904762	0.04761904762	0.05555555556		0.7	#DIV/0!
**PPIL2**	0.04761904762	0.04761904762	0.05555555556		0.7	#DIV/0!
**PPIL3**	0	0	0.05555555556		0	#DIV/0!
**PPIL4**	0	0	0			#DIV/0!
**PPIL6**	0	0	0			#DIV/0!
**PPIP5K2**	0.04761904762	0.04761904762	0		inf	#DIV/0!
**PPL**	0.04761904762	0.04761904762	0		inf	#DIV/0!
**PPM1B**	0	0	0.05555555556		0	#DIV/0!
**PPM1D**	0.1428571429	0.1428571429	0		inf	#DIV/0!
**PPM1E**	0.1428571429	0.1428571429	0		inf	#DIV/0!
**PPM1F**	0.04761904762	0.04761904762	0.05555555556		0.7	#DIV/0!
**PPM1H**	0.09523809524	0.09523809524	0.05555555556		1.473684211	#DIV/0!
**PPM1J**	0.04761904762	0.04761904762	0.05555555556		0.7	#DIV/0!
**PPM1K**	0	0	0.05555555556		0	#DIV/0!
**PM1K-D**	0	0	0.05555555556		0	#DIV/0!
**PPM1L**	0.1428571429	0.1428571429	0.05555555556		2.333333333	#DIV/0!
**PPM1N**	0.09523809524	0.09523809524	0.1111111111		0.6842105263	#DIV/0!
**PPOX**	0.1428571429	0.1428571429	0		inf	#DIV/0!
**PPP1CB**	0	0	0.05555555556		0	#DIV/0!
**PPP1CC**	0	0	0.1111111111		0	#DIV/0!
**PP1R12**	0.04761904762	0.04761904762	0.05555555556		0.7	#DIV/0!
**1R12A-**	0.04761904762	0.04761904762	0.05555555556		0.7	#DIV/0!
**PP1R12**	0.1428571429	0.1428571429	0		inf	#DIV/0!
**PP1R12**	0.09523809524	0.09523809524	0.05555555556		1.473684211	#DIV/0!
**PP1R13**	0.09523809524	0.09523809524	0.1111111111		0.6842105263	#DIV/0!
**PP1R14**	0.04761904762	0.04761904762	0.05555555556		0.7	#DIV/0!
**PP1R15**	0.09523809524	0.09523809524	0.1111111111		0.6842105263	#DIV/0!
**PP1R15**	0.2380952381	0.2380952381	0		inf	#DIV/0!
**PP1R16**	0.04761904762	0.04761904762	0.1666666667		0.2	#DIV/0!
**PP1R16**	0.1428571429	0.1428571429	0.2222222222		0.4583333333	#DIV/0!
**PPP1R17**	0.09523809524	0.09523809524	0.1111111111		0.6842105263	#DIV/0!
**PPP1R1B**	0.09523809524	0.09523809524	0		inf	#DIV/0!
**PPP1R1C**	0	0	0.05555555556		0	#DIV/0!
**PPP1R2**	0.1428571429	0.1428571429	0.05555555556		2.333333333	#DIV/0!

PPP1R21	0	0	0.05555555556		0	#DIV/0!
PPP1R26	0.04761904762	0.04761904762	0		inf	#DIV/0!
P1R26-A	0.04761904762	0.04761904762	0		inf	#DIV/0!
PPP1R27	0.04761904762	0.04761904762	0		inf	#DIV/0!
PPP1R2B	0.04761904762	0.04761904762	0.05555555556		0.7	#DIV/0!
PPP1R2P	0.04761904762	0.04761904762	0.1666666667		0.2	#DIV/0!
PPP1R35	0.04761904762	0.04761904762	0.05555555556		0.7	#DIV/0!
PPP1R36	0.09523809524	0.09523809524	0		inf	#DIV/0!
PPP1R37	0.09523809524	0.09523809524	0.1111111111		0.6842105263	#DIV/0!
PPP1R3A	0.04761904762	0.04761904762	0.05555555556		0.7	#DIV/0!
PPP1R3B	0.09523809524	0.09523809524	0.1111111111		0.6842105263	#DIV/0!
PPP1R3C	0.1428571429	0.1428571429	0.1111111111		1.083333333	#DIV/0!
PPP1R3D	0.1428571429	0.1428571429	0.1666666667		0.6666666667	#DIV/0!
PPP1R3E	0	0	0.1111111111		0	#DIV/0!
PPP1R42	0.1428571429	0.1428571429	0.1666666667		0.6666666667	#DIV/0!
PPP1R7	0	0	0.05555555556		0	#DIV/0!
PPP1R8	0.04761904762	0.04761904762	0.1111111111		0.325	#DIV/0!
PPP1R9A	0.04761904762	0.04761904762	0.1111111111		0.325	#DIV/0!
PPP1R9B	0.09523809524	0.09523809524	0		inf	#DIV/0!
PPP2CA	0.04761904762	0.04761904762	0		inf	#DIV/0!
PPP2CB	0.04761904762	0.04761904762	0.1111111111		0.325	#DIV/0!
PPP2R1A	0.09523809524	0.09523809524	0.05555555556		1.473684211	#DIV/0!
PPP2R2A	0.04761904762	0.04761904762	0.1111111111		0.325	#DIV/0!
PPP2R2D	0.2380952381	0.2380952381	0		inf	#DIV/0!
PPP2R3A	0.1904761905	0.1904761905	0.05555555556		3.294117647	#DIV/0!
PPP2R3C	0.09523809524	0.09523809524	0		inf	#DIV/0!
PPP2R5A	0.09523809524	0.09523809524	0		inf	#DIV/0!
PPP2R5D	0.04761904762	0.04761904762	0.05555555556		0.7	#DIV/0!
PPP2R5E	0.09523809524	0.09523809524	0		inf	#DIV/0!
PPP3CA	0	0	0.05555555556		0	#DIV/0!
PPP3CB	0.1904761905	0.1904761905	0.05555555556		3.294117647	#DIV/0!
P3CB-A	0.1904761905	0.1904761905	0.05555555556		3.294117647	#DIV/0!
PPP3CC	0.09523809524	0.09523809524	0.1111111111		0.6842105263	#DIV/0!
PPP3R1	0	0	0.05555555556		0	#DIV/0!
PPP3R2	0.04761904762	0.04761904762	0		inf	#DIV/0!
PPP4C	0.04761904762	0.04761904762	0		inf	#DIV/0!
PPP4R1	0.09523809524	0.09523809524	0		inf	#DIV/0!
P4R1-A	0.09523809524	0.09523809524	0		inf	#DIV/0!
PPP4R1L	0.1428571429	0.1428571429	0.2222222222		0.4583333333	#DIV/0!
PPP4R2	0.04761904762	0.04761904762	0		inf	#DIV/0!
PPP4R3B	0.04761904762	0.04761904762	0.05555555556		0.7	#DIV/0!
PPP5C	0.09523809524	0.09523809524	0.1111111111		0.6842105263	#DIV/0!
PPP5D1	0.09523809524	0.09523809524	0.1111111111		0.6842105263	#DIV/0!
PPP6C	0.09523809524	0.09523809524	0.05555555556		1.473684211	#DIV/0!
PPP6R1	0.09523809524	0.09523809524	0.05555555556		1.473684211	#DIV/0!
PPP6R2	0	0	0.1111111111		0	#DIV/0!
PPRC1	0.1428571429	0.1428571429	0.05555555556		2.333333333	#DIV/0!
PPT1	0.04761904762	0.04761904762	0.05555555556		0.7	#DIV/0!
PPT2	0.04761904762	0.04761904762	0		inf	#DIV/0!
T2-EGF	0.04761904762	0.04761904762	0		inf	#DIV/0!
PPTC7	0	0	0.1111111111		0	#DIV/0!

PPWD1	0.04761904762	0.04761904762	0		inf	#DIV/0!
PPY	0.09523809524	0.09523809524	0		inf	#DIV/0!
PPY2P	0.04761904762	0.04761904762	0.05555555556		0.7	#DIV/0!
PQLC1	0.04761904762	0.04761904762	0.05555555556		0.7	#DIV/0!
PQLC2	0.04761904762	0.04761904762	0.1111111111		0.325	#DIV/0!
PQLC2L	0.1428571429	0.1428571429	0.05555555556		2.333333333	#DIV/0!
PQLC3	0	0	0.05555555556		0	#DIV/0!
PRAC1	0.1428571429	0.1428571429	0		inf	#DIV/0!
PRAC2	0.1428571429	0.1428571429	0		inf	#DIV/0!
PRADC1	0	0	0.05555555556		0	#DIV/0!
PRAG1	0.09523809524	0.09523809524	0.1111111111		0.6842105263	#DIV/0!
PRAM1	0.1428571429	0.1428571429	0.05555555556		2.333333333	#DIV/0!
PRAME	0.04761904762	0.04761904762	0.05555555556		0.7	#DIV/0!
RAMEF	0.04761904762	0.04761904762	0.1111111111		0.325	#DIV/0!
RAMEF1	0.04761904762	0.04761904762	0.1111111111		0.325	#DIV/0!
RAMEF	0.04761904762	0.04761904762	0.1111111111		0.325	#DIV/0!
RAMEF1	0.04761904762	0.04761904762	0.1111111111		0.325	#DIV/0!
RAMEF1	0.04761904762	0.04761904762	0.1111111111		0.325	#DIV/0!
RAMEF1	0.04761904762	0.04761904762	0.1111111111		0.325	#DIV/0!
RAMEF1	0.04761904762	0.04761904762	0.1111111111		0.325	#DIV/0!
RAMEF1	0.04761904762	0.04761904762	0.1111111111		0.325	#DIV/0!
RAMEF1	0.04761904762	0.04761904762	0.1111111111		0.325	#DIV/0!
RAMEF1	0.04761904762	0.04761904762	0.1111111111		0.325	#DIV/0!
RAMEF	0.04761904762	0.04761904762	0.1111111111		0.325	#DIV/0!
RAMEF2	0.04761904762	0.04761904762	0.1111111111		0.325	#DIV/0!
RAMEF2	0.04761904762	0.04761904762	0.1111111111		0.325	#DIV/0!
RAMEF2	0.04761904762	0.04761904762	0.1111111111		0.325	#DIV/0!
RAMEF2	0.04761904762	0.04761904762	0.1111111111		0.325	#DIV/0!
RAMEF2	0.04761904762	0.04761904762	0.1111111111		0.325	#DIV/0!
RAMEF3	0.04761904762	0.04761904762	0.1111111111		0.325	#DIV/0!
AMEF3	0.04761904762	0.04761904762	0.1111111111		0.325	#DIV/0!
AMEF3	0.04761904762	0.04761904762	0.1111111111		0.325	#DIV/0!
RAMEF	0.04761904762	0.04761904762	0.1111111111		0.325	#DIV/0!
RAMEF	0.04761904762	0.04761904762	0.1111111111		0.325	#DIV/0!
RAMEF	0.04761904762	0.04761904762	0.1111111111		0.325	#DIV/0!
RAMEF	0.04761904762	0.04761904762	0.1111111111		0.325	#DIV/0!
RAMEF	0.04761904762	0.04761904762	0.1111111111		0.325	#DIV/0!
RAMEF	0.04761904762	0.04761904762	0.1111111111		0.325	#DIV/0!
RAMEN	0.04761904762	0.04761904762	0.05555555556		0.7	#DIV/0!
PRAP1	0.1904761905	0.1904761905	0		inf	#DIV/0!
PRB1	0.04761904762	0.04761904762	0.1111111111		0.325	#DIV/0!
PRB2	0.04761904762	0.04761904762	0.1111111111		0.325	#DIV/0!
PRB3	0.04761904762	0.04761904762	0.1111111111		0.325	#DIV/0!
PRB4	0.04761904762	0.04761904762	0.1111111111		0.325	#DIV/0!
PRC1	0.04761904762	0.04761904762	0.05555555556		0.7	#DIV/0!
RC1-AS	0.04761904762	0.04761904762	0.05555555556		0.7	#DIV/0!
PRCC	0.1428571429	0.1428571429	0.05555555556		2.333333333	#DIV/0!
PRCD	0.04761904762	0.04761904762	0		inf	#DIV/0!
PRDM12	0.09523809524	0.09523809524	0.05555555556		1.473684211	#DIV/0!
PRDM14	0.1428571429	0.1428571429	0.1666666667		0.6666666667	#DIV/0!
PRDM15	0.04761904762	0.04761904762	0.1111111111		0.325	#DIV/0!

PRDM16	0	0	0.05555555556		0	#DIV/0!
RDM16-I	0	0	0.05555555556		0	#DIV/0!
PRDM2	0.04761904762	0.04761904762	0.1111111111		0.325	#DIV/0!
PRDM4	0.04761904762	0.04761904762	0.05555555556		0.7	#DIV/0!
PRDM5	0	0	0.05555555556		0	#DIV/0!
PRDM6	0.04761904762	0.04761904762	0		inf	#DIV/0!
PRDM7	0.09523809524	0.09523809524	0		inf	#DIV/0!
PRDM8	0.04761904762	0.04761904762	0.05555555556		0.7	#DIV/0!
PRDM9	0.04761904762	0.04761904762	0.05555555556		0.7	#DIV/0!
PRDX1	0.04761904762	0.04761904762	0.05555555556		0.7	#DIV/0!
PRDX2	0.1428571429	0.1428571429	0.1111111111		1.083333333	#DIV/0!
PRDX3	0.2380952381	0.2380952381	0		inf	#DIV/0!
PRDX6	0.1428571429	0.1428571429	0		inf	#DIV/0!
PRELID1	0.09523809524	0.09523809524	0.05555555556		1.473684211	#DIV/0!
RELID3	0.09523809524	0.09523809524	0.05555555556		1.473684211	#DIV/0!
RELID3	0.1428571429	0.1428571429	0.2222222222		0.4583333333	#DIV/0!
PRELP	0.1904761905	0.1904761905	0		inf	#DIV/0!
PREPL	0	0	0.05555555556		0	#DIV/0!
PREX1	0.1428571429	0.1428571429	0.1666666667		0.6666666667	#DIV/0!
PREX2	0.1428571429	0.1428571429	0.1666666667		0.6666666667	#DIV/0!
PRF1	0.09523809524	0.09523809524	0		inf	#DIV/0!
PRG1	0.04761904762	0.04761904762	0.05555555556		0.7	#DIV/0!
PRG4	0.1428571429	0.1428571429	0.05555555556		2.333333333	#DIV/0!
PRH1	0.04761904762	0.04761904762	0.1111111111		0.325	#DIV/0!
RH1-PRI	0.04761904762	0.04761904762	0.1111111111		0.325	#DIV/0!
I1-TAS2	0.04761904762	0.04761904762	0.1111111111		0.325	#DIV/0!
PRH2	0.04761904762	0.04761904762	0.1111111111		0.325	#DIV/0!
RICKLE	0.04761904762	0.04761904762	0.1111111111		0.325	#DIV/0!
RICKLE	0.04761904762	0.04761904762	0		inf	#DIV/0!
CKLE2-	0.04761904762	0.04761904762	0		inf	#DIV/0!
CKLE2-	0.04761904762	0.04761904762	0		inf	#DIV/0!
CKLE2-	0.04761904762	0.04761904762	0		inf	#DIV/0!
RICKLE	0.04761904762	0.04761904762	0.05555555556		0.7	#DIV/0!
PRIM1	0.1428571429	0.1428571429	0.05555555556		2.333333333	#DIV/0!
PRIM2	0.04761904762	0.04761904762	0.05555555556		0.7	#DIV/0!
RIMPOI	0	0	0.05555555556		0	#DIV/0!
PRKAA1	0.04761904762	0.04761904762	0.05555555556		0.7	#DIV/0!
PRKAA2	0.04761904762	0.04761904762	0.05555555556		0.7	#DIV/0!
PRKAB1	0	0	0.05555555556		0	#DIV/0!
PRKAB2	0.1428571429	0.1428571429	0.05555555556		2.333333333	#DIV/0!
PRKACA	0.1428571429	0.1428571429	0.1111111111		1.083333333	#DIV/0!
PRKACB	0.04761904762	0.04761904762	0.05555555556		0.7	#DIV/0!
PRKACG	0	0	0.05555555556		0	#DIV/0!
PRKAG1	0.04761904762	0.04761904762	0.05555555556		0.7	#DIV/0!
PRKAG2	0.09523809524	0.09523809524	0.05555555556		1.473684211	#DIV/0!
KAG2-A	0.09523809524	0.09523809524	0.05555555556		1.473684211	#DIV/0!
PRKAG3	0	0	0.05555555556		0	#DIV/0!
RKAR1	0	0	0			#DIV/0!
RKAR1	0.04761904762	0.04761904762	0.1666666667		0.2	#DIV/0!
RKAR2	0.09523809524	0.09523809524	0		inf	#DIV/0!
KAR2A-	0.09523809524	0.09523809524	0		inf	#DIV/0!

RKAR2	0.04761904762	0.04761904762	0.05555555556		0.7	#DIV/0!
PRKCA	0	0	0			#DIV/0!
KCA-A	0	0	0			#DIV/0!
PRKCB	0.04761904762	0.04761904762	0		inf	#DIV/0!
PRKCD	0.04761904762	0.04761904762	0		inf	#DIV/0!
PRKCE	0	0	0.05555555556		0	#DIV/0!
PRKCG	0.09523809524	0.09523809524	0.05555555556		1.473684211	#DIV/0!
PRKCI	0.1904761905	0.1904761905	0.05555555556		3.294117647	#DIV/0!
PRKCQ	0.09523809524	0.09523809524	0.1111111111		0.6842105263	#DIV/0!
KCQ-A	0.09523809524	0.09523809524	0.1111111111		0.6842105263	#DIV/0!
PRKCSH	0.1428571429	0.1428571429	0.1111111111		1.083333333	#DIV/0!
PRKCZ	0	0	0.1111111111		0	#DIV/0!
KCZ-A	0	0	0.1111111111		0	#DIV/0!
PRKD2	0.09523809524	0.09523809524	0.1111111111		0.6842105263	#DIV/0!
PRKD3	0	0	0.05555555556		0	#DIV/0!
PRKDC	0.09523809524	0.09523809524	0.1666666667		0.4210526316	#DIV/0!
PRKG1	0.04761904762	0.04761904762	0.05555555556		0.7	#DIV/0!
KG1-A	0.04761904762	0.04761904762	0.05555555556		0.7	#DIV/0!
PRKG2	0	0	0.05555555556		0	#DIV/0!
PRKRA	0	0	0.1111111111		0	#DIV/0!
KRA-A	0	0	0.1111111111		0	#DIV/0!
RKRIP	0.04761904762	0.04761904762	0.05555555556		0.7	#DIV/0!
PRKXP1	0.04761904762	0.04761904762	0.05555555556		0.7	#DIV/0!
PRLH	0	0	0.05555555556		0	#DIV/0!
PRLHR	0.2380952381	0.2380952381	0		inf	#DIV/0!
PRLR	0.04761904762	0.04761904762	0.05555555556		0.7	#DIV/0!
PRM1	0.04761904762	0.04761904762	0		inf	#DIV/0!
PRM2	0.04761904762	0.04761904762	0		inf	#DIV/0!
PRM3	0.04761904762	0.04761904762	0		inf	#DIV/0!
PRMT1	0.09523809524	0.09523809524	0.1111111111		0.6842105263	#DIV/0!
PRMT2	0.04761904762	0.04761904762	0.1111111111		0.325	#DIV/0!
PRMT5	0	0	0.05555555556		0	#DIV/0!
MT5-A	0	0	0.05555555556		0	#DIV/0!
PRMT6	0.04761904762	0.04761904762	0.05555555556		0.7	#DIV/0!
PRMT7	0.1428571429	0.1428571429	0.05555555556		2.333333333	#DIV/0!
PRMT8	0.04761904762	0.04761904762	0.1111111111		0.325	#DIV/0!
PRMT9	0	0	0.05555555556		0	#DIV/0!
PRNCR1	0.09523809524	0.09523809524	0.1111111111		0.6842105263	#DIV/0!
PRND	0.1904761905	0.1904761905	0.05555555556		3.294117647	#DIV/0!
PRNP	0.1904761905	0.1904761905	0.05555555556		3.294117647	#DIV/0!
PRNT	0.1904761905	0.1904761905	0.05555555556		3.294117647	#DIV/0!
PRO180	0	0	0			#DIV/0!
PROB1	0.04761904762	0.04761904762	0		inf	#DIV/0!
PROC	0	0	0.05555555556		0	#DIV/0!
PROCA1	0.04761904762	0.04761904762	0.05555555556		0.7	#DIV/0!
PROCR	0.09523809524	0.09523809524	0.1111111111		0.6842105263	#DIV/0!
PRODH	0.04761904762	0.04761904762	0.05555555556		0.7	#DIV/0!
PRODH2	0.04761904762	0.04761904762	0.05555555556		0.7	#DIV/0!
PROK1	0.04761904762	0.04761904762	0		inf	#DIV/0!
PROK2	0.04761904762	0.04761904762	0		inf	#DIV/0!
PROKR1	0	0	0.05555555556		0	#DIV/0!

PROKR2	0.1904761905	0.1904761905	0.05555555556		3.294117647	#DIV/0!
PROM1	0	0	0			#DIV/0!
PROM2	0	0	0.1111111111		0	#DIV/0!
PROP1	0.09523809524	0.09523809524	0.05555555556		1.473684211	#DIV/0!
RORSD1	0.04761904762	0.04761904762	0.05555555556		0.7	#DIV/0!
PROS1	0.04761904762	0.04761904762	0.05555555556		0.7	#DIV/0!
ROSER	0.09523809524	0.09523809524	0.1111111111		0.6842105263	#DIV/0!
OSER2-A	0.09523809524	0.09523809524	0.1111111111		0.6842105263	#DIV/0!
ROSER	0.04761904762	0.04761904762	0.05555555556		0.7	#DIV/0!
PROX1	0.1428571429	0.1428571429	0		inf	#DIV/0!
ROX1-AS	0.1428571429	0.1428571429	0		inf	#DIV/0!
PRPF18	0.09523809524	0.09523809524	0.1111111111		0.6842105263	#DIV/0!
PRPF3	0.1904761905	0.1904761905	0.05555555556		3.294117647	#DIV/0!
PRPF31	0.09523809524	0.09523809524	0.05555555556		1.473684211	#DIV/0!
PRPF38A	0.04761904762	0.04761904762	0.05555555556		0.7	#DIV/0!
PRPF38B	0.04761904762	0.04761904762	0.05555555556		0.7	#DIV/0!
PRPF39	0.04761904762	0.04761904762	0		inf	#DIV/0!
PRPF4	0.04761904762	0.04761904762	0.05555555556		0.7	#DIV/0!
PRPF40A	0	0	0.05555555556		0	#DIV/0!
PRPF40B	0.04761904762	0.04761904762	0.05555555556		0.7	#DIV/0!
PRPF6	0.1428571429	0.1428571429	0.2222222222		0.4583333333	#DIV/0!
PRPF8	0	0	0.1111111111		0	#DIV/0!
PRPH	0.04761904762	0.04761904762	0.05555555556		0.7	#DIV/0!
PRPH2	0.04761904762	0.04761904762	0.05555555556		0.7	#DIV/0!
PRPS1L1	0.04761904762	0.04761904762	0.2222222222		0.1375	#DIV/0!
PRPSAP1	0.04761904762	0.04761904762	0		inf	#DIV/0!
PRPSAP2	0.04761904762	0.04761904762	0.1111111111		0.325	#DIV/0!
PRR11	0.1428571429	0.1428571429	0		inf	#DIV/0!
PRR12	0.09523809524	0.09523809524	0.1111111111		0.6842105263	#DIV/0!
PRR13	0.04761904762	0.04761904762	0.05555555556		0.7	#DIV/0!
PRR14	0.04761904762	0.04761904762	0		inf	#DIV/0!
PRR14L	0.04761904762	0.04761904762	0.1111111111		0.325	#DIV/0!
PRR15	0.04761904762	0.04761904762	0.1666666667		0.2	#DIV/0!
PRR15L	0.1428571429	0.1428571429	0		inf	#DIV/0!
PRR16	0.04761904762	0.04761904762	0		inf	#DIV/0!
PRR19	0.04761904762	0.04761904762	0.05555555556		0.7	#DIV/0!
PRR20G	0.09523809524	0.09523809524	0.05555555556		1.473684211	#DIV/0!
PRR22	0.1428571429	0.1428571429	0.05555555556		2.333333333	#DIV/0!
PRR23A	0.1428571429	0.1428571429	0.05555555556		2.333333333	#DIV/0!
PRR23B	0.1428571429	0.1428571429	0.05555555556		2.333333333	#DIV/0!
PRR23C	0.1428571429	0.1428571429	0.05555555556		2.333333333	#DIV/0!
PRR23D1	0.09523809524	0.09523809524	0.2222222222		0.2894736842	#DIV/0!
PRR23D2	0.09523809524	0.09523809524	0.2222222222		0.2894736842	#DIV/0!
PRR25	0.04761904762	0.04761904762	0.2222222222		0.1375	#DIV/0!
PRR26	0.04761904762	0.04761904762	0.1111111111		0.325	#DIV/0!
PRR27	0.04761904762	0.04761904762	0.05555555556		0.7	#DIV/0!
PRR29	0.04761904762	0.04761904762	0		inf	#DIV/0!
RR29-AS	0.04761904762	0.04761904762	0		inf	#DIV/0!
RR34-AS	0	0	0.1111111111		0	#DIV/0!
PRR35	0.04761904762	0.04761904762	0.2222222222		0.1375	#DIV/0!
PRR36	0.1428571429	0.1428571429	0.05555555556		2.333333333	#DIV/0!

PRR4	0.04761904762	0.04761904762	0.1111111111		0.325	#DIV/0!
PRR5	0	0	0.1666666667		0	#DIV/0!
5-ARHG	0	0	0.1666666667		0	#DIV/0!
PRR7	0.09523809524	0.09523809524	0.05555555556		1.473684211	#DIV/0!
RR7-AS	0.09523809524	0.09523809524	0.05555555556		1.473684211	#DIV/0!
PRR9	0.1904761905	0.1904761905	0		inf	#DIV/0!
PRRC1	0.04761904762	0.04761904762	0		inf	#DIV/0!
PRRC2A	0.04761904762	0.04761904762	0		inf	#DIV/0!
PRRC2B	0.09523809524	0.09523809524	0.05555555556		1.473684211	#DIV/0!
PRRC2C	0.1428571429	0.1428571429	0.05555555556		2.333333333	#DIV/0!
PRRG2	0.09523809524	0.09523809524	0.1111111111		0.6842105263	#DIV/0!
PRRG4	0.04761904762	0.04761904762	0		inf	#DIV/0!
PRRT1	0.04761904762	0.04761904762	0		inf	#DIV/0!
PRRT1B	0.09523809524	0.09523809524	0.05555555556		1.473684211	#DIV/0!
PRRT2	0.04761904762	0.04761904762	0		inf	#DIV/0!
PRRT3	0.09523809524	0.09523809524	0		inf	#DIV/0!
RRT3-AS	0.09523809524	0.09523809524	0		inf	#DIV/0!
PRRT4	0.04761904762	0.04761904762	0.05555555556		0.7	#DIV/0!
PRRX1	0.1428571429	0.1428571429	0.05555555556		2.333333333	#DIV/0!
PRRX2	0.09523809524	0.09523809524	0.05555555556		1.473684211	#DIV/0!
RRX2-AS	0.09523809524	0.09523809524	0.05555555556		1.473684211	#DIV/0!
PRSS1	0.04761904762	0.04761904762	0.05555555556		0.7	#DIV/0!
PRSS12	0	0	0.05555555556		0	#DIV/0!
PRSS21	0.04761904762	0.04761904762	0.1111111111		0.325	#DIV/0!
PRSS22	0.04761904762	0.04761904762	0.1111111111		0.325	#DIV/0!
PRSS27	0.04761904762	0.04761904762	0.1111111111		0.325	#DIV/0!
PRSS3	0.09523809524	0.09523809524	0.05555555556		1.473684211	#DIV/0!
PRSS30P	0.04761904762	0.04761904762	0.1111111111		0.325	#DIV/0!
PRSS33	0.04761904762	0.04761904762	0.1111111111		0.325	#DIV/0!
PRSS36	0.04761904762	0.04761904762	0		inf	#DIV/0!
PRSS37	0.04761904762	0.04761904762	0.05555555556		0.7	#DIV/0!
PRSS38	0.1428571429	0.1428571429	0		inf	#DIV/0!
PRSS3P2	0.04761904762	0.04761904762	0.05555555556		0.7	#DIV/0!
PRSS40A	0	0	0.05555555556		0	#DIV/0!
PRSS40B	0	0	0.05555555556		0	#DIV/0!
PRSS41	0.04761904762	0.04761904762	0.1111111111		0.325	#DIV/0!
PRSS47	0.04761904762	0.04761904762	0.05555555556		0.7	#DIV/0!
PRSS48	0	0	0.05555555556		0	#DIV/0!
PRSS53	0.04761904762	0.04761904762	0		inf	#DIV/0!
PRSS54	0.04761904762	0.04761904762	0		inf	#DIV/0!
PRSS55	0.09523809524	0.09523809524	0.1111111111		0.6842105263	#DIV/0!
PRSS56	0	0	0.05555555556		0	#DIV/0!
PRSS57	0.09523809524	0.09523809524	0.1111111111		0.6842105263	#DIV/0!
PRSS58	0.04761904762	0.04761904762	0.05555555556		0.7	#DIV/0!
PRSS8	0.04761904762	0.04761904762	0		inf	#DIV/0!
RTFDC	0.09523809524	0.09523809524	0.1111111111		0.6842105263	#DIV/0!
PRTG	0.04761904762	0.04761904762	0.05555555556		0.7	#DIV/0!
PRTN3	0.09523809524	0.09523809524	0.1111111111		0.6842105263	#DIV/0!
PRUNE1	0.1904761905	0.1904761905	0.05555555556		3.294117647	#DIV/0!
PRUNE2	0	0	0.05555555556		0	#DIV/0!
PRX	0.04761904762	0.04761904762	0.05555555556		0.7	#DIV/0!

**PRXL2A**	0.1428571429	0.1428571429	0		inf	#DIV/0!
**PRXL2B**	0	0	0.1111111111		0	#DIV/0!
**PRXL2C**	0.04761904762	0.04761904762	0.05555555556		0.7	#DIV/0!
**PSAP**	0.1428571429	0.1428571429	0		inf	#DIV/0!
**PSAT1**	0	0	0.05555555556		0	#DIV/0!
**PSCA**	0.04761904762	0.04761904762	0.1666666667		0.2	#DIV/0!
**PSD**	0.1428571429	0.1428571429	0.05555555556		2.333333333	#DIV/0!
**PSD2**	0.04761904762	0.04761904762	0		inf	#DIV/0!
**PSD2-AS**	0.04761904762	0.04761904762	0		inf	#DIV/0!
**PSD3**	0.09523809524	0.09523809524	0.1111111111		0.6842105263	#DIV/0!
**PSD4**	0	0	0.05555555556		0	#DIV/0!
**PSEN1**	0.09523809524	0.09523809524	0		inf	#DIV/0!
**PSEN2**	0.1428571429	0.1428571429	0		inf	#DIV/0!
**PSENEN**	0.04761904762	0.04761904762	0.05555555556		0.7	#DIV/0!
**PSG1**	0.04761904762	0.04761904762	0.05555555556		0.7	#DIV/0!
**PSG10P**	0.04761904762	0.04761904762	0.05555555556		0.7	#DIV/0!
**PSG11**	0.04761904762	0.04761904762	0.05555555556		0.7	#DIV/0!
**PSG2**	0.04761904762	0.04761904762	0.05555555556		0.7	#DIV/0!
**PSG3**	0.04761904762	0.04761904762	0.05555555556		0.7	#DIV/0!
**PSG4**	0.04761904762	0.04761904762	0.05555555556		0.7	#DIV/0!
**PSG5**	0.04761904762	0.04761904762	0.05555555556		0.7	#DIV/0!
**PSG6**	0.04761904762	0.04761904762	0.05555555556		0.7	#DIV/0!
**PSG7**	0.04761904762	0.04761904762	0.05555555556		0.7	#DIV/0!
**PSG8**	0.04761904762	0.04761904762	0.05555555556		0.7	#DIV/0!
**SG8-AS**	0.04761904762	0.04761904762	0.05555555556		0.7	#DIV/0!
**PSG9**	0.04761904762	0.04761904762	0.05555555556		0.7	#DIV/0!
**PSIP1**	0.04761904762	0.04761904762	0.05555555556		0.7	#DIV/0!
**PSKH1**	0.1428571429	0.1428571429	0.05555555556		2.333333333	#DIV/0!
**PSKH2**	0.1904761905	0.1904761905	0.1111111111		1.529411765	#DIV/0!
**PSMA2**	0.04761904762	0.04761904762	0.2222222222		0.1375	#DIV/0!
**PSMA3**	0.04761904762	0.04761904762	0		inf	#DIV/0!
**SMA3-AS**	0.04761904762	0.04761904762	0		inf	#DIV/0!
**PSMA4**	0.04761904762	0.04761904762	0.05555555556		0.7	#DIV/0!
**PSMA5**	0.04761904762	0.04761904762	0.05555555556		0.7	#DIV/0!
**PSMA6**	0.09523809524	0.09523809524	0		inf	#DIV/0!
**PSMA7**	0.1428571429	0.1428571429	0.2222222222		0.4583333333	#DIV/0!
**PSMA8**	0.09523809524	0.09523809524	0.05555555556		1.473684211	#DIV/0!
**PSMB10**	0.1428571429	0.1428571429	0.05555555556		2.333333333	#DIV/0!
**PSMB11**	0	0	0.05555555556		0	#DIV/0!
**PSMB2**	0.04761904762	0.04761904762	0.05555555556		0.7	#DIV/0!
**PSMB3**	0.04761904762	0.04761904762	0		inf	#DIV/0!
**PSMB4**	0.1904761905	0.1904761905	0.05555555556		3.294117647	#DIV/0!
**PSMB5**	0	0	0.05555555556		0	#DIV/0!
**PSMB7**	0.09523809524	0.09523809524	0.05555555556		1.473684211	#DIV/0!
**PSMB8**	0.04761904762	0.04761904762	0		inf	#DIV/0!
**SMB8-AS**	0.04761904762	0.04761904762	0		inf	#DIV/0!
**PSMB9**	0.04761904762	0.04761904762	0		inf	#DIV/0!
**PSMC2**	0.04761904762	0.04761904762	0.05555555556		0.7	#DIV/0!
**PSMC3**	0	0	0.05555555556		0	#DIV/0!
**PSMC3IP**	0.04761904762	0.04761904762	0		inf	#DIV/0!
**PSMC4**	0.04761904762	0.04761904762	0.05555555556		0.7	#DIV/0!

**PSMC5**	0.04761904762	0.04761904762	0		inf	#DIV/0!
**PSMC6**	0.04761904762	0.04761904762	0		inf	#DIV/0!
**PSMD1**	0	0	0.05555555556		0	#DIV/0!
**PSMD11**	0	0	0			#DIV/0!
**PSMD12**	0	0	0			#DIV/0!
**PSMD13**	0.04761904762	0.04761904762	0		inf	#DIV/0!
**PSMD14**	0	0	0.05555555556		0	#DIV/0!
**PSMD2**	0.1428571429	0.1428571429	0.05555555556		2.333333333	#DIV/0!
**PSMD3**	0.09523809524	0.09523809524	0		inf	#DIV/0!
**PSMD4**	0.1904761905	0.1904761905	0.05555555556		3.294117647	#DIV/0!
**PSMD5**	0.09523809524	0.09523809524	0.05555555556		1.473684211	#DIV/0!
**PSMD6**	0.04761904762	0.04761904762	0		inf	#DIV/0!
**SMD6-AS**	0.04761904762	0.04761904762	0		inf	#DIV/0!
**PSMD7**	0.04761904762	0.04761904762	0		inf	#DIV/0!
**PSMD8**	0.04761904762	0.04761904762	0.05555555556		0.7	#DIV/0!
**PSMD9**	0.04761904762	0.04761904762	0.1111111111		0.325	#DIV/0!
**PSME3**	0.04761904762	0.04761904762	0		inf	#DIV/0!
**PSME4**	0.04761904762	0.04761904762	0.05555555556		0.7	#DIV/0!
**PSMF1**	0.1904761905	0.1904761905	0.1111111111		1.529411765	#DIV/0!
**PSMG1**	0.04761904762	0.04761904762	0.1111111111		0.325	#DIV/0!
**PSMG2**	0.09523809524	0.09523809524	0.05555555556		1.473684211	#DIV/0!
**PSMG3**	0.04761904762	0.04761904762	0.1666666667		0.2	#DIV/0!
**SMG3-A**	0.04761904762	0.04761904762	0.1666666667		0.2	#DIV/0!
**PSPC1**	0.04761904762	0.04761904762	0		inf	#DIV/0!
**PSPH**	0.2380952381	0.2380952381	0.2222222222		0.859375	#DIV/0!
**PSPN**	0.1428571429	0.1428571429	0.05555555556		2.333333333	#DIV/0!
**PSRC1**	0.04761904762	0.04761904762	0.05555555556		0.7	#DIV/0!
**PSTK**	0.2380952381	0.2380952381	0.05555555556		4.375	#DIV/0!
**PSTPIP1**	0.04761904762	0.04761904762	0.05555555556		0.7	#DIV/0!
**PSTPIP2**	0.04761904762	0.04761904762	0.05555555556		0.7	#DIV/0!
**PTAFR**	0.04761904762	0.04761904762	0.1111111111		0.325	#DIV/0!
**PTAR1**	0	0	0.05555555556		0	#DIV/0!
**PTBP1**	0.09523809524	0.09523809524	0.1111111111		0.6842105263	#DIV/0!
**PTBP2**	0.04761904762	0.04761904762	0.05555555556		0.7	#DIV/0!
**PTBP3**	0.04761904762	0.04761904762	0.05555555556		0.7	#DIV/0!
**PTCD1**	0.04761904762	0.04761904762	0.05555555556		0.7	#DIV/0!
**PTCD2**	0	0	0.05555555556		0	#DIV/0!
**PTCD3**	0	0	0.05555555556		0	#DIV/0!
**PTCH1**	0.04761904762	0.04761904762	0.05555555556		0.7	#DIV/0!
**PTCH2**	0.04761904762	0.04761904762	0.05555555556		0.7	#DIV/0!
**PTCHD3**	0.09523809524	0.09523809524	0.1111111111		0.6842105263	#DIV/0!
**PTCHD4**	0.04761904762	0.04761904762	0.05555555556		0.7	#DIV/0!
**PTCRA**	0.04761904762	0.04761904762	0.05555555556		0.7	#DIV/0!
**PTCSC1**	0.04761904762	0.04761904762	0.2222222222		0.1375	#DIV/0!
**PTCSC2**	0.04761904762	0.04761904762	0		inf	#DIV/0!
**PTDSS1**	0.1904761905	0.1904761905	0.1666666667		0.9411764706	#DIV/0!
**PTDSS2**	0.04761904762	0.04761904762	0		inf	#DIV/0!
**PTEN**	0.1428571429	0.1428571429	0.1666666667		0.6666666667	#DIV/0!
**PTENP1**	0.09523809524	0.09523809524	0.05555555556		1.473684211	#DIV/0!
**TENP1-A**	0.09523809524	0.09523809524	0.05555555556		1.473684211	#DIV/0!
**PTER**	0.09523809524	0.09523809524	0.1111111111		0.6842105263	#DIV/0!

PTF1A	0.09523809524	0.09523809524	0.1111111111		0.6842105263	#DIV/0!
PTGDR	0.04761904762	0.04761904762	0		inf	#DIV/0!
PTGDS	0.1428571429	0.1428571429	0.05555555556		2.333333333	#DIV/0!
PTGER1	0.1428571429	0.1428571429	0.1111111111		1.083333333	#DIV/0!
PTGER2	0.04761904762	0.04761904762	0		inf	#DIV/0!
PTGER3	0.04761904762	0.04761904762	0.05555555556		0.7	#DIV/0!
PTGER4	0.04761904762	0.04761904762	0.05555555556		0.7	#DIV/0!
4P2-CDK	0.09523809524	0.09523809524	0.2222222222		0.2894736842	#DIV/0!
PTGES	0.09523809524	0.09523809524	0.05555555556		1.473684211	#DIV/0!
PTGES2	0.09523809524	0.09523809524	0.05555555556		1.473684211	#DIV/0!
GES2-A	0.09523809524	0.09523809524	0.05555555556		1.473684211	#DIV/0!
PTGES3	0.1428571429	0.1428571429	0.05555555556		2.333333333	#DIV/0!
TGES3L	0.04761904762	0.04761904762	0		inf	#DIV/0!
S3L-AA	0.04761904762	0.04761904762	0		inf	#DIV/0!
PTGFR	0.04761904762	0.04761904762	0.05555555556		0.7	#DIV/0!
TGFRN	0.04761904762	0.04761904762	0.05555555556		0.7	#DIV/0!
PTGIR	0.09523809524	0.09523809524	0.1111111111		0.6842105263	#DIV/0!
PTGIS	0.09523809524	0.09523809524	0.1666666667		0.4210526316	#DIV/0!
PTGR1	0.04761904762	0.04761904762	0.05555555556		0.7	#DIV/0!
PTGR2	0.09523809524	0.09523809524	0		inf	#DIV/0!
PTGS1	0.09523809524	0.09523809524	0.05555555556		1.473684211	#DIV/0!
PTGS2	0.1428571429	0.1428571429	0.05555555556		2.333333333	#DIV/0!
PTH2	0.09523809524	0.09523809524	0.1111111111		0.6842105263	#DIV/0!
PTH2R	0	0	0.05555555556		0	#DIV/0!
PTHLH	0.04761904762	0.04761904762	0.05555555556		0.7	#DIV/0!
PTK2	0.04761904762	0.04761904762	0.2222222222		0.1375	#DIV/0!
PTK2B	0.04761904762	0.04761904762	0.1111111111		0.325	#DIV/0!
PTK6	0.1428571429	0.1428571429	0.2222222222		0.4583333333	#DIV/0!
PTK7	0.04761904762	0.04761904762	0.05555555556		0.7	#DIV/0!
PTMA	0	0	0.05555555556		0	#DIV/0!
TMAP1	0.04761904762	0.04761904762	0		inf	#DIV/0!
PTMS	0.04761904762	0.04761904762	0.1111111111		0.325	#DIV/0!
PTN	0.04761904762	0.04761904762	0.05555555556		0.7	#DIV/0!
PTOV1	0.09523809524	0.09523809524	0.1111111111		0.6842105263	#DIV/0!
TOV1-A	0.09523809524	0.09523809524	0.1111111111		0.6842105263	#DIV/0!
TOV1-A	0.09523809524	0.09523809524	0.1111111111		0.6842105263	#DIV/0!
PTP4A2	0.04761904762	0.04761904762	0.1111111111		0.325	#DIV/0!
PTP4A3	0.04761904762	0.04761904762	0.2222222222		0.1375	#DIV/0!
PTPA	0.09523809524	0.09523809524	0.05555555556		1.473684211	#DIV/0!
PTPDC1	0.04761904762	0.04761904762	0.05555555556		0.7	#DIV/0!
PTPMT1	0	0	0.05555555556		0	#DIV/0!
PTPN1	0.09523809524	0.09523809524	0.1666666667		0.4210526316	#DIV/0!
PTPN11	0	0	0.1111111111		0	#DIV/0!
PTPN12	0.04761904762	0.04761904762	0.1111111111		0.325	#DIV/0!
PTPN13	0	0	0.05555555556		0	#DIV/0!
PTPN14	0.1428571429	0.1428571429	0		inf	#DIV/0!
PTPN18	0	0	0.05555555556		0	#DIV/0!
PTPN2	0.09523809524	0.09523809524	0.05555555556		1.473684211	#DIV/0!
PTPN20	0.1428571429	0.1428571429	0.05555555556		2.333333333	#DIV/0!
PTPN22	0.04761904762	0.04761904762	0.05555555556		0.7	#DIV/0!
PTPN23	0.09523809524	0.09523809524	0		inf	#DIV/0!

PTPN3	0.04761904762	0.04761904762	0.05555555556		0.7	#DIV/0!
PTPN4	0	0	0.05555555556		0	#DIV/0!
PTPN6	0.04761904762	0.04761904762	0.1111111111		0.325	#DIV/0!
PTPN7	0.1428571429	0.1428571429	0		inf	#DIV/0!
PTPN9	0.04761904762	0.04761904762	0.05555555556		0.7	#DIV/0!
PTPRA	0.1904761905	0.1904761905	0.05555555556		3.294117647	#DIV/0!
PTPRB	0.09523809524	0.09523809524	0.1111111111		0.6842105263	#DIV/0!
PTPRC	0.1428571429	0.1428571429	0.1111111111		1.083333333	#DIV/0!
PTPRD	0.09523809524	0.09523809524	0.05555555556		1.473684211	#DIV/0!
TPRD-A	0.09523809524	0.09523809524	0.05555555556		1.473684211	#DIV/0!
TPRD-A	0.04761904762	0.04761904762	0.05555555556		0.7	#DIV/0!
PTPRE	0.2380952381	0.2380952381	0.05555555556		4.375	#DIV/0!
PTPRF	0.04761904762	0.04761904762	0.05555555556		0.7	#DIV/0!
PTPRG	0.04761904762	0.04761904762	0		inf	#DIV/0!
TPRG-A	0.04761904762	0.04761904762	0		inf	#DIV/0!
PTPRH	0.09523809524	0.09523809524	0.05555555556		1.473684211	#DIV/0!
PTPRJ	0	0	0.05555555556		0	#DIV/0!
PTPRM	0.09523809524	0.09523809524	0		inf	#DIV/0!
PTPRN2	0.1428571429	0.1428571429	0.05555555556		2.333333333	#DIV/0!
PTPRO	0.04761904762	0.04761904762	0.1111111111		0.325	#DIV/0!
PTPRQ	0.04761904762	0.04761904762	0.05555555556		0.7	#DIV/0!
PTPRR	0.09523809524	0.09523809524	0.1111111111		0.6842105263	#DIV/0!
PTPRS	0.1428571429	0.1428571429	0.05555555556		2.333333333	#DIV/0!
PTPRT	0.1428571429	0.1428571429	0.1111111111		1.083333333	#DIV/0!
PTPRU	0.04761904762	0.04761904762	0.1111111111		0.325	#DIV/0!
PTPRVP	0.1428571429	0.1428571429	0		inf	#DIV/0!
PTPRZ1	0.04761904762	0.04761904762	0.05555555556		0.7	#DIV/0!
PTRH1	0.09523809524	0.09523809524	0.05555555556		1.473684211	#DIV/0!
PTRH2	0.1428571429	0.1428571429	0		inf	#DIV/0!
PTTG1	0.04761904762	0.04761904762	0.05555555556		0.7	#DIV/0!
PTTG1IP	0.04761904762	0.04761904762	0.1111111111		0.325	#DIV/0!
PTTG3P	0.1428571429	0.1428571429	0.1666666667		0.6666666667	#DIV/0!
PTX3	0.1428571429	0.1428571429	0.05555555556		2.333333333	#DIV/0!
PTX4	0.09523809524	0.09523809524	0.2222222222		0.2894736842	#DIV/0!
PUF60	0.04761904762	0.04761904762	0.1666666667		0.2	#DIV/0!
PUM1	0.04761904762	0.04761904762	0.1111111111		0.325	#DIV/0!
PUM2	0	0	0.05555555556		0	#DIV/0!
PUM3	0.04761904762	0.04761904762	0.05555555556		0.7	#DIV/0!
PURA	0.04761904762	0.04761904762	0		inf	#DIV/0!
PURB	0.04761904762	0.04761904762	0.2222222222		0.1375	#DIV/0!
PURG	0.04761904762	0.04761904762	0.1111111111		0.325	#DIV/0!
PURPL	0.04761904762	0.04761904762	0.05555555556		0.7	#DIV/0!
PUS1	0.09523809524	0.09523809524	0.05555555556		1.473684211	#DIV/0!
PUS10	0.09523809524	0.09523809524	0.05555555556		1.473684211	#DIV/0!
PUS7	0.04761904762	0.04761904762	0.05555555556		0.7	#DIV/0!
PUS7L	0.04761904762	0.04761904762	0.05555555556		0.7	#DIV/0!
PUSL1	0	0	0.1111111111		0	#DIV/0!
PVALB	0	0	0.1111111111		0	#DIV/0!
PVALEF	0.04761904762	0.04761904762	0		inf	#DIV/0!
PVR	0.09523809524	0.09523809524	0.05555555556		1.473684211	#DIV/0!
PVRIG	0.04761904762	0.04761904762	0.05555555556		0.7	#DIV/0!

VRIG2I	0.04761904762	0.04761904762	0.05555555556		0.7	#DIV/0!
PVT1	0.09523809524	0.09523809524	0.1111111111		0.6842105263	#DIV/0!
PWP1	0.04761904762	0.04761904762	0.05555555556		0.7	#DIV/0!
PWP2	0.04761904762	0.04761904762	0.1111111111		0.325	#DIV/0!
WWP2A	0.04761904762	0.04761904762	0.05555555556		0.7	#DIV/0!
WWP2I	0.2380952381	0.2380952381	0		inf	#DIV/0!
WWP3A	0.09523809524	0.09523809524	0.1111111111		0.6842105263	#DIV/0!
PXDN	0	0	0.05555555556		0	#DIV/0!
PXDNL	0.09523809524	0.09523809524	0.1111111111		0.6842105263	#DIV/0!
PXK	0.04761904762	0.04761904762	0		inf	#DIV/0!
PXMP2	0.09523809524	0.09523809524	0.05555555556		1.473684211	#DIV/0!
PXMP4	0.09523809524	0.09523809524	0.1111111111		0.6842105263	#DIV/0!
PXN	0	0	0.1111111111		0	#DIV/0!
XN-AS1	0	0	0.1111111111		0	#DIV/0!
PXT1	0.04761904762	0.04761904762	0.05555555556		0.7	#DIV/0!
PXYLP1	0.1428571429	0.1428571429	0.05555555556		2.333333333	#DIV/0!
YCARI	0.04761904762	0.04761904762	0		inf	#DIV/0!
CARD-A	0.04761904762	0.04761904762	0		inf	#DIV/0!
PYCR1	0.04761904762	0.04761904762	0		inf	#DIV/0!
PYCR2	0.1428571429	0.1428571429	0.05555555556		2.333333333	#DIV/0!
PYCR3	0.04761904762	0.04761904762	0.1666666667		0.2	#DIV/0!
PYDC1	0.04761904762	0.04761904762	0		inf	#DIV/0!
PYDC2	0.09523809524	0.09523809524	0.05555555556		1.473684211	#DIV/0!
PYDC5	0.1428571429	0.1428571429	0		inf	#DIV/0!
PYGB	0.1904761905	0.1904761905	0.05555555556		3.294117647	#DIV/0!
PYGO1	0.04761904762	0.04761904762	0.05555555556		0.7	#DIV/0!
PYGO2	0.1904761905	0.1904761905	0.1111111111		1.529411765	#DIV/0!
PYHIN1	0.1428571429	0.1428571429	0		inf	#DIV/0!
PYM1	0.04761904762	0.04761904762	0.05555555556		0.7	#DIV/0!
YROXD	0.04761904762	0.04761904762	0.05555555556		0.7	#DIV/0!
YROXD	0.1428571429	0.1428571429	0		inf	#DIV/0!
PYURF	0	0	0.05555555556		0	#DIV/0!
PYY	0.09523809524	0.09523809524	0		inf	#DIV/0!
PYY2	0.04761904762	0.04761904762	0.05555555556		0.7	#DIV/0!
PZP	0.04761904762	0.04761904762	0.1111111111		0.325	#DIV/0!
QARS	0.09523809524	0.09523809524	0		inf	#DIV/0!
QDPR	0	0	0			#DIV/0!
QKI	0	0	0.1111111111		0	#DIV/0!
QPCT	0	0	0.05555555556		0	#DIV/0!
QPCTL	0.09523809524	0.09523809524	0.1111111111		0.6842105263	#DIV/0!
QPRT	0.04761904762	0.04761904762	0		inf	#DIV/0!
QRFP	0.09523809524	0.09523809524	0.05555555556		1.473684211	#DIV/0!
QRFPR	0	0	0.05555555556		0	#DIV/0!
QRICH1	0.09523809524	0.09523809524	0		inf	#DIV/0!
QRICH2	0.04761904762	0.04761904762	0		inf	#DIV/0!
QRSL1	0	0	0			#DIV/0!
QSER1	0.04761904762	0.04761904762	0		inf	#DIV/0!
QSOX1	0.1428571429	0.1428571429	0		inf	#DIV/0!
QSOX2	0.1428571429	0.1428571429	0.05555555556		2.333333333	#DIV/0!
QTRT1	0.1428571429	0.1428571429	0.1111111111		1.083333333	#DIV/0!
QTRT2	0.09523809524	0.09523809524	0.05555555556		1.473684211	#DIV/0!

R3HCC1	0.04761904762	0.04761904762	0.1111111111		0.325	#DIV/0!
R3HCC1	0.1428571429	0.1428571429	0		inf	#DIV/0!
R3HDM1	0.04761904762	0.04761904762	0.05555555556		0.7	#DIV/0!
R3HDM2	0.1428571429	0.1428571429	0.05555555556		2.333333333	#DIV/0!
R3HDM4	0.09523809524	0.09523809524	0.1111111111		0.6842105263	#DIV/0!
R3HDMI	0.1428571429	0.1428571429	0.1666666667		0.6666666667	#DIV/0!
RAB11A	0.04761904762	0.04761904762	0.05555555556		0.7	#DIV/0!
RAB11B	0.1428571429	0.1428571429	0.05555555556		2.333333333	#DIV/0!
AB11B-A	0.1428571429	0.1428571429	0.05555555556		2.333333333	#DIV/0!
AB11FII	0.09523809524	0.09523809524	0.1111111111		0.6842105263	#DIV/0!
AB11FII	0.2380952381	0.2380952381	0		inf	#DIV/0!
AB11FII	0.04761904762	0.04761904762	0.2222222222		0.1375	#DIV/0!
AB11FII	0	0	0			#DIV/0!
AB11FII	0	0	0.05555555556		0	#DIV/0!
RAB12	0.09523809524	0.09523809524	0		inf	#DIV/0!
RAB13	0.1904761905	0.1904761905	0.05555555556		3.294117647	#DIV/0!
RAB14	0.09523809524	0.09523809524	0.05555555556		1.473684211	#DIV/0!
RAB17	0	0	0.05555555556		0	#DIV/0!
RAB18	0.09523809524	0.09523809524	0.1111111111		0.6842105263	#DIV/0!
RAB19	0.04761904762	0.04761904762	0.05555555556		0.7	#DIV/0!
RAB1A	0	0	0.05555555556		0	#DIV/0!
RAB21	0.04761904762	0.04761904762	0.1111111111		0.325	#DIV/0!
RAB22A	0.1428571429	0.1428571429	0.2222222222		0.4583333333	#DIV/0!
RAB23	0.04761904762	0.04761904762	0.05555555556		0.7	#DIV/0!
RAB24	0.09523809524	0.09523809524	0.05555555556		1.473684211	#DIV/0!
RAB25	0.1428571429	0.1428571429	0.1111111111		1.083333333	#DIV/0!
RAB26	0.09523809524	0.09523809524	0.2222222222		0.2894736842	#DIV/0!
RAB27A	0.04761904762	0.04761904762	0.05555555556		0.7	#DIV/0!
RAB27B	0.09523809524	0.09523809524	0.05555555556		1.473684211	#DIV/0!
RAB28	0	0	0			#DIV/0!
RAB29	0.1428571429	0.1428571429	0		inf	#DIV/0!
RAB2A	0.1428571429	0.1428571429	0.1666666667		0.6666666667	#DIV/0!
RAB31	0.09523809524	0.09523809524	0.05555555556		1.473684211	#DIV/0!
RAB33B	0	0	0.05555555556		0	#DIV/0!
RAB34	0.04761904762	0.04761904762	0.05555555556		0.7	#DIV/0!
RAB35	0	0	0.1111111111		0	#DIV/0!
RAB36	0	0	0.05555555556		0	#DIV/0!
RAB37	0.04761904762	0.04761904762	0		inf	#DIV/0!
RAB3A	0.1428571429	0.1428571429	0.1111111111		1.083333333	#DIV/0!
RAB3B	0.04761904762	0.04761904762	0.05555555556		0.7	#DIV/0!
RAB3C	0.04761904762	0.04761904762	0		inf	#DIV/0!
RAB3D	0.1428571429	0.1428571429	0.1111111111		1.083333333	#DIV/0!
AB3GAI	0.04761904762	0.04761904762	0.05555555556		0.7	#DIV/0!
AB3GAI	0.1428571429	0.1428571429	0.05555555556		2.333333333	#DIV/0!
RAB3IP	0.09523809524	0.09523809524	0.1111111111		0.6842105263	#DIV/0!
RAB40B	0	0	0			#DIV/0!
RAB40C	0.04761904762	0.04761904762	0.2222222222		0.1375	#DIV/0!
RAB42	0.04761904762	0.04761904762	0.1111111111		0.325	#DIV/0!
RAB43	0.09523809524	0.09523809524	0.05555555556		1.473684211	#DIV/0!
RAB44	0.04761904762	0.04761904762	0.05555555556		0.7	#DIV/0!
RAB4A	0.1428571429	0.1428571429	0		inf	#DIV/0!

**RAB5A**	0.04761904762	0.04761904762	0		inf	#DIV/0!
**RAB5B**	0.09523809524	0.09523809524	0.05555555556		1.473684211	#DIV/0!
**RAB5C**	0.04761904762	0.04761904762	0		inf	#DIV/0!
**RAB5IF**	0.09523809524	0.09523809524	0.1111111111		0.6842105263	#DIV/0!
**RAB6B**	0.1428571429	0.1428571429	0.05555555556		2.333333333	#DIV/0!
**RAB6C**	0	0	0.05555555556		0	#DIV/0!
**AB6C-AS**	0	0	0.05555555556		0	#DIV/0!
**RAB6D**	0	0	0.05555555556		0	#DIV/0!
**RAB7A**	0.09523809524	0.09523809524	0.05555555556		1.473684211	#DIV/0!
**RAB8A**	0.1428571429	0.1428571429	0.1111111111		1.083333333	#DIV/0!
**RAB8B**	0.04761904762	0.04761904762	0.05555555556		0.7	#DIV/0!
**RAB9BP**	0.04761904762	0.04761904762	0		inf	#DIV/0!
**RABAC1**	0.04761904762	0.04761904762	0.05555555556		0.7	#DIV/0!
**RABEP1**	0	0	0.1111111111		0	#DIV/0!
**RABEP2**	0.04761904762	0.04761904762	0		inf	#DIV/0!
**RABEPK**	0.09523809524	0.09523809524	0.05555555556		1.473684211	#DIV/0!
**ABGAP**	0.09523809524	0.09523809524	0.05555555556		1.473684211	#DIV/0!
**ABGAP1**	0.1428571429	0.1428571429	0		inf	#DIV/0!
**ABGEF**	0.04761904762	0.04761904762	0.1111111111		0.325	#DIV/0!
**ABGGT**	0.04761904762	0.04761904762	0.05555555556		0.7	#DIV/0!
**RABIF**	0.1428571429	0.1428571429	0		inf	#DIV/0!
**RABL2A**	0	0	0.05555555556		0	#DIV/0!
**RABL2B**	0	0	0.1111111111		0	#DIV/0!
**RABL3**	0.09523809524	0.09523809524	0.05555555556		1.473684211	#DIV/0!
**RABL6**	0.1428571429	0.1428571429	0.05555555556		2.333333333	#DIV/0!
**RAC2**	0	0	0.1111111111		0	#DIV/0!
**RAC3**	0.04761904762	0.04761904762	0		inf	#DIV/0!
**ACGAP**	0.04761904762	0.04761904762	0.05555555556		0.7	#DIV/0!
**ACGAP1**	0.04761904762	0.04761904762	0.05555555556		0.7	#DIV/0!
**RACK1**	0.09523809524	0.09523809524	0.05555555556		1.473684211	#DIV/0!
**RAD1**	0.04761904762	0.04761904762	0.05555555556		0.7	#DIV/0!
**RAD17**	0.04761904762	0.04761904762	0.05555555556		0.7	#DIV/0!
**RAD18**	0.04761904762	0.04761904762	0		inf	#DIV/0!
**RAD21**	0.1904761905	0.1904761905	0.1666666667		0.9411764706	#DIV/0!
**AD21-AS**	0.1904761905	0.1904761905	0.1666666667		0.9411764706	#DIV/0!
**RAD21L**	0.1904761905	0.1904761905	0.1111111111		1.529411765	#DIV/0!
**RAD23A**	0.1428571429	0.1428571429	0.1111111111		1.083333333	#DIV/0!
**RAD23B**	0.04761904762	0.04761904762	0.05555555556		0.7	#DIV/0!
**RAD50**	0.04761904762	0.04761904762	0		inf	#DIV/0!
**AD51AP**	0.04761904762	0.04761904762	0.1111111111		0.325	#DIV/0!
**AD51AP**	0	0	0.05555555556		0	#DIV/0!
**RAD51B**	0	0	0.1666666667		0	#DIV/0!
**RAD51C**	0.1428571429	0.1428571429	0		inf	#DIV/0!
**RAD52**	0.04761904762	0.04761904762	0.1111111111		0.325	#DIV/0!
**RAD54B**	0.1904761905	0.1904761905	0.1111111111		1.529411765	#DIV/0!
**RAD54L**	0.04761904762	0.04761904762	0.05555555556		0.7	#DIV/0!
**RAD54L**	0.04761904762	0.04761904762	0		inf	#DIV/0!
**RAD9B**	0	0	0.1111111111		0	#DIV/0!
**RADIL**	0.04761904762	0.04761904762	0.1666666667		0.2	#DIV/0!
**RAE1**	0.1428571429	0.1428571429	0.2222222222		0.4583333333	#DIV/0!
**RAF1**	0.09523809524	0.09523809524	0		inf	#DIV/0!

RAI1	0.04761904762	0.04761904762	0.1111111111		0.325	#DIV/0!
RAI1-AS	0.04761904762	0.04761904762	0.1111111111		0.325	#DIV/0!
RAI14	0.04761904762	0.04761904762	0.1111111111		0.325	#DIV/0!
RALA	0.04761904762	0.04761904762	0.2222222222		0.1375	#DIV/0!
RALB	0	0	0.05555555556		0	#DIV/0!
RALBP1	0.09523809524	0.09523809524	0		inf	#DIV/0!
ALGAPA	0.09523809524	0.09523809524	0		inf	#DIV/0!
LGAPA	0.04761904762	0.04761904762	0.05555555556		0.7	#DIV/0!
ALGAPA	0.1904761905	0.1904761905	0.05555555556		3.294117647	#DIV/0!
ALGAP	0.1428571429	0.1428571429	0.1666666667		0.6666666667	#DIV/0!
RALGDS	0.04761904762	0.04761904762	0		inf	#DIV/0!
RALGPS	0.09523809524	0.09523809524	0.05555555556		1.473684211	#DIV/0!
RALGPS	0.1428571429	0.1428571429	0		inf	#DIV/0!
RALY	0.09523809524	0.09523809524	0.1111111111		0.6842105263	#DIV/0!
ALY-AS	0.09523809524	0.09523809524	0.1111111111		0.6842105263	#DIV/0!
RALYL	0.1428571429	0.1428571429	0.1111111111		1.083333333	#DIV/0!
RAMAC	0.04761904762	0.04761904762	0.05555555556		0.7	#DIV/0!
RAMP1	0	0	0.05555555556		0	#DIV/0!
RAMP2	0.04761904762	0.04761904762	0		inf	#DIV/0!
AMP2-A	0.04761904762	0.04761904762	0		inf	#DIV/0!
RAMP3	0.04761904762	0.04761904762	0.2222222222		0.1375	#DIV/0!
RAN	0.09523809524	0.09523809524	0.05555555556		1.473684211	#DIV/0!
RANBP1	0.04761904762	0.04761904762	0.05555555556		0.7	#DIV/0!
RANBP1	0.1428571429	0.1428571429	0.05555555556		2.333333333	#DIV/0!
RANBP1	0	0	0.05555555556		0	#DIV/0!
RANBP2	0	0	0.05555555556		0	#DIV/0!
RANBP3	0.1428571429	0.1428571429	0.05555555556		2.333333333	#DIV/0!
RANBP3	0.04761904762	0.04761904762	0.05555555556		0.7	#DIV/0!
RANBP6	0.04761904762	0.04761904762	0.05555555556		0.7	#DIV/0!
ANGAP	0	0	0.1111111111		0	#DIV/0!
RANGRI	0.04761904762	0.04761904762	0.05555555556		0.7	#DIV/0!
RAP1A	0.04761904762	0.04761904762	0		inf	#DIV/0!
RAP1B	0.09523809524	0.09523809524	0.05555555556		1.473684211	#DIV/0!
RAP1GA	0.04761904762	0.04761904762	0.1111111111		0.325	#DIV/0!
AP1GAI	0	0	0.1111111111		0	#DIV/0!
AP1GDS	0	0	0.05555555556		0	#DIV/0!
RAP2B	0.1904761905	0.1904761905	0.1111111111		1.529411765	#DIV/0!
RAPGEF	0.09523809524	0.09523809524	0.05555555556		1.473684211	#DIV/0!
RAPGEF	0	0	0.05555555556		0	#DIV/0!
RAPGEF	0.04761904762	0.04761904762	0.05555555556		0.7	#DIV/0!
RAPGEF	0	0	0.1111111111		0	#DIV/0!
PGEF4-A	0	0	0.1111111111		0	#DIV/0!
RAPGEF	0.04761904762	0.04761904762	0.1666666667		0.2	#DIV/0!
RAPGEF	0.04761904762	0.04761904762	0		inf	#DIV/0!
APGEFI	0.09523809524	0.09523809524	0		inf	#DIV/0!
RAPH1	0.04761904762	0.04761904762	0.05555555556		0.7	#DIV/0!
RAPSN	0	0	0.05555555556		0	#DIV/0!
RARA	0.09523809524	0.09523809524	0		inf	#DIV/0!
ARA-AS	0.09523809524	0.09523809524	0		inf	#DIV/0!
RARB	0.04761904762	0.04761904762	0		inf	#DIV/0!
ARB-AS	0.04761904762	0.04761904762	0		inf	#DIV/0!

**RARG**	0.04761904762	0.04761904762	0.05555555556		0.7	#DIV/0!
**ARRES**	0.1428571429	0.1428571429	0.05555555556		2.333333333	#DIV/0!
**ARRES**	0.09523809524	0.09523809524	0.05555555556		1.473684211	#DIV/0!
**RARS**	0	0	0.05555555556		0	#DIV/0!
**RASA1**	0.04761904762	0.04761904762	0		inf	#DIV/0!
**RASA2**	0.1904761905	0.1904761905	0.05555555556		3.294117647	#DIV/0!
**RASA4**	0.04761904762	0.04761904762	0.05555555556		0.7	#DIV/0!
**RASA4B**	0.04761904762	0.04761904762	0.05555555556		0.7	#DIV/0!
**ASA4C**	0.04761904762	0.04761904762	0.1666666667		0.2	#DIV/0!
**ASA4D**	0.04761904762	0.04761904762	0.05555555556		0.7	#DIV/0!
**RASAL1**	0	0	0.05555555556		0	#DIV/0!
**RASAL2**	0.1428571429	0.1428571429	0		inf	#DIV/0!
**SAL2-A**	0.1428571429	0.1428571429	0		inf	#DIV/0!
**RASD1**	0.04761904762	0.04761904762	0.1111111111		0.325	#DIV/0!
**RASD2**	0	0	0.1111111111		0	#DIV/0!
**RASEF**	0	0	0.05555555556		0	#DIV/0!
**ASGEF1**	0.1428571429	0.1428571429	0.05555555556		2.333333333	#DIV/0!
**ASGEF1**	0	0	0.05555555556		0	#DIV/0!
**ASGEF1**	0.09523809524	0.09523809524	0.05555555556		1.473684211	#DIV/0!
**ASGRF**	0.04761904762	0.04761904762	0.05555555556		0.7	#DIV/0!
**ASGRF**	0.04761904762	0.04761904762	0		inf	#DIV/0!
**SGRF2-**	0.04761904762	0.04761904762	0		inf	#DIV/0!
**ASGRP**	0	0	0			#DIV/0!
**ASGRP**	0.04761904762	0.04761904762	0.05555555556		0.7	#DIV/0!
**RASIP1**	0.09523809524	0.09523809524	0.1111111111		0.6842105263	#DIV/0!
**ASL10**	0.04761904762	0.04761904762	0.1111111111		0.325	#DIV/0!
**ASL10**	0.09523809524	0.09523809524	0		inf	#DIV/0!
**ASL11**	0.2380952381	0.2380952381	0.05555555556		4.375	#DIV/0!
**RASL12**	0.04761904762	0.04761904762	0.05555555556		0.7	#DIV/0!
**RASSF1**	0.09523809524	0.09523809524	0		inf	#DIV/0!
**SSF1-A**	0.09523809524	0.09523809524	0		inf	#DIV/0!
**RASSF2**	0.1904761905	0.1904761905	0.05555555556		3.294117647	#DIV/0!
**RASSF3**	0.1428571429	0.1428571429	0.05555555556		2.333333333	#DIV/0!
**RASSF4**	0.1428571429	0.1428571429	0.05555555556		2.333333333	#DIV/0!
**RASSF5**	0.1428571429	0.1428571429	0		inf	#DIV/0!
**RASSF6**	0.09523809524	0.09523809524	0.05555555556		1.473684211	#DIV/0!
**RASSF7**	0.04761904762	0.04761904762	0		inf	#DIV/0!
**RASSF8**	0.04761904762	0.04761904762	0.05555555556		0.7	#DIV/0!
**SSF8-A**	0.04761904762	0.04761904762	0.05555555556		0.7	#DIV/0!
**RASSF9**	0.04761904762	0.04761904762	0.05555555556		0.7	#DIV/0!
**RAVER**	0.1428571429	0.1428571429	0.1111111111		1.083333333	#DIV/0!
**RAVER**	0.04761904762	0.04761904762	0.05555555556		0.7	#DIV/0!
**RAX**	0.09523809524	0.09523809524	0		inf	#DIV/0!
**RAX2**	0.09523809524	0.09523809524	0.05555555556		1.473684211	#DIV/0!
**RB1CC1**	0.09523809524	0.09523809524	0.1111111111		0.6842105263	#DIV/0!
**RBAK**	0.04761904762	0.04761904762	0.1666666667		0.2	#DIV/0!
**K-RBA**	0.04761904762	0.04761904762	0.1666666667		0.2	#DIV/0!
**RBAKD**	0.04761904762	0.04761904762	0.1666666667		0.2	#DIV/0!
**RBBP4**	0.04761904762	0.04761904762	0.1111111111		0.325	#DIV/0!
**RBBP5**	0.1904761905	0.1904761905	0		inf	#DIV/0!
**RBBP6**	0.04761904762	0.04761904762	0		inf	#DIV/0!

RBBP8	0.04761904762	0.04761904762	0.05555555556		0.7	#DIV/0!
RBBP8NL	0.1428571429	0.1428571429	0.2222222222		0.4583333333	#DIV/0!
RBBP9	0.1904761905	0.1904761905	0.05555555556		3.294117647	#DIV/0!
RBCK1	0.1904761905	0.1904761905	0.05555555556		3.294117647	#DIV/0!
RBFA	0.04761904762	0.04761904762	0.05555555556		0.7	#DIV/0!
RBFADN	0.04761904762	0.04761904762	0.05555555556		0.7	#DIV/0!
RBFOX1	0.04761904762	0.04761904762	0		inf	#DIV/0!
RBFOX2	0	0	0.1111111111		0	#DIV/0!
RBFOX3	0.04761904762	0.04761904762	0		inf	#DIV/0!
RBIS	0.1428571429	0.1428571429	0.1111111111		1.083333333	#DIV/0!
RBKS	0	0	0.1111111111		0	#DIV/0!
RBL1	0.09523809524	0.09523809524	0.1111111111		0.6842105263	#DIV/0!
RBL2	0.04761904762	0.04761904762	0.05555555556		0.7	#DIV/0!
RBM11	0.04761904762	0.04761904762	0.1111111111		0.325	#DIV/0!
RBM12	0.09523809524	0.09523809524	0.1111111111		0.6842105263	#DIV/0!
RBM12B	0.1904761905	0.1904761905	0.1111111111		1.529411765	#DIV/0!
M12B-A	0.1904761905	0.1904761905	0.1111111111		1.529411765	#DIV/0!
RBM15	0.04761904762	0.04761904762	0		inf	#DIV/0!
BM15-AS	0.04761904762	0.04761904762	0		inf	#DIV/0!
RBM15B	0.04761904762	0.04761904762	0		inf	#DIV/0!
RBM17	0.09523809524	0.09523809524	0.1111111111		0.6842105263	#DIV/0!
RBM18	0.09523809524	0.09523809524	0.05555555556		1.473684211	#DIV/0!
RBM19	0	0	0.05555555556		0	#DIV/0!
RBM20	0.1428571429	0.1428571429	0.05555555556		2.333333333	#DIV/0!
RBM23	0	0	0.05555555556		0	#DIV/0!
RBM25	0.09523809524	0.09523809524	0		inf	#DIV/0!
RBM28	0.04761904762	0.04761904762	0.05555555556		0.7	#DIV/0!
RBM33	0.1428571429	0.1428571429	0.05555555556		2.333333333	#DIV/0!
RBM34	0.1428571429	0.1428571429	0.05555555556		2.333333333	#DIV/0!
RBM38	0.1428571429	0.1428571429	0.2222222222		0.4583333333	#DIV/0!
RBM39	0.09523809524	0.09523809524	0.1111111111		0.6842105263	#DIV/0!
RBM42	0.04761904762	0.04761904762	0.05555555556		0.7	#DIV/0!
RBM43	0	0	0.05555555556		0	#DIV/0!
RBM44	0	0	0.05555555556		0	#DIV/0!
RBM45	0	0	0.1111111111		0	#DIV/0!
RBM46	0	0	0.05555555556		0	#DIV/0!
RBM47	0.1904761905	0.1904761905	0.05555555556		3.294117647	#DIV/0!
RBM48	0.04761904762	0.04761904762	0.1111111111		0.325	#DIV/0!
RBM5	0.09523809524	0.09523809524	0		inf	#DIV/0!
BM5-AS	0.09523809524	0.09523809524	0		inf	#DIV/0!
RBM6	0.09523809524	0.09523809524	0		inf	#DIV/0!
RBMS1	0	0	0.05555555556		0	#DIV/0!
RBMS2	0.1428571429	0.1428571429	0.05555555556		2.333333333	#DIV/0!
RBMS3	0.04761904762	0.04761904762	0		inf	#DIV/0!
BMS3-A	0.04761904762	0.04761904762	0		inf	#DIV/0!
BMS3-A	0.04761904762	0.04761904762	0		inf	#DIV/0!
RBMXL1	0.04761904762	0.04761904762	0.05555555556		0.7	#DIV/0!
RBMXL2	0.04761904762	0.04761904762	0		inf	#DIV/0!
RBP1	0.1428571429	0.1428571429	0.05555555556		2.333333333	#DIV/0!
RBP2	0.1428571429	0.1428571429	0.05555555556		2.333333333	#DIV/0!
RBP3	0.1428571429	0.1428571429	0.05555555556		2.333333333	#DIV/0!

RBP4	0.1428571429	0.1428571429	0.05555555556		2.333333333	#DIV/0!
RBP5	0.04761904762	0.04761904762	0.1111111111		0.325	#DIV/0!
RBP7	0	0	0.1111111111		0	#DIV/0!
RBPJL	0.1428571429	0.1428571429	0.1666666667		0.6666666667	#DIV/0!
RBPMS	0.04761904762	0.04761904762	0.1111111111		0.325	#DIV/0!
BPMS-A	0.04761904762	0.04761904762	0.1111111111		0.325	#DIV/0!
RBPMS2	0.04761904762	0.04761904762	0.05555555556		0.7	#DIV/0!
RBSN	0.04761904762	0.04761904762	0		inf	#DIV/0!
RBX1	0	0	0.1111111111		0	#DIV/0!
RC3H1	0.1428571429	0.1428571429	0		inf	#DIV/0!
RC3H2	0.09523809524	0.09523809524	0.05555555556		1.473684211	#DIV/0!
RCAN1	0.04761904762	0.04761904762	0.1666666667		0.2	#DIV/0!
RCAN2	0.04761904762	0.04761904762	0.05555555556		0.7	#DIV/0!
RCAN3	0.04761904762	0.04761904762	0.1111111111		0.325	#DIV/0!
CAN3A	0.04761904762	0.04761904762	0.1111111111		0.325	#DIV/0!
RCC1	0.04761904762	0.04761904762	0.1111111111		0.325	#DIV/0!
RCC1L	0.1428571429	0.1428571429	0.1666666667		0.6666666667	#DIV/0!
RCC2	0.04761904762	0.04761904762	0.1111111111		0.325	#DIV/0!
RCCD1	0.04761904762	0.04761904762	0.05555555556		0.7	#DIV/0!
RCHY1	0.04761904762	0.04761904762	0.05555555556		0.7	#DIV/0!
RCL1	0.04761904762	0.04761904762	0.05555555556		0.7	#DIV/0!
RCN2	0.04761904762	0.04761904762	0.05555555556		0.7	#DIV/0!
RCN3	0.09523809524	0.09523809524	0.1111111111		0.6842105263	#DIV/0!
RCOR3	0.1428571429	0.1428571429	0		inf	#DIV/0!
RCSD1	0.1428571429	0.1428571429	0		inf	#DIV/0!
RD3	0.1428571429	0.1428571429	0		inf	#DIV/0!
RDH10	0.1904761905	0.1904761905	0.1666666667		0.9411764706	#DIV/0!
DH10-AS	0.1904761905	0.1904761905	0.1666666667		0.9411764706	#DIV/0!
RDH13	0.09523809524	0.09523809524	0.05555555556		1.473684211	#DIV/0!
RDH14	0	0	0.05555555556		0	#DIV/0!
RDH16	0.1428571429	0.1428571429	0.05555555556		2.333333333	#DIV/0!
RDH5	0.04761904762	0.04761904762	0.05555555556		0.7	#DIV/0!
RDH8	0.1428571429	0.1428571429	0.1111111111		1.083333333	#DIV/0!
RDM1	0.09523809524	0.09523809524	0		inf	#DIV/0!
RDM1P5	0.09523809524	0.09523809524	0		inf	#DIV/0!
REC114	0.04761904762	0.04761904762	0.05555555556		0.7	#DIV/0!
RECK	0.09523809524	0.09523809524	0.05555555556		1.473684211	#DIV/0!
RECQL	0.04761904762	0.04761904762	0.05555555556		0.7	#DIV/0!
RECQL4	0.04761904762	0.04761904762	0.1666666667		0.2	#DIV/0!
RECQL5	0.04761904762	0.04761904762	0.05555555556		0.7	#DIV/0!
REELD1	0	0	0.05555555556		0	#DIV/0!
REEP1	0	0	0.05555555556		0	#DIV/0!
REEP2	0.04761904762	0.04761904762	0		inf	#DIV/0!
REEP3	0.09523809524	0.09523809524	0.1111111111		0.6842105263	#DIV/0!
REEP4	0.09523809524	0.09523809524	0.1111111111		0.6842105263	#DIV/0!
REEP5	0.04761904762	0.04761904762	0		inf	#DIV/0!
REEP6	0.09523809524	0.09523809524	0.1111111111		0.6842105263	#DIV/0!
REG1A	0	0	0.05555555556		0	#DIV/0!
REG1B	0	0	0.05555555556		0	#DIV/0!
REG1CP	0	0	0.05555555556		0	#DIV/0!
REG3A	0	0	0.05555555556		0	#DIV/0!

REG3G	0	0	0.05555555556		0	#DIV/0!
REG4	0.04761904762	0.04761904762	0.05555555556		0.7	#DIV/0!
REL	0.09523809524	0.09523809524	0.05555555556		1.473684211	#DIV/0!
RELB	0.09523809524	0.09523809524	0.1111111111		0.6842105263	#DIV/0!
RELCH	0.09523809524	0.09523809524	0		inf	#DIV/0!
RELL2	0.04761904762	0.04761904762	0		inf	#DIV/0!
RELN	0.04761904762	0.04761904762	0.05555555556		0.7	#DIV/0!
REM1	0.09523809524	0.09523809524	0.05555555556		1.473684211	#DIV/0!
REM2	0	0	0.05555555556		0	#DIV/0!
REN	0.2380952381	0.2380952381	0		inf	#DIV/0!
REP15	0.04761904762	0.04761904762	0.05555555556		0.7	#DIV/0!
REPIN1	0.09523809524	0.09523809524	0.05555555556		1.473684211	#DIV/0!
RER1	0	0	0.1111111111		0	#DIV/0!
RERE	0	0	0.05555555556		0	#DIV/0!
ERE-AS	0	0	0.05555555556		0	#DIV/0!
REREP3	0.2380952381	0.2380952381	0.1111111111		2.03125	#DIV/0!
RERG	0.04761904762	0.04761904762	0.1111111111		0.325	#DIV/0!
ERG-AS	0.04761904762	0.04761904762	0.1111111111		0.325	#DIV/0!
RESF1	0.1428571429	0.1428571429	0.1111111111		1.083333333	#DIV/0!
REST	0.1904761905	0.1904761905	0.05555555556		3.294117647	#DIV/0!
RET	0.1428571429	0.1428571429	0.05555555556		2.333333333	#DIV/0!
RETN	0.1428571429	0.1428571429	0.05555555556		2.333333333	#DIV/0!
RETNLE	0.09523809524	0.09523809524	0.05555555556		1.473684211	#DIV/0!
ETREG	0.04761904762	0.04761904762	0.05555555556		0.7	#DIV/0!
ETREG	0.04761904762	0.04761904762	0		inf	#DIV/0!
RETSAT	0	0	0.05555555556		0	#DIV/0!
REV1	0	0	0.05555555556		0	#DIV/0!
REV3L	0	0	0			#DIV/0!
REX1BD	0.1428571429	0.1428571429	0.1111111111		1.083333333	#DIV/0!
REXO1	0.09523809524	0.09523809524	0.1111111111		0.6842105263	#DIV/0!
EXO1L2	0.1904761905	0.1904761905	0.1111111111		1.529411765	#DIV/0!
REXO4	0.04761904762	0.04761904762	0		inf	#DIV/0!
REXO5	0.04761904762	0.04761904762	0		inf	#DIV/0!
RFC1	0.1904761905	0.1904761905	0.05555555556		3.294117647	#DIV/0!
RFC2	0.1428571429	0.1428571429	0.1111111111		1.083333333	#DIV/0!
RFC4	0.09523809524	0.09523809524	0.05555555556		1.473684211	#DIV/0!
RFC5	0	0	0.05555555556		0	#DIV/0!
RFESD	0.04761904762	0.04761904762	0		inf	#DIV/0!
RFK	0	0	0.05555555556		0	#DIV/0!
RFLNA	0.04761904762	0.04761904762	0.05555555556		0.7	#DIV/0!
RFLNB	0	0	0.1111111111		0	#DIV/0!
RFNG	0.04761904762	0.04761904762	0		inf	#DIV/0!
RFPL1	0.04761904762	0.04761904762	0.1111111111		0.325	#DIV/0!
RFPL1S	0.04761904762	0.04761904762	0.1111111111		0.325	#DIV/0!
RFPL2	0.04761904762	0.04761904762	0.1111111111		0.325	#DIV/0!
RFPL3	0.04761904762	0.04761904762	0.1111111111		0.325	#DIV/0!
RFPL3S	0.04761904762	0.04761904762	0.1111111111		0.325	#DIV/0!
RFPL4A	0.1428571429	0.1428571429	0.05555555556		2.333333333	#DIV/0!
FPL4AL	0.1428571429	0.1428571429	0.05555555556		2.333333333	#DIV/0!
RFT1	0.04761904762	0.04761904762	0		inf	#DIV/0!
RFTN1	0.04761904762	0.04761904762	0		inf	#DIV/0!

RFTN2	0	0	0.05555555556		0	#DIV/0!
RFWD3	0.04761904762	0.04761904762	0		inf	#DIV/0!
RFX1	0.1428571429	0.1428571429	0.1111111111		1.083333333	#DIV/0!
RFX2	0.1428571429	0.1428571429	0.05555555556		2.333333333	#DIV/0!
RFX3	0.04761904762	0.04761904762	0.05555555556		0.7	#DIV/0!
RFX3-AS	0.04761904762	0.04761904762	0.05555555556		0.7	#DIV/0!
RFX4	0.04761904762	0.04761904762	0.05555555556		0.7	#DIV/0!
RFX5	0.1904761905	0.1904761905	0.05555555556		3.294117647	#DIV/0!
RFX7	0.04761904762	0.04761904762	0.05555555556		0.7	#DIV/0!
RFX8	0	0	0.05555555556		0	#DIV/0!
RFXANK	0.1428571429	0.1428571429	0.1111111111		1.083333333	#DIV/0!
RGMA	0.04761904762	0.04761904762	0.05555555556		0.7	#DIV/0!
RGMB	0.04761904762	0.04761904762	0		inf	#DIV/0!
RGMB-AS	0.04761904762	0.04761904762	0		inf	#DIV/0!
RGP1	0.09523809524	0.09523809524	0.05555555556		1.473684211	#DIV/0!
RGPD1	0	0	0.05555555556		0	#DIV/0!
RGPD2	0	0	0.05555555556		0	#DIV/0!
RGPD3	0	0	0.05555555556		0	#DIV/0!
RGPD4	0	0	0.05555555556		0	#DIV/0!
RGPD4-AS	0	0	0.05555555556		0	#DIV/0!
RGPD5	0	0	0.05555555556		0	#DIV/0!
RGPD6	0	0	0.05555555556		0	#DIV/0!
RGPD8	0	0	0.05555555556		0	#DIV/0!
RGR	0.1428571429	0.1428571429	0		inf	#DIV/0!
RGS1	0.1428571429	0.1428571429	0		inf	#DIV/0!
RGS10	0.2380952381	0.2380952381	0		inf	#DIV/0!
RGS11	0.04761904762	0.04761904762	0.2222222222		0.1375	#DIV/0!
RGS12	0.04761904762	0.04761904762	0		inf	#DIV/0!
RGS13	0.1428571429	0.1428571429	0		inf	#DIV/0!
RGS14	0.09523809524	0.09523809524	0.05555555556		1.473684211	#DIV/0!
RGS16	0.1428571429	0.1428571429	0		inf	#DIV/0!
RGS18	0.1428571429	0.1428571429	0		inf	#DIV/0!
RGS19	0.1428571429	0.1428571429	0.2222222222		0.4583333333	#DIV/0!
RGS2	0.1428571429	0.1428571429	0		inf	#DIV/0!
RGS20	0.1428571429	0.1428571429	0.1111111111		1.083333333	#DIV/0!
RGS21	0.1428571429	0.1428571429	0		inf	#DIV/0!
RGS22	0.1904761905	0.1904761905	0.1111111111		1.529411765	#DIV/0!
RGS3	0.04761904762	0.04761904762	0.05555555556		0.7	#DIV/0!
RGS4	0.1428571429	0.1428571429	0		inf	#DIV/0!
RGS5	0.1428571429	0.1428571429	0		inf	#DIV/0!
RGS6	0.04761904762	0.04761904762	0		inf	#DIV/0!
RGS7	0.1428571429	0.1428571429	0		inf	#DIV/0!
RGS7BP	0.04761904762	0.04761904762	0		inf	#DIV/0!
RGS8	0.1428571429	0.1428571429	0		inf	#DIV/0!
RGS9	0.04761904762	0.04761904762	0		inf	#DIV/0!
RGS9BP	0.04761904762	0.04761904762	0.05555555556		0.7	#DIV/0!
RGSL1	0.1428571429	0.1428571429	0		inf	#DIV/0!
RHAG	0.04761904762	0.04761904762	0		inf	#DIV/0!
RHBDD1	0	0	0.05555555556		0	#DIV/0!
RHBDD2	0.1428571429	0.1428571429	0.1111111111		1.083333333	#DIV/0!
RHBDD3	0.04761904762	0.04761904762	0.1111111111		0.325	#DIV/0!

RHBDF1	0.04761904762	0.04761904762	0.2222222222		0.1375	#DIV/0!
RHBDF2	0.04761904762	0.04761904762	0		inf	#DIV/0!
RHBDL1	0.04761904762	0.04761904762	0.2222222222		0.1375	#DIV/0!
RHBDL2	0.04761904762	0.04761904762	0.05555555556		0.7	#DIV/0!
RHBDL3	0	0	0			#DIV/0!
RHBG	0.1428571429	0.1428571429	0.1111111111		1.083333333	#DIV/0!
RHCE	0.04761904762	0.04761904762	0.1111111111		0.325	#DIV/0!
RHCG	0.04761904762	0.04761904762	0.05555555556		0.7	#DIV/0!
RHD	0.04761904762	0.04761904762	0.1111111111		0.325	#DIV/0!
RHEB	0.09523809524	0.09523809524	0.05555555556		1.473684211	#DIV/0!
RHEBL1	0.04761904762	0.04761904762	0.05555555556		0.7	#DIV/0!
RHEX	0.1428571429	0.1428571429	0		inf	#DIV/0!
RHNO1	0.04761904762	0.04761904762	0.1666666667		0.2	#DIV/0!
RHO	0.09523809524	0.09523809524	0.05555555556		1.473684211	#DIV/0!
RHOA	0.1428571429	0.1428571429	0		inf	#DIV/0!
RHOB	0	0	0.05555555556		0	#DIV/0!
HOBTB	0.04761904762	0.04761904762	0.1111111111		0.325	#DIV/0!
HOBTB	0.04761904762	0.04761904762	0.1111111111		0.325	#DIV/0!
HOBTB	0.04761904762	0.04761904762	0		inf	#DIV/0!
RHOC	0.04761904762	0.04761904762	0.05555555556		0.7	#DIV/0!
RHOF	0.04761904762	0.04761904762	0.1111111111		0.325	#DIV/0!
RHOG	0.04761904762	0.04761904762	0		inf	#DIV/0!
RHOH	0.1904761905	0.1904761905	0.05555555556		3.294117647	#DIV/0!
RHOQ	0	0	0.05555555556		0	#DIV/0!
RHOT1	0	0	0			#DIV/0!
RHOT2	0.04761904762	0.04761904762	0.2222222222		0.1375	#DIV/0!
RHOU	0.1428571429	0.1428571429	0		inf	#DIV/0!
RHPN1	0.04761904762	0.04761904762	0.05555555556		0.7	#DIV/0!
HPN1-A	0.04761904762	0.04761904762	0.05555555556		0.7	#DIV/0!
RHPN2	0.04761904762	0.04761904762	0.05555555556		0.7	#DIV/0!
RIBC2	0	0	0.1111111111		0	#DIV/0!
RIC1	0.04761904762	0.04761904762	0.05555555556		0.7	#DIV/0!
RIC8A	0.04761904762	0.04761904762	0		inf	#DIV/0!
RIC8B	0.04761904762	0.04761904762	0.05555555556		0.7	#DIV/0!
RICTOR	0.04761904762	0.04761904762	0.05555555556		0.7	#DIV/0!
RIDA	0.1904761905	0.1904761905	0.1111111111		1.529411765	#DIV/0!
RIF1	0	0	0.05555555556		0	#DIV/0!
RIIAD1	0.1428571429	0.1428571429	0		inf	#DIV/0!
RILP	0	0	0.1111111111		0	#DIV/0!
RILPL1	0.04761904762	0.04761904762	0.1111111111		0.325	#DIV/0!
RILPL2	0.04761904762	0.04761904762	0.1111111111		0.325	#DIV/0!
RIMBP2	0.09523809524	0.09523809524	0.05555555556		1.473684211	#DIV/0!
RIMBP3	0.04761904762	0.04761904762	0.05555555556		0.7	#DIV/0!
IMBP3I	0.04761904762	0.04761904762	0.05555555556		0.7	#DIV/0!
IMBP3C	0.04761904762	0.04761904762	0.05555555556		0.7	#DIV/0!
RIMKLA	0.04761904762	0.04761904762	0.05555555556		0.7	#DIV/0!
RIMKLB	0.04761904762	0.04761904762	0.1111111111		0.325	#DIV/0!
RIMS2	0.1904761905	0.1904761905	0.1111111111		1.529411765	#DIV/0!
RIMS3	0.04761904762	0.04761904762	0.05555555556		0.7	#DIV/0!
RIMS4	0.1428571429	0.1428571429	0.1666666667		0.6666666667	#DIV/0!
RIN2	0.1904761905	0.1904761905	0.05555555556		3.294117647	#DIV/0!

**RINL**	0.04761904762	0.04761904762	0.05555555556		0.7	#DIV/0!
**RINT1**	0.04761904762	0.04761904762	0.05555555556		0.7	#DIV/0!
**RIOK2**	0.04761904762	0.04761904762	0		inf	#DIV/0!
**RIOK3**	0.04761904762	0.04761904762	0.05555555556		0.7	#DIV/0!
**RIOX1**	0.09523809524	0.09523809524	0		inf	#DIV/0!
**RIOX2**	0.04761904762	0.04761904762	0.05555555556		0.7	#DIV/0!
**RIPK2**	0.1904761905	0.1904761905	0.1111111111		1.529411765	#DIV/0!
**RIPK4**	0.04761904762	0.04761904762	0.1111111111		0.325	#DIV/0!
**RIPOR1**	0.1428571429	0.1428571429	0.05555555556		2.333333333	#DIV/0!
**RIPOR2**	0	0	0.05555555556		0	#DIV/0!
**RIPOR3**	0.09523809524	0.09523809524	0.1666666667		0.4210526316	#DIV/0!
**RIPPLY**	0.04761904762	0.04761904762	0.1666666667		0.2	#DIV/0!
**RIT1**	0.1904761905	0.1904761905	0.1111111111		1.529411765	#DIV/0!
**RIT2**	0.04761904762	0.04761904762	0		inf	#DIV/0!
**RITA1**	0	0	0.05555555556		0	#DIV/0!
**RLBP1**	0.04761904762	0.04761904762	0.05555555556		0.7	#DIV/0!
**RLF**	0.04761904762	0.04761904762	0.05555555556		0.7	#DIV/0!
**RLN1**	0.04761904762	0.04761904762	0.05555555556		0.7	#DIV/0!
**RLN2**	0.04761904762	0.04761904762	0.05555555556		0.7	#DIV/0!
**RLN3**	0.1428571429	0.1428571429	0.1111111111		1.083333333	#DIV/0!
**RMC1**	0.04761904762	0.04761904762	0.05555555556		0.7	#DIV/0!
**RMDN1**	0.1904761905	0.1904761905	0.1111111111		1.529411765	#DIV/0!
**RMDN2**	0	0	0.05555555556		0	#DIV/0!
**MDN2-A**	0	0	0.05555555556		0	#DIV/0!
**RMI1**	0	0	0.05555555556		0	#DIV/0!
**RMI2**	0.04761904762	0.04761904762	0		inf	#DIV/0!
**RMND5A**	0	0	0.05555555556		0	#DIV/0!
**RMND5B**	0.09523809524	0.09523809524	0.05555555556		1.473684211	#DIV/0!
**RMRP**	0.09523809524	0.09523809524	0.05555555556		1.473684211	#DIV/0!
**RMST**	0.1904761905	0.1904761905	0.05555555556		3.294117647	#DIV/0!
**RN7SK**	0.04761904762	0.04761904762	0		inf	#DIV/0!
**RN7SL1**	0.04761904762	0.04761904762	0		inf	#DIV/0!
**RN7SL2**	0.04761904762	0.04761904762	0		inf	#DIV/0!
**RN7SL3**	0.04761904762	0.04761904762	0		inf	#DIV/0!
**N7SL832**	0	0	0.05555555556		0	#DIV/0!
**RNA5S1**	0.1428571429	0.1428571429	0		inf	#DIV/0!
**RNA5S1(**	0.1428571429	0.1428571429	0		inf	#DIV/0!
**RNA5S1**	0.1428571429	0.1428571429	0		inf	#DIV/0!
**RNA5S1**	0.1428571429	0.1428571429	0		inf	#DIV/0!
**RNA5S1**	0.1428571429	0.1428571429	0		inf	#DIV/0!
**RNA5S1**	0.1428571429	0.1428571429	0		inf	#DIV/0!
**RNA5S1**	0.1428571429	0.1428571429	0		inf	#DIV/0!
**RNA5S1(**	0.1428571429	0.1428571429	0		inf	#DIV/0!
**RNA5S1**	0.1428571429	0.1428571429	0		inf	#DIV/0!
**RNA5S2**	0.1428571429	0.1428571429	0		inf	#DIV/0!
**RNA5S3**	0.1428571429	0.1428571429	0		inf	#DIV/0!
**RNA5S4**	0.1428571429	0.1428571429	0		inf	#DIV/0!
**RNA5S5**	0.1428571429	0.1428571429	0		inf	#DIV/0!
**RNA5S6**	0.1428571429	0.1428571429	0		inf	#DIV/0!
**RNA5S7**	0.1428571429	0.1428571429	0		inf	#DIV/0!
**RNA5S8**	0.1428571429	0.1428571429	0		inf	#DIV/0!

RNA5S9	0.1428571429	0.1428571429	0		inf	#DIV/0!
RNASEH	0	0	0.05555555556		0	#DIV/0!
ASEH1-A	0	0	0.05555555556		0	#DIV/0!
NASEH2	0.1428571429	0.1428571429	0.1111111111		1.083333333	#DIV/0!
RNASEK	0	0	0.05555555556		0	#DIV/0!
SEK-C17	0	0	0.05555555556		0	#DIV/0!
RNASEL	0.1428571429	0.1428571429	0		inf	#DIV/0!
RND1	0.04761904762	0.04761904762	0.05555555556		0.7	#DIV/0!
RND2	0.04761904762	0.04761904762	0		inf	#DIV/0!
RND3	0	0	0.05555555556		0	#DIV/0!
RNF10	0	0	0.1111111111		0	#DIV/0!
RNF103	0	0	0.05555555556		0	#DIV/0!
103-CH	0	0	0.05555555556		0	#DIV/0!
RNF11	0.04761904762	0.04761904762	0.05555555556		0.7	#DIV/0!
RNF111	0.04761904762	0.04761904762	0.05555555556		0.7	#DIV/0!
RNF112	0.04761904762	0.04761904762	0.05555555556		0.7	#DIV/0!
RNF114	0.09523809524	0.09523809524	0.1666666667		0.4210526316	#DIV/0!
RNF115	0.1428571429	0.1428571429	0.05555555556		2.333333333	#DIV/0!
RNF122	0.04761904762	0.04761904762	0.1111111111		0.325	#DIV/0!
RNF123	0.09523809524	0.09523809524	0		inf	#DIV/0!
RNF125	0.09523809524	0.09523809524	0		inf	#DIV/0!
RNF126	0.09523809524	0.09523809524	0.1111111111		0.6842105263	#DIV/0!
NF126P	0.09523809524	0.09523809524	0		inf	#DIV/0!
RNF13	0.1904761905	0.1904761905	0.1111111111		1.529411765	#DIV/0!
RNF130	0.09523809524	0.09523809524	0.05555555556		1.473684211	#DIV/0!
RNF133	0.04761904762	0.04761904762	0.05555555556		0.7	#DIV/0!
RNF135	0	0	0			#DIV/0!
RNF138	0.09523809524	0.09523809524	0		inf	#DIV/0!
NF138P	0.04761904762	0.04761904762	0.05555555556		0.7	#DIV/0!
RNF139	0.1428571429	0.1428571429	0.1111111111		1.083333333	#DIV/0!
NF139-A	0.1428571429	0.1428571429	0.1111111111		1.083333333	#DIV/0!
RNF14	0.04761904762	0.04761904762	0		inf	#DIV/0!
NF144A	0	0	0.05555555556		0	#DIV/0!
F144A-A	0	0	0.05555555556		0	#DIV/0!
RNF145	0.04761904762	0.04761904762	0.05555555556		0.7	#DIV/0!
RNF148	0.04761904762	0.04761904762	0.05555555556		0.7	#DIV/0!
RNF149	0	0	0.05555555556		0	#DIV/0!
RNF150	0	0	0.05555555556		0	#DIV/0!
RNF151	0.09523809524	0.09523809524	0.2222222222		0.2894736842	#DIV/0!
RNF152	0.09523809524	0.09523809524	0		inf	#DIV/0!
RNF157	0.04761904762	0.04761904762	0		inf	#DIV/0!
NF157-A	0.04761904762	0.04761904762	0		inf	#DIV/0!
RNF165	0.04761904762	0.04761904762	0.05555555556		0.7	#DIV/0!
RNF166	0.1904761905	0.1904761905	0		inf	#DIV/0!
RNF167	0	0	0.1111111111		0	#DIV/0!
RNF168	0.1428571429	0.1428571429	0.05555555556		2.333333333	#DIV/0!
RNF170	0.09523809524	0.09523809524	0.1666666667		0.4210526316	#DIV/0!
RNF175	0	0	0.05555555556		0	#DIV/0!
RNF180	0.04761904762	0.04761904762	0		inf	#DIV/0!
RNF181	0	0	0.05555555556		0	#DIV/0!
RNF183	0.04761904762	0.04761904762	0.05555555556		0.7	#DIV/0!

RNF185	0.04761904762	0.04761904762	0.1111111111		0.325	#DIV/0!
RNF186	0.04761904762	0.04761904762	0.1111111111		0.325	#DIV/0!
RNF187	0.1428571429	0.1428571429	0		inf	#DIV/0!
RNF19A	0.1904761905	0.1904761905	0.1111111111		1.529411765	#DIV/0!
RNF19B	0.04761904762	0.04761904762	0.1111111111		0.325	#DIV/0!
RNF2	0.1428571429	0.1428571429	0.05555555556		2.333333333	#DIV/0!
RNF20	0.04761904762	0.04761904762	0		inf	#DIV/0!
RNF207	0	0	0.05555555556		0	#DIV/0!
RNF208	0.1428571429	0.1428571429	0.05555555556		2.333333333	#DIV/0!
RNF212	0.04761904762	0.04761904762	0		inf	#DIV/0!
RNF212E	0	0	0.1111111111		0	#DIV/0!
RNF213	0.04761904762	0.04761904762	0		inf	#DIV/0!
RNF215	0.04761904762	0.04761904762	0.1111111111		0.325	#DIV/0!
RNF216	0.04761904762	0.04761904762	0.1666666667		0.2	#DIV/0!
NF216-IT	0.04761904762	0.04761904762	0.1666666667		0.2	#DIV/0!
RNF216P	0.04761904762	0.04761904762	0.1666666667		0.2	#DIV/0!
RNF220	0.04761904762	0.04761904762	0.05555555556		0.7	#DIV/0!
RNF222	0.04761904762	0.04761904762	0.05555555556		0.7	#DIV/0!
RNF223	0	0	0.1111111111		0	#DIV/0!
RNF224	0.1428571429	0.1428571429	0.05555555556		2.333333333	#DIV/0!
RNF225	0.1428571429	0.1428571429	0.05555555556		2.333333333	#DIV/0!
RNF227	0	0	0.05555555556		0	#DIV/0!
RNF24	0.1904761905	0.1904761905	0.05555555556		3.294117647	#DIV/0!
RNF25	0	0	0.05555555556		0	#DIV/0!
RNF32	0.1428571429	0.1428571429	0.05555555556		2.333333333	#DIV/0!
RNF34	0.04761904762	0.04761904762	0.1111111111		0.325	#DIV/0!
RNF38	0.09523809524	0.09523809524	0.05555555556		1.473684211	#DIV/0!
RNF4	0.04761904762	0.04761904762	0		inf	#DIV/0!
RNF40	0.04761904762	0.04761904762	0		inf	#DIV/0!
RNF41	0.09523809524	0.09523809524	0.05555555556		1.473684211	#DIV/0!
RNF43	0.09523809524	0.09523809524	0		inf	#DIV/0!
RNF44	0.09523809524	0.09523809524	0.05555555556		1.473684211	#DIV/0!
RNF5	0.04761904762	0.04761904762	0		inf	#DIV/0!
RNF5P1	0.09523809524	0.09523809524	0.1111111111		0.6842105263	#DIV/0!
RNF7	0.1904761905	0.1904761905	0.05555555556		3.294117647	#DIV/0!
RNF8	0.04761904762	0.04761904762	0.05555555556		0.7	#DIV/0!
RNFT1	0.1428571429	0.1428571429	0		inf	#DIV/0!
NFT1-D	0.1428571429	0.1428571429	0		inf	#DIV/0!
RNFT2	0	0	0.05555555556		0	#DIV/0!
RNH1	0.04761904762	0.04761904762	0		inf	#DIV/0!
RNLS	0.1428571429	0.1428571429	0.1666666667		0.6666666667	#DIV/0!
RNMT	0.09523809524	0.09523809524	0.05555555556		1.473684211	#DIV/0!
RNPC3	0.04761904762	0.04761904762	0.05555555556		0.7	#DIV/0!
RNPEP	0.1428571429	0.1428571429	0		inf	#DIV/0!
RNPEPL	0	0	0.05555555556		0	#DIV/0!
RNPS1	0.09523809524	0.09523809524	0.2222222222		0.2894736842	#DIV/0!
RNU1-1	0.1904761905	0.1904761905	0.1666666667		0.9411764706	#DIV/0!
RNU1-2	0.1904761905	0.1904761905	0.1666666667		0.9411764706	#DIV/0!
RNU1-3	0.1904761905	0.1904761905	0.1666666667		0.9411764706	#DIV/0!
RNU1-4	0.1904761905	0.1904761905	0.1666666667		0.9411764706	#DIV/0!
RNU105E	0.1904761905	0.1904761905	0.1111111111		1.529411765	#DIV/0!

RNU105C	0.1428571429	0.1428571429	0.1111111111		1.083333333	#DIV/0!
RNU11	0.04761904762	0.04761904762	0.1111111111		0.325	#DIV/0!
RNU4-1	0	0	0.1111111111		0	#DIV/0!
RNU4-2	0	0	0.1111111111		0	#DIV/0!
NU4ATA	0	0	0.05555555556		0	#DIV/0!
RNU5A-1	0.04761904762	0.04761904762	0.05555555556		0.7	#DIV/0!
RNU5B-1	0.04761904762	0.04761904762	0.05555555556		0.7	#DIV/0!
RNU5D-1	0.04761904762	0.04761904762	0.05555555556		0.7	#DIV/0!
RNU5E-1	0.04761904762	0.04761904762	0.1111111111		0.325	#DIV/0!
RNU5F-1	0.04761904762	0.04761904762	0.05555555556		0.7	#DIV/0!
RNU6-1	0.380952381	0.380952381	0.4444444444		0.5384615385	#DIV/0!
RNU6-2	0.380952381	0.380952381	0.4444444444		0.5384615385	#DIV/0!
RNU6-7	0.380952381	0.380952381	0.4444444444		0.5384615385	#DIV/0!
RNU6-8	0.380952381	0.380952381	0.4444444444		0.5384615385	#DIV/0!
RNU6-9	0.380952381	0.380952381	0.4444444444		0.5384615385	#DIV/0!
NU6ATA	0.04761904762	0.04761904762	0		inf	#DIV/0!
U6ATAC	0.1428571429	0.1428571429	0.05555555556		2.333333333	#DIV/0!
RNU7-1	0.04761904762	0.04761904762	0.1111111111		0.325	#DIV/0!
RNVU1-1	0.1428571429	0.1428571429	0.05555555556		2.333333333	#DIV/0!
RNVU1-1	0.1428571429	0.1428571429	0.05555555556		2.333333333	#DIV/0!
RNVU1-1	0.1428571429	0.1428571429	0.05555555556		2.333333333	#DIV/0!
RNVU1-1	0.09523809524	0.09523809524	0.05555555556		1.473684211	#DIV/0!
RNVU1-1	0.1904761905	0.1904761905	0.1666666667		0.9411764706	#DIV/0!
RNVU1-1	0.1428571429	0.1428571429	0.05555555556		2.333333333	#DIV/0!
RNVU1-2	0.09523809524	0.09523809524	0.05555555556		1.473684211	#DIV/0!
RNVU1-3	0.1428571429	0.1428571429	0.05555555556		2.333333333	#DIV/0!
RNVU1-4	0.09523809524	0.09523809524	0.05555555556		1.473684211	#DIV/0!
RNVU1-6	0.1428571429	0.1428571429	0.05555555556		2.333333333	#DIV/0!
RNVU1-7	0.1428571429	0.1428571429	0.05555555556		2.333333333	#DIV/0!
RNVU1-8	0.1428571429	0.1428571429	0.05555555556		2.333333333	#DIV/0!
RNY1	0.09523809524	0.09523809524	0.05555555556		1.473684211	#DIV/0!
RNY3	0.09523809524	0.09523809524	0.05555555556		1.473684211	#DIV/0!
RNY4	0.09523809524	0.09523809524	0.05555555556		1.473684211	#DIV/0!
RNY5	0.09523809524	0.09523809524	0.05555555556		1.473684211	#DIV/0!
ROBO1	0.04761904762	0.04761904762	0.05555555556		0.7	#DIV/0!
ROBO2	0.04761904762	0.04761904762	0		inf	#DIV/0!
ROCK1	0.04761904762	0.04761904762	0.1111111111		0.325	#DIV/0!
ROCK1P	0.09523809524	0.09523809524	0		inf	#DIV/0!
ROCK2	0	0	0.05555555556		0	#DIV/0!
ROCR	0	0	0			#DIV/0!
ROGDI	0.04761904762	0.04761904762	0		inf	#DIV/0!
ROMO1	0.09523809524	0.09523809524	0.1111111111		0.6842105263	#DIV/0!
ROPN1	0.04761904762	0.04761904762	0.05555555556		0.7	#DIV/0!
ROPN1B	0.04761904762	0.04761904762	0.05555555556		0.7	#DIV/0!
ROPN1L	0.04761904762	0.04761904762	0.05555555556		0.7	#DIV/0!
PN1L-A	0.04761904762	0.04761904762	0.05555555556		0.7	#DIV/0!
ROR1	0.04761904762	0.04761904762	0.05555555556		0.7	#DIV/0!
OR1-AS	0.04761904762	0.04761904762	0.05555555556		0.7	#DIV/0!
ROR2	0.04761904762	0.04761904762	0.05555555556		0.7	#DIV/0!
RORA	0.04761904762	0.04761904762	0.05555555556		0.7	#DIV/0!
ORA-AS	0.04761904762	0.04761904762	0.05555555556		0.7	#DIV/0!

ORA-AS	0.04761904762	0.04761904762	0.05555555556		0.7	#DIV/0!
RORB	0	0	0.05555555556		0	#DIV/0!
ORB-AS	0	0	0.05555555556		0	#DIV/0!
RORC	0.1904761905	0.1904761905	0		inf	#DIV/0!
RP1	0.1428571429	0.1428571429	0.1111111111		1.083333333	#DIV/0!
RP1L1	0.09523809524	0.09523809524	0.1111111111		0.6842105263	#DIV/0!
RP9	0.04761904762	0.04761904762	0.1666666667		0.2	#DIV/0!
RP9P	0.04761904762	0.04761904762	0.1666666667		0.2	#DIV/0!
RPA1	0	0	0.1111111111		0	#DIV/0!
RPA2	0.04761904762	0.04761904762	0.1111111111		0.325	#DIV/0!
RPA3	0.04761904762	0.04761904762	0.1666666667		0.2	#DIV/0!
RPAIN	0	0	0.05555555556		0	#DIV/0!
RPAP2	0.04761904762	0.04761904762	0.05555555556		0.7	#DIV/0!
RPAP3	0.04761904762	0.04761904762	0.05555555556		0.7	#DIV/0!
PARP-AS	0.1428571429	0.1428571429	0.05555555556		2.333333333	#DIV/0!
RPE	0	0	0.05555555556		0	#DIV/0!
RPE65	0.04761904762	0.04761904762	0.05555555556		0.7	#DIV/0!
RPEL1	0.1428571429	0.1428571429	0.05555555556		2.333333333	#DIV/0!
RPF1	0.04761904762	0.04761904762	0.05555555556		0.7	#DIV/0!
RPF2	0	0	0			#DIV/0!
PGRIP1	0.04761904762	0.04761904762	0.05555555556		0.7	#DIV/0!
RPH3A	0	0	0.05555555556		0	#DIV/0!
RPH3AL	0	0	0.1111111111		0	#DIV/0!
RPIA	0	0	0.05555555556		0	#DIV/0!
RPL10A	0.04761904762	0.04761904762	0.05555555556		0.7	#DIV/0!
RPL10L	0.04761904762	0.04761904762	0		inf	#DIV/0!
RPL11	0.04761904762	0.04761904762	0.1111111111		0.325	#DIV/0!
RPL12	0.09523809524	0.09523809524	0.05555555556		1.473684211	#DIV/0!
RPL13	0.09523809524	0.09523809524	0		inf	#DIV/0!
RPL13A	0.09523809524	0.09523809524	0.1111111111		0.6842105263	#DIV/0!
PL13AP	0.04761904762	0.04761904762	0.1111111111		0.325	#DIV/0!
PL13AP	0.04761904762	0.04761904762	0.05555555556		0.7	#DIV/0!
PL13AP	0.04761904762	0.04761904762	0		inf	#DIV/0!
PL13AP	0.1428571429	0.1428571429	0		inf	#DIV/0!
PL13AP	0.1428571429	0.1428571429	0.05555555556		2.333333333	#DIV/0!
RPL13P5	0.04761904762	0.04761904762	0.1111111111		0.325	#DIV/0!
RPL14	0.04761904762	0.04761904762	0		inf	#DIV/0!
RPL15	0.04761904762	0.04761904762	0		inf	#DIV/0!
RPL17	0.04761904762	0.04761904762	0		inf	#DIV/0!
17-C18o	0.04761904762	0.04761904762	0		inf	#DIV/0!
RPL18	0.09523809524	0.09523809524	0.1111111111		0.6842105263	#DIV/0!
RPL18A	0.1428571429	0.1428571429	0.1111111111		1.083333333	#DIV/0!
RPL19	0.09523809524	0.09523809524	0		inf	#DIV/0!
RPL19P1	0.04761904762	0.04761904762	0.05555555556		0.7	#DIV/0!
RPL21	0.2380952381	0.2380952381	0		inf	#DIV/0!
RPL21P2	0.1428571429	0.1428571429	0		inf	#DIV/0!
RPL21P4	0.2380952381	0.2380952381	0.05555555556		4.375	#DIV/0!
RPL22	0	0	0.05555555556		0	#DIV/0!
RPL22L1	0.1904761905	0.1904761905	0.05555555556		3.294117647	#DIV/0!
RPL23	0.09523809524	0.09523809524	0		inf	#DIV/0!
RPL23A	0.04761904762	0.04761904762	0.05555555556		0.7	#DIV/0!

PL23AP	0.04761904762	0.04761904762	0.05555555556		0.7	#DIV/0!
PL23AP	0.09523809524	0.09523809524	0.1111111111		0.6842105263	#DIV/0!
PL23AP	0	0	0.05555555556		0	#DIV/0!
PL23AP	0	0	0.1111111111		0	#DIV/0!
RPL23P	0.04761904762	0.04761904762	0.1666666667		0.2	#DIV/0!
RPL24	0.09523809524	0.09523809524	0.05555555556		1.473684211	#DIV/0!
RPL26	0.04761904762	0.04761904762	0.05555555556		0.7	#DIV/0!
RPL26L	0	0	0.05555555556		0	#DIV/0!
RPL27	0.04761904762	0.04761904762	0		inf	#DIV/0!
RPL28	0.09523809524	0.09523809524	0.05555555556		1.473684211	#DIV/0!
RPL29	0.04761904762	0.04761904762	0		inf	#DIV/0!
RPL29P	0	0	0.05555555556		0	#DIV/0!
RPL3	0	0	0.1111111111		0	#DIV/0!
RPL30	0.1904761905	0.1904761905	0.1111111111		1.529411765	#DIV/0!
RPL31	0	0	0.05555555556		0	#DIV/0!
RPL31P1	0.1428571429	0.1428571429	0		inf	#DIV/0!
RPL32	0.04761904762	0.04761904762	0		inf	#DIV/0!
RPL32P	0.09523809524	0.09523809524	0.05555555556		1.473684211	#DIV/0!
RPL34	0	0	0.05555555556		0	#DIV/0!
PL34-AS	0	0	0.05555555556		0	#DIV/0!
RPL35	0.09523809524	0.09523809524	0.05555555556		1.473684211	#DIV/0!
RPL35A	0.1428571429	0.1428571429	0.05555555556		2.333333333	#DIV/0!
RPL36	0.1428571429	0.1428571429	0.05555555556		2.333333333	#DIV/0!
RPL36A	0.04761904762	0.04761904762	0		inf	#DIV/0!
RPL37	0.04761904762	0.04761904762	0.05555555556		0.7	#DIV/0!
RPL37A	0	0	0.05555555556		0	#DIV/0!
RPL38	0.04761904762	0.04761904762	0		inf	#DIV/0!
RPL39L	0.09523809524	0.09523809524	0.05555555556		1.473684211	#DIV/0!
RPL3L	0.09523809524	0.09523809524	0.2222222222		0.2894736842	#DIV/0!
RPL4	0.04761904762	0.04761904762	0.05555555556		0.7	#DIV/0!
RPL41	0.09523809524	0.09523809524	0.05555555556		1.473684211	#DIV/0!
RPL5	0.04761904762	0.04761904762	0.05555555556		0.7	#DIV/0!
RPL6	0	0	0.1111111111		0	#DIV/0!
RPL7	0.1904761905	0.1904761905	0.1666666667		0.9411764706	#DIV/0!
RPL7A	0.04761904762	0.04761904762	0		inf	#DIV/0!
RPL7L1	0.04761904762	0.04761904762	0.05555555556		0.7	#DIV/0!
RPL8	0.09523809524	0.09523809524	0.2222222222		0.2894736842	#DIV/0!
RPL9	0.1904761905	0.1904761905	0.05555555556		3.294117647	#DIV/0!
RPLP0	0	0	0.1111111111		0	#DIV/0!
RPLP1	0.04761904762	0.04761904762	0.05555555556		0.7	#DIV/0!
RPLP2	0.04761904762	0.04761904762	0		inf	#DIV/0!
RPN1	0.09523809524	0.09523809524	0.05555555556		1.473684211	#DIV/0!
RPN2	0.09523809524	0.09523809524	0.1666666667		0.4210526316	#DIV/0!
RPP14	0.04761904762	0.04761904762	0		inf	#DIV/0!
RPP25	0.04761904762	0.04761904762	0.05555555556		0.7	#DIV/0!
RPP25L	0.09523809524	0.09523809524	0.05555555556		1.473684211	#DIV/0!
RPP30	0.1428571429	0.1428571429	0.1111111111		1.083333333	#DIV/0!
RPP38	0.09523809524	0.09523809524	0.1111111111		0.6842105263	#DIV/0!
PP38-D	0.09523809524	0.09523809524	0.1111111111		0.6842105263	#DIV/0!
RPPH1	0.04761904762	0.04761904762	0		inf	#DIV/0!
RPRD1A	0.04761904762	0.04761904762	0		inf	#DIV/0!

RPRD1B	0.1428571429	0.1428571429	0.1666666667		0.6666666667	#DIV/0!
RPRD2	0.1904761905	0.1904761905	0.05555555556		3.294117647	#DIV/0!
RPRM	0	0	0.05555555556		0	#DIV/0!
RPRML	0.1428571429	0.1428571429	0		inf	#DIV/0!
RPS10	0.04761904762	0.04761904762	0		inf	#DIV/0!
S10-NUD	0.04761904762	0.04761904762	0		inf	#DIV/0!
RPS10P7	0.1428571429	0.1428571429	0		inf	#DIV/0!
RPS11	0.09523809524	0.09523809524	0.1111111111		0.6842105263	#DIV/0!
RPS14P3	0.04761904762	0.04761904762	0.1111111111		0.325	#DIV/0!
RPS15	0.09523809524	0.09523809524	0.1111111111		0.6842105263	#DIV/0!
RPS15A	0.09523809524	0.09523809524	0		inf	#DIV/0!
PS15AP1	0.04761904762	0.04761904762	0.05555555556		0.7	#DIV/0!
RPS16	0.04761904762	0.04761904762	0.05555555556		0.7	#DIV/0!
RPS16P5	0.04761904762	0.04761904762	0		inf	#DIV/0!
RPS17	0.04761904762	0.04761904762	0.05555555556		0.7	#DIV/0!
RPS18P9	0	0	0			#DIV/0!
RPS19	0.04761904762	0.04761904762	0.05555555556		0.7	#DIV/0!
RPS19BP	0	0	0.1111111111		0	#DIV/0!
RPS2	0.09523809524	0.09523809524	0.2222222222		0.2894736842	#DIV/0!
RPS20	0.1428571429	0.1428571429	0.1111111111		1.083333333	#DIV/0!
RPS21	0.1428571429	0.1428571429	0.2222222222		0.4583333333	#DIV/0!
RPS23	0.04761904762	0.04761904762	0.05555555556		0.7	#DIV/0!
RPS24	0.1428571429	0.1428571429	0.05555555556		2.333333333	#DIV/0!
RPS26	0.09523809524	0.09523809524	0.05555555556		1.473684211	#DIV/0!
RPS27	0.1904761905	0.1904761905	0.05555555556		3.294117647	#DIV/0!
RPS27A	0.04761904762	0.04761904762	0.05555555556		0.7	#DIV/0!
RPS27L	0.04761904762	0.04761904762	0.05555555556		0.7	#DIV/0!
RPS28	0.1428571429	0.1428571429	0.05555555556		2.333333333	#DIV/0!
RPS29	0.04761904762	0.04761904762	0		inf	#DIV/0!
RPS2P32	0.04761904762	0.04761904762	0.1666666667		0.2	#DIV/0!
RPS3A	0	0	0.05555555556		0	#DIV/0!
RPS5	0.1428571429	0.1428571429	0.05555555556		2.333333333	#DIV/0!
RPS6	0.04761904762	0.04761904762	0.05555555556		0.7	#DIV/0!
RPS6KA	0.04761904762	0.04761904762	0.1111111111		0.325	#DIV/0!
RPS6KB	0.1428571429	0.1428571429	0		inf	#DIV/0!
RPS6KC	0.1428571429	0.1428571429	0		inf	#DIV/0!
RPS7	0	0	0.05555555556		0	#DIV/0!
RPS7P5	0.1428571429	0.1428571429	0		inf	#DIV/0!
RPS8	0.04761904762	0.04761904762	0.05555555556		0.7	#DIV/0!
RPS9	0.09523809524	0.09523809524	0.05555555556		1.473684211	#DIV/0!
RPSA	0.04761904762	0.04761904762	0		inf	#DIV/0!
RPSAP52	0.09523809524	0.09523809524	0.05555555556		1.473684211	#DIV/0!
RPSAP58	0.09523809524	0.09523809524	0		inf	#DIV/0!
RPSAP9	0	0	0.05555555556		0	#DIV/0!
RPTN	0.1904761905	0.1904761905	0		inf	#DIV/0!
RPTOR	0.04761904762	0.04761904762	0		inf	#DIV/0!
RPUSD1	0.04761904762	0.04761904762	0.2222222222		0.1375	#DIV/0!
RPUSD3	0.09523809524	0.09523809524	0		inf	#DIV/0!
RRAD	0.09523809524	0.09523809524	0.05555555556		1.473684211	#DIV/0!
RRAGA	0.04761904762	0.04761904762	0.05555555556		0.7	#DIV/0!
RRAGC	0.04761904762	0.04761904762	0.05555555556		0.7	#DIV/0!

**RRAS**	0.09523809524	0.09523809524	0.1111111111		0.6842105263	#DIV/0!
**RRBP1**	0.1904761905	0.1904761905	0.1111111111		1.529411765	#DIV/0!
**RRH**	0	0	0.05555555556		0	#DIV/0!
**RRM1**	0.04761904762	0.04761904762	0		inf	#DIV/0!
**RRM2**	0	0	0.05555555556		0	#DIV/0!
**RRM2B**	0.1904761905	0.1904761905	0.1111111111		1.529411765	#DIV/0!
**RRN3**	0.04761904762	0.04761904762	0		inf	#DIV/0!
**RRN3P1**	0.04761904762	0.04761904762	0		inf	#DIV/0!
**RRN3P2**	0.04761904762	0.04761904762	0		inf	#DIV/0!
**RRN3P3**	0.04761904762	0.04761904762	0		inf	#DIV/0!
**RRNAD1**	0.1428571429	0.1428571429	0.1111111111		1.083333333	#DIV/0!
**RRP1**	0.04761904762	0.04761904762	0.1111111111		0.325	#DIV/0!
**RRP12**	0.1428571429	0.1428571429	0		inf	#DIV/0!
**RRP15**	0.1428571429	0.1428571429	0.05555555556		2.333333333	#DIV/0!
**RRP1B**	0.04761904762	0.04761904762	0.1666666667		0.2	#DIV/0!
**RRP36**	0.04761904762	0.04761904762	0.05555555556		0.7	#DIV/0!
**RRP7A**	0	0	0.1666666667		0	#DIV/0!
**RRP7BP**	0	0	0.1666666667		0	#DIV/0!
**RRP8**	0.04761904762	0.04761904762	0		inf	#DIV/0!
**RRP9**	0.04761904762	0.04761904762	0		inf	#DIV/0!
**RRS1**	0.1428571429	0.1428571429	0.1666666667		0.6666666667	#DIV/0!
**RRS1-AS**	0.1428571429	0.1428571429	0.1666666667		0.6666666667	#DIV/0!
**RSAD1**	0.09523809524	0.09523809524	0		inf	#DIV/0!
**RSAD2**	0	0	0.05555555556		0	#DIV/0!
**RSBN1**	0.04761904762	0.04761904762	0.05555555556		0.7	#DIV/0!
**RSBN1L**	0.04761904762	0.04761904762	0.1111111111		0.325	#DIV/0!
**RSC1A1**	0.04761904762	0.04761904762	0.1111111111		0.325	#DIV/0!
**RSKR**	0.04761904762	0.04761904762	0.05555555556		0.7	#DIV/0!
**RSL1D1**	0.04761904762	0.04761904762	0		inf	#DIV/0!
**RSL24D1**	0.04761904762	0.04761904762	0.05555555556		0.7	#DIV/0!
**RSPH1**	0.04761904762	0.04761904762	0.1111111111		0.325	#DIV/0!
**RSPH10B**	0.04761904762	0.04761904762	0.1666666667		0.2	#DIV/0!
**SPH10B**	0.04761904762	0.04761904762	0.1666666667		0.2	#DIV/0!
**RSPH14**	0	0	0.05555555556		0	#DIV/0!
**RSPH6A**	0.09523809524	0.09523809524	0.1111111111		0.6842105263	#DIV/0!
**RSPH9**	0.04761904762	0.04761904762	0.05555555556		0.7	#DIV/0!
**RSPO1**	0.04761904762	0.04761904762	0.05555555556		0.7	#DIV/0!
**RSPO2**	0.1904761905	0.1904761905	0.1111111111		1.529411765	#DIV/0!
**RSPO4**	0.1904761905	0.1904761905	0.1111111111		1.529411765	#DIV/0!
**RSPRY1**	0.04761904762	0.04761904762	0		inf	#DIV/0!
**RSRC1**	0.1428571429	0.1428571429	0.05555555556		2.333333333	#DIV/0!
**RSRC2**	0.04761904762	0.04761904762	0.1111111111		0.325	#DIV/0!
**RSRP1**	0.04761904762	0.04761904762	0.1111111111		0.325	#DIV/0!
**RSU1**	0.09523809524	0.09523809524	0.1111111111		0.6842105263	#DIV/0!
**RSU1P2**	0.1428571429	0.1428571429	0.05555555556		2.333333333	#DIV/0!
**RTBDN**	0.1428571429	0.1428571429	0.1111111111		1.083333333	#DIV/0!
**RTCA**	0.04761904762	0.04761904762	0.05555555556		0.7	#DIV/0!
**TCA-AS**	0.04761904762	0.04761904762	0.05555555556		0.7	#DIV/0!
**RTCB**	0.04761904762	0.04761904762	0.1111111111		0.325	#DIV/0!
**RTEL1**	0.1428571429	0.1428571429	0.2222222222		0.4583333333	#DIV/0!
**1-TNFR**	0.1428571429	0.1428571429	0.2222222222		0.4583333333	#DIV/0!

RTF2	0.09523809524	0.09523809524	0.1666666667		0.4210526316	#DIV/0!
RTKN	0	0	0.05555555556		0	#DIV/0!
RTKN2	0.04761904762	0.04761904762	0.1666666667		0.2	#DIV/0!
RTL10	0.04761904762	0.04761904762	0.05555555556		0.7	#DIV/0!
RTL6	0	0	0.1666666667		0	#DIV/0!
RTN2	0.09523809524	0.09523809524	0.1111111111		0.6842105263	#DIV/0!
RTN4	0.04761904762	0.04761904762	0.05555555556		0.7	#DIV/0!
RTN4IP1	0	0	0			#DIV/0!
RTN4R	0.04761904762	0.04761904762	0.05555555556		0.7	#DIV/0!
RTN4RL	0	0	0.1111111111		0	#DIV/0!
RTP1	0.09523809524	0.09523809524	0.05555555556		1.473684211	#DIV/0!
RTP2	0.09523809524	0.09523809524	0.1111111111		0.6842105263	#DIV/0!
RTP4	0.09523809524	0.09523809524	0.05555555556		1.473684211	#DIV/0!
RTP5	0	0	0.05555555556		0	#DIV/0!
RTRAF	0.04761904762	0.04761904762	0		inf	#DIV/0!
RUBCN	0.1428571429	0.1428571429	0.05555555556		2.333333333	#DIV/0!
RUFY1	0.09523809524	0.09523809524	0.05555555556		1.473684211	#DIV/0!
RUFY2	0.1904761905	0.1904761905	0.05555555556		3.294117647	#DIV/0!
RUFY3	0.09523809524	0.09523809524	0.05555555556		1.473684211	#DIV/0!
RUFY4	0	0	0.1111111111		0	#DIV/0!
RUNDC1	0.04761904762	0.04761904762	0		inf	#DIV/0!
UNDC3	0.09523809524	0.09523809524	0		inf	#DIV/0!
NDC3A-	0.09523809524	0.09523809524	0		inf	#DIV/0!
UNDC3	0.09523809524	0.09523809524	0.05555555556		1.473684211	#DIV/0!
RUNX1	0.04761904762	0.04761904762	0.1666666667		0.2	#DIV/0!
UNX1-IT	0.04761904762	0.04761904762	0.1666666667		0.2	#DIV/0!
RUNX1T	0.1904761905	0.1904761905	0.1111111111		1.529411765	#DIV/0!
RUNX2	0.04761904762	0.04761904762	0.1111111111		0.325	#DIV/0!
RUNX3	0.04761904762	0.04761904762	0.1111111111		0.325	#DIV/0!
RUSC1	0.1904761905	0.1904761905	0.1111111111		1.529411765	#DIV/0!
USC1-AS	0.1904761905	0.1904761905	0.1111111111		1.529411765	#DIV/0!
RUSC2	0.09523809524	0.09523809524	0.05555555556		1.473684211	#DIV/0!
RUVBL1	0.09523809524	0.09523809524	0.05555555556		1.473684211	#DIV/0!
VBL1-A	0.09523809524	0.09523809524	0.05555555556		1.473684211	#DIV/0!
RUVBL2	0.09523809524	0.09523809524	0.1111111111		0.6842105263	#DIV/0!
RWDD2B	0.04761904762	0.04761904762	0.1111111111		0.325	#DIV/0!
RWDD3	0.04761904762	0.04761904762	0.05555555556		0.7	#DIV/0!
RWDD4	0	0	0.05555555556		0	#DIV/0!
RXFP1	0	0	0.05555555556		0	#DIV/0!
RXFP3	0.04761904762	0.04761904762	0.1111111111		0.325	#DIV/0!
RXFP4	0.1428571429	0.1428571429	0.1111111111		1.083333333	#DIV/0!
RXRA	0.04761904762	0.04761904762	0		inf	#DIV/0!
RXRB	0.04761904762	0.04761904762	0		inf	#DIV/0!
RXRG	0.1428571429	0.1428571429	0		inf	#DIV/0!
RXYLT1	0.1428571429	0.1428571429	0.05555555556		2.333333333	#DIV/0!
YLT1-A	0.1428571429	0.1428571429	0.05555555556		2.333333333	#DIV/0!
RYBP	0.04761904762	0.04761904762	0		inf	#DIV/0!
RYK	0.1428571429	0.1428571429	0.05555555556		2.333333333	#DIV/0!
RYR1	0.04761904762	0.04761904762	0.05555555556		0.7	#DIV/0!
RYR2	0.1428571429	0.1428571429	0		inf	#DIV/0!
S100A1	0.1904761905	0.1904761905	0.05555555556		3.294117647	#DIV/0!

**S100A10**	0.1904761905	0.1904761905	0		inf	#DIV/0!
**S100A11**	0.1904761905	0.1904761905	0		inf	#DIV/0!
**S100A13**	0.1904761905	0.1904761905	0.05555555556		3.294117647	#DIV/0!
**S100A14**	0.1904761905	0.1904761905	0.05555555556		3.294117647	#DIV/0!
**S100A16**	0.1904761905	0.1904761905	0.05555555556		3.294117647	#DIV/0!
**S100A2**	0.1904761905	0.1904761905	0.05555555556		3.294117647	#DIV/0!
**S100A3**	0.1904761905	0.1904761905	0.05555555556		3.294117647	#DIV/0!
**S100A4**	0.1904761905	0.1904761905	0.05555555556		3.294117647	#DIV/0!
**S100A5**	0.1904761905	0.1904761905	0.05555555556		3.294117647	#DIV/0!
**S100A6**	0.1904761905	0.1904761905	0		inf	#DIV/0!
**S100A7**	0.1904761905	0.1904761905	0		inf	#DIV/0!
**S100B**	0.04761904762	0.04761904762	0.1111111111		0.325	#DIV/0!
**S100PBP**	0.04761904762	0.04761904762	0.1111111111		0.325	#DIV/0!
**S100Z**	0.04761904762	0.04761904762	0		inf	#DIV/0!
**S1PR1**	0.04761904762	0.04761904762	0.05555555556		0.7	#DIV/0!
**S1PR2**	0.1428571429	0.1428571429	0.1111111111		1.083333333	#DIV/0!
**S1PR3**	0	0	0.05555555556		0	#DIV/0!
**S1PR4**	0.09523809524	0.09523809524	0.05555555556		1.473684211	#DIV/0!
**S1PR5**	0.1428571429	0.1428571429	0.1111111111		1.083333333	#DIV/0!
**SAE1**	0.09523809524	0.09523809524	0.1111111111		0.6842105263	#DIV/0!
**SAFB**	0.1428571429	0.1428571429	0.05555555556		2.333333333	#DIV/0!
**SAFB2**	0.1428571429	0.1428571429	0.05555555556		2.333333333	#DIV/0!
**SAG**	0	0	0.05555555556		0	#DIV/0!
**SALL1**	0.04761904762	0.04761904762	0		inf	#DIV/0!
**SALL3**	0.04761904762	0.04761904762	0.05555555556		0.7	#DIV/0!
**SALL4**	0.09523809524	0.09523809524	0.1111111111		0.6842105263	#DIV/0!
**ALRNA**	0.04761904762	0.04761904762	0.05555555556		0.7	#DIV/0!
**ALRNA**	0.04761904762	0.04761904762	0.05555555556		0.7	#DIV/0!
**SAMD1**	0.1428571429	0.1428571429	0.1111111111		1.083333333	#DIV/0!
**SAMD10**	0.1428571429	0.1428571429	0.2222222222		0.4583333333	#DIV/0!
**SAMD11**	0	0	0.1111111111		0	#DIV/0!
**SAMD12**	0.1428571429	0.1428571429	0.1666666667		0.6666666667	#DIV/0!
**MD12-A**	0.09523809524	0.09523809524	0.1666666667		0.4210526316	#DIV/0!
**SAMD13**	0.04761904762	0.04761904762	0.05555555556		0.7	#DIV/0!
**SAMD14**	0.09523809524	0.09523809524	0		inf	#DIV/0!
**SAMD4A**	0.04761904762	0.04761904762	0		inf	#DIV/0!
**SAMD4B**	0.04761904762	0.04761904762	0.05555555556		0.7	#DIV/0!
**SAMD7**	0.1904761905	0.1904761905	0.05555555556		3.294117647	#DIV/0!
**SAMD8**	0.1904761905	0.1904761905	0.05555555556		3.294117647	#DIV/0!
**SAMD9**	0.04761904762	0.04761904762	0.1111111111		0.325	#DIV/0!
**SAMD9L**	0.04761904762	0.04761904762	0.1111111111		0.325	#DIV/0!
**SAMHD1**	0.09523809524	0.09523809524	0.1111111111		0.6842105263	#DIV/0!
**SAMM50**	0	0	0.1666666667		0	#DIV/0!
**AMMSO**	0.04761904762	0.04761904762	0		inf	#DIV/0!
**SAMSN1**	0.04761904762	0.04761904762	0.1111111111		0.325	#DIV/0!
**MSN1-A**	0.04761904762	0.04761904762	0.1111111111		0.325	#DIV/0!
**SAP130**	0	0	0.05555555556		0	#DIV/0!
**SAP18**	0.04761904762	0.04761904762	0		inf	#DIV/0!
**SAP25**	0.04761904762	0.04761904762	0.05555555556		0.7	#DIV/0!
**SAP30**	0	0	0.05555555556		0	#DIV/0!
**SAP30BP**	0.04761904762	0.04761904762	0.05555555556		0.7	#DIV/0!

SAPCD1	0.04761904762	0.04761904762	0		inf	#DIV/0!
PCD1-A	0.04761904762	0.04761904762	0		inf	#DIV/0!
SAPCD2	0.1428571429	0.1428571429	0.05555555556		2.333333333	#DIV/0!
SAR1A	0.09523809524	0.09523809524	0		inf	#DIV/0!
SAR1B	0.04761904762	0.04761904762	0		inf	#DIV/0!
SARAF	0.04761904762	0.04761904762	0.1111111111		0.325	#DIV/0!
SARDH	0.04761904762	0.04761904762	0		inf	#DIV/0!
SARM1	0.04761904762	0.04761904762	0.05555555556		0.7	#DIV/0!
SARNP	0.04761904762	0.04761904762	0.05555555556		0.7	#DIV/0!
SARS	0.04761904762	0.04761904762	0.05555555556		0.7	#DIV/0!
SARS2	0.04761904762	0.04761904762	0.05555555556		0.7	#DIV/0!
SART3	0.04761904762	0.04761904762	0.05555555556		0.7	#DIV/0!
SASS6	0.04761904762	0.04761904762	0.05555555556		0.7	#DIV/0!
SAT2	0	0	0.05555555556		0	#DIV/0!
SATB1	0.04761904762	0.04761904762	0		inf	#DIV/0!
ATB1-AS	0.04761904762	0.04761904762	0		inf	#DIV/0!
SATB2	0	0	0.05555555556		0	#DIV/0!
ATB2-AS	0	0	0.05555555556		0	#DIV/0!
SAV1	0.04761904762	0.04761904762	0		inf	#DIV/0!
SAXO1	0.04761904762	0.04761904762	0.05555555556		0.7	#DIV/0!
SAXO2	0.04761904762	0.04761904762	0.05555555556		0.7	#DIV/0!
SAYSD1	0.04761904762	0.04761904762	0.05555555556		0.7	#DIV/0!
SBDS	0.04761904762	0.04761904762	0.1111111111		0.325	#DIV/0!
SBDSP1	0.1428571429	0.1428571429	0.1111111111		1.083333333	#DIV/0!
SBF1	0	0	0.1111111111		0	#DIV/0!
SBF1P1	0.1428571429	0.1428571429	0.1111111111		1.083333333	#DIV/0!
SBF2	0.04761904762	0.04761904762	0.05555555556		0.7	#DIV/0!
BF2-AS	0.04761904762	0.04761904762	0.05555555556		0.7	#DIV/0!
SBK1	0.04761904762	0.04761904762	0		inf	#DIV/0!
SBK2	0.09523809524	0.09523809524	0.05555555556		1.473684211	#DIV/0!
SBK3	0.09523809524	0.09523809524	0.05555555556		1.473684211	#DIV/0!
SBNO1	0.04761904762	0.04761904762	0.1111111111		0.325	#DIV/0!
SBNO2	0.09523809524	0.09523809524	0.1111111111		0.6842105263	#DIV/0!
SBSN	0.04761904762	0.04761904762	0.05555555556		0.7	#DIV/0!
SBSPON	0.1904761905	0.1904761905	0.1666666667		0.9411764706	#DIV/0!
CAANT	0.04761904762	0.04761904762	0		inf	#DIV/0!
SCAF1	0.09523809524	0.09523809524	0.1111111111		0.6842105263	#DIV/0!
SCAF11	0.04761904762	0.04761904762	0.05555555556		0.7	#DIV/0!
SCAF4	0.04761904762	0.04761904762	0.1111111111		0.325	#DIV/0!
SCAI	0.09523809524	0.09523809524	0.05555555556		1.473684211	#DIV/0!
SCAMP1	0.04761904762	0.04761904762	0		inf	#DIV/0!
AMP1-A	0.04761904762	0.04761904762	0		inf	#DIV/0!
SCAMP2	0.04761904762	0.04761904762	0.05555555556		0.7	#DIV/0!
SCAMP3	0.1904761905	0.1904761905	0.1111111111		1.529411765	#DIV/0!
SCAMP4	0.09523809524	0.09523809524	0.1111111111		0.6842105263	#DIV/0!
SCAMP5	0.04761904762	0.04761904762	0.05555555556		0.7	#DIV/0!
SCAND1	0.09523809524	0.09523809524	0.1111111111		0.6842105263	#DIV/0!
CAND2	0.04761904762	0.04761904762	0.05555555556		0.7	#DIV/0!
SCAP	0.09523809524	0.09523809524	0		inf	#DIV/0!
SCAPER	0.04761904762	0.04761904762	0.05555555556		0.7	#DIV/0!
SCARA3	0.04761904762	0.04761904762	0.1111111111		0.325	#DIV/0!

SCARA5	0.04761904762	0.04761904762	0.1111111111		0.325	#DIV/0!
SCARB1	0.04761904762	0.04761904762	0.05555555556		0.7	#DIV/0!
SCARB2	0.04761904762	0.04761904762	0.05555555556		0.7	#DIV/0!
SCARF1	0	0	0.1111111111		0	#DIV/0!
SCARF2	0.04761904762	0.04761904762	0.05555555556		0.7	#DIV/0!
CARNA	0.04761904762	0.04761904762	0.1111111111		0.325	#DIV/0!
CARNA1	0.04761904762	0.04761904762	0.1111111111		0.325	#DIV/0!
CARNA1	0.04761904762	0.04761904762	0.1111111111		0.325	#DIV/0!
CARNA1	0.04761904762	0.04761904762	0.1111111111		0.325	#DIV/0!
CARNA1	0.04761904762	0.04761904762	0.05555555556		0.7	#DIV/0!
CARNA1	0.04761904762	0.04761904762	0.05555555556		0.7	#DIV/0!
CARNA1	0.04761904762	0.04761904762	0		inf	#DIV/0!
CARNA1	0.04761904762	0.04761904762	0		inf	#DIV/0!
CARNA1	0.04761904762	0.04761904762	0.05555555556		0.7	#DIV/0!
CARNA	0.04761904762	0.04761904762	0.05555555556		0.7	#DIV/0!
CARNA2	0.1428571429	0.1428571429	0		inf	#DIV/0!
CARNA2	0	0	0.05555555556		0	#DIV/0!
ARNA2	0.04761904762	0.04761904762	0.1111111111		0.325	#DIV/0!
CARNA2	0.04761904762	0.04761904762	0		inf	#DIV/0!
ARNA2	0.1904761905	0.1904761905	0.1111111111		1.529411765	#DIV/0!
ARNA2	0.1904761905	0.1904761905	0.1111111111		1.529411765	#DIV/0!
CARNA2	0.04761904762	0.04761904762	0.05555555556		0.7	#DIV/0!
CARNA	0.1428571429	0.1428571429	0		inf	#DIV/0!
CARNA	0.1904761905	0.1904761905	0.1111111111		1.529411765	#DIV/0!
CARNA	0	0	0.05555555556		0	#DIV/0!
CARNA	0	0	0.05555555556		0	#DIV/0!
CARNA	0.1428571429	0.1428571429	0.05555555556		2.333333333	#DIV/0!
CARNA	0.04761904762	0.04761904762	0.05555555556		0.7	#DIV/0!
SCART1	0.1904761905	0.1904761905	0		inf	#DIV/0!
SCAT1	0.04761904762	0.04761904762	0		inf	#DIV/0!
SCAT2	0.04761904762	0.04761904762	0.05555555556		0.7	#DIV/0!
SCCPDH	0.1428571429	0.1428571429	0.05555555556		2.333333333	#DIV/0!
SCD	0.1428571429	0.1428571429	0		inf	#DIV/0!
SCD5	0	0	0.05555555556		0	#DIV/0!
SCFD2	0.2380952381	0.2380952381	0.05555555556		4.375	#DIV/0!
SCG2	0	0	0.05555555556		0	#DIV/0!
SCG3	0.04761904762	0.04761904762	0.05555555556		0.7	#DIV/0!
CGB1B2	0.04761904762	0.04761904762	0.05555555556		0.7	#DIV/0!
CGB1C	0.04761904762	0.04761904762	0		inf	#DIV/0!
CGB1C	0.04761904762	0.04761904762	0		inf	#DIV/0!
SCGB2B	0.04761904762	0.04761904762	0.05555555556		0.7	#DIV/0!
CGB2B3	0.04761904762	0.04761904762	0.05555555556		0.7	#DIV/0!
CGB3A	0.09523809524	0.09523809524	0.05555555556		1.473684211	#DIV/0!
SCGN	0	0	0.05555555556		0	#DIV/0!
SCHIP1	0.1428571429	0.1428571429	0.05555555556		2.333333333	#DIV/0!
CHLAP	0	0	0.05555555556		0	#DIV/0!
SCIMP	0	0	0.1111111111		0	#DIV/0!
SCIN	0.04761904762	0.04761904762	0.2222222222		0.1375	#DIV/0!
SCLT1	0	0	0.05555555556		0	#DIV/0!
SCLY	0	0	0.05555555556		0	#DIV/0!
SCMH1	0.04761904762	0.04761904762	0.05555555556		0.7	#DIV/0!

SCML4	0	0	0			#DIV/0!
SCN10A	0.04761904762	0.04761904762	0		inf	#DIV/0!
SCN11A	0.04761904762	0.04761904762	0		inf	#DIV/0!
SCN1A	0	0	0.05555555556		0	#DIV/0!
CN1A-AS	0	0	0.05555555556		0	#DIV/0!
SCN1B	0.04761904762	0.04761904762	0.05555555556		0.7	#DIV/0!
SCN2A	0	0	0.05555555556		0	#DIV/0!
SCN3A	0	0	0.05555555556		0	#DIV/0!
SCN4A	0.04761904762	0.04761904762	0		inf	#DIV/0!
SCN5A	0.04761904762	0.04761904762	0		inf	#DIV/0!
SCN7A	0	0	0.05555555556		0	#DIV/0!
SCN8A	0.04761904762	0.04761904762	0.05555555556		0.7	#DIV/0!
SCN9A	0	0	0.05555555556		0	#DIV/0!
SCNM1	0.1904761905	0.1904761905	0.05555555556		3.294117647	#DIV/0!
SCNN1A	0.04761904762	0.04761904762	0.1111111111		0.325	#DIV/0!
SCNN1B	0.04761904762	0.04761904762	0		inf	#DIV/0!
SCNN1D	0	0	0.1111111111		0	#DIV/0!
SCNN1G	0.04761904762	0.04761904762	0		inf	#DIV/0!
SCO1	0.04761904762	0.04761904762	0.05555555556		0.7	#DIV/0!
SCO2	0	0	0.1111111111		0	#DIV/0!
SCOC	0	0	0.05555555556		0	#DIV/0!
COC-AS	0	0	0.05555555556		0	#DIV/0!
SCP2	0.04761904762	0.04761904762	0.05555555556		0.7	#DIV/0!
SCP2D1	0.1904761905	0.1904761905	0.05555555556		3.294117647	#DIV/0!
SCPEP1	0.09523809524	0.09523809524	0		inf	#DIV/0!
SCRG1	0	0	0.05555555556		0	#DIV/0!
SCRIB	0.04761904762	0.04761904762	0.1666666667		0.2	#DIV/0!
SCRN1	0.04761904762	0.04761904762	0.1666666667		0.2	#DIV/0!
SCRN2	0.1428571429	0.1428571429	0		inf	#DIV/0!
SCRN3	0	0	0.1111111111		0	#DIV/0!
SCRT1	0.04761904762	0.04761904762	0.1666666667		0.2	#DIV/0!
SCRT2	0.1904761905	0.1904761905	0.05555555556		3.294117647	#DIV/0!
SCT	0.04761904762	0.04761904762	0		inf	#DIV/0!
SCTR	0	0	0.05555555556		0	#DIV/0!
SCUBE1	0	0	0.1666666667		0	#DIV/0!
SCUBE3	0.04761904762	0.04761904762	0		inf	#DIV/0!
SCX	0.04761904762	0.04761904762	0.1666666667		0.2	#DIV/0!
SCYL2	0.04761904762	0.04761904762	0.05555555556		0.7	#DIV/0!
SCYL3	0.1428571429	0.1428571429	0.05555555556		2.333333333	#DIV/0!
SDAD1	0.04761904762	0.04761904762	0.05555555556		0.7	#DIV/0!
SDAD1P	0.04761904762	0.04761904762	0.1111111111		0.325	#DIV/0!
SDC1	0	0	0.05555555556		0	#DIV/0!
SDC2	0.1904761905	0.1904761905	0.1666666667		0.9411764706	#DIV/0!
SDC3	0.04761904762	0.04761904762	0.1111111111		0.325	#DIV/0!
SDC4	0.1428571429	0.1428571429	0.1666666667		0.6666666667	#DIV/0!
SDC4P	0.04761904762	0.04761904762	0.1111111111		0.325	#DIV/0!
SDCBP	0.1428571429	0.1428571429	0.1666666667		0.6666666667	#DIV/0!
SDCBP2	0.1904761905	0.1904761905	0.1111111111		1.529411765	#DIV/0!
CBP2-A	0.1904761905	0.1904761905	0.1111111111		1.529411765	#DIV/0!
DCCAG	0.1428571429	0.1428571429	0.05555555556		2.333333333	#DIV/0!
SDE2	0.1428571429	0.1428571429	0.05555555556		2.333333333	#DIV/0!

**SDF2**	0.04761904762	0.04761904762	0.05555555556		0.7	#DIV/0!
**SDF2L1**	0.04761904762	0.04761904762	0.05555555556		0.7	#DIV/0!
**SDF4**	0	0	0.1111111111		0	#DIV/0!
**SDHA**	0	0	0.05555555556		0	#DIV/0!
**SDHAF1**	0.04761904762	0.04761904762	0.05555555556		0.7	#DIV/0!
**SDHAF3**	0.04761904762	0.04761904762	0.1111111111		0.325	#DIV/0!
**SDHAP1**	0.1428571429	0.1428571429	0.05555555556		2.333333333	#DIV/0!
**SDHAP2**	0.1428571429	0.1428571429	0.05555555556		2.333333333	#DIV/0!
**SDHAP3**	0	0	0.1111111111		0	#DIV/0!
**SDHB**	0.04761904762	0.04761904762	0.1111111111		0.325	#DIV/0!
**SDHC**	0.1428571429	0.1428571429	0		inf	#DIV/0!
**SDK1**	0.09523809524	0.09523809524	0.1666666667		0.4210526316	#DIV/0!
**SDK2**	0.04761904762	0.04761904762	0		inf	#DIV/0!
**SDR16C5**	0.1428571429	0.1428571429	0.1111111111		1.083333333	#DIV/0!
**DR16C6**	0.1428571429	0.1428571429	0.1111111111		1.083333333	#DIV/0!
**SDR42E1**	0.04761904762	0.04761904762	0		inf	#DIV/0!
**SDR42E2**	0.04761904762	0.04761904762	0		inf	#DIV/0!
**SDR9C7**	0.1428571429	0.1428571429	0.05555555556		2.333333333	#DIV/0!
**SDS**	0	0	0.05555555556		0	#DIV/0!
**SDSL**	0	0	0.05555555556		0	#DIV/0!
**SEBOX**	0.04761904762	0.04761904762	0.05555555556		0.7	#DIV/0!
**SEC11A**	0.04761904762	0.04761904762	0.05555555556		0.7	#DIV/0!
**SEC11C**	0.09523809524	0.09523809524	0		inf	#DIV/0!
**SEC13**	0.09523809524	0.09523809524	0		inf	#DIV/0!
**SEC14L1**	0.04761904762	0.04761904762	0		inf	#DIV/0!
**SEC14L2**	0.04761904762	0.04761904762	0.1111111111		0.325	#DIV/0!
**SEC14L3**	0.04761904762	0.04761904762	0.1111111111		0.325	#DIV/0!
**SEC14L4**	0.04761904762	0.04761904762	0.1111111111		0.325	#DIV/0!
**SEC14L5**	0.04761904762	0.04761904762	0		inf	#DIV/0!
**SEC14L6**	0.04761904762	0.04761904762	0.1111111111		0.325	#DIV/0!
**SEC16A**	0.1428571429	0.1428571429	0.05555555556		2.333333333	#DIV/0!
**SEC16B**	0.1428571429	0.1428571429	0		inf	#DIV/0!
**SEC1P**	0.09523809524	0.09523809524	0.1111111111		0.6842105263	#DIV/0!
**SEC22A**	0.04761904762	0.04761904762	0.05555555556		0.7	#DIV/0!
**SEC22B**	0.09523809524	0.09523809524	0.05555555556		1.473684211	#DIV/0!
**SEC22B2**	0.1428571429	0.1428571429	0.05555555556		2.333333333	#DIV/0!
**SEC22B3**	0.1428571429	0.1428571429	0.05555555556		2.333333333	#DIV/0!
**SEC22C**	0.04761904762	0.04761904762	0		inf	#DIV/0!
**SEC23A**	0.04761904762	0.04761904762	0.05555555556		0.7	#DIV/0!
**C23A-A**	0.04761904762	0.04761904762	0.05555555556		0.7	#DIV/0!
**SEC23B**	0.1904761905	0.1904761905	0.05555555556		3.294117647	#DIV/0!
**SEC23IP**	0.2380952381	0.2380952381	0		inf	#DIV/0!
**SEC24A**	0.04761904762	0.04761904762	0		inf	#DIV/0!
**SEC24B**	0	0	0.05555555556		0	#DIV/0!
**C24B-A**	0	0	0.05555555556		0	#DIV/0!
**SEC24D**	0	0	0.05555555556		0	#DIV/0!
**SEC31A**	0	0	0.05555555556		0	#DIV/0!
**SEC31B**	0.1428571429	0.1428571429	0		inf	#DIV/0!
**SEC61A1**	0.09523809524	0.09523809524	0.05555555556		1.473684211	#DIV/0!
**SEC61A2**	0.09523809524	0.09523809524	0.1111111111		0.6842105263	#DIV/0!
**SEC61B**	0.04761904762	0.04761904762	0		inf	#DIV/0!

SEC61G	0.380952381	0.380952381	0.1666666667		2.461538462	#DIV/0!
SEC62	0.1904761905	0.1904761905	0.05555555556		3.294117647	#DIV/0!
SEC63	0	0	0			#DIV/0!
ECISBP	0.04761904762	0.04761904762	0.05555555556		0.7	#DIV/0!
ECISBP2	0.04761904762	0.04761904762	0.05555555556		0.7	#DIV/0!
SECTM1	0.04761904762	0.04761904762	0		inf	#DIV/0!
SEH1L	0.09523809524	0.09523809524	0.05555555556		1.473684211	#DIV/0!
SEL1L2	0.1904761905	0.1904761905	0.1111111111		1.529411765	#DIV/0!
SELE	0.1428571429	0.1428571429	0		inf	#DIV/0!
ELENBI	0.1904761905	0.1904761905	0.05555555556		3.294117647	#DIV/0!
ELENO	0.04761904762	0.04761904762	0.05555555556		0.7	#DIV/0!
ELENOI	0.04761904762	0.04761904762	0		inf	#DIV/0!
ELENOI	0.04761904762	0.04761904762	0.1111111111		0.325	#DIV/0!
ELENOI	0.04761904762	0.04761904762	0.1111111111		0.325	#DIV/0!
ELENO(	0	0	0.1111111111		0	#DIV/0!
ELENO	0.04761904762	0.04761904762	0.05555555556		0.7	#DIV/0!
ELENO	0.04761904762	0.04761904762	0.05555555556		0.7	#DIV/0!
ELENOT	0.1904761905	0.1904761905	0.1111111111		1.529411765	#DIV/0!
ELENOT	0.04761904762	0.04761904762	0.05555555556		0.7	#DIV/0!
ELENOV	0.09523809524	0.09523809524	0.1111111111		0.6842105263	#DIV/0!
SELL	0.1428571429	0.1428571429	0		inf	#DIV/0!
SELP	0.1428571429	0.1428571429	0		inf	#DIV/0!
SELPLG	0.04761904762	0.04761904762	0.05555555556		0.7	#DIV/0!
SEM1	0.04761904762	0.04761904762	0.1111111111		0.325	#DIV/0!
SEMA3A	0.04761904762	0.04761904762	0.1111111111		0.325	#DIV/0!
SEMA3B	0.09523809524	0.09523809524	0		inf	#DIV/0!
MA3B-A	0.09523809524	0.09523809524	0		inf	#DIV/0!
SEMA3C	0.04761904762	0.04761904762	0.1111111111		0.325	#DIV/0!
SEMA3D	0.04761904762	0.04761904762	0.1111111111		0.325	#DIV/0!
SEMA3E	0.04761904762	0.04761904762	0.1111111111		0.325	#DIV/0!
SEMA3F	0.09523809524	0.09523809524	0		inf	#DIV/0!
MA3F-A	0.09523809524	0.09523809524	0		inf	#DIV/0!
SEMA3G	0.04761904762	0.04761904762	0		inf	#DIV/0!
SEMA4B	0.04761904762	0.04761904762	0.05555555556		0.7	#DIV/0!
SEMA4C	0	0	0.1111111111		0	#DIV/0!
SEMA4D	0.04761904762	0.04761904762	0.05555555556		0.7	#DIV/0!
SEMA4F	0	0	0.05555555556		0	#DIV/0!
SEMA4G	0.1428571429	0.1428571429	0		inf	#DIV/0!
SEMA5A	0	0	0.05555555556		0	#DIV/0!
MA5A-A	0	0	0.05555555556		0	#DIV/0!
SEMA5B	0.04761904762	0.04761904762	0.05555555556		0.7	#DIV/0!
SEMA6A	0.04761904762	0.04761904762	0		inf	#DIV/0!
MA6A-A	0.04761904762	0.04761904762	0		inf	#DIV/0!
MA6A-A	0.04761904762	0.04761904762	0		inf	#DIV/0!
SEMA6B	0.09523809524	0.09523809524	0.05555555556		1.473684211	#DIV/0!
SEMA6C	0.1904761905	0.1904761905	0.05555555556		3.294117647	#DIV/0!
SEMA6D	0.04761904762	0.04761904762	0.05555555556		0.7	#DIV/0!
SEMA7A	0.04761904762	0.04761904762	0.05555555556		0.7	#DIV/0!
SEMG1	0.1428571429	0.1428571429	0.1666666667		0.6666666667	#DIV/0!
SEMG2	0.1428571429	0.1428571429	0.1666666667		0.6666666667	#DIV/0!
SENP1	0.04761904762	0.04761904762	0.05555555556		0.7	#DIV/0!

**SENP2**	0.1428571429	0.1428571429	0.05555555556		2.333333333	#DIV/0!
**SENP3**	0	0	0.05555555556		0	#DIV/0!
**NP3-EIF**	0	0	0.05555555556		0	#DIV/0!
**SENP5**	0.1428571429	0.1428571429	0.05555555556		2.333333333	#DIV/0!
**SENP7**	0.09523809524	0.09523809524	0.05555555556		1.473684211	#DIV/0!
**SENP8**	0.04761904762	0.04761904762	0.05555555556		0.7	#DIV/0!
**SEPHS1**	0.09523809524	0.09523809524	0.1111111111		0.6842105263	#DIV/0!
**SEPHS2**	0.04761904762	0.04761904762	0		inf	#DIV/0!
**SEPT1**	0.04761904762	0.04761904762	0		inf	#DIV/0!
**SEPT10**	0	0	0.05555555556		0	#DIV/0!
**SEPT11**	0.04761904762	0.04761904762	0.05555555556		0.7	#DIV/0!
**SEPT12**	0.04761904762	0.04761904762	0		inf	#DIV/0!
**SEPT14**	0.3333333333	0.3333333333	0.2222222222		1.375	#DIV/0!
**SEPT2**	0	0	0.05555555556		0	#DIV/0!
**SEPT3**	0	0	0.1111111111		0	#DIV/0!
**SEPT4**	0.1428571429	0.1428571429	0		inf	#DIV/0!
**EPT4-AS**	0.1428571429	0.1428571429	0		inf	#DIV/0!
**SEPT5**	0.04761904762	0.04761904762	0.05555555556		0.7	#DIV/0!
**PT5-GP1**	0.04761904762	0.04761904762	0.05555555556		0.7	#DIV/0!
**SEPT7**	0.04761904762	0.04761904762	0.2222222222		0.1375	#DIV/0!
**EPT7-AS**	0.04761904762	0.04761904762	0.2222222222		0.1375	#DIV/0!
**SEPT7P2**	0.04761904762	0.04761904762	0.2222222222		0.1375	#DIV/0!
**SEPT7P9**	0.09523809524	0.09523809524	0.1111111111		0.6842105263	#DIV/0!
**SEPT8**	0.04761904762	0.04761904762	0		inf	#DIV/0!
**SEPT9**	0.04761904762	0.04761904762	0		inf	#DIV/0!
**SERBP1**	0.04761904762	0.04761904762	0.05555555556		0.7	#DIV/0!
**SERF1A**	0.09523809524	0.09523809524	0.1666666667		0.4210526316	#DIV/0!
**SERF1B**	0.09523809524	0.09523809524	0.1666666667		0.4210526316	#DIV/0!
**SERHL**	0	0	0.1666666667		0	#DIV/0!
**SERHL2**	0	0	0.1666666667		0	#DIV/0!
**SERINC2**	0.04761904762	0.04761904762	0.1111111111		0.325	#DIV/0!
**SERINC3**	0.1428571429	0.1428571429	0.1666666667		0.6666666667	#DIV/0!
**SERINC5**	0.04761904762	0.04761904762	0		inf	#DIV/0!
**SERP1**	0.1904761905	0.1904761905	0.1111111111		1.529411765	#DIV/0!
**ERPINB**	0.04761904762	0.04761904762	0		inf	#DIV/0!
**ERPINB**	0.04761904762	0.04761904762	0		inf	#DIV/0!
**ERPINB**	0.04761904762	0.04761904762	0		inf	#DIV/0!
**ERPINB**	0.04761904762	0.04761904762	0		inf	#DIV/0!
**ERPINB**	0.04761904762	0.04761904762	0		inf	#DIV/0!
**ERPINB**	0.04761904762	0.04761904762	0		inf	#DIV/0!
**ERPINB**	0.04761904762	0.04761904762	0		inf	#DIV/0!
**ERPINB**	0.04761904762	0.04761904762	0		inf	#DIV/0!
**ERPINB**	0.04761904762	0.04761904762	0		inf	#DIV/0!
**ERPINB**	0.04761904762	0.04761904762	0		inf	#DIV/0!
**ERPINC**	0.1428571429	0.1428571429	0		inf	#DIV/0!
**ERPIND**	0.04761904762	0.04761904762	0.05555555556		0.7	#DIV/0!
**ERPINE**	0.04761904762	0.04761904762	0.05555555556		0.7	#DIV/0!
**ERPINE**	0	0	0.05555555556		0	#DIV/0!
**ERPINF**	0	0	0.1111111111		0	#DIV/0!
**ERPINF**	0	0	0.1111111111		0	#DIV/0!
**ERPINI**	0.1428571429	0.1428571429	0.05555555556		2.333333333	#DIV/0!

ERPINI	0.1428571429	0.1428571429	0.05555555556		2.333333333	#DIV/0!
ERTAD	0.04761904762	0.04761904762	0.05555555556		0.7	#DIV/0!
ERTAD	0	0	0.05555555556		0	#DIV/0!
ERTAD	0.04761904762	0.04761904762	0.05555555556		0.7	#DIV/0!
ERTAD	0.1428571429	0.1428571429	0		inf	#DIV/0!
RTAD4-A	0.1428571429	0.1428571429	0		inf	#DIV/0!
SESN1	0	0	0			#DIV/0!
SESN2	0.04761904762	0.04761904762	0.1111111111		0.325	#DIV/0!
SESTD1	0	0	0.05555555556		0	#DIV/0!
SET	0.09523809524	0.09523809524	0.05555555556		1.473684211	#DIV/0!
SETBP1	0.04761904762	0.04761904762	0		inf	#DIV/0!
SETD1A	0.04761904762	0.04761904762	0		inf	#DIV/0!
SETD1B	0.04761904762	0.04761904762	0.1111111111		0.325	#DIV/0!
SETD2	0.09523809524	0.09523809524	0		inf	#DIV/0!
SETD4	0.04761904762	0.04761904762	0.1666666667		0.2	#DIV/0!
SETD5	0.09523809524	0.09523809524	0		inf	#DIV/0!
SETD6	0.04761904762	0.04761904762	0		inf	#DIV/0!
SETD7	0	0	0.05555555556		0	#DIV/0!
SETD9	0.04761904762	0.04761904762	0.05555555556		0.7	#DIV/0!
SETDB1	0.1904761905	0.1904761905	0.05555555556		3.294117647	#DIV/0!
SETMAR	0.04761904762	0.04761904762	0		inf	#DIV/0!
SETSIP	0.04761904762	0.04761904762	0.05555555556		0.7	#DIV/0!
SETX	0.09523809524	0.09523809524	0		inf	#DIV/0!
SEZ6	0.04761904762	0.04761904762	0.05555555556		0.7	#DIV/0!
SEZ6L	0.04761904762	0.04761904762	0.05555555556		0.7	#DIV/0!
SEZ6L2	0.04761904762	0.04761904762	0		inf	#DIV/0!
SF3A1	0.04761904762	0.04761904762	0.1111111111		0.325	#DIV/0!
SF3A2	0.09523809524	0.09523809524	0.1111111111		0.6842105263	#DIV/0!
SF3A3	0.04761904762	0.04761904762	0.05555555556		0.7	#DIV/0!
SF3B1	0	0	0.05555555556		0	#DIV/0!
SF3B3	0.1428571429	0.1428571429	0.05555555556		2.333333333	#DIV/0!
SF3B4	0.1428571429	0.1428571429	0.05555555556		2.333333333	#DIV/0!
SFI1	0.04761904762	0.04761904762	0.1111111111		0.325	#DIV/0!
SFMBT1	0.04761904762	0.04761904762	0		inf	#DIV/0!
SFMBT2	0.09523809524	0.09523809524	0.1111111111		0.6842105263	#DIV/0!
SFN	0.04761904762	0.04761904762	0.1111111111		0.325	#DIV/0!
SFPQ	0.04761904762	0.04761904762	0.05555555556		0.7	#DIV/0!
SFR1	0.1428571429	0.1428571429	0.05555555556		2.333333333	#DIV/0!
SFRP1	0.09523809524	0.09523809524	0.1666666667		0.4210526316	#DIV/0!
SFRP2	0	0	0.05555555556		0	#DIV/0!
SFRP4	0.04761904762	0.04761904762	0.2222222222		0.1375	#DIV/0!
SFRP5	0.1428571429	0.1428571429	0		inf	#DIV/0!
SFSWAP	0.09523809524	0.09523809524	0.05555555556		1.473684211	#DIV/0!
SFT2D2	0.1428571429	0.1428571429	0		inf	#DIV/0!
SFT2D3	0	0	0.05555555556		0	#DIV/0!
SFTA1P	0.09523809524	0.09523809524	0.1111111111		0.6842105263	#DIV/0!
SFTPA1	0.1428571429	0.1428571429	0.05555555556		2.333333333	#DIV/0!
SFTPA2	0.1428571429	0.1428571429	0.05555555556		2.333333333	#DIV/0!
SFTPB	0	0	0.05555555556		0	#DIV/0!
SFTPC	0.09523809524	0.09523809524	0.1111111111		0.6842105263	#DIV/0!
SFTPD	0.1428571429	0.1428571429	0		inf	#DIV/0!

SFXN1	0	0	0.05555555556		0	#DIV/0!
SFXN2	0.1428571429	0.1428571429	0.05555555556		2.333333333	#DIV/0!
SFXN3	0.1428571429	0.1428571429	0		inf	#DIV/0!
SFXN4	0.2380952381	0.2380952381	0		inf	#DIV/0!
SFXN5	0	0	0.05555555556		0	#DIV/0!
SGCA	0.09523809524	0.09523809524	0		inf	#DIV/0!
SGCB	0.2380952381	0.2380952381	0.05555555556		4.375	#DIV/0!
SGCD	0.04761904762	0.04761904762	0.05555555556		0.7	#DIV/0!
SGCE	0.04761904762	0.04761904762	0.1111111111		0.325	#DIV/0!
SGCZ	0.09523809524	0.09523809524	0.1111111111		0.6842105263	#DIV/0!
SGF29	0.04761904762	0.04761904762	0		inf	#DIV/0!
SGIP1	0.04761904762	0.04761904762	0.05555555556		0.7	#DIV/0!
SGK2	0.1428571429	0.1428571429	0.1111111111		1.083333333	#DIV/0!
SGK3	0.1428571429	0.1428571429	0.1666666667		0.6666666667	#DIV/0!
SGMS1	0.04761904762	0.04761904762	0.05555555556		0.7	#DIV/0!
GMS1-AS	0.04761904762	0.04761904762	0.05555555556		0.7	#DIV/0!
SGMS2	0	0	0.05555555556		0	#DIV/0!
SGO1	0.04761904762	0.04761904762	0		inf	#DIV/0!
GO1-AS	0.04761904762	0.04761904762	0		inf	#DIV/0!
SGO2	0	0	0.05555555556		0	#DIV/0!
SGPL1	0.09523809524	0.09523809524	0		inf	#DIV/0!
SGPP1	0.09523809524	0.09523809524	0		inf	#DIV/0!
SGPP2	0	0	0.05555555556		0	#DIV/0!
SGSH	0.04761904762	0.04761904762	0		inf	#DIV/0!
SGSM1	0.04761904762	0.04761904762	0.05555555556		0.7	#DIV/0!
SGSM2	0	0	0.1111111111		0	#DIV/0!
SGSM3	0	0	0.1111111111		0	#DIV/0!
SGTA	0.09523809524	0.09523809524	0.05555555556		1.473684211	#DIV/0!
SGTB	0.04761904762	0.04761904762	0		inf	#DIV/0!
SH2B1	0.04761904762	0.04761904762	0		inf	#DIV/0!
SH2B2	0.04761904762	0.04761904762	0.05555555556		0.7	#DIV/0!
SH2B3	0	0	0.1111111111		0	#DIV/0!
SH2D1B	0.1428571429	0.1428571429	0		inf	#DIV/0!
SH2D2A	0.1428571429	0.1428571429	0.05555555556		2.333333333	#DIV/0!
SH2D3A	0.1428571429	0.1428571429	0.05555555556		2.333333333	#DIV/0!
SH2D3C	0.09523809524	0.09523809524	0.05555555556		1.473684211	#DIV/0!
SH2D4A	0.09523809524	0.09523809524	0.1111111111		0.6842105263	#DIV/0!
SH2D4B	0.1428571429	0.1428571429	0		inf	#DIV/0!
SH2D5	0.04761904762	0.04761904762	0.1111111111		0.325	#DIV/0!
SH2D6	0	0	0.05555555556		0	#DIV/0!
SH2D7	0.04761904762	0.04761904762	0.05555555556		0.7	#DIV/0!
SH3BGR	0.04761904762	0.04761904762	0.05555555556		0.7	#DIV/0!
H3BGRL	0.04761904762	0.04761904762	0.1111111111		0.325	#DIV/0!
SH3BP1	0	0	0.1111111111		0	#DIV/0!
SH3BP2	0.04761904762	0.04761904762	0		inf	#DIV/0!
SH3BP4	0	0	0.05555555556		0	#DIV/0!
SH3BP5	0.04761904762	0.04761904762	0		inf	#DIV/0!
I3BP5-A	0.04761904762	0.04761904762	0		inf	#DIV/0!
H3BP5L	0.1428571429	0.1428571429	0		inf	#DIV/0!
SH3D19	0	0	0.05555555556		0	#DIV/0!
SH3D21	0.04761904762	0.04761904762	0.05555555556		0.7	#DIV/0!

H3PXD2	0.1428571429	0.1428571429	0.1111111111		1.083333333	#DIV/0!
PXD2A-	0.1428571429	0.1428571429	0.1111111111		1.083333333	#DIV/0!
H3PXD2	0	0	0.05555555556		0	#DIV/0!
SH3RF1	0	0	0.05555555556		0	#DIV/0!
SH3RF3	0	0	0.05555555556		0	#DIV/0!
I3RF3-A	0	0	0.05555555556		0	#DIV/0!
SH3YL1	0	0	0.05555555556		0	#DIV/0!
SHANK1	0.09523809524	0.09523809524	0.05555555556		1.473684211	#DIV/0!
SHANK3	0	0	0.1111111111		0	#DIV/0!
HARPI	0.04761904762	0.04761904762	0.1666666667		0.2	#DIV/0!
SHB	0.04761904762	0.04761904762	0.05555555556		0.7	#DIV/0!
SHBG	0	0	0.05555555556		0	#DIV/0!
SHC1	0.1904761905	0.1904761905	0.1111111111		1.529411765	#DIV/0!
SHC2	0.09523809524	0.09523809524	0.1111111111		0.6842105263	#DIV/0!
SHC3	0	0	0.05555555556		0	#DIV/0!
SHC4	0.04761904762	0.04761904762	0.05555555556		0.7	#DIV/0!
SHCBP1	0.04761904762	0.04761904762	0		inf	#DIV/0!
HCBP1I	0.1428571429	0.1428571429	0		inf	#DIV/0!
SHD	0.09523809524	0.09523809524	0.05555555556		1.473684211	#DIV/0!
SHE	0.1904761905	0.1904761905	0.05555555556		3.294117647	#DIV/0!
SHF	0.04761904762	0.04761904762	0		inf	#DIV/0!
SHH	0.1428571429	0.1428571429	0.05555555556		2.333333333	#DIV/0!
SHISA4	0.1428571429	0.1428571429	0		inf	#DIV/0!
SHISA5	0.09523809524	0.09523809524	0		inf	#DIV/0!
SHISA6	0.04761904762	0.04761904762	0.05555555556		0.7	#DIV/0!
SHISA7	0.09523809524	0.09523809524	0.05555555556		1.473684211	#DIV/0!
SHISA8	0	0	0.1111111111		0	#DIV/0!
SHISA9	0.04761904762	0.04761904762	0		inf	#DIV/0!
SHISAL1	0	0	0.1666666667		0	#DIV/0!
HISAL2	0.04761904762	0.04761904762	0.05555555556		0.7	#DIV/0!
HISAL2	0.04761904762	0.04761904762	0		inf	#DIV/0!
SHKBP1	0.04761904762	0.04761904762	0.05555555556		0.7	#DIV/0!
SHLD1	0.1904761905	0.1904761905	0.05555555556		3.294117647	#DIV/0!
SHLD2	0.1428571429	0.1428571429	0.05555555556		2.333333333	#DIV/0!
HLD2P	0.1428571429	0.1428571429	0.05555555556		2.333333333	#DIV/0!
HLD2P	0.1428571429	0.1428571429	0.05555555556		2.333333333	#DIV/0!
SHLD3	0.04761904762	0.04761904762	0		inf	#DIV/0!
SHMT1	0.04761904762	0.04761904762	0.1111111111		0.325	#DIV/0!
SHMT2	0.1428571429	0.1428571429	0.05555555556		2.333333333	#DIV/0!
SHOC1	0.04761904762	0.04761904762	0.05555555556		0.7	#DIV/0!
SHOC2	0.1428571429	0.1428571429	0.05555555556		2.333333333	#DIV/0!
SHOX2	0.1428571429	0.1428571429	0.05555555556		2.333333333	#DIV/0!
SHPK	0	0	0.1111111111		0	#DIV/0!
SHQ1	0.04761904762	0.04761904762	0		inf	#DIV/0!
HROOM	0.04761904762	0.04761904762	0		inf	#DIV/0!
HROOM	0.04761904762	0.04761904762	0.05555555556		0.7	#DIV/0!
SHTN1	0.2380952381	0.2380952381	0		inf	#DIV/0!
SI	0.1428571429	0.1428571429	0.05555555556		2.333333333	#DIV/0!
SIAH1	0.04761904762	0.04761904762	0		inf	#DIV/0!
SIAH2	0.1904761905	0.1904761905	0.1111111111		1.529411765	#DIV/0!
SIDT1	0.09523809524	0.09523809524	0.05555555556		1.473684211	#DIV/0!

SIGIRR	0.04761904762	0.04761904762	0		inf	#DIV/0!
IGMAR	0.09523809524	0.09523809524	0.05555555556		1.473684211	#DIV/0!
SIK1	0.04761904762	0.04761904762	0.1666666667		0.2	#DIV/0!
SIK1B	0.04761904762	0.04761904762	0.1666666667		0.2	#DIV/0!
SIKE1	0.04761904762	0.04761904762	0.05555555556		0.7	#DIV/0!
SIL1	0.04761904762	0.04761904762	0		inf	#DIV/0!
SILC1	0	0	0.05555555556		0	#DIV/0!
SIM2	0.04761904762	0.04761904762	0.1666666667		0.2	#DIV/0!
SIMC1	0.09523809524	0.09523809524	0.05555555556		1.473684211	#DIV/0!
SIN3A	0.04761904762	0.04761904762	0.05555555556		0.7	#DIV/0!
SIN3B	0.1428571429	0.1428571429	0.1111111111		1.083333333	#DIV/0!
INHCA	0.1428571429	0.1428571429	0.1111111111		1.083333333	#DIV/0!
SIPA1L2	0.1428571429	0.1428571429	0		inf	#DIV/0!
SIPA1L3	0.04761904762	0.04761904762	0.05555555556		0.7	#DIV/0!
SIRLNT	0.09523809524	0.09523809524	0.1111111111		0.6842105263	#DIV/0!
SIRPA	0.1904761905	0.1904761905	0.1111111111		1.529411765	#DIV/0!
SIRPB1	0.1904761905	0.1904761905	0.1111111111		1.529411765	#DIV/0!
SIRPB2	0.1904761905	0.1904761905	0.1111111111		1.529411765	#DIV/0!
SIRPD	0.1904761905	0.1904761905	0.1111111111		1.529411765	#DIV/0!
SIRPG	0.1904761905	0.1904761905	0.1111111111		1.529411765	#DIV/0!
RPG-AS	0.1904761905	0.1904761905	0.1111111111		1.529411765	#DIV/0!
SIRT1	0.1904761905	0.1904761905	0.05555555556		3.294117647	#DIV/0!
SIRT2	0.04761904762	0.04761904762	0.05555555556		0.7	#DIV/0!
SIRT3	0.04761904762	0.04761904762	0		inf	#DIV/0!
SIRT4	0	0	0.1111111111		0	#DIV/0!
SIRT6	0.09523809524	0.09523809524	0.05555555556		1.473684211	#DIV/0!
SIRT7	0.04761904762	0.04761904762	0		inf	#DIV/0!
SIT1	0.09523809524	0.09523809524	0.05555555556		1.473684211	#DIV/0!
SIX2	0	0	0.05555555556		0	#DIV/0!
SIX3	0	0	0.05555555556		0	#DIV/0!
IX3-AS	0	0	0.05555555556		0	#DIV/0!
SIX5	0.09523809524	0.09523809524	0.1111111111		0.6842105263	#DIV/0!
SKA1	0.04761904762	0.04761904762	0		inf	#DIV/0!
SKA2	0.1428571429	0.1428571429	0		inf	#DIV/0!
SKA3	0.04761904762	0.04761904762	0		inf	#DIV/0!
SKAP1	0.1428571429	0.1428571429	0		inf	#DIV/0!
SKAP2	0.04761904762	0.04761904762	0.2222222222		0.1375	#DIV/0!
SKI	0	0	0.1111111111		0	#DIV/0!
SKIDA1	0.09523809524	0.09523809524	0.1111111111		0.6842105263	#DIV/0!
SKIL	0.1904761905	0.1904761905	0.05555555556		3.294117647	#DIV/0!
KINT1I	0	0	0.05555555556		0	#DIV/0!
SKIV2L	0.04761904762	0.04761904762	0		inf	#DIV/0!
SKOR1	0.04761904762	0.04761904762	0.05555555556		0.7	#DIV/0!
SKOR2	0.04761904762	0.04761904762	0.05555555556		0.7	#DIV/0!
SKP1	0.04761904762	0.04761904762	0		inf	#DIV/0!
SKP1P2	0.04761904762	0.04761904762	0.1111111111		0.325	#DIV/0!
SKP2	0.04761904762	0.04761904762	0.05555555556		0.7	#DIV/0!
SLA	0.04761904762	0.04761904762	0.2222222222		0.1375	#DIV/0!
SLA2	0.09523809524	0.09523809524	0.1111111111		0.6842105263	#DIV/0!
SLAIN2	0.04761904762	0.04761904762	0.1111111111		0.325	#DIV/0!
SLAMF1	0.1428571429	0.1428571429	0		inf	#DIV/0!

SLAMF6	0.1428571429	0.1428571429	0		inf	#DIV/0!
SLAMF7	0.1428571429	0.1428571429	0		inf	#DIV/0!
SLAMF8	0.1428571429	0.1428571429	0		inf	#DIV/0!
SLAMF9	0.1428571429	0.1428571429	0		inf	#DIV/0!
SLBP	0.04761904762	0.04761904762	0		inf	#DIV/0!
SLC10A4	0.04761904762	0.04761904762	0.1111111111		0.325	#DIV/0!
SLC10A5	0.1428571429	0.1428571429	0.1111111111		1.083333333	#DIV/0!
SLC10A6	0	0	0.05555555556		0	#DIV/0!
SLC10A7	0	0	0.05555555556		0	#DIV/0!
SLC11A1	0	0	0.05555555556		0	#DIV/0!
SLC11A2	0.09523809524	0.09523809524	0.05555555556		1.473684211	#DIV/0!
SLC12A1	0.04761904762	0.04761904762	0.05555555556		0.7	#DIV/0!
SLC12A2	0.04761904762	0.04761904762	0		inf	#DIV/0!
SLC12A3	0.04761904762	0.04761904762	0		inf	#DIV/0!
SLC12A4	0.1428571429	0.1428571429	0.05555555556		2.333333333	#DIV/0!
SLC12A5	0.1428571429	0.1428571429	0.1666666667		0.6666666667	#DIV/0!
C12A5-A	0.1428571429	0.1428571429	0.1666666667		0.6666666667	#DIV/0!
SLC12A7	0	0	0.05555555556		0	#DIV/0!
SLC12A8	0.04761904762	0.04761904762	0.05555555556		0.7	#DIV/0!
SLC12A9	0.04761904762	0.04761904762	0.05555555556		0.7	#DIV/0!
C12A9-A	0.04761904762	0.04761904762	0.05555555556		0.7	#DIV/0!
SLC13A1	0.04761904762	0.04761904762	0.05555555556		0.7	#DIV/0!
SLC13A2	0.04761904762	0.04761904762	0.05555555556		0.7	#DIV/0!
SLC13A3	0.1428571429	0.1428571429	0.1666666667		0.6666666667	#DIV/0!
SLC13A4	0.04761904762	0.04761904762	0.05555555556		0.7	#DIV/0!
SLC13A5	0	0	0.05555555556		0	#DIV/0!
SLC14A1	0.04761904762	0.04761904762	0.05555555556		0.7	#DIV/0!
SLC14A2	0.04761904762	0.04761904762	0.05555555556		0.7	#DIV/0!
C14A2-A	0.04761904762	0.04761904762	0		inf	#DIV/0!
SLC15A2	0.09523809524	0.09523809524	0.05555555556		1.473684211	#DIV/0!
SLC15A4	0.09523809524	0.09523809524	0.05555555556		1.473684211	#DIV/0!
SLC15A5	0.04761904762	0.04761904762	0.1111111111		0.325	#DIV/0!
SLC16A1	0.04761904762	0.04761904762	0.05555555556		0.7	#DIV/0!
C16A1-A	0.04761904762	0.04761904762	0.05555555556		0.7	#DIV/0!
LC16A1	0	0	0			#DIV/0!
LC16A1	0	0	0.05555555556		0	#DIV/0!
LC16A1	0.1428571429	0.1428571429	0.1111111111		1.083333333	#DIV/0!
C16A12-	0.1428571429	0.1428571429	0.1111111111		1.083333333	#DIV/0!
LC16A1	0	0	0.05555555556		0	#DIV/0!
LC16A1	0	0	0.05555555556		0	#DIV/0!
SLC16A3	0.04761904762	0.04761904762	0		inf	#DIV/0!
SLC16A4	0.04761904762	0.04761904762	0		inf	#DIV/0!
SLC16A5	0.04761904762	0.04761904762	0.05555555556		0.7	#DIV/0!
SLC16A6	0	0	0			#DIV/0!
SLC16A7	0.09523809524	0.09523809524	0		inf	#DIV/0!
SLC16A8	0.04761904762	0.04761904762	0.1111111111		0.325	#DIV/0!
SLC16A9	0.04761904762	0.04761904762	0.05555555556		0.7	#DIV/0!
SLC17A5	0.04761904762	0.04761904762	0		inf	#DIV/0!
SLC17A7	0.09523809524	0.09523809524	0.1111111111		0.6842105263	#DIV/0!
SLC17A8	0.04761904762	0.04761904762	0.05555555556		0.7	#DIV/0!
SLC17A9	0.1428571429	0.1428571429	0.2222222222		0.4583333333	#DIV/0!

SLC18A1	0.09523809524	0.09523809524	0.1111111111		0.6842105263	#DIV/0!
SLC18A2	0.2380952381	0.2380952381	0		inf	#DIV/0!
SLC18A3	0.09523809524	0.09523809524	0.05555555556		1.473684211	#DIV/0!
SLC19A1	0.04761904762	0.04761904762	0.1111111111		0.325	#DIV/0!
SLC19A2	0.1428571429	0.1428571429	0		inf	#DIV/0!
SLC19A3	0	0	0.05555555556		0	#DIV/0!
SLC1A1	0.04761904762	0.04761904762	0.05555555556		0.7	#DIV/0!
SLC1A3	0.04761904762	0.04761904762	0.05555555556		0.7	#DIV/0!
SLC1A4	0	0	0.05555555556		0	#DIV/0!
SLC1A5	0.09523809524	0.09523809524	0.1111111111		0.6842105263	#DIV/0!
SLC1A6	0.1428571429	0.1428571429	0.1111111111		1.083333333	#DIV/0!
SLC1A7	0.04761904762	0.04761904762	0.05555555556		0.7	#DIV/0!
SLC20A1	0	0	0.05555555556		0	#DIV/0!
SLC20A2	0.09523809524	0.09523809524	0.1666666667		0.4210526316	#DIV/0!
LC22A1	0.04761904762	0.04761904762	0		inf	#DIV/0!
LC22A1	0.04761904762	0.04761904762	0		inf	#DIV/0!
LC22A1	0.04761904762	0.04761904762	0.05555555556		0.7	#DIV/0!
LC22A1	0	0	0			#DIV/0!
LC22A1	0	0	0.05555555556		0	#DIV/0!
LC22A1	0.04761904762	0.04761904762	0		inf	#DIV/0!
C22A18	0.04761904762	0.04761904762	0		inf	#DIV/0!
LC22A3	0.09523809524	0.09523809524	0		inf	#DIV/0!
SLC22A4	0.04761904762	0.04761904762	0		inf	#DIV/0!
SLC22A5	0.04761904762	0.04761904762	0		inf	#DIV/0!
SLC22A7	0.04761904762	0.04761904762	0.05555555556		0.7	#DIV/0!
SLC23A1	0.04761904762	0.04761904762	0		inf	#DIV/0!
SLC23A2	0.1904761905	0.1904761905	0.05555555556		3.294117647	#DIV/0!
SLC24A1	0.04761904762	0.04761904762	0.05555555556		0.7	#DIV/0!
SLC24A2	0.04761904762	0.04761904762	0.05555555556		0.7	#DIV/0!
SLC24A3	0.1904761905	0.1904761905	0.05555555556		3.294117647	#DIV/0!
SLC24A5	0.04761904762	0.04761904762	0.05555555556		0.7	#DIV/0!
SLC25A1	0.04761904762	0.04761904762	0.05555555556		0.7	#DIV/0!
LC25A1	0.04761904762	0.04761904762	0		inf	#DIV/0!
LC25A1	0	0	0.1111111111		0	#DIV/0!
LC25A1	0	0	0.05555555556		0	#DIV/0!
LC25A1	0.04761904762	0.04761904762	0.1111111111		0.325	#DIV/0!
LC25A1	0.1904761905	0.1904761905	0.05555555556		3.294117647	#DIV/0!
LC25A1	0	0	0.1111111111		0	#DIV/0!
LC25A1	0.04761904762	0.04761904762	0		inf	#DIV/0!
LC25A1	0.04761904762	0.04761904762	0.05555555556		0.7	#DIV/0!
SLC25A2	0.04761904762	0.04761904762	0		inf	#DIV/0!
LC25A2	0.09523809524	0.09523809524	0		inf	#DIV/0!
LC25A2	0.04761904762	0.04761904762	0		inf	#DIV/0!
LC25A2	0.1428571429	0.1428571429	0.05555555556		2.333333333	#DIV/0!
LC25A2	0.04761904762	0.04761904762	0.05555555556		0.7	#DIV/0!
LC25A2	0.09523809524	0.09523809524	0.05555555556		1.473684211	#DIV/0!
C25A25-	0.09523809524	0.09523809524	0.05555555556		1.473684211	#DIV/0!
LC25A2	0.04761904762	0.04761904762	0		inf	#DIV/0!
LC25A2	0.04761904762	0.04761904762	0.05555555556		0.7	#DIV/0!
LC25A2	0.1428571429	0.1428571429	0		inf	#DIV/0!
SLC25A3	0.04761904762	0.04761904762	0.05555555556		0.7	#DIV/0!

**LC25A3**	0	0	0.05555555556		0	#DIV/0!
**LC25A3**	0.1904761905	0.1904761905	0.1111111111		1.529411765	#DIV/0!
**LC25A3**	0	0	0.05555555556		0	#DIV/0!
**LC25A3**	0.04761904762	0.04761904762	0.1111111111		0.325	#DIV/0!
**C25A34-**	0.04761904762	0.04761904762	0.1111111111		0.325	#DIV/0!
**LC25A3**	0.04761904762	0.04761904762	0.05555555556		0.7	#DIV/0!
**LC25A3**	0.1428571429	0.1428571429	0.05555555556		2.333333333	#DIV/0!
**LC25A3**	0.04761904762	0.04761904762	0.1111111111		0.325	#DIV/0!
**LC25A3**	0.04761904762	0.04761904762	0		inf	#DIV/0!
**LC25A3**	0.09523809524	0.09523809524	0		inf	#DIV/0!
**LC25A3I**	0.04761904762	0.04761904762	0.05555555556		0.7	#DIV/0!
**SLC25A4**	0	0	0.05555555556		0	#DIV/0!
**LC25A4**	0.09523809524	0.09523809524	0.05555555556		1.473684211	#DIV/0!
**LC25A4**	0.1428571429	0.1428571429	0.05555555556		2.333333333	#DIV/0!
**LC25A4**	0.1428571429	0.1428571429	0.1111111111		1.083333333	#DIV/0!
**LC25A4**	0.04761904762	0.04761904762	0		inf	#DIV/0!
**LC25A4**	0.04761904762	0.04761904762	0		inf	#DIV/0!
**LC25A5**	0.04761904762	0.04761904762	0.05555555556		0.7	#DIV/0!
**LC25A5**	0.09523809524	0.09523809524	0		inf	#DIV/0!
**SLC26A1**	0.04761904762	0.04761904762	0		inf	#DIV/0!
**LC26A1**	0.1428571429	0.1428571429	0.05555555556		2.333333333	#DIV/0!
**LC26A1**	0.04761904762	0.04761904762	0		inf	#DIV/0!
**SLC26A3**	0.04761904762	0.04761904762	0.05555555556		0.7	#DIV/0!
**SLC26A4**	0.04761904762	0.04761904762	0.05555555556		0.7	#DIV/0!
**C26A4-A**	0.04761904762	0.04761904762	0.05555555556		0.7	#DIV/0!
**SLC26A5**	0.04761904762	0.04761904762	0.05555555556		0.7	#DIV/0!
**SLC26A6**	0.09523809524	0.09523809524	0		inf	#DIV/0!
**SLC26A7**	0.1904761905	0.1904761905	0.1111111111		1.529411765	#DIV/0!
**SLC26A8**	0.04761904762	0.04761904762	0.05555555556		0.7	#DIV/0!
**SLC26A9**	0.1428571429	0.1428571429	0		inf	#DIV/0!
**SLC27A2**	0.1428571429	0.1428571429	0.05555555556		2.333333333	#DIV/0!
**SLC27A3**	0.1904761905	0.1904761905	0.05555555556		3.294117647	#DIV/0!
**SLC27A4**	0.09523809524	0.09523809524	0.05555555556		1.473684211	#DIV/0!
**SLC27A5**	0.1428571429	0.1428571429	0.05555555556		2.333333333	#DIV/0!
**SLC27A6**	0.04761904762	0.04761904762	0		inf	#DIV/0!
**SLC28A1**	0.04761904762	0.04761904762	0.05555555556		0.7	#DIV/0!
**SLC28A2**	0.04761904762	0.04761904762	0		inf	#DIV/0!
**SLC28A3**	0	0	0.05555555556		0	#DIV/0!
**SLC29A1**	0.04761904762	0.04761904762	0.05555555556		0.7	#DIV/0!
**SLC29A3**	0.09523809524	0.09523809524	0		inf	#DIV/0!
**SLC29A4**	0.04761904762	0.04761904762	0.1666666667		0.2	#DIV/0!
**SLC2A1**	0.04761904762	0.04761904762	0.05555555556		0.7	#DIV/0!
**C2A1-A**	0.04761904762	0.04761904762	0.05555555556		0.7	#DIV/0!
**SLC2A10**	0.1428571429	0.1428571429	0.1666666667		0.6666666667	#DIV/0!
**SLC2A11**	0.04761904762	0.04761904762	0.1111111111		0.325	#DIV/0!
**SLC2A13**	0.04761904762	0.04761904762	0.05555555556		0.7	#DIV/0!
**SLC2A14**	0.04761904762	0.04761904762	0.1111111111		0.325	#DIV/0!
**SLC2A2**	0.1904761905	0.1904761905	0.05555555556		3.294117647	#DIV/0!
**SLC2A3**	0.04761904762	0.04761904762	0.1111111111		0.325	#DIV/0!
**SLC2A4**	0	0	0.05555555556		0	#DIV/0!
**LC2A4R**	0.1428571429	0.1428571429	0.2222222222		0.4583333333	#DIV/0!

SLC2A5	0	0	0.05555555556		0	#DIV/0!
SLC2A6	0.04761904762	0.04761904762	0		inf	#DIV/0!
SLC2A7	0	0	0.05555555556		0	#DIV/0!
SLC2A8	0.09523809524	0.09523809524	0.05555555556		1.473684211	#DIV/0!
SLC30A1	0.1428571429	0.1428571429	0		inf	#DIV/0!
LC30A1	0.1428571429	0.1428571429	0.05555555556		2.333333333	#DIV/0!
SLC30A2	0.04761904762	0.04761904762	0.1111111111		0.325	#DIV/0!
SLC30A4	0.04761904762	0.04761904762	0		inf	#DIV/0!
SLC30A5	0.04761904762	0.04761904762	0.05555555556		0.7	#DIV/0!
SLC30A6	0.1428571429	0.1428571429	0		inf	#DIV/0!
SLC30A7	0.04761904762	0.04761904762	0.05555555556		0.7	#DIV/0!
SLC30A8	0.1904761905	0.1904761905	0.1666666667		0.9411764706	#DIV/0!
SLC30A9	0.04761904762	0.04761904762	0		inf	#DIV/0!
SLC31A1	0.04761904762	0.04761904762	0.05555555556		0.7	#DIV/0!
SLC31A2	0.04761904762	0.04761904762	0.05555555556		0.7	#DIV/0!
SLC32A1	0.1428571429	0.1428571429	0.2222222222		0.4583333333	#DIV/0!
SLC33A1	0.1904761905	0.1904761905	0.05555555556		3.294117647	#DIV/0!
SLC34A1	0.09523809524	0.09523809524	0.05555555556		1.473684211	#DIV/0!
SLC34A3	0.1428571429	0.1428571429	0.05555555556		2.333333333	#DIV/0!
SLC35A3	0.04761904762	0.04761904762	0.05555555556		0.7	#DIV/0!
SLC35A4	0.04761904762	0.04761904762	0		inf	#DIV/0!
SLC35A5	0.09523809524	0.09523809524	0.05555555556		1.473684211	#DIV/0!
SLC35B1	0.09523809524	0.09523809524	0		inf	#DIV/0!
SLC35B2	0.04761904762	0.04761904762	0.05555555556		0.7	#DIV/0!
SLC35B4	0.04761904762	0.04761904762	0.05555555556		0.7	#DIV/0!
SLC35C2	0.1428571429	0.1428571429	0.1666666667		0.6666666667	#DIV/0!
SLC35D1	0.04761904762	0.04761904762	0.05555555556		0.7	#DIV/0!
SLC35D2	0.04761904762	0.04761904762	0.05555555556		0.7	#DIV/0!
SLC35E1	0.1428571429	0.1428571429	0.1111111111		1.083333333	#DIV/0!
LC35E2	0	0	0.1111111111		0	#DIV/0!
LC35E2	0	0	0.1111111111		0	#DIV/0!
SLC35E3	0.09523809524	0.09523809524	0.05555555556		1.473684211	#DIV/0!
SLC35E4	0.04761904762	0.04761904762	0.1111111111		0.325	#DIV/0!
SLC35F3	0.1428571429	0.1428571429	0		inf	#DIV/0!
SLC35F4	0.04761904762	0.04761904762	0		inf	#DIV/0!
SLC35F5	0	0	0.05555555556		0	#DIV/0!
SLC35G1	0.1428571429	0.1428571429	0		inf	#DIV/0!
SLC35G2	0.1904761905	0.1904761905	0.05555555556		3.294117647	#DIV/0!
SLC35G4	0.09523809524	0.09523809524	0		inf	#DIV/0!
SLC35G5	0.09523809524	0.09523809524	0.1111111111		0.6842105263	#DIV/0!
SLC35G6	0	0	0.05555555556		0	#DIV/0!
SLC37A1	0.04761904762	0.04761904762	0.1111111111		0.325	#DIV/0!
SLC37A3	0.04761904762	0.04761904762	0.05555555556		0.7	#DIV/0!
SLC38A1	0.04761904762	0.04761904762	0.05555555556		0.7	#DIV/0!
LC38A1	0.04761904762	0.04761904762	0		inf	#DIV/0!
LC38A1	0	0	0.05555555556		0	#DIV/0!
SLC38A2	0.04761904762	0.04761904762	0.05555555556		0.7	#DIV/0!
SLC38A3	0.09523809524	0.09523809524	0		inf	#DIV/0!
SLC38A4	0.04761904762	0.04761904762	0.05555555556		0.7	#DIV/0!
SLC38A7	0.04761904762	0.04761904762	0		inf	#DIV/0!
SLC38A8	0.04761904762	0.04761904762	0		inf	#DIV/0!

**SLC38A9**	0.04761904762	0.04761904762	0.05555555556		0.7	#DIV/0!
**SLC39A1**	0.1904761905	0.1904761905	0.05555555556		3.294117647	#DIV/0!
**LC39A1**	0	0	0.05555555556		0	#DIV/0!
**LC39A1**	0.04761904762	0.04761904762	0		inf	#DIV/0!
**LC39A1**	0.09523809524	0.09523809524	0.1111111111		0.6842105263	#DIV/0!
**C39A12-**	0.09523809524	0.09523809524	0.1111111111		0.6842105263	#DIV/0!
**LC39A1**	0	0	0.05555555556		0	#DIV/0!
**LC39A1**	0.09523809524	0.09523809524	0.1111111111		0.6842105263	#DIV/0!
**SLC39A3**	0.09523809524	0.09523809524	0.05555555556		1.473684211	#DIV/0!
**SLC39A4**	0.04761904762	0.04761904762	0.1666666667		0.2	#DIV/0!
**SLC39A5**	0.09523809524	0.09523809524	0.05555555556		1.473684211	#DIV/0!
**SLC39A6**	0.04761904762	0.04761904762	0		inf	#DIV/0!
**SLC39A7**	0.04761904762	0.04761904762	0		inf	#DIV/0!
**SLC39A8**	0	0	0.05555555556		0	#DIV/0!
**SLC3A1**	0	0	0.05555555556		0	#DIV/0!
**SLC40A1**	0	0	0.1666666667		0	#DIV/0!
**SLC41A1**	0.1428571429	0.1428571429	0		inf	#DIV/0!
**SLC41A2**	0.04761904762	0.04761904762	0.05555555556		0.7	#DIV/0!
**SLC41A3**	0.04761904762	0.04761904762	0.05555555556		0.7	#DIV/0!
**SLC43A2**	0	0	0.1111111111		0	#DIV/0!
**SLC44A1**	0.04761904762	0.04761904762	0.05555555556		0.7	#DIV/0!
**SLC44A2**	0.1428571429	0.1428571429	0.1111111111		1.083333333	#DIV/0!
**SLC44A3**	0.04761904762	0.04761904762	0.05555555556		0.7	#DIV/0!
**C44A3-A**	0.04761904762	0.04761904762	0.05555555556		0.7	#DIV/0!
**SLC44A4**	0.04761904762	0.04761904762	0		inf	#DIV/0!
**SLC44A5**	0.04761904762	0.04761904762	0.05555555556		0.7	#DIV/0!
**SLC45A1**	0	0	0.05555555556		0	#DIV/0!
**SLC45A2**	0.04761904762	0.04761904762	0.1111111111		0.325	#DIV/0!
**SLC45A3**	0.1428571429	0.1428571429	0		inf	#DIV/0!
**SLC45A4**	0.04761904762	0.04761904762	0.2222222222		0.1375	#DIV/0!
**SLC46A1**	0.04761904762	0.04761904762	0.05555555556		0.7	#DIV/0!
**SLC46A2**	0.04761904762	0.04761904762	0.05555555556		0.7	#DIV/0!
**SLC47A1**	0.09523809524	0.09523809524	0.05555555556		1.473684211	#DIV/0!
**SLC47A2**	0.09523809524	0.09523809524	0.05555555556		1.473684211	#DIV/0!
**SLC48A1**	0.04761904762	0.04761904762	0.05555555556		0.7	#DIV/0!
**SLC49A3**	0.04761904762	0.04761904762	0		inf	#DIV/0!
**SLC49A4**	0.09523809524	0.09523809524	0.05555555556		1.473684211	#DIV/0!
**SLC4A1**	0.09523809524	0.09523809524	0		inf	#DIV/0!
**SLC4A10**	0	0	0.05555555556		0	#DIV/0!
**SLC4A11**	0.1904761905	0.1904761905	0.05555555556		3.294117647	#DIV/0!
**LC4A1A**	0	0	0.05555555556		0	#DIV/0!
**SLC4A2**	0.09523809524	0.09523809524	0.05555555556		1.473684211	#DIV/0!
**SLC4A3**	0	0	0.05555555556		0	#DIV/0!
**SLC4A4**	0.09523809524	0.09523809524	0.05555555556		1.473684211	#DIV/0!
**SLC4A5**	0	0	0.05555555556		0	#DIV/0!
**SLC4A7**	0.04761904762	0.04761904762	0		inf	#DIV/0!
**SLC4A8**	0.04761904762	0.04761904762	0.05555555556		0.7	#DIV/0!
**SLC4A9**	0.04761904762	0.04761904762	0		inf	#DIV/0!
**SLC50A1**	0.1904761905	0.1904761905	0.1111111111		1.529411765	#DIV/0!
**SLC51A**	0.1428571429	0.1428571429	0.05555555556		2.333333333	#DIV/0!
**SLC51B**	0.04761904762	0.04761904762	0.05555555556		0.7	#DIV/0!

SLC52A1	0	0	0.1111111111		0	#DIV/0!
SLC52A2	0.04761904762	0.04761904762	0.1666666667		0.2	#DIV/0!
SLC52A3	0.1904761905	0.1904761905	0.05555555556		3.294117647	#DIV/0!
SLC5A1	0.04761904762	0.04761904762	0.1111111111		0.325	#DIV/0!
SLC5A10	0.04761904762	0.04761904762	0.1111111111		0.325	#DIV/0!
SLC5A11	0.04761904762	0.04761904762	0		inf	#DIV/0!
SLC5A2	0.04761904762	0.04761904762	0		inf	#DIV/0!
SLC5A3	0.04761904762	0.04761904762	0.1111111111		0.325	#DIV/0!
SLC5A4	0.04761904762	0.04761904762	0.1111111111		0.325	#DIV/0!
LC5A4-A	0.04761904762	0.04761904762	0.1111111111		0.325	#DIV/0!
SLC5A5	0.1428571429	0.1428571429	0.1111111111		1.083333333	#DIV/0!
SLC5A7	0	0	0.05555555556		0	#DIV/0!
SLC5A8	0.04761904762	0.04761904762	0.05555555556		0.7	#DIV/0!
SLC5A9	0	0	0.05555555556		0	#DIV/0!
SLC6A1	0.09523809524	0.09523809524	0		inf	#DIV/0!
LC6A1-A	0.04761904762	0.04761904762	0		inf	#DIV/0!
LC6A10	0.1428571429	0.1428571429	0		inf	#DIV/0!
SLC6A11	0.04761904762	0.04761904762	0		inf	#DIV/0!
SLC6A12	0.04761904762	0.04761904762	0.1111111111		0.325	#DIV/0!
SLC6A13	0.04761904762	0.04761904762	0.1111111111		0.325	#DIV/0!
SLC6A15	0.04761904762	0.04761904762	0.05555555556		0.7	#DIV/0!
SLC6A16	0.09523809524	0.09523809524	0.1111111111		0.6842105263	#DIV/0!
SLC6A17	0.04761904762	0.04761904762	0		inf	#DIV/0!
SLC6A18	0	0	0.1111111111		0	#DIV/0!
SLC6A19	0	0	0.1111111111		0	#DIV/0!
SLC6A2	0.04761904762	0.04761904762	0		inf	#DIV/0!
SLC6A3	0	0	0.1111111111		0	#DIV/0!
SLC6A4	0	0	0.05555555556		0	#DIV/0!
SLC6A6	0.04761904762	0.04761904762	0		inf	#DIV/0!
SLC6A9	0.04761904762	0.04761904762	0.05555555556		0.7	#DIV/0!
SLC7A10	0.04761904762	0.04761904762	0.05555555556		0.7	#DIV/0!
SLC7A11	0	0	0.05555555556		0	#DIV/0!
C7A11-A	0	0	0.05555555556		0	#DIV/0!
SLC7A13	0.1904761905	0.1904761905	0.1111111111		1.529411765	#DIV/0!
SLC7A14	0.1904761905	0.1904761905	0.05555555556		3.294117647	#DIV/0!
C7A14-A	0.1904761905	0.1904761905	0.05555555556		3.294117647	#DIV/0!
SLC7A2	0.09523809524	0.09523809524	0.1111111111		0.6842105263	#DIV/0!
SLC7A4	0.04761904762	0.04761904762	0.05555555556		0.7	#DIV/0!
SLC7A5	0.04761904762	0.04761904762	0		inf	#DIV/0!
LC7A5P	0.04761904762	0.04761904762	0		inf	#DIV/0!
SLC7A6	0.1428571429	0.1428571429	0.05555555556		2.333333333	#DIV/0!
LC7A6O	0.1428571429	0.1428571429	0.05555555556		2.333333333	#DIV/0!
SLC7A7	0	0	0.05555555556		0	#DIV/0!
SLC7A8	0	0	0.1111111111		0	#DIV/0!
SLC7A9	0.04761904762	0.04761904762	0.05555555556		0.7	#DIV/0!
SLC8A1	0	0	0.05555555556		0	#DIV/0!
LC8A1-A	0	0	0.05555555556		0	#DIV/0!
SLC8A2	0.09523809524	0.09523809524	0.1111111111		0.6842105263	#DIV/0!
SLC8B1	0	0	0.05555555556		0	#DIV/0!
SLC9A1	0.04761904762	0.04761904762	0.1111111111		0.325	#DIV/0!
SLC9A2	0	0	0.05555555556		0	#DIV/0!

**SLC9A3**	0	0	0.05555555556		0	#DIV/0!
**.C9A3-A**	0	0	0.05555555556		0	#DIV/0!
**LC9A3R**	0.04761904762	0.04761904762	0		inf	#DIV/0!
**LC9A3R**	0.09523809524	0.09523809524	0.2222222222		0.2894736842	#DIV/0!
**SLC9A4**	0	0	0.05555555556		0	#DIV/0!
**SLC9A5**	0.1428571429	0.1428571429	0.05555555556		2.333333333	#DIV/0!
**LC9A7P**	0.04761904762	0.04761904762	0.05555555556		0.7	#DIV/0!
**SLC9A8**	0.09523809524	0.09523809524	0.1666666667		0.4210526316	#DIV/0!
**SLC9A9**	0.1428571429	0.1428571429	0.1111111111		1.083333333	#DIV/0!
**.C9A9-A**	0.1428571429	0.1428571429	0.05555555556		2.333333333	#DIV/0!
**SLC9B1**	0	0	0.05555555556		0	#DIV/0!
**SLC9B2**	0	0	0.05555555556		0	#DIV/0!
**SLC9C1**	0.09523809524	0.09523809524	0.05555555556		1.473684211	#DIV/0!
**SLC9C2**	0.1428571429	0.1428571429	0		inf	#DIV/0!
**LCO1A**	0.04761904762	0.04761904762	0.05555555556		0.7	#DIV/0!
**LCO1B**	0.04761904762	0.04761904762	0.1111111111		0.325	#DIV/0!
**LCO1B**	0.04761904762	0.04761904762	0.1111111111		0.325	#DIV/0!
**LCO1B**	0.04761904762	0.04761904762	0.1111111111		0.325	#DIV/0!
**LCO1C**	0.04761904762	0.04761904762	0.1111111111		0.325	#DIV/0!
**LCO2A**	0.1428571429	0.1428571429	0.05555555556		2.333333333	#DIV/0!
**LCO3A**	0.04761904762	0.04761904762	0.05555555556		0.7	#DIV/0!
**LCO4A**	0.1428571429	0.1428571429	0.2222222222		0.4583333333	#DIV/0!
**CO4A1-A**	0.1428571429	0.1428571429	0.2222222222		0.4583333333	#DIV/0!
**LCO4C**	0.04761904762	0.04761904762	0		inf	#DIV/0!
**LCO5A**	0.1428571429	0.1428571429	0.1666666667		0.6666666667	#DIV/0!
**LCO6A**	0.04761904762	0.04761904762	0		inf	#DIV/0!
**SLED1**	0	0	0.05555555556		0	#DIV/0!
**SLF1**	0.04761904762	0.04761904762	0		inf	#DIV/0!
**SLF2**	0.1428571429	0.1428571429	0		inf	#DIV/0!
**LFN12I**	0.04761904762	0.04761904762	0		inf	#DIV/0!
**SLFN13**	0.04761904762	0.04761904762	0		inf	#DIV/0!
**SLFN14**	0.04761904762	0.04761904762	0		inf	#DIV/0!
**SLFNL1**	0.04761904762	0.04761904762	0.05555555556		0.7	#DIV/0!
**FNL1-A**	0.04761904762	0.04761904762	0.05555555556		0.7	#DIV/0!
**SLIT1**	0.1428571429	0.1428571429	0		inf	#DIV/0!
**LIT1-AS**	0.1428571429	0.1428571429	0		inf	#DIV/0!
**SLIT3**	0	0	0.05555555556		0	#DIV/0!
**LITRK**	0.1428571429	0.1428571429	0.05555555556		2.333333333	#DIV/0!
**SLK**	0.1428571429	0.1428571429	0.1111111111		1.083333333	#DIV/0!
**SLMAP**	0.09523809524	0.09523809524	0		inf	#DIV/0!
**IO2-AT**	0.1428571429	0.1428571429	0.2222222222		0.4583333333	#DIV/0!
**SLPI**	0.1428571429	0.1428571429	0.1666666667		0.6666666667	#DIV/0!
**SLTM**	0.04761904762	0.04761904762	0.05555555556		0.7	#DIV/0!
**SLU7**	0.04761904762	0.04761904762	0.05555555556		0.7	#DIV/0!
**SLURP1**	0.04761904762	0.04761904762	0.1666666667		0.2	#DIV/0!
**SLURP2**	0.04761904762	0.04761904762	0.1666666667		0.2	#DIV/0!
**SLX1A**	0.04761904762	0.04761904762	0		inf	#DIV/0!
**1A-SUL**	0.04761904762	0.04761904762	0		inf	#DIV/0!
**SLX1B**	0.04761904762	0.04761904762	0		inf	#DIV/0!
**1B-SUL**	0.04761904762	0.04761904762	0		inf	#DIV/0!
**SLX4**	0.04761904762	0.04761904762	0.1111111111		0.325	#DIV/0!

**SLX4IP**	0.1904761905	0.1904761905	0.1666666667		0.9411764706	#DIV/0!
**SMA4**	0.09523809524	0.09523809524	0.1666666667		0.4210526316	#DIV/0!
**SMA5**	0.09523809524	0.09523809524	0.1666666667		0.4210526316	#DIV/0!
**SMAD1**	0	0	0.05555555556		0	#DIV/0!
**IAD1-A**	0	0	0.05555555556		0	#DIV/0!
**IAD1-A**	0	0	0.05555555556		0	#DIV/0!
**SMAD2**	0.04761904762	0.04761904762	0.05555555556		0.7	#DIV/0!
**SMAD3**	0.04761904762	0.04761904762	0.05555555556		0.7	#DIV/0!
**SMAD4**	0.09523809524	0.09523809524	0		inf	#DIV/0!
**SMAD5**	0.04761904762	0.04761904762	0		inf	#DIV/0!
**IAD5-A**	0.04761904762	0.04761904762	0		inf	#DIV/0!
**SMAD6**	0.04761904762	0.04761904762	0.05555555556		0.7	#DIV/0!
**SMAD7**	0.04761904762	0.04761904762	0		inf	#DIV/0!
**SMAGP**	0.09523809524	0.09523809524	0.05555555556		1.473684211	#DIV/0!
**SMAP2**	0.04761904762	0.04761904762	0.05555555556		0.7	#DIV/0!
**MARCA**	0.04761904762	0.04761904762	0.05555555556		0.7	#DIV/0!
**MARCA**	0.1428571429	0.1428571429	0.1111111111		1.083333333	#DIV/0!
**MARCA**	0	0	0.05555555556		0	#DIV/0!
**ARCA5-**	0	0	0.05555555556		0	#DIV/0!
**IARCAI**	0	0	0.05555555556		0	#DIV/0!
**IARCAI**	0	0	0.05555555556		0	#DIV/0!
**MARCB**	0.04761904762	0.04761904762	0.1111111111		0.325	#DIV/0!
**MARCC**	0.09523809524	0.09523809524	0		inf	#DIV/0!
**MARCC**	0.09523809524	0.09523809524	0.05555555556		1.473684211	#DIV/0!
**MARCD**	0.04761904762	0.04761904762	0.05555555556		0.7	#DIV/0!
**MARCD**	0.04761904762	0.04761904762	0		inf	#DIV/0!
**MARCD**	0.09523809524	0.09523809524	0.05555555556		1.473684211	#DIV/0!
**MARCE**	0.09523809524	0.09523809524	0		inf	#DIV/0!
**SMC1B**	0	0	0.1111111111		0	#DIV/0!
**SMC2**	0.04761904762	0.04761904762	0		inf	#DIV/0!
**MC2-AS**	0.04761904762	0.04761904762	0		inf	#DIV/0!
**SMC3**	0.1428571429	0.1428571429	0.05555555556		2.333333333	#DIV/0!
**SMC4**	0.1428571429	0.1428571429	0.05555555556		2.333333333	#DIV/0!
**SMC5**	0	0	0.05555555556		0	#DIV/0!
**MC5-AS**	0	0	0.05555555556		0	#DIV/0!
**SMC6**	0	0	0.05555555556		0	#DIV/0!
**MCHD**	0.09523809524	0.09523809524	0		inf	#DIV/0!
**SMCO1**	0.1428571429	0.1428571429	0.05555555556		2.333333333	#DIV/0!
**SMCO2**	0.04761904762	0.04761904762	0.05555555556		0.7	#DIV/0!
**SMCO3**	0.04761904762	0.04761904762	0.1111111111		0.325	#DIV/0!
**SMCP**	0.1904761905	0.1904761905	0		inf	#DIV/0!
**SMCR2**	0.04761904762	0.04761904762	0.1111111111		0.325	#DIV/0!
**SMCR5**	0.04761904762	0.04761904762	0.1111111111		0.325	#DIV/0!
**SMCR8**	0.04761904762	0.04761904762	0.1111111111		0.325	#DIV/0!
**SMDT1**	0	0	0.1111111111		0	#DIV/0!
**SMG1**	0.04761904762	0.04761904762	0		inf	#DIV/0!
**SMG1P1**	0.04761904762	0.04761904762	0		inf	#DIV/0!
**SMG1P2**	0.04761904762	0.04761904762	0		inf	#DIV/0!
**SMG1P3**	0.04761904762	0.04761904762	0		inf	#DIV/0!
**SMG1P5**	0.04761904762	0.04761904762	0		inf	#DIV/0!
**SMG1P6**	0.04761904762	0.04761904762	0		inf	#DIV/0!

SMG1P7	0.1428571429	0.1428571429	0.05555555556		2.333333333	#DIV/0!
SMG6	0	0	0.1111111111		0	#DIV/0!
SMG7	0.1428571429	0.1428571429	0		inf	#DIV/0!
MG7-AS	0.1428571429	0.1428571429	0		inf	#DIV/0!
SMG8	0.1428571429	0.1428571429	0		inf	#DIV/0!
SMG9	0.09523809524	0.09523809524	0.05555555556		1.473684211	#DIV/0!
SMILR	0.1428571429	0.1428571429	0.1111111111		1.083333333	#DIV/0!
SMIM1	0	0	0.05555555556		0	#DIV/0!
MIM10L	0.04761904762	0.04761904762	0.1111111111		0.325	#DIV/0!
MIM11A	0.04761904762	0.04761904762	0.1111111111		0.325	#DIV/0!
MIM11B	0.04761904762	0.04761904762	0.1111111111		0.325	#DIV/0!
SMIM12	0.04761904762	0.04761904762	0		inf	#DIV/0!
SMIM13	0.04761904762	0.04761904762	0		inf	#DIV/0!
SMIM14	0.1904761905	0.1904761905	0.05555555556		3.294117647	#DIV/0!
SMIM15	0.04761904762	0.04761904762	0		inf	#DIV/0!
IIM15-A	0.04761904762	0.04761904762	0		inf	#DIV/0!
SMIM17	0.1428571429	0.1428571429	0.05555555556		2.333333333	#DIV/0!
SMIM18	0.04761904762	0.04761904762	0.1111111111		0.325	#DIV/0!
SMIM19	0.09523809524	0.09523809524	0.1666666667		0.4210526316	#DIV/0!
SMIM21	0.09523809524	0.09523809524	0		inf	#DIV/0!
SMIM22	0.04761904762	0.04761904762	0		inf	#DIV/0!
SMIM23	0	0	0.05555555556		0	#DIV/0!
SMIM24	0.09523809524	0.09523809524	0.05555555556		1.473684211	#DIV/0!
SMIM25	0.09523809524	0.09523809524	0.1666666667		0.4210526316	#DIV/0!
SMIM26	0.1904761905	0.1904761905	0.05555555556		3.294117647	#DIV/0!
SMIM27	0.09523809524	0.09523809524	0.05555555556		1.473684211	#DIV/0!
SMIM29	0.04761904762	0.04761904762	0		inf	#DIV/0!
SMIM30	0.04761904762	0.04761904762	0.05555555556		0.7	#DIV/0!
SMIM31	0.04761904762	0.04761904762	0.05555555556		0.7	#DIV/0!
SMIM32	0.04761904762	0.04761904762	0		inf	#DIV/0!
SMIM33	0.04761904762	0.04761904762	0		inf	#DIV/0!
MIM34A	0.04761904762	0.04761904762	0.1111111111		0.325	#DIV/0!
SMIM4	0.04761904762	0.04761904762	0		inf	#DIV/0!
SMIM40	0.04761904762	0.04761904762	0		inf	#DIV/0!
SMIM41	0.04761904762	0.04761904762	0.05555555556		0.7	#DIV/0!
SMIM7	0.1428571429	0.1428571429	0.1111111111		1.083333333	#DIV/0!
SMKR1	0.04761904762	0.04761904762	0.05555555556		0.7	#DIV/0!
SMN1	0.09523809524	0.09523809524	0.1666666667		0.4210526316	#DIV/0!
SMN2	0.09523809524	0.09523809524	0.1666666667		0.4210526316	#DIV/0!
MNDC1	0.1428571429	0.1428571429	0.05555555556		2.333333333	#DIV/0!
SMO	0.04761904762	0.04761904762	0.05555555556		0.7	#DIV/0!
SMOX	0.1904761905	0.1904761905	0.05555555556		3.294117647	#DIV/0!
SMPD1	0.04761904762	0.04761904762	0		inf	#DIV/0!
SMPD2	0	0	0			#DIV/0!
SMPD3	0.1428571429	0.1428571429	0.05555555556		2.333333333	#DIV/0!
SMPD4	0	0	0.05555555556		0	#DIV/0!
SMPD5	0.04761904762	0.04761904762	0.1666666667		0.2	#DIV/0!
MPDL3	0.04761904762	0.04761904762	0.1111111111		0.325	#DIV/0!
SMR3A	0.04761904762	0.04761904762	0.05555555556		0.7	#DIV/0!
SMR3B	0.04761904762	0.04761904762	0.05555555556		0.7	#DIV/0!
SMTN	0.04761904762	0.04761904762	0.1111111111		0.325	#DIV/0!

SMTNL2	0	0	0.1111111111		0	#DIV/0!
SMU1	0.09523809524	0.09523809524	0.05555555556		1.473684211	#DIV/0!
SMUG1	0.04761904762	0.04761904762	0.05555555556		0.7	#DIV/0!
SMURF1	0.04761904762	0.04761904762	0.05555555556		0.7	#DIV/0!
SMURF2	0.04761904762	0.04761904762	0		inf	#DIV/0!
SMYD1	0	0	0.05555555556		0	#DIV/0!
SMYD2	0.1428571429	0.1428571429	0		inf	#DIV/0!
SMYD3	0.1428571429	0.1428571429	0.05555555556		2.333333333	#DIV/0!
SMYD4	0	0	0.1111111111		0	#DIV/0!
SMYD5	0	0	0.05555555556		0	#DIV/0!
SNAI1	0.09523809524	0.09523809524	0.1666666667		0.4210526316	#DIV/0!
SNAI2	0.09523809524	0.09523809524	0.1666666667		0.4210526316	#DIV/0!
SNAI3	0.1904761905	0.1904761905	0		inf	#DIV/0!
NAI3-AS	0.1904761905	0.1904761905	0		inf	#DIV/0!
SNAP25	0.1904761905	0.1904761905	0.1111111111		1.529411765	#DIV/0!
AP25-A	0.1904761905	0.1904761905	0.1111111111		1.529411765	#DIV/0!
SNAP29	0.04761904762	0.04761904762	0.05555555556		0.7	#DIV/0!
SNAP47	0.1428571429	0.1428571429	0		inf	#DIV/0!
SNAPC2	0.1428571429	0.1428571429	0.05555555556		2.333333333	#DIV/0!
SNAPC3	0.04761904762	0.04761904762	0.05555555556		0.7	#DIV/0!
SNAPC4	0.1428571429	0.1428571429	0.05555555556		2.333333333	#DIV/0!
SNAPC5	0.04761904762	0.04761904762	0.05555555556		0.7	#DIV/0!
SNAPIN	0.1904761905	0.1904761905	0.05555555556		3.294117647	#DIV/0!
NAR-A1	0.09523809524	0.09523809524	0.1111111111		0.6842105263	#DIV/0!
NAR-A1	0.09523809524	0.09523809524	0.1111111111		0.6842105263	#DIV/0!
NAR-A1	0.09523809524	0.09523809524	0.1111111111		0.6842105263	#DIV/0!
NAR-A1	0.09523809524	0.09523809524	0.1111111111		0.6842105263	#DIV/0!
NAR-A1	0.09523809524	0.09523809524	0.1111111111		0.6842105263	#DIV/0!
NAR-A1	0.09523809524	0.09523809524	0.1111111111		0.6842105263	#DIV/0!
NAR-A2	0.09523809524	0.09523809524	0.1111111111		0.6842105263	#DIV/0!
NAR-A3	0.09523809524	0.09523809524	0.1111111111		0.6842105263	#DIV/0!
NAR-A4	0.09523809524	0.09523809524	0.1111111111		0.6842105263	#DIV/0!
NAR-A5	0.09523809524	0.09523809524	0.1111111111		0.6842105263	#DIV/0!
NAR-A6	0.09523809524	0.09523809524	0.1111111111		0.6842105263	#DIV/0!
NAR-A7	0.09523809524	0.09523809524	0.1111111111		0.6842105263	#DIV/0!
NAR-A8	0.09523809524	0.09523809524	0.1111111111		0.6842105263	#DIV/0!
NAR-A9	0.09523809524	0.09523809524	0.1111111111		0.6842105263	#DIV/0!
NAR-B1	0.09523809524	0.09523809524	0.1111111111		0.6842105263	#DIV/0!
NAR-B2	0.09523809524	0.09523809524	0.1111111111		0.6842105263	#DIV/0!
NAR-C1	0.09523809524	0.09523809524	0.1111111111		0.6842105263	#DIV/0!
NAR-C2	0.09523809524	0.09523809524	0.1111111111		0.6842105263	#DIV/0!
NAR-C3	0.09523809524	0.09523809524	0.1111111111		0.6842105263	#DIV/0!
NAR-C4	0.09523809524	0.09523809524	0.1111111111		0.6842105263	#DIV/0!
NAR-C5	0.09523809524	0.09523809524	0.1111111111		0.6842105263	#DIV/0!
SNAR-D	0.09523809524	0.09523809524	0.1111111111		0.6842105263	#DIV/0!
SNAR-E	0.09523809524	0.09523809524	0.1111111111		0.6842105263	#DIV/0!
SNAR-F	0.09523809524	0.09523809524	0.1111111111		0.6842105263	#DIV/0!
SNAR-G	0.09523809524	0.09523809524	0.1111111111		0.6842105263	#DIV/0!
NAR-G2	0.09523809524	0.09523809524	0.1111111111		0.6842105263	#DIV/0!
SNAR-H	0	0	0.05555555556		0	#DIV/0!
SNAR-I	0.09523809524	0.09523809524	0.05555555556		1.473684211	#DIV/0!

SNCA	0	0	0.05555555556		0	#DIV/0!
NCA-AS	0	0	0.05555555556		0	#DIV/0!
SNCAIP	0.04761904762	0.04761904762	0		inf	#DIV/0!
SNCB	0.09523809524	0.09523809524	0.05555555556		1.473684211	#DIV/0!
SNCG	0.1428571429	0.1428571429	0.05555555556		2.333333333	#DIV/0!
SND1	0.04761904762	0.04761904762	0.05555555556		0.7	#DIV/0!
ND1-IT	0.04761904762	0.04761904762	0.05555555556		0.7	#DIV/0!
SNED1	0	0	0.05555555556		0	#DIV/0!
SNF8	0.1428571429	0.1428571429	0		inf	#DIV/0!
SNHG11	0.1428571429	0.1428571429	0.1666666667		0.6666666667	#DIV/0!
SNHG12	0.04761904762	0.04761904762	0.1111111111		0.325	#DIV/0!
SNHG15	0.04761904762	0.04761904762	0.2222222222		0.1375	#DIV/0!
SNHG16	0.04761904762	0.04761904762	0		inf	#DIV/0!
SNHG17	0.1428571429	0.1428571429	0.1666666667		0.6666666667	#DIV/0!
SNHG18	0	0	0.05555555556		0	#DIV/0!
SNHG19	0.09523809524	0.09523809524	0.2222222222		0.2894736842	#DIV/0!
SNHG20	0.04761904762	0.04761904762	0		inf	#DIV/0!
SNHG21	0.04761904762	0.04761904762	0.05555555556		0.7	#DIV/0!
SNHG22	0.04761904762	0.04761904762	0		inf	#DIV/0!
SNHG25	0.04761904762	0.04761904762	0		inf	#DIV/0!
SNHG26	0.04761904762	0.04761904762	0.1666666667		0.2	#DIV/0!
SNHG27	0	0	0.05555555556		0	#DIV/0!
SNHG28	0.1428571429	0.1428571429	0		inf	#DIV/0!
SNHG29	0.04761904762	0.04761904762	0.05555555556		0.7	#DIV/0!
SNHG3	0.04761904762	0.04761904762	0.1111111111		0.325	#DIV/0!
SNHG30	0.04761904762	0.04761904762	0		inf	#DIV/0!
SNHG31	0	0	0.05555555556		0	#DIV/0!
SNHG32	0.04761904762	0.04761904762	0		inf	#DIV/0!
SNHG4	0.04761904762	0.04761904762	0		inf	#DIV/0!
SNHG6	0.1428571429	0.1428571429	0.1666666667		0.6666666667	#DIV/0!
SNHG7	0.1428571429	0.1428571429	0.05555555556		2.333333333	#DIV/0!
SNHG8	0	0	0.05555555556		0	#DIV/0!
SNHG9	0.09523809524	0.09523809524	0.2222222222		0.2894736842	#DIV/0!
SNIP1	0.04761904762	0.04761904762	0.05555555556		0.7	#DIV/0!
SNN	0.04761904762	0.04761904762	0		inf	#DIV/0!
NORA1	0.09523809524	0.09523809524	0.2222222222		0.2894736842	#DIV/0!
NORA10	0.1428571429	0.1428571429	0.05555555556		2.333333333	#DIV/0!
IORA10	0	0	0.05555555556		0	#DIV/0!
IORA10	0.09523809524	0.09523809524	0		inf	#DIV/0!
NORA10	0.1428571429	0.1428571429	0		inf	#DIV/0!
NORA10	0.1428571429	0.1428571429	0.1111111111		1.083333333	#DIV/0!
IORA10	0.04761904762	0.04761904762	0.1666666667		0.2	#DIV/0!
IORA10	0.04761904762	0.04761904762	0.1666666667		0.2	#DIV/0!
NORA10	0.09523809524	0.09523809524	0		inf	#DIV/0!
NORA10	0	0	0.05555555556		0	#DIV/0!
NORA11	0.04761904762	0.04761904762	0.05555555556		0.7	#DIV/0!
NORA11	0.04761904762	0.04761904762	0		inf	#DIV/0!
NORA11	0	0	0.1111111111		0	#DIV/0!
NORA11	0.09523809524	0.09523809524	0.1111111111		0.6842105263	#DIV/0!
NORA11	0.04761904762	0.04761904762	0.1666666667		0.2	#DIV/0!
NORA11	0	0	0.1111111111		0	#DIV/0!

NORA11	0.1428571429	0.1428571429	0.2222222222		0.4583333333	#DIV/0!
NORA11	0.1428571429	0.1428571429	0.1111111111		1.083333333	#DIV/0!
NORA11	0.09523809524	0.09523809524	0		inf	#DIV/0!
NORA11	0.1904761905	0.1904761905	0.05555555556		3.294117647	#DIV/0!
NORA1	0.1428571429	0.1428571429	0		inf	#DIV/0!
NORA12	0.04761904762	0.04761904762	0.1111111111		0.325	#DIV/0!
NORA1	0.04761904762	0.04761904762	0		inf	#DIV/0!
NORA14	0.1428571429	0.1428571429	0.1111111111		1.083333333	#DIV/0!
NORA14	0.1428571429	0.1428571429	0.05555555556		2.333333333	#DIV/0!
NORA1	0.2380952381	0.2380952381	0.2222222222		0.859375	#DIV/0!
ORA15I	0.04761904762	0.04761904762	0.1111111111		0.325	#DIV/0!
ORA15I	0.04761904762	0.04761904762	0.1111111111		0.325	#DIV/0!
NORA16	0.04761904762	0.04761904762	0.1111111111		0.325	#DIV/0!
NORA16	0.09523809524	0.09523809524	0		inf	#DIV/0!
NORA17	0.1428571429	0.1428571429	0.05555555556		2.333333333	#DIV/0!
NORA17	0.1428571429	0.1428571429	0.05555555556		2.333333333	#DIV/0!
NORA1	0.2380952381	0.2380952381	0		inf	#DIV/0!
NORA1	0.1428571429	0.1428571429	0.1111111111		1.083333333	#DIV/0!
NORA20	0.04761904762	0.04761904762	0.2222222222		0.1375	#DIV/0!
NORA2	0.09523809524	0.09523809524	0		inf	#DIV/0!
NORA21	0.09523809524	0.09523809524	0		inf	#DIV/0!
NORA2	0.04761904762	0.04761904762	0.1111111111		0.325	#DIV/0!
NORA22	0.2380952381	0.2380952381	0.2222222222		0.859375	#DIV/0!
NORA22	0.04761904762	0.04761904762	0.1111111111		0.325	#DIV/0!
NORA2	0.04761904762	0.04761904762	0.05555555556		0.7	#DIV/0!
NORA2	0	0	0.05555555556		0	#DIV/0!
NORA24	0.04761904762	0.04761904762	0.05555555556		0.7	#DIV/0!
NORA25	0.04761904762	0.04761904762	0.1111111111		0.325	#DIV/0!
NORA2	0.2380952381	0.2380952381	0.05555555556		4.375	#DIV/0!
NORA2	0.04761904762	0.04761904762	0.05555555556		0.7	#DIV/0!
NORA2	0.04761904762	0.04761904762	0.05555555556		0.7	#DIV/0!
NORA2	0.04761904762	0.04761904762	0.05555555556		0.7	#DIV/0!
NORA3	0.04761904762	0.04761904762	0		inf	#DIV/0!
NORA30	0.09523809524	0.09523809524	0.1111111111		0.6842105263	#DIV/0!
NORA36	0.1428571429	0.1428571429	0.05555555556		2.333333333	#DIV/0!
NORA36	0	0	0.05555555556		0	#DIV/0!
NORA3	0.09523809524	0.09523809524	0		inf	#DIV/0!
NORA3	0.04761904762	0.04761904762	0		inf	#DIV/0!
NORA38	0	0	0			#DIV/0!
NORA3	0.04761904762	0.04761904762	0.1111111111		0.325	#DIV/0!
SNORA4	0.09523809524	0.09523809524	0.05555555556		1.473684211	#DIV/0!
NORA40	0.04761904762	0.04761904762	0.05555555556		0.7	#DIV/0!
NORA40	0	0	0			#DIV/0!
NORA4	0	0	0.05555555556		0	#DIV/0!
NORA41	0.04761904762	0.04761904762	0		inf	#DIV/0!
NORA4	0.04761904762	0.04761904762	0.1111111111		0.325	#DIV/0!
NORA4	0.04761904762	0.04761904762	0		inf	#DIV/0!
NORA4	0.04761904762	0.04761904762	0		inf	#DIV/0!
NORA4	0	0	0.05555555556		0	#DIV/0!
NORA4	0.09523809524	0.09523809524	0.05555555556		1.473684211	#DIV/0!
NORA50	0.04761904762	0.04761904762	0		inf	#DIV/0!

NORA50	0.04761904762	0.04761904762	0.1666666667		0.2	#DIV/0!
NORA50	0.04761904762	0.04761904762	0		inf	#DIV/0!
NORA50	0.04761904762	0.04761904762	0.05555555556		0.7	#DIV/0!
NORA5	0.1904761905	0.1904761905	0.05555555556		3.294117647	#DIV/0!
NORA5	0.04761904762	0.04761904762	0		inf	#DIV/0!
NORA5	0.04761904762	0.04761904762	0.05555555556		0.7	#DIV/0!
NORA5	0.04761904762	0.04761904762	0		inf	#DIV/0!
NORA5	0.04761904762	0.04761904762	0.05555555556		0.7	#DIV/0!
NORA5	0.09523809524	0.09523809524	0.05555555556		1.473684211	#DIV/0!
NORA58	0.1904761905	0.1904761905	0.05555555556		3.294117647	#DIV/0!
NORA59	0.09523809524	0.09523809524	0.1111111111		0.6842105263	#DIV/0!
NORA59	0.09523809524	0.09523809524	0.1111111111		0.6842105263	#DIV/0!
NORA5	0.04761904762	0.04761904762	0.2222222222		0.1375	#DIV/0!
NORA5	0.04761904762	0.04761904762	0.2222222222		0.1375	#DIV/0!
NORA5	0.04761904762	0.04761904762	0.2222222222		0.1375	#DIV/0!
SNORA6	0.04761904762	0.04761904762	0		inf	#DIV/0!
NORA6	0.1428571429	0.1428571429	0.1666666667		0.6666666667	#DIV/0!
NORA6	0.04761904762	0.04761904762	0.1111111111		0.325	#DIV/0!
NORA6	0.04761904762	0.04761904762	0		inf	#DIV/0!
NORA6	0.09523809524	0.09523809524	0.05555555556		1.473684211	#DIV/0!
NORA63	0.09523809524	0.09523809524	0.05555555556		1.473684211	#DIV/0!
NORA63	0.04761904762	0.04761904762	0.05555555556		0.7	#DIV/0!
NORA63	0.1904761905	0.1904761905	0.05555555556		3.294117647	#DIV/0!
NORA63	0.1904761905	0.1904761905	0.05555555556		3.294117647	#DIV/0!
NORA6	0.09523809524	0.09523809524	0.2222222222		0.2894736842	#DIV/0!
NORA6	0.09523809524	0.09523809524	0.05555555556		1.473684211	#DIV/0!
NORA6	0.04761904762	0.04761904762	0.05555555556		0.7	#DIV/0!
NORA6	0	0	0.05555555556		0	#DIV/0!
NORA6	0.1428571429	0.1428571429	0.1111111111		1.083333333	#DIV/0!
NORA68	0.04761904762	0.04761904762	0.05555555556		0.7	#DIV/0!
NORA70	0.04761904762	0.04761904762	0.05555555556		0.7	#DIV/0!
NORA70	0.04761904762	0.04761904762	0		inf	#DIV/0!
NORA70	0.04761904762	0.04761904762	0		inf	#DIV/0!
NORA70	0	0	0.05555555556		0	#DIV/0!
NORA70	0.09523809524	0.09523809524	0.05555555556		1.473684211	#DIV/0!
NORA70	0.1428571429	0.1428571429	0		inf	#DIV/0!
NORA70	0	0	0.05555555556		0	#DIV/0!
NORA70	0	0	0.05555555556		0	#DIV/0!
NORA71	0.1428571429	0.1428571429	0.1666666667		0.6666666667	#DIV/0!
NORA71	0.1428571429	0.1428571429	0.1666666667		0.6666666667	#DIV/0!
NORA71	0.1428571429	0.1428571429	0.1666666667		0.6666666667	#DIV/0!
NORA71	0.1428571429	0.1428571429	0.1666666667		0.6666666667	#DIV/0!
NORA71	0.1428571429	0.1428571429	0.1666666667		0.6666666667	#DIV/0!
NORA7	0.1904761905	0.1904761905	0.1111111111		1.529411765	#DIV/0!
NORA73	0.04761904762	0.04761904762	0.1111111111		0.325	#DIV/0!
NORA73	0.04761904762	0.04761904762	0.1111111111		0.325	#DIV/0!
NORA74	0.04761904762	0.04761904762	0		inf	#DIV/0!
NORA74	0	0	0.05555555556		0	#DIV/0!
ORA74	0.04761904762	0.04761904762	0.05555555556		0.7	#DIV/0!
ORA74	0.04761904762	0.04761904762	0.05555555556		0.7	#DIV/0!
NORA74	0.04761904762	0.04761904762	0		inf	#DIV/0!

NORA7	0	0	0.05555555556		0	#DIV/0!
NORA75	0	0	0			#DIV/0!
NORA7	0.2380952381	0.2380952381	0		inf	#DIV/0!
NORA77	0.04761904762	0.04761904762	0.05555555556		0.7	#DIV/0!
NORA7	0.09523809524	0.09523809524	0.2222222222		0.2894736842	#DIV/0!
NORA79	0.04761904762	0.04761904762	0		inf	#DIV/0!
NORA7	0.04761904762	0.04761904762	0		inf	#DIV/0!
NORA7	0.09523809524	0.09523809524	0.05555555556		1.473684211	#DIV/0!
NORA80	0.04761904762	0.04761904762	0.1111111111		0.325	#DIV/0!
NORA80	0	0	0.05555555556		0	#DIV/0!
NORA80	0.04761904762	0.04761904762	0		inf	#DIV/0!
NORA80	0.04761904762	0.04761904762	0.1666666667		0.2	#DIV/0!
NORA80	0.1904761905	0.1904761905	0.1111111111		1.529411765	#DIV/0!
NORA8	0.09523809524	0.09523809524	0.05555555556		1.473684211	#DIV/0!
NORA8	0.04761904762	0.04761904762	0.05555555556		0.7	#DIV/0!
NORA8	0.09523809524	0.09523809524	0.2222222222		0.2894736842	#DIV/0!
NORA8	0.1428571429	0.1428571429	0.05555555556		2.333333333	#DIV/0!
SNORA9	0.04761904762	0.04761904762	0.2222222222		0.1375	#DIV/0!
NORA9	0.04761904762	0.04761904762	0		inf	#DIV/0!
NORA9	0.04761904762	0.04761904762	0.1111111111		0.325	#DIV/0!
NORA9	0.04761904762	0.04761904762	0.1111111111		0.325	#DIV/0!
NORA9	0.04761904762	0.04761904762	0		inf	#DIV/0!
NORA9	0.09523809524	0.09523809524	0		inf	#DIV/0!
NORA9	0.04761904762	0.04761904762	0		inf	#DIV/0!
NORA9	0.09523809524	0.09523809524	0.1111111111		0.6842105263	#DIV/0!
NORA9	0.04761904762	0.04761904762	0.1111111111		0.325	#DIV/0!
SNORC	0	0	0.05555555556		0	#DIV/0!
NORD1	0	0	0.05555555556		0	#DIV/0!
IORD10	0.04761904762	0.04761904762	0.1111111111		0.325	#DIV/0!
IORD10	0.04761904762	0.04761904762	0.1111111111		0.325	#DIV/0!
IORD10	0.04761904762	0.04761904762	0.1111111111		0.325	#DIV/0!
NORD10	0.04761904762	0.04761904762	0		inf	#DIV/0!
NORD10	0.1428571429	0.1428571429	0.1111111111		1.083333333	#DIV/0!
IORD10	0.1428571429	0.1428571429	0.1111111111		1.083333333	#DIV/0!
NORD1	0.09523809524	0.09523809524	0.05555555556		1.473684211	#DIV/0!
NORD11	0.1904761905	0.1904761905	0.05555555556		3.294117647	#DIV/0!
NORD11	0.1428571429	0.1428571429	0.05555555556		2.333333333	#DIV/0!
IORD11	0.1428571429	0.1428571429	0.05555555556		2.333333333	#DIV/0!
NORD11	0.04761904762	0.04761904762	0		inf	#DIV/0!
NORD11	0	0	0.05555555556		0	#DIV/0!
NORD11	0.1904761905	0.1904761905	0.05555555556		3.294117647	#DIV/0!
NORD11	0.09523809524	0.09523809524	0.05555555556		1.473684211	#DIV/0!
NORD1	0.09523809524	0.09523809524	0.1666666667		0.4210526316	#DIV/0!
IORD12	0.09523809524	0.09523809524	0.05555555556		1.473684211	#DIV/0!
IORD12	0.09523809524	0.09523809524	0.05555555556		1.473684211	#DIV/0!
NORD12	0	0	0.05555555556		0	#DIV/0!
NORD12	0.09523809524	0.09523809524	0		inf	#DIV/0!
NORD12	0.04761904762	0.04761904762	0.1111111111		0.325	#DIV/0!
NORD12	0.04761904762	0.04761904762	0		inf	#DIV/0!
NORD12	0.04761904762	0.04761904762	0		inf	#DIV/0!
NORD12	0	0	0.05555555556		0	#DIV/0!

NORD12	0.09523809524	0.09523809524	0.1111111111		0.6842105263	#DIV/0!
NORD12	0.09523809524	0.09523809524	0.1666666667		0.4210526316	#DIV/0!
NORD12	0.09523809524	0.09523809524	0.1666666667		0.4210526316	#DIV/0!
NORD1	0.04761904762	0.04761904762	0.1111111111		0.325	#DIV/0!
NORD13	0.09523809524	0.09523809524	0.1111111111		0.6842105263	#DIV/0!
NORD13	0.04761904762	0.04761904762	0		inf	#DIV/0!
NORD13	0	0	0.05555555556		0	#DIV/0!
NORD13	0.09523809524	0.09523809524	0.05555555556		1.473684211	#DIV/0!
NORD13	0.04761904762	0.04761904762	0		inf	#DIV/0!
NORD13	0.1428571429	0.1428571429	0.1111111111		1.083333333	#DIV/0!
NORD13	0.09523809524	0.09523809524	0.05555555556		1.473684211	#DIV/0!
NORD13	0.04761904762	0.04761904762	0		inf	#DIV/0!
NORD13	0	0	0.1111111111		0	#DIV/0!
ORD13I	0.09523809524	0.09523809524	0.1666666667		0.4210526316	#DIV/0!
ORD13I	0.09523809524	0.09523809524	0.1666666667		0.4210526316	#DIV/0!
NORD13	0.1904761905	0.1904761905	0.05555555556		3.294117647	#DIV/0!
NORD13	0.04761904762	0.04761904762	0.05555555556		0.7	#DIV/0!
NORD13	0.04761904762	0.04761904762	0.05555555556		0.7	#DIV/0!
NORD13	0.1428571429	0.1428571429	0.05555555556		2.333333333	#DIV/0!
NORD13	0.09523809524	0.09523809524	0		inf	#DIV/0!
NORD14	0	0	0.1111111111		0	#DIV/0!
ORD14	0.1428571429	0.1428571429	0.05555555556		2.333333333	#DIV/0!
ORD14	0.1428571429	0.1428571429	0.05555555556		2.333333333	#DIV/0!
NORD14	0.04761904762	0.04761904762	0.1666666667		0.2	#DIV/0!
NORD14	0	0	0.05555555556		0	#DIV/0!
NORD14	0	0	0.05555555556		0	#DIV/0!
NORD14	0.04761904762	0.04761904762	0.05555555556		0.7	#DIV/0!
NORD14	0.09523809524	0.09523809524	0		inf	#DIV/0!
NORD14	0.04761904762	0.04761904762	0		inf	#DIV/0!
NORD14	0.04761904762	0.04761904762	0.1111111111		0.325	#DIV/0!
NORD15	0.04761904762	0.04761904762	0.2222222222		0.1375	#DIV/0!
NORD15	0.04761904762	0.04761904762	0.05555555556		0.7	#DIV/0!
NORD15	0.1428571429	0.1428571429	0.1666666667		0.6666666667	#DIV/0!
NORD15	0.09523809524	0.09523809524	0.05555555556		1.473684211	#DIV/0!
NORD15	0.09523809524	0.09523809524	0.05555555556		1.473684211	#DIV/0!
NORD15	0.2380952381	0.2380952381	0		inf	#DIV/0!
NORD15	0.04761904762	0.04761904762	0.1111111111		0.325	#DIV/0!
NORD1	0.04761904762	0.04761904762	0.05555555556		0.7	#DIV/0!
NORD16	0.04761904762	0.04761904762	0.05555555556		0.7	#DIV/0!
NORD16	0.04761904762	0.04761904762	0.05555555556		0.7	#DIV/0!
NORD16	0.04761904762	0.04761904762	0.05555555556		0.7	#DIV/0!
NORD16	0.04761904762	0.04761904762	0.1666666667		0.2	#DIV/0!
NORD16	0	0	0			#DIV/0!
NORD16	0	0	0.1111111111		0	#DIV/0!
NORD16	0.1428571429	0.1428571429	0.1666666667		0.6666666667	#DIV/0!
NORD1	0.1904761905	0.1904761905	0.1111111111		1.529411765	#DIV/0!
NORD17	0.04761904762	0.04761904762	0.05555555556		0.7	#DIV/0!
NORD17	0.1904761905	0.1904761905	0.05555555556		3.294117647	#DIV/0!
NORD17	0.1904761905	0.1904761905	0.1111111111		1.529411765	#DIV/0!
NORD17	0.04761904762	0.04761904762	0.05555555556		0.7	#DIV/0!
NORD18	0.04761904762	0.04761904762	0.05555555556		0.7	#DIV/0!

NORD18	0.04761904762	0.04761904762	0.05555555556		0.7	#DIV/0!
NORD18	0.04761904762	0.04761904762	0.05555555556		0.7	#DIV/0!
NORD1	0.04761904762	0.04761904762	0		inf	#DIV/0!
NORD1	0.04761904762	0.04761904762	0		inf	#DIV/0!
NORD1	0.04761904762	0.04761904762	0		inf	#DIV/0!
SNORD2	0.09523809524	0.09523809524	0.05555555556		1.473684211	#DIV/0!
NORD2	0	0	0.05555555556		0	#DIV/0!
NORD2	0.04761904762	0.04761904762	0.05555555556		0.7	#DIV/0!
NORD2	0.09523809524	0.09523809524	0.1111111111		0.6842105263	#DIV/0!
NORD2	0.04761904762	0.04761904762	0		inf	#DIV/0!
NORD32	0.09523809524	0.09523809524	0.1111111111		0.6842105263	#DIV/0!
NORD3	0.09523809524	0.09523809524	0.1111111111		0.6842105263	#DIV/0!
NORD3	0.09523809524	0.09523809524	0.1111111111		0.6842105263	#DIV/0!
NORD35	0.09523809524	0.09523809524	0.1111111111		0.6842105263	#DIV/0!
NORD35	0.09523809524	0.09523809524	0.1111111111		0.6842105263	#DIV/0!
NORD36	0.04761904762	0.04761904762	0		inf	#DIV/0!
NORD36	0.04761904762	0.04761904762	0		inf	#DIV/0!
NORD36	0.04761904762	0.04761904762	0		inf	#DIV/0!
NORD3	0.09523809524	0.09523809524	0.05555555556		1.473684211	#DIV/0!
NORD38	0.04761904762	0.04761904762	0.05555555556		0.7	#DIV/0!
NORD38	0.04761904762	0.04761904762	0.05555555556		0.7	#DIV/0!
NORD38	0.04761904762	0.04761904762	0		inf	#DIV/0!
NORD38	0.09523809524	0.09523809524	0.1111111111		0.6842105263	#DIV/0!
NORD3	0.04761904762	0.04761904762	0.1111111111		0.325	#DIV/0!
NORD3B	0.04761904762	0.04761904762	0.1111111111		0.325	#DIV/0!
NORD3B	0.04761904762	0.04761904762	0.1111111111		0.325	#DIV/0!
NORD3	0.04761904762	0.04761904762	0.1111111111		0.325	#DIV/0!
NORD3	0.04761904762	0.04761904762	0.1111111111		0.325	#DIV/0!
NORD3	0.09523809524	0.09523809524	0.1111111111		0.6842105263	#DIV/0!
NORD3	0.04761904762	0.04761904762	0.05555555556		0.7	#DIV/0!
NORD3	0.1904761905	0.1904761905	0.1111111111		1.529411765	#DIV/0!
SNORD3	0.09523809524	0.09523809524	0.1111111111		0.6842105263	#DIV/0!
NORD3	0.1428571429	0.1428571429	0.05555555556		2.333333333	#DIV/0!
NORD3	0	0	0.05555555556		0	#DIV/0!
NORD4	0.1428571429	0.1428571429	0.1111111111		1.083333333	#DIV/0!
NORD42	0.04761904762	0.04761904762	0.05555555556		0.7	#DIV/0!
NORD42	0.04761904762	0.04761904762	0.05555555556		0.7	#DIV/0!
NORD4	0	0	0.1111111111		0	#DIV/0!
NORD4	0.1428571429	0.1428571429	0		inf	#DIV/0!
NORD45	0.04761904762	0.04761904762	0.05555555556		0.7	#DIV/0!
NORD45	0.04761904762	0.04761904762	0.05555555556		0.7	#DIV/0!
NORD45	0.04761904762	0.04761904762	0.05555555556		0.7	#DIV/0!
NORD4	0.04761904762	0.04761904762	0.05555555556		0.7	#DIV/0!
NORD4	0.1428571429	0.1428571429	0		inf	#DIV/0!
NORD4	0.04761904762	0.04761904762	0		inf	#DIV/0!
NORD49	0.04761904762	0.04761904762	0.05555555556		0.7	#DIV/0!
NORD49	0.04761904762	0.04761904762	0.05555555556		0.7	#DIV/0!
NORD4	0.04761904762	0.04761904762	0.05555555556		0.7	#DIV/0!
NORD4	0.04761904762	0.04761904762	0.05555555556		0.7	#DIV/0!
NORD5	0	0	0.05555555556		0	#DIV/0!
NORD5	0.04761904762	0.04761904762	0		inf	#DIV/0!

NORD5	0	0	0.05555555556		0	#DIV/0!
NORD53	0	0	0.05555555556		0	#DIV/0!
NORD5	0.1428571429	0.1428571429	0.1111111111		1.083333333	#DIV/0!
NORD5	0.04761904762	0.04761904762	0.05555555556		0.7	#DIV/0!
NORD5	0.1904761905	0.1904761905	0.05555555556		3.294117647	#DIV/0!
NORD5	0.1904761905	0.1904761905	0.05555555556		3.294117647	#DIV/0!
NORD58	0.04761904762	0.04761904762	0		inf	#DIV/0!
NORD58	0.04761904762	0.04761904762	0		inf	#DIV/0!
NORD58	0.04761904762	0.04761904762	0		inf	#DIV/0!
NORD59	0.1428571429	0.1428571429	0.05555555556		2.333333333	#DIV/0!
NORD59	0.1428571429	0.1428571429	0.05555555556		2.333333333	#DIV/0!
NORD6	0.09523809524	0.09523809524	0.2222222222		0.2894736842	#DIV/0!
NORD62	0.09523809524	0.09523809524	0.05555555556		1.473684211	#DIV/0!
NORD62	0.09523809524	0.09523809524	0.05555555556		1.473684211	#DIV/0!
NORD6	0.04761904762	0.04761904762	0		inf	#DIV/0!
NORD63	0.04761904762	0.04761904762	0		inf	#DIV/0!
NORD6	0.04761904762	0.04761904762	0.05555555556		0.7	#DIV/0!
NORD65	0.09523809524	0.09523809524	0.1666666667		0.4210526316	#DIV/0!
NORD65	0.04761904762	0.04761904762	0.1666666667		0.2	#DIV/0!
NORD6	0.1428571429	0.1428571429	0.05555555556		2.333333333	#DIV/0!
NORD6	0	0	0.05555555556		0	#DIV/0!
NORD6	0.09523809524	0.09523809524	0		inf	#DIV/0!
SNORD7	0.04761904762	0.04761904762	0		inf	#DIV/0!
NORD7	0.09523809524	0.09523809524	0.05555555556		1.473684211	#DIV/0!
NORD70	0.09523809524	0.09523809524	0.05555555556		1.473684211	#DIV/0!
NORD7	0.04761904762	0.04761904762	0		inf	#DIV/0!
NORD7	0.04761904762	0.04761904762	0.05555555556		0.7	#DIV/0!
NORD73	0	0	0.05555555556		0	#DIV/0!
NORD73	0	0	0.05555555556		0	#DIV/0!
NORD7	0.1428571429	0.1428571429	0		inf	#DIV/0!
NORD74	0.04761904762	0.04761904762	0.1111111111		0.325	#DIV/0!
NORD7	0.1428571429	0.1428571429	0		inf	#DIV/0!
NORD7	0.1428571429	0.1428571429	0		inf	#DIV/0!
NORD7	0.1428571429	0.1428571429	0		inf	#DIV/0!
NORD77	0.1904761905	0.1904761905	0.1111111111		1.529411765	#DIV/0!
NORD7	0.1428571429	0.1428571429	0		inf	#DIV/0!
NORD7	0.1428571429	0.1428571429	0		inf	#DIV/0!
NORD8	0.1428571429	0.1428571429	0		inf	#DIV/0!
NORD8	0.1428571429	0.1428571429	0		inf	#DIV/0!
NORD8	0	0	0.05555555556		0	#DIV/0!
NORD83	0	0	0.1111111111		0	#DIV/0!
NORD83	0	0	0.1111111111		0	#DIV/0!
NORD8	0.04761904762	0.04761904762	0		inf	#DIV/0!
NORD8	0.1904761905	0.1904761905	0.05555555556		3.294117647	#DIV/0!
NORD8	0.1428571429	0.1428571429	0.1666666667		0.6666666667	#DIV/0!
NORD88	0.09523809524	0.09523809524	0.05555555556		1.473684211	#DIV/0!
NORD88	0.09523809524	0.09523809524	0.05555555556		1.473684211	#DIV/0!
NORD88	0.09523809524	0.09523809524	0.05555555556		1.473684211	#DIV/0!
NORD8	0	0	0.05555555556		0	#DIV/0!
NORD9	0.09523809524	0.09523809524	0.05555555556		1.473684211	#DIV/0!
NORD91	0	0	0.1111111111		0	#DIV/0!

NORD91	0	0	0.1111111111		0	#DIV/0!
NORD9	0	0	0.05555555556		0	#DIV/0!
NORD9	0.04761904762	0.04761904762	0.1666666667		0.2	#DIV/0!
NORD9	0	0	0.05555555556		0	#DIV/0!
NORD9	0.09523809524	0.09523809524	0.05555555556		1.473684211	#DIV/0!
NORD96	0.09523809524	0.09523809524	0.05555555556		1.473684211	#DIV/0!
NORD9	0.1904761905	0.1904761905	0.05555555556		3.294117647	#DIV/0!
NORD9	0.04761904762	0.04761904762	0.1111111111		0.325	#DIV/0!
SNPH	0.1904761905	0.1904761905	0.1111111111		1.529411765	#DIV/0!
SNRK	0.04761904762	0.04761904762	0		inf	#DIV/0!
NRK-AS	0.04761904762	0.04761904762	0		inf	#DIV/0!
NRNP20	0	0	0.1111111111		0	#DIV/0!
NRNP2	0.04761904762	0.04761904762	0.2222222222		0.1375	#DIV/0!
NRNP2	0	0	0.05555555556		0	#DIV/0!
NRNP3	0.04761904762	0.04761904762	0.1111111111		0.325	#DIV/0!
NRNP4	0.04761904762	0.04761904762	0.1111111111		0.325	#DIV/0!
NRNP7	0.09523809524	0.09523809524	0.1111111111		0.6842105263	#DIV/0!
SNRPA1	0.04761904762	0.04761904762	0.05555555556		0.7	#DIV/0!
SNRPB	0.1904761905	0.1904761905	0.05555555556		3.294117647	#DIV/0!
SNRPB2	0.1904761905	0.1904761905	0.1111111111		1.529411765	#DIV/0!
SNRPC	0.04761904762	0.04761904762	0		inf	#DIV/0!
SNRPD1	0.04761904762	0.04761904762	0.1111111111		0.325	#DIV/0!
SNRPD2	0.09523809524	0.09523809524	0.1111111111		0.6842105263	#DIV/0!
NRPD2P	0.1428571429	0.1428571429	0		inf	#DIV/0!
SNRPD3	0.04761904762	0.04761904762	0.1111111111		0.325	#DIV/0!
SNRPE	0.2380952381	0.2380952381	0		inf	#DIV/0!
SNRPF	0.04761904762	0.04761904762	0.05555555556		0.7	#DIV/0!
SNRPG	0	0	0.05555555556		0	#DIV/0!
SNTA1	0.09523809524	0.09523809524	0.1111111111		0.6842105263	#DIV/0!
SNTB1	0.09523809524	0.09523809524	0.1111111111		0.6842105263	#DIV/0!
SNTB2	0.1428571429	0.1428571429	0.05555555556		2.333333333	#DIV/0!
SNTG1	0.09523809524	0.09523809524	0.1111111111		0.6842105263	#DIV/0!
SNTG2	0	0	0.05555555556		0	#DIV/0!
NTG2-AS	0	0	0.05555555556		0	#DIV/0!
SNTN	0.04761904762	0.04761904762	0		inf	#DIV/0!
SNU13	0	0	0.1111111111		0	#DIV/0!
SNUPN	0.04761904762	0.04761904762	0.05555555556		0.7	#DIV/0!
SNX1	0.04761904762	0.04761904762	0.05555555556		0.7	#DIV/0!
SNX10	0.04761904762	0.04761904762	0.2222222222		0.1375	#DIV/0!
SNX11	0.1428571429	0.1428571429	0		inf	#DIV/0!
SNX13	0.04761904762	0.04761904762	0.2222222222		0.1375	#DIV/0!
SNX16	0.1428571429	0.1428571429	0.1111111111		1.083333333	#DIV/0!
SNX18	0.04761904762	0.04761904762	0.05555555556		0.7	#DIV/0!
SNX2	0.04761904762	0.04761904762	0		inf	#DIV/0!
SNX20	0.04761904762	0.04761904762	0		inf	#DIV/0!
SNX21	0.1428571429	0.1428571429	0.1666666667		0.6666666667	#DIV/0!
SNX22	0.04761904762	0.04761904762	0.05555555556		0.7	#DIV/0!
SNX24	0.04761904762	0.04761904762	0		inf	#DIV/0!
SNX25	0	0	0.05555555556		0	#DIV/0!
SNX27	0.1428571429	0.1428571429	0.05555555556		2.333333333	#DIV/0!
SNX29	0.04761904762	0.04761904762	0		inf	#DIV/0!

SNX29P1	0.04761904762	0.04761904762	0		inf	#DIV/0!
SNX29P2	0.04761904762	0.04761904762	0		inf	#DIV/0!
SNX3	0	0	0			#DIV/0!
SNX30	0.04761904762	0.04761904762	0.05555555556		0.7	#DIV/0!
SNX31	0.1904761905	0.1904761905	0.1111111111		1.529411765	#DIV/0!
SNX33	0.04761904762	0.04761904762	0.05555555556		0.7	#DIV/0!
SNX4	0.04761904762	0.04761904762	0.05555555556		0.7	#DIV/0!
SNX5	0.1904761905	0.1904761905	0.1111111111		1.529411765	#DIV/0!
SNX6	0.09523809524	0.09523809524	0		inf	#DIV/0!
SNX7	0.04761904762	0.04761904762	0.05555555556		0.7	#DIV/0!
SNX8	0.04761904762	0.04761904762	0.1666666667		0.2	#DIV/0!
SOAT1	0.1428571429	0.1428571429	0		inf	#DIV/0!
SOAT2	0.04761904762	0.04761904762	0.05555555556		0.7	#DIV/0!
SOBP	0	0	0			#DIV/0!
SOCS1	0.04761904762	0.04761904762	0		inf	#DIV/0!
SOCS2	0.04761904762	0.04761904762	0.05555555556		0.7	#DIV/0!
OCS2-AS	0.04761904762	0.04761904762	0.05555555556		0.7	#DIV/0!
SOCS3	0.04761904762	0.04761904762	0		inf	#DIV/0!
SOCS4	0.04761904762	0.04761904762	0		inf	#DIV/0!
SOCS5	0	0	0.05555555556		0	#DIV/0!
SOD1	0.04761904762	0.04761904762	0.1111111111		0.325	#DIV/0!
SOGA1	0.09523809524	0.09523809524	0.1111111111		0.6842105263	#DIV/0!
SOHLH1	0.04761904762	0.04761904762	0		inf	#DIV/0!
SON	0.04761904762	0.04761904762	0.1111111111		0.325	#DIV/0!
SORBS1	0.1428571429	0.1428571429	0		inf	#DIV/0!
SORBS2	0	0	0.05555555556		0	#DIV/0!
SORBS3	0.09523809524	0.09523809524	0.1111111111		0.6842105263	#DIV/0!
SORCS1	0.1428571429	0.1428571429	0		inf	#DIV/0!
SORCS3	0.1428571429	0.1428571429	0		inf	#DIV/0!
RCS3-A	0.1428571429	0.1428571429	0		inf	#DIV/0!
SORD	0.04761904762	0.04761904762	0		inf	#DIV/0!
SORT1	0.04761904762	0.04761904762	0.05555555556		0.7	#DIV/0!
SOS1	0	0	0.05555555556		0	#DIV/0!
SOS2	0.04761904762	0.04761904762	0		inf	#DIV/0!
SOST	0.09523809524	0.09523809524	0		inf	#DIV/0!
OSTDC	0.04761904762	0.04761904762	0.2222222222		0.1375	#DIV/0!
OWAHA	0.04761904762	0.04761904762	0		inf	#DIV/0!
OWAHI	0.04761904762	0.04761904762	0.05555555556		0.7	#DIV/0!
OWAHC	0	0	0.05555555556		0	#DIV/0!
SOX10	0	0	0.1111111111		0	#DIV/0!
SOX11	0	0	0.05555555556		0	#DIV/0!
SOX12	0.1904761905	0.1904761905	0.05555555556		3.294117647	#DIV/0!
SOX13	0.2380952381	0.2380952381	0		inf	#DIV/0!
SOX14	0.1428571429	0.1428571429	0.05555555556		2.333333333	#DIV/0!
SOX15	0	0	0.05555555556		0	#DIV/0!
SOX17	0.1428571429	0.1428571429	0.1111111111		1.083333333	#DIV/0!
SOX18	0.1428571429	0.1428571429	0.2222222222		0.4583333333	#DIV/0!
SOX2	0.1428571429	0.1428571429	0.05555555556		2.333333333	#DIV/0!
OX2-OT	0.1428571429	0.1428571429	0.05555555556		2.333333333	#DIV/0!
SOX30	0.04761904762	0.04761904762	0.05555555556		0.7	#DIV/0!
SOX5	0.04761904762	0.04761904762	0.05555555556		0.7	#DIV/0!

**OX5-AS**	0.04761904762	0.04761904762	0.05555555556		0.7	#DIV/0!
**SOX7**	0.09523809524	0.09523809524	0.1111111111		0.6842105263	#DIV/0!
**SOX8**	0.04761904762	0.04761904762	0.2222222222		0.1375	#DIV/0!
**SOX9**	0	0	0			#DIV/0!
**OX9-AS**	0	0	0			#DIV/0!
**SP1**	0.04761904762	0.04761904762	0.05555555556		0.7	#DIV/0!
**SP100**	0	0	0.05555555556		0	#DIV/0!
**SP110**	0	0	0.05555555556		0	#DIV/0!
**SP140**	0	0	0.05555555556		0	#DIV/0!
**SP140L**	0	0	0.05555555556		0	#DIV/0!
**SP2**	0.1428571429	0.1428571429	0		inf	#DIV/0!
**SP2-AS1**	0.1428571429	0.1428571429	0		inf	#DIV/0!
**SP3**	0	0	0.1111111111		0	#DIV/0!
**SP4**	0.04761904762	0.04761904762	0.1666666667		0.2	#DIV/0!
**SP5**	0	0	0.05555555556		0	#DIV/0!
**SP6**	0.1428571429	0.1428571429	0		inf	#DIV/0!
**SP7**	0.04761904762	0.04761904762	0.05555555556		0.7	#DIV/0!
**SP8**	0.04761904762	0.04761904762	0.1666666667		0.2	#DIV/0!
**SP9**	0	0	0.1111111111		0	#DIV/0!
**SPAAR**	0.09523809524	0.09523809524	0.05555555556		1.473684211	#DIV/0!
**SPACA3**	0.04761904762	0.04761904762	0		inf	#DIV/0!
**SPACA4**	0.09523809524	0.09523809524	0.1111111111		0.6842105263	#DIV/0!
**SPACA9**	0.04761904762	0.04761904762	0		inf	#DIV/0!
**SPAG1**	0.1904761905	0.1904761905	0.1111111111		1.529411765	#DIV/0!
**SPAG11A**	0.09523809524	0.09523809524	0.2222222222		0.2894736842	#DIV/0!
**SPAG11B**	0.09523809524	0.09523809524	0.2222222222		0.2894736842	#DIV/0!
**SPAG16**	0	0	0.05555555556		0	#DIV/0!
**SPAG17**	0.04761904762	0.04761904762	0.05555555556		0.7	#DIV/0!
**SPAG4**	0.09523809524	0.09523809524	0.1111111111		0.6842105263	#DIV/0!
**SPAG5**	0.04761904762	0.04761904762	0.05555555556		0.7	#DIV/0!
**PAG5-AS**	0.04761904762	0.04761904762	0.05555555556		0.7	#DIV/0!
**SPAG6**	0.09523809524	0.09523809524	0.1111111111		0.6842105263	#DIV/0!
**SPAG7**	0	0	0.1111111111		0	#DIV/0!
**SPAG8**	0.09523809524	0.09523809524	0.05555555556		1.473684211	#DIV/0!
**SPAG9**	0.04761904762	0.04761904762	0		inf	#DIV/0!
**SPAM1**	0.04761904762	0.04761904762	0.05555555556		0.7	#DIV/0!
**PARCL**	0	0	0.05555555556		0	#DIV/0!
**SPAST**	0.1428571429	0.1428571429	0		inf	#DIV/0!
**SPATA1**	0.04761904762	0.04761904762	0.05555555556		0.7	#DIV/0!
**SPATA1**	0.04761904762	0.04761904762	0		inf	#DIV/0!
**SPATA1**	0.1904761905	0.1904761905	0.05555555556		3.294117647	#DIV/0!
**SPATA1**	0.1428571429	0.1428571429	0		inf	#DIV/0!
**ATA17-A**	0.1428571429	0.1428571429	0		inf	#DIV/0!
**SPATA1**	0.2380952381	0.2380952381	0.05555555556		4.375	#DIV/0!
**SPATA2**	0.09523809524	0.09523809524	0.1666666667		0.4210526316	#DIV/0!
**SPATA2**	0.09523809524	0.09523809524	0		inf	#DIV/0!
**SPATA2**	0.04761904762	0.04761904762	0.1111111111		0.325	#DIV/0!
**SPATA2**	0	0	0.1111111111		0	#DIV/0!
**SPATA2**	0.04761904762	0.04761904762	0		inf	#DIV/0!
**SPATA2**	0.1428571429	0.1428571429	0.1666666667		0.6666666667	#DIV/0!
**SPATA2**	0.09523809524	0.09523809524	0		inf	#DIV/0!

**SPATA3**	0	0	0.05555555556		0	#DIV/0!
**ATA3-A**	0	0	0.05555555556		0	#DIV/0!
**PATA31**	0.04761904762	0.04761904762	0.1111111111		0.325	#DIV/0!
**PATA31**	0.04761904762	0.04761904762	0.1111111111		0.325	#DIV/0!
**PATA31**	0.09523809524	0.09523809524	0.2222222222		0.2894736842	#DIV/0!
**PATA31**	0.1428571429	0.1428571429	0.05555555556		2.333333333	#DIV/0!
**PATA31**	0.09523809524	0.09523809524	0.2222222222		0.2894736842	#DIV/0!
**PATA31**	0	0	0.05555555556		0	#DIV/0!
**PATA31**	0	0	0.05555555556		0	#DIV/0!
**PATA31**	0	0	0.05555555556		0	#DIV/0!
**PATA31**	0	0	0.05555555556		0	#DIV/0!
**PATA31**	0	0	0.05555555556		0	#DIV/0!
**ATA31D**	0	0	0.05555555556		0	#DIV/0!
**PATA31**	0	0	0.05555555556		0	#DIV/0!
**SPATA3**	0.1428571429	0.1428571429	0		inf	#DIV/0!
**SPATA3**	0.09523809524	0.09523809524	0		inf	#DIV/0!
**SPATA4**	0	0	0.05555555556		0	#DIV/0!
**SPATA4**	0.04761904762	0.04761904762	0.05555555556		0.7	#DIV/0!
**SPATA4**	0.04761904762	0.04761904762	0.05555555556		0.7	#DIV/0!
**SPATA4**	0.09523809524	0.09523809524	0		inf	#DIV/0!
**SPATA4**	0.1428571429	0.1428571429	0		inf	#DIV/0!
**SPATA4**	0.09523809524	0.09523809524	0.2222222222		0.2894736842	#DIV/0!
**SPATA5**	0	0	0.05555555556		0	#DIV/0!
**PATA5L**	0.04761904762	0.04761904762	0		inf	#DIV/0!
**SPATA6**	0	0	0.05555555556		0	#DIV/0!
**SPATA6**	0.04761904762	0.04761904762	0.05555555556		0.7	#DIV/0!
**SPATA8**	0.04761904762	0.04761904762	0.05555555556		0.7	#DIV/0!
**ATA8-A**	0.04761904762	0.04761904762	0.05555555556		0.7	#DIV/0!
**SPATA9**	0.04761904762	0.04761904762	0		inf	#DIV/0!
**SPATC1**	0.04761904762	0.04761904762	0.1666666667		0.2	#DIV/0!
**SPATC1**	0.04761904762	0.04761904762	0.1111111111		0.325	#DIV/0!
**SPATS1**	0.04761904762	0.04761904762	0.05555555556		0.7	#DIV/0!
**SPATS2**	0.04761904762	0.04761904762	0.05555555556		0.7	#DIV/0!
**SPATS2L**	0	0	0.05555555556		0	#DIV/0!
**SPC24**	0.1428571429	0.1428571429	0.1111111111		1.083333333	#DIV/0!
**SPC25**	0.04761904762	0.04761904762	0.05555555556		0.7	#DIV/0!
**SPCS3**	0	0	0.05555555556		0	#DIV/0!
**SPDEF**	0.04761904762	0.04761904762	0		inf	#DIV/0!
**SPDL1**	0	0	0.05555555556		0	#DIV/0!
**SPDYA**	0	0	0.05555555556		0	#DIV/0!
**SPDYE1**	0.04761904762	0.04761904762	0.1666666667		0.2	#DIV/0!
**PDYE10**	0.1428571429	0.1428571429	0.1666666667		0.6666666667	#DIV/0!
**SPDYE1**	0.1428571429	0.1428571429	0.1666666667		0.6666666667	#DIV/0!
**PDYE13**	0.1428571429	0.1428571429	0.1666666667		0.6666666667	#DIV/0!
**PDYE14**	0.1428571429	0.1428571429	0.1666666667		0.6666666667	#DIV/0!
**PDYE15**	0.1428571429	0.1428571429	0.1666666667		0.6666666667	#DIV/0!
**SPDYE1**	0.09523809524	0.09523809524	0.1111111111		0.6842105263	#DIV/0!
**SPDYE1**	0.1428571429	0.1428571429	0.1666666667		0.6666666667	#DIV/0!
**SPDYE1**	0.04761904762	0.04761904762	0.1111111111		0.325	#DIV/0!
**SPDYE2**	0.04761904762	0.04761904762	0.05555555556		0.7	#DIV/0!
**SPDYE2**	0.04761904762	0.04761904762	0.05555555556		0.7	#DIV/0!

SPDYE3	0.04761904762	0.04761904762	0.05555555556		0.7	#DIV/0!
SPDYE4	0.04761904762	0.04761904762	0.05555555556		0.7	#DIV/0!
SPDYE5	0.1428571429	0.1428571429	0.1666666667		0.6666666667	#DIV/0!
SPDYE6	0.04761904762	0.04761904762	0.05555555556		0.7	#DIV/0!
PDYE7	0.1428571429	0.1428571429	0.1111111111		1.083333333	#DIV/0!
PDYE8	0.1428571429	0.1428571429	0.1666666667		0.6666666667	#DIV/0!
SPECC1	0.09523809524	0.09523809524	0.05555555556		1.473684211	#DIV/0!
PECC1I	0.04761904762	0.04761904762	0.1111111111		0.325	#DIV/0!
1L-ADC	0.04761904762	0.04761904762	0.1111111111		0.325	#DIV/0!
SPEF1	0.1904761905	0.1904761905	0.05555555556		3.294117647	#DIV/0!
SPEF2	0.04761904762	0.04761904762	0.05555555556		0.7	#DIV/0!
SPEG	0	0	0.05555555556		0	#DIV/0!
SPEGNB	0	0	0.05555555556		0	#DIV/0!
SPEM1	0	0	0.05555555556		0	#DIV/0!
SPEM2	0	0	0.05555555556		0	#DIV/0!
SPEM3	0	0	0.05555555556		0	#DIV/0!
SPEN	0.04761904762	0.04761904762	0.1111111111		0.325	#DIV/0!
SPESP1	0.04761904762	0.04761904762	0.05555555556		0.7	#DIV/0!
SPG21	0.04761904762	0.04761904762	0.05555555556		0.7	#DIV/0!
SPG7	0.09523809524	0.09523809524	0		inf	#DIV/0!
SPHAR	0.1428571429	0.1428571429	0		inf	#DIV/0!
SPHK1	0.04761904762	0.04761904762	0		inf	#DIV/0!
SPHK2	0.09523809524	0.09523809524	0.1111111111		0.6842105263	#DIV/0!
SPHKAP	0	0	0.05555555556		0	#DIV/0!
SPI1	0	0	0.05555555556		0	#DIV/0!
SPIB	0.09523809524	0.09523809524	0.1111111111		0.6842105263	#DIV/0!
SPIC	0.04761904762	0.04761904762	0.05555555556		0.7	#DIV/0!
SPICE1	0.09523809524	0.09523809524	0.05555555556		1.473684211	#DIV/0!
SPIDR	0.09523809524	0.09523809524	0.1666666667		0.4210526316	#DIV/0!
SPIN1	0	0	0.05555555556		0	#DIV/0!
SPINK2	0.1904761905	0.1904761905	0.05555555556		3.294117647	#DIV/0!
SPINK4	0.09523809524	0.09523809524	0.05555555556		1.473684211	#DIV/0!
SPINK8	0.09523809524	0.09523809524	0		inf	#DIV/0!
SPINT2	0.04761904762	0.04761904762	0.05555555556		0.7	#DIV/0!
SPINT3	0.1428571429	0.1428571429	0.1666666667		0.6666666667	#DIV/0!
SPINT4	0.1428571429	0.1428571429	0.1666666667		0.6666666667	#DIV/0!
SPIRE1	0.09523809524	0.09523809524	0.05555555556		1.473684211	#DIV/0!
SPIRE2	0.09523809524	0.09523809524	0		inf	#DIV/0!
SPN	0.04761904762	0.04761904762	0		inf	#DIV/0!
SPNS1	0.04761904762	0.04761904762	0		inf	#DIV/0!
SPNS2	0	0	0.1111111111		0	#DIV/0!
SPNS3	0	0	0.1111111111		0	#DIV/0!
SPO11	0.1428571429	0.1428571429	0.2222222222		0.4583333333	#DIV/0!
SPOCD1	0.04761904762	0.04761904762	0.1111111111		0.325	#DIV/0!
SPOCK1	0	0	0			#DIV/0!
SPOCK2	0.1428571429	0.1428571429	0		inf	#DIV/0!
SPOCK3	0	0	0.05555555556		0	#DIV/0!
SPON2	0.04761904762	0.04761904762	0		inf	#DIV/0!
SPOP	0.09523809524	0.09523809524	0		inf	#DIV/0!
SPOPL	0	0	0.05555555556		0	#DIV/0!
SPOUT1	0.09523809524	0.09523809524	0.05555555556		1.473684211	#DIV/0!

**SPP1**	0	0	0.05555555556		0	#DIV/0!
**SPP2**	0	0	0.05555555556		0	#DIV/0!
**SPPL2A**	0.1428571429	0.1428571429	0.05555555556		2.333333333	#DIV/0!
**SPPL2B**	0.09523809524	0.09523809524	0.1111111111		0.6842105263	#DIV/0!
**SPPL2C**	0.1428571429	0.1428571429	0		inf	#DIV/0!
**SPPL3**	0.04761904762	0.04761904762	0.1111111111		0.325	#DIV/0!
**SPR**	0	0	0.05555555556		0	#DIV/0!
**SPRED1**	0	0	0			#DIV/0!
**SPRED2**	0	0	0.05555555556		0	#DIV/0!
**SPRED3**	0.04761904762	0.04761904762	0.05555555556		0.7	#DIV/0!
**SPRN**	0.1904761905	0.1904761905	0		inf	#DIV/0!
**SPRNP1**	0.1904761905	0.1904761905	0		inf	#DIV/0!
**SPRR1A**	0.1904761905	0.1904761905	0		inf	#DIV/0!
**SPRR1B**	0.1904761905	0.1904761905	0		inf	#DIV/0!
**SPRR2A**	0.1904761905	0.1904761905	0		inf	#DIV/0!
**SPRR2B**	0.1904761905	0.1904761905	0		inf	#DIV/0!
**SPRR2C**	0.1904761905	0.1904761905	0		inf	#DIV/0!
**SPRR2D**	0.1904761905	0.1904761905	0		inf	#DIV/0!
**SPRR2E**	0.1904761905	0.1904761905	0		inf	#DIV/0!
**SPRR2F**	0.1904761905	0.1904761905	0		inf	#DIV/0!
**SPRR2G**	0.1904761905	0.1904761905	0		inf	#DIV/0!
**SPRR3**	0.1904761905	0.1904761905	0		inf	#DIV/0!
**SPRR4**	0.1904761905	0.1904761905	0		inf	#DIV/0!
**SPRTN**	0.1428571429	0.1428571429	0		inf	#DIV/0!
**SPRY1**	0	0	0.05555555556		0	#DIV/0!
**SPRY4**	0.04761904762	0.04761904762	0		inf	#DIV/0!
**PRY4-AS**	0.04761904762	0.04761904762	0		inf	#DIV/0!
**PRY4-IT**	0.04761904762	0.04761904762	0		inf	#DIV/0!
**SPRYD3**	0.04761904762	0.04761904762	0.05555555556		0.7	#DIV/0!
**SPSB1**	0	0	0.05555555556		0	#DIV/0!
**SPSB2**	0.04761904762	0.04761904762	0.1111111111		0.325	#DIV/0!
**SPSB3**	0.09523809524	0.09523809524	0.2222222222		0.2894736842	#DIV/0!
**SPSB4**	0.1428571429	0.1428571429	0.05555555556		2.333333333	#DIV/0!
**SPTA1**	0.1428571429	0.1428571429	0.05555555556		2.333333333	#DIV/0!
**SPTAN1**	0.09523809524	0.09523809524	0.05555555556		1.473684211	#DIV/0!
**SPTBN1**	0.04761904762	0.04761904762	0.05555555556		0.7	#DIV/0!
**SPTBN4**	0.04761904762	0.04761904762	0.05555555556		0.7	#DIV/0!
**SPTLC1**	0.04761904762	0.04761904762	0.05555555556		0.7	#DIV/0!
**SPTLC3**	0.1904761905	0.1904761905	0.1111111111		1.529411765	#DIV/0!
**SPTSSA**	0.09523809524	0.09523809524	0		inf	#DIV/0!
**SPTSSB**	0.1428571429	0.1428571429	0.05555555556		2.333333333	#DIV/0!
**SPX**	0.04761904762	0.04761904762	0.05555555556		0.7	#DIV/0!
**SPZ1**	0.04761904762	0.04761904762	0		inf	#DIV/0!
**SQLE**	0.1428571429	0.1428571429	0.1111111111		1.083333333	#DIV/0!
**SQOR**	0.04761904762	0.04761904762	0		inf	#DIV/0!
**SQSTM1**	0.09523809524	0.09523809524	0.05555555556		1.473684211	#DIV/0!
**SRA1**	0.04761904762	0.04761904762	0		inf	#DIV/0!
**SRARP**	0.04761904762	0.04761904762	0.1111111111		0.325	#DIV/0!
**SRBD1**	0	0	0.05555555556		0	#DIV/0!
**SRC**	0.09523809524	0.09523809524	0.1666666667		0.4210526316	#DIV/0!
**SRCAP**	0.04761904762	0.04761904762	0		inf	#DIV/0!

SRCIN1	0.04761904762	0.04761904762	0		inf	#DIV/0!
SRD5A1	0	0	0.05555555556		0	#DIV/0!
SRD5A2	0.04761904762	0.04761904762	0		inf	#DIV/0!
SRD5A3	0.1904761905	0.1904761905	0.05555555556		3.294117647	#DIV/0!
D5A3-A	0.1904761905	0.1904761905	0.05555555556		3.294117647	#DIV/0!
SREBF1	0.04761904762	0.04761904762	0.1111111111		0.325	#DIV/0!
SREBF2	0	0	0.1111111111		0	#DIV/0!
EBF2-A	0	0	0.1111111111		0	#DIV/0!
SREK1	0.04761904762	0.04761904762	0		inf	#DIV/0!
REK1IP	0.04761904762	0.04761904762	0		inf	#DIV/0!
SRF	0.04761904762	0.04761904762	0.05555555556		0.7	#DIV/0!
SRFBP1	0.04761904762	0.04761904762	0		inf	#DIV/0!
SRGAP1	0.1428571429	0.1428571429	0.05555555556		2.333333333	#DIV/0!
SRGAP2	0.1428571429	0.1428571429	0		inf	#DIV/0!
GAP2-A	0.04761904762	0.04761904762	0.1111111111		0.325	#DIV/0!
RGAP2I	0.04761904762	0.04761904762	0.1111111111		0.325	#DIV/0!
RGAP2C	0.04761904762	0.04761904762	0.1111111111		0.325	#DIV/0!
RGAP2I	0.1428571429	0.1428571429	0.1111111111		1.083333333	#DIV/0!
SRGAP3	0.04761904762	0.04761904762	0		inf	#DIV/0!
GAP3-A	0.04761904762	0.04761904762	0		inf	#DIV/0!
GAP3-A	0.04761904762	0.04761904762	0		inf	#DIV/0!
GAP3-A	0.04761904762	0.04761904762	0		inf	#DIV/0!
SRGN	0.1904761905	0.1904761905	0.05555555556		3.294117647	#DIV/0!
SRI	0.09523809524	0.09523809524	0.1111111111		0.6842105263	#DIV/0!
SRL	0.04761904762	0.04761904762	0.1111111111		0.325	#DIV/0!
SRM	0.04761904762	0.04761904762	0.1111111111		0.325	#DIV/0!
SRMS	0.1428571429	0.1428571429	0.2222222222		0.4583333333	#DIV/0!
SRP14	0	0	0			#DIV/0!
RP14-AS	0	0	0			#DIV/0!
SRP19	0.04761904762	0.04761904762	0		inf	#DIV/0!
SRP54	0.09523809524	0.09523809524	0		inf	#DIV/0!
RP54-AS	0.09523809524	0.09523809524	0		inf	#DIV/0!
SRP68	0.04761904762	0.04761904762	0		inf	#DIV/0!
SRP72	0.1904761905	0.1904761905	0.05555555556		3.294117647	#DIV/0!
SRP9	0.1428571429	0.1428571429	0.05555555556		2.333333333	#DIV/0!
SRPK1	0.04761904762	0.04761904762	0.05555555556		0.7	#DIV/0!
SRPK2	0.04761904762	0.04761904762	0.05555555556		0.7	#DIV/0!
SRPRB	0.1428571429	0.1428571429	0.05555555556		2.333333333	#DIV/0!
SRR	0	0	0.1111111111		0	#DIV/0!
SRRD	0.04761904762	0.04761904762	0.05555555556		0.7	#DIV/0!
SRRM1	0.04761904762	0.04761904762	0.1111111111		0.325	#DIV/0!
SRRM2	0.04761904762	0.04761904762	0.1111111111		0.325	#DIV/0!
RM2-A	0.04761904762	0.04761904762	0.1111111111		0.325	#DIV/0!
SRRM3	0.1428571429	0.1428571429	0.1111111111		1.083333333	#DIV/0!
SRRM4	0	0	0.05555555556		0	#DIV/0!
SRRM5	0.09523809524	0.09523809524	0.05555555556		1.473684211	#DIV/0!
SRRT	0.04761904762	0.04761904762	0.05555555556		0.7	#DIV/0!
SRSF1	0.09523809524	0.09523809524	0		inf	#DIV/0!
SRSF10	0.04761904762	0.04761904762	0.1111111111		0.325	#DIV/0!
SRSF11	0.04761904762	0.04761904762	0.05555555556		0.7	#DIV/0!
SRSF2	0.04761904762	0.04761904762	0		inf	#DIV/0!

**SRSF3**	0.04761904762	0.04761904762	0.05555555556		0.7	#DIV/0!
**SRSF4**	0.04761904762	0.04761904762	0.1111111111		0.325	#DIV/0!
**SRSF6**	0.1428571429	0.1428571429	0.1111111111		1.083333333	#DIV/0!
**SRSF7**	0	0	0.05555555556		0	#DIV/0!
**SRSF9**	0	0	0.1111111111		0	#DIV/0!
**SRXN1**	0.1904761905	0.1904761905	0.05555555556		3.294117647	#DIV/0!
**SS18**	0.09523809524	0.09523809524	0.05555555556		1.473684211	#DIV/0!
**SS18L1**	0.1428571429	0.1428571429	0.2222222222		0.4583333333	#DIV/0!
**SS18L2**	0.04761904762	0.04761904762	0		inf	#DIV/0!
**SSB**	0	0	0.05555555556		0	#DIV/0!
**SSBP1**	0.04761904762	0.04761904762	0.05555555556		0.7	#DIV/0!
**SSBP2**	0.04761904762	0.04761904762	0		inf	#DIV/0!
**SSBP3**	0.04761904762	0.04761904762	0.05555555556		0.7	#DIV/0!
**SBP3-AS**	0.04761904762	0.04761904762	0.05555555556		0.7	#DIV/0!
**SSBP4**	0.1428571429	0.1428571429	0.1111111111		1.083333333	#DIV/0!
**SSC4D**	0.09523809524	0.09523809524	0.1111111111		0.6842105263	#DIV/0!
**SSC5D**	0.09523809524	0.09523809524	0.05555555556		1.473684211	#DIV/0!
**SSH1**	0	0	0.05555555556		0	#DIV/0!
**SSH2**	0	0	0.05555555556		0	#DIV/0!
**SSMEM1**	0.04761904762	0.04761904762	0.05555555556		0.7	#DIV/0!
**SSNA1**	0.1428571429	0.1428571429	0.05555555556		2.333333333	#DIV/0!
**SSPN**	0.04761904762	0.04761904762	0.05555555556		0.7	#DIV/0!
**SSPO**	0.09523809524	0.09523809524	0.05555555556		1.473684211	#DIV/0!
**SSR2**	0.1428571429	0.1428571429	0.1111111111		1.083333333	#DIV/0!
**SSR3**	0.1428571429	0.1428571429	0.05555555556		2.333333333	#DIV/0!
**SSR4P1**	0.04761904762	0.04761904762	0.1111111111		0.325	#DIV/0!
**SST**	0.09523809524	0.09523809524	0.1111111111		0.6842105263	#DIV/0!
**SSTR2**	0.04761904762	0.04761904762	0		inf	#DIV/0!
**SSTR3**	0	0	0.1111111111		0	#DIV/0!
**SSTR4**	0.1904761905	0.1904761905	0.05555555556		3.294117647	#DIV/0!
**SSTR5**	0.04761904762	0.04761904762	0.2222222222		0.1375	#DIV/0!
**STR5-AS**	0.04761904762	0.04761904762	0.2222222222		0.1375	#DIV/0!
**SSU72**	0	0	0.1111111111		0	#DIV/0!
**SSUH2**	0.04761904762	0.04761904762	0		inf	#DIV/0!
**SSX2IP**	0.04761904762	0.04761904762	0.05555555556		0.7	#DIV/0!
**ST13**	0	0	0.1111111111		0	#DIV/0!
**ST18**	0.09523809524	0.09523809524	0.1111111111		0.6842105263	#DIV/0!
**ST20**	0.04761904762	0.04761904762	0.05555555556		0.7	#DIV/0!
**T20-AS**	0.04761904762	0.04761904762	0.05555555556		0.7	#DIV/0!
**20-MTH**	0.04761904762	0.04761904762	0.05555555556		0.7	#DIV/0!
**T3GAL**	0.04761904762	0.04761904762	0.2222222222		0.1375	#DIV/0!
**T3GAL**	0.1428571429	0.1428571429	0.05555555556		2.333333333	#DIV/0!
**T3GAL**	0.04761904762	0.04761904762	0.05555555556		0.7	#DIV/0!
**T3GAL**	0	0	0.05555555556		0	#DIV/0!
**GAL5-A**	0	0	0.05555555556		0	#DIV/0!
**T3GAL**	0.04761904762	0.04761904762	0.05555555556		0.7	#DIV/0!
**GAL6-A**	0.04761904762	0.04761904762	0.05555555556		0.7	#DIV/0!
**T6GAL**	0.09523809524	0.09523809524	0.05555555556		1.473684211	#DIV/0!
**T6GAL**	0	0	0.05555555556		0	#DIV/0!
**GALNA**	0.04761904762	0.04761904762	0		inf	#DIV/0!
**GALNA**	0.04761904762	0.04761904762	0		inf	#DIV/0!

GALNA	0.04761904762	0.04761904762	0.05555555556		0.7	#DIV/0!
GALNA	0.09523809524	0.09523809524	0.05555555556		1.473684211	#DIV/0!
GALNA	0.04761904762	0.04761904762	0.05555555556		0.7	#DIV/0!
GALNA	0.09523809524	0.09523809524	0.05555555556		1.473684211	#DIV/0!
ST7	0.09523809524	0.09523809524	0.2222222222		0.2894736842	#DIV/0!
ST7-AS1	0.09523809524	0.09523809524	0.2222222222		0.2894736842	#DIV/0!
ST7-AS2	0.09523809524	0.09523809524	0.1666666667		0.4210526316	#DIV/0!
ST7-OT3	0.09523809524	0.09523809524	0.1666666667		0.4210526316	#DIV/0!
ST7-OT4	0.09523809524	0.09523809524	0.2222222222		0.2894736842	#DIV/0!
ST7L	0.04761904762	0.04761904762	0.05555555556		0.7	#DIV/0!
ST8SIA1	0.04761904762	0.04761904762	0.05555555556		0.7	#DIV/0!
ST8SIA2	0.04761904762	0.04761904762	0.05555555556		0.7	#DIV/0!
ST8SIA3	0.09523809524	0.09523809524	0		inf	#DIV/0!
ST8SIA4	0.04761904762	0.04761904762	0		inf	#DIV/0!
ST8SIA5	0.04761904762	0.04761904762	0.05555555556		0.7	#DIV/0!
ST8SIA6	0.09523809524	0.09523809524	0.1111111111		0.6842105263	#DIV/0!
8SIA6-A	0.09523809524	0.09523809524	0.1111111111		0.6842105263	#DIV/0!
STAB1	0.04761904762	0.04761904762	0		inf	#DIV/0!
STAB2	0.04761904762	0.04761904762	0.05555555556		0.7	#DIV/0!
STAC	0.04761904762	0.04761904762	0		inf	#DIV/0!
STAC2	0.09523809524	0.09523809524	0		inf	#DIV/0!
STAC3	0.1428571429	0.1428571429	0.05555555556		2.333333333	#DIV/0!
STAG1	0.1904761905	0.1904761905	0.05555555556		3.294117647	#DIV/0!
STAG3	0.04761904762	0.04761904762	0.05555555556		0.7	#DIV/0!
TAG3L	0.1428571429	0.1428571429	0.1666666667		0.6666666667	#DIV/0!
TAG3L	0.1428571429	0.1428571429	0.1666666667		0.6666666667	#DIV/0!
TAG3L	0.1428571429	0.1428571429	0.1111111111		1.083333333	#DIV/0!
TAG3L	0.04761904762	0.04761904762	0.1111111111		0.325	#DIV/0!
TAG3L5	0.04761904762	0.04761904762	0.05555555556		0.7	#DIV/0!
-PVRIG	0.04761904762	0.04761904762	0.05555555556		0.7	#DIV/0!
STAM	0.09523809524	0.09523809524	0.1111111111		0.6842105263	#DIV/0!
TAM-AS	0.09523809524	0.09523809524	0.1111111111		0.6842105263	#DIV/0!
STAM2	0	0	0.05555555556		0	#DIV/0!
STAMBI	0	0	0.05555555556		0	#DIV/0!
TAMBPI	0.1428571429	0.1428571429	0.1666666667		0.6666666667	#DIV/0!
STAP1	0.04761904762	0.04761904762	0.05555555556		0.7	#DIV/0!
STAP2	0.09523809524	0.09523809524	0.05555555556		1.473684211	#DIV/0!
STAR	0.09523809524	0.09523809524	0.1111111111		0.6842105263	#DIV/0!
STARD3	0.09523809524	0.09523809524	0		inf	#DIV/0!
TARD3N	0.04761904762	0.04761904762	0.2222222222		0.1375	#DIV/0!
STARD4	0.04761904762	0.04761904762	0		inf	#DIV/0!
ARD4-A	0.04761904762	0.04761904762	0		inf	#DIV/0!
STARD5	0.04761904762	0.04761904762	0.05555555556		0.7	#DIV/0!
STARD6	0.09523809524	0.09523809524	0		inf	#DIV/0!
STARD7	0	0	0.1111111111		0	#DIV/0!
ARD7-A	0	0	0.1111111111		0	#DIV/0!
STAT2	0.1428571429	0.1428571429	0.05555555556		2.333333333	#DIV/0!
STAT3	0.04761904762	0.04761904762	0		inf	#DIV/0!
STAT4	0	0	0.05555555556		0	#DIV/0!
STAT5A	0.04761904762	0.04761904762	0		inf	#DIV/0!
STAT5B	0.04761904762	0.04761904762	0		inf	#DIV/0!

**STAT6**	0.1428571429	0.1428571429	0.05555555556		2.333333333	#DIV/0!
**STATH**	0.04761904762	0.04761904762	0.05555555556		0.7	#DIV/0!
**STAU1**	0.09523809524	0.09523809524	0.1666666667		0.4210526316	#DIV/0!
**STAU2**	0.1904761905	0.1904761905	0.1666666667		0.9411764706	#DIV/0!
**TAU2-AS**	0.1904761905	0.1904761905	0.1666666667		0.9411764706	#DIV/0!
**STBD1**	0.04761904762	0.04761904762	0.05555555556		0.7	#DIV/0!
**STC1**	0.04761904762	0.04761904762	0.1111111111		0.325	#DIV/0!
**STC2**	0	0	0.05555555556		0	#DIV/0!
**STEAP1**	0.09523809524	0.09523809524	0.1111111111		0.6842105263	#DIV/0!
**TEAP1B**	0.04761904762	0.04761904762	0.1666666667		0.2	#DIV/0!
**STEAP2**	0.09523809524	0.09523809524	0.1111111111		0.6842105263	#DIV/0!
**EAP2-A**	0.09523809524	0.09523809524	0.1111111111		0.6842105263	#DIV/0!
**STEAP3**	0	0	0.05555555556		0	#DIV/0!
**EAP3-A**	0	0	0.05555555556		0	#DIV/0!
**STEAP4**	0.09523809524	0.09523809524	0.1111111111		0.6842105263	#DIV/0!
**STH**	0.1428571429	0.1428571429	0		inf	#DIV/0!
**STIL**	0.04761904762	0.04761904762	0.05555555556		0.7	#DIV/0!
**STIM1**	0.04761904762	0.04761904762	0		inf	#DIV/0!
**TIMAT**	0.04761904762	0.04761904762	0		inf	#DIV/0!
**ATE-MU**	0.04761904762	0.04761904762	0		inf	#DIV/0!
**STK10**	0	0	0.05555555556		0	#DIV/0!
**STK11**	0.09523809524	0.09523809524	0.1111111111		0.6842105263	#DIV/0!
**STK11IP**	0	0	0.05555555556		0	#DIV/0!
**STK17A**	0.04761904762	0.04761904762	0.1666666667		0.2	#DIV/0!
**STK17B**	0	0	0.05555555556		0	#DIV/0!
**STK19**	0.04761904762	0.04761904762	0		inf	#DIV/0!
**STK25**	0	0	0.05555555556		0	#DIV/0!
**STK3**	0.1904761905	0.1904761905	0.1111111111		1.529411765	#DIV/0!
**STK31**	0.04761904762	0.04761904762	0.1666666667		0.2	#DIV/0!
**STK32C**	0.2380952381	0.2380952381	0		inf	#DIV/0!
**STK35**	0.1904761905	0.1904761905	0.1111111111		1.529411765	#DIV/0!
**STK36**	0	0	0.05555555556		0	#DIV/0!
**STK38**	0.04761904762	0.04761904762	0.05555555556		0.7	#DIV/0!
**STK38L**	0.04761904762	0.04761904762	0.05555555556		0.7	#DIV/0!
**STK39**	0.04761904762	0.04761904762	0.05555555556		0.7	#DIV/0!
**STK4**	0.1428571429	0.1428571429	0.1666666667		0.6666666667	#DIV/0!
**TK4-AS**	0.1428571429	0.1428571429	0.1666666667		0.6666666667	#DIV/0!
**STK40**	0.04761904762	0.04761904762	0.05555555556		0.7	#DIV/0!
**STKLD1**	0.04761904762	0.04761904762	0		inf	#DIV/0!
**STMN1**	0.04761904762	0.04761904762	0.1111111111		0.325	#DIV/0!
**STMN2**	0.1428571429	0.1428571429	0.1111111111		1.083333333	#DIV/0!
**STMN3**	0.1428571429	0.1428571429	0.2222222222		0.4583333333	#DIV/0!
**STMN4**	0.04761904762	0.04761904762	0.1111111111		0.325	#DIV/0!
**STMP1**	0.04761904762	0.04761904762	0.05555555556		0.7	#DIV/0!
**STN1**	0.1428571429	0.1428571429	0.1111111111		1.083333333	#DIV/0!
**STOM**	0.09523809524	0.09523809524	0.05555555556		1.473684211	#DIV/0!
**STOML1**	0.04761904762	0.04761904762	0.05555555556		0.7	#DIV/0!
**STOML2**	0.09523809524	0.09523809524	0.05555555556		1.473684211	#DIV/0!
**STON1**	0	0	0.05555555556		0	#DIV/0!
**N1-GTF2**	0	0	0.05555555556		0	#DIV/0!
**STOX1**	0.1904761905	0.1904761905	0.05555555556		3.294117647	#DIV/0!

**STOX2**	0	0	0.05555555556		0	#DIV/0!
**STPG1**	0.04761904762	0.04761904762	0.1111111111		0.325	#DIV/0!
**STPG2**	0	0	0.05555555556		0	#DIV/0!
**TPG2-AS**	0	0	0.05555555556		0	#DIV/0!
**STPG3**	0.1428571429	0.1428571429	0.05555555556		2.333333333	#DIV/0!
**TPG3-AS**	0.1428571429	0.1428571429	0.05555555556		2.333333333	#DIV/0!
**STPG4**	0	0	0.05555555556		0	#DIV/0!
**STRA6**	0.04761904762	0.04761904762	0.05555555556		0.7	#DIV/0!
**STRA8**	0.04761904762	0.04761904762	0.05555555556		0.7	#DIV/0!
**STRADA**	0.04761904762	0.04761904762	0		inf	#DIV/0!
**STRADB**	0	0	0.05555555556		0	#DIV/0!
**STRAP**	0.04761904762	0.04761904762	0.1111111111		0.325	#DIV/0!
**STRBP**	0.09523809524	0.09523809524	0.05555555556		1.473684211	#DIV/0!
**STRIP1**	0.04761904762	0.04761904762	0.05555555556		0.7	#DIV/0!
**STRIP2**	0.04761904762	0.04761904762	0.05555555556		0.7	#DIV/0!
**STRIT1**	0.1904761905	0.1904761905	0.05555555556		3.294117647	#DIV/0!
**STRN**	0	0	0.05555555556		0	#DIV/0!
**STRN3**	0	0	0			#DIV/0!
**STRN4**	0.09523809524	0.09523809524	0.1111111111		0.6842105263	#DIV/0!
**STT3B**	0.04761904762	0.04761904762	0		inf	#DIV/0!
**STUB1**	0.04761904762	0.04761904762	0.2222222222		0.1375	#DIV/0!
**STUM**	0.1428571429	0.1428571429	0		inf	#DIV/0!
**STX10**	0.1428571429	0.1428571429	0.1111111111		1.083333333	#DIV/0!
**STX12**	0.04761904762	0.04761904762	0.1111111111		0.325	#DIV/0!
**STX16**	0.1428571429	0.1428571429	0.2222222222		0.4583333333	#DIV/0!
**X16-NPE**	0.1428571429	0.1428571429	0.2222222222		0.4583333333	#DIV/0!
**STX17**	0.04761904762	0.04761904762	0		inf	#DIV/0!
**TX17-AS**	0.04761904762	0.04761904762	0		inf	#DIV/0!
**STX19**	0.04761904762	0.04761904762	0.05555555556		0.7	#DIV/0!
**STX1A**	0.1428571429	0.1428571429	0.1111111111		1.083333333	#DIV/0!
**STX1B**	0.04761904762	0.04761904762	0		inf	#DIV/0!
**STX2**	0.09523809524	0.09523809524	0.05555555556		1.473684211	#DIV/0!
**STX4**	0.04761904762	0.04761904762	0		inf	#DIV/0!
**STX6**	0.1428571429	0.1428571429	0		inf	#DIV/0!
**STX8**	0.04761904762	0.04761904762	0.05555555556		0.7	#DIV/0!
**STXBP1**	0.09523809524	0.09523809524	0.05555555556		1.473684211	#DIV/0!
**STXBP2**	0.1428571429	0.1428571429	0.05555555556		2.333333333	#DIV/0!
**STXBP3**	0.04761904762	0.04761904762	0.05555555556		0.7	#DIV/0!
**STXBP4**	0.09523809524	0.09523809524	0		inf	#DIV/0!
**TXBP5I**	0.1428571429	0.1428571429	0.05555555556		2.333333333	#DIV/0!
**STYK1**	0.04761904762	0.04761904762	0.1111111111		0.325	#DIV/0!
**STYX**	0.04761904762	0.04761904762	0		inf	#DIV/0!
**STYXL1**	0.1428571429	0.1428571429	0.1111111111		1.083333333	#DIV/0!
**SUB1**	0.04761904762	0.04761904762	0.05555555556		0.7	#DIV/0!
**SUCLG1**	0	0	0.05555555556		0	#DIV/0!
**SUCLG2**	0.04761904762	0.04761904762	0		inf	#DIV/0!
**CLG2-A**	0.04761904762	0.04761904762	0		inf	#DIV/0!
**SUCNR1**	0.1904761905	0.1904761905	0.1111111111		1.529411765	#DIV/0!
**SUCO**	0.1428571429	0.1428571429	0		inf	#DIV/0!
**SUDS3**	0	0	0.05555555556		0	#DIV/0!
**SUFU**	0.1428571429	0.1428571429	0.05555555556		2.333333333	#DIV/0!

SUGCT	0.04761904762	0.04761904762	0.2222222222		0.1375	#DIV/0!
SUGP1	0.1428571429	0.1428571429	0.1111111111		1.083333333	#DIV/0!
SUGP2	0.1428571429	0.1428571429	0.1111111111		1.083333333	#DIV/0!
UGT1P	0.09523809524	0.09523809524	0.05555555556		1.473684211	#DIV/0!
1P4-STR	0.04761904762	0.04761904762	0		inf	#DIV/0!
TRA6LI	0.04761904762	0.04761904762	0		inf	#DIV/0!
SULF1	0.1428571429	0.1428571429	0.1666666667		0.6666666667	#DIV/0!
SULF2	0.1428571429	0.1428571429	0.1666666667		0.6666666667	#DIV/0!
ULT1A	0.04761904762	0.04761904762	0		inf	#DIV/0!
ULT1A	0.04761904762	0.04761904762	0		inf	#DIV/0!
ULT1A	0.04761904762	0.04761904762	0		inf	#DIV/0!
ULT1A	0.04761904762	0.04761904762	0		inf	#DIV/0!
ULT1B	0.04761904762	0.04761904762	0.05555555556		0.7	#DIV/0!
ULT1C	0	0	0.05555555556		0	#DIV/0!
LT1C2	0	0	0.05555555556		0	#DIV/0!
ULT1C	0	0	0.05555555556		0	#DIV/0!
ULT1C	0	0	0.05555555556		0	#DIV/0!
ULT1E	0.04761904762	0.04761904762	0.05555555556		0.7	#DIV/0!
ULT2A	0.09523809524	0.09523809524	0.1111111111		0.6842105263	#DIV/0!
ULT2B	0.09523809524	0.09523809524	0.1111111111		0.6842105263	#DIV/0!
ULT4A	0	0	0.1666666667		0	#DIV/0!
ULT6B	0	0	0.05555555556		0	#DIV/0!
SUMF1	0.04761904762	0.04761904762	0		inf	#DIV/0!
SUMF2	0.2380952381	0.2380952381	0.2222222222		0.859375	#DIV/0!
SUMO1	0.09523809524	0.09523809524	0.05555555556		1.473684211	#DIV/0!
UMO1P	0.09523809524	0.09523809524	0.1111111111		0.6842105263	#DIV/0!
UMO1P	0.1428571429	0.1428571429	0		inf	#DIV/0!
SUMO2	0.04761904762	0.04761904762	0.05555555556		0.7	#DIV/0!
SUMO3	0.04761904762	0.04761904762	0.1111111111		0.325	#DIV/0!
SUN1	0.04761904762	0.04761904762	0.1666666667		0.2	#DIV/0!
SUN2	0.04761904762	0.04761904762	0.1111111111		0.325	#DIV/0!
SUN3	0.04761904762	0.04761904762	0.2222222222		0.1375	#DIV/0!
SUN5	0.09523809524	0.09523809524	0.1111111111		0.6842105263	#DIV/0!
SUOX	0.09523809524	0.09523809524	0.05555555556		1.473684211	#DIV/0!
SUPT3H	0.04761904762	0.04761904762	0.1111111111		0.325	#DIV/0!
UPT4H	0.09523809524	0.09523809524	0		inf	#DIV/0!
SUPT5H	0.04761904762	0.04761904762	0.05555555556		0.7	#DIV/0!
SUPT6H	0.04761904762	0.04761904762	0.05555555556		0.7	#DIV/0!
SUPT7L	0	0	0.05555555556		0	#DIV/0!
UPV3L	0.1904761905	0.1904761905	0.05555555556		3.294117647	#DIV/0!
SURF1	0.04761904762	0.04761904762	0		inf	#DIV/0!
SURF2	0.04761904762	0.04761904762	0		inf	#DIV/0!
SURF4	0.04761904762	0.04761904762	0		inf	#DIV/0!
SURF6	0.04761904762	0.04761904762	0		inf	#DIV/0!
SUSD1	0.04761904762	0.04761904762	0.05555555556		0.7	#DIV/0!
SUSD2	0.04761904762	0.04761904762	0.1111111111		0.325	#DIV/0!
SUSD3	0.04761904762	0.04761904762	0.05555555556		0.7	#DIV/0!
SUSD4	0.1428571429	0.1428571429	0.05555555556		2.333333333	#DIV/0!
SUSD5	0.09523809524	0.09523809524	0		inf	#DIV/0!
UV39H	0.09523809524	0.09523809524	0.1111111111		0.6842105263	#DIV/0!
SUZ12	0	0	0			#DIV/0!

SUZ12P1	0	0	0.05555555556		0	#DIV/0!
SV2A	0.1428571429	0.1428571429	0.05555555556		2.333333333	#DIV/0!
SV2B	0.04761904762	0.04761904762	0.05555555556		0.7	#DIV/0!
SV2C	0.04761904762	0.04761904762	0		inf	#DIV/0!
SVBP	0.04761904762	0.04761904762	0.05555555556		0.7	#DIV/0!
SVEP1	0.04761904762	0.04761904762	0.05555555556		0.7	#DIV/0!
SVIL	0.09523809524	0.09523809524	0.2222222222		0.2894736842	#DIV/0!
SVIL-AS	0.09523809524	0.09523809524	0.2222222222		0.2894736842	#DIV/0!
SVIL2P	0.09523809524	0.09523809524	0.2222222222		0.2894736842	#DIV/0!
SVOP	0	0	0.05555555556		0	#DIV/0!
SVOPL	0.04761904762	0.04761904762	0.05555555556		0.7	#DIV/0!
SWAP70	0.04761904762	0.04761904762	0.05555555556		0.7	#DIV/0!
SWI5	0.09523809524	0.09523809524	0.05555555556		1.473684211	#DIV/0!
SWT1	0.1428571429	0.1428571429	0.05555555556		2.333333333	#DIV/0!
SYBU	0.1904761905	0.1904761905	0.1111111111		1.529411765	#DIV/0!
SYCE1	0.1904761905	0.1904761905	0		inf	#DIV/0!
SYCE1L	0.04761904762	0.04761904762	0		inf	#DIV/0!
SYCE2	0.1428571429	0.1428571429	0.1111111111		1.083333333	#DIV/0!
SYCE3	0	0	0.1111111111		0	#DIV/0!
SYCN	0.04761904762	0.04761904762	0.05555555556		0.7	#DIV/0!
SYCP1	0.04761904762	0.04761904762	0.05555555556		0.7	#DIV/0!
SYCP2	0.1428571429	0.1428571429	0.1666666667		0.6666666667	#DIV/0!
SYCP2L	0.04761904762	0.04761904762	0		inf	#DIV/0!
SYCP3	0.04761904762	0.04761904762	0.05555555556		0.7	#DIV/0!
SYDE1	0.1428571429	0.1428571429	0.1111111111		1.083333333	#DIV/0!
SYDE2	0.04761904762	0.04761904762	0.05555555556		0.7	#DIV/0!
SYF2	0.04761904762	0.04761904762	0.1111111111		0.325	#DIV/0!
SYK	0.04761904762	0.04761904762	0.05555555556		0.7	#DIV/0!
SYMPK	0.09523809524	0.09523809524	0.1111111111		0.6842105263	#DIV/0!
SYN2	0.09523809524	0.09523809524	0		inf	#DIV/0!
SYN3	0.04761904762	0.04761904762	0.1666666667		0.2	#DIV/0!
SYNC	0.04761904762	0.04761904762	0.1111111111		0.325	#DIV/0!
SYNDIG	0.1904761905	0.1904761905	0.05555555556		3.294117647	#DIV/0!
YNDIG1	0.04761904762	0.04761904762	0		inf	#DIV/0!
SYNE2	0.09523809524	0.09523809524	0		inf	#DIV/0!
SYNE4	0.04761904762	0.04761904762	0.05555555556		0.7	#DIV/0!
YNGAP	0.04761904762	0.04761904762	0		inf	#DIV/0!
SYNGR1	0	0	0.1111111111		0	#DIV/0!
SYNGR2	0.04761904762	0.04761904762	0		inf	#DIV/0!
SYNGR3	0.09523809524	0.09523809524	0.2222222222		0.2894736842	#DIV/0!
SYNGR4	0.09523809524	0.09523809524	0.1111111111		0.6842105263	#DIV/0!
SYNJ1	0.04761904762	0.04761904762	0.1111111111		0.325	#DIV/0!
SYNM	0.04761904762	0.04761904762	0.05555555556		0.7	#DIV/0!
SYNPO2	0	0	0.05555555556		0	#DIV/0!
YNPO2I	0.1904761905	0.1904761905	0.05555555556		3.294117647	#DIV/0!
SYNPR	0.04761904762	0.04761904762	0		inf	#DIV/0!
YNPR-A	0.04761904762	0.04761904762	0		inf	#DIV/0!
SYPL1	0.04761904762	0.04761904762	0.05555555556		0.7	#DIV/0!
SYPL2	0.04761904762	0.04761904762	0.05555555556		0.7	#DIV/0!
SYS1	0.1428571429	0.1428571429	0.1666666667		0.6666666667	#DIV/0!
S1-DBNI	0.1428571429	0.1428571429	0.1666666667		0.6666666667	#DIV/0!

**SYT1**	0.04761904762	0.04761904762	0.05555555556		0.7	#DIV/0!
**SYT10**	0.04761904762	0.04761904762	0.05555555556		0.7	#DIV/0!
**SYT11**	0.1904761905	0.1904761905	0.1111111111		1.529411765	#DIV/0!
**SYT14**	0.1428571429	0.1428571429	0		inf	#DIV/0!
**YT14P1**	0.04761904762	0.04761904762	0.05555555556		0.7	#DIV/0!
**SYT15**	0.1428571429	0.1428571429	0.05555555556		2.333333333	#DIV/0!
**SYT17**	0.04761904762	0.04761904762	0		inf	#DIV/0!
**SYT2**	0.1428571429	0.1428571429	0		inf	#DIV/0!
**SYT3**	0.09523809524	0.09523809524	0.05555555556		1.473684211	#DIV/0!
**SYT4**	0.04761904762	0.04761904762	0		inf	#DIV/0!
**SYT5**	0.09523809524	0.09523809524	0.05555555556		1.473684211	#DIV/0!
**SYT6**	0.04761904762	0.04761904762	0.05555555556		0.7	#DIV/0!
**SYT8**	0.04761904762	0.04761904762	0		inf	#DIV/0!
**SYT9**	0.04761904762	0.04761904762	0		inf	#DIV/0!
**SYTL1**	0.04761904762	0.04761904762	0.1111111111		0.325	#DIV/0!
**SZRD1**	0.04761904762	0.04761904762	0.1111111111		0.325	#DIV/0!
**SZT2**	0.04761904762	0.04761904762	0.05555555556		0.7	#DIV/0!
**ZT2-AS**	0.04761904762	0.04761904762	0.05555555556		0.7	#DIV/0!
**TAB1**	0	0	0.1111111111		0	#DIV/0!
**TAC1**	0.04761904762	0.04761904762	0.05555555556		0.7	#DIV/0!
**TAC3**	0.1428571429	0.1428571429	0.05555555556		2.333333333	#DIV/0!
**TAC4**	0.09523809524	0.09523809524	0		inf	#DIV/0!
**TACC1**	0.09523809524	0.09523809524	0.1111111111		0.6842105263	#DIV/0!
**TACC2**	0.2380952381	0.2380952381	0.05555555556		4.375	#DIV/0!
**TACC3**	0.04761904762	0.04761904762	0		inf	#DIV/0!
**TACO1**	0.04761904762	0.04761904762	0		inf	#DIV/0!
**TACR1**	0	0	0.05555555556		0	#DIV/0!
**TACR2**	0.09523809524	0.09523809524	0		inf	#DIV/0!
**TACR3**	0	0	0.05555555556		0	#DIV/0!
**ACSTD**	0.04761904762	0.04761904762	0.05555555556		0.7	#DIV/0!
**TADA1**	0.1428571429	0.1428571429	0		inf	#DIV/0!
**TADA3**	0.09523809524	0.09523809524	0		inf	#DIV/0!
**TAF10**	0.04761904762	0.04761904762	0		inf	#DIV/0!
**TAF11**	0.04761904762	0.04761904762	0		inf	#DIV/0!
**TAF12**	0.04761904762	0.04761904762	0.1111111111		0.325	#DIV/0!
**TAF13**	0.04761904762	0.04761904762	0.05555555556		0.7	#DIV/0!
**TAF15**	0.09523809524	0.09523809524	0		inf	#DIV/0!
**TAF1A**	0.1428571429	0.1428571429	0.05555555556		2.333333333	#DIV/0!
**AF1A-AS**	0.1428571429	0.1428571429	0.05555555556		2.333333333	#DIV/0!
**TAF1B**	0	0	0.05555555556		0	#DIV/0!
**TAF1C**	0.04761904762	0.04761904762	0		inf	#DIV/0!
**TAF1L**	0.09523809524	0.09523809524	0.05555555556		1.473684211	#DIV/0!
**TAF2**	0.09523809524	0.09523809524	0.1666666667		0.4210526316	#DIV/0!
**TAF3**	0.09523809524	0.09523809524	0.1111111111		0.6842105263	#DIV/0!
**TAF4**	0.1428571429	0.1428571429	0.2222222222		0.4583333333	#DIV/0!
**TAF4B**	0.09523809524	0.09523809524	0.05555555556		1.473684211	#DIV/0!
**TAF5**	0.1428571429	0.1428571429	0.05555555556		2.333333333	#DIV/0!
**TAF5L**	0.1428571429	0.1428571429	0		inf	#DIV/0!
**TAF6**	0.04761904762	0.04761904762	0.05555555556		0.7	#DIV/0!
**TAF7**	0.04761904762	0.04761904762	0		inf	#DIV/0!
**TAF8**	0.04761904762	0.04761904762	0.05555555556		0.7	#DIV/0!

**TAF9**	0.04761904762	0.04761904762	0.05555555556		0.7	#DIV/0!
**TAFA1**	0.04761904762	0.04761904762	0		inf	#DIV/0!
**TAFA2**	0.09523809524	0.09523809524	0		inf	#DIV/0!
**TAFA3**	0.04761904762	0.04761904762	0.05555555556		0.7	#DIV/0!
**TAFA4**	0.04761904762	0.04761904762	0		inf	#DIV/0!
**TAFA5**	0.04761904762	0.04761904762	0.1666666667		0.2	#DIV/0!
**TAL1**	0.04761904762	0.04761904762	0.05555555556		0.7	#DIV/0!
**TAL2**	0.04761904762	0.04761904762	0.05555555556		0.7	#DIV/0!
**TALDO1**	0.04761904762	0.04761904762	0		inf	#DIV/0!
**TAMM4**	0.09523809524	0.09523809524	0		inf	#DIV/0!
**TANC1**	0	0	0.05555555556		0	#DIV/0!
**TANC2**	0.04761904762	0.04761904762	0		inf	#DIV/0!
**TANGO2**	0.04761904762	0.04761904762	0.05555555556		0.7	#DIV/0!
**TANGO6**	0.1428571429	0.1428571429	0.05555555556		2.333333333	#DIV/0!
**TANK**	0	0	0.05555555556		0	#DIV/0!
**TAOK1**	0.04761904762	0.04761904762	0.05555555556		0.7	#DIV/0!
**TAOK2**	0.04761904762	0.04761904762	0		inf	#DIV/0!
**TAOK3**	0	0	0.05555555556		0	#DIV/0!
**TAP1**	0.04761904762	0.04761904762	0		inf	#DIV/0!
**TAP2**	0.04761904762	0.04761904762	0		inf	#DIV/0!
**TAPBP**	0.04761904762	0.04761904762	0		inf	#DIV/0!
**TAPBPL**	0.04761904762	0.04761904762	0.1111111111		0.325	#DIV/0!
**TAPT1**	0	0	0			#DIV/0!
**APT1-AS**	0	0	0			#DIV/0!
**TARBP1**	0.09523809524	0.09523809524	0.05555555556		1.473684211	#DIV/0!
**TARBP2**	0.04761904762	0.04761904762	0.05555555556		0.7	#DIV/0!
**TARDBP**	0.04761904762	0.04761904762	0.1111111111		0.325	#DIV/0!
**TARM1**	0.09523809524	0.09523809524	0.05555555556		1.473684211	#DIV/0!
**TARP**	0.04761904762	0.04761904762	0.2222222222		0.1375	#DIV/0!
**TARS**	0.04761904762	0.04761904762	0.1111111111		0.325	#DIV/0!
**TARS2**	0.1904761905	0.1904761905	0.05555555556		3.294117647	#DIV/0!
**TARSL2**	0.04761904762	0.04761904762	0.05555555556		0.7	#DIV/0!
**TAS1R1**	0	0	0.05555555556		0	#DIV/0!
**TAS1R2**	0.04761904762	0.04761904762	0.1111111111		0.325	#DIV/0!
**TAS1R3**	0	0	0.1111111111		0	#DIV/0!
**TAS2R1**	0	0	0.05555555556		0	#DIV/0!
**TAS2R10**	0.04761904762	0.04761904762	0.1111111111		0.325	#DIV/0!
**TAS2R13**	0.04761904762	0.04761904762	0.1111111111		0.325	#DIV/0!
**TAS2R14**	0.04761904762	0.04761904762	0.1111111111		0.325	#DIV/0!
**TAS2R16**	0.04761904762	0.04761904762	0.05555555556		0.7	#DIV/0!
**TAS2R19**	0.04761904762	0.04761904762	0.1111111111		0.325	#DIV/0!
**TAS2R20**	0.04761904762	0.04761904762	0.1111111111		0.325	#DIV/0!
**TAS2R3**	0.04761904762	0.04761904762	0.05555555556		0.7	#DIV/0!
**TAS2R30**	0.04761904762	0.04761904762	0.1111111111		0.325	#DIV/0!
**TAS2R31**	0.04761904762	0.04761904762	0.1111111111		0.325	#DIV/0!
**TAS2R38**	0.04761904762	0.04761904762	0.05555555556		0.7	#DIV/0!
**TAS2R39**	0.04761904762	0.04761904762	0.05555555556		0.7	#DIV/0!
**TAS2R4**	0.04761904762	0.04761904762	0.05555555556		0.7	#DIV/0!
**TAS2R40**	0.04761904762	0.04761904762	0.05555555556		0.7	#DIV/0!
**TAS2R41**	0.04761904762	0.04761904762	0.05555555556		0.7	#DIV/0!
**TAS2R42**	0.04761904762	0.04761904762	0.1111111111		0.325	#DIV/0!

TAS2R43	0.04761904762	0.04761904762	0.1111111111		0.325	#DIV/0!
TAS2R46	0.04761904762	0.04761904762	0.1111111111		0.325	#DIV/0!
TAS2R5	0.04761904762	0.04761904762	0.05555555556		0.7	#DIV/0!
TAS2R50	0.04761904762	0.04761904762	0.1111111111		0.325	#DIV/0!
TAS2R60	0.04761904762	0.04761904762	0.05555555556		0.7	#DIV/0!
TAS2R7	0.04761904762	0.04761904762	0.1111111111		0.325	#DIV/0!
TAS2R8	0.04761904762	0.04761904762	0.1111111111		0.325	#DIV/0!
TAS2R9	0.04761904762	0.04761904762	0.1111111111		0.325	#DIV/0!
TASOR	0.04761904762	0.04761904762	0		inf	#DIV/0!
TASOR2	0.09523809524	0.09523809524	0.1666666667		0.4210526316	#DIV/0!
TASP1	0.1904761905	0.1904761905	0.1111111111		1.529411765	#DIV/0!
TAT	0.04761904762	0.04761904762	0		inf	#DIV/0!
TAT-AS1	0.04761904762	0.04761904762	0		inf	#DIV/0!
TATDN1	0.1428571429	0.1428571429	0.1111111111		1.083333333	#DIV/0!
TATDN2	0.09523809524	0.09523809524	0		inf	#DIV/0!
TATDN3	0.09523809524	0.09523809524	0		inf	#DIV/0!
TAX1BP	0.04761904762	0.04761904762	0.2222222222		0.1375	#DIV/0!
TAX1BP3	0	0	0.1111111111		0	#DIV/0!
TBATA	0.09523809524	0.09523809524	0		inf	#DIV/0!
BC1D10	0.04761904762	0.04761904762	0.1111111111		0.325	#DIV/0!
BC1D10	0.04761904762	0.04761904762	0		inf	#DIV/0!
TBC1D12	0.1428571429	0.1428571429	0		inf	#DIV/0!
TBC1D13	0.09523809524	0.09523809524	0.05555555556		1.473684211	#DIV/0!
TBC1D15	0.04761904762	0.04761904762	0.1111111111		0.325	#DIV/0!
TBC1D16	0.04761904762	0.04761904762	0		inf	#DIV/0!
TBC1D17	0.09523809524	0.09523809524	0.1111111111		0.6842105263	#DIV/0!
TBC1D2	0.04761904762	0.04761904762	0		inf	#DIV/0!
TBC1D20	0.1904761905	0.1904761905	0.05555555556		3.294117647	#DIV/0!
TBC1D21	0.04761904762	0.04761904762	0.05555555556		0.7	#DIV/0!
BC1D22	0.04761904762	0.04761904762	0.1666666667		0.2	#DIV/0!
C1D22A-	0.04761904762	0.04761904762	0.1111111111		0.325	#DIV/0!
BC1D22	0.04761904762	0.04761904762	0.05555555556		0.7	#DIV/0!
TBC1D23	0.09523809524	0.09523809524	0.1111111111		0.6842105263	#DIV/0!
TBC1D24	0.09523809524	0.09523809524	0.2222222222		0.2894736842	#DIV/0!
TBC1D26	0.04761904762	0.04761904762	0.05555555556		0.7	#DIV/0!
BC1D27	0.04761904762	0.04761904762	0.05555555556		0.7	#DIV/0!
TBC1D28	0.04761904762	0.04761904762	0.1111111111		0.325	#DIV/0!
TBC1D2B	0.04761904762	0.04761904762	0.05555555556		0.7	#DIV/0!
TBC1D30	0.1428571429	0.1428571429	0.05555555556		2.333333333	#DIV/0!
TBC1D31	0.1428571429	0.1428571429	0.1111111111		1.083333333	#DIV/0!
TBC1D3B	0.09523809524	0.09523809524	0.05555555556		1.473684211	#DIV/0!
TBC1D3D	0.09523809524	0.09523809524	0.05555555556		1.473684211	#DIV/0!
TBC1D3G	0.09523809524	0.09523809524	0.05555555556		1.473684211	#DIV/0!
TBC1D3H	0.09523809524	0.09523809524	0.05555555556		1.473684211	#DIV/0!
D3P1-DH	0.1428571429	0.1428571429	0		inf	#DIV/0!
BC1D3P	0.09523809524	0.09523809524	0		inf	#DIV/0!
BC1D3P	0.04761904762	0.04761904762	0.05555555556		0.7	#DIV/0!
TBC1D4	0	0	0.05555555556		0	#DIV/0!
TBC1D5	0.04761904762	0.04761904762	0		inf	#DIV/0!
TBC1D8	0	0	0.05555555556		0	#DIV/0!
C1D8-A	0	0	0.05555555556		0	#DIV/0!

**TBC1D9**	0	0	0.05555555556		0	#DIV/0!
**'BC1D9I**	0.09523809524	0.09523809524	0.05555555556		1.473684211	#DIV/0!
**TBCA**	0.04761904762	0.04761904762	0		inf	#DIV/0!
**TBCB**	0.04761904762	0.04761904762	0.05555555556		0.7	#DIV/0!
**TBCC**	0.04761904762	0.04761904762	0.05555555556		0.7	#DIV/0!
**TBCCD1**	0.09523809524	0.09523809524	0.05555555556		1.473684211	#DIV/0!
**TBCD**	0	0	0			#DIV/0!
**TBCE**	0.1428571429	0.1428571429	0.05555555556		2.333333333	#DIV/0!
**TBCK**	0	0	0.05555555556		0	#DIV/0!
**TBILA**	0.09523809524	0.09523809524	0.05555555556		1.473684211	#DIV/0!
**TBK1**	0.1428571429	0.1428571429	0.05555555556		2.333333333	#DIV/0!
**TBKBP1**	0.1428571429	0.1428571429	0		inf	#DIV/0!
**'BL1XR**	0.1904761905	0.1904761905	0.05555555556		3.294117647	#DIV/0!
**TBL2**	0.1428571429	0.1428571429	0.1111111111		1.083333333	#DIV/0!
**TBL3**	0.09523809524	0.09523809524	0.2222222222		0.2894736842	#DIV/0!
**TBPL2**	0.04761904762	0.04761904762	0		inf	#DIV/0!
**TBR1**	0	0	0.05555555556		0	#DIV/0!
**TBRG4**	0.04761904762	0.04761904762	0.2222222222		0.1375	#DIV/0!
**TBX1**	0.04761904762	0.04761904762	0.05555555556		0.7	#DIV/0!
**TBX15**	0.04761904762	0.04761904762	0.05555555556		0.7	#DIV/0!
**TBX19**	0.1428571429	0.1428571429	0		inf	#DIV/0!
**TBX2**	0.09523809524	0.09523809524	0		inf	#DIV/0!
**'BX2-AS**	0.09523809524	0.09523809524	0		inf	#DIV/0!
**TBX20**	0.04761904762	0.04761904762	0.2222222222		0.1375	#DIV/0!
**TBX21**	0.1428571429	0.1428571429	0		inf	#DIV/0!
**TBX3**	0	0	0.05555555556		0	#DIV/0!
**TBX4**	0.09523809524	0.09523809524	0		inf	#DIV/0!
**TBX5**	0	0	0.05555555556		0	#DIV/0!
**'BX5-AS**	0	0	0.05555555556		0	#DIV/0!
**TBX6**	0.04761904762	0.04761904762	0		inf	#DIV/0!
**TBXA2R**	0.09523809524	0.09523809524	0.05555555556		1.473684211	#DIV/0!
**TBXAS1**	0.04761904762	0.04761904762	0.05555555556		0.7	#DIV/0!
**TCAF1**	0.04761904762	0.04761904762	0.05555555556		0.7	#DIV/0!
**TCAF2**	0.04761904762	0.04761904762	0		inf	#DIV/0!
**'CAF2P**	0.04761904762	0.04761904762	0		inf	#DIV/0!
**TCAM1I**	0.04761904762	0.04761904762	0		inf	#DIV/0!
**TCAP**	0.09523809524	0.09523809524	0		inf	#DIV/0!
**TCEA1**	0.1428571429	0.1428571429	0.1111111111		1.083333333	#DIV/0!
**TCEA2**	0.1428571429	0.1428571429	0.2222222222		0.4583333333	#DIV/0!
**TCEA3**	0.04761904762	0.04761904762	0.1111111111		0.325	#DIV/0!
**CEANC**	0.04761904762	0.04761904762	0.05555555556		0.7	#DIV/0!
**CERG1**	0.2857142857	0.2857142857	0		inf	#DIV/0!
**ERG1L-**	0.2857142857	0.2857142857	0		inf	#DIV/0!
**TCF12**	0.04761904762	0.04761904762	0.05555555556		0.7	#DIV/0!
**TCF15**	0.1904761905	0.1904761905	0.05555555556		3.294117647	#DIV/0!
**TCF20**	0	0	0.1111111111		0	#DIV/0!
**TCF24**	0.1428571429	0.1428571429	0.1666666667		0.6666666667	#DIV/0!
**TCF25**	0.09523809524	0.09523809524	0		inf	#DIV/0!
**TCF3**	0.09523809524	0.09523809524	0.1111111111		0.6842105263	#DIV/0!
**TCF4**	0.09523809524	0.09523809524	0.05555555556		1.473684211	#DIV/0!
**'CF4-AS**	0.09523809524	0.09523809524	0.05555555556		1.473684211	#DIV/0!

**TCF7**	0.04761904762	0.04761904762	0		inf	#DIV/0!
**TCF7L1**	0	0	0.05555555556		0	#DIV/0!
**CF7L1-IT**	0	0	0.05555555556		0	#DIV/0!
**TCF7L2**	0.1428571429	0.1428571429	0.05555555556		2.333333333	#DIV/0!
**TCFL5**	0.1428571429	0.1428571429	0.2222222222		0.4583333333	#DIV/0!
**TCHH**	0.1904761905	0.1904761905	0		inf	#DIV/0!
**TCHHL1**	0.1904761905	0.1904761905	0		inf	#DIV/0!
**TCHP**	0	0	0.1111111111		0	#DIV/0!
**TCIM**	0.09523809524	0.09523809524	0.1111111111		0.6842105263	#DIV/0!
**TCN2**	0.04761904762	0.04761904762	0.1111111111		0.325	#DIV/0!
**TCP10L**	0.04761904762	0.04761904762	0.1111111111		0.325	#DIV/0!
**TCP11**	0.04761904762	0.04761904762	0		inf	#DIV/0!
**TCP11L1**	0.04761904762	0.04761904762	0		inf	#DIV/0!
**TCP11L2**	0.04761904762	0.04761904762	0.05555555556		0.7	#DIV/0!
**TCTA**	0.1428571429	0.1428571429	0		inf	#DIV/0!
**TCTE1**	0.04761904762	0.04761904762	0.05555555556		0.7	#DIV/0!
**CTEX1D**	0.04761904762	0.04761904762	0.05555555556		0.7	#DIV/0!
**CTEX1D**	0.1428571429	0.1428571429	0.05555555556		2.333333333	#DIV/0!
**CTEX1D**	0.04761904762	0.04761904762	0.05555555556		0.7	#DIV/0!
**TCTN1**	0	0	0.1111111111		0	#DIV/0!
**TCTN2**	0.04761904762	0.04761904762	0.1111111111		0.325	#DIV/0!
**TCTN3**	0.1428571429	0.1428571429	0		inf	#DIV/0!
**TDG**	0.04761904762	0.04761904762	0.05555555556		0.7	#DIV/0!
**TDH**	0.09523809524	0.09523809524	0.1111111111		0.6842105263	#DIV/0!
**TDO2**	0	0	0.1111111111		0	#DIV/0!
**TDP2**	0	0	0.05555555556		0	#DIV/0!
**TDRD1**	0.1904761905	0.1904761905	0.05555555556		3.294117647	#DIV/0!
**TDRD10**	0.1904761905	0.1904761905	0.1111111111		1.529411765	#DIV/0!
**TDRD12**	0.04761904762	0.04761904762	0.05555555556		0.7	#DIV/0!
**TDRD15**	0	0	0.05555555556		0	#DIV/0!
**TDRD5**	0.1428571429	0.1428571429	0		inf	#DIV/0!
**TDRD6**	0.04761904762	0.04761904762	0.05555555556		0.7	#DIV/0!
**TDRD7**	0.04761904762	0.04761904762	0		inf	#DIV/0!
**TDRG1**	0	0	0.05555555556		0	#DIV/0!
**TDRKH**	0.1904761905	0.1904761905	0		inf	#DIV/0!
**DRKH-A**	0.1904761905	0.1904761905	0		inf	#DIV/0!
**TDRP**	0.09523809524	0.09523809524	0.1111111111		0.6842105263	#DIV/0!
**TEAD2**	0.09523809524	0.09523809524	0.1111111111		0.6842105263	#DIV/0!
**TEAD3**	0.04761904762	0.04761904762	0.05555555556		0.7	#DIV/0!
**TEAD4**	0.04761904762	0.04761904762	0.1666666667		0.2	#DIV/0!
**TEC**	0.04761904762	0.04761904762	0.1111111111		0.325	#DIV/0!
**TECPR1**	0.04761904762	0.04761904762	0.05555555556		0.7	#DIV/0!
**TECR**	0.1428571429	0.1428571429	0.1111111111		1.083333333	#DIV/0!
**TECRL**	0.04761904762	0.04761904762	0.05555555556		0.7	#DIV/0!
**TECTB**	0.1428571429	0.1428571429	0.05555555556		2.333333333	#DIV/0!
**TEDC2**	0.09523809524	0.09523809524	0.2222222222		0.2894736842	#DIV/0!
**TEF**	0	0	0.1111111111		0	#DIV/0!
**TEFM**	0	0	0			#DIV/0!
**TEK**	0.04761904762	0.04761904762	0.05555555556		0.7	#DIV/0!
**TEKT1**	0	0	0.05555555556		0	#DIV/0!
**TEKT2**	0.04761904762	0.04761904762	0.05555555556		0.7	#DIV/0!

**TEKT3**	0.04761904762	0.04761904762	0.05555555556		0.7	#DIV/0!
**TEKT4**	0	0	0.1111111111		0	#DIV/0!
**EKT4P**	0.04761904762	0.04761904762	0		inf	#DIV/0!
**TEKT5**	0.04761904762	0.04761904762	0		inf	#DIV/0!
**TELO2**	0.09523809524	0.09523809524	0.2222222222		0.2894736842	#DIV/0!
**MN3-A**	0	0	0.05555555556		0	#DIV/0!
**TEN1**	0.04761904762	0.04761904762	0		inf	#DIV/0!
**N1-CD**	0.04761904762	0.04761904762	0		inf	#DIV/0!
**TENM2**	0	0	0.05555555556		0	#DIV/0!
**TENM3**	0	0	0.05555555556		0	#DIV/0!
**NM3-A**	0	0	0.05555555556		0	#DIV/0!
**TENT2**	0.04761904762	0.04761904762	0		inf	#DIV/0!
**TENT4A**	0	0	0.05555555556		0	#DIV/0!
**TENT4B**	0.04761904762	0.04761904762	0		inf	#DIV/0!
**TENT5B**	0.04761904762	0.04761904762	0.1111111111		0.325	#DIV/0!
**TENT5C**	0.04761904762	0.04761904762	0.05555555556		0.7	#DIV/0!
**TEP1**	0.04761904762	0.04761904762	0		inf	#DIV/0!
**TEPP**	0.04761904762	0.04761904762	0		inf	#DIV/0!
**TEPSIN**	0.04761904762	0.04761904762	0		inf	#DIV/0!
**TERB1**	0.09523809524	0.09523809524	0.05555555556		1.473684211	#DIV/0!
**TERC**	0.1904761905	0.1904761905	0.05555555556		3.294117647	#DIV/0!
**TERF1**	0.1904761905	0.1904761905	0.1666666667		0.9411764706	#DIV/0!
**TERF2**	0.1428571429	0.1428571429	0.05555555556		2.333333333	#DIV/0!
**TERF2II**	0.04761904762	0.04761904762	0		inf	#DIV/0!
**TERT**	0	0	0.1111111111		0	#DIV/0!
**TES**	0.04761904762	0.04761904762	0.1111111111		0.325	#DIV/0!
**TESC**	0	0	0.05555555556		0	#DIV/0!
**ESC-AS**	0	0	0.05555555556		0	#DIV/0!
**TESK1**	0.09523809524	0.09523809524	0.05555555556		1.473684211	#DIV/0!
**TESK2**	0.04761904762	0.04761904762	0.05555555556		0.7	#DIV/0!
**TESPA1**	0.04761904762	0.04761904762	0.05555555556		0.7	#DIV/0!
**TET1**	0.1904761905	0.1904761905	0.05555555556		3.294117647	#DIV/0!
**TET2**	0	0	0.05555555556		0	#DIV/0!
**ET2-AS**	0	0	0.05555555556		0	#DIV/0!
**TET3**	0	0	0.05555555556		0	#DIV/0!
**TEX10**	0.04761904762	0.04761904762	0		inf	#DIV/0!
**TEX101**	0.04761904762	0.04761904762	0.05555555556		0.7	#DIV/0!
**TEX14**	0.1428571429	0.1428571429	0		inf	#DIV/0!
**TEX15**	0.04761904762	0.04761904762	0.1111111111		0.325	#DIV/0!
**TEX19**	0.04761904762	0.04761904762	0		inf	#DIV/0!
**TEX2**	0.04761904762	0.04761904762	0		inf	#DIV/0!
**TEX21P**	0.09523809524	0.09523809524	0		inf	#DIV/0!
**TEX261**	0	0	0.05555555556		0	#DIV/0!
**TEX264**	0.04761904762	0.04761904762	0		inf	#DIV/0!
**TEX33**	0	0	0.1111111111		0	#DIV/0!
**TEX35**	0.1428571429	0.1428571429	0		inf	#DIV/0!
**TEX36**	0.2380952381	0.2380952381	0.05555555556		4.375	#DIV/0!
**EX36-AS**	0.2380952381	0.2380952381	0.05555555556		4.375	#DIV/0!
**TEX37**	0	0	0.05555555556		0	#DIV/0!
**TEX38**	0.04761904762	0.04761904762	0.05555555556		0.7	#DIV/0!
**TEX41**	0	0	0.05555555556		0	#DIV/0!

**TEX43**	0.04761904762	0.04761904762	0		inf	#DIV/0!
**TEX44**	0	0	0.05555555556		0	#DIV/0!
**TEX45**	0.1428571429	0.1428571429	0.05555555556		2.333333333	#DIV/0!
**TEX46**	0.04761904762	0.04761904762	0.1111111111		0.325	#DIV/0!
**TEX47**	0.09523809524	0.09523809524	0.1111111111		0.6842105263	#DIV/0!
**TEX48**	0.04761904762	0.04761904762	0.05555555556		0.7	#DIV/0!
**TEX49**	0.04761904762	0.04761904762	0.05555555556		0.7	#DIV/0!
**TEX50**	0.1428571429	0.1428571429	0		inf	#DIV/0!
**TEX51**	0	0	0.05555555556		0	#DIV/0!
**TEX52**	0.04761904762	0.04761904762	0.1666666667		0.2	#DIV/0!
**TEX53**	0.04761904762	0.04761904762	0.05555555556		0.7	#DIV/0!
**TEX55**	0.09523809524	0.09523809524	0.05555555556		1.473684211	#DIV/0!
**TEX9**	0.04761904762	0.04761904762	0.05555555556		0.7	#DIV/0!
**TF**	0.1428571429	0.1428571429	0.05555555556		2.333333333	#DIV/0!
**TFAM**	0.09523809524	0.09523809524	0.05555555556		1.473684211	#DIV/0!
**TFAMP1**	0.04761904762	0.04761904762	0.1666666667		0.2	#DIV/0!
**TFAP2B**	0.04761904762	0.04761904762	0		inf	#DIV/0!
**TFAP2C**	0.09523809524	0.09523809524	0.1666666667		0.4210526316	#DIV/0!
**TFAP2D**	0.04761904762	0.04761904762	0		inf	#DIV/0!
**TFAP2E**	0.04761904762	0.04761904762	0.05555555556		0.7	#DIV/0!
**TFAP4**	0.04761904762	0.04761904762	0		inf	#DIV/0!
**TFB2M**	0.1428571429	0.1428571429	0.05555555556		2.333333333	#DIV/0!
**TFCP2**	0.09523809524	0.09523809524	0.05555555556		1.473684211	#DIV/0!
**TFCP2L1**	0	0	0.05555555556		0	#DIV/0!
**TFDP2**	0.1904761905	0.1904761905	0.05555555556		3.294117647	#DIV/0!
**TFEB**	0.04761904762	0.04761904762	0.05555555556		0.7	#DIV/0!
**TFEC**	0.04761904762	0.04761904762	0.1111111111		0.325	#DIV/0!
**TFF1**	0.04761904762	0.04761904762	0.1111111111		0.325	#DIV/0!
**TFF2**	0.04761904762	0.04761904762	0.1111111111		0.325	#DIV/0!
**TFF3**	0.04761904762	0.04761904762	0.1111111111		0.325	#DIV/0!
**TFG**	0.09523809524	0.09523809524	0.05555555556		1.473684211	#DIV/0!
**TFIP11**	0.04761904762	0.04761904762	0.05555555556		0.7	#DIV/0!
**TFPI**	0	0	0.05555555556		0	#DIV/0!
**TFPI2**	0.04761904762	0.04761904762	0.1111111111		0.325	#DIV/0!
**TFPT**	0.09523809524	0.09523809524	0.05555555556		1.473684211	#DIV/0!
**TFR2**	0.04761904762	0.04761904762	0.05555555556		0.7	#DIV/0!
**TFRC**	0.1428571429	0.1428571429	0.05555555556		2.333333333	#DIV/0!
**TG**	0.04761904762	0.04761904762	0.2222222222		0.1375	#DIV/0!
**TGFA**	0	0	0.05555555556		0	#DIV/0!
**TGFA-IT**	0	0	0.05555555556		0	#DIV/0!
**TGFB1**	0.04761904762	0.04761904762	0.05555555556		0.7	#DIV/0!
**TGFB1I1**	0.04761904762	0.04761904762	0		inf	#DIV/0!
**TGFB2**	0.1428571429	0.1428571429	0.05555555556		2.333333333	#DIV/0!
**GFB2-AS**	0.1428571429	0.1428571429	0.05555555556		2.333333333	#DIV/0!
**GFB2-OT**	0.1428571429	0.1428571429	0.05555555556		2.333333333	#DIV/0!
**TGFBI**	0.04761904762	0.04761904762	0		inf	#DIV/0!
**TGFBR1**	0.04761904762	0.04761904762	0		inf	#DIV/0!
**TGFBR2**	0.04761904762	0.04761904762	0		inf	#DIV/0!
**TGFBR3**	0.04761904762	0.04761904762	0.05555555556		0.7	#DIV/0!
**TGFBR3L**	0.1428571429	0.1428571429	0.05555555556		2.333333333	#DIV/0!
**GFBRAP**	0	0	0.05555555556		0	#DIV/0!

**TGIF1**	0.09523809524	0.09523809524	0		inf	#DIV/0!
**TGIF2**	0.09523809524	0.09523809524	0.1111111111		0.6842105263	#DIV/0!
**IF2-RAB**	0.09523809524	0.09523809524	0.1111111111		0.6842105263	#DIV/0!
**TGM2**	0.1428571429	0.1428571429	0.1666666667		0.6666666667	#DIV/0!
**TGM3**	0.1904761905	0.1904761905	0.05555555556		3.294117647	#DIV/0!
**TGM6**	0.1904761905	0.1904761905	0.05555555556		3.294117647	#DIV/0!
**TGOLN2**	0	0	0.05555555556		0	#DIV/0!
**TGS1**	0.1428571429	0.1428571429	0.1111111111		1.083333333	#DIV/0!
**TH**	0.04761904762	0.04761904762	0		inf	#DIV/0!
**H2LCR**	0.04761904762	0.04761904762	0		inf	#DIV/0!
**THADA**	0	0	0.05555555556		0	#DIV/0!
**THAP1**	0.09523809524	0.09523809524	0.1666666667		0.4210526316	#DIV/0!
**THAP10**	0.04761904762	0.04761904762	0.05555555556		0.7	#DIV/0!
**THAP11**	0.1428571429	0.1428571429	0.05555555556		2.333333333	#DIV/0!
**THAP2**	0.04761904762	0.04761904762	0.1111111111		0.325	#DIV/0!
**THAP3**	0	0	0.05555555556		0	#DIV/0!
**THAP4**	0	0	0.05555555556		0	#DIV/0!
**THAP5**	0.04761904762	0.04761904762	0.05555555556		0.7	#DIV/0!
**THAP6**	0.04761904762	0.04761904762	0.05555555556		0.7	#DIV/0!
**THAP7**	0.04761904762	0.04761904762	0.05555555556		0.7	#DIV/0!
**IAP7-AS**	0.04761904762	0.04761904762	0.05555555556		0.7	#DIV/0!
**THAP8**	0.04761904762	0.04761904762	0.05555555556		0.7	#DIV/0!
**THAP9**	0	0	0.05555555556		0	#DIV/0!
**IAP9-AS**	0	0	0.05555555556		0	#DIV/0!
**THBD**	0.1904761905	0.1904761905	0.05555555556		3.294117647	#DIV/0!
**THBS1**	0	0	0			#DIV/0!
**THBS3**	0.1904761905	0.1904761905	0.1111111111		1.529411765	#DIV/0!
**THBS4**	0.04761904762	0.04761904762	0		inf	#DIV/0!
**HCAT15**	0.04761904762	0.04761904762	0.1111111111		0.325	#DIV/0!
**HCAT15**	0.1428571429	0.1428571429	0		inf	#DIV/0!
**THEG**	0.09523809524	0.09523809524	0.1111111111		0.6842105263	#DIV/0!
**THEG5**	0.04761904762	0.04761904762	0.05555555556		0.7	#DIV/0!
**THEM4**	0.1904761905	0.1904761905	0		inf	#DIV/0!
**THEM5**	0.1904761905	0.1904761905	0		inf	#DIV/0!
**THEM6**	0.04761904762	0.04761904762	0.1666666667		0.2	#DIV/0!
**THEMIS**	0.04761904762	0.04761904762	0.1111111111		0.325	#DIV/0!
**THG1L**	0.04761904762	0.04761904762	0.05555555556		0.7	#DIV/0!
**THNSL1**	0.09523809524	0.09523809524	0.1111111111		0.6842105263	#DIV/0!
**THNSL2**	0	0	0.05555555556		0	#DIV/0!
**THOC1**	0.09523809524	0.09523809524	0		inf	#DIV/0!
**THOC3**	0.04761904762	0.04761904762	0.05555555556		0.7	#DIV/0!
**THOC5**	0.04761904762	0.04761904762	0.1111111111		0.325	#DIV/0!
**THOC6**	0.04761904762	0.04761904762	0.1111111111		0.325	#DIV/0!
**THOC7**	0.04761904762	0.04761904762	0		inf	#DIV/0!
**IOC7-AS**	0.04761904762	0.04761904762	0		inf	#DIV/0!
**THOP1**	0.09523809524	0.09523809524	0.05555555556		1.473684211	#DIV/0!
**HORLN**	0	0	0.05555555556		0	#DIV/0!
**THPO**	0.1428571429	0.1428571429	0.05555555556		2.333333333	#DIV/0!
**THRA**	0.09523809524	0.09523809524	0		inf	#DIV/0!
**IRA1/BT**	0.1428571429	0.1428571429	0		inf	#DIV/0!
**THRAP3**	0.04761904762	0.04761904762	0.05555555556		0.7	#DIV/0!

THRB	0.04761904762	0.04761904762	0		inf	#DIV/0!
HRB-AS	0.04761904762	0.04761904762	0		inf	#DIV/0!
THRIL	0.04761904762	0.04761904762	0.05555555556		0.7	#DIV/0!
THSD4	0.04761904762	0.04761904762	0.05555555556		0.7	#DIV/0!
HSD4-AS	0.04761904762	0.04761904762	0.05555555556		0.7	#DIV/0!
HSD4-AS	0.04761904762	0.04761904762	0.05555555556		0.7	#DIV/0!
THSD7A	0.04761904762	0.04761904762	0.2222222222		0.1375	#DIV/0!
THSD7B	0	0	0.05555555556		0	#DIV/0!
THTPA	0	0	0.05555555556		0	#DIV/0!
HUMPD	0.04761904762	0.04761904762	0		inf	#DIV/0!
HUMPD	0	0	0.05555555556		0	#DIV/0!
HUMPD	0.09523809524	0.09523809524	0		inf	#DIV/0!
UMPD3-	0.09523809524	0.09523809524	0		inf	#DIV/0!
TIA1	0	0	0.05555555556		0	#DIV/0!
TIAF1	0.04761904762	0.04761904762	0.05555555556		0.7	#DIV/0!
TIAL1	0.2380952381	0.2380952381	0		inf	#DIV/0!
TIAM1	0.04761904762	0.04761904762	0.1111111111		0.325	#DIV/0!
TICAM1	0.09523809524	0.09523809524	0.05555555556		1.473684211	#DIV/0!
TICAM2	0.04761904762	0.04761904762	0		inf	#DIV/0!
TICRR	0.04761904762	0.04761904762	0.05555555556		0.7	#DIV/0!
TIE1	0.04761904762	0.04761904762	0.05555555556		0.7	#DIV/0!
TIFA	0	0	0.05555555556		0	#DIV/0!
TIFAB	0.04761904762	0.04761904762	0		inf	#DIV/0!
TIGAR	0.04761904762	0.04761904762	0.1111111111		0.325	#DIV/0!
TIGD1	0	0	0.05555555556		0	#DIV/0!
TIGD2	0	0	0.05555555556		0	#DIV/0!
TIGD4	0	0	0.05555555556		0	#DIV/0!
TIGD5	0.04761904762	0.04761904762	0.1666666667		0.2	#DIV/0!
TIGD7	0.04761904762	0.04761904762	0.1111111111		0.325	#DIV/0!
TIGIT	0.09523809524	0.09523809524	0.05555555556		1.473684211	#DIV/0!
TIMD4	0.04761904762	0.04761904762	0.05555555556		0.7	#DIV/0!
IMELES	0.1428571429	0.1428571429	0.05555555556		2.333333333	#DIV/0!
TIMM10	0.04761904762	0.04761904762	0		inf	#DIV/0!
TIMM13	0.09523809524	0.09523809524	0.05555555556		1.473684211	#DIV/0!
TIMM17	0.1428571429	0.1428571429	0		inf	#DIV/0!
TIMM22	0	0	0.1111111111		0	#DIV/0!
TIMM23	0.04761904762	0.04761904762	0.05555555556		0.7	#DIV/0!
TIMM23	0.04761904762	0.04761904762	0.05555555556		0.7	#DIV/0!
M23B-A	0.04761904762	0.04761904762	0.05555555556		0.7	#DIV/0!
TIMM29	0.1428571429	0.1428571429	0.1111111111		1.083333333	#DIV/0!
TIMM44	0.1428571429	0.1428571429	0.05555555556		2.333333333	#DIV/0!
TIMM50	0.04761904762	0.04761904762	0.05555555556		0.7	#DIV/0!
TIMM9	0.04761904762	0.04761904762	0		inf	#DIV/0!
IMMDC	0.09523809524	0.09523809524	0.05555555556		1.473684211	#DIV/0!
TIMP2	0.04761904762	0.04761904762	0		inf	#DIV/0!
TIMP3	0.04761904762	0.04761904762	0.1666666667		0.2	#DIV/0!
TIMP4	0.09523809524	0.09523809524	0		inf	#DIV/0!
TINAG	0.04761904762	0.04761904762	0		inf	#DIV/0!
TINCR	0.1428571429	0.1428571429	0.05555555556		2.333333333	#DIV/0!
TIPARP	0.1428571429	0.1428571429	0.05555555556		2.333333333	#DIV/0!
PARP-A	0.1428571429	0.1428571429	0.05555555556		2.333333333	#DIV/0!

**TIPIN**	0.04761904762	0.04761904762	0.05555555556		0.7	#DIV/0!
**TIPRL**	0.1428571429	0.1428571429	0		inf	#DIV/0!
**TJAP1**	0.04761904762	0.04761904762	0.05555555556		0.7	#DIV/0!
**TJP2**	0	0	0.05555555556		0	#DIV/0!
**TJP3**	0.09523809524	0.09523809524	0.05555555556		1.473684211	#DIV/0!
**TK1**	0.04761904762	0.04761904762	0		inf	#DIV/0!
**TK2**	0.04761904762	0.04761904762	0		inf	#DIV/0!
**TKT**	0.04761904762	0.04761904762	0		inf	#DIV/0!
**TKTL2**	0	0	0.05555555556		0	#DIV/0!
**TLCD1**	0.04761904762	0.04761904762	0.05555555556		0.7	#DIV/0!
**TLCD2**	0	0	0.1111111111		0	#DIV/0!
**TLDC2**	0.09523809524	0.09523809524	0.1111111111		0.6842105263	#DIV/0!
**TLE1**	0	0	0.05555555556		0	#DIV/0!
**TLE2**	0.09523809524	0.09523809524	0.05555555556		1.473684211	#DIV/0!
**TLE3**	0.04761904762	0.04761904762	0.05555555556		0.7	#DIV/0!
**TLE4**	0	0	0.05555555556		0	#DIV/0!
**TLE5**	0.09523809524	0.09523809524	0.05555555556		1.473684211	#DIV/0!
**TLE6**	0.09523809524	0.09523809524	0.05555555556		1.473684211	#DIV/0!
**TLK1**	0	0	0.05555555556		0	#DIV/0!
**TLK2**	0.04761904762	0.04761904762	0		inf	#DIV/0!
**TLL1**	0	0	0.1111111111		0	#DIV/0!
**TLL2**	0.1428571429	0.1428571429	0		inf	#DIV/0!
**TLN1**	0.09523809524	0.09523809524	0.05555555556		1.473684211	#DIV/0!
**TLN2**	0.04761904762	0.04761904762	0.05555555556		0.7	#DIV/0!
**TLNRD1**	0.04761904762	0.04761904762	0.05555555556		0.7	#DIV/0!
**TLR1**	0.04761904762	0.04761904762	0		inf	#DIV/0!
**TLR10**	0.04761904762	0.04761904762	0		inf	#DIV/0!
**TLR2**	0	0	0.05555555556		0	#DIV/0!
**TLR3**	0	0	0.05555555556		0	#DIV/0!
**TLR4**	0.04761904762	0.04761904762	0		inf	#DIV/0!
**TLR5**	0.1428571429	0.1428571429	0.05555555556		2.333333333	#DIV/0!
**TLR6**	0.1428571429	0.1428571429	0.05555555556		2.333333333	#DIV/0!
**TLR9**	0.04761904762	0.04761904762	0		inf	#DIV/0!
**TLX1**	0.1428571429	0.1428571429	0		inf	#DIV/0!
**TLX1NB**	0.1428571429	0.1428571429	0		inf	#DIV/0!
**TLX2**	0	0	0.05555555556		0	#DIV/0!
**TLX3**	0	0	0.05555555556		0	#DIV/0!
**TM2D1**	0.04761904762	0.04761904762	0.05555555556		0.7	#DIV/0!
**TM2D2**	0.09523809524	0.09523809524	0.1111111111		0.6842105263	#DIV/0!
**TM2D3**	0.04761904762	0.04761904762	0.05555555556		0.7	#DIV/0!
**TM4SF1**	0.1428571429	0.1428571429	0.1111111111		1.083333333	#DIV/0!
**M4SF1-A**	0.1428571429	0.1428571429	0.1111111111		1.083333333	#DIV/0!
**TM4SF18**	0.1428571429	0.1428571429	0.1111111111		1.083333333	#DIV/0!
**TM4SF19**	0.1428571429	0.1428571429	0.05555555556		2.333333333	#DIV/0!
**M4SF19-A**	0.1428571429	0.1428571429	0.05555555556		2.333333333	#DIV/0!
**19-TCT**	0.1428571429	0.1428571429	0.05555555556		2.333333333	#DIV/0!
**TM4SF20**	0	0	0.05555555556		0	#DIV/0!
**TM4SF4**	0.1428571429	0.1428571429	0.1111111111		1.083333333	#DIV/0!
**TM4SF5**	0	0	0.1111111111		0	#DIV/0!
**TM6SF1**	0.04761904762	0.04761904762	0.05555555556		0.7	#DIV/0!
**TM6SF2**	0.1428571429	0.1428571429	0.1111111111		1.083333333	#DIV/0!

TM7SF3	0.04761904762	0.04761904762	0.05555555556		0.7	#DIV/0!
TM9SF3	0.1428571429	0.1428571429	0		inf	#DIV/0!
TM9SF4	0.09523809524	0.09523809524	0.1111111111		0.6842105263	#DIV/0!
TMA16	0	0	0.05555555556		0	#DIV/0!
TMA7	0.09523809524	0.09523809524	0		inf	#DIV/0!
TMBIM1	0	0	0.05555555556		0	#DIV/0!
TMBIM4	0.09523809524	0.09523809524	0.05555555556		1.473684211	#DIV/0!
TMBIM6	0.04761904762	0.04761904762	0.05555555556		0.7	#DIV/0!
MBIM7	0.04761904762	0.04761904762	0.1111111111		0.325	#DIV/0!
TMC1	0	0	0.05555555556		0	#DIV/0!
TMC2	0.1904761905	0.1904761905	0.05555555556		3.294117647	#DIV/0!
TMC3	0.04761904762	0.04761904762	0.05555555556		0.7	#DIV/0!
MC3-AS	0.04761904762	0.04761904762	0.05555555556		0.7	#DIV/0!
TMC4	0.09523809524	0.09523809524	0.05555555556		1.473684211	#DIV/0!
TMC5	0.04761904762	0.04761904762	0		inf	#DIV/0!
TMC6	0.04761904762	0.04761904762	0		inf	#DIV/0!
TMC7	0.04761904762	0.04761904762	0		inf	#DIV/0!
TMC8	0.04761904762	0.04761904762	0		inf	#DIV/0!
TMCC1	0.09523809524	0.09523809524	0.05555555556		1.473684211	#DIV/0!
MCC1-A	0.09523809524	0.09523809524	0.05555555556		1.473684211	#DIV/0!
TMCC2	0.1904761905	0.1904761905	0		inf	#DIV/0!
TMCC3	0.04761904762	0.04761904762	0.05555555556		0.7	#DIV/0!
TMCO1	0.1428571429	0.1428571429	0		inf	#DIV/0!
MCO1-A	0.1428571429	0.1428571429	0		inf	#DIV/0!
TMCO2	0.04761904762	0.04761904762	0.05555555556		0.7	#DIV/0!
TMCO4	0.04761904762	0.04761904762	0.1111111111		0.325	#DIV/0!
TMCO5A	0	0	0			#DIV/0!
TMCO6	0.04761904762	0.04761904762	0		inf	#DIV/0!
TMED1	0.1428571429	0.1428571429	0.1111111111		1.083333333	#DIV/0!
MED10P	0.09523809524	0.09523809524	0.2222222222		0.2894736842	#DIV/0!
TMED11	0.04761904762	0.04761904762	0		inf	#DIV/0!
TMED2	0.04761904762	0.04761904762	0.1111111111		0.325	#DIV/0!
TMED3	0.04761904762	0.04761904762	0.05555555556		0.7	#DIV/0!
TMED4	0.04761904762	0.04761904762	0.2222222222		0.1375	#DIV/0!
TMED5	0.04761904762	0.04761904762	0.05555555556		0.7	#DIV/0!
TMED6	0.1428571429	0.1428571429	0.05555555556		2.333333333	#DIV/0!
TMED7	0.04761904762	0.04761904762	0		inf	#DIV/0!
D7-TIC	0.04761904762	0.04761904762	0		inf	#DIV/0!
TMED9	0.09523809524	0.09523809524	0.05555555556		1.473684211	#DIV/0!
TMEFF1	0.04761904762	0.04761904762	0		inf	#DIV/0!
TMEFF2	0	0	0.05555555556		0	#DIV/0!
MEM10	0.09523809524	0.09523809524	0		inf	#DIV/0!
MEM10	0.09523809524	0.09523809524	0		inf	#DIV/0!
MEM10	0	0	0.05555555556		0	#DIV/0!
MEM10	0.04761904762	0.04761904762	0		inf	#DIV/0!
MEM106	0.04761904762	0.04761904762	0		inf	#DIV/0!
MEM106	0.04761904762	0.04761904762	0.2222222222		0.1375	#DIV/0!
MEM106	0.04761904762	0.04761904762	0.05555555556		0.7	#DIV/0!
MEM10	0	0	0.05555555556		0	#DIV/0!
MEM10	0.1428571429	0.1428571429	0.05555555556		2.333333333	#DIV/0!
EM108-A	0.1428571429	0.1428571429	0.05555555556		2.333333333	#DIV/0!

TMEM11	0.09523809524	0.09523809524	0.05555555556		1.473684211	#DIV/0!
TMEM11	0.04761904762	0.04761904762	0		inf	#DIV/0!
TMEM11	0.09523809524	0.09523809524	0		inf	#DIV/0!
TMEM11	0	0	0.1111111111		0	#DIV/0!
TMEM11	0.04761904762	0.04761904762	0.05555555556		0.7	#DIV/0!
TMEM11	0.04761904762	0.04761904762	0.05555555556		0.7	#DIV/0!
MEM120	0.1428571429	0.1428571429	0.1111111111		1.083333333	#DIV/0!
MEM120	0.04761904762	0.04761904762	0.1111111111		0.325	#DIV/0!
MEM121	0.04761904762	0.04761904762	0		inf	#DIV/0!
MEM12	0.04761904762	0.04761904762	0.05555555556		0.7	#DIV/0!
MEM12	0	0	0.1111111111		0	#DIV/0!
MEM12	0.04761904762	0.04761904762	0		inf	#DIV/0!
MEM13	0.04761904762	0.04761904762	0.05555555556		0.7	#DIV/0!
MEM13	0	0	0.05555555556		0	#DIV/0!
MEM131	0	0	0.05555555556		0	#DIV/0!
MEM132	0.09523809524	0.09523809524	0.05555555556		1.473684211	#DIV/0!
MEM132	0.09523809524	0.09523809524	0.05555555556		1.473684211	#DIV/0!
MEM132	0.09523809524	0.09523809524	0.05555555556		1.473684211	#DIV/0!
EM132D-	0.09523809524	0.09523809524	0.05555555556		1.473684211	#DIV/0!
EM132D-	0.09523809524	0.09523809524	0.05555555556		1.473684211	#DIV/0!
MEM132	0.04761904762	0.04761904762	0		inf	#DIV/0!
MEM13	0.04761904762	0.04761904762	0.05555555556		0.7	#DIV/0!
MEM14	0.04761904762	0.04761904762	0.05555555556		0.7	#DIV/0!
MEM14	0.1428571429	0.1428571429	0.05555555556		2.333333333	#DIV/0!
MEM14	0.09523809524	0.09523809524	0.1111111111		0.6842105263	#DIV/0!
MEM14	0	0	0.05555555556		0	#DIV/0!
MEM14	0.04761904762	0.04761904762	0.05555555556		0.7	#DIV/0!
MEM14	0.04761904762	0.04761904762	0.05555555556		0.7	#DIV/0!
EM147-A	0.04761904762	0.04761904762	0.05555555556		0.7	#DIV/0!
MEM14	0.04761904762	0.04761904762	0		inf	#DIV/0!
MEM14B	0.1904761905	0.1904761905	0.1111111111		1.529411765	#DIV/0!
MEM150	0	0	0.05555555556		0	#DIV/0!
MEM150	0.09523809524	0.09523809524	0.05555555556		1.473684211	#DIV/0!
MEM150	0	0	0.05555555556		0	#DIV/0!
MEM151	0.04761904762	0.04761904762	0.05555555556		0.7	#DIV/0!
MEM15	0	0	0.05555555556		0	#DIV/0!
MEM15	0	0	0.05555555556		0	#DIV/0!
MEM15	0.1904761905	0.1904761905	0.05555555556		3.294117647	#DIV/0!
MEM15	0.04761904762	0.04761904762	0		inf	#DIV/0!
MEM16	0.09523809524	0.09523809524	0.1111111111		0.6842105263	#DIV/0!
MEM161	0.1428571429	0.1428571429	0.1111111111		1.083333333	#DIV/0!
MEM161	0.04761904762	0.04761904762	0		inf	#DIV/0!
EM161B-	0.04761904762	0.04761904762	0		inf	#DIV/0!
MEM16	0	0	0.05555555556		0	#DIV/0!
MEM16	0.1904761905	0.1904761905	0.05555555556		3.294117647	#DIV/0!
MEM167	0.04761904762	0.04761904762	0.05555555556		0.7	#DIV/0!
MEM167	0.04761904762	0.04761904762	0.05555555556		0.7	#DIV/0!
MEM16	0.04761904762	0.04761904762	0.05555555556		0.7	#DIV/0!
MEM16	0	0	0.05555555556		0	#DIV/0!
TMEM17	0	0	0.05555555556		0	#DIV/0!
MEM170	0.04761904762	0.04761904762	0		inf	#DIV/0!

MEM170	0.04761904762	0.04761904762	0		inf	#DIV/0!
MEM17	0	0	0.05555555556		0	#DIV/0!
MEM17	0.04761904762	0.04761904762	0		inf	#DIV/0!
MEM17	0	0	0.05555555556		0	#DIV/0!
MEM17	0.04761904762	0.04761904762	0		inf	#DIV/0!
MEM176	0.09523809524	0.09523809524	0.05555555556		1.473684211	#DIV/0!
MEM176	0.09523809524	0.09523809524	0.05555555556		1.473684211	#DIV/0!
MEM17	0	0	0.05555555556		0	#DIV/0!
MEM178	0	0	0.05555555556		0	#DIV/0!
MEM178	0.04761904762	0.04761904762	0.1111111111		0.325	#DIV/0!
TMEM18	0	0	0.05555555556		0	#DIV/0!
MEM18	0	0	0.05555555556		0	#DIV/0!
MEM183	0.1428571429	0.1428571429	0		inf	#DIV/0!
MEM183	0.1904761905	0.1904761905	0.1111111111		1.529411765	#DIV/0!
MEM184	0.04761904762	0.04761904762	0.1666666667		0.2	#DIV/0!
MEM184	0.04761904762	0.04761904762	0.1111111111		0.325	#DIV/0!
MEM184	0	0	0.05555555556		0	#DIV/0!
MEM185	0	0	0.05555555556		0	#DIV/0!
MEM18	0.09523809524	0.09523809524	0		inf	#DIV/0!
MEM18	0.09523809524	0.09523809524	0.1666666667		0.4210526316	#DIV/0!
M189-UE	0.09523809524	0.09523809524	0.1666666667		0.4210526316	#DIV/0!
TMEM19	0.04761904762	0.04761904762	0.1111111111		0.325	#DIV/0!
MEM19	0.09523809524	0.09523809524	0.05555555556		1.473684211	#DIV/0!
MEM191	0.04761904762	0.04761904762	0.05555555556		0.7	#DIV/0!
MEM191	0.04761904762	0.04761904762	0.05555555556		0.7	#DIV/0!
MEM191	0.04761904762	0.04761904762	0.05555555556		0.7	#DIV/0!
MEM19	0.04761904762	0.04761904762	0.05555555556		0.7	#DIV/0!
MEM19	0.04761904762	0.04761904762	0.2222222222		0.1375	#DIV/0!
MEM19	0	0	0.05555555556		0	#DIV/0!
MEM198	0.04761904762	0.04761904762	0.05555555556		0.7	#DIV/0!
MEM19	0.04761904762	0.04761904762	0.05555555556		0.7	#DIV/0!
MEM200	0.04761904762	0.04761904762	0.1111111111		0.325	#DIV/0!
MEM200	0.09523809524	0.09523809524	0		inf	#DIV/0!
MEM20	0	0	0.05555555556		0	#DIV/0!
MEM20	0.04761904762	0.04761904762	0.05555555556		0.7	#DIV/0!
EM202-/	0.04761904762	0.04761904762	0.05555555556		0.7	#DIV/0!
MEM20	0.1428571429	0.1428571429	0.05555555556		2.333333333	#DIV/0!
MEM20	0.09523809524	0.09523809524	0.2222222222		0.2894736842	#DIV/0!
MEM20	0.09523809524	0.09523809524	0		inf	#DIV/0!
MEM20	0.09523809524	0.09523809524	0.1111111111		0.6842105263	#DIV/0!
MEM20	0.1428571429	0.1428571429	0.05555555556		2.333333333	#DIV/0!
MEM20	0.04761904762	0.04761904762	0.05555555556		0.7	#DIV/0!
MEM21	0.1428571429	0.1428571429	0.05555555556		2.333333333	#DIV/0!
TMEM21	0.04761904762	0.04761904762	0.05555555556		0.7	#DIV/0!
MEM21	0.1904761905	0.1904761905	0.1111111111		1.529411765	#DIV/0!
EM212-/	0.1904761905	0.1904761905	0.1111111111		1.529411765	#DIV/0!
MEM21	0.04761904762	0.04761904762	0.05555555556		0.7	#DIV/0!
MEM21	0.09523809524	0.09523809524	0.05555555556		1.473684211	#DIV/0!
MEM21	0.04761904762	0.04761904762	0.05555555556		0.7	#DIV/0!
MEM21	0.04761904762	0.04761904762	0		inf	#DIV/0!
MEM22	0.04761904762	0.04761904762	0.05555555556		0.7	#DIV/0!

**EM220-**	0.04761904762	0.04761904762	0.05555555556		0.7	#DIV/0!
**MEM22**	0.04761904762	0.04761904762	0.1111111111		0.325	#DIV/0!
**MEM225**	0.04761904762	0.04761904762	0.05555555556		0.7	#DIV/0!
**MEM229**	0.04761904762	0.04761904762	0.05555555556		0.7	#DIV/0!
**MEM23**	0.1904761905	0.1904761905	0.05555555556		3.294117647	#DIV/0!
**MEM23**	0.04761904762	0.04761904762	0		inf	#DIV/0!
**MEM23**	0.04761904762	0.04761904762	0		inf	#DIV/0!
**MEM23**	0	0	0.05555555556		0	#DIV/0!
**MEM23**	0.04761904762	0.04761904762	0.1111111111		0.325	#DIV/0!
**MEM23**	0.04761904762	0.04761904762	0		inf	#DIV/0!
**MEM23**	0.09523809524	0.09523809524	0.1111111111		0.6842105263	#DIV/0!
**MEM23**	0	0	0.05555555556		0	#DIV/0!
**MEM23**	0.09523809524	0.09523809524	0.05555555556		1.473684211	#DIV/0!
**MEM238**	0.04761904762	0.04761904762	0.05555555556		0.7	#DIV/0!
**MEM23**	0.1904761905	0.1904761905	0.05555555556		3.294117647	#DIV/0!
**MEM24**	0	0	0.1111111111		0	#DIV/0!
**MEM24**	0.04761904762	0.04761904762	0.05555555556		0.7	#DIV/0!
**MEM24**	0.09523809524	0.09523809524	0.05555555556		1.473684211	#DIV/0!
**MEM24**	0.04761904762	0.04761904762	0.05555555556		0.7	#DIV/0!
**MEM24**	0.04761904762	0.04761904762	0		inf	#DIV/0!
**EM246-**	0.04761904762	0.04761904762	0		inf	#DIV/0!
**MEM24**	0	0	0.05555555556		0	#DIV/0!
**MEM24**	0.04761904762	0.04761904762	0.1111111111		0.325	#DIV/0!
**MEM24**	0.04761904762	0.04761904762	0.1666666667		0.2	#DIV/0!
**MEM25**	0.1428571429	0.1428571429	0.05555555556		2.333333333	#DIV/0!
**MEM25**	0	0	0.05555555556		0	#DIV/0!
**MEM25**	0.1428571429	0.1428571429	0		inf	#DIV/0!
**EM254-**	0.1428571429	0.1428571429	0		inf	#DIV/0!
**MEM25**	0	0	0.05555555556		0	#DIV/0!
**M256-PL**	0	0	0.05555555556		0	#DIV/0!
**MEM25**	0.09523809524	0.09523809524	0.1111111111		0.6842105263	#DIV/0!
**TMEM2**	0.04761904762	0.04761904762	0.1111111111		0.325	#DIV/0!
**IEM26-A**	0.04761904762	0.04761904762	0.1111111111		0.325	#DIV/0!
**MEM26**	0.04761904762	0.04761904762	0		inf	#DIV/0!
**MEM26**	0.04761904762	0.04761904762	0.05555555556		0.7	#DIV/0!
**MEM26**	0.04761904762	0.04761904762	0		inf	#DIV/0!
**MEM26**	0.04761904762	0.04761904762	0.05555555556		0.7	#DIV/0!
**MEM26**	0.04761904762	0.04761904762	0.05555555556		0.7	#DIV/0!
**MEM26**	0.04761904762	0.04761904762	0.05555555556		0.7	#DIV/0!
**MEM26**	0.04761904762	0.04761904762	0.05555555556		0.7	#DIV/0!
**MEM27**	0.1428571429	0.1428571429	0.1111111111		1.083333333	#DIV/0!
**MEM27**	0.04761904762	0.04761904762	0		inf	#DIV/0!
**MEM27**	0.09523809524	0.09523809524	0.05555555556		1.473684211	#DIV/0!
**MEM274**	0	0	0.1111111111		0	#DIV/0!
**MEM27**	0.04761904762	0.04761904762	0.05555555556		0.7	#DIV/0!
**MEM30C**	0.09523809524	0.09523809524	0.1111111111		0.6842105263	#DIV/0!
**TMEM3**	0.04761904762	0.04761904762	0		inf	#DIV/0!
**MEM35**	0.04761904762	0.04761904762	0.05555555556		0.7	#DIV/0!
**TMEM3**	0	0	0.05555555556		0	#DIV/0!
**MEM38**	0.1428571429	0.1428571429	0.1111111111		1.083333333	#DIV/0!
**MEM38**	0.04761904762	0.04761904762	0.05555555556		0.7	#DIV/0!

MEM39	0.04761904762	0.04761904762	0.1111111111		0.325	#DIV/0!
TMEM4	0.09523809524	0.09523809524	0		inf	#DIV/0!
MEM41	0.1428571429	0.1428571429	0.05555555556		2.333333333	#DIV/0!
MEM41	0.04761904762	0.04761904762	0.05555555556		0.7	#DIV/0!
TMEM4	0.04761904762	0.04761904762	0		inf	#DIV/0!
TMEM4	0.09523809524	0.09523809524	0.05555555556		1.473684211	#DIV/0!
IEM44-A	0.09523809524	0.09523809524	0.05555555556		1.473684211	#DIV/0!
MEM45	0.09523809524	0.09523809524	0.05555555556		1.473684211	#DIV/0!
MEM50	0.04761904762	0.04761904762	0.1111111111		0.325	#DIV/0!
MEM50	0.04761904762	0.04761904762	0.1111111111		0.325	#DIV/0!
TMEM5	0.04761904762	0.04761904762	0.1111111111		0.325	#DIV/0!
IEM51-A	0.04761904762	0.04761904762	0.1111111111		0.325	#DIV/0!
TMEM5	0	0	0.1111111111		0	#DIV/0!
MEM52	0.04761904762	0.04761904762	0.1111111111		0.325	#DIV/0!
TMEM5	0.04761904762	0.04761904762	0.05555555556		0.7	#DIV/0!
TMEM5	0.04761904762	0.04761904762	0.1111111111		0.325	#DIV/0!
TMEM5	0.04761904762	0.04761904762	0.05555555556		0.7	#DIV/0!
M56-RW	0.04761904762	0.04761904762	0.05555555556		0.7	#DIV/0!
TMEM5	0.04761904762	0.04761904762	0.05555555556		0.7	#DIV/0!
MEM59	0.1428571429	0.1428571429	0.1111111111		1.083333333	#DIV/0!
TMEM6	0.04761904762	0.04761904762	0.1111111111		0.325	#DIV/0!
TMEM6	0.04761904762	0.04761904762	0.05555555556		0.7	#DIV/0!
MEM63	0.1428571429	0.1428571429	0.05555555556		2.333333333	#DIV/0!
MEM63	0.04761904762	0.04761904762	0.05555555556		0.7	#DIV/0!
TMEM6	0.1904761905	0.1904761905	0.1111111111		1.529411765	#DIV/0!
TMEM6	0.1428571429	0.1428571429	0.1111111111		1.083333333	#DIV/0!
TMEM6	0.1904761905	0.1904761905	0.1111111111		1.529411765	#DIV/0!
TMEM6	0.1428571429	0.1428571429	0.1111111111		1.083333333	#DIV/0!
TMEM6	0.04761904762	0.04761904762	0.05555555556		0.7	#DIV/0!
TMEM7	0.1904761905	0.1904761905	0.1111111111		1.529411765	#DIV/0!
TMEM7	0.04761904762	0.04761904762	0.1666666667		0.2	#DIV/0!
TMEM7	0.1428571429	0.1428571429	0.05555555556		2.333333333	#DIV/0!
IEM72-A	0.1428571429	0.1428571429	0.05555555556		2.333333333	#DIV/0!
TMEM7	0.1904761905	0.1904761905	0.1111111111		1.529411765	#DIV/0!
MEM74	0.1904761905	0.1904761905	0.1111111111		1.529411765	#DIV/0!
TMEM7	0.09523809524	0.09523809524	0.1111111111		0.6842105263	#DIV/0!
TMEM8	0.04761904762	0.04761904762	0		inf	#DIV/0!
TMEM8	0.1904761905	0.1904761905	0		inf	#DIV/0!
TMEM8	0.04761904762	0.04761904762	0.1111111111		0.325	#DIV/0!
MEM86	0.09523809524	0.09523809524	0.05555555556		1.473684211	#DIV/0!
MEM87	0	0	0.05555555556		0	#DIV/0!
TMEM8	0	0	0.05555555556		0	#DIV/0!
MEM88	0	0	0.1111111111		0	#DIV/0!
TMEM8	0.09523809524	0.09523809524	0		inf	#DIV/0!
TMEM8A	0.04761904762	0.04761904762	0.2222222222		0.1375	#DIV/0!
TMEM8	0.09523809524	0.09523809524	0.05555555556		1.473684211	#DIV/0!
TMEM9	0.1428571429	0.1428571429	0		inf	#DIV/0!
TMEM9	0.04761904762	0.04761904762	0.05555555556		0.7	#DIV/0!
TMEM9	0.09523809524	0.09523809524	0		inf	#DIV/0!
IEM92-A	0.09523809524	0.09523809524	0		inf	#DIV/0!
TMEM9	0	0	0.05555555556		0	#DIV/0!

TMEM9	0.04761904762	0.04761904762	0.05555555556		0.7	#DIV/0!
TMEM9	0.04761904762	0.04761904762	0		inf	#DIV/0!
TMEM9	0.09523809524	0.09523809524	0		inf	#DIV/0!
TMF1	0.04761904762	0.04761904762	0		inf	#DIV/0!
TMIGD1	0	0	0.05555555556		0	#DIV/0!
TMIGD2	0.09523809524	0.09523809524	0.05555555556		1.473684211	#DIV/0!
TMIGD3	0.04761904762	0.04761904762	0		inf	#DIV/0!
TMOD1	0.04761904762	0.04761904762	0		inf	#DIV/0!
TMOD2	0.04761904762	0.04761904762	0.05555555556		0.7	#DIV/0!
TMOD3	0.04761904762	0.04761904762	0.05555555556		0.7	#DIV/0!
TMOD4	0.1904761905	0.1904761905	0.05555555556		3.294117647	#DIV/0!
TMPO	0.04761904762	0.04761904762	0.05555555556		0.7	#DIV/0!
MPO-AS	0.04761904762	0.04761904762	0.05555555556		0.7	#DIV/0!
IPRSS1	0.04761904762	0.04761904762	0.05555555556		0.7	#DIV/0!
IPRSS1	0.04761904762	0.04761904762	0.05555555556		0.7	#DIV/0!
PRSS11	0.04761904762	0.04761904762	0.05555555556		0.7	#DIV/0!
IPRSS1	0.04761904762	0.04761904762	0.05555555556		0.7	#DIV/0!
IPRSS1	0.04761904762	0.04761904762	0.05555555556		0.7	#DIV/0!
IPRSS1	0.04761904762	0.04761904762	0.05555555556		0.7	#DIV/0!
IPRSS11	0.04761904762	0.04761904762	0.05555555556		0.7	#DIV/0!
MPRSS1	0.09523809524	0.09523809524	0.05555555556		1.473684211	#DIV/0!
MPRSS1	0.04761904762	0.04761904762	0.05555555556		0.7	#DIV/0!
TMPRSS	0.04761904762	0.04761904762	0.1111111111		0.325	#DIV/0!
TMPRSS	0.04761904762	0.04761904762	0.1111111111		0.325	#DIV/0!
TMPRSS	0	0	0.1111111111		0	#DIV/0!
TMPRSS	0.09523809524	0.09523809524	0.05555555556		1.473684211	#DIV/0!
TMSB10	0	0	0.05555555556		0	#DIV/0!
TMTC1	0.04761904762	0.04761904762	0.05555555556		0.7	#DIV/0!
TMTC2	0.04761904762	0.04761904762	0.05555555556		0.7	#DIV/0!
TMTC3	0.04761904762	0.04761904762	0.05555555556		0.7	#DIV/0!
TMUB1	0.09523809524	0.09523809524	0.05555555556		1.473684211	#DIV/0!
TMUB2	0.09523809524	0.09523809524	0		inf	#DIV/0!
TMX1	0.04761904762	0.04761904762	0		inf	#DIV/0!
TMX4	0.1904761905	0.1904761905	0.1666666667		0.9411764706	#DIV/0!
TNC	0.04761904762	0.04761904762	0.05555555556		0.7	#DIV/0!
TNF	0.04761904762	0.04761904762	0		inf	#DIV/0!
TNFAIP1	0.04761904762	0.04761904762	0.05555555556		0.7	#DIV/0!
TNFAIP	0	0	0.05555555556		0	#DIV/0!
TNFAIP	0.04761904762	0.04761904762	0		inf	#DIV/0!
NFAIP8	0.09523809524	0.09523809524	0.05555555556		1.473684211	#DIV/0!
NFAIP8	0.1904761905	0.1904761905	0.05555555556		3.294117647	#DIV/0!
IP8L2-S	0.1904761905	0.1904761905	0.05555555556		3.294117647	#DIV/0!
NFAIP8	0.04761904762	0.04761904762	0.05555555556		0.7	#DIV/0!
NFRSF1	0.04761904762	0.04761904762	0.1111111111		0.325	#DIV/0!
NFRSF1	0.04761904762	0.04761904762	0.1111111111		0.325	#DIV/0!
NFRSF1	0.04761904762	0.04761904762	0.1111111111		0.325	#DIV/0!
NFRSF1	0.04761904762	0.04761904762	0.1111111111		0.325	#DIV/0!
NFRSF1	0.09523809524	0.09523809524	0		inf	#DIV/0!
NFRSF1	0.09523809524	0.09523809524	0.1666666667		0.4210526316	#DIV/0!
NFRSF1	0.04761904762	0.04761904762	0.1111111111		0.325	#DIV/0!
NFRSF1	0.04761904762	0.04761904762	0.05555555556		0.7	#DIV/0!

NFRSF13	0	0	0.1111111111		0	#DIV/0!
NFRSF1	0	0	0.1111111111		0	#DIV/0!
RSF14-	0	0	0.1111111111		0	#DIV/0!
NFRSF1	0.04761904762	0.04761904762	0		inf	#DIV/0!
NFRSF1	0	0	0.1111111111		0	#DIV/0!
NFRSF1	0.04761904762	0.04761904762	0.1111111111		0.325	#DIV/0!
NFRSF1	0.04761904762	0.04761904762	0.1111111111		0.325	#DIV/0!
NFRSF2	0.04761904762	0.04761904762	0.05555555556		0.7	#DIV/0!
NFRSF2	0	0	0.05555555556		0	#DIV/0!
TNFRSF4	0	0	0.1111111111		0	#DIV/0!
NFRSF6	0.1428571429	0.1428571429	0.2222222222		0.4583333333	#DIV/0!
TNFRSF	0.04761904762	0.04761904762	0.1111111111		0.325	#DIV/0!
TNFRSF	0	0	0.05555555556		0	#DIV/0!
TNFSF10	0.1904761905	0.1904761905	0.1111111111		1.529411765	#DIV/0!
TNFSF12	0	0	0.05555555556		0	#DIV/0!
F12-TN	0	0	0.05555555556		0	#DIV/0!
TNFSF13	0	0	0.05555555556		0	#DIV/0!
TNFSF14	0.1428571429	0.1428571429	0.05555555556		2.333333333	#DIV/0!
TNFSF15	0.04761904762	0.04761904762	0.05555555556		0.7	#DIV/0!
TNFSF18	0.1428571429	0.1428571429	0		inf	#DIV/0!
TNFSF4	0.1428571429	0.1428571429	0		inf	#DIV/0!
TNFSF8	0.04761904762	0.04761904762	0.05555555556		0.7	#DIV/0!
TNFSF9	0.1428571429	0.1428571429	0.05555555556		2.333333333	#DIV/0!
TNIK	0.1904761905	0.1904761905	0.05555555556		3.294117647	#DIV/0!
TNIP2	0.04761904762	0.04761904762	0		inf	#DIV/0!
TNIP3	0	0	0.05555555556		0	#DIV/0!
TNK1	0	0	0.05555555556		0	#DIV/0!
TNK2	0.1428571429	0.1428571429	0.05555555556		2.333333333	#DIV/0!
NK2-AS	0.1428571429	0.1428571429	0.05555555556		2.333333333	#DIV/0!
TNKS	0.09523809524	0.09523809524	0.1111111111		0.6842105263	#DIV/0!
TNKS2	0.1428571429	0.1428571429	0.1111111111		1.083333333	#DIV/0!
NKS2-AS	0.1428571429	0.1428571429	0.1111111111		1.083333333	#DIV/0!
TNN	0.1428571429	0.1428571429	0		inf	#DIV/0!
TNNC1	0.04761904762	0.04761904762	0		inf	#DIV/0!
TNNC2	0.1428571429	0.1428571429	0.1666666667		0.6666666667	#DIV/0!
TNNI1	0.1428571429	0.1428571429	0		inf	#DIV/0!
TNNI2	0.04761904762	0.04761904762	0		inf	#DIV/0!
TNNI3	0.09523809524	0.09523809524	0.05555555556		1.473684211	#DIV/0!
TNNI3K	0.04761904762	0.04761904762	0.05555555556		0.7	#DIV/0!
TNNT1	0.09523809524	0.09523809524	0.05555555556		1.473684211	#DIV/0!
TNNT2	0.1428571429	0.1428571429	0		inf	#DIV/0!
TNNT3	0.04761904762	0.04761904762	0		inf	#DIV/0!
TNP1	0	0	0.1111111111		0	#DIV/0!
TNP2	0.04761904762	0.04761904762	0		inf	#DIV/0!
TNPO1	0	0	0.05555555556		0	#DIV/0!
TNPO2	0.1428571429	0.1428571429	0.1111111111		1.083333333	#DIV/0!
TNPO3	0.04761904762	0.04761904762	0.05555555556		0.7	#DIV/0!
TNR	0.1428571429	0.1428571429	0		inf	#DIV/0!
TNRC17	0	0	0.05555555556		0	#DIV/0!
TNRC18	0.04761904762	0.04761904762	0.1666666667		0.2	#DIV/0!
NRC18P	0	0	0.05555555556		0	#DIV/0!

TNRC6A	0.04761904762	0.04761904762	0		inf	#DIV/0!
TNRC6B	0	0	0.1111111111		0	#DIV/0!
TNRC6C	0.04761904762	0.04761904762	0		inf	#DIV/0!
RC6C-A	0.04761904762	0.04761904762	0		inf	#DIV/0!
TNS1	0	0	0.1111111111		0	#DIV/0!
TNS2	0.04761904762	0.04761904762	0.05555555556		0.7	#DIV/0!
TNS3	0.04761904762	0.04761904762	0.2222222222		0.1375	#DIV/0!
TNS4	0.09523809524	0.09523809524	0		inf	#DIV/0!
TNXA	0.04761904762	0.04761904762	0		inf	#DIV/0!
TNXB	0.04761904762	0.04761904762	0		inf	#DIV/0!
TOB1	0.04761904762	0.04761904762	0		inf	#DIV/0!
OB1-AS	0.04761904762	0.04761904762	0		inf	#DIV/0!
TOB2	0	0	0.1111111111		0	#DIV/0!
TOE1	0.04761904762	0.04761904762	0.05555555556		0.7	#DIV/0!
GARAI	0.04761904762	0.04761904762	0		inf	#DIV/0!
GARAI	0	0	0.05555555556		0	#DIV/0!
TOLLIP	0.04761904762	0.04761904762	0		inf	#DIV/0!
LLIP-A	0.04761904762	0.04761904762	0		inf	#DIV/0!
TOM1	0	0	0.1111111111		0	#DIV/0!
TOM1L1	0.09523809524	0.09523809524	0		inf	#DIV/0!
TOM1L2	0.04761904762	0.04761904762	0.1111111111		0.325	#DIV/0!
TOMM2	0.1428571429	0.1428571429	0.05555555556		2.333333333	#DIV/0!
OMM20	0.04761904762	0.04761904762	0		inf	#DIV/0!
TOMM2	0.04761904762	0.04761904762	0.1111111111		0.325	#DIV/0!
TOMM3	0.1428571429	0.1428571429	0.1666666667		0.6666666667	#DIV/0!
TOMM4	0.09523809524	0.09523809524	0.1111111111		0.6842105263	#DIV/0!
OMM40	0.1428571429	0.1428571429	0		inf	#DIV/0!
TOMM5	0.09523809524	0.09523809524	0.05555555556		1.473684211	#DIV/0!
TOMM6	0.04761904762	0.04761904762	0.05555555556		0.7	#DIV/0!
TOMM7	0.04761904762	0.04761904762	0.1666666667		0.2	#DIV/0!
TOMM7	0.09523809524	0.09523809524	0.05555555556		1.473684211	#DIV/0!
TONSL	0.04761904762	0.04761904762	0.1666666667		0.2	#DIV/0!
NSL-A	0.04761904762	0.04761904762	0.1666666667		0.2	#DIV/0!
TOP1	0.1428571429	0.1428571429	0.1666666667		0.6666666667	#DIV/0!
TOP1MT	0.04761904762	0.04761904762	0.05555555556		0.7	#DIV/0!
TOP1P1	0.1428571429	0.1428571429	0.05555555556		2.333333333	#DIV/0!
TOP1P2	0.04761904762	0.04761904762	0.05555555556		0.7	#DIV/0!
TOP2A	0.09523809524	0.09523809524	0		inf	#DIV/0!
TOP2B	0.04761904762	0.04761904762	0		inf	#DIV/0!
TOP3A	0.04761904762	0.04761904762	0.1111111111		0.325	#DIV/0!
TOP3B	0.04761904762	0.04761904762	0.05555555556		0.7	#DIV/0!
TOPAZ1	0.04761904762	0.04761904762	0		inf	#DIV/0!
TOPBP1	0.1428571429	0.1428571429	0.05555555556		2.333333333	#DIV/0!
TOPORS	0.09523809524	0.09523809524	0.05555555556		1.473684211	#DIV/0!
TOR1A	0.09523809524	0.09523809524	0.05555555556		1.473684211	#DIV/0!
OR1AIP	0.1428571429	0.1428571429	0		inf	#DIV/0!
OR1AIP	0.1428571429	0.1428571429	0		inf	#DIV/0!
TOR1B	0.09523809524	0.09523809524	0.05555555556		1.473684211	#DIV/0!
TOR2A	0.09523809524	0.09523809524	0.05555555556		1.473684211	#DIV/0!
TOR3A	0.1428571429	0.1428571429	0		inf	#DIV/0!
TOR4A	0.1428571429	0.1428571429	0.05555555556		2.333333333	#DIV/0!

TOX	0.1428571429	0.1428571429	0.1666666667		0.6666666667	#DIV/0!
TOX2	0.1428571429	0.1428571429	0.1666666667		0.6666666667	#DIV/0!
TOX3	0.04761904762	0.04761904762	0		inf	#DIV/0!
TP53	0	0	0.05555555556		0	#DIV/0!
TP53BP2	0.1428571429	0.1428571429	0.05555555556		2.333333333	#DIV/0!
TP53I13	0	0	0.05555555556		0	#DIV/0!
TP53INP	0.1904761905	0.1904761905	0.1111111111		1.529411765	#DIV/0!
TP53INP	0.09523809524	0.09523809524	0.1111111111		0.6842105263	#DIV/0!
TP53RK	0.1428571429	0.1428571429	0.1666666667		0.6666666667	#DIV/0!
TP53TG1	0.09523809524	0.09523809524	0.05555555556		1.473684211	#DIV/0!
TP53TG3	0.1428571429	0.1428571429	0		inf	#DIV/0!
P53TG3	0.1428571429	0.1428571429	0		inf	#DIV/0!
P53TG3	0.1428571429	0.1428571429	0		inf	#DIV/0!
P53TG3	0.1428571429	0.1428571429	0		inf	#DIV/0!
P53TG3	0.1428571429	0.1428571429	0		inf	#DIV/0!
P53TG3	0.1428571429	0.1428571429	0		inf	#DIV/0!
P53TG3I	0.04761904762	0.04761904762	0		inf	#DIV/0!
TP53TG5	0.1428571429	0.1428571429	0.1666666667		0.6666666667	#DIV/0!
TP63	0.09523809524	0.09523809524	0.1111111111		0.6842105263	#DIV/0!
TP73	0	0	0.05555555556		0	#DIV/0!
TP73-AS	0	0	0.05555555556		0	#DIV/0!
TPCN1	0	0	0.05555555556		0	#DIV/0!
TPD52	0.1428571429	0.1428571429	0.1111111111		1.083333333	#DIV/0!
TPD52L2	0.1428571429	0.1428571429	0.2222222222		0.4583333333	#DIV/0!
TPD52L3	0.04761904762	0.04761904762	0.05555555556		0.7	#DIV/0!
TPGS1	0.09523809524	0.09523809524	0.1111111111		0.6842105263	#DIV/0!
TPGS2	0.04761904762	0.04761904762	0		inf	#DIV/0!
TPH2	0.04761904762	0.04761904762	0.05555555556		0.7	#DIV/0!
TPI1	0.04761904762	0.04761904762	0.1111111111		0.325	#DIV/0!
TPI1P2	0.04761904762	0.04761904762	0.05555555556		0.7	#DIV/0!
TPK1	0.09523809524	0.09523809524	0.05555555556		1.473684211	#DIV/0!
TPM1	0.04761904762	0.04761904762	0.05555555556		0.7	#DIV/0!
TPM1-AS	0.04761904762	0.04761904762	0.05555555556		0.7	#DIV/0!
TPM2	0.09523809524	0.09523809524	0.05555555556		1.473684211	#DIV/0!
TPM3	0.1904761905	0.1904761905	0.05555555556		3.294117647	#DIV/0!
TPM3P9	0.09523809524	0.09523809524	0.05555555556		1.473684211	#DIV/0!
TPM4	0.1428571429	0.1428571429	0.1111111111		1.083333333	#DIV/0!
TPO	0	0	0.05555555556		0	#DIV/0!
TPP1	0.04761904762	0.04761904762	0		inf	#DIV/0!
TPPP	0	0	0.05555555556		0	#DIV/0!
TPPP3	0.1428571429	0.1428571429	0.05555555556		2.333333333	#DIV/0!
TPR	0.1428571429	0.1428571429	0.05555555556		2.333333333	#DIV/0!
TPRA1	0.09523809524	0.09523809524	0.05555555556		1.473684211	#DIV/0!
TPRG1	0.09523809524	0.09523809524	0.1111111111		0.6842105263	#DIV/0!
PRG1-AS	0.09523809524	0.09523809524	0.1111111111		0.6842105263	#DIV/0!
PRG1-AS	0.09523809524	0.09523809524	0.1111111111		0.6842105263	#DIV/0!
TPRG1L	0	0	0.05555555556		0	#DIV/0!
TPRKB	0	0	0.05555555556		0	#DIV/0!
TPRN	0.1428571429	0.1428571429	0.05555555556		2.333333333	#DIV/0!
TPRX1	0.09523809524	0.09523809524	0.1111111111		0.6842105263	#DIV/0!
TPRXL	0.04761904762	0.04761904762	0		inf	#DIV/0!

TPSAB1	0.04761904762	0.04761904762	0.2222222222		0.1375	#DIV/0!
TPSB2	0.04761904762	0.04761904762	0.2222222222		0.1375	#DIV/0!
TPSD1	0.04761904762	0.04761904762	0.2222222222		0.1375	#DIV/0!
TPSG1	0.04761904762	0.04761904762	0.2222222222		0.1375	#DIV/0!
TPST1	0.04761904762	0.04761904762	0.1111111111		0.325	#DIV/0!
TPST2	0.04761904762	0.04761904762	0.05555555556		0.7	#DIV/0!
TPTE	0.04761904762	0.04761904762	0		inf	#DIV/0!
TPTE2	0.04761904762	0.04761904762	0		inf	#DIV/0!
TPTEP1	0.04761904762	0.04761904762	0		inf	#DIV/0!
TPTEP2	0.04761904762	0.04761904762	0.1111111111		0.325	#DIV/0!
EP2-CSN	0.04761904762	0.04761904762	0.1111111111		0.325	#DIV/0!
TPX2	0.09523809524	0.09523809524	0.1111111111		0.6842105263	#DIV/0!
TRA2A	0.04761904762	0.04761904762	0.1666666667		0.2	#DIV/0!
TRA2B	0.09523809524	0.09523809524	0.05555555556		1.473684211	#DIV/0!
TRABD	0	0	0.1111111111		0	#DIV/0!
RABD2	0	0	0.05555555556		0	#DIV/0!
RABD2	0	0	0.05555555556		0	#DIV/0!
TRADD	0.1428571429	0.1428571429	0.05555555556		2.333333333	#DIV/0!
TRAF1	0.09523809524	0.09523809524	0.05555555556		1.473684211	#DIV/0!
TRAF2	0.1428571429	0.1428571429	0.05555555556		2.333333333	#DIV/0!
RAF3IP	0	0	0.05555555556		0	#DIV/0!
RAF3IP	0	0	0			#DIV/0!
AF3IP2-	0	0	0			#DIV/0!
RAF3IP	0.1428571429	0.1428571429	0		inf	#DIV/0!
TRAF4	0.04761904762	0.04761904762	0.05555555556		0.7	#DIV/0!
TRAF5	0.1428571429	0.1428571429	0		inf	#DIV/0!
TRAF7	0.09523809524	0.09523809524	0.2222222222		0.2894736842	#DIV/0!
TRAFD1	0	0	0.1111111111		0	#DIV/0!
TRAIP	0.09523809524	0.09523809524	0		inf	#DIV/0!
TRAK1	0.04761904762	0.04761904762	0		inf	#DIV/0!
TRAK2	0	0	0.05555555556		0	#DIV/0!
TRAM1	0.1904761905	0.1904761905	0.1666666667		0.9411764706	#DIV/0!
RAM1L	0	0	0.05555555556		0	#DIV/0!
TRAM2	0.04761904762	0.04761904762	0		inf	#DIV/0!
RAM2-A	0.04761904762	0.04761904762	0		inf	#DIV/0!
TRANK1	0.04761904762	0.04761904762	0		inf	#DIV/0!
TRAP1	0.04761904762	0.04761904762	0.1111111111		0.325	#DIV/0!
TRAPPC	0	0	0.05555555556		0	#DIV/0!
RAPPC1	0.04761904762	0.04761904762	0.1111111111		0.325	#DIV/0!
RAPPC1	0	0	0.05555555556		0	#DIV/0!
RAPPC1	0	0	0.05555555556		0	#DIV/0!
RAPPC1	0.04761904762	0.04761904762	0		inf	#DIV/0!
RAPPC2	0.1428571429	0.1428571429	0.05555555556		2.333333333	#DIV/0!
RAPPC2	0.1904761905	0.1904761905	0		inf	#DIV/0!
TRAPPC	0.04761904762	0.04761904762	0.05555555556		0.7	#DIV/0!
TRAPPC	0.1428571429	0.1428571429	0.05555555556		2.333333333	#DIV/0!
RAPPC6	0.09523809524	0.09523809524	0.1111111111		0.6842105263	#DIV/0!
RAPPC6	0.04761904762	0.04761904762	0		inf	#DIV/0!
TRAPPC	0.09523809524	0.09523809524	0		inf	#DIV/0!
TRAPPC	0.04761904762	0.04761904762	0.1666666667		0.2	#DIV/0!
TRARG1	0	0	0.1111111111		0	#DIV/0!

TRAT1	0.09523809524	0.09523809524	0.05555555556		1.473684211	#DIV/0!
TRDMT1	0.09523809524	0.09523809524	0.1111111111		0.6842105263	#DIV/0!
TREM1	0	0	0.05555555556		0	#DIV/0!
TREM2	0	0	0.05555555556		0	#DIV/0!
TREML1	0	0	0.05555555556		0	#DIV/0!
TREML2	0	0	0.05555555556		0	#DIV/0!
TREML3	0	0	0.05555555556		0	#DIV/0!
TREML4	0	0	0.05555555556		0	#DIV/0!
TREML5	0	0	0.05555555556		0	#DIV/0!
TRERF1	0.04761904762	0.04761904762	0.05555555556		0.7	#DIV/0!
TRERNA	0.09523809524	0.09523809524	0.1666666667		0.4210526316	#DIV/0!
TREX1	0.09523809524	0.09523809524	0		inf	#DIV/0!
TRG-AS1	0.04761904762	0.04761904762	0.2222222222		0.1375	#DIV/0!
TRH	0.09523809524	0.09523809524	0.05555555556		1.473684211	#DIV/0!
TRHDE	0.04761904762	0.04761904762	0.05555555556		0.7	#DIV/0!
TRHDE-A	0.04761904762	0.04761904762	0.05555555556		0.7	#DIV/0!
TRHR	0.1904761905	0.1904761905	0.1111111111		1.529411765	#DIV/0!
TRIAP1	0	0	0.1111111111		0	#DIV/0!
TRIB1	0.1428571429	0.1428571429	0.1111111111		1.083333333	#DIV/0!
TRIB2	0	0	0.05555555556		0	#DIV/0!
TRIB3	0.1904761905	0.1904761905	0.05555555556		3.294117647	#DIV/0!
TRIL	0.04761904762	0.04761904762	0.2222222222		0.1375	#DIV/0!
TRIM11	0.1428571429	0.1428571429	0		inf	#DIV/0!
TRIM14	0.04761904762	0.04761904762	0		inf	#DIV/0!
TRIM16	0.04761904762	0.04761904762	0.05555555556		0.7	#DIV/0!
TRIM16L	0.04761904762	0.04761904762	0.1111111111		0.325	#DIV/0!
TRIM17	0.1428571429	0.1428571429	0		inf	#DIV/0!
TRIM2	0	0	0.05555555556		0	#DIV/0!
TRIM21	0.04761904762	0.04761904762	0		inf	#DIV/0!
TRIM22	0.04761904762	0.04761904762	0		inf	#DIV/0!
TRIM23	0.04761904762	0.04761904762	0		inf	#DIV/0!
TRIM24	0.04761904762	0.04761904762	0.05555555556		0.7	#DIV/0!
TRIM25	0.09523809524	0.09523809524	0		inf	#DIV/0!
TRIM28	0.1428571429	0.1428571429	0.05555555556		2.333333333	#DIV/0!
TRIM3	0.04761904762	0.04761904762	0		inf	#DIV/0!
TRIM32	0.04761904762	0.04761904762	0.05555555556		0.7	#DIV/0!
TRIM33	0.04761904762	0.04761904762	0.05555555556		0.7	#DIV/0!
TRIM34	0.04761904762	0.04761904762	0		inf	#DIV/0!
TRIM35	0.04761904762	0.04761904762	0.1111111111		0.325	#DIV/0!
TRIM36	0.04761904762	0.04761904762	0		inf	#DIV/0!
TRIM37	0.1428571429	0.1428571429	0		inf	#DIV/0!
TRIM38	0	0	0.05555555556		0	#DIV/0!
TRIM4	0.04761904762	0.04761904762	0.05555555556		0.7	#DIV/0!
TRIM41	0.09523809524	0.09523809524	0.05555555556		1.473684211	#DIV/0!
TRIM42	0.1428571429	0.1428571429	0.05555555556		2.333333333	#DIV/0!
TRIM43	0	0	0.1111111111		0	#DIV/0!
TRIM43B	0	0	0.1111111111		0	#DIV/0!
TRIM45	0.04761904762	0.04761904762	0.05555555556		0.7	#DIV/0!
TRIM46	0.1904761905	0.1904761905	0.1111111111		1.529411765	#DIV/0!
TRIM47	0.04761904762	0.04761904762	0		inf	#DIV/0!
TRIM48	0.09523809524	0.09523809524	0		inf	#DIV/0!

TRIM5	0.04761904762	0.04761904762	0		inf	#DIV/0!
TRIM50	0.1428571429	0.1428571429	0.1111111111		1.083333333	#DIV/0!
RIM51H	0.09523809524	0.09523809524	0		inf	#DIV/0!
TRIM52	0.09523809524	0.09523809524	0.05555555556		1.473684211	#DIV/0!
IM52-A	0.09523809524	0.09523809524	0.05555555556		1.473684211	#DIV/0!
TRIM55	0.1428571429	0.1428571429	0.1666666667		0.6666666667	#DIV/0!
TRIM56	0.04761904762	0.04761904762	0.05555555556		0.7	#DIV/0!
TRIM58	0.1428571429	0.1428571429	0		inf	#DIV/0!
TRIM59	0.1428571429	0.1428571429	0.05555555556		2.333333333	#DIV/0!
M59-IF	0.1428571429	0.1428571429	0.05555555556		2.333333333	#DIV/0!
TRIM6	0.04761904762	0.04761904762	0		inf	#DIV/0!
M6-TRI	0.04761904762	0.04761904762	0		inf	#DIV/0!
TRIM60	0.04761904762	0.04761904762	0.05555555556		0.7	#DIV/0!
TRIM61	0.04761904762	0.04761904762	0.05555555556		0.7	#DIV/0!
TRIM62	0.04761904762	0.04761904762	0.05555555556		0.7	#DIV/0!
TRIM63	0.04761904762	0.04761904762	0.1111111111		0.325	#DIV/0!
TRIM65	0.04761904762	0.04761904762	0		inf	#DIV/0!
TRIM67	0.1428571429	0.1428571429	0		inf	#DIV/0!
TRIM68	0.04761904762	0.04761904762	0		inf	#DIV/0!
TRIM7	0.09523809524	0.09523809524	0.05555555556		1.473684211	#DIV/0!
TRIM71	0.09523809524	0.09523809524	0		inf	#DIV/0!
TRIM72	0.04761904762	0.04761904762	0		inf	#DIV/0!
TRIM73	0.1428571429	0.1428571429	0.1666666667		0.6666666667	#DIV/0!
TRIM74	0.1428571429	0.1428571429	0.1111111111		1.083333333	#DIV/0!
TRIM8	0.1428571429	0.1428571429	0.05555555556		2.333333333	#DIV/0!
TRIM9	0.04761904762	0.04761904762	0		inf	#DIV/0!
TRIML1	0	0	0.05555555556		0	#DIV/0!
TRIML2	0	0	0.05555555556		0	#DIV/0!
TRIO	0.04761904762	0.04761904762	0.05555555556		0.7	#DIV/0!
TRIOBP	0	0	0.1111111111		0	#DIV/0!
TRIP10	0.1428571429	0.1428571429	0.05555555556		2.333333333	#DIV/0!
TRIP12	0	0	0.05555555556		0	#DIV/0!
TRIP13	0	0	0.05555555556		0	#DIV/0!
TRIP4	0.04761904762	0.04761904762	0.05555555556		0.7	#DIV/0!
TRIP6	0.04761904762	0.04761904762	0.05555555556		0.7	#DIV/0!
TRIQK	0.1904761905	0.1904761905	0.1111111111		1.529411765	#DIV/0!
TRIR	0.1428571429	0.1428571429	0.1111111111		1.083333333	#DIV/0!
TRIT1	0.04761904762	0.04761904762	0.05555555556		0.7	#DIV/0!
TRMO	0.04761904762	0.04761904762	0		inf	#DIV/0!
TRMT1	0.1428571429	0.1428571429	0.1111111111		1.083333333	#DIV/0!
RMT10	0	0	0.05555555556		0	#DIV/0!
RMT10	0.09523809524	0.09523809524	0.05555555556		1.473684211	#DIV/0!
RMT10	0.09523809524	0.09523809524	0.05555555556		1.473684211	#DIV/0!
TRMT12	0.1428571429	0.1428571429	0.1111111111		1.083333333	#DIV/0!
TRMT13	0.04761904762	0.04761904762	0.05555555556		0.7	#DIV/0!
TRMT1L	0.1428571429	0.1428571429	0.05555555556		2.333333333	#DIV/0!
TRMT2A	0.04761904762	0.04761904762	0.05555555556		0.7	#DIV/0!
TRMT6	0.1904761905	0.1904761905	0.05555555556		3.294117647	#DIV/0!
RMT61	0	0	0.05555555556		0	#DIV/0!
TRMT9B	0.09523809524	0.09523809524	0.1111111111		0.6842105263	#DIV/0!
TRMU	0	0	0.1111111111		0	#DIV/0!

RNAU1A	0.04761904762	0.04761904762	0.1111111111		0.325	#DIV/0!
TRNP1	0.04761904762	0.04761904762	0.1111111111		0.325	#DIV/0!
TRNT1	0.04761904762	0.04761904762	0		inf	#DIV/0!
TROAP	0.04761904762	0.04761904762	0.05555555556		0.7	#DIV/0!
TRPA1	0.1904761905	0.1904761905	0.1666666667		0.9411764706	#DIV/0!
TRPC1	0.1904761905	0.1904761905	0.05555555556		3.294117647	#DIV/0!
TRPC2	0.04761904762	0.04761904762	0		inf	#DIV/0!
TRPC3	0	0	0.05555555556		0	#DIV/0!
RPC4AI	0.09523809524	0.09523809524	0.1111111111		0.6842105263	#DIV/0!
TRPC7	0.04761904762	0.04761904762	0		inf	#DIV/0!
RPC7-AS	0.04761904762	0.04761904762	0		inf	#DIV/0!
TRPM2	0.04761904762	0.04761904762	0.1111111111		0.325	#DIV/0!
RPM2-A	0.04761904762	0.04761904762	0.1111111111		0.325	#DIV/0!
TRPM3	0	0	0.05555555556		0	#DIV/0!
TRPM4	0.09523809524	0.09523809524	0.1111111111		0.6842105263	#DIV/0!
TRPM5	0.04761904762	0.04761904762	0		inf	#DIV/0!
TRPM6	0	0	0.05555555556		0	#DIV/0!
TRPM7	0.1428571429	0.1428571429	0.05555555556		2.333333333	#DIV/0!
TRPM8	0	0	0.05555555556		0	#DIV/0!
TRPS1	0.1904761905	0.1904761905	0.1666666667		0.9411764706	#DIV/0!
TRPV1	0	0	0.1111111111		0	#DIV/0!
TRPV2	0.04761904762	0.04761904762	0.05555555556		0.7	#DIV/0!
TRPV3	0	0	0.1111111111		0	#DIV/0!
TRPV4	0	0	0.05555555556		0	#DIV/0!
TRPV5	0.04761904762	0.04761904762	0.05555555556		0.7	#DIV/0!
TRPV6	0.04761904762	0.04761904762	0.05555555556		0.7	#DIV/0!
TRRAP	0.04761904762	0.04761904762	0.05555555556		0.7	#DIV/0!
TRUB1	0.1904761905	0.1904761905	0.05555555556		3.294117647	#DIV/0!
TRUB2	0.09523809524	0.09523809524	0.05555555556		1.473684211	#DIV/0!
TRY2P	0.04761904762	0.04761904762	0.05555555556		0.7	#DIV/0!
TSACC	0.1428571429	0.1428571429	0.1111111111		1.083333333	#DIV/0!
TSBP1	0.04761904762	0.04761904762	0		inf	#DIV/0!
SBP1-AS	0.04761904762	0.04761904762	0		inf	#DIV/0!
TSC1	0.04761904762	0.04761904762	0		inf	#DIV/0!
TSC2	0.09523809524	0.09523809524	0.2222222222		0.2894736842	#DIV/0!
TSC22D2	0.1904761905	0.1904761905	0.1111111111		1.529411765	#DIV/0!
TSC22D4	0.04761904762	0.04761904762	0.05555555556		0.7	#DIV/0!
TSEN15	0.1428571429	0.1428571429	0		inf	#DIV/0!
TSEN2	0.09523809524	0.09523809524	0		inf	#DIV/0!
TSEN34	0.09523809524	0.09523809524	0.05555555556		1.473684211	#DIV/0!
TSFM	0.1428571429	0.1428571429	0.05555555556		2.333333333	#DIV/0!
TSG1	0	0	0			#DIV/0!
TSGA10	0	0	0.05555555556		0	#DIV/0!
TSGA13	0.04761904762	0.04761904762	0.05555555556		0.7	#DIV/0!
TSHB	0.04761904762	0.04761904762	0.05555555556		0.7	#DIV/0!
TSHZ1	0.09523809524	0.09523809524	0		inf	#DIV/0!
TSHZ2	0.09523809524	0.09523809524	0.1111111111		0.6842105263	#DIV/0!
TSHZ3	0.04761904762	0.04761904762	0.05555555556		0.7	#DIV/0!
TSKS	0.09523809524	0.09523809524	0.1111111111		0.6842105263	#DIV/0!
TSL	0.04761904762	0.04761904762	0.2222222222		0.1375	#DIV/0!
TSLP	0.04761904762	0.04761904762	0		inf	#DIV/0!

TSN	0	0	0.05555555556		0	#DIV/0!
TSNARE	0.04761904762	0.04761904762	0.1666666667		0.2	#DIV/0!
TSNAX	0.1428571429	0.1428571429	0		inf	#DIV/0!
NAX-DIS	0.1428571429	0.1428571429	0		inf	#DIV/0!
SNAXIP	0.1428571429	0.1428571429	0.05555555556		2.333333333	#DIV/0!
TSPAN1	0.04761904762	0.04761904762	0.05555555556		0.7	#DIV/0!
TSPAN1(	0.04761904762	0.04761904762	0		inf	#DIV/0!
TSPAN1	0.04761904762	0.04761904762	0.05555555556		0.7	#DIV/0!
TSPAN1	0.04761904762	0.04761904762	0.05555555556		0.7	#DIV/0!
TSPAN1	0.04761904762	0.04761904762	0.2222222222		0.1375	#DIV/0!
TSPAN1	0.1428571429	0.1428571429	0		inf	#DIV/0!
TSPAN1	0.09523809524	0.09523809524	0		inf	#DIV/0!
TSPAN1	0.1428571429	0.1428571429	0.1111111111		1.083333333	#DIV/0!
TSPAN1	0.09523809524	0.09523809524	0.05555555556		1.473684211	#DIV/0!
TSPAN1	0.04761904762	0.04761904762	0.05555555556		0.7	#DIV/0!
TSPAN2	0.04761904762	0.04761904762	0.05555555556		0.7	#DIV/0!
TSPAN3	0.04761904762	0.04761904762	0.05555555556		0.7	#DIV/0!
TSPAN3	0.1428571429	0.1428571429	0.05555555556		2.333333333	#DIV/0!
TSPAN3	0.04761904762	0.04761904762	0		inf	#DIV/0!
TSPAN3	0.04761904762	0.04761904762	0.05555555556		0.7	#DIV/0!
TSPAN4	0.04761904762	0.04761904762	0		inf	#DIV/0!
TSPAN5	0	0	0.05555555556		0	#DIV/0!
TSPAN8	0.09523809524	0.09523809524	0.1111111111		0.6842105263	#DIV/0!
TSPAN9	0.04761904762	0.04761904762	0.1666666667		0.2	#DIV/0!
TSPEAR	0.04761904762	0.04761904762	0.1111111111		0.325	#DIV/0!
PEAR-A	0.04761904762	0.04761904762	0.1111111111		0.325	#DIV/0!
PEAR-A	0.04761904762	0.04761904762	0.1111111111		0.325	#DIV/0!
TSPO	0	0	0.1666666667		0	#DIV/0!
TSPO2	0	0	0.05555555556		0	#DIV/0!
TSPOAP	0.09523809524	0.09523809524	0		inf	#DIV/0!
POAP1-A	0.09523809524	0.09523809524	0		inf	#DIV/0!
TSPYL5	0.1904761905	0.1904761905	0.1111111111		1.529411765	#DIV/0!
TSPYL6	0.04761904762	0.04761904762	0.05555555556		0.7	#DIV/0!
TSR1	0	0	0.1111111111		0	#DIV/0!
TSR3	0.04761904762	0.04761904762	0.2222222222		0.1375	#DIV/0!
TSSC2	0.04761904762	0.04761904762	0		inf	#DIV/0!
TSSC4	0.04761904762	0.04761904762	0		inf	#DIV/0!
TSSK1B	0.04761904762	0.04761904762	0		inf	#DIV/0!
TSSK2	0.04761904762	0.04761904762	0.05555555556		0.7	#DIV/0!
TSSK3	0.04761904762	0.04761904762	0.1111111111		0.325	#DIV/0!
TSSK6	0.1428571429	0.1428571429	0.1111111111		1.083333333	#DIV/0!
TST	0	0	0.1111111111		0	#DIV/0!
TSTA3	0.04761904762	0.04761904762	0.1666666667		0.2	#DIV/0!
TSTD1	0.1428571429	0.1428571429	0		inf	#DIV/0!
TSTD2	0.04761904762	0.04761904762	0		inf	#DIV/0!
TTBK1	0.04761904762	0.04761904762	0.05555555556		0.7	#DIV/0!
TTC1	0.04761904762	0.04761904762	0.05555555556		0.7	#DIV/0!
TTC13	0.1428571429	0.1428571429	0		inf	#DIV/0!
TTC14	0.1904761905	0.1904761905	0.05555555556		3.294117647	#DIV/0!
TTC16	0.09523809524	0.09523809524	0.05555555556		1.473684211	#DIV/0!
TTC19	0.04761904762	0.04761904762	0.05555555556		0.7	#DIV/0!

TTC21A	0.04761904762	0.04761904762	0		inf	#DIV/0!
TTC21B	0	0	0.05555555556		0	#DIV/0!
TC21B-A	0	0	0.05555555556		0	#DIV/0!
TTC22	0.04761904762	0.04761904762	0.05555555556		0.7	#DIV/0!
TTC23	0.04761904762	0.04761904762	0.05555555556		0.7	#DIV/0!
TTC23L	0.04761904762	0.04761904762	0.05555555556		0.7	#DIV/0!
TTC24	0.1428571429	0.1428571429	0.1111111111		1.083333333	#DIV/0!
TTC25	0.09523809524	0.09523809524	0		inf	#DIV/0!
TTC26	0.04761904762	0.04761904762	0.05555555556		0.7	#DIV/0!
TTC27	0.1428571429	0.1428571429	0		inf	#DIV/0!
TTC28	0.04761904762	0.04761904762	0.1111111111		0.325	#DIV/0!
TC28-AS	0.04761904762	0.04761904762	0.1111111111		0.325	#DIV/0!
TTC29	0	0	0.05555555556		0	#DIV/0!
TTC3	0.04761904762	0.04761904762	0.1666666667		0.2	#DIV/0!
TC3-AS	0.04761904762	0.04761904762	0.1666666667		0.2	#DIV/0!
TTC30A	0	0	0.1111111111		0	#DIV/0!
TTC30B	0	0	0.1111111111		0	#DIV/0!
TTC31	0	0	0.05555555556		0	#DIV/0!
TTC32	0	0	0.05555555556		0	#DIV/0!
TTC33	0.04761904762	0.04761904762	0.05555555556		0.7	#DIV/0!
TTC34	0	0	0.05555555556		0	#DIV/0!
TTC37	0.04761904762	0.04761904762	0		inf	#DIV/0!
TTC38	0	0	0.1111111111		0	#DIV/0!
TTC39A	0.04761904762	0.04761904762	0.05555555556		0.7	#DIV/0!
TC39A-A	0.04761904762	0.04761904762	0.05555555556		0.7	#DIV/0!
TTC39B	0.04761904762	0.04761904762	0.05555555556		0.7	#DIV/0!
TTC39C	0.09523809524	0.09523809524	0.05555555556		1.473684211	#DIV/0!
TC39C-A	0.09523809524	0.09523809524	0.05555555556		1.473684211	#DIV/0!
TTC4	0.04761904762	0.04761904762	0.05555555556		0.7	#DIV/0!
TTC41P	0.04761904762	0.04761904762	0.05555555556		0.7	#DIV/0!
TTC5	0.04761904762	0.04761904762	0		inf	#DIV/0!
TTC7A	0	0	0.05555555556		0	#DIV/0!
TTC9B	0.04761904762	0.04761904762	0.05555555556		0.7	#DIV/0!
TTF1	0.09523809524	0.09523809524	0		inf	#DIV/0!
TTF2	0.04761904762	0.04761904762	0.05555555556		0.7	#DIV/0!
TTI1	0.1428571429	0.1428571429	0.1666666667		0.6666666667	#DIV/0!
TTI2	0.04761904762	0.04761904762	0.1111111111		0.325	#DIV/0!
TTL	0	0	0.05555555556		0	#DIV/0!
TTLL1	0	0	0.1666666667		0	#DIV/0!
TTLL10	0	0	0.1111111111		0	#DIV/0!
TTLL11	0.09523809524	0.09523809524	0.05555555556		1.473684211	#DIV/0!
TTLL12	0	0	0.1666666667		0	#DIV/0!
TLL13P	0.04761904762	0.04761904762	0.05555555556		0.7	#DIV/0!
TTLL3	0.09523809524	0.09523809524	0		inf	#DIV/0!
TTLL4	0	0	0.05555555556		0	#DIV/0!
TTLL6	0.1428571429	0.1428571429	0		inf	#DIV/0!
TTLL7	0.04761904762	0.04761904762	0.05555555556		0.7	#DIV/0!
TTLL8	0	0	0.1111111111		0	#DIV/0!
TTLL9	0.09523809524	0.09523809524	0.1111111111		0.6842105263	#DIV/0!
TTN	0	0	0.1111111111		0	#DIV/0!
TN-AS1	0	0	0.1111111111		0	#DIV/0!

TTPA	0.1428571429	0.1428571429	0.1666666667		0.6666666667	#DIV/0!
TTPAL	0.1428571429	0.1428571429	0.1666666667		0.6666666667	#DIV/0!
TTR	0.09523809524	0.09523809524	0		inf	#DIV/0!
TTYH1	0.09523809524	0.09523809524	0.05555555556		1.473684211	#DIV/0!
TTYH2	0.04761904762	0.04761904762	0		inf	#DIV/0!
TTYH3	0.04761904762	0.04761904762	0.1666666667		0.2	#DIV/0!
TUBA1A	0.04761904762	0.04761904762	0.05555555556		0.7	#DIV/0!
TUBA1B	0.04761904762	0.04761904762	0.05555555556		0.7	#DIV/0!
TUBA1C	0.04761904762	0.04761904762	0.05555555556		0.7	#DIV/0!
TUBA3C	0.04761904762	0.04761904762	0		inf	#DIV/0!
TUBA3D	0	0	0.05555555556		0	#DIV/0!
TUBA3E	0	0	0.05555555556		0	#DIV/0!
TUBA3F	0.04761904762	0.04761904762	0.05555555556		0.7	#DIV/0!
TUBA8	0.04761904762	0.04761904762	0.05555555556		0.7	#DIV/0!
TUBAL3	0	0	0.1666666667		0	#DIV/0!
TUBB1	0.1428571429	0.1428571429	0.2222222222		0.4583333333	#DIV/0!
TUBB3	0.09523809524	0.09523809524	0		inf	#DIV/0!
TUBB4A	0.1428571429	0.1428571429	0.05555555556		2.333333333	#DIV/0!
TUBB4B	0.1428571429	0.1428571429	0.05555555556		2.333333333	#DIV/0!
TUBB6	0.09523809524	0.09523809524	0.05555555556		1.473684211	#DIV/0!
TUBB8	0.04761904762	0.04761904762	0.1111111111		0.325	#DIV/0!
UBB8P1	0.09523809524	0.09523809524	0		inf	#DIV/0!
TUBBP5	0.09523809524	0.09523809524	0		inf	#DIV/0!
TUBD1	0.1428571429	0.1428571429	0		inf	#DIV/0!
TUBG1	0.04761904762	0.04761904762	0		inf	#DIV/0!
TUBG2	0.04761904762	0.04761904762	0		inf	#DIV/0!
UBGCP	0.1904761905	0.1904761905	0		inf	#DIV/0!
UBGCP	0.09523809524	0.09523809524	0		inf	#DIV/0!
UBGCP	0	0	0.1111111111		0	#DIV/0!
TUFM	0.04761904762	0.04761904762	0		inf	#DIV/0!
TUFT1	0.1428571429	0.1428571429	0.05555555556		2.333333333	#DIV/0!
TUG1	0.04761904762	0.04761904762	0.1111111111		0.325	#DIV/0!
TULP1	0.04761904762	0.04761904762	0.05555555556		0.7	#DIV/0!
TULP2	0.09523809524	0.09523809524	0.1111111111		0.6842105263	#DIV/0!
TULP3	0.04761904762	0.04761904762	0.1666666667		0.2	#DIV/0!
TUSC1	0.04761904762	0.04761904762	0.05555555556		0.7	#DIV/0!
TUSC2	0.09523809524	0.09523809524	0		inf	#DIV/0!
TUSC3	0.09523809524	0.09523809524	0.1111111111		0.6842105263	#DIV/0!
TUSC7	0.09523809524	0.09523809524	0.05555555556		1.473684211	#DIV/0!
TUT4	0.04761904762	0.04761904762	0.05555555556		0.7	#DIV/0!
TUT7	0	0	0.05555555556		0	#DIV/0!
TVP23A	0.04761904762	0.04761904762	0		inf	#DIV/0!
TVP23B	0.04761904762	0.04761904762	0.1111111111		0.325	#DIV/0!
TVP23C	0.04761904762	0.04761904762	0.05555555556		0.7	#DIV/0!
23C-CD	0.04761904762	0.04761904762	0.05555555556		0.7	#DIV/0!
TWF1	0.04761904762	0.04761904762	0.05555555556		0.7	#DIV/0!
TWF2	0.04761904762	0.04761904762	0		inf	#DIV/0!
TWIST1	0.04761904762	0.04761904762	0.2222222222		0.1375	#DIV/0!
TWIST2	0	0	0.05555555556		0	#DIV/0!
WISTN	0.04761904762	0.04761904762	0.2222222222		0.1375	#DIV/0!
TWNK	0.1428571429	0.1428571429	0		inf	#DIV/0!

**TWSG1**	0.09523809524	0.09523809524	0		inf	#DIV/0!
**TXK**	0.04761904762	0.04761904762	0.1111111111		0.325	#DIV/0!
**TXLNA**	0.04761904762	0.04761904762	0.1111111111		0.325	#DIV/0!
**TXN**	0.04761904762	0.04761904762	0.05555555556		0.7	#DIV/0!
**TXN2**	0	0	0.1111111111		0	#DIV/0!
**TXNDC1**	0.04761904762	0.04761904762	0		inf	#DIV/0!
**TXNDC1**	0.04761904762	0.04761904762	0.05555555556		0.7	#DIV/0!
**NDC12-A**	0.04761904762	0.04761904762	0.05555555556		0.7	#DIV/0!
**TXNDC1**	0.04761904762	0.04761904762	0		inf	#DIV/0!
**TXNDC1**	0.04761904762	0.04761904762	0		inf	#DIV/0!
**TXNDC1**	0	0	0.05555555556		0	#DIV/0!
**TXNDC2**	0.09523809524	0.09523809524	0.05555555556		1.473684211	#DIV/0!
**TXNDC8**	0.04761904762	0.04761904762	0.05555555556		0.7	#DIV/0!
**TXNDC9**	0	0	0.05555555556		0	#DIV/0!
**TXNL1**	0.09523809524	0.09523809524	0		inf	#DIV/0!
**TXNL4A**	0.04761904762	0.04761904762	0.05555555556		0.7	#DIV/0!
**TXNL4B**	0.04761904762	0.04761904762	0		inf	#DIV/0!
**TXNRD1**	0.04761904762	0.04761904762	0.05555555556		0.7	#DIV/0!
**TXNRD2**	0.04761904762	0.04761904762	0.05555555556		0.7	#DIV/0!
**TXNRD3**	0.04761904762	0.04761904762	0.05555555556		0.7	#DIV/0!
**XNRD3N**	0.04761904762	0.04761904762	0.05555555556		0.7	#DIV/0!
**TYK2**	0.1428571429	0.1428571429	0.1111111111		1.083333333	#DIV/0!
**TYMP**	0	0	0.1111111111		0	#DIV/0!
**TYMS**	0.09523809524	0.09523809524	0		inf	#DIV/0!
**TYMSOS**	0.09523809524	0.09523809524	0		inf	#DIV/0!
**TYRO3P**	0.04761904762	0.04761904762	0.05555555556		0.7	#DIV/0!
**TYROBP**	0.04761904762	0.04761904762	0.05555555556		0.7	#DIV/0!
**TYRP1**	0.09523809524	0.09523809524	0.05555555556		1.473684211	#DIV/0!
**TYSND1**	0.09523809524	0.09523809524	0		inf	#DIV/0!
**TYW1**	0.04761904762	0.04761904762	0.1111111111		0.325	#DIV/0!
**TYW1B**	0.1428571429	0.1428571429	0.1111111111		1.083333333	#DIV/0!
**TYW3**	0.04761904762	0.04761904762	0.05555555556		0.7	#DIV/0!
**TYW5**	0	0	0.05555555556		0	#DIV/0!
**U2AF1**	0.04761904762	0.04761904762	0.1666666667		0.2	#DIV/0!
**U2AF1L4**	0.04761904762	0.04761904762	0.05555555556		0.7	#DIV/0!
**U2AF1L5**	0.04761904762	0.04761904762	0.1666666667		0.2	#DIV/0!
**U2AF2**	0.09523809524	0.09523809524	0.05555555556		1.473684211	#DIV/0!
**U2SURP**	0.1428571429	0.1428571429	0.05555555556		2.333333333	#DIV/0!
**UACA**	0.04761904762	0.04761904762	0.05555555556		0.7	#DIV/0!
**UAP1**	0.1428571429	0.1428571429	0		inf	#DIV/0!
**UAP1L1**	0.1428571429	0.1428571429	0.05555555556		2.333333333	#DIV/0!
**UBA2**	0.04761904762	0.04761904762	0.05555555556		0.7	#DIV/0!
**UBA3**	0.04761904762	0.04761904762	0		inf	#DIV/0!
**UBA5**	0.1428571429	0.1428571429	0.05555555556		2.333333333	#DIV/0!
**UBA52**	0.1428571429	0.1428571429	0.1111111111		1.083333333	#DIV/0!
**UBA6**	0.04761904762	0.04761904762	0.05555555556		0.7	#DIV/0!
**BA6-AS**	0.04761904762	0.04761904762	0.05555555556		0.7	#DIV/0!
**UBA7**	0.09523809524	0.09523809524	0		inf	#DIV/0!
**UBAC1**	0.04761904762	0.04761904762	0		inf	#DIV/0!
**UBALD1**	0.04761904762	0.04761904762	0		inf	#DIV/0!
**UBALD2**	0.04761904762	0.04761904762	0		inf	#DIV/0!

**UBAP1**	0.09523809524	0.09523809524	0.05555555556		1.473684211	#DIV/0!
**UBAP1L**	0.04761904762	0.04761904762	0.05555555556		0.7	#DIV/0!
**UBAP2**	0.09523809524	0.09523809524	0.05555555556		1.473684211	#DIV/0!
**UBAP2L**	0.1904761905	0.1904761905	0.05555555556		3.294117647	#DIV/0!
**UBASH3**	0.04761904762	0.04761904762	0.1111111111		0.325	#DIV/0!
**UBB**	0.04761904762	0.04761904762	0.05555555556		0.7	#DIV/0!
**UBBP4**	0.09523809524	0.09523809524	0.05555555556		1.473684211	#DIV/0!
**UBC**	0.04761904762	0.04761904762	0.05555555556		0.7	#DIV/0!
**UBE2B**	0.04761904762	0.04761904762	0		inf	#DIV/0!
**UBE2C**	0.1428571429	0.1428571429	0.1666666667		0.6666666667	#DIV/0!
**UBE2CP**	0.1428571429	0.1428571429	0.05555555556		2.333333333	#DIV/0!
**UBE2D1**	0.09523809524	0.09523809524	0.05555555556		1.473684211	#DIV/0!
**UBE2D2**	0.04761904762	0.04761904762	0		inf	#DIV/0!
**UBE2D3**	0	0	0.05555555556		0	#DIV/0!
**BE2D3-A**	0	0	0.05555555556		0	#DIV/0!
**UBE2D4**	0.04761904762	0.04761904762	0.1666666667		0.2	#DIV/0!
**UBE2E1**	0.04761904762	0.04761904762	0		inf	#DIV/0!
**BE2E1-A**	0.04761904762	0.04761904762	0		inf	#DIV/0!
**UBE2E2**	0.04761904762	0.04761904762	0		inf	#DIV/0!
**BE2E2-A**	0.04761904762	0.04761904762	0		inf	#DIV/0!
**UBE2E3**	0	0	0.05555555556		0	#DIV/0!
**UBE2F**	0	0	0.05555555556		0	#DIV/0!
**BE2F-SC**	0	0	0.05555555556		0	#DIV/0!
**UBE2G1**	0	0	0.1111111111		0	#DIV/0!
**UBE2G2**	0.04761904762	0.04761904762	0.1111111111		0.325	#DIV/0!
**UBE2H**	0.04761904762	0.04761904762	0.05555555556		0.7	#DIV/0!
**UBE2I**	0.04761904762	0.04761904762	0.2222222222		0.1375	#DIV/0!
**UBE2J2**	0	0	0.1111111111		0	#DIV/0!
**UBE2K**	0.1904761905	0.1904761905	0.05555555556		3.294117647	#DIV/0!
**UBE2L3**	0.04761904762	0.04761904762	0.05555555556		0.7	#DIV/0!
**UBE2M**	0.1428571429	0.1428571429	0.05555555556		2.333333333	#DIV/0!
**UBE2MP**	0.04761904762	0.04761904762	0		inf	#DIV/0!
**UBE2N**	0.04761904762	0.04761904762	0.05555555556		0.7	#DIV/0!
**UBE2O**	0.04761904762	0.04761904762	0		inf	#DIV/0!
**UBE2Q1**	0.1904761905	0.1904761905	0.1111111111		1.529411765	#DIV/0!
**BE2Q1-A**	0.1904761905	0.1904761905	0.1111111111		1.529411765	#DIV/0!
**UBE2Q2**	0.04761904762	0.04761904762	0.05555555556		0.7	#DIV/0!
**UBE2Q2I**	0.04761904762	0.04761904762	0.05555555556		0.7	#DIV/0!
**BE2Q2P**	0.04761904762	0.04761904762	0.05555555556		0.7	#DIV/0!
**BE2Q2P**	0.04761904762	0.04761904762	0.05555555556		0.7	#DIV/0!
**UBE2QL**	0	0	0.05555555556		0	#DIV/0!
**UBE2R2**	0.09523809524	0.09523809524	0.05555555556		1.473684211	#DIV/0!
**UBE2S**	0.09523809524	0.09523809524	0.05555555556		1.473684211	#DIV/0!
**UBE2T**	0.1428571429	0.1428571429	0		inf	#DIV/0!
**UBE2U**	0.04761904762	0.04761904762	0.05555555556		0.7	#DIV/0!
**UBE2V1**	0.09523809524	0.09523809524	0.1666666667		0.4210526316	#DIV/0!
**UBE2V2**	0.09523809524	0.09523809524	0.1666666667		0.4210526316	#DIV/0!
**UBE2W**	0.1904761905	0.1904761905	0.1111111111		1.529411765	#DIV/0!
**UBE2Z**	0.1428571429	0.1428571429	0		inf	#DIV/0!
**UBE3B**	0	0	0.05555555556		0	#DIV/0!
**UBE3C**	0.1428571429	0.1428571429	0.05555555556		2.333333333	#DIV/0!

**UBE4B**	0	0	0.1111111111		0	#DIV/0!
**UBFD1**	0.04761904762	0.04761904762	0		inf	#DIV/0!
**UBIAD1**	0.04761904762	0.04761904762	0.1111111111		0.325	#DIV/0!
**UBL4B**	0.04761904762	0.04761904762	0		inf	#DIV/0!
**UBL5**	0.1428571429	0.1428571429	0.1111111111		1.083333333	#DIV/0!
**UBL7**	0.04761904762	0.04761904762	0.05555555556		0.7	#DIV/0!
**JBL7-AS**	0.04761904762	0.04761904762	0.05555555556		0.7	#DIV/0!
**UBLCP1**	0.04761904762	0.04761904762	0.05555555556		0.7	#DIV/0!
**UBN2**	0.04761904762	0.04761904762	0.05555555556		0.7	#DIV/0!
**UBOX5**	0.1904761905	0.1904761905	0.05555555556		3.294117647	#DIV/0!
**3OX5-A**	0.1904761905	0.1904761905	0.05555555556		3.294117647	#DIV/0!
**UBP1**	0.09523809524	0.09523809524	0		inf	#DIV/0!
**JBQLN1**	0	0	0.05555555556		0	#DIV/0!
**JBQLN3**	0.04761904762	0.04761904762	0		inf	#DIV/0!
**JBQLN4**	0.1428571429	0.1428571429	0.1111111111		1.083333333	#DIV/0!
**JBQLNI**	0.04761904762	0.04761904762	0		inf	#DIV/0!
**UBR2**	0.04761904762	0.04761904762	0.05555555556		0.7	#DIV/0!
**UBR3**	0	0	0.05555555556		0	#DIV/0!
**UBR4**	0.04761904762	0.04761904762	0.1111111111		0.325	#DIV/0!
**UBR5**	0.1904761905	0.1904761905	0.1111111111		1.529411765	#DIV/0!
**BR5-AS**	0.1904761905	0.1904761905	0.1111111111		1.529411765	#DIV/0!
**UBTD1**	0.1428571429	0.1428571429	0		inf	#DIV/0!
**UBTD2**	0	0	0.05555555556		0	#DIV/0!
**UBTF**	0.09523809524	0.09523809524	0		inf	#DIV/0!
**UBXN10**	0.04761904762	0.04761904762	0.1111111111		0.325	#DIV/0!
**3XN10-A**	0.04761904762	0.04761904762	0.1111111111		0.325	#DIV/0!
**UBXN11**	0.04761904762	0.04761904762	0.1111111111		0.325	#DIV/0!
**JBXN2B**	0.1428571429	0.1428571429	0.1666666667		0.6666666667	#DIV/0!
**UBXN4**	0.04761904762	0.04761904762	0.05555555556		0.7	#DIV/0!
**UBXN6**	0.09523809524	0.09523809524	0.05555555556		1.473684211	#DIV/0!
**UBXN7**	0.1428571429	0.1428571429	0.05555555556		2.333333333	#DIV/0!
**3XN7-A**	0.1428571429	0.1428571429	0.05555555556		2.333333333	#DIV/0!
**UBXN8**	0.04761904762	0.04761904762	0.1111111111		0.325	#DIV/0!
**UC.134**	0.1428571429	0.1428571429	0.05555555556		2.333333333	#DIV/0!
**UCA1**	0.1428571429	0.1428571429	0.1111111111		1.083333333	#DIV/0!
**UCHL1**	0.04761904762	0.04761904762	0		inf	#DIV/0!
**CHL1-A**	0.04761904762	0.04761904762	0		inf	#DIV/0!
**UCHL3**	0	0	0.05555555556		0	#DIV/0!
**UCHL5**	0.1428571429	0.1428571429	0		inf	#DIV/0!
**UCK1**	0.09523809524	0.09523809524	0.05555555556		1.473684211	#DIV/0!
**UCK2**	0.1428571429	0.1428571429	0		inf	#DIV/0!
**UCKL1**	0.1428571429	0.1428571429	0.2222222222		0.4583333333	#DIV/0!
**CKL1-A**	0.1428571429	0.1428571429	0.2222222222		0.4583333333	#DIV/0!
**UCMA**	0.09523809524	0.09523809524	0.1111111111		0.6842105263	#DIV/0!
**UCN2**	0.09523809524	0.09523809524	0		inf	#DIV/0!
**UCN3**	0	0	0.1666666667		0	#DIV/0!
**UCP1**	0	0	0.05555555556		0	#DIV/0!
**UFC1**	0.1428571429	0.1428571429	0		inf	#DIV/0!
**UFD1**	0.04761904762	0.04761904762	0.05555555556		0.7	#DIV/0!
**UFSP1**	0.04761904762	0.04761904762	0.05555555556		0.7	#DIV/0!
**UFSP2**	0	0	0.05555555556		0	#DIV/0!

**UGCG**	0.04761904762	0.04761904762	0.05555555556		0.7	#DIV/0!
**UGDH**	0.1904761905	0.1904761905	0.05555555556		3.294117647	#DIV/0!
**GDH-AS**	0.1904761905	0.1904761905	0.05555555556		3.294117647	#DIV/0!
**UGGT1**	0	0	0.05555555556		0	#DIV/0!
**UGP2**	0	0	0.05555555556		0	#DIV/0!
**UGT1A1**	0	0	0.05555555556		0	#DIV/0!
**UGT1A1(**	0	0	0.05555555556		0	#DIV/0!
**UGT1A3**	0	0	0.05555555556		0	#DIV/0!
**UGT1A4**	0	0	0.05555555556		0	#DIV/0!
**UGT1A5**	0	0	0.05555555556		0	#DIV/0!
**UGT1A6**	0	0	0.05555555556		0	#DIV/0!
**UGT1A7**	0	0	0.05555555556		0	#DIV/0!
**UGT1A8**	0	0	0.05555555556		0	#DIV/0!
**UGT1A9**	0	0	0.05555555556		0	#DIV/0!
**UGT2A1**	0.04761904762	0.04761904762	0.05555555556		0.7	#DIV/0!
**UGT2A2**	0.04761904762	0.04761904762	0.05555555556		0.7	#DIV/0!
**UGT2A3**	0.04761904762	0.04761904762	0.05555555556		0.7	#DIV/0!
**JGT2B1(**	0.04761904762	0.04761904762	0.05555555556		0.7	#DIV/0!
**JGT2B1**	0.04761904762	0.04761904762	0.05555555556		0.7	#DIV/0!
**JGT2B1**	0.04761904762	0.04761904762	0.05555555556		0.7	#DIV/0!
**JGT2B1**	0.04761904762	0.04761904762	0.05555555556		0.7	#DIV/0!
**JGT2B2**	0.04761904762	0.04761904762	0.05555555556		0.7	#DIV/0!
**UGT2B4**	0.04761904762	0.04761904762	0.05555555556		0.7	#DIV/0!
**UGT2B7**	0.04761904762	0.04761904762	0.05555555556		0.7	#DIV/0!
**UGT3A1**	0.04761904762	0.04761904762	0.05555555556		0.7	#DIV/0!
**UGT3A2**	0.04761904762	0.04761904762	0.05555555556		0.7	#DIV/0!
**UGT8**	0	0	0.05555555556		0	#DIV/0!
**UHMK1**	0.1428571429	0.1428571429	0		inf	#DIV/0!
**UHRF1**	0.09523809524	0.09523809524	0.05555555556		1.473684211	#DIV/0!
**HRF1BF**	0.04761904762	0.04761904762	0		inf	#DIV/0!
**IRF1BP**	0.04761904762	0.04761904762	0.05555555556		0.7	#DIV/0!
**UHRF2**	0.04761904762	0.04761904762	0.05555555556		0.7	#DIV/0!
**UIMC1**	0.09523809524	0.09523809524	0.05555555556		1.473684211	#DIV/0!
**ULK1**	0.09523809524	0.09523809524	0.05555555556		1.473684211	#DIV/0!
**ULK2**	0.09523809524	0.09523809524	0.05555555556		1.473684211	#DIV/0!
**ULK3**	0.04761904762	0.04761904762	0.05555555556		0.7	#DIV/0!
**ULK4**	0.04761904762	0.04761904762	0		inf	#DIV/0!
**UMAD1**	0.04761904762	0.04761904762	0.1666666667		0.2	#DIV/0!
**UMOD**	0.04761904762	0.04761904762	0		inf	#DIV/0!
**JMODL**	0.04761904762	0.04761904762	0.1111111111		0.325	#DIV/0!
**IODL1-A**	0.04761904762	0.04761904762	0.1111111111		0.325	#DIV/0!
**UMPS**	0.04761904762	0.04761904762	0.05555555556		0.7	#DIV/0!
**UNC119**	0.04761904762	0.04761904762	0.05555555556		0.7	#DIV/0!
**JNC119I**	0	0	0.1111111111		0	#DIV/0!
**UNC13A**	0.1428571429	0.1428571429	0.1111111111		1.083333333	#DIV/0!
**UNC13B**	0.09523809524	0.09523809524	0.05555555556		1.473684211	#DIV/0!
**UNC13C**	0.04761904762	0.04761904762	0.05555555556		0.7	#DIV/0!
**UNC13D**	0.04761904762	0.04761904762	0		inf	#DIV/0!
**UNC45A**	0.04761904762	0.04761904762	0.05555555556		0.7	#DIV/0!
**UNC50**	0	0	0.05555555556		0	#DIV/0!
**UNC5A**	0.09523809524	0.09523809524	0.05555555556		1.473684211	#DIV/0!

**UNC5B**	0.09523809524	0.09523809524	0		inf	#DIV/0!
**NC5B-A**	0.09523809524	0.09523809524	0		inf	#DIV/0!
**UNC5C**	0	0	0.05555555556		0	#DIV/0!
**JNC5CI**	0	0	0.05555555556		0	#DIV/0!
**UNC5D**	0.04761904762	0.04761904762	0.1111111111		0.325	#DIV/0!
**UNC80**	0	0	0.05555555556		0	#DIV/0!
**UNCX**	0.04761904762	0.04761904762	0.1666666667		0.2	#DIV/0!
**UNG**	0	0	0.05555555556		0	#DIV/0!
**UNK**	0.04761904762	0.04761904762	0		inf	#DIV/0!
**UNKL**	0.09523809524	0.09523809524	0.2222222222		0.2894736842	#DIV/0!
**JNQ649**	0.04761904762	0.04761904762	0.05555555556		0.7	#DIV/0!
**UOX**	0.04761904762	0.04761904762	0.05555555556		0.7	#DIV/0!
**UPB1**	0.04761904762	0.04761904762	0.1111111111		0.325	#DIV/0!
**UPF1**	0.1428571429	0.1428571429	0.1111111111		1.083333333	#DIV/0!
**UPF2**	0.09523809524	0.09523809524	0.1111111111		0.6842105263	#DIV/0!
**UPK1A**	0.04761904762	0.04761904762	0.05555555556		0.7	#DIV/0!
**PK1A-A**	0.04761904762	0.04761904762	0.05555555556		0.7	#DIV/0!
**UPK1B**	0.09523809524	0.09523809524	0.05555555556		1.473684211	#DIV/0!
**UPK3A**	0	0	0.1111111111		0	#DIV/0!
**UPK3B**	0.09523809524	0.09523809524	0.1111111111		0.6842105263	#DIV/0!
**JPK3BL**	0.04761904762	0.04761904762	0.05555555556		0.7	#DIV/0!
**JPK3BL**	0.04761904762	0.04761904762	0.05555555556		0.7	#DIV/0!
**UPP1**	0.04761904762	0.04761904762	0.2222222222		0.1375	#DIV/0!
**UPP2**	0	0	0.05555555556		0	#DIV/0!
**UQCC1**	0.09523809524	0.09523809524	0.1111111111		0.6842105263	#DIV/0!
**UQCC2**	0.04761904762	0.04761904762	0		inf	#DIV/0!
**UQCR10**	0.04761904762	0.04761904762	0.1111111111		0.325	#DIV/0!
**UQCR11**	0.09523809524	0.09523809524	0.1111111111		0.6842105263	#DIV/0!
**UQCRB**	0.1904761905	0.1904761905	0.1666666667		0.9411764706	#DIV/0!
**JQCRC1**	0.09523809524	0.09523809524	0		inf	#DIV/0!
**JQCRC2**	0.04761904762	0.04761904762	0		inf	#DIV/0!
**JQCRFS**	0.04761904762	0.04761904762	0.1666666667		0.2	#DIV/0!
**UQCRH**	0.04761904762	0.04761904762	0.05555555556		0.7	#DIV/0!
**JQCRHI**	0.04761904762	0.04761904762	0.1111111111		0.325	#DIV/0!
**UQCRQ**	0.04761904762	0.04761904762	0		inf	#DIV/0!
**URAHP**	0.09523809524	0.09523809524	0		inf	#DIV/0!
**URB1**	0.04761904762	0.04761904762	0.1111111111		0.325	#DIV/0!
**RB1-AS**	0.04761904762	0.04761904762	0.1111111111		0.325	#DIV/0!
**URB2**	0.1428571429	0.1428571429	0		inf	#DIV/0!
**URGCP**	0.04761904762	0.04761904762	0.1666666667		0.2	#DIV/0!
**CP-MR**	0.04761904762	0.04761904762	0.1666666667		0.2	#DIV/0!
**URI1**	0.04761904762	0.04761904762	0.1666666667		0.2	#DIV/0!
**URM1**	0.09523809524	0.09523809524	0.05555555556		1.473684211	#DIV/0!
**UROC1**	0.04761904762	0.04761904762	0.05555555556		0.7	#DIV/0!
**UROD**	0.04761904762	0.04761904762	0.05555555556		0.7	#DIV/0!
**UROS**	0.2380952381	0.2380952381	0.05555555556		4.375	#DIV/0!
**USB1**	0.04761904762	0.04761904762	0		inf	#DIV/0!
**USE1**	0.1428571429	0.1428571429	0.1111111111		1.083333333	#DIV/0!
**USF1**	0.1428571429	0.1428571429	0		inf	#DIV/0!
**USF2**	0.04761904762	0.04761904762	0.05555555556		0.7	#DIV/0!
**USF3**	0.09523809524	0.09523809524	0.05555555556		1.473684211	#DIV/0!

**USH1G**	0.04761904762	0.04761904762	0		inf	#DIV/0!
**USH2A**	0.1904761905	0.1904761905	0		inf	#DIV/0!
**USHBP1**	0.1428571429	0.1428571429	0.1111111111		1.083333333	#DIV/0!
**USO1**	0.04761904762	0.04761904762	0.05555555556		0.7	#DIV/0!
**USP1**	0.04761904762	0.04761904762	0.05555555556		0.7	#DIV/0!
**USP10**	0.04761904762	0.04761904762	0		inf	#DIV/0!
**USP13**	0.1904761905	0.1904761905	0.05555555556		3.294117647	#DIV/0!
**USP14**	0.09523809524	0.09523809524	0		inf	#DIV/0!
**USP15**	0.09523809524	0.09523809524	0		inf	#DIV/0!
**USP16**	0.04761904762	0.04761904762	0.1111111111		0.325	#DIV/0!
**USP17L1**	0.09523809524	0.09523809524	0.2222222222		0.2894736842	#DIV/0!
**USP17L2**	0.09523809524	0.09523809524	0.1111111111		0.6842105263	#DIV/0!
**USP17L3**	0.09523809524	0.09523809524	0.2222222222		0.2894736842	#DIV/0!
**USP17L4**	0.09523809524	0.09523809524	0.2222222222		0.2894736842	#DIV/0!
**USP17L7**	0.09523809524	0.09523809524	0.1111111111		0.6842105263	#DIV/0!
**USP17L8**	0.09523809524	0.09523809524	0.2222222222		0.2894736842	#DIV/0!
**USP18**	0.04761904762	0.04761904762	0.05555555556		0.7	#DIV/0!
**USP19**	0.09523809524	0.09523809524	0		inf	#DIV/0!
**USP20**	0.09523809524	0.09523809524	0.05555555556		1.473684211	#DIV/0!
**USP21**	0.1428571429	0.1428571429	0		inf	#DIV/0!
**USP22**	0.09523809524	0.09523809524	0.05555555556		1.473684211	#DIV/0!
**USP24**	0.04761904762	0.04761904762	0.05555555556		0.7	#DIV/0!
**USP25**	0.04761904762	0.04761904762	0.1111111111		0.325	#DIV/0!
**USP29**	0.1428571429	0.1428571429	0.05555555556		2.333333333	#DIV/0!
**USP3**	0.04761904762	0.04761904762	0.05555555556		0.7	#DIV/0!
**USP3-AS**	0.04761904762	0.04761904762	0.05555555556		0.7	#DIV/0!
**USP30**	0	0	0.05555555556		0	#DIV/0!
**USP30-AS**	0	0	0.05555555556		0	#DIV/0!
**USP31**	0.04761904762	0.04761904762	0		inf	#DIV/0!
**USP32**	0.1428571429	0.1428571429	0		inf	#DIV/0!
**USP32P1**	0.04761904762	0.04761904762	0.05555555556		0.7	#DIV/0!
**USP32P2**	0.04761904762	0.04761904762	0.1111111111		0.325	#DIV/0!
**USP32P3**	0.09523809524	0.09523809524	0.05555555556		1.473684211	#DIV/0!
**USP33**	0.04761904762	0.04761904762	0.05555555556		0.7	#DIV/0!
**USP34**	0.09523809524	0.09523809524	0.05555555556		1.473684211	#DIV/0!
**USP36**	0.04761904762	0.04761904762	0		inf	#DIV/0!
**USP37**	0	0	0.05555555556		0	#DIV/0!
**USP38**	0	0	0.05555555556		0	#DIV/0!
**USP39**	0	0	0.05555555556		0	#DIV/0!
**USP4**	0.1428571429	0.1428571429	0		inf	#DIV/0!
**USP40**	0	0	0.05555555556		0	#DIV/0!
**USP42**	0.04761904762	0.04761904762	0.1666666667		0.2	#DIV/0!
**USP43**	0.04761904762	0.04761904762	0.05555555556		0.7	#DIV/0!
**USP44**	0.04761904762	0.04761904762	0.05555555556		0.7	#DIV/0!
**USP46**	0.2380952381	0.2380952381	0.05555555556		4.375	#DIV/0!
**USP46-AS**	0.2380952381	0.2380952381	0.05555555556		4.375	#DIV/0!
**USP48**	0.04761904762	0.04761904762	0.1111111111		0.325	#DIV/0!
**USP49**	0.04761904762	0.04761904762	0.05555555556		0.7	#DIV/0!
**USP5**	0.04761904762	0.04761904762	0.1111111111		0.325	#DIV/0!
**USP50**	0.1428571429	0.1428571429	0.05555555556		2.333333333	#DIV/0!
**USP53**	0	0	0.05555555556		0	#DIV/0!

**USP54**	0.1904761905	0.1904761905	0.05555555556		3.294117647	#DIV/0!
**USP6**	0	0	0.1111111111		0	#DIV/0!
**USP6NL**	0.09523809524	0.09523809524	0.1111111111		0.6842105263	#DIV/0!
**USP7**	0.09523809524	0.09523809524	0		inf	#DIV/0!
**USP8**	0.1428571429	0.1428571429	0.05555555556		2.333333333	#DIV/0!
**UTAT33**	0	0	0.05555555556		0	#DIV/0!
**UTF1**	0.1904761905	0.1904761905	0		inf	#DIV/0!
**UTP11**	0.04761904762	0.04761904762	0.05555555556		0.7	#DIV/0!
**UTP15**	0	0	0.05555555556		0	#DIV/0!
**UTP18**	0.04761904762	0.04761904762	0		inf	#DIV/0!
**UTP20**	0.04761904762	0.04761904762	0.05555555556		0.7	#DIV/0!
**UTP23**	0.1904761905	0.1904761905	0.1666666667		0.9411764706	#DIV/0!
**UTP25**	0.1428571429	0.1428571429	0		inf	#DIV/0!
**UTP3**	0.09523809524	0.09523809524	0.05555555556		1.473684211	#DIV/0!
**UTP4**	0.1428571429	0.1428571429	0.05555555556		2.333333333	#DIV/0!
**UTP6**	0	0	0			#DIV/0!
**UTS2**	0	0	0.05555555556		0	#DIV/0!
**UTS2B**	0.09523809524	0.09523809524	0.05555555556		1.473684211	#DIV/0!
**UTS2R**	0.04761904762	0.04761904762	0		inf	#DIV/0!
**UVSSA**	0.04761904762	0.04761904762	0		inf	#DIV/0!
**UXS1**	0	0	0.05555555556		0	#DIV/0!
**VAC14**	0.04761904762	0.04761904762	0		inf	#DIV/0!
**AC14-AS**	0.04761904762	0.04761904762	0		inf	#DIV/0!
**VAMP1**	0.04761904762	0.04761904762	0.1111111111		0.325	#DIV/0!
**VAMP2**	0	0	0.05555555556		0	#DIV/0!
**VAMP3**	0	0	0.05555555556		0	#DIV/0!
**VAMP4**	0.1428571429	0.1428571429	0.05555555556		2.333333333	#DIV/0!
**VAMP5**	0	0	0.05555555556		0	#DIV/0!
**VAMP8**	0	0	0.05555555556		0	#DIV/0!
**VAPA**	0.09523809524	0.09523809524	0.05555555556		1.473684211	#DIV/0!
**VAPB**	0.1428571429	0.1428571429	0.2222222222		0.4583333333	#DIV/0!
**VARS**	0.04761904762	0.04761904762	0		inf	#DIV/0!
**VASH2**	0.09523809524	0.09523809524	0		inf	#DIV/0!
**VASP**	0.09523809524	0.09523809524	0.1111111111		0.6842105263	#DIV/0!
**VAT1**	0.04761904762	0.04761904762	0		inf	#DIV/0!
**VAT1L**	0.09523809524	0.09523809524	0		inf	#DIV/0!
**VAV1**	0.1428571429	0.1428571429	0.05555555556		2.333333333	#DIV/0!
**VAV2**	0.04761904762	0.04761904762	0		inf	#DIV/0!
**VAV3**	0.04761904762	0.04761904762	0.05555555556		0.7	#DIV/0!
**AV3-AS**	0.04761904762	0.04761904762	0.05555555556		0.7	#DIV/0!
**VAX1**	0.2380952381	0.2380952381	0		inf	#DIV/0!
**VAX2**	0	0	0.05555555556		0	#DIV/0!
**VCAM1**	0.04761904762	0.04761904762	0.05555555556		0.7	#DIV/0!
**VCAN**	0.04761904762	0.04761904762	0.05555555556		0.7	#DIV/0!
**CAN-AS**	0.04761904762	0.04761904762	0.05555555556		0.7	#DIV/0!
**VCL**	0.1904761905	0.1904761905	0.05555555556		3.294117647	#DIV/0!
**VCP**	0.09523809524	0.09523809524	0.05555555556		1.473684211	#DIV/0!
**VCPIP1**	0.1428571429	0.1428571429	0.1666666667		0.6666666667	#DIV/0!
**CPKMT**	0.04761904762	0.04761904762	0		inf	#DIV/0!
**VDAC1**	0.04761904762	0.04761904762	0		inf	#DIV/0!
**VDAC2**	0.1904761905	0.1904761905	0.05555555556		3.294117647	#DIV/0!

**VDAC3**	0.09523809524	0.09523809524	0.1666666667		0.4210526316	#DIV/0!
**VDR**	0.04761904762	0.04761904762	0.05555555556		0.7	#DIV/0!
**VEGFA**	0.04761904762	0.04761904762	0.05555555556		0.7	#DIV/0!
**VEGFC**	0	0	0.05555555556		0	#DIV/0!
**VENTX**	0.1904761905	0.1904761905	0		inf	#DIV/0!
**/ENTXP**	0.04761904762	0.04761904762	0		inf	#DIV/0!
**VEPH1**	0.1428571429	0.1428571429	0.05555555556		2.333333333	#DIV/0!
**VEZF1**	0.09523809524	0.09523809524	0		inf	#DIV/0!
**VEZT**	0.04761904762	0.04761904762	0.05555555556		0.7	#DIV/0!
**VGF**	0.04761904762	0.04761904762	0.05555555556		0.7	#DIV/0!
**VHL**	0.09523809524	0.09523809524	0		inf	#DIV/0!
**VIL1**	0	0	0.05555555556		0	#DIV/0!
**VILL**	0.04761904762	0.04761904762	0		inf	#DIV/0!
**VIM**	0.09523809524	0.09523809524	0.1111111111		0.6842105263	#DIV/0!
**/IM-AS1**	0.09523809524	0.09523809524	0.1111111111		0.6842105263	#DIV/0!
**VIPR1**	0.04761904762	0.04761904762	0		inf	#DIV/0!
**IPR1-AS**	0.04761904762	0.04761904762	0		inf	#DIV/0!
**VIPR2**	0.1428571429	0.1428571429	0.05555555556		2.333333333	#DIV/0!
**VIRMA**	0.1904761905	0.1904761905	0.1111111111		1.529411765	#DIV/0!
**VIT**	0	0	0.05555555556		0	#DIV/0!
**/KORC1**	0.04761904762	0.04761904762	0		inf	#DIV/0!
**KORC1L**	0.04761904762	0.04761904762	0.1111111111		0.325	#DIV/0!
**VLDLR**	0.04761904762	0.04761904762	0.05555555556		0.7	#DIV/0!
**LDLR-A**	0.04761904762	0.04761904762	0.05555555556		0.7	#DIV/0!
**VMAC**	0.1428571429	0.1428571429	0.05555555556		2.333333333	#DIV/0!
**VMO1**	0	0	0.1111111111		0	#DIV/0!
**VMP1**	0.1428571429	0.1428571429	0		inf	#DIV/0!
**VN1R1**	0.1428571429	0.1428571429	0.05555555556		2.333333333	#DIV/0!
**VN1R2**	0.09523809524	0.09523809524	0.05555555556		1.473684211	#DIV/0!
**VN1R3**	0.04761904762	0.04761904762	0		inf	#DIV/0!
**VN1R4**	0.09523809524	0.09523809524	0.05555555556		1.473684211	#DIV/0!
**VN1R5**	0.1428571429	0.1428571429	0		inf	#DIV/0!
**VOPP1**	0.3333333333	0.3333333333	0.2222222222		1.375	#DIV/0!
**/PREB1**	0.04761904762	0.04761904762	0.05555555556		0.7	#DIV/0!
**VPS13A**	0	0	0.05555555556		0	#DIV/0!
**PS13A-A**	0	0	0.05555555556		0	#DIV/0!
**VPS13B**	0.1904761905	0.1904761905	0.1111111111		1.529411765	#DIV/0!
**VPS13C**	0.04761904762	0.04761904762	0.05555555556		0.7	#DIV/0!
**VPS13D**	0.04761904762	0.04761904762	0.1111111111		0.325	#DIV/0!
**VPS16**	0.1904761905	0.1904761905	0.05555555556		3.294117647	#DIV/0!
**VPS25**	0.04761904762	0.04761904762	0		inf	#DIV/0!
**VPS26A**	0.1904761905	0.1904761905	0.05555555556		3.294117647	#DIV/0!
**VPS26C**	0.04761904762	0.04761904762	0.1666666667		0.2	#DIV/0!
**VPS28**	0.04761904762	0.04761904762	0.1666666667		0.2	#DIV/0!
**VPS29**	0	0	0.1111111111		0	#DIV/0!
**VPS33A**	0.04761904762	0.04761904762	0.1111111111		0.325	#DIV/0!
**VPS33B**	0.04761904762	0.04761904762	0.05555555556		0.7	#DIV/0!
**PS33B-D**	0.04761904762	0.04761904762	0.05555555556		0.7	#DIV/0!
**VPS35**	0.04761904762	0.04761904762	0		inf	#DIV/0!
**VPS35L**	0.04761904762	0.04761904762	0		inf	#DIV/0!
**VPS37A**	0.09523809524	0.09523809524	0.1111111111		0.6842105263	#DIV/0!

VPS37B	0.04761904762	0.04761904762	0.1111111111		0.325	#DIV/0!
VPS37D	0.1428571429	0.1428571429	0.1111111111		1.083333333	#DIV/0!
VPS41	0.04761904762	0.04761904762	0.2222222222		0.1375	#DIV/0!
VPS45	0.1904761905	0.1904761905	0.05555555556		3.294117647	#DIV/0!
VPS4A	0.1428571429	0.1428571429	0.05555555556		2.333333333	#DIV/0!
VPS4B	0.04761904762	0.04761904762	0		inf	#DIV/0!
VPS50	0.04761904762	0.04761904762	0.1111111111		0.325	#DIV/0!
VPS53	0	0	0.1111111111		0	#DIV/0!
VPS54	0	0	0.05555555556		0	#DIV/0!
VPS72	0.1904761905	0.1904761905	0.05555555556		3.294117647	#DIV/0!
VPS8	0.1428571429	0.1428571429	0.05555555556		2.333333333	#DIV/0!
VPS9D1	0.09523809524	0.09523809524	0		inf	#DIV/0!
PS9D1-A	0.09523809524	0.09523809524	0		inf	#DIV/0!
VRK2	0.04761904762	0.04761904762	0.05555555556		0.7	#DIV/0!
VRK3	0.09523809524	0.09523809524	0.1111111111		0.6842105263	#DIV/0!
VRTN	0.04761904762	0.04761904762	0		inf	#DIV/0!
VSIG10	0	0	0.05555555556		0	#DIV/0!
VSIR	0.09523809524	0.09523809524	0		inf	#DIV/0!
VSNL1	0	0	0.05555555556		0	#DIV/0!
VSTM1	0.09523809524	0.09523809524	0.05555555556		1.473684211	#DIV/0!
VSTM2A	0.380952381	0.380952381	0.1666666667		2.461538462	#DIV/0!
TM2A-O	0.380952381	0.380952381	0.1666666667		2.461538462	#DIV/0!
VSTM2B	0.04761904762	0.04761904762	0.1666666667		0.2	#DIV/0!
VSTM2L	0.1428571429	0.1428571429	0.1666666667		0.6666666667	#DIV/0!
VSTM4	0.09523809524	0.09523809524	0.05555555556		1.473684211	#DIV/0!
VSX1	0.1904761905	0.1904761905	0.05555555556		3.294117647	#DIV/0!
VSX2	0.04761904762	0.04761904762	0		inf	#DIV/0!
VTCN1	0.04761904762	0.04761904762	0.05555555556		0.7	#DIV/0!
VTI1A	0.1428571429	0.1428571429	0.05555555556		2.333333333	#DIV/0!
VTN	0.04761904762	0.04761904762	0.05555555556		0.7	#DIV/0!
TRNA1-	0.04761904762	0.04761904762	0		inf	#DIV/0!
TRNA1-	0.04761904762	0.04761904762	0		inf	#DIV/0!
TRNA1-	0.04761904762	0.04761904762	0		inf	#DIV/0!
TRNA2-	0.04761904762	0.04761904762	0		inf	#DIV/0!
VWA1	0	0	0.1111111111		0	#DIV/0!
VWA2	0.1904761905	0.1904761905	0.05555555556		3.294117647	#DIV/0!
VWA3A	0.04761904762	0.04761904762	0		inf	#DIV/0!
VWA3B	0	0	0.05555555556		0	#DIV/0!
VWA5B1	0.04761904762	0.04761904762	0.1111111111		0.325	#DIV/0!
VWA5B2	0.1428571429	0.1428571429	0.05555555556		2.333333333	#DIV/0!
VWA7	0.04761904762	0.04761904762	0		inf	#DIV/0!
VWC2	0.09523809524	0.09523809524	0.2222222222		0.2894736842	#DIV/0!
VWC2L	0	0	0.05555555556		0	#DIV/0!
WC2L-IT	0	0	0.05555555556		0	#DIV/0!
VWDE	0.04761904762	0.04761904762	0.2222222222		0.1375	#DIV/0!
VWF	0.04761904762	0.04761904762	0.1111111111		0.325	#DIV/0!
VXN	0.1428571429	0.1428571429	0.1666666667		0.6666666667	#DIV/0!
WAC	0.09523809524	0.09523809524	0.1111111111		0.6842105263	#DIV/0!
VAC-AS	0.09523809524	0.09523809524	0.1111111111		0.6842105263	#DIV/0!
WAPL	0.1428571429	0.1428571429	0.05555555556		2.333333333	#DIV/0!
WARS2	0.04761904762	0.04761904762	0.05555555556		0.7	#DIV/0!

ARS2-A	0.04761904762	0.04761904762	0.05555555556		0.7	#DIV/0!
ARS2-IT	0.04761904762	0.04761904762	0.05555555556		0.7	#DIV/0!
WASF1	0	0	0			#DIV/0!
WASF2	0.04761904762	0.04761904762	0.1111111111		0.325	#DIV/0!
WASH2I	0	0	0.05555555556		0	#DIV/0!
WASH3I	0.04761904762	0.04761904762	0.05555555556		0.7	#DIV/0!
WASH5I	0.09523809524	0.09523809524	0.1111111111		0.6842105263	#DIV/0!
WASH7I	0.04761904762	0.04761904762	0.1666666667		0.2	#DIV/0!
WASH8I	0.04761904762	0.04761904762	0.1111111111		0.325	#DIV/0!
WASHC	0.04761904762	0.04761904762	0.05555555556		0.7	#DIV/0!
VASHC2	0.04761904762	0.04761904762	0.05555555556		0.7	#DIV/0!
VASHC2	0.1428571429	0.1428571429	0.05555555556		2.333333333	#DIV/0!
WASHC	0.04761904762	0.04761904762	0.05555555556		0.7	#DIV/0!
WASHC	0.04761904762	0.04761904762	0.05555555556		0.7	#DIV/0!
WASHC	0.1428571429	0.1428571429	0.1111111111		1.083333333	#DIV/0!
WASIR2	0.04761904762	0.04761904762	0.2222222222		0.1375	#DIV/0!
WASL	0.04761904762	0.04761904762	0.05555555556		0.7	#DIV/0!
WBP1	0	0	0.05555555556		0	#DIV/0!
WBP11	0.04761904762	0.04761904762	0.1111111111		0.325	#DIV/0!
VBP11P	0.09523809524	0.09523809524	0		inf	#DIV/0!
WBP1L	0.1428571429	0.1428571429	0.05555555556		2.333333333	#DIV/0!
WBP2	0.04761904762	0.04761904762	0		inf	#DIV/0!
VBP2NI	0	0	0.1111111111		0	#DIV/0!
WDFY1	0	0	0.05555555556		0	#DIV/0!
WDFY3	0	0	0.05555555556		0	#DIV/0!
DFY3-A	0	0	0.05555555556		0	#DIV/0!
WDFY4	0.09523809524	0.09523809524	0.05555555556		1.473684211	#DIV/0!
WDHD1	0.04761904762	0.04761904762	0		inf	#DIV/0!
WDPCP	0	0	0.05555555556		0	#DIV/0!
WDR11	0.2380952381	0.2380952381	0		inf	#DIV/0!
DR11-A	0.2380952381	0.2380952381	0		inf	#DIV/0!
WDR12	0.09523809524	0.09523809524	0.05555555556		1.473684211	#DIV/0!
WDR17	0	0	0.05555555556		0	#DIV/0!
WDR18	0.09523809524	0.09523809524	0.1111111111		0.6842105263	#DIV/0!
WDR19	0.1904761905	0.1904761905	0.05555555556		3.294117647	#DIV/0!
WDR24	0.04761904762	0.04761904762	0.2222222222		0.1375	#DIV/0!
WDR26	0.1428571429	0.1428571429	0.05555555556		2.333333333	#DIV/0!
WDR3	0.04761904762	0.04761904762	0.05555555556		0.7	#DIV/0!
WDR31	0.04761904762	0.04761904762	0.05555555556		0.7	#DIV/0!
WDR33	0	0	0.05555555556		0	#DIV/0!
WDR34	0.09523809524	0.09523809524	0.05555555556		1.473684211	#DIV/0!
WDR35	0	0	0.05555555556		0	#DIV/0!
WDR36	0.04761904762	0.04761904762	0		inf	#DIV/0!
WDR37	0.04761904762	0.04761904762	0.1111111111		0.325	#DIV/0!
WDR38	0.09523809524	0.09523809524	0.05555555556		1.473684211	#DIV/0!
WDR4	0.04761904762	0.04761904762	0.1666666667		0.2	#DIV/0!
WDR41	0.04761904762	0.04761904762	0		inf	#DIV/0!
WDR43	0	0	0.05555555556		0	#DIV/0!
VDR45E	0	0	0			#DIV/0!
WDR47	0.04761904762	0.04761904762	0.05555555556		0.7	#DIV/0!
WDR48	0.04761904762	0.04761904762	0		inf	#DIV/0!

**WDR49**	0.1428571429	0.1428571429	0.05555555556		2.333333333	#DIV/0!
**WDR5**	0.04761904762	0.04761904762	0		inf	#DIV/0!
**WDR53**	0.1428571429	0.1428571429	0.05555555556		2.333333333	#DIV/0!
**WDR54**	0	0	0.05555555556		0	#DIV/0!
**WDR55**	0.04761904762	0.04761904762	0		inf	#DIV/0!
**WDR59**	0.04761904762	0.04761904762	0		inf	#DIV/0!
**WDR5B**	0.09523809524	0.09523809524	0.05555555556		1.473684211	#DIV/0!
**WDR6**	0.09523809524	0.09523809524	0		inf	#DIV/0!
**WDR60**	0.1428571429	0.1428571429	0.05555555556		2.333333333	#DIV/0!
**WDR61**	0.04761904762	0.04761904762	0.05555555556		0.7	#DIV/0!
**WDR62**	0.04761904762	0.04761904762	0.05555555556		0.7	#DIV/0!
**WDR63**	0.04761904762	0.04761904762	0.05555555556		0.7	#DIV/0!
**WDR64**	0.1428571429	0.1428571429	0		inf	#DIV/0!
**WDR66**	0.04761904762	0.04761904762	0.1111111111		0.325	#DIV/0!
**WDR7**	0.09523809524	0.09523809524	0		inf	#DIV/0!
**WDR70**	0.09523809524	0.09523809524	0.05555555556		1.473684211	#DIV/0!
**WDR72**	0.04761904762	0.04761904762	0.05555555556		0.7	#DIV/0!
**WDR73**	0.04761904762	0.04761904762	0.05555555556		0.7	#DIV/0!
**WDR75**	0	0	0.1666666667		0	#DIV/0!
**WDR77**	0.04761904762	0.04761904762	0		inf	#DIV/0!
**WDR78**	0.04761904762	0.04761904762	0.05555555556		0.7	#DIV/0!
**WDR81**	0	0	0.1111111111		0	#DIV/0!
**WDR83**	0.1428571429	0.1428571429	0.1111111111		1.083333333	#DIV/0!
**VDR83O**	0.1428571429	0.1428571429	0.1111111111		1.083333333	#DIV/0!
**WDR86**	0.09523809524	0.09523809524	0.05555555556		1.473684211	#DIV/0!
**DR86-A**	0.09523809524	0.09523809524	0.05555555556		1.473684211	#DIV/0!
**WDR87**	0.04761904762	0.04761904762	0.05555555556		0.7	#DIV/0!
**WDR88**	0.04761904762	0.04761904762	0.05555555556		0.7	#DIV/0!
**WDR89**	0.09523809524	0.09523809524	0		inf	#DIV/0!
**WDR90**	0.04761904762	0.04761904762	0.2222222222		0.1375	#DIV/0!
**WDR91**	0.04761904762	0.04761904762	0.05555555556		0.7	#DIV/0!
**WDR92**	0	0	0.05555555556		0	#DIV/0!
**WDR93**	0.04761904762	0.04761904762	0.05555555556		0.7	#DIV/0!
**WDR97**	0.04761904762	0.04761904762	0.1666666667		0.2	#DIV/0!
**VDSUB1**	0	0	0.05555555556		0	#DIV/0!
**WDTC1**	0.04761904762	0.04761904762	0.1111111111		0.325	#DIV/0!
**VDYHV**	0.1428571429	0.1428571429	0.1111111111		1.083333333	#DIV/0!
**WEE1**	0.04761904762	0.04761904762	0.05555555556		0.7	#DIV/0!
**WEE2**	0.04761904762	0.04761904762	0.1111111111		0.325	#DIV/0!
**VEE2-AS**	0.04761904762	0.04761904762	0.1111111111		0.325	#DIV/0!
**WFDC1**	0.04761904762	0.04761904762	0		inf	#DIV/0!
**VFDC10**	0.1428571429	0.1428571429	0.1666666667		0.6666666667	#DIV/0!
**VFDC10**	0.1428571429	0.1428571429	0.1666666667		0.6666666667	#DIV/0!
**WFDC11**	0.1428571429	0.1428571429	0.1666666667		0.6666666667	#DIV/0!
**WFDC12**	0.1428571429	0.1428571429	0.1666666667		0.6666666667	#DIV/0!
**WFDC13**	0.1428571429	0.1428571429	0.1666666667		0.6666666667	#DIV/0!
**WFDC2**	0.1428571429	0.1428571429	0.1666666667		0.6666666667	#DIV/0!
**VFDC21**	0.1428571429	0.1428571429	0		inf	#DIV/0!
**WFDC3**	0.1428571429	0.1428571429	0.1666666667		0.6666666667	#DIV/0!
**WFDC5**	0.1428571429	0.1428571429	0.1666666667		0.6666666667	#DIV/0!
**WFDC6**	0.1428571429	0.1428571429	0.1666666667		0.6666666667	#DIV/0!

WFDC8	0.1428571429	0.1428571429	0.1666666667		0.6666666667	#DIV/0!
WFDC9	0.1428571429	0.1428571429	0.1666666667		0.6666666667	#DIV/0!
VFIKKN	0.04761904762	0.04761904762	0.2222222222		0.1375	#DIV/0!
VFIKKN	0.09523809524	0.09523809524	0		inf	#DIV/0!
VHAMM	0.04761904762	0.04761904762	0.05555555556		0.7	#DIV/0!
HAMM	0.09523809524	0.09523809524	0		inf	#DIV/0!
WHRN	0.04761904762	0.04761904762	0.05555555556		0.7	#DIV/0!
WIF1	0.09523809524	0.09523809524	0.05555555556		1.473684211	#DIV/0!
WIPF1	0	0	0.1111111111		0	#DIV/0!
WIPF2	0.09523809524	0.09523809524	0		inf	#DIV/0!
WIPF3	0.04761904762	0.04761904762	0.1666666667		0.2	#DIV/0!
WIPI1	0	0	0			#DIV/0!
WIPI2	0.04761904762	0.04761904762	0.1666666667		0.2	#DIV/0!
WLS	0.04761904762	0.04761904762	0.05555555556		0.7	#DIV/0!
WNK1	0.04761904762	0.04761904762	0.1111111111		0.325	#DIV/0!
WNK2	0.04761904762	0.04761904762	0.05555555556		0.7	#DIV/0!
WNK4	0.04761904762	0.04761904762	0		inf	#DIV/0!
WNT1	0.04761904762	0.04761904762	0.05555555556		0.7	#DIV/0!
VNT10A	0	0	0.05555555556		0	#DIV/0!
VNT10B	0.04761904762	0.04761904762	0.05555555556		0.7	#DIV/0!
WNT16	0.04761904762	0.04761904762	0.05555555556		0.7	#DIV/0!
WNT2	0.09523809524	0.09523809524	0.05555555556		1.473684211	#DIV/0!
WNT2B	0.04761904762	0.04761904762	0.05555555556		0.7	#DIV/0!
WNT3	0.1428571429	0.1428571429	0		inf	#DIV/0!
WNT3A	0.1428571429	0.1428571429	0		inf	#DIV/0!
WNT4	0.04761904762	0.04761904762	0.1111111111		0.325	#DIV/0!
WNT5A	0.04761904762	0.04761904762	0		inf	#DIV/0!
WNT5B	0.04761904762	0.04761904762	0.1111111111		0.325	#DIV/0!
WNT6	0	0	0.05555555556		0	#DIV/0!
WNT7A	0.04761904762	0.04761904762	0		inf	#DIV/0!
WNT7B	0	0	0.1111111111		0	#DIV/0!
WNT8A	0.04761904762	0.04761904762	0		inf	#DIV/0!
WNT8B	0.1428571429	0.1428571429	0		inf	#DIV/0!
WNT9A	0.1428571429	0.1428571429	0		inf	#DIV/0!
WNT9B	0.1428571429	0.1428571429	0		inf	#DIV/0!
VRAP53	0	0	0.05555555556		0	#DIV/0!
VRAP73	0	0	0.05555555556		0	#DIV/0!
WRB	0.04761904762	0.04761904762	0.1111111111		0.325	#DIV/0!
B-SH3B	0.04761904762	0.04761904762	0.1111111111		0.325	#DIV/0!
WRN	0.04761904762	0.04761904762	0.1111111111		0.325	#DIV/0!
WSB1	0.04761904762	0.04761904762	0.05555555556		0.7	#DIV/0!
WSB2	0	0	0.05555555556		0	#DIV/0!
WSCD1	0	0	0.05555555556		0	#DIV/0!
WSCD2	0.04761904762	0.04761904762	0.05555555556		0.7	#DIV/0!
WSPAR	0.04761904762	0.04761904762	0		inf	#DIV/0!
WTIP	0.04761904762	0.04761904762	0.05555555556		0.7	#DIV/0!
WWC1	0	0	0.05555555556		0	#DIV/0!
WWC2	0	0	0.05555555556		0	#DIV/0!
WC2-AS	0	0	0.05555555556		0	#DIV/0!
WC2-AS	0	0	0.05555555556		0	#DIV/0!
WWOX	0.09523809524	0.09523809524	0		inf	#DIV/0!

WWP1	0.1904761905	0.1904761905	0.1111111111		1.529411765	#DIV/0!
WWP2	0.1428571429	0.1428571429	0.05555555556		2.333333333	#DIV/0!
WWTR1	0.1904761905	0.1904761905	0.1111111111		1.529411765	#DIV/0!
WTR1-A	0.1904761905	0.1904761905	0.1111111111		1.529411765	#DIV/0!
XAB2	0.1428571429	0.1428571429	0.05555555556		2.333333333	#DIV/0!
XAF1	0	0	0.05555555556		0	#DIV/0!
XBP1	0.04761904762	0.04761904762	0.1111111111		0.325	#DIV/0!
XCL1	0.1428571429	0.1428571429	0		inf	#DIV/0!
XCL2	0.1428571429	0.1428571429	0		inf	#DIV/0!
XDH	0	0	0.05555555556		0	#DIV/0!
XIRP1	0.04761904762	0.04761904762	0		inf	#DIV/0!
XIRP2	0	0	0.05555555556		0	#DIV/0!
IRP2-AS	0	0	0.05555555556		0	#DIV/0!
XKR3	0.04761904762	0.04761904762	0		inf	#DIV/0!
XKR4	0.1428571429	0.1428571429	0.1111111111		1.083333333	#DIV/0!
XKR5	0.09523809524	0.09523809524	0.1111111111		0.6842105263	#DIV/0!
XKR6	0.09523809524	0.09523809524	0.1111111111		0.6842105263	#DIV/0!
XKR7	0.09523809524	0.09523809524	0.1111111111		0.6842105263	#DIV/0!
XKR8	0.04761904762	0.04761904762	0.1111111111		0.325	#DIV/0!
XKR9	0.1904761905	0.1904761905	0.1666666667		0.9411764706	#DIV/0!
OC_007	0.09523809524	0.09523809524	0.05555555556		1.473684211	#DIV/0!
OC_008	0.1428571429	0.1428571429	0.1111111111		1.083333333	#DIV/0!
OC_009	0.04761904762	0.04761904762	0.1111111111		0.325	#DIV/0!
XPA	0.04761904762	0.04761904762	0		inf	#DIV/0!
XPC	0.04761904762	0.04761904762	0		inf	#DIV/0!
XPNPEP	0.1428571429	0.1428571429	0.05555555556		2.333333333	#DIV/0!
XPNPEP	0	0	0.1111111111		0	#DIV/0!
XPO1	0.04761904762	0.04761904762	0.05555555556		0.7	#DIV/0!
XPO4	0.04761904762	0.04761904762	0		inf	#DIV/0!
XPO5	0.04761904762	0.04761904762	0.05555555556		0.7	#DIV/0!
XPO6	0.04761904762	0.04761904762	0		inf	#DIV/0!
XPO7	0.09523809524	0.09523809524	0.1111111111		0.6842105263	#DIV/0!
XPOT	0.1428571429	0.1428571429	0.05555555556		2.333333333	#DIV/0!
XPR1	0.1428571429	0.1428571429	0		inf	#DIV/0!
XRCC1	0.09523809524	0.09523809524	0.05555555556		1.473684211	#DIV/0!
XRCC2	0.09523809524	0.09523809524	0.05555555556		1.473684211	#DIV/0!
XRCC4	0.04761904762	0.04761904762	0.05555555556		0.7	#DIV/0!
XRCC5	0	0	0.05555555556		0	#DIV/0!
XRCC6	0	0	0.1111111111		0	#DIV/0!
XRN1	0.2380952381	0.2380952381	0.05555555556		4.375	#DIV/0!
XRN2	0.1904761905	0.1904761905	0.05555555556		3.294117647	#DIV/0!
XXYLT1	0.1428571429	0.1428571429	0.05555555556		2.333333333	#DIV/0!
XYLT1-A	0.09523809524	0.09523809524	0.05555555556		1.473684211	#DIV/0!
XYLT1-A	0.09523809524	0.09523809524	0.05555555556		1.473684211	#DIV/0!
XYLB	0.04761904762	0.04761904762	0		inf	#DIV/0!
XYLT1	0.04761904762	0.04761904762	0		inf	#DIV/0!
XYLT2	0.09523809524	0.09523809524	0		inf	#DIV/0!
YAE1	0.04761904762	0.04761904762	0.2222222222		0.1375	#DIV/0!
YAF2	0.04761904762	0.04761904762	0.1111111111		0.325	#DIV/0!
YARS	0.04761904762	0.04761904762	0.1111111111		0.325	#DIV/0!
YARS2	0.1428571429	0.1428571429	0.1111111111		1.083333333	#DIV/0!

YBEY	0.04761904762	0.04761904762	0.1111111111		0.325	#DIV/0!
YBX1	0.04761904762	0.04761904762	0.05555555556		0.7	#DIV/0!
YBX2	0	0	0.05555555556		0	#DIV/0!
YBX3	0.04761904762	0.04761904762	0.1111111111		0.325	#DIV/0!
YBX3P1	0.04761904762	0.04761904762	0		inf	#DIV/0!
YDJC	0.04761904762	0.04761904762	0.05555555556		0.7	#DIV/0!
YEATS2	0.1904761905	0.1904761905	0.05555555556		3.294117647	#DIV/0!
ATS2-A	0.1904761905	0.1904761905	0.05555555556		3.294117647	#DIV/0!
YEATS4	0.09523809524	0.09523809524	0.1111111111		0.6842105263	#DIV/0!
YES1	0.09523809524	0.09523809524	0		inf	#DIV/0!
YIF1B	0.04761904762	0.04761904762	0.05555555556		0.7	#DIV/0!
YIPF1	0.04761904762	0.04761904762	0.05555555556		0.7	#DIV/0!
YIPF2	0.1428571429	0.1428571429	0.1111111111		1.083333333	#DIV/0!
YIPF3	0.04761904762	0.04761904762	0.05555555556		0.7	#DIV/0!
YIPF4	0.1428571429	0.1428571429	0		inf	#DIV/0!
YIPF7	0.04761904762	0.04761904762	0.05555555556		0.7	#DIV/0!
YJEFN3	0.1428571429	0.1428571429	0.1111111111		1.083333333	#DIV/0!
YJU2	0.09523809524	0.09523809524	0.05555555556		1.473684211	#DIV/0!
YKT6	0.04761904762	0.04761904762	0.1666666667		0.2	#DIV/0!
YME1L1	0.09523809524	0.09523809524	0.1111111111		0.6842105263	#DIV/0!
YOD1	0.1428571429	0.1428571429	0		inf	#DIV/0!
YPEL1	0.04761904762	0.04761904762	0.05555555556		0.7	#DIV/0!
YPEL2	0.1428571429	0.1428571429	0		inf	#DIV/0!
YPEL3	0.04761904762	0.04761904762	0		inf	#DIV/0!
YPEL5	0	0	0.05555555556		0	#DIV/0!
YRDC	0.04761904762	0.04761904762	0.05555555556		0.7	#DIV/0!
YTHDC1	0.04761904762	0.04761904762	0.05555555556		0.7	#DIV/0!
YTHDC2	0.04761904762	0.04761904762	0		inf	#DIV/0!
YTHDF1	0.1428571429	0.1428571429	0.2222222222		0.4583333333	#DIV/0!
YTHDF2	0.04761904762	0.04761904762	0.1111111111		0.325	#DIV/0!
YTHDF3	0.1428571429	0.1428571429	0.1666666667		0.6666666667	#DIV/0!
HDF3-A	0.1428571429	0.1428571429	0.1666666667		0.6666666667	#DIV/0!
YWHAB	0.1428571429	0.1428571429	0.1666666667		0.6666666667	#DIV/0!
YWHAE	0	0	0.1111111111		0	#DIV/0!
WHAEP	0.04761904762	0.04761904762	0.05555555556		0.7	#DIV/0!
YWHAG	0.09523809524	0.09523809524	0.1111111111		0.6842105263	#DIV/0!
YWHAH	0.04761904762	0.04761904762	0.1111111111		0.325	#DIV/0!
YWHAQ	0	0	0.05555555556		0	#DIV/0!
YWHAZ	0.1904761905	0.1904761905	0.1111111111		1.529411765	#DIV/0!
YY1AP1	0.1904761905	0.1904761905	0.1111111111		1.529411765	#DIV/0!
YY1P2	0	0	0.05555555556		0	#DIV/0!
ZACN	0.04761904762	0.04761904762	0		inf	#DIV/0!
ZADH2	0.09523809524	0.09523809524	0		inf	#DIV/0!
ZAN	0.04761904762	0.04761904762	0.05555555556		0.7	#DIV/0!
ZAP70	0	0	0.05555555556		0	#DIV/0!
ZAR1	0.04761904762	0.04761904762	0.1111111111		0.325	#DIV/0!
ZASP	0.04761904762	0.04761904762	0.05555555556		0.7	#DIV/0!
ZBBX	0.1428571429	0.1428571429	0.05555555556		2.333333333	#DIV/0!
ZBED2	0.09523809524	0.09523809524	0.05555555556		1.473684211	#DIV/0!
ZBED3	0.04761904762	0.04761904762	0		inf	#DIV/0!
BED3-AS	0.04761904762	0.04761904762	0		inf	#DIV/0!

**ZBED4**	0	0	0.1111111111		0	#DIV/0!
**ZBED6**	0.2380952381	0.2380952381	0		inf	#DIV/0!
**BED6C**	0.09523809524	0.09523809524	0.05555555556		1.473684211	#DIV/0!
**ZBED8**	0.04761904762	0.04761904762	0.05555555556		0.7	#DIV/0!
**ZBP1**	0.1428571429	0.1428571429	0.2222222222		0.4583333333	#DIV/0!
**ZBTB1**	0.09523809524	0.09523809524	0		inf	#DIV/0!
**ZBTB10**	0.1428571429	0.1428571429	0.1111111111		1.083333333	#DIV/0!
**ZBTB11**	0.09523809524	0.09523809524	0.05555555556		1.473684211	#DIV/0!
**TB11-A**	0.09523809524	0.09523809524	0.05555555556		1.473684211	#DIV/0!
**ZBTB12**	0.04761904762	0.04761904762	0		inf	#DIV/0!
**ZBTB14**	0.09523809524	0.09523809524	0		inf	#DIV/0!
**ZBTB17**	0.04761904762	0.04761904762	0.1111111111		0.325	#DIV/0!
**ZBTB18**	0.1428571429	0.1428571429	0.05555555556		2.333333333	#DIV/0!
**ZBTB20**	0.09523809524	0.09523809524	0.05555555556		1.473684211	#DIV/0!
**TB20-A**	0.09523809524	0.09523809524	0.05555555556		1.473684211	#DIV/0!
**TB20-A**	0.09523809524	0.09523809524	0.05555555556		1.473684211	#DIV/0!
**TB20-A**	0.09523809524	0.09523809524	0.05555555556		1.473684211	#DIV/0!
**TB20-A**	0.09523809524	0.09523809524	0.05555555556		1.473684211	#DIV/0!
**ZBTB21**	0.04761904762	0.04761904762	0.1111111111		0.325	#DIV/0!
**ZBTB22**	0.04761904762	0.04761904762	0		inf	#DIV/0!
**ZBTB24**	0	0	0			#DIV/0!
**ZBTB25**	0.09523809524	0.09523809524	0		inf	#DIV/0!
**ZBTB26**	0.09523809524	0.09523809524	0.05555555556		1.473684211	#DIV/0!
**ZBTB32**	0.04761904762	0.04761904762	0.05555555556		0.7	#DIV/0!
**ZBTB34**	0.09523809524	0.09523809524	0.05555555556		1.473684211	#DIV/0!
**ZBTB37**	0.1428571429	0.1428571429	0		inf	#DIV/0!
**ZBTB38**	0.1904761905	0.1904761905	0.05555555556		3.294117647	#DIV/0!
**ZBTB39**	0.1428571429	0.1428571429	0.05555555556		2.333333333	#DIV/0!
**ZBTB4**	0	0	0.05555555556		0	#DIV/0!
**ZBTB40**	0.04761904762	0.04761904762	0.1111111111		0.325	#DIV/0!
**ZBTB41**	0.1428571429	0.1428571429	0		inf	#DIV/0!
**ZBTB43**	0.09523809524	0.09523809524	0.05555555556		1.473684211	#DIV/0!
**ZBTB45**	0.1428571429	0.1428571429	0.05555555556		2.333333333	#DIV/0!
**ZBTB46**	0.1428571429	0.1428571429	0.2222222222		0.4583333333	#DIV/0!
**TB46-A**	0.1428571429	0.1428571429	0.2222222222		0.4583333333	#DIV/0!
**ZBTB47**	0.04761904762	0.04761904762	0		inf	#DIV/0!
**ZBTB48**	0	0	0.05555555556		0	#DIV/0!
**ZBTB5**	0.09523809524	0.09523809524	0.05555555556		1.473684211	#DIV/0!
**ZBTB6**	0.09523809524	0.09523809524	0.05555555556		1.473684211	#DIV/0!
**ZBTB7A**	0.09523809524	0.09523809524	0.05555555556		1.473684211	#DIV/0!
**ZBTB7B**	0.1904761905	0.1904761905	0.1111111111		1.529411765	#DIV/0!
**ZBTB7C**	0.04761904762	0.04761904762	0.05555555556		0.7	#DIV/0!
**ZBTB8A**	0.04761904762	0.04761904762	0.1111111111		0.325	#DIV/0!
**ZBTB8B**	0.04761904762	0.04761904762	0.1111111111		0.325	#DIV/0!
**BTB8O**	0.04761904762	0.04761904762	0.1111111111		0.325	#DIV/0!
**ZBTB9**	0.04761904762	0.04761904762	0		inf	#DIV/0!
**C2HC1**	0.1428571429	0.1428571429	0.1111111111		1.083333333	#DIV/0!
**ZC3H10**	0.09523809524	0.09523809524	0.05555555556		1.473684211	#DIV/0!
**C3H11**	0.2380952381	0.2380952381	0		inf	#DIV/0!
**C3H11**	0.1428571429	0.1428571429	0.05555555556		2.333333333	#DIV/0!
**C3H12**	0.04761904762	0.04761904762	0.05555555556		0.7	#DIV/0!

ZC3H12I	0	0	0			#DIV/0!
ZC3H15	0	0	0.05555555556		0	#DIV/0!
ZC3H18	0.1428571429	0.1428571429	0		inf	#DIV/0!
C3H18-A	0.1428571429	0.1428571429	0		inf	#DIV/0!
ZC3H3	0.04761904762	0.04761904762	0.1111111111		0.325	#DIV/0!
ZC3H4	0.09523809524	0.09523809524	0.1111111111		0.6842105263	#DIV/0!
ZC3H6	0	0	0.05555555556		0	#DIV/0!
ZC3H7A	0.04761904762	0.04761904762	0		inf	#DIV/0!
ZC3H7B	0	0	0.1111111111		0	#DIV/0!
ZC3H8	0	0	0.05555555556		0	#DIV/0!
ZC3HAV	0.04761904762	0.04761904762	0.05555555556		0.7	#DIV/0!
C3HAV1	0.04761904762	0.04761904762	0.05555555556		0.7	#DIV/0!
ZC3HC1	0.04761904762	0.04761904762	0.05555555556		0.7	#DIV/0!
CCHC1	0.04761904762	0.04761904762	0		inf	#DIV/0!
CCHC1	0.04761904762	0.04761904762	0		inf	#DIV/0!
CCHC1	0.04761904762	0.04761904762	0.1111111111		0.325	#DIV/0!
ZCCHC2	0.09523809524	0.09523809524	0		inf	#DIV/0!
CCHC2	0	0	0.05555555556		0	#DIV/0!
CCHC2	0.1428571429	0.1428571429	0.05555555556		2.333333333	#DIV/0!
ZCCHC3	0.1904761905	0.1904761905	0.05555555556		3.294117647	#DIV/0!
ZCCHC7	0.09523809524	0.09523809524	0.05555555556		1.473684211	#DIV/0!
ZCCHC8	0.04761904762	0.04761904762	0.1111111111		0.325	#DIV/0!
ZCCHC9	0.04761904762	0.04761904762	0		inf	#DIV/0!
ZCRB1	0.04761904762	0.04761904762	0.1111111111		0.325	#DIV/0!
ZCWPW	0.04761904762	0.04761904762	0.05555555556		0.7	#DIV/0!
ZCWPW	0.04761904762	0.04761904762	0		inf	#DIV/0!
ZDBF2	0	0	0.05555555556		0	#DIV/0!
ZDHHC1	0.1428571429	0.1428571429	0.05555555556		2.333333333	#DIV/0!
DHHC1	0	0	0.05555555556		0	#DIV/0!
DHHC11	0	0	0.05555555556		0	#DIV/0!
DHHC1	0.09523809524	0.09523809524	0.05555555556		1.473684211	#DIV/0!
DHHC1	0.1428571429	0.1428571429	0		inf	#DIV/0!
DHHC1	0.04761904762	0.04761904762	0.1111111111		0.325	#DIV/0!
DHHC1	0.04761904762	0.04761904762	0.1111111111		0.325	#DIV/0!
DHHC1	0.1428571429	0.1428571429	0.05555555556		2.333333333	#DIV/0!
ZDHHC2	0.09523809524	0.09523809524	0.1111111111		0.6842105263	#DIV/0!
DHHC2	0.1428571429	0.1428571429	0.05555555556		2.333333333	#DIV/0!
DHHC2	0.09523809524	0.09523809524	0.05555555556		1.473684211	#DIV/0!
ZDHHC4	0.04761904762	0.04761904762	0.1666666667		0.2	#DIV/0!
ZDHHC6	0.1428571429	0.1428571429	0.05555555556		2.333333333	#DIV/0!
ZDHHC7	0.04761904762	0.04761904762	0		inf	#DIV/0!
ZDHHC8	0.04761904762	0.04761904762	0.05555555556		0.7	#DIV/0!
DHHC8I	0	0	0.05555555556		0	#DIV/0!
ZEB1	0.09523809524	0.09523809524	0.2222222222		0.2894736842	#DIV/0!
EB1-AS	0.09523809524	0.09523809524	0.2222222222		0.2894736842	#DIV/0!
ZEB2	0	0	0.05555555556		0	#DIV/0!
EB2-AS	0	0	0.05555555556		0	#DIV/0!
ZER1	0.09523809524	0.09523809524	0.05555555556		1.473684211	#DIV/0!
ZFAND1	0.1428571429	0.1428571429	0.1111111111		1.083333333	#DIV/0!
FAND2	0.04761904762	0.04761904762	0.1666666667		0.2	#DIV/0!
ZFAND3	0.04761904762	0.04761904762	0.05555555556		0.7	#DIV/0!

ZFAND4	0.1428571429	0.1428571429	0.05555555556		2.333333333	#DIV/0!
ZFAND5	0	0	0.05555555556		0	#DIV/0!
ZFAND6	0.04761904762	0.04761904762	0.05555555556		0.7	#DIV/0!
ZFAS1	0.09523809524	0.09523809524	0.1666666667		0.4210526316	#DIV/0!
ZFAT	0.04761904762	0.04761904762	0.2222222222		0.1375	#DIV/0!
ZFAT-AS	0.04761904762	0.04761904762	0.2222222222		0.1375	#DIV/0!
ZFC3H1	0.04761904762	0.04761904762	0.1111111111		0.325	#DIV/0!
ZFHX2	0	0	0.05555555556		0	#DIV/0!
ZFHX3	0.04761904762	0.04761904762	0		inf	#DIV/0!
ZFHX4	0.1904761905	0.1904761905	0.1111111111		1.529411765	#DIV/0!
FHX4-AS	0.1904761905	0.1904761905	0.1111111111		1.529411765	#DIV/0!
ZFP1	0.04761904762	0.04761904762	0		inf	#DIV/0!
ZFP14	0.04761904762	0.04761904762	0.05555555556		0.7	#DIV/0!
ZFP2	0.09523809524	0.09523809524	0.05555555556		1.473684211	#DIV/0!
ZFP28	0.1428571429	0.1428571429	0.05555555556		2.333333333	#DIV/0!
ZFP3	0	0	0.1111111111		0	#DIV/0!
ZFP30	0.04761904762	0.04761904762	0.05555555556		0.7	#DIV/0!
ZFP36	0.04761904762	0.04761904762	0.05555555556		0.7	#DIV/0!
ZFP36L1	0	0	0.1111111111		0	#DIV/0!
ZFP36L2	0	0	0.05555555556		0	#DIV/0!
ZFP37	0.04761904762	0.04761904762	0.05555555556		0.7	#DIV/0!
ZFP42	0	0	0.05555555556		0	#DIV/0!
ZFP62	0.09523809524	0.09523809524	0.05555555556		1.473684211	#DIV/0!
ZFP64	0.09523809524	0.09523809524	0.1111111111		0.6842105263	#DIV/0!
ZFP69	0.04761904762	0.04761904762	0.05555555556		0.7	#DIV/0!
ZFP69B	0.04761904762	0.04761904762	0.05555555556		0.7	#DIV/0!
ZFP82	0.04761904762	0.04761904762	0.05555555556		0.7	#DIV/0!
ZFP90	0.1428571429	0.1428571429	0.05555555556		2.333333333	#DIV/0!
ZFPM1	0.1428571429	0.1428571429	0		inf	#DIV/0!
ZFPM2	0.1904761905	0.1904761905	0.1111111111		1.529411765	#DIV/0!
FPM2-AS	0.1904761905	0.1904761905	0.1111111111		1.529411765	#DIV/0!
ZFR	0.04761904762	0.04761904762	0.05555555556		0.7	#DIV/0!
ZFR2	0.09523809524	0.09523809524	0.05555555556		1.473684211	#DIV/0!
ZFYVE1	0.09523809524	0.09523809524	0		inf	#DIV/0!
ZFYVE1	0.04761904762	0.04761904762	0		inf	#DIV/0!
ZFYVE2	0.1428571429	0.1428571429	0		inf	#DIV/0!
ZFYVE2	0.04761904762	0.04761904762	0		inf	#DIV/0!
ZFYVE9	0.04761904762	0.04761904762	0.05555555556		0.7	#DIV/0!
ZG16	0.04761904762	0.04761904762	0		inf	#DIV/0!
ZG16B	0.04761904762	0.04761904762	0.1111111111		0.325	#DIV/0!
ZGPAT	0.1428571429	0.1428571429	0.2222222222		0.4583333333	#DIV/0!
ZGRF1	0	0	0.05555555556		0	#DIV/0!
ZHX1	0.1428571429	0.1428571429	0.1111111111		1.083333333	#DIV/0!
X1-C8or	0.1428571429	0.1428571429	0.1111111111		1.083333333	#DIV/0!
ZHX2	0.1428571429	0.1428571429	0.1111111111		1.083333333	#DIV/0!
ZHX3	0.1428571429	0.1428571429	0.1666666667		0.6666666667	#DIV/0!
ZIC1	0.1428571429	0.1428571429	0.1111111111		1.083333333	#DIV/0!
ZIC4	0.1428571429	0.1428571429	0.1111111111		1.083333333	#DIV/0!
ZIK1	0.1428571429	0.1428571429	0.05555555556		2.333333333	#DIV/0!
ZIM2	0.1428571429	0.1428571429	0.05555555556		2.333333333	#DIV/0!
IM2-AS	0.1428571429	0.1428571429	0.05555555556		2.333333333	#DIV/0!

ZIM3	0.1428571429	0.1428571429	0.05555555556		2.333333333	#DIV/0!
KSCAN	0.04761904762	0.04761904762	0.05555555556		0.7	#DIV/0!
KSCAN	0.04761904762	0.04761904762	0		inf	#DIV/0!
KSCAN	0.04761904762	0.04761904762	0.05555555556		0.7	#DIV/0!
ZMAT2	0.04761904762	0.04761904762	0		inf	#DIV/0!
ZMAT3	0.1904761905	0.1904761905	0.05555555556		3.294117647	#DIV/0!
ZMAT4	0.09523809524	0.09523809524	0.1111111111		0.6842105263	#DIV/0!
ZMAT5	0.04761904762	0.04761904762	0.1111111111		0.325	#DIV/0!
ZMIZ1	0.1428571429	0.1428571429	0.05555555556		2.333333333	#DIV/0!
MIZ1-AS	0.1428571429	0.1428571429	0.05555555556		2.333333333	#DIV/0!
ZMIZ2	0.04761904762	0.04761904762	0.2222222222		0.1375	#DIV/0!
MPSTE2	0.04761904762	0.04761904762	0.05555555556		0.7	#DIV/0!
ZMYM1	0.04761904762	0.04761904762	0.05555555556		0.7	#DIV/0!
ZMYM2	0.04761904762	0.04761904762	0		inf	#DIV/0!
ZMYM4	0.04761904762	0.04761904762	0.05555555556		0.7	#DIV/0!
IYM4-A	0.04761904762	0.04761904762	0.05555555556		0.7	#DIV/0!
ZMYM5	0.04761904762	0.04761904762	0		inf	#DIV/0!
ZMYM6	0.04761904762	0.04761904762	0.05555555556		0.7	#DIV/0!
MYND1	0.09523809524	0.09523809524	0		inf	#DIV/0!
MYND1	0.04761904762	0.04761904762	0.1111111111		0.325	#DIV/0!
MYND1	0.04761904762	0.04761904762	0.05555555556		0.7	#DIV/0!
MYND1	0	0	0.1111111111		0	#DIV/0!
MYND1	0.09523809524	0.09523809524	0		inf	#DIV/0!
ZMYND8	0.1428571429	0.1428571429	0.1666666667		0.6666666667	#DIV/0!
ZNF10	0.09523809524	0.09523809524	0.05555555556		1.473684211	#DIV/0!
ZNF100	0.09523809524	0.09523809524	0		inf	#DIV/0!
ZNF101	0.1428571429	0.1428571429	0.1111111111		1.083333333	#DIV/0!
ZNF107	0.04761904762	0.04761904762	0.05555555556		0.7	#DIV/0!
ZNF112	0.09523809524	0.09523809524	0.05555555556		1.473684211	#DIV/0!
ZNF114	0.09523809524	0.09523809524	0.1111111111		0.6842105263	#DIV/0!
ZNF117	0.04761904762	0.04761904762	0.1111111111		0.325	#DIV/0!
ZNF12	0.04761904762	0.04761904762	0.1666666667		0.2	#DIV/0!
ZNF121	0.1428571429	0.1428571429	0.1111111111		1.083333333	#DIV/0!
ZNF124	0.1428571429	0.1428571429	0		inf	#DIV/0!
ZNF131	0.04761904762	0.04761904762	0.05555555556		0.7	#DIV/0!
ZNF132	0.1428571429	0.1428571429	0.05555555556		2.333333333	#DIV/0!
ZNF133	0.1904761905	0.1904761905	0.05555555556		3.294117647	#DIV/0!
ZNF134	0.1428571429	0.1428571429	0.05555555556		2.333333333	#DIV/0!
ZNF135	0.1428571429	0.1428571429	0.05555555556		2.333333333	#DIV/0!
ZNF136	0.1428571429	0.1428571429	0.1111111111		1.083333333	#DIV/0!
ZNF137P	0.09523809524	0.09523809524	0.05555555556		1.473684211	#DIV/0!
ZNF138	0.04761904762	0.04761904762	0.05555555556		0.7	#DIV/0!
ZNF14	0.1428571429	0.1428571429	0.1111111111		1.083333333	#DIV/0!
ZNF140	0.09523809524	0.09523809524	0.05555555556		1.473684211	#DIV/0!
ZNF141	0.04761904762	0.04761904762	0		inf	#DIV/0!
ZNF142	0	0	0.05555555556		0	#DIV/0!
ZNF143	0.04761904762	0.04761904762	0.05555555556		0.7	#DIV/0!
ZNF146	0.04761904762	0.04761904762	0.05555555556		0.7	#DIV/0!
ZNF148	0.04761904762	0.04761904762	0.05555555556		0.7	#DIV/0!
ZNF154	0.1428571429	0.1428571429	0.05555555556		2.333333333	#DIV/0!
ZNF155	0.09523809524	0.09523809524	0.05555555556		1.473684211	#DIV/0!

**ZNF16**	0.09523809524	0.09523809524	0.2222222222		0.2894736842	#DIV/0!
**ZNF160**	0.09523809524	0.09523809524	0.05555555556		1.473684211	#DIV/0!
**ZNF169**	0.04761904762	0.04761904762	0.05555555556		0.7	#DIV/0!
**ZNF17**	0.1428571429	0.1428571429	0.05555555556		2.333333333	#DIV/0!
**ZNF174**	0.04761904762	0.04761904762	0.1111111111		0.325	#DIV/0!
**ZNF177**	0.1428571429	0.1428571429	0.05555555556		2.333333333	#DIV/0!
**ZNF18**	0.04761904762	0.04761904762	0.05555555556		0.7	#DIV/0!
**ZNF180**	0.09523809524	0.09523809524	0.05555555556		1.473684211	#DIV/0!
**ZNF181**	0.04761904762	0.04761904762	0.05555555556		0.7	#DIV/0!
**ZNF189**	0.04761904762	0.04761904762	0		inf	#DIV/0!
**ZNF19**	0.04761904762	0.04761904762	0		inf	#DIV/0!
**ZNF195**	0.04761904762	0.04761904762	0		inf	#DIV/0!
**ZNF2**	0	0	0.1111111111		0	#DIV/0!
**ZNF20**	0.1428571429	0.1428571429	0.1111111111		1.083333333	#DIV/0!
**ZNF200**	0.04761904762	0.04761904762	0.1111111111		0.325	#DIV/0!
**ZNF205**	0.04761904762	0.04761904762	0.1111111111		0.325	#DIV/0!
**NF205-A**	0.04761904762	0.04761904762	0.1111111111		0.325	#DIV/0!
**ZNF207**	0	0	0			#DIV/0!
**ZNF208**	0.09523809524	0.09523809524	0		inf	#DIV/0!
**ZNF211**	0.1428571429	0.1428571429	0.05555555556		2.333333333	#DIV/0!
**ZNF212**	0.09523809524	0.09523809524	0.05555555556		1.473684211	#DIV/0!
**ZNF213**	0.04761904762	0.04761904762	0.1111111111		0.325	#DIV/0!
**NF213-A**	0.04761904762	0.04761904762	0.1111111111		0.325	#DIV/0!
**ZNF214**	0.04761904762	0.04761904762	0		inf	#DIV/0!
**ZNF215**	0.04761904762	0.04761904762	0		inf	#DIV/0!
**ZNF217**	0.09523809524	0.09523809524	0.1111111111		0.6842105263	#DIV/0!
**ZNF22**	0.1428571429	0.1428571429	0.05555555556		2.333333333	#DIV/0!
**ZNF221**	0.09523809524	0.09523809524	0.05555555556		1.473684211	#DIV/0!
**ZNF222**	0.09523809524	0.09523809524	0.05555555556		1.473684211	#DIV/0!
**ZNF223**	0.09523809524	0.09523809524	0.05555555556		1.473684211	#DIV/0!
**ZNF224**	0.09523809524	0.09523809524	0.05555555556		1.473684211	#DIV/0!
**ZNF225**	0.09523809524	0.09523809524	0.05555555556		1.473684211	#DIV/0!
**ZNF226**	0.09523809524	0.09523809524	0.05555555556		1.473684211	#DIV/0!
**ZNF227**	0.09523809524	0.09523809524	0.05555555556		1.473684211	#DIV/0!
**ZNF229**	0.09523809524	0.09523809524	0.05555555556		1.473684211	#DIV/0!
**ZNF23**	0.04761904762	0.04761904762	0		inf	#DIV/0!
**ZNF230**	0.09523809524	0.09523809524	0.05555555556		1.473684211	#DIV/0!
**ZNF232**	0	0	0.1111111111		0	#DIV/0!
**ZNF233**	0.09523809524	0.09523809524	0.05555555556		1.473684211	#DIV/0!
**ZNF234**	0.09523809524	0.09523809524	0.05555555556		1.473684211	#DIV/0!
**ZNF235**	0.09523809524	0.09523809524	0.05555555556		1.473684211	#DIV/0!
**ZNF236**	0.09523809524	0.09523809524	0.05555555556		1.473684211	#DIV/0!
**NF236-D**	0.09523809524	0.09523809524	0.05555555556		1.473684211	#DIV/0!
**ZNF239**	0.1428571429	0.1428571429	0.05555555556		2.333333333	#DIV/0!
**ZNF24**	0.09523809524	0.09523809524	0		inf	#DIV/0!
**ZNF248**	0.09523809524	0.09523809524	0.1111111111		0.6842105263	#DIV/0!
**ZNF25**	0.09523809524	0.09523809524	0.1111111111		0.6842105263	#DIV/0!
**ZNF250**	0.09523809524	0.09523809524	0.2222222222		0.2894736842	#DIV/0!
**ZNF251**	0.09523809524	0.09523809524	0.2222222222		0.2894736842	#DIV/0!
**ZNF252I**	0.09523809524	0.09523809524	0.2222222222		0.2894736842	#DIV/0!
**F252P-A**	0.09523809524	0.09523809524	0.2222222222		0.2894736842	#DIV/0!

ZNF253	0.1428571429	0.1428571429	0.05555555556		2.333333333	#DIV/0!
ZNF254	0.09523809524	0.09523809524	0		inf	#DIV/0!
ZNF256	0.1428571429	0.1428571429	0.05555555556		2.333333333	#DIV/0!
ZNF257	0.09523809524	0.09523809524	0		inf	#DIV/0!
ZNF26	0.09523809524	0.09523809524	0.05555555556		1.473684211	#DIV/0!
ZNF260	0.04761904762	0.04761904762	0.05555555556		0.7	#DIV/0!
ZNF263	0.04761904762	0.04761904762	0.1111111111		0.325	#DIV/0!
ZNF264	0.1428571429	0.1428571429	0.05555555556		2.333333333	#DIV/0!
ZNF266	0.1428571429	0.1428571429	0.05555555556		2.333333333	#DIV/0!
ZNF267	0.04761904762	0.04761904762	0		inf	#DIV/0!
ZNF268	0.09523809524	0.09523809524	0.05555555556		1.473684211	#DIV/0!
ZNF271P	0.09523809524	0.09523809524	0		inf	#DIV/0!
ZNF273	0.04761904762	0.04761904762	0.1111111111		0.325	#DIV/0!
ZNF274	0.1428571429	0.1428571429	0.05555555556		2.333333333	#DIV/0!
ZNF276	0.09523809524	0.09523809524	0		inf	#DIV/0!
ZNF277	0.04761904762	0.04761904762	0.05555555556		0.7	#DIV/0!
ZNF28	0.09523809524	0.09523809524	0.05555555556		1.473684211	#DIV/0!
ZNF280A	0.04761904762	0.04761904762	0.05555555556		0.7	#DIV/0!
ZNF280B	0.04761904762	0.04761904762	0.05555555556		0.7	#DIV/0!
ZNF280D	0.04761904762	0.04761904762	0.05555555556		0.7	#DIV/0!
ZNF281	0.1428571429	0.1428571429	0		inf	#DIV/0!
ZNF282	0.09523809524	0.09523809524	0.05555555556		1.473684211	#DIV/0!
ZNF283	0.09523809524	0.09523809524	0.05555555556		1.473684211	#DIV/0!
ZNF284	0.09523809524	0.09523809524	0.05555555556		1.473684211	#DIV/0!
ZNF285	0.09523809524	0.09523809524	0.05555555556		1.473684211	#DIV/0!
ZNF286A	0.04761904762	0.04761904762	0.05555555556		0.7	#DIV/0!
ZNF286B	0.04761904762	0.04761904762	0.1111111111		0.325	#DIV/0!
ZNF287	0.04761904762	0.04761904762	0.05555555556		0.7	#DIV/0!
NF295-A	0.04761904762	0.04761904762	0.1111111111		0.325	#DIV/0!
ZNF296	0.09523809524	0.09523809524	0.1111111111		0.6842105263	#DIV/0!
ZNF3	0.04761904762	0.04761904762	0.05555555556		0.7	#DIV/0!
ZNF30	0.04761904762	0.04761904762	0.05555555556		0.7	#DIV/0!
NF30-AS	0.04761904762	0.04761904762	0.05555555556		0.7	#DIV/0!
ZNF302	0.04761904762	0.04761904762	0.05555555556		0.7	#DIV/0!
ZNF304	0.1428571429	0.1428571429	0.05555555556		2.333333333	#DIV/0!
ZNF316	0.04761904762	0.04761904762	0.1666666667		0.2	#DIV/0!
ZNF317	0.1428571429	0.1428571429	0.05555555556		2.333333333	#DIV/0!
ZNF318	0.04761904762	0.04761904762	0.05555555556		0.7	#DIV/0!
ZNF319	0.04761904762	0.04761904762	0		inf	#DIV/0!
ZNF32	0.1428571429	0.1428571429	0.05555555556		2.333333333	#DIV/0!
NF32-AS	0.1428571429	0.1428571429	0.05555555556		2.333333333	#DIV/0!
NF32-AS	0.1428571429	0.1428571429	0.05555555556		2.333333333	#DIV/0!
NF32-AS	0.1428571429	0.1428571429	0.05555555556		2.333333333	#DIV/0!
ZNF320	0.09523809524	0.09523809524	0.05555555556		1.473684211	#DIV/0!
ZNF321P	0.09523809524	0.09523809524	0.05555555556		1.473684211	#DIV/0!
ZNF324	0.1428571429	0.1428571429	0.05555555556		2.333333333	#DIV/0!
ZNF324B	0.1428571429	0.1428571429	0.05555555556		2.333333333	#DIV/0!
ZNF326	0.04761904762	0.04761904762	0.05555555556		0.7	#DIV/0!
ZNF329	0.1428571429	0.1428571429	0.05555555556		2.333333333	#DIV/0!
ZNF330	0	0	0.05555555556		0	#DIV/0!
ZNF331	0.09523809524	0.09523809524	0.05555555556		1.473684211	#DIV/0!

ZNF333	0.1428571429	0.1428571429	0.1111111111		1.083333333	#DIV/0!
ZNF334	0.1428571429	0.1428571429	0.1666666667		0.6666666667	#DIV/0!
ZNF335	0.1428571429	0.1428571429	0.1666666667		0.6666666667	#DIV/0!
ZNF337	0.1904761905	0.1904761905	0.05555555556		3.294117647	#DIV/0!
NF337-A	0.1904761905	0.1904761905	0.05555555556		3.294117647	#DIV/0!
ZNF33A	0.09523809524	0.09523809524	0.1111111111		0.6842105263	#DIV/0!
ZNF33B	0.1428571429	0.1428571429	0.05555555556		2.333333333	#DIV/0!
NF33BP	0.09523809524	0.09523809524	0.1111111111		0.6842105263	#DIV/0!
ZNF34	0.09523809524	0.09523809524	0.2222222222		0.2894736842	#DIV/0!
ZNF341	0.09523809524	0.09523809524	0.1111111111		0.6842105263	#DIV/0!
NF341-A	0.09523809524	0.09523809524	0.1111111111		0.6842105263	#DIV/0!
ZNF343	0.1904761905	0.1904761905	0.05555555556		3.294117647	#DIV/0!
ZNF345	0.04761904762	0.04761904762	0.05555555556		0.7	#DIV/0!
ZNF346	0.09523809524	0.09523809524	0.05555555556		1.473684211	#DIV/0!
ZNF347	0.09523809524	0.09523809524	0.05555555556		1.473684211	#DIV/0!
ZNF350	0.09523809524	0.09523809524	0.05555555556		1.473684211	#DIV/0!
NF350-A	0.09523809524	0.09523809524	0.05555555556		1.473684211	#DIV/0!
ZNF354A	0.09523809524	0.09523809524	0.05555555556		1.473684211	#DIV/0!
ZNF354B	0.09523809524	0.09523809524	0.05555555556		1.473684211	#DIV/0!
ZNF354C	0.09523809524	0.09523809524	0.05555555556		1.473684211	#DIV/0!
ZNF358	0.1428571429	0.1428571429	0.05555555556		2.333333333	#DIV/0!
ZNF362	0.04761904762	0.04761904762	0.05555555556		0.7	#DIV/0!
ZNF365	0.04761904762	0.04761904762	0.1666666667		0.2	#DIV/0!
ZNF366	0	0	0.05555555556		0	#DIV/0!
ZNF367	0.04761904762	0.04761904762	0.05555555556		0.7	#DIV/0!
ZNF37A	0.09523809524	0.09523809524	0.1111111111		0.6842105263	#DIV/0!
ZNF37BP	0.1428571429	0.1428571429	0.05555555556		2.333333333	#DIV/0!
ZNF382	0.04761904762	0.04761904762	0.05555555556		0.7	#DIV/0!
ZNF383	0.04761904762	0.04761904762	0.05555555556		0.7	#DIV/0!
ZNF384	0.04761904762	0.04761904762	0.1111111111		0.325	#DIV/0!
ZNF385A	0.04761904762	0.04761904762	0.05555555556		0.7	#DIV/0!
ZNF385B	0	0	0.05555555556		0	#DIV/0!
ZNF385C	0.09523809524	0.09523809524	0		inf	#DIV/0!
ZNF385D	0.04761904762	0.04761904762	0		inf	#DIV/0!
F385D-A	0.04761904762	0.04761904762	0		inf	#DIV/0!
F385D-A	0.04761904762	0.04761904762	0		inf	#DIV/0!
ZNF394	0.04761904762	0.04761904762	0.05555555556		0.7	#DIV/0!
ZNF395	0.04761904762	0.04761904762	0.1111111111		0.325	#DIV/0!
ZNF396	0.09523809524	0.09523809524	0		inf	#DIV/0!
ZNF397	0.09523809524	0.09523809524	0		inf	#DIV/0!
ZNF398	0.09523809524	0.09523809524	0.05555555556		1.473684211	#DIV/0!
ZNF404	0.09523809524	0.09523809524	0.05555555556		1.473684211	#DIV/0!
ZNF407	0.09523809524	0.09523809524	0		inf	#DIV/0!
ZNF408	0	0	0.05555555556		0	#DIV/0!
ZNF410	0.09523809524	0.09523809524	0		inf	#DIV/0!
ZNF414	0.1428571429	0.1428571429	0.05555555556		2.333333333	#DIV/0!
ZNF415	0.09523809524	0.09523809524	0.05555555556		1.473684211	#DIV/0!
ZNF416	0.1428571429	0.1428571429	0.05555555556		2.333333333	#DIV/0!
ZNF417	0.1428571429	0.1428571429	0.05555555556		2.333333333	#DIV/0!
ZNF418	0.1428571429	0.1428571429	0.05555555556		2.333333333	#DIV/0!
ZNF419	0.1428571429	0.1428571429	0.05555555556		2.333333333	#DIV/0!

**ZNF420**	0.04761904762	0.04761904762	0.05555555556		0.7	#DIV/0!
**ZNF423**	0.04761904762	0.04761904762	0		inf	#DIV/0!
**ZNF425**	0.09523809524	0.09523809524	0.05555555556		1.473684211	#DIV/0!
**ZNF426**	0.1428571429	0.1428571429	0.1111111111		1.083333333	#DIV/0!
**NF426-D**	0.1428571429	0.1428571429	0.1111111111		1.083333333	#DIV/0!
**ZNF428**	0.09523809524	0.09523809524	0.05555555556		1.473684211	#DIV/0!
**ZNF429**	0.09523809524	0.09523809524	0		inf	#DIV/0!
**ZNF43**	0.09523809524	0.09523809524	0		inf	#DIV/0!
**ZNF430**	0.09523809524	0.09523809524	0		inf	#DIV/0!
**ZNF431**	0.09523809524	0.09523809524	0		inf	#DIV/0!
**ZNF432**	0.09523809524	0.09523809524	0.05555555556		1.473684211	#DIV/0!
**ZNF433**	0.1428571429	0.1428571429	0.1111111111		1.083333333	#DIV/0!
**NF433-A**	0.1428571429	0.1428571429	0.1111111111		1.083333333	#DIV/0!
**ZNF436**	0.04761904762	0.04761904762	0.1111111111		0.325	#DIV/0!
**NF436-A**	0.04761904762	0.04761904762	0.1111111111		0.325	#DIV/0!
**ZNF438**	0.09523809524	0.09523809524	0.2222222222		0.2894736842	#DIV/0!
**ZNF439**	0.1428571429	0.1428571429	0.1111111111		1.083333333	#DIV/0!
**ZNF44**	0.1428571429	0.1428571429	0.1111111111		1.083333333	#DIV/0!
**ZNF440**	0.1428571429	0.1428571429	0.1111111111		1.083333333	#DIV/0!
**ZNF441**	0.1428571429	0.1428571429	0.1111111111		1.083333333	#DIV/0!
**ZNF442**	0.1428571429	0.1428571429	0.1111111111		1.083333333	#DIV/0!
**ZNF443**	0.1428571429	0.1428571429	0.1111111111		1.083333333	#DIV/0!
**ZNF444**	0.1428571429	0.1428571429	0.05555555556		2.333333333	#DIV/0!
**ZNF446**	0.1428571429	0.1428571429	0.05555555556		2.333333333	#DIV/0!
**ZNF45**	0.09523809524	0.09523809524	0.05555555556		1.473684211	#DIV/0!
**ZNF451**	0.04761904762	0.04761904762	0.05555555556		0.7	#DIV/0!
**NF451-A**	0.04761904762	0.04761904762	0.05555555556		0.7	#DIV/0!
**ZNF454**	0.09523809524	0.09523809524	0.05555555556		1.473684211	#DIV/0!
**ZNF460**	0.1428571429	0.1428571429	0.05555555556		2.333333333	#DIV/0!
**NF460-A**	0.1428571429	0.1428571429	0.05555555556		2.333333333	#DIV/0!
**ZNF461**	0.04761904762	0.04761904762	0.05555555556		0.7	#DIV/0!
**ZNF462**	0.04761904762	0.04761904762	0.05555555556		0.7	#DIV/0!
**ZNF467**	0.09523809524	0.09523809524	0.05555555556		1.473684211	#DIV/0!
**ZNF468**	0.09523809524	0.09523809524	0.05555555556		1.473684211	#DIV/0!
**ZNF469**	0.1428571429	0.1428571429	0		inf	#DIV/0!
**ZNF470**	0.1428571429	0.1428571429	0.05555555556		2.333333333	#DIV/0!
**ZNF471**	0.1428571429	0.1428571429	0.05555555556		2.333333333	#DIV/0!
**ZNF473**	0.09523809524	0.09523809524	0.1111111111		0.6842105263	#DIV/0!
**ZNF474**	0.04761904762	0.04761904762	0		inf	#DIV/0!
**ZNF479**	0.1428571429	0.1428571429	0.1666666667		0.6666666667	#DIV/0!
**ZNF48**	0.04761904762	0.04761904762	0		inf	#DIV/0!
**ZNF480**	0.09523809524	0.09523809524	0.05555555556		1.473684211	#DIV/0!
**ZNF483**	0.04761904762	0.04761904762	0.05555555556		0.7	#DIV/0!
**ZNF484**	0.04761904762	0.04761904762	0.05555555556		0.7	#DIV/0!
**ZNF485**	0.1428571429	0.1428571429	0.05555555556		2.333333333	#DIV/0!
**ZNF486**	0.1428571429	0.1428571429	0		inf	#DIV/0!
**ZNF487**	0.1428571429	0.1428571429	0.05555555556		2.333333333	#DIV/0!
**ZNF488**	0.1428571429	0.1428571429	0.05555555556		2.333333333	#DIV/0!
**ZNF490**	0.1428571429	0.1428571429	0.1111111111		1.083333333	#DIV/0!
**ZNF491**	0.1428571429	0.1428571429	0.1111111111		1.083333333	#DIV/0!
**ZNF492**	0.09523809524	0.09523809524	0		inf	#DIV/0!

ZNF493	0.09523809524	0.09523809524	0		inf	#DIV/0!
ZNF496	0.1428571429	0.1428571429	0		inf	#DIV/0!
ZNF497	0.1428571429	0.1428571429	0.05555555556		2.333333333	#DIV/0!
ZNF500	0.04761904762	0.04761904762	0		inf	#DIV/0!
ZNF503	0.1904761905	0.1904761905	0.05555555556		3.294117647	#DIV/0!
NF503-A	0.1904761905	0.1904761905	0.05555555556		3.294117647	#DIV/0!
NF503-A	0.1428571429	0.1428571429	0.05555555556		2.333333333	#DIV/0!
ZNF506	0.1428571429	0.1428571429	0.1111111111		1.083333333	#DIV/0!
ZNF507	0.04761904762	0.04761904762	0.05555555556		0.7	#DIV/0!
ZNF510	0.04761904762	0.04761904762	0		inf	#DIV/0!
ZNF511	0.1904761905	0.1904761905	0		inf	#DIV/0!
ZNF512E	0.1428571429	0.1428571429	0.2222222222		0.4583333333	#DIV/0!
ZNF514	0	0	0.1111111111		0	#DIV/0!
ZNF516	0.09523809524	0.09523809524	0.05555555556		1.473684211	#DIV/0!
ZNF517	0.09523809524	0.09523809524	0.2222222222		0.2894736842	#DIV/0!
ZNF518A	0.1428571429	0.1428571429	0		inf	#DIV/0!
ZNF518E	0	0	0			#DIV/0!
ZNF519	0.09523809524	0.09523809524	0.05555555556		1.473684211	#DIV/0!
ZNF521	0.09523809524	0.09523809524	0.1111111111		0.6842105263	#DIV/0!
ZNF524	0.09523809524	0.09523809524	0.05555555556		1.473684211	#DIV/0!
ZNF525	0.09523809524	0.09523809524	0.05555555556		1.473684211	#DIV/0!
ZNF526	0.04761904762	0.04761904762	0.05555555556		0.7	#DIV/0!
ZNF527	0.04761904762	0.04761904762	0.05555555556		0.7	#DIV/0!
ZNF528	0.09523809524	0.09523809524	0.05555555556		1.473684211	#DIV/0!
NF528-A	0.09523809524	0.09523809524	0.05555555556		1.473684211	#DIV/0!
ZNF529	0.04761904762	0.04761904762	0.05555555556		0.7	#DIV/0!
NF529-A	0.04761904762	0.04761904762	0.05555555556		0.7	#DIV/0!
ZNF530	0.1428571429	0.1428571429	0.05555555556		2.333333333	#DIV/0!
ZNF532	0.09523809524	0.09523809524	0		inf	#DIV/0!
ZNF534	0.09523809524	0.09523809524	0.05555555556		1.473684211	#DIV/0!
ZNF536	0.04761904762	0.04761904762	0.05555555556		0.7	#DIV/0!
ZNF540	0.04761904762	0.04761904762	0.05555555556		0.7	#DIV/0!
ZNF541	0.09523809524	0.09523809524	0.1111111111		0.6842105263	#DIV/0!
ZNF542I	0.1428571429	0.1428571429	0.05555555556		2.333333333	#DIV/0!
ZNF543	0.1428571429	0.1428571429	0.05555555556		2.333333333	#DIV/0!
ZNF544	0.1428571429	0.1428571429	0.05555555556		2.333333333	#DIV/0!
ZNF546	0.04761904762	0.04761904762	0.05555555556		0.7	#DIV/0!
ZNF547	0.1428571429	0.1428571429	0.05555555556		2.333333333	#DIV/0!
ZNF548	0.1428571429	0.1428571429	0.05555555556		2.333333333	#DIV/0!
ZNF549	0.1428571429	0.1428571429	0.05555555556		2.333333333	#DIV/0!
ZNF550	0.1428571429	0.1428571429	0.05555555556		2.333333333	#DIV/0!
ZNF551	0.1428571429	0.1428571429	0.05555555556		2.333333333	#DIV/0!
ZNF552	0.1428571429	0.1428571429	0.05555555556		2.333333333	#DIV/0!
ZNF554	0.09523809524	0.09523809524	0.05555555556		1.473684211	#DIV/0!
ZNF555	0.09523809524	0.09523809524	0.05555555556		1.473684211	#DIV/0!
ZNF556	0.09523809524	0.09523809524	0.05555555556		1.473684211	#DIV/0!
ZNF557	0.1428571429	0.1428571429	0.05555555556		2.333333333	#DIV/0!
ZNF558	0.1428571429	0.1428571429	0.05555555556		2.333333333	#DIV/0!
ZNF559	0.1428571429	0.1428571429	0.05555555556		2.333333333	#DIV/0!
'559-ZNI	0.1428571429	0.1428571429	0.05555555556		2.333333333	#DIV/0!
ZNF56	0.1428571429	0.1428571429	0.1111111111		1.083333333	#DIV/0!

ZNF560	0.1428571429	0.1428571429	0.05555555556		2.333333333	#DIV/0!
ZNF561	0.1428571429	0.1428571429	0.1111111111		1.083333333	#DIV/0!
NF561-A	0.1428571429	0.1428571429	0.1111111111		1.083333333	#DIV/0!
ZNF562	0.1428571429	0.1428571429	0.1111111111		1.083333333	#DIV/0!
ZNF563	0.1428571429	0.1428571429	0.1111111111		1.083333333	#DIV/0!
ZNF564	0.1428571429	0.1428571429	0.1111111111		1.083333333	#DIV/0!
ZNF565	0.04761904762	0.04761904762	0.05555555556		0.7	#DIV/0!
ZNF566	0.04761904762	0.04761904762	0.05555555556		0.7	#DIV/0!
ZNF567	0.04761904762	0.04761904762	0.05555555556		0.7	#DIV/0!
ZNF568	0.04761904762	0.04761904762	0.05555555556		0.7	#DIV/0!
ZNF569	0.04761904762	0.04761904762	0.05555555556		0.7	#DIV/0!
ZNF57	0.09523809524	0.09523809524	0.05555555556		1.473684211	#DIV/0!
ZNF570	0.04761904762	0.04761904762	0.05555555556		0.7	#DIV/0!
ZNF571	0.04761904762	0.04761904762	0.05555555556		0.7	#DIV/0!
NF571-A	0.04761904762	0.04761904762	0.05555555556		0.7	#DIV/0!
ZNF572	0.1428571429	0.1428571429	0.1111111111		1.083333333	#DIV/0!
ZNF573	0.04761904762	0.04761904762	0.05555555556		0.7	#DIV/0!
ZNF574	0.04761904762	0.04761904762	0.05555555556		0.7	#DIV/0!
ZNF575	0.09523809524	0.09523809524	0.05555555556		1.473684211	#DIV/0!
ZNF576	0.09523809524	0.09523809524	0.05555555556		1.473684211	#DIV/0!
ZNF577	0.09523809524	0.09523809524	0.05555555556		1.473684211	#DIV/0!
ZNF578	0.09523809524	0.09523809524	0.05555555556		1.473684211	#DIV/0!
ZNF579	0.09523809524	0.09523809524	0.05555555556		1.473684211	#DIV/0!
ZNF580	0.09523809524	0.09523809524	0.05555555556		1.473684211	#DIV/0!
ZNF581	0.09523809524	0.09523809524	0.05555555556		1.473684211	#DIV/0!
ZNF582	0.1428571429	0.1428571429	0.05555555556		2.333333333	#DIV/0!
NF582-A	0.1428571429	0.1428571429	0.05555555556		2.333333333	#DIV/0!
ZNF583	0.1428571429	0.1428571429	0.05555555556		2.333333333	#DIV/0!
ZNF584	0.1428571429	0.1428571429	0.05555555556		2.333333333	#DIV/0!
ZNF585A	0.04761904762	0.04761904762	0.05555555556		0.7	#DIV/0!
ZNF585B	0.04761904762	0.04761904762	0.05555555556		0.7	#DIV/0!
ZNF586	0.1428571429	0.1428571429	0.05555555556		2.333333333	#DIV/0!
ZNF587	0.1428571429	0.1428571429	0.05555555556		2.333333333	#DIV/0!
ZNF587B	0.1428571429	0.1428571429	0.05555555556		2.333333333	#DIV/0!
ZNF589	0.09523809524	0.09523809524	0		inf	#DIV/0!
ZNF592	0.04761904762	0.04761904762	0.05555555556		0.7	#DIV/0!
ZNF593	0.04761904762	0.04761904762	0.1111111111		0.325	#DIV/0!
ZNF594	0	0	0.1111111111		0	#DIV/0!
ZNF595	0.04761904762	0.04761904762	0		inf	#DIV/0!
ZNF596	0.09523809524	0.09523809524	0.1111111111		0.6842105263	#DIV/0!
ZNF597	0.04761904762	0.04761904762	0.1111111111		0.325	#DIV/0!
ZNF598	0.09523809524	0.09523809524	0.2222222222		0.2894736842	#DIV/0!
ZNF599	0.04761904762	0.04761904762	0.05555555556		0.7	#DIV/0!
ZNF600	0.09523809524	0.09523809524	0.05555555556		1.473684211	#DIV/0!
ZNF605	0.09523809524	0.09523809524	0.05555555556		1.473684211	#DIV/0!
ZNF606	0.1428571429	0.1428571429	0.05555555556		2.333333333	#DIV/0!
ZNF607	0.04761904762	0.04761904762	0.05555555556		0.7	#DIV/0!
ZNF608	0.04761904762	0.04761904762	0		inf	#DIV/0!
ZNF609	0.04761904762	0.04761904762	0.05555555556		0.7	#DIV/0!
ZNF610	0.09523809524	0.09523809524	0.05555555556		1.473684211	#DIV/0!
ZNF611	0.09523809524	0.09523809524	0.05555555556		1.473684211	#DIV/0!

ZNF613	0.09523809524	0.09523809524	0.05555555556		1.473684211	#DIV/0!
ZNF614	0.09523809524	0.09523809524	0.05555555556		1.473684211	#DIV/0!
ZNF615	0.09523809524	0.09523809524	0.05555555556		1.473684211	#DIV/0!
ZNF616	0.09523809524	0.09523809524	0.05555555556		1.473684211	#DIV/0!
ZNF618	0.04761904762	0.04761904762	0.05555555556		0.7	#DIV/0!
ZNF619	0.04761904762	0.04761904762	0		inf	#DIV/0!
ZNF620	0.04761904762	0.04761904762	0		inf	#DIV/0!
ZNF621	0.04761904762	0.04761904762	0		inf	#DIV/0!
ZNF622	0.04761904762	0.04761904762	0.05555555556		0.7	#DIV/0!
ZNF623	0.04761904762	0.04761904762	0.1666666667		0.2	#DIV/0!
ZNF624	0.04761904762	0.04761904762	0.05555555556		0.7	#DIV/0!
ZNF625	0.1428571429	0.1428571429	0.1111111111		1.083333333	#DIV/0!
F625-ZN	0.1428571429	0.1428571429	0.1111111111		1.083333333	#DIV/0!
ZNF626	0.09523809524	0.09523809524	0		inf	#DIV/0!
ZNF627	0.1428571429	0.1428571429	0.1111111111		1.083333333	#DIV/0!
ZNF628	0.09523809524	0.09523809524	0.05555555556		1.473684211	#DIV/0!
ZNF629	0.04761904762	0.04761904762	0		inf	#DIV/0!
ZNF638	0	0	0.05555555556		0	#DIV/0!
ZNF639	0.1904761905	0.1904761905	0.05555555556		3.294117647	#DIV/0!
ZNF641	0.04761904762	0.04761904762	0.05555555556		0.7	#DIV/0!
ZNF644	0.04761904762	0.04761904762	0.05555555556		0.7	#DIV/0!
ZNF646	0.04761904762	0.04761904762	0		inf	#DIV/0!
ZNF648	0.1428571429	0.1428571429	0		inf	#DIV/0!
ZNF649	0.09523809524	0.09523809524	0.05555555556		1.473684211	#DIV/0!
NF649-A	0.09523809524	0.09523809524	0.05555555556		1.473684211	#DIV/0!
ZNF652	0.1428571429	0.1428571429	0		inf	#DIV/0!
ZNF653	0.1428571429	0.1428571429	0.1111111111		1.083333333	#DIV/0!
ZNF654	0.04761904762	0.04761904762	0		inf	#DIV/0!
ZNF655	0.04761904762	0.04761904762	0.05555555556		0.7	#DIV/0!
ZNF658	0.04761904762	0.04761904762	0.1111111111		0.325	#DIV/0!
ZNF658B	0.04761904762	0.04761904762	0.1111111111		0.325	#DIV/0!
ZNF66	0.09523809524	0.09523809524	0		inf	#DIV/0!
ZNF662	0.04761904762	0.04761904762	0		inf	#DIV/0!
ZNF663P	0.1428571429	0.1428571429	0.1666666667		0.6666666667	#DIV/0!
ZNF664	0.04761904762	0.04761904762	0.05555555556		0.7	#DIV/0!
664-RFI	0.04761904762	0.04761904762	0.05555555556		0.7	#DIV/0!
ZNF665	0.09523809524	0.09523809524	0.05555555556		1.473684211	#DIV/0!
ZNF667	0.1428571429	0.1428571429	0.05555555556		2.333333333	#DIV/0!
NF667-A	0.1428571429	0.1428571429	0.05555555556		2.333333333	#DIV/0!
ZNF668	0.04761904762	0.04761904762	0		inf	#DIV/0!
ZNF669	0.1428571429	0.1428571429	0		inf	#DIV/0!
ZNF670	0.1428571429	0.1428571429	0		inf	#DIV/0!
670-ZNI	0.1428571429	0.1428571429	0.05555555556		2.333333333	#DIV/0!
ZNF671	0.1428571429	0.1428571429	0.05555555556		2.333333333	#DIV/0!
ZNF672	0.1428571429	0.1428571429	0		inf	#DIV/0!
ZNF675	0.09523809524	0.09523809524	0		inf	#DIV/0!
ZNF676	0.09523809524	0.09523809524	0		inf	#DIV/0!
ZNF677	0.09523809524	0.09523809524	0.05555555556		1.473684211	#DIV/0!
ZNF678	0.1428571429	0.1428571429	0		inf	#DIV/0!
ZNF679	0.09523809524	0.09523809524	0.05555555556		1.473684211	#DIV/0!
ZNF680	0.04761904762	0.04761904762	0.05555555556		0.7	#DIV/0!

ZNF681	0.09523809524	0.09523809524	0		inf	#DIV/0!
ZNF682	0.1428571429	0.1428571429	0		inf	#DIV/0!
ZNF683	0.04761904762	0.04761904762	0.1111111111		0.325	#DIV/0!
ZNF684	0.04761904762	0.04761904762	0.05555555556		0.7	#DIV/0!
ZNF687	0.1904761905	0.1904761905	0.05555555556		3.294117647	#DIV/0!
NF687-A	0.1904761905	0.1904761905	0.05555555556		3.294117647	#DIV/0!
ZNF688	0.04761904762	0.04761904762	0		inf	#DIV/0!
ZNF689	0.04761904762	0.04761904762	0		inf	#DIV/0!
ZNF69	0.1428571429	0.1428571429	0.1111111111		1.083333333	#DIV/0!
ZNF691	0.04761904762	0.04761904762	0.05555555556		0.7	#DIV/0!
ZNF692	0.1428571429	0.1428571429	0		inf	#DIV/0!
ZNF695	0.1428571429	0.1428571429	0.05555555556		2.333333333	#DIV/0!
ZNF697	0.04761904762	0.04761904762	0.05555555556		0.7	#DIV/0!
ZNF699	0.1428571429	0.1428571429	0.05555555556		2.333333333	#DIV/0!
ZNF7	0.09523809524	0.09523809524	0.2222222222		0.2894736842	#DIV/0!
ZNF700	0.1428571429	0.1428571429	0.1111111111		1.083333333	#DIV/0!
ZNF701	0.09523809524	0.09523809524	0.05555555556		1.473684211	#DIV/0!
ZNF702I	0.09523809524	0.09523809524	0.05555555556		1.473684211	#DIV/0!
ZNF703	0.04761904762	0.04761904762	0.1111111111		0.325	#DIV/0!
ZNF704	0.1428571429	0.1428571429	0.1111111111		1.083333333	#DIV/0!
ZNF705A	0.04761904762	0.04761904762	0.1111111111		0.325	#DIV/0!
ZNF705E	0.09523809524	0.09523809524	0.2222222222		0.2894736842	#DIV/0!
ZNF705I	0.09523809524	0.09523809524	0.1111111111		0.6842105263	#DIV/0!
ZNF705C	0.09523809524	0.09523809524	0.2222222222		0.2894736842	#DIV/0!
ZNF706	0.1904761905	0.1904761905	0.1111111111		1.529411765	#DIV/0!
ZNF707	0.04761904762	0.04761904762	0.1666666667		0.2	#DIV/0!
ZNF708	0.09523809524	0.09523809524	0		inf	#DIV/0!
ZNF709	0.1428571429	0.1428571429	0.1111111111		1.083333333	#DIV/0!
ZNF71	0.1428571429	0.1428571429	0.05555555556		2.333333333	#DIV/0!
ZNF710	0.04761904762	0.04761904762	0.05555555556		0.7	#DIV/0!
NF710-A	0.04761904762	0.04761904762	0.05555555556		0.7	#DIV/0!
ZNF713	0.3333333333	0.3333333333	0.2222222222		1.375	#DIV/0!
ZNF714	0.09523809524	0.09523809524	0		inf	#DIV/0!
ZNF716	0.1428571429	0.1428571429	0.1666666667		0.6666666667	#DIV/0!
ZNF717	0.04761904762	0.04761904762	0		inf	#DIV/0!
ZNF718	0.04761904762	0.04761904762	0		inf	#DIV/0!
ZNF720	0.04761904762	0.04761904762	0		inf	#DIV/0!
ZNF721	0.04761904762	0.04761904762	0		inf	#DIV/0!
ZNF722I	0.09523809524	0.09523809524	0.05555555556		1.473684211	#DIV/0!
ZNF723	0.09523809524	0.09523809524	0		inf	#DIV/0!
ZNF724	0.09523809524	0.09523809524	0		inf	#DIV/0!
ZNF726	0.09523809524	0.09523809524	0		inf	#DIV/0!
ZNF727	0.09523809524	0.09523809524	0.05555555556		1.473684211	#DIV/0!
ZNF728	0.09523809524	0.09523809524	0		inf	#DIV/0!
ZNF729	0.09523809524	0.09523809524	0		inf	#DIV/0!
ZNF730	0.09523809524	0.09523809524	0		inf	#DIV/0!
ZNF732	0.04761904762	0.04761904762	0		inf	#DIV/0!
ZNF733I	0.09523809524	0.09523809524	0.05555555556		1.473684211	#DIV/0!
ZNF735	0.09523809524	0.09523809524	0.05555555556		1.473684211	#DIV/0!
ZNF736	0.09523809524	0.09523809524	0.05555555556		1.473684211	#DIV/0!
ZNF737	0.09523809524	0.09523809524	0		inf	#DIV/0!

ZNF738	0.09523809524	0.09523809524	0		inf	#DIV/0!
ZNF74	0.04761904762	0.04761904762	0.05555555556		0.7	#DIV/0!
ZNF740	0.04761904762	0.04761904762	0.05555555556		0.7	#DIV/0!
ZNF746	0.09523809524	0.09523809524	0.05555555556		1.473684211	#DIV/0!
ZNF747	0.04761904762	0.04761904762	0		inf	#DIV/0!
ZNF749	0.1428571429	0.1428571429	0.05555555556		2.333333333	#DIV/0!
ZNF75A	0.04761904762	0.04761904762	0.1111111111		0.325	#DIV/0!
ZNF76	0.04761904762	0.04761904762	0		inf	#DIV/0!
ZNF761	0.09523809524	0.09523809524	0.05555555556		1.473684211	#DIV/0!
ZNF763	0.1428571429	0.1428571429	0.1111111111		1.083333333	#DIV/0!
ZNF764	0.04761904762	0.04761904762	0		inf	#DIV/0!
ZNF765	0.09523809524	0.09523809524	0.05555555556		1.473684211	#DIV/0!
765-ZNI	0.09523809524	0.09523809524	0.05555555556		1.473684211	#DIV/0!
ZNF766	0.09523809524	0.09523809524	0.05555555556		1.473684211	#DIV/0!
ZNF767I	0.09523809524	0.09523809524	0.05555555556		1.473684211	#DIV/0!
ZNF768	0.04761904762	0.04761904762	0		inf	#DIV/0!
ZNF77	0.09523809524	0.09523809524	0.05555555556		1.473684211	#DIV/0!
ZNF771	0.04761904762	0.04761904762	0		inf	#DIV/0!
ZNF772	0.1428571429	0.1428571429	0.05555555556		2.333333333	#DIV/0!
ZNF773	0.1428571429	0.1428571429	0.05555555556		2.333333333	#DIV/0!
ZNF774	0.04761904762	0.04761904762	0.05555555556		0.7	#DIV/0!
ZNF775	0.09523809524	0.09523809524	0.05555555556		1.473684211	#DIV/0!
ZNF776	0.1428571429	0.1428571429	0.05555555556		2.333333333	#DIV/0!
ZNF777	0.09523809524	0.09523809524	0.05555555556		1.473684211	#DIV/0!
ZNF778	0.09523809524	0.09523809524	0		inf	#DIV/0!
ZNF780A	0.04761904762	0.04761904762	0.05555555556		0.7	#DIV/0!
ZNF780I	0.04761904762	0.04761904762	0.05555555556		0.7	#DIV/0!
ZNF781	0.04761904762	0.04761904762	0.05555555556		0.7	#DIV/0!
ZNF782	0.04761904762	0.04761904762	0		inf	#DIV/0!
ZNF783	0.09523809524	0.09523809524	0.05555555556		1.473684211	#DIV/0!
ZNF784	0.09523809524	0.09523809524	0.05555555556		1.473684211	#DIV/0!
ZNF785	0.04761904762	0.04761904762	0		inf	#DIV/0!
ZNF786	0.09523809524	0.09523809524	0.05555555556		1.473684211	#DIV/0!
ZNF787	0.1428571429	0.1428571429	0.05555555556		2.333333333	#DIV/0!
ZNF788I	0.1428571429	0.1428571429	0.1111111111		1.083333333	#DIV/0!
ZNF789	0.04761904762	0.04761904762	0.05555555556		0.7	#DIV/0!
ZNF79	0.09523809524	0.09523809524	0.05555555556		1.473684211	#DIV/0!
ZNF790	0.04761904762	0.04761904762	0.05555555556		0.7	#DIV/0!
NF790-A	0.04761904762	0.04761904762	0.05555555556		0.7	#DIV/0!
ZNF791	0.1428571429	0.1428571429	0.1111111111		1.083333333	#DIV/0!
ZNF792	0.04761904762	0.04761904762	0.05555555556		0.7	#DIV/0!
ZNF793	0.04761904762	0.04761904762	0.05555555556		0.7	#DIV/0!
NF793-A	0.04761904762	0.04761904762	0.05555555556		0.7	#DIV/0!
ZNF799	0.1428571429	0.1428571429	0.1111111111		1.083333333	#DIV/0!
ZNF8	0.1428571429	0.1428571429	0.05555555556		2.333333333	#DIV/0!
F8-ERVI	0.1428571429	0.1428571429	0.05555555556		2.333333333	#DIV/0!
ZNF80	0.09523809524	0.09523809524	0.05555555556		1.473684211	#DIV/0!
ZNF800	0.04761904762	0.04761904762	0.05555555556		0.7	#DIV/0!
ZNF804A	0	0	0.05555555556		0	#DIV/0!
ZNF804I	0.09523809524	0.09523809524	0.1111111111		0.6842105263	#DIV/0!
ZNF805	0.1428571429	0.1428571429	0.05555555556		2.333333333	#DIV/0!

ZNF806	0	0	0.05555555556		0	#DIV/0!
ZNF807	0.04761904762	0.04761904762	0.05555555556		0.7	#DIV/0!
ZNF808	0.09523809524	0.09523809524	0.05555555556		1.473684211	#DIV/0!
ZNF812I	0.1428571429	0.1428571429	0.1111111111		1.083333333	#DIV/0!
ZNF813	0.09523809524	0.09523809524	0.05555555556		1.473684211	#DIV/0!
ZNF814	0.1428571429	0.1428571429	0.05555555556		2.333333333	#DIV/0!
ZNF815I	0.04761904762	0.04761904762	0.1666666667		0.2	#DIV/0!
ZNF816	0.09523809524	0.09523809524	0.05555555556		1.473684211	#DIV/0!
816-ZNF	0.09523809524	0.09523809524	0.05555555556		1.473684211	#DIV/0!
ZNF818I	0.09523809524	0.09523809524	0.05555555556		1.473684211	#DIV/0!
ZNF821	0.04761904762	0.04761904762	0		inf	#DIV/0!
ZNF823	0.1428571429	0.1428571429	0.1111111111		1.083333333	#DIV/0!
ZNF826I	0.1428571429	0.1428571429	0		inf	#DIV/0!
ZNF827	0	0	0.05555555556		0	#DIV/0!
ZNF829	0.04761904762	0.04761904762	0.05555555556		0.7	#DIV/0!
ZNF83	0.09523809524	0.09523809524	0.05555555556		1.473684211	#DIV/0!
ZNF831	0.1428571429	0.1428571429	0.1666666667		0.6666666667	#DIV/0!
ZNF833I	0.1428571429	0.1428571429	0.1111111111		1.083333333	#DIV/0!
ZNF835	0.1428571429	0.1428571429	0.05555555556		2.333333333	#DIV/0!
ZNF836	0.09523809524	0.09523809524	0.05555555556		1.473684211	#DIV/0!
ZNF837	0.1428571429	0.1428571429	0.05555555556		2.333333333	#DIV/0!
ZNF84	0.09523809524	0.09523809524	0.05555555556		1.473684211	#DIV/0!
ZNF841	0.09523809524	0.09523809524	0.05555555556		1.473684211	#DIV/0!
ZNF843	0.04761904762	0.04761904762	0		inf	#DIV/0!
ZNF844	0.1428571429	0.1428571429	0.1111111111		1.083333333	#DIV/0!
ZNF845	0.09523809524	0.09523809524	0.05555555556		1.473684211	#DIV/0!
ZNF846	0.1428571429	0.1428571429	0.1111111111		1.083333333	#DIV/0!
ZNF847I	0.1428571429	0.1428571429	0		inf	#DIV/0!
ZNF85	0.09523809524	0.09523809524	0		inf	#DIV/0!
ZNF850	0.04761904762	0.04761904762	0.05555555556		0.7	#DIV/0!
ZNF853	0.04761904762	0.04761904762	0.1666666667		0.2	#DIV/0!
ZNF860	0.09523809524	0.09523809524	0		inf	#DIV/0!
ZNF862	0.09523809524	0.09523809524	0.05555555556		1.473684211	#DIV/0!
ZNF865	0.09523809524	0.09523809524	0.05555555556		1.473684211	#DIV/0!
ZNF875	0.04761904762	0.04761904762	0.05555555556		0.7	#DIV/0!
ZNF876I	0.04761904762	0.04761904762	0		inf	#DIV/0!
ZNF878	0.1428571429	0.1428571429	0.1111111111		1.083333333	#DIV/0!
ZNF879	0.09523809524	0.09523809524	0.05555555556		1.473684211	#DIV/0!
ZNF880	0.09523809524	0.09523809524	0.05555555556		1.473684211	#DIV/0!
ZNF883	0.04761904762	0.04761904762	0.05555555556		0.7	#DIV/0!
ZNF888	0.09523809524	0.09523809524	0.05555555556		1.473684211	#DIV/0!
ZNF890I	0.04761904762	0.04761904762	0.1666666667		0.2	#DIV/0!
ZNF891	0.09523809524	0.09523809524	0.05555555556		1.473684211	#DIV/0!
ZNF90	0.1428571429	0.1428571429	0		inf	#DIV/0!
ZNF91	0.09523809524	0.09523809524	0		inf	#DIV/0!
ZNF92	0.04761904762	0.04761904762	0.1111111111		0.325	#DIV/0!
ZNF93	0.1428571429	0.1428571429	0.05555555556		2.333333333	#DIV/0!
ZNF98	0.09523809524	0.09523809524	0		inf	#DIV/0!
ZNF99	0.09523809524	0.09523809524	0		inf	#DIV/0!
ZNFX1	0.09523809524	0.09523809524	0.1666666667		0.4210526316	#DIV/0!
ZNHIT1	0.04761904762	0.04761904762	0.05555555556		0.7	#DIV/0!

ZNHIT3	0.04761904762	0.04761904762	0		inf	#DIV/0!
ZNHIT6	0.04761904762	0.04761904762	0.05555555556		0.7	#DIV/0!
ZNRF1	0.04761904762	0.04761904762	0		inf	#DIV/0!
ZNRF2	0.04761904762	0.04761904762	0.1666666667		0.2	#DIV/0!
ZNRF2P	0.04761904762	0.04761904762	0.1666666667		0.2	#DIV/0!
ZNRF2P	0.04761904762	0.04761904762	0.1666666667		0.2	#DIV/0!
ZNRF3	0.04761904762	0.04761904762	0.1111111111		0.325	#DIV/0!
NRF3-AS	0.04761904762	0.04761904762	0.1111111111		0.325	#DIV/0!
ZNRF4	0.1428571429	0.1428571429	0.05555555556		2.333333333	#DIV/0!
ZP2	0.04761904762	0.04761904762	0		inf	#DIV/0!
ZP3	0.09523809524	0.09523809524	0.1111111111		0.6842105263	#DIV/0!
ZP4	0.1428571429	0.1428571429	0		inf	#DIV/0!
ZPBP	0.09523809524	0.09523809524	0.2222222222		0.2894736842	#DIV/0!
ZPBP2	0.09523809524	0.09523809524	0		inf	#DIV/0!
ZPLD1	0.09523809524	0.09523809524	0.05555555556		1.473684211	#DIV/0!
ZRANB1	0.2380952381	0.2380952381	0.05555555556		4.375	#DIV/0!
ZRANB2	0.04761904762	0.04761904762	0.05555555556		0.7	#DIV/0!
ANB2-A	0.04761904762	0.04761904762	0.05555555556		0.7	#DIV/0!
ANB2-A	0.04761904762	0.04761904762	0.05555555556		0.7	#DIV/0!
ZRANB3	0.04761904762	0.04761904762	0.05555555556		0.7	#DIV/0!
ZSCAN1	0.1428571429	0.1428571429	0.05555555556		2.333333333	#DIV/0!
ZSCAN1	0.04761904762	0.04761904762	0.1111111111		0.325	#DIV/0!
ZSCAN1	0.1428571429	0.1428571429	0.05555555556		2.333333333	#DIV/0!
ZSCAN2	0.04761904762	0.04761904762	0.05555555556		0.7	#DIV/0!
ZSCAN2	0.04761904762	0.04761904762	0		inf	#DIV/0!
ZSCAN2	0.04761904762	0.04761904762	0.05555555556		0.7	#DIV/0!
ZSCAN2	0.1428571429	0.1428571429	0.05555555556		2.333333333	#DIV/0!
ZSCAN2	0.04761904762	0.04761904762	0.05555555556		0.7	#DIV/0!
ZSCAN3	0.09523809524	0.09523809524	0		inf	#DIV/0!
ZSCAN3	0.04761904762	0.04761904762	0.1111111111		0.325	#DIV/0!
ZSCAN4	0.1428571429	0.1428571429	0.05555555556		2.333333333	#DIV/0!
ZSCAN5	0.1428571429	0.1428571429	0.05555555556		2.333333333	#DIV/0!
ZSCAN5	0.1428571429	0.1428571429	0.05555555556		2.333333333	#DIV/0!
ZSCAN5	0.1428571429	0.1428571429	0.05555555556		2.333333333	#DIV/0!
ZSWIM1	0.1428571429	0.1428571429	0.1666666667		0.6666666667	#DIV/0!
ZSWIM2	0	0	0.05555555556		0	#DIV/0!
ZSWIM3	0.1428571429	0.1428571429	0.1666666667		0.6666666667	#DIV/0!
ZSWIM4	0.1428571429	0.1428571429	0.1111111111		1.083333333	#DIV/0!
ZSWIM5	0.04761904762	0.04761904762	0.05555555556		0.7	#DIV/0!
ZSWIM6	0.04761904762	0.04761904762	0		inf	#DIV/0!
ZSWIM7	0.04761904762	0.04761904762	0.05555555556		0.7	#DIV/0!
ZSWIM8	0.1904761905	0.1904761905	0.05555555556		3.294117647	#DIV/0!
WIM8-A	0.1904761905	0.1904761905	0.05555555556		3.294117647	#DIV/0!
ZSWIM9	0.09523809524	0.09523809524	0.1111111111		0.6842105263	#DIV/0!
ZWILCH	0.04761904762	0.04761904762	0.05555555556		0.7	#DIV/0!
ZWINT	0.09523809524	0.09523809524	0.05555555556		1.473684211	#DIV/0!
ZXDC	0.04761904762	0.04761904762	0.05555555556		0.7	#DIV/0!
ZYG11A	0.04761904762	0.04761904762	0.05555555556		0.7	#DIV/0!
ZYG11B	0.04761904762	0.04761904762	0.05555555556		0.7	#DIV/0!
ZYX	0.04761904762	0.04761904762	0.05555555556		0.7	#DIV/0!
ZZEF1	0	0	0.1111111111		0	#DIV/0!

**ZZZ3**	0.04761904762	0.04761904762	0.05555555556		0.7	#DIV/0!

Pvalue
0.6256684492
0.6256684492
1
0.5588235294
0.5588235294
0.5588235294
0.5588235294
1
0.0637254902
0.6256684492
1
1
1
1
1
1
1
0.5588235294
0.5588235294
1
0.4166666667
1
0.4166666667
0.4166666667
1
1
1
0.6256684492
1
1
0.376114082
1
0.5588235294
1
1
1
0.5
1
1
1
0.4166666667
0.209972112
0.209972112
0.6256684492
0.209972112
1
1
1
1

1
1
1
1
0.25
1
1
0.1379790553
1
0.5588235294
1
1
1
1
0.5588235294
0.25
0.5
0.376114082
0.6256684492
1
0.5
1
1
1
1
1
0.4166666667
1
0.6256684492
0.5588235294
0.4166666667
0.4166666667
0.4166666667
1
1
1
0.376114082
1
1
1
0.4166666667
1
0.4178238744
1
0.4166666667
1
0.4166666667
1
0.5588235294
0.4166666667
1

1
1
1
1
0.25
1
1
1
0.25
0.376114082
1
0.1666666667
1
0.4166666667
0.3666803232
1
1
1
0.4166666667
0.1666666667
0.6256684492
1
0.4166666667
1
0.1666666667
0.3666803232
0.4166666667
1
0.4166666667
0.6256684492
0.1666666667
0.6256684492
0.6256684492
0.25
1
0.25
1
1
0.6256684492
1
0.6256684492
1
1
0.4166666667
0.25
1
0.4166666667
0.6256684492
0.5
1
1

0.1666666667
0.4166666667
0.5
1
1
0.4166666667
0.5
0.5
0.5
0.4166666667
0.6773654476
1
1
0.4166666667
0.4166666667
0.4166666667
0.4166666667
1
1
0.6256684492
1
0.6256684492
0.1666666667
0.5588235294
1
0.6256684492
0.5
0.5
0.4166666667
0.4166666667
0.6256684492
1
1
1
1
1
0.5588235294
1
0.25
0.376114082
1
1
0.25
0.6773654476
0.6773654476
0.2869875223
1
1
1
0.5588235294
1

0.4166666667
1
0.376114082
1
1
1
1
0.6256684492
0.25
1
1
0.6256684492
0.1666666667
0.4166666667
0.4166666667
0.4166666667
1
1
1
1
0.4178238744
1
1
0.4166666667
0.376114082
0.4166666667
1
0.4166666667
0.4166666667
1
1
1
1
1
0.6773654476
1
0.4166666667
1
1
0.5
0.3666803232
1
0.4166666667
1
1
0.1666666667
1
1
0.4166666667
0.5588235294
0.4166666667

1
1
0.376114082
1
1
1
1
0.5588235294
0.1247771836
1
0.5588235294
0.5588235294
0.6256684492
0.5588235294
1
0.5
0.1666666667
1
0.5
1
1
1
1
1
0.25
0.5588235294
1
1
1
1
1
1
1
1
1
0.376114082
0.376114082
1
0.2869875223
1
1
0.5588235294
0.5588235294
0.5588235294
1
1
1
0.2869875223
0.1379790553
0.25
0.4166666667

0.4166666667
1
1
1
0.5588235294
0.5588235294
0.5
0.5588235294
1
0.4166666667
0.6256684492
0.6256684492
0.1247771836
0.1247771836
0.5588235294
0.2869875223
0.5588235294
1
1
0.6256684492
1
1
0.6256684492
1
1
1
1
1
1
1
1
1
1
1
1
1
1
1
0.4166666667
0.4166666667
0.4166666667
0.4166666667
0.4166666667
0.4166666667
0.4166666667
0.6773654476
0.4166666667
0.6773654476
1
1
0.25

0.2869875223
0.6256684492
0.376114082
0.1666666667
1
0.6295677362
0.6295677362
1
0.2869875223
0.25
0.5588235294
0.5588235294
1
1
1
1
1
1
1
0.5588235294
1
0.376114082
0.6256684492
0.1666666667
1
0.6256684492
1
0.4178238744
0.1666666667
0.6256684492
1
0.2869875223
0.5588235294
1
0.376114082
0.4166666667
0.4166666667
0.4166666667
1
1
0.5
1
1
1
1
0.4166666667
0.4166666667
0.4166666667
0.4166666667
0.6256684492
0.6256684492

0.6256684492
0.6256684492
0.6256684492
1
0.6256684492
0.376114082
1
1
0.6256684492
1
1
0.4166666667
1
0.4166666667
1
1
0.4166666667
1
1
0.5588235294
0.5588235294
0.1379790553
1
0.1379790553
1
1
1
0.6256684492
0.5588235294
1
0.1666666667
0.1379790553
0.1379790553
0.1666666667
0.6256684492
0.25
0.4166666667
1
0.5588235294
0.4166666667
1
0.6256684492
0.4166666667
1
1
1
0.5588235294
0.1379790553
0.4166666667
1
1

1
0.5588235294
0.6256684492
1
1
0.5
1
0.25
0.4166666667
0.2869875223
0.4166666667
0.5588235294
0.4166666667
0.6256684492
0.4166666667
1
1
1
1
1
1
1
1
0.5
0.5
1
1
1
0.5588235294
0.5
1
1
0.5588235294
1
1
1
1
1
1
1
0.0637254902
0.0637254902
0.0637254902
0.0637254902
0.2869875223
0.0637254902
1
0.2869875223
0.5588235294
1
0.5588235294

0.5588235294
1
1
0.6256684492
1
1
1
1
1
1
0.25
0.4166666667
1
1
1
1
1
1
1
0.1666666667
1
1
0.5588235294
0.4166666667
0.5
1
0.25
1
1
1
1
1
1
0.1666666667
1
1
1
1
0.6256684492
1
0.4166666667
1
0.4166666667
0.4166666667
1
1
0.6256684492
0.4166666667
0.4166666667
0.4166666667
0.4166666667

0.4166666667
0.4166666667
0.4166666667
0.4166666667
0.1666666667
0.4166666667
0.4166666667
0.6256684492
0.4166666667
0.4166666667
0.4166666667
0.4166666667
0.5
1
0.5588235294
0.4166666667
0.4166666667
1
1
1
0.5588235294
1
1
0.4166666667
1
1
0.5588235294
0.4166666667
1
1
1
1
1
0.5
0.4166666667
1
0.6256684492
1
1
0.1379790553
0.25
1
1
1
1
1
1
0.2869875223
1
1
0.4166666667

0.4166666667
1
0.6256684492
0.376114082
0.4166666667
0.6256684492
0.5588235294
0.1666666667
0.25
1
1
1
0.25
1
1
0.6256684492
1
0.5588235294
1
1
0.6295677362
0.4166666667
0.5588235294
0.4166666667
1
1
1
0.5
0.1666666667
1
1
1
0.5588235294
1
1
1
0.1379790553
0.4166666667
1
0.5
0.5
0.1666666667
0.4166666667
1
1
0.6256684492
1
1
1
0.6256684492
1

0.25
0.6940287188
1
1
0.376114082
0.6940287188
0.209972112
0.209972112
1
0.1666666667
1
0.6773654476
0.1666666667
1
1
1
1
1
1
1
1
1
0.4166666667
1
0.6256684492
1
1
1
1
1
1
0.6256684492
0.1666666667
0.1666666667
0.25
0.4166666667
0.1666666667
0.4166666667
0.1666666667
0.5
0.5
0.4166666667
0.4166666667
0.25
1
0.4166666667
1
0.4166666667
0.1666666667
1
1

0.4178238744
0.2869875223
1
1
0.1666666667
1
1
1
1
1
1
1
1
0.1379790553
1
0.5588235294
1
1
0.4166666667
1
1
1
1
1
1
0.4166666667
1
0.376114082
1
0.4166666667
1
0.6256684492
0.6256684492
0.4166666667
0.4166666667
0.25
1
1
0.4166666667
1
0.6256684492
1
1
0.4166666667
0.4166666667
0.376114082
0.6256684492
0.6256684492
0.376114082
0.1379790553
0.1379790553

1
1
1
1
1
0.4166666667
0.4166666667
0.4166666667
0.5588235294
0.5588235294
1
1
1
1
0.4166666667
1
1
0.5
0.6256684492
1
1
1
1
0.376114082
0.6295677362
1
1
1
1
0.6256684492
1
1
1
0.376114082
0.2869875223
1
0.4166666667
1
1
1
0.376114082
1
1
1
1
0.4178238744
0.4178238744
0.25
0.5
1
0.376114082

1
0.4166666667
1
0.376114082
0.25
0.4166666667
0.5588235294
0.4166666667
0.5588235294
0.5588235294
0.5588235294
0.5588235294
0.5588235294
0.5588235294
0.5588235294
0.5588235294
0.5588235294
1
1
1
1
1
1
0.6256684492
1
0.6256684492
1
0.1666666667
0.1666666667
0.1666666667
0.1666666667
0.1666666667
0.1666666667
1
1
1
0.5588235294
0.25
0.5
1
0.1247771836
0.5588235294
1
0.5588235294
0.376114082
0.4166666667
0.4166666667
1
1
1
1

1
1
1
0.209972112
0.6940287188
1
1
1
0.2869875223
1
0.25
1
0.5
0.5
1
1
0.4178238744
0.4166666667
0.0637254902
0.6773654476
0.6295677362
0.4166666667
1
0.4178238744
0.6256684492
1
0.4166666667
0.4166666667
0.209972112
0.4166666667
1
0.25
0.25
1
1
1
0.4166666667
0.4166666667
1
1
1
0.25
1
1
1
0.5
1
0.25
1
1
1

1
0.209972112
0.6773654476
0.5588235294
1
0.0637254902
1
0.5588235294
0.1379790553
1
1
0.5588235294
0.6256684492
1
0.4166666667
0.6256684492
0.5588235294
1
0.6256684492
0.376114082
0.376114082
0.4166666667
1
1
0.4166666667
1
1
0.4166666667
0.4166666667
1
0.4166666667
1
0.5588235294
1
1
1
1
1
0.6256684492
0.1666666667
0.2869875223
1
0.5
0.5
1
1
1
0.6256684492
1
1
1

0.061942959
0.061942959
1
0.6256684492
0.1379790553
0.4166666667
1
0.4166666667
1
0.5
1
1
0.5588235294
1
0.4166666667
0.25
1
0.5
0.6773654476
1
1
1
1
1
1
1
1
1
1
0.6256684492
0.4166666667
1
0.4166666667
0.6256684492
1
0.6256684492
0.376114082
1
1
1
1
1
0.4166666667
0.1666666667
0.5
0.5
1
1
1
1
1

0.1666666667
0.5
0.5
1
1
1
1
1
0.1666666667
1
1
0.4166666667
1
1
1
0.5588235294
1
1
0.25
1
0.3666803232
0.6256684492
1
1
1
0.5588235294
0.5588235294
0.5588235294
0.4166666667
0.5588235294
0.4166666667
1
0.2869875223
0.5
1
0.5
0.5
1
0.4166666667
1
0.5588235294
0.4166666667
1
1
1
1
0.376114082
0.5588235294
0.5588235294
1
1

0.25
0.4166666667
1
0.4166666667
0.4166666667
1
1
1
1
0.5
1
0.4166666667
0.1666666667
1
0.5588235294
1
1
0.4166666667
0.6295677362
0.1666666667
1
0.6773654476
1
1
0.25
1
0.4166666667
1
1
1
0.1666666667
0.25
1
1
1
0.5
1
0.6773654476
1
0.4166666667
0.1666666667
0.1666666667
0.1666666667
0.4166666667
1
0.3666803232
0.3666803232
1
0.1666666667
0.5
0.1666666667

0.25
1
1
1
0.5588235294
1
1
1
1
0.4166666667
1
0.4166666667
1
0.4166666667
1
1
0.5
1
0.4166666667
1
0.4166666667
0.5
0.5588235294
0.4166666667
0.376114082
0.4166666667
1
1
0.376114082
1
0.5588235294
1
1
1
1
1
1
1
0.25
1
0.25
0.25
0.4166666667
0.376114082
0.6256684492
1
1
0.1666666667
0.1666666667
1
1

1
1
0.061942959
1
1
1
1
1
0.6256684492
1
1
0.4178238744
0.5588235294
0.25
1
0.1666666667
0.6256684492
1
1
1
1
1
0.5588235294
0.5588235294
1
1
1
1
1
0.5588235294
1
0.6256684492
1
0.4166666667
1
1
1
0.4166666667
0.4166666667
1
1
0.4166666667
1
0.4166666667
1
1
1
1
1
1
1

0.5
0.376114082
1
1
1
0.6295677362
1
1
0.5588235294
1
0.3666803232
0.5
0.5
0.376114082
0.061942959
1
0.1666666667
1
0.1666666667
0.1666666667
1
1
1
1
0.5
1
1
0.5588235294
0.4166666667
0.6295677362
0.1666666667
0.6256684492
1
0.6256684492
0.5588235294
0.6256684492
0.2869875223
0.376114082
0.25
1
0.25
0.25
0.1379790553
1
1
1
1
1
1
1
1

1
0.6256684492
0.6256684492
1
0.25
1
1
0.1666666667
1
0.4166666667
1
0.5588235294
0.376114082
0.4166666667
1
0.6256684492
0.6256684492
0.25
0.5588235294
1
1
1
1
0.25
1
1
0.6295677362
0.5
1
0.5588235294
1
1
1
1
1
1
0.1666666667
0.1666666667
0.5588235294
0.5588235294
0.5588235294
1
1
1
1
0.061942959
1
1
1
1
1

1
1
1
1
1
1
0.5588235294
0.1379790553
1
1
0.4166666667
1
0.4166666667
1
1
0.4166666667
1
1
1
1
1
1
0.5
1
0.5
1
0.6256684492
0.4166666667
1
0.6256684492
0.5
0.5588235294
0.4166666667
1
1
0.4166666667
0.4166666667
0.2869875223
1
1
1
1
1
1
1
1
1
0.25
0.6295677362
1
1

0.3666803232
1
1
0.6256684492
1
1
1
1
0.5
1
1
1
0.4166666667
1
1
1
1
0.1666666667
0.1666666667
1
1
0.4166666667
0.5588235294
1
1
0.6256684492
1
0.4166666667
1
0.5588235294
0.4166666667
0.4166666667
0.6256684492
0.4166666667
1
1
1
1
0.25
1
0.6256684492
1
0.4166666667
1
1
1
1
1
0.5588235294
1
1

1
0.6256684492
0.6256684492
1
0.1666666667
0.6773654476
0.4178238744
1
1
1
1
1
1
1
1
1
0.1666666667
0.5588235294
1
1
1
0.0637254902
0.4166666667
1
1
1
1
0.25
0.25
0.4178238744
1
1
0.1247771836
0.6256684492
0.6295677362
1
1
0.4166666667
0.25
1
1
0.25
1
1
0.2869875223
1
0.25
1
1
1
1

0.4166666667
1
1
0.4166666667
1
1
0.4178238744
0.4178238744
1
1
0.2869875223
0.6256684492
0.4166666667
0.4166666667
1
0.6256684492
0.6256684492
0.209972112
1
1
0.6256684492
0.6256684492
1
0.25
1
0.376114082
1
1
1
1
0.4166666667
1
0.061942959
0.5588235294
0.376114082
1
0.4166666667
1
0.5
0.4166666667
0.4166666667
0.6256684492
1
1
1
0.4166666667
0.4166666667
0.4166666667
0.2869875223
1
1

0.6256684492
0.6256684492
1
1
0.6256684492
1
0.6773654476
1
1
1
1
1
1
1
1
1
0.5588235294
0.6256684492
1
0.5588235294
0.1666666667
0.2869875223
1
0.1666666667
1
1
1
1
1
1
0.4166666667
1
0.2869875223
0.5588235294
1
1
0.209972112
1
0.25
0.25
0.5
1
0.5
0.5
0.6256684492
0.5
1
1
1
0.5588235294
0.5588235294

0.5588235294
0.5588235294
1
0.5
0.5
1
1
1
1
1
1
1
1
0.376114082
1
1
1
1
1
1
1
1
1
0.4166666667
1
0.4166666667
1
0.25
0.5588235294
0.5588235294
1
0.4166666667
0.4166666667
0.4166666667
0.4166666667
0.4166666667
0.4166666667
0.4166666667
0.25
1
1
1
1
0.25
0.4166666667
0.3666803232
1
1
1
0.4166666667
0.1379790553

0.5
1
0.3666803232
1
0.6256684492
0.0272914151
0.6256684492
1
0.376114082
0.25
1
1
1
0.061942959
0.3666803232
0.3666803232
0.061942959
0.6256684492
0.25
1
1
0.4166666667
1
1
0.5588235294
1
1
0.5588235294
1
0.4166666667
1
1
0.6256684492
0.5588235294
0.5588235294
0.5588235294
0.6256684492
1
1
1
1
0.1666666667
1
1
0.4166666667
1
0.4166666667
1
1
1
1

1
1
1
1
0.5
1
1
1
0.6256684492
1
0.5
1
1
1
0.6256684492
1
1
0.5588235294
0.209972112
1
1
1
1
0.4166666667
1
0.1666666667
1
0.5
0.4166666667
0.5
1
0.5
1
0.25
0.5
1
1
1
0.5
0.1666666667
0.5
1
1
1
1
1
1
0.5
1
1
0.2869875223

0.6256684492
1
1
1
1
1
1
1
1
1
1
1
1
1
1
0.4166666667
0.2869875223
0.4166666667
0.5588235294
0.5588235294
0.4166666667
0.5588235294
0.25
0.4166666667
1
1
1
1
0.1666666667
1
0.5588235294
0.5588235294
0.4166666667
0.1666666667
1
0.1379790553
0.5588235294
0.5588235294
0.5588235294
0.5588235294
0.6256684492
0.6256684492
1
0.6256684492
0.6256684492
0.25
1
0.5588235294
0.25
1
0.376114082

1
0.6256684492
0.5588235294
0.1666666667
1
0.5588235294
1
1
0.376114082
1
0.5588235294
0.25
0.25
1
1
0.25
0.25
0.25
0.5588235294
0.25
0.376114082
1
1
0.25
0.376114082
0.376114082
1
0.1247771836
0.25
1
1
1
1
0.376114082
1
1
1
0.376114082
0.6773654476
1
1
0.4178238744
0.376114082
0.4178238744
0.376114082
0.5588235294
0.5588235294
0.5588235294
0.5588235294
1
1

0.5588235294
0.1666666667
0.5588235294
0.5588235294
0.2869875223
1
0.5588235294
0.1666666667
0.5588235294
1
1
1
0.1247771836
0.1247771836
1
0.4166666667
0.4166666667
0.4166666667
0.4166666667
0.4166666667
0.4166666667
0.4166666667
0.4166666667
0.4166666667
0.4166666667
0.4166666667
0.4166666667
0.4166666667
1
1
0.4166666667
0.4166666667
0.4166666667
0.4166666667
0.4166666667
0.4166666667
0.4166666667
0.1666666667
0.6256684492
0.5588235294
1
1
0.5
1
1
0.376114082
1
0.6256684492
1
1
1

1
0.5
1
1
0.6256684492
1
0.6256684492
0.5
1
1
1
0.25
0.25
1
0.4166666667
0.4166666667
0.4166666667
0.4166666667
0.4166666667
0.4166666667
0.4166666667
1
0.4166666667
0.4166666667
1
1
1
1
1
1
1
1
1
1
0.1666666667
0.4166666667
0.4166666667
1
1
1
0.4166666667
1
1
1
1
1
1
1
1
1
1

1
1
1
1
1
0.4166666667
1
1
0.1379790553
0.2869875223
0.1379790553
1
0.2869875223
0.1379790553
1
0.1379790553
1
0.1379790553
0.1379790553
1
1
1
0.6256684492
0.2869875223
0.209972112
0.6773654476
0.6773654476
1
1
0.6773654476
0.6773654476
1
1
1
1
1
0.2869875223
1
1
1
1
1
1
1
1
1
0.4166666667
0.4166666667
0.6256684492
1
1

0.4166666667
1
0.6256684492
0.4166666667
1
0.4166666667
1
0.4166666667
1
0.4166666667
1
0.4166666667
1
1
1
0.4166666667
1
1
1
1
1
1
0.376114082
1
1
1
0.25
1
0.4166666667
1
0.6773654476
1
1
0.4166666667
0.2869875223
0.5588235294
1
0.4178238744
0.1666666667
1
0.5588235294
1
1
1
1
1
0.5
0.2869875223
0.2869875223
0.2869875223
0.2869875223

0.2869875223
0.2869875223
1
0.25
0.5
0.5
0.1379790553
0.1666666667
0.25
0.2869875223
0.5
1
1
0.2869875223
0.5
1
1
0.4166666667
1
0.1666666667
1
1
1
1
1
1
1
1
0.061942959
0.25
1
1
0.25
0.25
1
1
1
0.1666666667
1
1
1
0.25
0.5588235294
0.4166666667
1
0.6256684492
0.6256684492
0.6256684492
0.4166666667
1
0.0637254902

0.0637254902
1
0.0637254902
0.1666666667
1
1
1
1
0.1247771836
0.5
1
0.25
0.2869875223
0.4166666667
0.376114082
0.5588235294
0.6256684492
1
0.1666666667
0.5588235294
0.4166666667
0.5
1
0.5
1
0.25
0.6256684492
0.4166666667
0.4166666667
0.1666666667
1
0.5
1
1
1
0.4166666667
0.4166666667
0.4166666667
1
1
0.4166666667
0.4166666667
0.1379790553
0.6256684492
1
0.6256684492
0.25
1
1
1
0.6256684492

1
1
0.209972112
0.5588235294
0.5588235294
0.1666666667
0.6295677362
1
1
1
1
1
0.6256684492
1
0.5
1
0.4166666667
0.6256684492
0.4166666667
0.4166666667
1
1
1
1
1
1
1
1
1
0.061942959
1
1
1
0.5
1
1
1
0.5588235294
0.209972112
0.4166666667
1
0.5588235294
1
0.4166666667
0.4166666667
0.6256684492
0.4166666667
1
0.5588235294
0.25
1

1
0.6295677362
1
0.5588235294
0.6773654476
1
0.5588235294
0.4166666667
0.4166666667
0.4166666667
0.5588235294
0.6256684492
0.6256684492
1
0.5588235294
0.5588235294
1
1
0.4166666667
1
1
1
1
0.1247771836
0.6256684492
1
1
1
1
0.6295677362
0.2869875223
0.2869875223
0.2869875223
0.4166666667
0.2869875223
0.2869875223
1
0.4166666667
0.209972112
0.209972112
0.209972112
0.25
1
0.1379790553
0.5
1
1
0.5588235294
0.5588235294
1
0.5588235294

1
1
1
1
0.25
0.376114082
1
1
1
0.5
1
0.25
1
1
1
0.4166666667
1
1
1
0.4166666667
1
0.5588235294
1
1
0.2869875223
0.4166666667
1
1
1
0.1666666667
1
1
0.4166666667
1
0.4166666667
0.1666666667
0.4166666667
1
0.5588235294
1
1
1
0.5588235294
0.4166666667
0.4166666667
0.4166666667
1
1
0.209972112
1
0.5588235294

1
1
1
0.2869875223
1
1
1
0.061942959
0.4166666667
1
0.5
1
1
1
0.25
0.5
0.6256684492
0.6256684492
1
0.6256684492
0.6256684492
1
1
0.25
1
1
1
1
1
0.5588235294
0.5588235294
0.4166666667
0.5588235294
1
1
1
0.5
1
1
0.376114082
1
1
0.5
1
1
0.5
1
1
1
1
1

0.4166666667
0.5588235294
0.1666666667
1
1
1
0.209972112
0.5
1
1
0.4166666667
0.4166666667
0.5588235294
0.1379790553
1
1
1
1
1
1
1
0.1666666667
0.4166666667
1
1
1
1
1
1
1
1
1
1
0.5
0.5
0.5
0.5
1
0.5
1
1
0.4166666667
1
1
0.5
1
0.6256684492
1
1
1
0.5

1
1
0.5
1
1
0.5
1
1
0.1379790553
1
1
0.6295677362
0.1379790553
0.6773654476
0.4166666667
1
1
1
0.5588235294
0.5588235294
1
0.2869875223
1
0.209972112
0.4166666667
1
1
1
1
0.25
1
0.6256684492
0.1666666667
1
1
1
1
0.6295677362
0.4166666667
0.6256684492
1
1
1
1
0.5
1
0.25
0.4166666667
0.25
1
1

1
1
1
0.5588235294
0.5588235294
0.4166666667
0.5588235294
1
0.2869875223
0.2869875223
1
1
1
1
1
0.6256684492
0.5588235294
0.5588235294
1
0.5588235294
1
1
1
0.6256684492
0.6256684492
0.6256684492
0.6256684492
0.6256684492
1
1
1
1
1
0.6256684492
1
0.25
0.25
0.5588235294
0.5588235294
1
1
0.4166666667
1
1
1
1
1
1
1
1
1

0.5
0.4166666667
0.6256684492
0.25
0.5588235294
1
1
1
0.5588235294
0.6773654476
0.25
1
0.25
0.5588235294
1
0.25
1
0.6256684492
1
0.4166666667
0.5588235294
1
0.6256684492
1
1
1
1
1
1
0.25
1
0.4166666667
0.4166666667
0.4166666667
0.5588235294
0.376114082
1
0.5588235294
1
1
1
0.209972112
1
1
1
0.5
0.376114082
1
1
0.25
1

1
1
1
1
0.5588235294
0.25
0.1666666667
0.4166666667
1
1
0.2869875223
0.376114082
1
1
1
0.5
1
0.25
0.5588235294
0.5588235294
0.1666666667
0.2869875223
1
1
0.6256684492
1
1
0.5588235294
1
0.5
1
1
0.5588235294
1
0.5
0.5
0.6773654476
0.25
0.5
0.4166666667
0.6773654476
0.6256684492
1
1
1
1
1
0.25
1
1
0.5

1
1
1
1
0.5588235294
0.5
0.1666666667
0.1666666667
0.5
0.1379790553
1
0.4166666667
1
0.25
1
1
0.4166666667
0.5588235294
1
0.6256684492
1
1
0.4166666667
1
1
0.25
0.5588235294
1
1
1
1
1
0.4166666667
1
1
1
0.4166666667
1
1
1
1
1
1
1
0.1666666667
1
1
1
1
1
1

1
1
1
1
1
0.4166666667
0.376114082
0.1247771836
0.6256684492
0.5
1
1
1
1
1
1
1
1
1
1
1
1
1
1
0.6295677362
0.6295677362
0.6295677362
0.4166666667
1
0.4166666667
0.4166666667
1
1
1
1
1
0.5588235294
0.5588235294
0.5588235294
0.5588235294
0.4166666667
1
1
1
1
0.25
1
1
1
1
0.1666666667

1
0.5
1
0.4166666667
0.5588235294
1
0.376114082
0.5
0.6256684492
1
0.4166666667
0.25
1
1
0.25
0.1666666667
0.5
1
0.5588235294
0.5588235294
0.6256684492
0.4166666667
1
0.4166666667
1
1
1
0.376114082
1
0.6256684492
0.4166666667
0.6256684492
1
1
1
1
0.25
1
0.4166666667
1
1
0.6256684492
0.4166666667
0.6256684492
0.4166666667
1
0.4166666667
1
1
0.5588235294
1

1
1
1
0.6256684492
0.1666666667
0.4166666667
1
0.25
1
1
0.376114082
1
1
0.6256684492
1
1
1
1
1
1
1
0.6256684492
0.6256684492
1
0.4166666667
0.5
1
0.4166666667
1
1
0.25
1
1
1
0.4166666667
0.5588235294
1
1
0.5588235294
0.6256684492
1
1
0.061942959
1
1
1
1
0.6256684492
0.6256684492
0.376114082
0.4166666667

1
0.376114082
0.4166666667
0.1666666667
0.5
0.4166666667
0.5
1
1
0.4166666667
0.4166666667
1
1
0.25
0.25
0.25
0.25
0.25
0.25
0.4166666667
0.6773654476
0.5
0.4166666667
0.4166666667
1
1
1
1
1
1
1
1
1
1
0.376114082
1
1
0.6773654476
1
0.5
0.1666666667
0.2869875223
1
0.5588235294
1
1
1
0.4166666667
1
1
1

0.6256684492
1
0.4166666667
0.5588235294
0.4166666667
0.6773654476
0.6773654476
1
1
0.5588235294
0.4240540589
1
1
0.376114082
0.25
1
1
1
0.3666803232
1
0.25
0.1666666667
0.1666666667
0.1666666667
1
1
1
0.25
0.5
1
0.6256684492
1
0.4166666667
1
1
1
1
0.5588235294
0.1666666667
0.2869875223
1
1
1
0.4166666667
1
1
0.2869875223
0.6256684492
1
0.25
0.25

0.25
0.4166666667
0.1666666667
1
0.5588235294
1
0.4178238744
1
0.6295677362
0.376114082
0.4166666667
1
0.6295677362
1
0.4166666667
0.1666666667
0.4166666667
0.4166666667
1
0.2869875223
1
0.3666803232
0.6256684492
0.25
1
1
1
1
1
1
1
0.1379790553
0.6256684492
0.376114082
0.25
0.1666666667
1
1
0.1379790553
1
0.376114082
1
1
1
1
1
0.5
0.5
1
1
1

1
0.1666666667
1
1
1
0.4166666667
1
0.5
1
0.1666666667
1
1
0.4166666667
1
0.4166666667
1
1
1
1
1
1
0.4166666667
0.4166666667
0.5
1
1
1
1
1
1
1
1
1
0.6256684492
0.4166666667
0.5588235294
0.209972112
0.5588235294
0.5588235294
1
0.376114082
1
0.2869875223
1
1
1
0.6256684492
1
0.4166666667
1
0.4166666667

1
1
1
0.5588235294
0.4166666667
1
0.5588235294
1
0.4166666667
1
0.5588235294
0.5588235294
0.5588235294
1
1
0.6256684492
0.6256684492
1
0.5588235294
0.5588235294
0.5588235294
0.5588235294
0.5588235294
1
0.5
0.5588235294
0.5588235294
0.5588235294
0.5588235294
0.6256684492
0.6256684492
0.6256684492
1
0.5588235294
0.5588235294
0.5588235294
0.5588235294
0.4166666667
1
1
0.6256684492
0.5588235294
0.5588235294
0.2869875223
1
0.5588235294
0.5588235294
1
1
0.4166666667
0.4166666667

1
0.2869875223
1
1
1
1
1
0.4166666667
1
1
1
0.376114082
0.6256684492
1
1
1
1
0.1666666667
1
1
1
1
0.3666803232
1
0.4166666667
0.6256684492
0.6256684492
0.5588235294
1
1
0.25
1
0.5588235294
0.5588235294
0.1666666667
1
0.5
0.6773654476
0.4166666667
1
1
1
0.5
1
1
1
0.4166666667
1
1
1
1

1
0.4166666667
0.5
0.5
0.5
1
1
1
1
1
1
1
1
0.5588235294
0.4166666667
1
1
1
1
0.6256684492
0.6256684492
0.5588235294
1
1
1
0.25
0.6256684492
0.1666666667
0.1666666667
0.1666666667
1
0.5
0.4166666667
1
1
1
1
1
1
0.4166666667
0.4166666667
0.5
0.6256684492
0.6256684492
1
1
1
0.5588235294
0.4166666667
0.6256684492
1

1
1
1
1
1
0.061942959
1
1
1
1
1
1
1
0.5588235294
0.6256684492
0.6256684492
1
0.4166666667
1
0.4166666667
0.2869875223
1
0.4166666667
0.5
0.5
1
0.6773654476
0.4166666667
1
1
0.25
0.6256684492
1
1
0.6256684492
0.5
1
1
0.5
0.6295677362
1
0.5588235294
1
0.5588235294
0.5588235294
0.5588235294
0.5
0.5588235294
0.5588235294
0.4178238744
1

0.5588235294
1
1
0.4166666667
1
1
0.2869875223
1
0.0637254902
0.4166666667
1
0.4166666667
1
1
0.0637254902
1
0.5588235294
0.5588235294
0.4166666667
1
1
1
1
0.5
1
1
1
0.4178238744
0.6295677362
0.4166666667
0.5
0.25
1
1
1
1
1
1
1
0.209972112
0.4166666667
1
1
1
1
0.376114082
0.25
0.25
0.6256684492
1
1

1
1
1
0.5588235294
0.4166666667
0.6773654476
1
0.5588235294
0.4166666667
0.4166666667
1
1
0.25
0.6256684492
0.4166666667
0.4166666667
1
0.1666666667
0.5
1
0.25
1
1
0.5588235294
1
1
1
1
0.4166666667
1
1
0.5588235294
1
1
1
1
1
0.25
1
0.6256684492
0.2869875223
0.6256684492
1
1
1
0.1666666667
0.1666666667
1
1
1
1

1
0.4166666667
1
1
1
1
1
1
1
1
0.6773654476
1
1
0.4166666667
0.1666666667
1
1
1
1
1
0.4166666667
1
1
1
0.5588235294
1
1
1
0.5
1
0.25
1
1
1
1
0.6256684492
1
0.5
1
0.5
0.4166666667
1
1
1
0.4166666667
0.4166666667
0.2869875223
0.4166666667
1
1
1

0.1666666667
1
1
0.1666666667
0.1379790553
0.376114082
0.3666803232
0.25
0.25
0.25
1
1
0.5588235294
1
1
0.209972112
1
0.25
1
0.6256684492
1
0.5588235294
0.1247771836
0.4166666667
1
0.4166666667
1
1
0.376114082
0.1379790553
0.5588235294
1
0.4166666667
0.25
0.4166666667
0.5
0.1666666667
0.6295677362
0.6773654476
1
0.25
0.5588235294
0.4166666667
0.4166666667
1
1
0.4166666667
1
1
1
1

1
0.1666666667
1
1
0.4166666667
0.376114082
1
0.376114082
0.1247771836
0.5588235294
0.4166666667
0.5588235294
0.5588235294
1
0.25
0.1379790553
0.1379790553
1
0.25
0.5
1
0.376114082
1
1
1
1
0.2869875223
0.2869875223
1
0.4166666667
1
1
1
1
1
0.5588235294
1
0.4166666667
0.4166666667
0.4166666667
0.4166666667
1
1
1
1
1
1
0.5588235294
0.25
0.25
0.6256684492

1
0.1666666667
1
0.6295677362
0.6295677362
1
1
0.1666666667
0.5
1
1
0.6256684492
1
1
1
1
1
1
1
1
1
1
1
1
1
1
1
0.5588235294
1
1
1
1
0.376114082
1
1
1
1
0.5
0.6773654476
1
1
0.4166666667
0.25
0.5588235294
0.376114082
0.376114082
0.376114082
0.376114082
0.376114082
0.376114082
0.376114082

0.376114082
0.376114082
0.376114082
0.376114082
1
0.5588235294
0.6295677362
1
1
1
0.376114082
1
0.5588235294
0.4166666667
1
1
1
1
1
0.4166666667
1
1
1
0.3666803232
1
1
1
0.6256684492
0.4178238744
0.1666666667
1
0.4166666667
0.1666666667
1
0.4166666667
0.4166666667
1
0.4166666667
0.6256684492
1
1
1
1
1
0.4166666667
1
0.4166666667
0.6256684492
1
1
0.1666666667

0.6773654476
1
0.1666666667
1
1
1
0.5588235294
0.6256684492
0.6773654476
1
1
0.25
1
0.376114082
0.4166666667
0.4166666667
0.6256684492
0.4166666667
0.1666666667
0.376114082
1
0.4178238744
1
1
1
0.1247771836
0.6256684492
1
1
1
0.376114082
1
0.5
0.6256684492
1
0.6295677362
0.4166666667
1
1
1
1
0.25
1
0.1666666667
0.3666803232
0.4166666667
0.5
1
0.25
1
1

1
0.5588235294
0.4240540589
1
1
1
1
0.6256684492
1
1
0.1666666667
1
1
1
1
1
1
1
0.4166666667
0.4166666667
0.4166666667
0.4166666667
1
0.4166666667
0.4166666667
1
1
1
1
0.5
0.6256684492
0.4166666667
0.1666666667
0.25
1
1
0.25
1
0.4166666667
0.2869875223
0.1379790553
0.6256684492
0.5
1
1
0.2869875223
1
1
1
1
1

0.2869875223
0.2869875223
0.6256684492
1
1
1
0.4166666667
0.4166666667
1
1
1
1
1
0.4166666667
1
0.4166666667
0.6256684492
0.4166666667
1
1
1
1
1
0.25
0.25
0.25
0.25
0.1666666667
0.1666666667
0.1247771836
1
1
1
1
0.4166666667
0.2869875223
1
1
1
1
1
1
1
1
1
1
1
1
1
1
1

1
0.4166666667
1
0.4166666667
1
0.4166666667
1
1
1
0.5588235294
0.5588235294
0.6773654476
0.6773654476
1
1
0.4166666667
0.6256684492
1
1
1
0.2869875223
0.1666666667
0.4166666667
0.4166666667
0.5588235294
0.5588235294
1
1
0.5588235294
0.4166666667
1
1
1
1
1
1
1
0.4166666667
0.25
0.5
0.3666803232
1
0.4166666667
1
1
0.4166666667
0.4166666667
1
1
0.4166666667
0.4166666667

1
1
0.25
0.4166666667
1
1
1
1
1
1
0.5
1
1
0.4166666667
0.4166666667
1
0.5
0.6773654476
0.2869875223
1
0.6256684492
1
1
1
1
1
1
0.6256684492
1
1
0.4166666667
0.5
1
1
0.25
1
0.25
0.25
1
1
0.5
0.4166666667
0.5588235294
0.4166666667
1
0.4166666667
1
0.4166666667
1
0.6256684492
1

1
1
1
1
0.2869875223
1
1
1
1
1
0.4166666667
0.4166666667
0.4166666667
1
1
0.5588235294
1
0.376114082
1
0.3666803232
1
1
1
1
1
0.4166666667
0.4178238744
0.4178238744
1
1
0.5588235294
0.376114082
0.4166666667
1
1
1
0.5588235294
0.25
0.376114082
0.5588235294
0.5588235294
0.4166666667
1
1
0.2869875223
0.1379790553
0.4166666667
1
1
0.5588235294
0.5588235294

0.4166666667
0.6256684492
0.6256684492
1
0.376114082
1
0.6295677362
0.6256684492
1
1
1
1
1
1
1
1
1
1
1
1
0.376114082
1
0.4166666667
0.5588235294
0.1379790553
1
0.25
0.4166666667
0.4166666667
1
1
0.1379790553
0.25
1
1
0.5
1
1
1
1
1
1
1
1
1
1
1
0.209972112
0.209972112
0.209972112
0.209972112

0.209972112
0.209972112
0.209972112
0.209972112
0.209972112
0.209972112
1
0.209972112
1
1
1
1
1
1
1
1
1
1
1
1
0.376114082
0.376114082
0.376114082
0.376114082
0.376114082
1
1
0.376114082
1
1
1
1
0.209972112
0.209972112
1
1
0.6256684492
1
1
0.25
0.6256684492
1
1
1
0.1379790553
1
0.376114082
1
1
1
1

0.5
0.5
0.1666666667
0.5588235294
1
1
1
1
0.5588235294
1
0.6256684492
0.6295677362
0.5588235294
1
0.4166666667
0.5588235294
0.6256684492
0.4166666667
0.1666666667
0.6256684492
1
1
0.5588235294
1
0.2869875223
1
1
1
1
1
1
1
1
1
0.1379790553
0.4166666667
0.5
1
1
1
0.4166666667
0.4166666667
0.4166666667
1
0.5588235294
1
1
1
0.4166666667
1
1

1
1
1
0.5588235294
1
1
1
1
1
0.5
0.3666803232
0.4166666667
1
0.4178238744
0.376114082
1
1
0.25
0.4166666667
1
1
0.25
0.5588235294
1
0.4178238744
1
1
1
0.5588235294
0.5588235294
1
0.5588235294
1
0.6256684492
1
1
1
1
0.0637254902
0.1666666667
0.1666666667
1
0.4166666667
0.25
0.25
0.25
0.6256684492
0.5588235294
0.6256684492
1
0.2869875223

0.6773654476
0.1666666667
1
0.4166666667
0.6295677362
1
1
1
1
1
0.6256684492
0.6256684492
0.4166666667
0.6256684492
0.6256684492
0.5
0.5
0.5
0.5
0.5
0.5
1
1
1
1
1
1
1
1
1
0.4166666667
0.4166666667
0.4166666667
0.5
0.5
0.5588235294
0.5588235294
0.5588235294
1
1
1
1
1
0.3666803232
0.3666803232
1
0.5588235294
1
1
0.4166666667
1

1
1
1
1
1
1
1
1
1
1
1
1
0.376114082
1
1
1
1
1
0.2869875223
1
1
0.2869875223
0.5
0.6256684492
1
1
0.4166666667
1
1
0.4166666667
0.4166666667
1
1
1
1
1
1
1
1
1
1
1
0.376114082
0.4166666667
0.4166666667
1
1
1
0.6256684492
0.1666666667
1

1
1
1
0.4166666667
0.4166666667
0.376114082
0.6256684492
1
0.5588235294
1
0.376114082
1
1
1
1
1
0.5588235294
1
0.4178238744
0.6773654476
1
0.5
0.5588235294
0.376114082
0.376114082
0.5
0.5588235294
1
0.5588235294
0.209972112
1
1
1
1
0.4166666667
1
0.6256684492
1
1
1
1
1
1
0.6256684492
0.6256684492
0.6256684492
0.25
0.25
1
1
0.5588235294

0.4166666667
1
0.25
0.6773654476
1
1
0.3666803232
0.4166666667
0.4166666667
0.5
1
1
0.5588235294
1
1
1
1
0.4166666667
1
1
1
1
1
0.5588235294
0.2869875223
1
0.25
0.5
0.6256684492
0.6256684492
0.6256684492
1
1
0.1666666667
1
1
0.4178238744
1
0.4166666667
0.4166666667
0.5
0.6295677362
1
1
0.4166666667
0.4166666667
0.4166666667
0.4166666667
0.6256684492
0.6256684492
1

1
1
0.6256684492
1
0.5588235294
0.6256684492
1
1
1
0.25
0.1379790553
0.2869875223
0.2869875223
1
0.1379790553
1
0.2869875223
1
1
1
0.25
1
1
1
1
0.5588235294
0.061942959
0.4166666667
1
1
1
1
0.5588235294
0.5588235294
1
0.4166666667
1
1
0.6256684492
0.5588235294
0.5588235294
1
1
0.5
0.5
0.5
1
1
1
0.5
0.6295677362

0.5588235294
0.5588235294
0.5588235294
0.2869875223
0.5
0.5
0.5
0.5
0.5
0.5
1
0.4166666667
1
1
0.5588235294
0.1247771836
0.376114082
1
0.25
0.5
1
1
0.4166666667
1
0.5588235294
0.6256684492
1
0.4166666667
1
1
1
1
1
0.376114082
1
0.6256684492
0.6256684492
1
0.4166666667
0.376114082
0.4166666667
0.25
0.376114082
1
1
0.5588235294
0.4166666667
0.1666666667
0.25
0.5588235294
0.25

1
0.5
0.5588235294
0.6256684492
0.25
1
1
1
1
0.6256684492
1
1
1
1
0.1666666667
0.4166666667
0.6256684492
1
0.25
0.25
1
0.5
0.5588235294
0.4166666667
0.5
1
0.4166666667
0.1666666667
0.5
1
0.5
0.2869875223
1
1
0.25
0.5588235294
0.4166666667
0.4166666667
0.376114082
0.6256684492
1
1
0.5588235294
0.6256684492
1
0.4166666667
0.5588235294
0.209972112
1
1
0.5

1
1
1
0.5588235294
0.03042881375
0.376114082
1
1
1
1
1
0.376114082
0.5588235294
0.6256684492
0.4166666667
0.4166666667
1
1
1
0.1247771836
0.209972112
0.376114082
1
1
1
1
0.376114082
0.4166666667
0.25
1
0.6256684492
0.6256684492
1
1
0.6256684492
0.6256684492
1
1
1
0.6773654476
0.4166666667
0.3666803232
0.3666803232
0.3666803232
1
1
0.4178238744
1
0.3666803232
0.6256684492
0.6256684492

0.6256684492
0.6256684492
0.4166666667
0.2869875223
1
1
1
1
0.1379790553
0.6295677362
1
1
0.25
1
1
0.6256684492
1
0.4166666667
0.0637254902
0.0637254902
1
1
0.4166666667
1
1
1
1
0.4166666667
0.5588235294
1
1
1
1
1
1
1
0.4166666667
0.5588235294
0.4166666667
0.25
0.4166666667
0.6256684492
0.6256684492
1
1
1
1
0.7210233593
0.7210233593
1
1

0.2869875223
0.5588235294
0.4166666667
0.4166666667
1
0.4166666667
0.6256684492
0.6256684492
0.5
1
1
1
1
1
0.5
1
1
1
0.2869875223
0.4166666667
0.4166666667
0.4166666667
1
0.5588235294
1
0.6256684492
0.25
1
0.5588235294
0.061942959
0.2869875223
1
1
0.1666666667
1
1
1
0.5588235294
1
1
0.1666666667
1
0.4166666667
1
1
1
0.4166666667
1
0.4166666667
1
1

0.5
1
0.5588235294
0.6256684492
0.5588235294
1
0.4166666667
0.376114082
0.6256684492
0.4166666667
1
0.4166666667
1
0.5588235294
1
0.6256684492
1
1
1
0.7210233593
1
0.25
0.25
0.2869875223
0.2869875223
0.1666666667
1
0.25
1
1
0.1379790553
0.1379790553
0.6773654476
0.6256684492
0.4166666667
0.4166666667
0.5
1
0.5588235294
0.5588235294
1
1
1
1
1
0.5588235294
1
1
1
1
1

0.6256684492
1
0.4166666667
1
1
1
0.5588235294
0.4166666667
0.5
1
1
0.5588235294
0.5588235294
0.5588235294
1
1
0.5
0.5
0.1666666667
1
0.4166666667
0.5
0.209972112
0.5588235294
0.5588235294
0.5
0.6773654476
1
1
0.4166666667
1
1
1
1
0.4166666667
0.061942959
0.061942959
0.4166666667
0.6256684492
0.6256684492
1
1
1
1
1
1
1
1
0.6256684492
1
0.4166666667

0.4166666667
0.5588235294
0.1666666667
0.061942959
0.4166666667
0.5
0.4166666667
0.6295677362
1
1
0.4166666667
1
0.25
0.376114082
0.1666666667
0.25
0.25
0.6256684492
1
1
0.5588235294
0.5
0.376114082
0.25
0.5
0.6256684492
1
1
1
0.1666666667
0.1666666667
1
1
0.4166666667
0.5588235294
1
0.5
1
1
1
1
0.4166666667
0.209972112
0.4166666667
0.4166666667
0.4166666667
0.1379790553
1
1
1
1

1
0.5588235294
1
0.4166666667
1
1
0.5588235294
1
0.5588235294
0.25
0.5588235294
0.6256684492
1
1
0.6256684492
0.5588235294
1
1
1
1
0.5588235294
0.5588235294
0.5588235294
0.5
1
1
0.6773654476
0.6773654476
0.2869875223
0.6256684492
1
1
0.5588235294
1
1
1
0.5
1
1
1
1
1
0.5
1
0.4166666667
1
0.5588235294
1
1
1
1

0.4166666667
1
1
1
1
1
1
0.4166666667
0.5588235294
0.4166666667
1
1
1
1
1
1
1
0.4166666667
1
1
1
1
1
1
1
0.25
0.5588235294
0.061942959
1
0.4166666667
1
1
1
1
0.25
0.5588235294
0.1666666667
1
0.4166666667
0.5588235294
0.5588235294
1
0.2869875223
0.5588235294
0.6256684492
0.3666803232
1
1
0.5588235294
0.5588235294
0.5588235294

1
1
1
0.4166666667
0.4166666667
0.5588235294
0.5
1
1
0.6256684492
0.25
1
1
0.6256684492
0.6256684492
0.4166666667
1
1
1
0.4166666667
1
1
1
0.061942959
0.4166666667
0.5588235294
0.1379790553
1
0.6256684492
0.6256684492
1
1
0.5588235294
1
0.4166666667
1
0.5588235294
1
1
1
0.6256684492
1
0.5588235294
0.1379790553
0.1379790553
0.1666666667
1
0.5588235294
0.6256684492
0.25
1

0.376114082
0.376114082
0.4166666667
0.4166666667
0.6256684492
1
1
1
1
0.4166666667
1
0.25
1
0.25
0.5588235294
0.5588235294
1
1
0.2869875223
1
0.6256684492
0.4166666667
0.6773654476
0.5588235294
1
0.5588235294
0.5588235294
1
0.6773654476
0.5588235294
1
1
0.4166666667
0.4166666667
0.25
1
0.25
0.4166666667
1
1
1
1
1
0.25
1
1
1
1
0.1666666667
1
0.4166666667

1
0.4166666667
0.5588235294
1
1
1
1
1
1
1
1
1
0.209972112
0.1666666667
0.1666666667
0.1666666667
0.6256684492
1
0.376114082
1
1
1
0.5588235294
1
1
1
1
0.376114082
0.6773654476
0.4166666667
0.5588235294
0.376114082
0.5
1
0.1666666667
1
1
0.4166666667
0.4166666667
0.2869875223
0.4166666667
0.6256684492
1
0.6256684492
1
0.5588235294
0.5588235294
1
0.4166666667
0.5588235294
0.4166666667

0.4178238744
0.4166666667
0.4178238744
0.5588235294
1
0.4178238744
0.4166666667
0.4166666667
1
1
0.4166666667
0.376114082
1
1
1
1
1
0.6256684492
0.3666803232
0.5
0.4166666667
1
0.376114082
1
1
0.5
1
0.25
1
0.6256684492
1
1
1
0.5588235294
0.4166666667
1
1
1
1
1
1
0.5
0.4166666667
1
1
0.1379790553
1
1
0.5
0.6256684492
0.1666666667

1
0.4166666667
0.376114082
0.376114082
1
0.1379790553
1
1
0.5588235294
1
1
0.25
1
0.4166666667
1
1
1
1
0.4166666667
1
1
1
0.4166666667
0.061942959
1
1
1
0.5588235294
0.6295677362
0.6295677362
1
0.25
0.2869875223
1
0.6295677362
1
1
0.5
0.1666666667
0.6773654476
1
1
1
1
0.2869875223
0.2869875223
1
0.4166666667
0.4166666667
1
1

1
0.5588235294
1
0.5588235294
1
1
1
1
1
1
1
0.4166666667
1
0.1379790553
1
0.4166666667
1
1
1
0.4166666667
0.5
1
0.6256684492
0.5588235294
0.5588235294
1
0.3666803232
0.3666803232
0.3666803232
0.6256684492
0.6256684492
0.6256684492
0.6256684492
0.209972112
0.6256684492
0.6256684492
0.209972112
1
1
0.4240540589
1
0.5588235294
1
1
1
0.1666666667
1
0.5588235294
0.061942959
1
1

0.4166666667
0.5588235294
1
0.3666803232
0.3666803232
1
1
1
0.209972112
0.5588235294
1
0.209972112
0.5
1
1
1
1
1
1
0.25
0.5588235294
0.6256684492
1
0.209972112
0.5588235294
0.209972112
0.209972112
0.6256684492
0.5588235294
1
0.25
1
1
1
1
1
1
1
0.4178238744
1
0.1666666667
0.5588235294
0.2869875223
0.2869875223
1
0.6295677362
1
1
0.6295677362
1
0.5588235294

1
1
1
0.1666666667
0.25
0.5588235294
0.209972112
1
1
1
0.209972112
1
1
0.4166666667
1
0.1666666667
0.6940287188
1
1
1
1
1
0.5
1
0.5
0.5
1
1
1
1
1
0.3666803232
0.3666803232
0.4166666667
1
0.4166666667
0.4166666667
0.4166666667
1
1
0.4166666667
0.6773654476
0.6773654476
0.25
1
1
0.4166666667
0.4166666667
0.4166666667
0.376114082
0.4166666667

0.4166666667
0.4166666667
1
1
1
0.5588235294
0.4166666667
0.1666666667
1
0.4166666667
1
1
0.6256684492
1
1
1
1
1
1
0.5588235294
0.6256684492
0.1379790553
1
0.2869875223
1
1
0.5
0.5
1
1
0.4166666667
1
0.2869875223
1
0.6256684492
1
0.4166666667
0.5588235294
1
0.5588235294
0.4166666667
1
1
1
1
0.6256684492
1
1
1
1
0.4166666667

0.4166666667
1
0.6256684492
0.4166666667
0.5588235294
1
0.5588235294
0.6256684492
1
0.5
0.4166666667
0.5588235294
0.5588235294
0.4166666667
1
0.5588235294
0.4166666667
0.5
1
0.25
0.4166666667
0.6256684492
0.4166666667
0.4166666667
0.4166666667
1
0.25
1
0.25
0.25
0.6256684492
0.5
1
0.6256684492
0.5588235294
1
0.25
0.25
0.25
0.25
0.25
1
1
0.4166666667
1
0.25
1
1
0.5588235294
0.6256684492
0.6256684492

0.6256684492
0.6256684492
0.6256684492
0.25
0.25
0.25
0.6256684492
1
1
1
1
1
0.5
1
1
1
1
1
1
0.1666666667
1
1
1
0.1379790553
0.376114082
1
1
1
0.4166666667
0.4166666667
1
1
1
1
1
1
0.6256684492
0.4166666667
0.4166666667
1
1
1
1
1
1
1
1
1
1
0.4166666667
1

1
1
1
0.4166666667
0.4166666667
1
1
1
0.5588235294
1
0.5588235294
1
0.376114082
0.6256684492
0.25
1
1
1
1
1
0.061942959
1
1
1
0.4166666667
1
0.5588235294
0.25
0.5588235294
0.4166666667
1
0.4166666667
1
0.6256684492
1
1
1
1
0.4166666667
0.4178238744
1
1
0.3666803232
1
0.6773654476
1
0.5
1
0.2869875223
1
1

1
0.6256684492
1
0.2869875223
1
1
0.1666666667
1
0.2869875223
0.7287727104
1
1
1
0.4166666667
1
0.5588235294
0.1247771836
0.1247771836
0.1247771836
1
1
0.4178238744
0.4166666667
0.6256684492
1
0.0637254902
1
0.4166666667
1
0.4166666667
1
1
0.5588235294
0.4166666667
0.25
0.25
1
0.4166666667
1
0.5588235294
1
0.5
1
0.4166666667
0.5
1
1
1
1
1
1

1
0.5
0.5
0.5588235294
0.5588235294
1
1
0.25
0.25
0.4166666667
1
0.6256684492
0.6256684492
0.6256684492
0.6256684492
0.6256684492
0.6256684492
0.25
0.25
0.4166666667
1
1
1
1
0.4166666667
0.1666666667
0.4178238744
1
0.5588235294
1
1
1
0.4166666667
0.6295677362
1
1
1
1
0.4166666667
0.376114082
1
1
0.4166666667
1
1
0.4166666667
0.4166666667
0.4166666667
1
1
1

0.4166666667
0.209972112
0.6940287188
0.209972112
0.209972112
1
0.4166666667
1
0.2869875223
0.4166666667
0.3666803232
0.4166666667
1
0.5588235294
1
0.2869875223
1
1
0.6256684492
0.6256684492
0.2869875223
1
0.4166666667
0.4166666667
1
0.5588235294
1
1
1
0.5588235294
0.4166666667
0.4166666667
0.1666666667
1
1
1
1
1
1
1
0.25
1
0.25
0.25
0.6256684492
0.4166666667
1
1
1
1
0.209972112

0.209972112
1
0.1247771836
1
1
1
1
0.2869875223
0.4166666667
1
1
1
1
1
1
0.6256684492
0.6256684492
1
1
1
1
0.5588235294
0.4166666667
1
1
0.2869875223
1
1
1
1
1
0.4166666667
1
0.4166666667
0.4166666667
0.4166666667
1
1
1
1
0.4166666667
0.5588235294
0.5588235294
0.4166666667
1
1
1
1
1
1
1

1
0.5588235294
0.4166666667
0.1247771836
1
1
1
0.5588235294
1
0.6256684492
0.6256684492
0.6256684492
0.6256684492
1
0.4166666667
0.376114082
0.4166666667
1
1
0.6256684492
1
1
1
1
0.6256684492
0.5588235294
1
1
0.5
0.5588235294
0.4166666667
1
0.4166666667
0.6295677362
1
1
0.25
1
1
1
0.5
1
0.4166666667
1
0.4166666667
0.5588235294
1
1
1
0.5588235294
0.6256684492

0.6256684492
0.6256684492
0.376114082
1
1
1
1
1
1
0.1666666667
1
1
0.4166666667
1
0.4166666667
0.5
0.4166666667
0.4166666667
1
1
1
1
1
1
1
0.5588235294
0.4166666667
1
0.5588235294
1
1
0.4166666667
0.1247771836
0.5
1
1
0.4166666667
0.4166666667
1
1
0.25
0.4166666667
1
0.4166666667
1
0.4166666667
0.5588235294
1
1
0.4166666667
0.4166666667

0.6256684492
1
1
0.1666666667
1
1
1
1
0.5588235294
1
0.5
1
1
0.4166666667
0.5
1
0.2869875223
0.2869875223
0.5588235294
0.4166666667
0.4166666667
0.5588235294
1
0.5
1
0.25
0.25
1
0.5
0.5
1
1
0.4166666667
0.5
1
1
1
1
1
0.4178238744
1
1
0.5588235294
1
0.376114082
0.4166666667
0.1666666667
1
0.5588235294
0.5588235294
1

0.5588235294
1
1
1
0.376114082
1
0.6256684492
1
1
1
1
1
1
1
1
1
1
0.4166666667
1
0.4166666667
0.1666666667
1
0.4166666667
0.4166666667
1
0.4166666667
0.4166666667
1
1
0.2869875223
1
1
1
1
0.1666666667
0.4166666667
1
1
0.6295677362
1
1
0.25
0.25
0.5
0.4166666667
0.4166666667
1
0.6773654476
1
1
1

0.6256684492
1
1
0.6256684492
0.5588235294
1
1
1
0.4166666667
1
1
1
1
0.25
1
1
1
1
0.4166666667
0.4166666667
1
1
1
0.4166666667
0.2869875223
0.25
0.209972112
1
1
0.6256684492
1
0.6256684492
0.4166666667
1
0.061942959
1
1
0.376114082
1
1
0.1666666667
1
1
0.2869875223
0.4166666667
0.6773654476
1
1
1
0.6256684492
0.5588235294

1
1
0.5588235294
0.1666666667
1
1
1
0.376114082
1
1
1
1
1
1
0.25
1
0.6295677362
0.5588235294
0.5
0.5
1
0.5588235294
0.4178238744
1
0.4166666667
0.6256684492
1
1
1
1
1
1
1
1
0.4166666667
1
1
0.376114082
1
1
0.6295677362
0.25
1
1
1
1
0.4166666667
0.1666666667
0.4166666667
1
1

1
0.6256684492
0.6256684492
1
1
1
1
1
1
1
0.5
0.25
1
0.5
0.6295677362
1
0.1666666667
0.376114082
0.4166666667
0.4166666667
0.4166666667
0.4166666667
1
0.5588235294
0.4178238744
1
1
1
0.2869875223
1
0.4166666667
0.4166666667
0.5
0.376114082
1
0.2869875223
1
0.4166666667
0.4166666667
1
0.5588235294
0.5
1
1
1
0.4166666667
0.4178238744
0.4178238744
0.5
1
0.5588235294

0.4166666667
0.1666666667
1
1
0.5588235294
0.376114082
1
1
1
0.5588235294
1
1
0.1379790553
1
1
0.6256684492
1
1
1
0.5588235294
0.25
1
0.4166666667
1
0.25
1
1
1
1
0.1379790553
0.5588235294
0.376114082
1
0.6256684492
1
1
1
1
1
0.209972112
1
0.5
1
1
1
1
1
1
0.1247771836
1
1

1
0.1247771836
1
1
1
0.4240540589
1
0.6295677362
0.25
0.4240540589
0.25
1
0.5
1
1
0.6256684492
0.4166666667
1
0.376114082
0.061942959
1
1
0.6256684492
0.6256684492
1
0.4166666667
0.4166666667
0.25
0.25
1
1
0.1666666667
1
1
1
1
1
0.25
0.2869875223
0.6256684492
1
0.1666666667
0.4166666667
0.6295677362
1
0.4166666667
0.25
0.5588235294
1
0.5
0.4166666667

1
1
1
1
0.376114082
1
0.5
0.4166666667
0.2869875223
1
0.2869875223
0.4166666667
0.4166666667
0.5588235294
0.1666666667
0.2869875223
0.4166666667
0.4166666667
1
0.6256684492
0.25
1
1
0.1379790553
1
0.2869875223
0.4166666667
0.376114082
1
1
0.4166666667
0.1666666667
1
0.4166666667
1
1
0.376114082
0.25
0.5588235294
0.4166666667
1
1
0.6256684492
1
0.1379790553
1
1
0.25
0.3666803232
1
0.5588235294

1
1
0.4166666667
1
0.25
0.4166666667
1
1
0.4166666667
0.25
0.4166666667
1
1
1
1
1
1
1
1
1
0.6256684492
0.6256684492
1
1
1
1
1
1
1
0.6256684492
0.4166666667
1
0.4166666667
0.6256684492
1
1
0.2869875223
1
0.25
1
1
1
1
1
1
1
0.1666666667
0.5588235294
0.1666666667
0.5588235294
1

1
0.4178238744
0.4166666667
1
0.5
0.4166666667
0.5588235294
0.25
0.4166666667
1
0.5588235294
1
0.4166666667
0.6256684492
0.25
0.4166666667
0.2869875223
0.2869875223
1
1
1
1
1
0.6256684492
1
1
1
1
1
1
0.2869875223
1
1
1
1
0.061942959
1
0.376114082
1
1
1
1
1
1
1
1
1
0.5
0.5
1
1

0.5588235294
0.5588235294
1
0.6256684492
0.5588235294
1
0.5
0.5
0.5588235294
0.2869875223
0.1379790553
1
1
1
1
1
1
1
1
0.5588235294
1
1
1
1
1
1
1
0.4166666667
1
1
1
1
1
1
1
0.6256684492
0.6256684492
0.5588235294
0.5588235294
0.5588235294
0.5588235294
0.5588235294
0.5588235294
0.3666803232
0.4166666667
0.4166666667
1
1
1
0.5588235294
0.6256684492

0.6295677362
0.6295677362
0.6295677362
0.6295677362
0.5588235294
0.6773654476
0.6773654476
0.4166666667
0.6256684492
0.5588235294
0.6773654476
1
1
0.5588235294
0.6773654476
0.6773654476
1
0.4166666667
1
1
1
0.5588235294
1
1
1
0.5588235294
1
0.1666666667
0.1666666667
1
1
1
1
1
1
1
0.5588235294
0.1666666667
0.1666666667
0.5588235294
0.4166666667
0.6256684492
1
0.25
0.0637254902
0.5588235294
0.5588235294
0.6773654476
0.6295677362
1
0.4166666667

0.6295677362
1
0.5588235294
0.376114082
1
1
0.4166666667
0.4166666667
0.4166666667
0.4166666667
1
1
0.376114082
1
1
1
1
0.1666666667
1
1
1
1
0.5588235294
0.1379790553
1
0.5
0.4166666667
0.2869875223
1
0.6256684492
0.6773654476
1
1
0.4166666667
0.4166666667
0.6256684492
1
1
1
1
1
1
0.4166666667
0.209972112
0.1379790553
1
1
0.4166666667
0.4166666667
1
1

1
0.5
1
1
1
1
0.4178238744
0.4178238744
0.4166666667
1
1
1
1
1
0.6256684492
0.1666666667
0.4166666667
1
1
0.4166666667
1
1
1
1
0.5
1
0.376114082
0.1379790553
0.1379790553
1
0.1379790553
1
0.1379790553
0.1379790553
0.5588235294
0.5588235294
0.5588235294
1
0.5588235294
1
0.4166666667
0.4166666667
1
1
1
1
1
1
1
1
1

1
1
1
1
0.5588235294
0.1666666667
1
1
1
0.4166666667
0.4166666667
0.5
1
0.1379790553
0.6256684492
1
0.6256684492
1
1
1
1
1
1
0.4166666667
0.6256684492
0.25
1
0.5
1
0.4166666667
0.25
0.5
1
1
1
1
1
0.1666666667
0.2869875223
0.2869875223
0.4166666667
0.4166666667
1
1
1
0.25
0.4166666667
0.4166666667
1
0.4178238744
1

0.5
1
0.5588235294
1
0.5
0.4240540589
0.25
1
0.4166666667
0.376114082
0.4166666667
0.4166666667
1
0.1379790553
1
0.4166666667
0.4166666667
0.1666666667
0.1666666667
0.4166666667
0.4166666667
1
1
1
1
1
0.25
0.25
1
1
0.4166666667
1
1
0.2869875223
1
0.2869875223
1
0.6295677362
0.25
1
1
1
0.4166666667
0.4166666667
0.6256684492
0.5588235294
1
1
0.1379790553
0.4166666667
0.1666666667

1
1
1
0.25
1
1
0.25
1
1
1
1
0.5
1
1
0.6773654476
0.5588235294
1
1
1
1
1
1
1
0.4166666667
0.4166666667
0.4166666667
0.4166666667
0.4166666667
0.4166666667
0.4166666667
0.4166666667
0.4166666667
0.4166666667
0.4166666667
0.4166666667
0.4166666667
0.4166666667
0.4166666667
0.4166666667
0.4166666667
0.4166666667
0.4166666667
0.4166666667
0.4166666667
0.4166666667
0.4166666667
0.4166666667
0.4166666667
0.4166666667
0.4166666667
0.4166666667

0.4166666667
0.4166666667
0.4166666667
0.4166666667
0.6256684492
0.6256684492
0.6256684492
0.6256684492
0.5588235294
1
0.6256684492
0.6256684492
0.6256684492
0.6256684492
0.6256684492
0.6256684492
1
1
0.6256684492
0.6256684492
0.25
0.25
0.25
0.5588235294
1
1
0.4166666667
0.6256684492
0.1247771836
0.4166666667
1
0.1247771836
1
1
1
1
1
1
1
1
1
1
1
1
1
1
1
1
0.2869875223
0.5
1

1
0.6256684492
0.6256684492
1
1
0.5588235294
1
0.6256684492
1
1
1
1
1
1
0.4166666667
1
1
0.5588235294
1
1
1
1
0.5588235294
0.5588235294
1
0.4166666667
0.0637254902
0.4166666667
0.4166666667
0.1666666667
1
1
0.3666803232
0.3666803232
0.5588235294
0.5588235294
0.6773654476
0.6773654476
1
0.4166666667
1
1
1
0.6256684492
0.1379790553
0.1666666667
0.6256684492
1
0.5588235294
0.5588235294
0.5588235294

0.5588235294
0.4166666667
0.4166666667
0.6256684492
1
0.376114082
0.4166666667
1
0.4166666667
0.6256684492
0.5588235294
0.6256684492
1
0.25
1
1
1
1
0.1666666667
1
1
0.6295677362
0.376114082
0.376114082
0.5588235294
0.5588235294
1
0.1379790553
1
0.1379790553
0.1379790553
0.1379790553
0.1379790553
0.1379790553
0.1379790553
0.1379790553
0.1379790553
0.1379790553
0.1379790553
0.1379790553
0.1379790553
0.1379790553
0.1379790553
0.1379790553
0.1379790553
0.1379790553
0.25
0.25
0.25
0.25
0.25

0.25
0.25
0.25
0.25
0.25
0.25
0.25
1
1
1
1
1
1
1
1
1
1
1
1
1
0.1666666667
0.1666666667
0.1666666667
0.1666666667
0.1666666667
0.1666666667
1
1
0.5588235294
0.5588235294
0.4166666667
1
0.5588235294
1
0.4166666667
0.4166666667
0.4166666667
1
1
1
0.25
1
1
0.6256684492
0.4166666667
0.25
1
0.5588235294
1
1
1

0.4166666667
0.2869875223
1
1
1
0.5
0.4166666667
0.4178238744
1
0.4166666667
0.1247771836
0.4166666667
0.4166666667
1
1
1
0.5588235294
1
1
0.209972112
0.4166666667
1
1
0.5588235294
0.25
0.25
0.6256684492
0.6256684492
0.4166666667
0.4166666667
1
1
1
1
1
0.6256684492
0.25
1
1
1
1
1
1
1
1
1
0.2869875223
0.2869875223
0.6256684492
0.5
1

1
1
1
1
1
0.061942959
0.5588235294
1
1
1
1
0.5
1
0.4166666667
1
0.25
0.25
1
1
1
1
1
0.5588235294
0.4166666667
1
1
1
0.6256684492
0.4166666667
0.4166666667
0.4166666667
0.5588235294
1
1
1
1
0.5588235294
1
0.4166666667
0.6256684492
0.6256684492
0.6256684492
0.6256684492
0.6256684492
0.6256684492
0.5588235294
1
1
0.376114082
0.4166666667
1

1
1
0.5588235294
0.1379790553
0.1666666667
0.5
0.5
0.5
1
1
1
1
1
0.4166666667
1
1
0.6256684492
0.209972112
0.25
0.25
0.4166666667
0.2869875223
1
1
1
0.1666666667
1
1
0.4166666667
0.4166666667
0.5588235294
1
0.4166666667
0.4166666667
0.5588235294
1
0.4166666667
0.4166666667
1
1
1
0.376114082
0.5588235294
0.5588235294
0.5588235294
0.4166666667
1
1
1
1
1

0.25
1
0.5588235294
0.5588235294
0.25
1
1
1
1
0.5588235294
0.4166666667
0.6773654476
0.6773654476
0.6773654476
0.6773654476
1
1
1
1
1
1
1
1
1
1
1
1
1
1
1
1
1
1
1
1
0.5588235294
0.5588235294
1
1
1
1
0.5588235294
1
1
1
1
1
0.5588235294
1
1
0.5

1
0.209972112
1
1
0.1666666667
1
1
1
1
0.6256684492
0.1666666667
1
0.5
1
1
1
1
1
1
0.25
0.6256684492
0.6256684492
0.2869875223
0.209972112
0.1379790553
0.1666666667
0.1379790553
0.5
0.1666666667
1
0.376114082
0.376114082
1
1
1
1
1
1
0.25
0.6737806193
0.6737806193
1
1
1
1
1
1
1
1
1
0.25

0.25
0.4166666667
1
1
0.6295677362
0.25
0.209972112
0.4166666667
0.5
1
0.3666803232
0.25
0.5588235294
0.5588235294
1
1
0.6256684492
0.6256684492
1
1
1
1
0.4166666667
1
1
1
0.25
1
1
1
1
0.5
1
0.5
0.1666666667
0.4166666667
0.4166666667
0.25
0.4166666667
0.4166666667
0.4166666667
0.4166666667
0.4166666667
1
0.4166666667
0.4166666667
0.4166666667
0.4166666667
0.25
0.376114082
0.4166666667

0.4166666667
1
1
1
0.5588235294
0.6256684492
1
0.25
0.4166666667
1
1
1
1
0.1666666667
1
0.5588235294
1
0.5588235294
1
0.6256684492
0.4166666667
0.4166666667
0.4166666667
0.4166666667
0.4166666667
1
1
1
1
1
0.2869875223
0.376114082
0.376114082
1
1
1
1
1
0.25
0.376114082
1
1
1
0.4166666667
1
1
1
0.4166666667
1
0.4166666667
1

1
1
1
1
1
0.5
1
0.6256684492
1
0.25
0.1666666667
0.4166666667
1
0.5588235294
0.4166666667
0.4166666667
0.209972112
0.209972112
0.4166666667
1
0.5
1
1
0.5588235294
0.5588235294
0.4166666667
0.4166666667
0.5
0.4166666667
1
0.4166666667
0.4166666667
0.4166666667
0.061942959
1
0.4166666667
0.6256684492
0.061942959
0.5588235294
0.1666666667
1
1
0.6256684492
0.4166666667
1
1
1
1
0.376114082
0.5
0.6256684492

0.6256684492
1
1
0.3666803232
0.4166666667
0.2869875223
1
0.1666666667
0.4166666667
1
1
0.5
0.376114082
0.5588235294
0.5588235294
0.25
1
0.5588235294
0.4166666667
1
0.5
0.5
1
1
1
1
1
0.5
1
1
0.25
0.25
1
1
0.4166666667
1
1
0.5588235294
0.4166666667
0.2869875223
1
1
1
1
1
1
1
0.6256684492
1
1
0.6256684492

0.6256684492
0.6256684492
1
0.4166666667
1
1
1
1
1
0.5588235294
1
1
0.5
1
1
1
1
0.4166666667
1
1
1
1
0.25
1
1
1
1
0.4166666667
0.1666666667
0.1666666667
1
0.1666666667
1
1
0.4166666667
1
0.1666666667
1
1
1
1
1
1
1
1
1
1
1
0.0637254902
0.0637254902
1

1
1
1
1
1
0.2869875223
0.2869875223
1
0.6256684492
1
1
0.5
0.5
0.4166666667
1
0.1666666667
0.1666666667
1
1
1
1
1
0.1666666667
1
1
0.4166666667
1
0.209972112
0.4166666667
0.5588235294
0.5588235294
0.25
1
1
1
0.4166666667
1
0.1379790553
1
1
1
1
1
1
1
0.25
0.25
0.4166666667
0.376114082
0.25
0.25

1
1
1
1
1
1
0.4166666667
0.6256684492
0.6256684492
0.1666666667
1
0.5588235294
0.1247771836
0.6256684492
0.4166666667
1
1
1
1
0.4166666667
1
1
0.5
1
1
1
0.061942959
0.5588235294
0.1379790553
0.1379790553
0.209972112
1
1
1
0.25
1
0.1379790553
1
0.5588235294
1
1
0.6773654476
1
1
0.209972112
0.2869875223
1
0.376114082
1
1
1

0.5
1
1
1
0.6256684492
1
0.25
0.25
0.4166666667
1
0.1666666667
1
0.376114082
1
1
0.6295677362
0.376114082
0.5
1
1
1
1
0.25
0.5588235294
0.5588235294
1
1
1
1
0.2869875223
0.4166666667
1
1
1
0.1379790553
0.4166666667
0.5588235294
1
1
1
0.5588235294
0.5588235294
1
0.6256684492
0.6256684492
0.6256684492
0.4166666667
0.4166666667
0.6295677362
1
0.5588235294

1
1
1
1
1
1
0.2869875223
0.5588235294
0.5588235294
0.4166666667
0.4166666667
0.6295677362
1
0.4166666667
1
0.25
0.25
1
1
1
1
0.4166666667
1
0.4166666667
0.25
0.25
0.1666666667
0.25
1
0.4166666667
1
0.5588235294
1
1
1
1
0.4166666667
0.5588235294
0.5588235294
1
0.25
0.25
0.4166666667
0.6773654476
0.6773654476
1
1
0.061942959
0.25
1
1

0.5588235294
1
1
1
1
0.4166666667
0.376114082
0.376114082
0.376114082
1
1
1
1
1
1
1
1
1
0.4178238744
0.5588235294
1
1
0.6773654476
1
0.4166666667
1
0.25
0.5588235294
1
1
1
1
0.4166666667
0.4166666667
0.4166666667
1
1
0.1666666667
0.4166666667
0.6256684492
1
1
0.25
0.5588235294
1
0.5588235294
1
0.5588235294
1
0.5588235294
0.5588235294

0.5588235294
0.5588235294
0.4166666667
1
1
1
0.6256684492
1
0.5588235294
0.25
0.4166666667
1
1
1
0.376114082
0.5
1
1
1
1
1
1
1
0.5588235294
0.1379790553
0.6256684492
0.25
0.1379790553
1
0.4166666667
1
1
0.5
1
1
1
0.4166666667
1
1
0.1379790553
0.6256684492
0.1666666667
1
0.4166666667
0.5588235294
1
0.376114082
0.4166666667
1
1
1

1
1
0.5588235294
1
1
0.25
0.25
0.5588235294
1
0.6773654476
1
1
1
0.4166666667
0.5588235294
1
0.4166666667
1
1
0.5588235294
0.25
1
0.5588235294
0.25
1
0.4166666667
0.1666666667
0.376114082
0.1666666667
1
1
1
0.25
1
1
1
0.6256684492
0.6256684492
0.4166666667
1
0.5
1
1
1
0.4166666667
0.6256684492
0.209972112
0.4166666667
1
1
0.5

0.5
0.6256684492
1
0.2869875223
1
1
1
1
1
1
1
1
1
1
0.6256684492
0.6256684492
1
0.061942959
1
0.376114082
1
0.376114082
0.376114082
0.376114082
1
1
1
1
0.4166666667
1
1
1
1
1
1
1
1
1
0.2869875223
0.4166666667
0.4166666667
1
1
1
1
1
1
0.1666666667
0.1247771836
0.5
0.25

0.5
0.5
0.25
0.5
0.1666666667
0.5
0.1666666667
0.25
0.4166666667
1
0.4166666667
0.376114082
1
1
1
0.4166666667
1
0.4166666667
1
1
1
1
1
0.4166666667
1
0.376114082
0.376114082
0.376114082
0.2869875223
0.2869875223
0.4166666667
1
1
1
1
1
1
1
1
1
1
1
1
1
1
1
1
0.4166666667
1
0.6773654476
0.5588235294

0.5588235294
0.5588235294
0.5588235294
0.5588235294
0.5588235294
0.5588235294
0.5588235294
0.5588235294
0.5588235294
0.5588235294
1
0.5588235294
0.25
1
1
1
1
1
0.5588235294
1
1
0.1247771836
1
1
0.5588235294
1
1
0.6256684492
0.5588235294
1
0.25
0.1247771836
1
1
1
1
1
1
0.4166666667
0.5588235294
0.5588235294
1
0.5588235294
1
0.5
0.5
0.5
0.5
0.5
0.5
0.5588235294

1
1
0.5
1
0.5588235294
0.5588235294
1
1
0.5
1
1
0.5
0.5
0.5
0.5
0.5
0.5
0.5
0.5
1
0.5
0.5
0.5
0.5
0.5
0.5
0.5
0.5
0.5
0.5
1
0.5
0.5
1
1
1
1
1
1
1
1
1
1
1
1
1
1
1
1
1
1

1
1
1
1
1
1
1
0.5
0.5
0.5
0.5
0.5
0.5588235294
0.5588235294
0.5588235294
0.5588235294
0.5588235294
0.5588235294
0.5588235294
0.5588235294
0.5588235294
0.5588235294
0.5588235294
0.5588235294
1
0.5588235294
0.5588235294
0.5588235294
0.5588235294
1
1
1
1
1
0.5
0.5
1
1
1
1
1
1
1
1
0.5
0.5
0.5
0.5
1
1
1

1
1
1
1
1
1
1
1
1
1
1
0.5
0.5
0.5
0.5
0.5
0.5
0.5
0.5
0.5
0.5
0.5
0.5
0.5
0.5
0.5
1
1
1
1
1
1
1
1
1
1
1
1
0.5
0.5
0.5
0.5
0.5
0.5
0.5
0.5
1
1
1
0.4166666667
1

1
1
1
0.6256684492
1
1
0.4166666667
1
1
1
0.1666666667
0.5
0.5
1
1
1
0.5588235294
0.25
0.5588235294
1
1
1
0.5
1
0.4178238744
0.4178238744
1
0.5
0.5
0.25
1
0.25
0.25
0.25
1
0.376114082
1
1
1
0.4166666667
1
1
1
0.4166666667
0.4166666667
0.7210233593
1
0.4166666667
1
0.5588235294
0.2869875223

0.2869875223
0.4166666667
0.4166666667
1
0.5588235294
1
0.4166666667
0.5
1
1
1
1
0.061942959
0.4166666667
0.6773654476
0.6256684492
0.25
0.25
0.4166666667
0.4166666667
1
0.6256684492
0.1247771836
0.1247771836
0.1247771836
0.1247771836
0.1247771836
0.1247771836
0.1247771836
0.1247771836
0.1247771836
0.1247771836
0.1247771836
0.1247771836
0.1247771836
0.1247771836
0.1247771836
0.1247771836
0.1247771836
0.1247771836
0.5588235294
0.4166666667
1
1
1
1
0.6256684492
0.6256684492
0.6256684492
1
0.6256684492

0.6256684492
0.6256684492
0.25
1
0.4166666667
1
1
0.4166666667
0.6256684492
1
0.6256684492
1
1
1
1
1
0.5588235294
1
1
0.5588235294
1
1
0.4166666667
0.4166666667
0.6256684492
0.6256684492
0.6256684492
0.1247771836
0.1247771836
0.1247771836
1
1
1
1
1
1
1
1
1
1
1
0.5588235294
1
1
1
1
1
0.2869875223
0.1666666667
1
1

1
1
0.1666666667
1
1
1
1
1
0.25
0.25
1
1
0.5588235294
0.4166666667
0.25
1
1
0.5588235294
0.25
1
0.4166666667
1
1
1
1
0.5
1
0.3666803232
1
1
1
0.6256684492
0.25
0.25
0.4166666667
0.4166666667
1
1
0.25
0.376114082
0.5588235294
0.5588235294
1
1
1
1
1
1
1
1
1

1
1
1
1
1
1
1
1
1
0.4178238744
1
0.5588235294
0.4166666667
0.4166666667
0.4166666667
0.4166666667
0.4166666667
0.4166666667
0.5588235294
1
0.5
0.4166666667
1
1
0.1247771836
1
0.5
1
0.4178238744
1
0.2869875223
1
1
0.5588235294
0.5588235294
0.5588235294
0.5588235294
0.1666666667
0.5588235294
0.5588235294
0.2869875223
0.5588235294
0.5588235294
0.4166666667
0.5588235294
1
0.5588235294
0.5588235294
0.5588235294
0.0637254902
1

0.25
0.4166666667
1
1
1
1
0.0637254902
0.1379790553
0.376114082
0.6256684492
0.6773654476
0.209972112
0.061942959
0.376114082
0.1379790553
0.4178238744
0.4166666667
0.4166666667
0.6295677362
1
0.4166666667
0.4166666667
0.1247771836
0.061942959
0.5
1
1
0.5588235294
0.4166666667
0.5588235294
1
1
0.2869875223
0.5588235294
0.5588235294
0.5588235294
0.2869875223
0.2869875223
0.2869875223
0.5588235294
1
0.5588235294
1
0.4166666667
0.5588235294
0.1666666667
1
1
1
1
1

1
1
1
0.25
1
0.5
0.4166666667
1
1
1
1
0.5588235294
1
1
0.5588235294
0.4166666667
1
0.6773654476
1
1
0.6773654476
0.4166666667
1
1
1
1
1
1
1
1
0.5588235294
0.5588235294
1
1
0.1379790553
0.5
1
1
1
1
0.209972112
0.25
0.4166666667
0.4166666667
0.4166666667
1
0.25
1
1
1
0.5588235294

1
1
0.6256684492
1
1
0.3666803232
1
1
0.4166666667
0.4166666667
0.5588235294
0.4166666667
1
0.4166666667
0.6256684492
1
1
0.6256684492
0.6256684492
0.25
0.061942959
0.1666666667
1
1
1
1
1
0.5588235294
0.376114082
0.376114082
0.376114082
0.376114082
0.376114082
0.4178238744
1
1
1
0.5
1
0.5
0.5
1
1
0.5
1
1
1
1
1
1
1

1
0.6256684492
1
1
1
1
1
1
0.5588235294
0.0637254902
0.2869875223
0.2869875223
0.2869875223
1
1
1
1
1
1
1
1
0.209972112
0.6256684492
0.6256684492
0.6256684492
0.6256684492
1
0.5588235294
1
0.376114082
0.5
1
0.6256684492
0.25
0.25
1
0.25
1
1
0.25
0.061942959
1
0.6256684492
1
1
1
1
0.6256684492
0.6256684492
1
1

0.6256684492
0.6256684492
1
0.376114082
1
1
0.2869875223
0.1666666667
1
1
1
1
0.2869875223
1
1
1
1
1
1
0.5588235294
1
1
1
1
1
1
1
1
0.4166666667
0.5588235294
1
1
0.2869875223
1
0.5588235294
1
1
0.5588235294
1
0.4166666667
0.4166666667
1
0.2869875223
1
1
1
0.209972112
0.6773654476
1
0.25
1

1
0.5
1
1
0.5588235294
1
1
1
1
1
0.2869875223
1
1
1
1
1
1
1
0.6256684492
1
0.376114082
1
0.1666666667
0.4166666667
0.1666666667
0.1666666667
0.6256684492
1
1
1
1
0.25
0.25
1
0.25
0.25
1
0.4178238744
0.4166666667
0.4166666667
0.6256684492
1
1
1
1
0.4166666667
1
0.5588235294
0.4166666667
0.4166666667
0.4166666667

1
0.4166666667
1
1
0.4166666667
0.4166666667
0.6256684492
0.4166666667
0.4166666667
0.4166666667
0.4166666667
0.4166666667
0.4166666667
1
0.4166666667
0.4166666667
0.1666666667
0.1666666667
0.4166666667
0.4166666667
0.4166666667
0.4166666667
1
0.4166666667
0.4166666667
0.4166666667
0.4166666667
0.1666666667
1
0.25
1
1
0.25
1
1
0.25
1
0.5588235294
0.6256684492
0.4166666667
1
0.6256684492
1
1
1
1
0.4166666667
1
0.2869875223
0.03042881375
0.03042881375

0.1247771836
0.1247771836
0.1247771836
1
1
0.4166666667
0.2869875223
1
0.1666666667
0.5
1
1
1
1
0.4166666667
0.6256684492
0.4166666667
0.6256684492
0.4240540589
1
1
1
1
0.6256684492
0.6256684492
0.376114082
0.376114082
0.6256684492
1
1
1
0.4166666667
1
1
1
0.5
0.5588235294
0.5588235294
0.5
0.5
1
1
0.5
0.4166666667
1
0.4166666667
0.4166666667
1
1
1
1

0.4166666667
0.4166666667
0.4166666667
0.4166666667
0.4166666667
1
0.5588235294
1
0.5
0.4166666667
1
1
1
0.6773654476
1
0.6256684492
1
1
1
0.6295677362
0.6295677362
0.6295677362
0.4166666667
1
0.4166666667
0.6256684492
0.5588235294
0.6773654476
1
0.4166666667
1
1
0.6773654476
0.1379790553
0.6773654476
0.4166666667
0.1666666667
1
0.2869875223
1
0.1666666667
0.6256684492
0.6256684492
0.6256684492
0.4166666667
0.4166666667
0.4166666667
1
1
1
1

0.6256684492
0.1666666667
1
0.25
0.4166666667
0.4166666667
0.25
1
0.6256684492
0.25
0.6256684492
0.25
1
0.5588235294
1
1
1
1
1
1
0.25
1
0.4166666667
0.4166666667
0.6773654476
1
1
1
0.4166666667
0.1666666667
0.4166666667
0.4166666667
0.5
0.4166666667
0.6256684492
0.5588235294
0.5588235294
1
1
0.0637254902
0.1666666667
0.209972112
0.4166666667
0.4166666667
1
0.6773654476
0.5
0.5
1
1
0.5588235294

0.5588235294
0.5588235294
0.2869875223
0.376114082
1
1
0.6773654476
0.376114082
0.376114082
0.376114082
0.1247771836
0.2869875223
0.6295677362
0.6295677362
1
1
0.4681927731
0.6773654476
0.1379790553
0.209972112
0.1379790553
0.1379790553
0.6256684492
1
0.4166666667
1
0.5
1
0.4166666667
0.4166666667
0.25
0.25
1
1
1
1
1
1
1
0.4166666667
0.4166666667
0.4166666667
0.376114082
0.5588235294
1
1
1
1
0.4166666667
1
1

1
1
1
0.4166666667
1
0.4166666667
0.4166666667
1
1
0.2869875223
0.1666666667
1
0.25
0.25
1
1
0.6256684492
0.25
0.25
0.5588235294
0.6773654476
0.6773654476
1
1
1
1
0.2869875223
1
1
1
1
0.5
1
0.5
0.5588235294
0.5588235294
0.5588235294
1
1
1
1
0.061942959
1
1
1
1
1
1
1
1
1

1
1
1
1
1
1
1
1
1
1
1
1
1
0.1666666667
0.1379790553
0.6773654476
0.4166666667
0.4166666667
1
1
1
1
0.6773654476
0.6773654476
0.5588235294
1
1
1
1
0.4166666667
1
0.4166666667
1
0.3666803232
1
0.6773654476
0.4166666667
0.5
0.6256684492
1
0.5588235294
1
0.5588235294
0.0637254902
0.2869875223
0.2869875223
0.25
0.4166666667
0.5588235294
0.4166666667
1

1
0.25
1
0.376114082
0.0637254902
0.25
0.4166666667
1
1
1
0.2869875223
0.2869875223
0.2869875223
0.4166666667
1
0.25
1
1
0.5588235294
0.2869875223
0.25
0.6256684492
0.25
0.5588235294
0.5588235294
1
0.25
0.5588235294
0.25
0.5588235294
0.5588235294
0.5588235294
0.25
0.5588235294
0.5588235294
0.25
0.5588235294
0.25
0.25
0.5588235294
0.6256684492
0.6256684492
1
1
1
0.6256684492
0.4178238744
1
1
0.4166666667
0.5588235294

0.6295677362
0.25
1
0.6256684492
0.25
0.376114082
1
1
0.25
1
0.376114082
0.376114082
0.6773654476
0.376114082
0.376114082
0.25
0.376114082
0.6773654476
0.25
0.25
1
0.5
0.25
0.4178238744
0.6256684492
0.25
0.25
0.376114082
1
0.4178238744
1
1
1
1
0.6773654476
1
0.4166666667
0.5588235294
1
1
1
1
1
1
1
0.1666666667
1
0.5588235294
0.25
1
1

0.4166666667
0.5588235294
1
1
1
0.5588235294
0.5588235294
0.5
1
1
0.4166666667
1
0.4166666667
1
1
0.4166666667
0.4166666667
0.4166666667
0.4166666667
0.4166666667
1
0.4166666667
0.4166666667
0.4166666667
0.4166666667
0.4166666667
0.4166666667
0.4166666667
0.4166666667
0.4166666667
1
0.4166666667
0.4166666667
0.4166666667
0.4166666667
0.4166666667
0.4166666667
0.4166666667
0.4166666667
0.4166666667
0.4166666667
0.4166666667
0.4166666667
0.4166666667
0.4166666667
1
1
0.6256684492
1
1
1

1
1
1
1
1
0.4166666667
0.4166666667
1
0.4166666667
1
0.4166666667
0.4166666667
0.5
0.5
0.6256684492
0.4166666667
1
1
0.4166666667
0.4166666667
0.4166666667
0.4166666667
0.4166666667
0.4166666667
1
0.4166666667
0.4166666667
1
0.4166666667
0.4166666667
0.4166666667
0.5
0.4166666667
0.5
1
1
0.5
0.5
0.5
0.5
0.5
0.4166666667
0.4166666667
0.4166666667
1
0.5
0.4166666667
0.5
0.4166666667
0.1666666667
0.4166666667

1
0.5
0.5
1
0.5
1
1
0.4166666667
1
0.4166666667
0.4166666667
0.4166666667
0.4166666667
0.4166666667
0.4166666667
0.4166666667
0.4166666667
0.4166666667
0.5
0.4166666667
1
1
1
0.4166666667
0.4166666667
0.4166666667
1
1
0.1666666667
1
0.4166666667
0.4166666667
1
0.5
1
1
1
0.1666666667
0.5
1
1
1
1
1
1
0.6256684492
1
1
1
1
1

1
1
1
0.6256684492
0.6256684492
0.1666666667
0.25
0.6256684492
1
0.6256684492
1
1
1
1
1
0.6256684492
1
1
1
1
1
1
1
1
1
1
1
1
1
0.6256684492
1
1
1
1
1
1
1
1
1
1
1
1
1
1
1
0.376114082
0.6256684492
0.5588235294
0.4166666667
1
1

1
1
1
1
0.6256684492
1
0.6256684492
1
1
1
1
0.5
0.5588235294
1
1
0.5
1
0.6256684492
1
1
1
1
1
0.1666666667
1
0.5588235294
1
0.5588235294
1
0.4166666667
1
1
1
1
1
1
1
1
1
0.4166666667
1
0.1666666667
1
0.1666666667
1
1
1
0.1666666667
0.4166666667
0.209972112
1

1
1
1
1
1
1
1
0.5
1
1
1
0.4166666667
0.1666666667
0.5588235294
1
1
1
1
1
0.6773654476
1
1
1
0.5588235294
0.1666666667
1
1
1
1
0.4166666667
0.4166666667
0.4166666667
1
1
1
1
1
0.5
1
1
1
1
1
1
0.6295677362
1
0.4166666667
1
1
1
0.4240540589

1
1
1
1
1
0.5588235294
0.25
0.25
1
1
1
1
1
1
1
1
1
0.4166666667
1
1
1
1
0.4166666667
1
1
1
0.4166666667
1
1
0.4166666667
1
0.4166666667
1
1
1
1
0.5588235294
1
0.4166666667
0.5588235294
1
1
1
1
0.376114082
1
1
0.376114082
0.4166666667
0.4166666667
0.4166666667

0.376114082
0.4166666667
0.4166666667
0.4166666667
0.4166666667
1
1
0.4166666667
0.5
0.4166666667
1
0.3666803232
1
1
1
1
1
0.4166666667
1
1
1
1
1
1
0.4166666667
1
1
1
0.4166666667
0.4166666667
0.4166666667
0.5588235294
0.5588235294
1
1
1
1
0.4166666667
1
1
0.5588235294
0.376114082
1
0.4166666667
1
1
1
0.5
1
1
1

1
1
1
0.5588235294
1
0.5588235294
1
1
1
1
1
1
1
1
1
1
0.5588235294
1
1
1
1
1
0.4166666667
1
1
1
0.4166666667
0.4166666667
1
0.4166666667
0.4166666667
0.4166666667
0.4166666667
0.4166666667
1
0.5588235294
1
0.5588235294
0.5588235294
1
1
1
1
1
0.4166666667
1
0.4166666667
0.4166666667
0.4166666667
0.5588235294
0.5588235294

1
0.376114082
1
1
0.4166666667
0.4166666667
0.3666803232
1
0.4166666667
1
1
0.4166666667
1
0.6256684492
1
0.4166666667
0.4166666667
0.4166666667
0.4166666667
0.4166666667
0.4166666667
0.4166666667
0.4166666667
1
1
1
1
0.5588235294
0.4166666667
0.5588235294
0.6256684492
0.0637254902
1
1
0.5
0.5
1
1
0.4166666667
0.5588235294
0.5588235294
0.5588235294
1
0.4166666667
0.4166666667
0.4166666667
0.5
1
0.1379790553
0.1666666667
1

0.5588235294
1
0.4166666667
1
0.5
1
0.4166666667
0.4166666667
0.4166666667
0.4166666667
1
0.4166666667
0.5588235294
0.376114082
1
0.6256684492
0.5
0.6256684492
0.25
0.376114082
0.25
0.2869875223
1
0.209972112
0.0637254902
0.25
1
1
0.25
1
1
0.25
1
0.0637254902
0.6256684492
0.209972112
0.03042881375
0.2869875223
1
0.6256684492
1
0.6256684492
0.25
1
0.6256684492
1
1
1
1
1
1

1
1
1
1
1
0.1247771836
1
1
0.6773654476
1
1
1
1
0.6773654476
1
0.6256684492
0.5588235294
0.6773654476
0.6773654476
0.6773654476
0.6773654476
0.4166666667
1
1
0.4178238744
0.1666666667
1
1
1
1
0.5
1
1
0.1666666667
0.6256684492
1
1
1
1
0.1379790553
0.1379790553
0.1666666667
0.6256684492
0.6256684492
0.4166666667
1
1
1
1
1
1

0.5588235294
1
0.4166666667
0.4166666667
0.4166666667
0.4166666667
0.25
1
1
1
1
0.25
1
1
0.376114082
0.2869875223
0.5588235294
1
0.4166666667
1
1
0.4166666667
1
1
0.1666666667
0.3666803232
0.3666803232
0.3666803232
0.5588235294
1
1
0.4166666667
1
1
1
0.376114082
0.2869875223
1
1
0.2869875223
1
1
1
0.6256684492
1
0.6256684492
0.6256684492
0.2869875223
0.5588235294
0.25
1

1
0.6773654476
1
0.5588235294
1
1
1
1
1
1
0.4166666667
1
1
0.2869875223
0.1666666667
1
0.6295677362
1
1
0.1247771836
1
1
1
0.209972112
1
1
1
1
0.376114082
0.5588235294
0.5
0.6773654476
0.25
1
1
1
0.4166666667
0.4166666667
1
1
0.4178238744
0.2869875223
0.1666666667
0.25
1
0.6773654476
1
1
0.1666666667
0.1666666667
1

1
1
0.1247771836
0.2869875223
0.1379790553
0.6773654476
0.4166666667
1
0.6256684492
0.6295677362
1
0.4166666667
0.209972112
0.6295677362
1
1
1
1
0.5
1
0.2869875223
0.2869875223
0.209972112
1
1
0.1379790553
1
0.376114082
0.4166666667
1
0.5
0.6773654476
1
1
0.4166666667
1
0.6256684492
0.4166666667
1
0.4166666667
1
0.5
1
0.25
1
0.1379790553
1
0.4166666667
0.6256684492
1
0.6773654476

1
1
0.6295677362
1
0.2869875223
1
0.5
0.1666666667
0.4166666667
0.4166666667
0.1666666667
1
1
1
0.5
1
1
0.6256684492
1
1
0.1247771836
0.4178238744
1
1
1
0.2869875223
1
1
0.4166666667
1
0.25
1
0.4166666667
1
1
0.6773654476
0.209972112
0.5
1
1
1
1
0.4166666667
1
1
0.1666666667
0.1666666667
0.4166666667
0.5
0.25
0.6256684492

1
0.5
1
0.25
0.2869875223
0.6256684492
1
0.25
1
0.1666666667
1
0.6256684492
0.1379790553
1
0.1666666667
0.4166666667
1
0.4166666667
0.6295677362
0.2869875223
1
1
0.1666666667
0.4166666667
1
0.6256684492
0.6773654476
0.1666666667
0.2869875223
1
1
0.3666803232
0.1666666667
0.4166666667
0.5
0.1379790553
0.1666666667
0.4166666667
0.25
0.6256684492
1
1
0.0637254902
1
0.4166666667
0.5588235294
0.1379790553
0.5588235294
0.25
0.5588235294
0.1666666667

0.5
1
0.5588235294
1
1
0.4166666667
0.4166666667
1
1
0.5588235294
1
0.6256684492
0.4166666667
1
1
1
1
1
1
0.6256684492
0.4166666667
1
1
1
1
1
1
1
1
1
0.2869875223
1
1
1
1
1
1
1
0.5588235294
0.4166666667
0.5
1
1
1
0.5588235294
0.6256684492
0.1666666667
0.209972112
0.7210233593
0.0637254902
1

1
1
0.6256684492
0.5
0.4166666667
1
0.6256684492
1
1
1
0.1666666667
0.5588235294
0.376114082
1
1
1
1
0.4166666667
1
0.4166666667
1
0.376114082
0.6773654476
1
1
1
1
0.2869875223
0.5588235294
1
0.2869875223
1
1
0.2869875223
1
1
1
1
0.1666666667
0.1666666667
0.1666666667
1
0.4166666667
1
0.6737806193
1
1
1
1
1
1

1
1
0.4166666667
0.6256684492
1
0.5588235294
1
0.6256684492
0.25
0.5
0.5
0.5588235294
0.5
1
1
1
0.5588235294
1
1
0.5588235294
1
0.25
1
1
1
0.5
1
0.2869875223
0.2869875223
1
0.4166666667
0.4166666667
1
0.6773654476
0.5588235294
0.2869875223
1
0.4166666667
0.5
0.5588235294
0.5588235294
1
1
1
0.6256684492
1
1
0.0637254902
0.4166666667
0.6256684492
0.25

1
1
0.5588235294
0.4166666667
0.6295677362
0.4166666667
0.5
1
1
0.0637254902
0.5
1
1
1
0.4166666667
0.6256684492
1
0.4166666667
0.25
1
0.4166666667
0.1379790553
0.4166666667
1
0.4166666667
0.5
0.1379790553
1
0.376114082
0.6256684492
1
0.4166666667
0.1666666667
1
1
1
0.5588235294
0.1379790553
1
1
0.1247771836
0.4166666667
0.1379790553
1
0.1379790553
1
1
0.5
0.1379790553
0.2869875223
1

1
0.1666666667
1
0.1379790553
1
1
1
0.5
1
1
0.4166666667
0.4166666667
0.4166666667
0.4166666667
0.1666666667
0.6256684492
0.5588235294
0.4178238744
0.4166666667
1
0.4166666667
0.2869875223
0.4166666667
0.4166666667
1
0.4166666667
1
0.1666666667
1
1
0.1247771836
1
0.4166666667
1
0.5588235294
0.376114082
1
1
0.4166666667
1
1
0.6256684492
0.2869875223
0.4166666667
1
0.5588235294
1
0.5588235294
1
1
1

0.5
0.5588235294
0.1666666667
1
0.5
0.2869875223
0.5588235294
1
0.1379790553
1
1
0.6940287188
1
0.4166666667
0.25
1
1
1
1
0.2869875223
1
0.4166666667
1
1
0.5588235294
1
0.5588235294
1
1
1
0.4166666667
0.25
0.6940287188
0.4166666667
0.5588235294
0.6773654476
0.25
1
1
1
1
1
1
1
1
1
1
1
1
1
1

1
1
1
1
1
0.25
0.4166666667
1
0.25
1
1
1
0.1379790553
0.2869875223
1
1
0.4166666667
0.25
1
1
0.25
1
1
1
1
1
0.5588235294
1
1
0.376114082
1
1
1
0.5
0.5588235294
1
1
1
1
1
0.6256684492
1
1
1
0.4166666667
0.376114082
0.4166666667
0.2869875223
1
1
0.5

1
0.4166666667
0.4166666667
0.25
1
0.4166666667
1
0.1247771836
1
1
0.4166666667
1
1
1
1
1
1
1
1
1
0.5
1
1
1
1
0.376114082
1
1
0.4166666667
0.6295677362
0.25
1
0.4166666667
0.6256684492
0.5588235294
1
0.6295677362
1
0.209972112
0.5588235294
0.5588235294
0.4166666667
0.5588235294
1
1
0.4166666667
1
1
0.5588235294
1
1

1
0.209972112
1
0.4166666667
1
1
0.5588235294
1
1
0.4166666667
1
0.376114082
0.6773654476
0.4166666667
0.4166666667
1
0.5588235294
0.4166666667
1
0.4166666667
1
0.4166666667
1
0.25
0.4166666667
1
0.6940287188
1
1
0.4166666667
0.6295677362
0.5588235294
0.4166666667
0.5588235294
1
0.4166666667
1
0.25
1
1
1
1
1
1
1
1
0.4166666667
1
0.6256684492
0.1666666667
0.4166666667

0.6295677362
1
1
0.6256684492
1
1
0.1379790553
0.5588235294
0.4166666667
0.5588235294
0.209972112
1
1
1
1
1
1
0.4166666667
1
0.4166666667
1
1
1
1
1
1
0.5
1
1
0.376114082
1
1
0.4240540589
0.5588235294
1
1
1
0.25
1
0.25
0.2869875223
1
0.5588235294
0.25
0.4166666667
0.4178238744
0.25
0.6295677362
0.25
1
0.5

0.4166666667
0.1247771836
1
1
0.5588235294
0.1666666667
0.5588235294
1
1
0.4166666667
0.5
1
1
0.1666666667
0.4166666667
0.5588235294
1
0.376114082
0.6256684492
0.1666666667
1
1
0.5588235294
0.4166666667
1
1
0.2869875223
0.25
1
0.5588235294
0.4166666667
0.209972112
0.6256684492
0.25
0.25
0.6256684492
0.6773654476
0.5588235294
0.4166666667
0.6773654476
1
1
1
1
0.6737806193
1
0.4166666667
1
1
0.4166666667
1

0.209972112
1
0.6256684492
0.5588235294
0.2869875223
0.6295677362
1
1
0.1379790553
0.1379790553
0.4166666667
1
0.1666666667
1
1
0.6256684492
0.6256684492
0.6256684492
1
1
0.5
1
1
0.2869875223
1
1
1
1
0.4178238744
1
0.25
0.6737806193
0.2869875223
0.5588235294
0.5588235294
1
0.5588235294
1
1
1
1
1
1
1
0.1666666667
0.4166666667
1
0.4166666667
1
1
1

1
1
1
1
1
1
0.1379790553
0.209972112
0.209972112
0.209972112
1
0.1379790553
0.6256684492
1
0.6256684492
1
0.5
0.1666666667
0.376114082
0.6256684492
0.1666666667
0.4166666667
0.1666666667
1
1
1
0.5
0.25
0.25
0.5
0.25
1
1
1
1
1
1
1
1
1
1
1
1
1
1
1
1
0.6256684492
0.6256684492
1
0.376114082

0.6773654476
0.6773654476
0.6295677362
1
0.4178238744
0.2869875223
0.5588235294
0.5588235294
0.5588235294
0.1666666667
0.5588235294
0.1666666667
0.0637254902
0.1666666667
0.1666666667
0.25
0.4166666667
0.4166666667
0.1666666667
0.4166666667
0.4166666667
0.4166666667
0.4166666667
0.1666666667
0.1666666667
0.4166666667
0.1666666667
0.1666666667
1
1
0.6256684492
0.6256684492
0.6256684492
1
1
1
1
0.4166666667
0.4166666667
1
0.1666666667
1
1
0.4166666667
0.4166666667
1
0.2869875223
0.6295677362
0.1379790553
0.2869875223
0.6773654476

0.1379790553
0.5588235294
1
1
1
1
1
1
1
1
1
1
0.1379790553
0.1379790553
0.2869875223
1
1
0.4166666667
1
1
1
0.5
0.2869875223
1
1
1
0.5588235294
0.5
1
0.5
1
1
1
1
0.4166666667
0.4166666667
1
0.4166666667
0.4166666667
0.4166666667
0.4166666667
0.4166666667
0.4166666667
1
1
1
0.6256684492
0.6256684492
1
1
1

0.6256684492
1
0.6256684492
0.1666666667
0.1666666667
0.5588235294
1
1
1
1
0.4166666667
1
1
1
1
1
0.6940287188
0.5588235294
0.6295677362
0.6940287188
0.5588235294
0.376114082
1
1
0.4178238744
1
1
0.4166666667
0.209972112
1
0.2869875223
0.6256684492
0.4166666667
0.6256684492
0.4166666667
1
1
0.5588235294
0.6256684492
0.061942959
0.5588235294
1
0.6256684492
1
1
1
0.25
0.25
0.5588235294
0.4166666667
0.4166666667

1
0.4166666667
1
0.6256684492
1
0.6773654476
1
1
1
0.4166666667
1
1
1
1
1
0.5588235294
1
0.1379790553
1
0.5588235294
0.5
0.5
1
1
0.25
0.3666803232
0.5
1
1
1
1
1
1
1
0.25
0.25
0.25
0.6773654476
0.5588235294
0.4166666667
0.4166666667
0.4166666667
1
1
1
1
1
1
1
1
0.4166666667

1
0.6256684492
0.2869875223
0.5588235294
0.4166666667
1
0.6256684492
0.3666803232
0.2869875223
1
1
0.6256684492
1
1
0.5
1
1
1
0.1666666667
1
1
1
1
1
0.2869875223
1
1
0.25
0.25
0.5588235294
0.376114082
0.376114082
1
1
0.2869875223
0.2869875223
0.4166666667
0.4166666667
0.4166666667
0.4166666667
0.4166666667
0.4166666667
0.4166666667
1
1
0.4166666667
1
0.6256684492
0.5588235294
1
1

0.6773654476
0.6940287188
1
1
0.1247771836
0.4166666667
0.25
1
1
0.6256684492
1
1
0.5588235294
0.2869875223
1
1
1
1
0.4166666667
0.4166666667
1
1
0.376114082
0.376114082
1
0.5588235294
0.4166666667
1
1
0.5588235294
0.376114082
0.5588235294
0.4166666667
0.6256684492
0.5588235294
1
0.4166666667
0.25
0.6256684492
1
0.5588235294
1
1
0.4166666667
0.3666803232
1
0.6256684492
1
1
1
0.5

1
1
0.5588235294
1
0.5588235294
0.4166666667
0.376114082
1
1
0.2869875223
0.1379790553
0.6773654476
1
0.6773654476
0.5588235294
1
0.209972112
1
1
1
0.5588235294
0.4166666667
1
1
1
1
0.5588235294
0.25
0.4166666667
0.209972112
0.4166666667
1
0.4166666667
0.6295677362
1
1
0.1379790553
0.5588235294
1
0.6256684492
0.1666666667
0.4166666667
0.2869875223
0.4166666667
0.4166666667
1
0.2869875223
0.6773654476
1
1
0.376114082

0.5588235294
1
1
1
1
0.5
1
0.6737806193
0.25
1
0.5
0.1666666667
0.5588235294
0.376114082
1
0.4166666667
1
1
0.4166666667
0.376114082
1
0.376114082
1
1
1
0.25
1
1
1
1
1
1
1
1
0.4166666667
1
1
1
0.4240540589
0.5588235294
0.1379790553
1
0.5588235294
0.4166666667
0.2869875223
1
1
0.25
1
1
0.6773654476

0.5
0.5588235294
0.6295677362
1
1
0.25
1
1
0.4166666667
1
1
0.5
0.5588235294
0.4166666667
0.3666803232
0.4166666667
1
1
0.1666666667
0.209972112
0.5588235294
1
1
1
0.6256684492
1
0.4166666667
0.4166666667
1
0.6773654476
0.4166666667
0.209972112
1
1
0.2869875223
0.6256684492
0.25
0.4166666667
1
1
0.4166666667
1
1
1
1
1
0.1379790553
0.4166666667
0.1379790553
0.1666666667
1

1
0.5588235294
1
0.5588235294
1
0.25
1
0.4166666667
1
0.6256684492
1
1
0.4166666667
1
1
1
1
0.5588235294
0.4166666667
0.25
1
1
1
0.5588235294
0.1666666667
1
0.5588235294
0.25
0.25
0.4166666667
0.5
0.6773654476
1
0.5
1
1
1
0.4166666667
0.4166666667
1
0.4166666667
0.6256684492
1
1
1
0.4166666667
1
1
1
1
1

1
1
0.5
0.25
0.25
0.4166666667
1
1
0.6256684492
0.6256684492
0.4166666667
1
1
0.4166666667
1
0.4166666667
1
0.4166666667
0.4166666667
1
1
1
0.4166666667
1
1
1
0.2869875223
0.4166666667
1
1
1
1
0.5
0.5588235294
0.2869875223
1
0.6256684492
0.061942959
1
0.6256684492
0.5588235294
0.5588235294
0.5
0.376114082
0.376114082
0.6256684492
0.25
0.5
0.061942959
1
0.25

1
1
0.5
1
0.5588235294
1
1
0.5
1
1
1
1
0.5588235294
1
0.25
0.4166666667
1
1
0.25
0.25
1
1
1
0.5
0.5588235294
1
0.3666803232
1
1
1
0.6256684492
0.1379790553
1
1
1
0.5588235294
0.6256684492
1
1
1
1
1
0.6256684492
1
0.5588235294
0.4166666667
1
1
1
0.376114082
1

1
1
0.061942959
1
0.376114082
0.4166666667
1
0.25
0.4166666667
1
1
0.2869875223
1
1
1
1
0.1666666667
1
1
1
0.5
1
0.4178238744
1
1
1
0.2869875223
0.4166666667
1
1
1
0.5
1
1
1
0.6256684492
1
0.5588235294
1
1
1
1
1
0.5588235294
1
0.5588235294
0.2869875223
0.1379790553
1
0.5
1

1
1
1
1
1
0.5588235294
1
1
0.6256684492
0.2869875223
0.2869875223
0.2869875223
1
1
1
1
1
1
1
0.2869875223
0.2869875223
0.2869875223
0.4166666667
0.4166666667
0.25
1
0.4166666667
0.4166666667
1
1
0.2869875223
0.2869875223
0.4166666667
0.2869875223
1
1
1
0.4166666667
0.4166666667
0.25
1
0.5588235294
0.6256684492
0.6256684492
0.6256684492
0.6256684492
1
1
1
1
0.376114082

1
1
1
0.6256684492
1
0.209972112
0.209972112
1
0.5
0.1666666667
1
1
1
0.25
0.376114082
0.4166666667
0.5588235294
1
0.4166666667
1
0.4166666667
0.1379790553
0.1379790553
1
0.5588235294
1
1
1
0.2869875223
0.4166666667
1
0.5588235294
1
0.4166666667
1
0.25
0.5
0.2869875223
1
1
0.6773654476
0.5588235294
1
1
0.2869875223
0.5
1
0.6256684492
1
1
1

0.5588235294
0.5588235294
0.5588235294
1
1
1
0.5588235294
0.4166666667
1
0.5588235294
0.1666666667
0.6295677362
0.6295677362
1
0.4166666667
1
0.2869875223
0.5
0.4166666667
0.4166666667
0.6256684492
1
0.4166666667
1
1
0.6256684492
0.6256684492
0.5588235294
1
1
1
1
0.4166666667
0.6295677362
0.2869875223
1
1
1
1
0.25
1
0.4166666667
1
1
0.4166666667
0.25
1
0.4166666667
1
1
1

1
1
1
0.6256684492
1
1
1
0.6256684492
0.25
0.25
1
0.4166666667
0.1666666667
0.1666666667
0.25
1
0.5588235294
1
0.5588235294
0.209972112
0.5
1
0.4166666667
0.4166666667
0.4166666667
1
0.376114082
1
0.4166666667
1
0.4166666667
0.1666666667
0.1666666667
1
1
0.2869875223
1
1
1
1
1
1
1
0.25
0.1666666667
0.209972112
1
1
0.25
0.5
0.1666666667

0.1666666667
1
0.5
0.25
0.25
0.25
0.6256684492
0.6256684492
1
1
1
0.6256684492
1
0.4166666667
1
1
0.4166666667
0.6256684492
0.6256684492
0.5588235294
0.4166666667
1
0.6256684492
1
1
0.4166666667
0.25
1
1
1
0.5588235294
1
1
1
1
1
1
0.25
0.4166666667
0.4166666667
1
0.1666666667
0.1666666667
1
0.4166666667
0.6773654476
1
1
0.376114082
1
0.1666666667

1
1
0.5
1
0.6256684492
0.6256684492
0.6256684492
0.6256684492
0.6256684492
0.6256684492
1
0.4166666667
0.25
1
1
1
1
1
0.4166666667
0.5588235294
1
1
1
1
0.5
1
0.6295677362
0.5
1
0.0637254902
1
0.376114082
1
1
0.4166666667
0.6256684492
0.376114082
0.4166666667
0.1666666667
1
0.376114082
1
1
1
0.5588235294
0.5588235294
0.6295677362
0.1666666667
1
1
0.376114082

0.376114082
0.061942959
0.6773654476
0.6256684492
1
1
1
1
1
0.1379790553
1
1
1
0.376114082
0.4166666667
0.4166666667
0.5588235294
1
1
1
0.4166666667
0.4166666667
1
0.4166666667
1
1
0.061942959
1
0.5588235294
1
1
1
1
0.6256684492
0.5588235294
0.5
0.1666666667
0.1666666667
1
0.5
0.4166666667
1
0.4166666667
1
0.5588235294
1
1
1
0.5
1
1

0.5
1
1
0.6773654476
0.4166666667
1
1
0.5588235294
1
1
1
1
1
1
0.5588235294
1
1
0.4166666667
1
1
0.1666666667
1
1
0.209972112
0.5
0.4166666667
0.4166666667
0.4166666667
1
1
1
1
0.6256684492
0.6256684492
0.25
1
0.1379790553
1
0.5
1
0.4166666667
0.4166666667
1
1
1
1
1
0.209972112
0.4166666667
0.4166666667
1

0.1379790553
0.6256684492
0.6256684492
0.4166666667
0.4166666667
0.1666666667
0.6256684492
0.4166666667
1
1
1
1
0.1379790553
1
1
1
0.5
0.4166666667
1
1
1
0.4166666667
1
1
1
0.5
1
0.5588235294
0.4166666667
1
0.5588235294
0.4166666667
1
1
0.376114082
0.5588235294
0.1666666667
0.6256684492
1
1
1
0.6256684492
1
1
1
1
1
0.2869875223
0.25
0.25
1

1
0.4166666667
1
0.4166666667
0.4166666667
1
1
1
1
1
0.1666666667
0.1666666667
0.4166666667
1
1
0.4166666667
0.25
0.4166666667
1
1
1
0.4166666667
1
1
1
1
1
1
0.1379790553
1
0.03042881375
0.5588235294
1
0.5588235294
0.4166666667
0.25
0.4178238744
0.4166666667
1
0.6256684492
1
1
0.5588235294
0.1666666667
1
1
1
0.1666666667
0.2869875223
1
1

0.5588235294
0.5588235294
0.6256684492
0.376114082
1
1
0.1666666667
0.5
1
1
1
0.5588235294
0.5588235294
1
1
1
0.5588235294
1
0.6256684492
1
1
0.376114082
1
1
0.5588235294
1
0.1666666667
1
0.2869875223
0.1666666667
0.6256684492
0.4178238744
0.4178238744
0.4178238744
0.5588235294
1
1
0.4166666667
0.376114082
0.4166666667
0.376114082
1
1
0.25
0.4166666667
1
0.5588235294
1
0.25
0.2869875223
1

0.1379790553
0.25
1
1
1
1
1
0.5
0.6256684492
0.209972112
0.5
1
0.6256684492
1
0.25
1
1
1
1
0.6773654476
0.6773654476
0.4178238744
0.4166666667
0.6295677362
0.6295677362
0.6295677362
0.6295677362
0.0637254902
0.0637254902
0.1666666667
1
0.1666666667
1
0.376114082
1
0.1666666667
0.4166666667
0.25
0.5588235294
1
0.4166666667
0.5588235294
0.6256684492
1
0.6256684492
1
1
1
1
1
0.5588235294

0.25
1
1
1
1
1
1
1
1
0.1666666667
1
1
1
0.5588235294
0.4166666667
1
0.25
1
1
1
0.5588235294
1
0.376114082
1
0.6256684492
0.6256684492
1
0.209972112
0.4166666667
0.2357640397
0.2357640397
0.2357640397
0.4166666667
0.4166666667
0.6295677362
0.1379790553
0.2869875223
1
0.2357640397
0.4166666667
0.4166666667
1
0.6256684492
1
0.1666666667
1
1
1
0.5588235294
0.5588235294
0.4178238744

1
0.376114082
0.1247771836
1
1
1
1
0.6256684492
0.5588235294
0.5
1
1
1
0.6256684492
0.4166666667
1
1
0.1379790553
1
1
1
0.1379790553
0.25
0.6256684492
0.6256684492
0.6256684492
1
0.5588235294
0.5588235294
0.6256684492
0.6256684492
1
1
1
1
1
1
1
1
1
1
1
1
1
1
1
0.5
1
1
0.376114082
0.5

1
0.4178238744
1
1
1
1
0.6256684492
0.4166666667
0.25
1
0.1379790553
1
0.6256684492
0.6256684492
0.4166666667
0.5588235294
1
1
0.6256684492
1
0.1666666667
0.1666666667
0.5588235294
0.5588235294
0.1247771836
0.1247771836
0.4166666667
0.25
1
1
1
0.4166666667
0.25
1
0.4166666667
0.4166666667
0.25
1
1
0.6256684492
0.5
1
1
1
0.1666666667
0.6256684492
0.6256684492
1
1
1
1

0.4166666667
1
1
1
0.1666666667
1
1
0.1666666667
0.4166666667
1
1
1
1
0.4166666667
1
1
1
0.6256684492
0.4166666667
1
1
1
1
1
0.4178238744
0.4166666667
0.4178238744
1
1
0.25
0.25
0.25
0.25
1
0.4166666667
0.4166666667
0.4166666667
0.4166666667
0.4166666667
1
0.5
1
1
1
0.1379790553
1
0.1379790553
1
1
0.5588235294
1

1
1
1
0.4240540589
0.4240540589
0.4240540589
0.6256684492
0.6256684492
0.6256684492
0.25
0.25
0.25
0.25
0.4166666667
0.4166666667
0.1666666667
0.1666666667
0.0637254902
0.4166666667
0.4166666667
0.4166666667
0.4166666667
0.4166666667
1
1
1
1
1
0.4166666667
0.4166666667
0.4166666667
1
1
0.1379790553
0.6773654476
0.5588235294
1
1
1
1
1
1
0.4166666667
1
1
1
0.6256684492
1
1
0.25
0.25

0.25
0.4166666667
0.4166666667
0.4166666667
1
1
0.1379790553
0.209972112
0.5588235294
0.5
0.5
0.5
0.5
0.5
0.5
0.5
1
0.209972112
1
1
0.1666666667
0.4166666667
0.25
1
1
1
1
1
1
0.376114082
1
0.6295677362
0.4178238744
0.4178238744
0.5588235294
1
1
0.5588235294
0.5588235294
1
1
0.5588235294
0.2869875223
1
1
0.6256684492
0.5588235294
1
1
0.4166666667
0.6256684492

1
1
1
1
0.2869875223
0.1666666667
0.5588235294
1
0.6256684492
0.4166666667
0.4166666667
1
0.5
1
0.0637254902
1
1
0.6295677362
1
0.5
1
0.6773654476
0.6773654476
1
0.1666666667
0.25
0.6256684492
0.5588235294
0.5588235294
0.6773654476
1
1
0.5588235294
1
1
1
1
1
0.1666666667
0.25
1
1
1
1
1
0.061942959
0.061942959
0.5588235294
0.2869875223
0.4166666667
0.5

0.5
0.5
0.5
1
1
0.5588235294
0.209972112
1
0.4166666667
1
1
0.4166666667
0.4166666667
1
0.2869875223
0.4166666667
1
0.5588235294
0.4166666667
0.4166666667
1
1
1
1
1
1
1
1
1
1
1
1
1
0.4166666667
1
0.03042881375
1
0.4166666667
0.5588235294
1
0.4166666667
0.1666666667
1
1
0.5588235294
0.1666666667
1
1
1
0.4166666667
1

1
1
1
0.25
0.5588235294
0.6256684492
1
1
1
1
1
1
1
1
0.5588235294
0.5
1
1
1
1
1
0.6256684492
0.6256684492
1
0.2869875223
0.1247771836
0.4166666667
0.4166666667
1
0.5588235294
0.5588235294
0.5
0.5
0.5
1
1
0.4166666667
0.5
0.4166666667
0.4166666667
0.5588235294
0.5588235294
1
1
0.376114082
1
0.25
0.4166666667
0.4166666667
0.1666666667
0.4166666667

0.4166666667
0.4166666667
0.4166666667
0.4166666667
0.4166666667
1
0.5
1
1
0.4166666667
0.1666666667
0.4166666667
1
1
1
0.6773654476
0.6773654476
1
1
1
0.5588235294
0.5588235294
0.4166666667
0.1666666667
1
1
1
0.6256684492
1
1
0.6256684492
0.3666803232
0.03042881375
1
1
0.376114082
0.5588235294
1
1
1
1
0.4166666667
0.25
0.25
1
0.5
1
1
1
1
1

1
0.4178238744
0.4178238744
1
0.5588235294
0.1666666667
1
1
1
1
0.25
0.5588235294
0.3666803232
0.25
0.25
0.4166666667
1
1
1
0.4166666667
0.4166666667
0.4166666667
0.4166666667
0.4166666667
0.4166666667
0.4166666667
0.4166666667
0.4166666667
0.4166666667
0.4166666667
0.4166666667
0.4166666667
0.4166666667
0.5
0.5588235294
0.5588235294
1
1
1
0.376114082
0.3666803232
1
0.4166666667
0.4166666667
0.4166666667
0.4166666667
1
0.4166666667
0.4166666667
0.1666666667
0.4166666667

0.4166666667
1
1
1
1
1
0.6295677362
0.6773654476
1
0.2869875223
0.4166666667
1
1
1
1
0.209972112
0.209972112
1
0.6256684492
1
1
0.6256684492
0.6256684492
0.3666803232
0.4166666667
1
0.376114082
1
0.1666666667
1
1
1
1
1
0.5
1
0.25
0.25
0.25
1
1
1
1
1
1
0.209972112
1
1
1
1
1

0.4166666667
0.4166666667
1
1
1
1
1
1
1
1
1
0.5
1
1
1
1
0.5588235294
0.2869875223
1
0.5
0.6295677362
0.25
1
1
0.5588235294
1
0.4166666667
0.4166666667
0.4166666667
1
1
0.4166666667
1
1
1
1
0.2869875223
0.2869875223
1
1
1
0.5588235294
1
0.25
0.2869875223
0.2869875223
0.1379790553
1
1
1
1

1
1
1
1
1
0.2869875223
1
0.25
1
1
1
1
0.5
1
0.6256684492
0.6256684492
1
0.376114082
0.6256684492
1
0.6773654476
0.6773654476
0.6256684492
0.061942959
0.061942959
0.6295677362
0.5588235294
0.25
1
1
0.4166666667
0.4166666667
0.5588235294
1
1
0.1666666667
1
0.4166666667
1
0.6256684492
1
1
1
1
0.209972112
1
1
1
1
0.1247771836
1

1
1
1
0.5
0.5
0.25
1
1
1
1
1
0.25
0.5
0.25
1
1
1
1
0.6256684492
1
1
1
1
1
1
1
1
0.6256684492
0.6256684492
1
1
0.4166666667
0.4178238744
0.5588235294
1
1
0.1666666667
0.1666666667
0.2869875223
0.25
0.1666666667
0.4166666667
0.4166666667
0.4166666667
0.4166666667
0.4166666667
1
0.4166666667
0.4166666667
0.4166666667
0.4166666667

0.4166666667
0.4166666667
0.4166666667
1
0.4166666667
0.4166666667
0.4166666667
0.4166666667
0.4166666667
0.5
0.6256684492
0.376114082
1
1
0.5
1
1
1
0.6256684492
0.4166666667
1
0.376114082
0.4166666667
0.4166666667
1
1
0.6295677362
0.6295677362
0.25
0.5588235294
1
1
1
0.4166666667
0.4166666667
1
0.6256684492
1
1
0.4166666667
0.4166666667
1
0.4166666667
1
0.25
1
1
0.6256684492
1
0.25
1

1
1
1
1
0.6256684492
1
1
1
1
1
1
1
1
1
1
1
1
1
0.25
1
0.25
1
1
1
1
1
1
1
1
0.25
0.4166666667
0.5
0.5588235294
1
1
1
1
1
1
1
1
1
1
1
1
1
1
1
1
1
1

1
1
1
1
1
1
1
1
1
1
1
1
1
1
0.4166666667
1
1
1
1
1
0.6295677362
1
0.6295677362
0.5588235294
1
0.5
0.6295677362
0.4166666667
1
0.6256684492
1
1
0.6256684492
1
0.4166666667
0.376114082
1
0.4166666667
0.5
1
0.5588235294
0.5588235294
1
0.6295677362
1
0.209972112
1
0.6773654476
1
1
0.4166666667

0.4166666667
1
1
1
0.5588235294
0.5588235294
1
0.6256684492
1
0.2869875223
0.2869875223
0.2869875223
0.2869875223
0.2869875223
0.4166666667
0.6256684492
1
1
0.6256684492
1
0.25
0.25
1
0.25
1
1
0.4166666667
0.5
0.5
1
0.5588235294
0.1379790553
0.6256684492
1
0.4166666667
1
0.376114082
0.4166666667
0.4166666667
1
1
1
1
1
1
0.6256684492
1
1
1
1
0.376114082

1
1
1
0.2869875223
1
1
1
0.4166666667
0.1666666667
0.376114082
0.5588235294
0.6256684492
1
0.4240540589
0.4240540589
0.4240540589
0.4166666667
0.4166666667
1
0.4166666667
1
0.6256684492
1
0.6256684492
1
1
0.5588235294
0.6256684492
0.4166666667
0.4166666667
0.4166666667
0.1666666667
0.5
1
1
1
1
1
0.4166666667
0.5588235294
1
0.2869875223
1
0.5588235294
1
1
0.6256684492
1
1
1
1

1
1
1
0.5
1
0.209972112
1
1
1
0.1666666667
0.25
0.2869875223
0.4166666667
0.1666666667
1
0.1666666667
1
0.6256684492
0.2869875223
1
0.25
1
1
0.4166666667
1
0.5588235294
1
1
0.6256684492
0.5
0.5588235294
0.5588235294
0.25
1
0.5588235294
1
0.6295677362
1
1
1
0.5
1
1
0.4166666667
0.4166666667
1
1
1
1
1
1

1
1
1
1
1
1
1
1
1
1
1
1
1
0.6295677362
1
0.6773654476
0.4178238744
1
1
0.4166666667
1
0.5588235294
1
1
1
1
0.25
1
1
0.5
0.5
0.5
0.5
0.5
0.209972112
0.209972112
0.1666666667
0.4166666667
1
1
0.5588235294
1
0.1666666667
0.1666666667
0.2869875223
0.1379790553
0.376114082
0.376114082
0.4166666667
0.6256684492
1

0.6256684492
0.6256684492
0.5
1
1
0.6256684492
0.1666666667
0.1666666667
0.4166666667
0.5588235294
1
0.5588235294
1
1
1
1
0.6256684492
0.376114082
1
0.25
0.25
0.6256684492
0.25
1
1
0.4166666667
1
1
0.6256684492
0.1666666667
0.1666666667
0.4166666667
1
1
0.209972112
0.209972112
1
0.25
0.5
0.5
0.5
1
1
0.6256684492
1
1
0.1666666667
0.5588235294
0.5588235294
0.5
1

1
1
0.5
0.25
0.25
1
1
0.5
1
0.6256684492
0.6256684492
0.6256684492
1
1
1
1
1
1
1
1
1
1
1
1
1
0.6256684492
0.6256684492
0.1666666667
0.1666666667
0.4166666667
0.4166666667
0.6773654476
0.4178238744
0.5588235294
0.5588235294
1
1
0.5588235294
0.1666666667
0.1666666667
0.1666666667
1
0.5
0.5
1
1
0.6256684492
0.376114082
0.6256684492
1
1

1
1
1
0.2869875223
0.2869875223
0.2869875223
1
1
0.5588235294
0.5588235294
0.5588235294
1
0.2869875223
0.2869875223
0.2869875223
0.2869875223
0.2869875223
0.209972112
1
1
1
1
1
1
0.0189649224
0.0189649224
0.0189649224
0.0189649224
0.1666666667
1
1
1
0.1666666667
0.1666666667
0.5
0.5
1
1
1
0.6773654476
0.5
0.2869875223
1
0.5588235294
1
0.376114082
1
1
1
0.4166666667
0.5

0.6256684492
1
1
0.4166666667
0.1666666667
0.5
1
0.2869875223
0.4166666667
1
1
1
0.4166666667
0.5588235294
1
0.5588235294
0.5
1
1
0.2869875223
0.5
1
0.25
0.5
1
1
0.4166666667
0.4166666667
1
1
0.209972112
1
0.4166666667
0.4166666667
1
1
1
1
0.6256684492
1
1
1
1
1
0.4166666667
0.5588235294
1
0.2869875223
0.4166666667
1
0.5588235294

1
1
1
1
1
1
1
1
0.376114082
0.2869875223
1
1
0.4166666667
1
1
0.5
1
1
1
1
1
1
0.4166666667
1
1
1
0.5588235294
1
1
0.5
0.4166666667
1
0.4166666667
0.376114082
1
1
1
1
0.4166666667
1
1
1
1
1
1
1
1
1
1
1
0.25

0.6256684492
0.5
0.5
1
1
0.1666666667
1
1
0.2869875223
0.209972112
0.2869875223
0.209972112
0.4178238744
0.4178238744
0.4178238744
0.4178238744
0.4178238744
1
1
1
0.4166666667
1
0.5588235294
0.5588235294
1
0.1666666667
0.1666666667
0.1666666667
0.5588235294
1
1
0.5
0.4166666667
0.5588235294
1
1
1
0.4166666667
1
0.25
0.3666803232
1
1
1
1
1
1
1
0.5
0.5
0.6773654476

1
0.5
1
1
1
0.1666666667
0.1666666667
0.6256684492
0.5588235294
1
1
1
1
1
0.6256684492
1
1
0.376114082
1
1
1
0.4166666667
0.209972112
1
0.5588235294
1
1
0.4166666667
0.4166666667
1
0.4166666667
0.5
0.2869875223
0.376114082
0.1666666667
0.5588235294
0.4166666667
1
1
1
1
1
1
0.3666803232
0.1666666667
0.1666666667
1
1
1
0.5588235294
0.5588235294

1
0.5
0.6773654476
0.4166666667
0.6256684492
0.25
0.5588235294
0.4166666667
0.25
1
0.1666666667
0.6256684492
0.6256684492
0.6256684492
0.4166666667
1
1
1
1
1
0.4166666667
1
1
0.4166666667
1
0.6295677362
0.4166666667
1
0.4166666667
0.5
1
0.5
1
1
0.5588235294
0.5588235294
0.2869875223
1
0.1666666667
0.4166666667
0.5588235294
0.25
1
1
1
1
1
0.1666666667
1
0.25
0.4166666667

1
0.1379790553
0.4166666667
1
1
0.5588235294
1
1
1
0.1379790553
1
0.5
0.5
0.5
0.4166666667
0.1379790553
1
1
0.0637254902
0.5588235294
0.1666666667
1
1
0.25
0.25
0.25
0.5588235294
0.6256684492
1
1
0.4166666667
0.4178238744
1
1
1
1
1
1
0.2869875223
1
1
0.2869875223
0.4166666667
1
0.25
0.1379790553
0.2869875223
1
1
1
0.6256684492

1
0.25
1
1
1
1
1
0.4166666667
1
0.1666666667
0.1666666667
0.4166666667
1
1
1
0.5
0.1379790553
1
0.4166666667
0.1379790553
0.1666666667
1
0.4166666667
0.1666666667
1
1
0.5588235294
1
0.5
1
0.25
0.4166666667
0.25
1
0.376114082
1
0.5588235294
0.4166666667
0.4166666667
1
0.25
1
1
0.1247771836
1
1
1
0.25
1
0.376114082
0.7287727104

1
0.4166666667
1
0.376114082
0.6256684492
0.5
0.2869875223
1
0.376114082
0.4166666667
1
1
1
0.5588235294
0.209972112
1
1
0.1666666667
0.5588235294
1
0.4166666667
1
0.4166666667
0.5588235294
0.1666666667
0.1666666667
1
0.5588235294
1
1
1
1
1
0.4166666667
0.4166666667
1
1
1
1
0.4166666667
0.1666666667
0.5
0.5
0.376114082
0.4166666667
0.1379790553
1
0.1666666667
1
1
1

0.209972112
1
1
0.376114082
0.5
0.5588235294
0.5588235294
0.5
0.4166666667
1
1
0.4166666667
0.5
1
1
1
1
1
1
1
0.25
1
1
1
1
1
1
1
1
0.4166666667
1
1
0.4166666667
0.5
1
0.5588235294
1
1
0.4166666667
1
1
1
0.5588235294
0.6773654476
0.5588235294
0.1247771836
0.4178238744
0.5
0.4166666667
1
1

0.5588235294
1
1
1
0.4166666667
0.6256684492
1
0.5
0.5588235294
0.5
1
1
0.5588235294
0.4166666667
1
0.5588235294
0.5588235294
0.209972112
1
0.25
1
1
1
0.4178238744
0.5588235294
1
1
1
0.4166666667
1
1
0.4166666667
0.2869875223
1
0.5
1
0.1666666667
1
1
1
1
0.6256684492
1
1
1
0.6256684492
1
0.6256684492
1
1
1

1
1
1
0.5588235294
1
1
1
1
1
0.25
0.4166666667
1
0.5588235294
0.5588235294
0.4166666667
1
1
0.6256684492
1
0.1666666667
1
1
0.1666666667
0.6773654476
1
1
1
0.4166666667
0.25
1
1
1
0.4166666667
0.4166666667
0.5
1
0.4166666667
0.4166666667
0.4166666667
1
1
1
1
1
1
1
1
1
1
1
1

1
1
1
0.4166666667
0.4166666667
1
1
0.1666666667
1
0.4166666667
1
0.5
0.5
0.1666666667
0.25
1
1
1
0.2869875223
1
1
1
1
1
1
1
1
1
0.376114082
1
0.5588235294
1
1
1
0.6256684492
0.4166666667
0.1666666667
1
1
0.6256684492
0.1379790553
0.4166666667
1
0.4166666667
0.4166666667
1
1
1
0.4166666667
1
1

0.6256684492
1
0.6256684492
1
0.25
0.5
1
0.5588235294
0.25
1
0.4166666667
0.4166666667
1
0.376114082
0.4166666667
0.376114082
1
1
1
1
0.4178238744
0.4166666667
0.4166666667
1
1
0.6256684492
0.6256684492
0.4166666667
0.4166666667
1
0.376114082
1
0.5588235294
1
1
0.376114082
0.1666666667
1
0.4166666667
0.4166666667
1
0.5588235294
0.5
1
0.376114082
0.376114082
0.376114082
0.376114082
0.4166666667
0.6256684492
0.4166666667

1
0.6256684492
0.5
1
0.1379790553
1
0.6256684492
0.1666666667
1
1
0.4166666667
0.1666666667
0.4166666667
1
1
0.5
1
0.6295677362
0.6256684492
1
1
0.5588235294
0.5588235294
0.061942959
1
1
0.376114082
1
0.5588235294
1
0.4166666667
1
1
0.376114082
1
0.5
0.2869875223
1
1
1
0.5
1
1
1
1
1
0.4166666667
0.4166666667
1
1
1

0.25
1
1
0.4166666667
1
0.4240540589
0.5588235294
1
0.5588235294
0.6256684492
0.6256684492
0.6256684492
0.6256684492
0.6256684492
0.6256684492
0.1247771836
0.6256684492
0.6256684492
1
0.6256684492
0.6256684492
0.5588235294
1
1
1
1
0.6256684492
1
1
1
0.2869875223
1
0.5588235294
1
0.6256684492
0.1666666667
0.1666666667
1
0.6256684492
0.6256684492
0.6256684492
0.1666666667
1
1
0.376114082
0.6773654476
1
0.6773654476
0.1666666667
0.1666666667
0.376114082

0.376114082
0.4166666667
0.4166666667
1
0.4166666667
0.4166666667
0.4166666667
1
0.5
0.4166666667
1
0.5588235294
1
0.6773654476
1
0.6773654476
1
1
0.376114082
1
1
0.4166666667
1
0.1379790553
1
1
1
0.5
1
1
1
0.4166666667
0.6256684492
0.1379790553
1
1
0.376114082
0.4166666667
0.4166666667
0.4166666667
0.6256684492
1
1
1
1
0.1379790553
0.6256684492
1
0.1666666667
0.1666666667
0.6256684492

1
0.5588235294
1
1
0.5
1
1
1
0.4166666667
1
0.209972112
1
1
0.4166666667
1
0.376114082
1
1
0.25
1
1
0.4166666667
0.25
0.4166666667
1
1
0.1666666667
1
0.5
0.5
0.2869875223
0.4166666667
1
1
1
1
1
0.5588235294
0.5588235294
1
1
1
0.25
1
1
0.5
1
0.5588235294
0.5588235294
0.5588235294
1

1
1
1
0.4166666667
0.4166666667
1
1
0.25
1
1
1
1
1
0.6256684492
0.4178238744
1
1
1
0.6256684492
0.4166666667
0.5
1
1
1
0.0637254902
1
1
0.4166666667
0.4166666667
0.6256684492
0.6256684492
0.4166666667
0.6773654476
0.1666666667
0.4166666667
0.4166666667
0.5
1
0.5588235294
1
0.4166666667
0.5
1
1
1
0.4240540589
0.5588235294
0.0637254902
0.061942959
0.6256684492
1

0.6295677362
1
0.6256684492
1
0.25
0.1666666667
0.1379790553
1
0.5588235294
0.5588235294
0.5588235294
0.6256684492
1
1
1
0.4166666667
0.6256684492
0.5
1
1
1
1
1
1
0.4166666667
1
0.5588235294
0.4166666667
1
1
1
0.4166666667
0.4166666667
1
1
0.5
1
0.4166666667
1
0.6256684492
0.1379790553
1
0.25
0.6256684492
1
0.4166666667
0.4166666667
0.4166666667
1
1
1

0.5588235294
0.376114082
0.6256684492
0.5
0.5
0.5588235294
1
0.5588235294
1
1
1
0.5588235294
0.7287727104
1
1
1
0.25
1
0.5588235294
0.6773654476
0.6773654476
0.4178238744
1
0.4166666667
0.4178238744
1
0.5
1
1
1
0.3666803232
0.376114082
0.25
0.376114082
0.4166666667
0.5588235294
0.5588235294
1
0.4166666667
0.061942959
0.6295677362
0.376114082
0.376114082
0.4166666667
1
1
0.5588235294
0.25
1
0.4166666667
0.6256684492

1
0.6256684492
1
1
0.25
0.6256684492
0.6256684492
1
1
0.6256684492
0.6256684492
1
0.6256684492
1
1
1
0.209972112
0.1379790553
1
0.5
0.25
0.1379790553
0.6256684492
0.4166666667
0.1666666667
0.25
0.6256684492
1
1
0.4166666667
1
0.4166666667
0.25
1
0.376114082
0.4166666667
0.6295677362
1
1
0.376114082
0.6256684492
1
0.1666666667
0.4166666667
0.25
1
1
0.2869875223
1
0.5
0.5

0.4166666667
1
0.1666666667
0.6256684492
0.5
1
1
1
1
0.4166666667
0.6256684492
0.6256684492
1
0.5
1
1
1
1
0.5588235294
1
1
0.376114082
1
1
0.4166666667
0.25
1
1
1
1
1
0.6256684492
0.5588235294
0.6256684492
0.6256684492
0.6256684492
1
1
1
0.4166666667
1
1
0.5
0.209972112
1
1
0.4166666667
1
1
0.4178238744
1

0.1379790553
0.6256684492
0.4178238744
0.25
1
0.5
1
0.4166666667
0.6256684492
0.6256684492
0.6256684492
0.4166666667
1
0.25
1
1
1
1
0.5
0.5
1
1
1
1
1
1
1
1
1
1
0.25
1
0.4166666667
1
0.25
0.4166666667
0.5588235294
0.5588235294
0.5588235294
0.4166666667
0.376114082
1
1
0.5
0.1379790553
0.3666803232
0.2869875223
0.2869875223
1
1
1

1
0.5588235294
0.1379790553
0.209972112
0.2869875223
0.4166666667
0.4166666667
0.6256684492
0.6256684492
0.4166666667
1
0.6256684492
0.5588235294
0.5
1
1
0.4166666667
1
1
0.25
1
1
1
1
1
1
1
1
1
1
1
0.4166666667
1
0.4166666667
1
1
0.25
1
0.6256684492
0.6256684492
1
0.1666666667
1
0.2869875223
1
1
1
1
1
0.5588235294
0.5588235294

0.5588235294
1
0.25
0.25
1
0.5588235294
0.2869875223
0.4166666667
0.6256684492
1
0.209972112
0.4166666667
0.6256684492
0.376114082
0.376114082
0.6256684492
1
1
1
1
1
0.061942959
1
0.061942959
0.4166666667
0.5
0.6773654476
1
1
1
0.3666803232
0.5
0.4166666667
0.4166666667
1
1
1
0.6773654476
1
1
0.376114082
1
1
0.4166666667
0.4166666667
0.6256684492
0.2869875223
0.5
1
1
0.4166666667

0.5588235294
1
0.5588235294
1
0.209972112
1
1
0.209972112
1
1
1
0.5
0.25
0.6256684492
0.4166666667
1
1
1
0.4178238744
0.4166666667
1
0.1247771836
1
1
0.209972112
1
1
1
0.25
0.5588235294
1
0.4166666667
0.1379790553
0.2869875223
1
0.376114082
1
1
1
0.6256684492
1
1
1
1
1
1
1
1
1
0.4166666667
0.5

0.4166666667
1
0.25
1
0.5588235294
0.5
1
1
0.25
1
0.4166666667
1
1
0.376114082
1
1
0.4166666667
1
1
0.1666666667
0.1666666667
1
1
1
0.1666666667
1
1
0.2869875223
1
1
0.6256684492
1
1
0.25
0.6256684492
1
0.25
1
1
0.6256684492
1
0.1666666667
0.4166666667
1
0.2869875223
0.4166666667
0.5
0.6256684492
0.376114082
1
0.4166666667

0.5
0.4166666667
0.4166666667
0.4166666667
0.4166666667
0.5588235294
0.3666803232
1
1
0.1247771836
1
1
0.25
0.25
0.4166666667
0.5588235294
1
1
1
1
0.6256684492
0.6295677362
0.6295677362
0.2869875223
1
0.6773654476
1
1
0.4166666667
0.4166666667
1
1
1
0.1666666667
1
1
1
0.6256684492
0.25
0.1379790553
1
1
0.5588235294
1
0.4178238744
0.4178238744
0.1666666667
0.5
1
0.376114082
0.1666666667

1
1
1
1
1
1
1
0.25
1
0.5588235294
0.5588235294
0.25
0.5588235294
1
1
1
1
0.5
1
1
1
1
1
0.25
0.2869875223
1
0.25
0.6256684492
1
0.5588235294
1
0.4178238744
1
0.1247771836
1
1
1
1
1
1
1
1
1
1
1
1
0.25
0.25
0.25
0.25
0.6256684492

0.6256684492
1
0.6256684492
0.6256684492
0.6256684492
0.6256684492
1
1
1
1
1
1
1
0.25
0.6256684492
1
1
1
1
1
1
1
1
0.25
1
0.25
0.25
0.25
0.25
0.25
0.1666666667
0.1666666667
1
0.25
0.1666666667
0.1666666667
0.1666666667
0.1666666667
0.1666666667
0.1666666667
0.5588235294
0.5588235294
0.1666666667
1
1
1
1
1
1
1
1

1
1
0.6256684492
1
1
1
1
1
1
1
1
1
1
1
1
1
1
1
1
1
0.25
0.25
1
0.25
0.5588235294
0.25
1
1
1
1
0.25
0.25
0.25
1
0.25
0.25
0.25
0.25
0.25
0.25
0.25
0.25
0.25
0.25
0.25
0.25
1
0.25
0.25
0.25
0.25

0.25
0.25
0.25
0.25
0.25
0.25
0.25
0.25
0.25
0.25
0.25
0.25
1
1
0.25
0.25
1
0.6256684492
0.1666666667
0.1666666667
0.1666666667
0.1666666667
1
1
0.4166666667
0.5
0.4166666667
1
1
1
0.4166666667
0.4166666667
0.5
0.4166666667
0.5
0.5
1
1
0.6295677362
1
1
0.6295677362
0.6295677362
1
0.2869875223
1
0.5
1
0.5
0.5
1

1
1
1
1
1
0.6737806193
1
0.6737806193
0.6737806193
1
0.5
1
0.4166666667
0.4166666667
0.4166666667
1
1
1
1
1
1
1
1
1
1
1
1
1
1
1
1
1
1
1
1
1
1
1
1
1
1
1
1
1
1
1
1
1
1
1
1

1
1
1
1
1
1
1
1
1
1
1
1
1
1
1
1
1
1
1
1
1
1
1
1
1
1
1
1
1
1
1
1
1
1
1
1
0.4166666667
0.4166666667
1
1
1
1
1
1
1
1
1
1
1
0.25
0.4166666667

0.6256684492
0.6256684492
0.25
0.25
0.25
0.6256684492
1
1
0.6256684492
1
1
1
1
1
0.6256684492
0.6256684492
1
0.6256684492
1
0.4166666667
0.6256684492
0.6256684492
0.6256684492
1
1
1
1
0.5588235294
1
1
1
0.4166666667
0.4166666667
1
1
1
1
0.4166666667
1
0.5
0.6256684492
0.5588235294
0.5
1
1
1
0.4178238744
0.1379790553
1
0.1666666667
0.25

0.5588235294
1
1
1
0.6773654476
0.6773654476
0.4166666667
0.4166666667
1
1
0.5588235294
1
1
0.4166666667
1
0.4166666667
0.4166666667
1
1
1
1
1
1
0.6256684492
1
1
1
1
1
1
0.5588235294
0.4166666667
1
1
1
1
1
0.4166666667
1
1
0.25
0.1666666667
1
1
1
1
1
1
0.5588235294
1
0.4166666667

1
1
1
1
0.4166666667
1
1
1
1
0.376114082
1
0.1666666667
1
0.5588235294
0.1666666667
0.1666666667
1
1
0.5588235294
1
1
1
1
1
1
1
1
0.5588235294
0.5
1
0.376114082
1
1
1
0.5
1
0.6256684492
1
0.6773654476
0.4166666667
1
1
0.4166666667
0.1666666667
0.1247771836
0.6256684492
0.1666666667
0.1666666667
0.4166666667
0.1666666667
1

0.0637254902
0.4166666667
0.5588235294
0.5588235294
0.5588235294
0.5588235294
0.5588235294
1
1
0.1666666667
1
0.5588235294
1
1
0.5588235294
0.376114082
1
1
0.4166666667
0.6256684492
1
1
1
0.5
0.4166666667
1
1
1
1
1
1
0.6256684492
0.4166666667
1
1
0.376114082
0.4166666667
0.1666666667
1
0.4166666667
0.1666666667
0.1247771836
0.5
0.2869875223
1
1
1
0.25
0.4166666667
0.6773654476
1

0.5588235294
1
0.5588235294
1
0.6256684492
0.6256684492
1
0.209972112
0.6295677362
0.4166666667
0.6256684492
0.6295677362
1
1
1
1
1
0.4166666667
0.376114082
1
1
0.1247771836
0.2869875223
0.5588235294
1
1
1
1
1
1
1
1
1
1
1
1
0.4166666667
0.0637254902
0.0637254902
0.4166666667
1
0.5588235294
1
0.376114082
0.25
0.4166666667
1
1
1
0.4166666667
0.4166666667

0.5588235294
0.5588235294
0.6256684492
0.6256684492
0.6256684492
0.6256684492
0.5588235294
0.376114082
1
0.25
1
1
1
1
0.4166666667
0.4166666667
1
0.4166666667
1
1
1
1
1
0.6295677362
0.25
1
0.5
1
0.4166666667
0.4166666667
1
1
0.5588235294
1
0.376114082
1
0.4166666667
1
1
0.4166666667
1
1
1
1
1
1
1
1
1
1
1

1
1
1
1
1
1
1
1
1
1
1
1
1
1
1
1
1
1
1
1
1
1
1
1
1
1
1
1
1
1
1
1
1
1
1
1
1
1
1
1
1
1
1
1
1
1
0.376114082
1
1
0.4166666667
0.4166666667

1
1
1
0.6256684492
0.6773654476
0.4178238744
1
0.5588235294
1
1
1
0.4178238744
0.376114082
0.376114082
0.4166666667
1
0.5588235294
0.25
1
1
0.6256684492
0.6256684492
1
0.25
1
1
1
0.4166666667
1
1
1
0.5
0.4166666667
0.6256684492
1
1
0.6256684492
0.4166666667
0.6256684492
0.6256684492
1
1
0.6256684492
0.6256684492
1
0.4166666667
0.5
1
1
0.376114082
0.4166666667

1
0.1666666667
0.5
0.4166666667
1
1
0.5588235294
1
1
1
1
1
0.6256684492
0.4166666667
1
0.25
0.4166666667
1
0.5588235294
0.6773654476
1
1
0.2869875223
0.6256684492
0.2869875223
0.5588235294
0.1666666667
0.3666803232
1
0.4166666667
1
0.1379790553
0.6256684492
1
1
0.4166666667
0.5588235294
1
0.1666666667
0.5
0.5588235294
0.25
1
0.4166666667
1
0.4166666667
1
1
1
0.5588235294
0.5588235294

0.6256684492
1
0.376114082
1
1
1
0.6256684492
0.5588235294
0.1666666667
1
1
1
0.25
0.061942959
1
0.6256684492
1
0.6256684492
1
1
1
0.25
1
1
0.6256684492
0.4166666667
0.5588235294
1
0.4166666667
0.5588235294
0.5588235294
0.6256684492
0.6256684492
0.4166666667
1
1
0.1666666667
0.5588235294
1
1
0.4166666667
0.4166666667
0.4166666667
0.1379790553
0.1666666667
0.5588235294
0.6256684492
0.376114082
0.5588235294
0.1666666667
1

0.6256684492
1
1
0.5588235294
0.25
1
1
0.5588235294
0.376114082
0.376114082
1
1
1
1
1
0.25
1
1
0.25
1
0.5
0.5588235294
1
0.2869875223
0.1666666667
1
1
1
0.25
1
0.2869875223
1
0.4166666667
1
0.5
0.25
1
0.25
1
1
0.5588235294
1
1
1
1
0.6295677362
0.6295677362
0.209972112
0.5588235294
0.4166666667
0.4166666667

0.209972112
1
1
0.4166666667
1
0.6773654476
0.5588235294
1
0.25
0.5588235294
0.5588235294
1
0.376114082
0.1666666667
0.1666666667
1
1
0.4166666667
0.1379790553
1
1
1
0.2869875223
0.0637254902
0.4166666667
1
0.1666666667
1
1
1
1
1
0.5588235294
1
0.25
1
1
0.5
1
0.25
0.4166666667
0.4166666667
1
0.6256684492
1
1
0.5588235294
1
1
1
1

1
1
0.6773654476
0.25
1
1
1
0.376114082
0.5588235294
1
1
0.6256684492
1
0.5
0.5588235294
0.5588235294
0.4166666667
1
0.1247771836
0.5
1
1
1
1
0.6256684492
0.4166666667
1
1
0.25
0.5
1
0.2869875223
0.1379790553
0.25
1
0.6773654476
1
0.5588235294
1
0.6256684492
0.4166666667
0.4166666667
0.6256684492
1
0.25
0.061942959
0.5588235294
1
0.376114082
0.6256684492
0.4166666667

0.4166666667
0.4166666667
1
0.5588235294
0.5588235294
1
1
1
1
1
1
0.4166666667
1
1
1
0.1666666667
0.4166666667
1
1
0.5588235294
0.5588235294
1
1
1
1
0.5
0.6256684492
1
0.376114082
0.4166666667
1
1
1
0.25
1
0.5588235294
0.6256684492
0.5588235294
0.1666666667
0.1666666667
0.5588235294
1
0.5588235294
0.5588235294
0.4166666667
0.2869875223
0.2869875223
0.4166666667
0.6256684492
1
1

1
1
0.1666666667
0.209972112
0.1379790553
0.5
1
1
1
0.5
0.5
1
0.4166666667
0.25
1
0.4166666667
0.1666666667
1
1
1
1
0.6773654476
1
1
0.5588235294
1
1
1
1
0.2869875223
0.25
1
1
0.4166666667
0.4166666667
1
1
0.4166666667
0.376114082
0.6256684492
0.1666666667
0.5588235294
0.5588235294
0.5588235294
0.5588235294
0.5588235294
0.5588235294
0.6256684492
1
1
0.5588235294

0.5588235294
1
0.4166666667
1
1
0.5588235294
0.4166666667
0.25
0.4166666667
1
0.6295677362
0.376114082
1
0.4166666667
0.5588235294
0.5588235294
0.4166666667
1
1
1
1
0.25
0.4166666667
0.25
0.25
0.25
0.6773654476
0.6773654476
1
0.376114082
0.376114082
0.1666666667
0.4166666667
1
1
1
1
0.5588235294
1
1
0.25
0.5588235294
0.2869875223
0.4166666667
0.376114082
1
0.1666666667
1
0.5588235294
0.061942959
0.2869875223

1
0.4166666667
0.2869875223
1
1
0.6256684492
0.4166666667
0.4166666667
0.5588235294
1
0.4166666667
1
1
0.25
1
0.5588235294
0.4166666667
0.1666666667
0.376114082
1
0.6256684492
1
1
1
1
1
1
1
1
1
0.4166666667
1
1
0.5588235294
1
1
0.5588235294
1
1
1
0.061942959
1
1
1
1
1
1
1
0.4166666667
0.3666803232
1

1
0.4166666667
1
1
0.6773654476
1
0.5
1
1
0.25
1
0.5
0.1666666667
1
1
0.25
0.5
1
0.5588235294
1
1
0.4178238744
1
1
0.1666666667
0.5
1
1
0.6256684492
1
0.4166666667
0.2869875223
0.2869875223
1
1
0.6773654476
0.5588235294
0.6773654476
0.6773654476
0.5588235294
1
0.4166666667
1
0.061942959
0.061942959
0.061942959
0.061942959
0.5
0.5588235294
1
1

0.5
1
0.4166666667
0.5588235294
1
1
0.0637254902
0.0637254902
0.6256684492
0.5
1
0.25
1
0.5588235294
1
1
1
1
1
1
1
1
1
0.5588235294
0.25
0.376114082
0.6295677362
1
0.2869875223
1
1
1
1
0.4166666667
1
1
1
0.5
1
0.25
0.2869875223
1
0.6256684492
0.4166666667
0.4166666667
1
1
0.4166666667
0.376114082
1
0.4166666667

1
0.5588235294
0.6256684492
1
1
1
1
0.2869875223
1
1
1
0.6256684492
1
0.6256684492
1
1
0.376114082
1
0.1666666667
0.1379790553
1
1
0.6773654476
0.5588235294
0.6773654476
1
1
1
0.5588235294
1
1
0.6295677362
1
1
0.5588235294
0.5588235294
0.5588235294
1
0.2869875223
0.1666666667
1
1
1
1
1
0.6295677362
0.4240540589
0.4240540589
0.4240540589
1
1

0.4166666667
0.4166666667
1
1
1
0.4166666667
0.4166666667
0.4166666667
1
1
0.25
1
1
0.4166666667
0.4166666667
1
0.5588235294
1
0.376114082
1
1
0.1379790553
0.1379790553
0.1379790553
0.4166666667
1
1
0.4166666667
0.5
0.4166666667
1
1
0.1666666667
1
0.5
0.376114082
1
1
1
1
0.4178238744
1
1
1
1
1
0.25
1
1
0.5588235294
0.1379790553

0.6256684492
0.6256684492
0.6256684492
0.6256684492
0.6256684492
1
0.6256684492
1
1
1
1
0.4166666667
1
1
0.6256684492
0.4166666667
1
1
1
0.4166666667
1
1
1
1
0.4166666667
0.25
0.25
1
1
1
0.4166666667
0.4166666667
0.6256684492
1
0.25
0.4166666667
0.1666666667
1
1
0.25
1
1
1
1
0.061942959
0.2869875223
0.4178238744
1
0.5
0.4166666667
0.6256684492

0.4166666667
1
1
1
1
0.2869875223
1
0.5
1
1
1
1
0.6773654476
0.1666666667
0.6773654476
0.4166666667
0.5588235294
0.5588235294
0.5
1
0.5588235294
1
0.5588235294
0.061942959
0.376114082
0.5
0.5
1
0.5
0.4166666667
0.376114082
0.376114082
1
0.4166666667
1
1
0.5
0.5
0.4178238744
1
1
1
1
1
1
0.1666666667
0.6256684492
1
1
1
0.1666666667

1
0.5
1
1
0.5588235294
0.6256684492
0.4166666667
0.25
0.25
0.4166666667
1
0.6256684492
1
0.5588235294
0.5588235294
0.5588235294
0.5588235294
0.5588235294
0.5588235294
0.5588235294
0.5588235294
0.5588235294
0.5588235294
0.5588235294
0.5588235294
0.5588235294
0.5588235294
0.5588235294
0.5588235294
0.5588235294
0.5588235294
0.5588235294
0.5588235294
0.5588235294
0.5588235294
0.5588235294
0.5588235294
0.5588235294
1
0.1247771836
0.5588235294
0.5588235294
0.5588235294
0.5588235294
1
1
0.6256684492
1
1
0.6773654476
0.5588235294

0.4166666667
0.4166666667
0.5588235294
1
0.4166666667
1
0.5
1
1
1
1
0.061942959
0.25
1
1
0.4178238744
0.1247771836
0.4166666667
0.6773654476
0.6773654476
0.5
1
0.6256684492
0.5588235294
0.5588235294
0.5588235294
0.5588235294
0.5588235294
1
1
1
1
1
0.6256684492
1
0.4166666667
1
1
0.4166666667
0.6256684492
1
1
0.4166666667
1
1
1
0.4166666667
1
0.2869875223
0.5
0.5

1
1
1
1
1
0.4166666667
1
0.376114082
1
1
1
0.1666666667
0.1666666667
1
0.4166666667
0.6295677362
1
1
0.4166666667
0.1666666667
0.1666666667
1
1
0.4166666667
0.061942959
1
1
1
1
1
0.5588235294
0.4166666667
0.4166666667
1
0.6256684492
0.5588235294
0.4166666667
1
0.376114082
0.376114082
0.376114082
1
1
0.4166666667
1
1
1
1
1
1
0.4166666667

0.376114082
1
0.1666666667
1
1
1
1
1
1
0.25
0.25
1
0.376114082
1
1
1
1
1
0.4166666667
1
0.4178238744
0.1666666667
1
1
0.1379790553
1
0.5588235294
0.25
1
1
1
0.5588235294
0.2869875223
0.25
1
1
1
0.6256684492
0.6256684492
0.6256684492
0.6256684492
0.209972112
0.209972112
0.1379790553
0.5588235294
1
1
1
0.1666666667
0.1379790553
0.6256684492

0.5588235294
0.0637254902
0.0637254902
1
1
0.1247771836
1
1
1
0.6256684492
1
1
1
1
1
0.5
0.5
1
0.6256684492
1
1
1
0.4166666667
0.5588235294
0.5588235294
0.5588235294
1
0.5588235294
0.5588235294
1
1
0.25
1
0.4166666667
0.4166666667
0.5588235294
1
0.4166666667
1
1
1
0.4166666667
1
1
1
1
1
1
0.376114082
0.4166666667
1

0.25
0.1666666667
1
0.25
0.4166666667
0.2869875223
0.6256684492
1
1
1
0.4166666667
0.5
0.25
1
1
1
1
1
1
1
1
1
1
1
1
1
1
0.6256684492
1
0.1379790553
1
1
1
1
0.5
0.4178238744
1
0.6256684492
0.4166666667
1
1
0.376114082
0.4166666667
1
1
1
1
1
0.4166666667
1
1

1
1
0.4166666667
1
1
1
0.4166666667
0.6256684492
0.5
0.376114082
1
1
1
1
1
0.5588235294
1
1
1
0.5588235294
1
0.2869875223
0.2869875223
1
1
0.6256684492
1
0.3666803232
1
1
0.5588235294
0.4166666667
1
1
1
1
0.4166666667
0.4166666667
1
1
1
1
1
0.1379790553
1
1
1
0.6773654476
1
1
1

1
1
0.6256684492
1
1
1
1
0.209972112
1
1
1
0.6256684492
1
1
1
1
1
0.6295677362
1
0.5
1
0.6256684492
1
0.4166666667
1
0.1379790553
0.5588235294
0.4178238744
1
0.4166666667
1
0.5588235294
1
1
1
1
0.5588235294
0.1379790553
1
1
0.4166666667
0.6295677362
0.1666666667
0.5588235294
0.4166666667
0.25
0.4166666667
1
0.6256684492
1
0.5

1
0.4166666667
0.5588235294
0.25
1
0.376114082
1
1
1
1
1
0.3666803232
1
1
1
1
0.4166666667
0.5
0.6256684492
0.5588235294
1
1
0.6256684492
1
0.5588235294
0.25
1
1
0.25
1
0.5588235294
0.6773654476
0.6256684492
0.209972112
0.2869875223
0.5588235294
0.4166666667
1
1
0.1379790553
0.5588235294
1
1
1
1
1
0.1666666667
0.1666666667
1
1
1

1
1
1
0.5588235294
1
0.061942959
1
0.4166666667
1
1
1
1
0.1666666667
0.1666666667
1
0.6256684492
1
1
1
0.6256684492
0.2869875223
1
1
0.25
0.376114082
1
1
0.25
1
1
0.25
0.4166666667
0.5
1
0.5588235294
0.5
1
0.1666666667
0.4166666667
1
1
1
0.4166666667
0.5
1
1
1
0.25
0.6256684492
1
1

0.5588235294
0.25
1
0.6256684492
1
0.6773654476
1
0.6256684492
0.6256684492
1
0.061942959
0.1379790553
1
0.4166666667
0.5
0.376114082
1
0.4166666667
1
1
0.4166666667
0.5588235294
0.4178238744
1
1
1
0.209972112
1
1
1
0.25
0.6773654476
1
0.4166666667
1
0.1666666667
0.4166666667
1
1
1
1
1
1
0.6256684492
1
1
0.1379790553
0.5588235294
1
1
0.25

1
1
1
1
0.6256684492
0.4166666667
0.4166666667
0.4166666667
1
1
1
1
1
0.1666666667
1
1
1
0.25
0.5588235294
1
0.25
0.4166666667
0.1666666667
1
0.6256684492
0.1666666667
1
1
1
1
1
1
1
1
1
1
1
1
1
0.5588235294
0.4166666667
0.0637254902
0.25
0.5588235294
1
1
1
0.1666666667
0.2869875223
0.4178238744
0.5

0.5588235294
0.5588235294
0.5588235294
0.1379790553
0.4166666667
0.5
0.5
1
0.376114082
0.6773654476
1
1
0.25
1
1
1
1
0.4166666667
1
1
0.1379790553
1
1
0.6256684492
0.4166666667
0.4166666667
0.6256684492
1
1
0.1666666667
1
1
1
0.5588235294
0.1666666667
0.4166666667
1
1
0.4166666667
1
0.1666666667
0.1666666667
0.2869875223
1
0.5
1
0.4166666667
0.5
0.5
1
1

1
0.6256684492
1
0.4166666667
1
0.376114082
1
1
0.2869875223
1
0.4166666667
0.25
0.25
0.5588235294
0.1666666667
0.4166666667
0.6256684492
0.4166666667
1
1
1
1
1
1
1
0.5588235294
0.5
0.3666803232
1
0.5
0.5
0.376114082
0.6256684492
0.6256684492
0.25
1
1
1
1
1
1
1
0.5
1
1
0.2869875223
0.2869875223
0.2869875223
0.5588235294
0.1247771836
1

1
0.4178238744
0.376114082
0.376114082
1
1
1
0.1666666667
1
1
0.1666666667
1
1
0.5588235294
1
1
1
1
1
1
1
1
0.4166666667
0.6256684492
0.4166666667
0.5
1
0.6256684492
0.6256684492
0.4178238744
1
1
0.4166666667
0.4166666667
0.1666666667
0.4166666667
0.376114082
0.5588235294
0.5
0.5
0.5
0.4166666667
0.6256684492
1
1
1
1
1
0.6256684492
0.6256684492
0.6256684492

0.6256684492
0.5588235294
0.1666666667
0.6773654476
0.5588235294
0.5588235294
1
1
0.1666666667
0.25
1
0.2869875223
1
0.5588235294
0.5588235294
0.5588235294
0.6773654476
0.5588235294
1
1
1
1
1
0.25
0.25
0.25
1
1
1
0.4166666667
0.6256684492
1
1
0.5
0.5
1
1
1
0.2869875223
1
0.4166666667
0.4166666667
1
1
1
1
1
0.4166666667
0.4166666667
0.4166666667
0.4166666667

0.4166666667
1
1
1
0.5
1
1
1
0.4166666667
0.061942959
1
1
0.1666666667
0.4166666667
0.4166666667
0.6737806193
0.5588235294
0.5588235294
1
0.376114082
0.6256684492
0.6256684492
1
1
1
0.4166666667
0.4166666667
1
1
1
1
1
1
0.376114082
1
1
0.4166666667
1
0.4166666667
1
0.1666666667
1
0.5588235294
0.5588235294
0.5588235294
0.5588235294
0.5588235294
0.6256684492
0.6256684492
1
1

0.4166666667
1
1
0.6256684492
1
1
1
0.376114082
1
0.4166666667
1
1
1
1
1
0.4166666667
0.4166666667
0.4166666667
0.4166666667
0.4166666667
0.4166666667
0.4166666667
0.4166666667
0.25
0.25
0.061942959
0.1379790553
1
0.25
1
0.25
0.25
0.4178238744
0.25
1
0.25
1
1
0.25
0.25
1
0.25
1
0.25
1
1
0.25
1
0.4166666667
1
0.5588235294

0.1379790553
1
0.1379790553
1
1
1
0.5588235294
1
0.5588235294
1
1
0.25
0.2869875223
1
0.25
0.4166666667
0.5588235294
0.5588235294
1
1
0.5588235294
1
0.376114082
0.4166666667
1
0.1379790553
0.25
1
1
1
0.1666666667
1
1
1
1
1
0.4166666667
0.25
0.1666666667
0.5588235294
0.5588235294
1
1
1
1
1
0.5588235294
1
1
0.6773654476
0.376114082

1
1
1
1
0.5
1
1
0.5588235294
0.6256684492
0.4166666667
0.6295677362
0.2869875223
1
1
0.4166666667
1
1
1
1
1
1
1
0.4166666667
0.4166666667
0.4166666667
1
0.4166666667
1
1
0.376114082
1
1
1
1
0.4166666667
0.25
0.25
0.25
0.25
0.25
0.25
0.25
0.25
0.25
0.25
0.25
0.25
0.25
0.25
0.25
0.25

0.25
0.4166666667
0.4166666667
1
0.4166666667
0.4166666667
0.25
1
1
0.4166666667
0.1666666667
0.4166666667
0.4166666667
1
1
1
0.6295677362
0.6256684492
0.5588235294
0.5
0.5
1
0.5
1
1
1
1
0.5
1
1
1
1
0.4166666667
0.4166666667
1
1
0.4166666667
0.4166666667
0.209972112
0.5
1
1
1
0.1247771836
0.1666666667
0.6256684492
0.6295677362
0.4166666667
1
0.4166666667
1

0.5588235294
0.5588235294
0.25
1
0.5588235294
0.6256684492
1
0.4166666667
0.6256684492
1
0.1666666667
1
0.5588235294
0.2869875223
0.2869875223
0.2869875223
1
1
0.1666666667
0.6256684492
0.6256684492
0.4166666667
0.376114082
0.4166666667
0.6256684492
0.5588235294
1
1
1
1
0.5
1
1
1
0.376114082
1
0.25
0.25
0.4166666667
1
0.6773654476
1
1
0.25
0.4166666667
0.209972112
1
1
1
1
1

1
0.5588235294
0.1666666667
0.1666666667
0.4166666667
1
1
1
0.5588235294
1
0.4996104098
0.4996104098
0.4996104098
0.4996104098
0.4996104098
1
0.6256684492
0.5588235294
0.6256684492
0.6256684492
0.6256684492
1
1
0.6256684492
1
0.6256684492
1
0.6256684492
0.6256684492
0.6256684492
1
1
1
1
1
1
0.5588235294
0.5
0.4166666667
1
1
1
1
1
1
1
1
1
1
1
1

1
0.4166666667
0.4166666667
0.1247771836
1
1
0.2869875223
0.2869875223
0.1666666667
0.5588235294
0.2869875223
0.4166666667
1
1
0.6256684492
0.4166666667
1
0.6256684492
1
1
1
0.4166666667
0.1666666667
0.4166666667
1
1
0.5588235294
1
0.5
1
0.5588235294
1
1
0.25
0.6256684492
0.5588235294
1
1
1
1
1
1
0.5
1
0.061942959
0.25
0.3666803232
0.4166666667
0.376114082
0.5
1

1
1
0.4166666667
0.1666666667
0.2869875223
1
1
0.4166666667
1
1
1
0.4166666667
0.1666666667
1
0.4166666667
0.25
1
1
0.4166666667
0.4166666667
1
0.6256684492
0.6256684492
1
1
0.4166666667
1
1
0.209972112
1
1
1
0.1666666667
1
1
1
0.209972112
0.376114082
0.1666666667
1
1
1
0.6295677362
1
1
1
1
1
1
1
1

0.6773654476
0.376114082
0.4166666667
0.25
1
1
0.25
1
0.5588235294
1
0.5
1
1
1
1
1
1
0.1666666667
0.209972112
1
0.4178238744
1
0.6256684492
1
0.376114082
1
1
0.6256684492
1
0.2869875223
0.4166666667
0.6256684492
1
0.5588235294
0.25
0.25
0.4166666667
0.25
1
1
1
1
0.5
0.4166666667
0.1247771836
1
0.1379790553
0.5
1
1
1

1
1
0.4166666667
1
0.4166666667
1
1
1
1
1
1
0.5588235294
0.25
0.6256684492
0.2869875223
1
0.0637254902
0.0637254902
1
1
0.6773654476
0.6773654476
0.5
0.4166666667
1
0.5588235294
0.5588235294
1
1
1
0.5588235294
0.2869875223
0.2869875223
0.4166666667
1
1
1
1
1
1
0.6256684492
0.5588235294
0.5588235294
1
0.6256684492
1
1
1
0.5588235294
0.4178238744
0.4178238744

0.6295677362
0.4166666667
0.2869875223
1
0.0637254902
1
1
1
1
0.1666666667
1
1
1
0.4166666667
1
0.6256684492
1
0.376114082
1
0.1666666667
1
0.5
0.5
1
0.2869875223
0.2869875223
1
0.5588235294
0.5588235294
1
1
1
1
1
1
0.5588235294
1
0.4166666667
0.4166666667
0.5588235294
1
1
1
0.25
0.6256684492
0.6256684492
1
0.6256684492
1
0.25
0.376114082

0.1247771836
0.1247771836
0.376114082
0.376114082
0.376114082
0.376114082
0.376114082
0.376114082
0.376114082
0.1247771836
0.1247771836
0.5588235294
0.5588235294
1
1
1
0.4166666667
1
1
1
0.6256684492
0.6256684492
0.4166666667
1
1
1
1
1
1
0.4178238744
0.1666666667
0.6773654476
0.6295677362
1
0.5
1
1
0.376114082
0.376114082
0.5588235294
0.5588235294
1
0.0637254902
1
0.5588235294
0.5588235294
0.4166666667
1
1
0.4166666667
1

1
1
0.6256684492
0.5
1
0.5588235294
1
1
1
1
1
1
1
0.4166666667
1
1
0.4166666667
0.4166666667
1
1
1
1
0.5588235294
1
0.1666666667
1
1
1
1
1
1
0.5588235294
1
1
1
1
1
1
0.5588235294
1
1
1
1
1
1
1
1
1
0.5
1
0.5588235294

0.5588235294
1
1
0.1666666667
1
0.5588235294
0.5588235294
0.5588235294
0.5588235294
1
1
1
1
1
1
0.25
0.4166666667
0.5588235294
1
1
1
1
0.25
1
0.4166666667
0.4166666667
0.6256684492
1
0.1247771836
1
1
0.6256684492
0.25
0.4166666667
0.3666803232
0.4166666667
1
1
1
1
1
1
1
0.4166666667
0.6256684492
0.4166666667
0.1666666667
0.1379790553
0.4166666667
0.4166666667
1

1
1
1
0.4166666667
0.4166666667
1
0.4166666667
0.4166666667
1
1
0.4166666667
1
0.4166666667
0.376114082
0.5588235294
1
0.1666666667
1
1
0.1666666667
0.4166666667
0.4166666667
1
0.376114082
0.5
0.4166666667
0.2869875223
0.2869875223
0.25
0.1666666667
0.2869875223
0.376114082
1
0.4166666667
0.0637254902
1
0.2869875223
1
0.6256684492
1
0.5588235294
0.4166666667
1
0.5588235294
0.6773654476
0.5588235294
0.6773654476
1
1
0.6256684492
0.6256684492

1
1
0.1666666667
0.4166666667
1
0.5588235294
0.6256684492
0.6256684492
0.1666666667
0.5588235294
0.25
0.6295677362
1
1
1
1
1
0.6256684492
0.4166666667
0.4166666667
1
1
0.5
0.5
1
0.5588235294
0.5588235294
0.5588235294
1
0.5588235294
0.6256684492
0.25
1
1
1
0.6256684492
0.6256684492
1
1
1
0.376114082
0.061942959
1
0.4166666667
0.4166666667
0.4166666667
0.4166666667
0.25
1
1
1

0.2950732295
0.376114082
1
1
1
1
1
1
0.25
0.376114082
1
1
0.5588235294
0.5588235294
0.1666666667
1
1
1
1
1
0.25
0.25
1
0.5588235294
0.5588235294
0.5
0.5
0.5588235294
0.5588235294
0.5588235294
0.5
0.5
1
1
0.1666666667
1
0.4166666667
0.25
0.4166666667
0.4166666667
1
1
1
1
1
0.376114082
1
1
0.6773654476
0.6773654476
1

0.6256684492
0.4166666667
0.4166666667
0.6256684492
1
1
1
1
1
0.4166666667
1
1
0.7287727104
0.4166666667
0.1666666667
0.25
0.25
1
1
0.1379790553
0.1379790553
0.1379790553
1
1
1
1
0.6295677362
0.6295677362
0.0637254902
0.0637254902
0.5588235294
0.6773654476
1
1
1
1
1
1
1
1
1
1
1
1
0.25
1
1
0.4166666667
0.1666666667
0.1666666667
0.6256684492

0.6256684492
1
0.4166666667
1
0.25
0.25
1
0.5588235294
0.4166666667
1
1
1
0.5588235294
0.5
0.2869875223
0.5
1
0.4166666667
1
0.376114082
1
1
0.5
1
1
1
0.5588235294
1
1
0.4166666667
0.6256684492
0.6256684492
0.5588235294
1
1
0.5588235294
1
0.6256684492
0.6295677362
0.4166666667
0.1379790553
0.25
1
0.25
0.4166666667
1
0.6256684492
0.6256684492
0.4166666667
1
0.25

0.4166666667
0.6256684492
0.25
0.061942959
0.4166666667
0.5
0.3666803232
1
0.5588235294
1
1
1
1
0.6773654476
1
1
0.4166666667
1
1
0.4166666667
0.5
0.5
0.4166666667
1
1
0.1666666667
0.1666666667
1
1
1
1
0.1666666667
0.25
0.6256684492
0.6256684492
1
1
0.25
0.5588235294
0.4166666667
1
1
0.5588235294
0.1666666667
1
0.4166666667
1
1
0.25
0.4166666667
1

1
1
0.4166666667
0.4166666667
0.4166666667
0.4166666667
0.4166666667
1
0.1666666667
0.2869875223
1
0.4166666667
1
1
0.4166666667
1
1
0.25
1
0.376114082
1
0.6256684492
0.25
0.5
1
1
0.1666666667
1
0.0637254902
1
1
1
0.376114082
0.6256684492
0.6256684492
0.6256684492
1
0.5588235294
0.6256684492
1
0.6256684492
0.6256684492
0.1666666667
1
1
1
0.061942959
0.6256684492
1
1
1

1
1
0.2869875223
0.2869875223
1
1
0.4166666667
0.2869875223
1
1
1
1
0.25
1
1
1
1
1
1
1
1
0.376114082
1
1
0.1666666667
1
1
1
0.4166666667
0.4166666667
0.4166666667
1
1
0.25
1
0.25
0.1379790553
0.1666666667
1
0.376114082
0.4166666667
1
1
1
1
0.5588235294
1
0.1379790553
1
0.5588235294
0.25

0.25
0.25
0.25
0.25
1
0.5588235294
1
0.4166666667
0.4166666667
0.4166666667
1
1
1
1
0.6256684492
0.6773654476
0.6773654476
0.4166666667
1
1
1
1
1
0.6773654476
1
0.4166666667
1
1
1
1
1
0.5588235294
1
1
1
0.4166666667
1
1
0.4166666667
0.4166666667
1
1
1
1
0.5
0.5588235294
1
1
1
1
0.4178238744

1
0.061942959
1
0.5588235294
0.25
0.4166666667
1
1
0.4166666667
1
1
1
0.4166666667
0.6295677362
1
1
1
1
0.4166666667
1
1
0.5
1
1
1
1
0.376114082
1
1
0.376114082
1
1
1
0.1666666667
0.4166666667
0.5588235294
0.376114082
0.1666666667
1
1
1
0.5
1
0.6256684492
1
1
1
1
1
0.25
1

0.4166666667
1
0.4166666667
0.5588235294
0.5588235294
1
0.6256684492
0.5588235294
1
0.5
1
0.4166666667
1
0.6256684492
1
1
1
1
0.5
1
0.6256684492
1
1
1
1
1
0.5
1
1
0.25
0.6256684492
0.376114082
1
0.6256684492
1
1
1
0.4166666667
1
0.5
0.2869875223
1
1
0.6773654476
0.5588235294
1
0.5588235294
0.376114082
0.5588235294
0.4166666667
0.4178238744

0.4166666667
1
0.4166666667
1
0.25
0.6256684492
0.5588235294
1
1
0.25
1
1
1
1
1
0.4178238744
0.376114082
1
0.6256684492
1
1
1
0.5
1
1
0.6773654476
1
1
1
0.1666666667
0.1666666667
1
0.5588235294
0.25
1
0.4166666667
0.25
0.376114082
0.5
1
0.4166666667
0.5588235294
1
1
1
0.4166666667
1
0.5
1
1
1

1
0.376114082
0.4166666667
1
1
1
0.4166666667
1
1
0.2869875223
1
1
1
0.4166666667
0.4166666667
0.0637254902
0.25
1
1
0.1666666667
1
1
1
1
1
1
0.4166666667
0.5588235294
0.25
0.1379790553
1
1
1
1
1
1
1
0.5
0.4166666667
0.376114082
0.4166666667
1
0.4166666667
1
0.4166666667
1
1
1
1
0.6256684492
1

0.1666666667
0.2869875223
0.376114082
0.5588235294
0.5588235294
1
1
0.5588235294
0.5588235294
0.5588235294
1
0.4166666667
1
0.4166666667
0.5
1
0.25
1
0.5588235294
0.5588235294
1
1
1
0.1666666667
0.1666666667
1
0.1666666667
0.4166666667
1
1
1
0.4166666667
0.4166666667
1
0.376114082
0.376114082
1
1
1
1
0.6256684492
0.6256684492
0.4166666667
0.1666666667
1
0.4166666667
0.4166666667
1
0.4166666667
0.5588235294
0.4166666667

0.4166666667
0.4166666667
1
0.209972112
0.4166666667
0.6256684492
1
0.6295677362
1
0.6256684492
0.4166666667
0.4166666667
1
0.25
1
0.5588235294
0.5588235294
0.5588235294
0.5588235294
0.6256684492
1
0.4178238744
0.4178238744
1
0.6773654476
1
0.4166666667
1
0.25
1
1
1
1
1
0.25
0.25
0.4166666667
0.6256684492
1
0.5
0.4178238744
0.6773654476
1
1
0.2869875223
0.2869875223
1
1
1
1
0.5588235294

1
0.6295677362
0.6295677362
0.4166666667
0.4166666667
0.4166666667
1
1
0.5
1
1
1
1
1
1
1
1
0.4166666667
0.4166666667
0.4166666667
0.4166666667
0.5588235294
0.5
1
1
1
1
0.5
0.1666666667
1
1
0.6256684492
0.6256684492
0.4166666667
0.4166666667
0.4166666667
0.5
0.6256684492
1
0.5588235294
0.1247771836
0.5588235294
0.5588235294
0.5588235294
0.1666666667
1
1
1
1
1
1

0.6256684492
0.1666666667
0.25
0.25
0.25
1
1
0.4166666667
0.5588235294
0.5588235294
0.5588235294
1
1
0.376114082
1
1
0.6256684492
0.5588235294
0.6295677362
0.5
1
0.4166666667
1
0.6295677362
0.376114082
1
1
1
1
1
1
0.5588235294
1
1
1
1
1
0.6295677362
0.6295677362
0.6256684492
1
0.376114082
1
1
0.6256684492
0.4166666667
0.2869875223
0.5588235294
1
1
0.5588235294

0.1666666667
1
1
1
1
0.4166666667
0.25
0.6256684492
0.1666666667
0.4166666667
0.6295677362
0.6295677362
0.1247771836
0.1247771836
1
1
1
0.25
0.6256684492
1
0.6256684492
1
0.376114082
1
1
1
1
1
1
1
1
1
1
1
1
1
1
1
1
1
1
1
1
1
1
1
1
1
1
0.4166666667
1

0.4166666667
0.4166666667
1
1
0.6256684492
1
1
0.4166666667
0.25
0.6773654476
0.5588235294
0.1379790553
1
0.6773654476
0.4166666667
0.209972112
1
1
1
1
0.2869875223
0.4166666667
0.25
1
0.5588235294
1
0.4166666667
1
1
0.6773654476
0.6256684492
0.4166666667
0.209972112
1
1
0.209972112
0.6256684492
0.4166666667
0.5
0.25
1
0.2869875223
0.2869875223
0.5
0.4166666667
1
1
0.1666666667
1
0.2869875223
0.1666666667

0.4178238744
1
0.5
0.376114082
0.25
0.5588235294
1
1
0.6256684492
1
0.5588235294
0.5588235294
0.5588235294
0.5
0.6256684492
0.6256684492
0.061942959
1
0.1379790553
0.5
0.5
0.5588235294
1
0.5588235294
1
0.4166666667
1
0.5588235294
0.3666803232
1
1
1
1
1
0.6256684492
0.4166666667
0.5
1
1
0.5588235294
1
1
1
0.4166666667
1
0.5588235294
1
1
0.4166666667
1
1

0.2869875223
1
1
0.376114082
1
1
1
1
1
0.376114082
1
1
0.1379790553
0.1379790553
0.1379790553
1
0.6773654476
0.5588235294
1
1
1
1
0.376114082
0.376114082
0.209972112
1
1
0.4166666667
1
1
1
1
1
0.4166666667
1
0.25
0.4166666667
0.4166666667
0.6773654476
0.6773654476
0.6773654476
0.6773654476
0.6773654476
1
0.5588235294
0.5588235294
1
0.4166666667
1
1
1

0.4166666667
1
0.061942959
1
0.209972112
1
1
1
0.5588235294
0.4166666667
1
0.2869875223
1
1
1
0.209972112
0.6256684492
0.1379790553
1
0.5588235294
0.5588235294
1
0.5
1
1
0.5588235294
0.4166666667
0.4166666667
0.5588235294
0.5588235294
0.5588235294
1
1
1
1
0.376114082
0.6256684492
0.6256684492
1
0.4166666667
0.376114082
1
0.6295677362
1
1
0.4166666667
0.5
0.5588235294
1
1
0.4166666667

1
0.6295677362
0.6295677362
0.5588235294
1
1
0.4166666667
1
1
1
1
1
0.1666666667
0.6295677362
0.6295677362
0.376114082
1
1
0.6256684492
0.5
0.1666666667
0.6256684492
0.6256684492
0.2869875223
0.4166666667
0.4166666667
1
0.5
1
0.5588235294
0.1379790553
1
0.6773654476
1
1
0.061942959
0.5588235294
1
1
1
1
0.2869875223
1
0.1666666667
0.6773654476
1
1
0.376114082
1
1
1

1
1
1
1
1
1
0.4166666667
1
1
1
1
1
1
1
1
1
1
1
1
1
1
1
0.5588235294
0.5588235294
0.5588235294
0.5588235294
0.5588235294
1
1
1
1
0.6256684492
0.4166666667
1
1
1
0.1666666667
0.25
1
1
1
1
0.25
1
1
1
1
1
0.4166666667
1

0.4166666667
0.4166666667
1
1
0.376114082
0.376114082
1
1
1
0.6256684492
0.6256684492
0.209972112
1
1
1
1
1
0.6295677362
0.2869875223
0.6256684492
0.4166666667
0.5
1
1
1
1
1
0.4166666667
0.4166666667
0.25
0.5588235294
0.25
0.25
0.25
1
0.25
0.25
0.25
0.25
0.4166666667
0.1666666667
0.1666666667
1
0.376114082
0.6773654476
1
1
1
0.4166666667
1
0.1666666667

0.1666666667
0.4166666667
0.2869875223
0.4166666667
1
1
0.376114082
0.5588235294
1
1
1
0.1666666667
0.1379790553
0.4166666667
0.5588235294
0.5588235294
1
1
0.376114082
1
1
0.5588235294
1
0.25
0.5588235294
0.061942959
1
0.4166666667
1
1
0.6256684492
1
0.4166666667
0.4166666667
1
0.1666666667
1
1
0.1379790553
0.25
0.1379790553
1
1
1
1
0.6773654476
1
1
0.4166666667
0.6256684492
1

1
1
1
1
1
1
1
1
0.5
1
0.2869875223
0.25
1
1
1
1
1
1
1
0.4166666667
0.5588235294
1
1
0.5588235294
0.25
0.4166666667
1
0.25
0.25
0.25
1
1
0.4166666667
1
0.5
0.1379790553
1
1
0.4166666667
0.1666666667
0.4166666667
0.376114082
0.061942959
0.6256684492
0.4166666667
1
0.4178238744
0.6256684492
0.6256684492
1
1

1
1
0.1379790553
1
1
1
0.4166666667
0.4166666667
0.4166666667
0.4166666667
0.25
0.25
0.1666666667
0.2869875223
0.4166666667
0.25
1
0.2869875223
0.1666666667
1
1
1
1
1
0.209972112
0.209972112
0.4166666667
1
1
1
1
1
0.1666666667
1
1
1
0.4166666667
0.25
1
1
0.376114082
0.25
0.25
0.3666803232
0.6295677362
0.5
0.5588235294
0.1666666667
1
0.6773654476
0.5

0.4166666667
0.4166666667
0.5588235294
0.5588235294
0.209972112
0.6256684492
0.209972112
0.4166666667
0.4166666667
0.4166666667
0.4166666667
0.4166666667
0.4166666667
0.4166666667
0.25
0.5
0.4166666667
1
1
0.5
0.25
0.209972112
0.4166666667
1
0.4166666667
1
1
1
1
0.2869875223
0.5588235294
1
1
0.4166666667
1
1
0.4166666667
1
0.4166666667
0.4166666667
0.2869875223
0.6773654476
0.6773654476
0.6773654476
0.6773654476
0.6773654476
1
0.6773654476
0.5588235294
1
1

1
1
0.6773654476
1
1
0.6773654476
1
0.5588235294
0.5588235294
0.376114082
1
0.4166666667
0.4166666667
0.4166666667
0.4166666667
0.4166666667
0.5588235294
1
1
0.5
0.25
1
1
0.4166666667
0.4166666667
1
1
1
0.6295677362
0.4166666667
0.376114082
1
0.5
1
0.6773654476
0.6773654476
1
0.5
1
1
0.1666666667
0.1666666667
0.4178238744
0.5588235294
1
0.25
0.4166666667
1
0.5
0.4166666667
1

0.4166666667
0.4166666667
0.6256684492
1
0.25
0.5588235294
0.4166666667
1
0.4166666667
1
0.1247771836
0.1247771836
0.1247771836
0.1247771836
0.1247771836
0.1247771836
0.1247771836
0.1247771836
0.1247771836
0.1247771836
0.1247771836
0.1247771836
0.1247771836
0.25
0.4166666667
1
1
1
1
0.4166666667
0.5588235294
0.209972112
0.6256684492
0.6256684492
1
1
1
1
1
0.5
0.6256684492
1
1
1
1
1
1
0.5588235294
0.4166666667
0.6295677362
1

1
0.4166666667
1
0.376114082
0.376114082
0.5588235294
0.1666666667
0.1666666667
1
1
1
1
0.6256684492
0.25
0.5588235294
0.5588235294
0.5588235294
1
1
1
1
1
0.376114082
1
0.5588235294
0.5588235294
0.4178238744
1
1
1
0.5
0.5
1
0.376114082
0.6256684492
1
1
0.6256684492
0.1666666667
1
0.5588235294
0.5588235294
0.5588235294
1
0.4166666667
1
1
0.5
0.5588235294
1
1

1
0.5588235294
1
0.4166666667
0.1666666667
0.376114082
1
0.4178238744
1
0.4166666667
1
1
1
1
1
1
1
0.4166666667
0.4166666667
1
0.6256684492
1
1
1
0.6256684492
0.5588235294
1
1
0.1666666667
0.376114082
0.1379790553
0.1379790553
0.1666666667
1
1
0.1666666667
1
1
1
1
0.1379790553
0.6256684492
1
0.4166666667
0.4166666667
1
1
1
0.4166666667
1
1

1
1
1
1
0.209972112
0.209972112
0.6295677362
0.6295677362
0.209972112
1
1
1
0.5
1
1
1
1
1
1
1
0.5
0.6256684492
0.376114082
1
0.6773654476
0.6773654476
1
0.5588235294
1
1
1
1
0.4166666667
0.4166666667
0.6773654476
1
1
1
0.5
0.1379790553
1
1
1
0.5
0.1666666667
0.1666666667
0.6256684492
1
0.4166666667
1
1

0.6256684492
1
0.6295677362
1
1
1
0.5588235294
0.4166666667
1
0.2869875223
1
1
0.4166666667
0.4166666667
1
0.25
1
1
1
1
0.4166666667
1
0.4166666667
0.2869875223
0.4166666667
1
0.4166666667
1
0.2869875223
0.061942959
1
0.4166666667
1
1
1
0.6773654476
0.6773654476
1
1
0.5588235294
1
0.4178238744
0.5588235294
1
1
1
1
1
0.4166666667
0.4166666667
0.376114082

0.4166666667
0.5588235294
0.4166666667
0.4166666667
0.6256684492
0.6256684492
0.4166666667
1
1
1
0.4166666667
0.5588235294
1
1
1
0.376114082
0.4166666667
1
1
1
0.1379790553
0.25
1
0.5588235294
0.4178238744
0.4178238744
1
1
1
1
1
1
1
0.25
1
1
0.6256684492
1
0.5
0.6256684492
0.5588235294
1
1
1
0.4166666667
1
1
1
0.25
0.4166666667
0.6256684492

0.1379790553
1
1
1
1
1
0.6773654476
0.6773654476
1
1
1
1
1
0.4166666667
0.4166666667
0.4166666667
0.4166666667
1
1
1
0.0637254902
0.4166666667
1
1
1
1
0.25
1
0.5588235294
0.2869875223
0.5588235294
0.1379790553
1
1
0.5588235294
0.5
1
1
0.4166666667
0.376114082
1
1
1
1
1
0.5588235294
1
0.6256684492
0.5
1
1

0.4166666667
0.6256684492
1
1
1
1
0.209972112
0.209972112
0.209972112
0.4166666667
1
1
1
0.6256684492
1
0.1247771836
1
1
0.1666666667
1
1
0.6773654476
1
1
1
1
0.5588235294
1
1
0.5
0.2869875223
0.5588235294
0.376114082
1
0.5
1
1
0.1666666667
1
0.209972112
1
0.5588235294
1
0.4166666667
0.376114082
1
1
1
1
0.6773654476
0.6773654476

1
1
1
0.25
1
0.6256684492
1
0.25
1
1
1
1
1
1
0.5588235294
0.5588235294
1
1
0.1666666667
1
0.6256684492
0.5
1
0.3666803232
1
1
0.4166666667
0.5
0.4166666667
1
0.25
0.5
1
1
0.5588235294
1
0.5
0.6256684492
0.6256684492
0.4166666667
1
1
0.6295677362
1
0.4178238744
1
0.6256684492
0.25
1
1
1

1
1
0.5
1
1
0.2869875223
1
1
1
0.5
0.4166666667
1
1
0.6256684492
0.4166666667
1
1
0.4166666667
1
1
1
0.5588235294
1
1
1
1
0.5588235294
1
0.1379790553
0.5588235294
0.376114082
1
0.4166666667
0.5588235294
0.1666666667
0.4166666667
0.5588235294
0.5588235294
0.5588235294
1
0.5588235294
0.5588235294
1
0.5588235294
0.5588235294
1
1
1
1
1
0.5588235294

0.5588235294
0.5588235294
1
0.5588235294
1
0.5588235294
0.5588235294
0.5588235294
1
0.6295677362
1
1
1
1
0.5
0.5
0.1379790553
0.1666666667
0.5
0.5588235294
1
0.25
1
0.5588235294
1
1
1
0.376114082
1
0.2869875223
0.5588235294
1
1
0.209972112
1
1
0.5588235294
1
0.6256684492
1
1
1
1
1
0.25
0.5
1
0.4166666667
1
0.4166666667
0.4166666667

0.4166666667
1
1
1
1
1
1
0.6256684492
0.4166666667
1
0.6256684492
0.25
0.376114082
1
0.209972112
1
0.4166666667
0.1379790553
1
1
0.25
0.5
0.5
0.1379790553
0.25
0.4166666667
0.5
0.4166666667
0.4166666667
1
1
1
1
1
1
1
0.5
1
0.4178238744
0.5588235294
1
0.03042881375
0.03042881375
1
0.376114082
0.1666666667
0.6773654476
0.5
1
1
1

1
0.4166666667
0.4166666667
0.6256684492
0.4178238744
0.1247771836
0.1247771836
0.1666666667
1
0.5588235294
0.5588235294
1
1
1
0.25
1
1
0.6256684492
1
0.1666666667
0.5588235294
0.25
1
1
0.1666666667
0.4166666667
0.376114082
1
1
0.4166666667
0.25
1
1
0.4166666667
0.1247771836
0.1247771836
1
1
1
0.2869875223
0.5588235294
1
1
1
0.6256684492
0.209972112
0.1666666667
1
1
0.4166666667
1

1
0.1666666667
1
1
0.209972112
0.4166666667
1
1
0.4166666667
0.4166666667
0.4166666667
1
0.4166666667
1
0.5588235294
1
1
1
1
1
0.376114082
1
0.6256684492
1
0.1666666667
0.5588235294
0.4166666667
0.4166666667
1
1
1
0.376114082
0.4166666667
0.4166666667
0.4166666667
1
1
0.25
0.5588235294
1
1
0.5
0.4166666667
1
0.1666666667
0.25
0.3666803232
0.3666803232
0.4166666667
1
0.4166666667

1
0.4166666667
0.6256684492
0.5588235294
1
1
1
0.25
0.4166666667
0.2869875223
1
1
1
0.6256684492
1
0.2869875223
1
0.6295677362
1
1
1
0.6256684492
1
0.4166666667
0.376114082
1
0.5588235294
0.5588235294
0.5588235294
0.5588235294
1
1
0.4166666667
0.5588235294
1
1
0.6256684492
0.1379790553
0.4166666667
0.4166666667
1
1
0.6256684492
0.6256684492
0.6256684492
1
1
1
1
0.6256684492
0.4166666667

0.5
1
1
0.6773654476
0.376114082
0.376114082
0.4166666667
1
1
1
0.4166666667
0.6295677362
1
0.6256684492
0.5588235294
0.4166666667
0.4166666667
1
1
1
1
1
0.4166666667
0.4166666667
0.376114082
1
1
1
0.5588235294
0.25
1
1
0.1247771836
0.1247771836
0.2869875223
0.5588235294
1
1
0.4166666667
0.5
1
0.5588235294
0.5588235294
1
1
1
0.4166666667
0.6256684492
0.5
0.25
1

1
1
1
1
1
1
0.1379790553
0.4166666667
0.4166666667
1
0.4166666667
0.5
0.5
0.4166666667
1
0.061942959
0.5588235294
1
1
1
1
0.4166666667
1
0.5588235294
0.4166666667
0.4166666667
0.4166666667
0.2869875223
0.5588235294
1
1
0.6256684492
1
1
0.25
0.1666666667
1
1
1
1
0.6256684492
1
1
1
1
0.2869875223
0.5
1
0.6256684492
0.6256684492
0.6256684492

1
0.25
1
0.4166666667
1
1
1
1
0.4166666667
1
0.1666666667
1
0.4166666667
1
1
0.4166666667
1
1
0.4166666667
1
0.1666666667
0.25
1
1
1
1
1
0.4166666667
0.4166666667
1
0.6256684492
0.6256684492
1
0.25
0.25
0.4166666667
0.4166666667
1
1
1
1
1
1
0.6256684492
0.6256684492
0.6256684492
0.4166666667
1
0.1666666667
1
1

1
0.25
1
0.4166666667
0.5
0.4166666667
1
1
0.5588235294
0.4166666667
0.376114082
1
1
1
1
1
1
1
1
1
0.1247771836
1
0.25
0.25
1
0.5588235294
1
1
1
0.209972112
1
0.5588235294
1
0.1379790553
1
0.6256684492
1
1
1
1
0.4166666667
0.5
0.5
0.4166666667
1
1
0.1379790553
1
0.4166666667
0.6256684492
0.6256684492

1
1
0.5
0.1666666667
1
1
1
0.5588235294
1
1
0.1666666667
1
1
0.4166666667
0.4166666667
1
1
1
1
1
1
1
1
0.6256684492
1
0.4166666667
1
1
1
1
1
0.4166666667
1
0.4166666667
1
0.4166666667
0.4166666667
0.376114082
1
1
1
1
1
0.4166666667
0.376114082
1
1
1
0.4166666667
0.4166666667
1

1
0.4166666667
1
0.4166666667
1
1
1
0.4166666667
0.4166666667
0.5588235294
0.4166666667
0.4166666667
0.25
1
0.2869875223
0.5588235294
0.4166666667
0.4166666667
0.5
0.6295677362
0.6295677362
0.5588235294
1
1
1
1
1
0.1379790553
0.4166666667
1
1
0.5588235294
0.5
0.4166666667
1
1
0.6256684492
0.209972112
0.5
1
0.6256684492
1
0.6256684492
1
1
1
1
1
1
1
1

1
0.5588235294
1
1
0.376114082
1
1
0.4166666667
0.5588235294
1
1
0.4166666667
1
1
0.376114082
0.1666666667
1
1
1
1
1
0.4166666667
0.5588235294
0.2869875223
0.6256684492
0.4166666667
0.25
0.25
0.4166666667
0.4166666667
1
0.5588235294
0.5588235294
1
1
1
1
1
1
1
1
1
1
0.1666666667
1
1
1
1
0.4166666667
1
1

0.5588235294
0.5
0.6256684492
1
1
1
1
1
0.5588235294
0.5588235294
0.5588235294
0.5588235294
0.1666666667
0.5588235294
1
0.5588235294
1
1
1
1
0.5588235294
1
0.6256684492
1
1
1
1
1
1
1
0.2869875223
0.6256684492
0.6256684492
1
1
1
1
0.1247771836
0.5588235294
1
0.4166666667
0.4166666667
0.1666666667
0.5
0.1379790553
1
0.25
1
0.5
0.5
0.4166666667

1
1
0.5
1
0.4166666667
1
1
1
1
1
0.376114082
1
1
1
1
1
1
1
1
1
1
1
0.5588235294
0.5588235294
0.1666666667
1
0.4166666667
1
1
1
1
0.5
1
1
1
1
1
0.4166666667
1
1
0.376114082
0.376114082
1
0.5588235294
0.5588235294
0.5588235294
0.5588235294
0.5
0.6295677362
0.5588235294
1

0.1666666667
0.1666666667
0.1666666667
1
0.1666666667
0.5588235294
0.5588235294
1
0.4166666667
0.1666666667
0.4178238744
0.5588235294
0.4166666667
1
0.4166666667
0.4166666667
0.4166666667
0.6256684492
1
0.25
0.25
1
0.6256684492
0.376114082
1
0.4166666667
0.4166666667
0.6256684492
0.6256684492
1
1
1
0.25
1
0.6773654476
0.25
1
1
1
1
0.25
1
0.1666666667
1
0.4166666667
1
1
0.25
0.4166666667
0.2869875223
0.4166666667

1
0.1666666667
1
1
0.1666666667
1
0.1379790553
0.5
1
1
1
1
0.1666666667
1
1
0.4166666667
1
1
0.1666666667
0.5
0.5588235294
0.6256684492
1
0.5588235294
0.6773654476
1
0.25
1
1
0.2869875223
1
0.2869875223
0.2869875223
0.6773654476
1
0.6256684492
1
0.5
1
0.5588235294
1
1
0.6256684492
1
1
0.25
0.25
1
1
0.25
0.6256684492

0.6773654476
0.6773654476
1
0.4166666667
0.6256684492
0.4166666667
1
1
0.6773654476
1
0.25
0.25
0.25
0.25
0.25
0.25
1
0.6773654476
1
0.4166666667
0.4166666667
0.4166666667
1
0.4178238744
1
1
1
1
0.5588235294
1
1
1
1
1
0.376114082
1
1
0.4166666667
1
0.4166666667
0.6256684492
0.6256684492
1
1
1
1
0.4166666667
0.4166666667
0.6256684492
1
1

0.1379790553
0.1379790553
0.1379790553
0.1379790553
0.5588235294
1
1
1
1
0.5588235294
0.5588235294
1
0.2869875223
1
0.1666666667
0.4166666667
0.4166666667
0.6256684492
1
0.6256684492
0.4166666667
1
1
0.25
1
0.25
0.209972112
0.1666666667
0.5
1
0.4166666667
1
0.4166666667
1
1
1
0.5588235294
0.4166666667
0.5588235294
0.4166666667
0.4166666667
1
0.6256684492
0.1247771836
1
0.6256684492
1
1
0.5
0.2869875223
0.1666666667

1
1
0.4166666667
0.4166666667
0.4166666667
0.4166666667
0.4166666667
0.4166666667
0.4166666667
1
0.6295677362
0.5
0.1379790553
1
1
1
1
0.1666666667
1
0.4166666667
0.376114082
0.1379790553
0.25
1
1
0.5588235294
0.25
0.4166666667
1
1
1
1
0.5
0.6256684492
1
1
1
1
0.5588235294
1
0.25
0.4166666667
1
1
0.6256684492
0.1666666667
0.1666666667
1
1
1
0.5

1
1
0.5
1
1
0.6773654476
1
0.25
0.6256684492
0.6256684492
1
1
1
1
1
0.5588235294
1
0.25
1
1
0.5
1
0.6773654476
1
0.6256684492
1
0.4166666667
0.4166666667
1
0.1666666667
0.6256684492
0.4166666667
0.4166666667
1
1
1
1
1
1
1
0.4166666667
1
1
1
1
0.6256684492
1
0.376114082
0.4166666667
1
0.1666666667

0.5588235294
0.5588235294
1
1
1
0.376114082
1
0.4166666667
1
1
1
0.5588235294
0.5588235294
0.4166666667
1
1
0.4166666667
0.6256684492
0.4166666667
1
0.1666666667
1
0.1666666667
0.4166666667
1
1
1
0.376114082
1
1
1
1
1
1
0.209972112
1
1
0.25
0.5
1
0.6256684492
1
0.4166666667
1
1
0.5
1
1
1
0.1379790553
1

0.4166666667
0.2869875223
0.25
0.25
0.6256684492
1
1
1
1
0.1379790553
0.25
0.5
1
1
1
1
1
0.6256684492
1
1
1
0.4166666667
1
0.2869875223
0.5588235294
0.5588235294
0.5588235294
0.0637254902
0.4166666667
0.5
0.5
1
1
0.1666666667
0.1379790553
1
1
1
1
0.5588235294
1
0.1666666667
0.2869875223
0.25
1
1
1
0.25
0.376114082
1
1

1
0.4166666667
0.4166666667
1
1
1
1
0.5
1
0.25
0.5588235294
0.5588235294
0.4166666667
0.2869875223
0.2869875223
0.1666666667
0.1666666667
0.4166666667
0.4166666667
1
0.4166666667
1
0.1666666667
1
1
1
1
1
1
1
1
0.4166666667
1
0.5
1
0.6773654476
0.5588235294
0.4166666667
0.0637254902
0.1666666667
1
0.0637254902
1
0.5
0.4166666667
0.25
1
0.1666666667
1
0.1666666667
0.1666666667

0.6773654476
0.6773654476
0.5
1
1
0.2869875223
1
1
1
1
0.4166666667
0.4166666667
1
1
0.0637254902
0.4178238744
0.5
0.6256684492
0.6256684492
1
0.5588235294
0.5
0.5
0.25
1
1
0.1247771836
0.5
0.1666666667
1
0.6256684492
0.5588235294
1
1
0.2869875223
1
0.5
1
1
1
0.4166666667
1
0.5588235294
1
1
1
1
0.1379790553
0.4166666667
0.1379790553
0.25

0.5
0.5588235294
0.5588235294
1
0.1666666667
1
1
1
1
1
0.4166666667
1
1
0.4166666667
0.5
1
1
1
1
1
1
1
0.1666666667
0.5
0.5
1
1
1
0.5
0.5588235294
1
1
0.4166666667
0.2869875223
1
0.2869875223
1
0.6256684492
1
0.25
0.6256684492
1
1
0.6256684492
1
1
1
0.5
1
1
1

1
1
1
0.376114082
0.5588235294
1
1
1
1
0.6773654476
0.6256684492
1
1
0.4166666667
0.4166666667
0.2869875223
1
1
1
1
0.4166666667
0.4166666667
0.4166666667
0.1666666667
0.5588235294
1
0.1379790553
0.1666666667
0.376114082
1
0.6256684492
1
1
1
1
1
1
1
1
1
0.4166666667
1
1
0.25
1
0.6295677362
0.6295677362
1
0.25
0.4166666667
0.6256684492

0.1666666667
1
0.5588235294
1
1
1
1
1
1
0.376114082
0.376114082
0.5
0.4166666667
1
1
1
1
0.4166666667
0.5588235294
1
1
0.25
0.4166666667
0.5
0.5588235294
0.5588235294
0.5588235294
0.6773654476
1
1
0.6256684492
0.6256684492
0.5588235294
0.6256684492
1
1
1
0.4166666667
0.25
1
0.25
0.4178238744
0.4178238744
1
0.5
0.0637254902
0.4166666667
0.25
1
1
0.4166666667

1
0.376114082
0.376114082
0.4166666667
0.4166666667
0.4166666667
0.4166666667
0.4166666667
0.4166666667
0.4166666667
0.4166666667
0.4166666667
0.4166666667
0.4166666667
1
1
1
1
1
1
1
1
1
1
1
1
0.4166666667
0.25
1
1
1
1
1
1
1
1
1
0.2869875223
1
0.5588235294
0.5588235294
1
1
0.1666666667
1
1
1
1
1
0.4166666667
1

0.5
0.5
0.4166666667
0.4166666667
0.5588235294
0.4166666667
0.2869875223
0.4166666667
1
0.209972112
1
1
0.5588235294
1
1
1
1
1
0.1666666667
1
1
1
0.1379790553
0.4166666667
1
1
0.5588235294
1
1
0.5
1
0.2869875223
1
0.5588235294
1
0.5
0.5588235294
0.5588235294
0.25
0.2869875223
0.2869875223
0.2869875223
1
1
1
0.3666803232
1
1
0.25
1
1

1
0.1247771836
1
1
1
1
0.376114082
0.5
0.5
0.5588235294
0.209972112
1
0.209972112
0.209972112
1
0.209972112
1
0.5
1
0.25
1
1
0.5588235294
0.6256684492
1
1
0.4166666667
0.4166666667
1
0.25
1
0.5588235294
1
1
1
1
0.4166666667
0.4166666667
0.4166666667
0.25
0.4166666667
0.2869875223
1
1
0.3666803232
0.3666803232
0.5588235294
1
0.5588235294
0.6256684492
0.4166666667

0.376114082
0.1666666667
1
0.5
0.6256684492
0.4166666667
0.1247771836
1
0.4166666667
1
1
1
0.25
1
0.6256684492
1
0.4166666667
1
1
1
0.4166666667
1
1
0.5588235294
0.4166666667
0.4166666667
0.6256684492
0.4166666667
0.4166666667
1
0.4178238744
1
0.5
1
1
0.5
0.6256684492
1
1
1
0.061942959
0.4166666667
1
1
1
0.376114082
1
0.6773654476
1
1
0.376114082

0.6295677362
1
1
0.4166666667
0.1247771836
1
0.6256684492
0.5
1
1
0.5
0.4166666667
1
1
1
1
1
0.6256684492
1
0.4166666667
1
0.5588235294
1
1
0.6256684492
0.1666666667
0.25
0.6256684492
1
1
1
0.25
0.7287727104
1
0.4166666667
0.4166666667
1
1
0.5588235294
0.376114082
1
0.376114082
0.2869875223
0.2869875223
0.1666666667
0.5588235294
1
1
1
1
1

0.5588235294
1
0.1379790553
0.376114082
0.6256684492
1
0.5588235294
0.1666666667
0.4166666667
0.376114082
0.6256684492
0.5
0.5
1
1
1
0.4166666667
0.5
0.4166666667
1
0.2950732295
0.2950732295
0.2869875223
0.6773654476
1
0.376114082
1
1
0.6256684492
1
1
1
1
1
0.1666666667
0.376114082
1
0.4166666667
0.5588235294
0.6256684492
1
0.209972112
0.4166666667
0.4166666667
0.1379790553
0.5588235294
0.6773654476
1
1
0.6256684492
1

1
1
1
0.5588235294
0.4166666667
1
1
0.2869875223
0.5588235294
1
1
0.6256684492
1
1
1
0.1379790553
1
0.4166666667
0.5588235294
0.5
0.6256684492
1
0.1666666667
0.4166666667
0.4166666667
0.4166666667
1
1
0.4166666667
0.061942959
0.061942959
1
0.4166666667
1
0.376114082
0.1379790553
0.6256684492
1
1
0.4166666667
1
0.4166666667
1
0.5588235294
1
0.2869875223
1
0.4166666667
1
1
1

0.6256684492
1
0.6256684492
0.4166666667
1
1
1
0.5
0.6256684492
1
1
1
0.25
0.5588235294
0.5
1
1
1
0.0637254902
1
1
0.1666666667
1
1
1
1
1
1
0.5
0.1379790553
1
0.4166666667
1
0.2869875223
0.4166666667
0.5588235294
1
1
0.5588235294
0.5588235294
1
0.6773654476
0.6773654476
0.6773654476
0.6773654476
0.6773654476
0.6773654476
0.25
0.6773654476
0.6773654476
0.6773654476

0.6773654476
0.6773654476
0.1379790553
0.5
1
0.5
1
1
0.1666666667
0.5
0.2869875223
1
0.2869875223
1
0.5588235294
1
1
1
0.4166666667
1
1
1
1
0.25
0.25
0.5588235294
1
0.5588235294
0.4166666667
1
0.1666666667
1
0.25
0.25
0.25
0.4166666667
0.4166666667
0.5588235294
0.5588235294
0.5588235294
1
0.4166666667
0.4166666667
1
1
1
0.4166666667
0.4166666667
0.4166666667
0.4166666667
0.5

1
0.6256684492
1
1
0.6256684492
0.4166666667
0.5588235294
0.25
0.25
0.4166666667
1
0.4166666667
0.4166666667
1
1
1
1
1
0.5588235294
1
1
1
0.5588235294
1
1
0.6256684492
0.1666666667
1
1
1
1
1
0.6256684492
0.25
1
1
1
0.4166666667
0.1666666667
0.3666803232
0.376114082
0.6256684492
1
1
1
1
0.5
0.1379790553
0.5588235294
0.5588235294
1

0.5588235294
1
0.4166666667
0.5588235294
1
1
0.376114082
0.376114082
1
0.5
1
1
1
1
0.25
1
1
1
0.2869875223
1
0.25
1
0.25
1
0.4166666667
1
1
1
0.4178238744
0.5588235294
0.6773654476
0.6773654476
0.6773654476
0.1666666667
1
1
0.5588235294
0.4166666667
1
1
0.4166666667
1
0.5
1
0.4166666667
0.5588235294
1
0.6256684492
1
1
1

0.1666666667
0.061942959
1
1
0.4178238744
0.5
1
1
1
1
0.5
0.5588235294
0.6256684492
1
1
1
1
1
0.5588235294
1
1
0.5
1
1
1
0.25
0.376114082
0.6256684492
0.4166666667
0.5588235294
0.25
1
0.6256684492
0.4178238744
0.4178238744
1
0.4166666667
1
1
1
1
1
0.5588235294
0.5588235294
0.5588235294
1
1
1
0.061942959
0.6256684492
1

1
0.4166666667
0.25
0.25
0.5588235294
1
0.4166666667
1
0.1666666667
0.4166666667
1
1
1
1
1
0.5588235294
0.5
0.4166666667
0.6256684492
0.376114082
1
0.5588235294
1
0.5588235294
1
1
0.4166666667
0.6256684492
0.4166666667
0.4166666667
1
0.25
0.5588235294
0.5588235294
0.6256684492
1
0.6256684492
1
0.2869875223
0.6256684492
1
1
0.4166666667
0.209972112
0.209972112
0.4166666667
0.4166666667
1
1
0.2869875223
1

0.6256684492
0.4166666667
1
0.6295677362
0.1379790553
0.1379790553
0.5588235294
0.4166666667
1
1
1
1
1
1
0.6256684492
0.1666666667
1
1
0.1666666667
0.4166666667
1
0.4166666667
1
1
1
1
1
0.6256684492
0.25
1
1
1
1
0.5
1
0.25
1
1
1
0.5588235294
0.4178238744
0.4166666667
1
1
1
0.6773654476
1
1
0.6256684492
0.6256684492
0.6256684492

0.6256684492
1
1
1
1
0.376114082
1
0.5588235294
0.6256684492
0.6256684492
0.1379790553
1
1
1
1
1
1
1
0.5
0.5588235294
1
0.1666666667
0.5
0.6773654476
1
0.5
1
1
1
1
0.5588235294
0.2869875223
1
0.25
1
0.6256684492
0.376114082
0.6256684492
0.6256684492
1
1
1
1
1
1
0.4166666667
1
1
1
0.6256684492
1

0.209972112
1
1
0.6256684492
0.5588235294
0.6256684492
1
1
1
1
1
1
0.1666666667
1
0.5588235294
0.5588235294
0.5588235294
1
0.5
0.6256684492
1
0.5588235294
0.5588235294
1
1
1
0.6256684492
1
1
1
1
1
1
1
1
1
1
0.1666666667
1
1
1
1
1
0.6256684492
0.5
1
1
0.209972112
0.209972112
0.209972112
0.209972112

0.6256684492
0.5
0.6256684492
0.5
1
1
0.5588235294
0.6256684492
0.6256684492
1
1
0.5
0.5588235294
0.6256684492
0.5
1
1
1
1
1
0.25
1
1
1
1
1
0.5588235294
1
0.5588235294
1
1
1
1
1
0.6256684492
0.2869875223
0.6256684492
1
1
0.6256684492
0.6256684492
0.6256684492
0.6256684492
1
1
0.6256684492
0.6256684492
1
0.6256684492
0.4166666667
1

1
0.6773654476
0.6773654476
0.376114082
0.376114082
1
0.6256684492
1
0.209972112
1
1
0.376114082
1
1
1
1
1
1
1
1
0.6256684492
1
0.2869875223
0.4166666667
1
1
0.6256684492
1
1
0.5588235294
1
0.4166666667
0.5
1
1
1
1
0.5588235294
0.5
0.5
1
1
0.5
0.4166666667
0.5
0.6256684492
1
0.6256684492
0.6256684492
0.6256684492
0.6256684492

1
1
1
1
1
1
0.5
0.5
0.5
0.5
1
1
1
0.5588235294
0.5588235294
0.209972112
1
1
1
1
1
1
0.6256684492
0.6256684492
1
1
1
1
0.6256684492
0.6256684492
1
1
1
1
0.25
0.6256684492
0.6256684492
1
1
0.6773654476
1
1
1
1
0.6256684492
0.25
0.6256684492
0.6256684492
1
1
0.5

0.5
0.25
0.6256684492
1
0.376114082
0.376114082
0.6256684492
1
1
1
0.1247771836
0.4178238744
0.1666666667
1
0.209972112
0.25
1
1
1
1
1
1
1
1
1
1
1
0.6256684492
0.5
1
1
1
1
0.6256684492
0.6256684492
0.6256684492
1
0.6256684492
0.6256684492
0.6256684492
0.6256684492
0.6256684492
0.6256684492
1
1
1
0.6256684492
0.6256684492
0.6256684492
0.6256684492
1

0.6256684492
1
1
1
1
1
1
1
1
1
1
1
1
1
1
1
1
1
1
1
1
1
1
1
1
0.6256684492
0.6256684492
0.6256684492
0.6256684492
1
1
0.6256684492
0.6256684492
0.6256684492
0.5
1
0.5588235294
0.1666666667
1
1
0.5588235294
0.209972112
1
1
1
0.6256684492
1
1
1
1
1

1
1
1
1
1
1
1
1
1
0.2869875223
1
1
1
0.5
1
1
1
0.4166666667
0.376114082
1
1
1
0.25
1
1
0.25
1
1
1
0.5588235294
0.5588235294
0.5
1
0.6773654476
1
1
1
0.6256684492
0.6256684492
1
0.25
0.25
0.6256684492
0.6256684492
0.25
0.5
0.5
1
0.25
1
1

0.5
0.25
0.5588235294
1
0.376114082
0.376114082
1
1
1
1
0.25
0.6256684492
1
0.6256684492
0.209972112
1
1
1
0.5588235294
1
0.5588235294
0.209972112
1
0.209972112
1
0.2869875223
0.5
1
0.6256684492
1
1
0.7287727104
0.5
0.6773654476
1
1
1
1
1
0.5
0.5
0.5
1
0.5
0.5
0.5
1
1
1
1
0.5

0.5
1
1
1
1
0.6256684492
0.5588235294
1
1
1
1
1
1
1
1
1
1
1
0.6256684492
0.6256684492
1
1
0.6256684492
1
0.5
1
1
1
1
1
1
1
1
0.6256684492
1
1
1
1
1
1
1
1
1
1
0.6256684492
0.6256684492
1
1
0.4166666667
1
0.6256684492

0.4166666667
1
1
1
1
0.6256684492
0.2869875223
1
1
1
1
1
0.25
0.4166666667
1
1
0.6773654476
1
0.6256684492
1
0.6256684492
1
1
1
1
1
1
0.25
0.5
1
0.2869875223
0.5
1
1
1
1
1
1
1
1
1
0.2869875223
1
0.25
0.5
0.5588235294
0.6256684492
0.5
0.5
0.6295677362
1

1
1
1
0.2869875223
0.2869875223
0.2869875223
0.5588235294
0.5588235294
0.6256684492
1
1
0.25
0.209972112
0.5
1
0.3666803232
1
1
1
1
0.6256684492
0.5588235294
0.6256684492
1
1
1
0.6256684492
1
0.5
0.5588235294
0.6256684492
0.6256684492
0.6256684492
0.6256684492
0.6773654476
0.4166666667
0.6773654476
1
1
1
1
0.376114082
0.376114082
1
1
1
1
1
1
1
0.1666666667
